# Supplementary material for: Genetic diversity of Schima superba based on physiological traits and SSR markers
Source: PLoS One. 2026 Apr 10;21(4):e0344465. doi: 10.1371/journal.pone.0344465 (PMC13068225; doi:10.1371/journal.pone.0344465)
Supplement: S1 Table — (DOCX) [file pone.0344465.s001.docx]

| **Population** | **Longitude** | **Latitude** |
| --- | --- | --- |
| HBB | 109.9812E | 22.2723N |
| HBN | 111.6924E | 23.8589N |
| HTHC | 111.5137E | 23.9546N |
| HQZ | 108.6230E | 22.0172N |
| HGC | 110.6305E | 24.9971N |
| HGY | 111.1206E | 25.5638N |
| HCW | 111.4765E | 23.8816N |
| HRS | 109.2322E | 25.1053N |

**Table S1** The longitude and latitude of the 8 *S. superba* provenance collection sites
