# Supplementary material for: Genetic diversity of Schima superba based on physiological traits and SSR markers
Source: PLoS One. 2026 Apr 10;21(4):e0344465. doi: 10.1371/journal.pone.0344465 (PMC13068225; doi:10.1371/journal.pone.0344465)
Supplement: S1 File — (ZIP) [file pone.0344465.s003.zip › SS02.pdf]

Allele Report

Project Comments:

Sample 1: SSS13_SS20_SS11_SS21_SS02_SS19_HBB10_E05.fsa

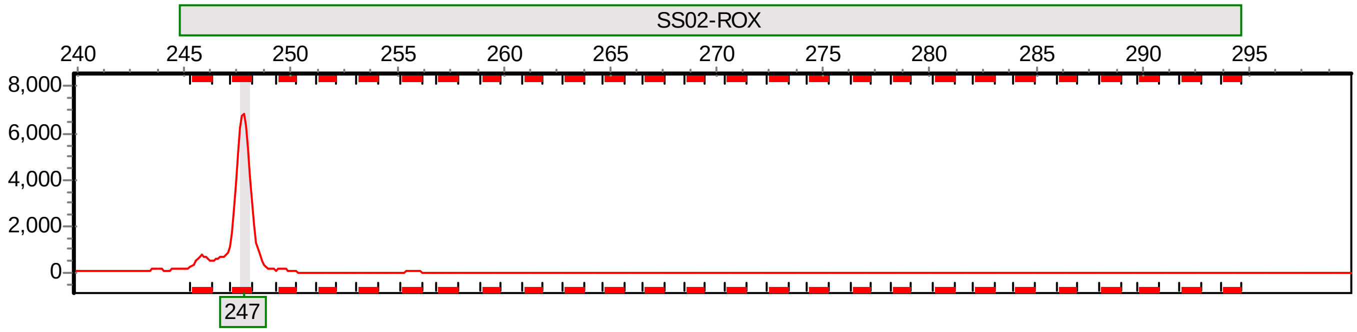

No	Size	Height	Area	Marker	Allele	Difference	Quality	Score	Allele Comments	Sample Comments
1	247.8	6845	54870	SS02-ROX	247	0.10	Pass	500.0	[<Confirmed>]	

Sample 2: SSS13_SS20_SS11_SS21_SS02_SS19_HBB12-2_D11.fsa

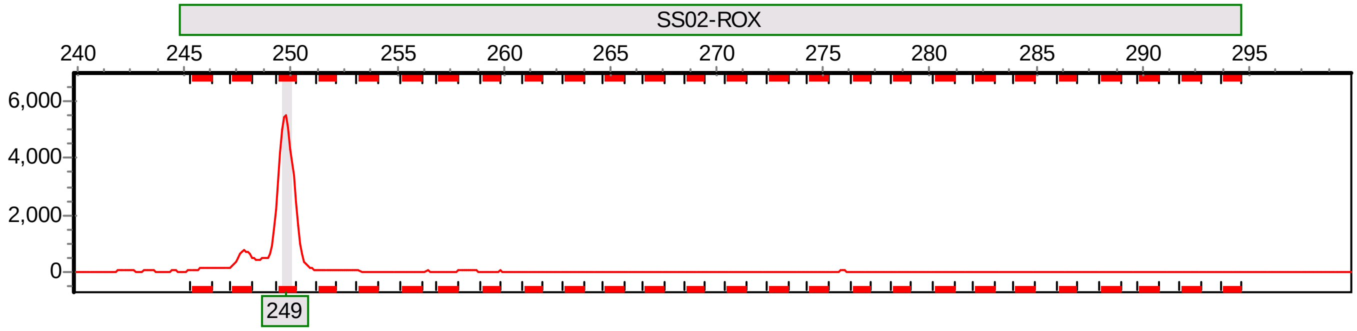

No	Size	Height	Area	Marker	Allele	Difference	Quality	Score	Allele Comments	Sample Comments
1	249.8	5489	44431	SS02-ROX	249	0.00	Pass	500.0	[<Confirmed>]	

Sample 3: SSS13_SS20_SS11_SS21_SS02_SS19_HBB13_C17.fsa

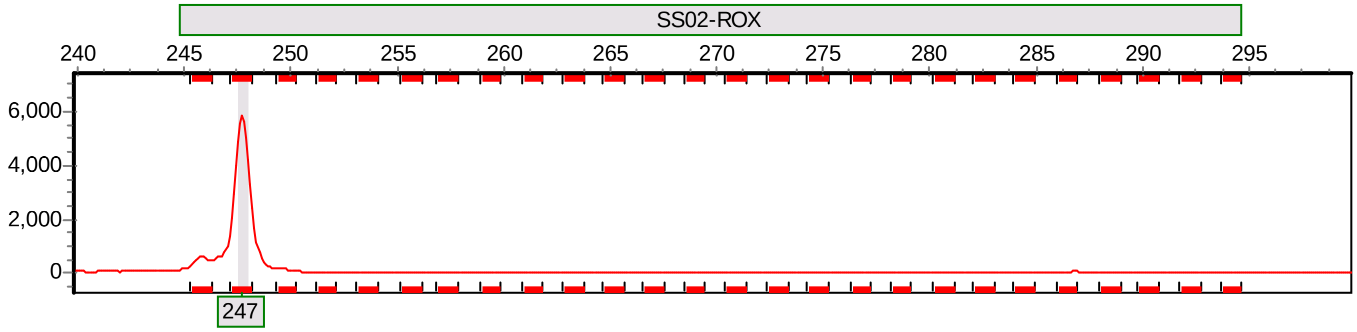

No	Size	Height	Area	Marker	Allele	Difference	Quality	Score	Allele Comments	Sample Comments
1	247.7	5849	49577	SS02-ROX	247	0.00	Pass	500.0	[<Confirmed>]	

Allele Report

Sample 4: SSS13_SS20_SS11_SS21_SS02_SS19_HBB14_G03.fsa

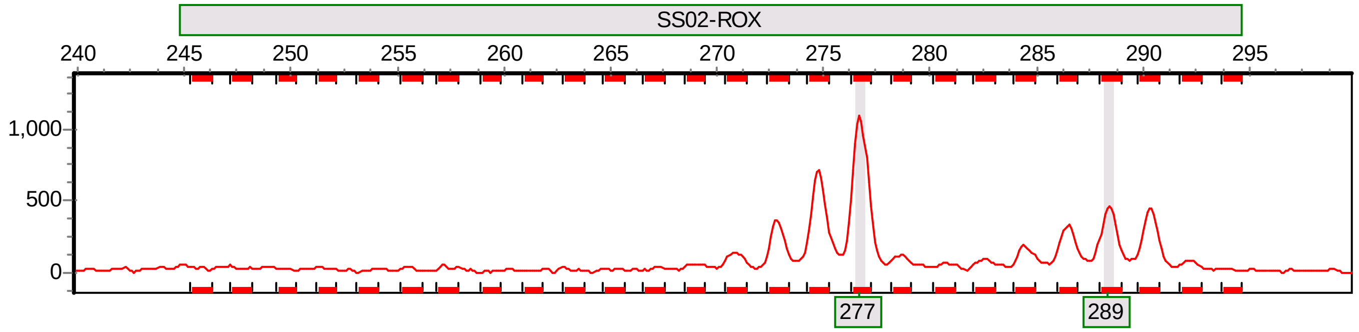

No	Size	Height	Area	Marker	Allele	Difference	Quality	Score	Allele Comments	Sample Comments
1	276.7	1099	9093	SS02-ROX	277	0.10	Pass	97.5	[<Confirmed>]	
2	288.4	469	4064	SS02-ROX	289	0.10	Pass	20.6	[<Confirmed>]	

Sample 5: SSS13_SS20_SS11_SS21_SS02_SS19_HBB15_B07.fsa

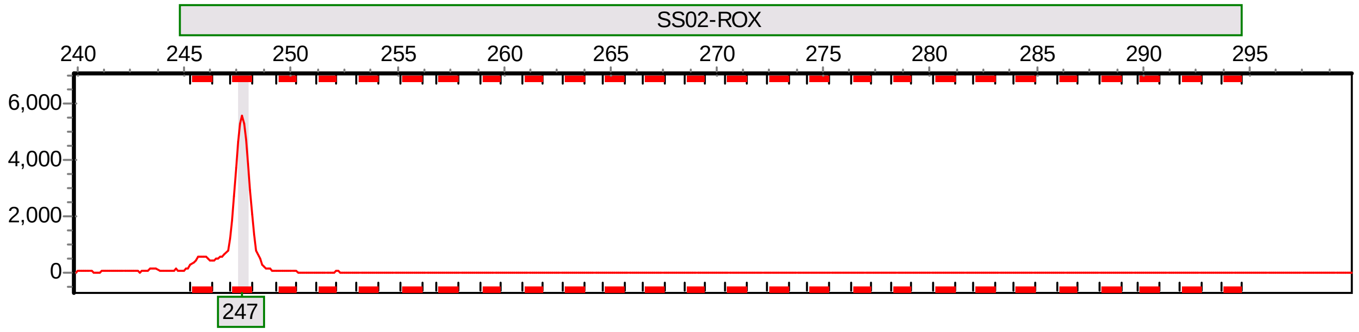

No	Size	Height	Area	Marker	Allele	Difference	Quality	Score	Allele Comments	Sample Comments
1	247.7	5525	44841	SS02-ROX	247	0.00	Pass	500.0	[<Confirmed>]	

Sample 6: SSS13_SS20_SS11_SS21_SS02_SS19_HBB16_G07.fsa

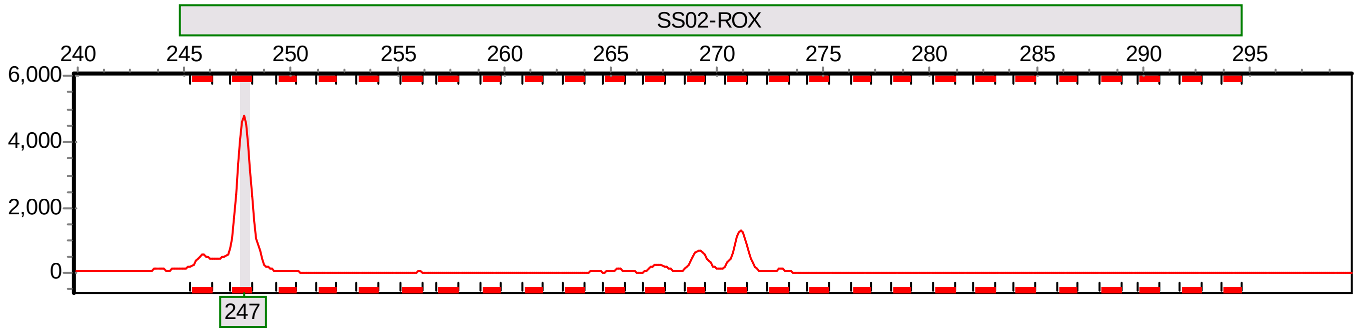

No	Size	Height	Area	Marker	Allele	Difference	Quality	Score	Allele Comments	Sample Comments
1	247.8	4769	37998	SS02-ROX	247	0.10	Pass	500.0	[<Confirmed>]	

Allele Report

Sample 7: SSS13_SS20_SS11_SS21_SS02_SS19_HBB17_K15.fsa

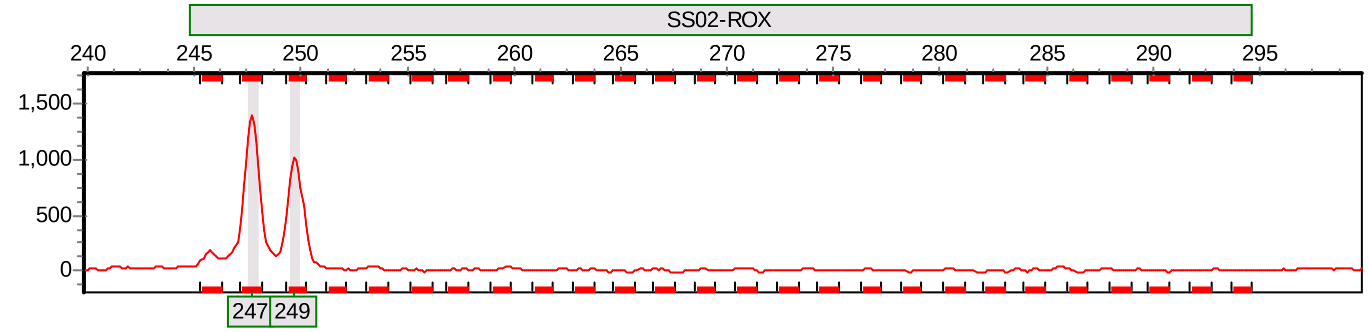

No	Size	Height	Area	Marker	Allele	Difference	Quality	Score	Allele Comments	Sample Comments
1	247.7	1386	11858	SS02-ROX	247	0.00	Pass	134.0	[<Confirmed>]	
2	249.7	1018	8194	SS02-ROX	249	0.10	Pass	88.4	[<Confirmed>]	

Sample 8: SSS13_SS20_SS11_SS21_SS02_SS19_HBB18_O05.fsa

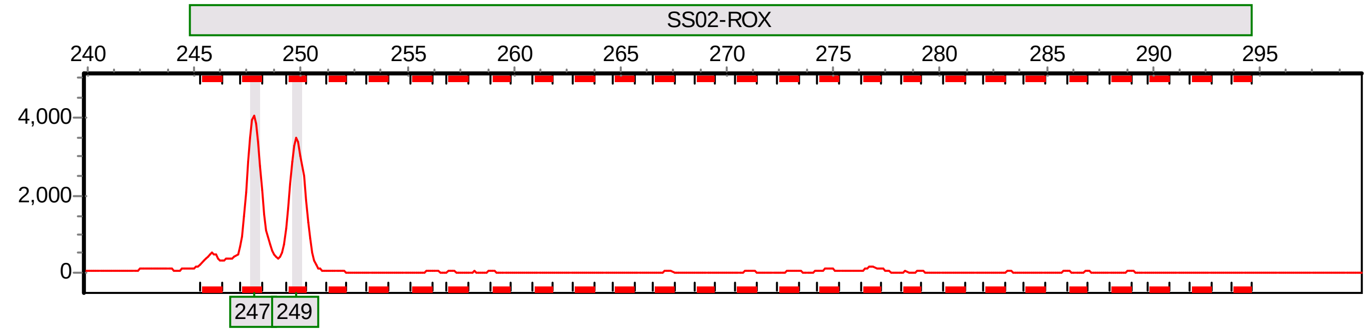

No	Size	Height	Area	Marker	Allele	Difference	Quality	Score	Allele Comments	Sample Comments
1	247.8	4033	33647	SS02-ROX	247	0.10	Pass	500.0	[<Confirmed>]	
2	249.8	3480	28136	SS02-ROX	249	0.00	Pass	500.0	[<Confirmed>]	

Sample 9: SSS13_SS20_SS11_SS21_SS02_SS19_HBB19_O07.fsa

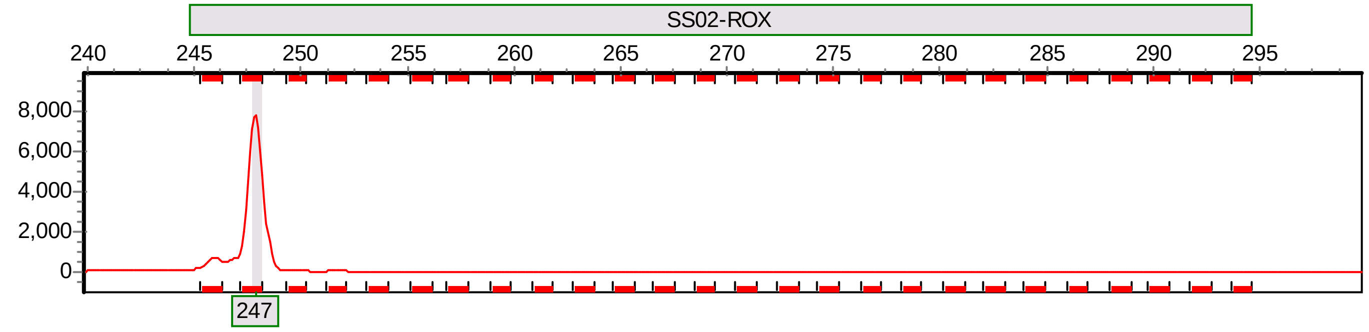

No	Size	Height	Area	Marker	Allele	Difference	Quality	Score	Allele Comments	Sample Comments
1	247.9	7748	62980	SS02-ROX	247	0.20	Pass	500.0	[<Confirmed>]	

Sample 10: SSS13_SS20_SS11_SS21_SS02_SS19_HBB1_B11.fsa

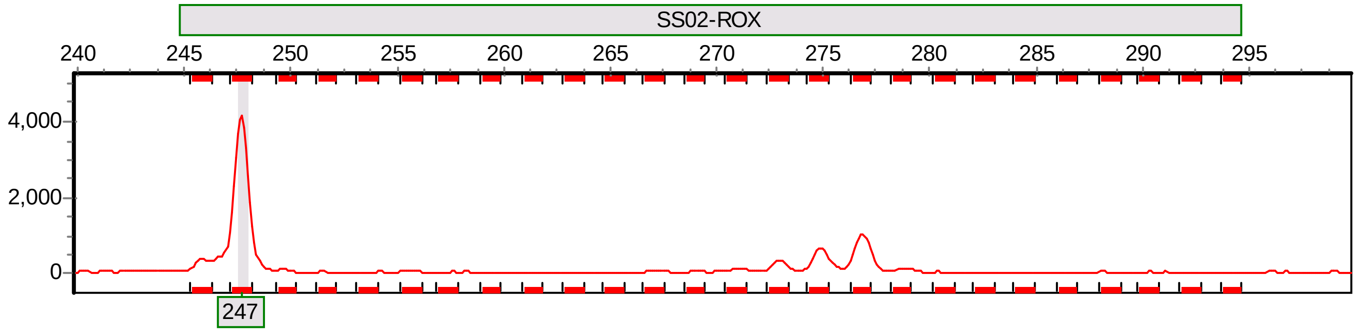

No	Size	Height	Area	Marker	Allele	Difference	Quality	Score	Allele Comments	Sample Comments
1	247.7	4164	33219	SS02-ROX	247	0.00	Pass	500.0	[<Confirmed>]	

Sample 11: SSS13_SS20_SS11_SS21_SS02_SS19_HBB20_F11.fsa

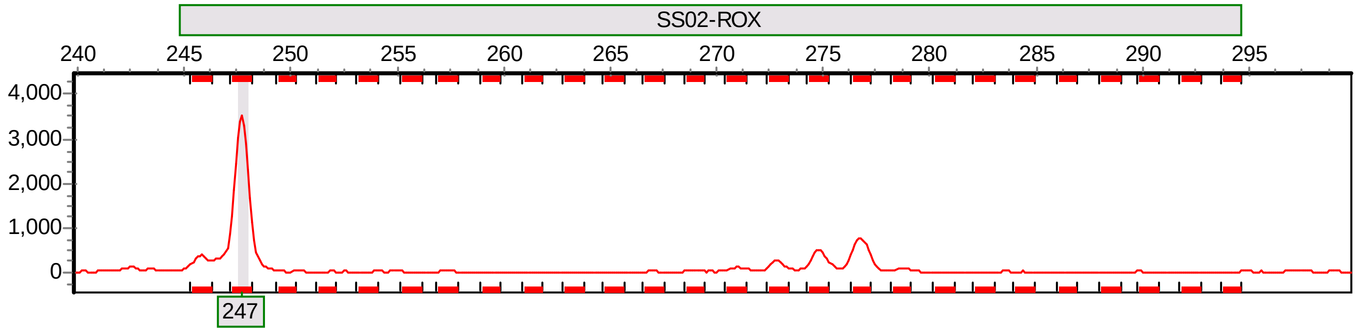

No	Size	Height	Area	Marker	Allele	Difference	Quality	Score	Allele Comments	Sample Comments
1	247.7	3502	28074	SS02-ROX	247	0.00	Pass	500.0	[<Confirmed>]	

Sample 12: SSS13_SS20_SS11_SS21_SS02_SS19_HBB21_J09.fsa

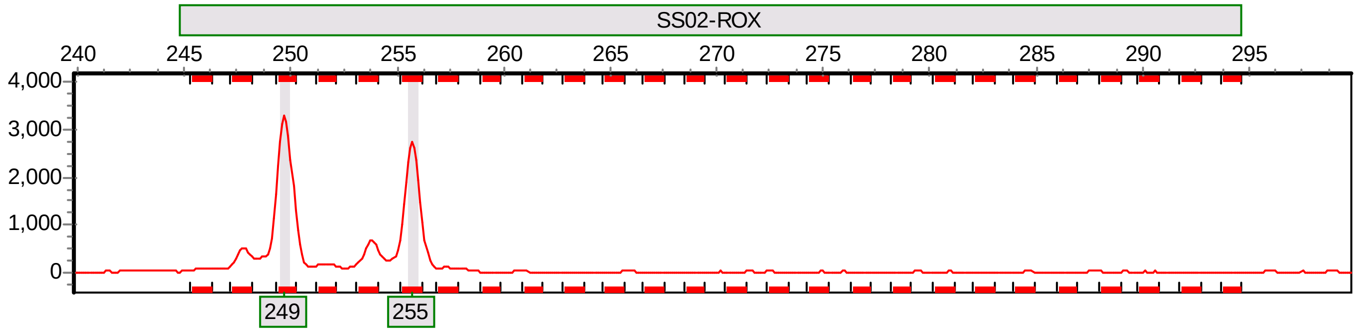

No	Size	Height	Area	Marker	Allele	Difference	Quality	Score	Allele Comments	Sample Comments
1	249.7	3266	27214	SS02-ROX	249	0.10	Pass	465.0	[<Confirmed>]	
2	255.7	2728	22824	SS02-ROX	255	0.00	Pass	360.1	[<Confirmed>]	

Allele Report

Sample 13: SSS13_SS20_SS11_SS21_SS02_SS19_HBB22_K05.fsa

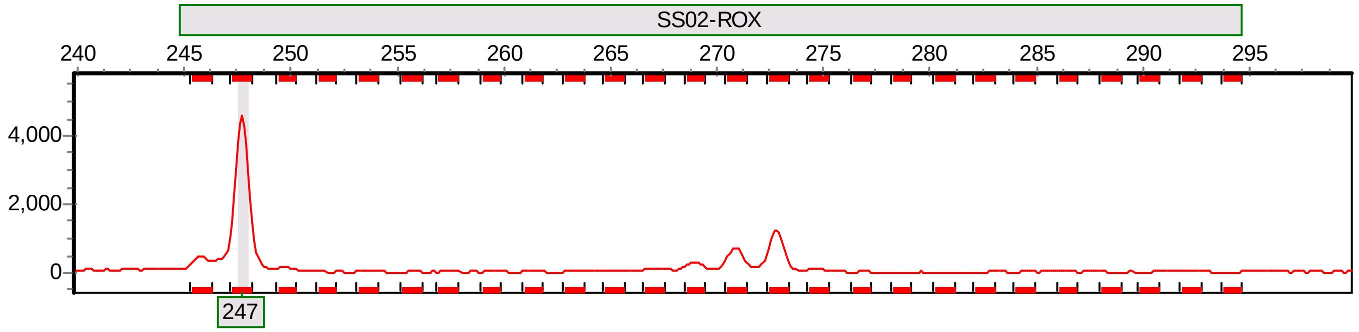

No	Size	Height	Area	Marker	Allele	Difference	Quality	Score	Allele Comments	Sample Comments
1	247.7	4561	35237	SS02-ROX	247	0.00	Pass	500.0	[<Confirmed>]	

Sample 14: SSS13_SS20_SS11_SS21_SS02_SS19_HBB23_B09.fsa

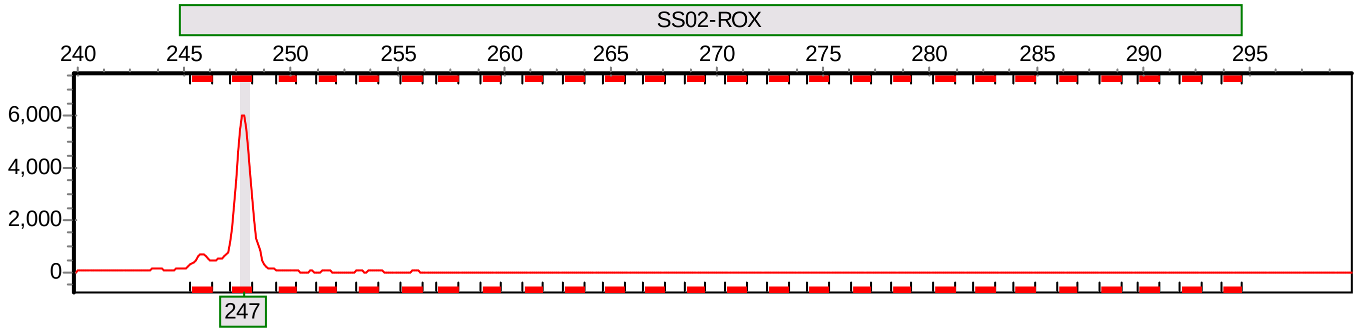

No	Size	Height	Area	Marker	Allele	Difference	Quality	Score	Allele Comments	Sample Comments
1	247.8	5970	50574	SS02-ROX	247	0.10	Pass	500.0	[<Confirmed>]	

Sample 15: SSS13_SS20_SS11_SS21_SS02_SS19_HBB25-1_F09.fsa

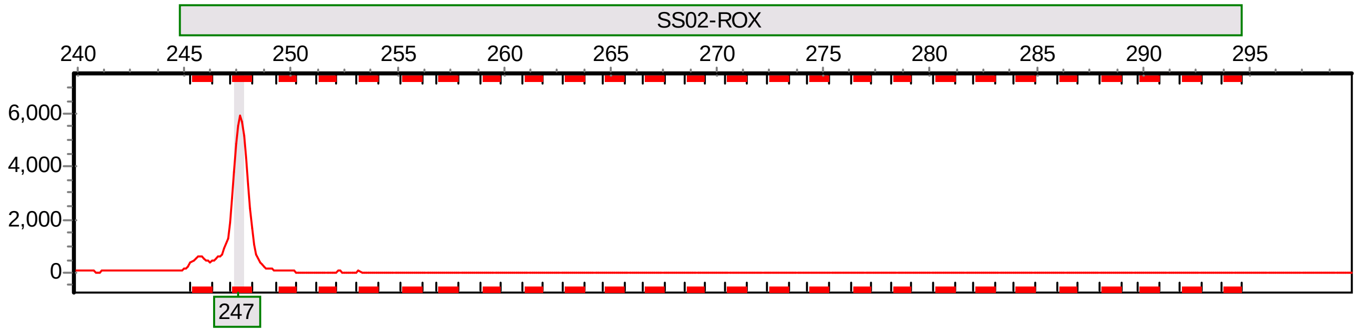

No	Size	Height	Area	Marker	Allele	Difference	Quality	Score	Allele Comments	Sample Comments
1	247.6	5857	47206	SS02-ROX	247	0.10	Pass	500.0	[<Confirmed>]	

Sample 16: SSS13_SS20_SS11_SS21_SS02_SS19_HBB25-2_N11.fsa

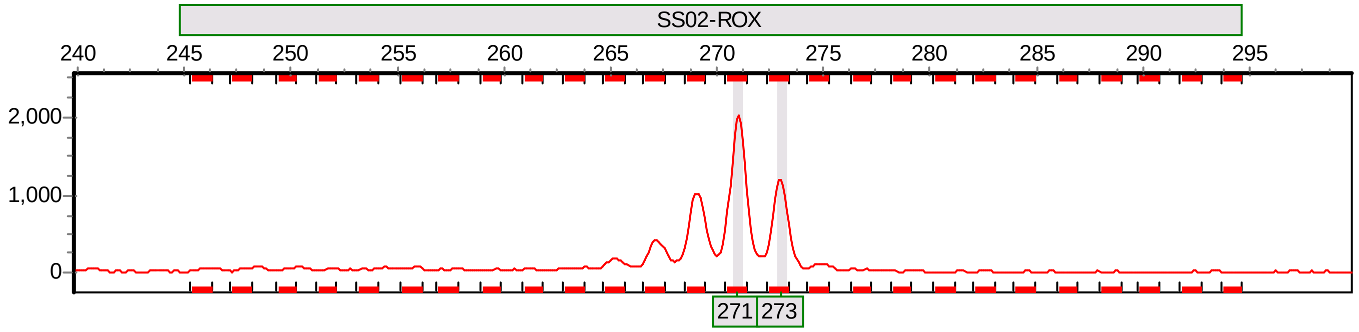

No	Size	Height	Area	Marker	Allele	Difference	Quality	Score	Allele Comments	Sample Comments
1	271.0	2016	17320	SS02-ROX	271	0.10	Pass	228.0	[<Confirmed>]	
2	273.0	1209	10349	SS02-ROX	273	0.10	Pass	102.3	[<Confirmed>]	

Sample 17: SSS13_SS20_SS11_SS21_SS02_SS19_HBB26_D07.fsa

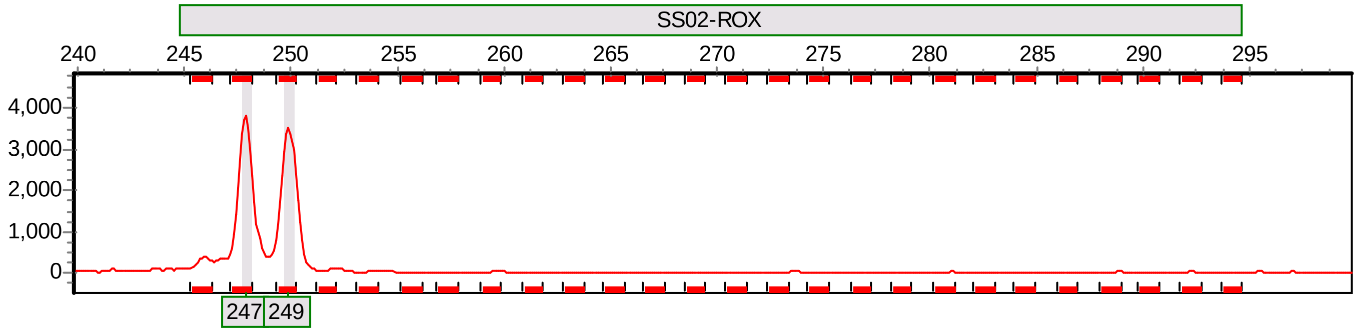

No	Size	Height	Area	Marker	Allele	Difference	Quality	Score	Allele Comments	Sample Comments
1	247.9	3805	29833	SS02-ROX	247	0.20	Pass	500.0	[<Confirmed>]	
2	249.9	3532	27977	SS02-ROX	249	0.10	Pass	500.0	[<Confirmed>]	

Sample 18: SSS13_SS20_SS11_SS21_SS02_SS19_HBB27_H05.fsa

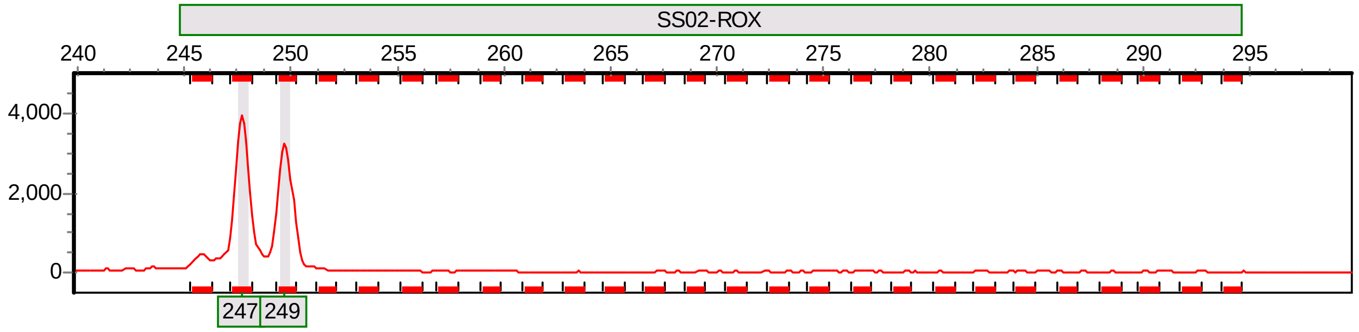

No	Size	Height	Area	Marker	Allele	Difference	Quality	Score	Allele Comments	Sample Comments
1	247.7	3951	31760	SS02-ROX	247	0.00	Pass	500.0	[<Confirmed>]	
2	249.7	3244	25583	SS02-ROX	249	0.10	Pass	500.0	[<Confirmed>]	

Allele Report

Sample 19: SSS13_SS20_SS11_SS21_SS02_SS19_HBB28_L11.fsa

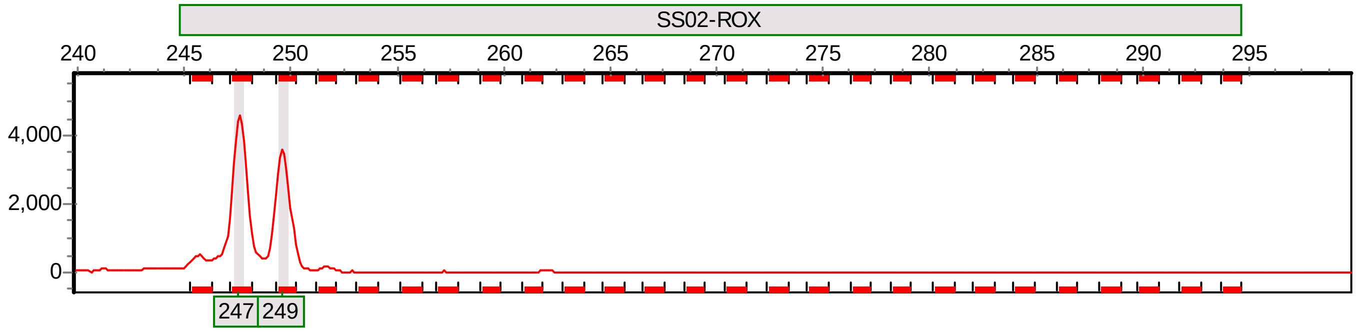

No	Size	Height	Area	Marker	Allele	Difference	Quality	Score	Allele Comments	Sample Comments
1	247.6	4535	37178	SS02-ROX	247	0.10	Pass	500.0	[<Confirmed>]	
2	249.6	3548	27623	SS02-ROX	249	0.20	Pass	500.0	[<Confirmed>]	

Sample 20: SSS13_SS20_SS11_SS21_SS02_SS19_HBB2_C07.fsa

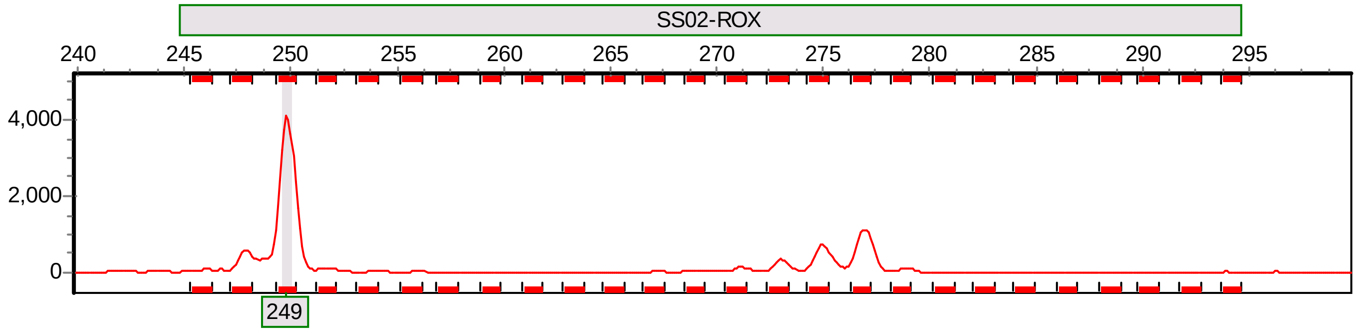

No	Size	Height	Area	Marker	Allele	Difference	Quality	Score	Allele Comments	Sample Comments
1	249.8	4070	32546	SS02-ROX	249	0.00	Pass	500.0	[<Confirmed>]	

Sample 21: SSS13_SS20_SS11_SS21_SS02_SS19_HBB30_L13.fsa

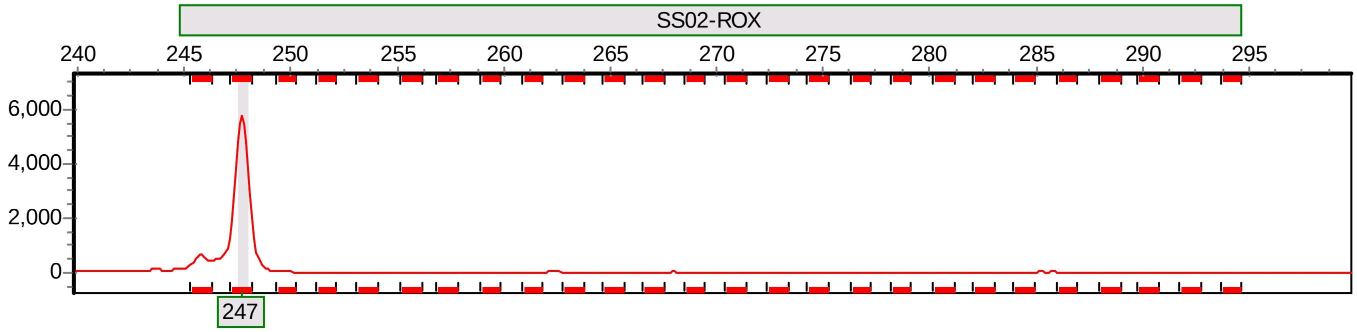

No	Size	Height	Area	Marker	Allele	Difference	Quality	Score	Allele Comments	Sample Comments
1	247.7	5713	45457	SS02-ROX	247	0.00	Pass	500.0	[<Confirmed>]	

Allele Report

Sample 22: SSS13_SS20_SS11_SS21_SS02_SS19_HBB31_P07.fsa

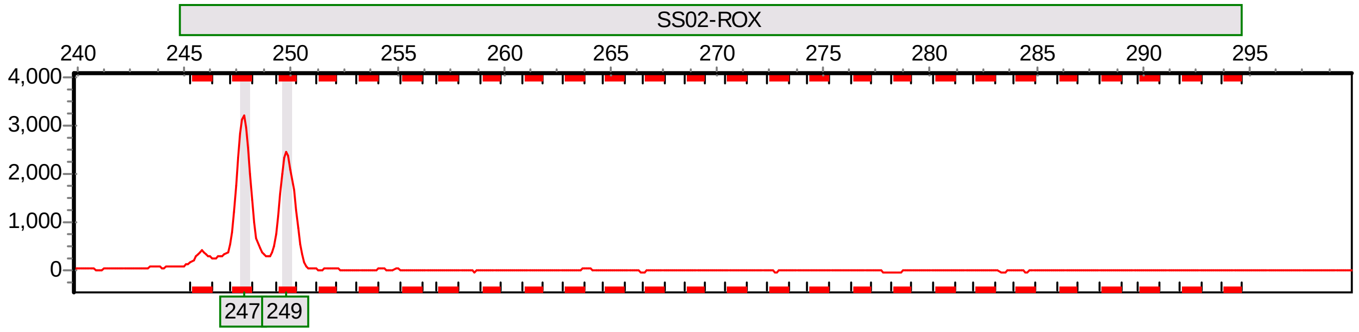

No	Size	Height	Area	Marker	Allele	Difference	Quality	Score	Allele Comments	Sample Comments
1	247.8	3189	25272	SS02-ROX	247	0.10	Pass	483.8	[<Confirmed>]	
2	249.8	2463	18968	SS02-ROX	249	0.00	Pass	361.0	[<Confirmed>]	

Sample 23: SSS13_SS20_SS11_SS21_SS02_SS19_HBB32_B15.fsa

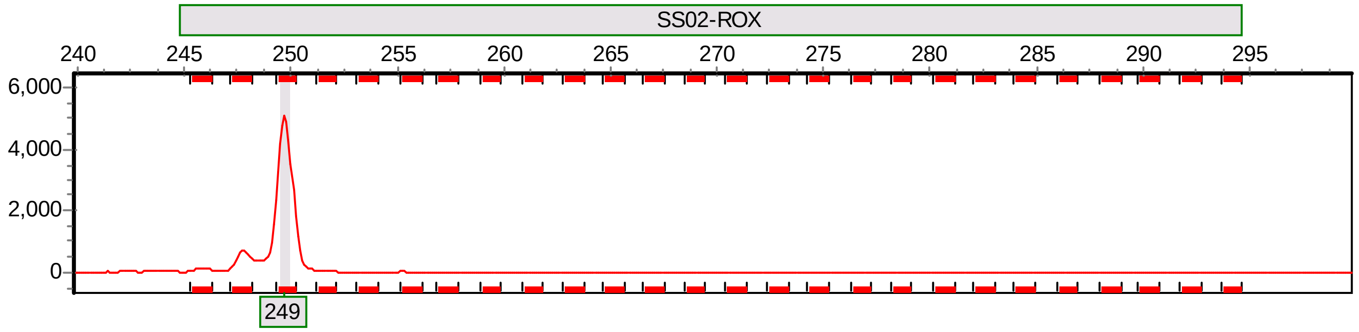

No	Size	Height	Area	Marker	Allele	Difference	Quality	Score	Allele Comments	Sample Comments
1	249.7	5055	39915	SS02-ROX	249	0.10	Pass	500.0	[<Confirmed>]	

Sample 24: SSS13_SS20_SS11_SS21_SS02_SS19_HBB33_N13.fsa

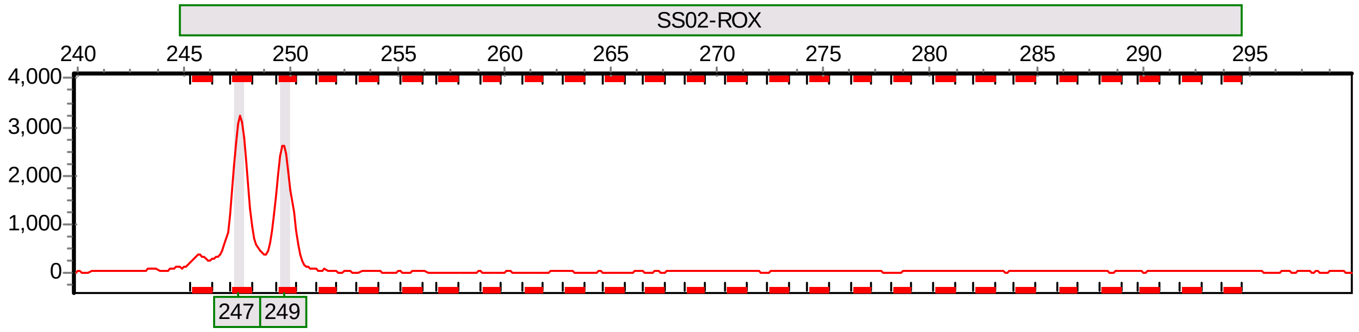

No	Size	Height	Area	Marker	Allele	Difference	Quality	Score	Allele Comments	Sample Comments
1	247.6	3213	27125	SS02-ROX	247	0.10	Pass	446.4	[<Confirmed>]	
2	249.7	2604	22570	SS02-ROX	249	0.10	Pass	319.5	[<Confirmed>]	

Sample 25: SSS13_SS20_SS11_SS21_SS02_SS19_HBB34_P13.fsa

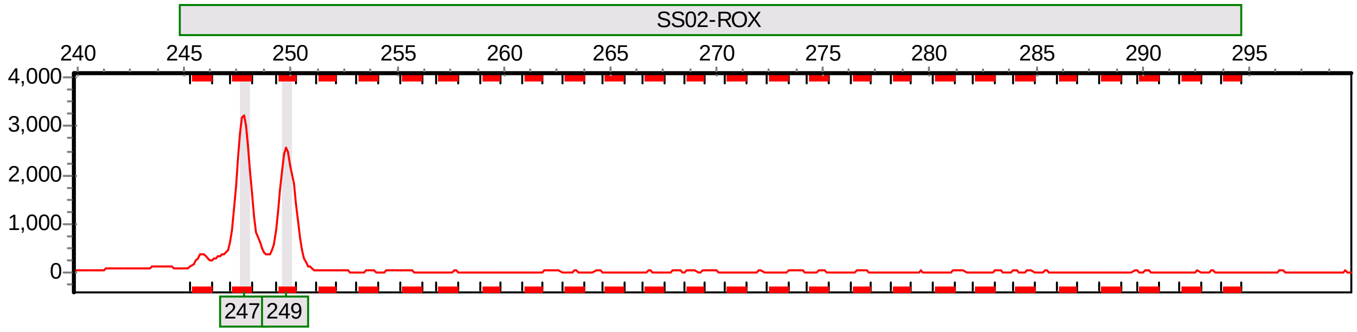

No	Size	Height	Area	Marker	Allele	Difference	Quality	Score	Allele Comments	Sample Comments
1	247.8	3203	27245	SS02-ROX	247	0.10	Pass	457.9	[<Confirmed>]	
2	249.8	2540	21141	SS02-ROX	249	0.00	Pass	342.4	[<Confirmed>]	

Sample 26: SSS13_SS20_SS11_SS21_SS02_SS19_HBB35_C15.fsa

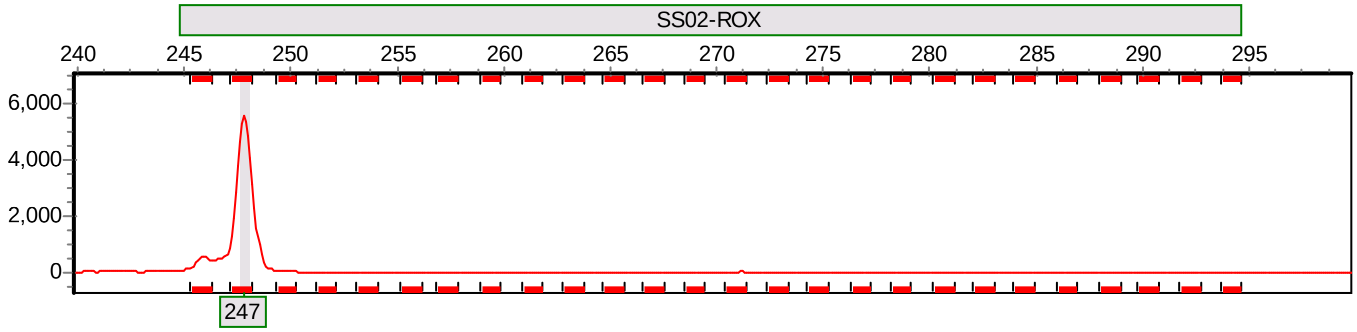

No	Size	Height	Area	Marker	Allele	Difference	Quality	Score	Allele Comments	Sample Comments
1	247.8	5572	47163	SS02-ROX	247	0.10	Pass	500.0	[<Confirmed>]	

Sample 27: SSS13_SS20_SS11_SS21_SS02_SS19_HBB36_G05.fsa

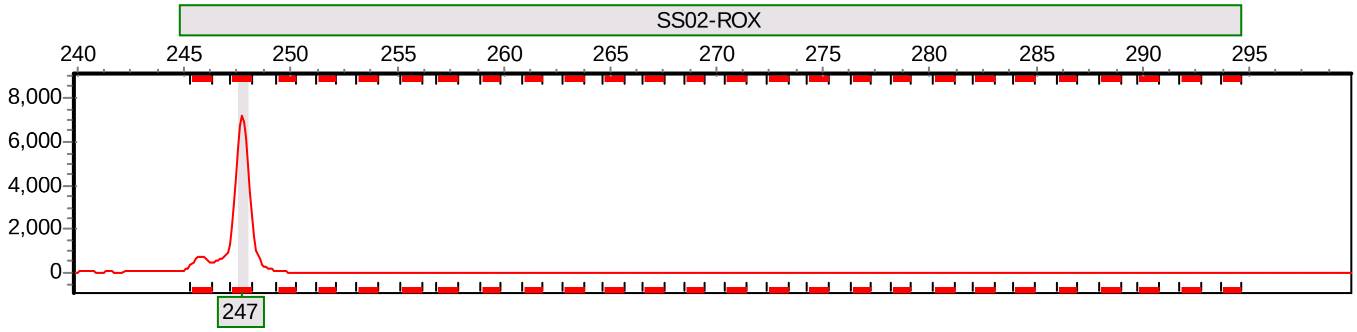

No	Size	Height	Area	Marker	Allele	Difference	Quality	Score	Allele Comments	Sample Comments
1	247.7	7175	55899	SS02-ROX	247	0.00	Pass	500.0	[<Confirmed>]	

Allele Report

Sample 28: SSS13_SS20_SS11_SS21_SS02_SS19_HBB37_N09.fsa

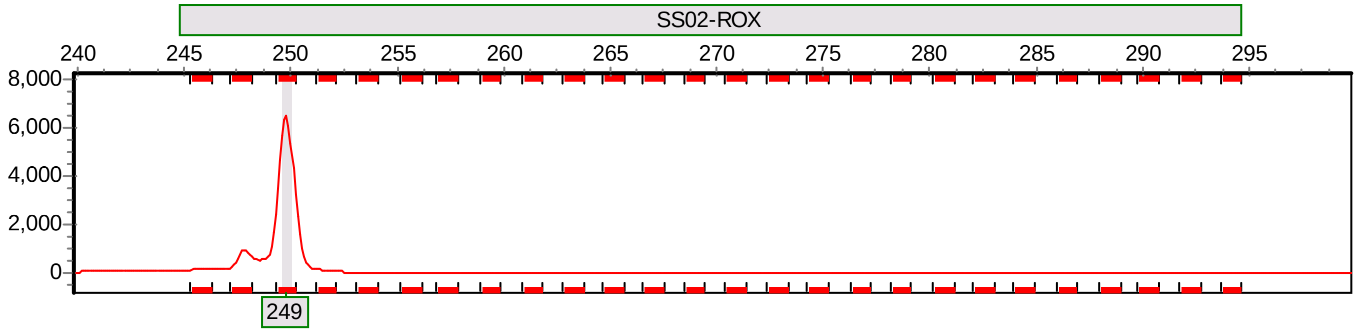

No	Size	Height	Area	Marker	Allele	Difference	Quality	Score	Allele Comments	Sample Comments
1	249.8	6450	53576	SS02-ROX	249	0.00	Pass	500.0	[<Confirmed>]	

Sample 29: SSS13_SS20_SS11_SS21_SS02_SS19_HBB38_M07.fsa

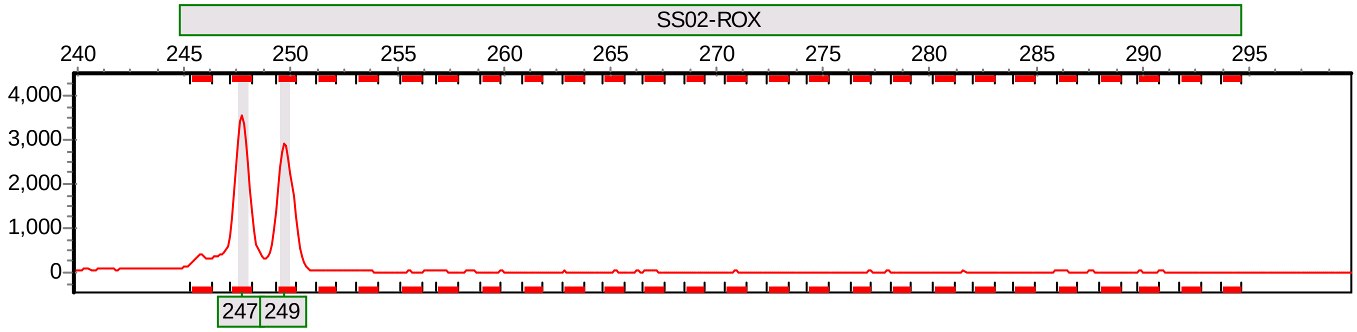

No	Size	Height	Area	Marker	Allele	Difference	Quality	Score	Allele Comments	Sample Comments
1	247.7	3517	29129	SS02-ROX	247	0.00	Pass	500.0	[<Confirmed>]	
2	249.7	2886	24381	SS02-ROX	249	0.10	Pass	384.9	[<Confirmed>]	

Sample 30: SSS13_SS20_SS11_SS21_SS02_SS19_HBB39_F05.fsa

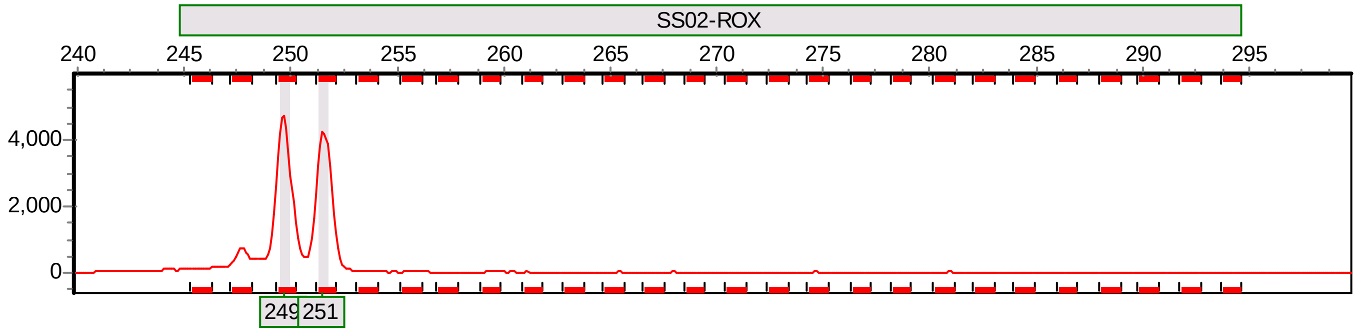

No	Size	Height	Area	Marker	Allele	Difference	Quality	Score	Allele Comments	Sample Comments
1	249.7	4696	36818	SS02-ROX	249	0.10	Pass	500.0	[<Confirmed>]	
2	251.5	4216	33307	SS02-ROX	251	0.20	Pass	500.0	[<Confirmed>]	

Sample 31: SSS13_SS20_SS11_SS21_SS02_SS19_HBB40_D15.fsa

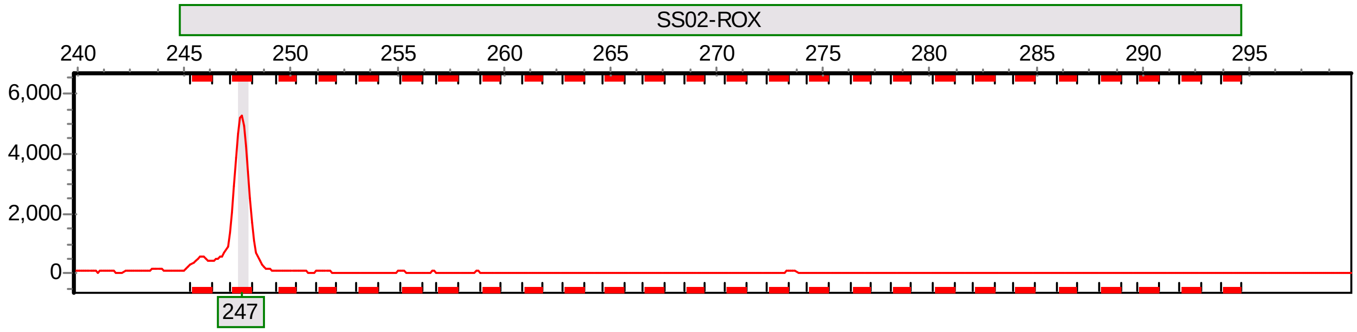

No	Size	Height	Area	Marker	Allele	Difference	Quality	Score	Allele Comments	Sample Comments
1	247.7	5256	42962	SS02-ROX	247	0.00	Pass	500.0	[<Confirmed>]	

Sample 32: SSS13_SS20_SS11_SS21_SS02_SS19_HBB41_L07.fsa

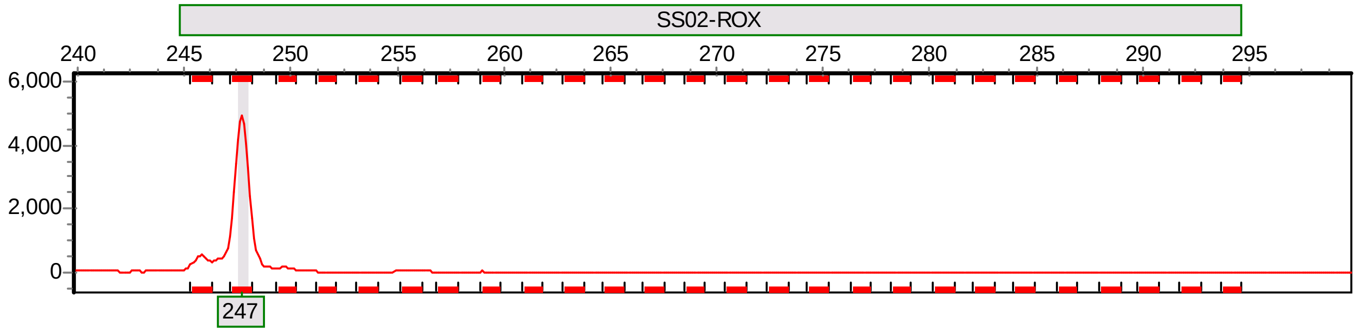

No	Size	Height	Area	Marker	Allele	Difference	Quality	Score	Allele Comments	Sample Comments
1	247.7	4902	39463	SS02-ROX	247	0.00	Pass	500.0	[<Confirmed>]	

Sample 33:

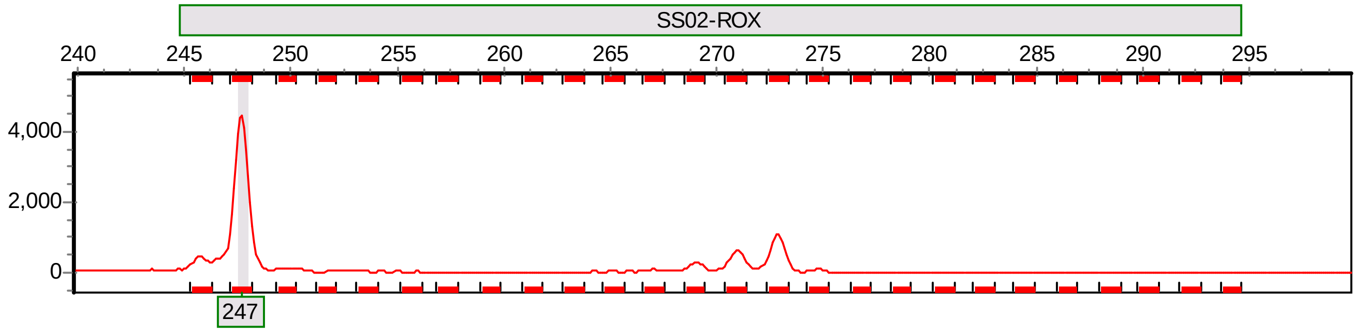

No	Size	Height	Area	Marker	Allele	Difference	Quality	Score	Allele Comments	Sample Comments
1	247.7	4462	35364	SS02-ROX	247	0.00	Pass	500.0	[<Confirmed>]	

SoftGenetics

Allele Report

GeneMarker V3.0.0

Sample 34: SSS13_SS20_SS11_SS21_SS02_SS19_HBB43_D09.fsa

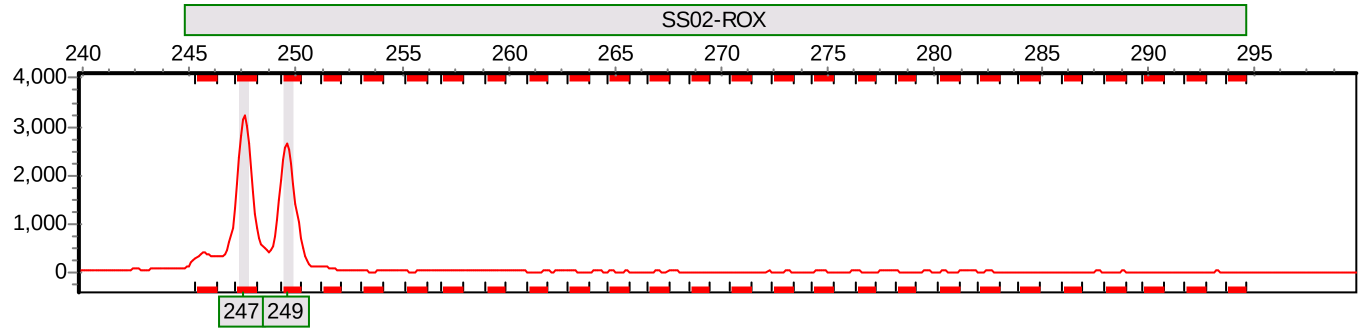

No	Size	Height	Area	Marker	Allele	Difference	Quality	Score	Allele Comments	Sample Comments
1	247.6	3212	26491	SS02-ROX	247	0.10	Pass	444.1	[<Confirmed>]	
2	249.6	2655	22602	SS02-ROX	249	0.20	Pass	332.9	[<Confirmed>]	

Sample 35: SSS13_SS20_SS11_SS21_SS02_SS19_HBB44_H11.fsa

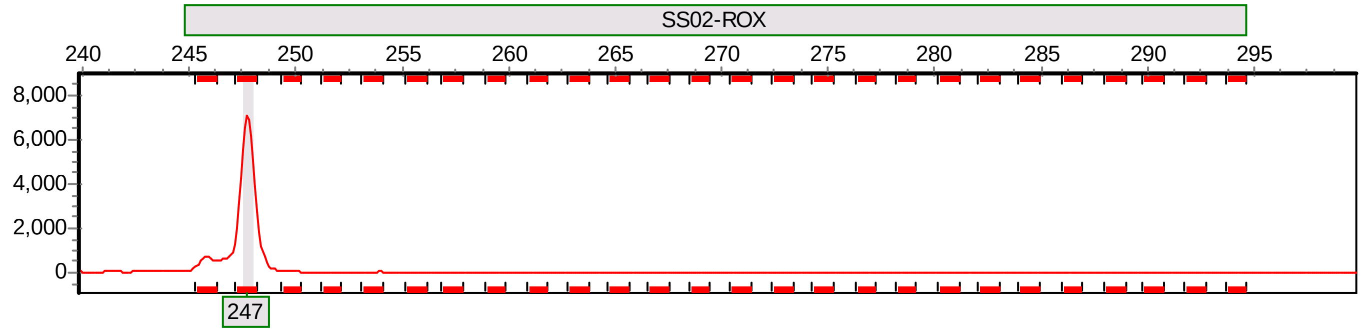

No	Size	Height	Area	Marker	Allele	Difference	Quality	Score	Allele Comments	Sample Comments
1	247.7	7081	55909	SS02-ROX	247	0.00	Pass	500.0	[<Confirmed>]	

Sample 36: SSS13_SS20_SS11_SS21_SS02_SS19_HBB45_F13.fsa

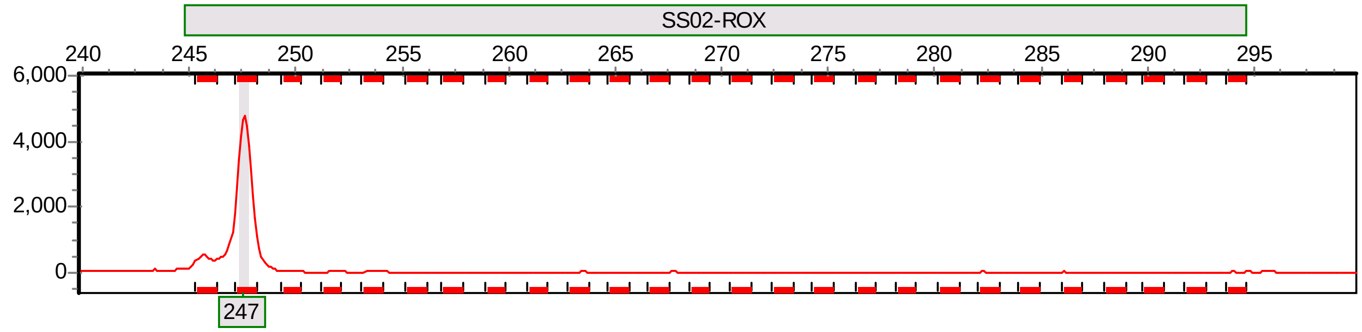

No	Size	Height	Area	Marker	Allele	Difference	Quality	Score	Allele Comments	Sample Comments
1	247.6	4752	39116	SS02-ROX	247	0.10	Pass	500.0	[<Confirmed>]	

Sample 37: SSS13_SS20_SS11_SS21_SS02_SS19_HBB46_F07.fsa

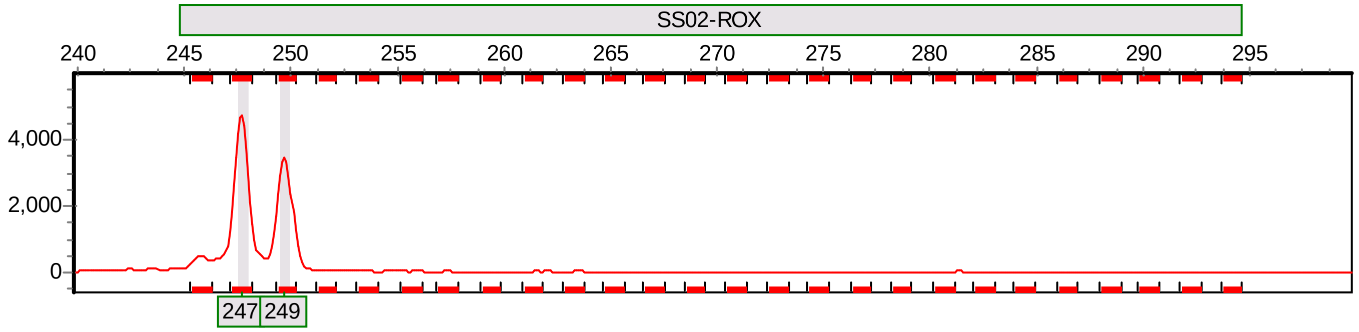

No	Size	Height	Area	Marker	Allele	Difference	Quality	Score	Allele Comments	Sample Comments
1	247.7	4672	36960	SS02-ROX	247	0.00	Pass	500.0	[<Confirmed>]	
2	249.7	3440	27730	SS02-ROX	249	0.10	Pass	500.0	[<Confirmed>]	

Sample 38: SSS13_SS20_SS11_SS21_SS02_SS19_HBB47_A05.fsa

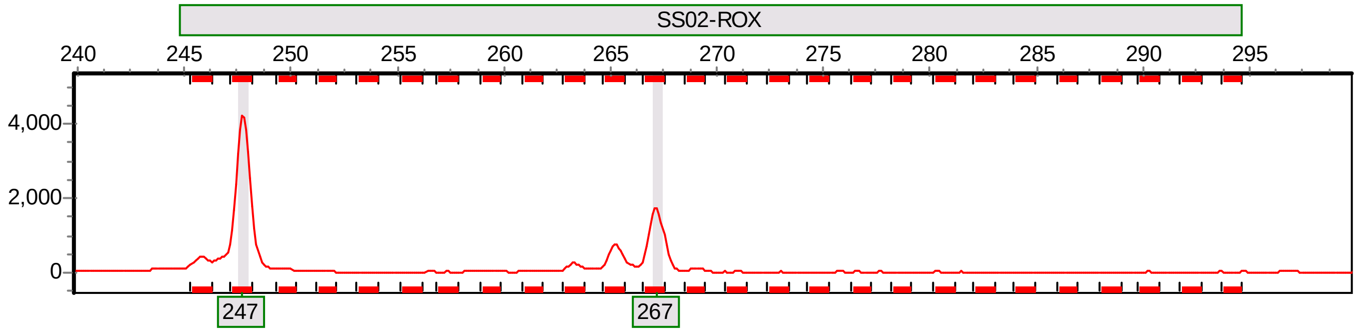

No	Size	Height	Area	Marker	Allele	Difference	Quality	Score	Allele Comments	Sample Comments
1	247.7	4211	33581	SS02-ROX	247	0.00	Pass	500.0	[<Confirmed>]	
2	267.2	1721	13850	SS02-ROX	267	0.10	Pass	199.6	[<Confirmed>]	

Sample 39: SSS13_SS20_SS11_SS21_SS02_SS19_HBB48_B13.fsa

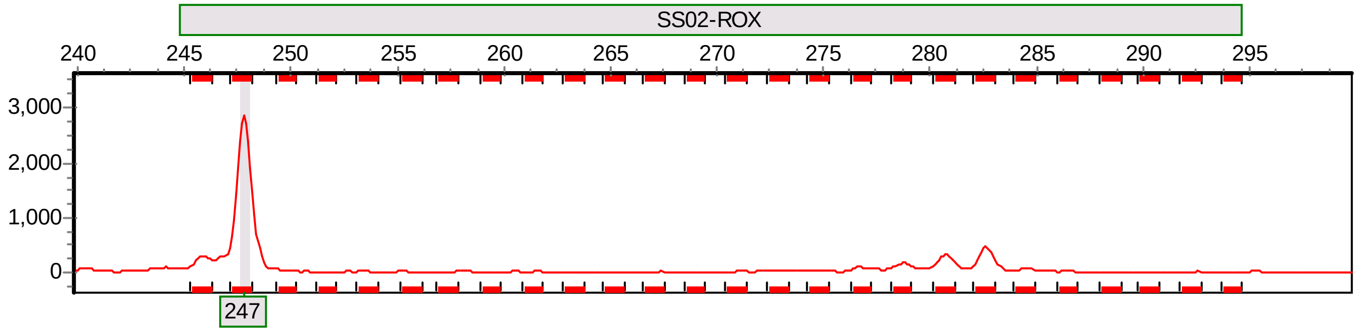

No	Size	Height	Area	Marker	Allele	Difference	Quality	Score	Allele Comments	Sample Comments
1	247.8	2846	23068	SS02-ROX	247	0.10	Pass	419.7	[<Confirmed>]	

Allele Report

Sample 40: SSS13_SS20_SS11_SS21_SS02_SS19_HBB49_L05.fsa

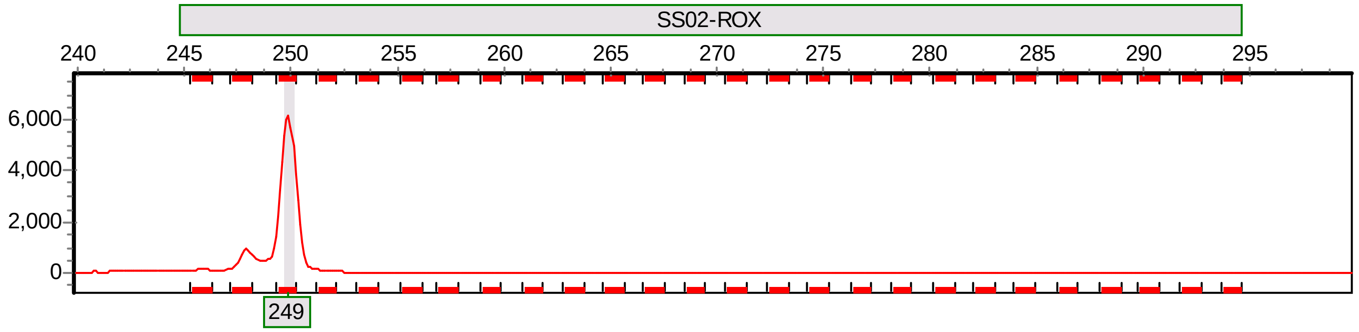

No	Size	Height	Area	Marker	Allele	Difference	Quality	Score	Allele Comments	Sample Comments
1	249.9	6140	48551	SS02-ROX	249	0.10	Pass	500.0	[<Confirmed>]	

Sample 41: SSS13_SS20_SS11_SS21_SS02_SS19_HBB4_E07.fsa

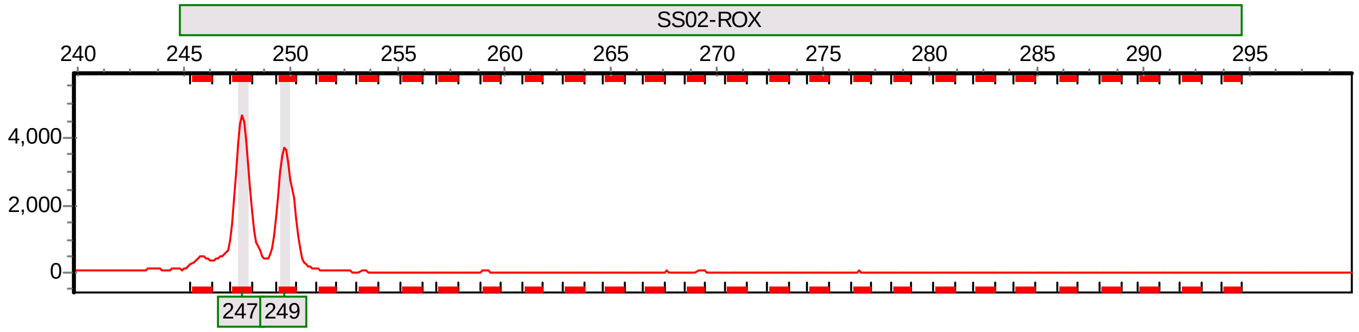

No	Size	Height	Area	Marker	Allele	Difference	Quality	Score	Allele Comments	Sample Comments
1	247.7	4658	37068	SS02-ROX	247	0.00	Pass	500.0	[<Confirmed>]	
2	249.7	3710	30581	SS02-ROX	249	0.10	Pass	500.0	[<Confirmed>]	

Sample 42: SSS13_SS20_SS11_SS21_SS02_SS19_HBB5_H13.fsa

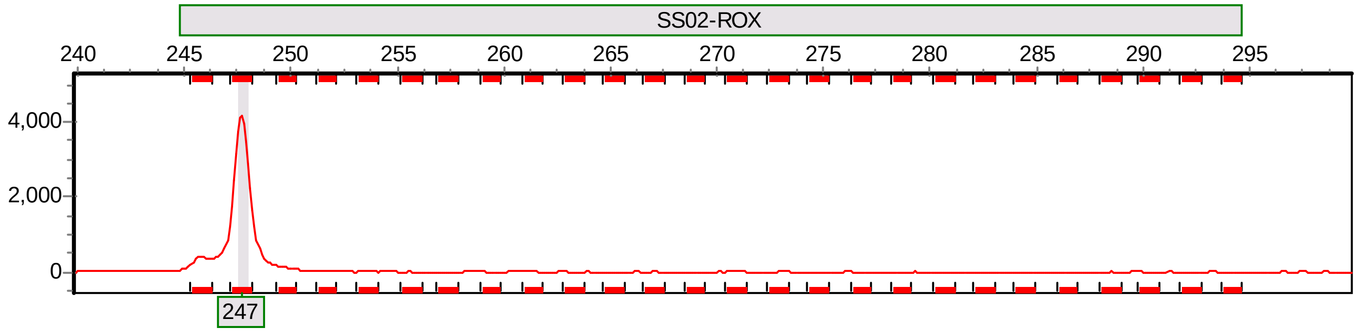

No	Size	Height	Area	Marker	Allele	Difference	Quality	Score	Allele Comments	Sample Comments
1	247.7	4135	35642	SS02-ROX	247	0.00	Pass	500.0	[<Confirmed>]	

Sample 43: SSS13_SS20_SS11_SS21_SS02_SS19_HBB6_C05.fsa

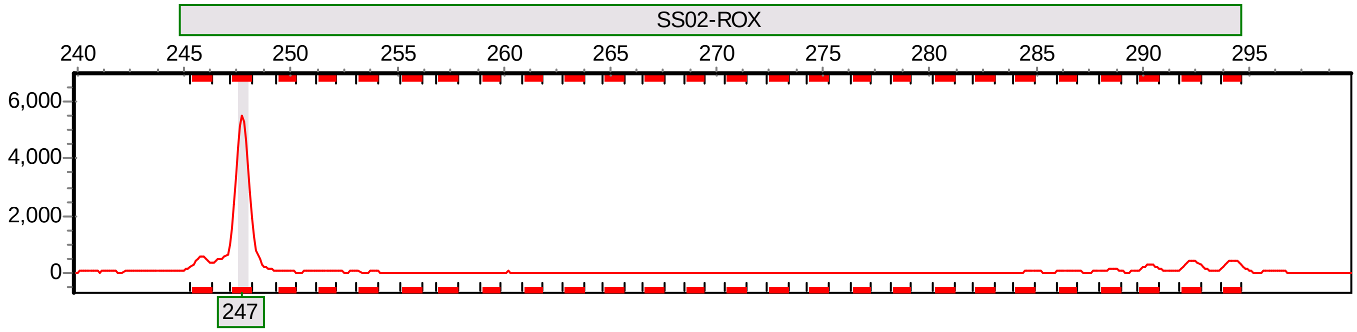

No	Size	Height	Area	Marker	Allele	Difference	Quality	Score	Allele Comments	Sample Comments
1	247.7	5467	41913	SS02-ROX	247	0.00	Pass	500.0	[<Confirmed>]	

Sample 44: SSS13_SS20_SS11_SS21_SS02_SS19_HBB7_N05.fsa

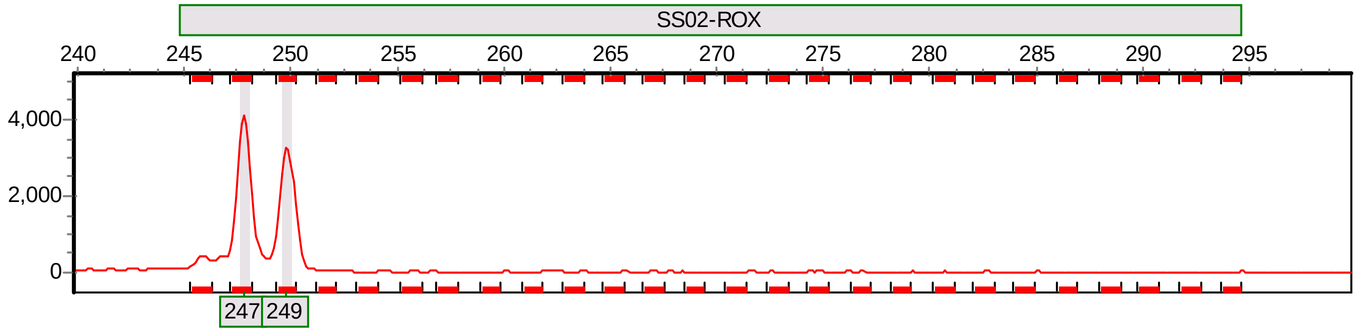

No	Size	Height	Area	Marker	Allele	Difference	Quality	Score	Allele Comments	Sample Comments
1	247.8	4078	32117	SS02-ROX	247	0.10	Pass	500.0	[<Confirmed>]	
2	249.8	3255	25628	SS02-ROX	249	0.00	Pass	500.0	[<Confirmed>]	

Sample 45: SSS13_SS20_SS11_SS21_SS02_SS19_HBB8_P11.fsa

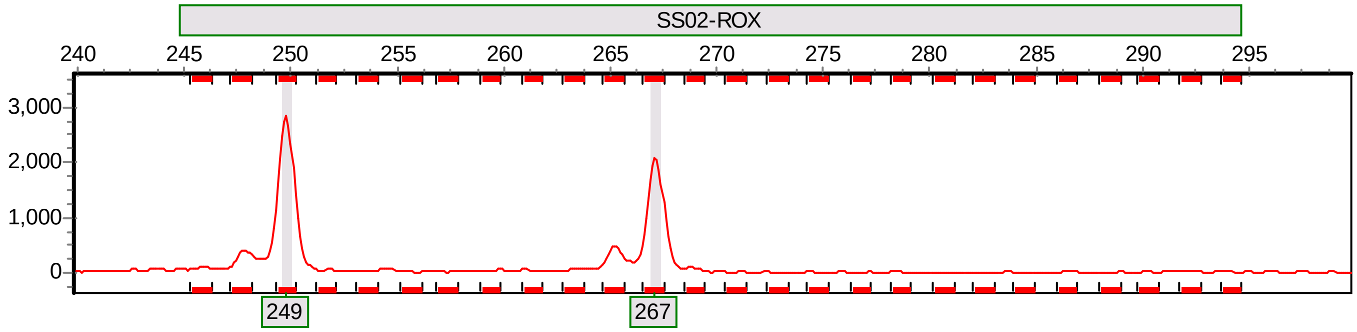

No	Size	Height	Area	Marker	Allele	Difference	Quality	Score	Allele Comments	Sample Comments
1	249.8	2848	23857	SS02-ROX	249	0.00	Pass	386.5	[<Confirmed>]	
2	267.1	2078	17403	SS02-ROX	267	0.00	Pass	237.1	[<Confirmed>]	

Allele Report

Sample 46: SSS13_SS20_SS11_SS21_SS02_SS19_HBB9_I05.fsa

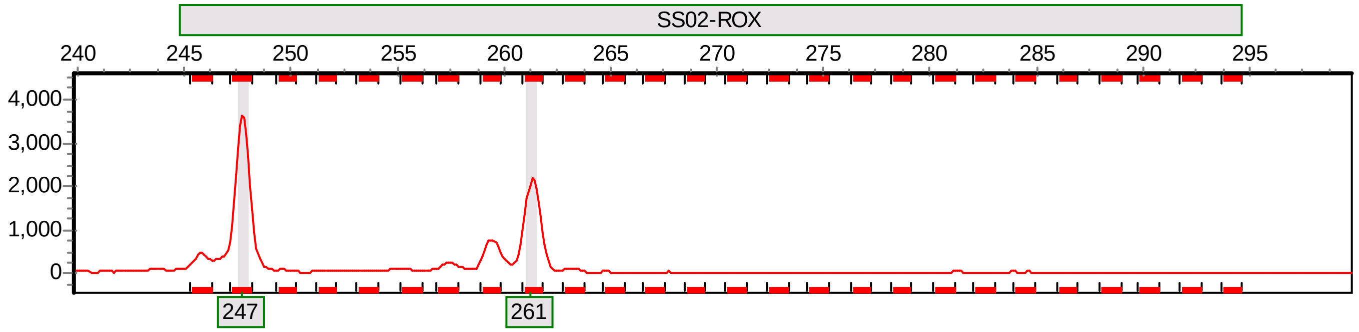

No	Size	Height	Area	Marker	Allele	Difference	Quality	Score	Allele Comments	Sample Comments
1	247.7	3629	29233	SS02-ROX	247	0.00	Pass	500.0	[<Confirmed>]	
2	261.3	2185	18148	SS02-ROX	261	0.10	Pass	236.9	[<Confirmed>]	

Sample 47: SSS13_SS20_SS11_SS21_SS02_SS19_HBN10_G11.fsa

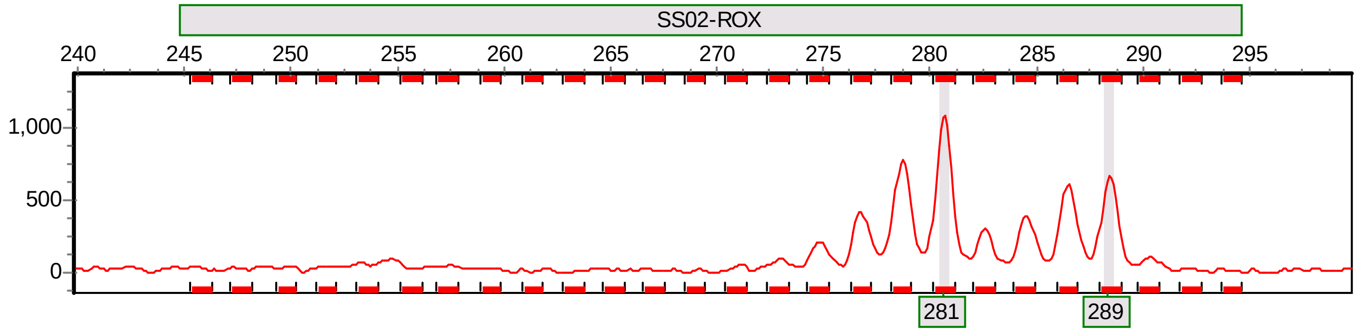

No	Size	Height	Area	Marker	Allele	Difference	Quality	Score	Allele Comments	Sample Comments
1	280.7	1076	9548	SS02-ROX	281	0.00	Pass	81.3	[<Confirmed>]	
2	286.5	612	5164	SS02-ROX	287	0.00	Undetermined	34.4	[<Deleted>]	
3	288.4	660	6001	SS02-ROX	289	0.10	Pass	34.8	[<Confirmed>]	

Sample 48: SSS13_SS20_SS11_SS21_SS02_SS19_HBN6_O01.fsa

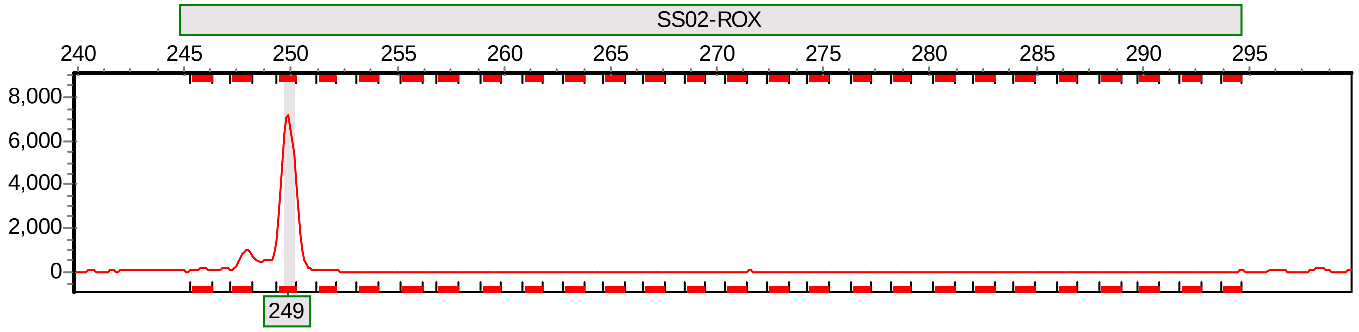

No	Size	Height	Area	Marker	Allele	Difference	Quality	Score	Allele Comments	Sample Comments
1	249.9	7122	52824	SS02-ROX	249	0.10	Pass	500.0	[<Confirmed>]	

Sample 49: SSS13_SS20_SS11_SS21_SS02_SS19_HBN9_D03.fsa

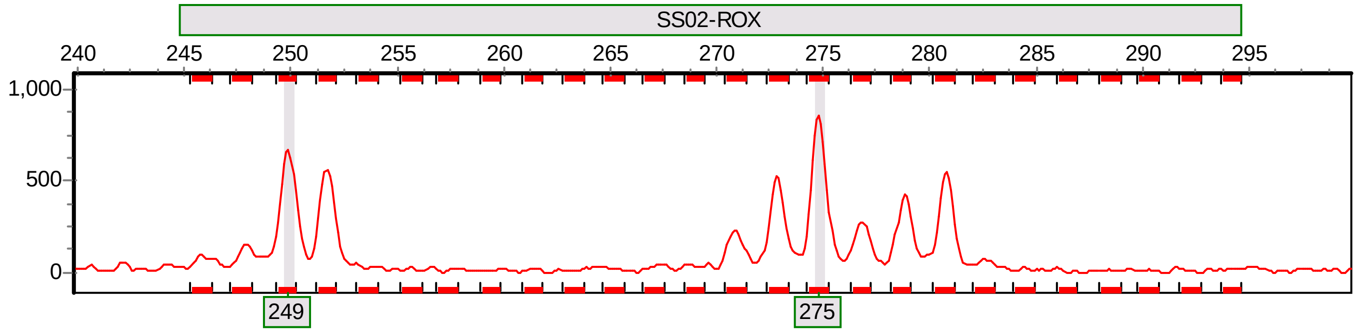

No	Size	Height	Area	Marker	Allele	Difference	Quality	Score	Allele Comments	Sample Comments
1	249.9	665	5584	SS02-ROX	249	0.10	Pass	42.4	[<Confirmed>]	
2	251.7	558	4643	SS02-ROX	251	0.00	Undetermined	32.8	[<Deleted>]	
3	274.8	853	7132	SS02-ROX	275	0.00	Pass	64.0	[<Confirmed>]	
4	280.8	544	4510	SS02-ROX	281	0.10	Undetermined	30.6	[<Deleted>]	

Sample 50: SSS13_SS20_SS11_SS21_SS02_SS19_HCW1_M09.fsa

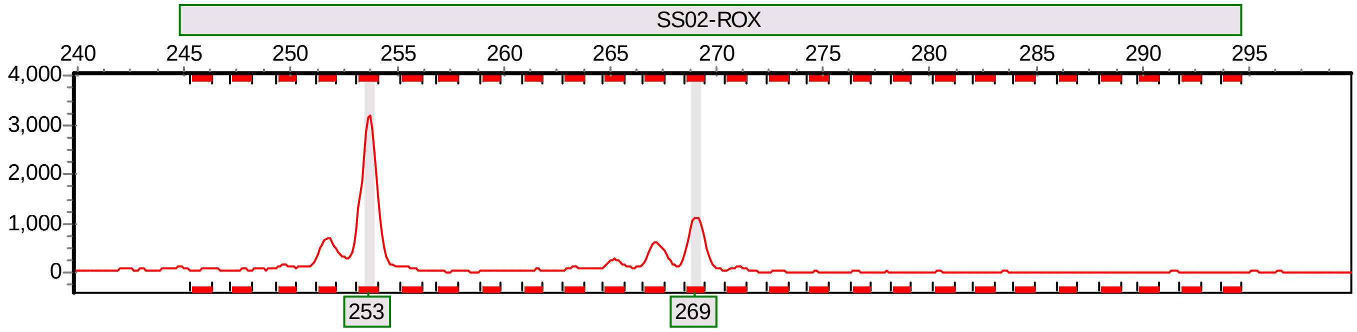

No	Size	Height	Area	Marker	Allele	Difference	Quality	Score	Allele Comments	Sample Comments
1	253.7	3183	25820	SS02-ROX	253	0.10	Pass	455.2	[<Confirmed>]	
2	269.0	1131	9495	SS02-ROX	269	0.00	Pass	97.0	[<Confirmed>]	

Sample 51: SSS13_SS20_SS11_SS21_SS02_SS19_HCW2_A11.fsa

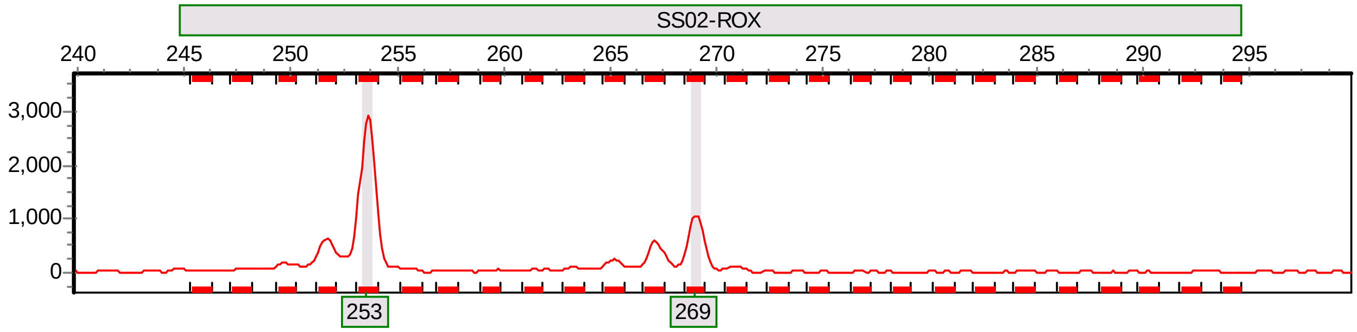

No	Size	Height	Area	Marker	Allele	Difference	Quality	Score	Allele Comments	Sample Comments
1	253.6	2909	23875	SS02-ROX	253	0.00	Pass	407.9	[<Confirmed>]	
2	269.0	1057	8521	SS02-ROX	269	0.00	Pass	94.7	[<Confirmed>]	

Allele Report

Sample 52: SSS13_SS20_SS11_SS21_SS02_SS19_HCW3_G13.fsa

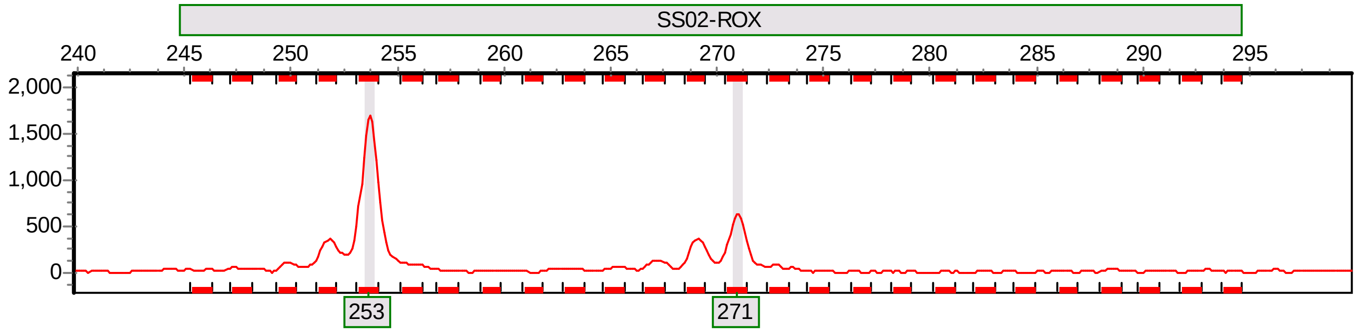

No	Size	Height	Area	Marker	Allele	Difference	Quality	Score	Allele Comments	Sample Comments
1	253.7	1693	14773	SS02-ROX	253	0.10	Pass	157.9	[<Confirmed>]	
2	271.0	634	5828	SS02-ROX	271	0.10	Pass	31.2	[<Confirmed>]	

Sample 53: SSS13_SS20_SS11_SS21_SS02_SS19_HCW4_E09.fsa

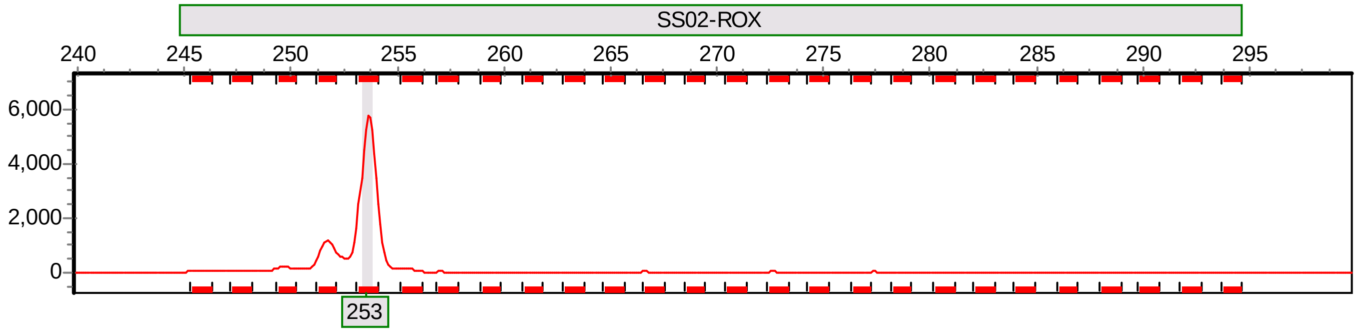

No	Size	Height	Area	Marker	Allele	Difference	Quality	Score	Allele Comments	Sample Comments
1	253.6	5733	47271	SS02-ROX	253	0.00	Pass	500.0	[<Confirmed>]	

Sample 54: SSS13_SS20_SS11_SS21_SS02_SS19_HCW5_C11.fsa

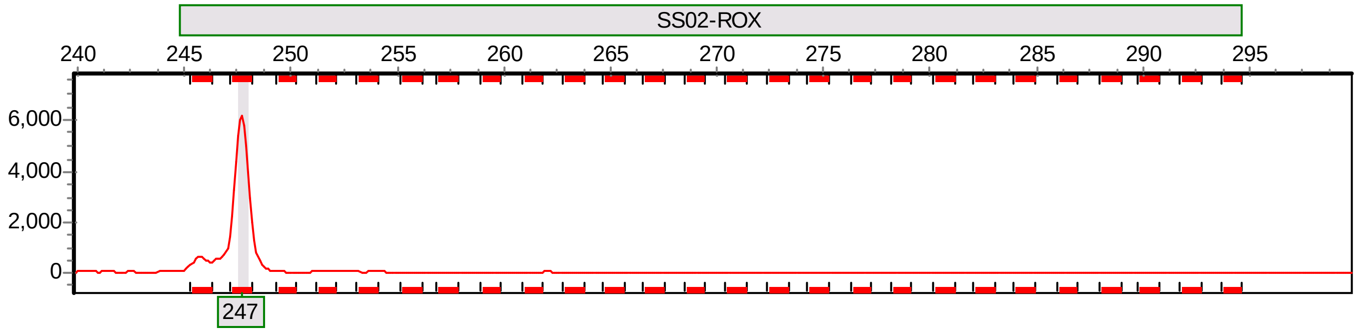

No	Size	Height	Area	Marker	Allele	Difference	Quality	Score	Allele Comments	Sample Comments
1	247.7	6161	49128	SS02-ROX	247	0.00	Pass	500.0	[<Confirmed>]	

Allele Report

Sample 55: SSS13_SS20_SS11_SS21_SS02_SS19_HCW6_D01.fsa

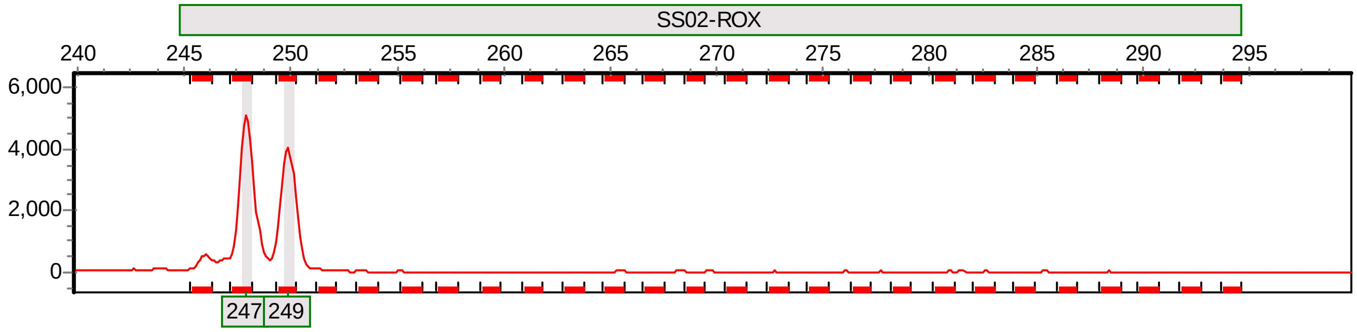

No	Size	Height	Area	Marker	Allele	Difference	Quality	Score	Allele Comments	Sample Comments
1	247.9	5071	39719	SS02-ROX	247	0.20	Pass	500.0	[<Confirmed>]	
2	249.9	4023	31636	SS02-ROX	249	0.10	Pass	500.0	[<Confirmed>]	

Sample 56: SSS13_SS20_SS11_SS21_SS02_SS19_HCW7_G17.fsa

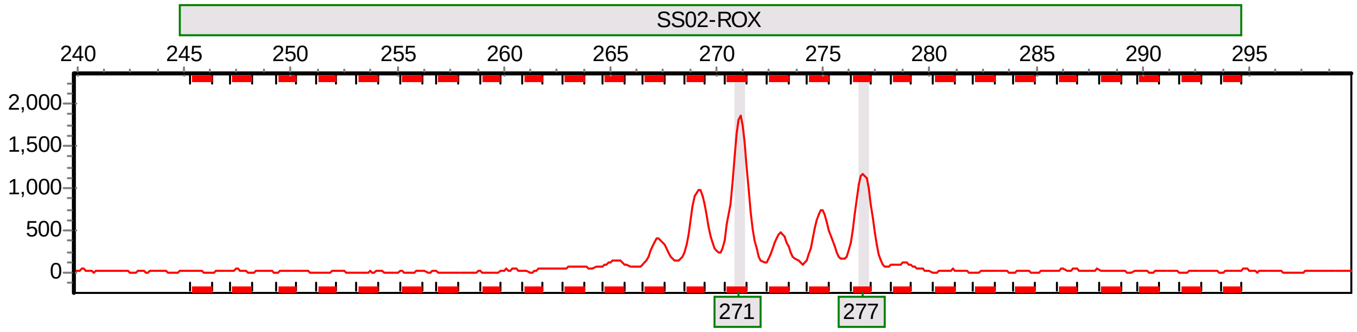

No	Size	Height	Area	Marker	Allele	Difference	Quality	Score	Allele Comments	Sample Comments
1	271.1	1842	16040	SS02-ROX	271	0.20	Pass	190.5	[<Confirmed>]	
2	276.9	1172	10321	SS02-ROX	277	0.10	Pass	95.1	[<Confirmed>]	

Sample 57: SSS13_SS20_SS11_SS21_SS02_SS19_HCW8_A17.fsa

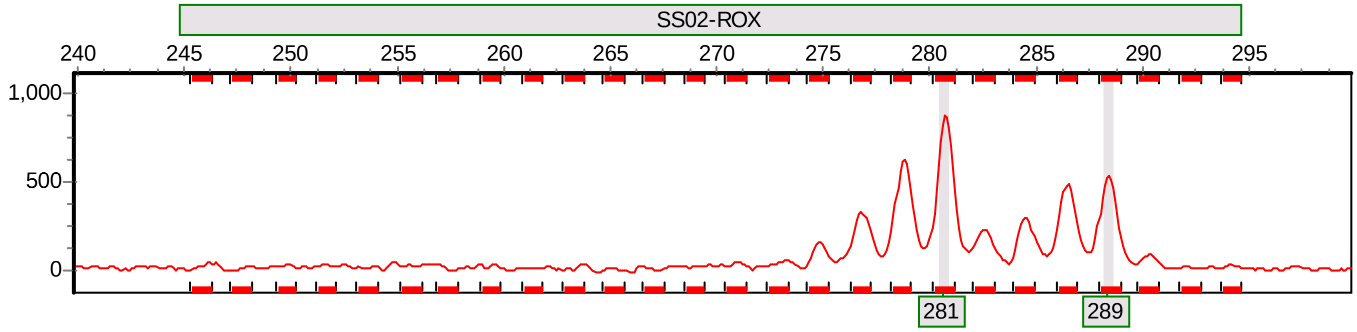

No	Size	Height	Area	Marker	Allele	Difference	Quality	Score	Allele Comments	Sample Comments
1	280.7	871	7832	SS02-ROX	281	0.00	Pass	51.3	[<Confirmed>]	
2	288.4	537	4759	SS02-ROX	289	0.10	Pass	23.6	[<Confirmed>]	

Allele Report

Sample 58: SSS13_SS20_SS11_SS21_SS02_SS19_HGC1_C01.fsa

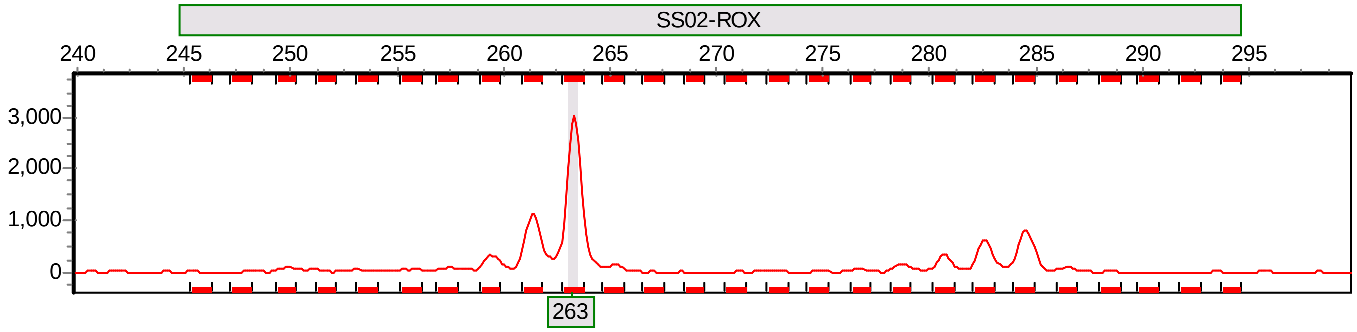

No	Size	Height	Area	Marker	Allele	Difference	Quality	Score	Allele Comments	Sample Comments
1	263.3	3006	23947	SS02-ROX	263	0.00	Pass	458.2	[<Confirmed>]	

Sample 59: SSS13_SS20_SS11_SS21_SS02_SS19_HGC3_E17.fsa

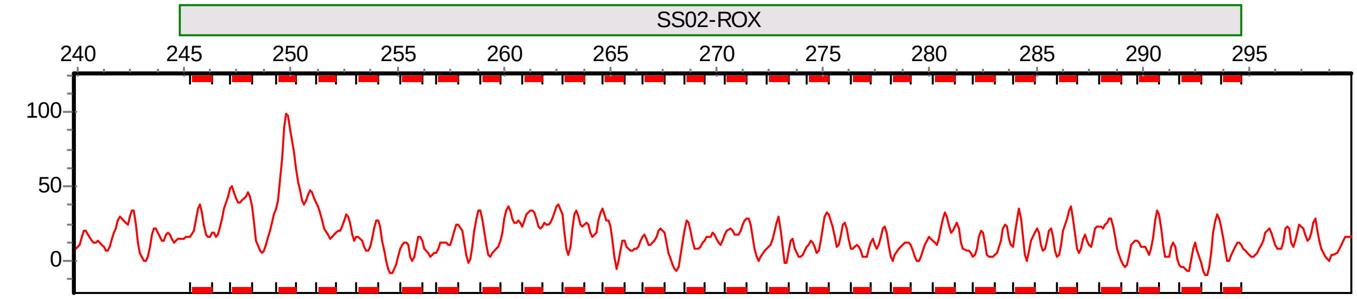

No	Size	Height	Area	Marker	Allele	Difference	Quality	Score	Allele Comments	Sample Comments

Sample 60: SSS13_SS20_SS11_SS21_SS02_SS19_HGC4_A13.fsa

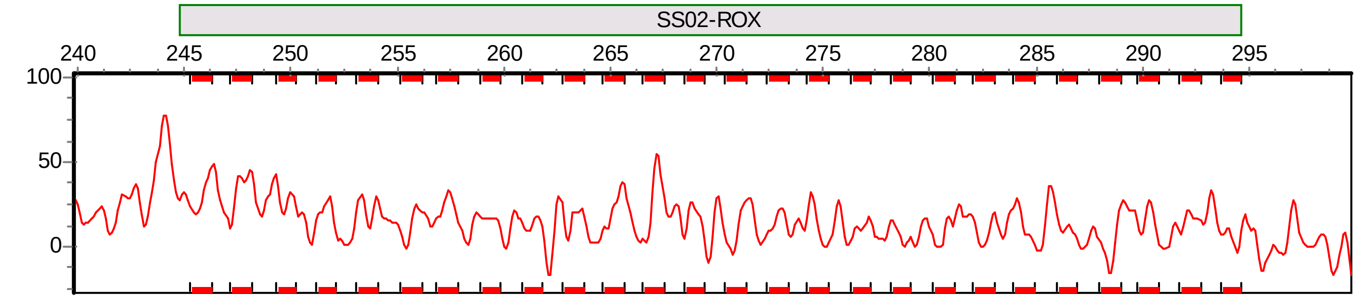

No	Size	Height	Area	Marker	Allele	Difference	Quality	Score	Allele Comments	Sample Comments

Sample 61: SSS13_SS20_SS11_SS21_SS02_SS19_HGY1_A09.fsa

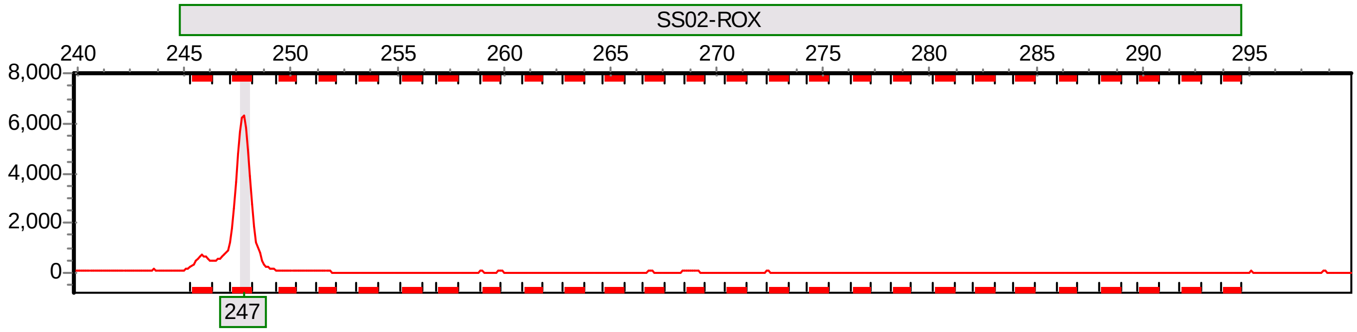

No	Size	Height	Area	Marker	Allele	Difference	Quality	Score	Allele Comments	Sample Comments

1	247.8	6288	51302	SS02-ROX	247	0.10	Pass	500.0	[<Confirmed>]	

Sample 62: SSS13_SS20_SS11_SS21_SS02_SS19_HGY2_I13.fsa

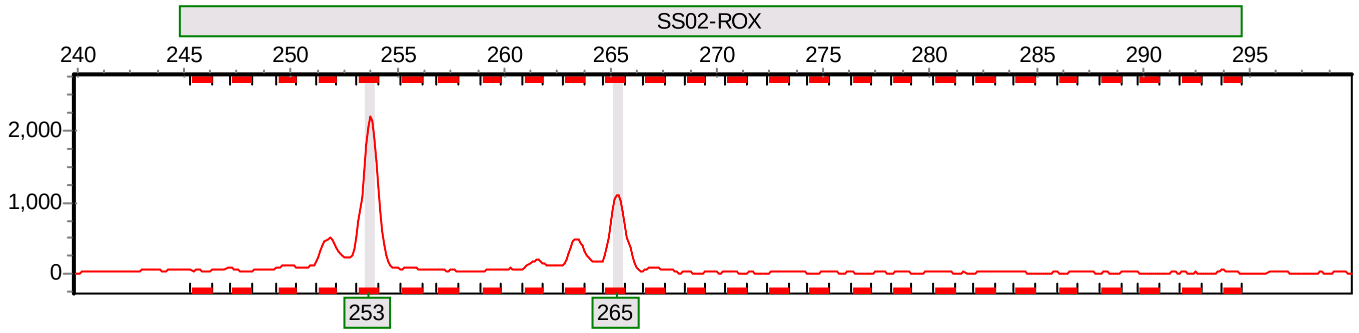

No	Size	Height	Area	Marker	Allele	Difference	Quality	Score	Allele Comments	Sample Comments
1	253.7	2194	18233	SS02-ROX	253	0.10	Pass	274.5	[<Confirmed>]	
2	265.3	1107	9300	SS02-ROX	265	0.10	Pass	90.7	[<Confirmed>]	

Sample 63: SSS13_SS20_SS11_SS21_SS02_SS19_HGY3_I11.fsa

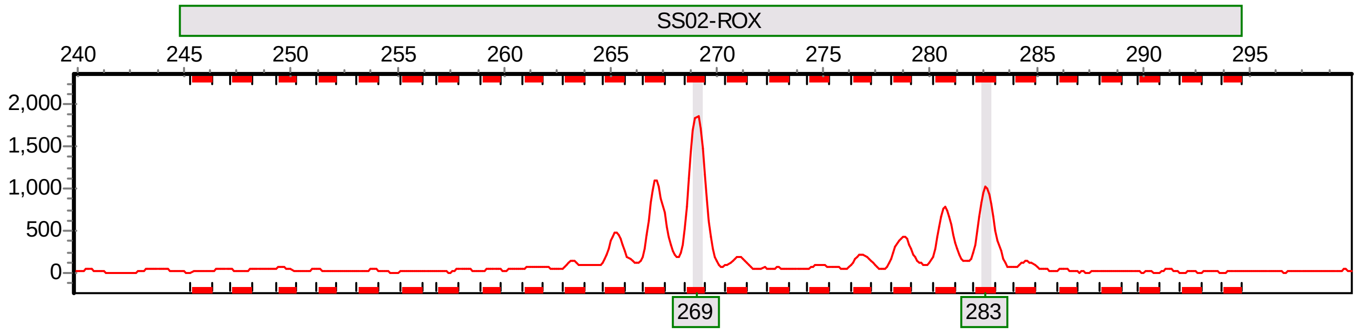

No	Size	Height	Area	Marker	Allele	Difference	Quality	Score	Allele Comments	Sample Comments
1	269.1	1858	15146	SS02-ROX	269	0.10	Pass	206.2	[<Confirmed>]	
2	282.6	1013	8754	SS02-ROX	283	0.00	Pass	74.8	[<Confirmed>]	

Sample 64: SSS13_SS20_SS11_SS21_SS02_SS19_HGY4_I09.fsa

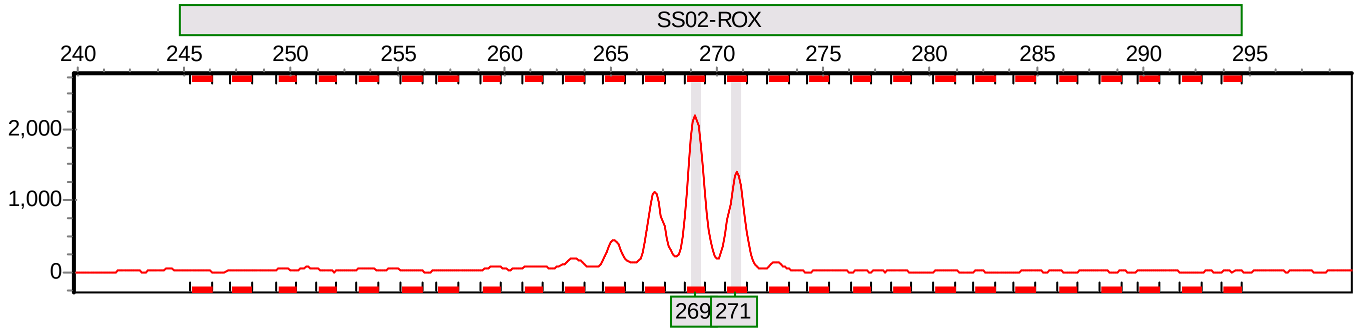

No	Size	Height	Area	Marker	Allele	Difference	Quality	Score	Allele Comments	Sample Comments
1	269.0	2183	18028	SS02-ROX	269	0.00	Pass	277.5	[<Confirmed>]	
2	270.9	1396	11918	SS02-ROX	271	0.00	Pass	135.7	[<Confirmed>]	

Allele Report

Sample 65: SSS13_SS20_SS11_SS21_SS02_SS19_HGY5_I15.fsa

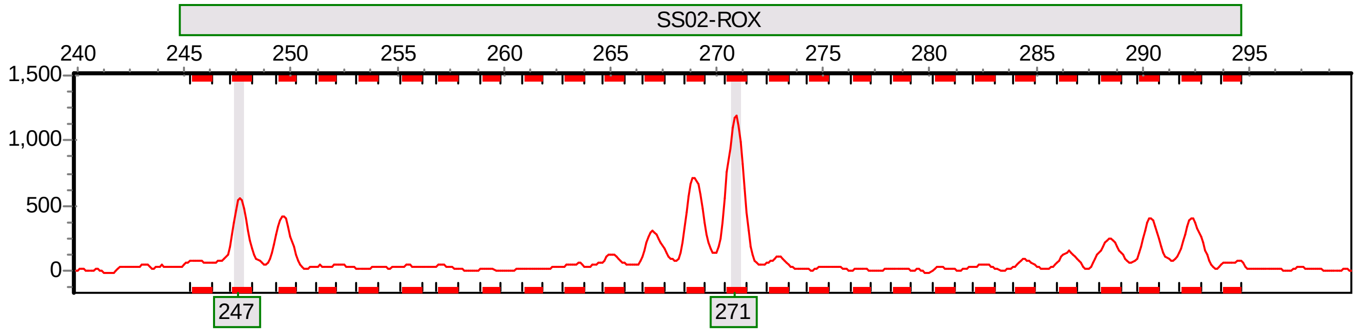

No	Size	Height	Area	Marker	Allele	Difference	Quality	Score	Allele Comments	Sample Comments
1	247.6	565	4688	SS02-ROX	247	0.10	Pass	34.4	[<Confirmed>]	
2	270.9	1183	10619	SS02-ROX	271	0.00	Pass	85.0	[<Confirmed>]	

Sample 66: SSS13_SS20_SS11_SS21_SS02_SS19_HQZ11_O11.fsa

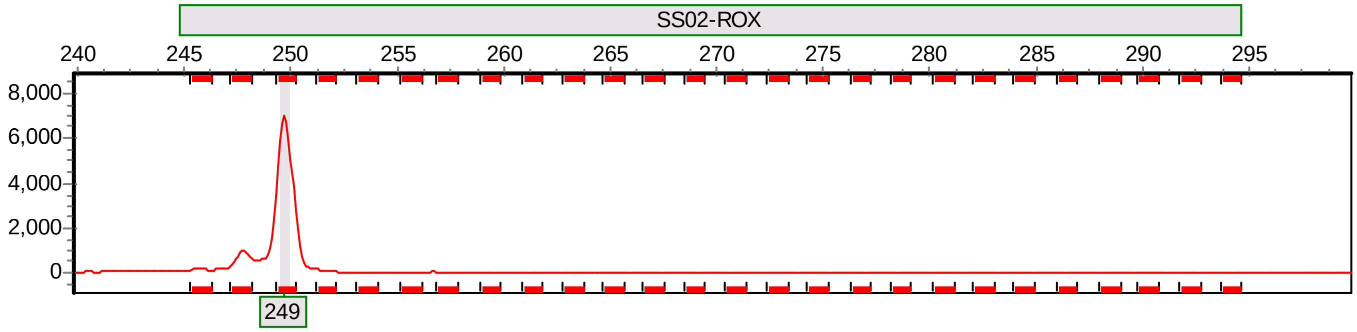

No	Size	Height	Area	Marker	Allele	Difference	Quality	Score	Allele Comments	Sample Comments
1	249.7	6974	57765	SS02-ROX	249	0.10	Pass	500.0	[<Confirmed>]	

Sample 67: SSS13_SS20_SS11_SS21_SS02_SS19_HQZ13-1_C09.fsa

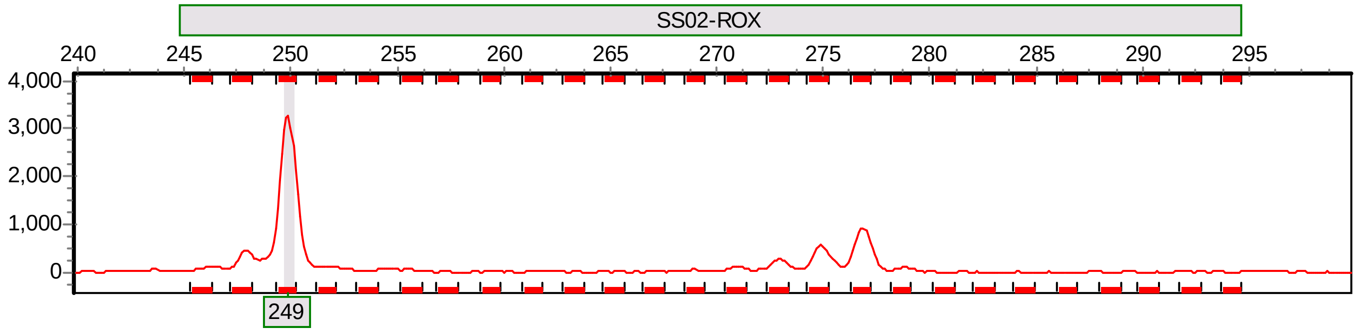

No	Size	Height	Area	Marker	Allele	Difference	Quality	Score	Allele Comments	Sample Comments
1	249.9	3251	27649	SS02-ROX	249	0.10	Pass	449.6	[<Confirmed>]	

Allele Report

Sample 68: SSS13_SS20_SS11_SS21_SS02_SS19_HQZ13-2_J03.fsa

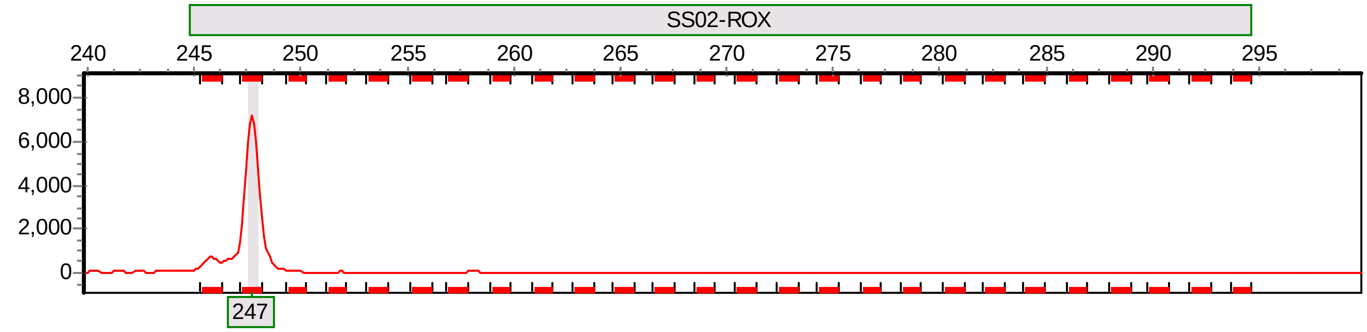

No	Size	Height	Area	Marker	Allele	Difference	Quality	Score	Allele Comments	Sample Comments
1	247.7	7160	56269	SS02-ROX	247	0.00	Pass	500.0	[<Confirmed>]	

Sample 69: SSS13_SS20_SS11_SS21_SS02_SS19_HQZ14_H03.fsa

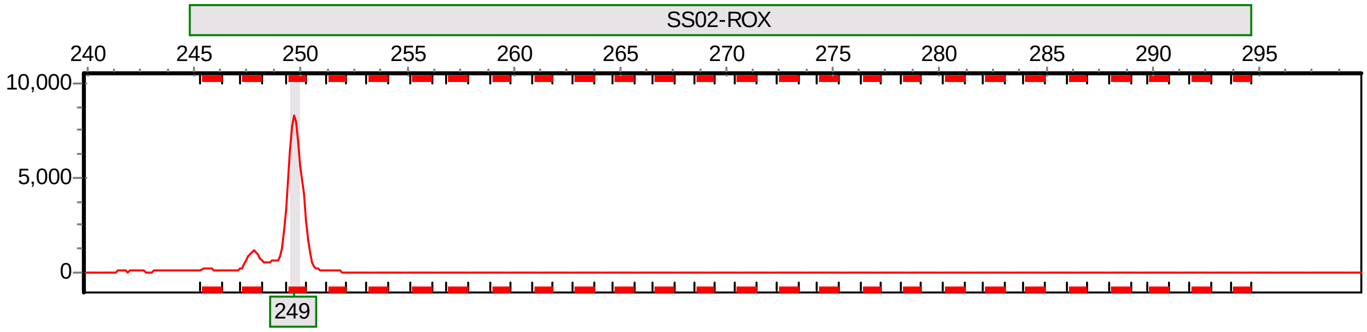

No	Size	Height	Area	Marker	Allele	Difference	Quality	Score	Allele Comments	Sample Comments
1	249.7	8230	61504	SS02-ROX	249	0.10	Pass	500.0	[<Confirmed>]	

Sample 70: SSS13_SS20_SS11_SS21_SS02_SS19_HQZ15_B01.fsa

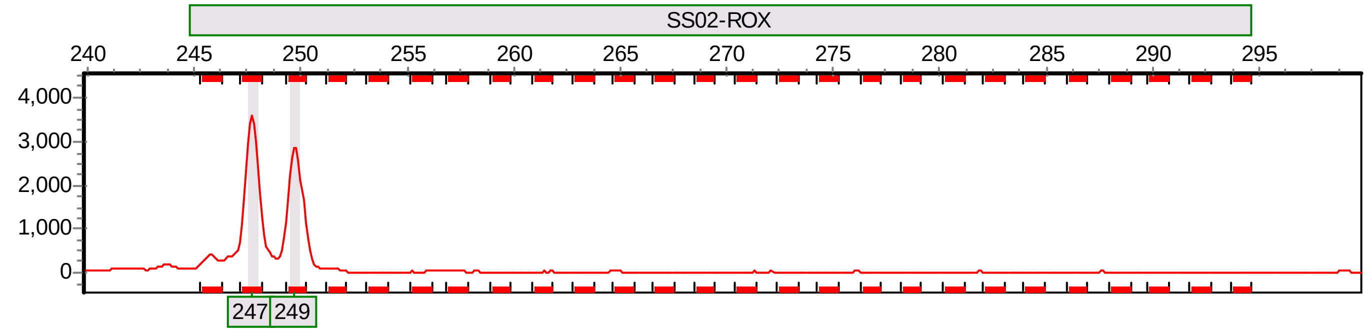

No	Size	Height	Area	Marker	Allele	Difference	Quality	Score	Allele Comments	Sample Comments
1	247.7	3577	28179	SS02-ROX	247	0.00	Pass	500.0	[<Confirmed>]	
2	249.7	2875	22641	SS02-ROX	249	0.10	Pass	448.5	[<Confirmed>]	

Allele Report

Sample 71: SSS13_SS20_SS11_SS21_SS02_SS19_HQZ16_P01.fsa

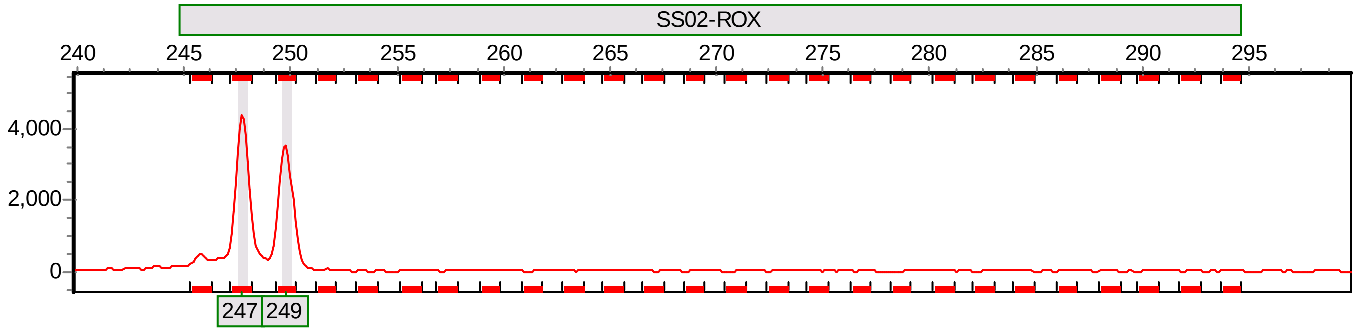

No	Size	Height	Area	Marker	Allele	Difference	Quality	Score	Allele Comments	Sample Comments
1	247.7	4372	32872	SS02-ROX	247	0.00	Pass	500.0	[<Confirmed>]	
2	249.8	3560	26556	SS02-ROX	249	0.00	Pass	500.0	[<Confirmed>]	

Sample 72: SSS13_SS20_SS11_SS21_SS02_SS19_HQZ17-1_O03.fsa

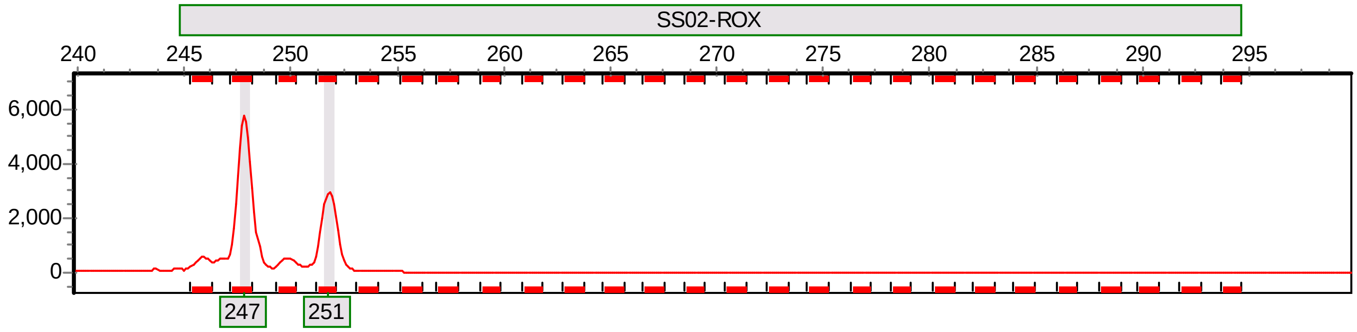

No	Size	Height	Area	Marker	Allele	Difference	Quality	Score	Allele Comments	Sample Comments
1	247.8	5714	44988	SS02-ROX	247	0.10	Pass	500.0	[<Confirmed>]	
2	251.8	2969	23754	SS02-ROX	251	0.10	Pass	439.2	[<Confirmed>]	

Sample 73: SSS13_SS20_SS11_SS21_SS02_SS19_HQZ17-2_N03.fsa

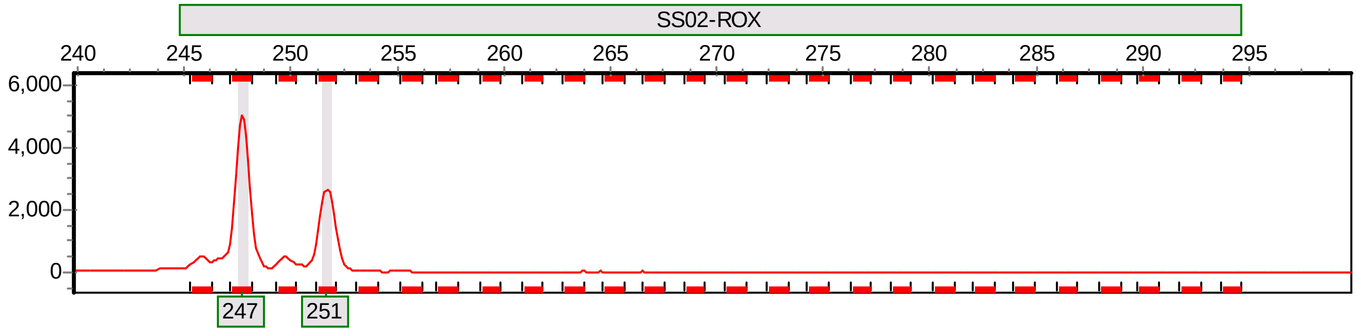

No	Size	Height	Area	Marker	Allele	Difference	Quality	Score	Allele Comments	Sample Comments
1	247.7	5028	39577	SS02-ROX	247	0.00	Pass	500.0	[<Confirmed>]	
2	251.7	2682	22018	SS02-ROX	251	0.00	Pass	369.5	[<Confirmed>]	

Sample 74: SSS13_SS20_SS11_SS21_SS02_SS19_HQZ18_K09.fsa

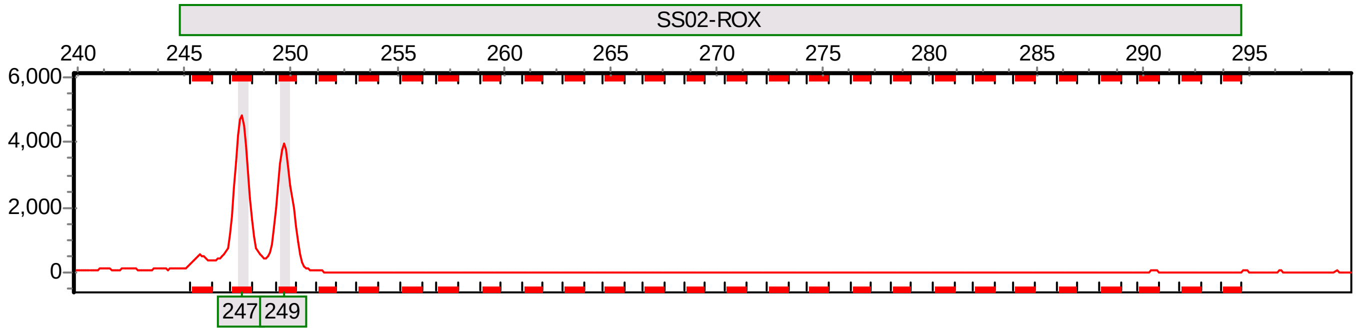

No	Size	Height	Area	Marker	Allele	Difference	Quality	Score	Allele Comments	Sample Comments
1	247.7	4800	39044	SS02-ROX	247	0.00	Pass	500.0	[<Confirmed>]	
2	249.7	3949	31820	SS02-ROX	249	0.10	Pass	500.0	[<Confirmed>]	

Sample 75: SSS13_SS20_SS11_SS21_SS02_SS19_HQZ19_L03.fsa

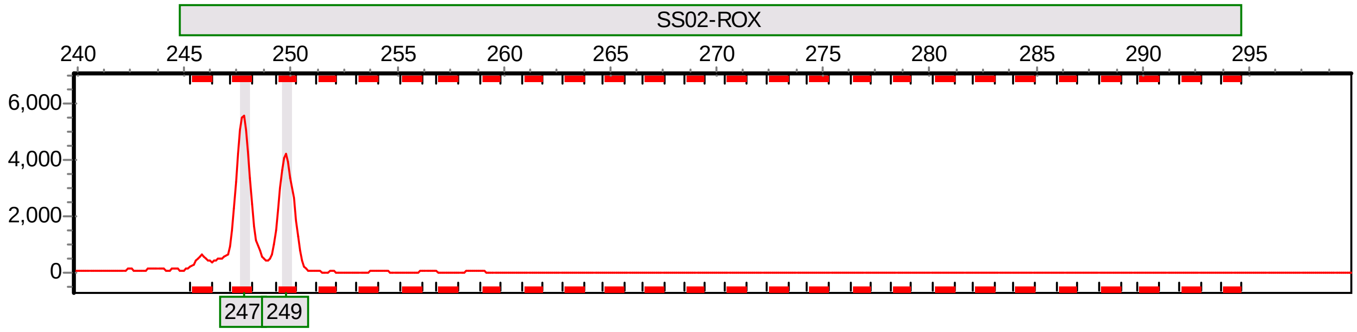

No	Size	Height	Area	Marker	Allele	Difference	Quality	Score	Allele Comments	Sample Comments
1	247.8	5564	44365	SS02-ROX	247	0.10	Pass	500.0	[<Confirmed>]	
2	249.8	4200	33078	SS02-ROX	249	0.00	Pass	500.0	[<Confirmed>]	

Sample 76: SSS13_SS20_SS11_SS21_SS02_SS19_HQZ21_B03.fsa

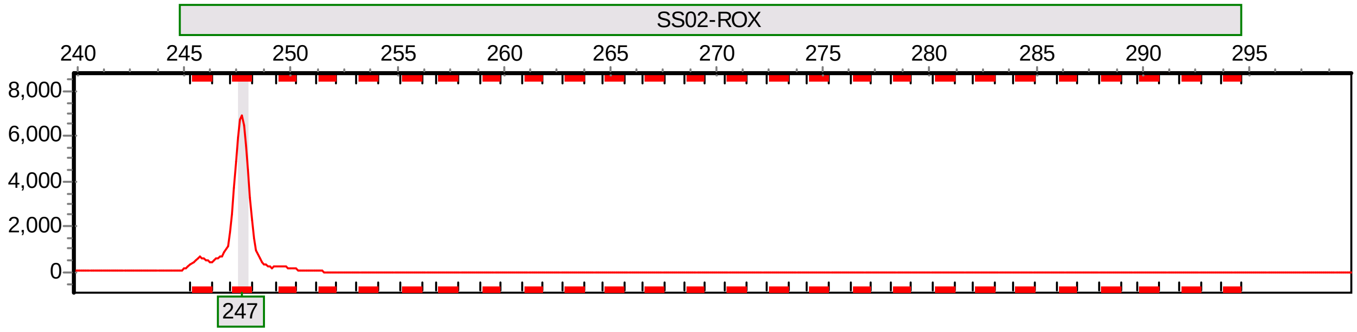

No	Size	Height	Area	Marker	Allele	Difference	Quality	Score	Allele Comments	Sample Comments
1	247.7	6896	56090	SS02-ROX	247	0.00	Pass	500.0	[<Confirmed>]	

Sample 77: SSS13_SS20_SS11_SS21_SS02_SS19_HQZ22-1_M01.fsa

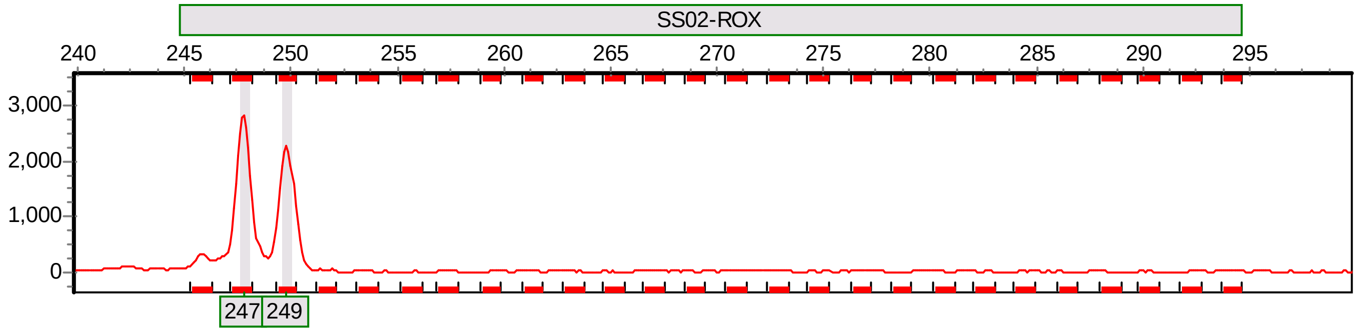

No	Size	Height	Area	Marker	Allele	Difference	Quality	Score	Allele Comments	Sample Comments
1	247.8	2806	22500	SS02-ROX	247	0.10	Pass	399.3	[<Confirmed>]	
2	249.8	2272	18565	SS02-ROX	249	0.00	Pass	299.7	[<Confirmed>]	

Sample 78: SSS13_SS20_SS11_SS21_SS02_SS19_HQZ22-2_O13.fsa

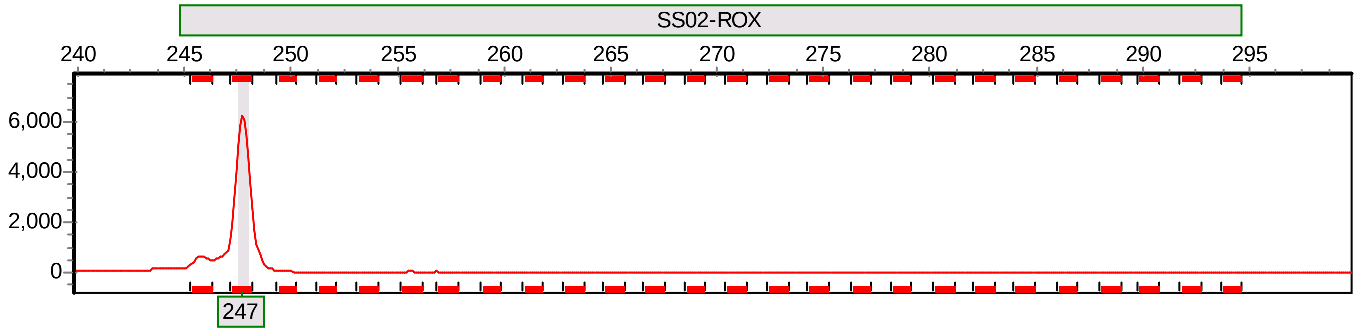

No	Size	Height	Area	Marker	Allele	Difference	Quality	Score	Allele Comments	Sample Comments
1	247.7	6227	50103	SS02-ROX	247	0.00	Pass	500.0	[<Confirmed>]	

Sample 79: SSS13_SS20_SS11_SS21_SS02_SS19_HQZ23_A15.fsa

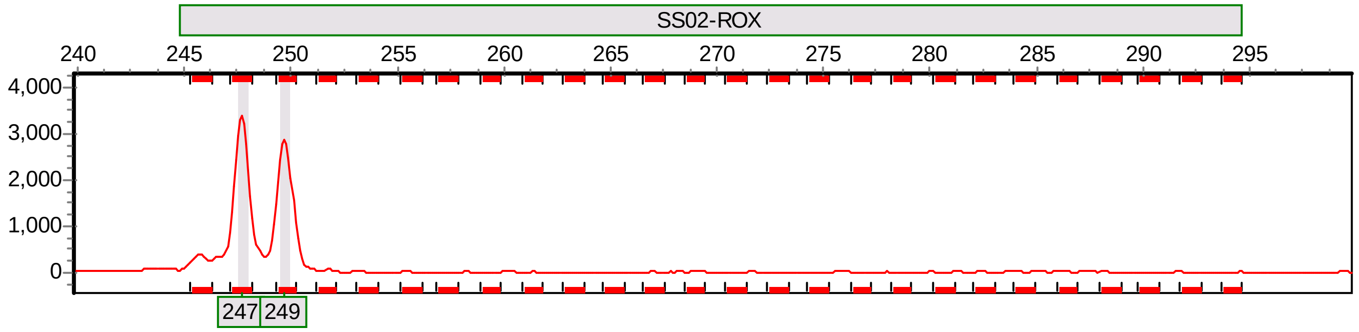

No	Size	Height	Area	Marker	Allele	Difference	Quality	Score	Allele Comments	Sample Comments
1	247.7	3368	28134	SS02-ROX	247	0.00	Pass	497.3	[<Confirmed>]	
2	249.7	2877	23933	SS02-ROX	249	0.10	Pass	396.8	[<Confirmed>]	

Sample 80: SSS13_SS20_SS11_SS21_SS02_SS19_HQZ24_E11.fsa

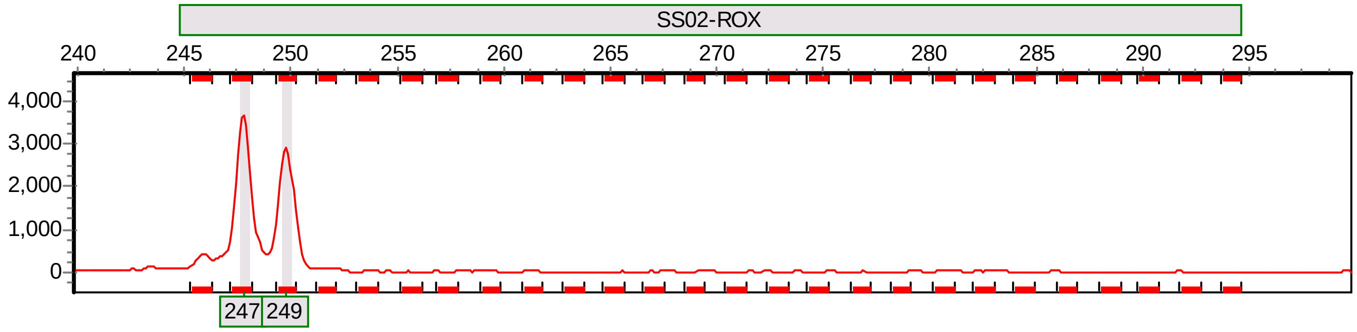

No	Size	Height	Area	Marker	Allele	Difference	Quality	Score	Allele Comments	Sample Comments
1	247.8	3635	29723	SS02-ROX	247	0.10	Pass	500.0	[<Confirmed>]	
2	249.8	2896	24300	SS02-ROX	249	0.00	Pass	401.0	[<Confirmed>]	

Sample 81: SSS13_SS20_SS11_SS21_SS02_SS19_HQZ25_N01.fsa

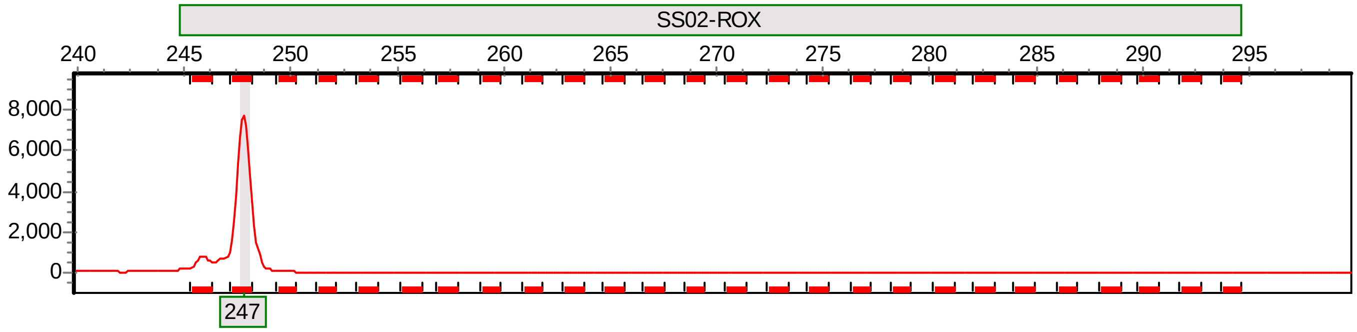

No	Size	Height	Area	Marker	Allele	Difference	Quality	Score	Allele Comments	Sample Comments
1	247.8	7658	58789	SS02-ROX	247	0.10	Pass	500.0	[<Confirmed>]	

Sample 82: SSS13_SS20_SS11_SS21_SS02_SS19_HQZ26_E03.fsa

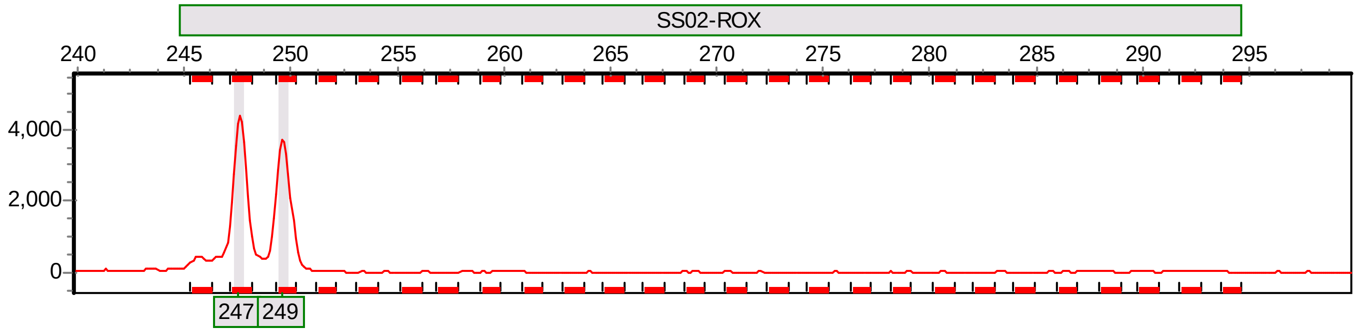

No	Size	Height	Area	Marker	Allele	Difference	Quality	Score	Allele Comments	Sample Comments
1	247.6	4357	33644	SS02-ROX	247	0.10	Pass	500.0	[<Confirmed>]	
2	249.6	3714	28907	SS02-ROX	249	0.20	Pass	500.0	[<Confirmed>]	

Allele Report

Sample 83: SSS13_SS20_SS11_SS21_SS02_SS19_HQZ27_G09.fsa

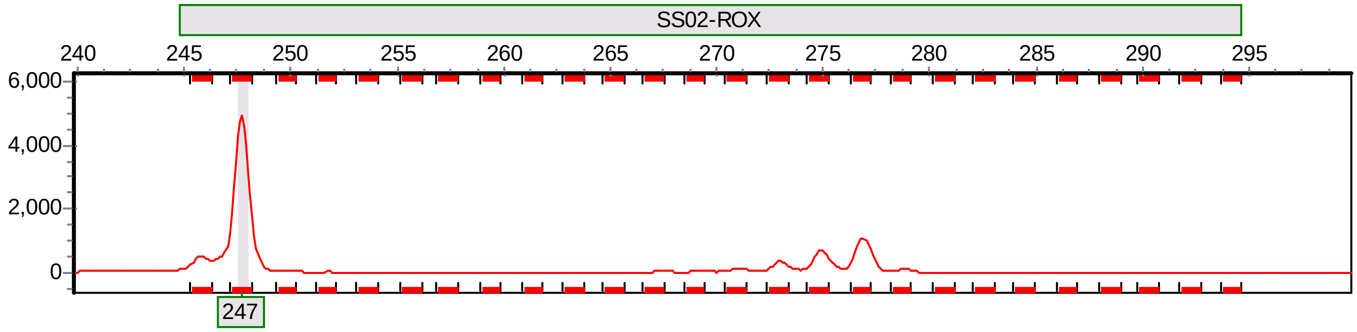

No	Size	Height	Area	Marker	Allele	Difference	Quality	Score	Allele Comments	Sample Comments
1	247.7	4897	40382	SS02-ROX	247	0.00	Pass	500.0	[<Confirmed>]	

Sample 84: SSS13_SS20_SS11_SS21_SS02_SS19_HQZ28_B05.fsa

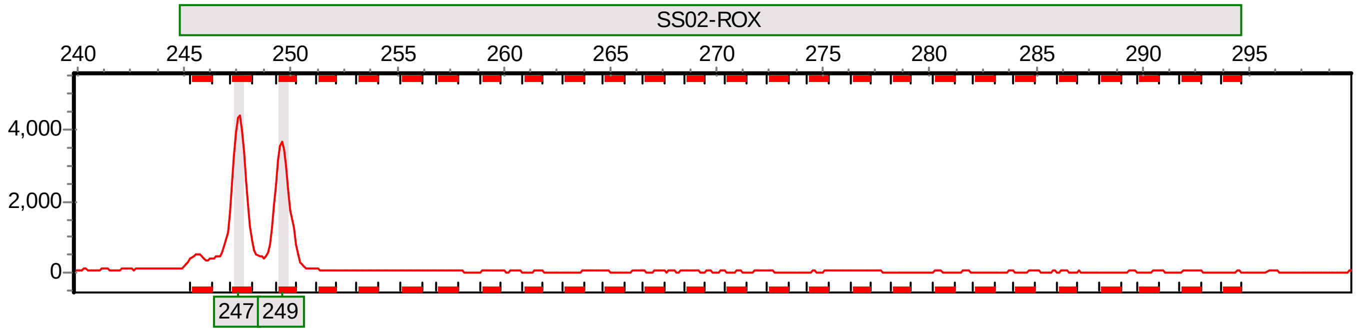

No	Size	Height	Area	Marker	Allele	Difference	Quality	Score	Allele Comments	Sample Comments
1	247.6	4401	34590	SS02-ROX	247	0.10	Pass	500.0	[<Confirmed>]	
2	249.6	3692	28989	SS02-ROX	249	0.20	Pass	500.0	[<Confirmed>]	

Sample 85: SSS13_SS20_SS11_SS21_SS02_SS19_HQZ29_C13.fsa

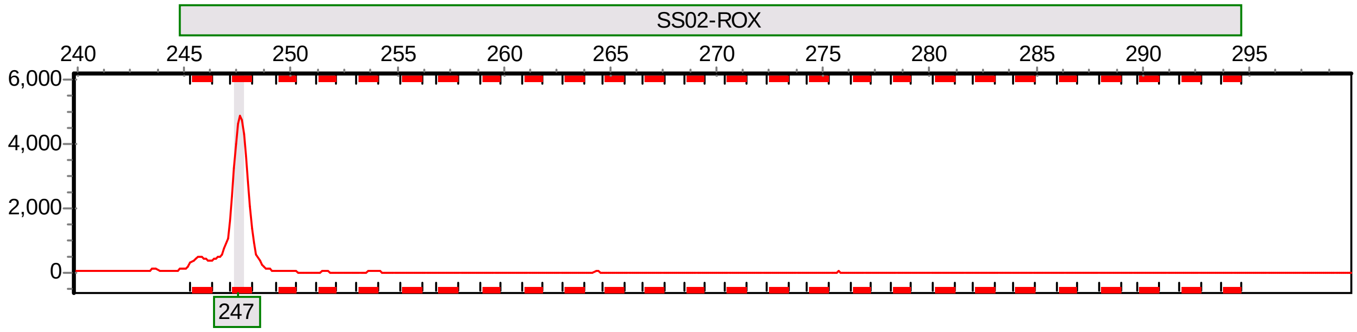

No	Size	Height	Area	Marker	Allele	Difference	Quality	Score	Allele Comments	Sample Comments
1	247.6	4829	40900	SS02-ROX	247	0.10	Pass	500.0	[<Confirmed>]	

Allele Report

Sample 86: SSS13_SS20_SS11_SS21_SS02_SS19_HQZ2_K03.fsa

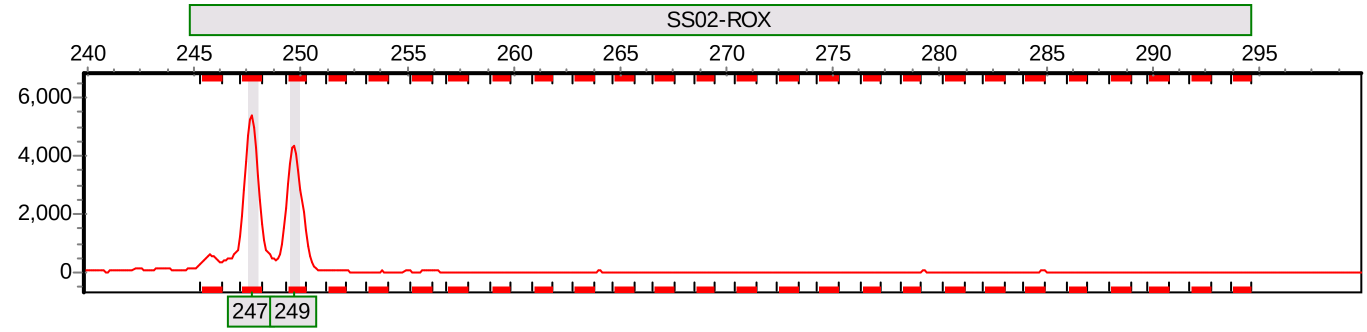

No	Size	Height	Area	Marker	Allele	Difference	Quality	Score	Allele Comments	Sample Comments
1	247.7	5374	41646	SS02-ROX	247	0.00	Pass	500.0	[<Confirmed>]	
2	249.7	4362	34502	SS02-ROX	249	0.10	Pass	500.0	[<Confirmed>]	

Sample 87: SSS13_SS20_SS11_SS21_SS02_SS19_HQZ30_G15.fsa

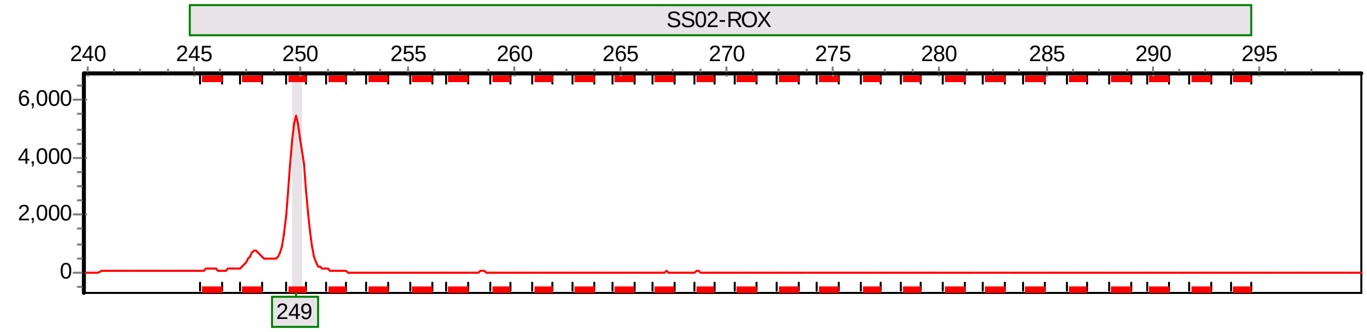

No	Size	Height	Area	Marker	Allele	Difference	Quality	Score	Allele Comments	Sample Comments
1	249.8	5432	45191	SS02-ROX	249	0.00	Pass	500.0	[<Confirmed>]	

Sample 88: SSS13_SS20_SS11_SS21_SS02_SS19_HQZ31_K13.fsa

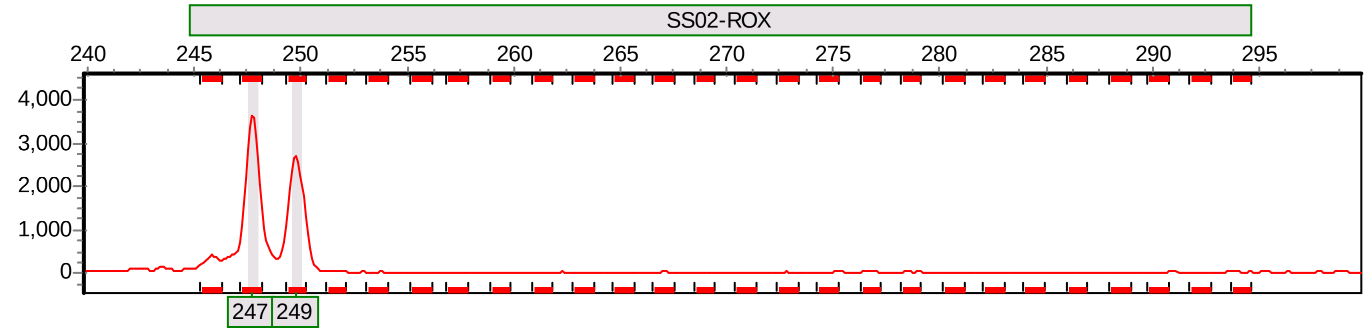

No	Size	Height	Area	Marker	Allele	Difference	Quality	Score	Allele Comments	Sample Comments
1	247.7	3612	28441	SS02-ROX	247	0.00	Pass	500.0	[<Confirmed>]	
2	249.8	2714	22300	SS02-ROX	249	0.00	Pass	371.4	[<Confirmed>]	

Allele Report

Sample 89: SSS13_SS20_SS11_SS21_SS02_SS19_HQZ32_J01.fsa

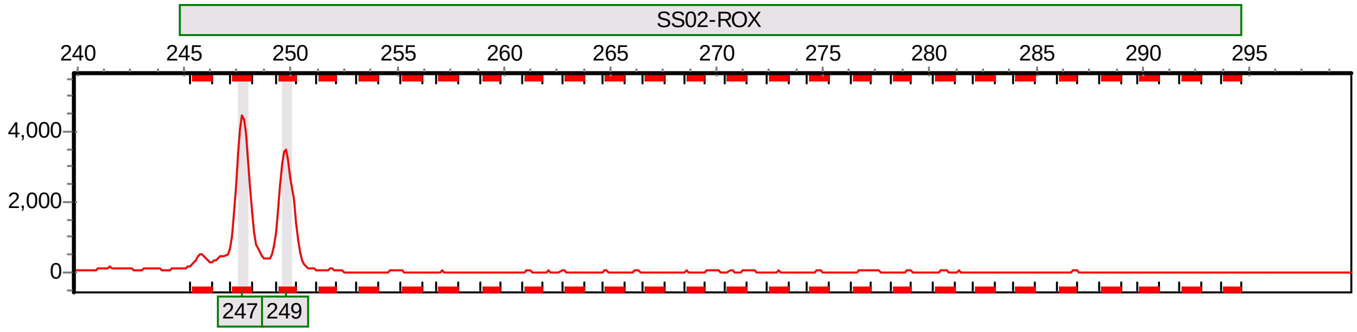

No	Size	Height	Area	Marker	Allele	Difference	Quality	Score	Allele Comments	Sample Comments
1	247.7	4414	33734	SS02-ROX	247	0.00	Pass	500.0	[<Confirmed>]	
2	249.8	3482	26112	SS02-ROX	249	0.00	Pass	500.0	[<Confirmed>]	

Sample 90: SSS13_SS20_SS11_SS21_SS02_SS19_HQZ33_I03.fsa

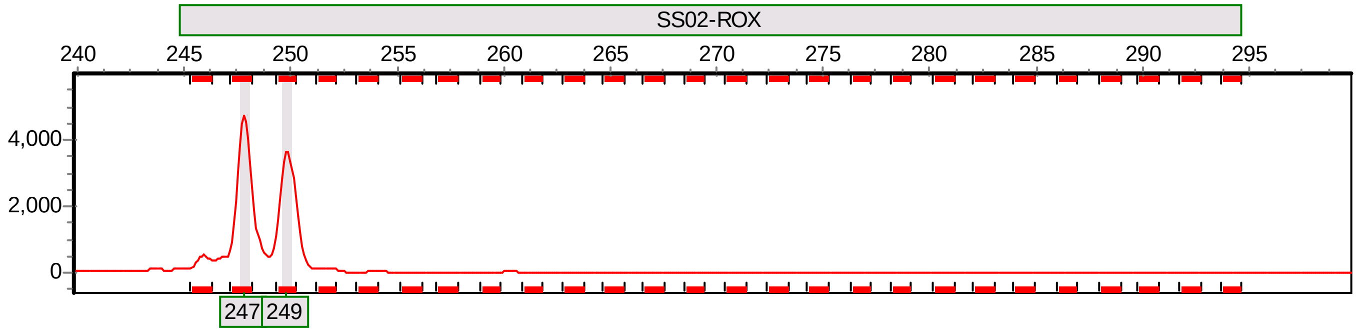

No	Size	Height	Area	Marker	Allele	Difference	Quality	Score	Allele Comments	Sample Comments
1	247.8	4668	37357	SS02-ROX	247	0.10	Pass	500.0	[<Confirmed>]	
2	249.8	3617	30121	SS02-ROX	249	0.00	Pass	500.0	[<Confirmed>]	

Sample 91: SSS13_SS20_SS11_SS21_SS02_SS19_HQZ34_M03.fsa

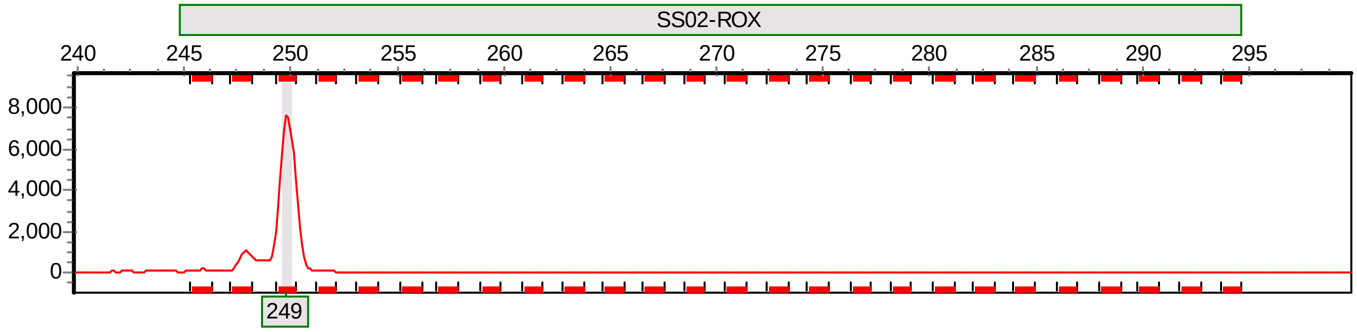

No	Size	Height	Area	Marker	Allele	Difference	Quality	Score	Allele Comments	Sample Comments
1	249.8	7573	60811	SS02-ROX	249	0.00	Pass	500.0	[<Confirmed>]	

Sample 92: SSS13_SS20_SS11_SS21_SS02_SS19_HQZ35_A01.fsa

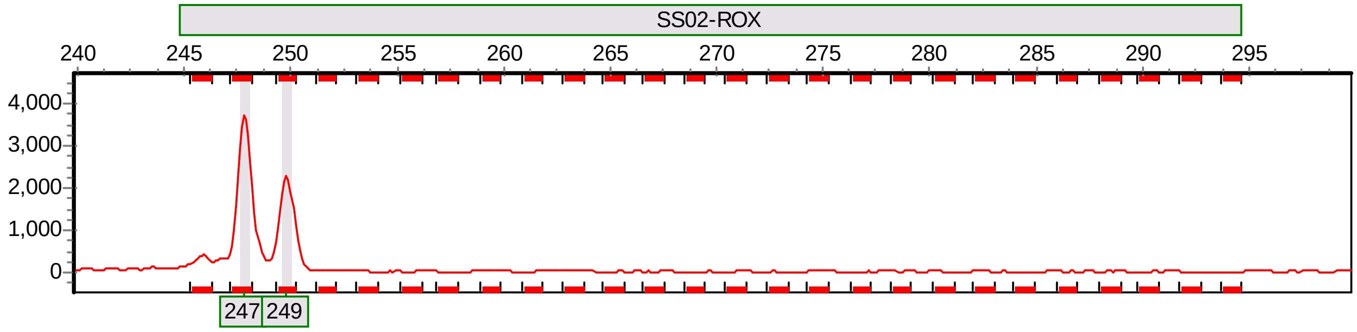

No	Size	Height	Area	Marker	Allele	Difference	Quality	Score	Allele Comments	Sample Comments
1	247.8	3680	29062	SS02-ROX	247	0.10	Pass	500.0	[<Confirmed>]	
2	249.8	2274	17205	SS02-ROX	249	0.00	Pass	336.4	[<Confirmed>]	

Sample 93: SSS13_SS20_SS11_SS21_SS02_SS19_HQZ36_E15.fsa

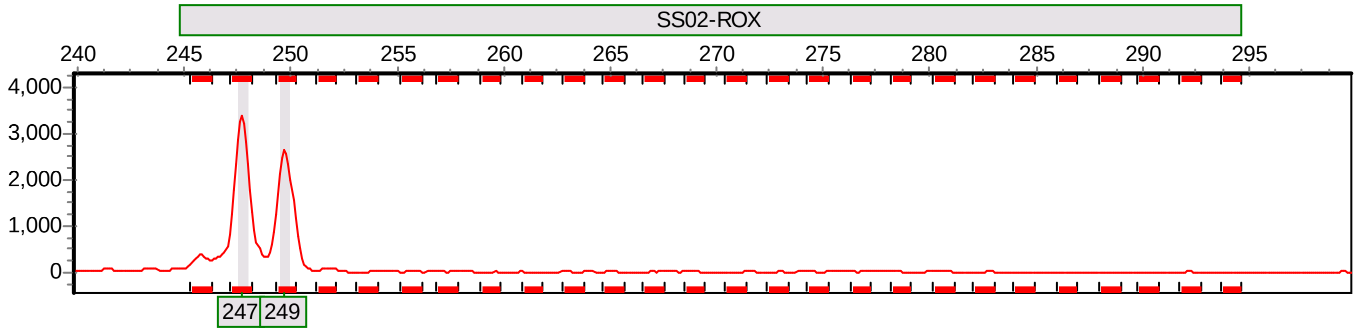

No	Size	Height	Area	Marker	Allele	Difference	Quality	Score	Allele Comments	Sample Comments
1	247.7	3388	28620	SS02-ROX	247	0.00	Pass	498.7	[<Confirmed>]	
2	249.7	2645	21765	SS02-ROX	249	0.10	Pass	340.8	[<Confirmed>]	

Sample 94: SSS13_SS20_SS11_SS21_SS02_SS19_HQZ37_F03.fsa

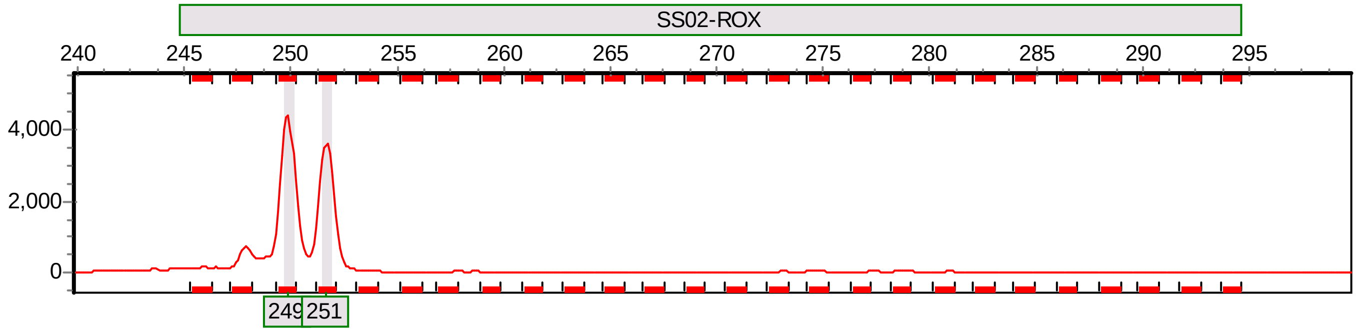

No	Size	Height	Area	Marker	Allele	Difference	Quality	Score	Allele Comments	Sample Comments
1	249.9	4374	34311	SS02-ROX	249	0.10	Pass	500.0	[<Confirmed>]	
2	251.7	3587	28003	SS02-ROX	251	0.00	Pass	500.0	[<Confirmed>]	

Sample 95: SSS13_SS20_SS11_SS21_SS02_SS19_HQZ38_L01.fsa

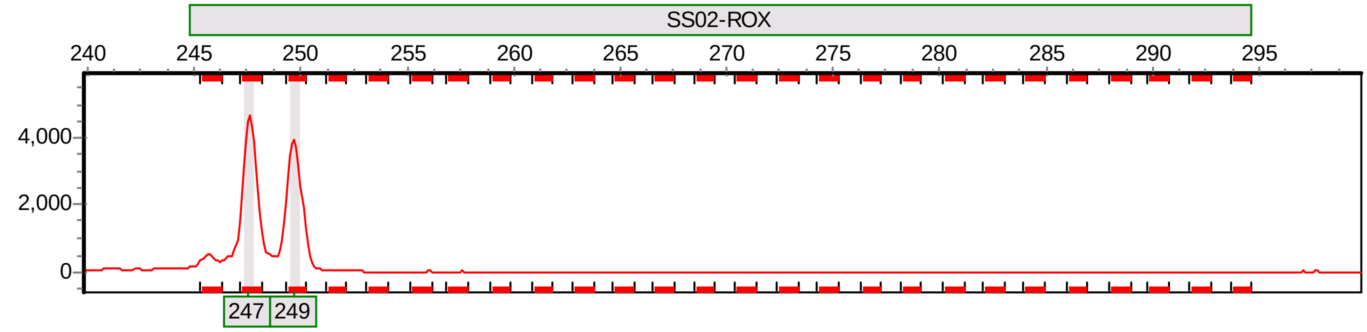

No	Size	Height	Area	Marker	Allele	Difference	Quality	Score	Allele Comments	Sample Comments
1	247.6	4623	37162	SS02-ROX	247	0.10	Pass	500.0	[<Confirmed>]	
2	249.7	3917	31583	SS02-ROX	249	0.10	Pass	500.0	[<Confirmed>]	

Sample 96: SSS13_SS20_SS11_SS21_SS02_SS19_HQZ39_M15.fsa

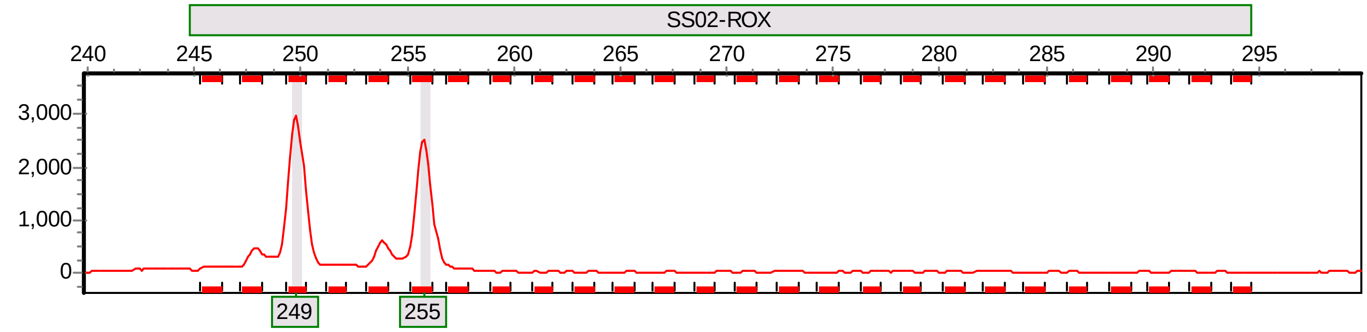

No	Size	Height	Area	Marker	Allele	Difference	Quality	Score	Allele Comments	Sample Comments
1	249.8	2959	25490	SS02-ROX	249	0.00	Pass	384.4	[<Confirmed>]	
2	255.8	2497	21581	SS02-ROX	255	0.10	Pass	292.0	[<Confirmed>]	

Sample 97: SSS13_SS20_SS11_SS21_SS02_SS19_HQZ7_C03.fsa

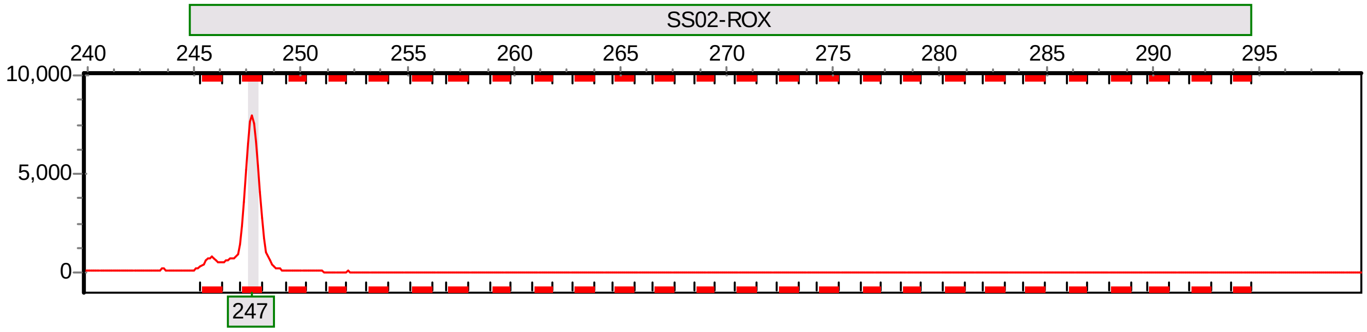

No	Size	Height	Area	Marker	Allele	Difference	Quality	Score	Allele Comments	Sample Comments
1	247.7	7913	61475	SS02-ROX	247	0.00	Pass	500.0	[<Confirmed>]	

SoftGenetics

Allele Report

GeneMarker V3.0.0

Sample 98: SSS13_SS20_SS11_SS21_SS02_SS19_HQZ9_K11.fsa

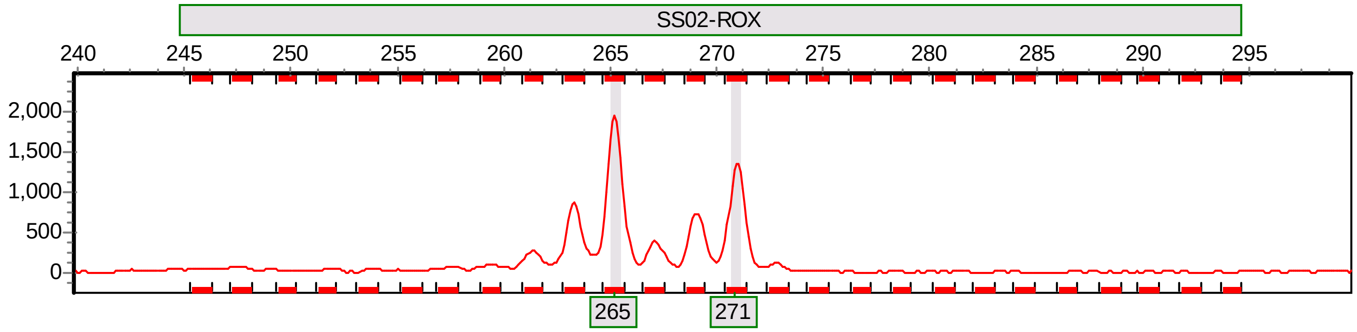

No	Size	Height	Area	Marker	Allele	Difference	Quality	Score	Allele Comments	Sample Comments
1	265.2	1941	16648	SS02-ROX	265	0.00	Pass	212.9	[<Confirmed>]	
2	270.9	1360	11442	SS02-ROX	271	0.00	Pass	134.0	[<Confirmed>]	

Sample 99: SSS13_SS20_SS11_SS21_SS02_SS19_HRS24_I17.fsa

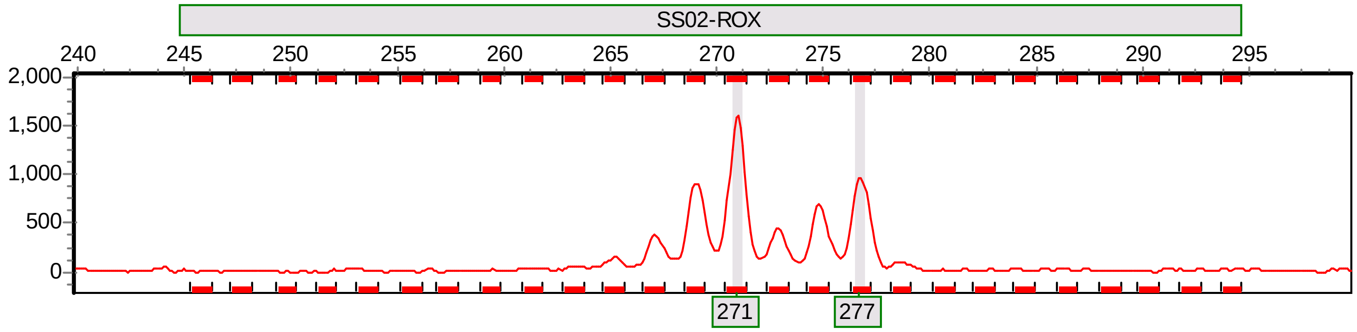

No	Size	Height	Area	Marker	Allele	Difference	Quality	Score	Allele Comments	Sample Comments
1	271.0	1594	13632	SS02-ROX	271	0.10	Pass	153.2	[<Confirmed>]	
2	276.7	969	8912	SS02-ROX	277	0.10	Pass	61.1	[<Confirmed>]	

Sample 100: SSS13_SS20_SS11_SS21_SS02_SS19_HRS26_H09.fsa

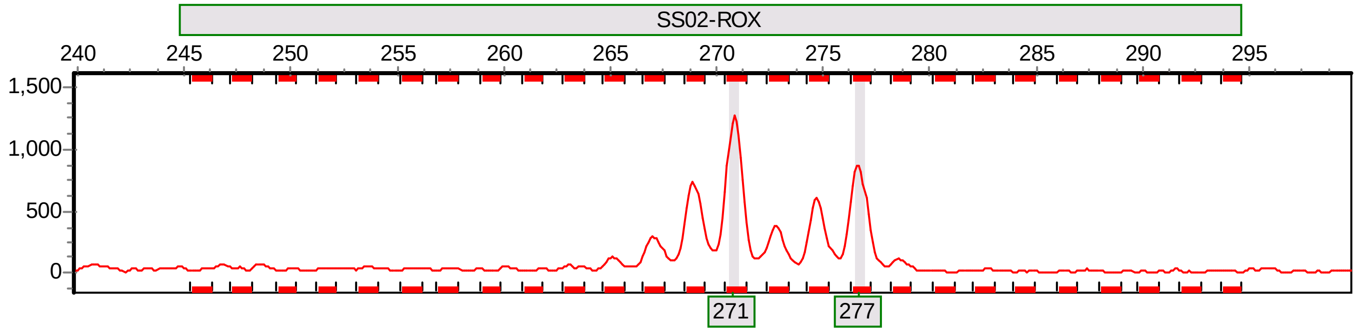

No	Size	Height	Area	Marker	Allele	Difference	Quality	Score	Allele Comments	Sample Comments
1	270.8	1268	11025	SS02-ROX	271	0.10	Pass	111.1	[<Confirmed>]	
2	276.7	872	8068	SS02-ROX	277	0.10	Pass	50.5	[<Confirmed>]	

Allele Report

Sample 101: SSS13_SS20_SS11_SS21_SS02_SS19_HRS28_D13.fsa

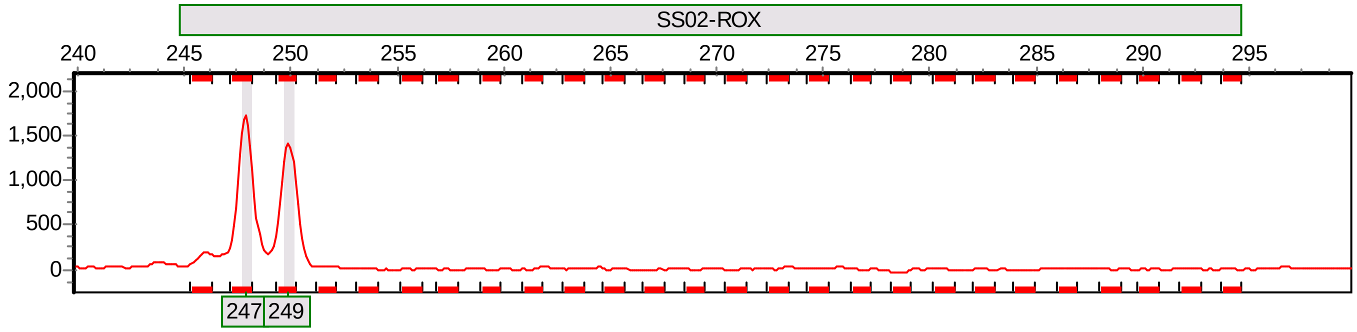

No	Size	Height	Area	Marker	Allele	Difference	Quality	Score	Allele Comments	Sample Comments
1	247.9	1717	13953	SS02-ROX	247	0.20	Pass	189.8	[<Confirmed>]	
2	249.9	1417	11794	SS02-ROX	249	0.10	Pass	145.3	[<Confirmed>]	

Sample 102: SSS13_SS20_SS11_SS21_SS02_SS19_HRS29_J05.fsa

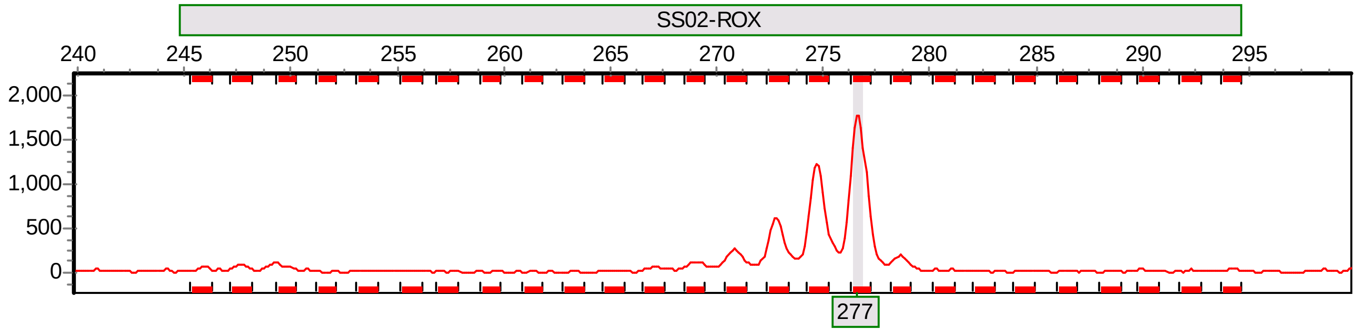

No	Size	Height	Area	Marker	Allele	Difference	Quality	Score	Allele Comments	Sample Comments
1	276.6	1758	15023	SS02-ROX	277	0.20	Pass	180.4	[<Confirmed>]	

Sample 103: SSS13_SS20_SS11_SS21_SS02_SS19_HRS30_P05.fsa

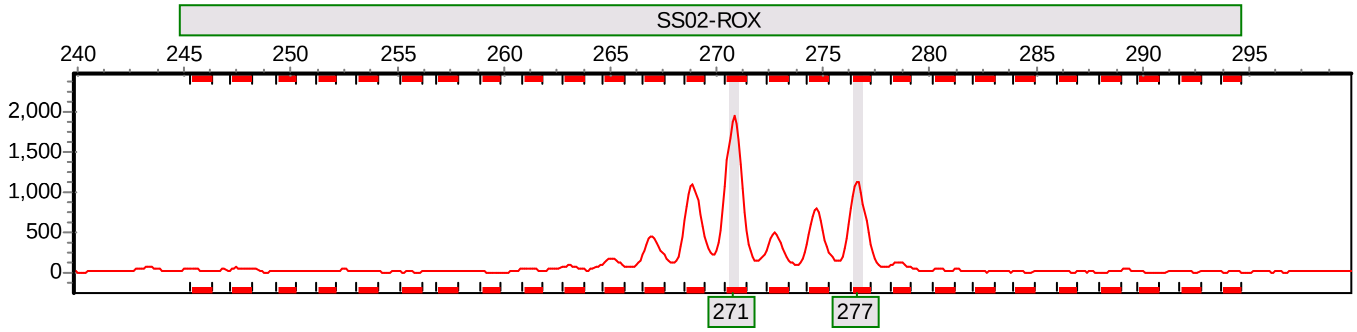

No	Size	Height	Area	Marker	Allele	Difference	Quality	Score	Allele Comments	Sample Comments
1	270.8	1948	16688	SS02-ROX	271	0.10	Pass	215.5	[<Confirmed>]	
2	276.6	1139	9786	SS02-ROX	277	0.20	Pass	92.4	[<Confirmed>]	

Allele Report

Sample 104: SSS13_SS20_SS11_SS21_SS02_SS19_HRS31_J11.fsa

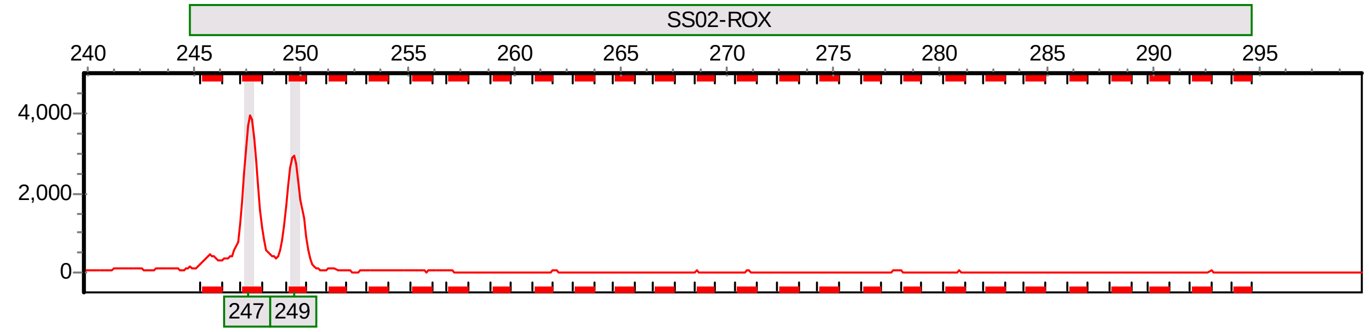

No	Size	Height	Area	Marker	Allele	Difference	Quality	Score	Allele Comments	Sample Comments
1	247.6	3933	31546	SS02-ROX	247	0.10	Pass	500.0	[<Confirmed>]	
2	249.7	2959	23902	SS02-ROX	249	0.10	Pass	438.1	[<Confirmed>]	

Sample 105: SSS13_SS20_SS11_SS21_SS02_SS19_HRS33_H07.fsa

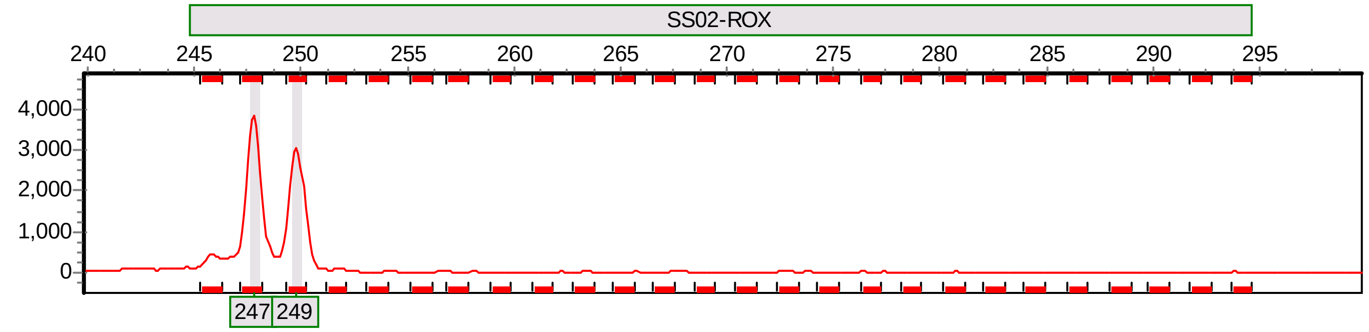

No	Size	Height	Area	Marker	Allele	Difference	Quality	Score	Allele Comments	Sample Comments
1	247.8	3815	31436	SS02-ROX	247	0.10	Pass	500.0	[<Confirmed>]	
2	249.8	3034	25057	SS02-ROX	249	0.00	Pass	434.7	[<Confirmed>]	

Sample 106: SSS13_SS20_SS11_SS21_SS02_SS19_HRS34_L09.fsa

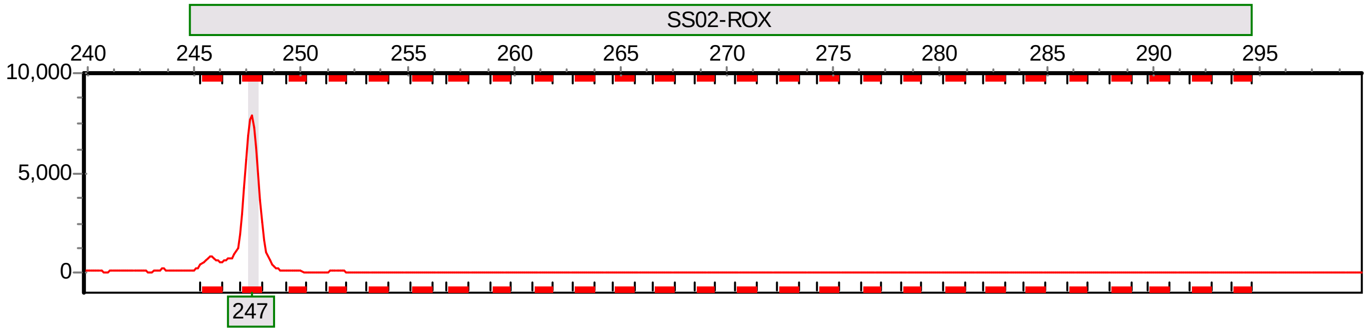

No	Size	Height	Area	Marker	Allele	Difference	Quality	Score	Allele Comments	Sample Comments
1	247.7	7869	62822	SS02-ROX	247	0.00	Pass	500.0	[<Confirmed>]	

Allele Report

Sample 107: SSS13_SS20_SS11_SS21_SS02_SS19_HRS35_P09.fsa

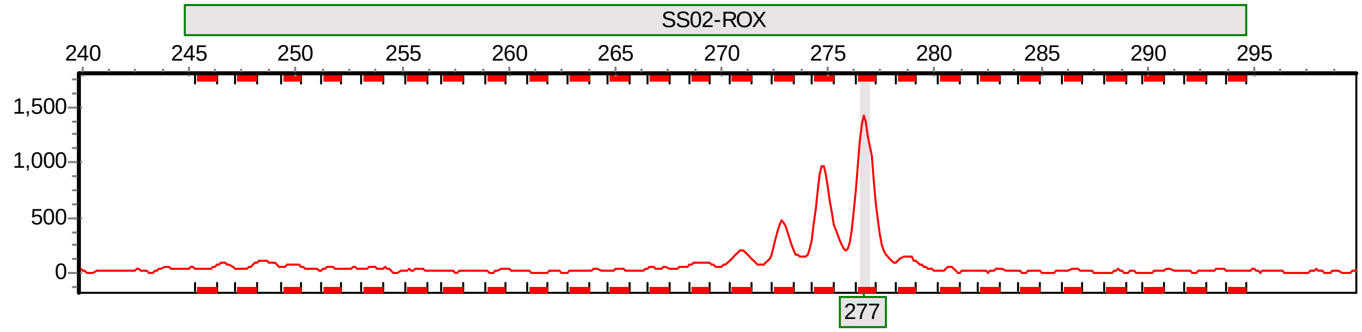

No	Size	Height	Area	Marker	Allele	Difference	Quality	Score	Allele Comments	Sample Comments
1	276.7	1424	12770	SS02-ROX	277	0.10	Pass	128.5	[<Confirmed>]	

Sample 108: SSS13_SS20_SS11_SS21_SS02_SS19_HRS37_J13.fsa

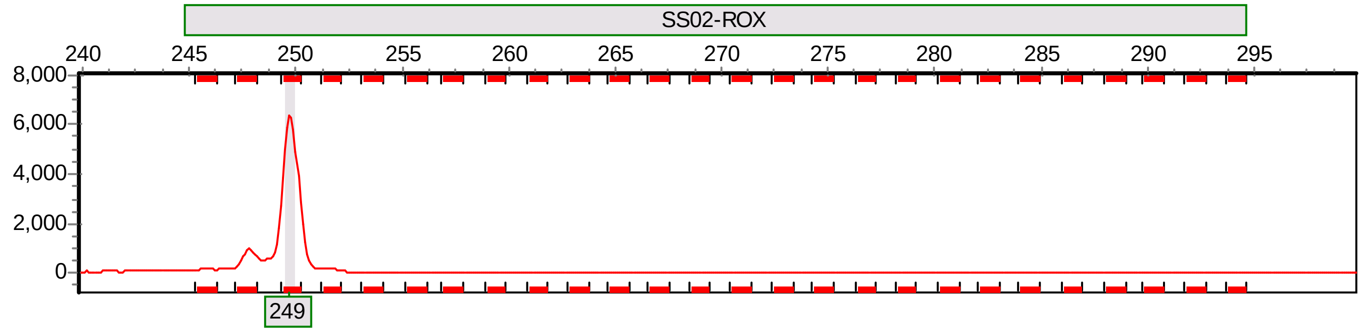

No	Size	Height	Area	Marker	Allele	Difference	Quality	Score	Allele Comments	Sample Comments
1	249.7	6323	51990	SS02-ROX	249	0.10	Pass	500.0	[<Confirmed>]	

Sample 109: SSS13_SS20_SS11_SS21_SS02_SS19_HRS38_J07.fsa

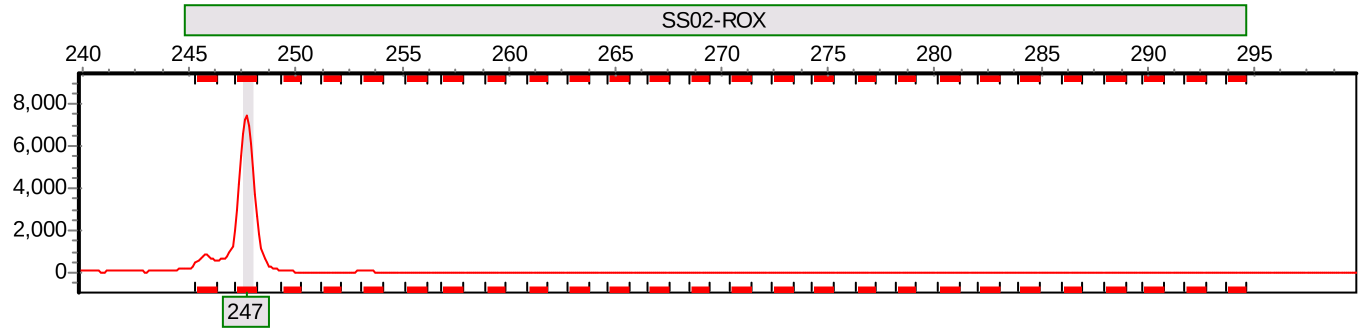

No	Size	Height	Area	Marker	Allele	Difference	Quality	Score	Allele Comments	Sample Comments
1	247.7	7349	61810	SS02-ROX	247	0.00	Pass	500.0	[<Confirmed>]	

Sample 110: SSS13_SS20_SS11_SS21_SS02_SS19_HRS39_N07.fsa

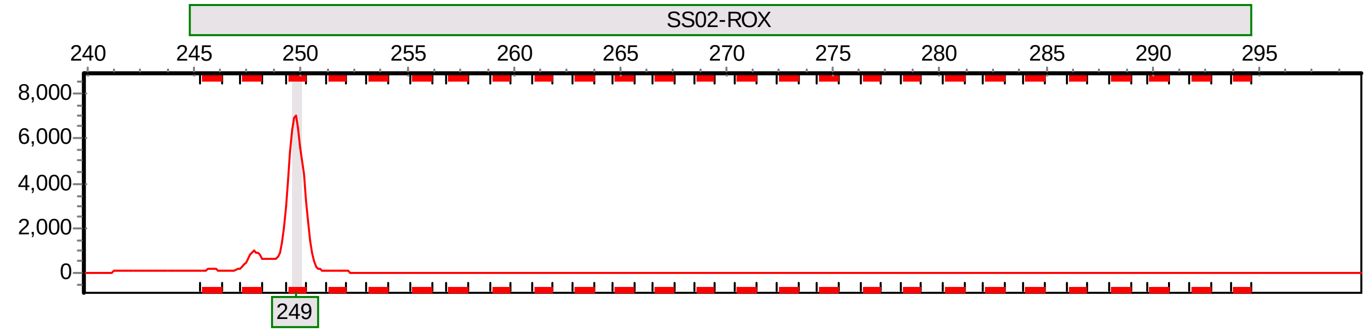

No	Size	Height	Area	Marker	Allele	Difference	Quality	Score	Allele Comments	Sample Comments
1	249.8	6965	57966	SS02-ROX	249	0.00	Pass	500.0	[<Confirmed>]	

Sample 111: SSS13_SS20_SS11_SS21_SS02_SS19_HRS40_A07.fsa

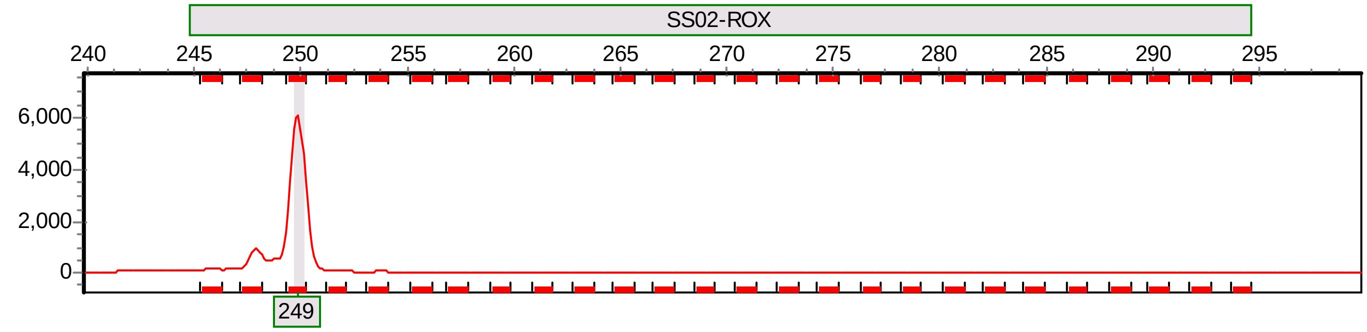

No	Size	Height	Area	Marker	Allele	Difference	Quality	Score	Allele Comments	Sample Comments
1	249.9	6083	48312	SS02-ROX	249	0.10	Pass	500.0	[<Confirmed>]	

Sample 112: SSS13_SS20_SS11_SS21_SS02_SS19_HRS41_K07.fsa

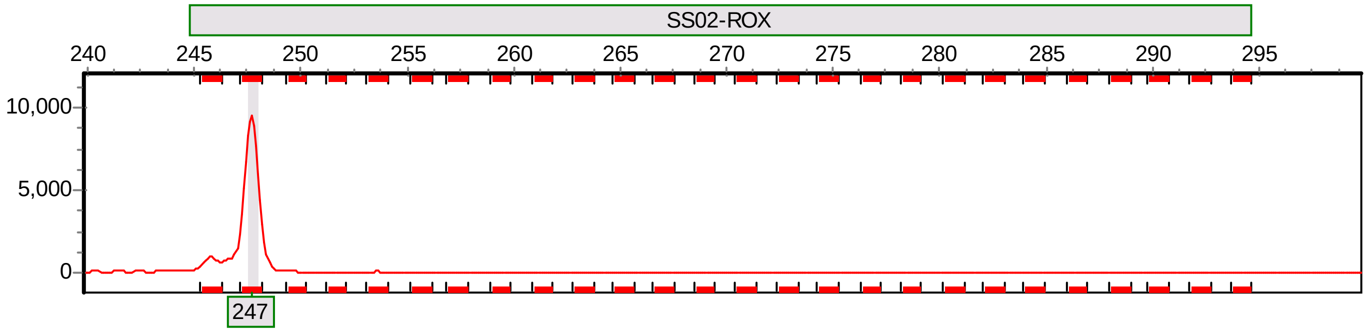

No	Size	Height	Area	Marker	Allele	Difference	Quality	Score	Allele Comments	Sample Comments
1	247.7	9415	74968	SS02-ROX	247	0.00	Pass	500.0	[<Confirmed>]	

Sample 113: SSS13_SS20_SS11_SS21_SS02_SS19_HRS42_I07.fsa

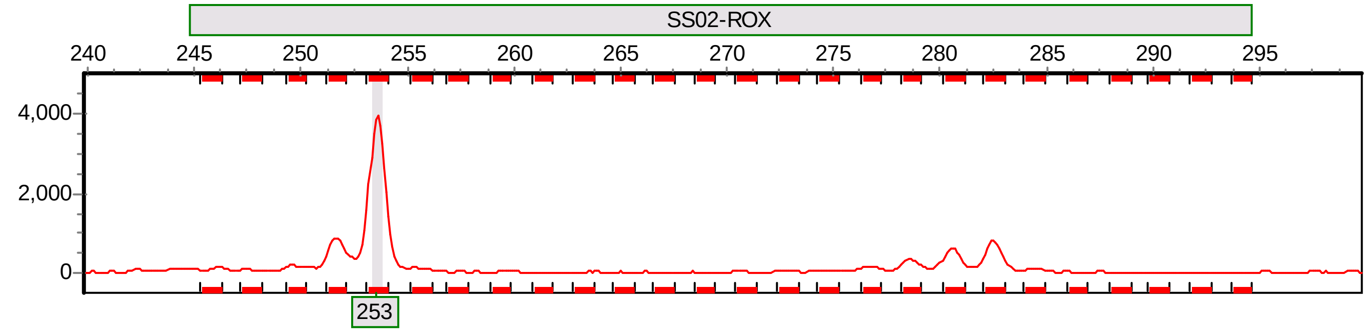

No	Size	Height	Area	Marker	Allele	Difference	Quality	Score	Allele Comments	Sample Comments
1	253.6	3936	33146	SS02-ROX	253	0.00	Pass	500.0	[<Confirmed>]	

Sample 114: SSS13_SS20_SS11_SS21_SS02_SS19_HTHL11_M11.fsa

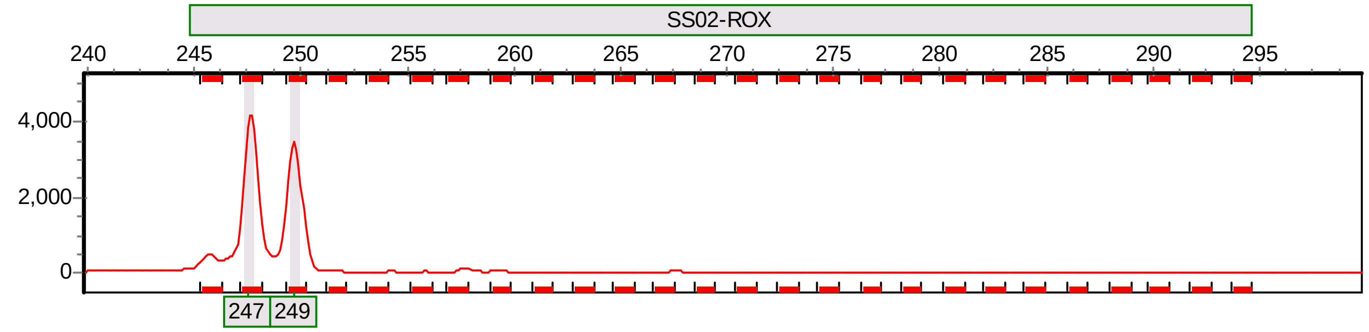

No	Size	Height	Area	Marker	Allele	Difference	Quality	Score	Allele Comments	Sample Comments
1	247.6	4131	34229	SS02-ROX	247	0.10	Pass	500.0	[<Confirmed>]	
2	249.7	3444	27997	SS02-ROX	249	0.10	Pass	500.0	[<Confirmed>]	

Sample 115: SSS13_SS20_SS11_SS21_SS02_SS19_HTHL13_H01.fsa

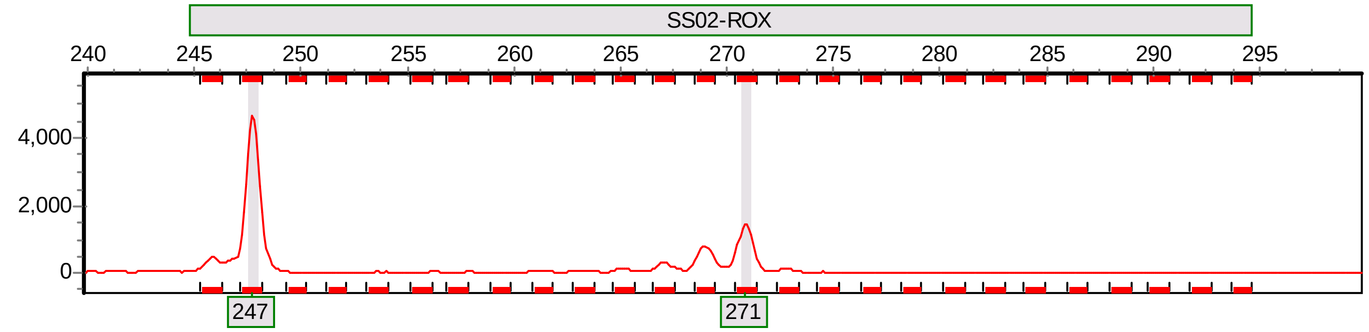

No	Size	Height	Area	Marker	Allele	Difference	Quality	Score	Allele Comments	Sample Comments
1	247.7	4647	35455	SS02-ROX	247	0.00	Pass	500.0	[<Confirmed>]	
2	270.9	1441	11794	SS02-ROX	271	0.00	Pass	129.2	[<Confirmed>]	

Allele Report

Sample 116: SSS13_SS20_SS11_SS21_SS02_SS19_HTHL14_F15.fsa

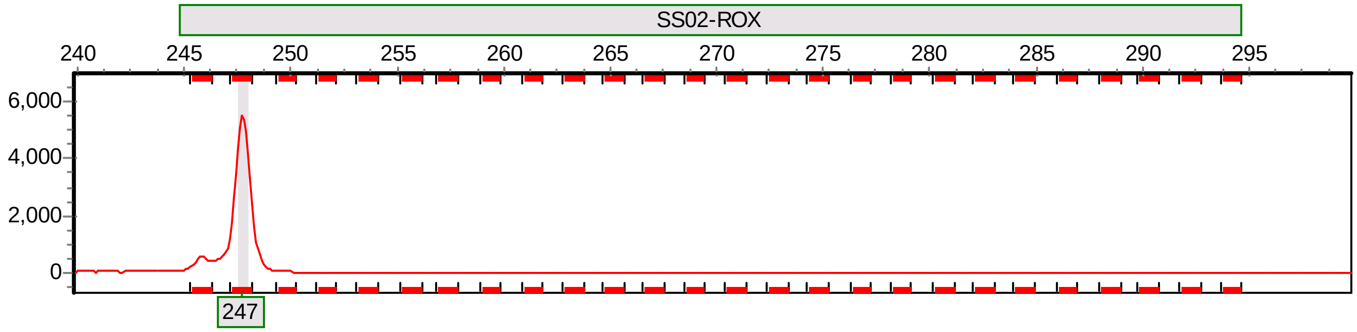

No	Size	Height	Area	Marker	Allele	Difference	Quality	Score	Allele Comments	Sample Comments
1	247.7	5453	44909	SS02-ROX	247	0.00	Pass	500.0	[<Confirmed>]	

Sample 117: SSS13_SS20_SS11_SS21_SS02_SS19_HTHL15_F01.fsa

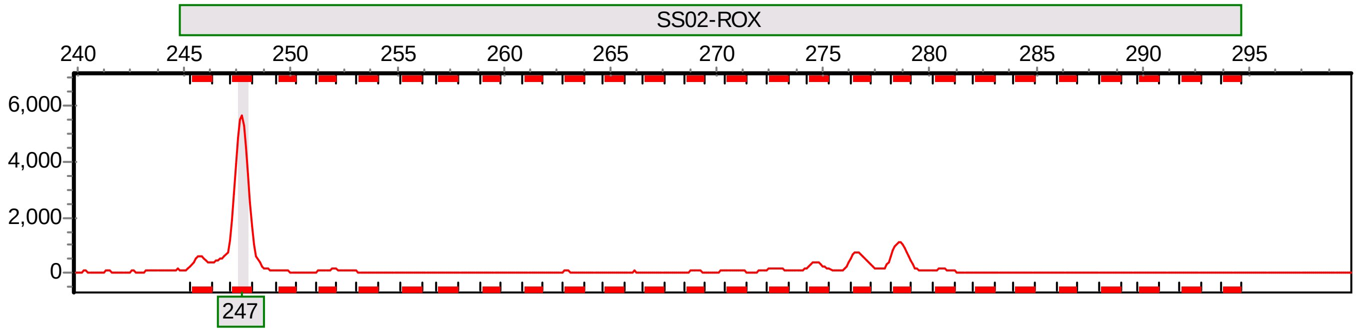

No	Size	Height	Area	Marker	Allele	Difference	Quality	Score	Allele Comments	Sample Comments
1	247.7	5631	43358	SS02-ROX	247	0.00	Pass	500.0	[<Confirmed>]	

Sample 118: SSS13_SS20_SS11_SS21_SS02_SS19_HTHL1_E13.fsa

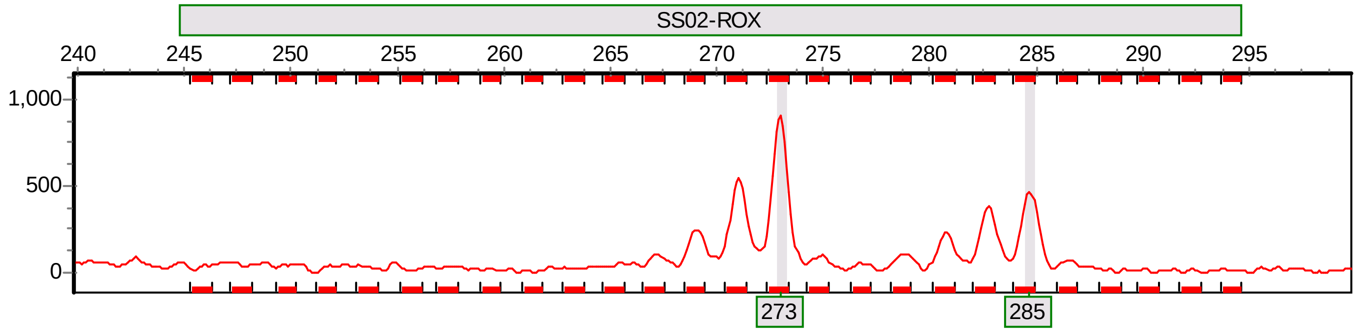

No	Size	Height	Area	Marker	Allele	Difference	Quality	Score	Allele Comments	Sample Comments
1	273.0	902	8015	SS02-ROX	273	0.10	Pass	61.9	[<Confirmed>]	
2	284.7	463	4188	SS02-ROX	285	0.20	Pass	18.8	[<Confirmed>]	

Allele Report

Sample 119: SSS13_SS20_SS11_SS21_SS02_SS19_HTHL3_D05.fsa

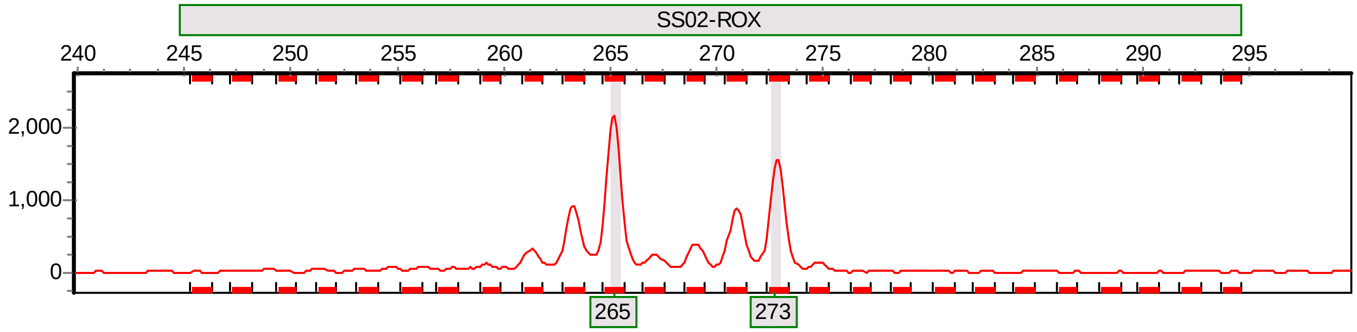

No	Size	Height	Area	Marker	Allele	Difference	Quality	Score	Allele Comments	Sample Comments
1	265.2	2166	17754	SS02-ROX	265	0.00	Pass	262.0	[<Confirmed>]	
2	272.8	1564	12888	SS02-ROX	273	0.10	Pass	161.2	[<Confirmed>]	

Sample 120: SSS13_SS20_SS11_SS21_SS02_SS19_HTHL4_O15.fsa

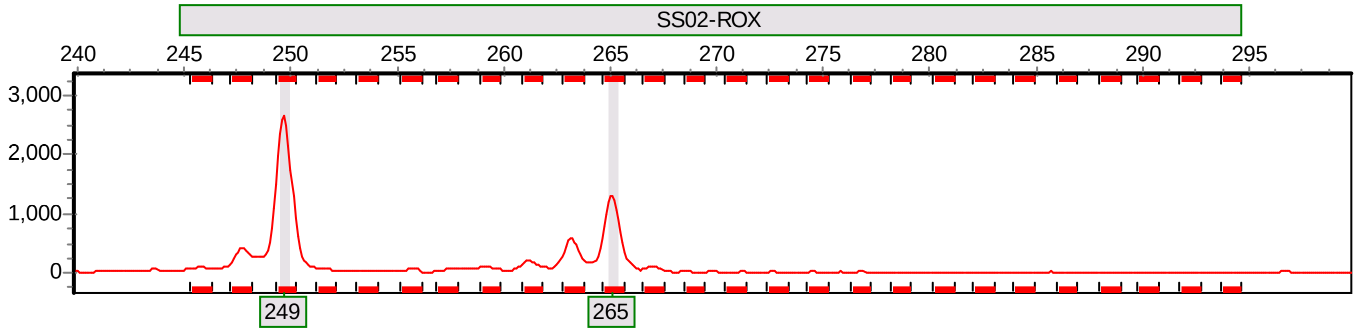

No	Size	Height	Area	Marker	Allele	Difference	Quality	Score	Allele Comments	Sample Comments
1	249.7	2650	22395	SS02-ROX	249	0.10	Pass	347.3	[<Confirmed>]	
2	265.1	1307	11345	SS02-ROX	265	0.10	Pass	111.7	[<Confirmed>]	

Sample 121: SSS13_SS20_SS11_SS21_SS02_SS19_HTHL5_M13.fsa

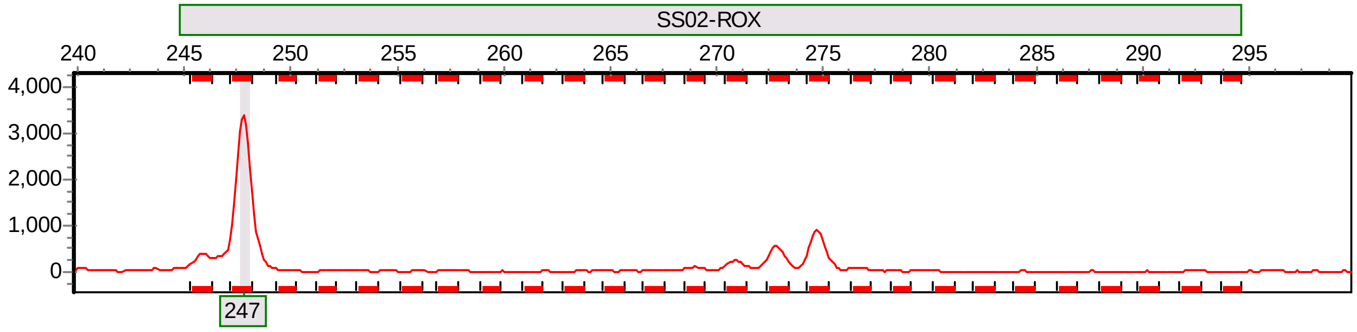

No	Size	Height	Area	Marker	Allele	Difference	Quality	Score	Allele Comments	Sample Comments
1	247.8	3366	29217	SS02-ROX	247	0.10	Pass	452.4	[<Confirmed>]	

Allele Report

Sample 122: SSS13_SS20_SS11_SS21_SS02_SS19_HTHL6_P03.fsa

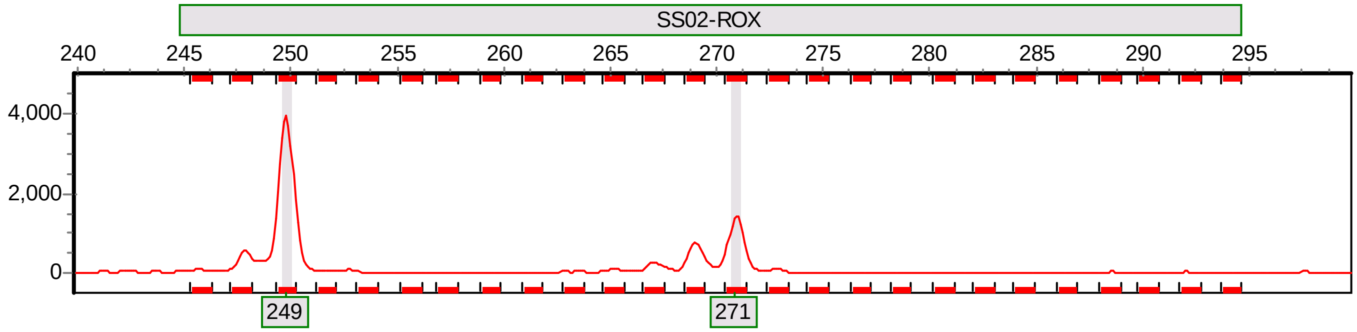

No	Size	Height	Area	Marker	Allele	Difference	Quality	Score	Allele Comments	Sample Comments
1	249.8	3938	30886	SS02-ROX	249	0.00	Pass	500.0	[<Confirmed>]	
2	270.9	1438	11716	SS02-ROX	271	0.00	Pass	133.0	[<Confirmed>]	

Sample 123: SSS13_SS20_SS11_SS21_SS02_SS19_HTHL8_A03.fsa

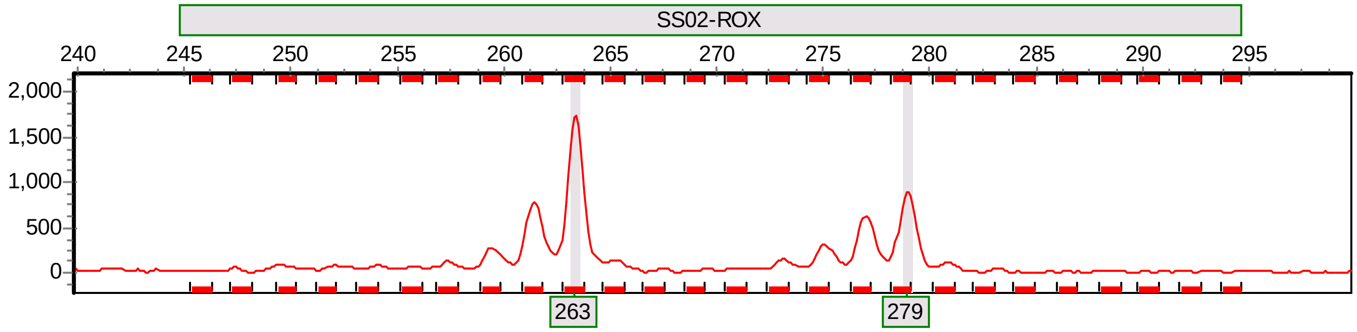

No	Size	Height	Area	Marker	Allele	Difference	Quality	Score	Allele Comments	Sample Comments
1	263.4	1734	15319	SS02-ROX	263	0.10	Pass	167.4	[<Confirmed>]	
2	279.0	892	8320	SS02-ROX	279	0.30	Pass	51.6	[<Confirmed>]	

Sample 124: SSS13_SS20_SS11_SS21_SS02_SS19_HTHL9_O09.fsa

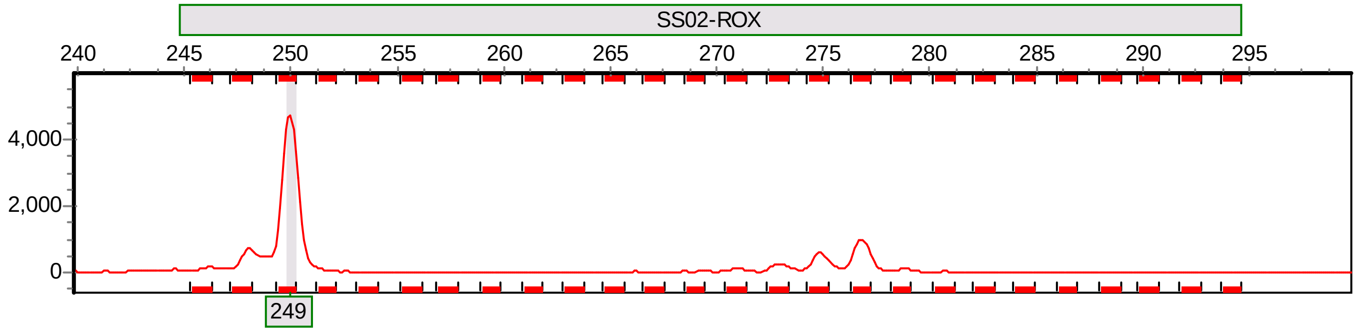

No	Size	Height	Area	Marker	Allele	Difference	Quality	Score	Allele Comments	Sample Comments
1	250.0	4686	37643	SS02-ROX	249	0.20	Pass	500.0	[<Confirmed>]	
