# Supplementary material for: Genetic diversity of Schima superba based on physiological traits and SSR markers
Source: PLoS One. 2026 Apr 10;21(4):e0344465. doi: 10.1371/journal.pone.0344465 (PMC13068225; doi:10.1371/journal.pone.0344465)
Supplement: S1 File — (ZIP) [file pone.0344465.s003.zip › SS10.pdf]

Allele Report

Project Comments:

Sample 1: SS08_SS10_SSS42_SS16_SS27_SS36_SS22_HBB10_E05.fsa

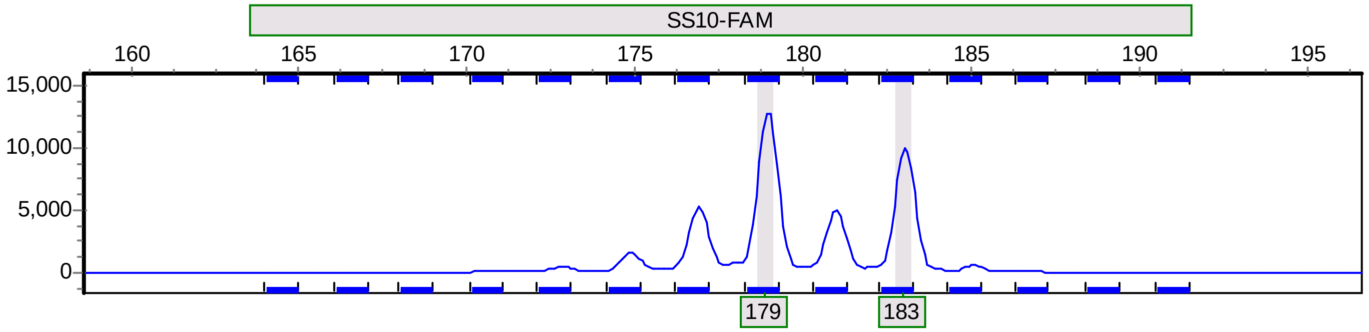

No	Size	Height	Area	Marker	Allele	Difference	Quality	Score	Allele Comments	Sample Comments
1	115.5	16673	110843	SS08-FAM	115	0.10	Pass	500.0	[<Confirmed>]	
2	117.5	12320	81840	SS08-FAM	117	0.10	Pass	500.0	[<Confirmed>]	
3	178.9	12710	86162	SS10-FAM	179	0.10	Pass	500.0	[<Confirmed>]	
4	183.0	9987	66819	SS10-FAM	183	0.20	Pass	500.0	[<Confirmed>]	
5	251.4	6639	49371	SSS42-FAM	252	0.10	Pass	500.0		

Sample 2: SS08_SS10_SSS42_SS16_SS27_SS36_SS22_HBB12-2_D11.fsa

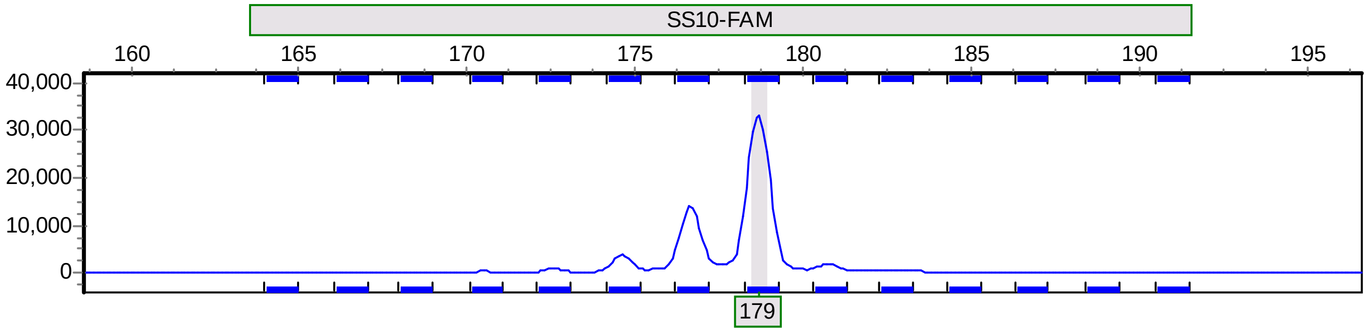

No	Size	Height	Area	Marker	Allele	Difference	Quality	Score	Allele Comments	Sample Comments
1	115.2	13271	91630	SS08-FAM	115	0.20	Pass	500.0	[<Confirmed>]	
2	119.3	10401	71418	SS08-FAM	119	0.00	Pass	500.0	[<Confirmed>]	
3	178.7	32937	250146	SS10-FAM	179	0.10	Pass	500.0	[<SAT (Repaired)><Confirmed>]	
4	224.3	24305	181368	SSS42-FAM	224	0.00	Pass	500.0		
5	294.6	17543	156335	SS16-FAM	295	0.00	Pass	500.0		

Sample 3: SS08_SS10_SSS42_SS16_SS27_SS36_SS22_HBB13_C17.fsa

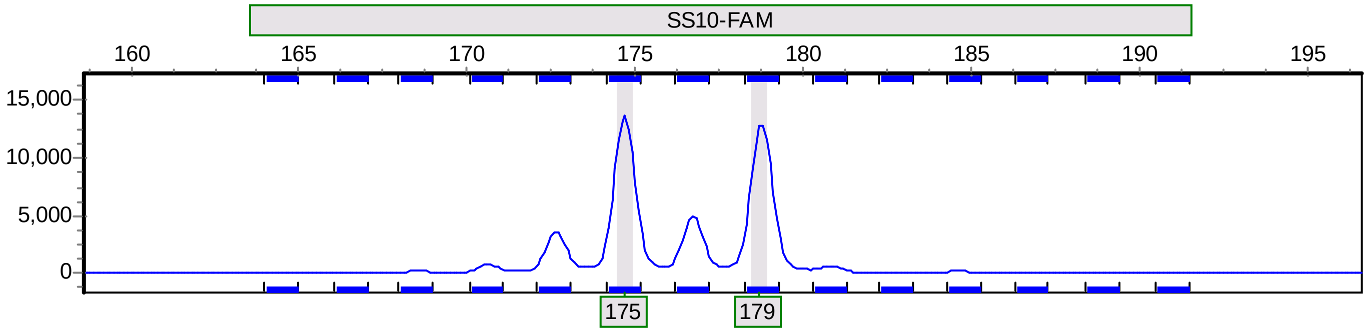

No	Size	Height	Area	Marker	Allele	Difference	Quality	Score	Allele Comments	Sample Comments
1	109.3	12871	85993	SS08-FAM	109	0.00	Pass	500.0	[<Confirmed>]	
2	115.4	15237	101137	SS08-FAM	115	0.00	Pass	500.0	[<Confirmed>]	
3	174.7	13569	97374	SS10-FAM	175	0.00	Pass	500.0	[<Confirmed>]	
4	178.7	12739	93190	SS10-FAM	179	0.10	Pass	500.0	[<Confirmed>]	

5	230.1	18018	137858	SSS42-FAM	230	0.10	Pass	500.0		
6	303.6	5350	52895	SS16-FAM	303	0.00	Pass	500.0		

Sample 4: SS08_SS10_SSS42_SS16_SS27_SS36_SS22_HBB14_G03.fsa

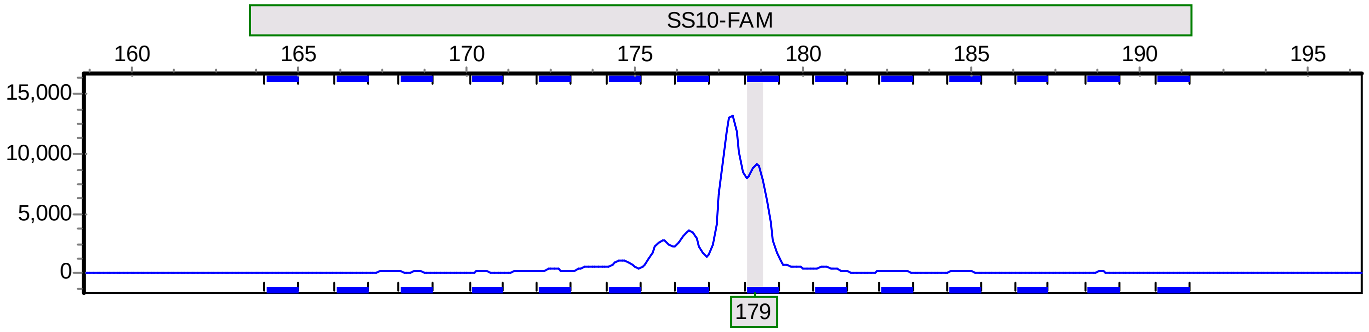

No	Size	Height	Area	Marker	Allele	Difference	Quality	Score	Allele Comments	Sample Comments
1	103.8	11187	71888	SS08-FAM	103	0.10	Pass	500.0	[<Confirmed>]	
2	113.4	7455	49368	SS08-FAM	113	0.00	Pass	500.0	[<Confirmed>]	
3	178.6	9139	154950	SS10-FAM	179	0.20	Pass	500.0	[<Confirmed><Inserted>]	
4	218.3	10017	69754	SSS42-FAM	218	0.10	Pass	500.0		
5	294.6	2489	22455	SS16-FAM	295	0.00	Pass	282.0		

Sample 5: SS08_SS10_SSS42_SS16_SS27_SS36_SS22_HBB15_B07.fsa

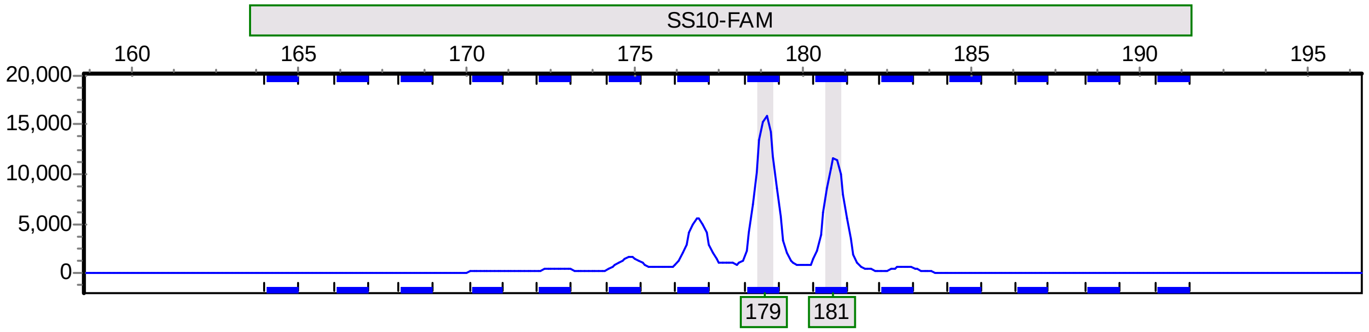

No	Size	Height	Area	Marker	Allele	Difference	Quality	Score	Allele Comments	Sample Comments
1	115.4	11333	76575	SS08-FAM	115	0.00	Pass	500.0	[<Confirmed>]	
2	117.4	8835	59288	SS08-FAM	117	0.00	Pass	500.0	[<Confirmed>]	
3	178.9	15811	108146	SS10-FAM	179	0.10	Pass	500.0	[<Confirmed>]	
4	180.9	11668	80839	SS10-FAM	181	0.10	Pass	500.0	[<Confirmed>]	
5	222.5	8751	63603	SSS42-FAM	222	0.20	Pass	500.0		
6	230.3	10073	74598	SSS42-FAM	230	0.10	Pass	500.0		
7	303.5	3393	31886	SS16-FAM	303	0.10	Pass	368.8		

Sample 6: SS08_SS10_SSS42_SS16_SS27_SS36_SS22_HBB16_G07.fsa

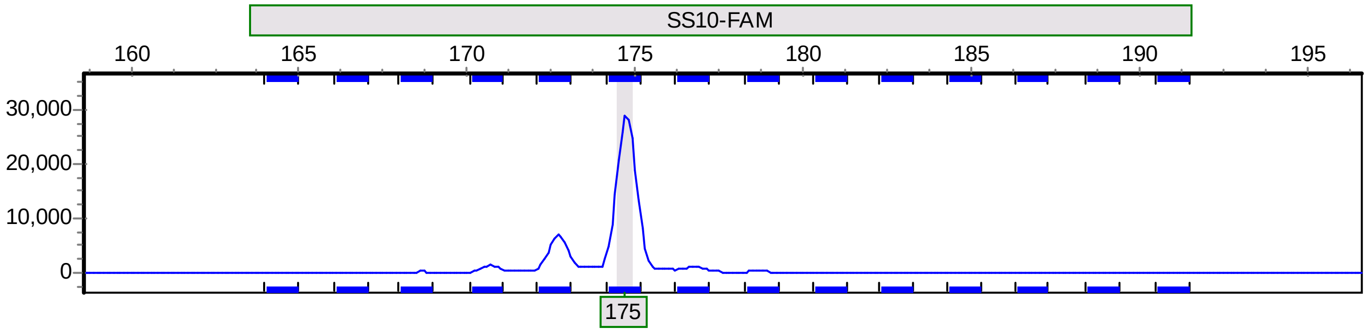

No	Size	Height	Area	Marker	Allele	Difference	Quality	Score	Allele Comments	Sample Comments
1	117.2	19793	142150	SS08-FAM	117	0.20	Pass	500.0	[<Confirmed>]	

2	174.7	28914	194959	SS10-FAM	175	0.00	Pass	500.0	[<Confirmed>]	
3	228.1	5752	42231	SSS42-FAM	228	0.10	Pass	500.0		
4	232.0	8026	58905	SSS42-FAM	232	0.00	Pass	500.0		
5	286.6	15602	132180	SS16-FAM	287	0.10	Pass	500.0		

Sample 7: SS08_SS10_SSS42_SS16_SS27_SS36_SS22_HBB17_K15.fsa

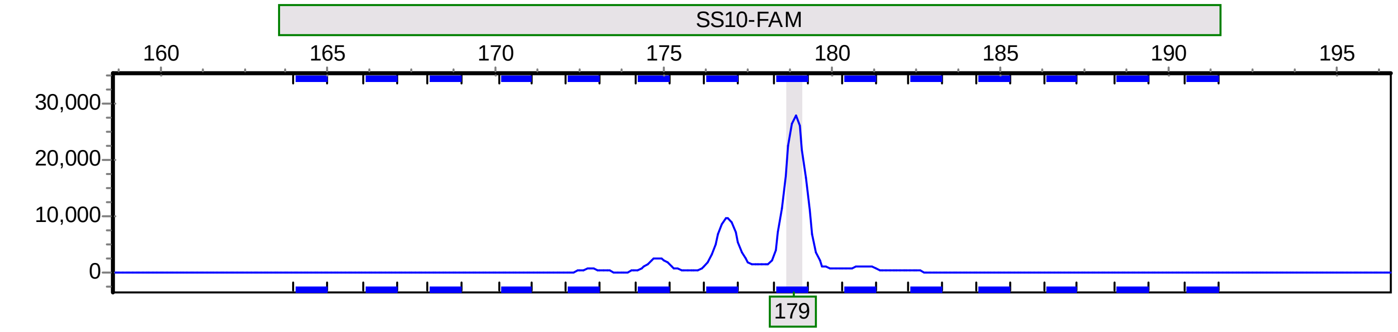

No	Size	Height	Area	Marker	Allele	Difference	Quality	Score	Allele Comments	Sample Comments
1	117.4	26538	179563	SS08-FAM	117	0.00	Pass	500.0	[<Confirmed>]	
2	178.9	27557	193198	SS10-FAM	179	0.10	Pass	500.0	[<Confirmed>]	
3	224.3	7670	56971	SSS42-FAM	224	0.00	Pass	500.0		
4	230.2	10102	76782	SSS42-FAM	230	0.00	Pass	500.0		
5	303.6	9343	89691	SS16-FAM	303	0.00	Pass	500.0		

Sample 8: SS08_SS10_SSS42_SS16_SS27_SS36_SS22_HBB18_O05.fsa

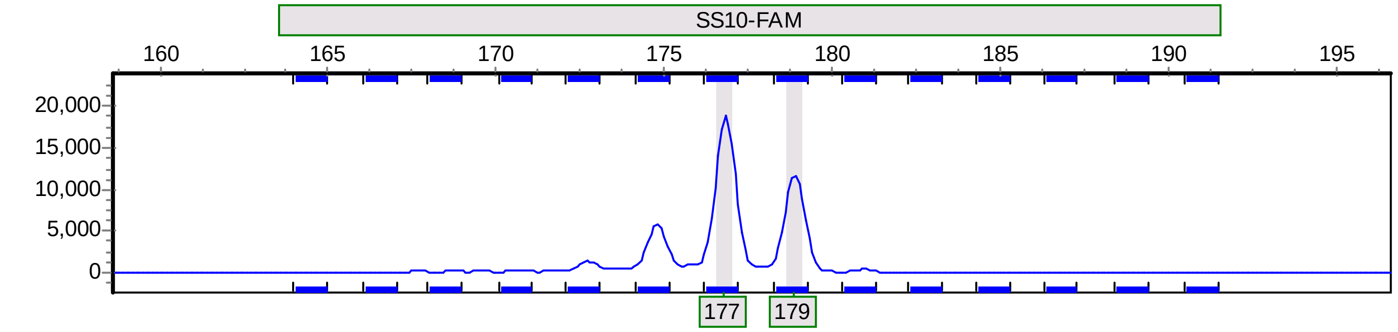

No	Size	Height	Area	Marker	Allele	Difference	Quality	Score	Allele Comments	Sample Comments
1	115.5	13247	90002	SS08-FAM	115	0.10	Pass	500.0	[<Confirmed>]	
2	117.3	7015	47058	SS08-FAM	117	0.10	Pass	500.0	[<Confirmed>]	
3	176.8	18706	126846	SS10-FAM	177	0.10	Pass	500.0	[<Confirmed>]	
4	178.9	11711	78954	SS10-FAM	179	0.10	Pass	500.0	[<Confirmed>]	
5	222.4	6405	45676	SSS42-FAM	222	0.10	Pass	500.0		
6	249.6	3425	25997	SSS42-FAM	250	0.00	Pass	500.0		
7	282.6	5206	42683	SS16-FAM	283	0.00	Pass	500.0		
8	294.8	6529	56197	SS16-FAM	295	0.20	Pass	500.0		

Sample 9: SS08_SS10_SSS42_SS16_SS27_SS36_SS22_HBB19_O07.fsa

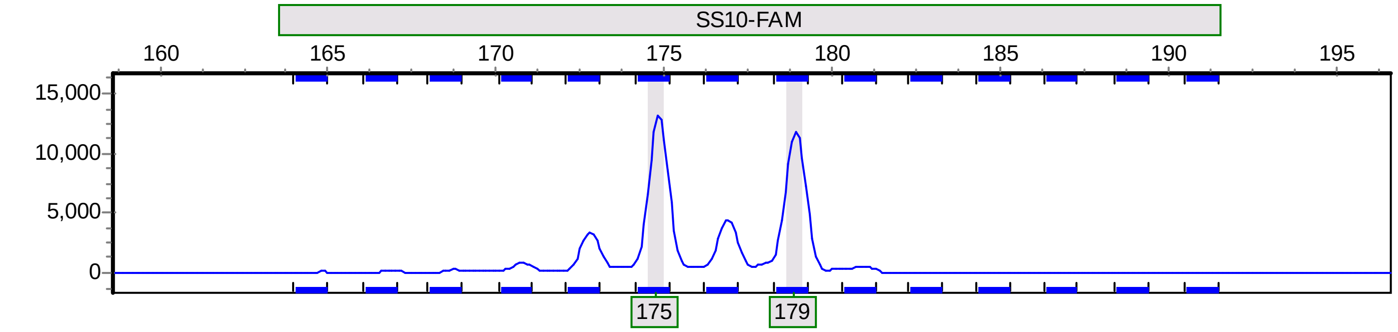

No	Size	Height	Area	Marker	Allele	Difference	Quality	Score	Allele Comments	Sample Comments
1	115.3	12016	82145	SS08-FAM	115	0.10	Pass	500.0	[<Confirmed>]	
2	117.5	11905	78807	SS08-FAM	117	0.10	Pass	500.0	[<Confirmed>]	
3	174.8	13038	87412	SS10-FAM	175	0.10	Pass	500.0	[<Confirmed>]	
4	178.9	11720	80072	SS10-FAM	179	0.10	Pass	500.0	[<Confirmed>]	
5	230.3	15308	107890	SSS42-FAM	230	0.10	Pass	500.0		
6	303.7	7315	66538	SS16-FAM	303	0.10	Pass	500.0		

Sample 10: SS08_SS10_SSS42_SS16_SS27_SS36_SS22_HBB1_B11.fsa

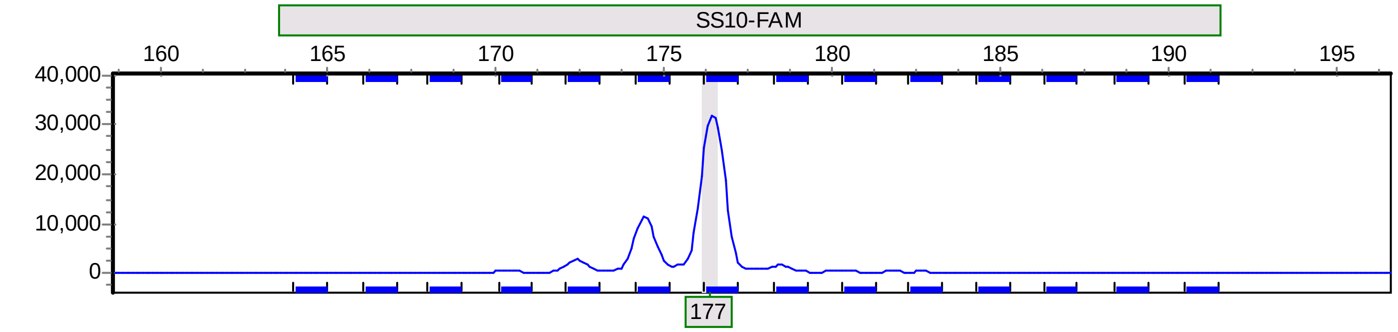

No	Size	Height	Area	Marker	Allele	Difference	Quality	Score	Allele Comments	Sample Comments
1	115.6	29586	204586	SS08-FAM	115	0.20	Pass	500.0	[<Confirmed>]	
2	176.4	31535	249820	SS10-FAM	177	0.30	Pass	500.0	[<Confirmed>]	
3	222.2	8195	61352	SSS42-FAM	222	0.10	Pass	500.0		
4	239.8	9765	74865	SSS42-FAM	240	0.00	Pass	500.0		
5	282.4	26504	223266	SS16-FAM	283	0.20	Pass	500.0		

Sample 11: SS08_SS10_SSS42_SS16_SS27_SS36_SS22_HBB20_F11.fsa

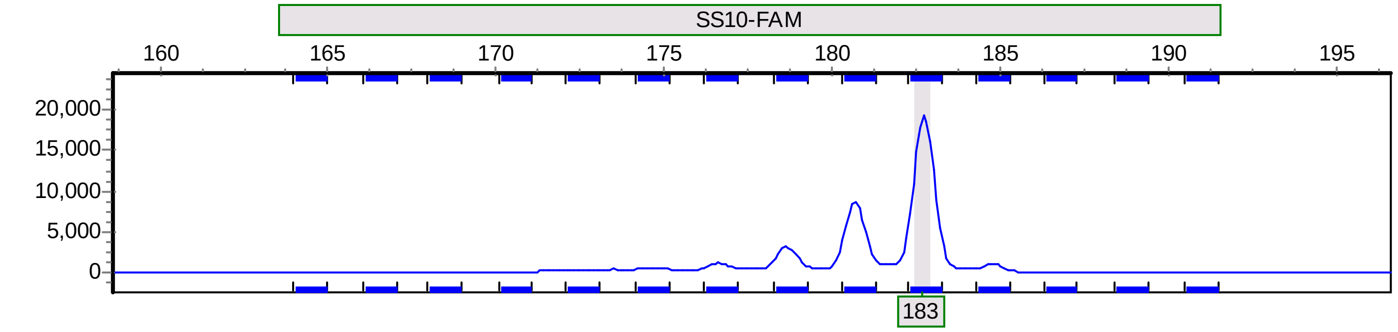

No	Size	Height	Area	Marker	Allele	Difference	Quality	Score	Allele Comments	Sample Comments
1	115.6	21047	143895	SS08-FAM	115	0.20	Pass	500.0	[<Confirmed>]	
2	182.7	19192	135443	SS10-FAM	183	0.10	Pass	500.0	[<Confirmed>]	
3	218.3	13076	95915	SSS42-FAM	218	0.10	Pass	500.0		
4	236.0	6552	50336	SSS42-FAM	236	0.10	Pass	500.0		

5	290.3	11796	107840	SS16-FAM	291	0.20	Pass	500.0		

Sample 12: SS08_SS10_SSS42_SS16_SS27_SS36_SS22_HBB21_J09.fsa

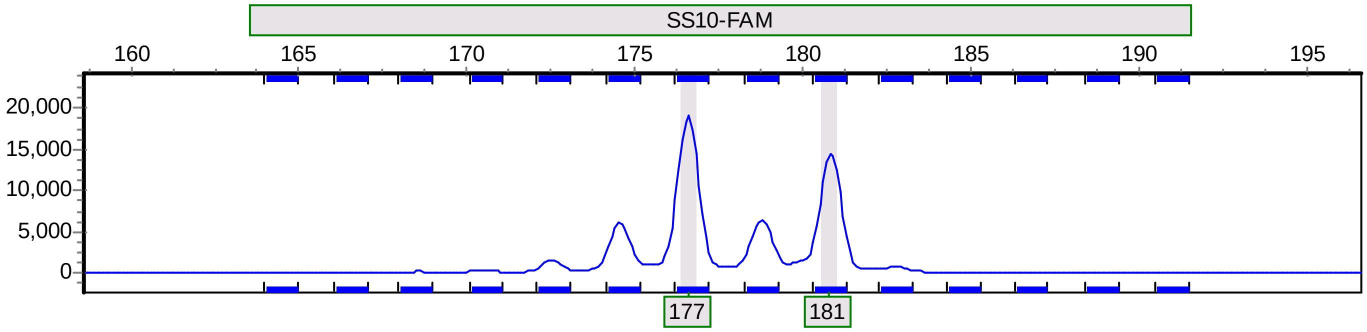

No	Size	Height	Area	Marker	Allele	Difference	Quality	Score	Allele Comments	Sample Comments
1	111.8	16327	109867	SS08-FAM	111	0.30	Pass	500.0	[<Confirmed>]	
2	117.4	5015	36158	SS08-FAM	117	0.00	Pass	500.0	[<Confirmed>]	
3	176.6	18905	133013	SS10-FAM	177	0.10	Pass	500.0	[<Confirmed>]	
4	180.8	14511	105414	SS10-FAM	181	0.00	Pass	500.0	[<Confirmed>]	
5	215.6	13782	99182	SSS42-FAM	216	0.10	Pass	500.0		
6	282.6	20004	172474	SS16-FAM	283	0.00	Pass	500.0		

Sample 13: SS08_SS10_SSS42_SS16_SS27_SS36_SS22_HBB22_K05.fsa

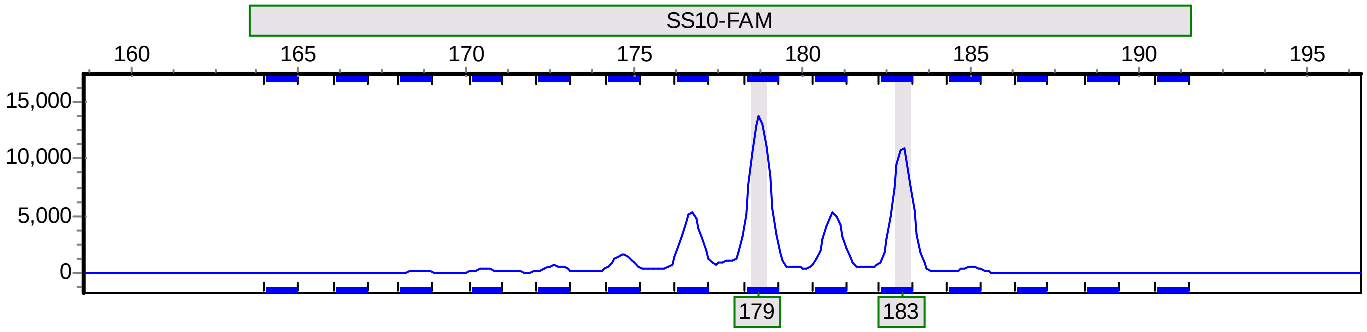

No	Size	Height	Area	Marker	Allele	Difference	Quality	Score	Allele Comments	Sample Comments
1	111.5	12134	79624	SS08-FAM	111	0.00	Pass	500.0	[<Confirmed>]	
2	115.5	12363	79502	SS08-FAM	115	0.10	Pass	500.0	[<Confirmed>]	
3	178.7	13639	93392	SS10-FAM	179	0.10	Pass	500.0	[<Confirmed>]	
4	183.0	10829	73374	SS10-FAM	183	0.20	Pass	500.0	[<Confirmed>]	
5	222.3	13363	97304	SSS42-FAM	222	0.00	Pass	500.0		
6	286.4	11108	89807	SS16-FAM	287	0.10	Pass	500.0		

Sample 14: SS08_SS10_SSS42_SS16_SS27_SS36_SS22_HBB23_B09.fsa

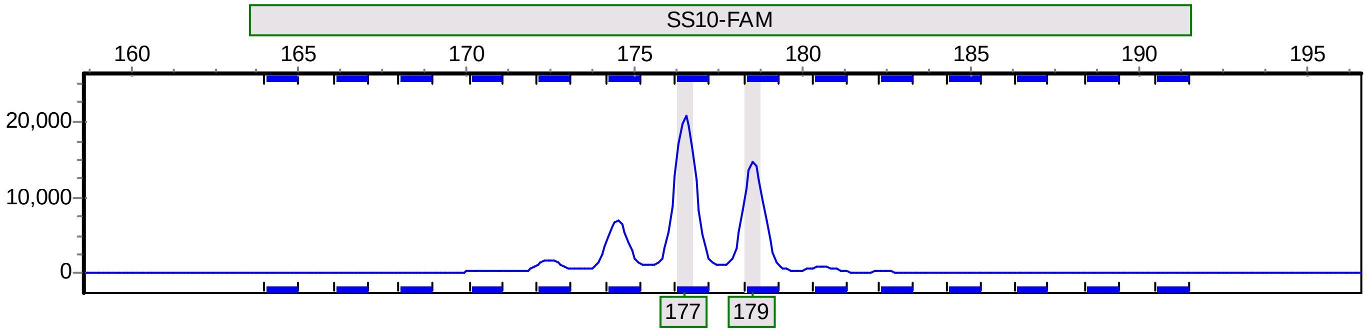

No	Size	Height	Area	Marker	Allele	Difference	Quality	Score	Allele Comments	Sample Comments
1	115.4	19410	131702	SS08-FAM	115	0.00	Pass	500.0	[<Confirmed>]	
2	117.4	13964	95119	SS08-FAM	117	0.00	Pass	500.0	[<Confirmed>]	

3	176.5	20692	145723	SS10-FAM	177	0.20	Pass	500.0	[<Confirmed>]	
4	178.5	14561	103917	SS10-FAM	179	0.30	Pass	500.0	[<Confirmed>]	
5	224.2	12974	98916	SSS42-FAM	224	0.10	Pass	500.0		
6	294.4	10085	87928	SS16-FAM	295	0.20	Pass	500.0		
7	303.4	3840	38487	SS16-FAM	303	0.20	Pass	402.5		

Sample 15: SS08_SS10_SSS42_SS16_SS27_SS36_SS22_HBB25-1_F09.fsa

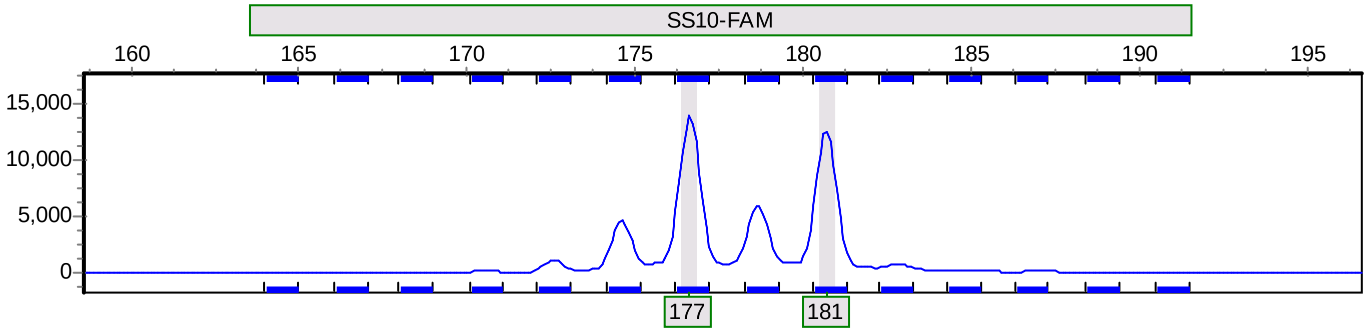

No	Size	Height	Area	Marker	Allele	Difference	Quality	Score	Allele Comments	Sample Comments
1	117.4	17802	121200	SS08-FAM	117	0.00	Pass	500.0	[<Confirmed>]	
2	176.6	13792	98139	SS10-FAM	177	0.10	Pass	500.0	[<Confirmed>]	
3	180.7	12411	90297	SS10-FAM	181	0.10	Pass	500.0	[<Confirmed>]	
4	222.3	3659	28252	SSS42-FAM	222	0.00	Pass	500.0		
5	243.7	8517	68258	SSS42-FAM	244	0.00	Pass	500.0		
6	303.5	3437	31986	SS16-FAM	303	0.10	Pass	385.3		

Sample 16: SS08_SS10_SSS42_SS16_SS27_SS36_SS22_HBB25-2_N11.fsa

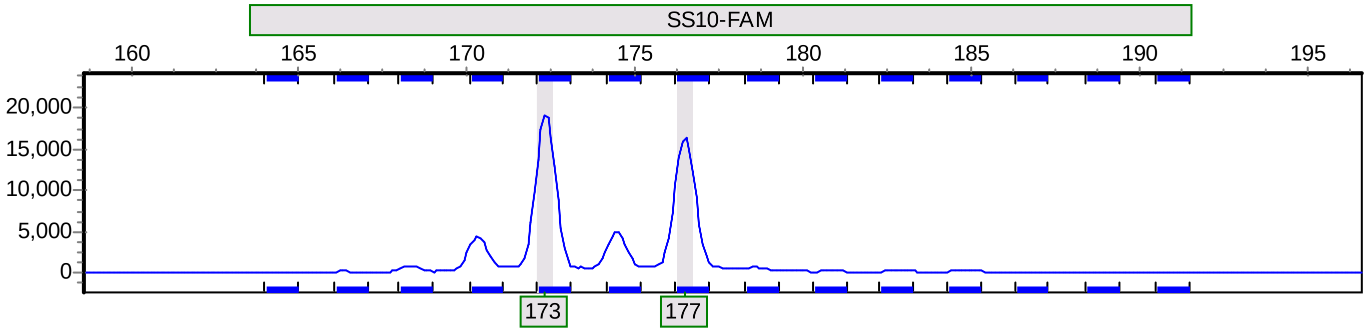

No	Size	Height	Area	Marker	Allele	Difference	Quality	Score	Allele Comments	Sample Comments
1	101.8	26045	170848	SS08-FAM	101	0.00	Pass	500.0	[<Confirmed>]	
2	172.3	19029	130768	SS10-FAM	173	0.30	Pass	500.0	[<Confirmed>]	
3	176.5	16457	113218	SS10-FAM	177	0.20	Pass	500.0	[<Confirmed>]	
4	215.4	9196	69416	SSS42-FAM	216	0.10	Pass	500.0		
5	218.3	9212	65636	SSS42-FAM	218	0.10	Pass	500.0		
6	290.2	18794	157585	SS16-FAM	291	0.30	Pass	500.0		

Allele Report

Sample 17: SS08_SS10_SSS42_SS16_SS27_SS36_SS22_HBB26_D07.fsa

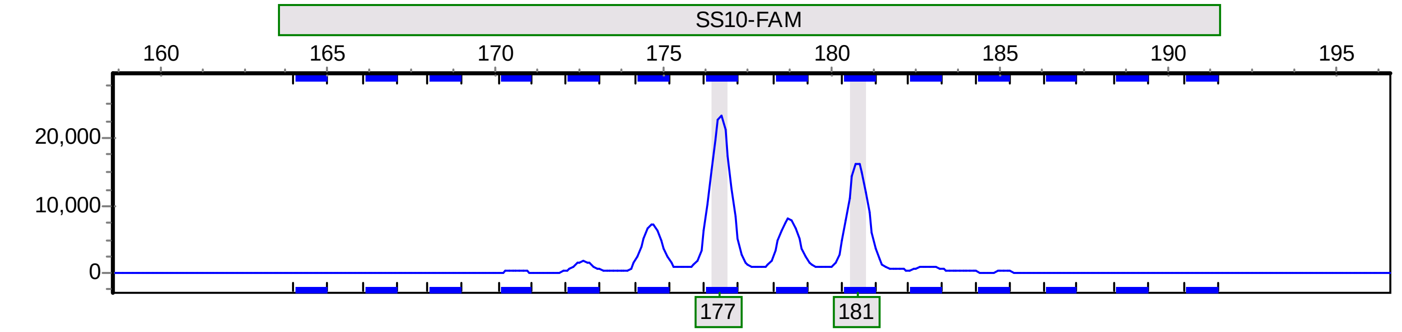

No	Size	Height	Area	Marker	Allele	Difference	Quality	Score	Allele Comments	Sample Comments
1	113.5	15818	104231	SS08-FAM	113	0.10	Pass	500.0	[<Confirmed>]	
2	117.4	12343	84228	SS08-FAM	117	0.00	Pass	500.0	[<Confirmed>]	
3	176.7	23203	160567	SS10-FAM	177	0.00	Pass	500.0	[<Confirmed>]	
4	180.8	16315	115741	SS10-FAM	181	0.00	Pass	500.0	[<Confirmed>]	
5	224.4	10219	74382	SSS42-FAM	224	0.10	Pass	500.0		
6	230.3	13424	98755	SSS42-FAM	230	0.10	Pass	500.0		
7	303.5	5121	50280	SS16-FAM	303	0.10	Pass	500.0		

Sample 18: SS08_SS10_SSS42_SS16_SS27_SS36_SS22_HBB27_H05.fsa

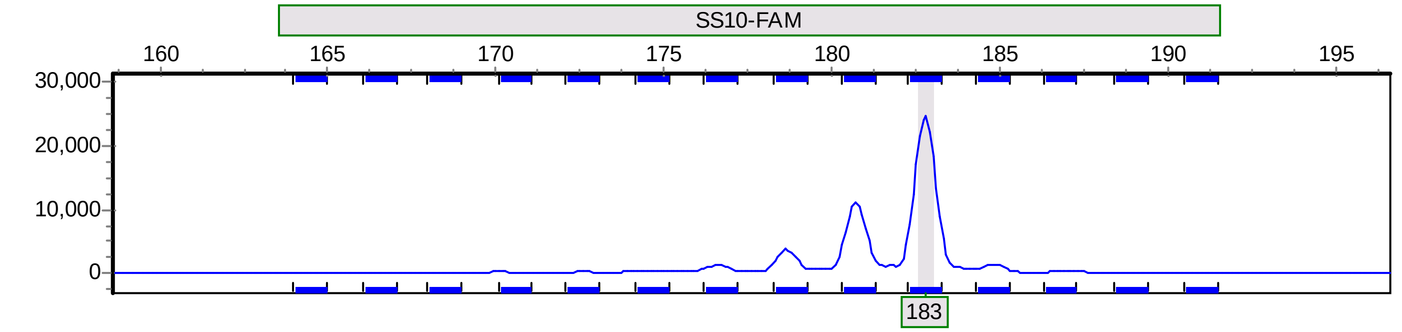

No	Size	Height	Area	Marker	Allele	Difference	Quality	Score	Allele Comments	Sample Comments
1	115.3	12858	89290	SS08-FAM	115	0.10	Pass	500.0	[<Confirmed>]	
2	117.5	13017	89432	SS08-FAM	117	0.10	Pass	500.0	[<Confirmed>]	
3	182.8	24569	175091	SS10-FAM	183	0.00	Pass	500.0	[<Confirmed>]	
4	222.5	10540	75388	SSS42-FAM	222	0.20	Pass	500.0		
5	228.3	15465	113144	SSS42-FAM	228	0.10	Pass	500.0		
6	294.6	11207	96158	SS16-FAM	295	0.00	Pass	500.0		

Sample 19: SS08_SS10_SSS42_SS16_SS27_SS36_SS22_HBB28_L11.fsa

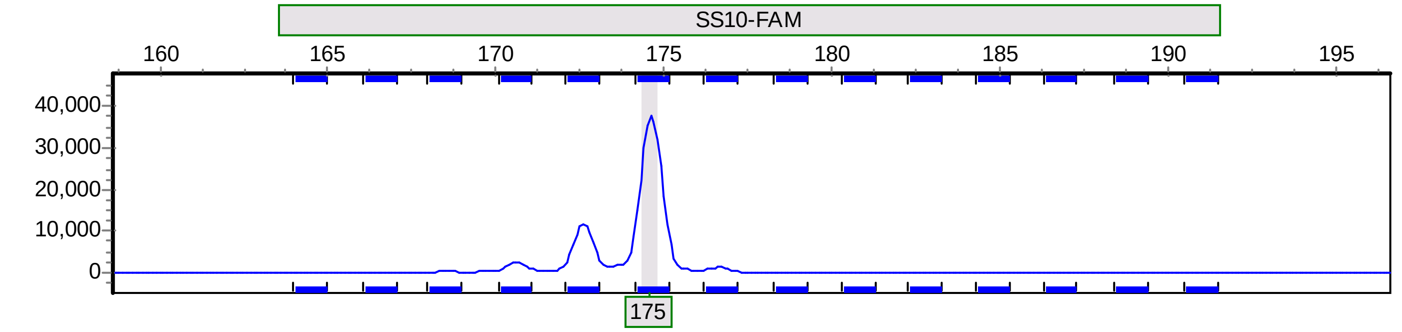

No	Size	Height	Area	Marker	Allele	Difference	Quality	Score	Allele Comments	Sample Comments
1	113.6	20848	139482	SS08-FAM	113	0.20	Pass	500.0	[<Confirmed>]	
2	117.6	16621	111489	SS08-FAM	117	0.20	Pass	500.0	[<Confirmed>]	

3	174.6	37313	273158	SS10-FAM	175	0.10	Pass	500.0	[<SAT (Repaired)><Confirmed>]	
4	230.2	22359	164010	SSS42-FAM	230	0.00	Pass	500.0		
5	294.4	13829	117072	SS16-FAM	295	0.20	Pass	500.0		

Sample 20: SS08_SS10_SSS42_SS16_SS27_SS36_SS22_HBB2_C07.fsa

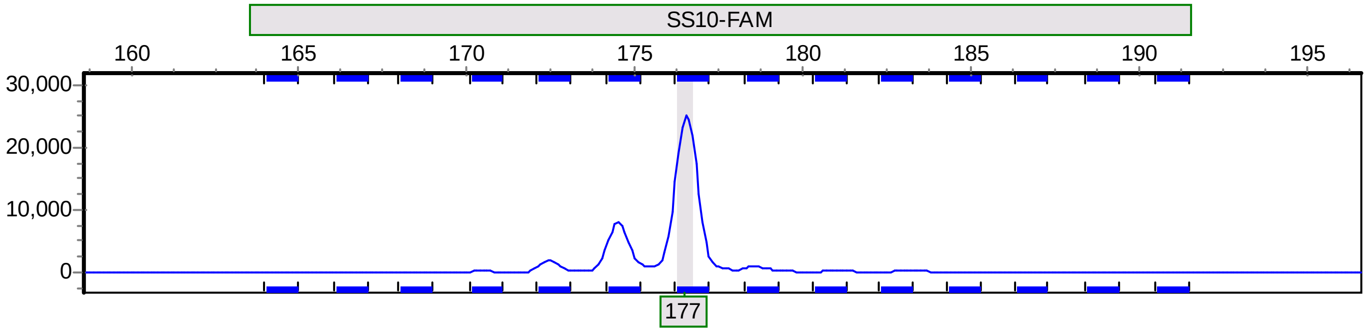

No	Size	Height	Area	Marker	Allele	Difference	Quality	Score	Allele Comments	Sample Comments
1	115.5	21105	142708	SS08-FAM	115	0.10	Pass	500.0	[<Confirmed>]	
2	176.5	24959	182587	SS10-FAM	177	0.20	Pass	500.0	[<Confirmed>]	
3	215.4	11194	79098	SSS42-FAM	216	0.10	Pass	500.0		
4	282.6	14920	121831	SS16-FAM	283	0.00	Pass	500.0		

Sample 21: SS08_SS10_SSS42_SS16_SS27_SS36_SS22_HBB30_L13.fsa 8

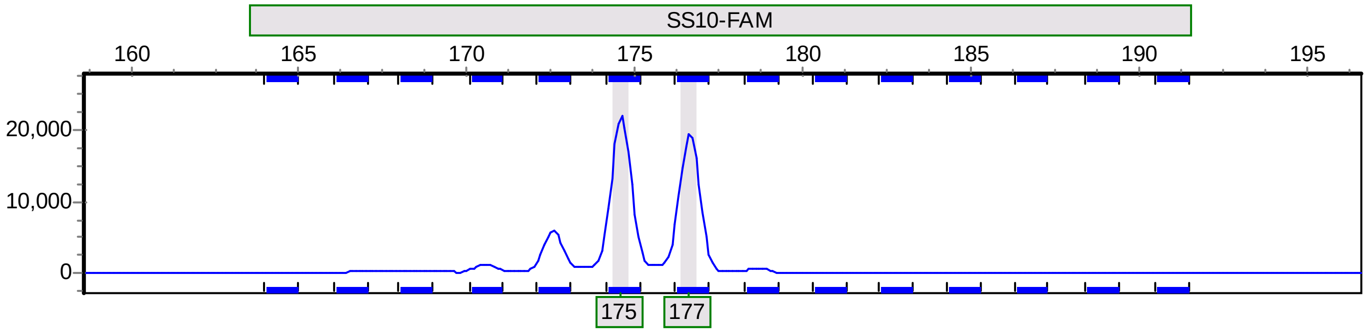

No	Size	Height	Area	Marker	Allele	Difference	Quality	Score	Allele Comments	Sample Comments
1	117.3	16610	107770	SS08-FAM	117	0.10	Pass	500.0	[<Confirmed>]	
2	174.6	21998	150543	SS10-FAM	175	0.10	Pass	500.0	[<Confirmed>]	
3	176.6	19497	133034	SS10-FAM	177	0.10	Pass	500.0	[<Confirmed>]	
4	224.2	9542	70059	SSS42-FAM	224	0.10	Pass	500.0		
5	228.2	14480	107263	SSS42-FAM	228	0.00	Pass	500.0		
6	303.5	9413	88969	SS16-FAM	303	0.10	Pass	500.0		

Sample 22: SS08_SS10_SSS42_SS16_SS27_SS36_SS22_HBB31_P07.fsa

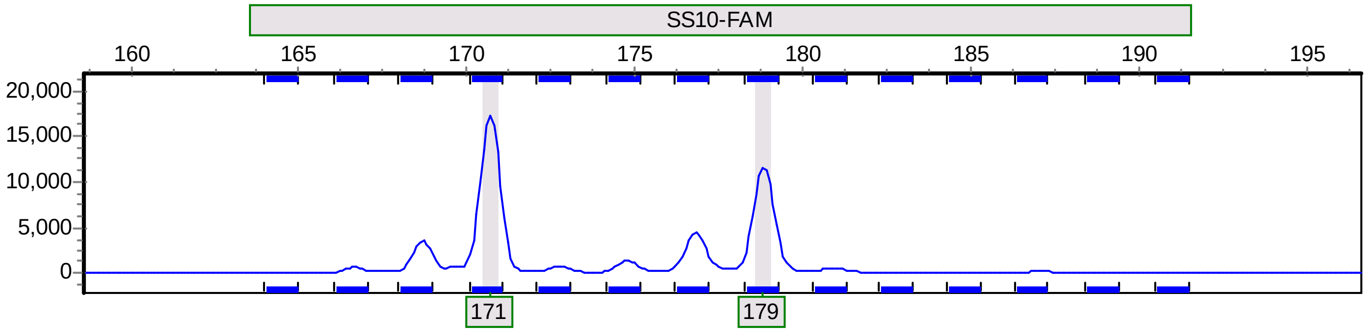

No	Size	Height	Area	Marker	Allele	Difference	Quality	Score	Allele Comments	Sample Comments
1	115.4	17693	118272	SS08-FAM	115	0.00	Pass	500.0	[<Confirmed>]	
2	170.7	17172	112216	SS10-FAM	171	0.10	Pass	500.0	[<Confirmed>]	

3	178.8	11552	79825	SS10-FAM	179	0.00	Pass	500.0	[<Confirmed>]	
4	224.4	8341	59632	SSS42-FAM	224	0.10	Pass	500.0		
5	230.3	10519	74586	SSS42-FAM	230	0.10	Pass	500.0		
6	294.6	8085	70245	SS16-FAM	295	0.00	Pass	500.0		

Sample 23: SS08_SS10_SSS42_SS16_SS27_SS36_SS22_HBB32_B15.fsa

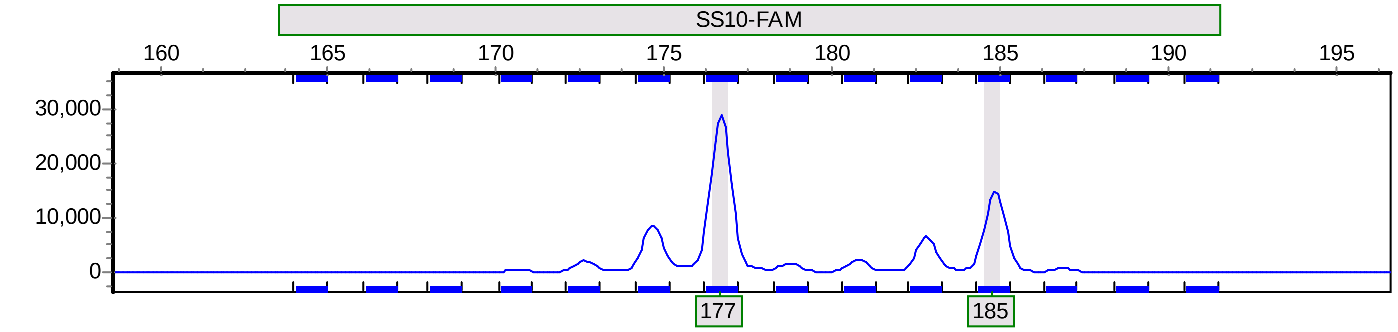

No	Size	Height	Area	Marker	Allele	Difference	Quality	Score	Allele Comments	Sample Comments
1	115.4	28024	188833	SS08-FAM	115	0.00	Pass	500.0	[<Confirmed>]	
2	176.7	28563	198000	SS10-FAM	177	0.00	Pass	500.0	[<Confirmed>]	
3	184.8	14599	104160	SS10-FAM	185	0.00	Pass	500.0	[<Confirmed>]	
4	230.2	30888	231352	SSS42-FAM	230	0.00	Check	500.0		
5	303.6	11773	113592	SS16-FAM	303	0.00	Pass	500.0		

Sample 24: SS08_SS10_SSS42_SS16_SS27_SS36_SS22_HBB33_N13.fsa

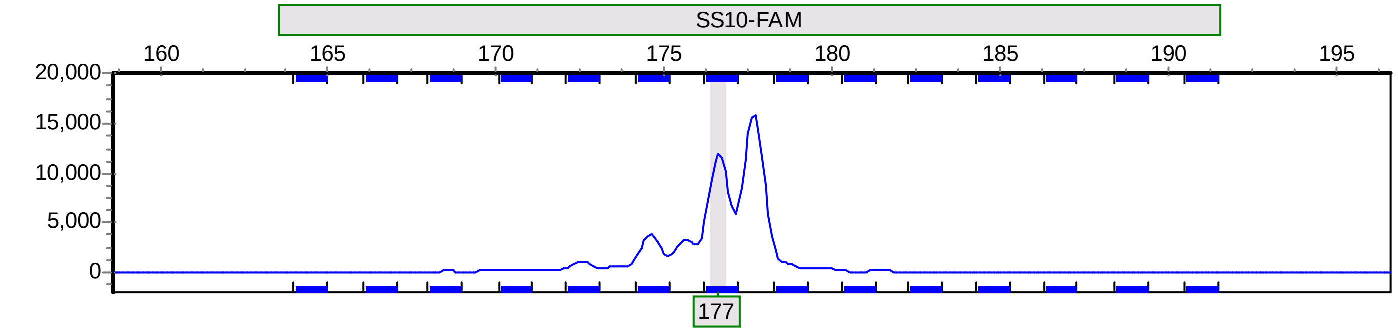

No	Size	Height	Area	Marker	Allele	Difference	Quality	Score	Allele Comments	Sample Comments
1	117.4	4826	36626	SS08-FAM	117	0.00	Pass	500.0	[<Confirmed>]	
2	176.6	11941	84041	SS10-FAM	177	0.10	Pass	500.0	[<Confirmed>]	
3	243.6	3369	26579	SSS42-FAM	244	0.10	Pass	500.0		
4	294.4	11434	105263	SS16-FAM	295	0.20	Pass	500.0		

Sample 25: SS08_SS10_SSS42_SS16_SS27_SS36_SS22_HBB34_P13.fsa

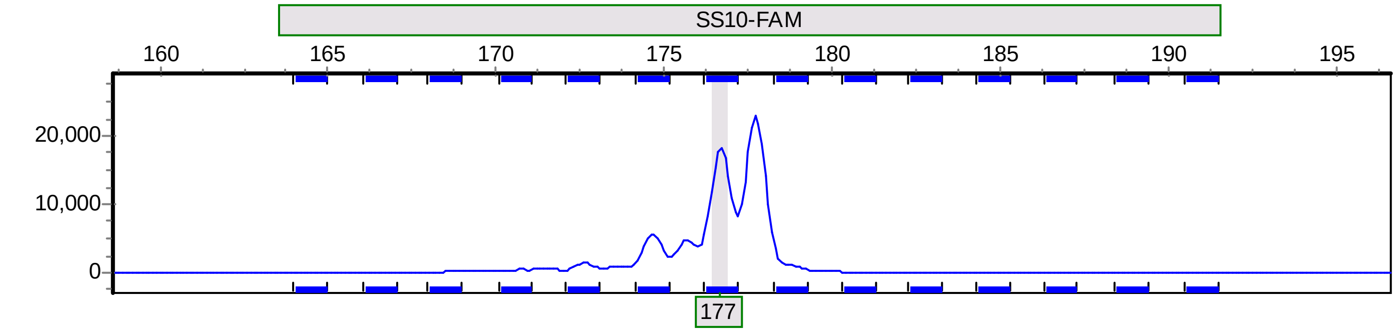

No	Size	Height	Area	Marker	Allele	Difference	Quality	Score	Allele Comments	Sample Comments
1	115.4	14323	100548	SS08-FAM	115	0.00	Pass	500.0	[<Confirmed>]	
2	117.6	14184	97564	SS08-FAM	117	0.20	Pass	500.0	[<Confirmed>]	

3	176.7	18055	125887	SS10-FAM	177	0.00	Pass	500.0	[<Confirmed>]	
4	243.6	4402	35374	SSS42-FAM	244	0.10	Pass	500.0		
5	294.5	10383	95611	SS16-FAM	295	0.10	Pass	500.0		
6	303.5	4270	42482	SS16-FAM	303	0.10	Pass	466.6		

Sample 26: SS08_SS10_SSS42_SS16_SS27_SS36_SS22_HBB35_C15.fsa

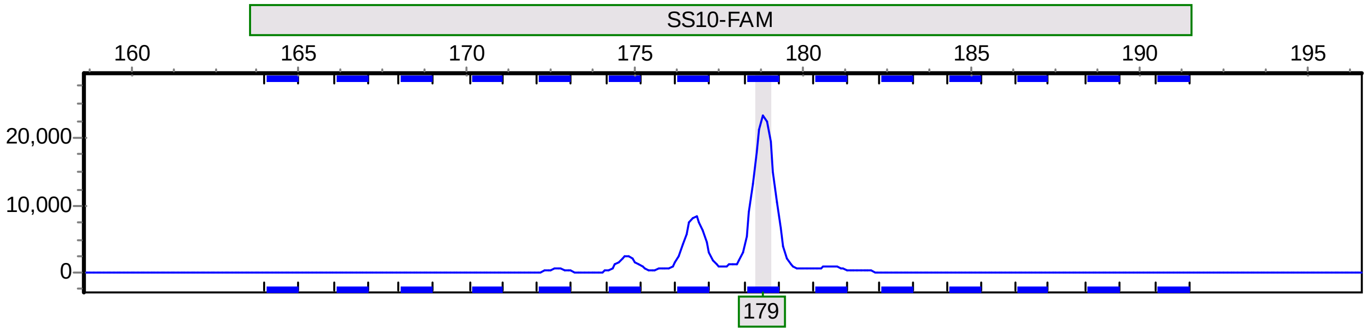

No	Size	Height	Area	Marker	Allele	Difference	Quality	Score	Allele Comments	Sample Comments
1	115.4	14327	97693	SS08-FAM	115	0.00	Pass	500.0	[<Confirmed>]	
2	117.4	11293	76111	SS08-FAM	117	0.00	Pass	500.0	[<Confirmed>]	
3	178.8	23216	163306	SS10-FAM	179	0.00	Pass	500.0	[<Confirmed>]	
4	224.2	8978	66702	SSS42-FAM	224	0.10	Pass	500.0		
5	249.5	3875	30040	SSS42-FAM	250	0.10	Pass	500.0		
6	303.6	4045	39006	SS16-FAM	303	0.00	Pass	465.3		

Sample 27: SS08_SS10_SSS42_SS16_SS27_SS36_SS22_HBB36_G05.fsa

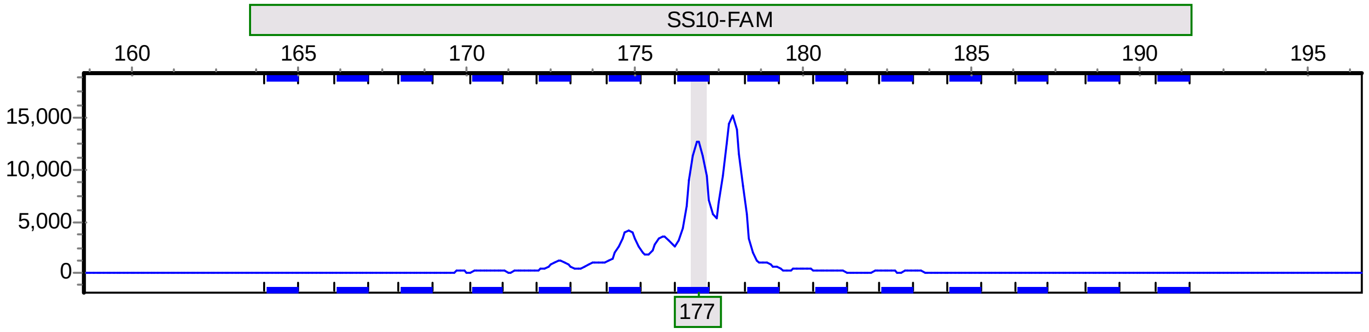

No	Size	Height	Area	Marker	Allele	Difference	Quality	Score	Allele Comments	Sample Comments
1	115.3	10943	75644	SS08-FAM	115	0.10	Pass	500.0	[<Confirmed>]	
2	117.5	10465	71682	SS08-FAM	117	0.10	Pass	500.0	[<Confirmed>]	
3	176.9	12759	87034	SS10-FAM	177	0.20	Pass	500.0	[<Confirmed>]	
4	230.1	16486	118177	SSS42-FAM	230	0.10	Pass	500.0		
5	303.6	5119	48614	SS16-FAM	303	0.00	Pass	500.0		

Sample 28: SS08_SS10_SSS42_SS16_SS27_SS36_SS22_HBB37_N09.fsa

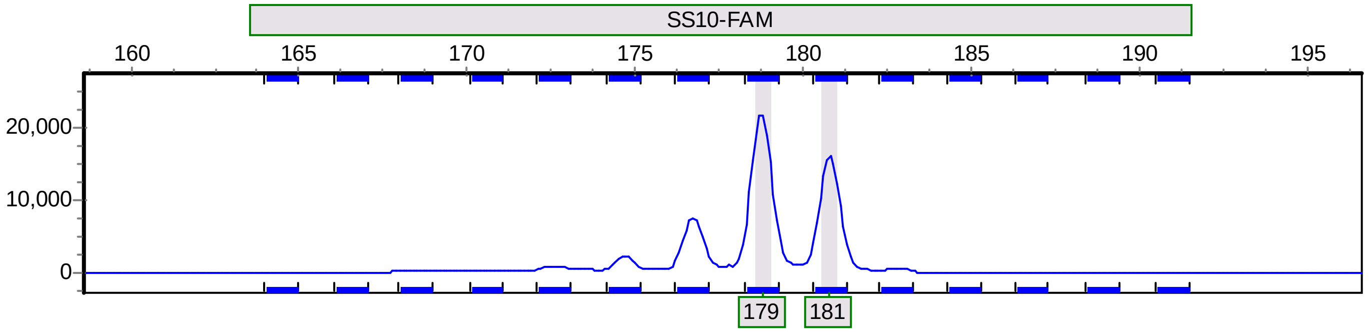

No	Size	Height	Area	Marker	Allele	Difference	Quality	Score	Allele Comments	Sample Comments

1	115.3	5170	37198	SS08-FAM	115	0.10	Pass	500.0	[<Confirmed>]	
2	178.8	21431	152144	SS10-FAM	179	0.00	Pass	500.0	[<Confirmed>]	
3	180.8	15927	112637	SS10-FAM	181	0.00	Pass	500.0	[<Confirmed>]	
4	224.3	10307	78209	SSS42-FAM	224	0.00	Pass	500.0		
5	243.7	4212	34221	SSS42-FAM	244	0.00	Pass	500.0		

Sample 29: SS08_SS10_SSS42_SS16_SS27_SS36_SS22_HBB38_M07.fsa

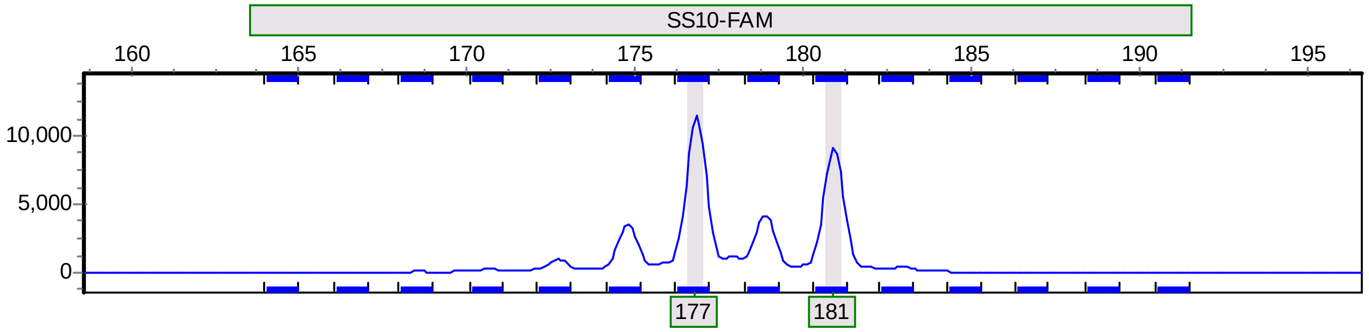

No	Size	Height	Area	Marker	Allele	Difference	Quality	Score	Allele Comments	Sample Comments
1	117.5	15497	103783	SS08-FAM	117	0.10	Pass	500.0	[<Confirmed>]	
2	176.8	11483	78748	SS10-FAM	177	0.10	Pass	500.0	[<Confirmed>]	
3	180.9	9126	63848	SS10-FAM	181	0.10	Pass	500.0	[<Confirmed>]	
4	222.4	3060	22319	SSS42-FAM	222	0.10	Pass	225.7		
5	294.7	5232	46322	SS16-FAM	295	0.10	Pass	500.0		

Sample 30: SS08_SS10_SSS42_SS16_SS27_SS36_SS22_HBB39_F05.fsa

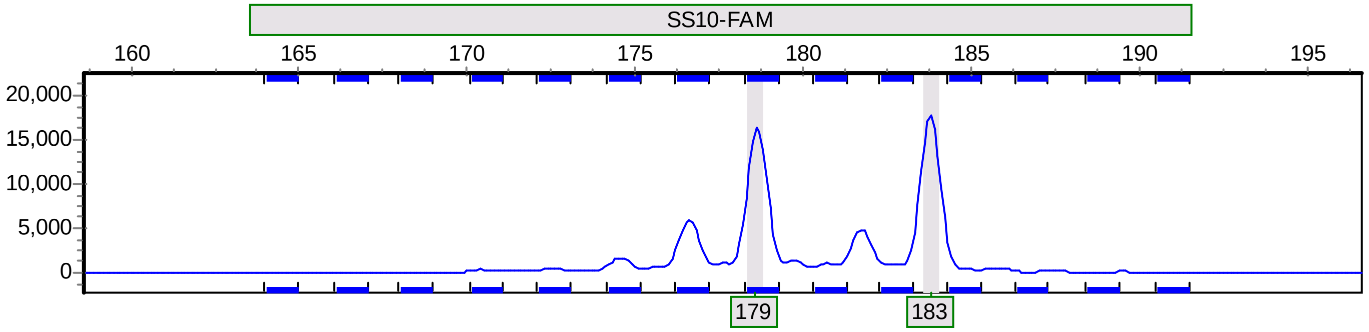

No	Size	Height	Area	Marker	Allele	Difference	Quality	Score	Allele Comments	Sample Comments
1	115.4	27358	188064	SS08-FAM	115	0.00	Pass	500.0	[<Confirmed>]	
2	178.6	16306	110261	SS10-FAM	179	0.20	Pass	500.0	[<Confirmed>]	
3	183.8	17622	119342	SS10-FAM	183	1.00	Pass	500.0	[<Confirmed><Edited>]	
4	222.3	7291	52238	SSS42-FAM	222	0.00	Pass	500.0		
5	249.6	9901	75283	SSS42-FAM	250	0.00	Pass	500.0		
6	294.5	15770	132056	SS16-FAM	295	0.10	Pass	500.0		

Allele Report

Sample 31: SS08_SS10_SSS42_SS16_SS27_SS36_SS22_HBB40_D15.fsa

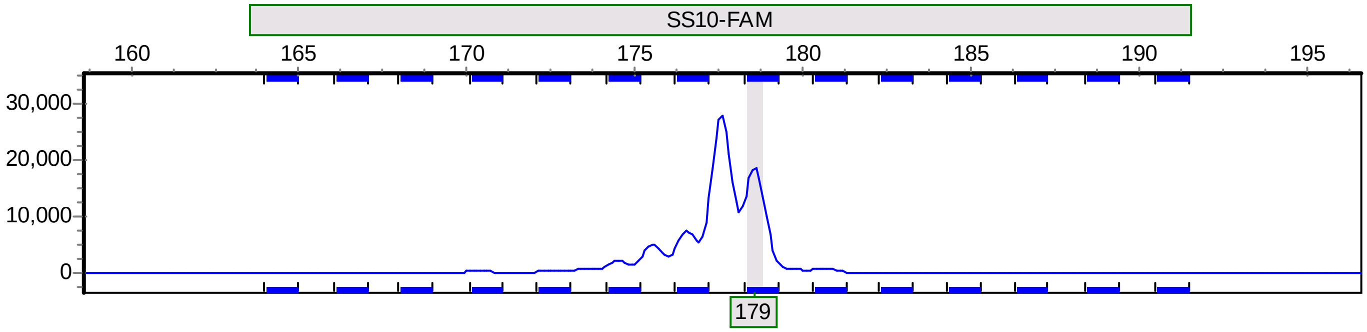

No	Size	Height	Area	Marker	Allele	Difference	Quality	Score	Allele Comments	Sample Comments
1	115.5	21947	148897	SS08-FAM	115	0.10	Pass	500.0	[<Confirmed>]	
2	117.5	17292	117422	SS08-FAM	117	0.10	Pass	500.0	[<Confirmed>]	
3	178.6	18739	129759	SS10-FAM	179	0.20	Pass	500.0	[<Confirmed>]	
4	230.2	13405	102013	SSS42-FAM	230	0.00	Pass	500.0		
5	294.5	23039	206795	SS16-FAM	295	0.10	Pass	500.0		

Sample 32: SS08_SS10_SSS42_SS16_SS27_SS36_SS22_HBB41_L07.fsa

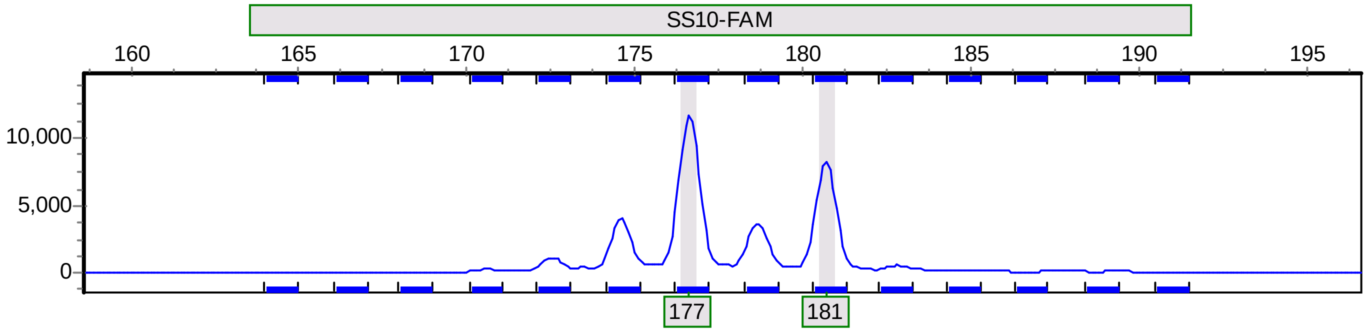

No	Size	Height	Area	Marker	Allele	Difference	Quality	Score	Allele Comments	Sample Comments
1	115.4	7991	54773	SS08-FAM	115	0.00	Pass	500.0	[<Confirmed>]	
2	117.4	7088	49537	SS08-FAM	117	0.00	Pass	500.0	[<Confirmed>]	
3	176.6	11641	82118	SS10-FAM	177	0.10	Pass	500.0	[<Confirmed>]	
4	180.7	8211	58344	SS10-FAM	181	0.10	Pass	500.0	[<Confirmed>]	
5	230.2	8237	63701	SSS42-FAM	230	0.00	Pass	500.0		
6	245.7	3891	30841	SSS42-FAM	246	0.00	Pass	500.0		
7	294.5	5644	51814	SS16-FAM	295	0.10	Pass	500.0		

Sample 33: SS08_SS10_SSS42_SS16_SS27_SS36_SS22_HBB42_M05.fsa

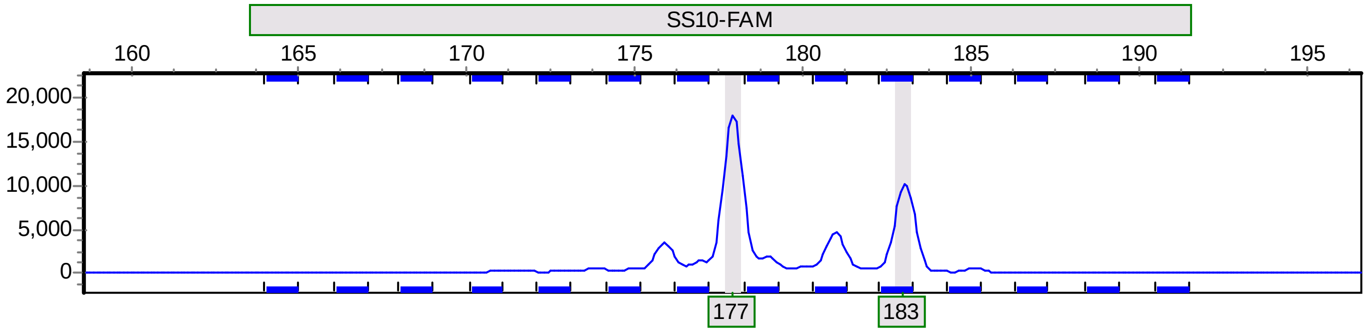

No	Size	Height	Area	Marker	Allele	Difference	Quality	Score	Allele Comments	Sample Comments
1	117.4	11983	78847	SS08-FAM	117	0.00	Pass	500.0	[<Confirmed>]	
2	125.1	6945	46277	SS08-FAM	125	0.00	Pass	500.0	[<Confirmed>]	
3	177.9	17941	120285	SS10-FAM	177	1.00	Pass	500.0	[<Confirmed><Edited>]	

Allele Report

4	183.0	10150	69606	SS10-FAM	183	0.20	Pass	500.0	[<Confirmed>]	
5	228.4	10050	70592	SSS42-FAM	228	0.20	Pass	500.0		
6	245.9	4461	32924	SSS42-FAM	246	0.20	Pass	500.0		
7	294.8	12978	109072	SS16-FAM	295	0.20	Pass	500.0		

Sample 34: SS08_SS10_SSS42_SS16_SS27_SS36_SS22_HBB43_D09.fsa

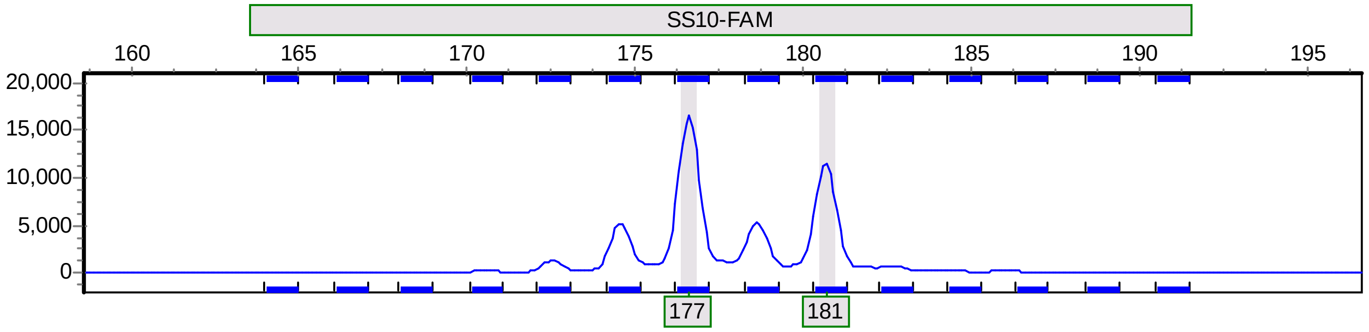

No	Size	Height	Area	Marker	Allele	Difference	Quality	Score	Allele Comments	Sample Comments
1	115.3	11690	84989	SS08-FAM	115	0.10	Pass	500.0	[<Confirmed>]	
2	117.5	12549	88460	SS08-FAM	117	0.10	Pass	500.0	[<Confirmed>]	
3	176.6	16436	117472	SS10-FAM	177	0.10	Pass	500.0	[<Confirmed>]	
4	180.7	11420	85287	SS10-FAM	181	0.10	Pass	500.0	[<Confirmed>]	
5	230.1	14714	114476	SSS42-FAM	230	0.10	Pass	500.0		
6	299.4	3198	31693	SS16-FAM	299	0.10	Pass	304.9		

Sample 35: SS08_SS10_SSS42_SS16_SS27_SS36_SS22_HBB44_H11.fsa

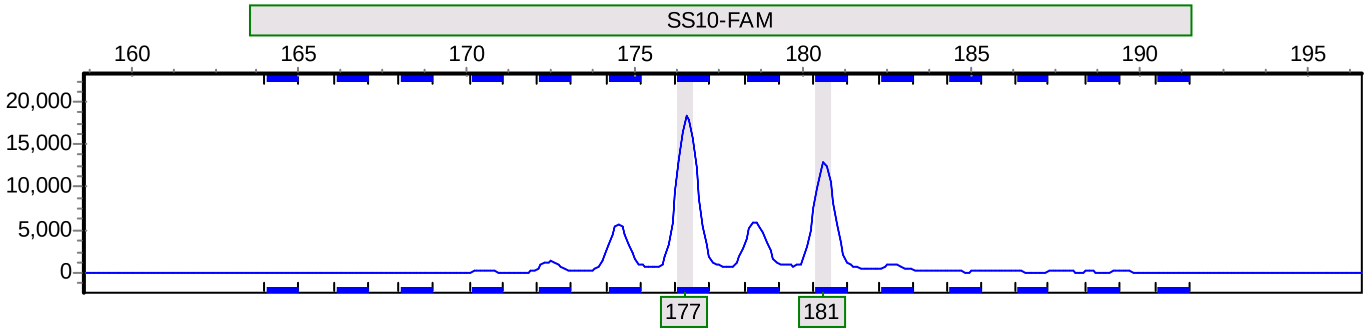

No	Size	Height	Area	Marker	Allele	Difference	Quality	Score	Allele Comments	Sample Comments
1	115.5	14508	99072	SS08-FAM	115	0.10	Pass	500.0	[<Confirmed>]	
2	117.5	10966	78805	SS08-FAM	117	0.10	Pass	500.0	[<Confirmed>]	
3	176.5	18156	125346	SS10-FAM	177	0.20	Pass	500.0	[<Confirmed>]	
4	180.6	12793	90030	SS10-FAM	181	0.20	Pass	500.0	[<Confirmed>]	
5	230.2	11448	86703	SSS42-FAM	230	0.00	Pass	500.0		
6	294.5	16336	140126	SS16-FAM	295	0.10	Pass	500.0		

Allele Report

Sample 36: SS08_SS10_SSS42_SS16_SS27_SS36_SS22_HBB45_F13.fsa

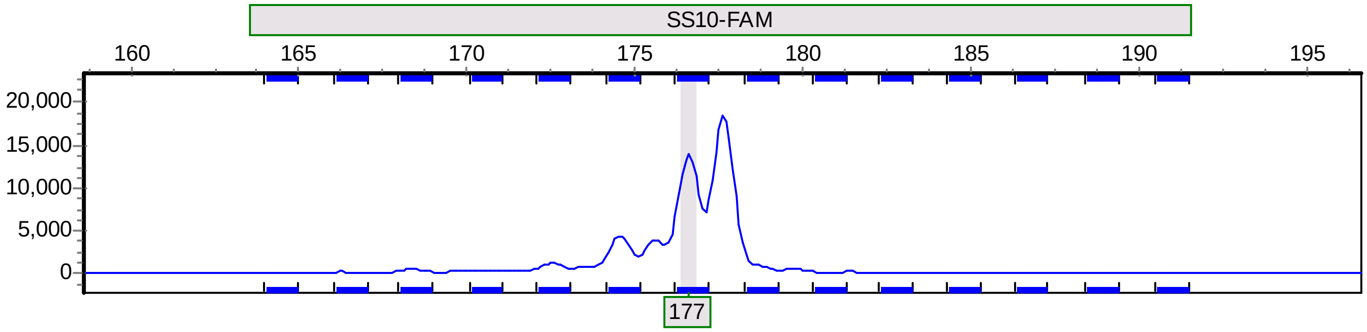

No	Size	Height	Area	Marker	Allele	Difference	Quality	Score	Allele Comments	Sample Comments
1	115.5	14697	100498	SS08-FAM	115	0.10	Pass	500.0	[<Confirmed>]	
2	117.5	10917	76512	SS08-FAM	117	0.10	Pass	500.0	[<Confirmed>]	
3	176.6	13905	99861	SS10-FAM	177	0.10	Pass	500.0	[<Confirmed>]	
4	230.2	11781	91801	SSS42-FAM	230	0.00	Pass	500.0		
5	294.5	11782	105837	SS16-FAM	295	0.10	Pass	500.0		

Sample 37: SS08_SS10_SSS42_SS16_SS27_SS36_SS22_HBB46_F07.fsa

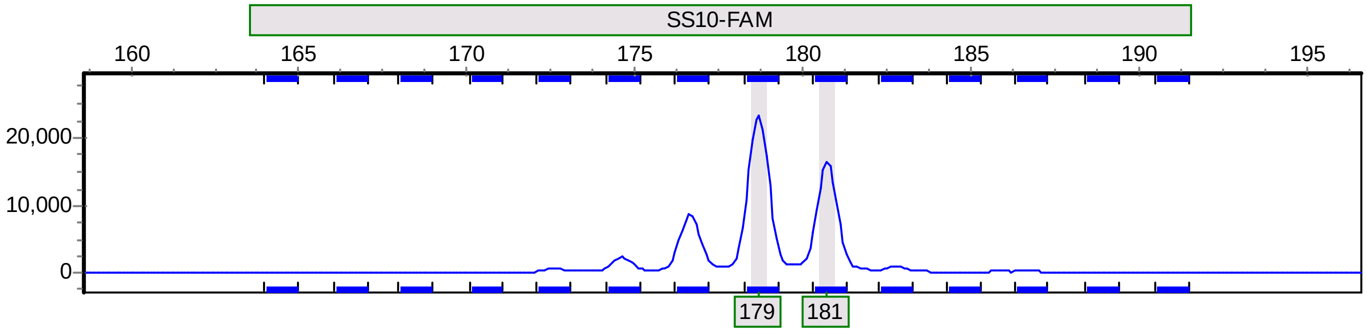

No	Size	Height	Area	Marker	Allele	Difference	Quality	Score	Allele Comments	Sample Comments
1	117.5	19380	132170	SS08-FAM	117	0.10	Pass	500.0	[<Confirmed>]	
2	119.4	11179	77625	SS08-FAM	119	0.10	Pass	500.0	[<Confirmed>]	
3	178.7	23233	162227	SS10-FAM	179	0.10	Pass	500.0	[<Confirmed>]	
4	180.7	16542	114431	SS10-FAM	181	0.10	Pass	500.0	[<Confirmed>]	
5	228.3	15363	113979	SSS42-FAM	228	0.10	Pass	500.0		
6	294.5	15763	139555	SS16-FAM	295	0.10	Pass	500.0		
7	303.3	5809	55340	SS16-FAM	303	0.30	Pass	500.0		

Sample 38: SS08_SS10_SSS42_SS16_SS27_SS36_SS22_HBB47_A05.fsa

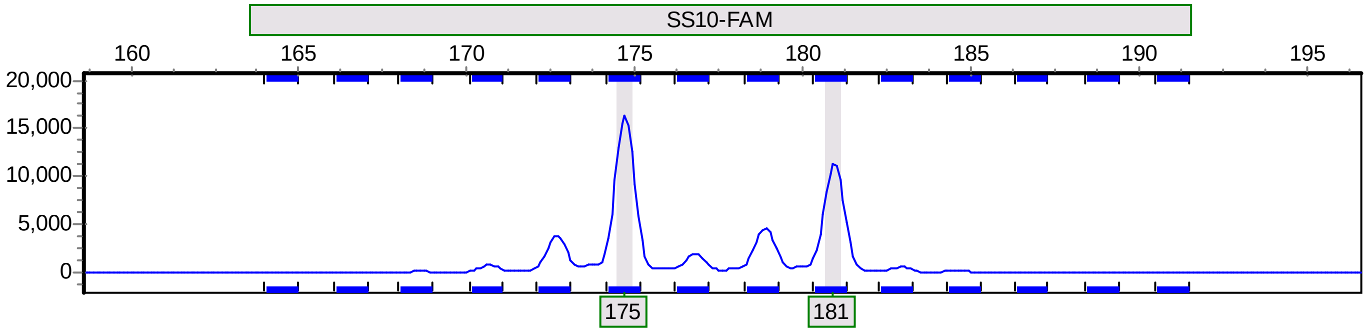

No	Size	Height	Area	Marker	Allele	Difference	Quality	Score	Allele Comments	Sample Comments
1	117.4	25356	167734	SS08-FAM	117	0.00	Pass	500.0	[<Confirmed>]	
2	174.7	16265	108121	SS10-FAM	175	0.00	Pass	500.0	[<Confirmed>]	
3	180.9	11390	78518	SS10-FAM	181	0.10	Pass	500.0	[<Confirmed>]	

No	Size	Height	Area	Marker	Allele	Difference	Quality	Score	Allele Comments	Sample Comments
4	222.3	11428	81119	SSS42-FAM	222	0.00	Pass	500.0		
5	228.0	3884	27402	SSS42-FAM	228	0.20	Pass	500.0		

Sample 39: SS08_SS10_SSS42_SS16_SS27_SS36_SS22_HBB48_B13.fsa

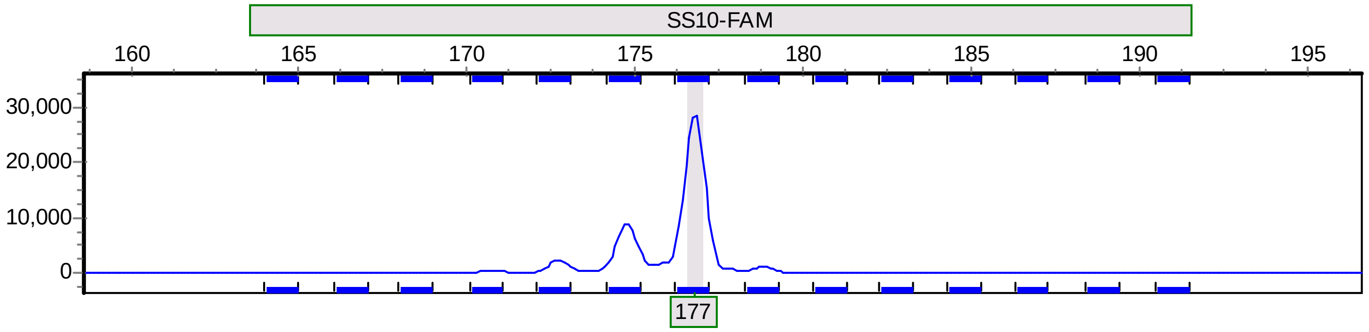

No	Size	Height	Area	Marker	Allele	Difference	Quality	Score	Allele Comments	Sample Comments
1	111.7	16780	113009	SS08-FAM	111	0.20	Pass	500.0	[<Confirmed>]	
2	117.6	16568	110558	SS08-FAM	117	0.20	Pass	500.0	[<Confirmed>]	
3	176.8	28283	198010	SS10-FAM	177	0.10	Pass	500.0	[<Confirmed>]	
4	222.5	8033	59565	SSS42-FAM	222	0.20	Pass	500.0		
5	228.1	9781	75610	SSS42-FAM	228	0.10	Pass	500.0		
6	286.4	17480	155078	SS16-FAM	287	0.10	Pass	500.0		

Sample 40: SS08_SS10_SSS42_SS16_SS27_SS36_SS22_HBB49_L05.fsa

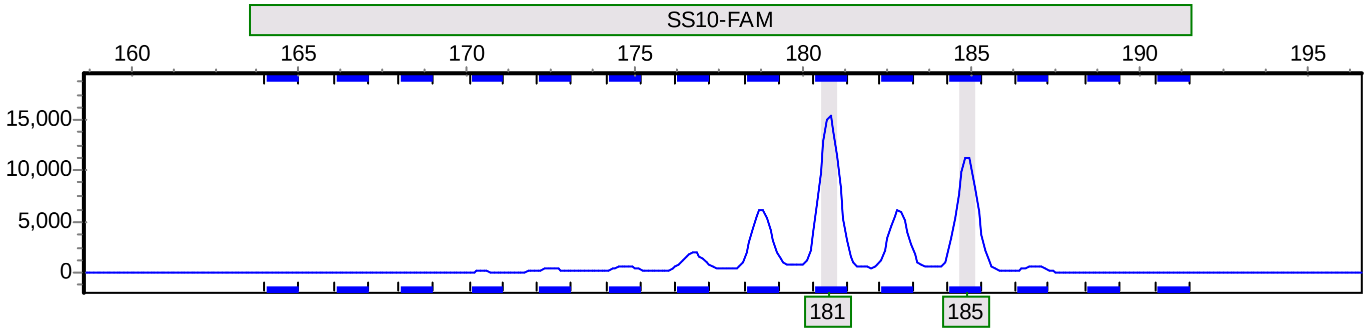

No	Size	Height	Area	Marker	Allele	Difference	Quality	Score	Allele Comments	Sample Comments
1	115.3	11089	76838	SS08-FAM	115	0.10	Pass	500.0	[<Confirmed>]	
2	117.4	11828	78669	SS08-FAM	117	0.00	Pass	500.0	[<Confirmed>]	
3	180.8	15240	103853	SS10-FAM	181	0.00	Pass	500.0	[<Confirmed>]	
4	184.9	11263	78143	SS10-FAM	185	0.10	Pass	500.0	[<Confirmed>]	
5	224.2	7507	54848	SSS42-FAM	224	0.10	Pass	500.0		
6	230.1	10542	77227	SSS42-FAM	230	0.10	Pass	500.0		
7	303.5	5349	51621	SS16-FAM	303	0.10	Pass	500.0		

Sample 41: SS08_SS10_SSS42_SS16_SS27_SS36_SS22_HBB4_E07.fsa

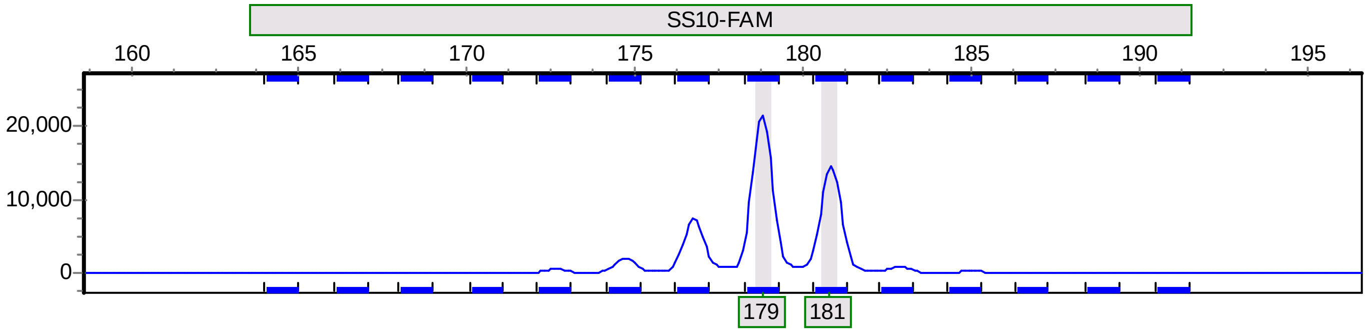

No	Size	Height	Area	Marker	Allele	Difference	Quality	Score	Allele Comments	Sample Comments

Allele Report

1	109.2	13711	89366	SS08-FAM	109	0.10	Pass	500.0	[<Confirmed>]	
2	115.2	13478	89501	SS08-FAM	115	0.20	Pass	500.0	[<Confirmed>]	
3	178.8	21247	144132	SS10-FAM	179	0.00	Pass	500.0	[<Confirmed>]	
4	180.8	14605	101757	SS10-FAM	181	0.00	Pass	500.0	[<Confirmed>]	
5	230.1	13389	94780	SSS42-FAM	230	0.10	Pass	500.0		
6	243.6	4155	31576	SSS42-FAM	244	0.10	Pass	500.0		
7	299.5	4204	38012	SS16-FAM	299	0.00	Pass	500.0		
8	303.6	6687	63844	SS16-FAM	303	0.00	Pass	500.0		

Sample 42: SS08_SS10_SSS42_SS16_SS27_SS36_SS22_HBB5_H13.fsa

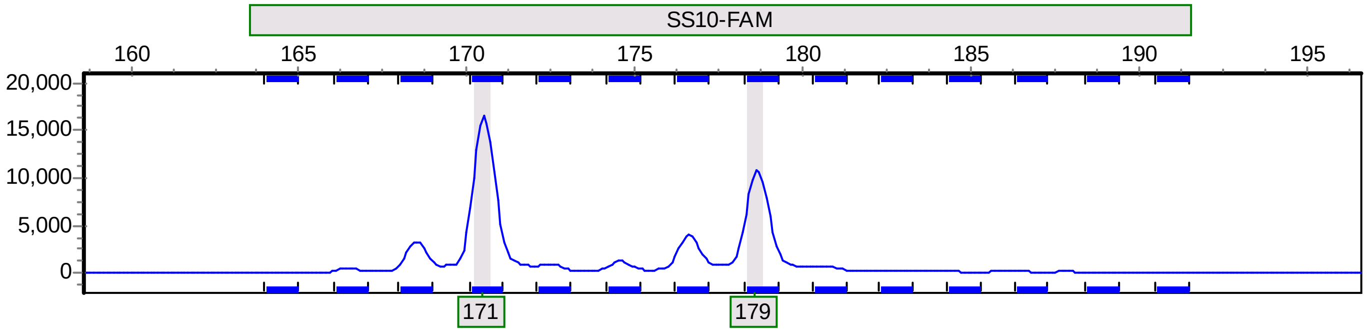

No	Size	Height	Area	Marker	Allele	Difference	Quality	Score	Allele Comments	Sample Comments
1	109.4	10273	73943	SS08-FAM	109	0.10	Pass	500.0	[<Confirmed>]	
2	117.5	10953	77853	SS08-FAM	117	0.10	Pass	500.0	[<Confirmed>]	
3	170.5	16460	120339	SS10-FAM	171	0.10	Pass	500.0	[<Confirmed>]	
4	178.6	10798	83539	SS10-FAM	179	0.20	Pass	500.0	[<Confirmed>]	
5	222.3	13651	113621	SSS42-FAM	222	0.00	Pass	500.0		
6	294.5	6327	61958	SS16-FAM	295	0.10	Pass	500.0		
7	303.4	2620	28112	SS16-FAM	303	0.20	Pass	186.7		

Sample 43: SS08_SS10_SSS42_SS16_SS27_SS36_SS22_HBB6_C05.fsa

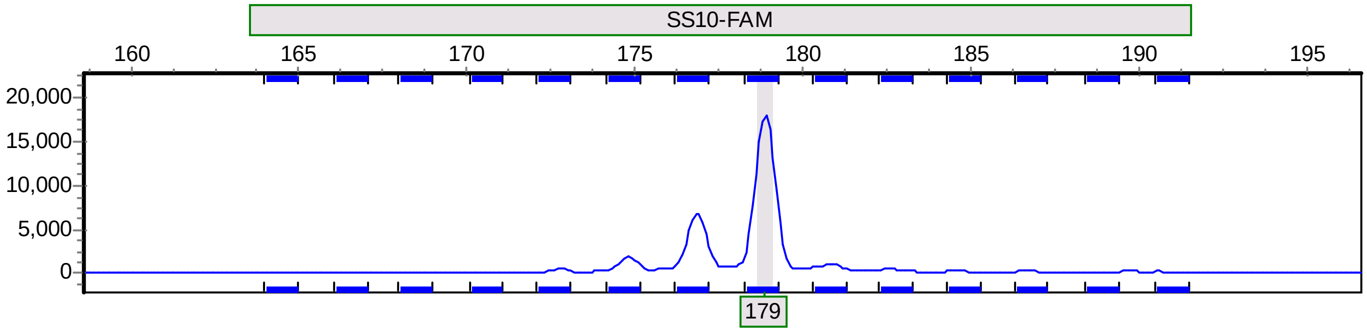

No	Size	Height	Area	Marker	Allele	Difference	Quality	Score	Allele Comments	Sample Comments
1	117.2	9262	62448	SS08-FAM	117	0.20	Pass	500.0	[<Confirmed>]	
2	121.1	10865	70810	SS08-FAM	121	0.00	Pass	500.0	[<Confirmed>]	
3	178.9	17926	119405	SS10-FAM	179	0.10	Pass	500.0	[<Confirmed>]	
4	215.5	13520	90571	SSS42-FAM	216	0.00	Pass	500.0		
5	294.6	7211	58813	SS16-FAM	295	0.00	Pass	500.0		

Allele Report

Sample 44: SS08_SS10_SSS42_SS16_SS27_SS36_SS22_HBB7_N05.fsa

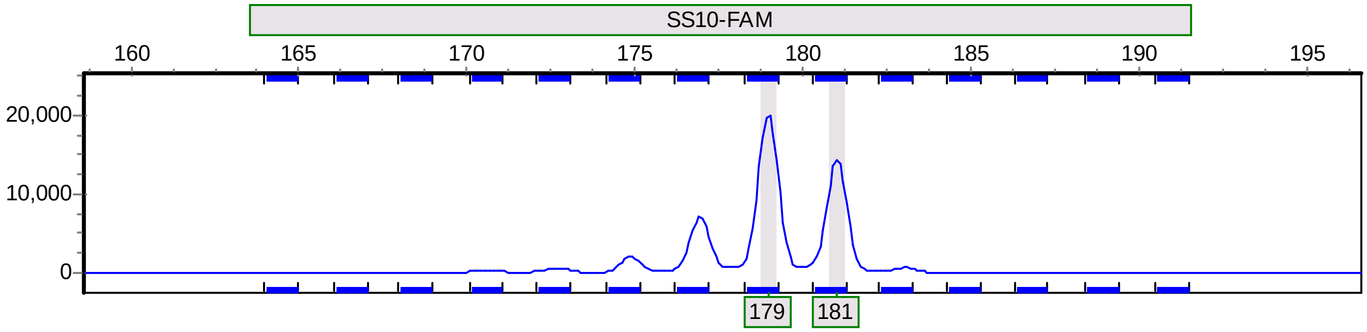

No	Size	Height	Area	Marker	Allele	Difference	Quality	Score	Allele Comments	Sample Comments
1	117.6	26829	175066	SS08-FAM	117	0.20	Pass	500.0	[<Confirmed>]	
2	179.0	20004	137678	SS10-FAM	179	0.20	Pass	500.0	[<Confirmed>]	
3	181.0	14468	99741	SS10-FAM	181	0.20	Pass	500.0	[<Confirmed>]	
4	224.4	15409	110667	SSS42-FAM	224	0.10	Pass	500.0		
5	294.5	8678	76495	SS16-FAM	295	0.10	Pass	500.0		
6	303.5	3759	35453	SS16-FAM	303	0.10	Pass	442.6		

Sample 45: SS08_SS10_SSS42_SS16_SS27_SS36_SS22_HBB8_P11.fsa

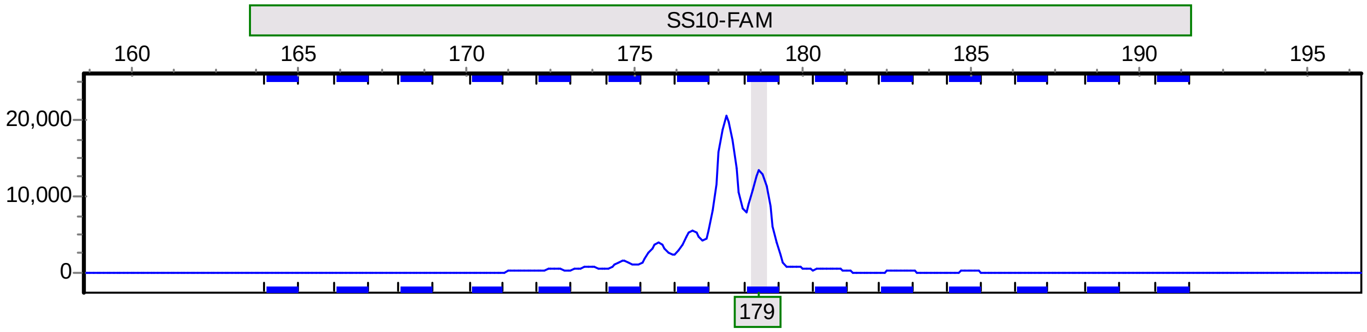

No	Size	Height	Area	Marker	Allele	Difference	Quality	Score	Allele Comments	Sample Comments
1	115.4	31837	225362	SS08-FAM	115	0.00	Pass	500.0	[<SAT (Repaired)><Confirmed>]	
2	178.7	13402	89063	SS10-FAM	179	0.10	Pass	500.0	[<Confirmed>]	
3	218.4	13198	96724	SSS42-FAM	218	0.00	Pass	500.0		
4	230.2	9145	70274	SSS42-FAM	230	0.00	Pass	500.0		
5	282.4	18022	162346	SS16-FAM	283	0.20	Pass	500.0		

Sample 46: SS08_SS10_SSS42_SS16_SS27_SS36_SS22_HBB9_I05.fsa

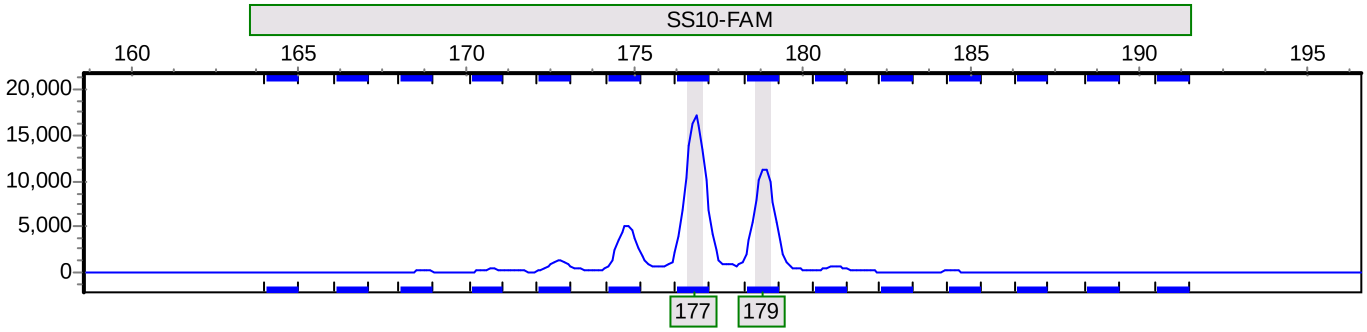

No	Size	Height	Area	Marker	Allele	Difference	Quality	Score	Allele Comments	Sample Comments
1	117.5	17107	114893	SS08-FAM	117	0.10	Pass	500.0	[<Confirmed>]	
2	176.8	17109	118469	SS10-FAM	177	0.10	Pass	500.0	[<Confirmed>]	
3	178.8	11360	77704	SS10-FAM	179	0.00	Pass	500.0	[<Confirmed>]	
4	224.3	8104	59155	SSS42-FAM	224	0.00	Pass	500.0		

5	286.6	10679	91354	SS16-FAM	287	0.10	Pass	500.0		

Sample 47: SS08_SS10_SSS42_SS16_SS27_SS36_SS22_HBN10_G11.fsa

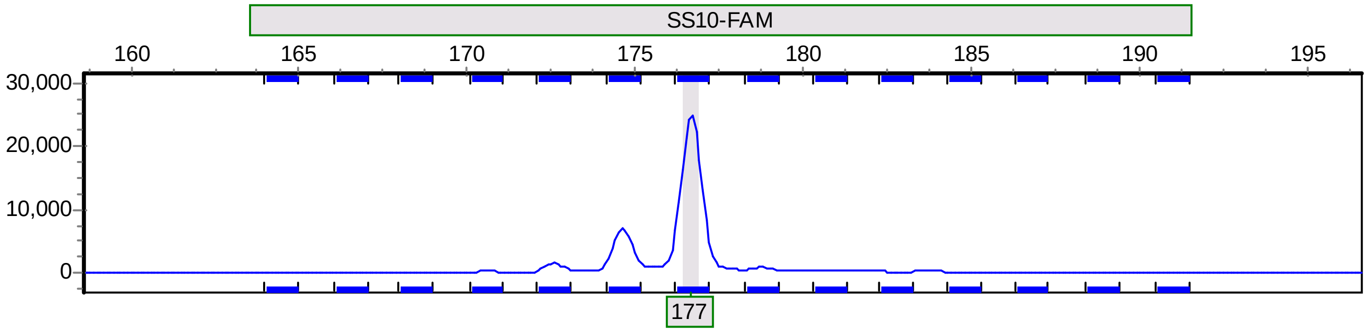

No	Size	Height	Area	Marker	Allele	Difference	Quality	Score	Allele Comments	Sample Comments
1	101.8	12184	83209	SS08-FAM	101	0.00	Pass	500.0	[<Confirmed>]	
2	111.4	6945	48805	SS08-FAM	111	0.10	Pass	500.0	[<Confirmed>]	
3	176.7	24702	168602	SS10-FAM	177	0.00	Pass	500.0	[<Confirmed>]	
4	220.2	12796	92374	SSS42-FAM	220	0.10	Pass	500.0		
5	286.8	9930	94084	SS16-FAM	287	0.30	Pass	500.0		

Sample 48: SS08_SS10_SSS42_SS16_SS27_SS36_SS22_HBN6_O01.fsa

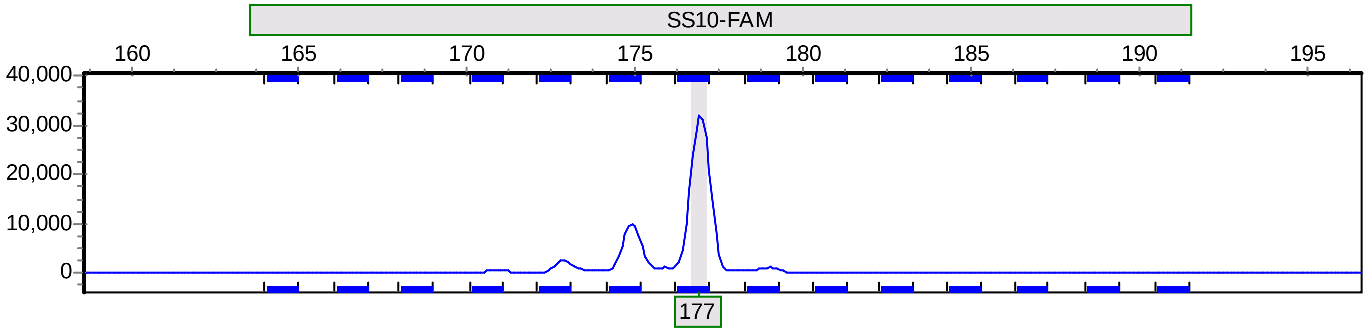

No	Size	Height	Area	Marker	Allele	Difference	Quality	Score	Allele Comments	Sample Comments
1	115.2	13410	82260	SS08-FAM	115	0.20	Pass	500.0	[<Confirmed>]	
2	121.2	9788	60843	SS08-FAM	121	0.10	Pass	500.0	[<Confirmed>]	
3	176.9	31664	209988	SS10-FAM	177	0.20	Pass	500.0	[<Confirmed>]	
4	228.0	4567	29837	SSS42-FAM	228	0.20	Pass	500.0		
5	243.7	7782	53760	SSS42-FAM	244	0.00	Pass	500.0		
6	288.6	13200	101261	SS16-FAM	289	0.40	Pass	500.0		

Sample 49: SS08_SS10_SSS42_SS16_SS27_SS36_SS22_HBN9_D03.fsa

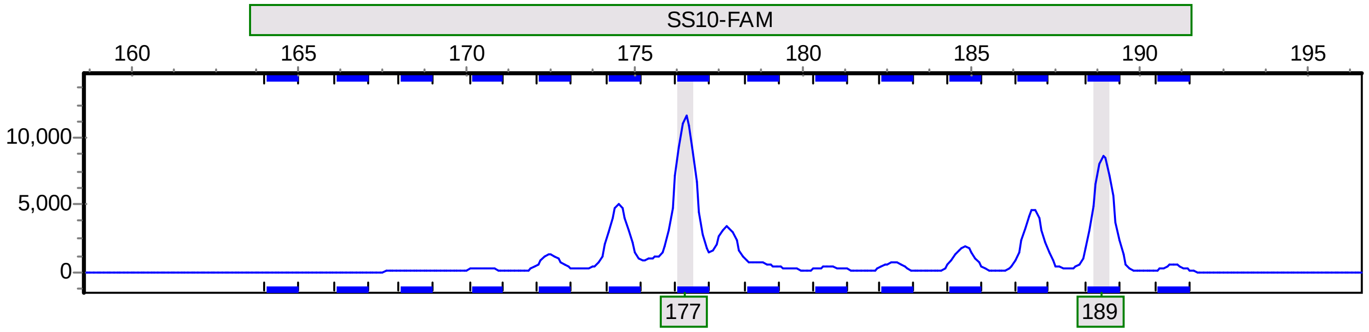

No	Size	Height	Area	Marker	Allele	Difference	Quality	Score	Allele Comments	Sample Comments
1	101.8	14423	93443	SS08-FAM	101	0.00	Pass	500.0	[<Confirmed>]	
2	125.3	4382	29122	SS08-FAM	125	0.20	Pass	500.0	[<Confirmed>]	
3	176.5	11499	80796	SS10-FAM	177	0.20	Pass	500.0	[<Confirmed>]	

4	188.9	8600	59888	SS10-FAM	189	0.00	Pass	500.0	[<Confirmed>]	
5	218.4	17915	125034	SSS42-FAM	218	0.00	Pass	500.0		
6	282.4	10063	84239	SS16-FAM	283	0.20	Pass	500.0		
7	286.5	8904	76668	SS16-FAM	287	0.00	Pass	500.0		

Sample 50: SS08_SS10_SSS42_SS16_SS27_SS36_SS22_HCW1_M09.fsa

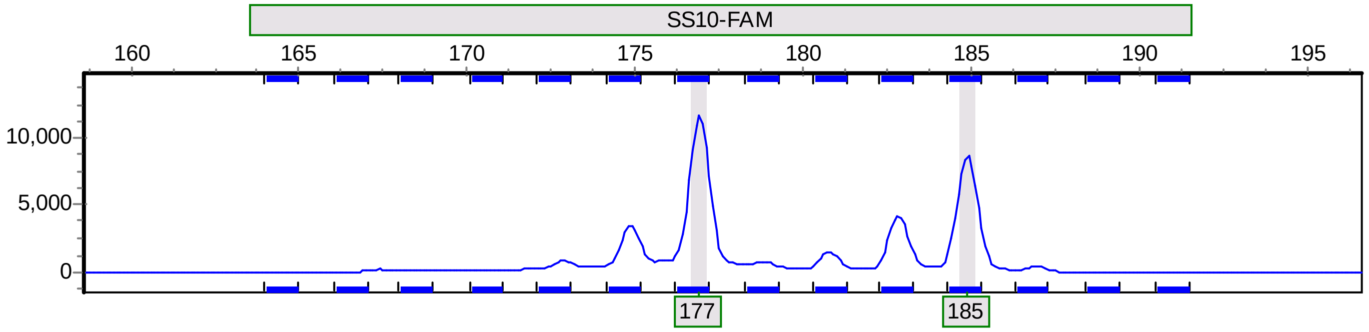

No	Size	Height	Area	Marker	Allele	Difference	Quality	Score	Allele Comments	Sample Comments
1	105.6	11047	75441	SS08-FAM	105	0.00	Pass	500.0	[<Confirmed>]	
2	113.4	9792	67200	SS08-FAM	113	0.00	Pass	500.0	[<Confirmed>]	
3	176.9	11499	81051	SS10-FAM	177	0.20	Pass	500.0	[<Confirmed>]	
4	184.9	8538	60788	SS10-FAM	185	0.10	Pass	500.0	[<Confirmed>]	
5	218.4	7931	59528	SSS42-FAM	218	0.00	Pass	500.0		
6	220.4	6199	46381	SSS42-FAM	220	0.10	Pass	500.0		
7	282.7	7561	66231	SS16-FAM	283	0.10	Pass	500.0		
8	286.8	6689	59509	SS16-FAM	287	0.30	Pass	500.0		

Sample 51: SS08_SS10_SSS42_SS16_SS27_SS36_SS22_HCW2_A11.fsa

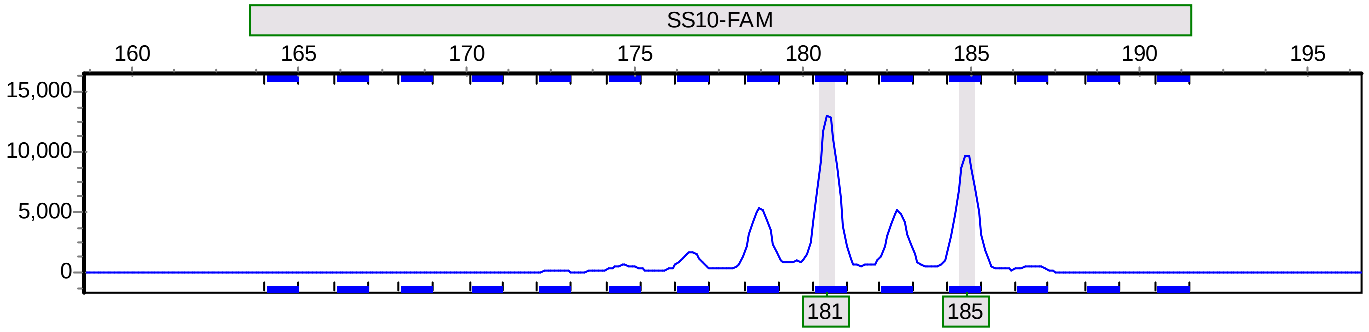

No	Size	Height	Area	Marker	Allele	Difference	Quality	Score	Allele Comments	Sample Comments
1	105.7	13933	94584	SS08-FAM	105	0.10	Pass	500.0	[<Confirmed>]	
2	113.5	11026	73039	SS08-FAM	113	0.10	Pass	500.0	[<Confirmed>]	
3	180.7	12990	90402	SS10-FAM	181	0.10	Pass	500.0	[<Confirmed>]	
4	184.9	9784	68317	SS10-FAM	185	0.10	Pass	500.0	[<Confirmed>]	
5	218.5	11817	82838	SSS42-FAM	218	0.10	Pass	500.0		
6	282.6	15703	132020	SS16-FAM	283	0.00	Pass	500.0		

Sample 52: SS08_SS10_SSS42_SS16_SS27_SS36_SS22_HCW3_G13.fsa

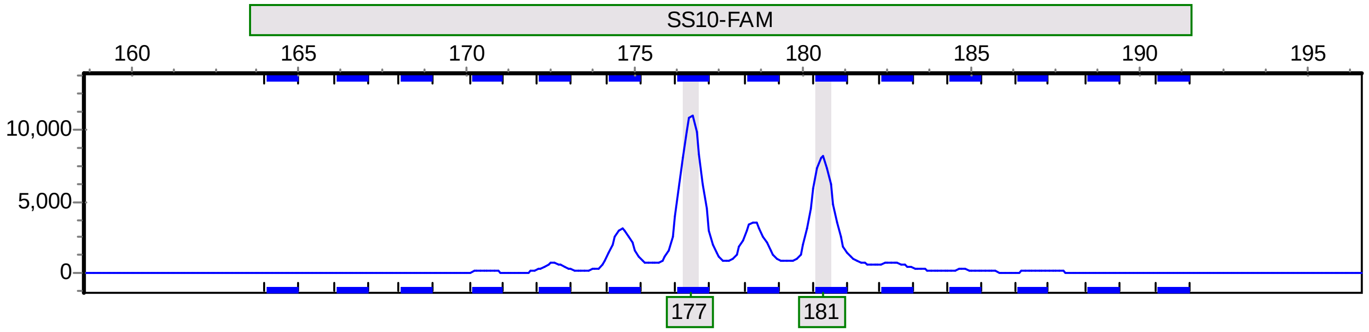

No	Size	Height	Area	Marker	Allele	Difference	Quality	Score	Allele Comments	Sample Comments
1	104.0	9255	66697	SS08-FAM	103	0.10	Pass	500.0	[<Confirmed>]	
2	111.4	6688	47805	SS08-FAM	111	0.10	Pass	500.0	[<Confirmed>]	
3	176.7	10956	84627	SS10-FAM	177	0.00	Pass	500.0	[<Confirmed>]	
4	180.6	8140	63502	SS10-FAM	181	0.20	Pass	500.0	[<Confirmed>]	
5	220.4	11550	90840	SSS42-FAM	220	0.10	Pass	500.0		
6	282.6	11563	101311	SS16-FAM	283	0.00	Pass	500.0		

Sample 53: SS08_SS10_SSS42_SS16_SS27_SS36_SS22_HCW4_E09.fsa

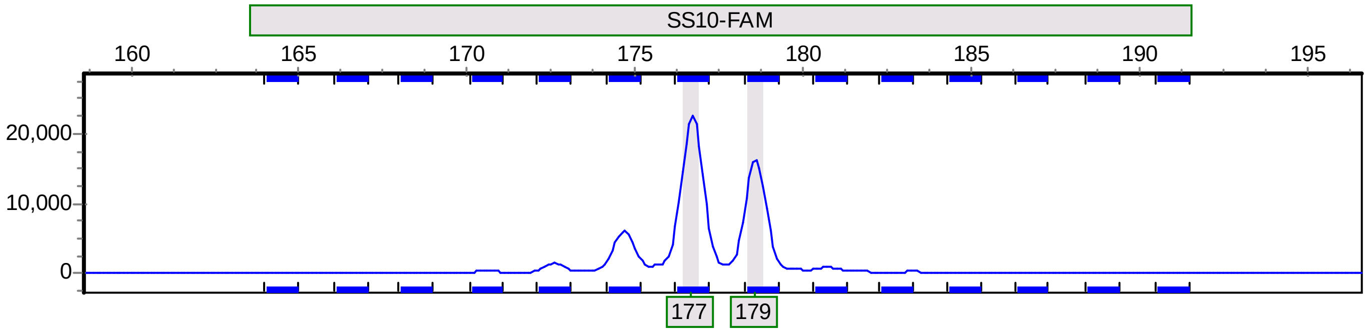

No	Size	Height	Area	Marker	Allele	Difference	Quality	Score	Allele Comments	Sample Comments
1	111.3	27021	178103	SS08-FAM	111	0.20	Pass	500.0	[<Confirmed>]	
2	176.7	22463	165943	SS10-FAM	177	0.00	Pass	500.0	[<Confirmed>]	
3	178.6	16211	115156	SS10-FAM	179	0.20	Pass	500.0	[<Confirmed>]	
4	220.3	16280	119909	SSS42-FAM	220	0.00	Pass	500.0		
5	282.5	11904	103336	SS16-FAM	283	0.10	Pass	500.0		
6	286.6	9615	82768	SS16-FAM	287	0.10	Pass	500.0		

Sample 54: SS08_SS10_SSS42_SS16_SS27_SS36_SS22_HCW5_C11.fsa

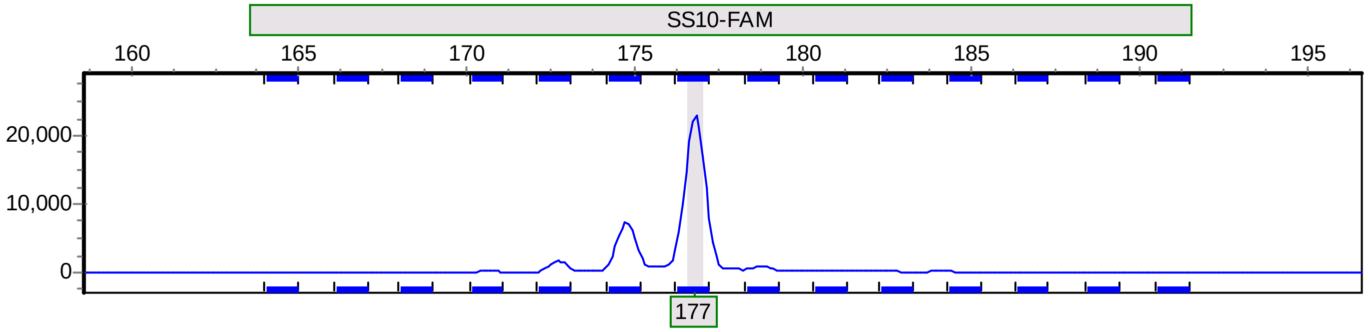

No	Size	Height	Area	Marker	Allele	Difference	Quality	Score	Allele Comments	Sample Comments
1	117.2	18497	123240	SS08-FAM	117	0.20	Pass	500.0	[<Confirmed>]	
2	176.8	22716	154973	SS10-FAM	177	0.10	Pass	500.0	[<Confirmed>]	
3	224.2	6226	45344	SSS42-FAM	224	0.10	Pass	500.0		

4	230.1	8413	62786	SSS42-FAM	230	0.10	Pass	500.0		
5	303.7	5368	50813	SS16-FAM	303	0.10	Pass	500.0		

Sample 55: SS08_SS10_SSS42_SS16_SS27_SS36_SS22_HCW6_D01.fsa

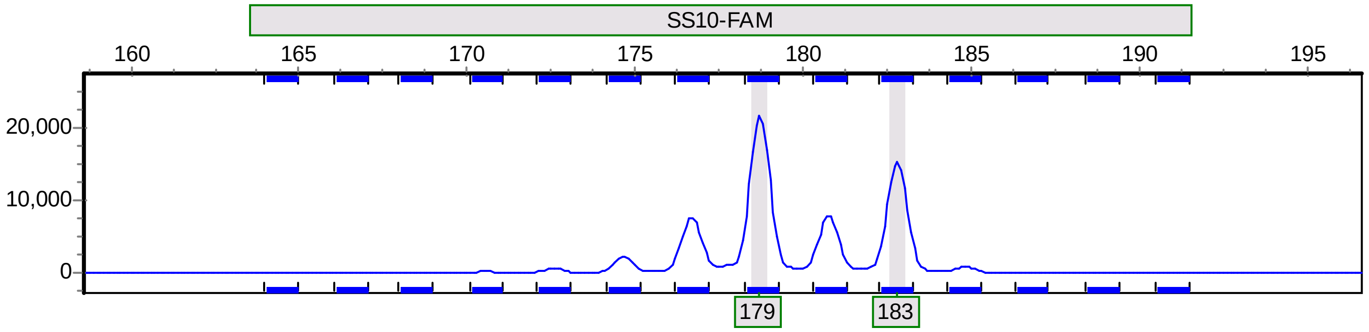

No	Size	Height	Area	Marker	Allele	Difference	Quality	Score	Allele Comments	Sample Comments
1	117.4	27561	189392	SS08-FAM	117	0.00	Pass	500.0	[<Confirmed>]	
2	178.7	21407	142787	SS10-FAM	179	0.10	Pass	500.0	[<Confirmed>]	
3	182.8	15274	103423	SS10-FAM	183	0.00	Pass	500.0	[<Confirmed>]	
4	228.2	19452	143186	SSS42-FAM	228	0.00	Pass	500.0		

Sample 56: SS08_SS10_SSS42_SS16_SS27_SS36_SS22_HCW7_G17.fsa

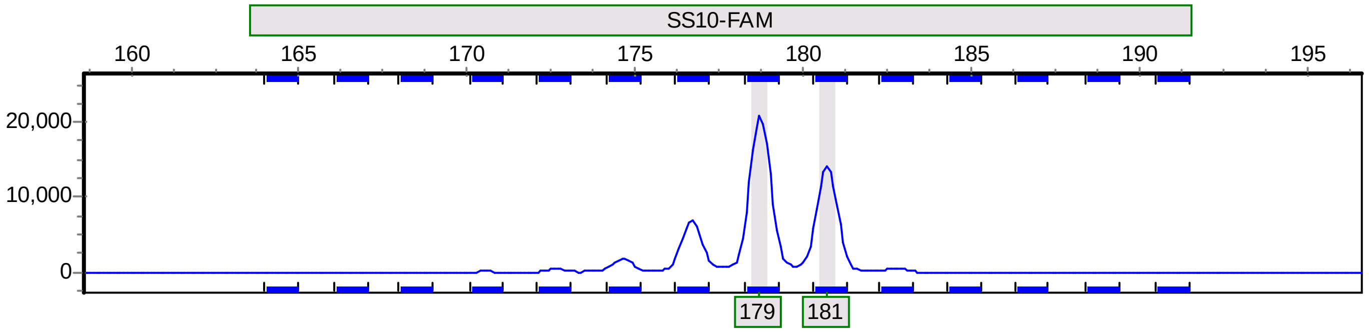

No	Size	Height	Area	Marker	Allele	Difference	Quality	Score	Allele Comments	Sample Comments
1	104.0	19288	128661	SS08-FAM	103	0.10	Pass	500.0	[<Confirmed>]	
2	111.6	14426	96814	SS08-FAM	111	0.10	Pass	500.0	[<Confirmed>]	
3	178.7	20608	143743	SS10-FAM	179	0.10	Pass	500.0	[<Confirmed>]	
4	180.7	14144	101231	SS10-FAM	181	0.10	Pass	500.0	[<Confirmed>]	
5	220.3	10492	78358	SSS42-FAM	220	0.00	Pass	500.0		
6	222.3	8240	62821	SSS42-FAM	222	0.00	Pass	500.0		
7	296.6	16881	150852	SS16-FAM	297	0.40	Pass	500.0		

Sample 57: SS08_SS10_SSS42_SS16_SS27_SS36_SS22_HCW8_A17.fsa

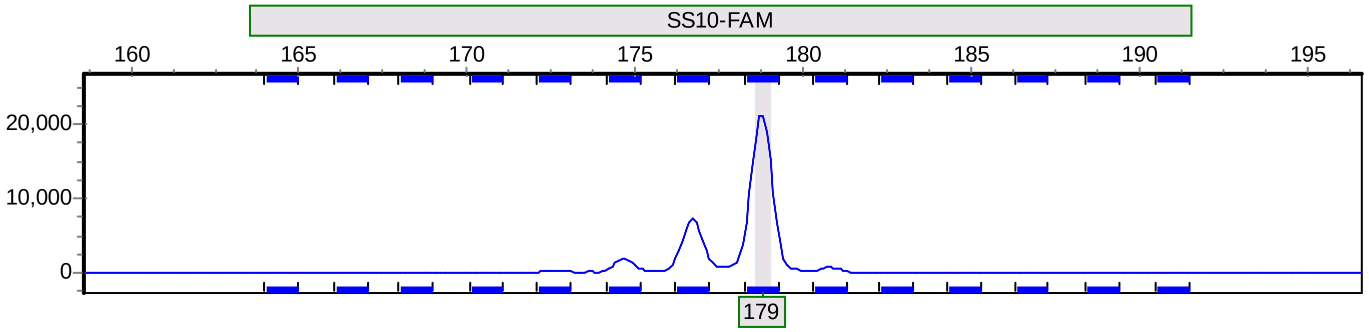

No	Size	Height	Area	Marker	Allele	Difference	Quality	Score	Allele Comments	Sample Comments
1	101.8	16452	108280	SS08-FAM	101	0.00	Pass	500.0	[<Confirmed>]	
2	111.6	12283	83557	SS08-FAM	111	0.10	Pass	500.0	[<Confirmed>]	

3	178.8	20841	146760	SS10-FAM	179	0.00	Pass	500.0	[<Confirmed>]	
4	222.3	6735	48690	SSS42-FAM	222	0.00	Pass	500.0		
5	230.0	4231	31125	SSS42-FAM	230	0.20	Pass	500.0		
6	286.6	7264	65923	SS16-FAM	287	0.10	Pass	500.0		

Sample 58: SS08_SS10_SSS42_SS16_SS27_SS36_SS22_HGC1_C01.fsa

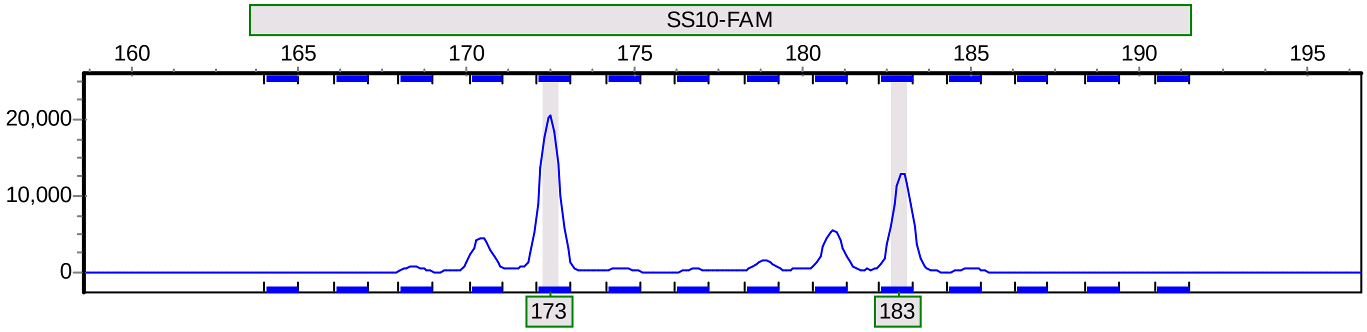

No	Size	Height	Area	Marker	Allele	Difference	Quality	Score	Allele Comments	Sample Comments
1	106.7	21373	138847	SS08-FAM	107	1.00	Pass	500.0	[<Confirmed><Edited>]	
2	111.4	15615	99628	SS08-FAM	111	0.10	Pass	500.0	[<Confirmed>]	
3	172.5	20326	134521	SS10-FAM	173	0.10	Pass	500.0	[<Confirmed>]	
4	182.9	12956	86598	SS10-FAM	183	0.10	Pass	500.0	[<Confirmed>]	
5	215.5	7820	54317	SSS42-FAM	216	0.00	Pass	500.0		
6	218.3	2966	21688	SSS42-FAM	218	0.10	Pass	121.9		
7	303.7	6565	57320	SS16-FAM	303	0.10	Pass	500.0		

Sample 59: SS08_SS10_SSS42_SS16_SS27_SS36_SS22_HGC3_E17.fsa

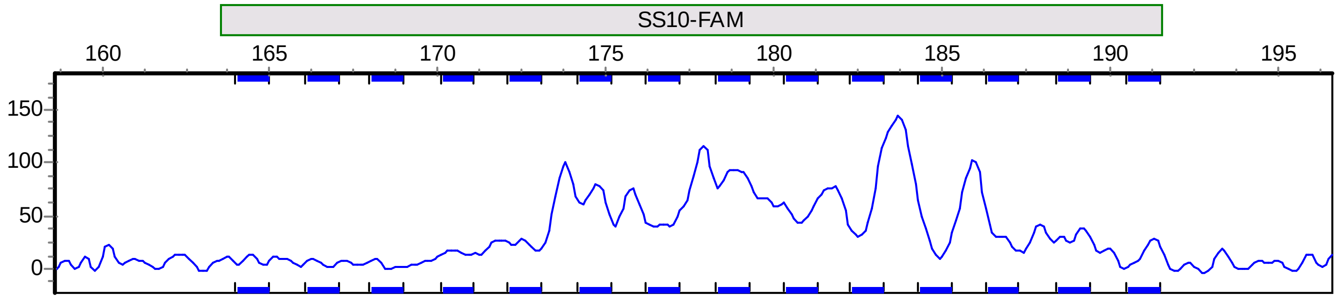

No	Size	Height	Area	Marker	Allele	Difference	Quality	Score	Allele Comments	Sample Comments

Sample 60: SS08_SS10_SSS42_SS16_SS27_SS36_SS22_HGC4_A13.fsa

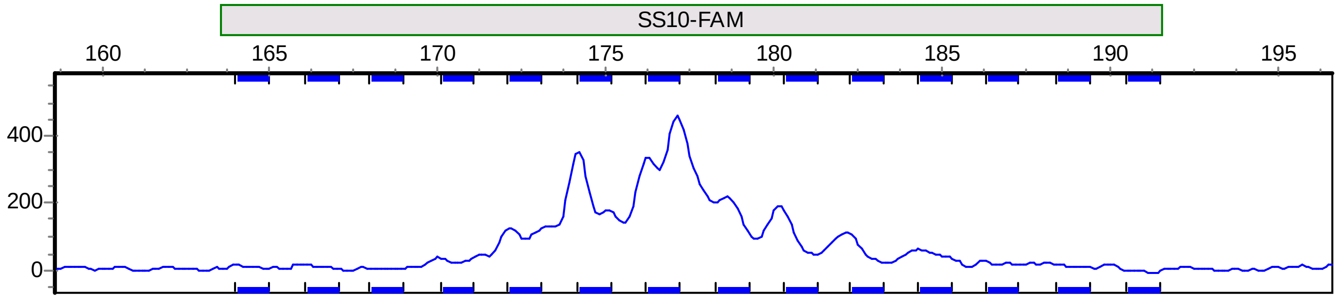

No	Size	Height	Area	Marker	Allele	Difference	Quality	Score	Allele Comments	Sample Comments

Allele Report

Sample 61: SS08_SS10_SSS42_SS16_SS27_SS36_SS22_HGY1_A09.fsa

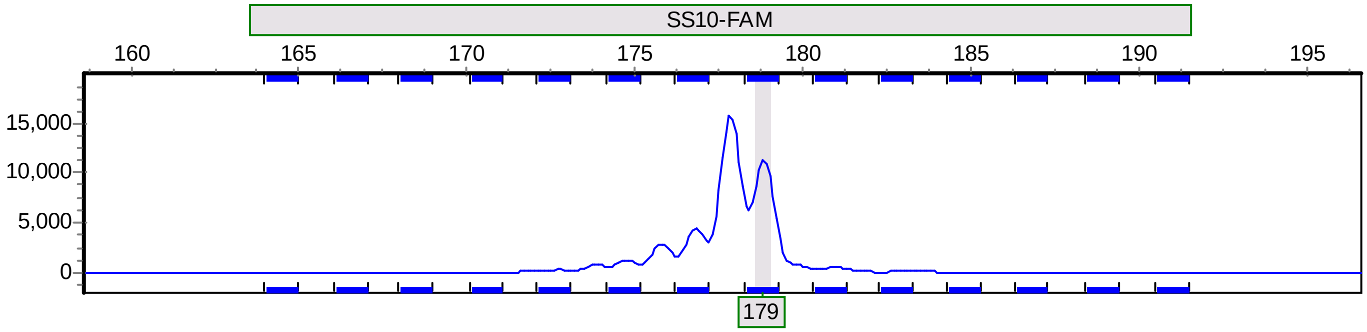

No	Size	Height	Area	Marker	Allele	Difference	Quality	Score	Allele Comments	Sample Comments
1	117.4	28052	190929	SS08-FAM	117	0.00	Pass	500.0	[<Confirmed>]	
2	178.8	11255	75206	SS10-FAM	179	0.00	Pass	500.0	[<Confirmed>]	
3	228.2	11077	80700	SSS42-FAM	228	0.00	Pass	500.0		
4	294.7	8006	69170	SS16-FAM	295	0.10	Pass	500.0		
5	303.7	3033	29603	SS16-FAM	303	0.10	Pass	312.9		

Sample 62: SS08_SS10_SSS42_SS16_SS27_SS36_SS22_HGY2_I13.fsa

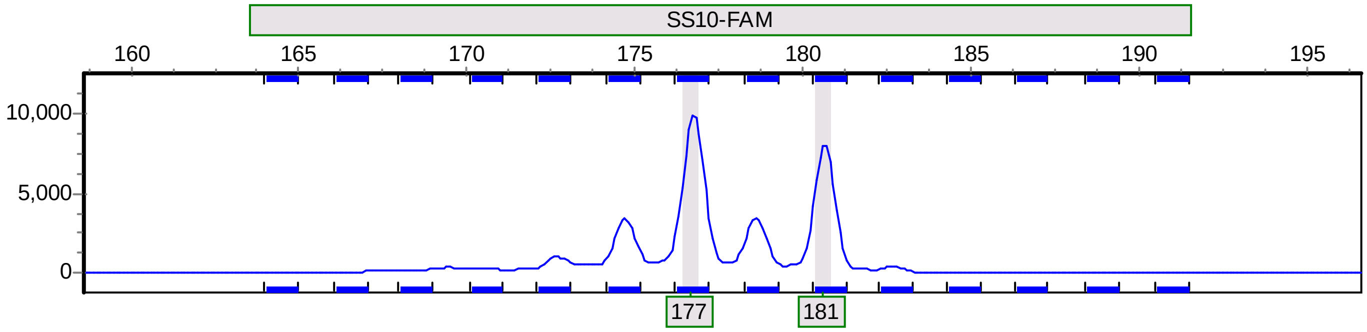

No	Size	Height	Area	Marker	Allele	Difference	Quality	Score	Allele Comments	Sample Comments
1	104.0	7458	51412	SS08-FAM	103	0.10	Pass	500.0	[<Confirmed>]	
2	113.2	5601	41860	SS08-FAM	113	0.20	Pass	500.0	[<Confirmed>]	
3	176.7	9837	72946	SS10-FAM	177	0.00	Pass	500.0	[<Confirmed>]	
4	180.6	8037	56352	SS10-FAM	181	0.20	Pass	500.0	[<Confirmed>]	
5	224.2	9402	69864	SSS42-FAM	224	0.10	Pass	500.0		
6	282.7	6403	56525	SS16-FAM	283	0.10	Pass	500.0		
7	290.5	4453	39535	SS16-FAM	291	0.00	Pass	500.0		

Sample 63: SS08_SS10_SSS42_SS16_SS27_SS36_SS22_HGY3_I11.fsa

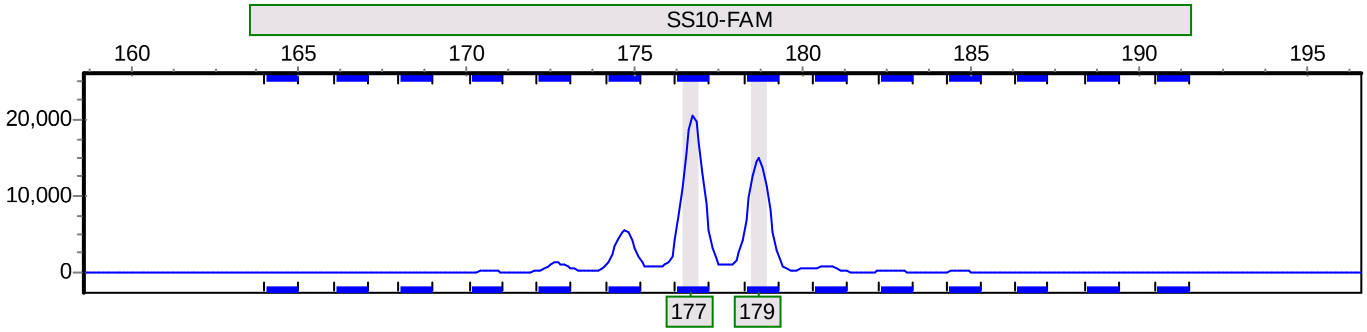

No	Size	Height	Area	Marker	Allele	Difference	Quality	Score	Allele Comments	Sample Comments
1	111.6	12300	93261	SS08-FAM	111	0.10	Pass	500.0	[<Confirmed>]	
2	113.2	10142	73307	SS08-FAM	113	0.20	Pass	500.0	[<Confirmed>]	
3	176.7	20470	140330	SS10-FAM	177	0.00	Pass	500.0	[<Confirmed>]	

4	178.7	14996	104241	SS10-FAM	179	0.10	Pass	500.0	[<Confirmed>]	
5	218.4	17391	121378	SSS42-FAM	218	0.00	Pass	500.0		
6	282.6	12130	100718	SS16-FAM	283	0.00	Pass	500.0		
7	294.3	6429	55856	SS16-FAM	295	0.30	Pass	500.0		

Sample 64: SS08_SS10_SSS42_SS16_SS27_SS36_SS22_HGY4_I09.fsa

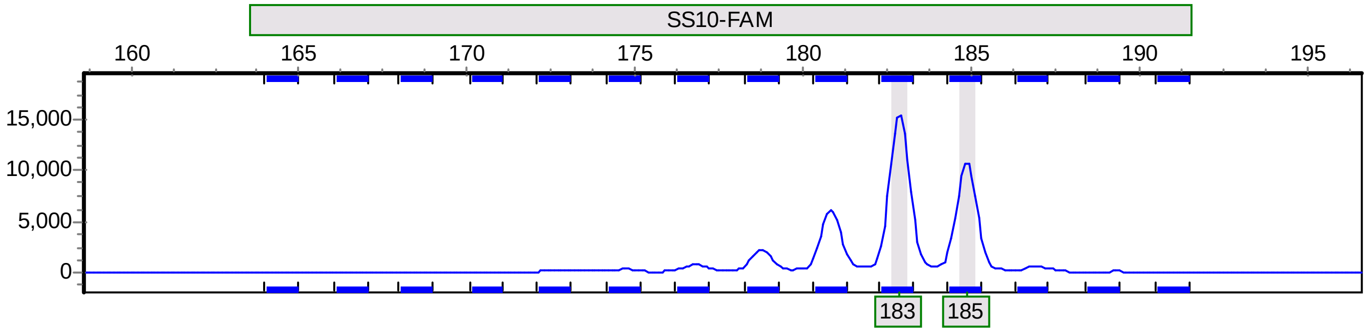

No	Size	Height	Area	Marker	Allele	Difference	Quality	Score	Allele Comments	Sample Comments
1	103.9	15189	101249	SS08-FAM	103	0.00	Pass	500.0	[<Confirmed>]	
2	113.2	10881	72779	SS08-FAM	113	0.20	Pass	500.0	[<Confirmed>]	
3	182.9	15247	106530	SS10-FAM	183	0.10	Pass	500.0	[<Confirmed>]	
4	184.9	10604	74288	SS10-FAM	185	0.10	Pass	500.0	[<Confirmed>]	
5	230.0	9676	69284	SSS42-FAM	230	0.20	Pass	500.0		
6	282.6	10083	83794	SS16-FAM	283	0.00	Pass	500.0		

Sample 65: SS08_SS10_SSS42_SS16_SS27_SS36_SS22_HGY5_I15.fsa

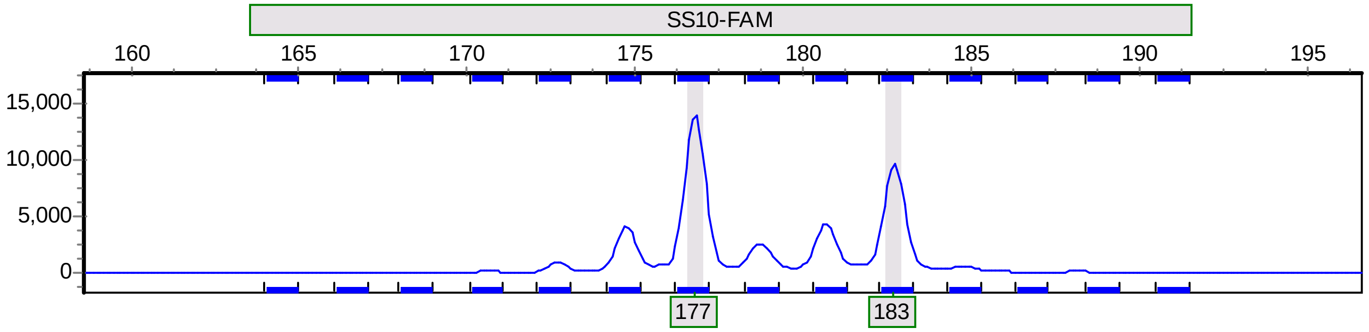

No	Size	Height	Area	Marker	Allele	Difference	Quality	Score	Allele Comments	Sample Comments
1	105.5	18712	128286	SS08-FAM	105	0.10	Pass	500.0	[<Confirmed>]	
2	176.8	13922	99071	SS10-FAM	177	0.10	Pass	500.0	[<Confirmed>]	
3	182.7	9662	70198	SS10-FAM	183	0.10	Pass	500.0	[<Confirmed>]	
4	215.5	7630	55259	SSS42-FAM	216	0.00	Pass	500.0		
5	224.3	4500	33448	SSS42-FAM	224	0.00	Pass	500.0		
6	282.7	8656	74584	SS16-FAM	283	0.10	Pass	500.0		
7	290.5	6174	53730	SS16-FAM	291	0.00	Pass	500.0		

Sample 66: SS08_SS10_SSS42_SS16_SS27_SS36_SS22_HQZ11_O11.fsa

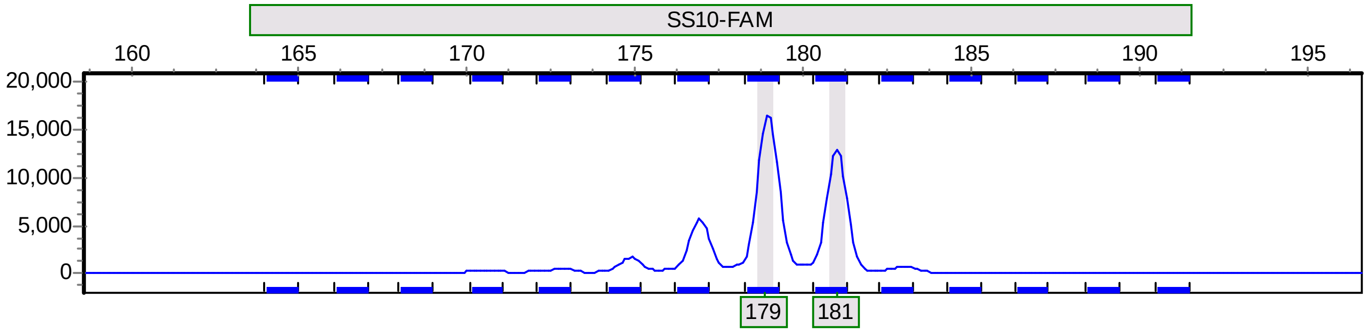

No	Size	Height	Area	Marker	Allele	Difference	Quality	Score	Allele Comments	Sample Comments
1	109.2	13050	86808	SS08-FAM	109	0.10	Pass	500.0	[<Confirmed>]	
2	115.2	12146	82994	SS08-FAM	115	0.20	Pass	500.0	[<Confirmed>]	
3	178.9	16406	116388	SS10-FAM	179	0.10	Pass	500.0	[<Confirmed>]	
4	181.0	12956	90227	SS10-FAM	181	0.20	Pass	500.0	[<Confirmed>]	
5	224.3	8377	62262	SSS42-FAM	224	0.00	Pass	500.0		
6	243.7	3372	25859	SSS42-FAM	244	0.00	Pass	500.0		
7	294.8	8877	79920	SS16-FAM	295	0.20	Pass	500.0		
8	303.7	3530	34352	SS16-FAM	303	0.10	Pass	357.3		

Sample 67: SS08_SS10_SSS42_SS16_SS27_SS36_SS22_HQZ13-1_C09.fsa

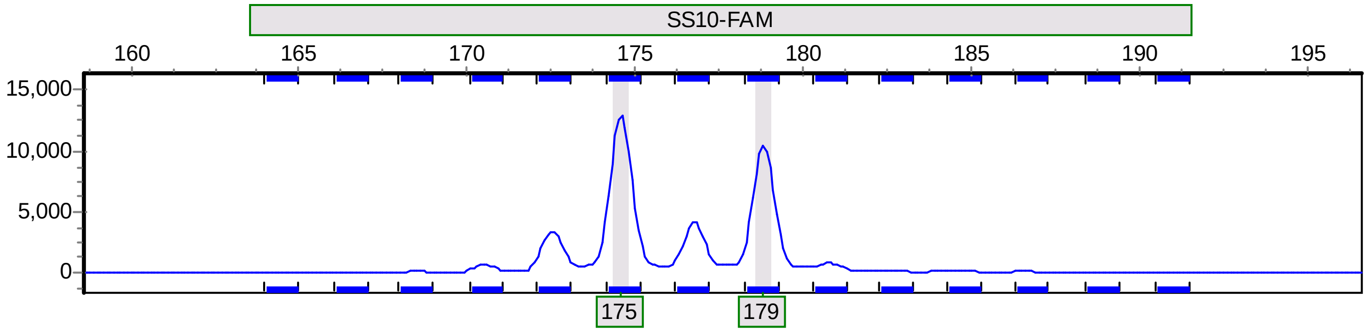

No	Size	Height	Area	Marker	Allele	Difference	Quality	Score	Allele Comments	Sample Comments
1	111.6	10674	72504	SS08-FAM	111	0.10	Pass	500.0	[<Confirmed>]	
2	117.4	9509	65977	SS08-FAM	117	0.00	Pass	500.0	[<Confirmed>]	
3	174.6	12837	95341	SS10-FAM	175	0.10	Pass	500.0	[<Confirmed>]	
4	178.8	10380	75340	SS10-FAM	179	0.00	Pass	500.0	[<Confirmed>]	
5	220.2	7555	56880	SSS42-FAM	220	0.10	Pass	500.0		
6	224.2	4978	37747	SSS42-FAM	224	0.10	Pass	500.0		
7	289.5	15244	133640	SS16-FAM	289	0.50	Pass	500.0		

Sample 68: SS08_SS10_SSS42_SS16_SS27_SS36_SS22_HQZ13-2_J03.fsa

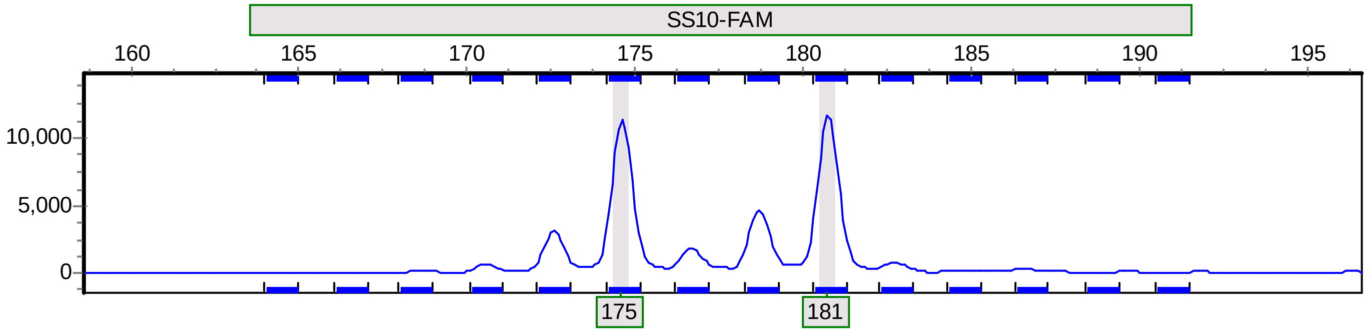

No	Size	Height	Area	Marker	Allele	Difference	Quality	Score	Allele Comments	Sample Comments

1	115.5	11534	76750	SS08-FAM	115	0.10	Pass	500.0	[<Confirmed>]	
2	117.5	8371	57188	SS08-FAM	117	0.10	Pass	500.0	[<Confirmed>]	
3	174.6	11329	78415	SS10-FAM	175	0.10	Pass	500.0	[<Confirmed>]	
4	180.7	11550	82284	SS10-FAM	181	0.10	Pass	500.0	[<Confirmed>]	
5	228.3	10008	75240	SSS42-FAM	228	0.10	Pass	500.0		
6	294.5	11891	111045	SS16-FAM	295	0.10	Pass	500.0		

Sample 69: SS08_SS10_SSS42_SS16_SS27_SS36_SS22_HQZ14_H03.fsa

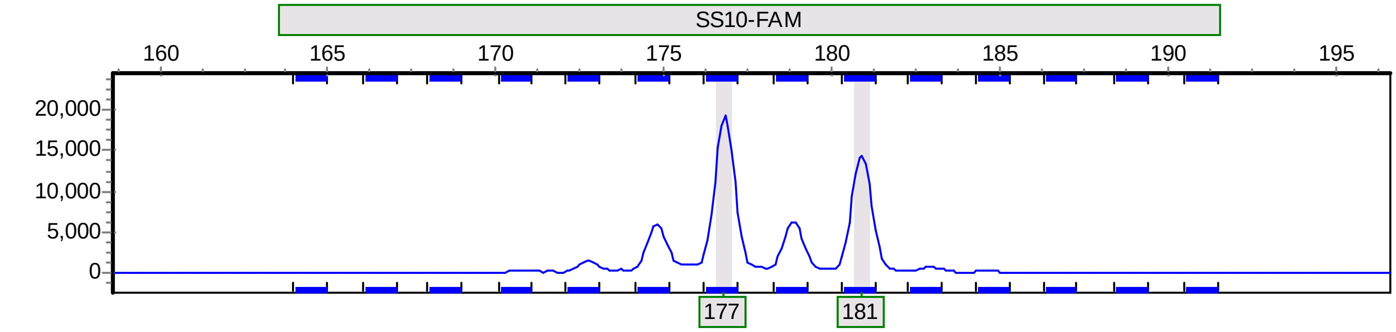

No	Size	Height	Area	Marker	Allele	Difference	Quality	Score	Allele Comments	Sample Comments
1	115.5	13434	91067	SS08-FAM	115	0.10	Pass	500.0	[<Confirmed>]	
2	117.6	13692	90126	SS08-FAM	117	0.20	Pass	500.0	[<Confirmed>]	
3	176.8	19160	128683	SS10-FAM	177	0.10	Pass	500.0	[<Confirmed>]	
4	180.9	14444	99650	SS10-FAM	181	0.10	Pass	500.0	[<Confirmed>]	
5	224.3	9796	67241	SSS42-FAM	224	0.00	Pass	500.0		
6	294.6	13829	114019	SS16-FAM	295	0.00	Pass	500.0		

Sample 70: SS08_SS10_SSS42_SS16_SS27_SS36_SS22_HQZ15_B01.fsa

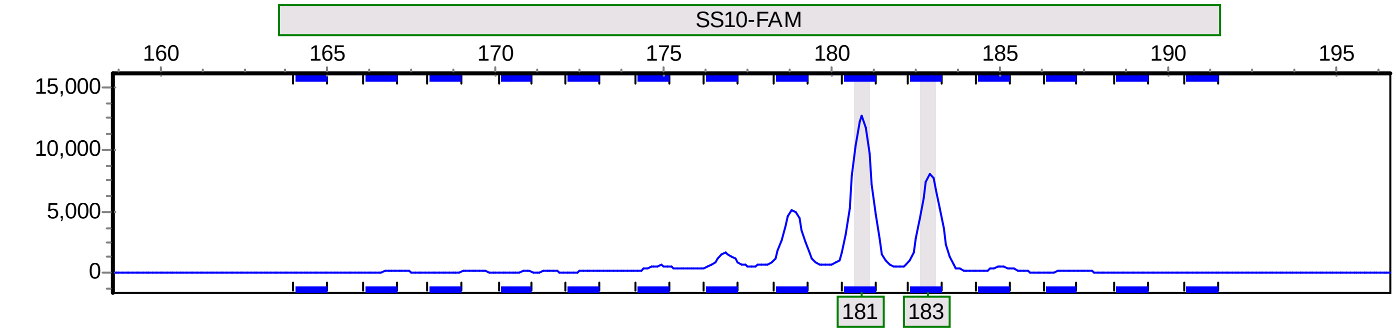

No	Size	Height	Area	Marker	Allele	Difference	Quality	Score	Allele Comments	Sample Comments
1	115.5	11216	79794	SS08-FAM	115	0.10	Pass	500.0	[<Confirmed>]	
2	117.6	10963	73800	SS08-FAM	117	0.20	Pass	500.0	[<Confirmed>]	
3	180.9	12718	86882	SS10-FAM	181	0.10	Pass	500.0	[<Confirmed>]	
4	182.9	7999	55524	SS10-FAM	183	0.10	Pass	500.0	[<Confirmed>]	
5	224.3	10893	79034	SSS42-FAM	224	0.00	Pass	500.0		
6	294.7	4743	42129	SS16-FAM	295	0.10	Pass	500.0		

Allele Report

Sample 71: SS08_SS10_SSS42_SS16_SS27_SS36_SS22_HQZ16_P01.fsa

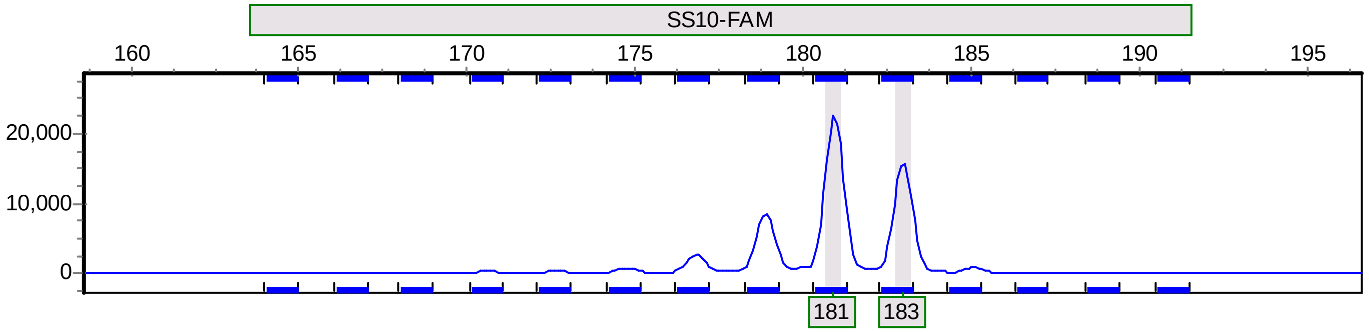

No	Size	Height	Area	Marker	Allele	Difference	Quality	Score	Allele Comments	Sample Comments
1	115.4	12078	78881	SS08-FAM	115	0.00	Pass	500.0	[<Confirmed>]	
2	117.2	7675	48997	SS08-FAM	117	0.20	Pass	500.0	[<Confirmed>]	
3	180.9	22489	144516	SS10-FAM	181	0.10	Pass	500.0	[<Confirmed>]	
4	183.0	15669	101156	SS10-FAM	183	0.20	Pass	500.0	[<Confirmed>]	
5	222.5	3533	23661	SSS42-FAM	222	0.20	Pass	500.0		
6	245.8	6869	48707	SSS42-FAM	246	0.10	Pass	500.0		
7	294.7	4068	33437	SS16-FAM	295	0.10	Pass	500.0		

Sample 72: SS08_SS10_SSS42_SS16_SS27_SS36_SS22_HQZ17-1_O03.fsa

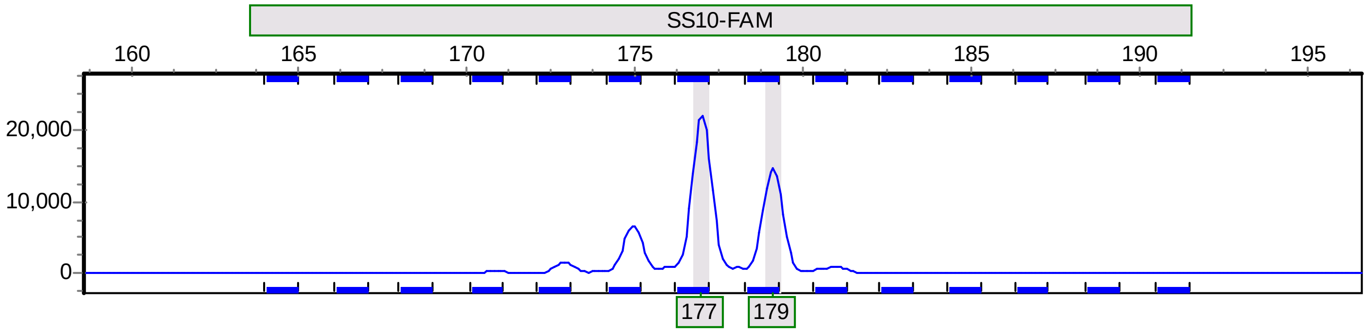

No	Size	Height	Area	Marker	Allele	Difference	Quality	Score	Allele Comments	Sample Comments
1	115.4	18910	120565	SS08-FAM	115	0.00	Pass	500.0	[<Confirmed>]	
2	117.4	14723	93432	SS08-FAM	117	0.00	Pass	500.0	[<Confirmed>]	
3	177.0	21803	144789	SS10-FAM	177	0.30	Pass	500.0	[<Confirmed>]	
4	179.1	14506	96928	SS10-FAM	179	0.30	Pass	500.0	[<Confirmed>]	
5	222.4	5507	38431	SSS42-FAM	222	0.10	Pass	500.0		
6	251.6	5674	42363	SSS42-FAM	252	0.10	Pass	500.0		
7	294.9	11567	96454	SS16-FAM	295	0.30	Pass	500.0		
8	303.8	4595	41125	SS16-FAM	303	0.20	Pass	500.0		

Sample 73: SS08_SS10_SSS42_SS16_SS27_SS36_SS22_HQZ17-2_N03.fsa

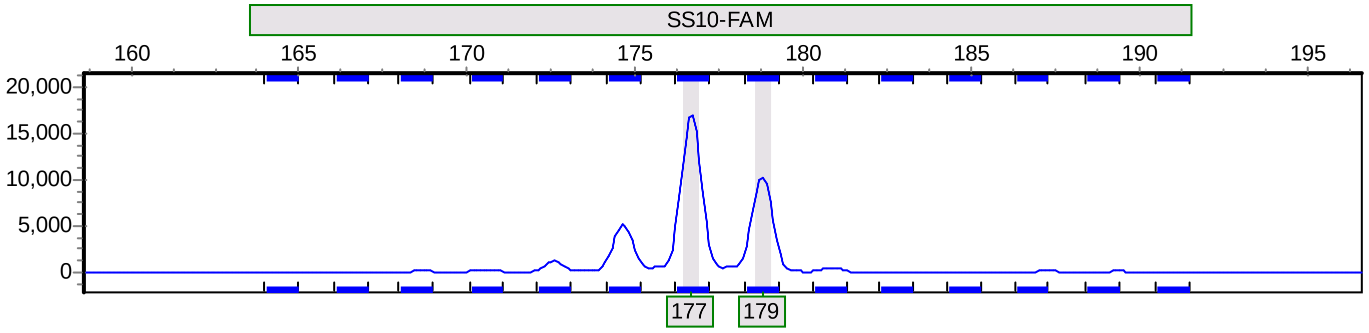

No	Size	Height	Area	Marker	Allele	Difference	Quality	Score	Allele Comments	Sample Comments

1	115.4	16384	107683	SS08-FAM	115	0.00	Pass	500.0	[<Confirmed>]	
2	117.4	12186	81995	SS08-FAM	117	0.00	Pass	500.0	[<Confirmed>]	
3	176.7	16825	114480	SS10-FAM	177	0.00	Pass	500.0	[<Confirmed>]	
4	178.8	10260	70207	SS10-FAM	179	0.00	Pass	500.0	[<Confirmed>]	
5	222.3	5589	40454	SSS42-FAM	222	0.00	Pass	500.0		
6	251.5	6286	49312	SSS42-FAM	252	0.00	Pass	500.0		
7	294.7	10730	95223	SS16-FAM	295	0.10	Pass	500.0		
8	303.6	4493	43215	SS16-FAM	303	0.00	Pass	500.0		

Sample 74: SS08_SS10_SSS42_SS16_SS27_SS36_SS22_HQZ18_K09.fsa

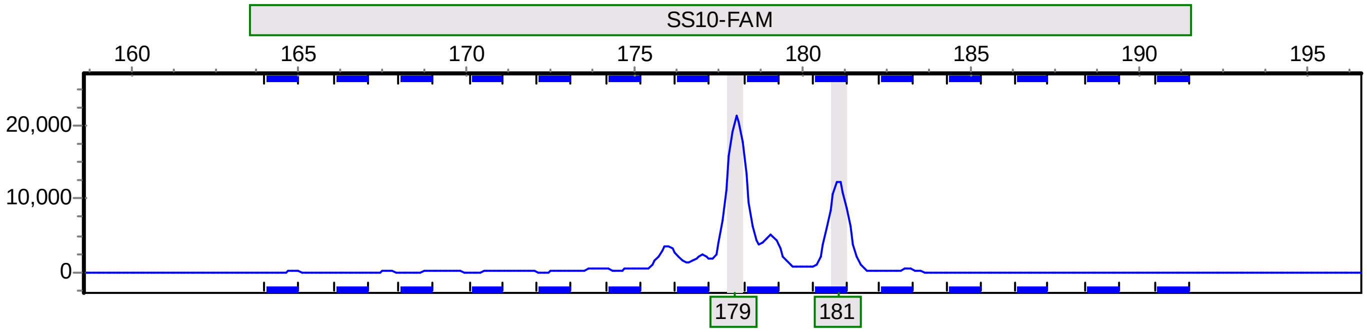

No	Size	Height	Area	Marker	Allele	Difference	Quality	Score	Allele Comments	Sample Comments
1	115.4	17852	116892	SS08-FAM	115	0.00	Pass	500.0	[<Confirmed>]	
2	117.4	13356	87335	SS08-FAM	117	0.00	Pass	500.0	[<Confirmed>]	
3	178.0	21138	143218	SS10-FAM	179	1.00	Pass	500.0	[<Confirmed><Edited>]	
4	181.1	12244	85056	SS10-FAM	181	0.30	Pass	500.0	[<Confirmed>]	
5	222.4	4673	33784	SSS42-FAM	222	0.10	Pass	500.0		
6	245.8	8025	60205	SSS42-FAM	246	0.10	Pass	500.0		
7	294.8	13456	113947	SS16-FAM	295	0.20	Pass	500.0		

Sample 75: SS08_SS10_SSS42_SS16_SS27_SS36_SS22_HQZ19_L03.fsa

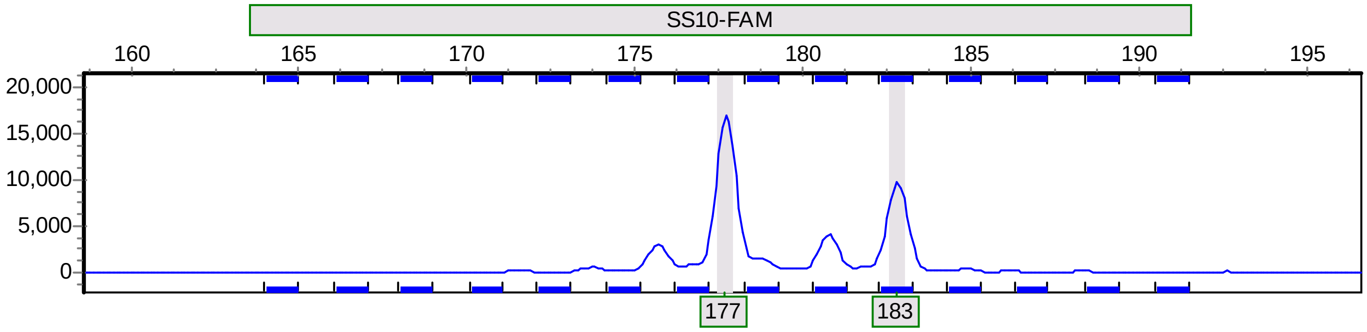

No	Size	Height	Area	Marker	Allele	Difference	Quality	Score	Allele Comments	Sample Comments
1	117.2	17705	115264	SS08-FAM	117	0.20	Pass	500.0	[<Confirmed>]	
2	177.7	16846	114889	SS10-FAM	177	1.00	Pass	500.0	[<Confirmed><Edited>]	
3	182.8	9778	68925	SS10-FAM	183	0.00	Pass	500.0	[<Confirmed>]	
4	222.4	4793	34741	SSS42-FAM	222	0.10	Pass	425.3		
5	294.5	14707	128597	SS16-FAM	295	0.10	Pass	500.0		

Allele Report

Sample 76: SS08_SS10_SSS42_SS16_SS27_SS36_SS22_HQZ21_B03.fsa

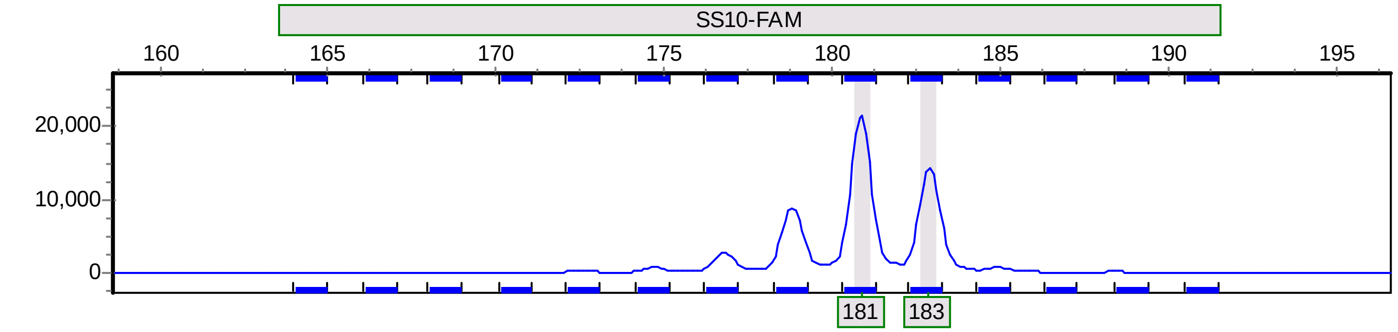

No	Size	Height	Area	Marker	Allele	Difference	Quality	Score	Allele Comments	Sample Comments
1	115.4	17601	119615	SS08-FAM	115	0.00	Pass	500.0	[<Confirmed>]	
2	117.4	13575	94631	SS08-FAM	117	0.00	Pass	500.0	[<Confirmed>]	
3	180.9	21196	145759	SS10-FAM	181	0.10	Pass	500.0	[<Confirmed>]	
4	182.9	14267	104316	SS10-FAM	183	0.10	Pass	500.0	[<Confirmed>]	
5	230.2	10621	82374	SSS42-FAM	230	0.00	Pass	500.0		
6	245.7	5288	43416	SSS42-FAM	246	0.00	Pass	500.0		
7	294.5	12110	108917	SS16-FAM	295	0.10	Pass	500.0		
8	301.2	4286	43674	SS16-FAM	301	0.30	Pass	464.9		

Sample 77: SS08_SS10_SSS42_SS16_SS27_SS36_SS22_HQZ22-1_M01.fsa

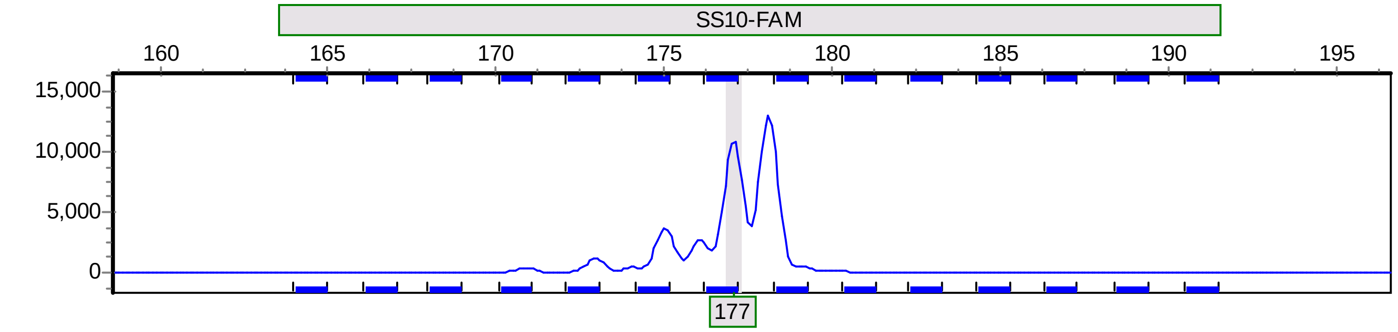

No	Size	Height	Area	Marker	Allele	Difference	Quality	Score	Allele Comments	Sample Comments
1	117.5	18586	118685	SS08-FAM	117	0.10	Pass	500.0	[<Confirmed>]	
2	177.1	10876	72954	SS10-FAM	177	0.40	Pass	500.0	[<Confirmed>]	
3	243.7	2603	18724	SSS42-FAM	244	0.00	Pass	452.7		
4	294.8	4615	37682	SS16-FAM	295	0.20	Pass	500.0		
5	303.8	2091	18628	SS16-FAM	303	0.20	Pass	212.9		

Sample 78: SS08_SS10_SSS42_SS16_SS27_SS36_SS22_HQZ22-2_O13.fsa

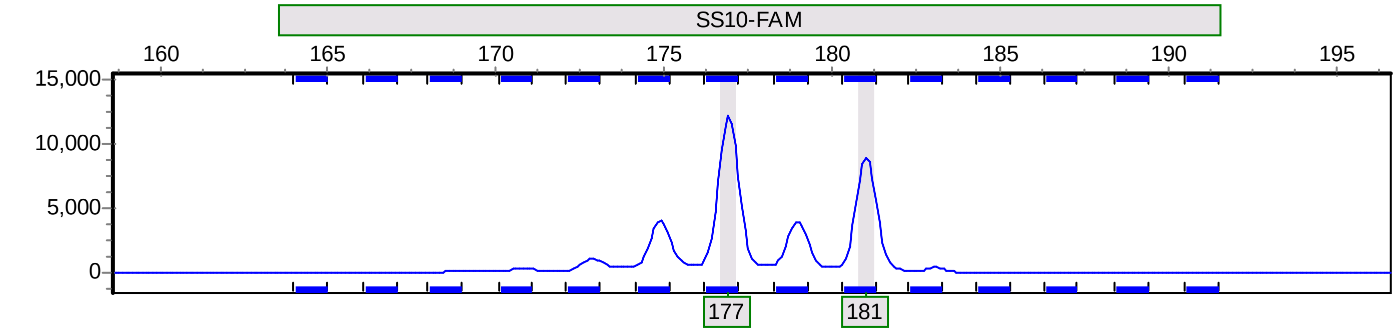

No	Size	Height	Area	Marker	Allele	Difference	Quality	Score	Allele Comments	Sample Comments
1	115.5	15694	109677	SS08-FAM	115	0.10	Pass	500.0	[<Confirmed>]	
2	117.3	8244	60064	SS08-FAM	117	0.10	Pass	500.0	[<Confirmed>]	

3	176.9	12166	84558	SS10-FAM	177	0.20	Pass	500.0	[<Confirmed>]	
4	181.0	9018	63264	SS10-FAM	181	0.20	Pass	500.0	[<Confirmed>]	
5	230.3	7158	52947	SSS42-FAM	230	0.10	Pass	500.0		
6	294.8	11180	98069	SS16-FAM	295	0.20	Pass	500.0		

Sample 79: SS08_SS10_SSS42_SS16_SS27_SS36_SS22_HQZ23_A15.fsa

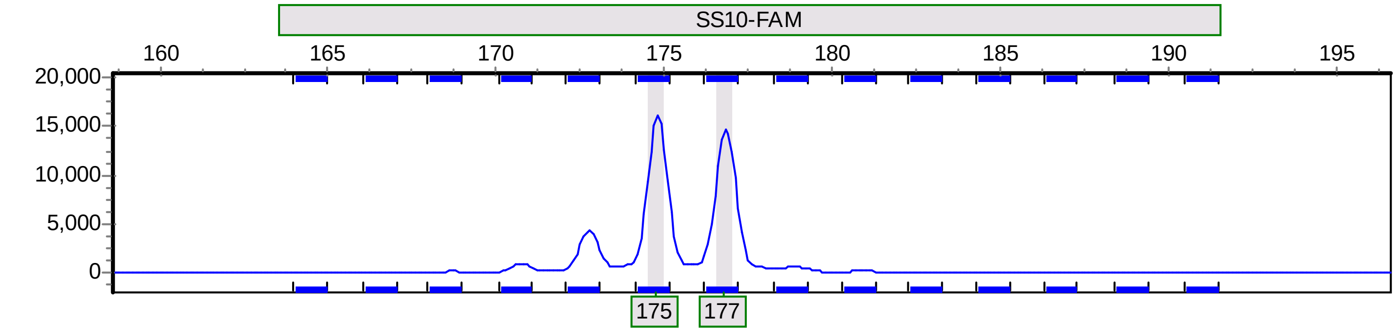

No	Size	Height	Area	Marker	Allele	Difference	Quality	Score	Allele Comments	Sample Comments
1	115.3	23022	164114	SS08-FAM	115	0.10	Pass	500.0	[<Confirmed>]	
2	174.8	16032	108157	SS10-FAM	175	0.10	Pass	500.0	[<Confirmed>]	
3	176.8	14720	101471	SS10-FAM	177	0.10	Pass	500.0	[<Confirmed>]	
4	230.1	12975	93502	SSS42-FAM	230	0.10	Pass	500.0		
5	294.8	6338	55163	SS16-FAM	295	0.20	Pass	500.0		
6	301.5	2577	24003	SS16-FAM	301	0.00	Pass	261.3		

Sample 80: SS08_SS10_SSS42_SS16_SS27_SS36_SS22_HQZ24_E11.fsa

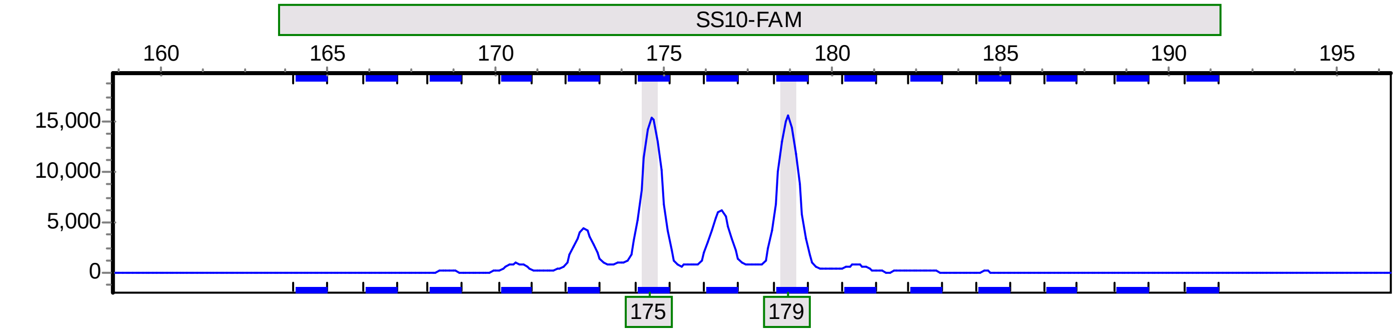

No	Size	Height	Area	Marker	Allele	Difference	Quality	Score	Allele Comments	Sample Comments
1	117.4	21249	142583	SS08-FAM	117	0.00	Pass	500.0	[<Confirmed>]	
2	174.6	15405	105352	SS10-FAM	175	0.10	Pass	500.0	[<Confirmed>]	
3	178.7	15432	107379	SS10-FAM	179	0.10	Pass	500.0	[<Confirmed>]	
4	222.2	8530	62370	SSS42-FAM	222	0.10	Pass	500.0		
5	230.1	10800	77697	SSS42-FAM	230	0.10	Pass	500.0		
6	301.6	3825	36014	SS16-FAM	301	0.10	Pass	456.2		

Allele Report

Sample 81: SS08_SS10_SSS42_SS16_SS27_SS36_SS22_HQZ25_N01.fsa

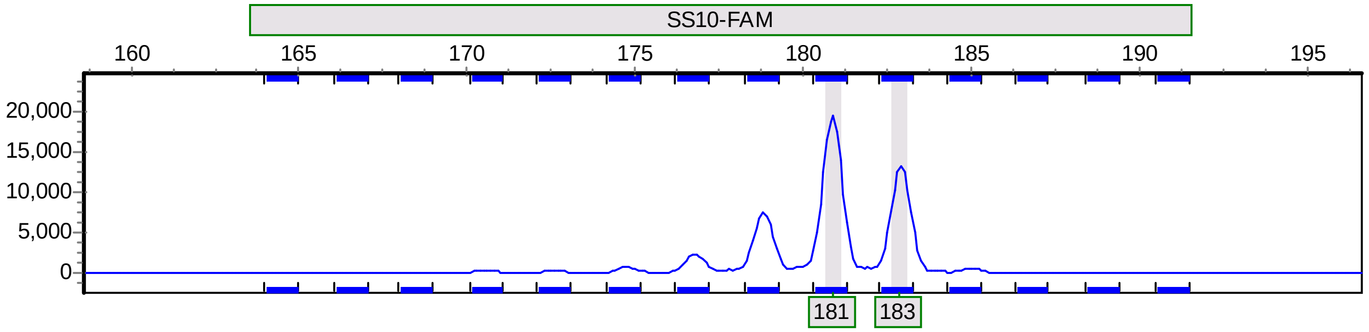

No	Size	Height	Area	Marker	Allele	Difference	Quality	Score	Allele Comments	Sample Comments
1	115.3	13149	87734	SS08-FAM	115	0.10	Pass	500.0	[<Confirmed>]	
2	117.4	14197	93384	SS08-FAM	117	0.00	Pass	500.0	[<Confirmed>]	
3	180.9	19457	129176	SS10-FAM	181	0.10	Pass	500.0	[<Confirmed>]	
4	182.9	13259	88495	SS10-FAM	183	0.10	Pass	500.0	[<Confirmed>]	
5	249.6	3659	27534	SSS42-FAM	250	0.00	Pass	500.0		
6	294.6	17765	147271	SS16-FAM	295	0.00	Pass	500.0		

Sample 82: SS08_SS10_SSS42_SS16_SS27_SS36_SS22_HQZ26_E03.fsa

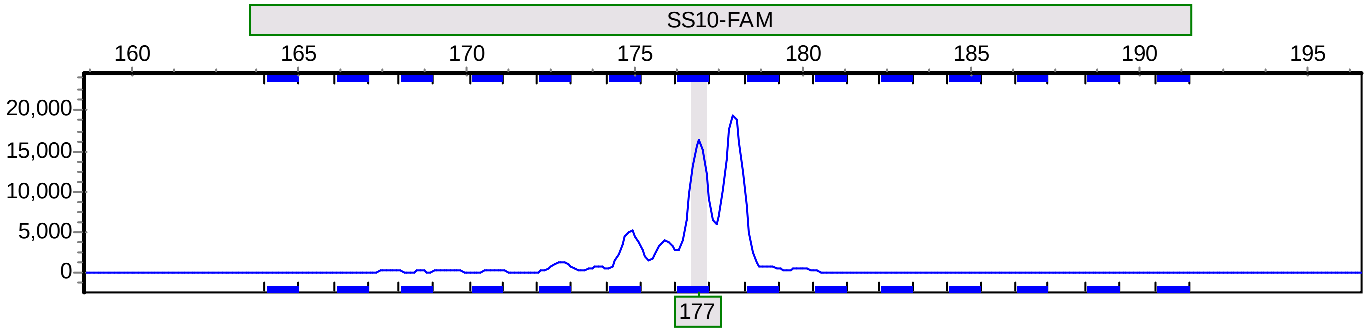

No	Size	Height	Area	Marker	Allele	Difference	Quality	Score	Allele Comments	Sample Comments
1	109.4	14048	90616	SS08-FAM	109	0.10	Pass	500.0	[<Confirmed>]	
2	115.4	15308	96241	SS08-FAM	115	0.00	Pass	500.0	[<Confirmed>]	
3	176.9	16323	108625	SS10-FAM	177	0.20	Pass	500.0	[<Confirmed>]	
4	230.2	15296	105966	SSS42-FAM	230	0.00	Pass	500.0		
5	294.8	10259	84199	SS16-FAM	295	0.20	Pass	500.0		
6	301.5	3659	32474	SS16-FAM	301	0.00	Pass	466.8		

Sample 83: SS08_SS10_SSS42_SS16_SS27_SS36_SS22_HQZ27_G09.fsa

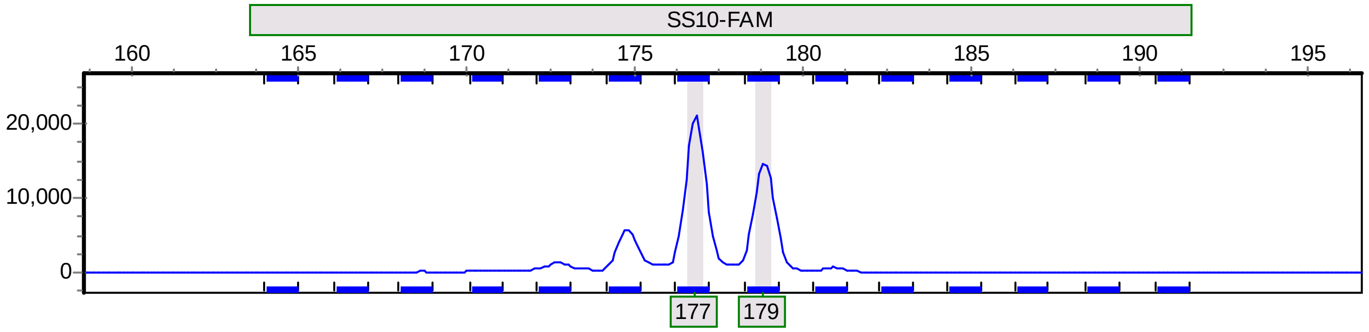

No	Size	Height	Area	Marker	Allele	Difference	Quality	Score	Allele Comments	Sample Comments
1	101.8	17606	116183	SS08-FAM	101	0.00	Pass	500.0	[<Confirmed>]	
2	117.5	12942	86461	SS08-FAM	117	0.10	Pass	500.0	[<Confirmed>]	
3	176.8	20944	142956	SS10-FAM	177	0.10	Pass	500.0	[<Confirmed>]	

Allele Report

4	178.8	14519	103279	SS10-FAM	179	0.00	Pass	500.0	[<Confirmed>]	
5	215.5	11963	84547	SSS42-FAM	216	0.00	Pass	500.0		
6	224.3	4738	34707	SSS42-FAM	224	0.00	Pass	500.0		
7	286.5	15520	131271	SS16-FAM	287	0.00	Pass	500.0		

Sample 84: SS08_SS10_SSS42_SS16_SS27_SS36_SS22_HQZ28_B05.fsa

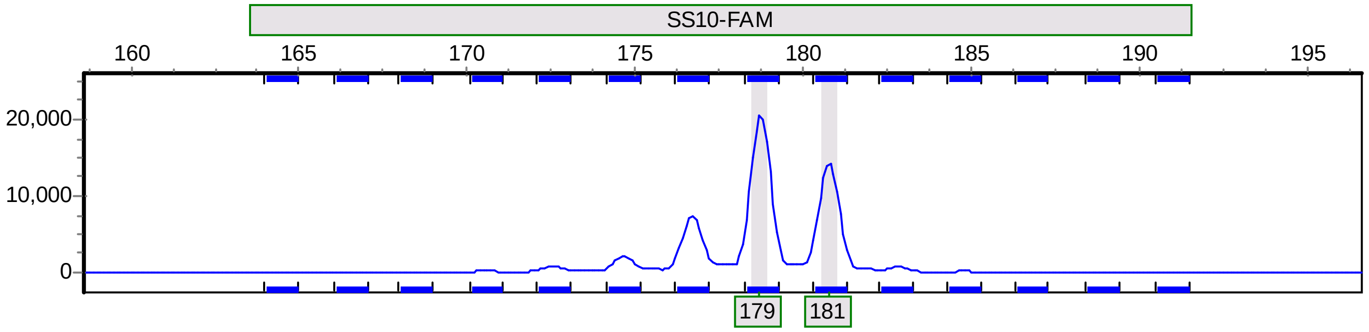

No	Size	Height	Area	Marker	Allele	Difference	Quality	Score	Allele Comments	Sample Comments
1	115.4	20483	136603	SS08-FAM	115	0.00	Pass	500.0	[<Confirmed>]	
2	117.4	15177	100294	SS08-FAM	117	0.00	Pass	500.0	[<Confirmed>]	
3	178.7	20526	138126	SS10-FAM	179	0.10	Pass	500.0	[<Confirmed>]	
4	180.8	14252	100496	SS10-FAM	181	0.00	Pass	500.0	[<Confirmed>]	
5	224.3	12867	93891	SSS42-FAM	224	0.00	Pass	500.0		
6	245.7	7734	58188	SSS42-FAM	246	0.00	Pass	500.0		
7	294.5	11178	99797	SS16-FAM	295	0.10	Pass	500.0		
8	303.5	4763	45717	SS16-FAM	303	0.10	Pass	500.0		

Sample 85: SS08_SS10_SSS42_SS16_SS27_SS36_SS22_HQZ29_C13.fsa

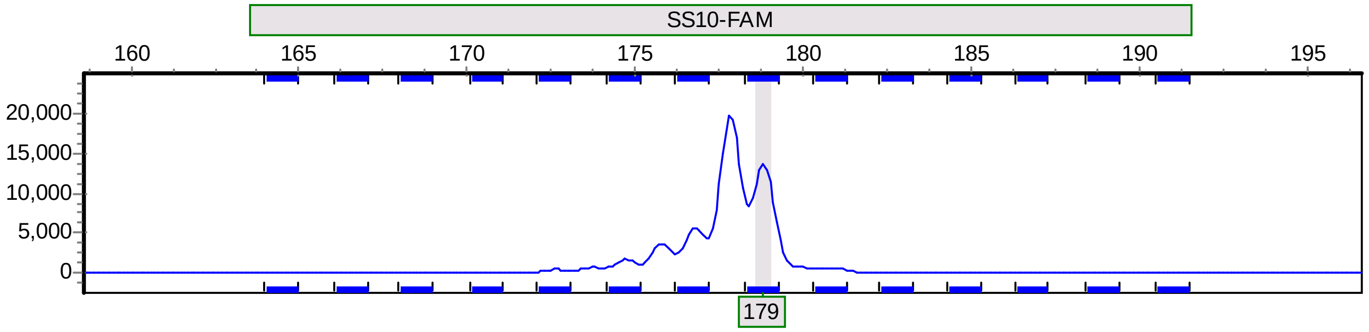

No	Size	Height	Area	Marker	Allele	Difference	Quality	Score	Allele Comments	Sample Comments
1	115.5	6362	45232	SS08-FAM	115	0.10	Pass	500.0	[<Confirmed>]	
2	117.5	5200	37694	SS08-FAM	117	0.10	Pass	500.0	[<Confirmed>]	
3	178.8	13543	99950	SS10-FAM	179	0.00	Pass	500.0	[<Confirmed>]	
4	245.7	5576	44498	SSS42-FAM	246	0.00	Pass	500.0		
5	294.7	12853	118604	SS16-FAM	295	0.10	Pass	500.0		

Allele Report

Sample 86: SS08_SS10_SSS42_SS16_SS27_SS36_SS22_HQZ2_K03.fsa

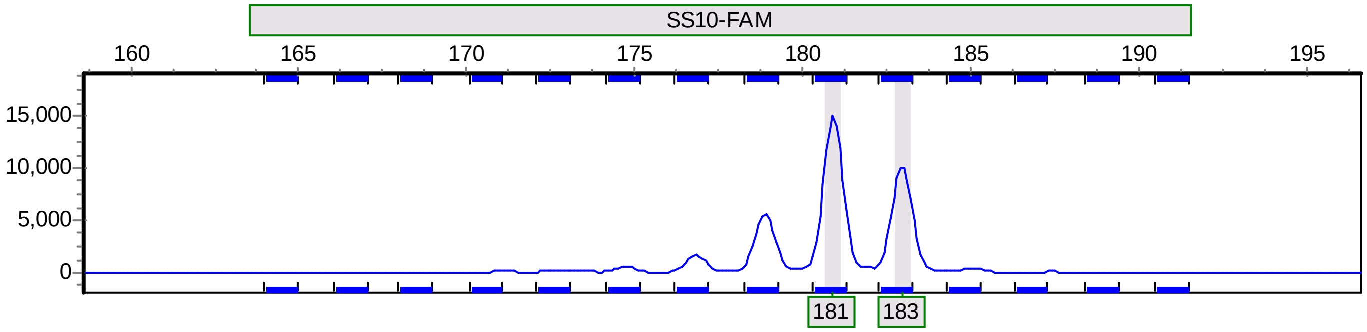

No	Size	Height	Area	Marker	Allele	Difference	Quality	Score	Allele Comments	Sample Comments
1	115.3	16472	108466	SS08-FAM	115	0.10	Pass	500.0	[<Confirmed>]	
2	180.9	14980	100489	SS10-FAM	181	0.10	Pass	500.0	[<Confirmed>]	
3	183.0	10119	70916	SS10-FAM	183	0.20	Pass	500.0	[<Confirmed>]	
4	230.1	8618	61352	SSS42-FAM	230	0.10	Pass	500.0		
5	303.6	7591	71519	SS16-FAM	303	0.00	Pass	500.0		

Sample 87: SS08_SS10_SSS42_SS16_SS27_SS36_SS22_HQZ30_G15.fsa

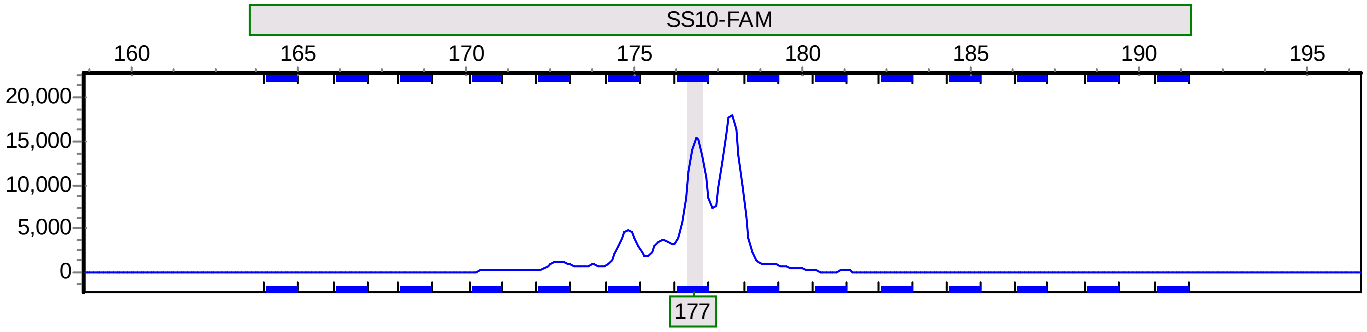

No	Size	Height	Area	Marker	Allele	Difference	Quality	Score	Allele Comments	Sample Comments
1	115.4	15529	106161	SS08-FAM	115	0.00	Pass	500.0	[<Confirmed>]	
2	117.4	11097	77974	SS08-FAM	117	0.00	Pass	500.0	[<Confirmed>]	
3	176.8	15400	110058	SS10-FAM	177	0.10	Pass	500.0	[<Confirmed>]	
4	230.2	17602	133779	SSS42-FAM	230	0.00	Pass	500.0		
5	303.6	4269	42271	SS16-FAM	303	0.00	Pass	482.3		

Sample 88: SS08_SS10_SSS42_SS16_SS27_SS36_SS22_HQZ31_K13.fsa

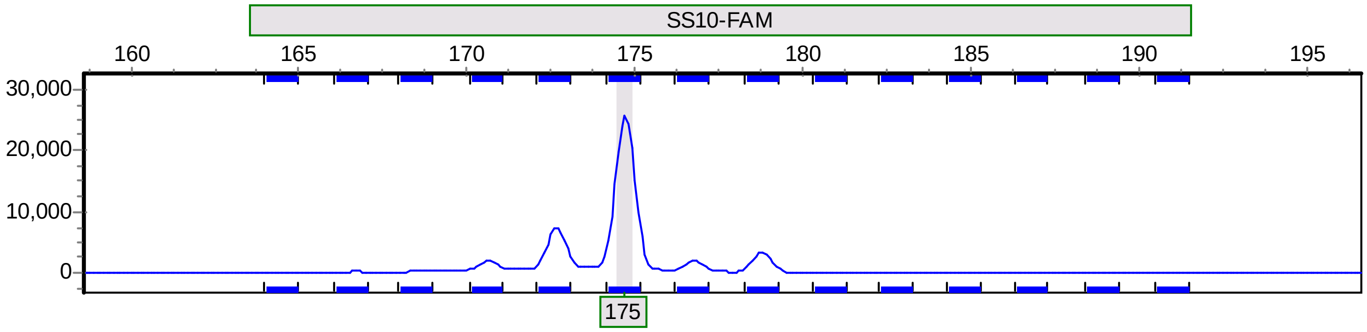

No	Size	Height	Area	Marker	Allele	Difference	Quality	Score	Allele Comments	Sample Comments
1	115.3	19504	137739	SS08-FAM	115	0.10	Pass	500.0	[<Confirmed>]	
2	174.7	25528	169921	SS10-FAM	175	0.00	Pass	500.0	[<Confirmed>]	
3	230.1	11003	79512	SSS42-FAM	230	0.10	Pass	500.0		
4	249.5	3604	27623	SSS42-FAM	250	0.10	Pass	500.0		
5	294.7	8812	75411	SS16-FAM	295	0.10	Pass	500.0		

Allele Report

Sample 89: SS08_SS10_SSS42_SS16_SS27_SS36_SS22_HQZ32_J01.fsa

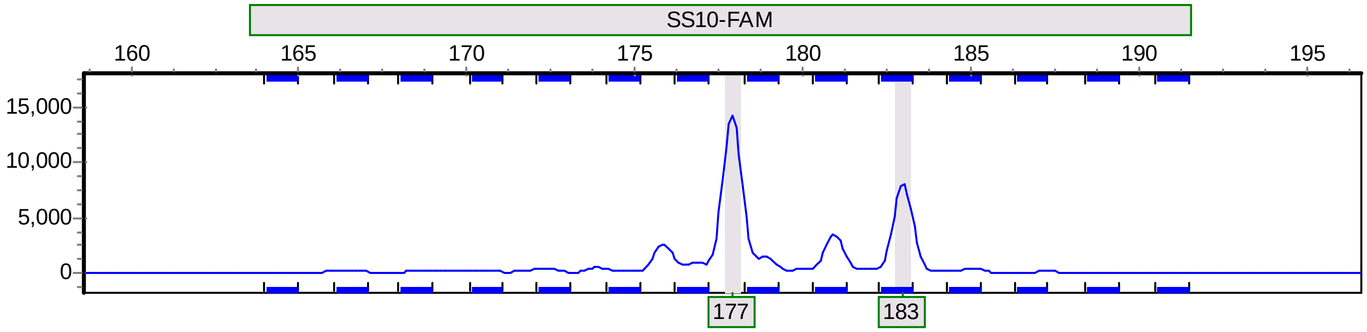

No	Size	Height	Area	Marker	Allele	Difference	Quality	Score	Allele Comments	Sample Comments
1	115.4	10904	70750	SS08-FAM	115	0.00	Pass	500.0	[<Confirmed>]	
2	117.3	8160	53201	SS08-FAM	117	0.10	Pass	500.0	[<Confirmed>]	
3	177.9	14153	94080	SS10-FAM	177	1.00	Pass	500.0	[<Confirmed><Edited>]	
4	183.0	8011	54002	SS10-FAM	183	0.20	Pass	500.0	[<Confirmed>]	
5	249.7	2993	22945	SSS42-FAM	250	0.10	Pass	483.4		
6	294.8	11855	99611	SS16-FAM	295	0.20	Pass	500.0		

Sample 90: SS08_SS10_SSS42_SS16_SS27_SS36_SS22_HQZ33_I03.fsa

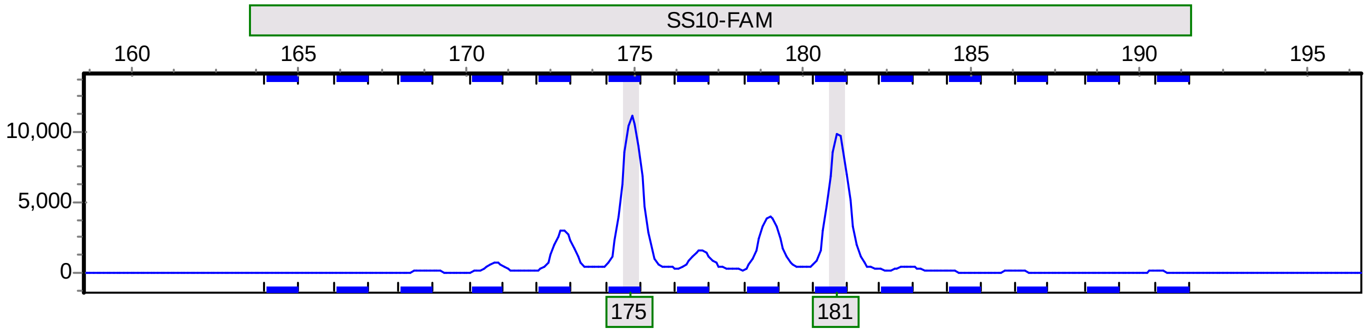

No	Size	Height	Area	Marker	Allele	Difference	Quality	Score	Allele Comments	Sample Comments
1	109.3	9710	64920	SS08-FAM	109	0.00	Pass	500.0	[<Confirmed>]	
2	115.3	9675	63412	SS08-FAM	115	0.10	Pass	500.0	[<Confirmed>]	
3	174.9	11038	75393	SS10-FAM	175	0.20	Pass	500.0	[<Confirmed>]	
4	181.0	9764	68308	SS10-FAM	181	0.20	Pass	500.0	[<Confirmed>]	
5	224.4	6495	48101	SSS42-FAM	224	0.10	Pass	500.0		
6	243.8	2963	23050	SSS42-FAM	244	0.10	Pass	471.0		
7	303.7	4317	40382	SS16-FAM	303	0.10	Pass	500.0		

Sample 91: SS08_SS10_SSS42_SS16_SS27_SS36_SS22_HQZ34_M03.fsa

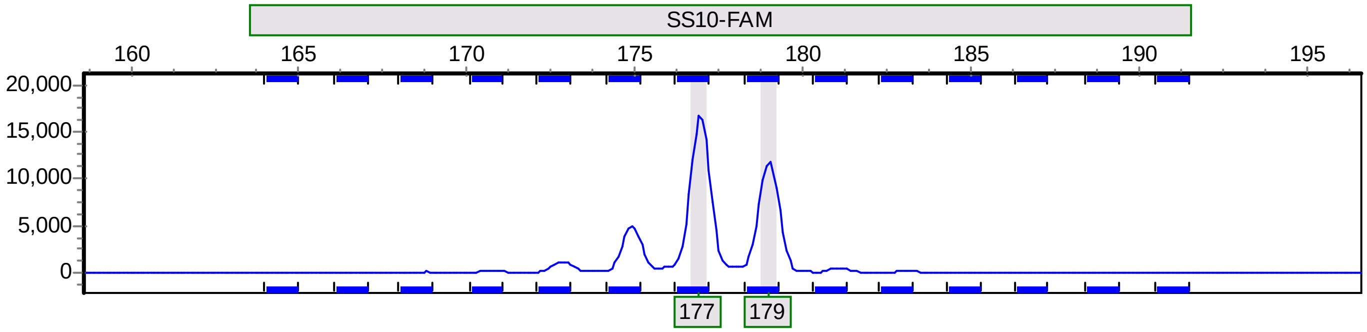

No	Size	Height	Area	Marker	Allele	Difference	Quality	Score	Allele Comments	Sample Comments
1	115.3	11542	78457	SS08-FAM	115	0.10	Pass	500.0	[<Confirmed>]	
2	117.4	11687	77680	SS08-FAM	117	0.00	Pass	500.0	[<Confirmed>]	

3	176.9	16607	111248	SS10-FAM	177	0.20	Pass	500.0	[<Confirmed>]	
4	179.0	11856	80497	SS10-FAM	179	0.20	Pass	500.0	[<Confirmed>]	
5	224.4	6651	47225	SSS42-FAM	224	0.10	Pass	500.0		
6	243.7	2916	21980	SSS42-FAM	244	0.00	Pass	485.9		
7	294.8	9582	81323	SS16-FAM	295	0.20	Pass	500.0		

Sample 92: SS08_SS10_SSS42_SS16_SS27_SS36_SS22_HQZ35_A01.fsa

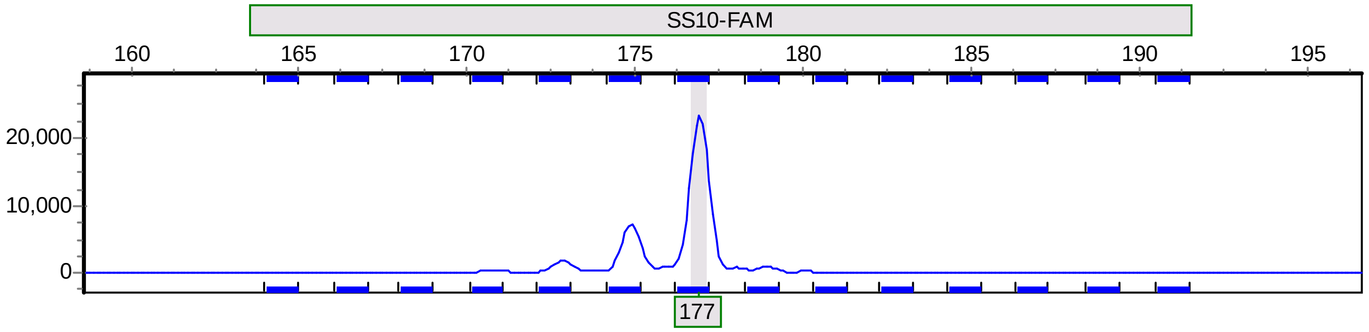

No	Size	Height	Area	Marker	Allele	Difference	Quality	Score	Allele Comments	Sample Comments
1	109.3	13622	88578	SS08-FAM	109	0.00	Pass	500.0	[<Confirmed>]	
2	115.5	14279	93482	SS08-FAM	115	0.10	Pass	500.0	[<Confirmed>]	
3	176.9	23112	149819	SS10-FAM	177	0.20	Pass	500.0	[<Confirmed>]	
4	224.3	6621	45292	SSS42-FAM	224	0.00	Pass	500.0		
5	249.6	3202	22980	SSS42-FAM	250	0.00	Pass	500.0		
6	294.8	10130	82989	SS16-FAM	295	0.20	Pass	500.0		

Sample 93: SS08_SS10_SSS42_SS16_SS27_SS36_SS22_HQZ36_E15.fsa

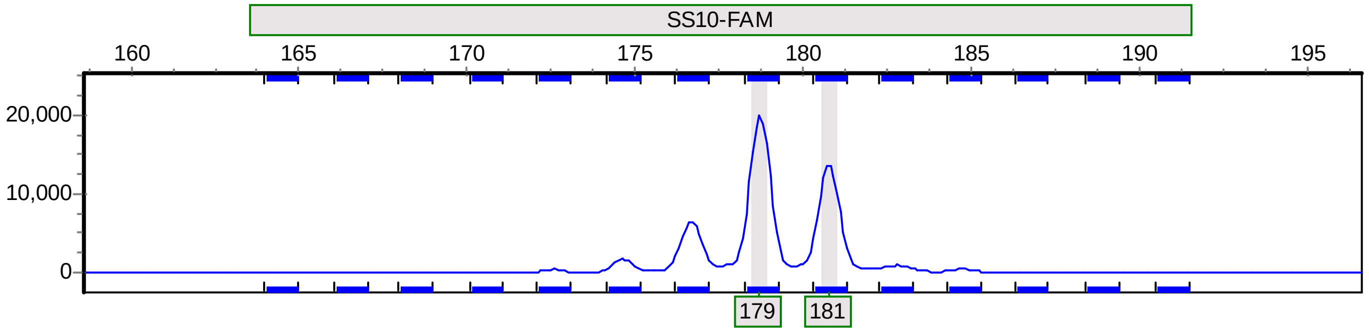

No	Size	Height	Area	Marker	Allele	Difference	Quality	Score	Allele Comments	Sample Comments
1	109.3	13636	91038	SS08-FAM	109	0.00	Pass	500.0	[<Confirmed>]	
2	117.1	11488	78195	SS08-FAM	117	0.30	Pass	500.0	[<Confirmed>]	
3	178.7	19781	136816	SS10-FAM	179	0.10	Pass	500.0	[<Confirmed>]	
4	180.8	13507	98732	SS10-FAM	181	0.00	Pass	500.0	[<Confirmed>]	
5	245.7	6558	51182	SSS42-FAM	246	0.00	Pass	500.0		
6	294.6	11881	104105	SS16-FAM	295	0.00	Pass	500.0		
7	303.5	4554	44782	SS16-FAM	303	0.10	Pass	500.0		

Allele Report

Sample 94: SS08_SS10_SSS42_SS16_SS27_SS36_SS22_HQZ37_F03.fsa

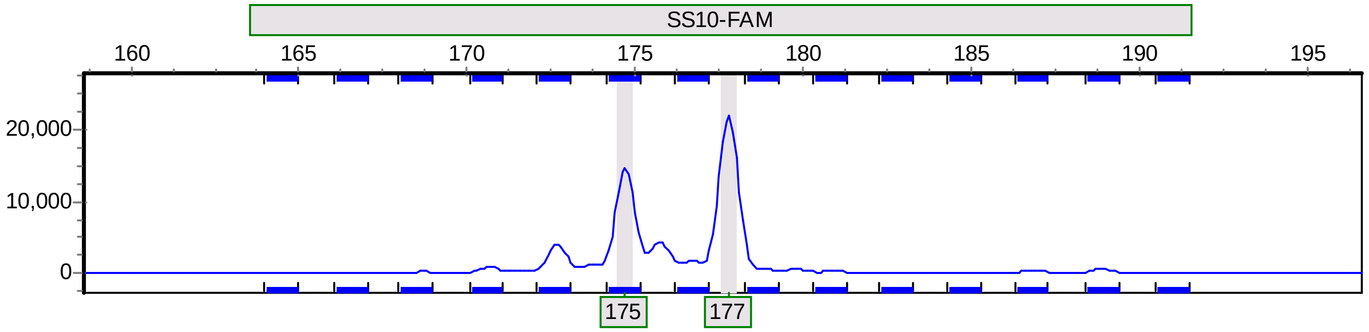

No	Size	Height	Area	Marker	Allele	Difference	Quality	Score	Allele Comments	Sample Comments
1	117.5	26876	170966	SS08-FAM	117	0.10	Pass	500.0	[<Confirmed>]	
2	174.7	14783	100085	SS10-FAM	175	0.00	Pass	500.0	[<Confirmed>]	
3	177.8	21908	145581	SS10-FAM	177	1.00	Pass	500.0	[<Confirmed><Edited>]	
4	224.2	21425	147335	SSS42-FAM	224	0.10	Pass	500.0		
5	282.5	2634	22608	SS16-FAM	283	0.10	Undetermined	317.7		
6	286.5	4259	34803	SS16-FAM	287	0.00	Pass	500.0		
7	299.5	3206	27737	SS16-FAM	299	0.00	Pass	420.0		

Sample 95: SS08_SS10_SSS42_SS16_SS27_SS36_SS22_HQZ38_L01.fsa

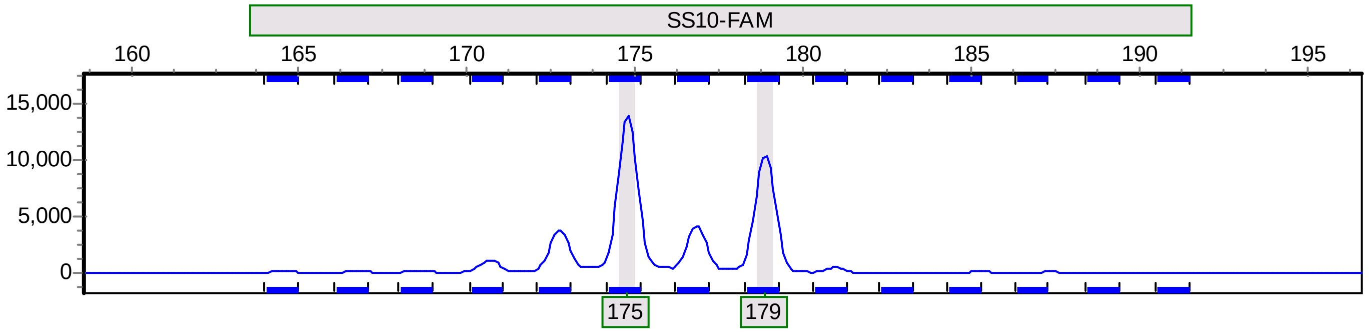

No	Size	Height	Area	Marker	Allele	Difference	Quality	Score	Allele Comments	Sample Comments
1	115.4	9266	64370	SS08-FAM	115	0.00	Pass	500.0	[<Confirmed>]	
2	117.5	9612	63756	SS08-FAM	117	0.10	Pass	500.0	[<Confirmed>]	
3	174.8	13938	93116	SS10-FAM	175	0.10	Pass	500.0	[<Confirmed>]	
4	178.9	10429	70354	SS10-FAM	179	0.10	Pass	500.0	[<Confirmed>]	
5	224.4	7286	52983	SSS42-FAM	224	0.10	Pass	500.0		
6	243.7	3050	23664	SSS42-FAM	244	0.00	Pass	480.6		
7	303.6	4069	38905	SS16-FAM	303	0.00	Pass	478.9		

Sample 96: SS08_SS10_SSS42_SS16_SS27_SS36_SS22_HQZ39_M15.fsa

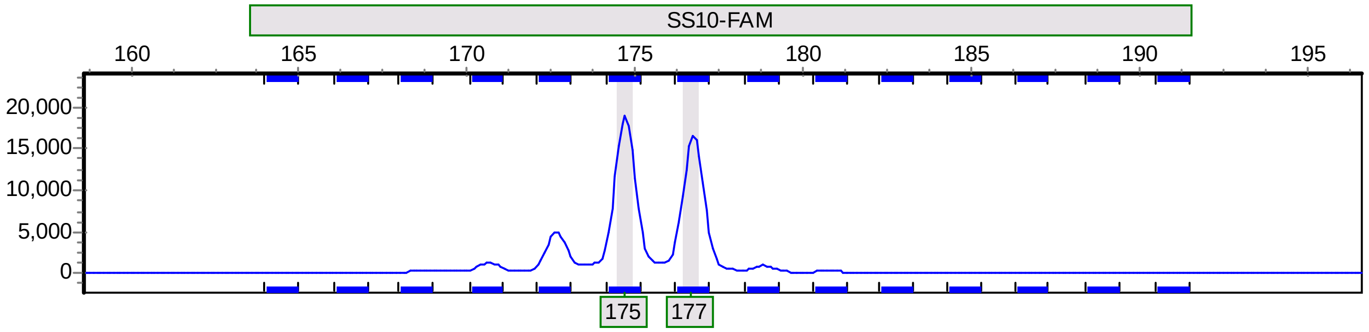

No	Size	Height	Area	Marker	Allele	Difference	Quality	Score	Allele Comments	Sample Comments
1	111.7	14363	99045	SS08-FAM	111	0.20	Pass	500.0	[<Confirmed>]	

2	115.3	10485	73853	SS08-FAM	115	0.10	Pass	500.0	[<Confirmed>]	
3	174.7	18898	133665	SS10-FAM	175	0.00	Pass	500.0	[<Confirmed>]	
4	176.7	16526	117339	SS10-FAM	177	0.00	Pass	500.0	[<Confirmed>]	
5	215.4	11603	84688	SSS42-FAM	216	0.10	Pass	500.0		
6	282.6	16774	144649	SS16-FAM	283	0.00	Pass	500.0		

Sample 97: SS08_SS10_SSS42_SS16_SS27_SS36_SS22_HQZ7_C03.fsa

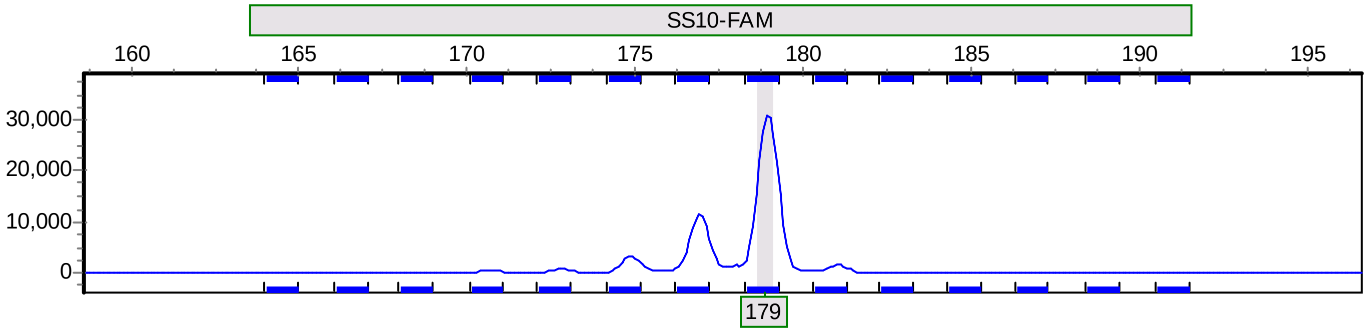

No	Size	Height	Area	Marker	Allele	Difference	Quality	Score	Allele Comments	Sample Comments
1	115.5	28684	198651	SS08-FAM	115	0.10	Pass	500.0	[<Confirmed>]	
2	178.9	30448	210298	SS10-FAM	179	0.10	Pass	500.0	[<Confirmed>]	
3	228.4	13333	92819	SSS42-FAM	228	0.20	Pass	500.0		
4	230.3	11026	76143	SSS42-FAM	230	0.10	Pass	500.0		
5	301.5	5795	50190	SS16-FAM	301	0.00	Pass	500.0		

Sample 98: SS08_SS10_SSS42_SS16_SS27_SS36_SS22_HQZ9_K11.fsa

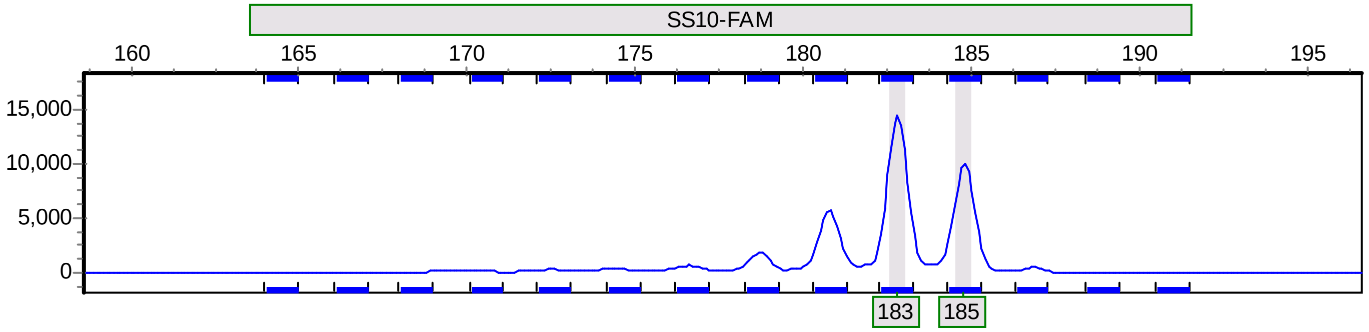

No	Size	Height	Area	Marker	Allele	Difference	Quality	Score	Allele Comments	Sample Comments
1	103.8	12646	85201	SS08-FAM	103	0.10	Pass	500.0	[<Confirmed>]	
2	113.4	8462	58743	SS08-FAM	113	0.00	Pass	500.0	[<Confirmed>]	
3	182.8	14323	98651	SS10-FAM	183	0.00	Pass	500.0	[<Confirmed>]	
4	184.8	9943	69121	SS10-FAM	185	0.00	Pass	500.0	[<Confirmed>]	
5	224.1	5998	42992	SSS42-FAM	224	0.20	Pass	500.0		
6	228.0	4346	31255	SSS42-FAM	228	0.20	Pass	500.0		
7	289.5	13216	115753	SS16-FAM	289	0.50	Pass	500.0		

Allele Report

Sample 99: SS08_SS10_SSS42_SS16_SS27_SS36_SS22_HRS24_I17.fsa

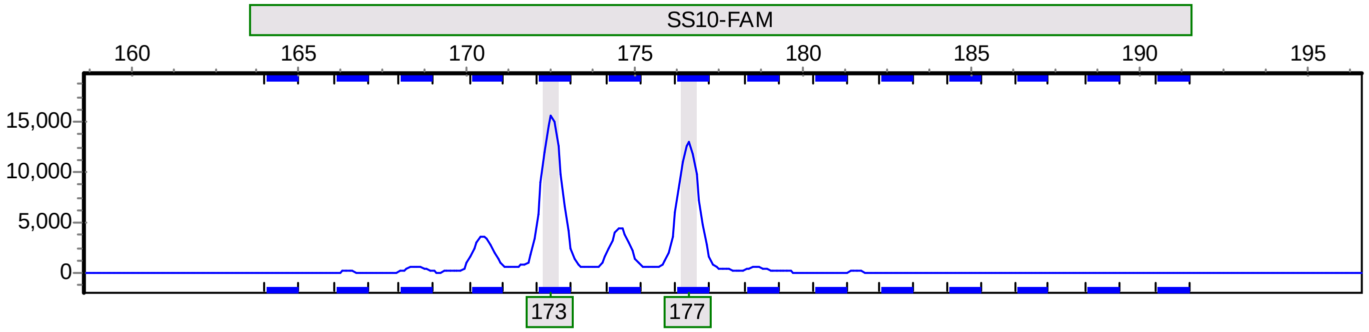

No	Size	Height	Area	Marker	Allele	Difference	Quality	Score	Allele Comments	Sample Comments
1	101.8	17867	120605	SS08-FAM	101	0.00	Pass	500.0	[<Confirmed>]	
2	172.5	15560	107817	SS10-FAM	173	0.10	Pass	500.0	[<Confirmed>]	
3	176.6	13045	90923	SS10-FAM	177	0.10	Pass	500.0	[<Confirmed>]	
4	220.4	10681	79915	SSS42-FAM	220	0.10	Pass	500.0		
5	222.3	8004	60946	SSS42-FAM	222	0.00	Pass	500.0		
6	290.4	13295	120092	SS16-FAM	291	0.10	Pass	500.0		

Sample 100: SS08_SS10_SSS42_SS16_SS27_SS36_SS22_HRS26_H09.fsa

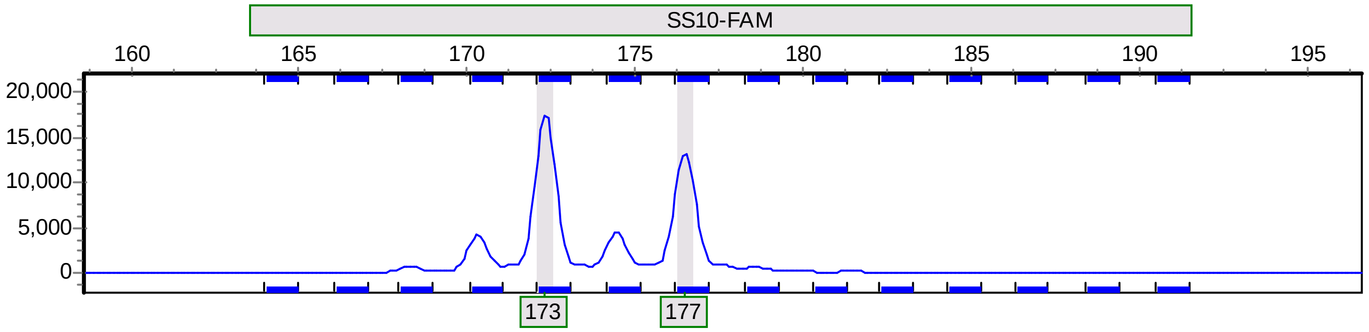

No	Size	Height	Area	Marker	Allele	Difference	Quality	Score	Allele Comments	Sample Comments
1	113.4	15633	107010	SS08-FAM	113	0.00	Pass	500.0	[<Confirmed>]	
2	172.3	17264	123771	SS10-FAM	173	0.30	Pass	500.0	[<Confirmed>]	
3	176.5	13243	95666	SS10-FAM	177	0.20	Pass	500.0	[<Confirmed>]	
4	215.5	10191	79110	SSS42-FAM	216	0.00	Pass	500.0		
5	218.4	10728	79950	SSS42-FAM	218	0.00	Pass	500.0		
6	290.2	13260	116067	SS16-FAM	291	0.30	Pass	500.0		

Sample 101: SS08_SS10_SSS42_SS16_SS27_SS36_SS22_HRS28_D13.fsa

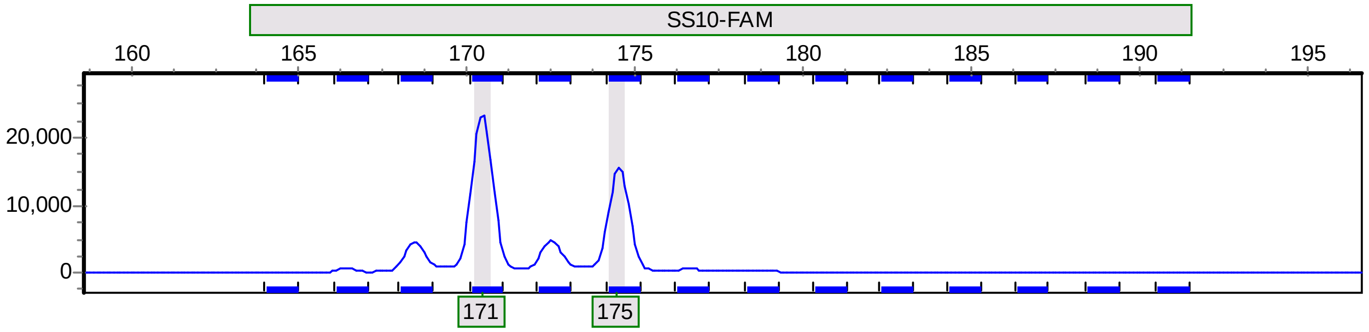

No	Size	Height	Area	Marker	Allele	Difference	Quality	Score	Allele Comments	Sample Comments
1	109.4	19834	133057	SS08-FAM	109	0.10	Pass	500.0	[<Confirmed>]	
2	170.5	23276	164087	SS10-FAM	171	0.10	Pass	500.0	[<Confirmed>]	
3	174.5	15593	110074	SS10-FAM	175	0.20	Pass	500.0	[<Confirmed>]	

4	230.2	13012	98919	SSS42-FAM	230	0.00	Pass	500.0		
5	243.7	4196	32109	SSS42-FAM	244	0.00	Pass	500.0		
6	303.4	3946	38525	SS16-FAM	303	0.20	Pass	434.7		

Sample 102: SS08_SS10_SSS42_SS16_SS27_SS36_SS22_HRS29_J05.fsa

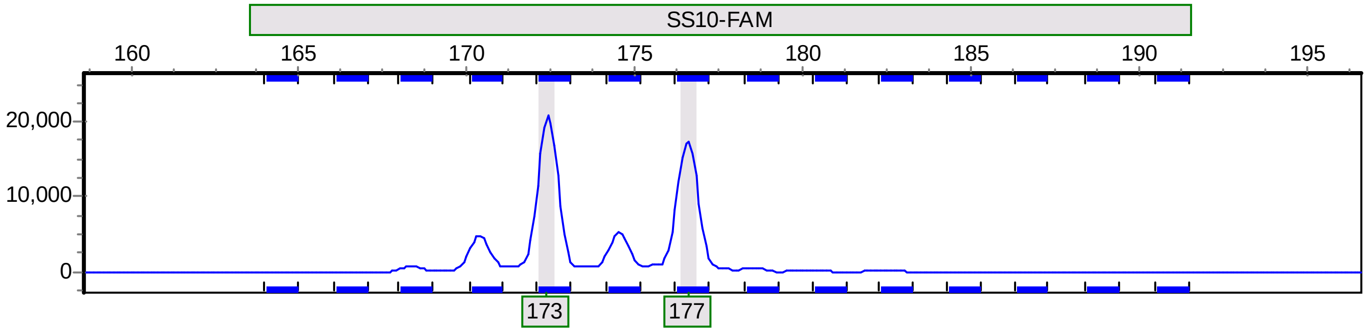

No	Size	Height	Area	Marker	Allele	Difference	Quality	Score	Allele Comments	Sample Comments
1	101.9	14427	96820	SS08-FAM	101	0.10	Pass	500.0	[<Confirmed>]	
2	113.5	8805	59528	SS08-FAM	113	0.10	Pass	500.0	[<Confirmed>]	
3	172.4	20549	140014	SS10-FAM	173	0.20	Pass	500.0	[<Confirmed>]	
4	176.6	17243	120675	SS10-FAM	177	0.10	Pass	500.0	[<Confirmed>]	
5	218.4	13891	102667	SSS42-FAM	218	0.00	Pass	500.0		
6	220.4	11443	85188	SSS42-FAM	220	0.10	Pass	500.0		
7	290.3	17194	151897	SS16-FAM	291	0.20	Pass	500.0		

Sample 103: SS08_SS10_SSS42_SS16_SS27_SS36_SS22_HRS30_P05.fsa

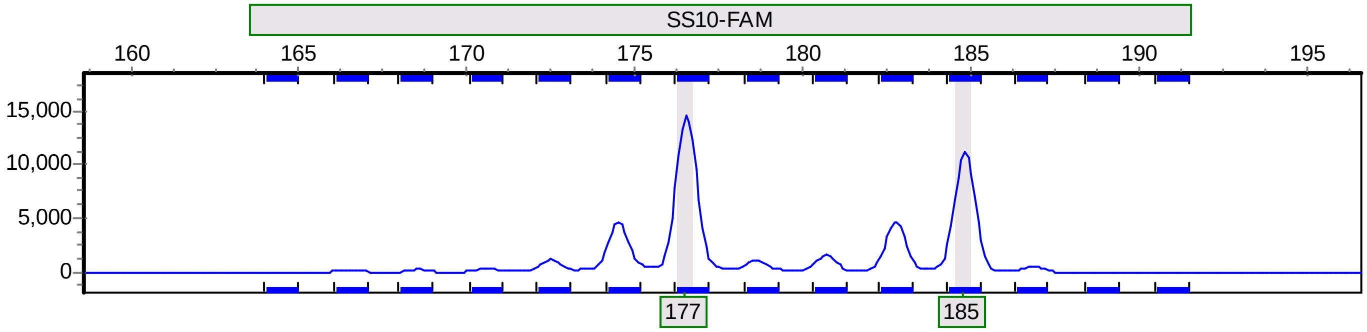

No	Size	Height	Area	Marker	Allele	Difference	Quality	Score	Allele Comments	Sample Comments
1	102.0	12829	84075	SS08-FAM	101	0.20	Pass	500.0	[<Confirmed>]	
2	113.5	8160	55731	SS08-FAM	113	0.10	Pass	500.0	[<Confirmed>]	
3	176.5	14471	100172	SS10-FAM	177	0.20	Pass	500.0	[<Confirmed>]	
4	184.8	11143	78081	SS10-FAM	185	0.00	Pass	500.0	[<Confirmed>]	
5	215.6	8814	64039	SSS42-FAM	216	0.10	Pass	500.0		
6	218.5	8319	61244	SSS42-FAM	218	0.10	Pass	500.0		
7	286.4	11675	102437	SS16-FAM	287	0.10	Pass	500.0		
8	290.3	8165	72643	SS16-FAM	291	0.20	Pass	500.0		

Sample 104: SS08_SS10_SSS42_SS16_SS27_SS36_SS22_HRS31_J11.fsa

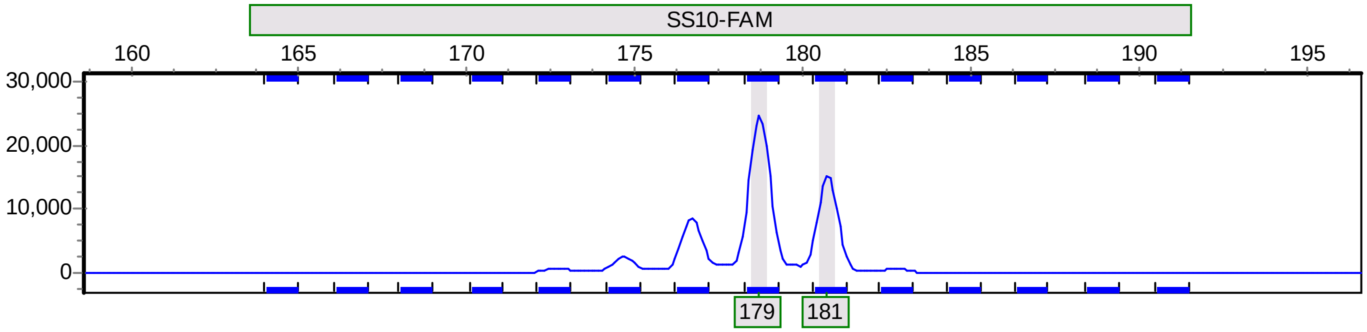

No	Size	Height	Area	Marker	Allele	Difference	Quality	Score	Allele Comments	Sample Comments
1	115.3	10052	72485	SS08-FAM	115	0.10	Pass	500.0	[<Confirmed>]	
2	117.4	10386	71419	SS08-FAM	117	0.00	Pass	500.0	[<Confirmed>]	
3	178.7	24447	171141	SS10-FAM	179	0.10	Pass	500.0	[<Confirmed>]	
4	180.7	15007	104841	SS10-FAM	181	0.10	Pass	500.0	[<Confirmed>]	
5	230.1	20213	146159	SSS42-FAM	230	0.10	Pass	500.0		
6	294.5	10084	82918	SS16-FAM	295	0.10	Pass	500.0		

Sample 105: SS08_SS10_SSS42_SS16_SS27_SS36_SS22_HRS33_H07.fsa

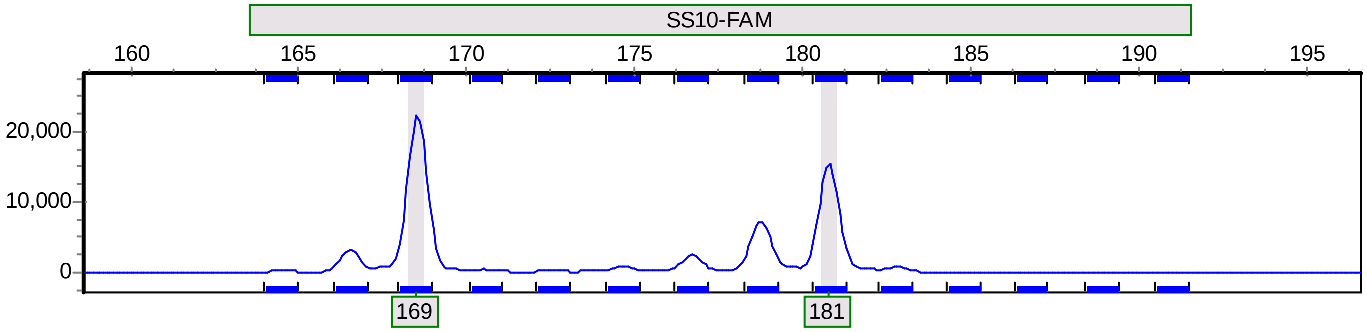

No	Size	Height	Area	Marker	Allele	Difference	Quality	Score	Allele Comments	Sample Comments
1	115.3	13944	97114	SS08-FAM	115	0.10	Pass	500.0	[<Confirmed>]	
2	117.4	14201	94483	SS08-FAM	117	0.00	Pass	500.0	[<Confirmed>]	
3	168.5	22257	150465	SS10-FAM	169	0.00	Pass	500.0	[<Confirmed>]	
4	180.8	15377	106605	SS10-FAM	181	0.00	Pass	500.0	[<Confirmed>]	
5	230.2	11164	84052	SSS42-FAM	230	0.00	Pass	500.0		
6	243.8	4008	31823	SSS42-FAM	244	0.10	Pass	500.0		
7	294.6	10035	90193	SS16-FAM	295	0.00	Pass	500.0		

Sample 106: SS08_SS10_SSS42_SS16_SS27_SS36_SS22_HRS34_L09.fsa

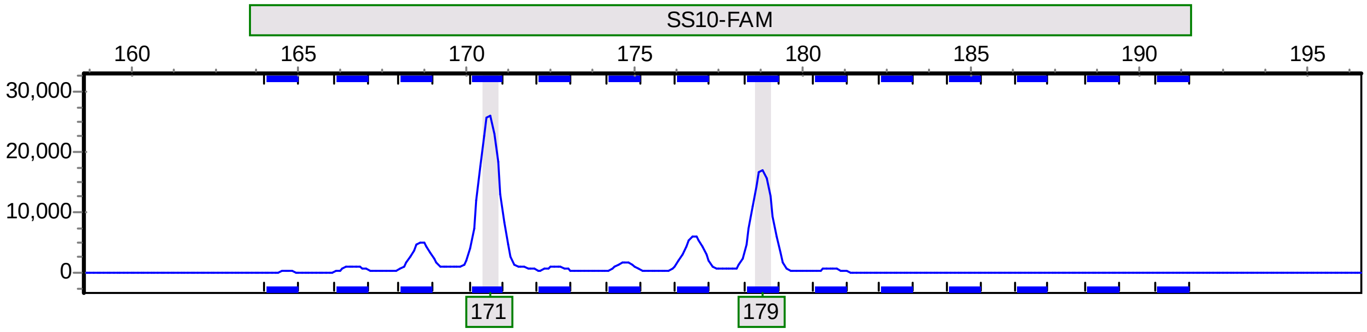

No	Size	Height	Area	Marker	Allele	Difference	Quality	Score	Allele Comments	Sample Comments
1	115.3	23930	155647	SS08-FAM	115	0.10	Pass	500.0	[<Confirmed>]	
2	117.3	17319	114087	SS08-FAM	117	0.10	Pass	500.0	[<Confirmed>]	

3	170.7	26011	176994	SS10-FAM	171	0.10	Pass	500.0	[<Confirmed>]	
4	178.8	17233	117957	SS10-FAM	179	0.00	Pass	500.0	[<Confirmed>]	
5	230.1	27772	205313	SSS42-FAM	230	0.10	Pass	500.0		
6	293.2	14292	124828	SS16-FAM	293	0.20	Pass	500.0		

Sample 107: SS08_SS10_SSS42_SS16_SS27_SS36_SS22_HRS35_P09.fsa

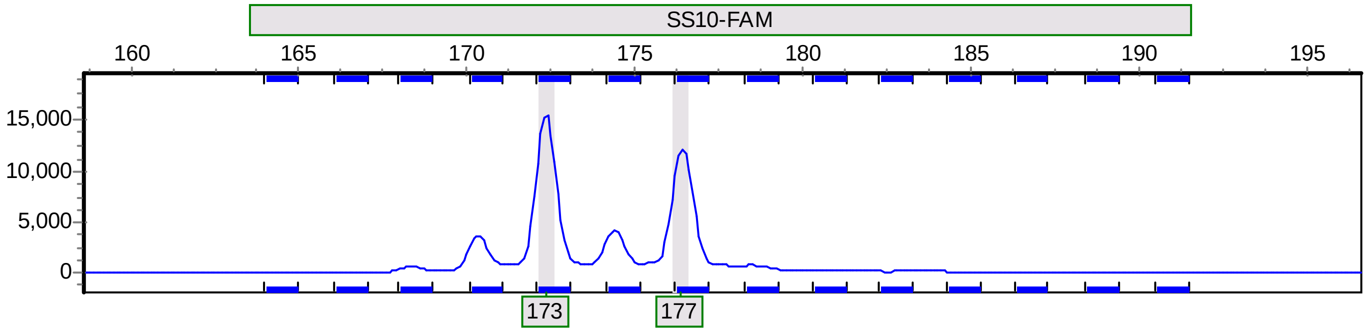

No	Size	Height	Area	Marker	Allele	Difference	Quality	Score	Allele Comments	Sample Comments
1	101.8	15483	103642	SS08-FAM	101	0.00	Pass	500.0	[<Confirmed>]	
2	113.5	9874	66999	SS08-FAM	113	0.10	Pass	500.0	[<Confirmed>]	
3	172.4	15360	104405	SS10-FAM	173	0.20	Pass	500.0	[<Confirmed>]	
4	176.4	12190	87523	SS10-FAM	177	0.30	Pass	500.0	[<Confirmed>]	
5	220.3	10966	83308	SSS42-FAM	220	0.00	Pass	500.0		
6	222.3	8259	63332	SSS42-FAM	222	0.00	Pass	500.0		
7	290.2	14600	129033	SS16-FAM	291	0.30	Pass	500.0		

Sample 108: SS08_SS10_SSS42_SS16_SS27_SS36_SS22_HRS37_J13.fsa

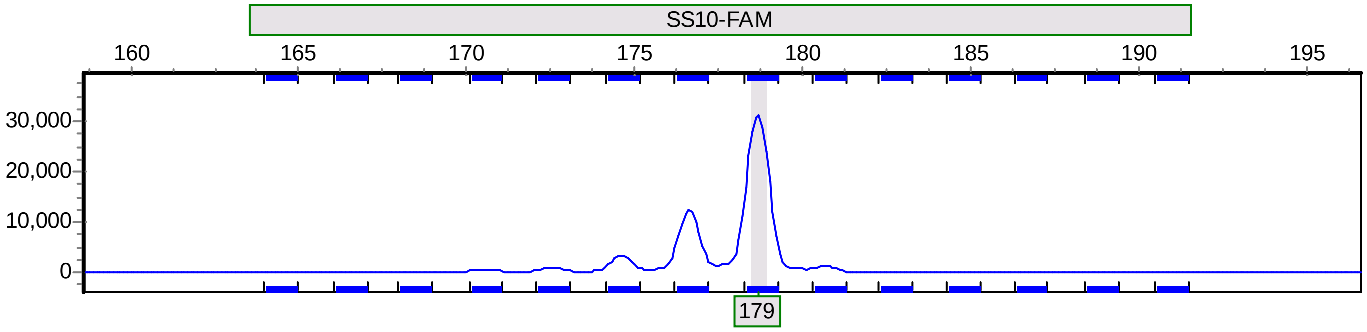

No	Size	Height	Area	Marker	Allele	Difference	Quality	Score	Allele Comments	Sample Comments
1	115.3	14198	98386	SS08-FAM	115	0.10	Pass	500.0	[<Confirmed>]	
2	117.3	10394	72077	SS08-FAM	117	0.10	Pass	500.0	[<Confirmed>]	
3	178.7	31213	234968	SS10-FAM	179	0.10	Pass	500.0	[<Confirmed>]	
4	224.2	11105	85989	SSS42-FAM	224	0.10	Pass	500.0		
5	251.6	3426	27710	SSS42-FAM	252	0.10	Pass	500.0		
6	299.5	2832	26096	SS16-FAM	299	0.00	Pass	295.4		
7	303.6	4974	48506	SS16-FAM	303	0.00	Pass	500.0		

Allele Report

Sample 109: SS08_SS10_SSS42_SS16_SS27_SS36_SS22_HRS38_J07.fsa

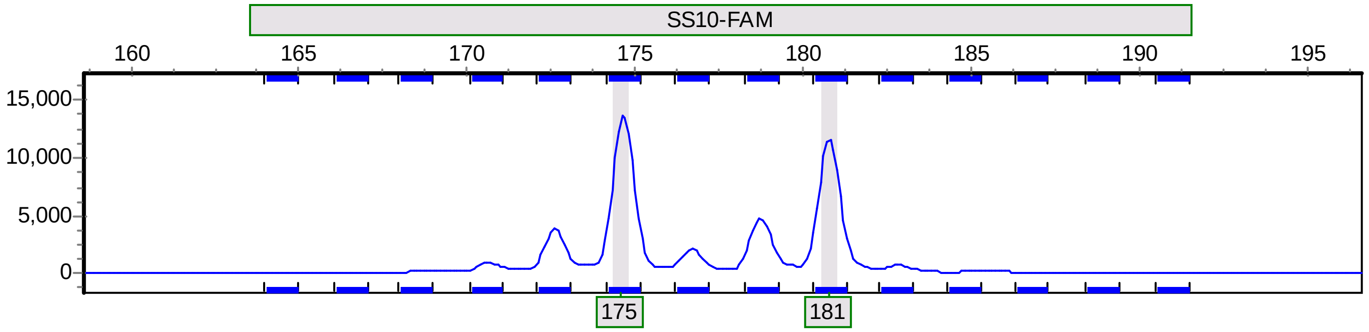

No	Size	Height	Area	Marker	Allele	Difference	Quality	Score	Allele Comments	Sample Comments
1	115.5	20067	145924	SS08-FAM	115	0.10	Pass	500.0	[<Confirmed>]	
2	174.6	13614	99090	SS10-FAM	175	0.10	Pass	500.0	[<Confirmed>]	
3	180.8	11665	85385	SS10-FAM	181	0.00	Pass	500.0	[<Confirmed>]	
4	222.4	12075	92091	SSS42-FAM	222	0.10	Pass	500.0		

Sample 110: SS08_SS10_SSS42_SS16_SS27_SS36_SS22_HRS39_N07.fsa

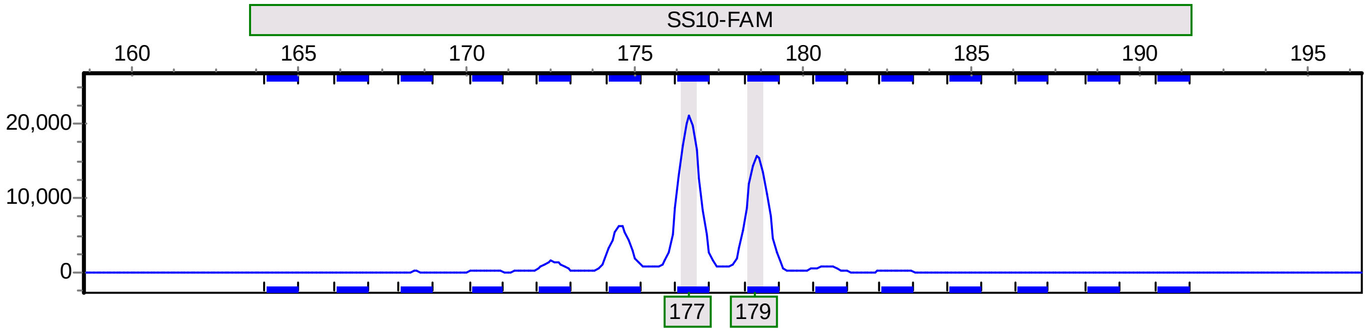

No	Size	Height	Area	Marker	Allele	Difference	Quality	Score	Allele Comments	Sample Comments
1	115.4	26503	185068	SS08-FAM	115	0.00	Pass	500.0	[<Confirmed>]	
2	176.6	20897	145728	SS10-FAM	177	0.10	Pass	500.0	[<Confirmed>]	
3	178.6	15723	110632	SS10-FAM	179	0.20	Pass	500.0	[<Confirmed>]	
4	228.2	17798	135367	SSS42-FAM	228	0.00	Pass	500.0		
5	294.5	11991	109580	SS16-FAM	295	0.10	Pass	500.0		
6	303.5	5078	49956	SS16-FAM	303	0.10	Pass	500.0		

Sample 111: SS08_SS10_SSS42_SS16_SS27_SS36_SS22_HRS40_A07.fsa

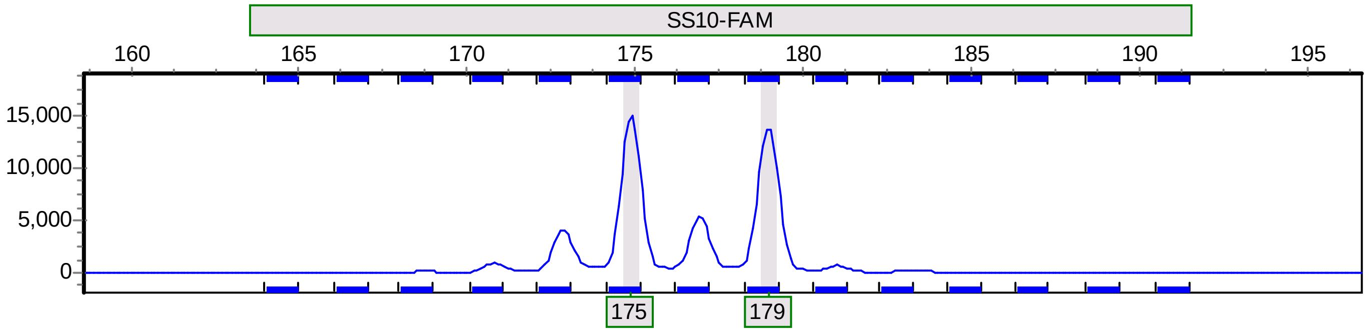

No	Size	Height	Area	Marker	Allele	Difference	Quality	Score	Allele Comments	Sample Comments
1	115.3	25622	174765	SS08-FAM	115	0.10	Pass	500.0	[<Confirmed>]	
2	174.9	14934	100106	SS10-FAM	175	0.20	Pass	500.0	[<Confirmed>]	
3	179.0	13683	96115	SS10-FAM	179	0.20	Pass	500.0	[<Confirmed>]	
4	224.3	11426	82550	SSS42-FAM	224	0.00	Pass	500.0		
5	294.7	9660	80995	SS16-FAM	295	0.10	Pass	500.0		

6	303.6	3833	35020	SS16-FAM	303	0.00	Pass	480.8	

Sample 112:

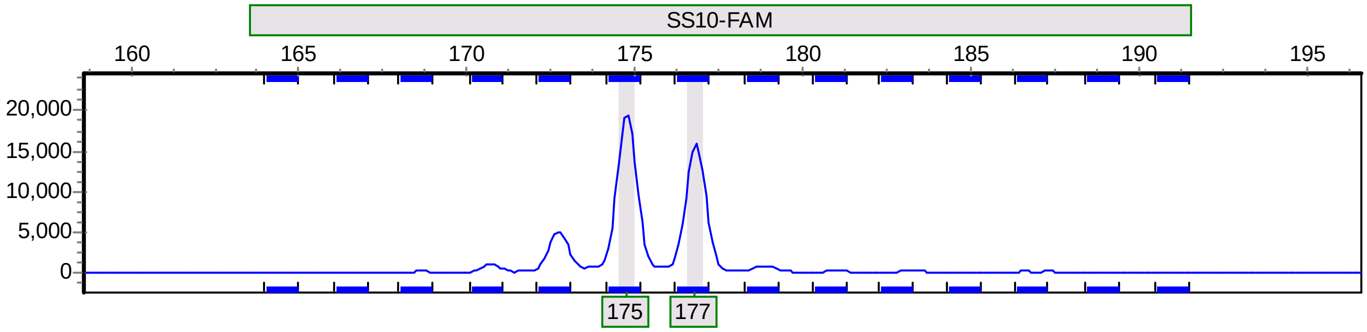

No	Size	Height	Area	Marker	Allele	Difference	Quality	Score	Allele Comments	Sample Comments
1	109.2	27957	184947	SS08-FAM	109	0.10	Pass	500.0	[<Confirmed>]	
2	174.8	19237	131786	SS10-FAM	175	0.10	Pass	500.0	[<Confirmed>]	
3	176.8	15892	106987	SS10-FAM	177	0.10	Pass	500.0	[<Confirmed>]	
4	228.4	10773	79635	SSS42-FAM	228	0.20	Pass	500.0		
5	230.3	8737	64987	SSS42-FAM	230	0.10	Pass	500.0		
6	303.7	7166	68688	SS16-FAM	303	0.10	Pass	500.0		

Sample 113:

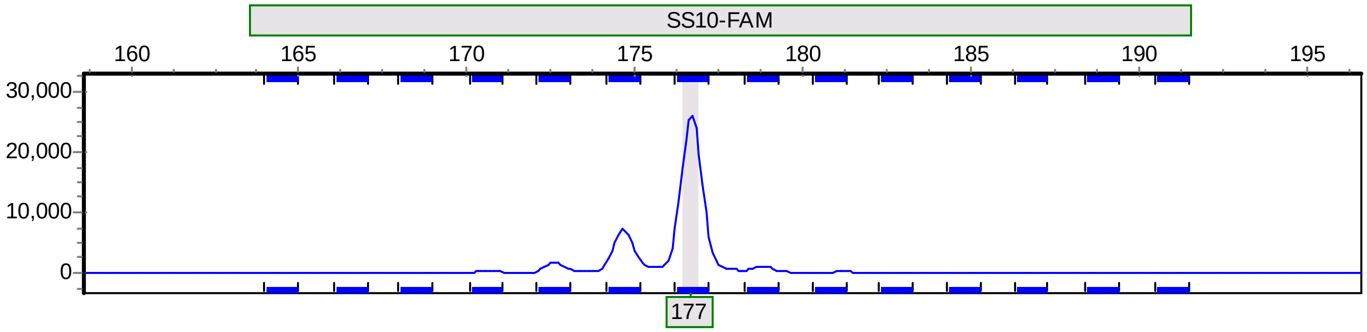

No	Size	Height	Area	Marker	Allele	Difference	Quality	Score	Allele Comments	Sample Comments
1	104.0	12458	83451	SS08-FAM	103	0.10	Pass	500.0	[<Confirmed>]	
2	113.4	9077	61790	SS08-FAM	113	0.00	Pass	500.0	[<Confirmed>]	
3	176.7	25970	184322	SS10-FAM	177	0.00	Pass	500.0	[<Confirmed>]	
4	218.3	9724	73545	SSS42-FAM	218	0.10	Pass	500.0		
5	220.3	8376	62815	SSS42-FAM	220	0.00	Pass	500.0		
6	282.5	8666	75320	SS16-FAM	283	0.10	Pass	500.0		
7	289.5	12562	110860	SS16-FAM	289	0.50	Pass	500.0		

Sample 114:

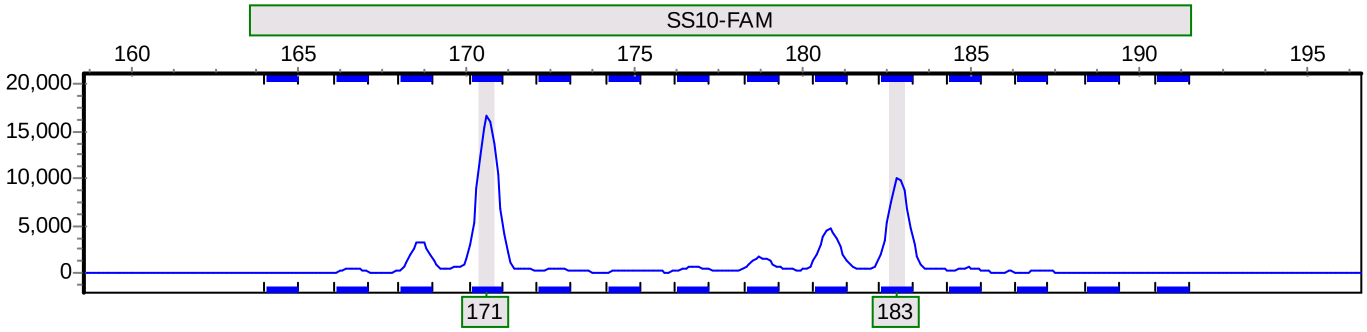

No	Size	Height	Area	Marker	Allele	Difference	Quality	Score	Allele Comments	Sample Comments
1	115.3	24963	164266	SS08-FAM	115	0.10	Pass	500.0	[<Confirmed>]	

2	170.6	16575	110654	SS10-FAM	171	0.00	Pass	500.0	[<Confirmed>]	
3	182.8	10064	70486	SS10-FAM	183	0.00	Pass	500.0	[<Confirmed>]	
4	224.2	8192	60106	SSS42-FAM	224	0.10	Pass	500.0		
5	249.6	3671	28311	SSS42-FAM	250	0.00	Pass	500.0		
6	294.7	7495	65410	SS16-FAM	295	0.10	Pass	500.0		
7	303.7	3142	29401	SS16-FAM	303	0.10	Pass	335.4		

Sample 115: SS08_SS10_SSS42_SS16_SS27_SS36_SS22_HTHL13_H01.fsa

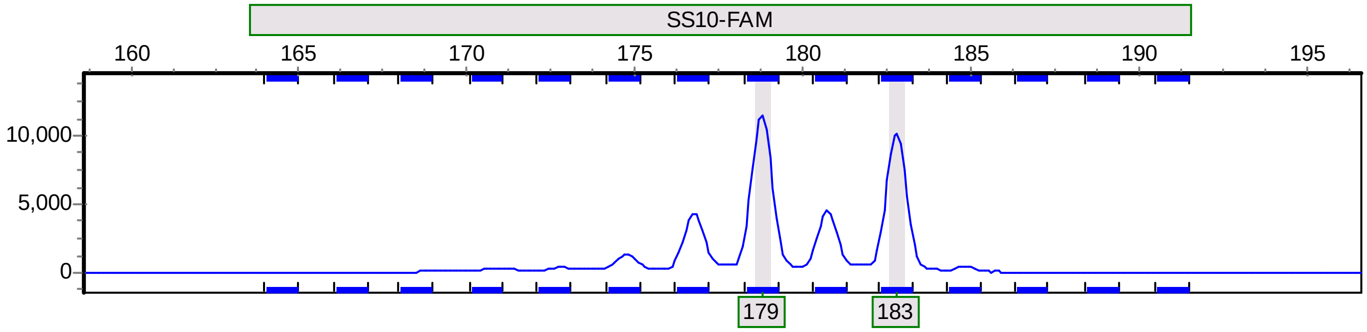

No	Size	Height	Area	Marker	Allele	Difference	Quality	Score	Allele Comments	Sample Comments
1	115.2	9188	61621	SS08-FAM	115	0.20	Pass	500.0	[<Confirmed>]	
2	125.1	5676	37743	SS08-FAM	125	0.00	Pass	500.0	[<Confirmed>]	
3	178.8	11459	80072	SS10-FAM	179	0.00	Pass	500.0	[<Confirmed>]	
4	182.8	10240	71206	SS10-FAM	183	0.00	Pass	500.0	[<Confirmed>]	
5	224.3	11139	77982	SSS42-FAM	224	0.00	Pass	500.0		
6	289.4	22162	186523	SS16-FAM	289	0.40	Pass	500.0		

Sample 116: SS08_SS10_SSS42_SS16_SS27_SS36_SS22_HTHL14_F15.fsa

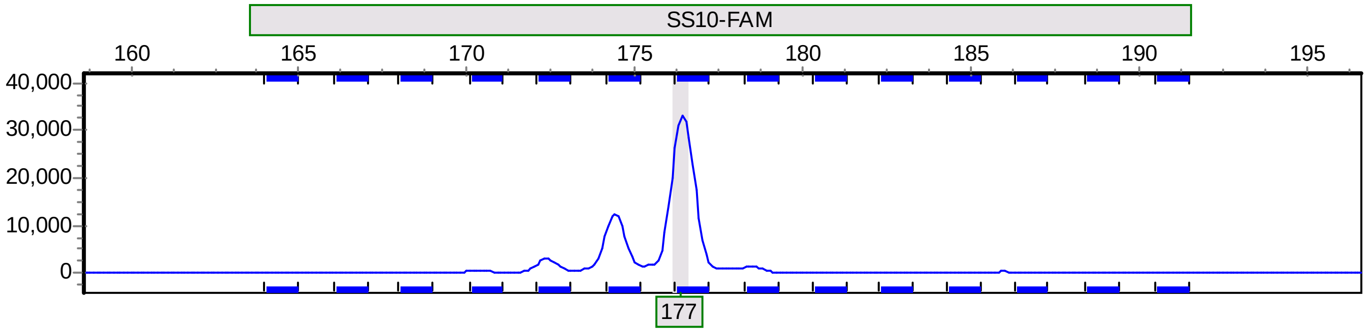

No	Size	Height	Area	Marker	Allele	Difference	Quality	Score	Allele Comments	Sample Comments
1	115.5	15955	108160	SS08-FAM	115	0.10	Pass	500.0	[<Confirmed>]	
2	117.5	11885	83742	SS08-FAM	117	0.10	Pass	500.0	[<Confirmed>]	
3	176.4	32958	249615	SS10-FAM	177	0.30	Pass	500.0	[<SAT (Repaired)><Confirmed>]	
4	230.1	12563	97459	SSS42-FAM	230	0.10	Pass	500.0		
5	251.5	3842	31761	SSS42-FAM	252	0.00	Pass	500.0		
6	303.4	6034	61754	SS16-FAM	303	0.20	Pass	500.0		

Sample 117: SS08_SS10_SSS42_SS16_SS27_SS36_SS22_HTHL15_F01.fsa

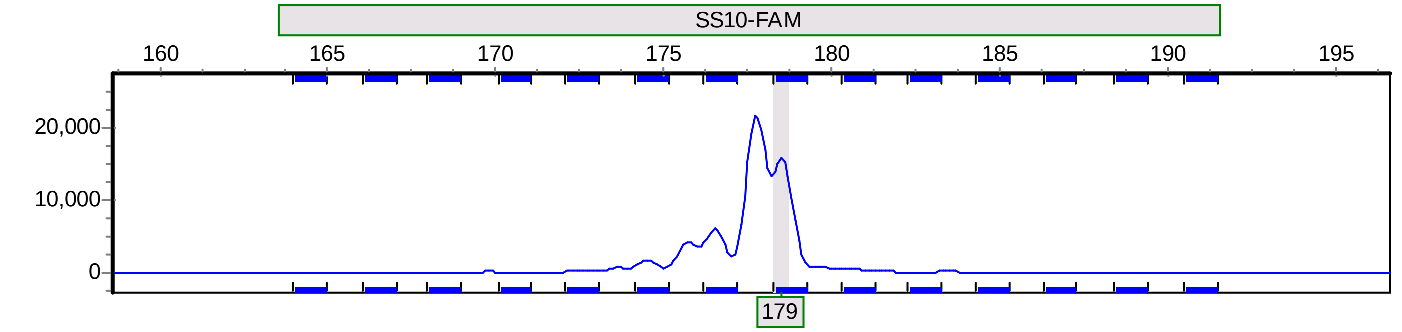

No	Size	Height	Area	Marker	Allele	Difference	Quality	Score	Allele Comments	Sample Comments
1	102.1	19016	121174	SS08-FAM	101	0.30	Pass	500.0	[<Confirmed>]	
2	115.5	16245	104808	SS08-FAM	115	0.10	Pass	500.0	[<Confirmed>]	
3	178.5	15707	258069	SS10-FAM	179	0.30	Pass	500.0	[<Confirmed><Inserted>]	
4	220.4	16329	113375	SSS42-FAM	220	0.10	Pass	500.0		
5	230.2	9596	69006	SSS42-FAM	230	0.00	Pass	500.0		
6	286.4	19348	157243	SS16-FAM	287	0.10	Pass	500.0		

Sample 118: SS08_SS10_SSS42_SS16_SS27_SS36_SS22_HTHL1_E13.fsa

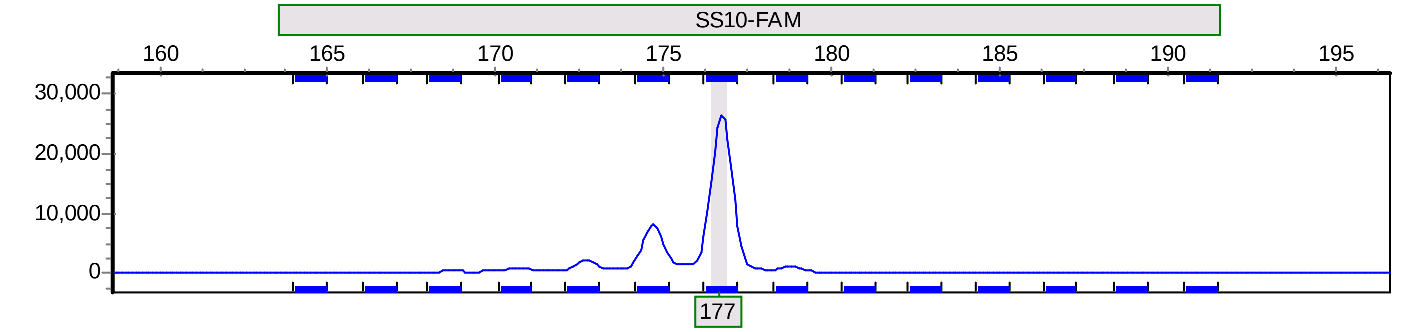

No	Size	Height	Area	Marker	Allele	Difference	Quality	Score	Allele Comments	Sample Comments
1	115.2	13807	95603	SS08-FAM	115	0.20	Pass	500.0	[<Confirmed>]	
2	176.7	26252	187692	SS10-FAM	177	0.00	Pass	500.0	[<Confirmed>]	
3	218.3	9498	73058	SSS42-FAM	218	0.10	Pass	500.0		
4	220.3	8123	60312	SSS42-FAM	220	0.00	Pass	500.0		
5	288.5	10287	90332	SS16-FAM	289	0.50	Pass	500.0		

Sample 119: SS08_SS10_SSS42_SS16_SS27_SS36_SS22_HTHL3_D05.fsa

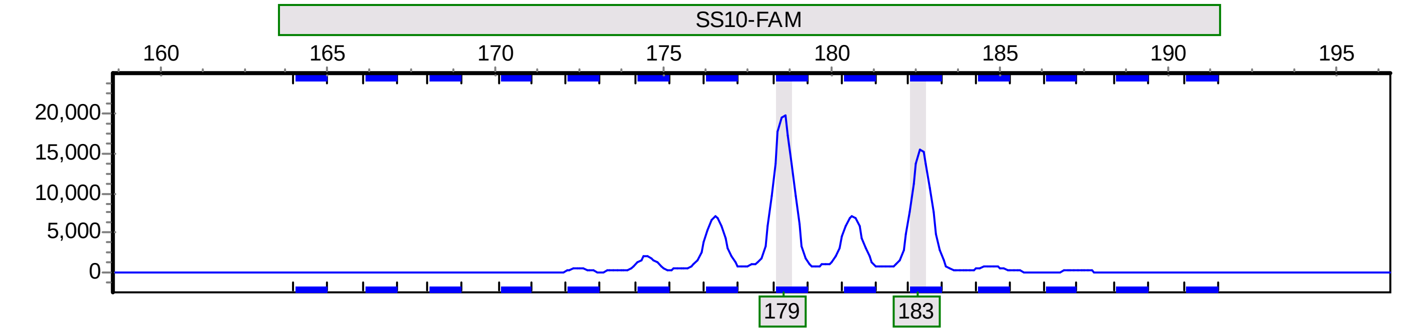

No	Size	Height	Area	Marker	Allele	Difference	Quality	Score	Allele Comments	Sample Comments
1	101.9	18896	126583	SS08-FAM	101	0.10	Pass	500.0	[<Confirmed>]	
2	178.6	19590	134908	SS10-FAM	179	0.20	Pass	500.0	[<Confirmed>]	
3	182.6	15377	106614	SS10-FAM	183	0.20	Pass	500.0	[<Confirmed>]	
4	218.4	18876	131480	SSS42-FAM	218	0.00	Pass	500.0		

5	311.7	5364	50349	SS16-FAM	311	0.00	Pass	500.0		

Sample 120: SS08_SS10_SSS42_SS16_SS27_SS36_SS22_HTHL4_O15.fsa

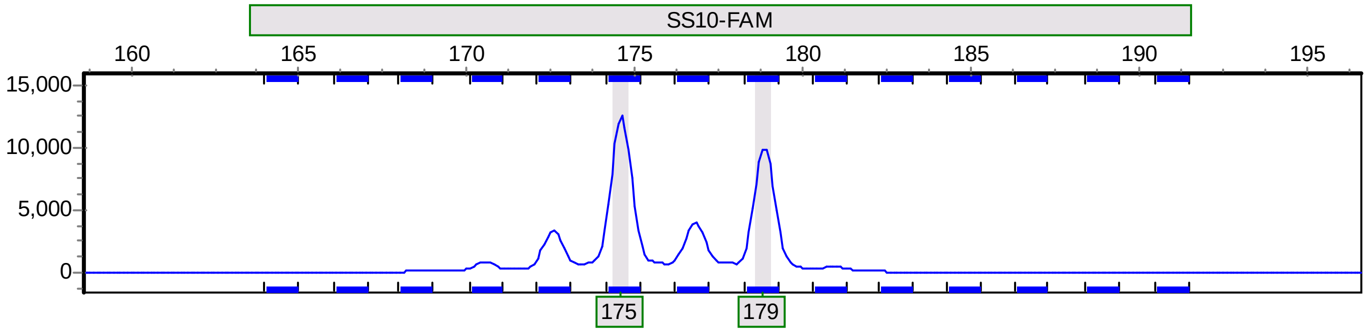

No	Size	Height	Area	Marker	Allele	Difference	Quality	Score	Allele Comments	Sample Comments
1	105.6	10641	72018	SS08-FAM	105	0.00	Pass	500.0	[<Confirmed>]	
2	117.3	8844	60473	SS08-FAM	117	0.10	Pass	500.0	[<Confirmed>]	
3	174.6	12527	90419	SS10-FAM	175	0.10	Pass	500.0	[<Confirmed>]	
4	178.8	9858	70708	SS10-FAM	179	0.00	Pass	500.0	[<Confirmed>]	
5	248.2	7129	56440	SSS42-FAM	248	0.50	Pass	500.0		
6	288.5	9304	82237	SS16-FAM	289	0.50	Pass	500.0		

Sample 121: SS08_SS10_SSS42_SS16_SS27_SS36_SS22_HTHL5_M13.fsa

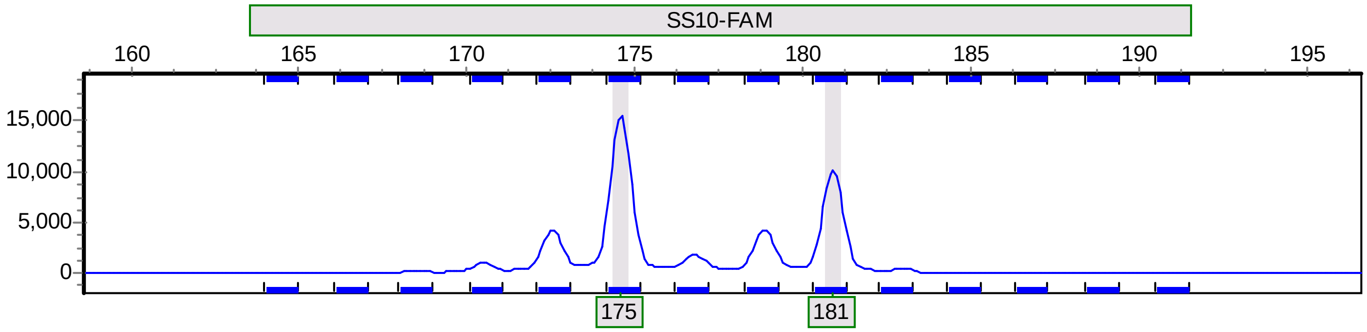

No	Size	Height	Area	Marker	Allele	Difference	Quality	Score	Allele Comments	Sample Comments
1	115.3	15735	108694	SS08-FAM	115	0.10	Pass	500.0	[<Confirmed>]	
2	174.6	15407	110773	SS10-FAM	175	0.10	Pass	500.0	[<Confirmed>]	
3	180.9	10119	72575	SS10-FAM	181	0.10	Pass	500.0	[<Confirmed>]	
4	215.5	9335	69428	SSS42-FAM	216	0.00	Pass	500.0		
5	228.2	5955	45027	SSS42-FAM	228	0.00	Pass	500.0		
6	286.5	12578	114140	SS16-FAM	287	0.00	Pass	500.0		

Sample 122: SS08_SS10_SSS42_SS16_SS27_SS36_SS22_HTHL6_P03.fsa

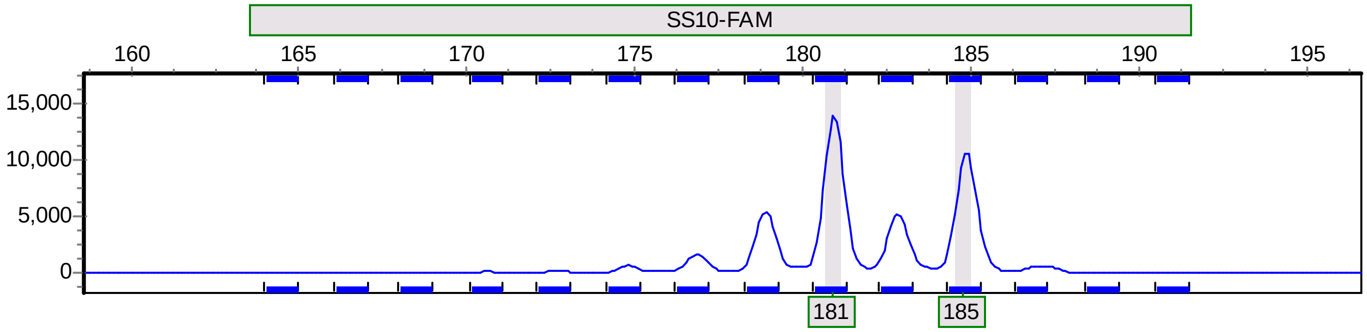

No	Size	Height	Area	Marker	Allele	Difference	Quality	Score	Allele Comments	Sample Comments
1	115.4	14461	92496	SS08-FAM	115	0.00	Pass	500.0	[<Confirmed>]	
2	180.9	13929	94620	SS10-FAM	181	0.10	Pass	500.0	[<Confirmed>]	

3	184.8	10519	75054	SS10-FAM	185	0.00	Pass	500.0	[<Confirmed>]	
4	222.3	6595	46251	SSS42-FAM	222	0.00	Pass	500.0		
5	243.8	7391	56276	SSS42-FAM	244	0.10	Pass	500.0		
6	286.4	15863	137435	SS16-FAM	287	0.10	Pass	500.0		

Sample 123: SS08_SS10_SSS42_SS16_SS27_SS36_SS22_HTHL8_A03.fsa

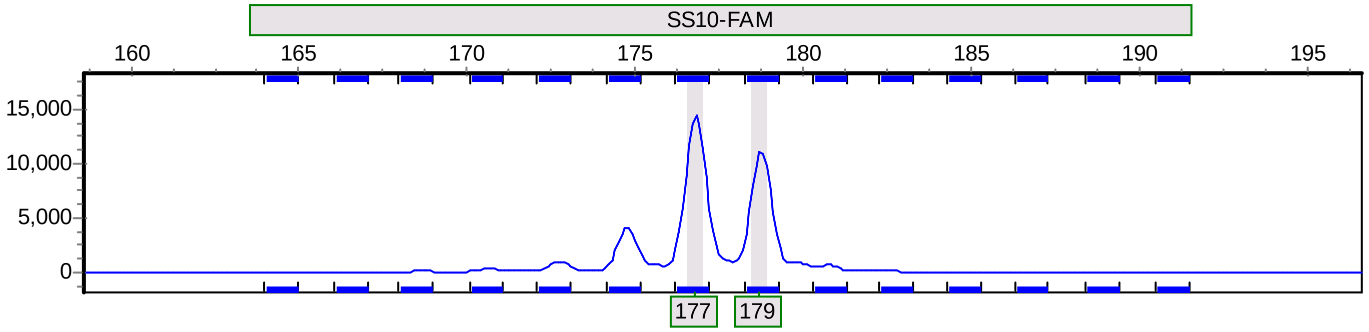

No	Size	Height	Area	Marker	Allele	Difference	Quality	Score	Allele Comments	Sample Comments
1	111.6	19112	129628	SS08-FAM	111	0.10	Pass	500.0	[<Confirmed>]	
2	176.8	14326	101669	SS10-FAM	177	0.10	Pass	500.0	[<Confirmed>]	
3	178.7	11048	77730	SS10-FAM	179	0.10	Pass	500.0	[<Confirmed>]	
4	215.5	6090	43165	SSS42-FAM	216	0.00	Pass	500.0		
5	220.3	5632	40947	SSS42-FAM	220	0.00	Pass	500.0		

Sample 124: SS08_SS10_SSS42_SS16_SS27_SS36_SS22_HTHL9_O09.fsa

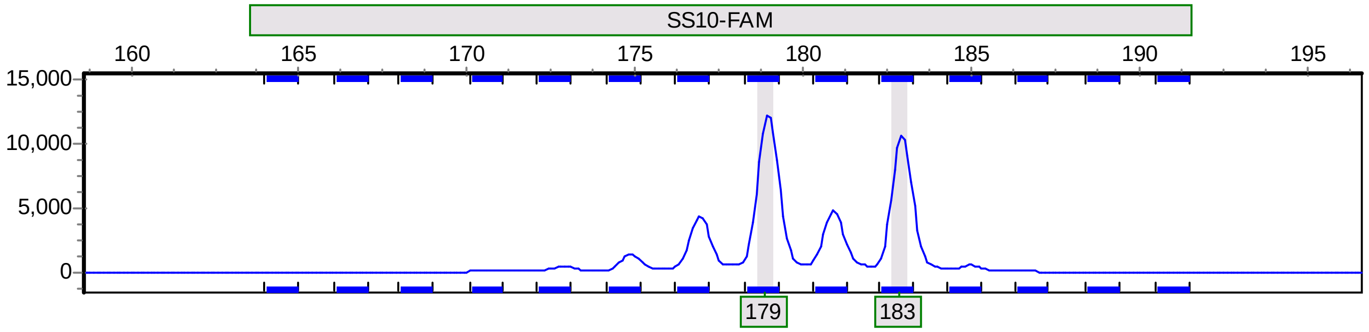

No	Size	Height	Area	Marker	Allele	Difference	Quality	Score	Allele Comments	Sample Comments
1	109.2	12964	88629	SS08-FAM	109	0.10	Pass	500.0	[<Confirmed>]	
2	113.4	10244	69496	SS08-FAM	113	0.00	Pass	500.0	[<Confirmed>]	
3	178.9	12109	87111	SS10-FAM	179	0.10	Pass	500.0	[<Confirmed>]	
4	182.9	10620	74604	SS10-FAM	183	0.10	Pass	500.0	[<Confirmed>]	
5	222.2	3535	26706	SSS42-FAM	222	0.10	Pass	500.0		
6	230.2	9088	67163	SSS42-FAM	230	0.00	Pass	500.0		
7	286.5	12150	105250	SS16-FAM	287	0.00	Pass	500.0		
