# Supplementary material for: Genetic diversity of Schima superba based on physiological traits and SSR markers
Source: PLoS One. 2026 Apr 10;21(4):e0344465. doi: 10.1371/journal.pone.0344465 (PMC13068225; doi:10.1371/journal.pone.0344465)
Supplement: S1 File — (ZIP) [file pone.0344465.s003.zip › SS19.pdf]

**SoftGenetics**

# Allele Report

10/11/2024 11:24:33 AM

GeneMarker V3.0.0



**Project Comments:**

**Sample 1:** SSS13_SS20_SS11_SS21_SS02_SS19_HBB10_E05.fsa

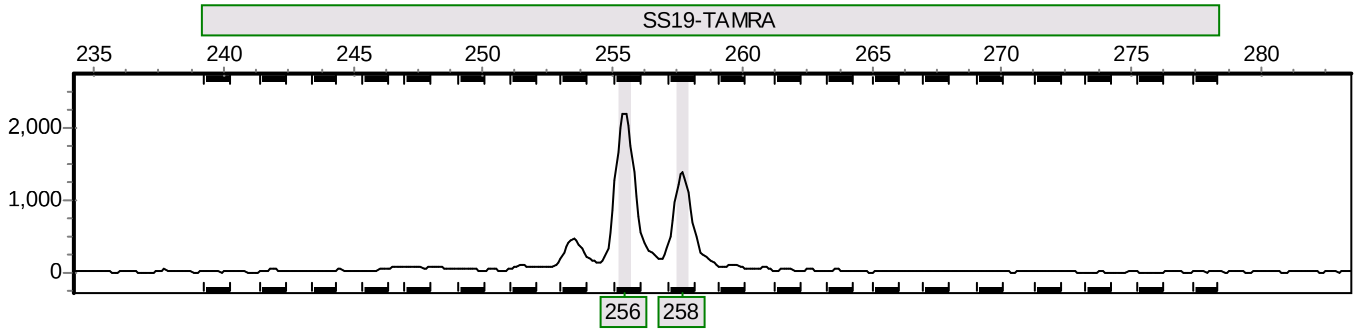


| No | Size | Height | Area | Marker | Allele | Difference | Quality | Score | Allele Comments | Sample Comments |
|---|---|---|---|---|---|---|---|---|---|---|
| 1 | 255.5 | 2202 | 17682 | SS19-TAMRA | 256 | 0.10 | Pass | 269.4 | [<Confirmed>] | |
| 2 | 257.7 | 1390 | 10965 | SS19-TAMRA | 258 | 0.00 | Pass | 147.7 | [<Confirmed>] | |

**Sample 2:** SSS13_SS20_SS11_SS21_SS02_SS19_HBB12-2_D11.fsa

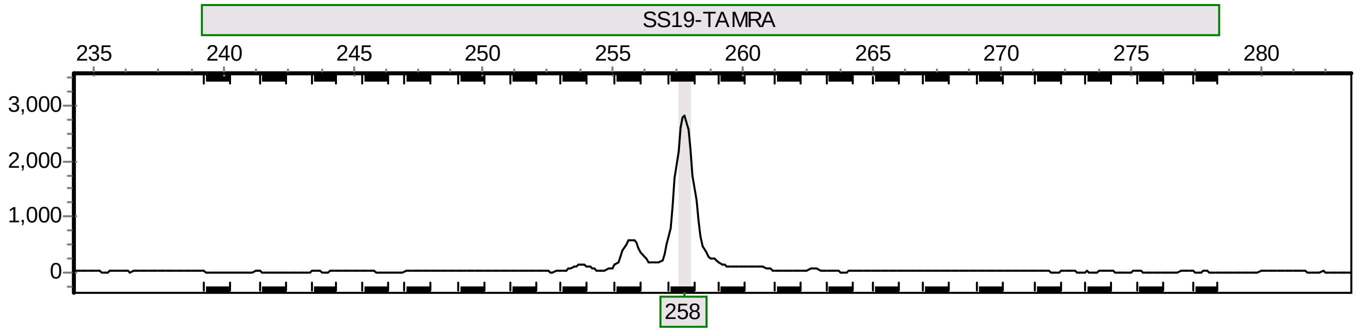


| No | Size | Height | Area | Marker | Allele | Difference | Quality | Score | Allele Comments | Sample Comments |
|---|---|---|---|---|---|---|---|---|---|---|
| 1 | 257.8 | 2807 | 22860 | SS19-TAMRA | 258 | 0.10 | Pass | 375.7 | [<Confirmed>] | |

**Sample 3:** SSS13_SS20_SS11_SS21_SS02_SS19_HBB13_C17.fsa

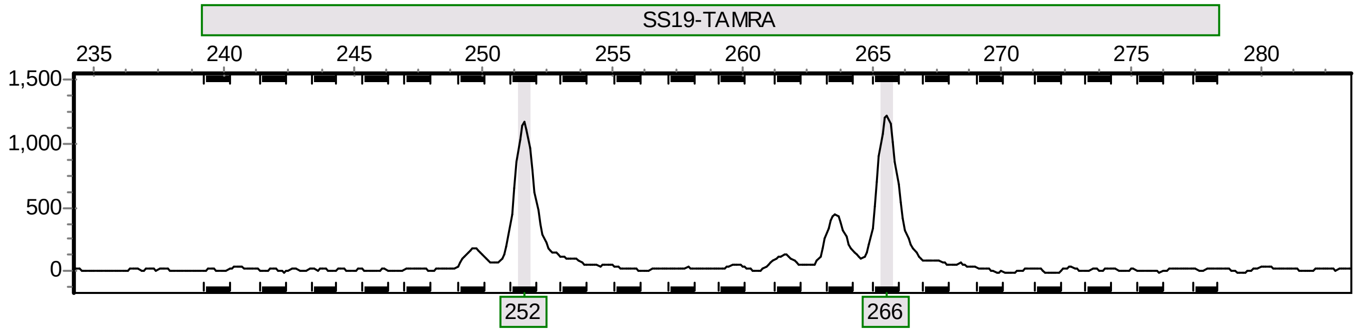


| No | Size | Height | Area | Marker | Allele | Difference | Quality | Score | Allele Comments | Sample Comments |
|---|---|---|---|---|---|---|---|---|---|---|
| 1 | 251.6 | 1168 | 9898 | SS19-TAMRA | 252 | 0.00 | Pass | 100.4 | [<Confirmed>] | |
| 2 | 265.6 | 1214 | 10626 | SS19-TAMRA | 266 | 0.00 | Pass | 97.7 | [<Confirmed>] | |

**Sample 4:** SSS13_SS20_SS11_SS21_SS02_SS19_HBB14_G03.fsa

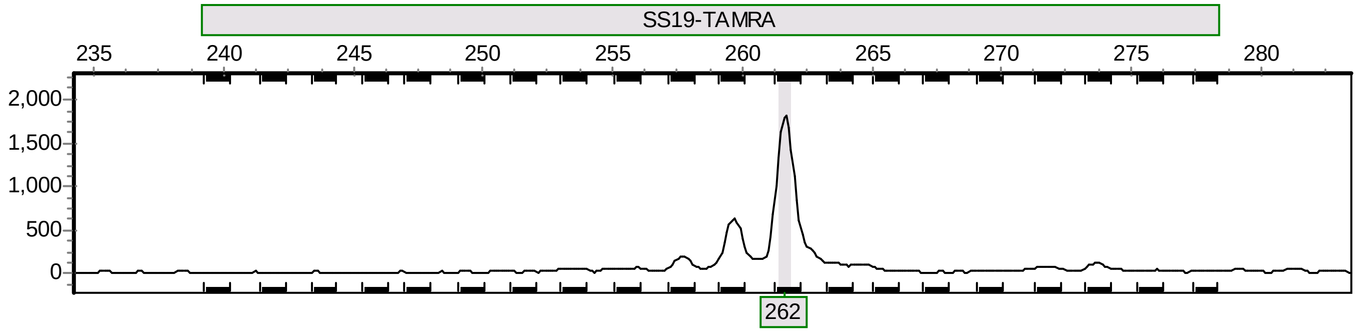


| No | Size | Height | Area | Marker | Allele | Difference | Quality | Score | Allele Comments | Sample Comments |
|---|---|---|---|---|---|---|---|---|---|---|
| 1 | 261.7 | 1811 | 14178 | SS19-TAMRA | 262 | 0.10 | Pass | 218.7 | [<Confirmed>] | |

**Sample 5:** SSS13_SS20_SS11_SS21_SS02_SS19_HBB15_B07.fsa

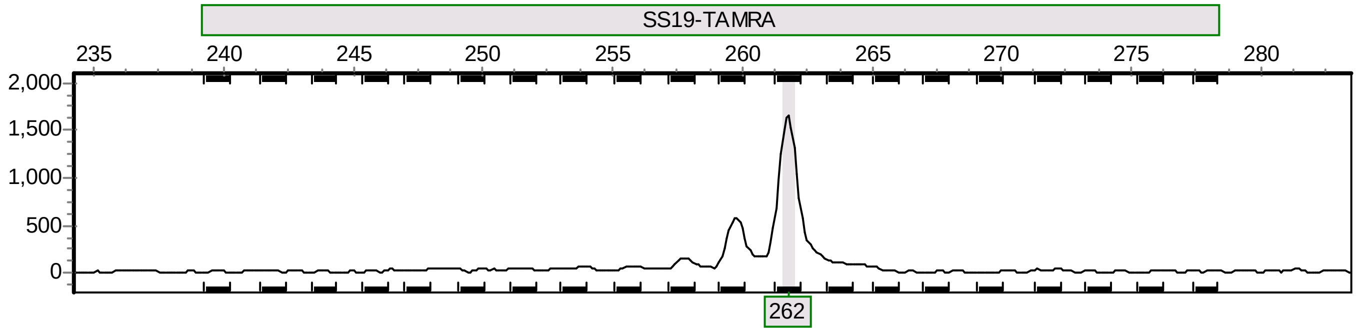


| No | Size | Height | Area | Marker | Allele | Difference | Quality | Score | Allele Comments | Sample Comments |
|---|---|---|---|---|---|---|---|---|---|---|
| 1 | 261.8 | 1645 | 13464 | SS19-TAMRA | 262 | 0.00 | Pass | 174.0 | [<Confirmed>] | |

**Sample 6:** SSS13_SS20_SS11_SS21_SS02_SS19_HBB16_G07.fsa

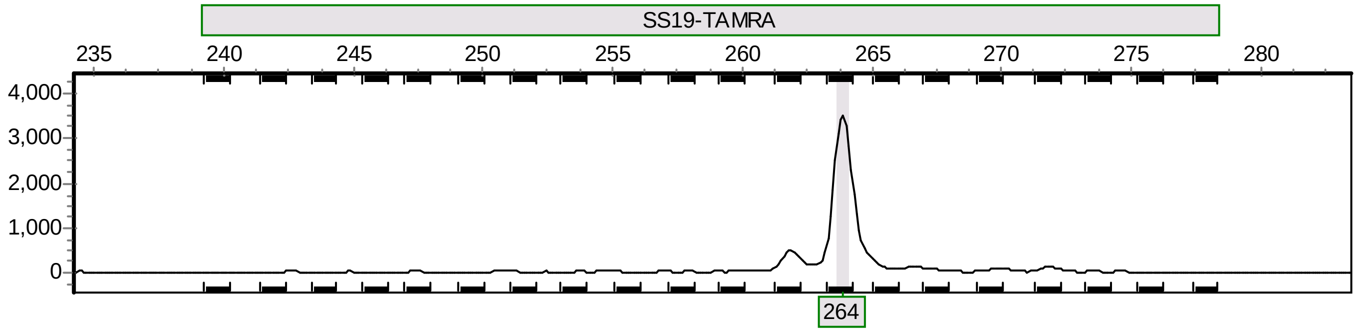


| No | Size | Height | Area | Marker | Allele | Difference | Quality | Score | Allele Comments | Sample Comments |
|---|---|---|---|---|---|---|---|---|---|---|
| 1 | 263.9 | 3496 | 27661 | SS19-TAMRA | 264 | 0.10 | Pass | 500.0 | [<Confirmed>] | |

**Sample 7:** SSS13_SS20_SS11_SS21_SS02_SS19_HBB17_K15.fsa

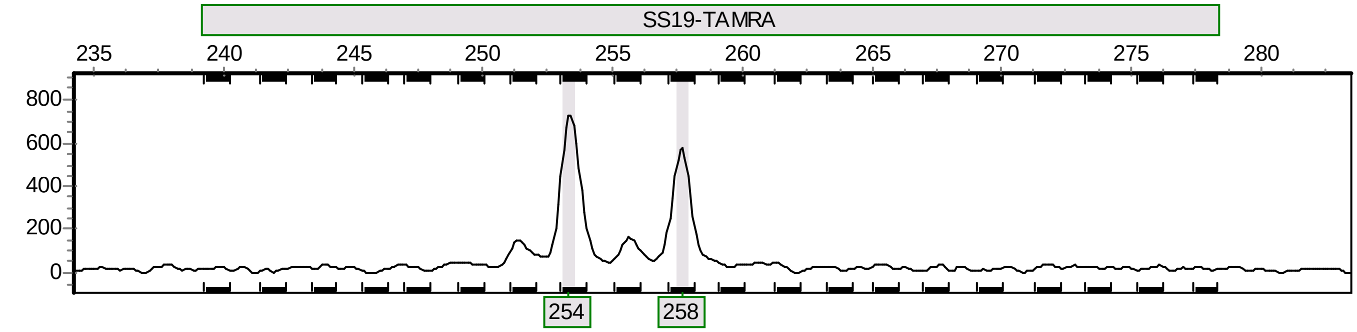


| No | Size | Height | Area | Marker | Allele | Difference | Quality | Score | Allele Comments | Sample Comments |
|---|---|---|---|---|---|---|---|---|---|---|
| 1 | 253.3 | 725 | 6310 | SS19-TAMRA | 254 | 0.20 | Pass | 45.6 | [<Confirmed>] | |
| 2 | 257.7 | 574 | 4760 | SS19-TAMRA | 258 | 0.00 | Pass | 34.1 | [<Confirmed>] | |

**Sample 8:** SSS13_SS20_SS11_SS21_SS02_SS19_HBB18_O05.fsa

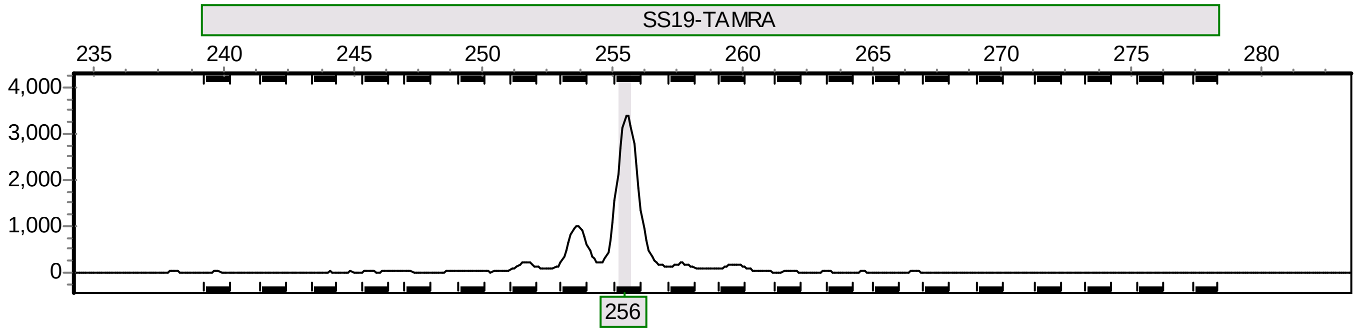


| No | Size | Height | Area | Marker | Allele | Difference | Quality | Score | Allele Comments | Sample Comments |
|---|---|---|---|---|---|---|---|---|---|---|
| 1 | 255.5 | 3375 | 30599 | SS19-TAMRA | 256 | 0.10 | Pass | 422.0 | [<Confirmed>] | |

**Sample 9:** SSS13_SS20_SS11_SS21_SS02_SS19_HBB19_O07.fsa

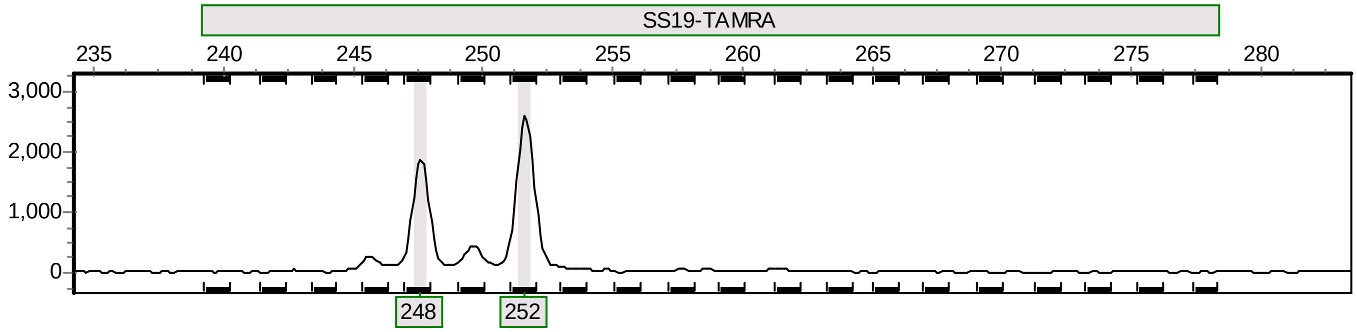


| No | Size | Height | Area | Marker | Allele | Difference | Quality | Score | Allele Comments | Sample Comments |
|---|---|---|---|---|---|---|---|---|---|---|
| 1 | 247.6 | 1882 | 14010 | SS19-TAMRA | 248 | 0.10 | Pass | 267.4 | [<Confirmed>] | |
| 2 | 251.6 | 2595 | 20034 | SS19-TAMRA | 252 | 0.00 | Pass | 395.8 | [<Confirmed>] | |

**SoftGenetics**

GeneMarker V3.0.0

# Allele Report



**Sample 10:** SSS13_SS20_SS11_SS21_SS02_SS19_HBB1_B11.fsa

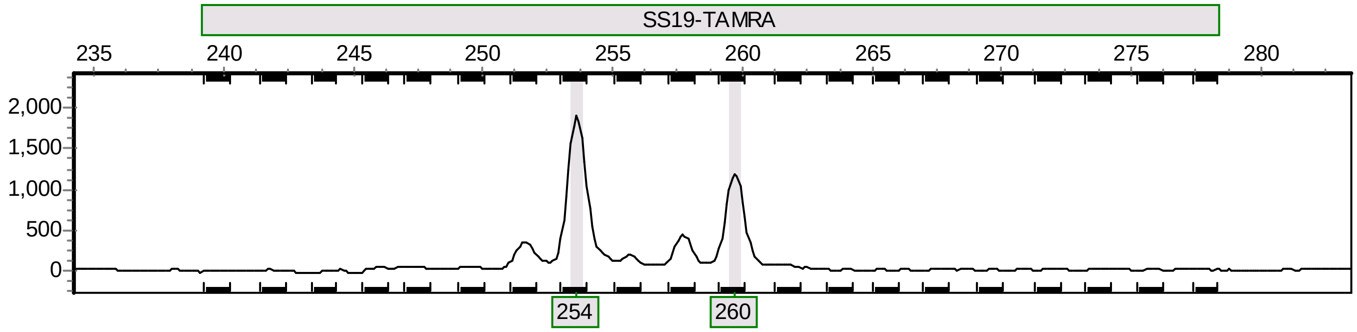


| No | Size | Height | Area | Marker | Allele | Difference | Quality | Score | Allele Comments | Sample Comments |
|---|---|---|---|---|---|---|---|---|---|---|
| 1 | 253.6 | 1899 | 15320 | SS19-TAMRA | 254 | 0.10 | Pass | 232.0 | [<Confirmed>] | |
| 2 | 259.7 | 1198 | 9882 | SS19-TAMRA | 260 | 0.10 | Pass | 110.1 | [<Confirmed>] | |

**Sample 11:** SSS13_SS20_SS11_SS21_SS02_SS19_HBB20_F11.fsa

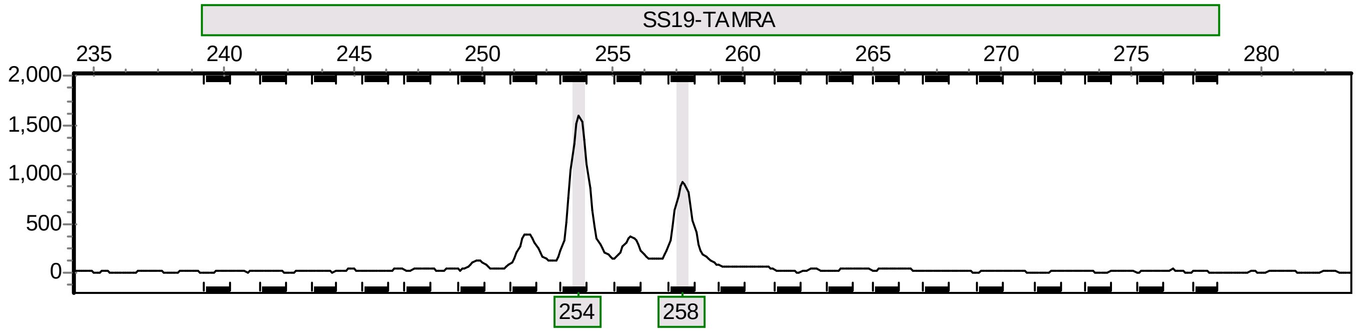


| No | Size | Height | Area | Marker | Allele | Difference | Quality | Score | Allele Comments | Sample Comments |
|---|---|---|---|---|---|---|---|---|---|---|
| 1 | 253.7 | 1590 | 12866 | SS19-TAMRA | 254 | 0.20 | Pass | 180.2 | [<Confirmed>] | |
| 2 | 257.7 | 926 | 7927 | SS19-TAMRA | 258 | 0.00 | Pass | 69.4 | [<Confirmed>] | |

**Sample 12:** SSS13_SS20_SS11_SS21_SS02_SS19_HBB21_J09.fsa

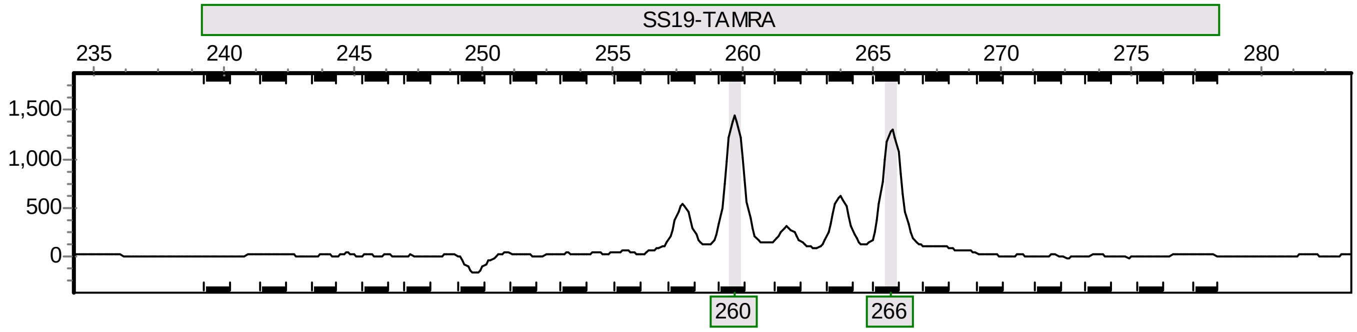


| No | Size | Height | Area | Marker | Allele | Difference | Quality | Score | Allele Comments | Sample Comments |
|---|---|---|---|---|---|---|---|---|---|---|
| 1 | 259.7 | 1434 | 11871 | SS19-TAMRA | 260 | 0.10 | Pass | 145.5 | [<Confirmed>] | |
| 2 | 265.8 | 1307 | 10753 | SS19-TAMRA | 266 | 0.20 | Pass | 121.5 | [<Confirmed>] | |

**Sample 13:** SSS13_SS20_SS11_SS21_SS02_SS19_HBB22_K05.fsa

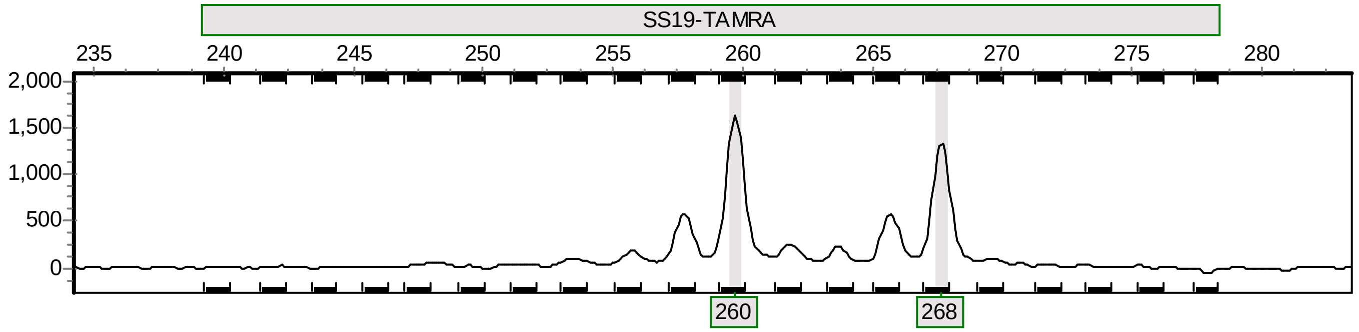


| No | Size | Height | Area | Marker | Allele | Difference | Quality | Score | Allele Comments | Sample Comments |
|---|---|---|---|---|---|---|---|---|---|---|
| 1 | 259.7 | 1618 | 13161 | SS19-TAMRA | 260 | 0.10 | Pass | 183.1 | [<Confirmed>] | |
| 2 | 267.7 | 1322 | 10496 | SS19-TAMRA | 268 | 0.20 | Pass | 133.9 | [<Confirmed>] | |

**Sample 14:** SSS13_SS20_SS11_SS21_SS02_SS19_HBB23_B09.fsa

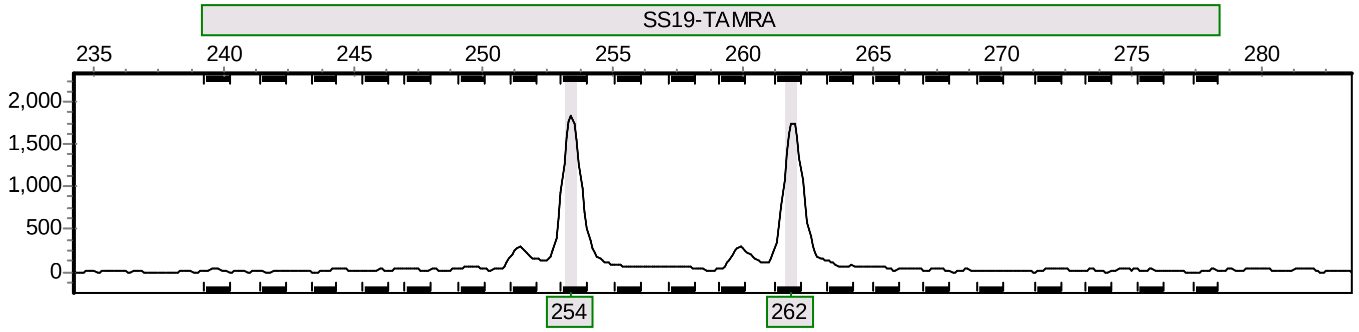


| No | Size | Height | Area | Marker | Allele | Difference | Quality | Score | Allele Comments | Sample Comments |
|---|---|---|---|---|---|---|---|---|---|---|
| 1 | 253.4 | 1823 | 15146 | SS19-TAMRA | 254 | 0.10 | Pass | 207.3 | [<Confirmed>] | |
| 2 | 261.9 | 1742 | 14800 | SS19-TAMRA | 262 | 0.10 | Pass | 192.9 | [<Confirmed>] | |

**Sample 15:** SSS13_SS20_SS11_SS21_SS02_SS19_HBB25-1_F09.fsa

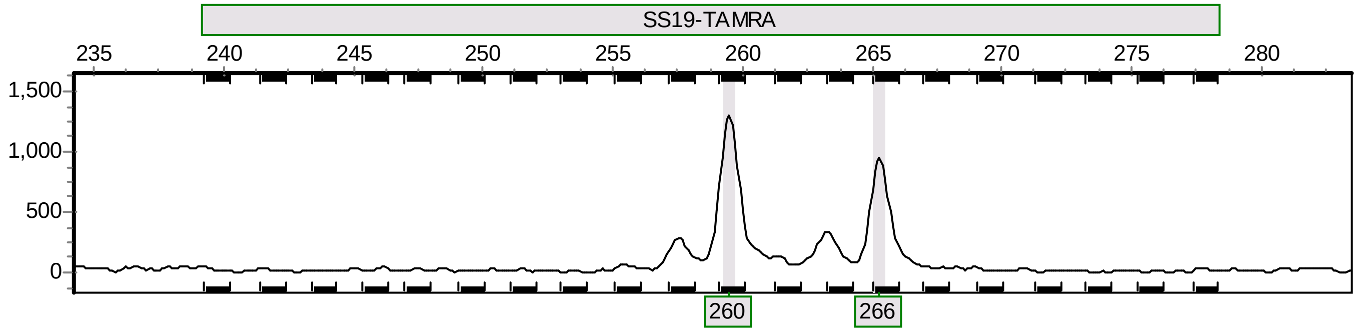


| No | Size | Height | Area | Marker | Allele | Difference | Quality | Score | Allele Comments | Sample Comments |
|---|---|---|---|---|---|---|---|---|---|---|
| 1 | 259.5 | 1297 | 10984 | SS19-TAMRA | 260 | 0.10 | Pass | 118.3 | [<Confirmed>] | |
| 2 | 265.3 | 945 | 7870 | SS19-TAMRA | 266 | 0.30 | Pass | 76.1 | [<Confirmed>] | |

**SoftGenetics**

# Allele Report

GeneMarker V3.0.0

**Sample 16:** SSS13_SS20_SS11_SS21_SS02_SS19_HBB25-2_N11.fsa Run date and time: 10/09/2021 - 22:22:10 -> 10/09/2021 - 22:49:12

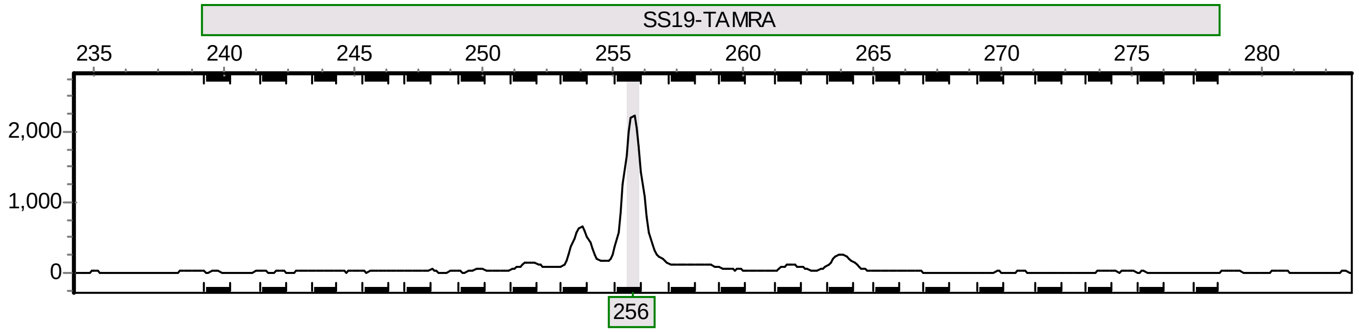


| No | Size | Height | Area | Marker | Allele | Difference | Quality | Score | Allele Comments | Sample Comments |
|---|---|---|---|---|---|---|---|---|---|---|
| 1 | 255.8 | 2205 | 17775 | SS19-TAMRA | 256 | 0.20 | Pass | 268.8 | [<Confirmed>] | |

**Sample 17:** SSS13_SS20_SS11_SS21_SS02_SS19_HBB26_D07.fsa

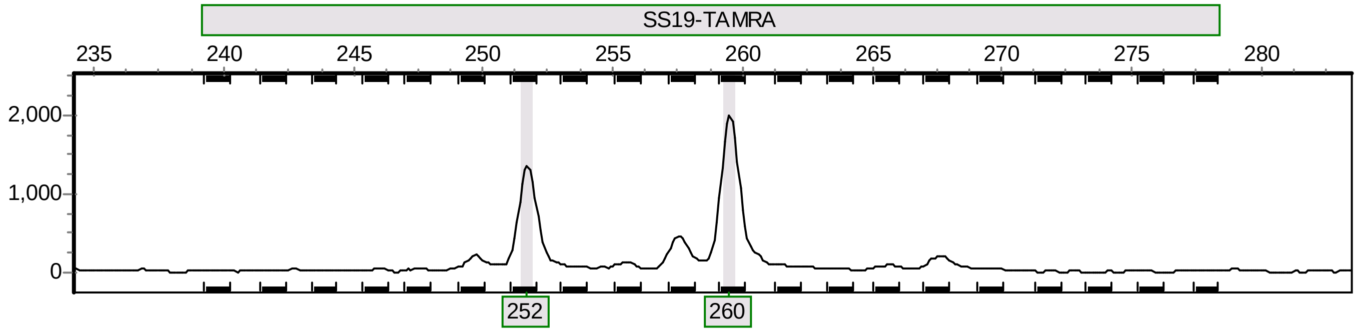


| No | Size | Height | Area | Marker | Allele | Difference | Quality | Score | Allele Comments | Sample Comments |
|---|---|---|---|---|---|---|---|---|---|---|
| 1 | 251.7 | 1354 | 10913 | SS19-TAMRA | 252 | 0.10 | Pass | 145.3 | [<Confirmed>] | |
| 2 | 259.5 | 1979 | 16035 | SS19-TAMRA | 260 | 0.10 | Pass | 241.7 | [<Confirmed>] | |

**Sample 18:** SSS13_SS20_SS11_SS21_SS02_SS19_HBB27_H05.fsa

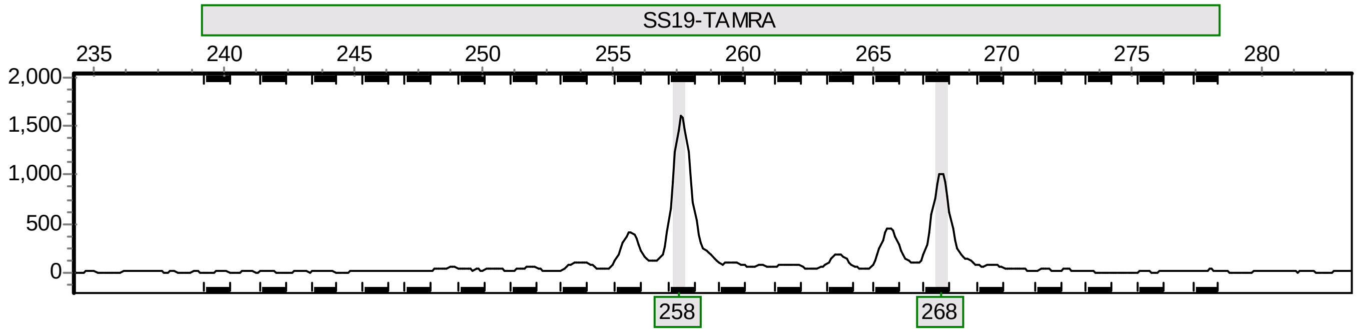


| No | Size | Height | Area | Marker | Allele | Difference | Quality | Score | Allele Comments | Sample Comments |
|---|---|---|---|---|---|---|---|---|---|---|
| 1 | 257.6 | 1597 | 13270 | SS19-TAMRA | 258 | 0.10 | Pass | 181.8 | [<Confirmed>] | |
| 2 | 267.7 | 1005 | 8107 | SS19-TAMRA | 268 | 0.20 | Pass | 87.4 | [<Confirmed>] | |

# Allele Report

**Sample 19:** SSS13_SS20_SS11_SS21_SS02_SS19_HBB28_L11.fsa

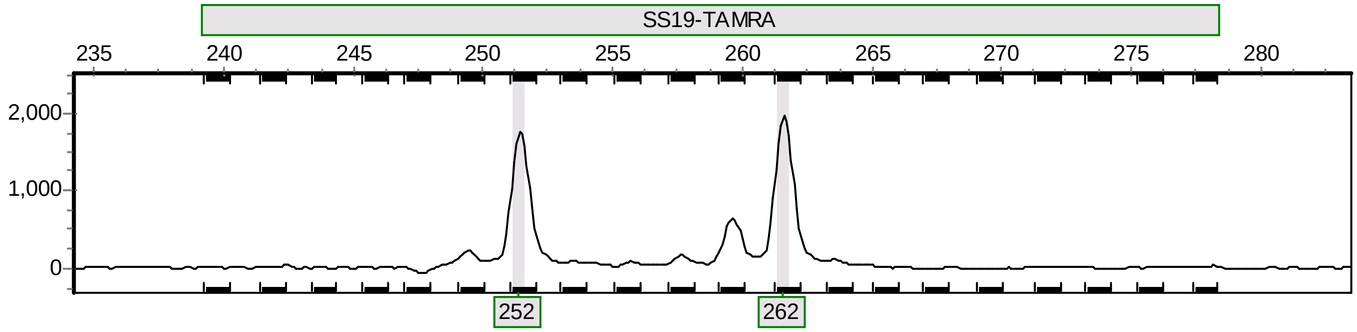


| No | Size | Height | Area | Marker | Allele | Difference | Quality | Score | Allele Comments | Sample Comments |
|---|---|---|---|---|---|---|---|---|---|---|
| 1 | 251.4 | 1758 | 14019 | SS19-TAMRA | 252 | 0.20 | Pass | 215.8 | [<Confirmed>] | |
| 2 | 261.6 | 1958 | 15781 | SS19-TAMRA | 262 | 0.20 | Pass | 243.0 | [<Confirmed>] | |

**Sample 20:** SSS13_SS20_SS11_SS21_SS02_SS19_HBB2_C07.fsa

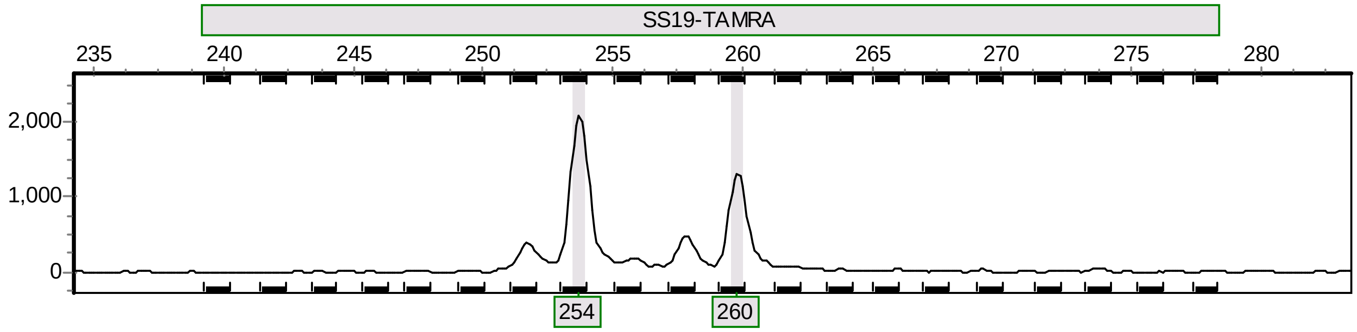


| No | Size | Height | Area | Marker | Allele | Difference | Quality | Score | Allele Comments | Sample Comments |
|---|---|---|---|---|---|---|---|---|---|---|
| 1 | 253.7 | 2060 | 16785 | SS19-TAMRA | 254 | 0.20 | Pass | 259.9 | [<Confirmed>] | |
| 2 | 259.8 | 1296 | 10555 | SS19-TAMRA | 260 | 0.20 | Pass | 126.1 | [<Confirmed>] | |

**Sample 21:** SSS13_SS20_SS11_SS21_SS02_SS19_HBB30_L13.fsa

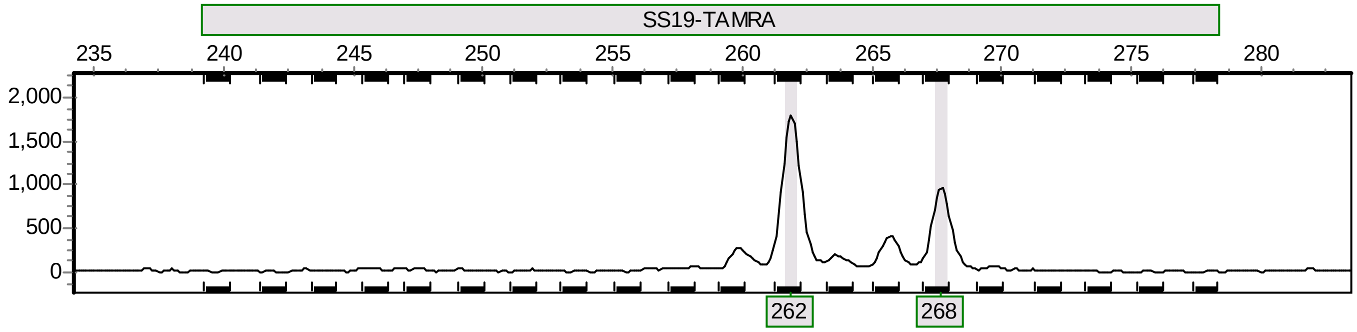


| No | Size | Height | Area | Marker | Allele | Difference | Quality | Score | Allele Comments | Sample Comments |
|---|---|---|---|---|---|---|---|---|---|---|
| 1 | 261.9 | 1786 | 14644 | SS19-TAMRA | 262 | 0.10 | Pass | 206.0 | [<Confirmed>] | |
| 2 | 267.7 | 950 | 7899 | SS19-TAMRA | 268 | 0.20 | Pass | 76.9 | [<Confirmed>] | |

# SoftGenetics

# Allele Report

GeneMarker V3.0.0



**Sample 22:** SSS13_SS20_SS11_SS21_SS02_SS19_HBB31_P07.fsa

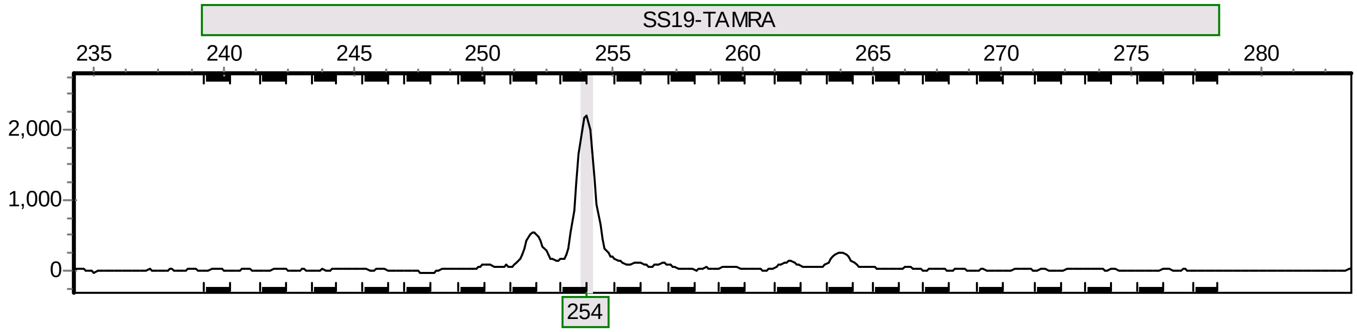


| No | Size | Height | Area | Marker | Allele | Difference | Quality | Score | Allele Comments | Sample Comments |
|---|---|---|---|---|---|---|---|---|---|---|
| 1 | 254.0 | 2187 | 17174 | SS19-TAMRA | 254 | 0.50 | Pass | 288.6 | [<Confirmed>] | |

**Sample 23:** SSS13_SS20_SS11_SS21_SS02_SS19_HBB32_B15.fsa

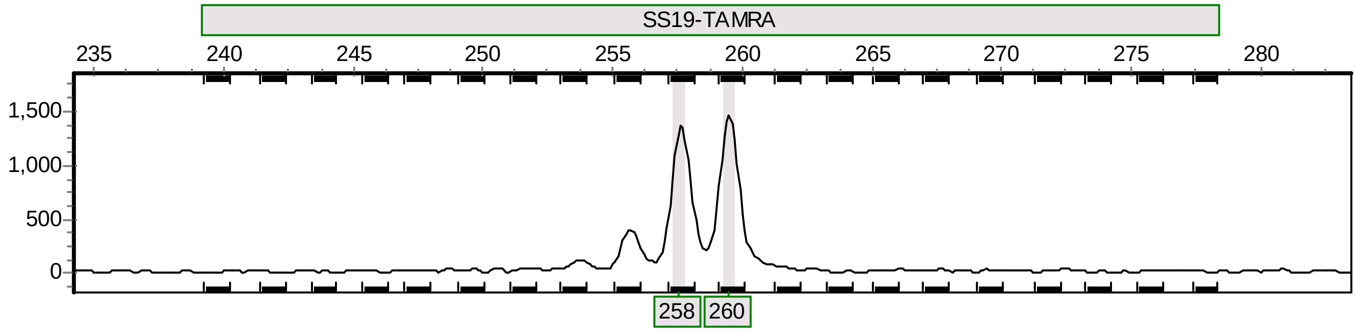


| No | Size | Height | Area | Marker | Allele | Difference | Quality | Score | Allele Comments | Sample Comments |
|---|---|---|---|---|---|---|---|---|---|---|
| 1 | 257.6 | 1376 | 11645 | SS19-TAMRA | 258 | 0.10 | Pass | 132.6 | [<Confirmed>] | |
| 2 | 259.5 | 1462 | 12621 | SS19-TAMRA | 260 | 0.10 | Pass | 141.4 | [<Confirmed>] | |

**Sample 24:** SSS13_SS20_SS11_SS21_SS02_SS19_HBB33_N13.fsa

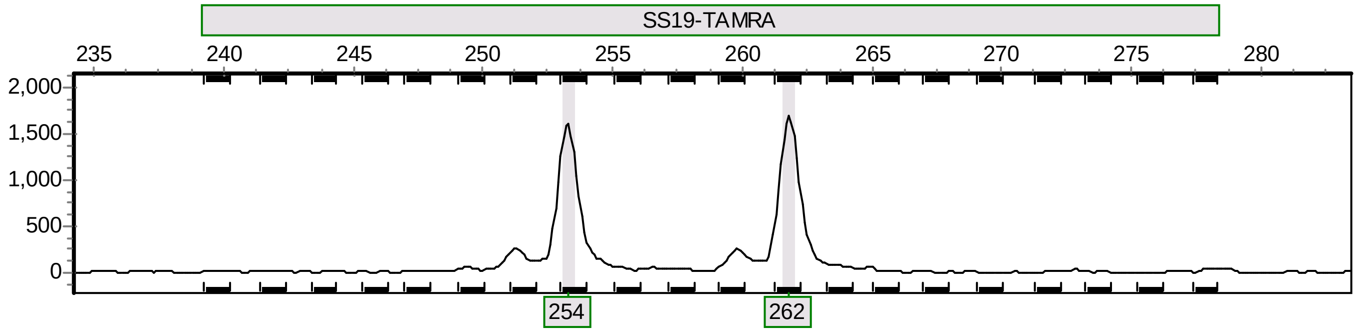


| No | Size | Height | Area | Marker | Allele | Difference | Quality | Score | Allele Comments | Sample Comments |
|---|---|---|---|---|---|---|---|---|---|---|
| 1 | 253.3 | 1597 | 13380 | SS19-TAMRA | 254 | 0.20 | Pass | 150.5 | [<Confirmed>] | |
| 2 | 261.8 | 1681 | 14405 | SS19-TAMRA | 262 | 0.00 | Pass | 166.8 | [<Confirmed>] | |

# Allele Report

**Sample 25:** SSS13_SS20_SS11_SS21_SS02_SS19_HBB34_P13.fsa 2

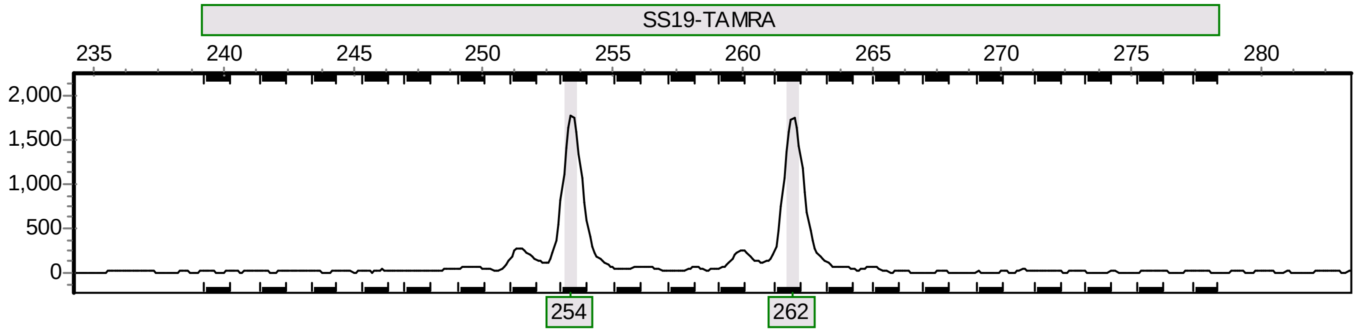


| No | Size | Height | Area | Marker | Allele | Difference | Quality | Score | Allele Comments | Sample Comments |
|---|---|---|---|---|---|---|---|---|---|---|
| 1 | 253.4 | 1764 | 14886 | SS19-TAMRA | 254 | 0.10 | Pass | 191.0 | [<Confirmed>] | |
| 2 | 262.0 | 1751 | 15126 | SS19-TAMRA | 262 | 0.20 | Pass | 171.2 | [<Confirmed>] | |

**Sample 26:** SSS13_SS20_SS11_SS21_SS02_SS19_HBB35_C15.fsa

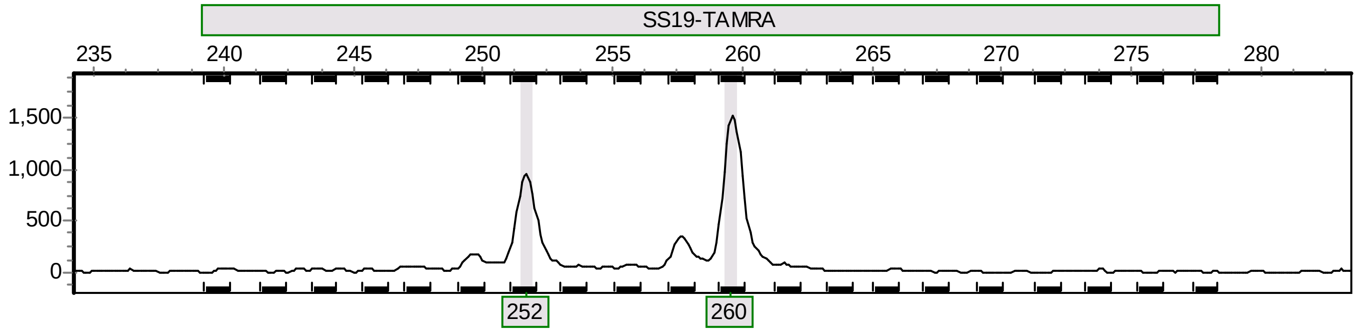


| No | Size | Height | Area | Marker | Allele | Difference | Quality | Score | Allele Comments | Sample Comments |
|---|---|---|---|---|---|---|---|---|---|---|
| 1 | 251.7 | 944 | 7970 | SS19-TAMRA | 252 | 0.10 | Pass | 66.0 | [<Confirmed>] | |
| 2 | 259.6 | 1511 | 13151 | SS19-TAMRA | 260 | 0.00 | Pass | 146.6 | [<Confirmed>] | |

**Sample 27:** SSS13_SS20_SS11_SS21_SS02_SS19_HBB36_G05.fsa

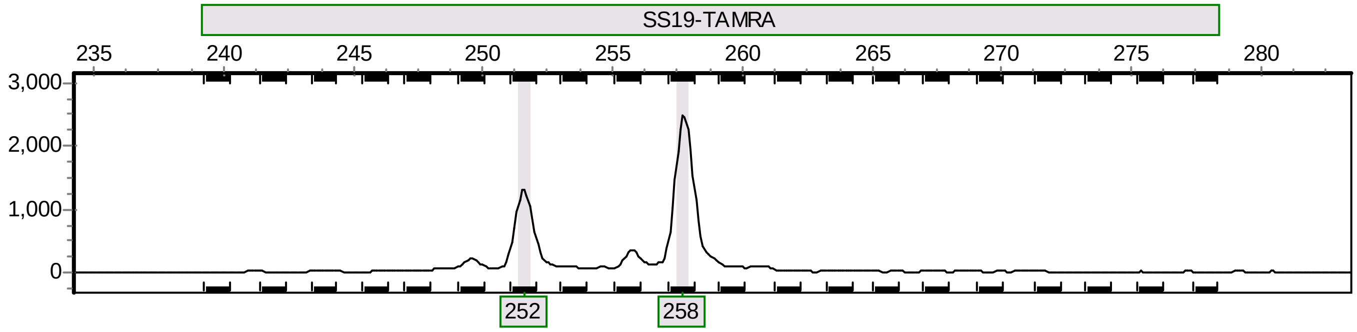


| No | Size | Height | Area | Marker | Allele | Difference | Quality | Score | Allele Comments | Sample Comments |
|---|---|---|---|---|---|---|---|---|---|---|
| 1 | 251.6 | 1306 | 10282 | SS19-TAMRA | 252 | 0.00 | Pass | 130.8 | [<Confirmed>] | |
| 2 | 257.7 | 2479 | 20580 | SS19-TAMRA | 258 | 0.00 | Pass | 331.9 | [<Confirmed>] | |

# Allele Report



**Sample 28:** SSS13_SS20_SS11_SS21_SS02_SS19_HBB37_N09.fsa

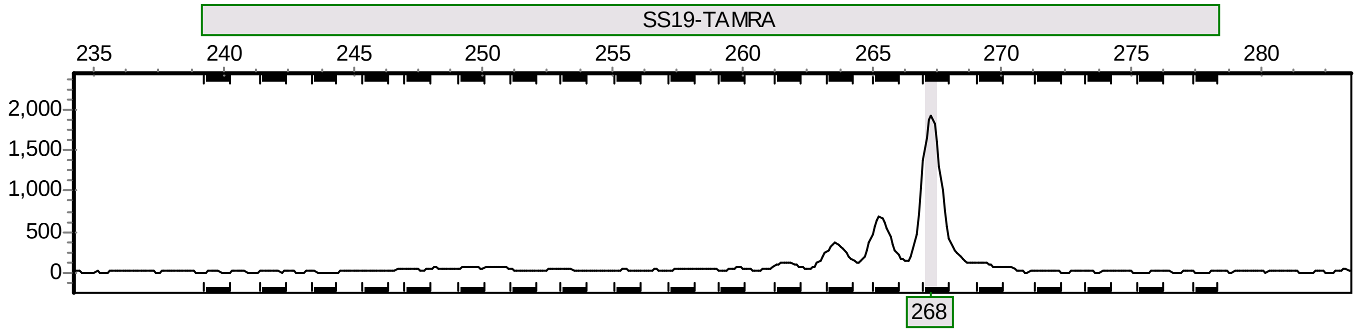


| No | Size | Height | Area | Marker | Allele | Difference | Quality | Score | Allele Comments | Sample Comments |
|---|---|---|---|---|---|---|---|---|---|---|
| 1 | 267.3 | 1920 | 16068 | SS19-TAMRA | 268 | 0.20 | Pass | 218.1 | [<Confirmed>] | |

**Sample 29:** SSS13_SS20_SS11_SS21_SS02_SS19_HBB38_M07.fsa

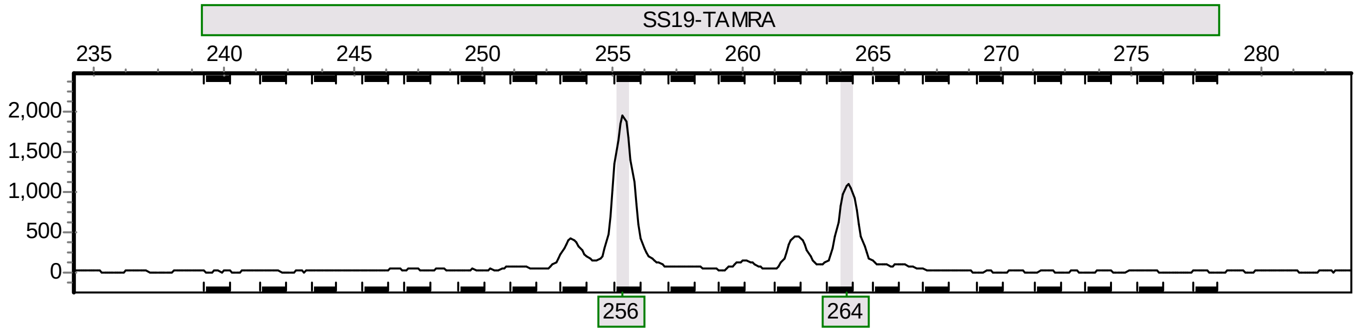


| No | Size | Height | Area | Marker | Allele | Difference | Quality | Score | Allele Comments | Sample Comments |
|---|---|---|---|---|---|---|---|---|---|---|
| 1 | 255.4 | 1944 | 16420 | SS19-TAMRA | 256 | 0.20 | Pass | 212.7 | [<Confirmed>] | |
| 2 | 264.1 | 1107 | 9559 | SS19-TAMRA | 264 | 0.30 | Pass | 88.3 | [<Confirmed>] | |

**Sample 30:** SSS13_SS20_SS11_SS21_SS02_SS19_HBB39_F05.fsa

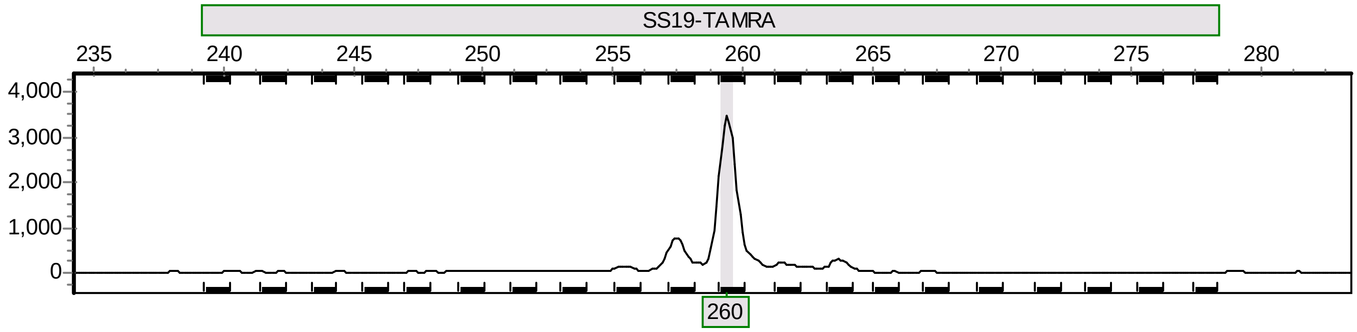


| No | Size | Height | Area | Marker | Allele | Difference | Quality | Score | Allele Comments | Sample Comments |
|---|---|---|---|---|---|---|---|---|---|---|
| 1 | 259.4 | 3461 | 27048 | SS19-TAMRA | 260 | 0.20 | Pass | 500.0 | [<Confirmed>] | |

# Allele Report

**Sample 31:** SSS13_SS20_SS11_SS21_SS02_SS19_HBB40_D15.fsa

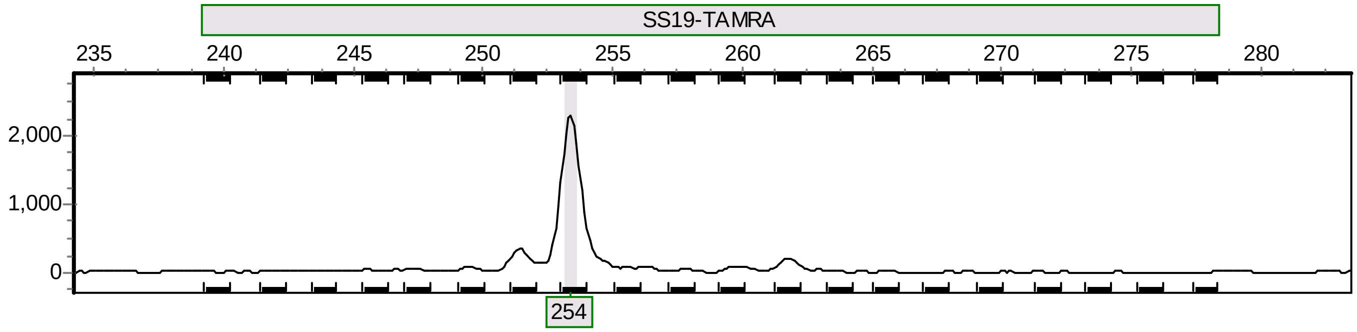


| No | Size | Height | Area | Marker | Allele | Difference | Quality | Score | Allele Comments | Sample Comments |
|---|---|---|---|---|---|---|---|---|---|---|
| 1 | 253.4 | 2270 | 19490 | SS19-TAMRA | 254 | 0.10 | Pass | 259.4 | [<Confirmed>] | |

**Sample 32:** SSS13_SS20_SS11_SS21_SS02_SS19_HBB41_L07.fsa

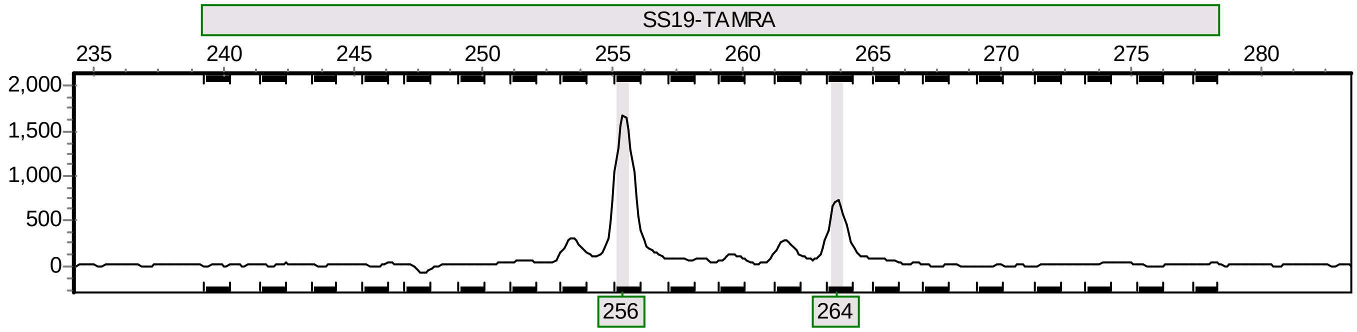


| No | Size | Height | Area | Marker | Allele | Difference | Quality | Score | Allele Comments | Sample Comments |
|---|---|---|---|---|---|---|---|---|---|---|
| 1 | 255.4 | 1664 | 14168 | SS19-TAMRA | 256 | 0.20 | Pass | 176.5 | [<Confirmed>] | |
| 2 | 263.7 | 722 | 5882 | SS19-TAMRA | 264 | 0.10 | Pass | 48.7 | [<Confirmed>] | |

**Sample 33:** SSS13_SS20_SS11_SS21_SS02_SS19_HBB42_M05.fsa

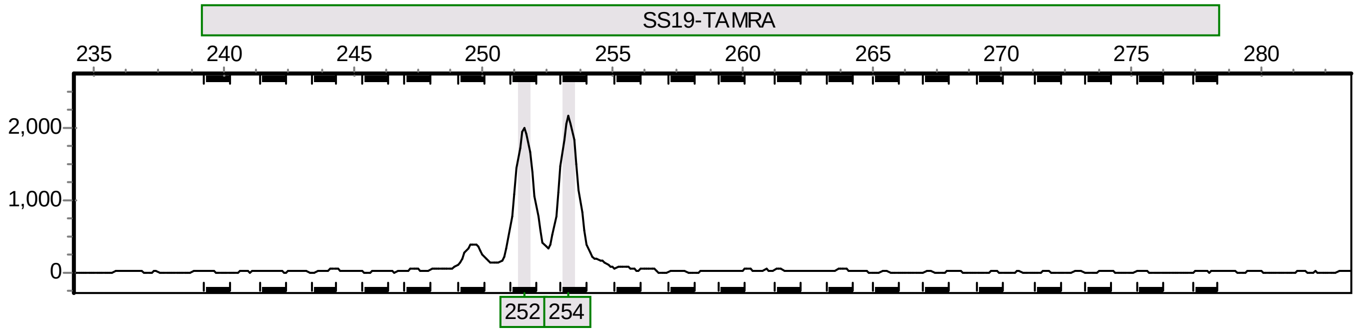


| No | Size | Height | Area | Marker | Allele | Difference | Quality | Score | Allele Comments | Sample Comments |
|---|---|---|---|---|---|---|---|---|---|---|
| 1 | 251.6 | 2010 | 16926 | SS19-TAMRA | 252 | 0.00 | Pass | 230.4 | [<Confirmed>] | |
| 2 | 253.3 | 2169 | 18096 | SS19-TAMRA | 254 | 0.20 | Pass | 273.6 | [<Confirmed>] | |

**Sample 34:** SSS13_SS20_SS11_SS21_SS02_SS19_HBB43_D09.fsa

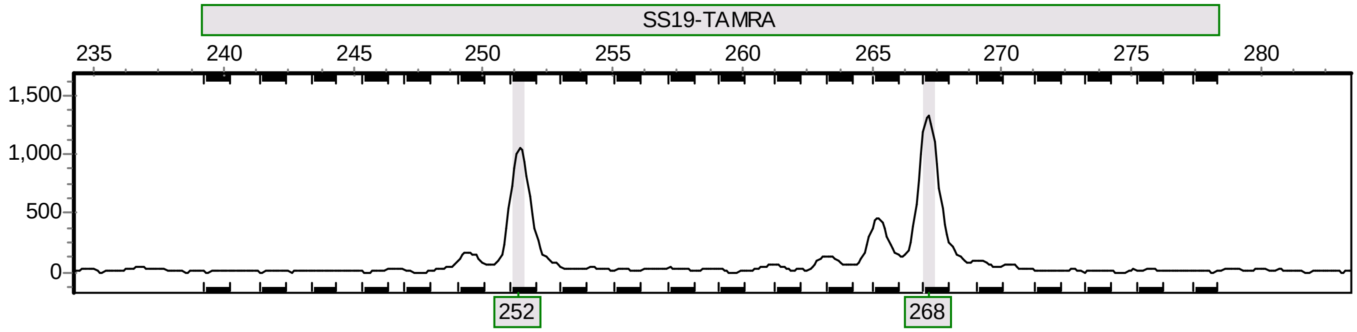


| No | Size | Height | Area | Marker | Allele | Difference | Quality | Score | Allele Comments | Sample Comments |
|---|---|---|---|---|---|---|---|---|---|---|
| 1 | 251.4 | 1042 | 9328 | SS19-TAMRA | 252 | 0.20 | Pass | 75.3 | [<Confirmed>] | |
| 2 | 267.2 | 1317 | 11521 | SS19-TAMRA | 268 | 0.30 | Pass | 111.1 | [<Confirmed>] | |

**Sample 35:** SSS13_SS20_SS11_SS21_SS02_SS19_HBB44_H11.fsa

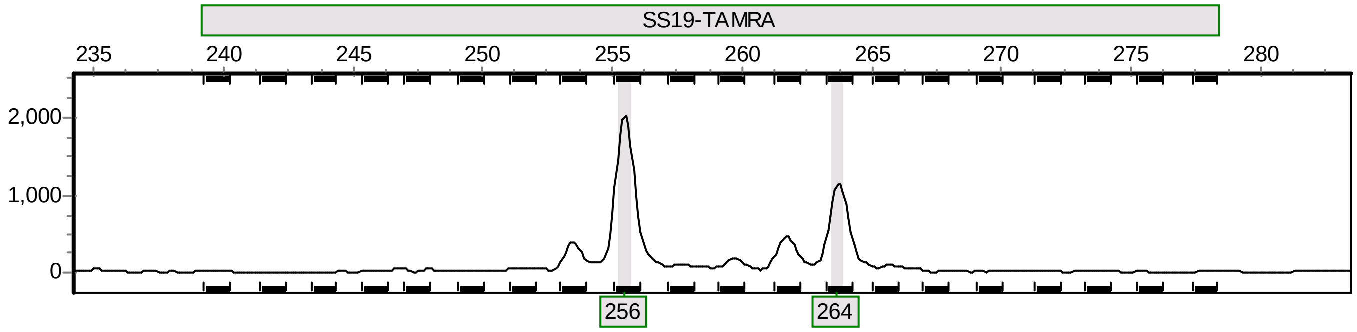


| No | Size | Height | Area | Marker | Allele | Difference | Quality | Score | Allele Comments | Sample Comments |
|---|---|---|---|---|---|---|---|---|---|---|
| 1 | 255.5 | 2028 | 16882 | SS19-TAMRA | 256 | 0.10 | Pass | 245.0 | [<Confirmed>] | |
| 2 | 263.7 | 1155 | 9960 | SS19-TAMRA | 264 | 0.10 | Pass | 99.4 | [<Confirmed>] | |

**Sample 36:** SSS13_SS20_SS11_SS21_SS02_SS19_HBB45_F13.fsa

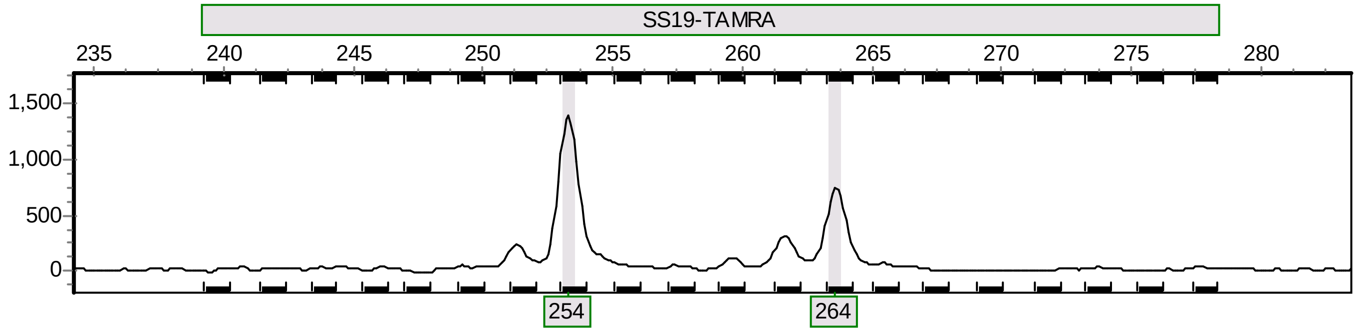


| No | Size | Height | Area | Marker | Allele | Difference | Quality | Score | Allele Comments | Sample Comments |
|---|---|---|---|---|---|---|---|---|---|---|
| 1 | 253.3 | 1387 | 12079 | SS19-TAMRA | 254 | 0.20 | Pass | 117.4 | [<Confirmed>] | |
| 2 | 263.6 | 742 | 6737 | SS19-TAMRA | 264 | 0.20 | Pass | 44.0 | [<Confirmed>] | |

# Allele Report



**Sample 37:** SSS13_SS20_SS11_SS21_SS02_SS19_HBB46_F07.fsa

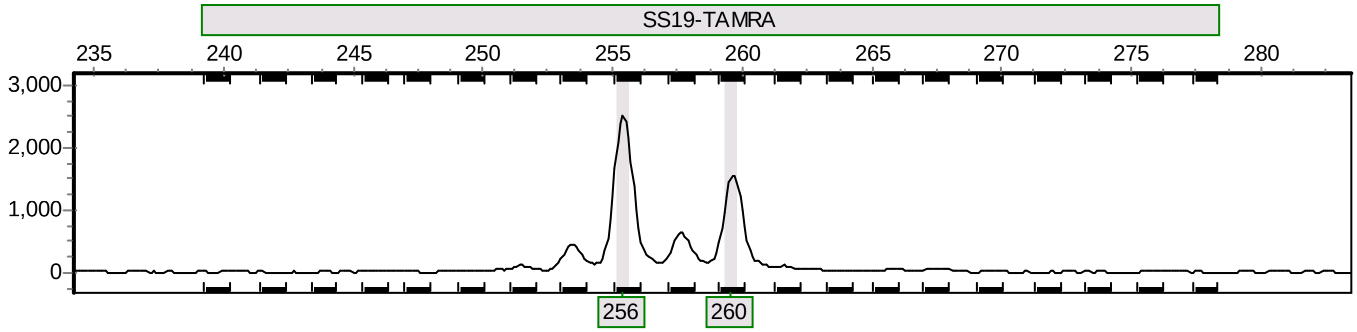


| No | Size | Height | Area | Marker | Allele | Difference | Quality | Score | Allele Comments | Sample Comments |
|---|---|---|---|---|---|---|---|---|---|---|
| 1 | 255.4 | 2515 | 20828 | SS19-TAMRA | 256 | 0.20 | Pass | 337.0 | [<Confirmed>] | |
| 2 | 259.6 | 1566 | 13213 | SS19-TAMRA | 260 | 0.00 | Pass | 156.1 | [<Confirmed>] | |

**Sample 38:** SSS13_SS20_SS11_SS21_SS02_SS19_HBB47_A05.fsa

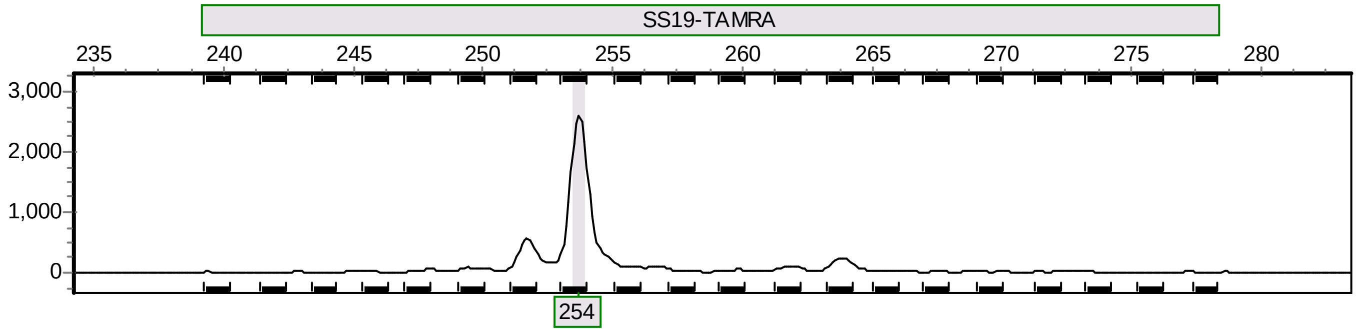


| No | Size | Height | Area | Marker | Allele | Difference | Quality | Score | Allele Comments | Sample Comments |
|---|---|---|---|---|---|---|---|---|---|---|
| 1 | 253.7 | 2598 | 20548 | SS19-TAMRA | 254 | 0.20 | Pass | 395.6 | [<Confirmed>] | |

**Sample 39:** SSS13_SS20_SS11_SS21_SS02_SS19_HBB48_B13.fsa

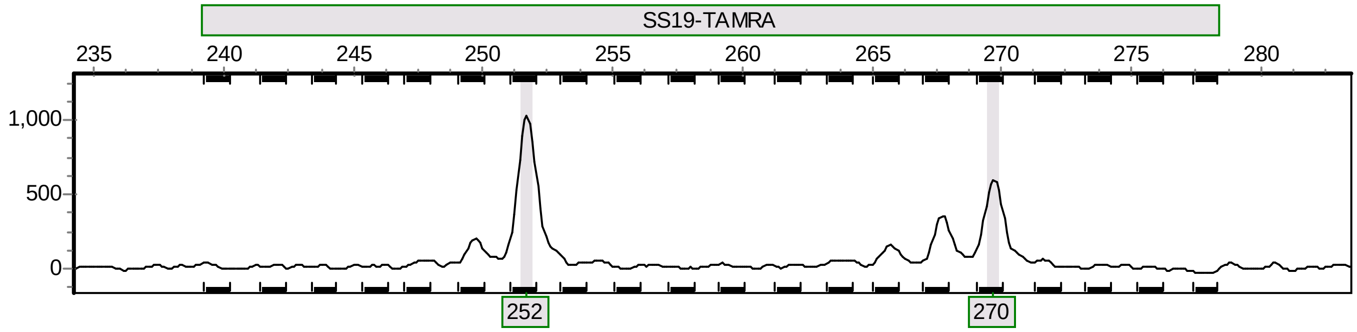


| No | Size | Height | Area | Marker | Allele | Difference | Quality | Score | Allele Comments | Sample Comments |
|---|---|---|---|---|---|---|---|---|---|---|
| 1 | 251.7 | 1022 | 8534 | SS19-TAMRA | 252 | 0.10 | Pass | 84.3 | [<Confirmed>] | |
| 2 | 269.7 | 600 | 5179 | SS19-TAMRA | 270 | 0.10 | Pass | 33.1 | [<Confirmed>] | |

# Allele Report

**Sample 40:** SSS13_SS20_SS11_SS21_SS02_SS19_HBB49_L05.fsa

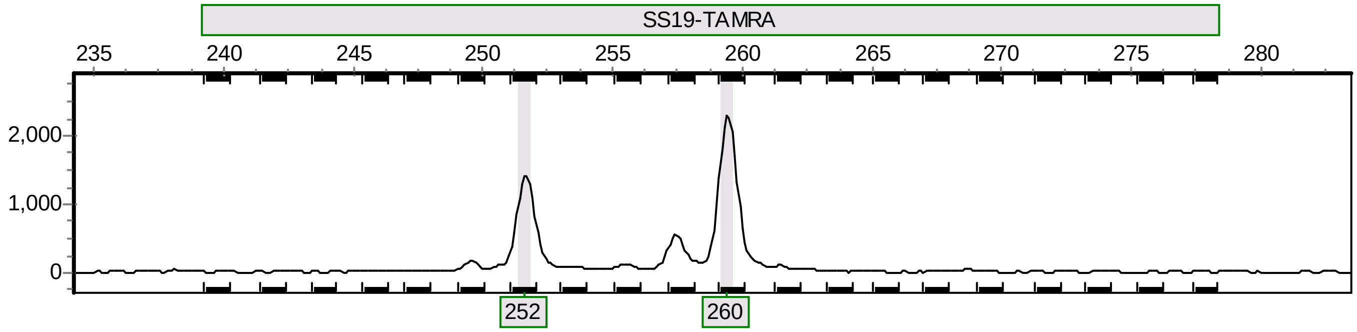


| No | Size | Height | Area | Marker | Allele | Difference | Quality | Score | Allele Comments | Sample Comments |
|---|---|---|---|---|---|---|---|---|---|---|
| 1 | 251.6 | 1418 | 11390 | SS19-TAMRA | 252 | 0.00 | Pass | 155.4 | [<Confirmed>] | |
| 2 | 259.4 | 2283 | 18350 | SS19-TAMRA | 260 | 0.20 | Pass | 308.3 | [<Confirmed>] | |

**Sample 41:** SSS13_SS20_SS11_SS21_SS02_SS19_HBB4_E07.fsa

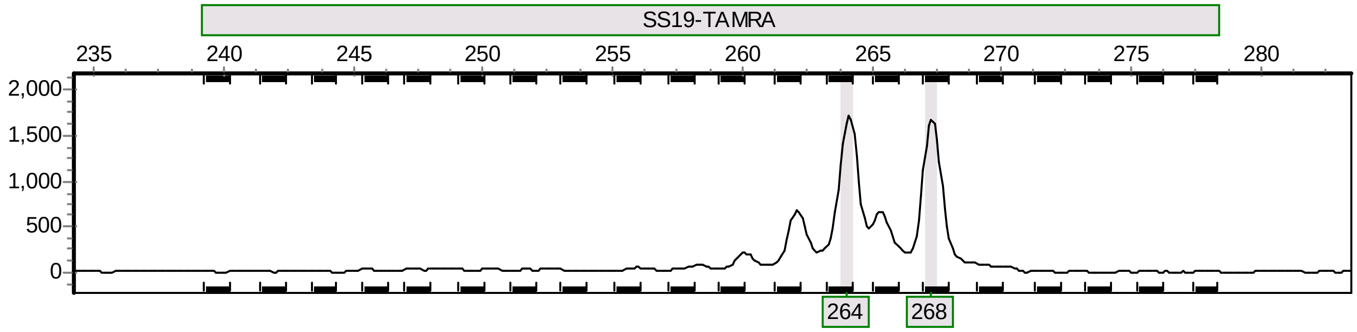


| No | Size | Height | Area | Marker | Allele | Difference | Quality | Score | Allele Comments | Sample Comments |
|---|---|---|---|---|---|---|---|---|---|---|
| 1 | 264.1 | 1712 | 14708 | SS19-TAMRA | 264 | 0.30 | Pass | 173.8 | [<Confirmed>] | |
| 2 | 267.3 | 1682 | 14025 | SS19-TAMRA | 268 | 0.20 | Pass | 181.4 | [<Confirmed>] | |

**Sample 42:** SSS13_SS20_SS11_SS21_SS02_SS19_HBB5_H13.fsa

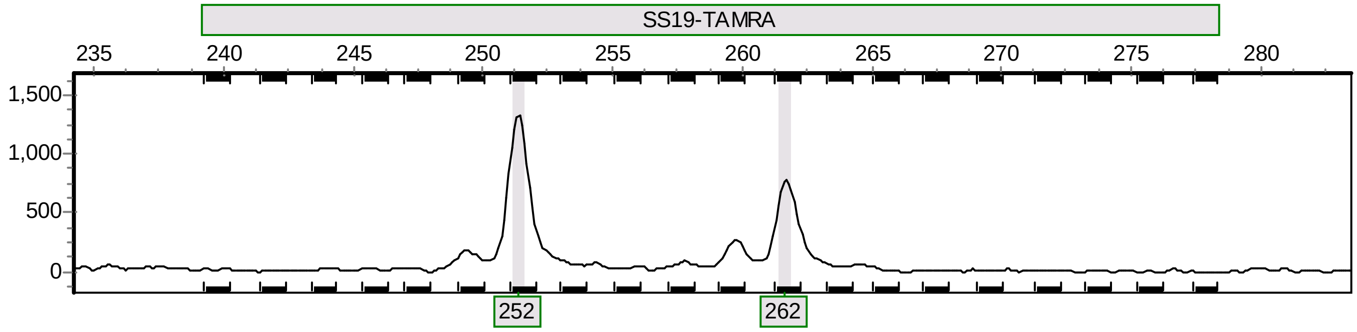


| No | Size | Height | Area | Marker | Allele | Difference | Quality | Score | Allele Comments | Sample Comments |
|---|---|---|---|---|---|---|---|---|---|---|
| 1 | 251.4 | 1319 | 11913 | SS19-TAMRA | 252 | 0.20 | Pass | 102.7 | [<Confirmed>] | |
| 2 | 261.7 | 779 | 7257 | SS19-TAMRA | 262 | 0.10 | Pass | 43.1 | [<Confirmed>] | |

# Allele Report



**Sample 43:** SSS13_SS20_SS11_SS21_SS02_SS19_HBB6_C05.fsa

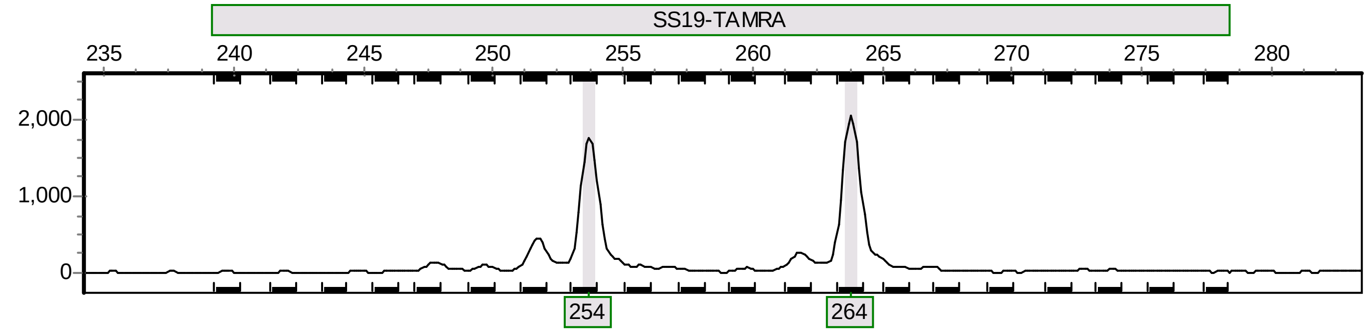


| No | Size | Height | Area | Marker | Allele | Difference | Quality | Score | Allele Comments | Sample Comments |
|---|---|---|---|---|---|---|---|---|---|---|
| 1 | 253.7 | 1746 | 13821 | SS19-TAMRA | 254 | 0.20 | Pass | 218.1 | [<Confirmed>] | |
| 2 | 263.8 | 2029 | 16177 | SS19-TAMRA | 264 | 0.00 | Pass | 268.8 | [<Confirmed>] | |

**Sample 44:** SSS13_SS20_SS11_SS21_SS02_SS19_HBB7_N05.fsa

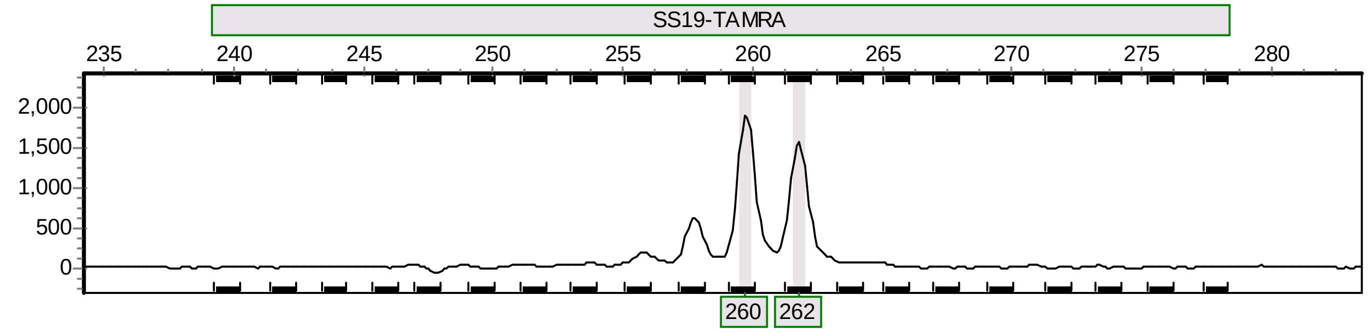


| No | Size | Height | Area | Marker | Allele | Difference | Quality | Score | Allele Comments | Sample Comments |
|---|---|---|---|---|---|---|---|---|---|---|
| 1 | 259.7 | 1898 | 15425 | SS19-TAMRA | 260 | 0.10 | Pass | 241.7 | [<Confirmed>] | |
| 2 | 261.8 | 1572 | 13004 | SS19-TAMRA | 262 | 0.00 | Pass | 173.1 | [<Confirmed>] | |

**Sample 45:** SSS13_SS20_SS11_SS21_SS02_SS19_HBB8_P11.fsa

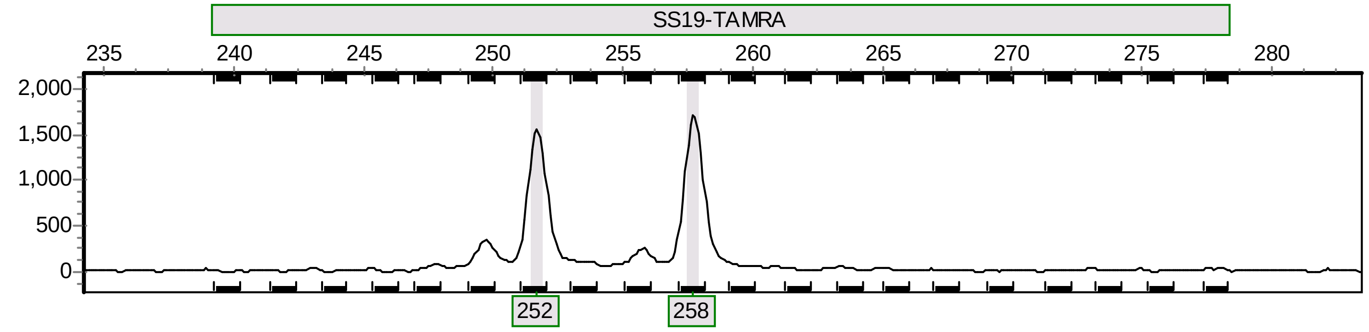


| No | Size | Height | Area | Marker | Allele | Difference | Quality | Score | Allele Comments | Sample Comments |
|---|---|---|---|---|---|---|---|---|---|---|
| 1 | 251.7 | 1551 | 12985 | SS19-TAMRA | 252 | 0.10 | Pass | 160.8 | [<Confirmed>] | |
| 2 | 257.7 | 1701 | 14434 | SS19-TAMRA | 258 | 0.00 | Pass | 183.8 | [<Confirmed>] | |

**Sample 46:** SSS13_SS20_SS11_SS21_SS02_SS19_HBB9_I05.fsa

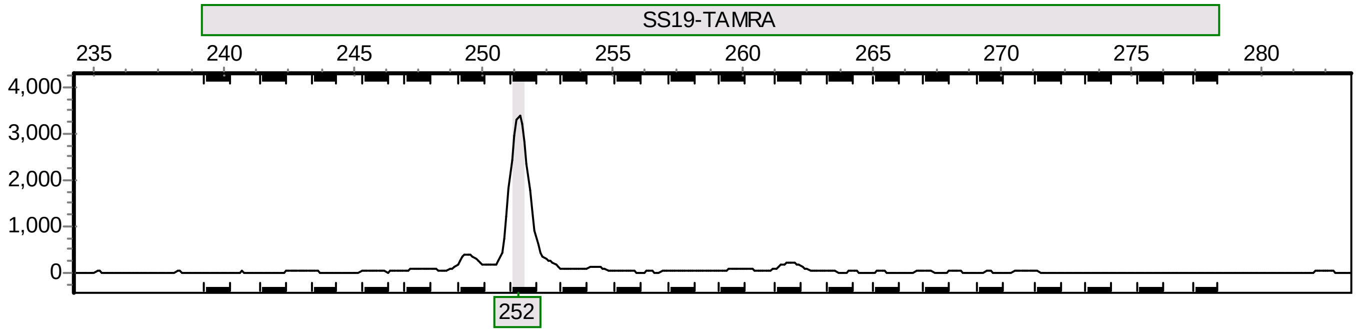


| No | Size | Height | Area | Marker | Allele | Difference | Quality | Score | Allele Comments | Sample Comments |
|---|---|---|---|---|---|---|---|---|---|---|
| 1 | 251.4 | 3377 | 28303 | SS19-TAMRA | 252 | 0.20 | Pass | 479.6 | [<Confirmed>] | |

**Sample 47:** SSS13_SS20_SS11_SS21_SS02_SS19_HBN10_G11.fsa

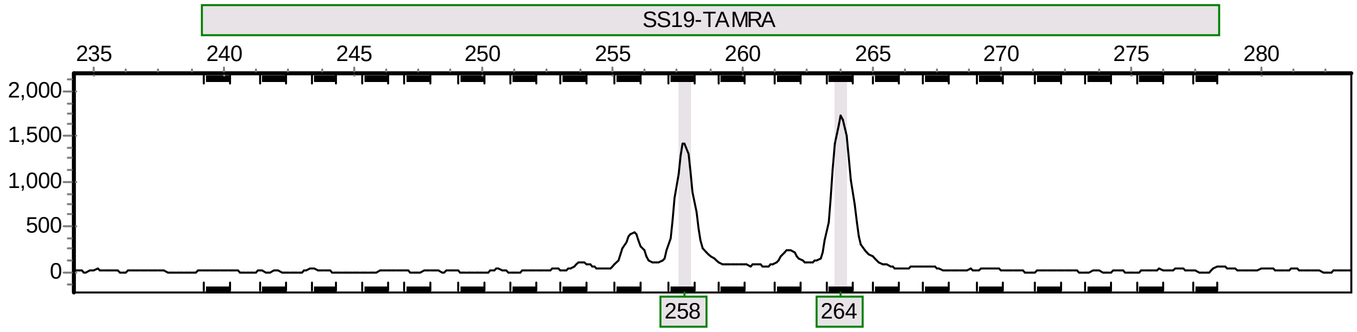


| No | Size | Height | Area | Marker | Allele | Difference | Quality | Score | Allele Comments | Sample Comments |
|---|---|---|---|---|---|---|---|---|---|---|
| 1 | 257.8 | 1415 | 11440 | SS19-TAMRA | 258 | 0.10 | Pass | 143.7 | [<Confirmed>] | |
| 2 | 263.8 | 1724 | 14679 | SS19-TAMRA | 264 | 0.00 | Pass | 186.7 | [<Confirmed>] | |

**Sample 48:** SSS13_SS20_SS11_SS21_SS02_SS19_HBN6_O01.fsa

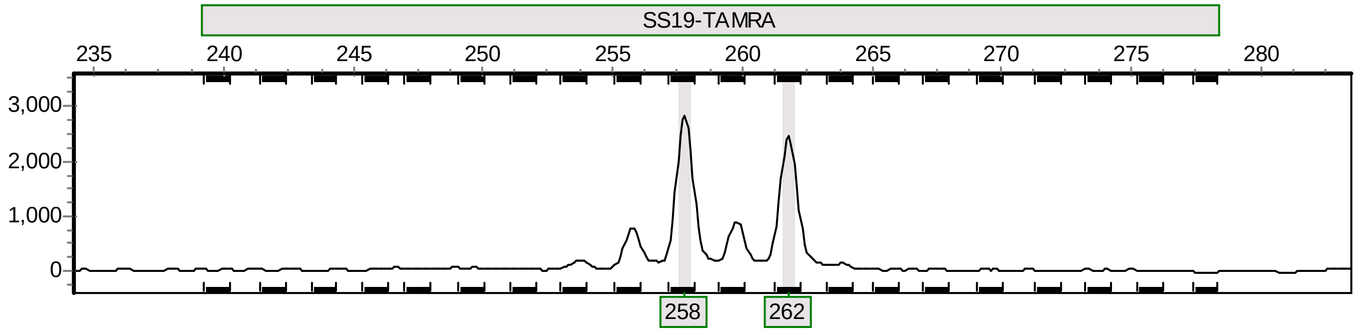


| No | Size | Height | Area | Marker | Allele | Difference | Quality | Score | Allele Comments | Sample Comments |
|---|---|---|---|---|---|---|---|---|---|---|
| 1 | 257.8 | 2816 | 21408 | SS19-TAMRA | 258 | 0.10 | Pass | 451.7 | [<Confirmed>] | |
| 2 | 261.8 | 2451 | 18563 | SS19-TAMRA | 262 | 0.00 | Pass | 366.8 | [<Confirmed>] | |

**Sample 49:** SSS13_SS20_SS11_SS21_SS02_SS19_HBN9_D03.fsa

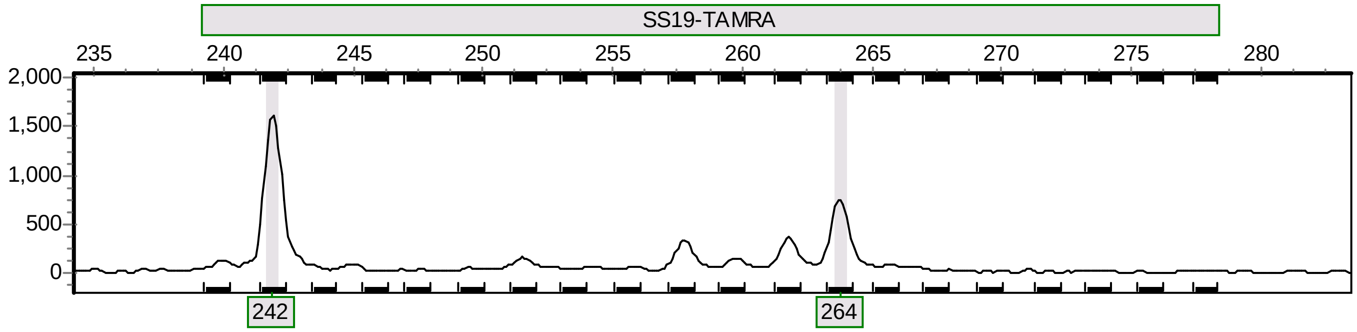


| No | Size | Height | Area | Marker | Allele | Difference | Quality | Score | Allele Comments | Sample Comments |
|---|---|---|---|---|---|---|---|---|---|---|
| 1 | 241.9 | 1605 | 12035 | SS19-TAMRA | 242 | 0.00 | Pass | 201.5 | [<Confirmed>] | |
| 2 | 263.8 | 750 | 6088 | SS19-TAMRA | 264 | 0.00 | Pass | 53.9 | [<Confirmed>] | |

**Sample 50:** SSS13_SS20_SS11_SS21_SS02_SS19_HCW1_M09.fsa

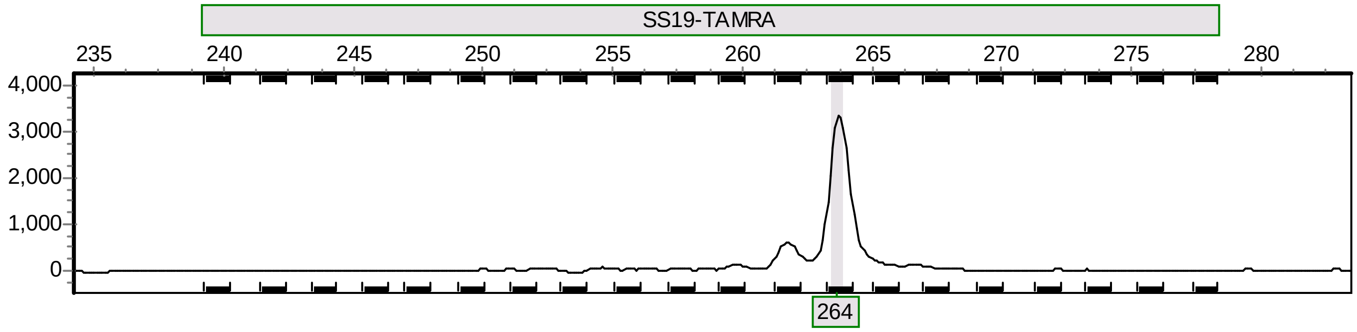


| No | Size | Height | Area | Marker | Allele | Difference | Quality | Score | Allele Comments | Sample Comments |
|---|---|---|---|---|---|---|---|---|---|---|
| 1 | 263.7 | 3327 | 29246 | SS19-TAMRA | 264 | 0.10 | Pass | 451.1 | [<Confirmed>] | |

**Sample 51:** SSS13_SS20_SS11_SS21_SS02_SS19_HCW2_A11.fsa

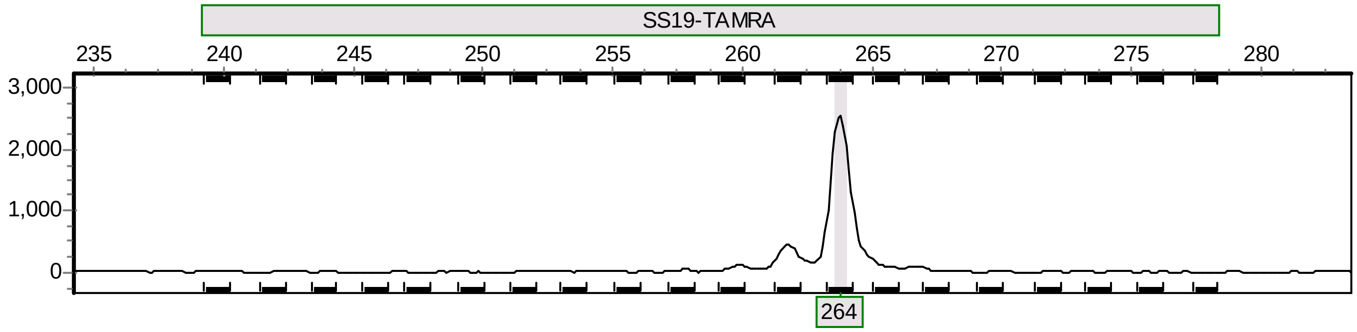


| No | Size | Height | Area | Marker | Allele | Difference | Quality | Score | Allele Comments | Sample Comments |
|---|---|---|---|---|---|---|---|---|---|---|
| 1 | 263.8 | 2543 | 21637 | SS19-TAMRA | 264 | 0.00 | Pass | 310.4 | [<Confirmed>] | |

**Sample 52:** SSS13_SS20_SS11_SS21_SS02_SS19_HCW3_G13.fsa

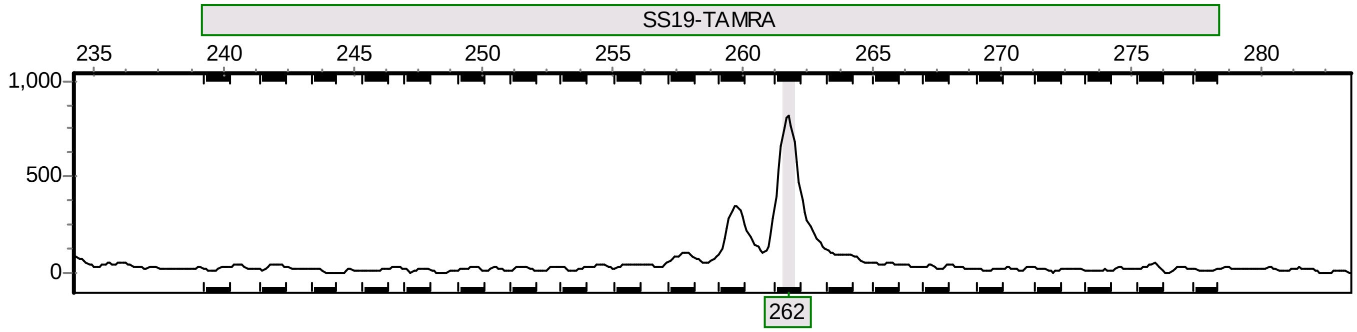


| No | Size | Height | Area | Marker | Allele | Difference | Quality | Score | Allele Comments | Sample Comments |
|---|---|---|---|---|---|---|---|---|---|---|
| 1 | 261.8 | 816 | 7330 | SS19-TAMRA | 262 | 0.00 | Pass | 43.0 | [<Confirmed>] | |

**Sample 53:** SSS13_SS20_SS11_SS21_SS02_SS19_HCW4_E09.fsa

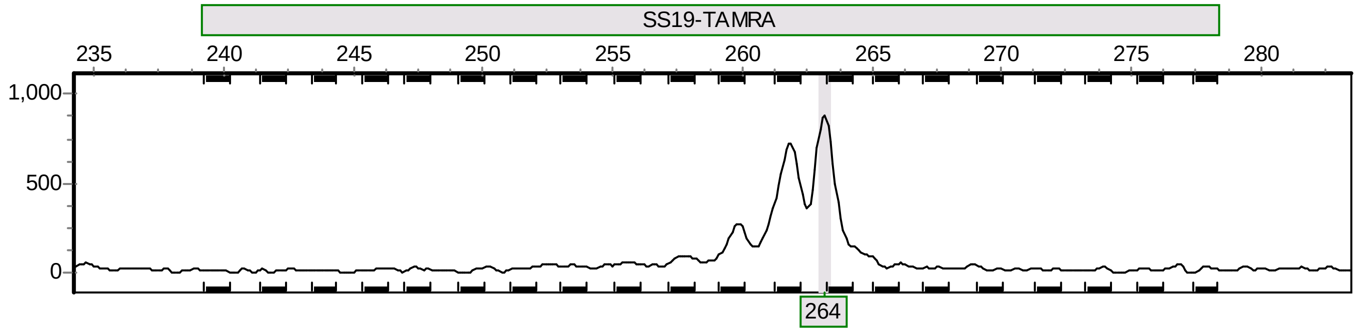


| No | Size | Height | Area | Marker | Allele | Difference | Quality | Score | Allele Comments | Sample Comments |
|---|---|---|---|---|---|---|---|---|---|---|
| 1 | 261.8 | 722 | 7202 | SS19-TAMRA | 262 | 0.00 | Pass | 23.3 | [<Deleted>] | |
| 2 | 263.2 | 876 | 8012 | SS19-TAMRA | 264 | 1.00 | Pass | 49.3 | [<Confirmed><Edited>] | |

**Sample 54:** SSS13_SS20_SS11_SS21_SS02_SS19_HCW5_C11.fsa

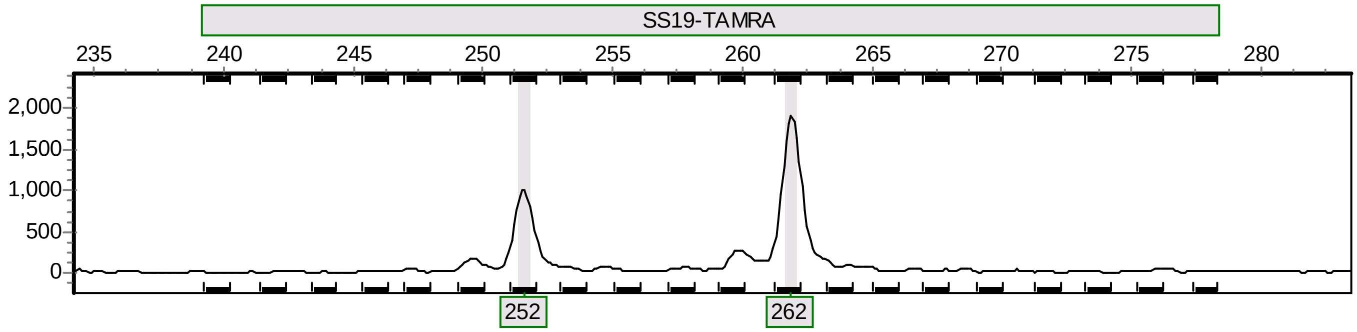


| No | Size | Height | Area | Marker | Allele | Difference | Quality | Score | Allele Comments | Sample Comments |
|---|---|---|---|---|---|---|---|---|---|---|
| 1 | 251.6 | 1009 | 8175 | SS19-TAMRA | 252 | 0.00 | Pass | 81.3 | [<Confirmed>] | |
| 2 | 261.9 | 1897 | 15716 | SS19-TAMRA | 262 | 0.10 | Pass | 217.9 | [<Confirmed>] | |

# Allele Report



**Sample 55:** SSS13_SS20_SS11_SS21_SS02_SS19_HCW6_D01.fsa

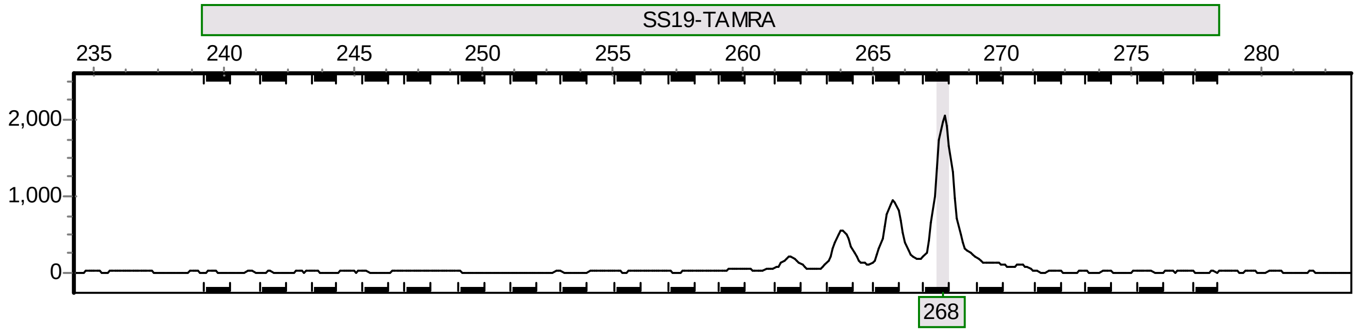


| No | Size | Height | Area | Marker | Allele | Difference | Quality | Score | Allele Comments | Sample Comments |
|---|---|---|---|---|---|---|---|---|---|---|
| 1 | 267.8 | 2048 | 15542 | SS19-TAMRA | 268 | 0.30 | Pass | 279.4 | [<Confirmed>] | |

**Sample 56:** SSS13_SS20_SS11_SS21_SS02_SS19_HCW7_G17.fsa

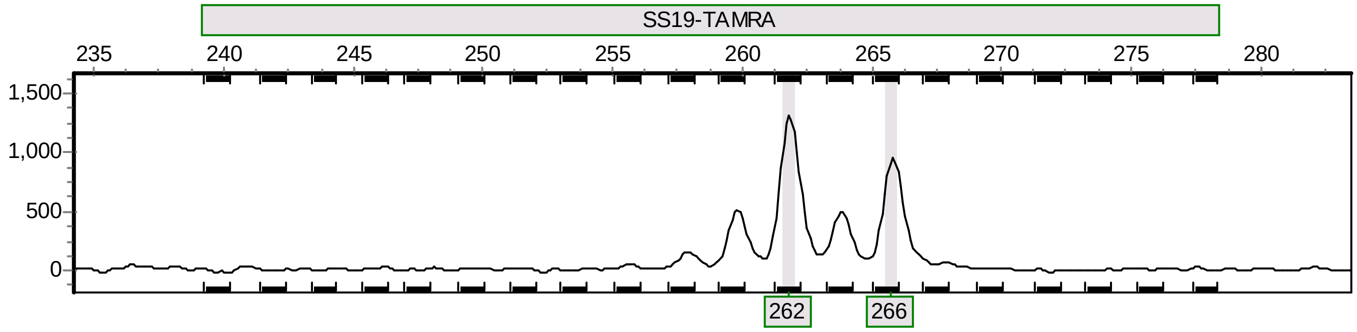


| No | Size | Height | Area | Marker | Allele | Difference | Quality | Score | Allele Comments | Sample Comments |
|---|---|---|---|---|---|---|---|---|---|---|
| 1 | 261.8 | 1300 | 11564 | SS19-TAMRA | 262 | 0.00 | Pass | 111.3 | [<Confirmed>] | |
| 2 | 265.8 | 948 | 8458 | SS19-TAMRA | 266 | 0.20 | Pass | 67.6 | [<Confirmed>] | |

**Sample 57:** SSS13_SS20_SS11_SS21_SS02_SS19_HCW8_A17.fsa

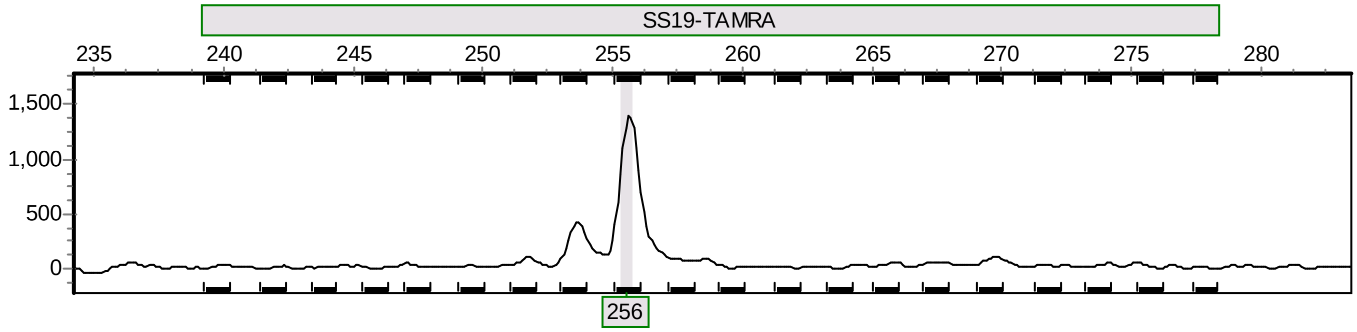


| No | Size | Height | Area | Marker | Allele | Difference | Quality | Score | Allele Comments | Sample Comments |
|---|---|---|---|---|---|---|---|---|---|---|
| 1 | 255.6 | 1391 | 12001 | SS19-TAMRA | 256 | 0.00 | Pass | 129.7 | [<Confirmed>] | |

# Allele Report

**Sample 58:** SSS13_SS20_SS11_SS21_SS02_SS19_HGC1_C01.fsa

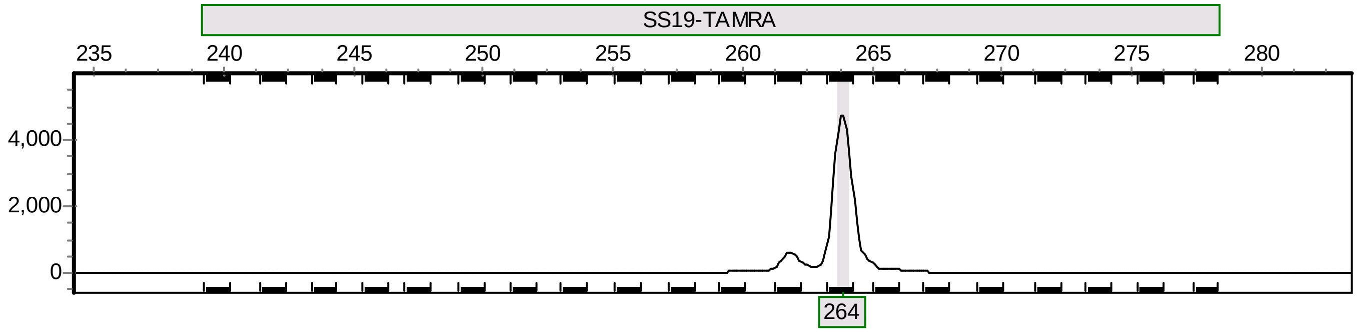


| No | Size | Height | Area | Marker | Allele | Difference | Quality | Score | Allele Comments | Sample Comments |
|---|---|---|---|---|---|---|---|---|---|---|
| 1 | 263.9 | 4693 | 37132 | SS19-TAMRA | 264 | 0.10 | Pass | 500.0 | [<Confirmed>] | |

**Sample 59:** SSS13_SS20_SS11_SS21_SS02_SS19_HGC3_E17.fsa

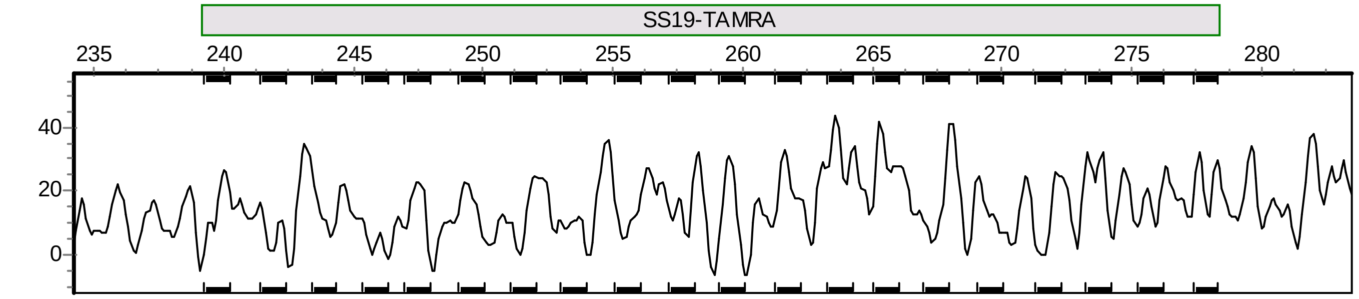


| No | Size | Height | Area | Marker | Allele | Difference | Quality | Score | Allele Comments | Sample Comments |
|---|---|---|---|---|---|---|---|---|---|---|

**Sample 60:** SSS13_SS20_SS11_SS21_SS02_SS19_HGC4_A13.fsa

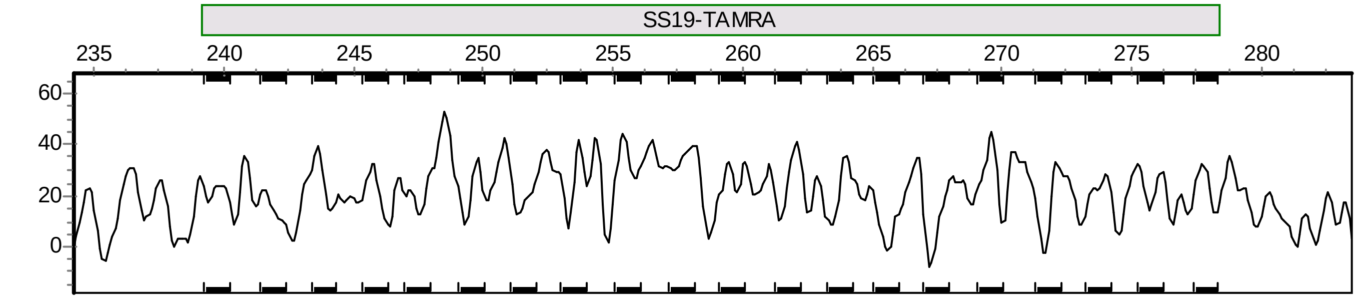


| No | Size | Height | Area | Marker | Allele | Difference | Quality | Score | Allele Comments | Sample Comments |
|---|---|---|---|---|---|---|---|---|---|---|

**Sample 61:** SSS13_SS20_SS11_SS21_SS02_SS19_HGY1_A09.fsa

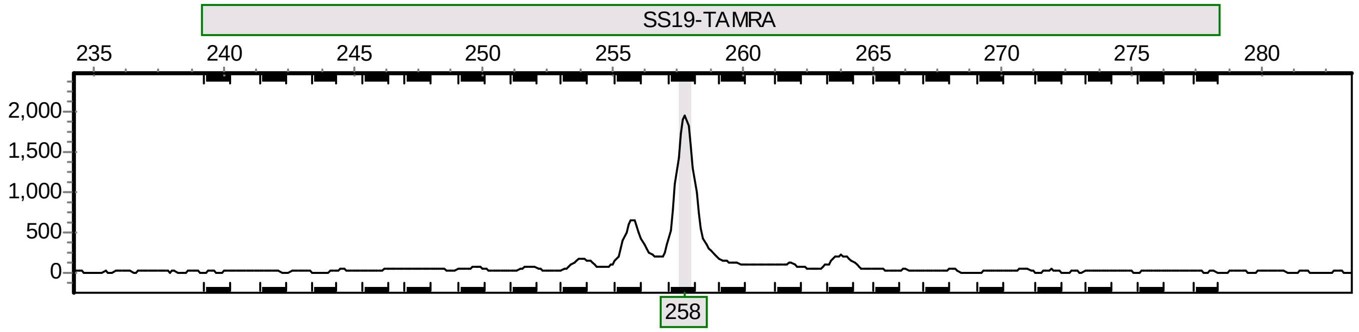


| No | Size | Height | Area | Marker | Allele | Difference | Quality | Score | Allele Comments | Sample Comments |
|---|---|---|---|---|---|---|---|---|---|---|

| | | | | | | | | | | |
|---|---|---|---|---|---|---|---|---|---|---|
| 1 | 257.8 | 1941 | 15964 | SS19-TAMRA | 258 | 0.10 | Pass | 215.8 | [<Confirmed>] | |

**Sample 62:** SSS13_SS20_SS11_SS21_SS02_SS19_HGY2_I13.fsa

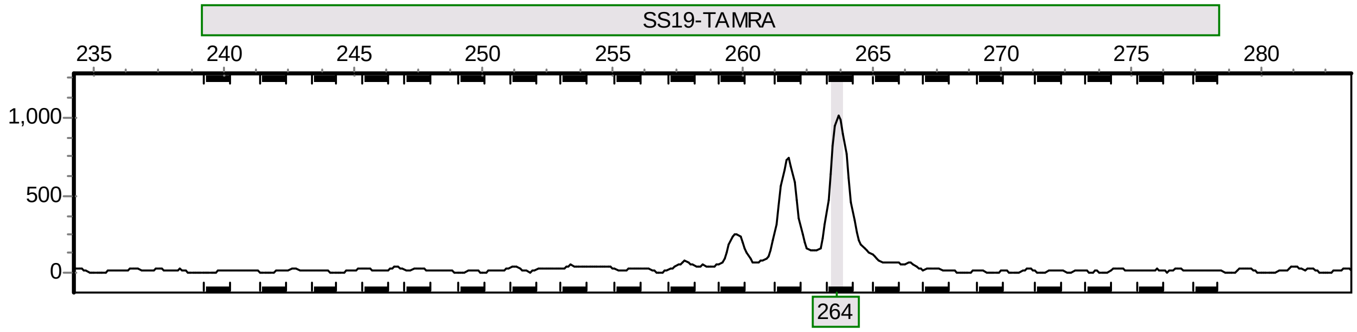


| No | Size | Height | Area | Marker | Allele | Difference | Quality | Score | Allele Comments | Sample Comments |
|---|---|---|---|---|---|---|---|---|---|---|
| 1 | 263.7 | 1006 | 8188 | SS19-TAMRA | 264 | 0.10 | Pass | 83.2 | [<Confirmed>] | |

**Sample 63:** SSS13_SS20_SS11_SS21_SS02_SS19_HGY3_I11.fsa

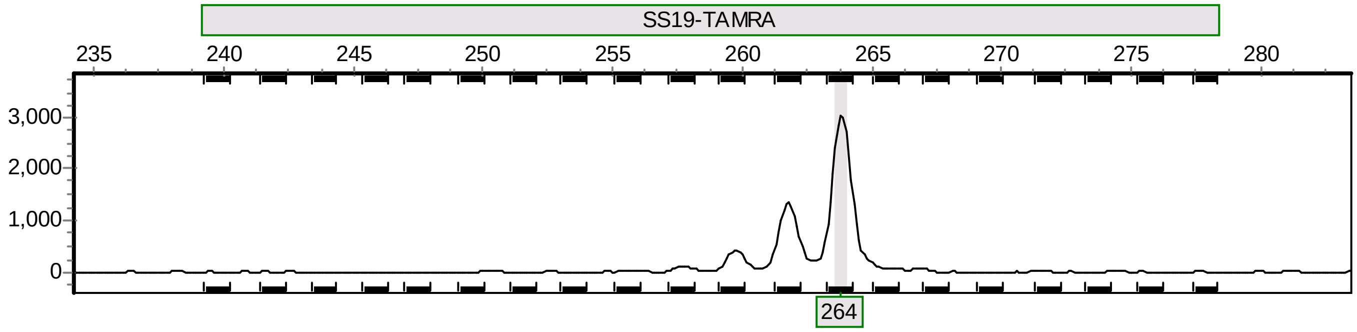


| No | Size | Height | Area | Marker | Allele | Difference | Quality | Score | Allele Comments | Sample Comments |
|---|---|---|---|---|---|---|---|---|---|---|
| 1 | 263.8 | 3011 | 25207 | SS19-TAMRA | 264 | 0.00 | Pass | 420.7 | [<Confirmed>] | |

**Sample 64:** SSS13_SS20_SS11_SS21_SS02_SS19_HGY4_I09.fsa

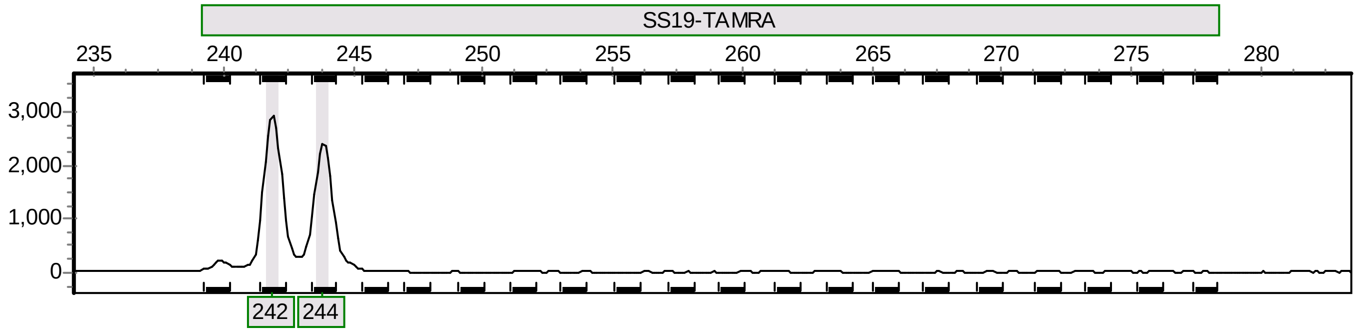


| No | Size | Height | Area | Marker | Allele | Difference | Quality | Score | Allele Comments | Sample Comments |
|---|---|---|---|---|---|---|---|---|---|---|
| 1 | 241.9 | 2918 | 22285 | SS19-TAMRA | 242 | 0.00 | Pass | 449.4 | [<Confirmed>] | |
| 2 | 243.8 | 2391 | 19231 | SS19-TAMRA | 244 | 0.10 | Pass | 328.0 | [<Confirmed>] | |

**Sample 65:** SSS13_SS20_SS11_SS21_SS02_SS19_HGY5_I15.fsa

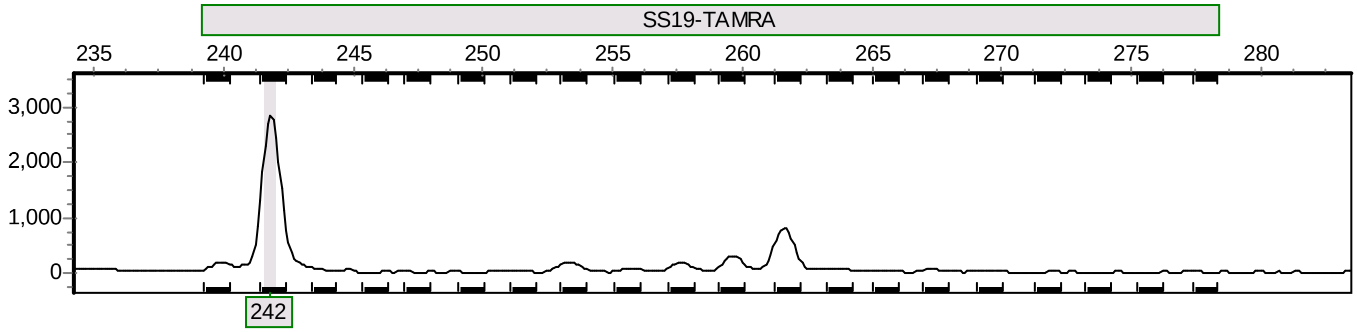


| No | Size | Height | Area | Marker | Allele | Difference | Quality | Score | Allele Comments | Sample Comments |
|---|---|---|---|---|---|---|---|---|---|---|
| 1 | 241.8 | 2828 | 22922 | SS19-TAMRA | 242 | 0.10 | Pass | 410.5 | [<Confirmed>] | |

**Sample 66:** SSS13_SS20_SS11_SS21_SS02_SS19_HQZ11_O11.fsa

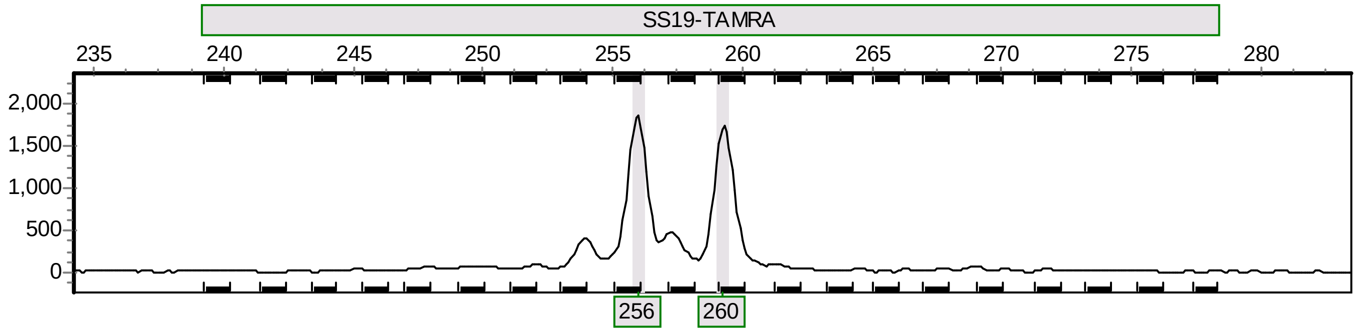


| No | Size | Height | Area | Marker | Allele | Difference | Quality | Score | Allele Comments | Sample Comments |
|---|---|---|---|---|---|---|---|---|---|---|
| 1 | 256.0 | 1850 | 15832 | SS19-TAMRA | 256 | 0.40 | Pass | 190.2 | [<Confirmed>] | |
| 2 | 259.3 | 1745 | 14969 | SS19-TAMRA | 260 | 0.30 | Pass | 176.9 | [<Confirmed>] | |

**Sample 67:** SSS13_SS20_SS11_SS21_SS02_SS19_HQZ13-1_C09.fsa

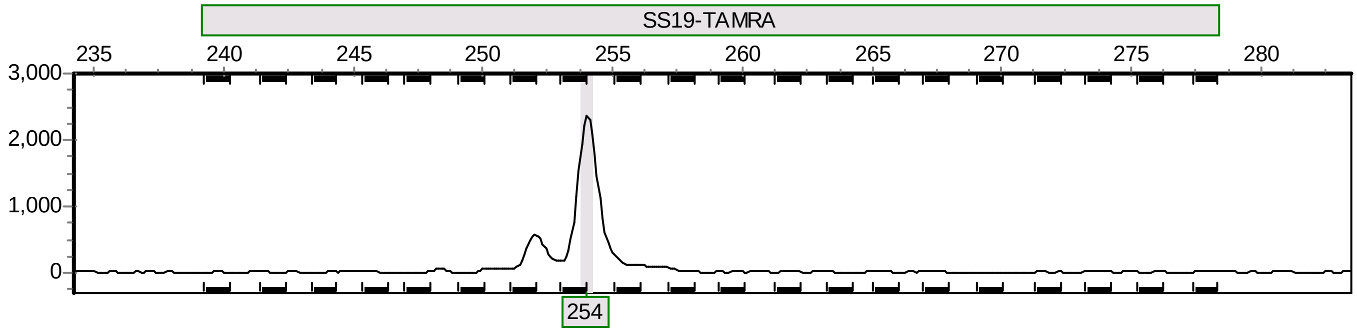


| No | Size | Height | Area | Marker | Allele | Difference | Quality | Score | Allele Comments | Sample Comments |
|---|---|---|---|---|---|---|---|---|---|---|
| 1 | 254.0 | 2357 | 20733 | SS19-TAMRA | 254 | 0.50 | Pass | 277.6 | [<Confirmed>] | |

# Allele Report



**Sample 68:** SSS13_SS20_SS11_SS21_SS02_SS19_HQZ13-2_J03.fsa

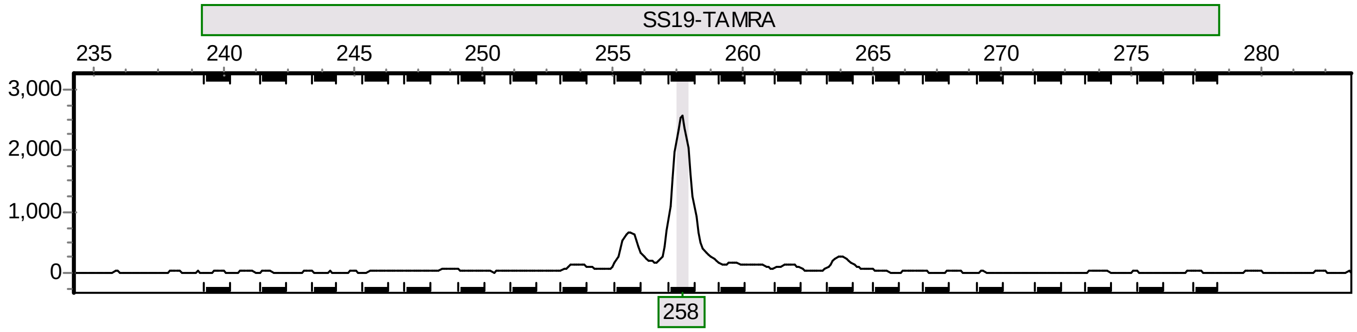


| No | Size | Height | Area | Marker | Allele | Difference | Quality | Score | Allele Comments | Sample Comments |
|---|---|---|---|---|---|---|---|---|---|---|
| 1 | 257.7 | 2551 | 20875 | SS19-TAMRA | 258 | 0.00 | Pass | 320.0 | [<Confirmed>] | |

**Sample 69:** SSS13_SS20_SS11_SS21_SS02_SS19_HQZ14_H03.fsa

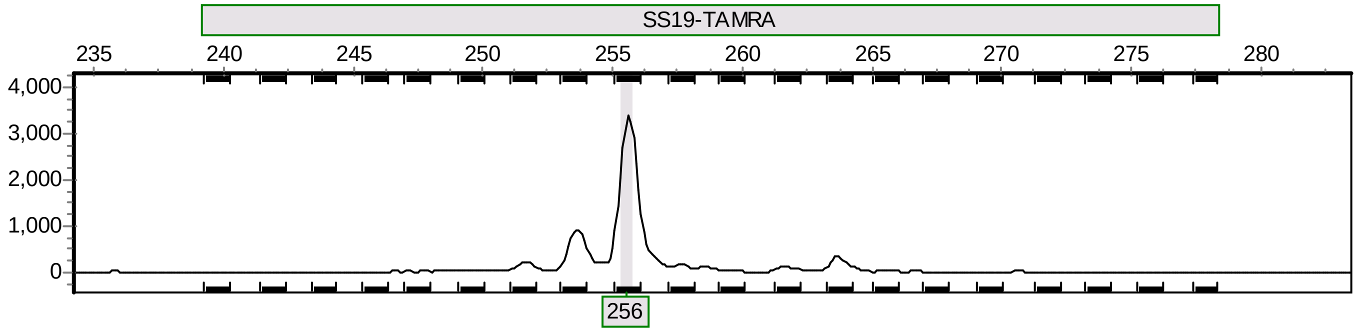


| No | Size | Height | Area | Marker | Allele | Difference | Quality | Score | Allele Comments | Sample Comments |
|---|---|---|---|---|---|---|---|---|---|---|
| 1 | 255.6 | 3358 | 25996 | SS19-TAMRA | 256 | 0.00 | Pass | 500.0 | [<Confirmed>] | |

**Sample 70:** SSS13_SS20_SS11_SS21_SS02_SS19_HQZ15_B01.fsa

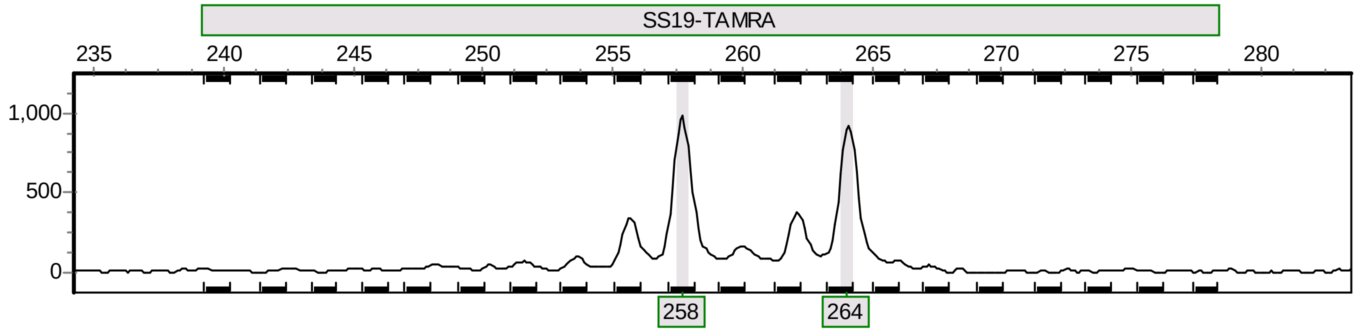


| No | Size | Height | Area | Marker | Allele | Difference | Quality | Score | Allele Comments | Sample Comments |
|---|---|---|---|---|---|---|---|---|---|---|
| 1 | 257.7 | 976 | 8093 | SS19-TAMRA | 258 | 0.00 | Pass | 81.0 | [<Confirmed>] | |
| 2 | 264.1 | 923 | 7516 | SS19-TAMRA | 264 | 0.30 | Pass | 81.5 | [<Confirmed>] | |

**Sample 71:** SSS13_SS20_SS11_SS21_SS02_SS19_HQZ16_P01.fsa

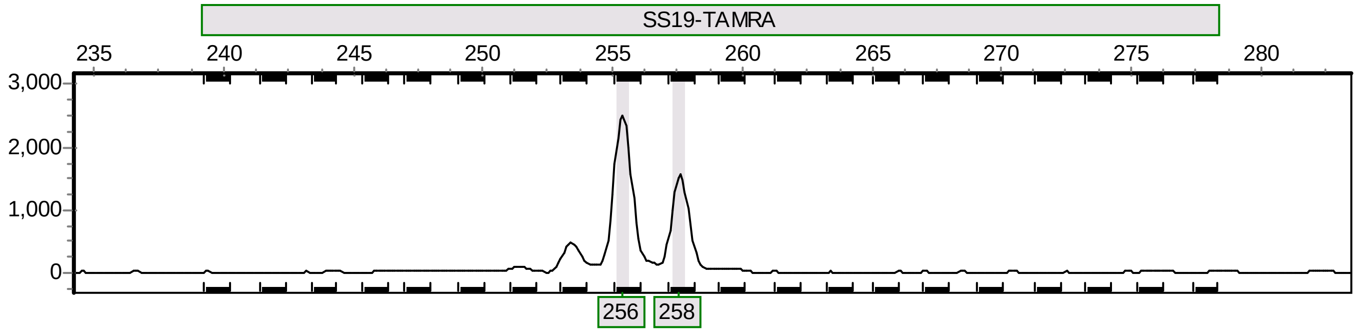


| No | Size | Height | Area | Marker | Allele | Difference | Quality | Score | Allele Comments | Sample Comments |
|---|---|---|---|---|---|---|---|---|---|---|
| 1 | 255.4 | 2485 | 18946 | SS19-TAMRA | 256 | 0.20 | Pass | 363.4 | [<Confirmed>] | |
| 2 | 257.6 | 1562 | 11745 | SS19-TAMRA | 258 | 0.10 | Pass | 201.6 | [<Confirmed>] | |

**Sample 72:** SSS13_SS20_SS11_SS21_SS02_SS19_HQZ17-1_O03.fsa

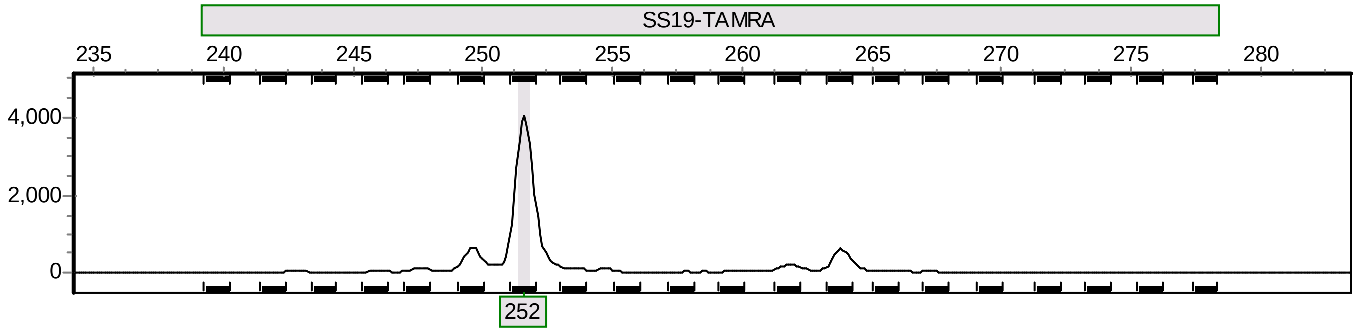


| No | Size | Height | Area | Marker | Allele | Difference | Quality | Score | Allele Comments | Sample Comments |
|---|---|---|---|---|---|---|---|---|---|---|
| 1 | 251.6 | 4040 | 31930 | SS19-TAMRA | 252 | 0.00 | Pass | 500.0 | [<Confirmed>] | |

**Sample 73:** SSS13_SS20_SS11_SS21_SS02_SS19_HQZ17-2_N03.fsa

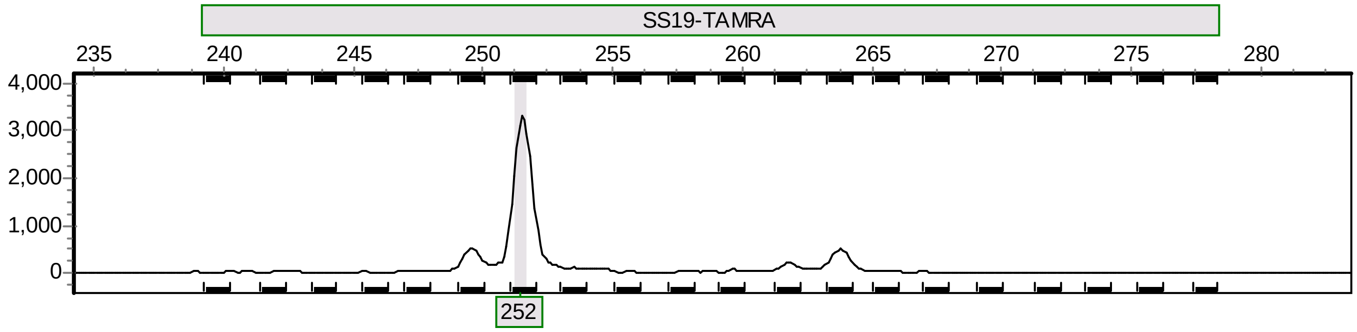


| No | Size | Height | Area | Marker | Allele | Difference | Quality | Score | Allele Comments | Sample Comments |
|---|---|---|---|---|---|---|---|---|---|---|
| 1 | 251.5 | 3299 | 26432 | SS19-TAMRA | 252 | 0.10 | Pass | 500.0 | [<Confirmed>] | |

**Sample 74:** SSS13_SS20_SS11_SS21_SS02_SS19_HQZ18_K09.fsa

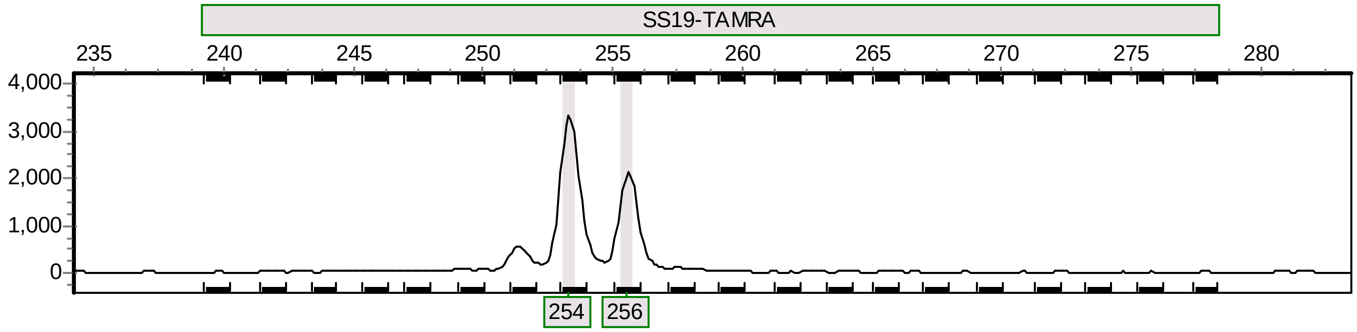


| No | Size | Height | Area | Marker | Allele | Difference | Quality | Score | Allele Comments | Sample Comments |
|---|---|---|---|---|---|---|---|---|---|---|
| 1 | 253.3 | 3310 | 28504 | SS19-TAMRA | 254 | 0.20 | Pass | 455.0 | [<Confirmed>] | |
| 2 | 255.6 | 2121 | 17457 | SS19-TAMRA | 256 | 0.00 | Pass | 261.5 | [<Confirmed>] | |

**Sample 75:** SSS13_SS20_SS11_SS21_SS02_SS19_HQZ19_L03.fsa

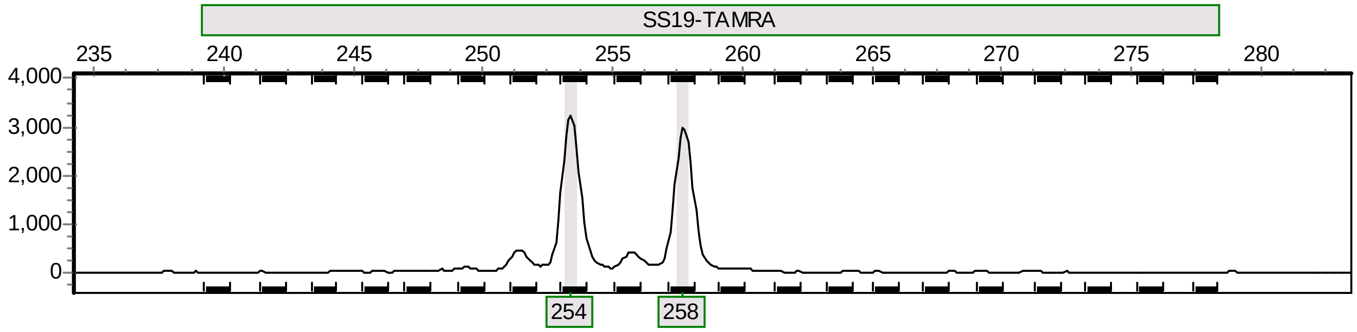


| No | Size | Height | Area | Marker | Allele | Difference | Quality | Score | Allele Comments | Sample Comments |
|---|---|---|---|---|---|---|---|---|---|---|
| 1 | 253.4 | 3212 | 24699 | SS19-TAMRA | 254 | 0.10 | Pass | 500.0 | [<Confirmed>] | |
| 2 | 257.7 | 2970 | 24006 | SS19-TAMRA | 258 | 0.00 | Pass | 435.5 | [<Confirmed>] | |

**Sample 76:** SSS13_SS20_SS11_SS21_SS02_SS19_HQZ21_B03.fsa

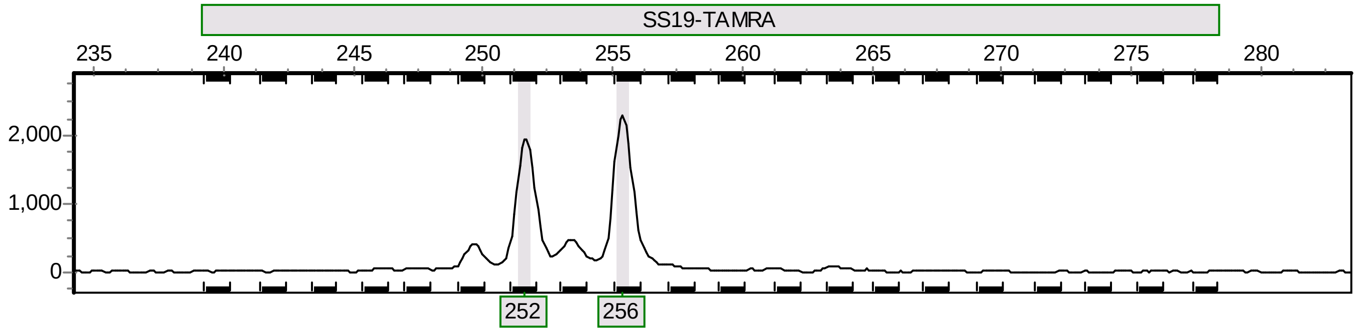


| No | Size | Height | Area | Marker | Allele | Difference | Quality | Score | Allele Comments | Sample Comments |
|---|---|---|---|---|---|---|---|---|---|---|
| 1 | 251.6 | 1946 | 16587 | SS19-TAMRA | 252 | 0.00 | Pass | 224.7 | [<Confirmed>] | |
| 2 | 255.4 | 2276 | 18636 | SS19-TAMRA | 256 | 0.20 | Pass | 289.9 | [<Confirmed>] | |

**Sample 77:** SSS13_SS20_SS11_SS21_SS02_SS19_HQZ22-1_M01.fsa

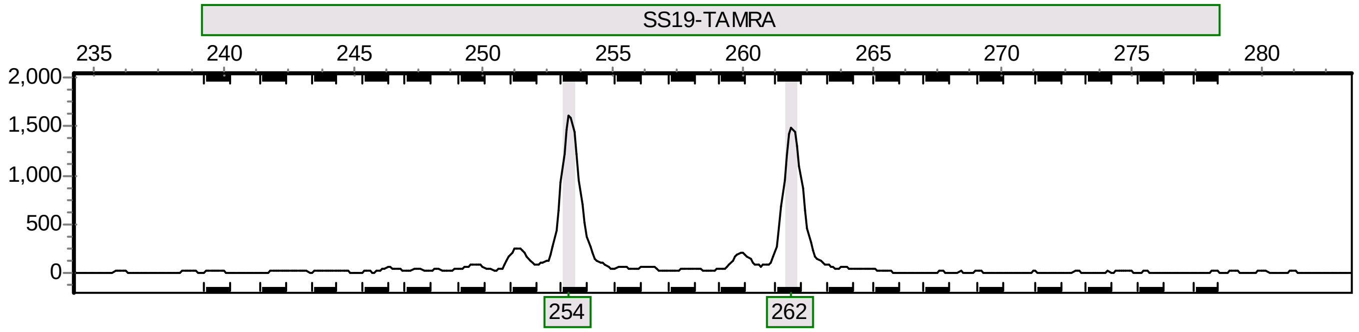


| No | Size | Height | Area | Marker | Allele | Difference | Quality | Score | Allele Comments | Sample Comments |
|---|---|---|---|---|---|---|---|---|---|---|
| 1 | 253.3 | 1597 | 13074 | SS19-TAMRA | 254 | 0.20 | Pass | 187.5 | [<Confirmed>] | |
| 2 | 261.9 | 1489 | 12333 | SS19-TAMRA | 262 | 0.10 | Pass | 160.2 | [<Confirmed>] | |

**Sample 78:** SSS13_SS20_SS11_SS21_SS02_SS19_HQZ22-2_O13.fsa

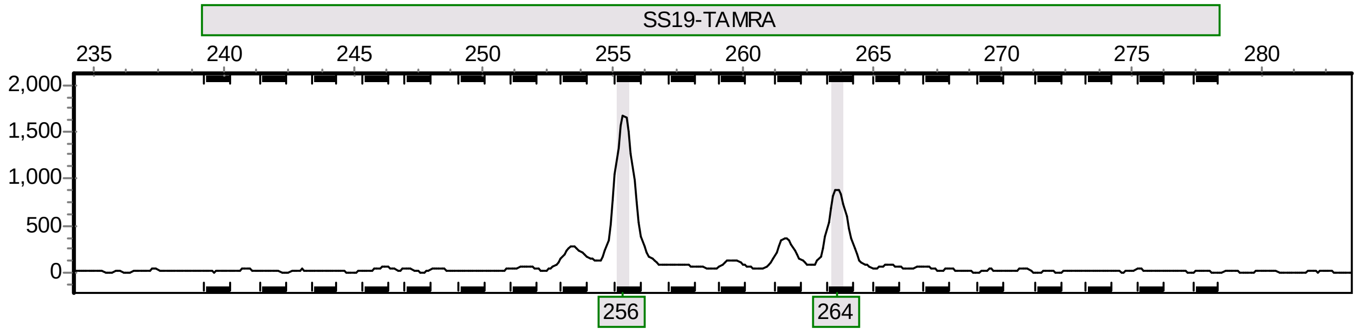


| No | Size | Height | Area | Marker | Allele | Difference | Quality | Score | Allele Comments | Sample Comments |
|---|---|---|---|---|---|---|---|---|---|---|
| 1 | 255.4 | 1666 | 13861 | SS19-TAMRA | 256 | 0.20 | Pass | 180.5 | [<Confirmed>] | |
| 2 | 263.7 | 883 | 7727 | SS19-TAMRA | 264 | 0.10 | Pass | 57.7 | [<Confirmed>] | |

**Sample 79:** SSS13_SS20_SS11_SS21_SS02_SS19_HQZ23_A15.fsa

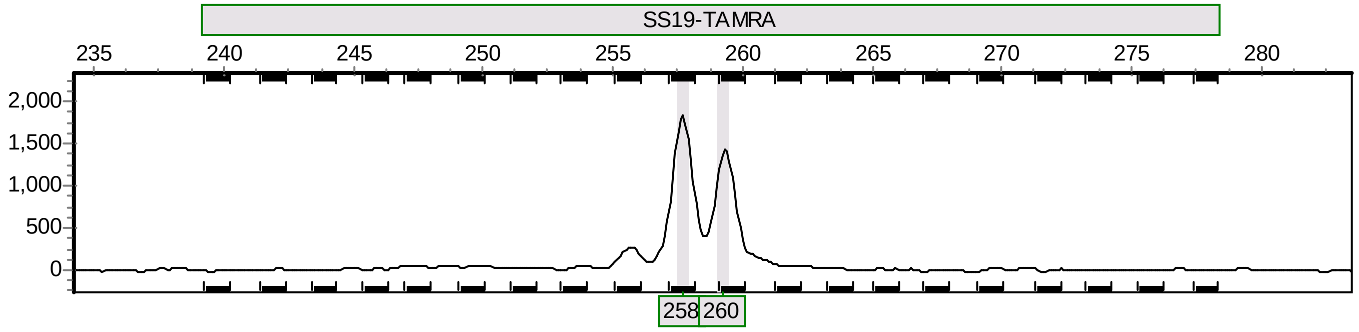


| No | Size | Height | Area | Marker | Allele | Difference | Quality | Score | Allele Comments | Sample Comments |
|---|---|---|---|---|---|---|---|---|---|---|
| 1 | 257.7 | 1823 | 16516 | SS19-TAMRA | 258 | 0.00 | Pass | 168.0 | [<Confirmed>] | |
| 2 | 259.3 | 1421 | 12641 | SS19-TAMRA | 260 | 0.30 | Pass | 115.4 | [<Confirmed>] | |

# Allele Report

**Sample 80:** SSS13_SS20_SS11_SS21_SS02_SS19_HQZ24_E11.fsa

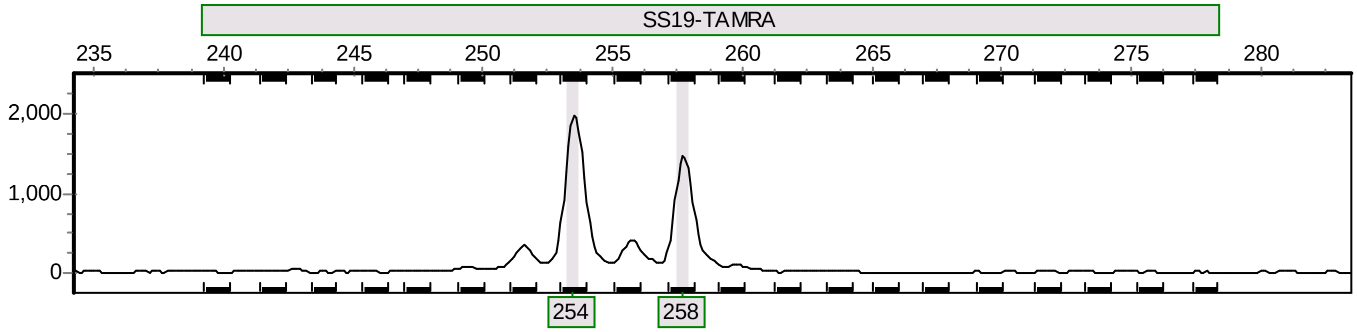


| No | Size | Height | Area | Marker | Allele | Difference | Quality | Score | Allele Comments | Sample Comments |
|---|---|---|---|---|---|---|---|---|---|---|
| 1 | 253.5 | 1967 | 16790 | SS19-TAMRA | 254 | 0.00 | Pass | 223.4 | [<Confirmed>] | |
| 2 | 257.7 | 1461 | 12360 | SS19-TAMRA | 258 | 0.00 | Pass | 150.2 | [<Confirmed>] | |

**Sample 81:** SSS13_SS20_SS11_SS21_SS02_SS19_HQZ25_N01.fsa

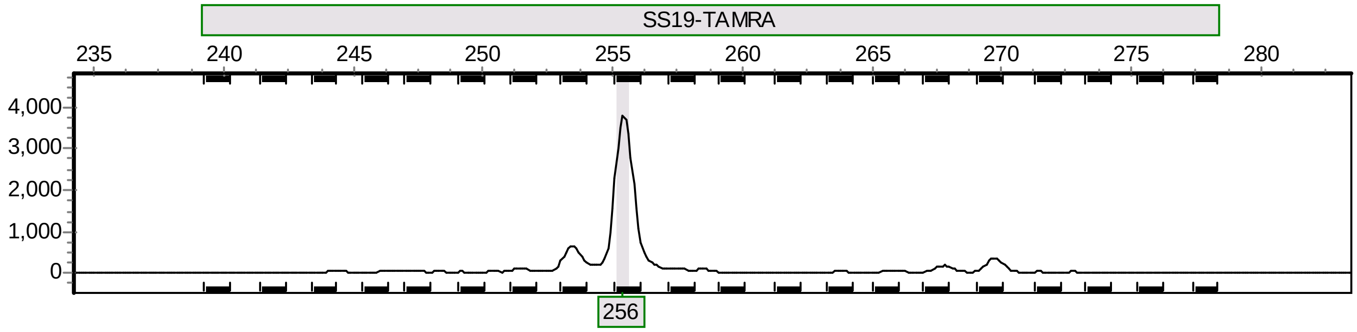


| No | Size | Height | Area | Marker | Allele | Difference | Quality | Score | Allele Comments | Sample Comments |
|---|---|---|---|---|---|---|---|---|---|---|
| 1 | 255.4 | 3771 | 29925 | SS19-TAMRA | 256 | 0.20 | Pass | 500.0 | [<Confirmed>] | |

**Sample 82:** SSS13_SS20_SS11_SS21_SS02_SS19_HQZ26_E03.fsa

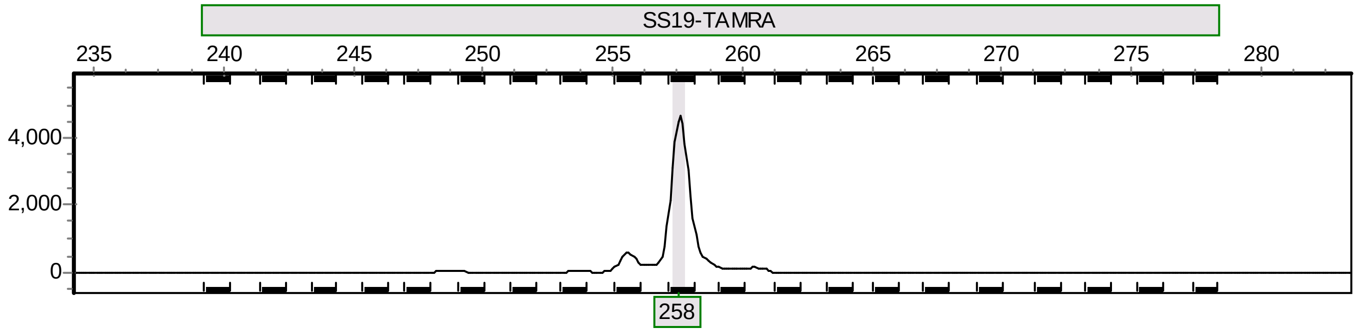


| No | Size | Height | Area | Marker | Allele | Difference | Quality | Score | Allele Comments | Sample Comments |
|---|---|---|---|---|---|---|---|---|---|---|
| 1 | 257.6 | 4610 | 36082 | SS19-TAMRA | 258 | 0.10 | Pass | 500.0 | [<Confirmed>] | |

**Sample 83:** SSS13_SS20_SS11_SS21_SS02_SS19_HQZ27_G09.fsa

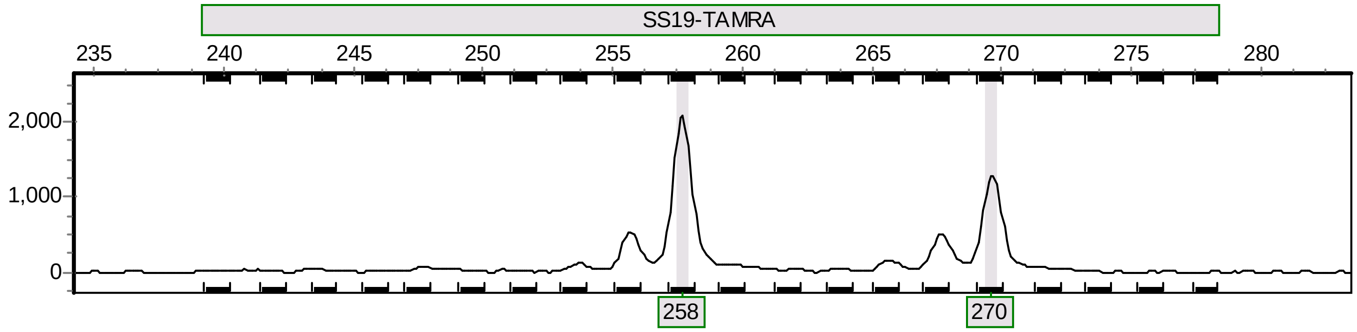


| No | Size | Height | Area | Marker | Allele | Difference | Quality | Score | Allele Comments | Sample Comments |
|---|---|---|---|---|---|---|---|---|---|---|
| 1 | 257.7 | 2064 | 17539 | SS19-TAMRA | 258 | 0.00 | Pass | 242.6 | [<Confirmed>] | |
| 2 | 269.6 | 1281 | 10868 | SS19-TAMRA | 270 | 0.00 | Pass | 114.5 | [<Confirmed>] | |

**Sample 84:** SSS13_SS20_SS11_SS21_SS02_SS19_HQZ28_B05.fsa

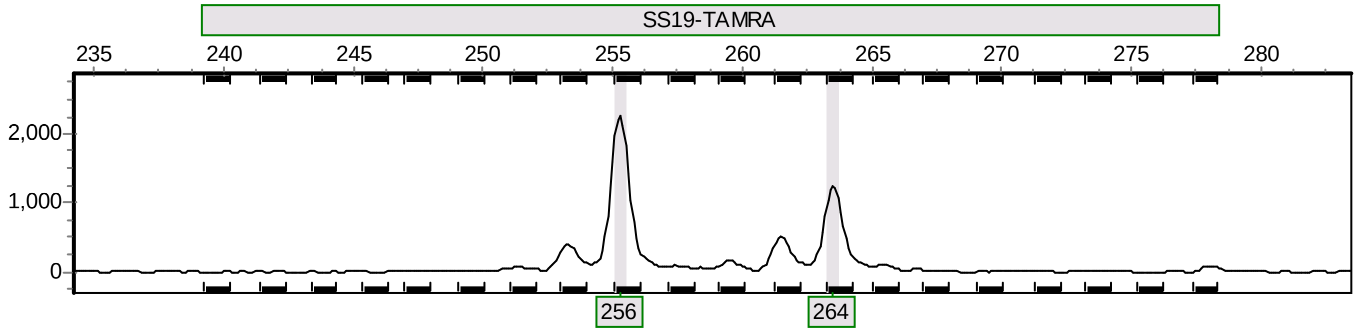


| No | Size | Height | Area | Marker | Allele | Difference | Quality | Score | Allele Comments | Sample Comments |
|---|---|---|---|---|---|---|---|---|---|---|
| 1 | 255.3 | 2236 | 17372 | SS19-TAMRA | 256 | 0.30 | Pass | 301.9 | [<Confirmed>] | |
| 2 | 263.5 | 1245 | 10051 | SS19-TAMRA | 264 | 0.30 | Pass | 127.9 | [<Confirmed>] | |

**Sample 85:** SSS13_SS20_SS11_SS21_SS02_SS19_HQZ29_C13.fsa

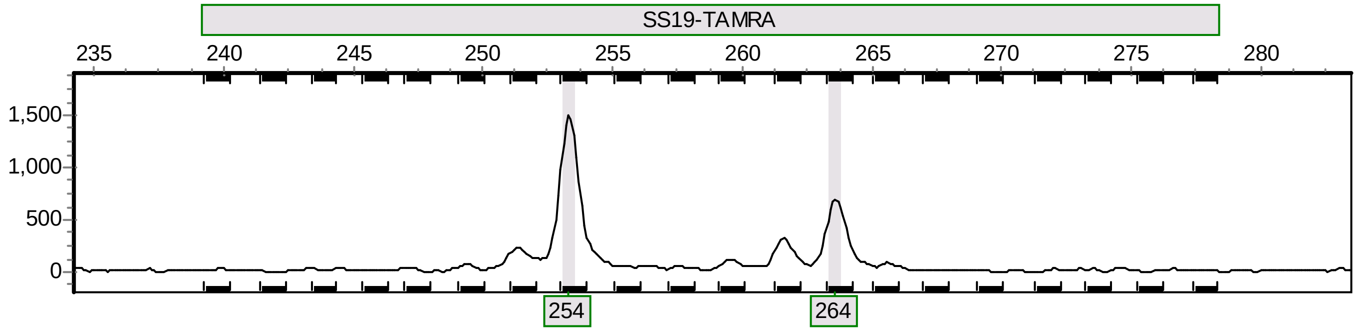


| No | Size | Height | Area | Marker | Allele | Difference | Quality | Score | Allele Comments | Sample Comments |
|---|---|---|---|---|---|---|---|---|---|---|
| 1 | 253.3 | 1495 | 12364 | SS19-TAMRA | 254 | 0.20 | Pass | 153.2 | [<Confirmed>] | |
| 2 | 263.6 | 698 | 6237 | SS19-TAMRA | 264 | 0.20 | Pass | 41.6 | [<Confirmed>] | |

**Sample 86:** SSS13_SS20_SS11_SS21_SS02_SS19_HQZ2_K03.fsa

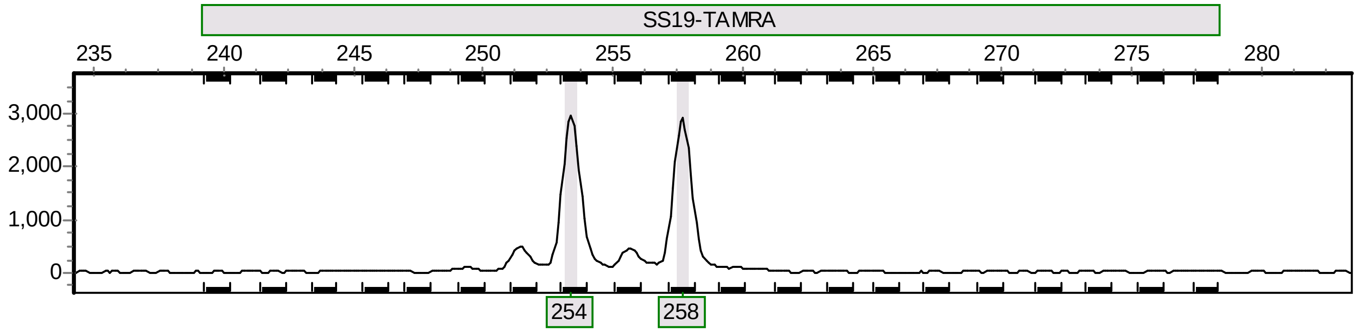


| No | Size | Height | Area | Marker | Allele | Difference | Quality | Score | Allele Comments | Sample Comments |
|---|---|---|---|---|---|---|---|---|---|---|
| 1 | 253.4 | 2958 | 23315 | SS19-TAMRA | 254 | 0.10 | Pass | 453.7 | [<Confirmed>] | |
| 2 | 257.7 | 2928 | 23375 | SS19-TAMRA | 258 | 0.00 | Pass | 431.3 | [<Confirmed>] | |

**Sample 87:** SSS13_SS20_SS11_SS21_SS02_SS19_HQZ30_G15.fsa

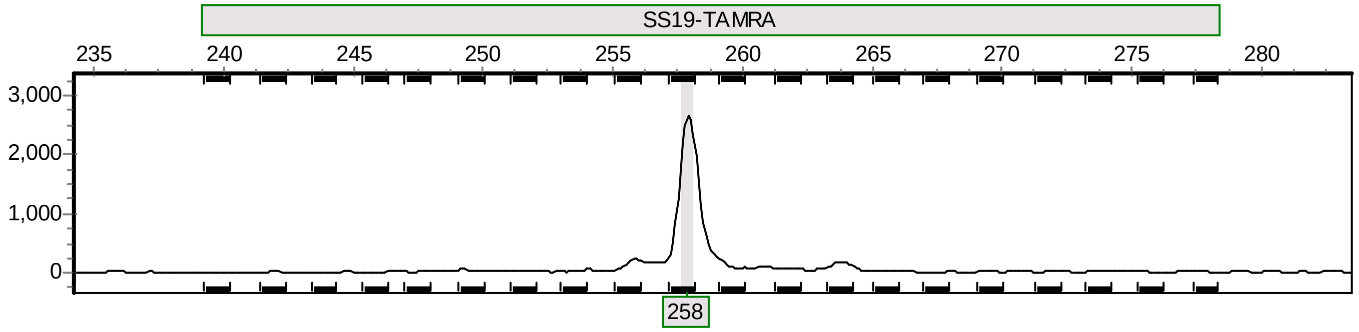


| No | Size | Height | Area | Marker | Allele | Difference | Quality | Score | Allele Comments | Sample Comments |
|---|---|---|---|---|---|---|---|---|---|---|
| 1 | 257.9 | 2632 | 22355 | SS19-TAMRA | 258 | 0.20 | Pass | 340.9 | [<Confirmed>] | |

**Sample 88:** SSS13_SS20_SS11_SS21_SS02_SS19_HQZ31_K13.fsa

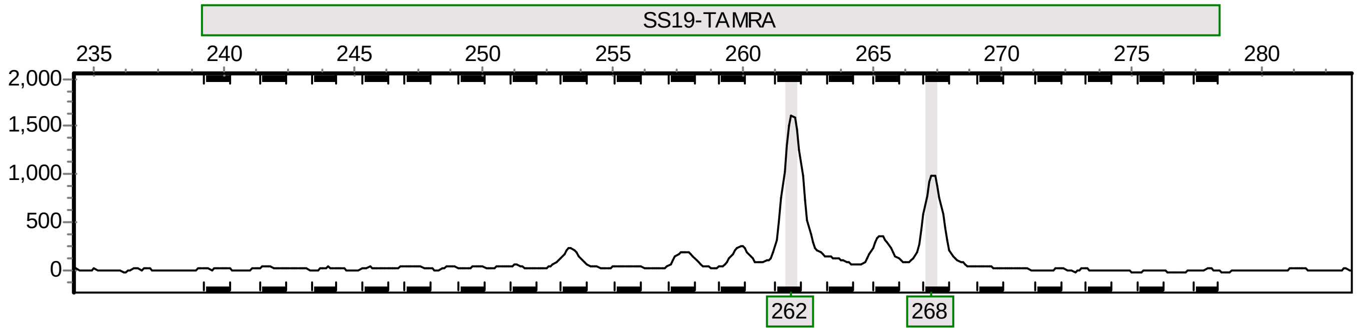


| No | Size | Height | Area | Marker | Allele | Difference | Quality | Score | Allele Comments | Sample Comments |
|---|---|---|---|---|---|---|---|---|---|---|
| 1 | 261.9 | 1606 | 13873 | SS19-TAMRA | 262 | 0.10 | Pass | 159.1 | [<Confirmed>] | |
| 2 | 267.3 | 993 | 8197 | SS19-TAMRA | 268 | 0.20 | Pass | 88.3 | [<Confirmed>] | |

**Sample 89:** SSS13_SS20_SS11_SS21_SS02_SS19_HQZ32_J01.fsa

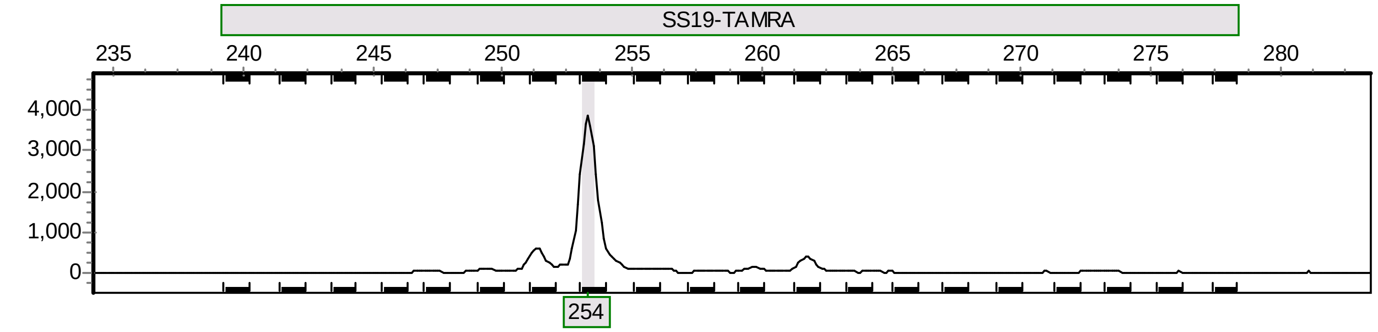


| No | Size | Height | Area | Marker | Allele | Difference | Quality | Score | Allele Comments | Sample Comments |
|---|---|---|---|---|---|---|---|---|---|---|
| 1 | 253.3 | 3830 | 27879 | SS19-TAMRA | 254 | 0.20 | Pass | 500.0 | [<Confirmed>] | |

**Sample 90:** SSS13_SS20_SS11_SS21_SS02_SS19_HQZ33_I03.fsa

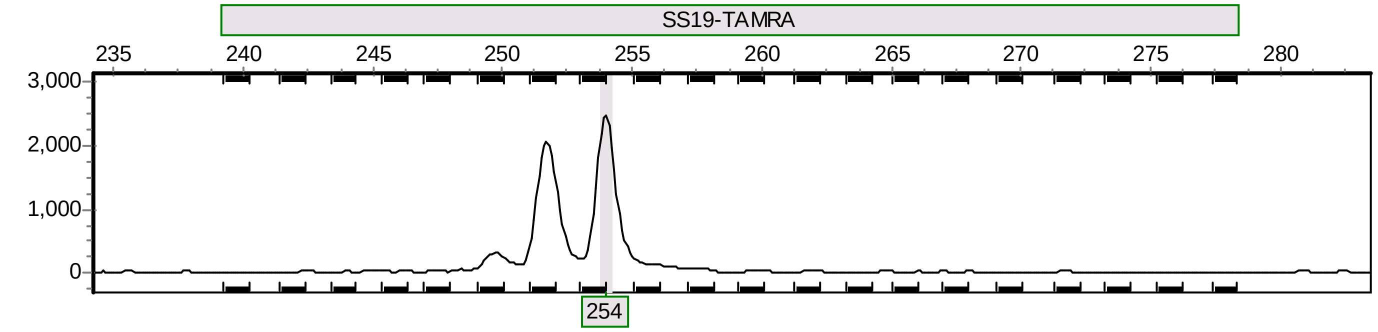


| No | Size | Height | Area | Marker | Allele | Difference | Quality | Score | Allele Comments | Sample Comments |
|---|---|---|---|---|---|---|---|---|---|---|
| 1 | 254.0 | 2456 | 19639 | SS19-TAMRA | 254 | 0.50 | Pass | 317.1 | [<Confirmed>] | |

**Sample 91:** SSS13_SS20_SS11_SS21_SS02_SS19_HQZ34_M03.fsa

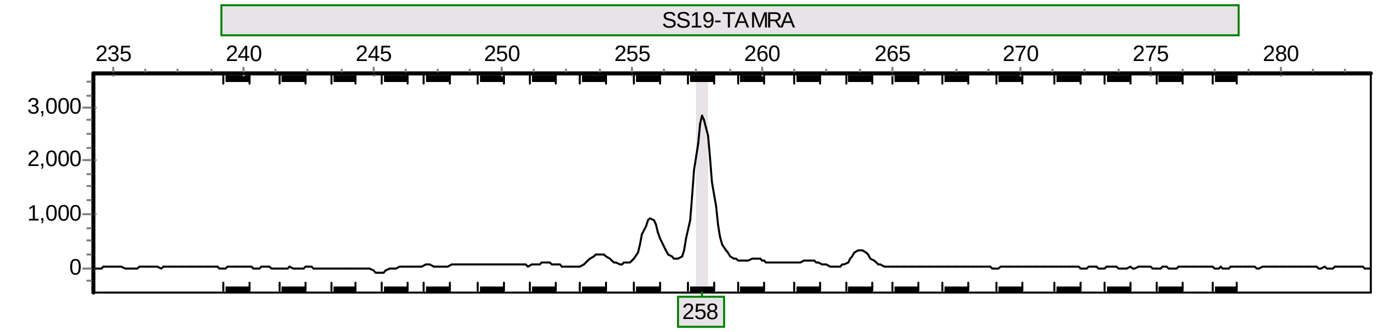


| No | Size | Height | Area | Marker | Allele | Difference | Quality | Score | Allele Comments | Sample Comments |
|---|---|---|---|---|---|---|---|---|---|---|
| 1 | 257.7 | 2831 | 22883 | SS19-TAMRA | 258 | 0.00 | Pass | 400.0 | [<Confirmed>] | |

**Sample 92:** SSS13_SS20_SS11_SS21_SS02_SS19_HQZ35_A01.fsa

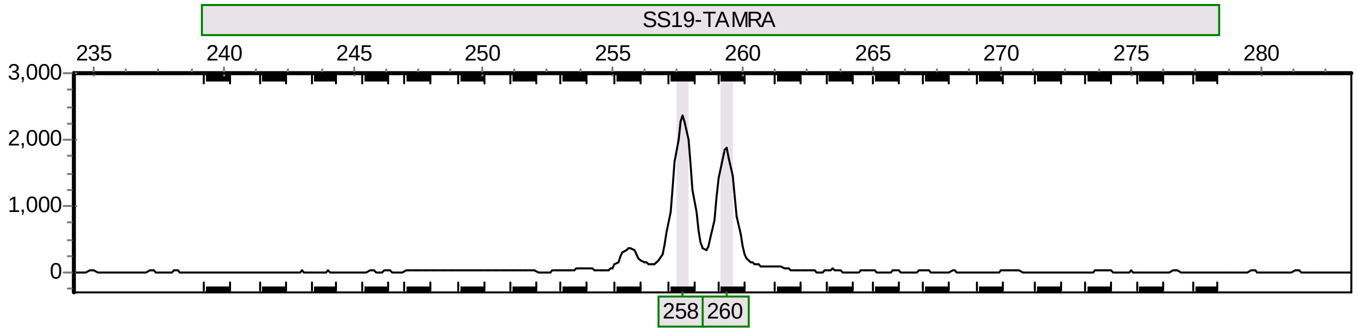


| No | Size | Height | Area | Marker | Allele | Difference | Quality | Score | Allele Comments | Sample Comments |
|---|---|---|---|---|---|---|---|---|---|---|
| 1 | 257.7 | 2355 | 20094 | SS19-TAMRA | 258 | 0.00 | Pass | 291.5 | [<Confirmed>] | |
| 2 | 259.4 | 1867 | 15037 | SS19-TAMRA | 260 | 0.20 | Pass | 214.2 | [<Confirmed>] | |

**Sample 93:** SSS13_SS20_SS11_SS21_SS02_SS19_HQZ36_E15.fsa

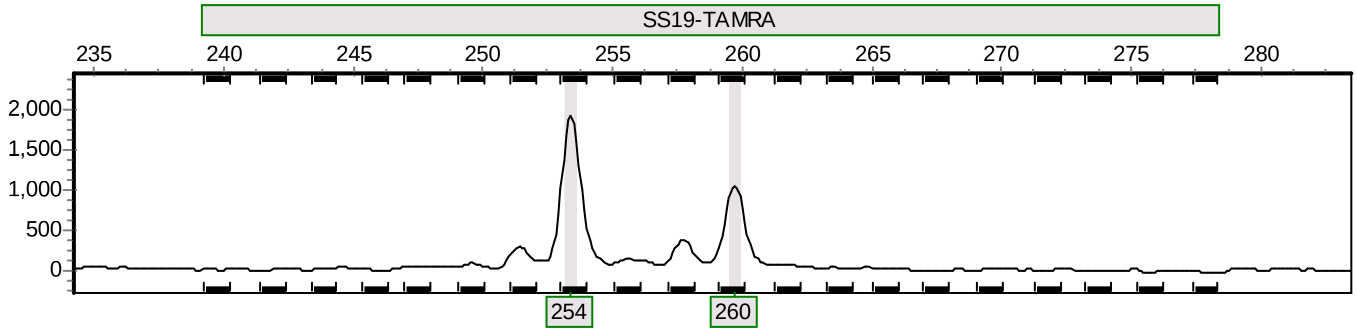


| No | Size | Height | Area | Marker | Allele | Difference | Quality | Score | Allele Comments | Sample Comments |
|---|---|---|---|---|---|---|---|---|---|---|
| 1 | 253.4 | 1905 | 16027 | SS19-TAMRA | 254 | 0.10 | Pass | 215.3 | [<Confirmed>] | |
| 2 | 259.7 | 1047 | 9115 | SS19-TAMRA | 260 | 0.10 | Pass | 77.2 | [<Confirmed>] | |

**Sample 94:** SSS13_SS20_SS11_SS21_SS02_SS19_HQZ37_F03.fsa

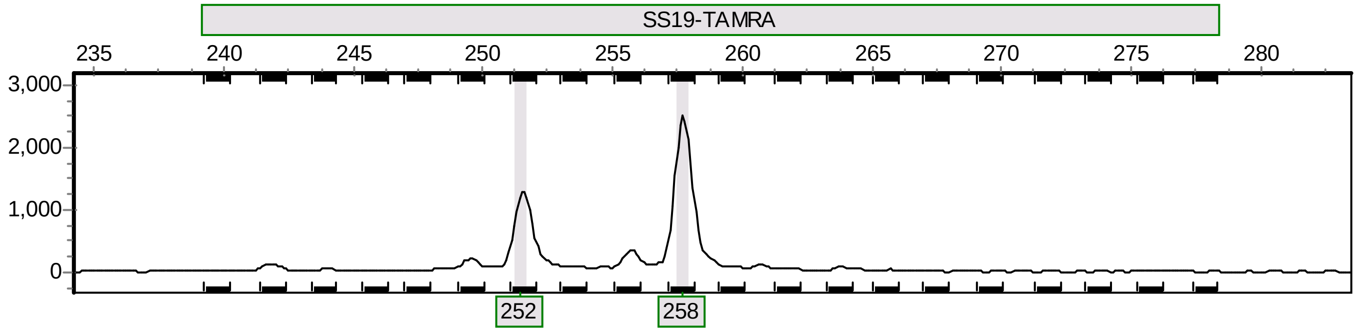


| No | Size | Height | Area | Marker | Allele | Difference | Quality | Score | Allele Comments | Sample Comments |
|---|---|---|---|---|---|---|---|---|---|---|
| 1 | 251.5 | 1306 | 10195 | SS19-TAMRA | 252 | 0.10 | Pass | 144.0 | [<Confirmed>] | |
| 2 | 257.7 | 2512 | 19690 | SS19-TAMRA | 258 | 0.00 | Pass | 376.0 | [<Confirmed>] | |

# Allele Report

**Sample 95:** SSS13_SS20_SS11_SS21_SS02_SS19_HQZ38_L01.fsa

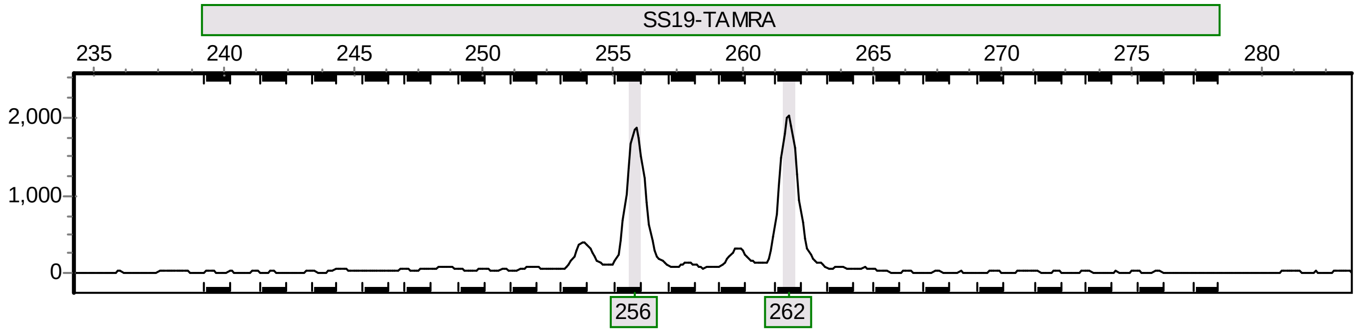


| No | Size | Height | Area | Marker | Allele | Difference | Quality | Score | Allele Comments | Sample Comments |
|---|---|---|---|---|---|---|---|---|---|---|
| 1 | 255.9 | 1874 | 15150 | SS19-TAMRA | 256 | 0.30 | Pass | 226.4 | [<Confirmed>] | |
| 2 | 261.8 | 2017 | 15880 | SS19-TAMRA | 262 | 0.00 | Pass | 253.3 | [<Confirmed>] | |

**Sample 96:** SSS13_SS20_SS11_SS21_SS02_SS19_HQZ39_M15.fsa

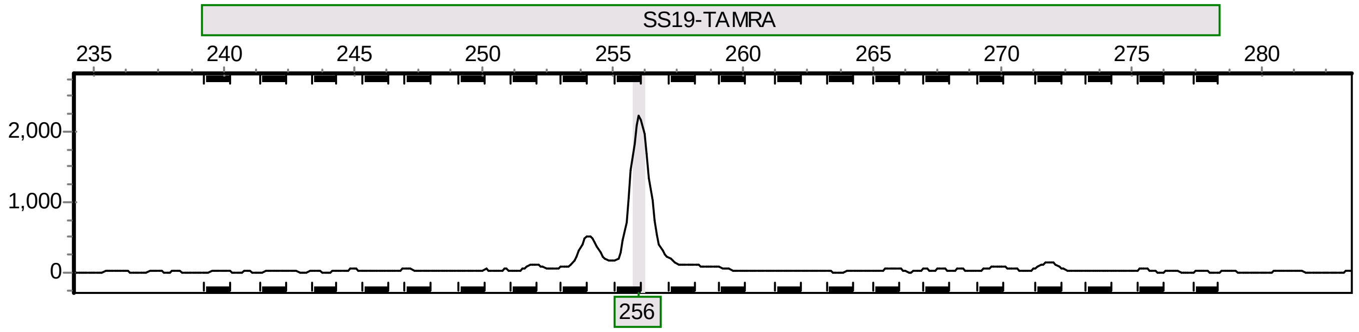


| No | Size | Height | Area | Marker | Allele | Difference | Quality | Score | Allele Comments | Sample Comments |
|---|---|---|---|---|---|---|---|---|---|---|
| 1 | 256.0 | 2216 | 19140 | SS19-TAMRA | 256 | 0.40 | Pass | 261.7 | [<Confirmed>] | |

**Sample 97:** SSS13_SS20_SS11_SS21_SS02_SS19_HQZ7_C03.fsa

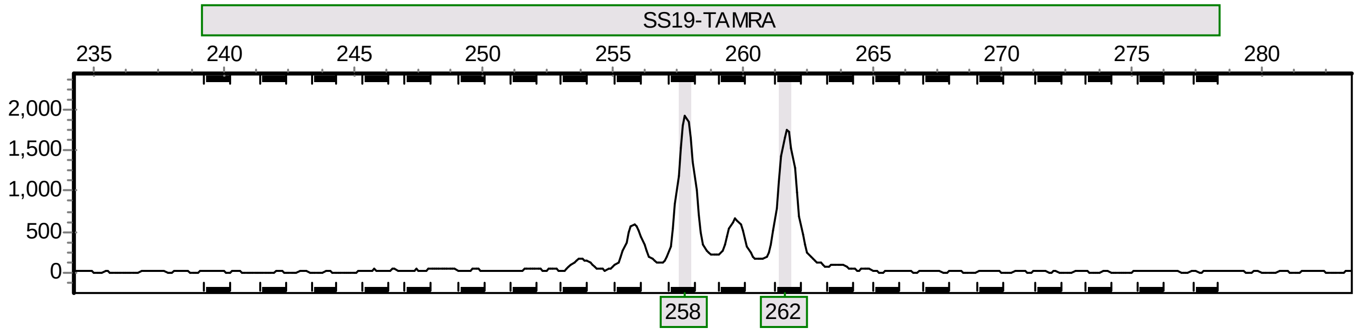


| No | Size | Height | Area | Marker | Allele | Difference | Quality | Score | Allele Comments | Sample Comments |
|---|---|---|---|---|---|---|---|---|---|---|
| 1 | 257.8 | 1911 | 15034 | SS19-TAMRA | 258 | 0.10 | Pass | 253.3 | [<Confirmed>] | |
| 2 | 261.7 | 1758 | 14214 | SS19-TAMRA | 262 | 0.10 | Pass | 211.7 | [<Confirmed>] | |

# Allele Report



**Sample 98:** SSS13_SS20_SS11_SS21_SS02_SS19_HQZ9_K11.fsa

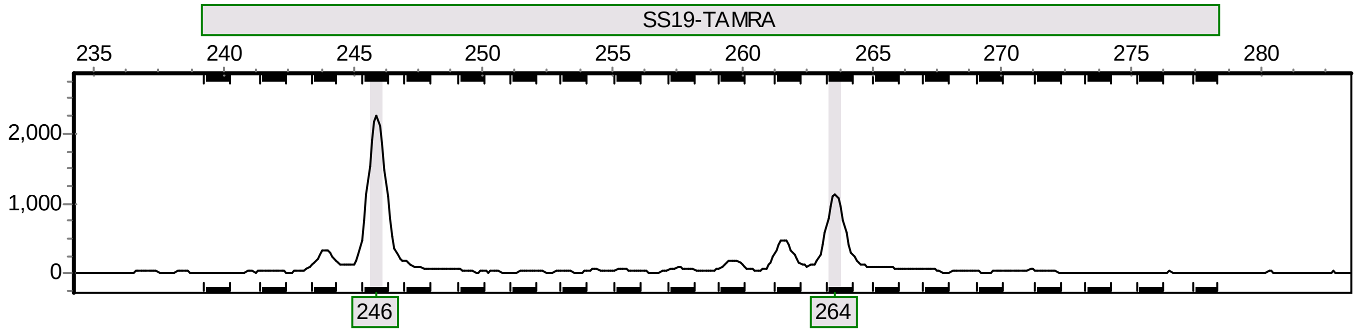


| No | Size | Height | Area | Marker | Allele | Difference | Quality | Score | Allele Comments | Sample Comments |
|---|---|---|---|---|---|---|---|---|---|---|
| 1 | 245.9 | 2258 | 17978 | SS19-TAMRA | 246 | 0.00 | Pass | 305.3 | [<Confirmed>] | |
| 2 | 263.6 | 1145 | 9536 | SS19-TAMRA | 264 | 0.20 | Pass | 105.8 | [<Confirmed>] | |

**Sample 99:** SSS13_SS20_SS11_SS21_SS02_SS19_HRS24_I17.fsa

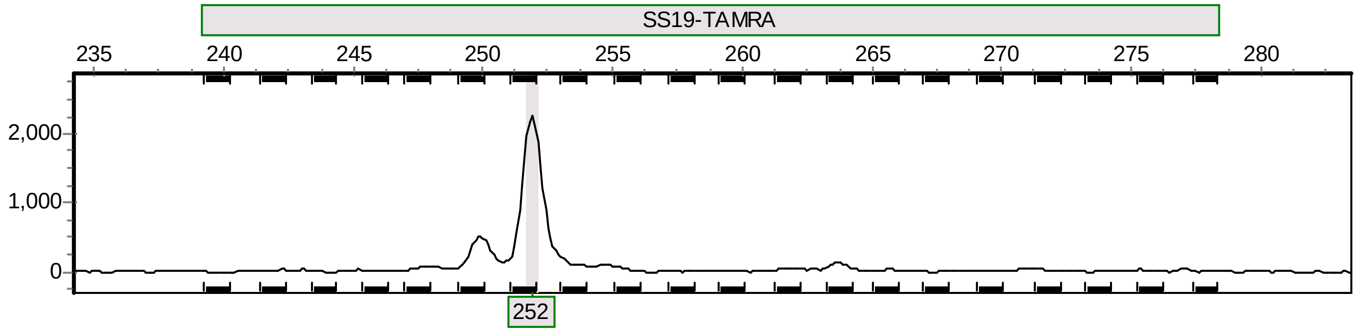


| No | Size | Height | Area | Marker | Allele | Difference | Quality | Score | Allele Comments | Sample Comments |
|---|---|---|---|---|---|---|---|---|---|---|
| 1 | 251.9 | 2235 | 18992 | SS19-TAMRA | 252 | 0.30 | Pass | 258.8 | [<Confirmed>] | |

**Sample 100:** SSS13_SS20_SS11_SS21_SS02_SS19_HRS26_H09.fsa

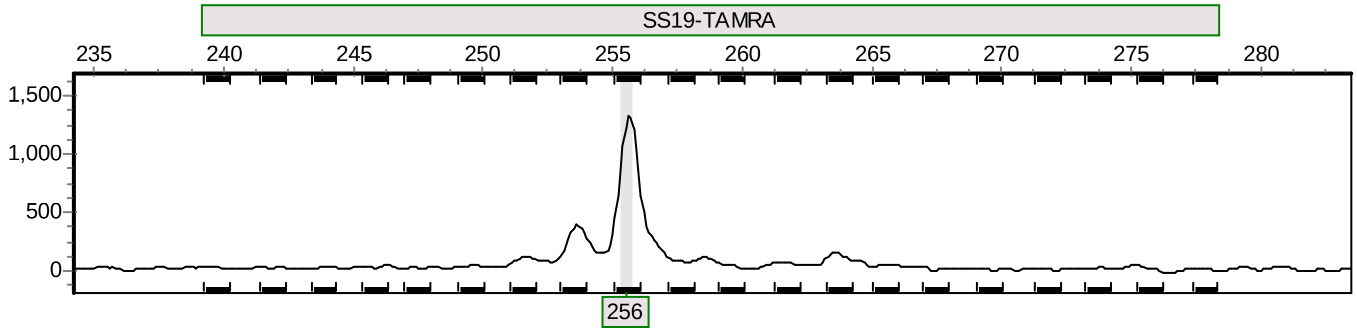


| No | Size | Height | Area | Marker | Allele | Difference | Quality | Score | Allele Comments | Sample Comments |
|---|---|---|---|---|---|---|---|---|---|---|
| 1 | 255.6 | 1323 | 11407 | SS19-TAMRA | 256 | 0.00 | Pass | 116.5 | [<Confirmed>] | |

**Sample 101:** SSS13_SS20_SS11_SS21_SS02_SS19_HRS28_D13.fsa

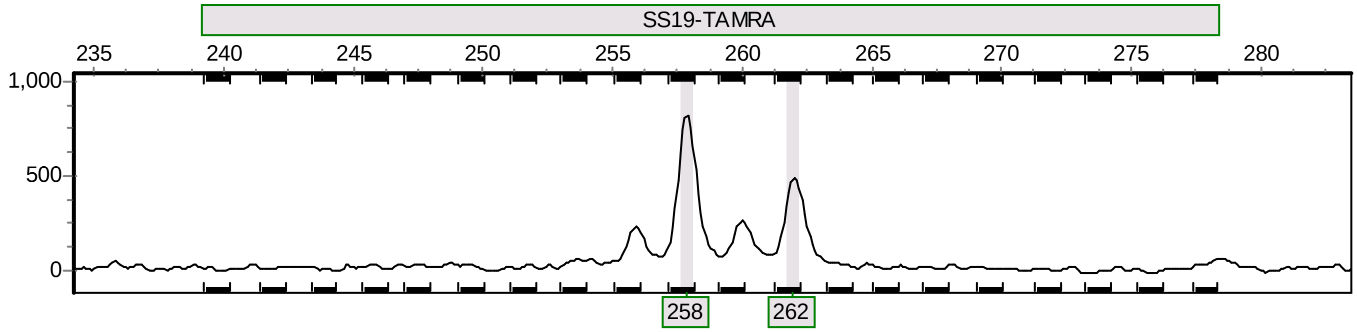


| No | Size | Height | Area | Marker | Allele | Difference | Quality | Score | Allele Comments | Sample Comments |
|---|---|---|---|---|---|---|---|---|---|---|
| 1 | 257.9 | 816 | 6704 | SS19-TAMRA | 258 | 0.20 | Pass | 57.4 | [<Confirmed>] | |
| 2 | 262.0 | 493 | 4436 | SS19-TAMRA | 262 | 0.20 | Pass | 22.1 | [<Confirmed>] | |

**Sample 102:** SSS13_SS20_SS11_SS21_SS02_SS19_HRS29_J05.fsa

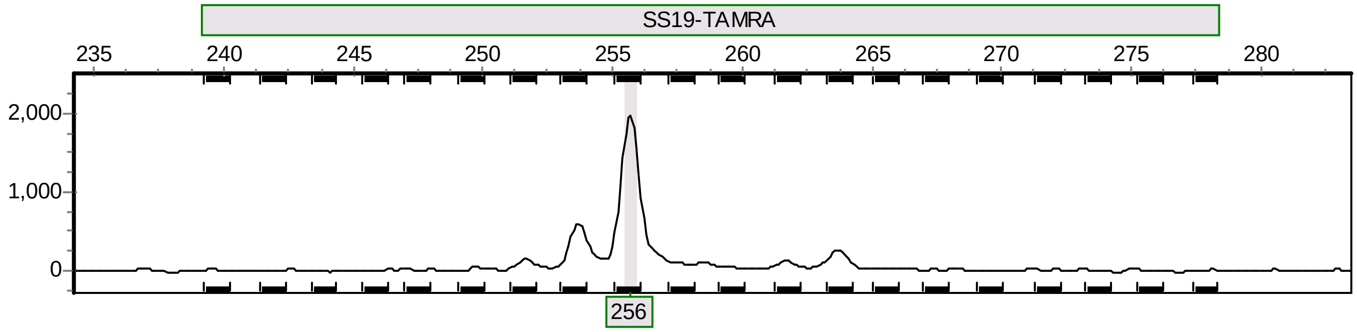


| No | Size | Height | Area | Marker | Allele | Difference | Quality | Score | Allele Comments | Sample Comments |
|---|---|---|---|---|---|---|---|---|---|---|
| 1 | 255.7 | 1975 | 15639 | SS19-TAMRA | 256 | 0.10 | Pass | 243.0 | [<Confirmed>] | |

**Sample 103:** SSS13_SS20_SS11_SS21_SS02_SS19_HRS30_P05.fsa

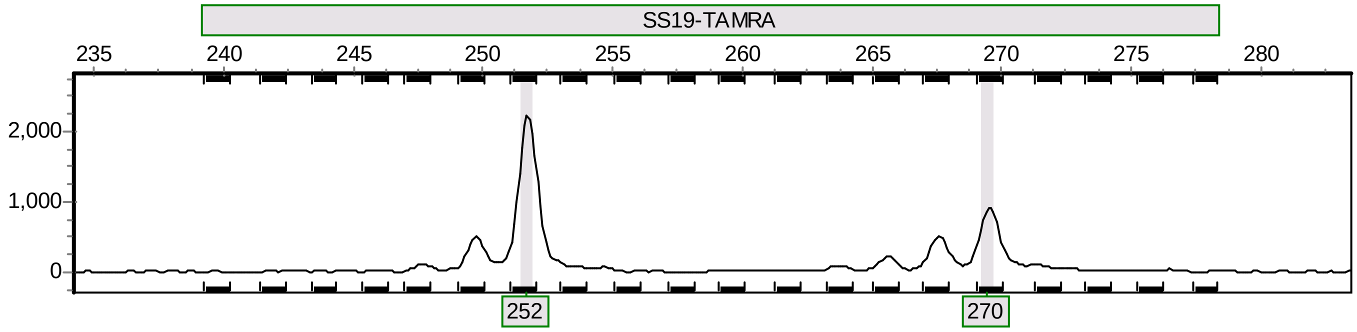


| No | Size | Height | Area | Marker | Allele | Difference | Quality | Score | Allele Comments | Sample Comments |
|---|---|---|---|---|---|---|---|---|---|---|
| 1 | 251.7 | 2213 | 18189 | SS19-TAMRA | 252 | 0.10 | Pass | 281.2 | [<Confirmed>] | |
| 2 | 269.5 | 914 | 8164 | SS19-TAMRA | 270 | 0.10 | Pass | 61.9 | [<Confirmed>] | |

**Sample 104:** SSS13_SS20_SS11_SS21_SS02_SS19_HRS31_J11.fsa

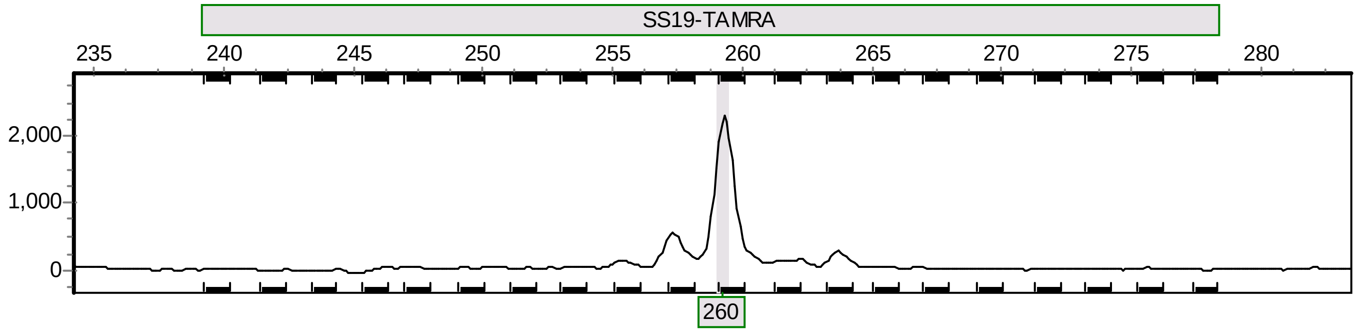


| No | Size | Height | Area | Marker | Allele | Difference | Quality | Score | Allele Comments | Sample Comments |
|---|---|---|---|---|---|---|---|---|---|---|
| 1 | 259.3 | 2285 | 18764 | SS19-TAMRA | 260 | 0.30 | Pass | 290.2 | [<Confirmed>] | |

**Sample 105:** SSS13_SS20_SS11_SS21_SS02_SS19_HRS33_H07.fsa

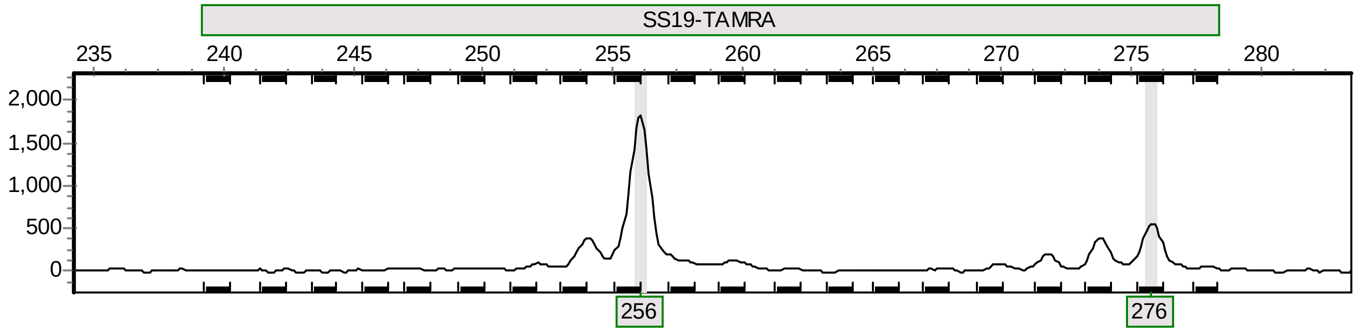


| No | Size | Height | Area | Marker | Allele | Difference | Quality | Score | Allele Comments | Sample Comments |
|---|---|---|---|---|---|---|---|---|---|---|
| 1 | 256.1 | 1808 | 15569 | SS19-TAMRA | 256 | 0.50 | Pass | 186.4 | [<Confirmed>] | |
| 2 | 275.8 | 561 | 4832 | SS19-TAMRA | 276 | 0.00 | Pass | 28.8 | [<Confirmed>] | |

**Sample 106:** SSS13_SS20_SS11_SS21_SS02_SS19_HRS34_L09.fsa

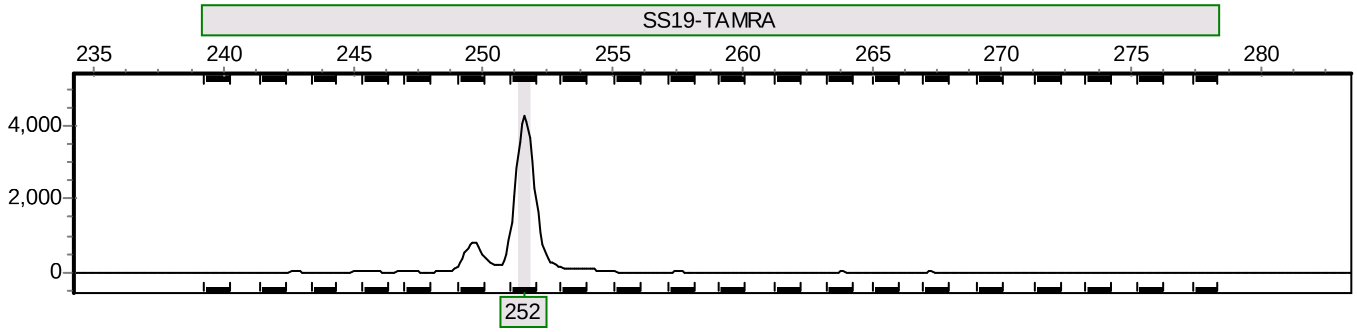


| No | Size | Height | Area | Marker | Allele | Difference | Quality | Score | Allele Comments | Sample Comments |
|---|---|---|---|---|---|---|---|---|---|---|
| 1 | 251.6 | 4235 | 34502 | SS19-TAMRA | 252 | 0.00 | Pass | 500.0 | [<Confirmed>] | |

# Allele Report



**Sample 107:** SSS13_SS20_SS11_SS21_SS02_SS19_HRS35_P09.fsa

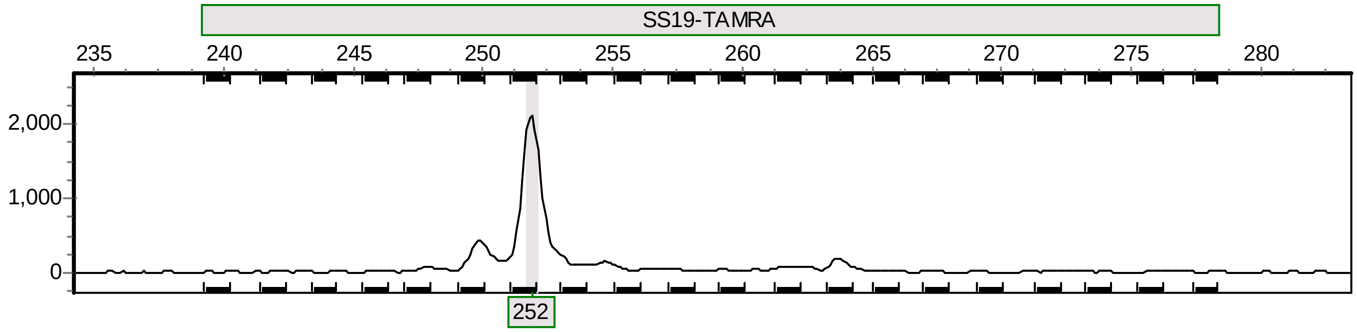


| No | Size | Height | Area | Marker | Allele | Difference | Quality | Score | Allele Comments | Sample Comments |
|---|---|---|---|---|---|---|---|---|---|---|
| 1 | 251.9 | 2085 | 16959 | SS19-TAMRA | 252 | 0.30 | Pass | 246.7 | [<Confirmed>] | |

**Sample 108:** SSS13_SS20_SS11_SS21_SS02_SS19_HRS37_J13.fsa

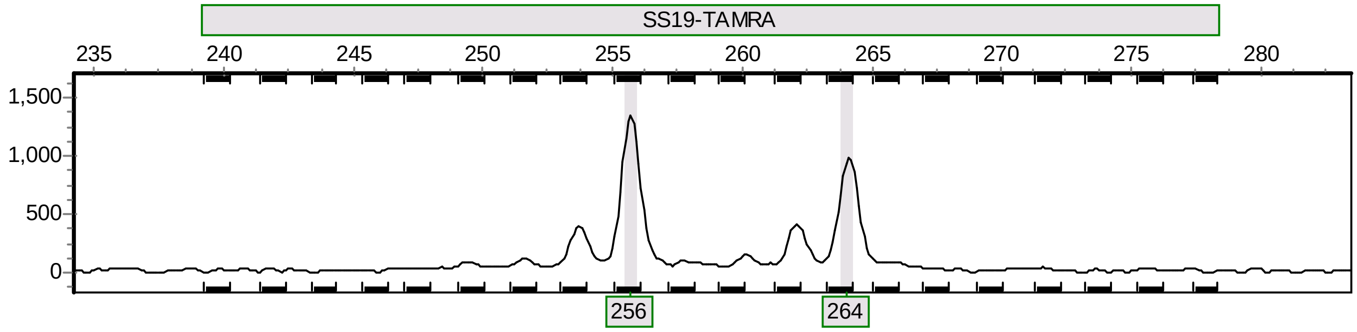


| No | Size | Height | Area | Marker | Allele | Difference | Quality | Score | Allele Comments | Sample Comments |
|---|---|---|---|---|---|---|---|---|---|---|
| 1 | 255.7 | 1333 | 11242 | SS19-TAMRA | 256 | 0.10 | Pass | 127.9 | [<Confirmed>] | |
| 2 | 264.1 | 986 | 8538 | SS19-TAMRA | 264 | 0.30 | Pass | 78.2 | [<Confirmed>] | |

**Sample 109:** SSS13_SS20_SS11_SS21_SS02_SS19_HRS38_J07.fsa

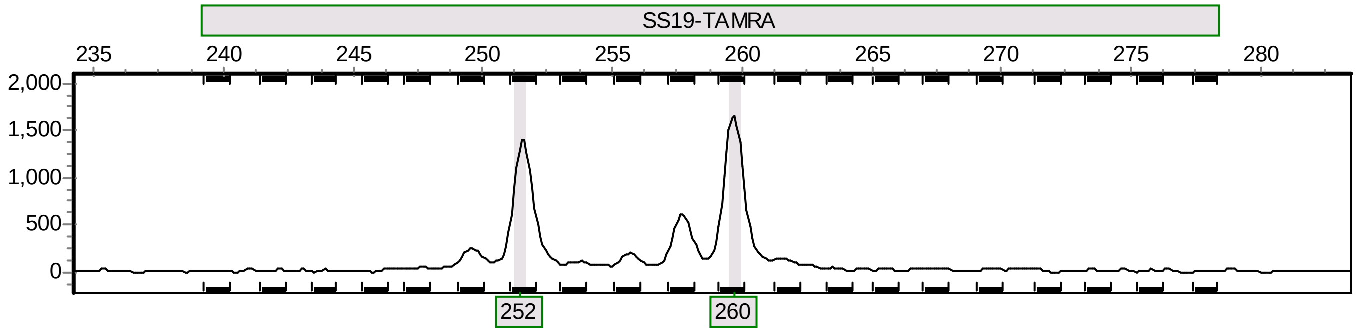


| No | Size | Height | Area | Marker | Allele | Difference | Quality | Score | Allele Comments | Sample Comments |
|---|---|---|---|---|---|---|---|---|---|---|
| 1 | 251.5 | 1397 | 11922 | SS19-TAMRA | 252 | 0.10 | Pass | 137.0 | [<Confirmed>] | |
| 2 | 259.7 | 1642 | 14390 | SS19-TAMRA | 260 | 0.10 | Pass | 151.8 | [<Confirmed>] | |

# Allele Report

**Sample 110:** SSS13_SS20_SS11_SS21_SS02_SS19_HRS39_N07.fsa

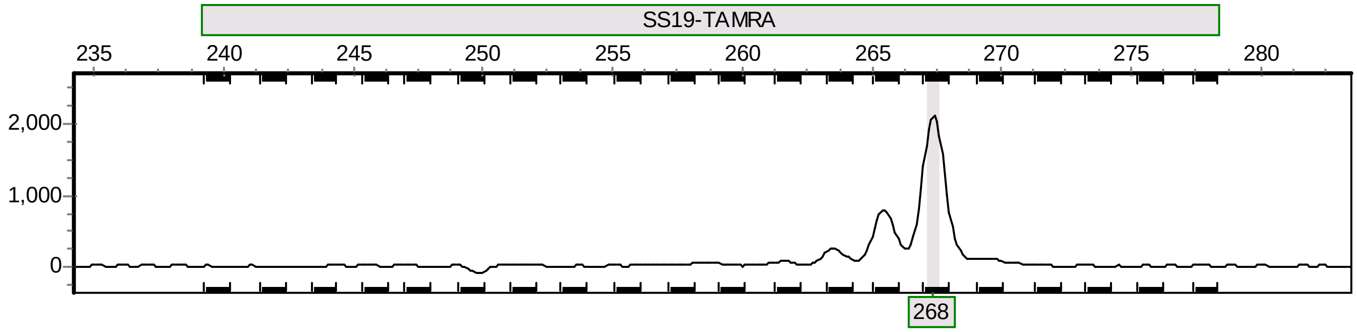


| No | Size | Height | Area | Marker | Allele | Difference | Quality | Score | Allele Comments | Sample Comments |
|---|---|---|---|---|---|---|---|---|---|---|
| 1 | 267.4 | 2110 | 21149 | SS19-TAMRA | 268 | 0.10 | Pass | 168.9 | [<Confirmed>] | |

**Sample 111:** SSS13_SS20_SS11_SS21_SS02_SS19_HRS40_A07.fsa

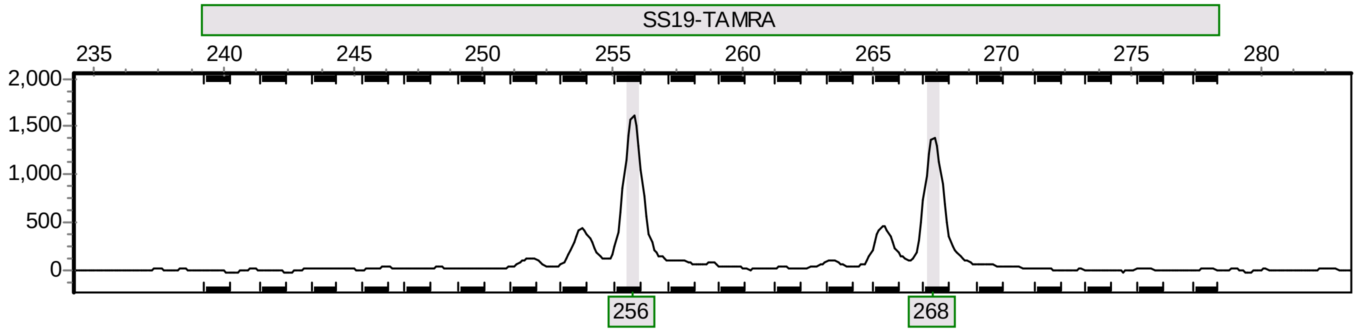


| No | Size | Height | Area | Marker | Allele | Difference | Quality | Score | Allele Comments | Sample Comments |
|---|---|---|---|---|---|---|---|---|---|---|
| 1 | 255.8 | 1607 | 12779 | SS19-TAMRA | 256 | 0.20 | Pass | 179.3 | [<Confirmed>] | |
| 2 | 267.4 | 1386 | 10925 | SS19-TAMRA | 268 | 0.10 | Pass | 143.5 | [<Confirmed>] | |

**Sample 112:** SSS13_SS20_SS11_SS21_SS02_SS19_HRS41_K07.fsa

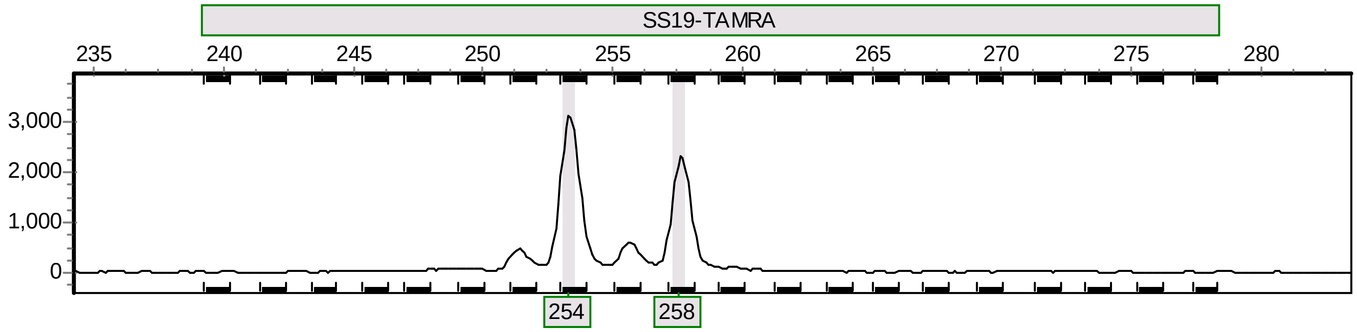


| No | Size | Height | Area | Marker | Allele | Difference | Quality | Score | Allele Comments | Sample Comments |
|---|---|---|---|---|---|---|---|---|---|---|
| 1 | 253.3 | 3100 | 26210 | SS19-TAMRA | 254 | 0.20 | Pass | 425.1 | [<Confirmed>] | |
| 2 | 257.6 | 2308 | 19064 | SS19-TAMRA | 258 | 0.10 | Pass | 293.1 | [<Confirmed>] | |

# Allele Report



**Sample 113:** SSS13_SS20_SS11_SS21_SS02_SS19_HRS42_I07.fsa

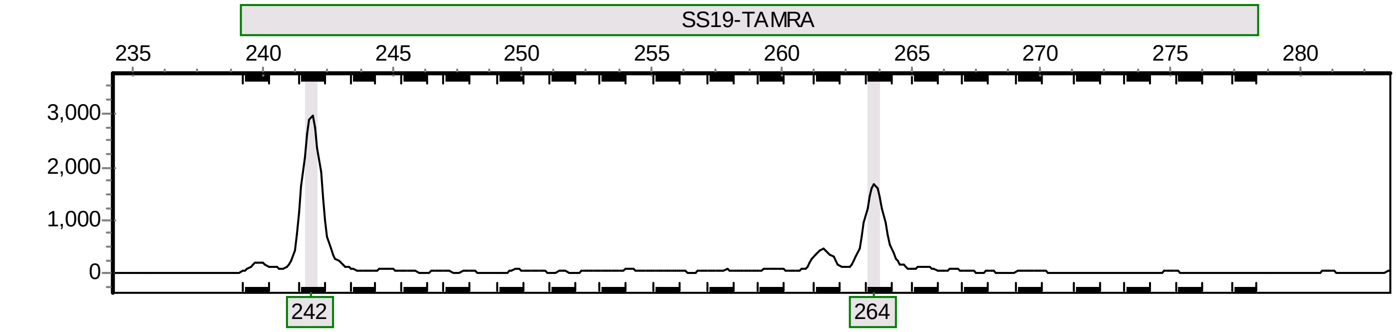


| No | Size | Height | Area | Marker | Allele | Difference | Quality | Score | Allele Comments | Sample Comments |
|---|---|---|---|---|---|---|---|---|---|---|
| 1 | 241.9 | 2963 | 23509 | SS19-TAMRA | 242 | 0.00 | Pass | 424.6 | [<Confirmed>] | |
| 2 | 263.6 | 1676 | 14716 | SS19-TAMRA | 264 | 0.20 | Pass | 157.4 | [<Confirmed>] | |

**Sample 114:** SSS13_SS20_SS11_SS21_SS02_SS19_HTHL11_M11.fsa

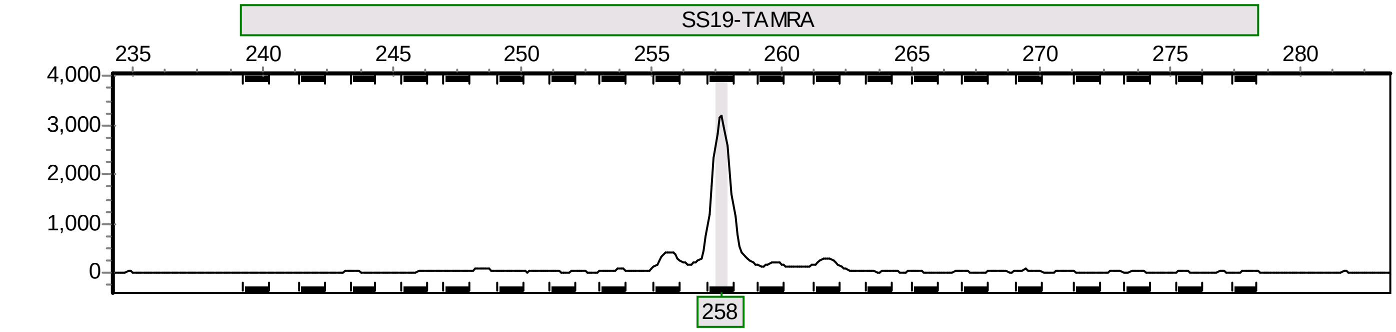


| No | Size | Height | Area | Marker | Allele | Difference | Quality | Score | Allele Comments | Sample Comments |
|---|---|---|---|---|---|---|---|---|---|---|
| 1 | 257.7 | 3184 | 26424 | SS19-TAMRA | 258 | 0.00 | Pass | 455.2 | [<Confirmed>] | |

**Sample 115:** SSS13_SS20_SS11_SS21_SS02_SS19_HTHL13_H01.fsa

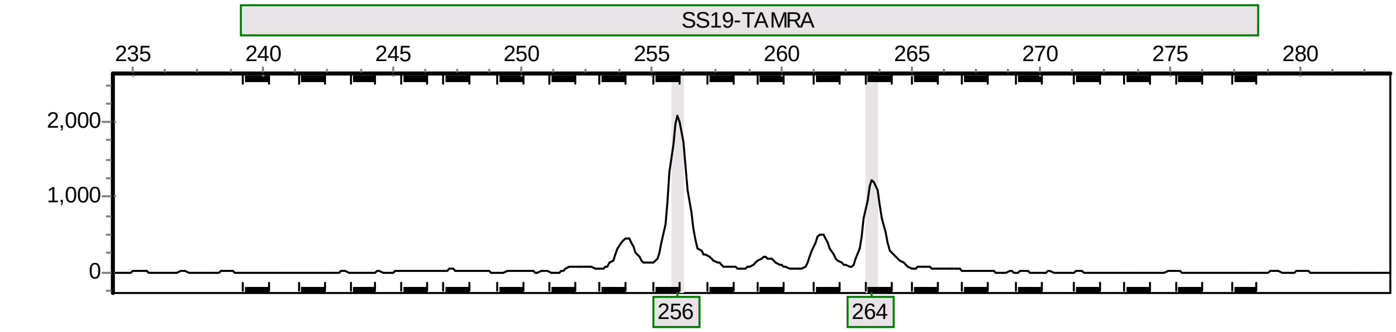


| No | Size | Height | Area | Marker | Allele | Difference | Quality | Score | Allele Comments | Sample Comments |
|---|---|---|---|---|---|---|---|---|---|---|
| 1 | 256.0 | 2063 | 16390 | SS19-TAMRA | 256 | 0.40 | Pass | 275.3 | [<Confirmed>] | |
| 2 | 263.5 | 1225 | 9703 | SS19-TAMRA | 264 | 0.30 | Pass | 125.7 | [<Confirmed>] | |

**Sample 116:** SSS13_SS20_SS11_SS21_SS02_SS19_HTHL14_F15.fsa

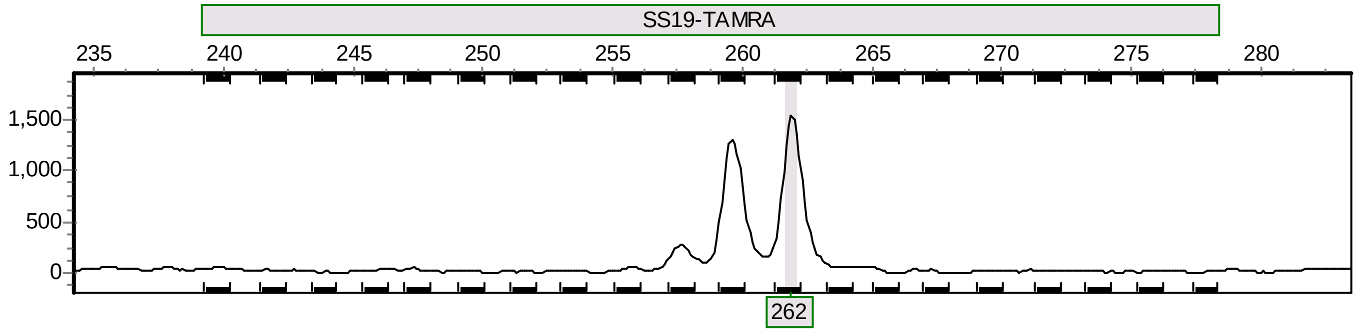

| No | Size | Height | Area | Marker | Allele | Difference | Quality | Score | Allele Comments | Sample Comments |
|---|---|---|---|---|---|---|---|---|---|---|
| 1 | 261.9 | 1530 | 13269 | SS19-TAMRA | 262 | 0.10 | Pass | 155.9 | [<Confirmed>] | |

**Sample 117:** SSS13_SS20_SS11_SS21_SS02_SS19_HTHL15_F01.fsa

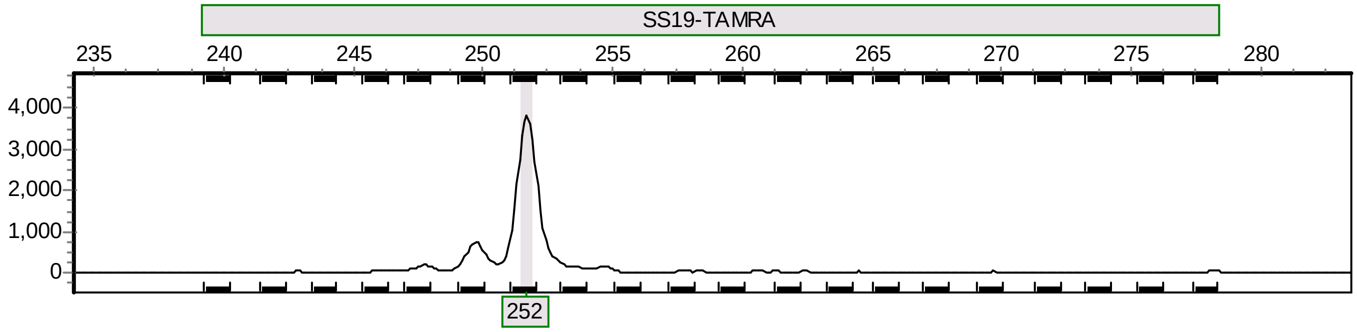

| No | Size | Height | Area | Marker | Allele | Difference | Quality | Score | Allele Comments | Sample Comments |
|---|---|---|---|---|---|---|---|---|---|---|
| 1 | 251.7 | 3788 | 32402 | SS19-TAMRA | 252 | 0.10 | Pass | 500.0 | [<Confirmed>] | |

**Sample 118:** SSS13_SS20_SS11_SS21_SS02_SS19_HTHL1_E13.fsa

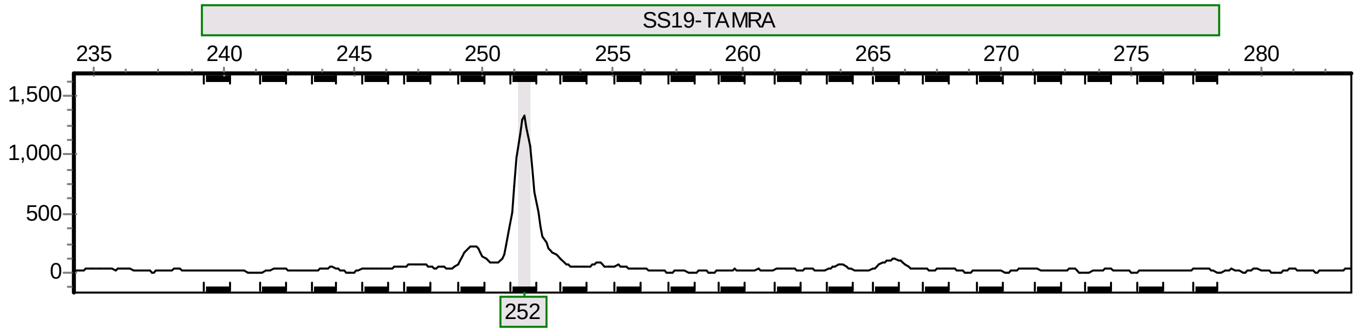

| No | Size | Height | Area | Marker | Allele | Difference | Quality | Score | Allele Comments | Sample Comments |
|---|---|---|---|---|---|---|---|---|---|---|
| 1 | 251.6 | 1326 | 10748 | SS19-TAMRA | 252 | 0.00 | Pass | 126.1 | [<Confirmed>] | |

# Allele Report

**Sample 119:** SSS13_SS20_SS11_SS21_SS02_SS19_HTHL3_D05.fsa

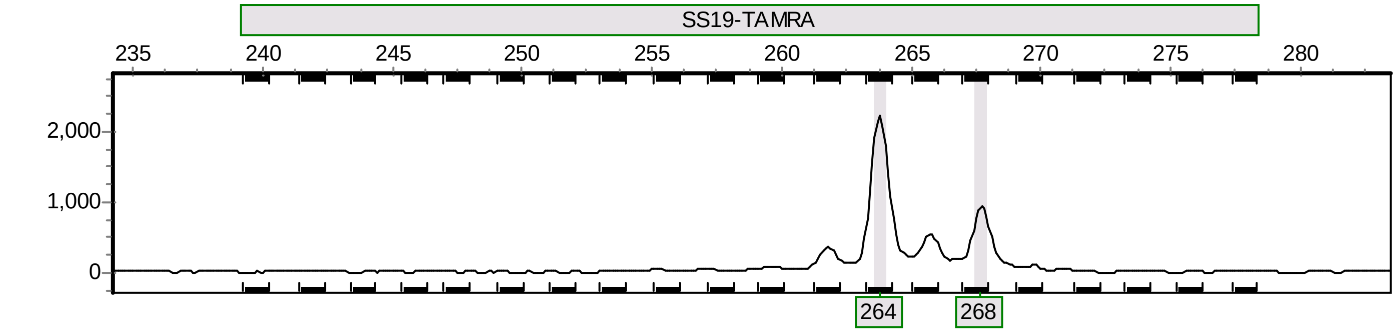


| No | Size | Height | Area | Marker | Allele | Difference | Quality | Score | Allele Comments | Sample Comments |
|---|---|---|---|---|---|---|---|---|---|---|
| 1 | 263.8 | 2232 | 17283 | SS19-TAMRA | 264 | 0.00 | Pass | 301.2 | [<Confirmed>] | |
| 2 | 267.7 | 936 | 7469 | SS19-TAMRA | 268 | 0.20 | Pass | 79.0 | [<Confirmed>] | |

**Sample 120:** SSS13_SS20_SS11_SS21_SS02_SS19_HTHL4_O15.fsa

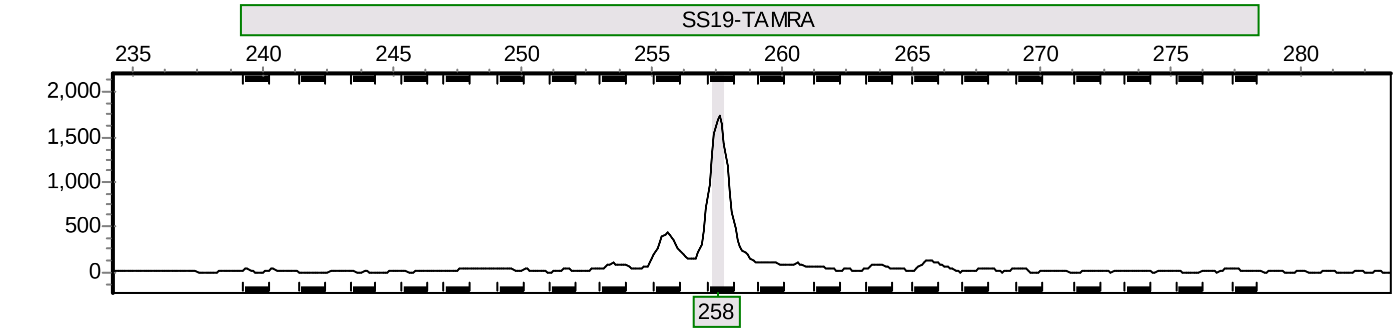


| No | Size | Height | Area | Marker | Allele | Difference | Quality | Score | Allele Comments | Sample Comments |
|---|---|---|---|---|---|---|---|---|---|---|
| 1 | 257.6 | 1730 | 14722 | SS19-TAMRA | 258 | 0.10 | Pass | 180.9 | [<Confirmed>] | |

**Sample 121:** SSS13_SS20_SS11_SS21_SS02_SS19_HTHL5_M13.fsa

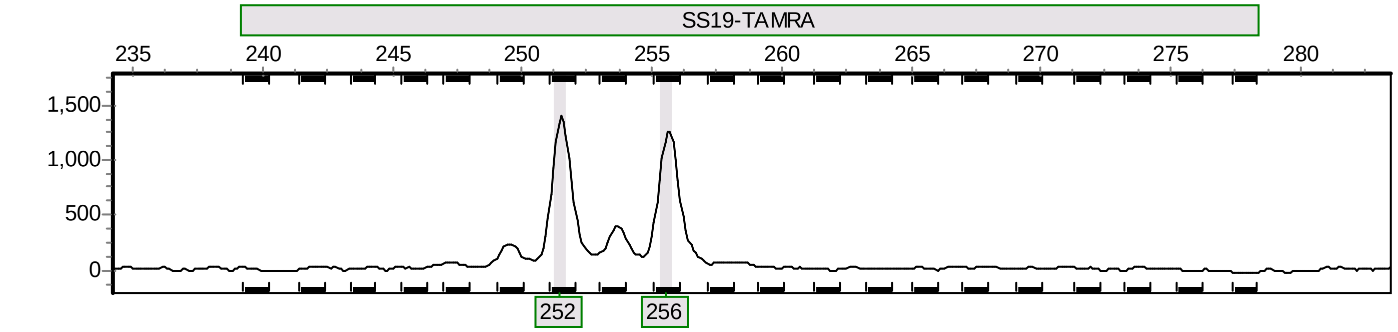


| No | Size | Height | Area | Marker | Allele | Difference | Quality | Score | Allele Comments | Sample Comments |
|---|---|---|---|---|---|---|---|---|---|---|
| 1 | 251.5 | 1393 | 11627 | SS19-TAMRA | 252 | 0.10 | Pass | 134.2 | [<Confirmed>] | |
| 2 | 255.6 | 1249 | 11270 | SS19-TAMRA | 256 | 0.00 | Pass | 100.0 | [<Confirmed>] | |

# Allele Report



**Sample 122:** SSS13_SS20_SS11_SS21_SS02_SS19_HTHL6_P03.fsa

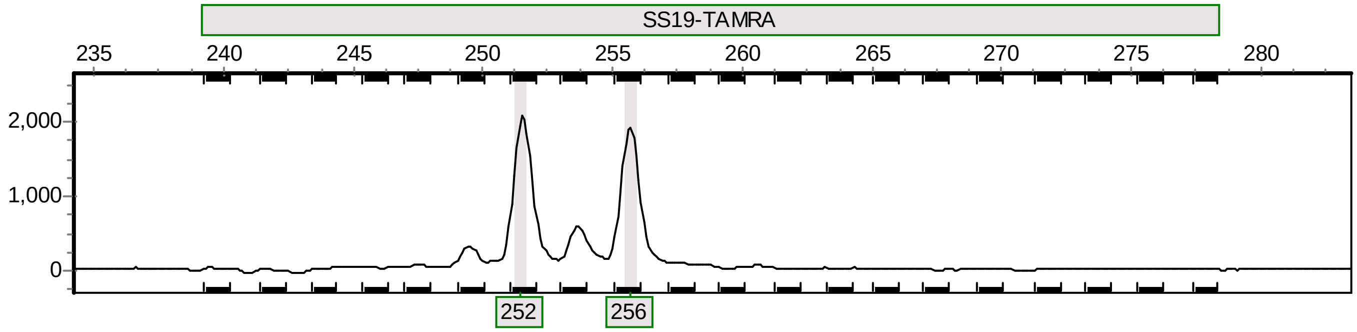


| No | Size | Height | Area | Marker | Allele | Difference | Quality | Score | Allele Comments | Sample Comments |
|---|---|---|---|---|---|---|---|---|---|---|
| 1 | 251.5 | 2077 | 16520 | SS19-TAMRA | 252 | 0.10 | Pass | 267.6 | [<Confirmed>] | |
| 2 | 255.7 | 1919 | 15107 | SS19-TAMRA | 256 | 0.10 | Pass | 235.8 | [<Confirmed>] | |

**Sample 123:** SSS13_SS20_SS11_SS21_SS02_SS19_HTHL8_A03.fsa

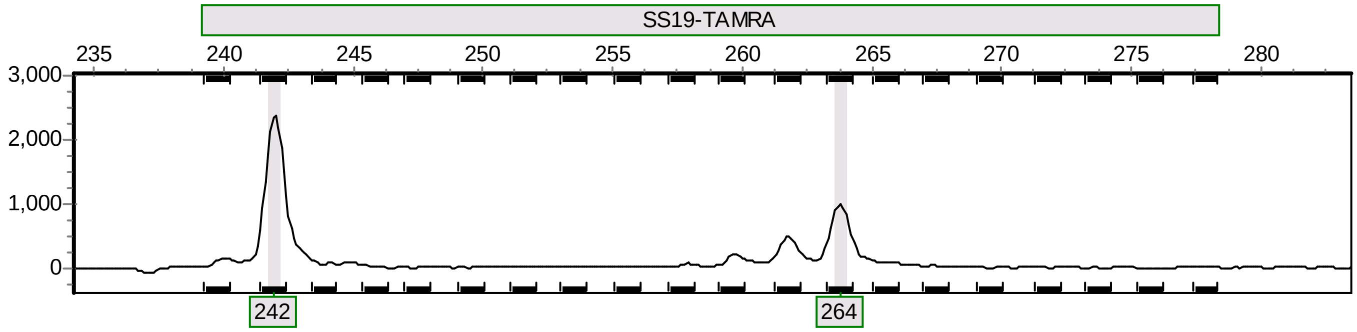


| No | Size | Height | Area | Marker | Allele | Difference | Quality | Score | Allele Comments | Sample Comments |
|---|---|---|---|---|---|---|---|---|---|---|
| 1 | 242.0 | 2360 | 18802 | SS19-TAMRA | 242 | 0.10 | Pass | 305.7 | [<Confirmed>] | |
| 2 | 263.8 | 998 | 8835 | SS19-TAMRA | 264 | 0.00 | Pass | 71.4 | [<Confirmed>] | |

**Sample 124:** SSS13_SS20_SS11_SS21_SS02_SS19_HTHL9_O09.fsa

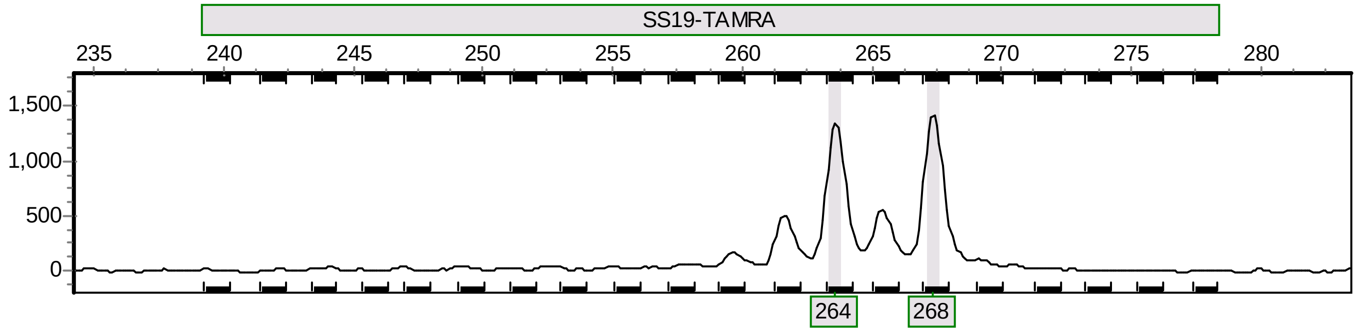


| No | Size | Height | Area | Marker | Allele | Difference | Quality | Score | Allele Comments | Sample Comments |
|---|---|---|---|---|---|---|---|---|---|---|
| 1 | 263.6 | 1340 | 11274 | SS19-TAMRA | 264 | 0.20 | Pass | 123.0 | [<Confirmed>] | |
| 2 | 267.4 | 1403 | 12068 | SS19-TAMRA | 268 | 0.10 | Pass | 126.1 | [<Confirmed>] | |
