# Supplementary material for: Genetic diversity of Schima superba based on physiological traits and SSR markers
Source: PLoS One. 2026 Apr 10;21(4):e0344465. doi: 10.1371/journal.pone.0344465 (PMC13068225; doi:10.1371/journal.pone.0344465)
Supplement: S1 File — (ZIP) [file pone.0344465.s003.zip › SS20.pdf]

SoftGenetics

Allele Report

10/11/2024 11:09:34 AM

GeneMarker V3.0.0

Project Comments:

Sample 1: SSS13_SS20_SS11_SS21_SS02_SS19_HBB10_E05.fsa

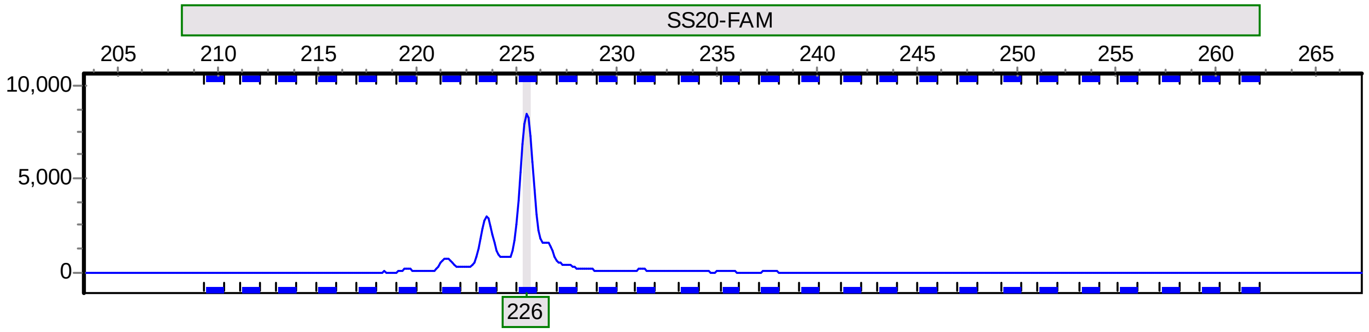

No	Size	Height	Area	Marker	Allele	Difference	Quality	Score	Allele Comments	Sample Comments
1	134.5	13602	64030	SSS13-FAM	135	1.00	Pass	500.0	[<Confirmed><Edited>]	
2	137.4	10653	52619	SSS13-FAM	137	0.10	Pass	500.0	[<Deleted>]	
3	138.9	24773	120533	SSS13-FAM	139	0.60	Pass	500.0	[<Confirmed>]	
4	225.5	8506	67423	SS20-FAM	226	0.00	Pass	500.0	[<Confirmed>]	

Sample 2: SSS13_SS20_SS11_SS21_SS02_SS19_HBB12-2_D11.fsa

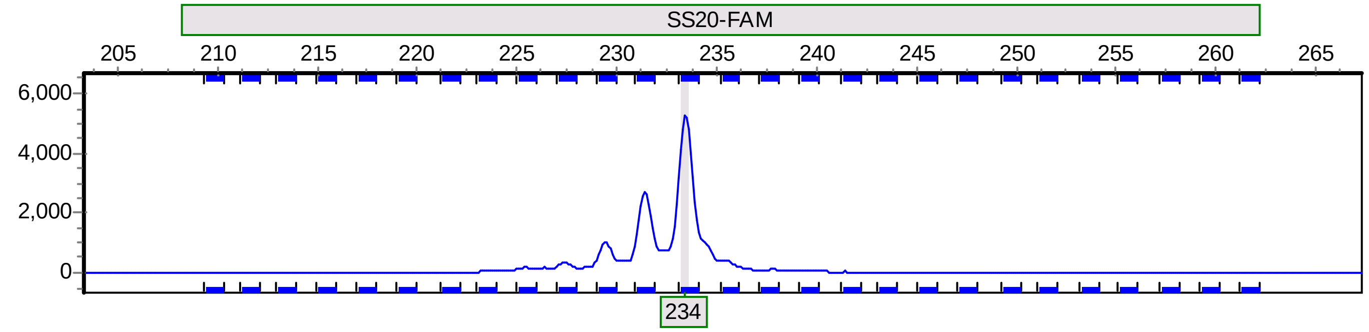

No	Size	Height	Area	Marker	Allele	Difference	Quality	Score	Allele Comments	Sample Comments
1	126.9	32524	242375	SSS13-FAM	127	0.30	Pass	500.0	[<SAT (Repaired)><Confirmed>]	
2	128.9	12866	87149	SSS13-FAM	129	0.40	Pass	500.0	[<Confirmed>]	
3	233.4	5215	41995	SS20-FAM	234	0.20	Pass	500.0	[<Confirmed>]	

Sample 3: SSS13_SS20_SS11_SS21_SS02_SS19_HBB13_C17.fsa

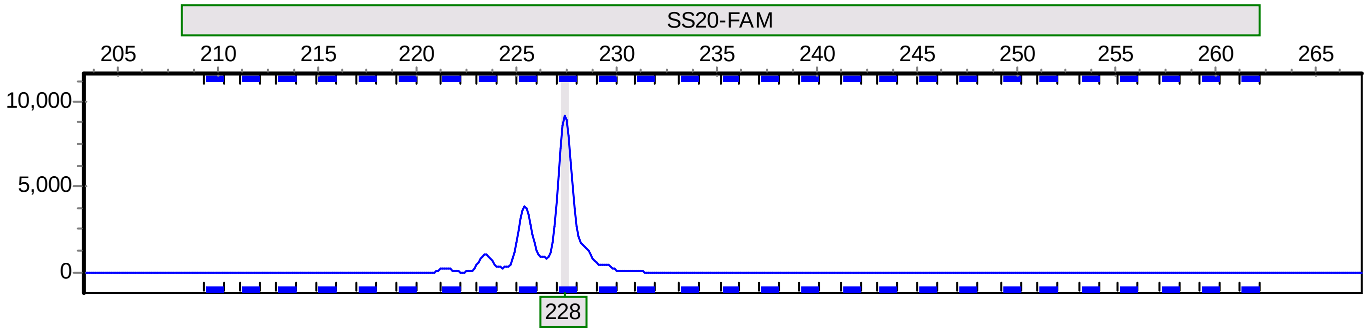

No	Size	Height	Area	Marker	Allele	Difference	Quality	Score	Allele Comments	Sample Comments
1	129.2	31339	254805	SSS13-FAM	129	0.10	Pass	500.0	[<Confirmed>]	
2	227.4	9089	72704	SS20-FAM	228	0.10	Pass	500.0	[<Confirmed>]	

Sample 4: SSS13_SS20_SS11_SS21_SS02_SS19_HBB14_G03.fsa

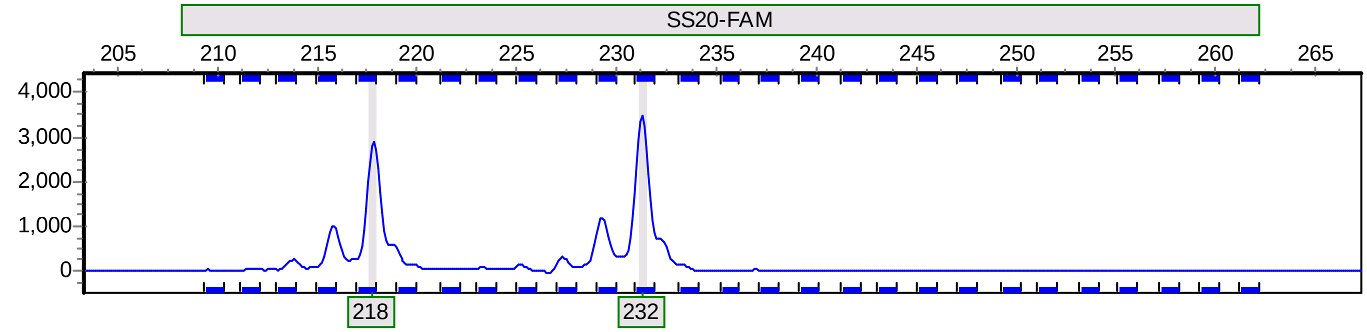

No	Size	Height	Area	Marker	Allele	Difference	Quality	Score	Allele Comments	Sample Comments
1	128.9	20122	144317	SSS13-FAM	129	0.40	Pass	500.0	[<Confirmed>]	
2	131.2	16550	107368	SSS13-FAM	131	0.10	Pass	500.0	[<Confirmed>]	
3	217.8	2883	21715	SS20-FAM	218	0.30	Pass	466.1	[<Confirmed>]	
4	231.3	3459	26292	SS20-FAM	232	0.10	Pass	500.0	[<Confirmed>]	

Sample 5: SSS13_SS20_SS11_SS21_SS02_SS19_HBB15_B07.fsa

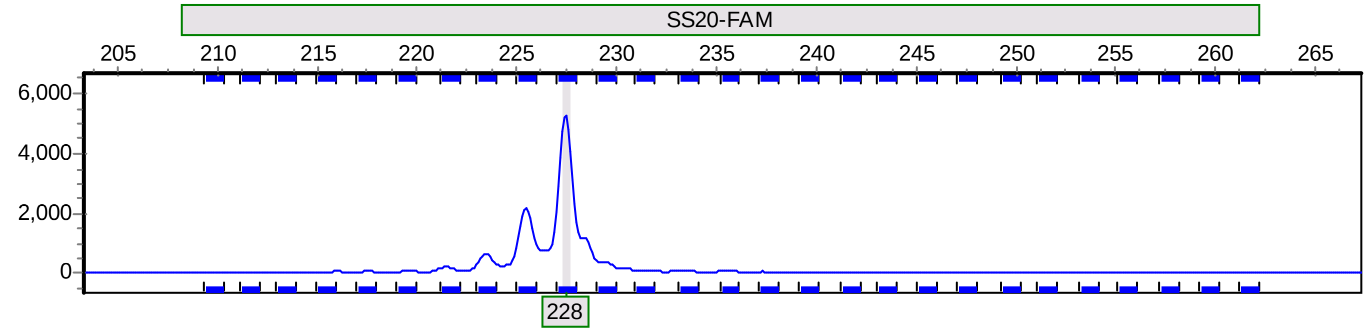

No	Size	Height	Area	Marker	Allele	Difference	Quality	Score	Allele Comments	Sample Comments
1	129.3	31559	230028	SSS13-FAM	129	0.00	Pass	500.0	[<Confirmed>]	
2	227.5	5261	41625	SS20-FAM	228	0.00	Pass	500.0	[<Confirmed>]	

Sample 6: SSS13_SS20_SS11_SS21_SS02_SS19_HBB16_G07.fsa

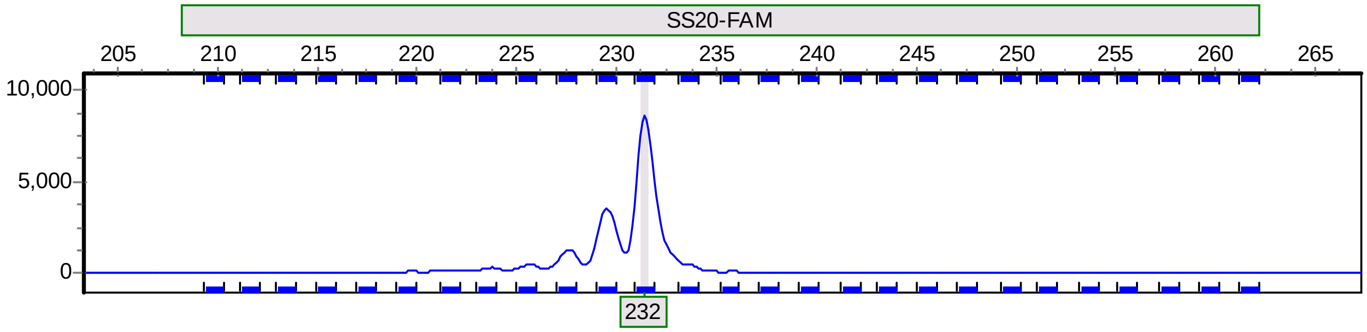

No	Size	Height	Area	Marker	Allele	Difference	Quality	Score	Allele Comments	Sample Comments
1	129.0	28180	188036	SSS13-FAM	129	0.30	Pass	500.0	[<Confirmed>]	
2	139.8	16963	129615	SSS13-FAM	139	0.30	Pass	500.0	[<Confirmed>]	
3	231.4	8521	85896	SS20-FAM	232	0.00	Pass	500.0	[<Confirmed>]	

Allele Report

Sample 7: SSS13_SS20_SS11_SS21_SS02_SS19_HBB17_K15.fsa

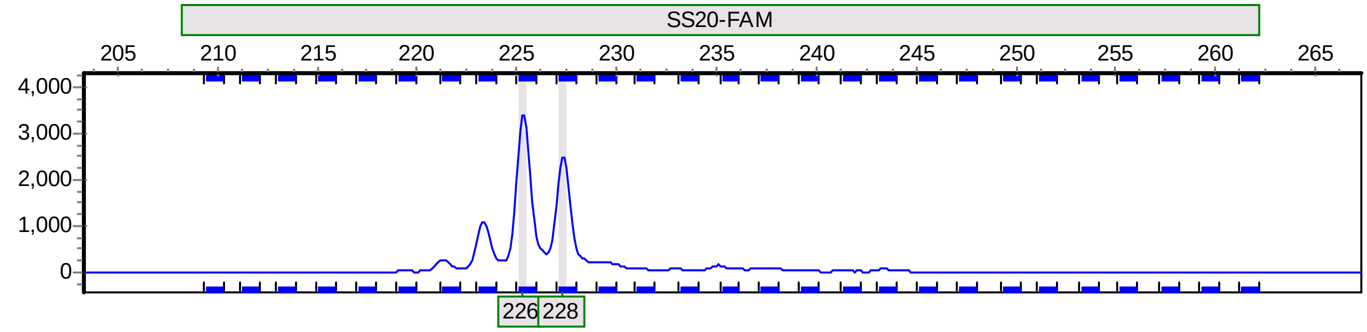

No	Size	Height	Area	Marker	Allele	Difference	Quality	Score	Allele Comments	Sample Comments
1	123.3	16081	106815	SSS13-FAM	123	0.10	Pass	500.0	[<Confirmed>]	
2	225.3	3371	27744	SS20-FAM	226	0.20	Pass	500.0	[<Confirmed>]	
3	227.3	2497	20076	SS20-FAM	228	0.20	Pass	357.3	[<Confirmed>]	

Sample 8: SSS13_SS20_SS11_SS21_SS02_SS19_HBB18_O05.fsa

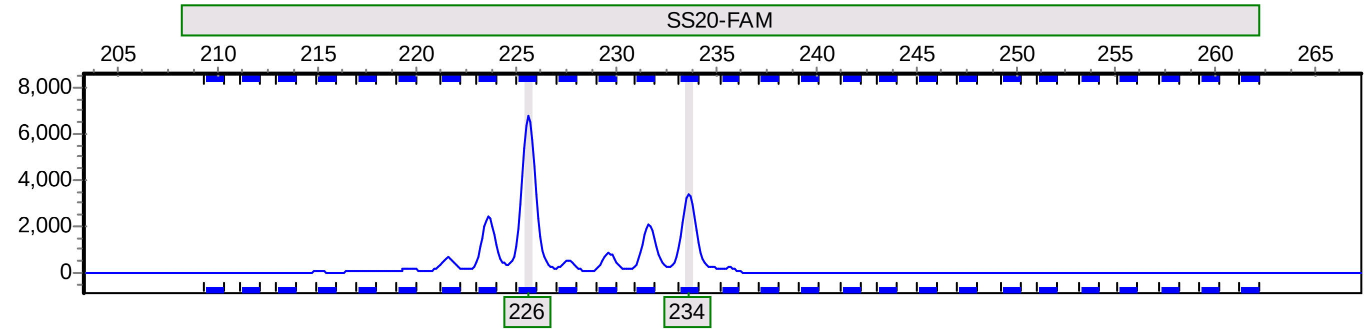

No	Size	Height	Area	Marker	Allele	Difference	Quality	Score	Allele Comments	Sample Comments
1	137.5	28289	189245	SSS13-FAM	137	0.00	Pass	500.0	[<Confirmed>]	
2	225.6	6761	51932	SS20-FAM	226	0.10	Pass	500.0	[<Confirmed>]	
3	233.6	3404	27099	SS20-FAM	234	0.00	Pass	500.0	[<Confirmed>]	

Sample 9: SSS13_SS20_SS11_SS21_SS02_SS19_HBB19_O07.fsa

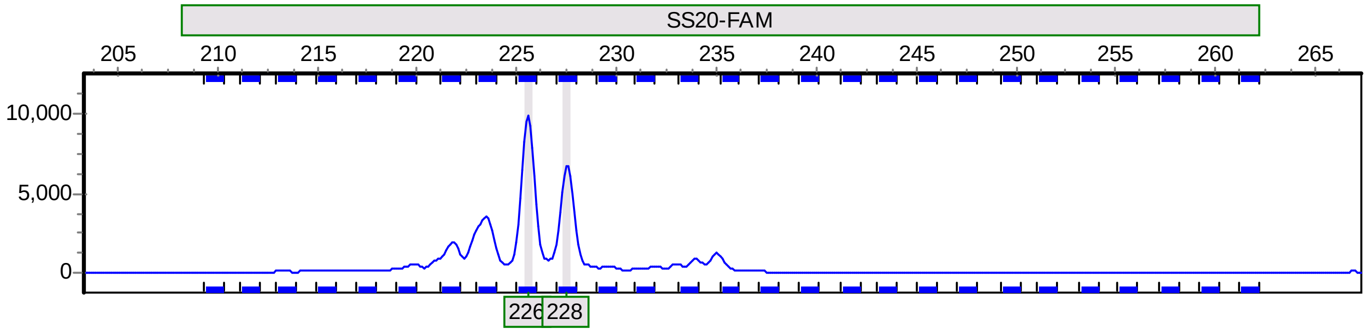

No	Size	Height	Area	Marker	Allele	Difference	Quality	Score	Allele Comments	Sample Comments
1	127.2	5674	37811	SSS13-FAM	127	0.00	Pass	500.0	[<Confirmed>]	
2	225.6	9825	73475	SS20-FAM	226	0.10	Pass	500.0	[<Confirmed>]	
3	227.5	6752	52378	SS20-FAM	228	0.00	Pass	500.0	[<Confirmed>]	

Sample 10: SSS13_SS20_SS11_SS21_SS02_SS19_HBB1_B11.fsa

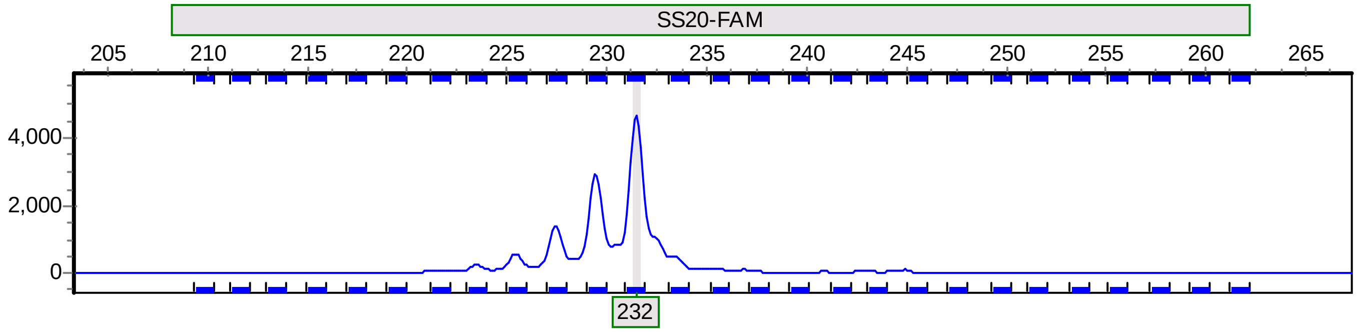

No	Size	Height	Area	Marker	Allele	Difference	Quality	Score	Allele Comments	Sample Comments
1	143.5	30976	228019	SSS13-FAM	143	0.30	Pass	500.0	[<Confirmed>]	
2	231.5	4645	36629	SS20-FAM	232	0.10	Pass	500.0	[<Confirmed>]	

Sample 11: SSS13_SS20_SS11_SS21_SS02_SS19_HBB20_F11.fsa

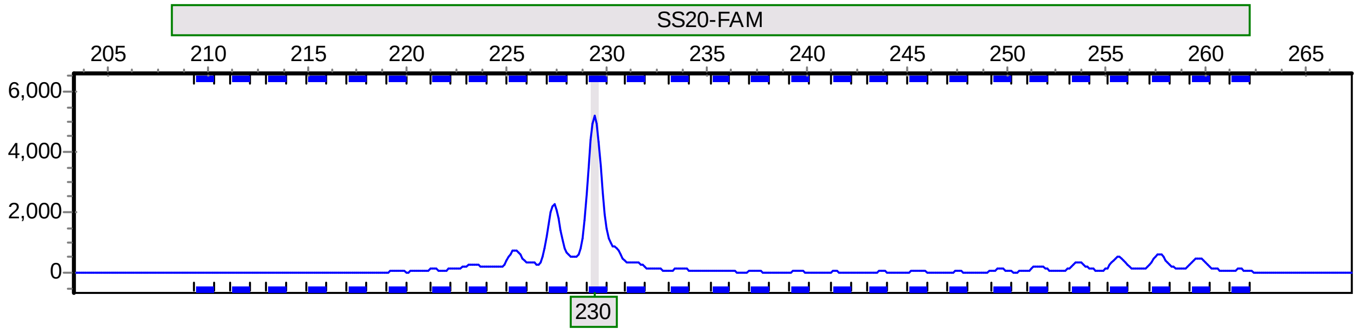

No	Size	Height	Area	Marker	Allele	Difference	Quality	Score	Allele Comments	Sample Comments
1	127.0	31132	218117	SSS13-FAM	127	0.20	Pass	500.0	[<Confirmed>]	
2	143.3	14003	104530	SSS13-FAM	143	0.50	Pass	500.0	[<Confirmed>]	
3	229.4	5189	42205	SS20-FAM	230	0.10	Pass	500.0	[<Confirmed>]	

Sample 12: SSS13_SS20_SS11_SS21_SS02_SS19_HBB21_J09.fsa

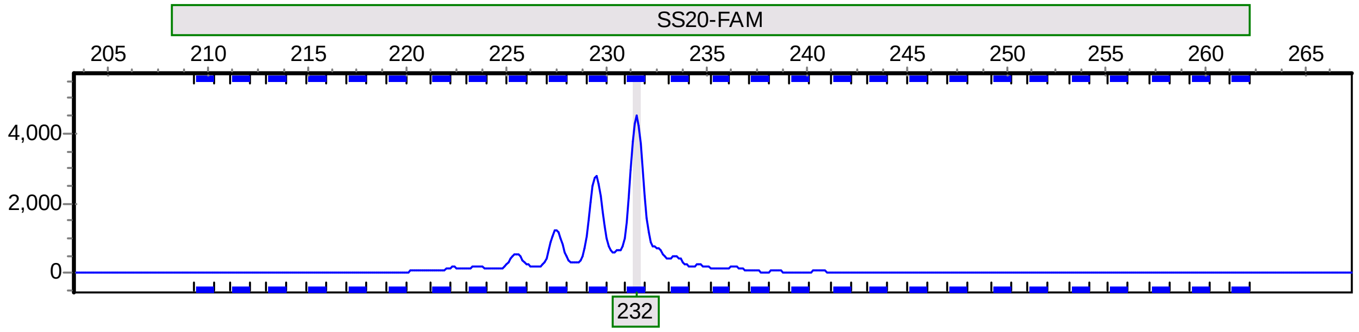

No	Size	Height	Area	Marker	Allele	Difference	Quality	Score	Allele Comments	Sample Comments
1	133.3	27303	185947	SSS13-FAM	133	0.10	Pass	500.0	[<Confirmed>]	
2	137.5	10733	72657	SSS13-FAM	137	0.00	Pass	500.0	[<Confirmed>]	
3	231.5	4492	34686	SS20-FAM	232	0.10	Pass	500.0	[<Confirmed>]	

Allele Report

Sample 13: SSS13_SS20_SS11_SS21_SS02_SS19_HBB22_K05.fsa

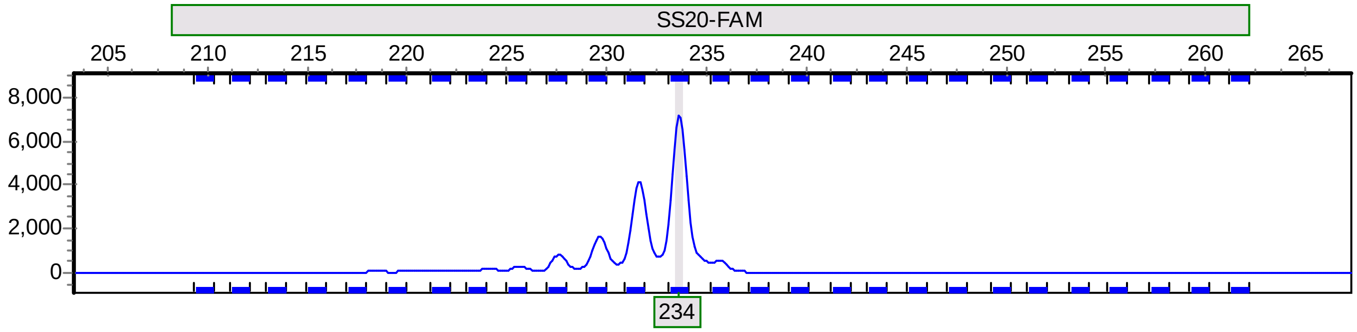

No	Size	Height	Area	Marker	Allele	Difference	Quality	Score	Allele Comments	Sample Comments
1	139.4	23810	178667	SSS13-FAM	139	0.10	Pass	500.0	[<Confirmed>]	
2	233.6	7117	59916	SS20-FAM	234	0.00	Pass	500.0	[<Confirmed>]	

Sample 14: SSS13_SS20_SS11_SS21_SS02_SS19_HBB23_B09.fsa

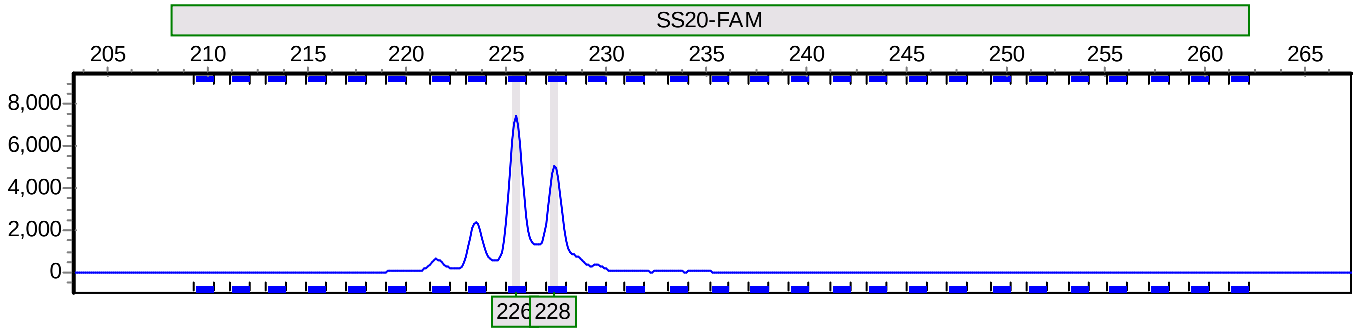

No	Size	Height	Area	Marker	Allele	Difference	Quality	Score	Allele Comments	Sample Comments
1	129.3	35045	258140	SSS13-FAM	129	0.00	Pass	500.0	[<SAT (Repaired)><Confirmed>]	
2	225.5	7348	56412	SS20-FAM	226	0.00	Pass	500.0	[<Confirmed>]	
3	227.4	4990	40266	SS20-FAM	228	0.10	Pass	500.0	[<Confirmed>]	

Sample 15: SSS13_SS20_SS11_SS21_SS02_SS19_HBB25-1_F09.fsa

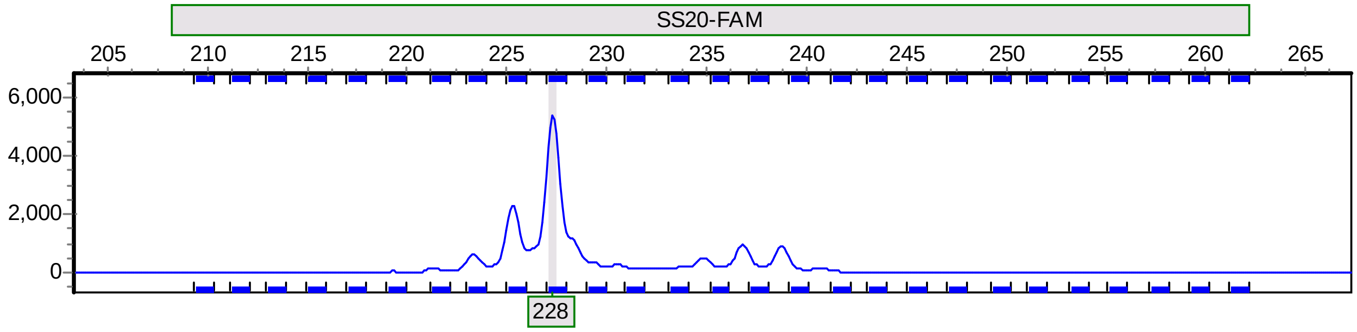

No	Size	Height	Area	Marker	Allele	Difference	Quality	Score	Allele Comments	Sample Comments
1	141.9	5162	41661	SSS13-FAM	141	0.40	Pass	500.0	[<Confirmed>]	
2	144.1	1982	16458	SSS13-FAM	143	0.30	Pass	173.9	[<Confirmed>]	
3	227.3	5366	42943	SS20-FAM	228	0.20	Pass	500.0	[<Confirmed>]	

Allele Report

Sample 16: SSS13_SS20_SS11_SS21_SS02_SS19_HBB25-2_N11.fsa

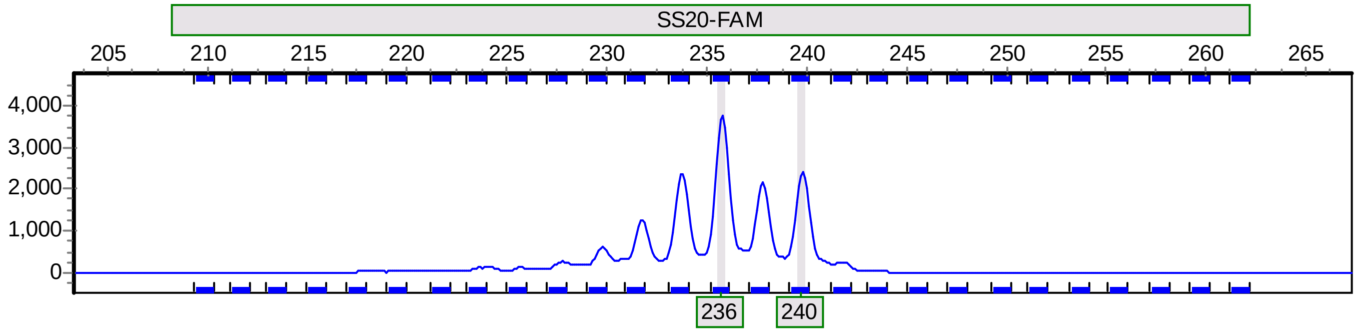

No	Size	Height	Area	Marker	Allele	Difference	Quality	Score	Allele Comments	Sample Comments
1	129.4	24675	174600	SSS13-FAM	129	0.10	Pass	500.0	[<Confirmed>]	
2	133.2	18726	127177	SSS13-FAM	133	0.20	Pass	500.0	[<Confirmed>]	
3	235.8	3733	29199	SS20-FAM	236	0.10	Pass	500.0	[<Confirmed>]	
4	239.8	2409	19937	SS20-FAM	240	0.10	Pass	325.8	[<Confirmed>]	

Sample 17: SSS13_SS20_SS11_SS21_SS02_SS19_HBB26_D07.fsa

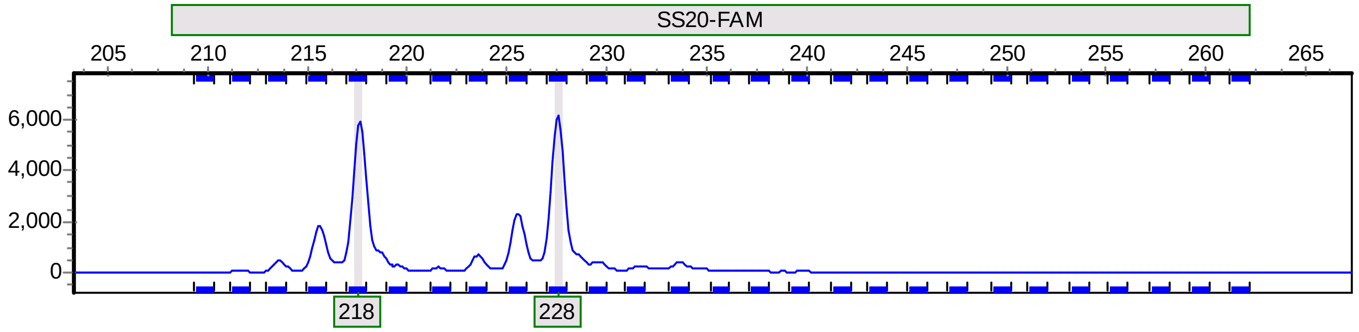

No	Size	Height	Area	Marker	Allele	Difference	Quality	Score	Allele Comments	Sample Comments
1	135.3	7326	51685	SSS13-FAM	135	0.10	Pass	500.0	[<Confirmed>]	
2	141.9	19341	144871	SSS13-FAM	141	0.40	Pass	500.0	[<Confirmed>]	
3	217.6	5881	44453	SS20-FAM	218	0.10	Pass	500.0	[<Confirmed>]	
4	227.6	6086	46435	SS20-FAM	228	0.10	Pass	500.0	[<Confirmed>]	

Sample 18: SSS13_SS20_SS11_SS21_SS02_SS19_HBB27_H05.fsa

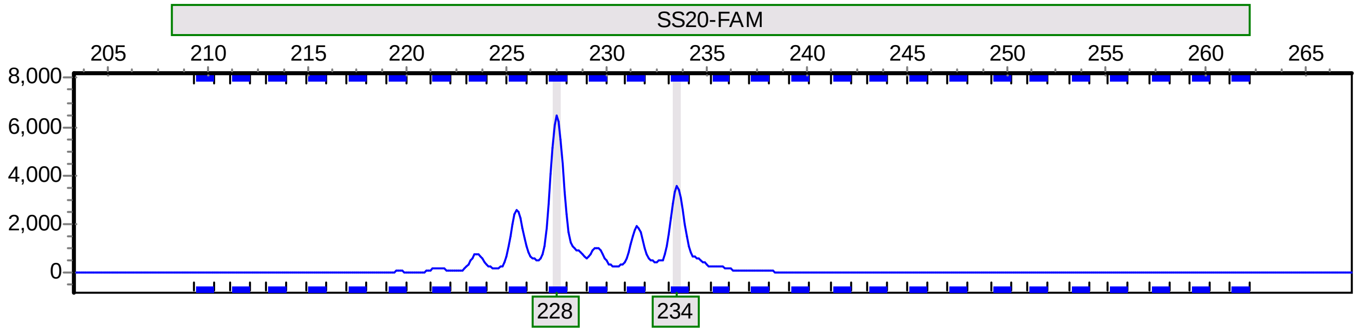

No	Size	Height	Area	Marker	Allele	Difference	Quality	Score	Allele Comments	Sample Comments
1	127.2	31575	261198	SSS13-FAM	127	0.00	Pass	500.0	[<Confirmed>]	
2	227.5	6431	50819	SS20-FAM	228	0.00	Pass	500.0	[<Confirmed>]	
3	233.5	3541	28065	SS20-FAM	234	0.10	Pass	500.0	[<Confirmed>]	

Sample 19: SSS13_SS20_SS11_SS21_SS02_SS19_HBB28_L11.fsa

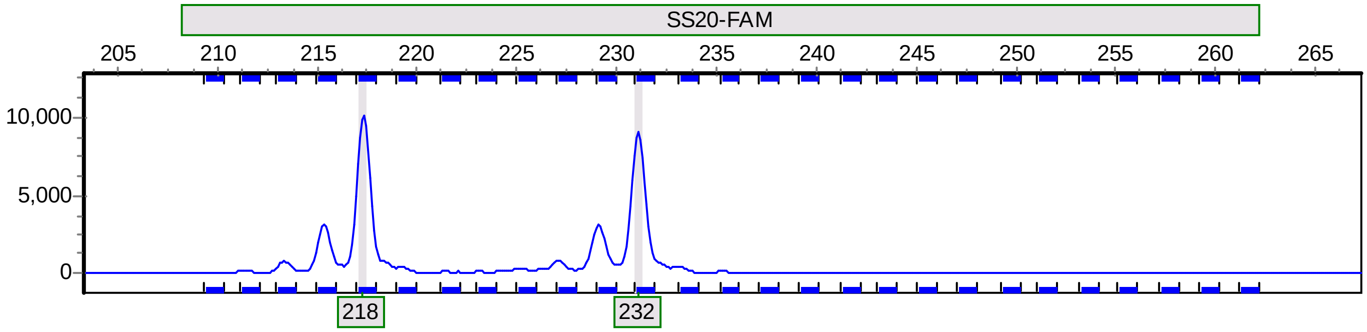

No	Size	Height	Area	Marker	Allele	Difference	Quality	Score	Allele Comments	Sample Comments
1	129.3	31507	270299	SSS13-FAM	129	0.00	Pass	500.0	[<Confirmed>]	
2	217.3	10106	75358	SS20-FAM	218	0.20	Pass	500.0	[<Confirmed>]	
3	231.1	9086	70329	SS20-FAM	232	0.30	Pass	500.0	[<Confirmed>]	

Sample 20: SSS13_SS20_SS11_SS21_SS02_SS19_HBB2_C07.fsa

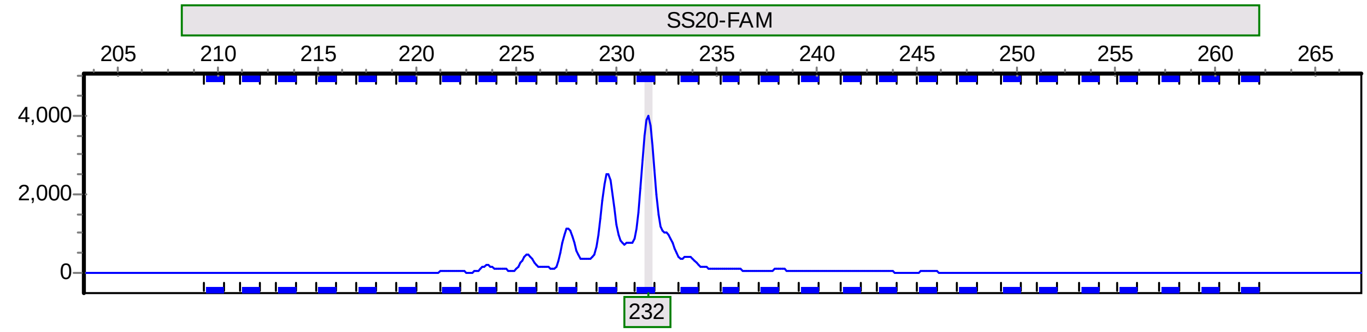

No	Size	Height	Area	Marker	Allele	Difference	Quality	Score	Allele Comments	Sample Comments
1	137.4	8724	61290	SSS13-FAM	137	0.10	Pass	500.0	[<Confirmed>]	
2	143.5	20072	149557	SSS13-FAM	143	0.30	Pass	500.0	[<Confirmed>]	
3	231.6	3979	32089	SS20-FAM	232	0.20	Pass	500.0	[<Confirmed>]	

Sample 21: SSS13_SS20_SS11_SS21_SS02_SS19_HBB30_L13.fsa

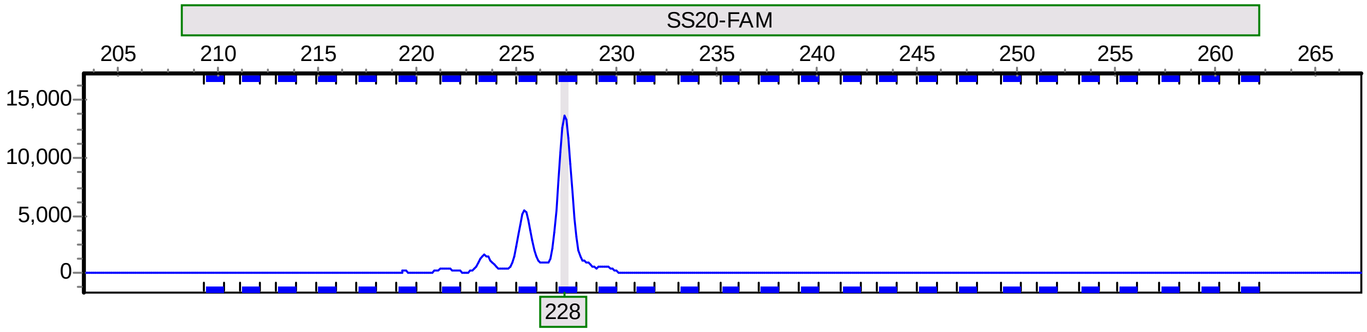

No	Size	Height	Area	Marker	Allele	Difference	Quality	Score	Allele Comments	Sample Comments
1	129.2	31685	252101	SSS13-FAM	129	0.10	Pass	500.0	[<Confirmed>]	
2	227.4	13552	102397	SS20-FAM	228	0.10	Pass	500.0	[<Confirmed>]	

Allele Report

Sample 22: SSS13_SS20_SS11_SS21_SS02_SS19_HBB31_P07.fsa

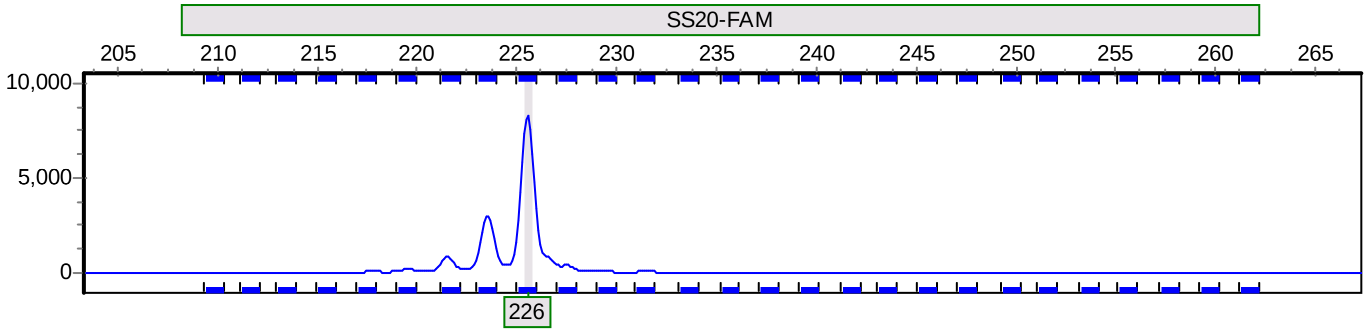

No	Size	Height	Area	Marker	Allele	Difference	Quality	Score	Allele Comments	Sample Comments
1	129.2	20699	134152	SSS13-FAM	129	0.10	Pass	500.0	[<Confirmed>]	
2	144.3	11270	82166	SSS13-FAM	143	0.50	Pass	500.0	[<Confirmed>]	
3	225.6	8268	62241	SS20-FAM	226	0.10	Pass	500.0	[<Confirmed>]	

Sample 23: SSS13_SS20_SS11_SS21_SS02_SS19_HBB32_B15.fsa

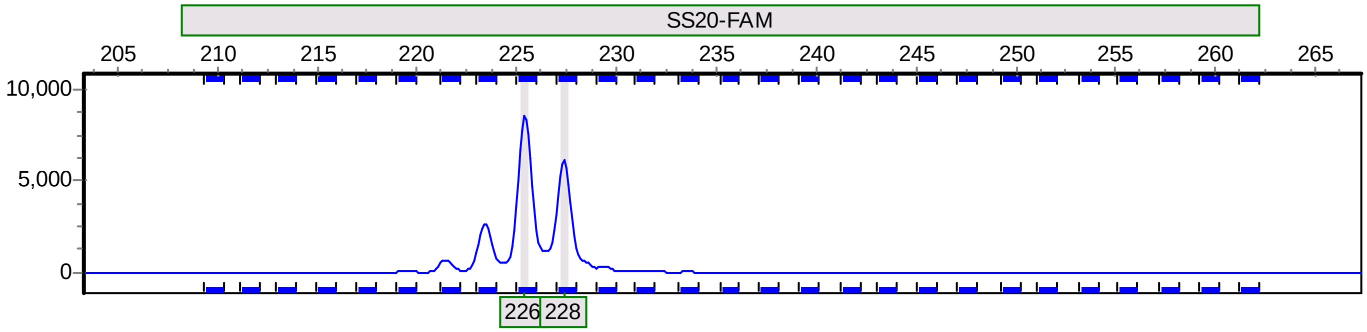

No	Size	Height	Area	Marker	Allele	Difference	Quality	Score	Allele Comments	Sample Comments
1	137.4	31505	241112	SSS13-FAM	137	0.10	Pass	500.0	[<Confirmed>]	
2	225.4	8506	66626	SS20-FAM	226	0.10	Pass	500.0	[<Confirmed>]	
3	227.4	6116	47791	SS20-FAM	228	0.10	Pass	500.0	[<Confirmed>]	

Sample 24: SSS13_SS20_SS11_SS21_SS02_SS19_HBB33_N13.fsa

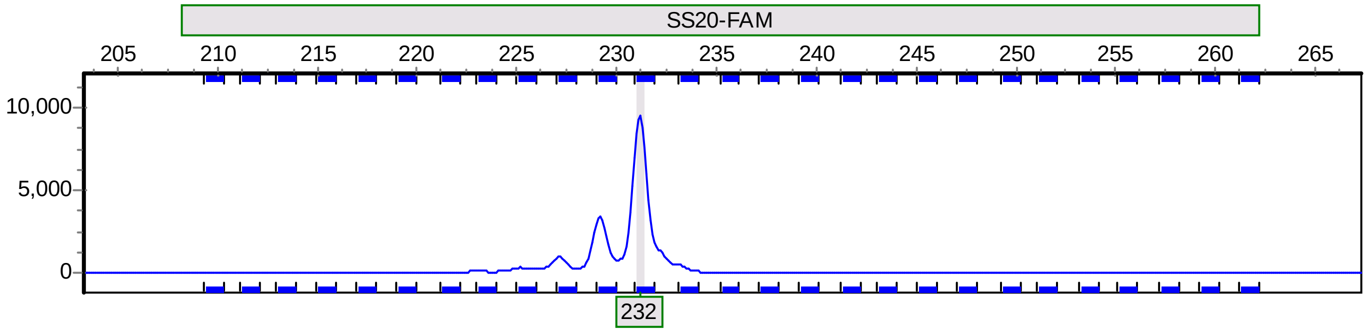

No	Size	Height	Area	Marker	Allele	Difference	Quality	Score	Allele Comments	Sample Comments
1	137.4	9099	44565	SSS13-FAM	137	0.10	Pass	500.0	[<Deleted>]	
2	138.9	20070	103303	SSS13-FAM	139	0.60	Pass	500.0	[<Confirmed>]	
3	231.2	9453	75492	SS20-FAM	232	0.20	Pass	500.0	[<Confirmed>]	

Allele Report

Sample 25: SSS13_SS20_SS11_SS21_SS02_SS19_HBB34_P13.fsa

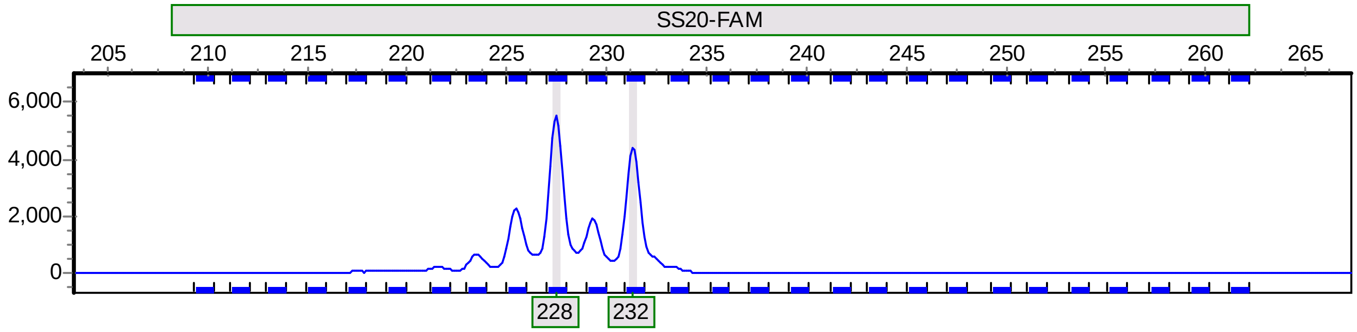

No	Size	Height	Area	Marker	Allele	Difference	Quality	Score	Allele Comments	Sample Comments
1	135.4	17650	120223	SSS13-FAM	135	0.00	Pass	500.0	[<Confirmed>]	
2	144.4	11449	85495	SSS13-FAM	145	1.00	Pass	500.0	[<Confirmed><Edited>]	
3	227.5	5505	43045	SS20-FAM	228	0.00	Pass	500.0	[<Confirmed>]	
4	231.3	4404	35371	SS20-FAM	232	0.10	Pass	500.0	[<Confirmed>]	

Sample 26: SSS13_SS20_SS11_SS21_SS02_SS19_HBB35_C15.fsa

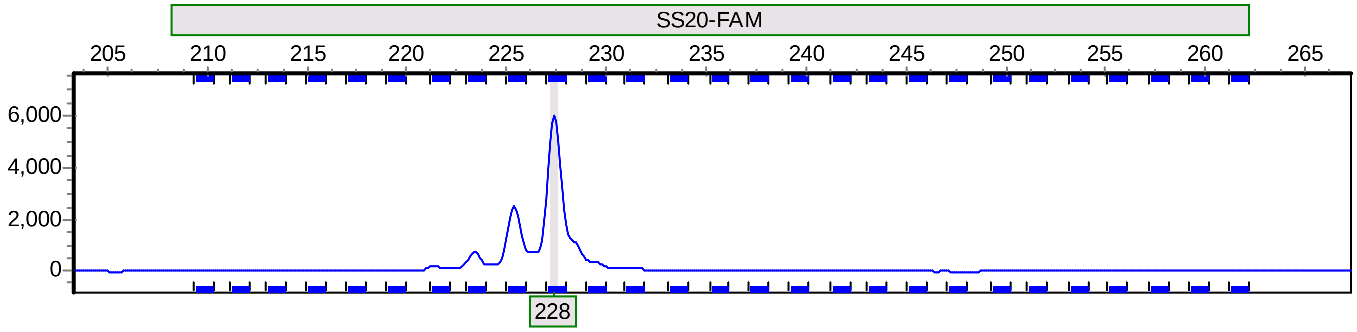

No	Size	Height	Area	Marker	Allele	Difference	Quality	Score	Allele Comments	Sample Comments
1	137.5	12033	60980	SSS13-FAM	137	0.00	Pass	500.0	[<Deleted>]	
2	139.0	33093	172688	SSS13-FAM	139	0.50	Pass	500.0	[<SAT (Repaired)><Confirmed>]	
3	227.4	5968	47111	SS20-FAM	228	0.10	Pass	500.0	[<Confirmed>]	

Sample 27: SSS13_SS20_SS11_SS21_SS02_SS19_HBB36_G05.fsa

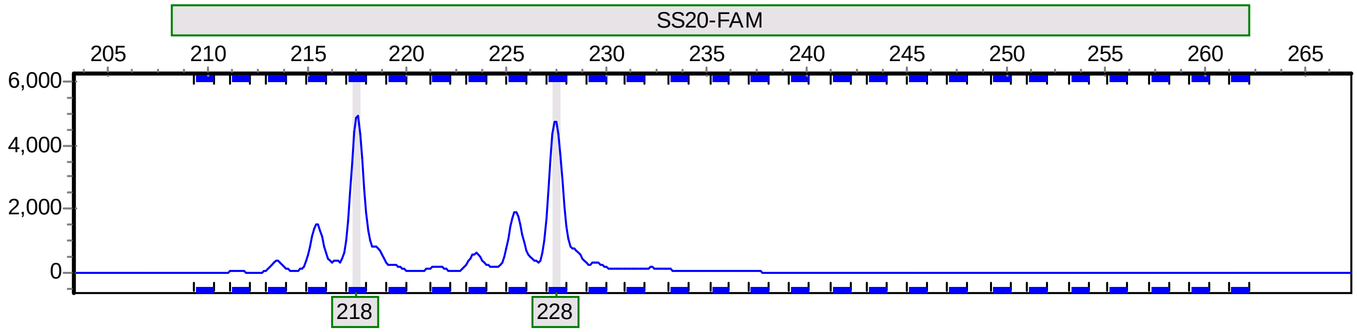

No	Size	Height	Area	Marker	Allele	Difference	Quality	Score	Allele Comments	Sample Comments
1	129.2	31477	234270	SSS13-FAM	129	0.10	Pass	500.0	[<Confirmed>]	
2	217.5	4895	34804	SS20-FAM	218	0.00	Pass	500.0	[<Confirmed>]	
3	227.5	4757	36893	SS20-FAM	228	0.00	Pass	500.0	[<Confirmed>]	

SoftGenetics

GeneMarker V3.0.0

Allele Report

Sample 28: SSS13_SS20_SS11_SS21_SS02_SS19_HBB37_N09.fsa

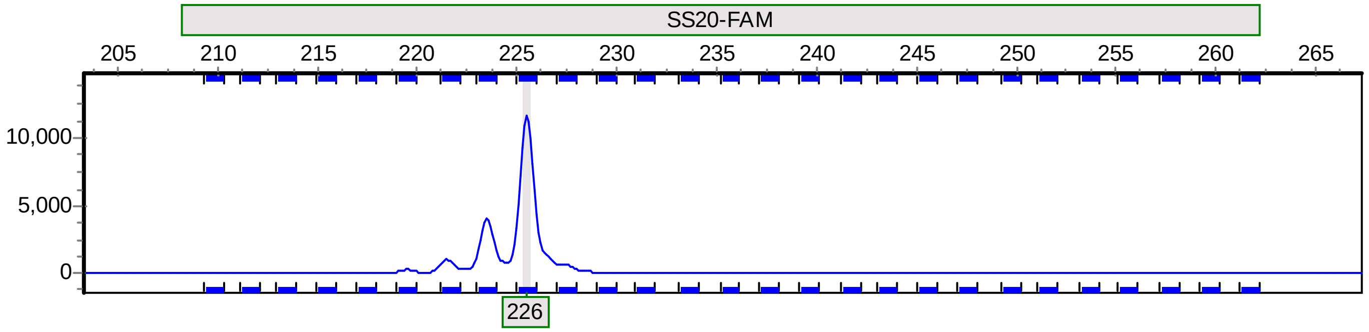

No	Size	Height	Area	Marker	Allele	Difference	Quality	Score	Allele Comments	Sample Comments
1	135.4	22273	157422	SSS13-FAM	135	0.00	Pass	500.0	[<Confirmed>]	
2	137.5	18234	130134	SSS13-FAM	137	0.00	Pass	500.0	[<Confirmed>]	
3	139.7	14602	113885	SSS13-FAM	139	0.20	Undetermined	500.0	[<Deleted>]	
4	225.5	11636	91855	SS20-FAM	226	0.00	Pass	500.0	[<Confirmed>]	

Sample 29: SSS13_SS20_SS11_SS21_SS02_SS19_HBB38_M07.fsa

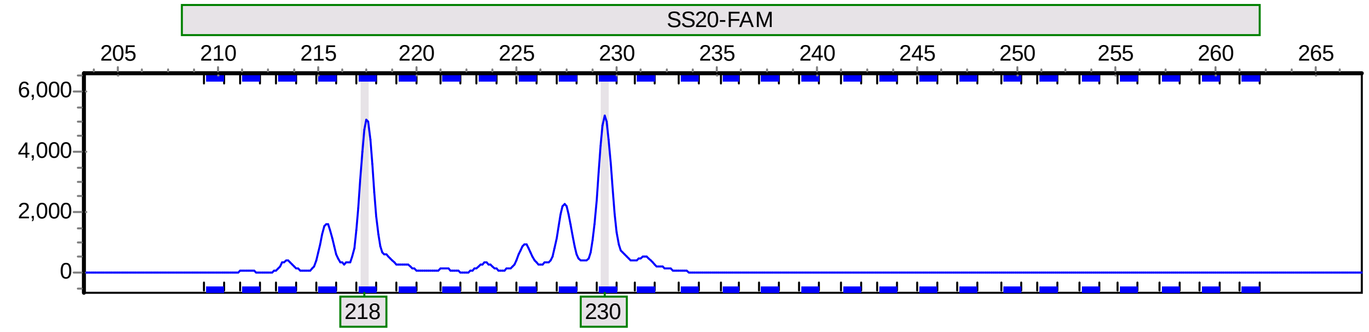

No	Size	Height	Area	Marker	Allele	Difference	Quality	Score	Allele Comments	Sample Comments
1	129.4	20487	136836	SSS13-FAM	129	0.10	Pass	500.0	[<Confirmed>]	
2	144.4	13139	98128	SSS13-FAM	145	1.00	Pass	500.0	[<Confirmed><Edited>]	
3	217.4	5110	39817	SS20-FAM	218	0.10	Pass	500.0	[<Confirmed>]	
4	229.4	5193	41837	SS20-FAM	230	0.10	Pass	500.0	[<Confirmed>]	

Sample 30: SSS13_SS20_SS11_SS21_SS02_SS19_HBB39_F05.fsa

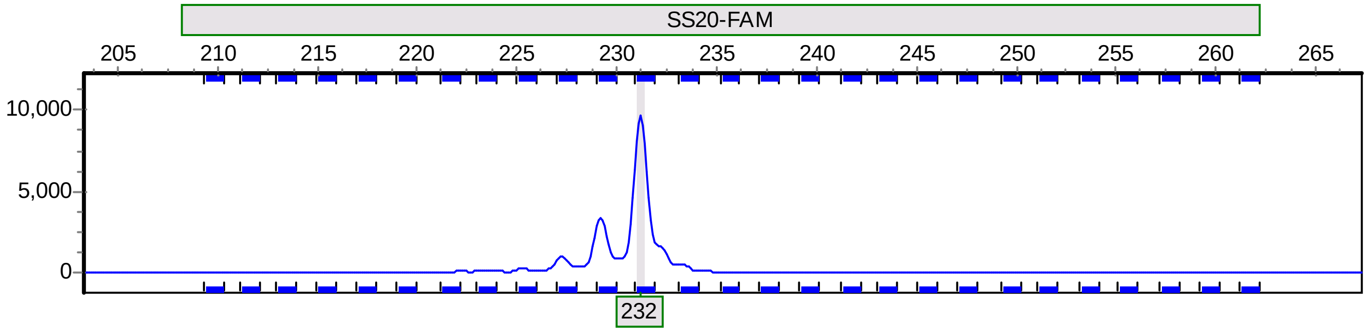

No	Size	Height	Area	Marker	Allele	Difference	Quality	Score	Allele Comments	Sample Comments
1	139.0	12144	56779	SSS13-FAM	139	0.50	Pass	500.0	[<Deleted>]	
2	140.3	29972	132379	SSS13-FAM	139	1.00	Pass	500.0	[<Confirmed><Edited>]	
3	141.8	10994	52172	SSS13-FAM	141	0.30	Undetermined	500.0	[<Deleted>]	
4	143.2	19914	91166	SSS13-FAM	143	0.60	Pass	500.0	[<Confirmed>]	
5	231.2	9629	72648	SS20-FAM	232	0.20	Pass	500.0	[<Confirmed>]	

Allele Report

Sample 31: SSS13_SS20_SS11_SS21_SS02_SS19_HBB40_D15.fsa

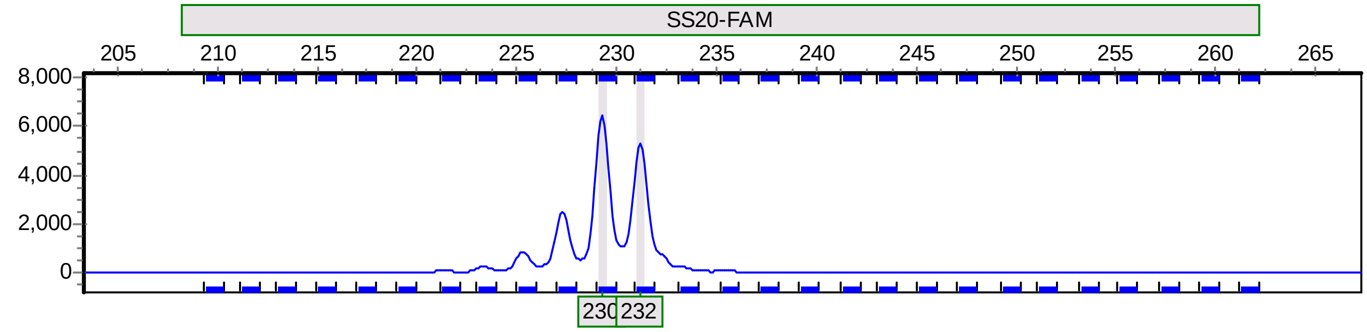

No	Size	Height	Area	Marker	Allele	Difference	Quality	Score	Allele Comments	Sample Comments
1	129.1	30145	210416	SSS13-FAM	129	0.20	Pass	500.0	[<Confirmed>]	
2	229.3	6402	51099	SS20-FAM	230	0.20	Pass	500.0	[<Confirmed>]	
3	231.2	5308	44646	SS20-FAM	232	0.20	Pass	500.0	[<Confirmed>]	

Sample 32: SSS13_SS20_SS11_SS21_SS02_SS19_HBB41_L07.fsa

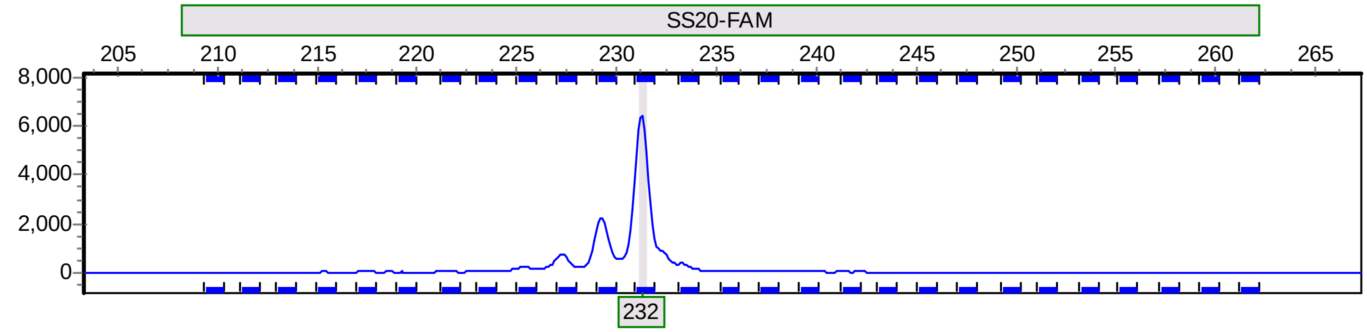

No	Size	Height	Area	Marker	Allele	Difference	Quality	Score	Allele Comments	Sample Comments
1	129.3	17141	114086	SSS13-FAM	129	0.00	Pass	500.0	[<Confirmed>]	
2	144.4	11160	83750	SSS13-FAM	143	1.00	Pass	500.0	[<Confirmed><Edited>]	
3	231.3	6375	50529	SS20-FAM	232	0.10	Pass	500.0	[<Confirmed>]	

Sample 33: SSS13_SS20_SS11_SS21_SS02_SS19_HBB42_M05.fsa

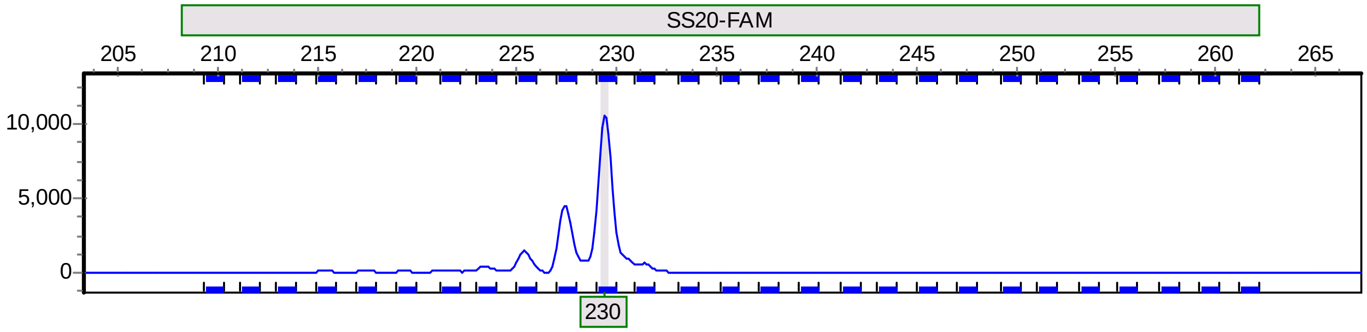

No	Size	Height	Area	Marker	Allele	Difference	Quality	Score	Allele Comments	Sample Comments
1	129.5	23787	152734	SSS13-FAM	129	0.20	Pass	500.0	[<Confirmed>]	
2	144.4	13500	95235	SSS13-FAM	143	1.00	Pass	500.0	[<Confirmed><Edited>]	
3	229.4	10426	80652	SS20-FAM	230	0.10	Pass	500.0	[<Confirmed>]	

Sample 34: SSS13_SS20_SS11_SS21_SS02_SS19_HBB43_D09.fsa

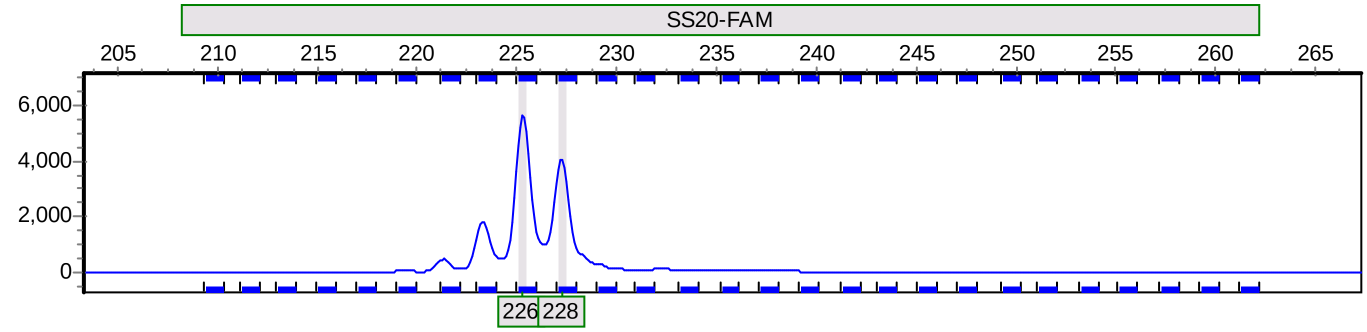

No	Size	Height	Area	Marker	Allele	Difference	Quality	Score	Allele Comments	Sample Comments
1	129.1	31944	247541	SSS13-FAM	129	0.20	Pass	500.0	[<Confirmed>]	
2	225.3	5591	45891	SS20-FAM	226	0.20	Pass	500.0	[<Confirmed>]	
3	227.3	4049	33743	SS20-FAM	228	0.20	Pass	500.0	[<Confirmed>]	

Sample 35: SSS13_SS20_SS11_SS21_SS02_SS19_HBB44_H11.fsa

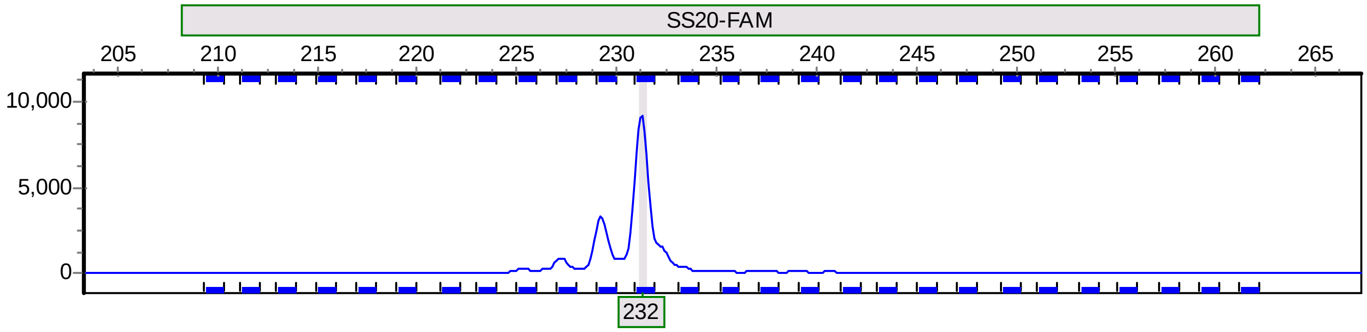

No	Size	Height	Area	Marker	Allele	Difference	Quality	Score	Allele Comments	Sample Comments
1	129.2	29554	198411	SSS13-FAM	129	0.10	Pass	500.0	[<Confirmed>]	
2	144.3	17242	131881	SSS13-FAM	143	0.50	Pass	500.0	[<Confirmed>]	
3	231.3	9120	71922	SS20-FAM	232	0.10	Pass	500.0	[<Confirmed>]	

Sample 36: SSS13_SS20_SS11_SS21_SS02_SS19_HBB45_F13.fsa

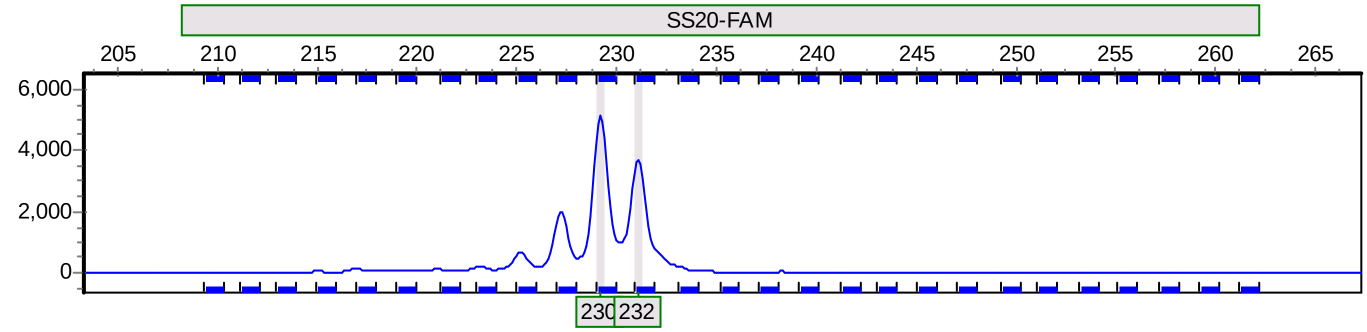

No	Size	Height	Area	Marker	Allele	Difference	Quality	Score	Allele Comments	Sample Comments
1	129.2	26370	182525	SSS13-FAM	129	0.10	Pass	500.0	[<Confirmed>]	
2	229.2	5121	43046	SS20-FAM	230	0.30	Pass	500.0	[<Confirmed>]	
3	231.1	3712	32230	SS20-FAM	232	0.30	Pass	500.0	[<Confirmed>]	

Allele Report

Sample 37: SSS13_SS20_SS11_SS21_SS02_SS19_HBB46_F07.fsa

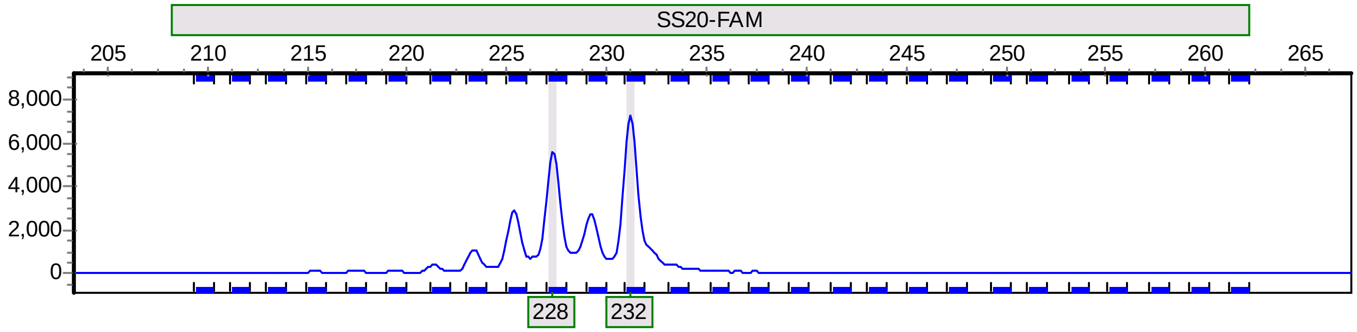

No	Size	Height	Area	Marker	Allele	Difference	Quality	Score	Allele Comments	Sample Comments
1	144.2	26993	205581	SSS13-FAM	143	0.40	Pass	500.0	[<Confirmed>]	
2	146.3	9855	75219	SSS13-FAM	147	0.20	Pass	500.0	[<Confirmed>]	
3	227.3	5570	44268	SS20-FAM	228	0.20	Pass	500.0	[<Confirmed>]	
4	231.2	7252	57640	SS20-FAM	232	0.20	Pass	500.0	[<Confirmed>]	

Sample 38: SSS13_SS20_SS11_SS21_SS02_SS19_HBB47_A05.fsa

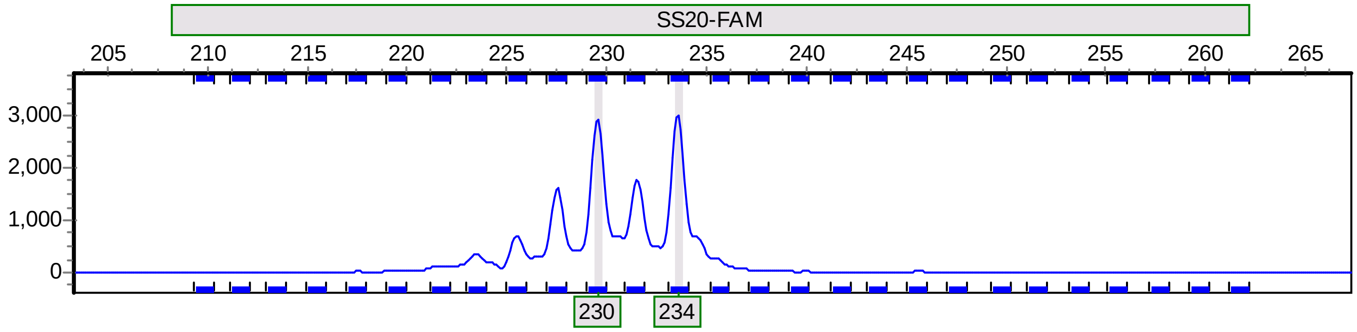

No	Size	Height	Area	Marker	Allele	Difference	Quality	Score	Allele Comments	Sample Comments
1	143.5	28323	209453	SSS13-FAM	143	0.30	Pass	500.0	[<Confirmed>]	
2	229.6	2927	23178	SS20-FAM	230	0.10	Pass	449.4	[<Confirmed>]	
3	233.6	2987	23658	SS20-FAM	234	0.00	Pass	463.3	[<Confirmed>]	

Sample 39: SSS13_SS20_SS11_SS21_SS02_SS19_HBB48_B13.fsa

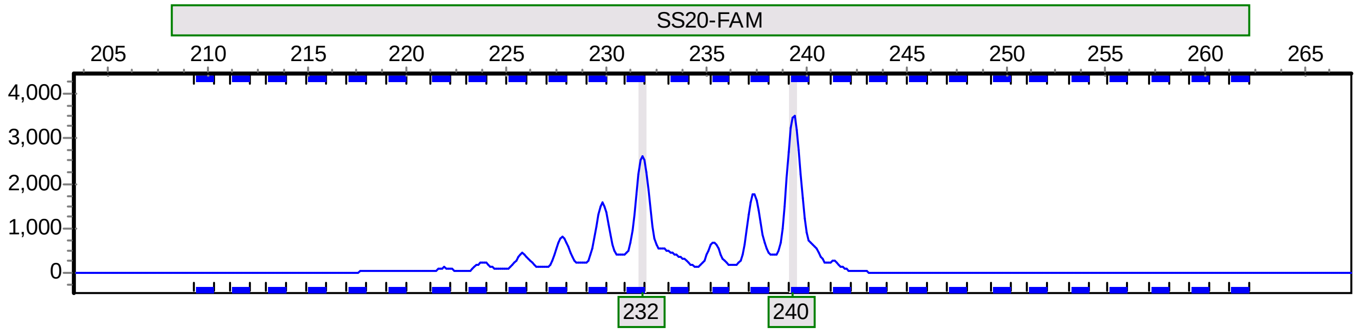

No	Size	Height	Area	Marker	Allele	Difference	Quality	Score	Allele Comments	Sample Comments
1	127.1	27144	184608	SSS13-FAM	127	0.10	Pass	500.0	[<Confirmed>]	
2	143.5	12136	92498	SSS13-FAM	143	0.30	Pass	500.0	[<Confirmed>]	
3	231.8	2637	21184	SS20-FAM	232	0.40	Pass	356.9	[<Confirmed>]	
4	239.4	3496	28830	SS20-FAM	240	0.30	Pass	500.0	[<Confirmed>]	

Allele Report

Sample 40: SSS13_SS20_SS11_SS21_SS02_SS19_HBB49_L05.fsa

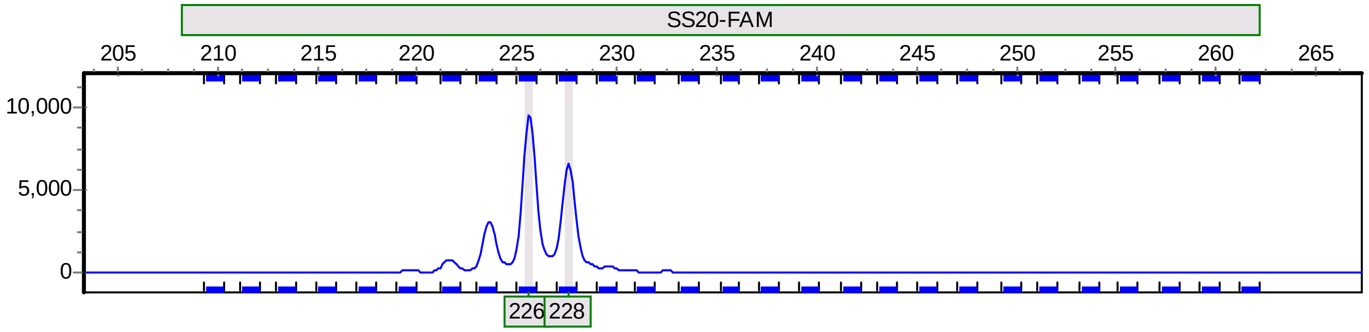

No	Size	Height	Area	Marker	Allele	Difference	Quality	Score	Allele Comments	Sample Comments
1	127.1	26406	176933	SSS13-FAM	127	0.10	Pass	500.0	[<Confirmed>]	
2	129.3	20534	133535	SSS13-FAM	129	0.00	Pass	500.0	[<Confirmed>]	
3	225.6	9455	72528	SS20-FAM	226	0.10	Pass	500.0	[<Confirmed>]	
4	227.6	6556	50044	SS20-FAM	228	0.10	Pass	500.0	[<Confirmed>]	

Sample 41: SSS13_SS20_SS11_SS21_SS02_SS19_HBB4_E07.fsa

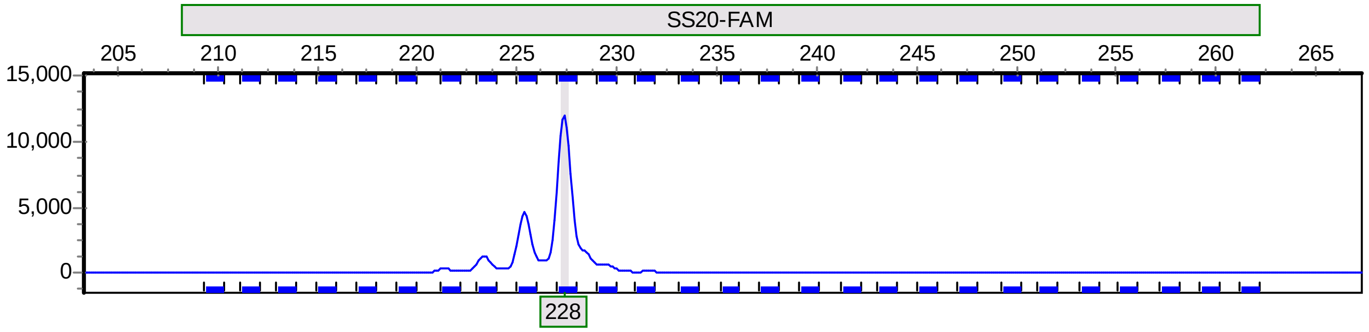

No	Size	Height	Area	Marker	Allele	Difference	Quality	Score	Allele Comments	Sample Comments
1	137.5	32610	240815	SSS13-FAM	137	0.00	Pass	500.0	[<SAT (Repaired)><Confirmed>]	
2	227.4	11924	91668	SS20-FAM	228	0.10	Pass	500.0	[<Confirmed>]	

Sample 42: SSS13_SS20_SS11_SS21_SS02_SS19_HBB5_H13.fsa

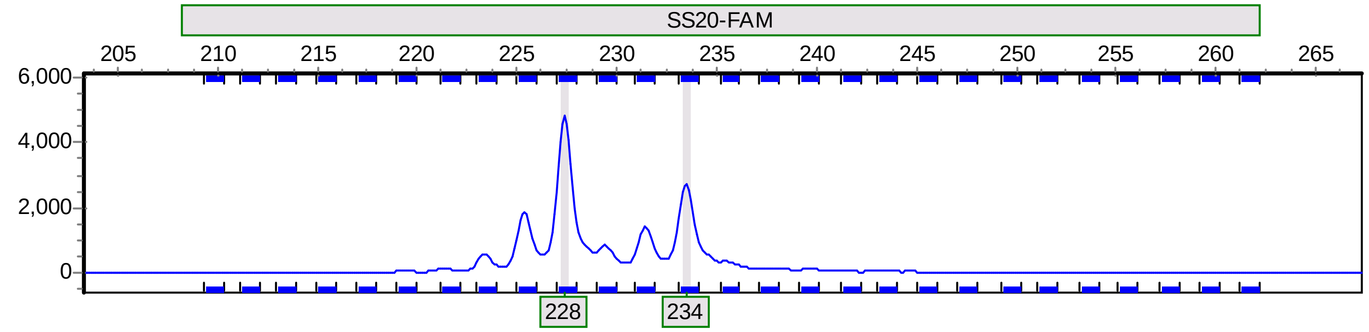

No	Size	Height	Area	Marker	Allele	Difference	Quality	Score	Allele Comments	Sample Comments
1	129.1	31196	235509	SSS13-FAM	129	0.20	Pass	500.0	[<Confirmed>]	
2	227.4	4793	40554	SS20-FAM	228	0.10	Pass	500.0	[<Confirmed>]	
3	233.5	2690	23741	SS20-FAM	234	0.10	Pass	301.2	[<Confirmed>]	

Allele Report

Sample 43: SSS13_SS20_SS11_SS21_SS02_SS19_HBB6_C05.fsa

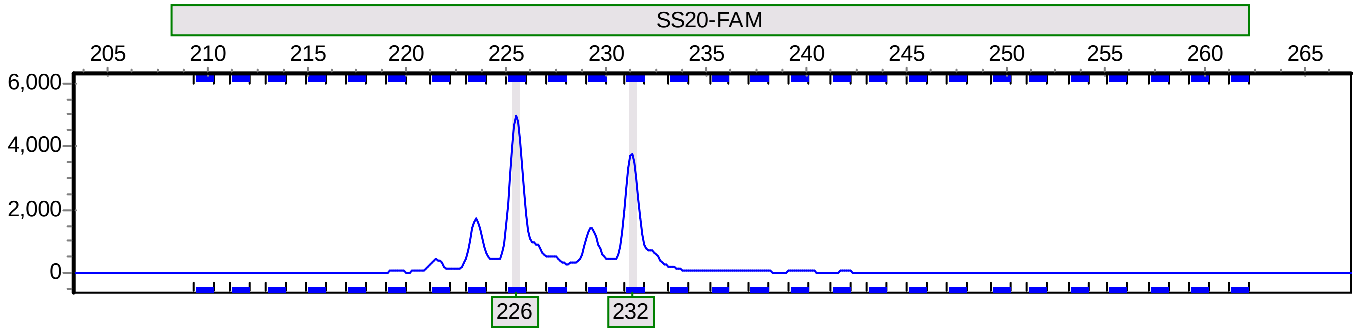

No	Size	Height	Area	Marker	Allele	Difference	Quality	Score	Allele Comments	Sample Comments
1	129.0	32169	233938	SSS13-FAM	129	0.30	Pass	500.0	[<SAT (Repaired)><Confirmed>]	
2	225.5	4936	38653	SS20-FAM	226	0.00	Pass	500.0	[<Confirmed>]	
3	231.3	3745	28959	SS20-FAM	232	0.10	Pass	500.0	[<Confirmed>]	

Sample 44: SSS13_SS20_SS11_SS21_SS02_SS19_HBB7_N05.fsa

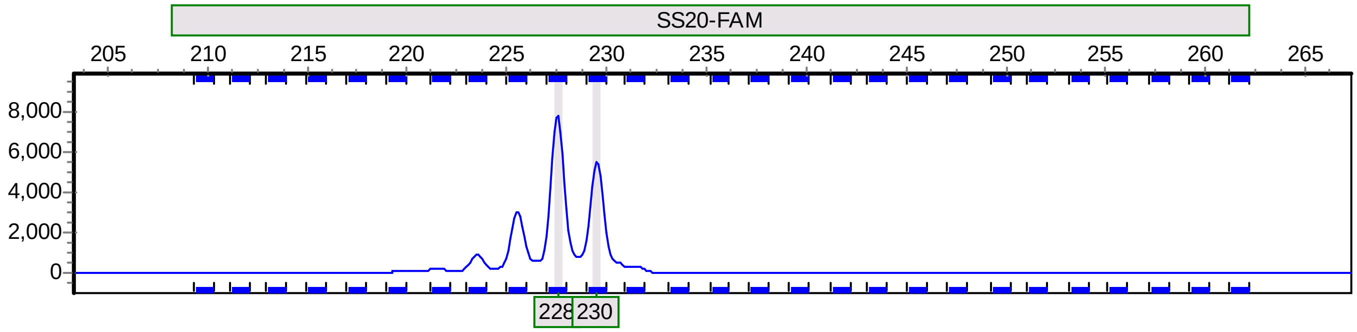

No	Size	Height	Area	Marker	Allele	Difference	Quality	Score	Allele Comments	Sample Comments
1	129.3	14650	96388	SSS13-FAM	129	0.00	Pass	500.0	[<Confirmed>]	
2	137.6	21179	138384	SSS13-FAM	137	0.10	Pass	500.0	[<Confirmed>]	
3	227.6	7785	60041	SS20-FAM	228	0.10	Pass	500.0	[<Confirmed>]	
4	229.5	5531	43252	SS20-FAM	230	0.00	Pass	500.0	[<Confirmed>]	

Sample 45: SSS13_SS20_SS11_SS21_SS02_SS19_HBB8_P11.fsa

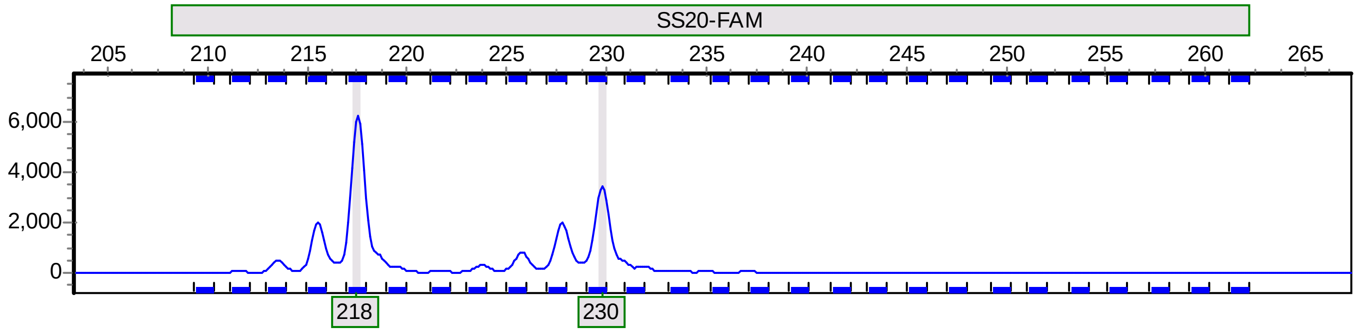

No	Size	Height	Area	Marker	Allele	Difference	Quality	Score	Allele Comments	Sample Comments
1	129.0	15685	106935	SSS13-FAM	129	0.30	Pass	500.0	[<Confirmed>]	
2	144.1	17194	127780	SSS13-FAM	143	0.30	Pass	500.0	[<Confirmed>]	
3	217.5	6238	49038	SS20-FAM	218	0.00	Pass	500.0	[<Confirmed>]	
4	229.8	3474	29183	SS20-FAM	230	0.30	Pass	500.0	[<Confirmed>]	

Allele Report

Sample 46: SSS13_SS20_SS11_SS21_SS02_SS19_HBB9_I05.fsa

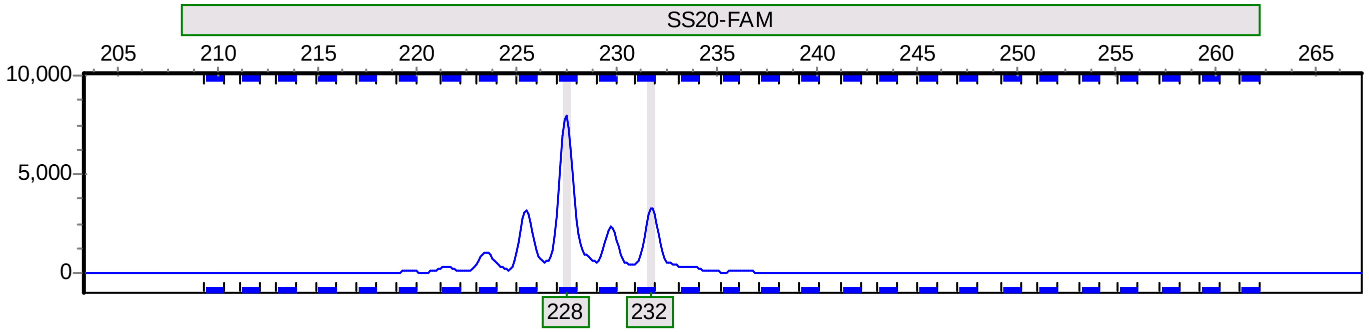

No	Size	Height	Area	Marker	Allele	Difference	Quality	Score	Allele Comments	Sample Comments
1	120.3	36065	257571	SSS13-FAM	121	0.00	Pass	500.0	[<SAT (Repaired)><Confirmed>]	
2	227.5	7935	62227	SS20-FAM	228	0.00	Pass	500.0	[<Confirmed>]	
3	231.7	3316	26428	SS20-FAM	232	0.30	Pass	500.0	[<Confirmed>]	

Sample 47: SSS13_SS20_SS11_SS21_SS02_SS19_HBN10_G11.fsa

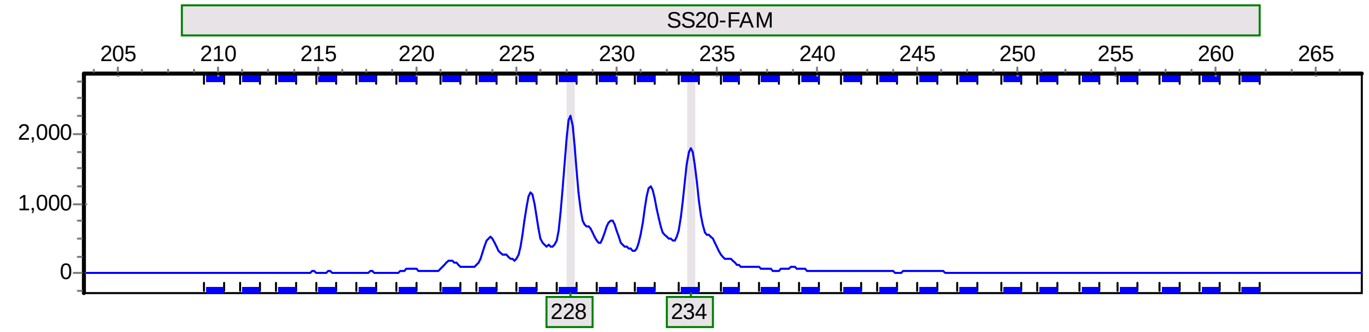

No	Size	Height	Area	Marker	Allele	Difference	Quality	Score	Allele Comments	Sample Comments
1	131.2	31108	215149	SSS13-FAM	131	0.10	Pass	500.0	[<Confirmed>]	
2	133.3	20373	137784	SSS13-FAM	133	0.10	Pass	500.0	[<Confirmed>]	
3	227.7	2265	18188	SS20-FAM	228	0.20	Pass	289.1	[<Confirmed>]	
4	233.7	1801	16130	SS20-FAM	234	0.10	Pass	164.0	[<Confirmed>]	

Sample 48: SSS13_SS20_SS11_SS21_SS02_SS19_HBN6_O01.fsa

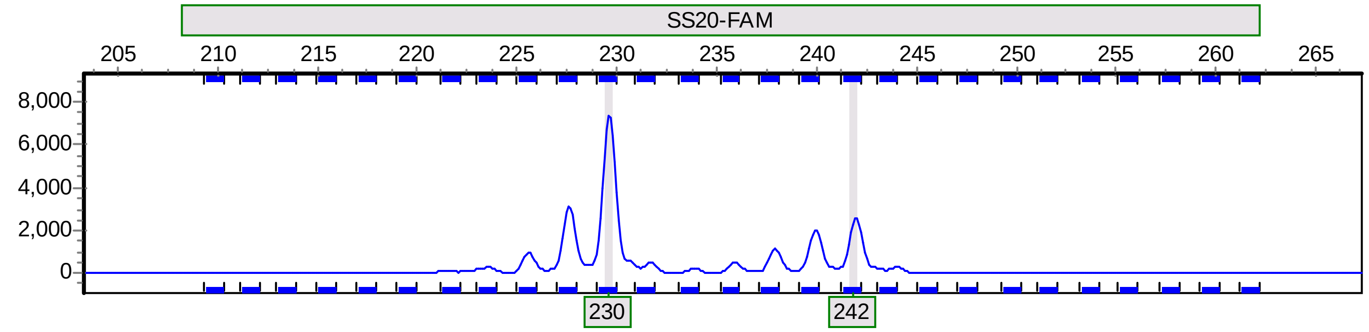

No	Size	Height	Area	Marker	Allele	Difference	Quality	Score	Allele Comments	Sample Comments
1	131.7	26043	160748	SSS13-FAM	131	0.40	Pass	500.0	[<Confirmed>]	
2	144.7	15071	103135	SSS13-FAM	145	1.00	Pass	500.0	[<Confirmed><Edited>]	
3	229.6	7305	52828	SS20-FAM	230	0.10	Pass	500.0	[<Confirmed>]	
4	241.9	2524	19050	SS20-FAM	242	0.10	Pass	401.3	[<Confirmed>]	

Allele Report

Sample 49: SSS13_SS20_SS11_SS21_SS02_SS19_HBN9_D03.fsa

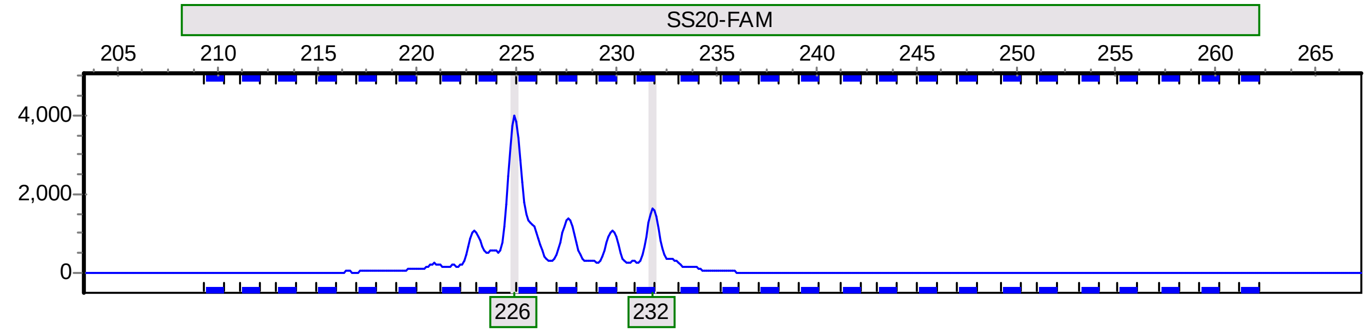

No	Size	Height	Area	Marker	Allele	Difference	Quality	Score	Allele Comments	Sample Comments
1	129.5	18727	119403	SSS13-FAM	129	0.20	Pass	500.0	[<Confirmed>]	
2	143.6	11671	82766	SSS13-FAM	143	0.20	Pass	500.0	[<Confirmed>]	
3	224.9	3992	31012	SS20-FAM	226	1.00	Pass	500.0	[<Confirmed><Edited>]	
4	227.6	1379	12080	SS20-FAM	228	0.10	Pass	116.7	[<Deleted>]	
5	231.8	1647	12070	SS20-FAM	232	0.40	Pass	217.3	[<Confirmed>]	

Sample 50: SSS13_SS20_SS11_SS21_SS02_SS19_HCW1_M09.fsa

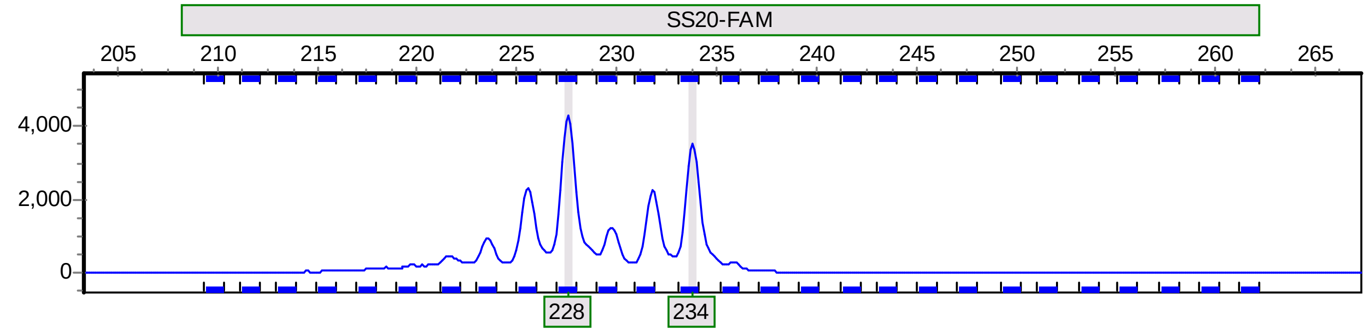

No	Size	Height	Area	Marker	Allele	Difference	Quality	Score	Allele Comments	Sample Comments
1	133.7	26423	190504	SSS13-FAM	133	0.30	Pass	500.0	[<Confirmed>]	
2	135.5	17714	124900	SSS13-FAM	135	0.10	Pass	500.0	[<Confirmed>]	
3	227.6	4245	34343	SS20-FAM	228	0.10	Pass	500.0	[<Confirmed>]	
4	233.8	3498	27607	SS20-FAM	234	0.20	Pass	500.0	[<Confirmed>]	

Sample 51: SSS13_SS20_SS11_SS21_SS02_SS19_HCW2_A11.fsa

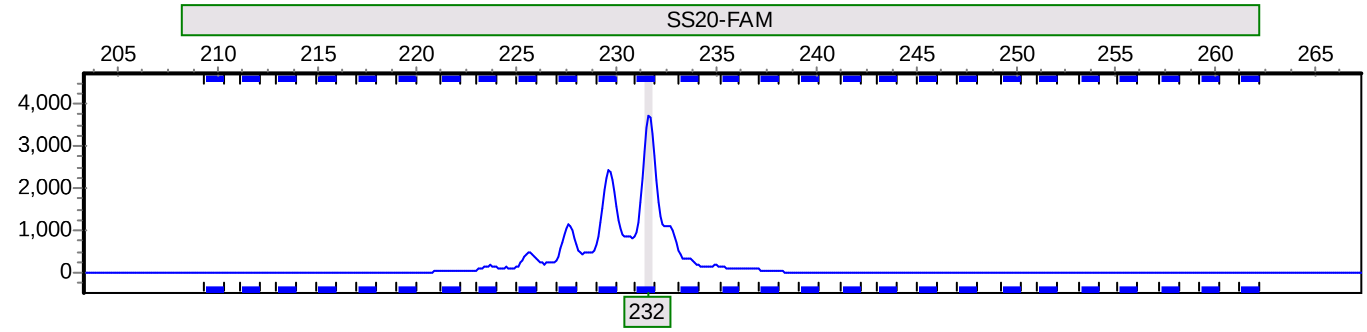

No	Size	Height	Area	Marker	Allele	Difference	Quality	Score	Allele Comments	Sample Comments
1	135.6	22947	172371	SSS13-FAM	135	0.20	Pass	500.0	[<Confirmed>]	
2	137.8	19570	132628	SSS13-FAM	137	0.30	Pass	500.0	[<Confirmed>]	
3	231.6	3695	29965	SS20-FAM	232	0.20	Pass	500.0	[<Confirmed>]	

Allele Report

Sample 52: SSS13_SS20_SS11_SS21_SS02_SS19_HCW3_G13.fsa

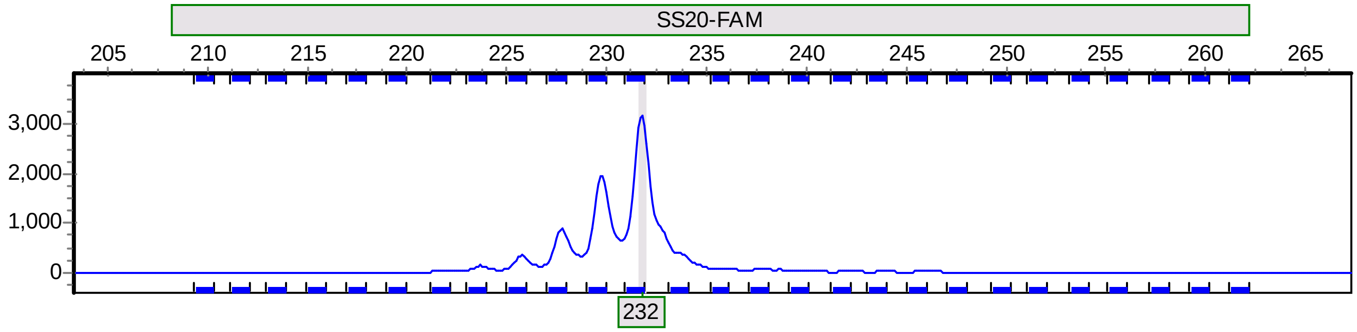

No	Size	Height	Area	Marker	Allele	Difference	Quality	Score	Allele Comments	Sample Comments
1	133.3	15339	121298	SSS13-FAM	133	0.10	Pass	500.0	[<Confirmed>]	
2	135.2	9117	70711	SSS13-FAM	135	0.20	Pass	500.0	[<Confirmed>]	
3	231.8	3131	27018	SS20-FAM	232	0.40	Pass	354.5	[<Confirmed>]	

Sample 53: SSS13_SS20_SS11_SS21_SS02_SS19_HCW4_E09.fsa

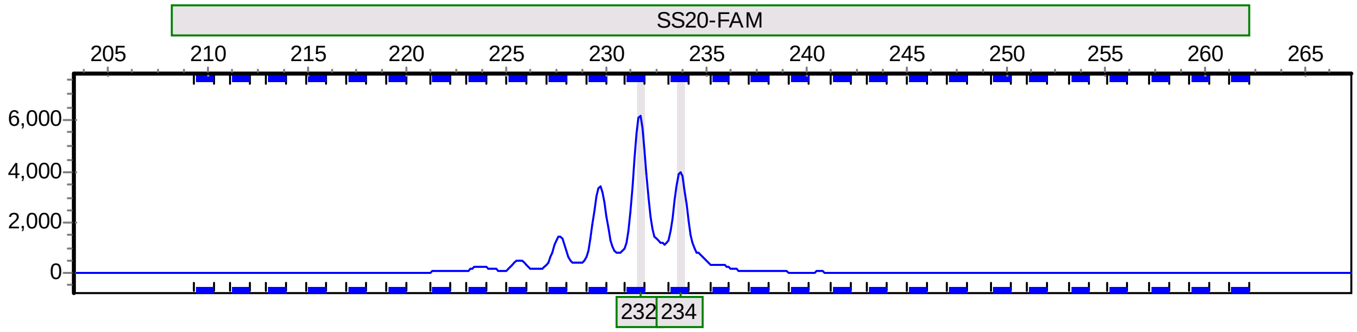

No	Size	Height	Area	Marker	Allele	Difference	Quality	Score	Allele Comments	Sample Comments
1	137.4	31906	235094	SSS13-FAM	137	0.10	Pass	500.0	[<Confirmed>]	
2	231.7	6145	49679	SS20-FAM	232	0.30	Pass	500.0	[<Confirmed>]	
3	233.7	3988	32507	SS20-FAM	234	0.10	Pass	500.0	[<Confirmed>]	

Sample 54: SSS13_SS20_SS11_SS21_SS02_SS19_HCW5_C11.fsa

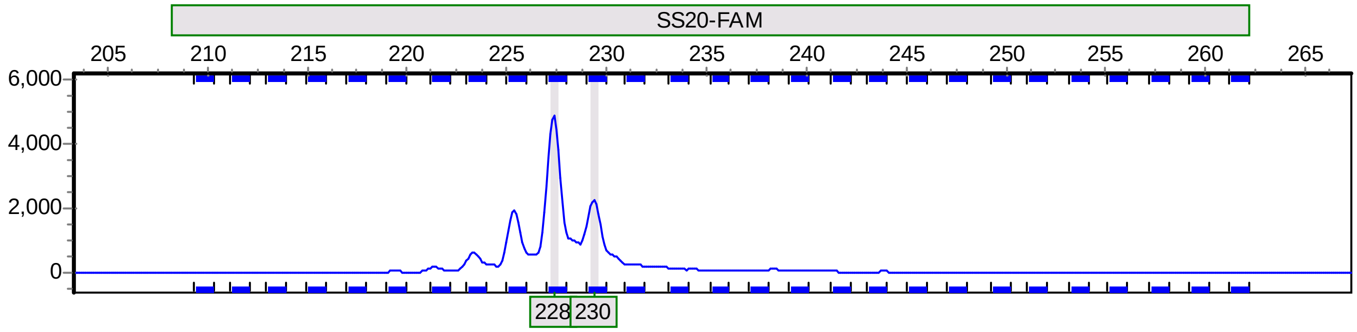

No	Size	Height	Area	Marker	Allele	Difference	Quality	Score	Allele Comments	Sample Comments
1	129.1	24387	164570	SSS13-FAM	129	0.20	Pass	500.0	[<Confirmed>]	
2	146.4	14499	108243	SSS13-FAM	147	0.10	Pass	500.0	[<Confirmed>]	
3	227.4	4840	38391	SS20-FAM	228	0.10	Pass	500.0	[<Confirmed>]	
4	229.4	2246	19218	SS20-FAM	230	0.10	Pass	239.7	[<Confirmed>]	

Allele Report

Sample 55: SSS13_SS20_SS11_SS21_SS02_SS19_HCW6_D01.fsa

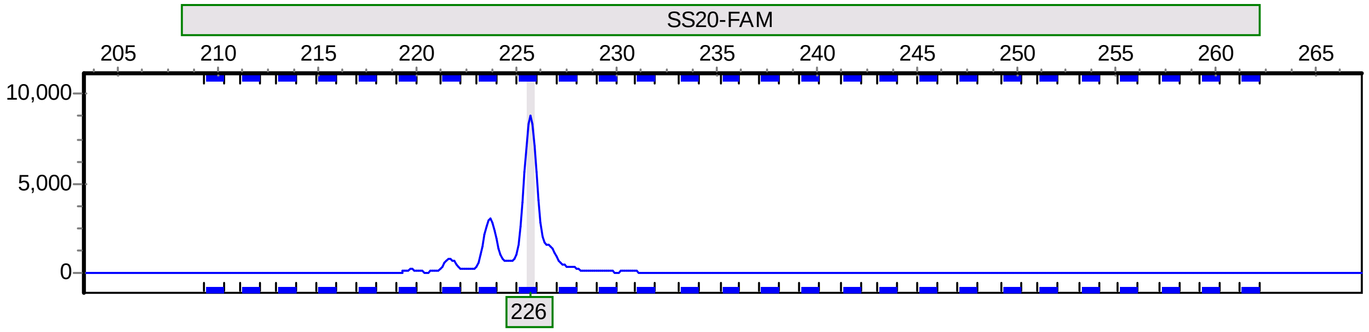

No	Size	Height	Area	Marker	Allele	Difference	Quality	Score	Allele Comments	Sample Comments
1	123.3	31062	200522	SSS13-FAM	123	0.10	Pass	500.0	[<Confirmed>]	
2	135.5	23712	152369	SSS13-FAM	135	0.10	Pass	500.0	[<Confirmed>]	
3	225.7	8736	64876	SS20-FAM	226	0.20	Pass	500.0	[<Confirmed>]	

Sample 56: SSS13_SS20_SS11_SS21_SS02_SS19_HCW7_G17.fsa

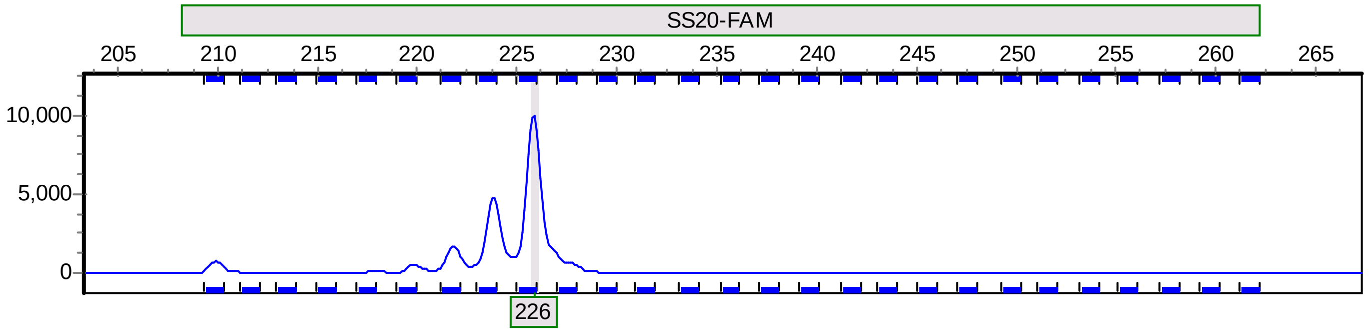

No	Size	Height	Area	Marker	Allele	Difference	Quality	Score	Allele Comments	Sample Comments
1	125.2	23470	158100	SSS13-FAM	125	0.10	Pass	500.0	[<Confirmed>]	
2	129.4	31446	227098	SSS13-FAM	129	0.10	Pass	500.0	[<Confirmed>]	
3	225.9	9995	79962	SS20-FAM	226	0.40	Pass	500.0	[<Confirmed>]	

Sample 57: SSS13_SS20_SS11_SS21_SS02_SS19_HCW8_A17.fsa

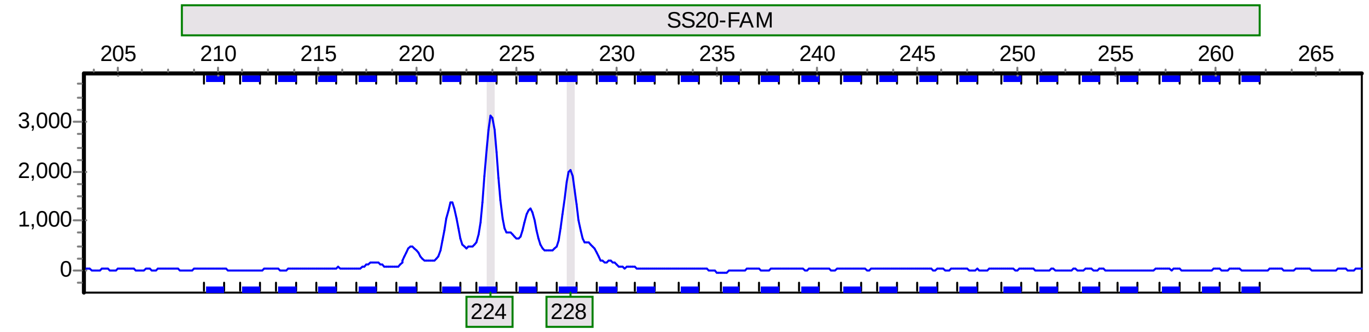

No	Size	Height	Area	Marker	Allele	Difference	Quality	Score	Allele Comments	Sample Comments
1	130.7	23179	161686	SSS13-FAM	131	1.00	Pass	500.0	[<Confirmed><Edited>]	
2	137.4	14548	98011	SSS13-FAM	137	0.10	Pass	500.0	[<Confirmed>]	
3	223.7	3103	25141	SS20-FAM	224	0.20	Pass	444.4	[<Confirmed>]	
4	227.7	2031	16406	SS20-FAM	228	0.20	Pass	244.2	[<Confirmed>]	

Allele Report

Sample 58: SSS13_SS20_SS11_SS21_SS02_SS19_HGC1_C01.fsa

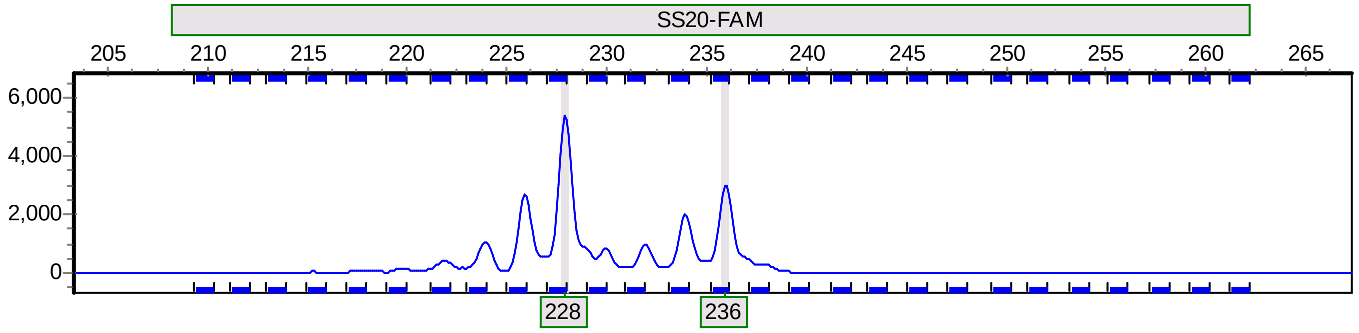

No	Size	Height	Area	Marker	Allele	Difference	Quality	Score	Allele Comments	Sample Comments
1	137.6	37590	301177	SSS13-FAM	137	0.10	Pass	500.0	[<SAT (Repaired)><Confirmed>]	
2	227.9	5352	41063	SS20-FAM	228	0.40	Pass	500.0	[<Confirmed>]	
3	236.0	2970	23456	SS20-FAM	236	0.30	Pass	456.0	[<Confirmed>]	

Sample 59: SSS13_SS20_SS11_SS21_SS02_SS19_HGC3_E17.fsa

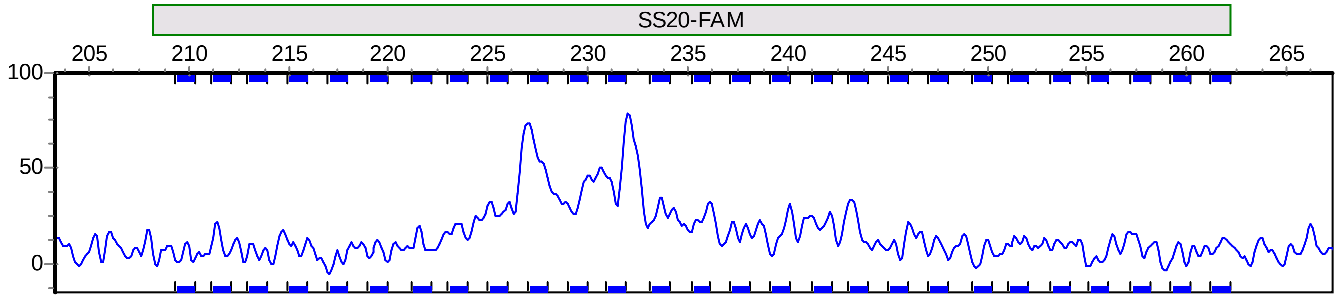

No	Size	Height	Area	Marker	Allele	Difference	Quality	Score	Allele Comments	Sample Comments

Sample 60: SSS13_SS20_SS11_SS21_SS02_SS19_HGC4_A13.fsa

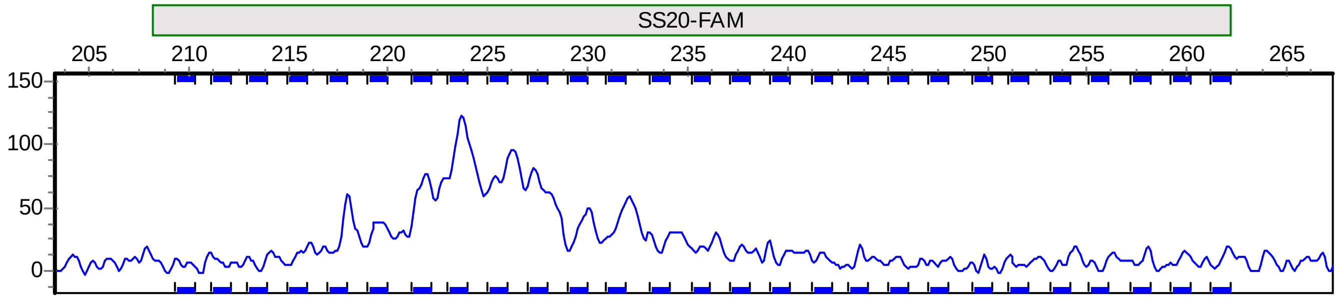

No	Size	Height	Area	Marker	Allele	Difference	Quality	Score	Allele Comments	Sample Comments
1	132.0	892	7008	SSS13-FAM	131	1.00	Pass	79.5	[<Confirmed><Edited>]	

Sample 61: SSS13_SS20_SS11_SS21_SS02_SS19_HGY1_A09.fsa

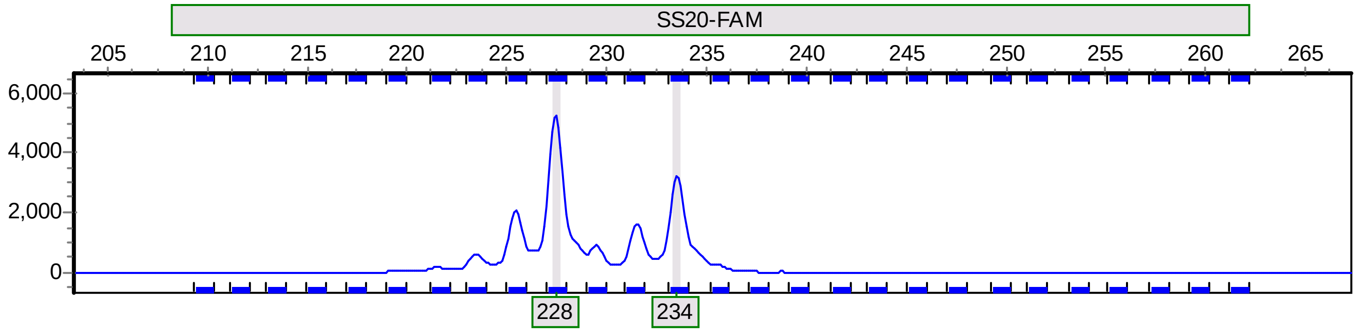

No	Size	Height	Area	Marker	Allele	Difference	Quality	Score	Allele Comments	Sample Comments
1	129.3	31692	275376	SSS13-FAM	129	0.00	Pass	500.0	[<Confirmed>]	
2	227.5	5205	42553	SS20-FAM	228	0.00	Pass	500.0	[<Confirmed>]	
3	233.5	3243	26498	SS20-FAM	234	0.10	Pass	466.6	[<Confirmed>]	

Sample 62: SSS13_SS20_SS11_SS21_SS02_SS19_HGY2_I13.fsa

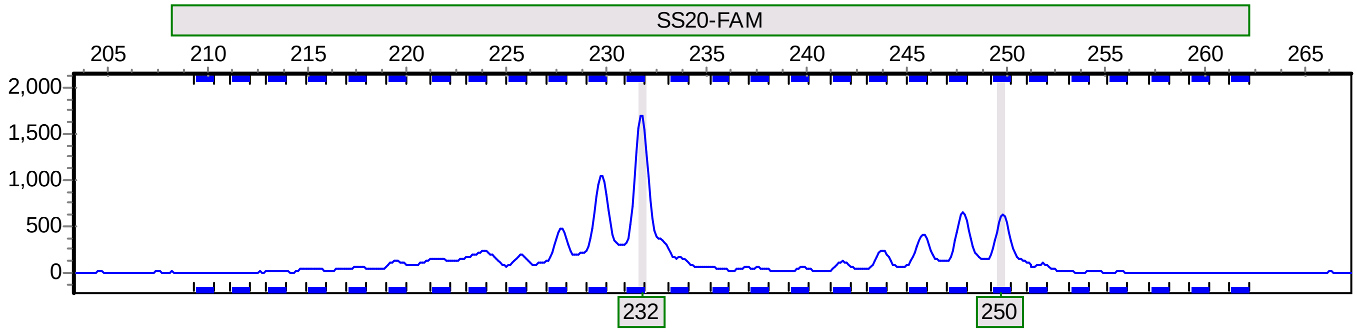

No	Size	Height	Area	Marker	Allele	Difference	Quality	Score	Allele Comments	Sample Comments
1	137.6	15463	107802	SSS13-FAM	137	0.10	Pass	500.0	[<Confirmed>]	
2	139.8	11132	86380	SSS13-FAM	139	0.30	Pass	500.0	[<Confirmed>]	
3	231.8	1690	13682	SS20-FAM	232	0.40	Pass	183.1	[<Confirmed>]	
4	247.8	652	5564	SS20-FAM	248	0.20	Undetermined	39.8	[<Deleted>]	
5	249.8	639	5004	SS20-FAM	250	0.00	Pass	40.1	[<Confirmed>]	

Sample 63: SSS13_SS20_SS11_SS21_SS02_SS19_HGY3_I11.fsa

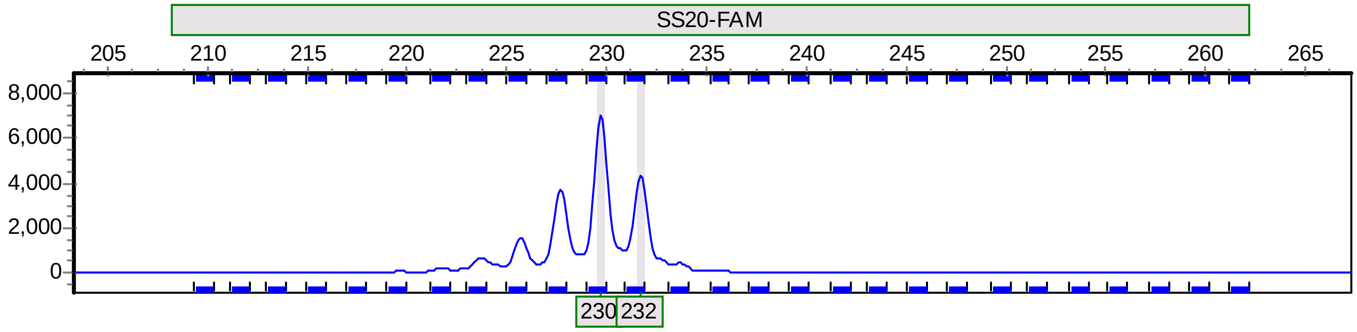

No	Size	Height	Area	Marker	Allele	Difference	Quality	Score	Allele Comments	Sample Comments
1	133.5	20041	137613	SSS13-FAM	133	0.10	Pass	500.0	[<Confirmed>]	
2	137.5	24327	166547	SSS13-FAM	137	0.00	Pass	500.0	[<Confirmed>]	
3	229.7	6991	54844	SS20-FAM	230	0.20	Pass	500.0	[<Confirmed>]	
4	231.7	4319	34633	SS20-FAM	232	0.30	Pass	500.0	[<Confirmed>]	

Allele Report

Sample 64: SSS13_SS20_SS11_SS21_SS02_SS19_HGY4_I09.fsa

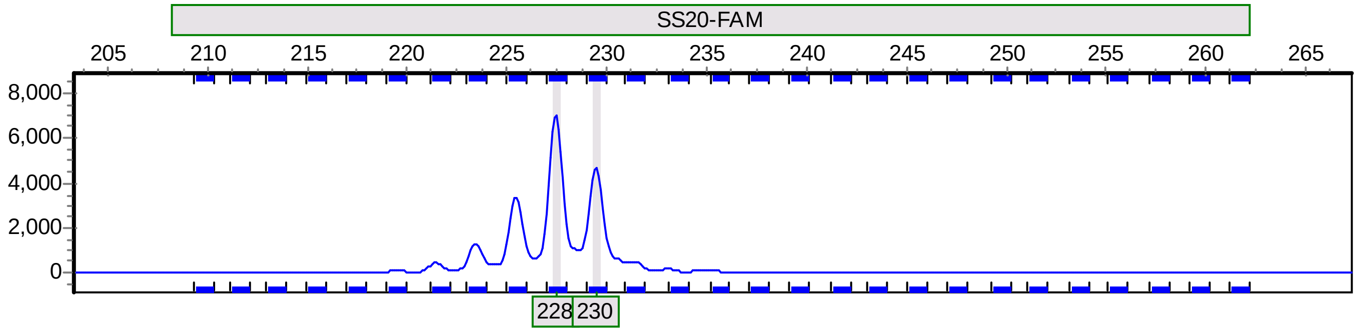

No	Size	Height	Area	Marker	Allele	Difference	Quality	Score	Allele Comments	Sample Comments
1	133.6	15305	104565	SSS13-FAM	133	0.20	Pass	500.0	[<Confirmed>]	
2	141.0	27831	206401	SSS13-FAM	141	0.50	Pass	500.0	[<Confirmed>]	
3	227.5	6986	54740	SS20-FAM	228	0.00	Pass	500.0	[<Confirmed>]	
4	229.5	4684	37235	SS20-FAM	230	0.00	Pass	500.0	[<Confirmed>]	

Sample 65: SSS13_SS20_SS11_SS21_SS02_SS19_HGY5_I15.fsa

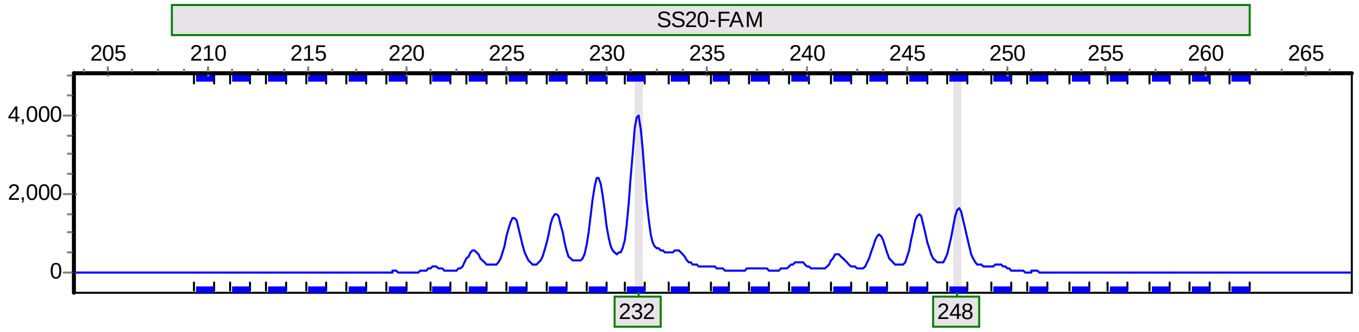

No	Size	Height	Area	Marker	Allele	Difference	Quality	Score	Allele Comments	Sample Comments
1	133.2	21750	149840	SSS13-FAM	133	0.20	Pass	500.0	[<Confirmed>]	
2	137.4	15551	109073	SSS13-FAM	137	0.10	Pass	500.0	[<Confirmed>]	
3	225.4	1407	12830	SS20-FAM	226	0.10	Undetermined	114.3	[<Deleted>]	
4	231.6	3983	32990	SS20-FAM	232	0.20	Pass	500.0	[<Confirmed>]	
5	245.6	1507	12315	SS20-FAM	246	0.00	Undetermined	153.7	[<Deleted>]	
6	247.6	1629	13907	SS20-FAM	248	0.00	Pass	160.2	[<Confirmed>]	

Sample 66: SSS13_SS20_SS11_SS21_SS02_SS19_HQZ11_O11.fsa

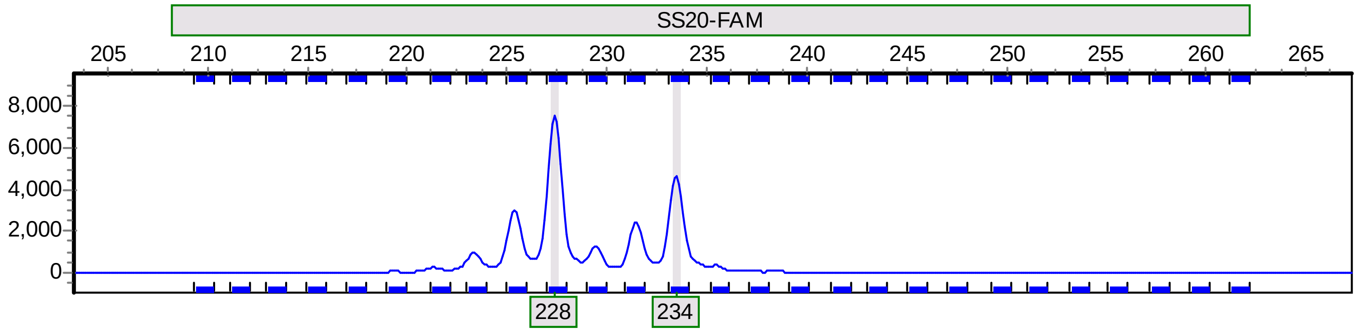

No	Size	Height	Area	Marker	Allele	Difference	Quality	Score	Allele Comments	Sample Comments
1	129.2	28615	194431	SSS13-FAM	129	0.10	Pass	500.0	[<Confirmed>]	
2	135.3	20654	140452	SSS13-FAM	135	0.10	Pass	500.0	[<Confirmed>]	
3	227.4	7483	61153	SS20-FAM	228	0.10	Pass	500.0	[<Confirmed>]	
4	233.5	4624	37315	SS20-FAM	234	0.10	Pass	500.0	[<Confirmed>]	

Allele Report

Sample 67: SSS13_SS20_SS11_SS21_SS02_SS19_HQZ13-1_C09.fsa

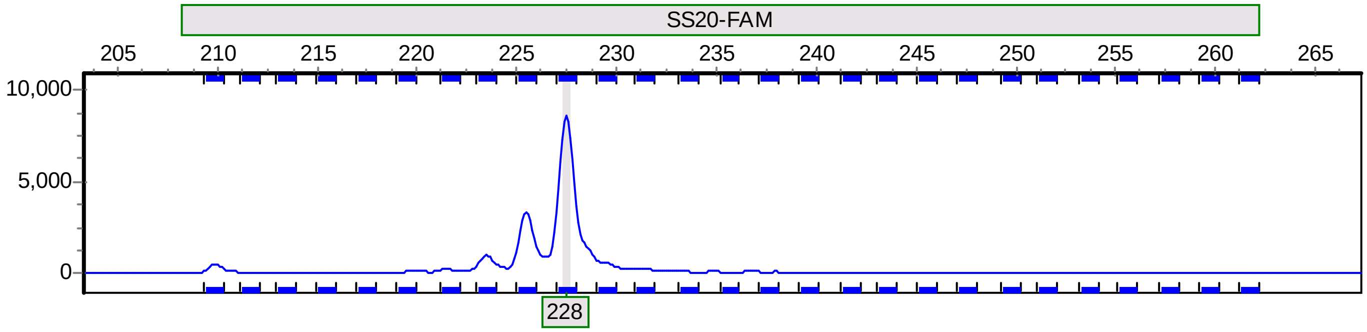

No	Size	Height	Area	Marker	Allele	Difference	Quality	Score	Allele Comments	Sample Comments
1	129.3	28302	199215	SSS13-FAM	129	0.00	Pass	500.0	[<Confirmed>]	
2	131.4	19189	132236	SSS13-FAM	131	0.10	Pass	500.0	[<Confirmed>]	
3	227.5	8515	72992	SS20-FAM	228	0.00	Pass	500.0	[<Confirmed>]	

Sample 68: SSS13_SS20_SS11_SS21_SS02_SS19_HQZ13-2_J03.fsa

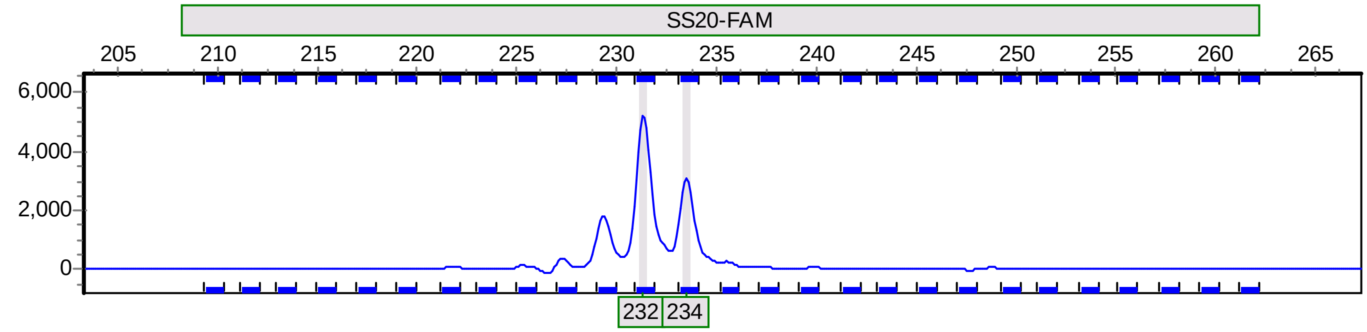

No	Size	Height	Area	Marker	Allele	Difference	Quality	Score	Allele Comments	Sample Comments
1	137.4	8163	56119	SSS13-FAM	137	0.10	Pass	500.0	[<Confirmed>]	
2	231.3	5176	43848	SS20-FAM	232	0.10	Pass	500.0	[<Confirmed>]	
3	233.5	3078	24849	SS20-FAM	234	0.10	Pass	442.1	[<Confirmed>]	

Sample 69: SSS13_SS20_SS11_SS21_SS02_SS19_HQZ14_H03.fsa

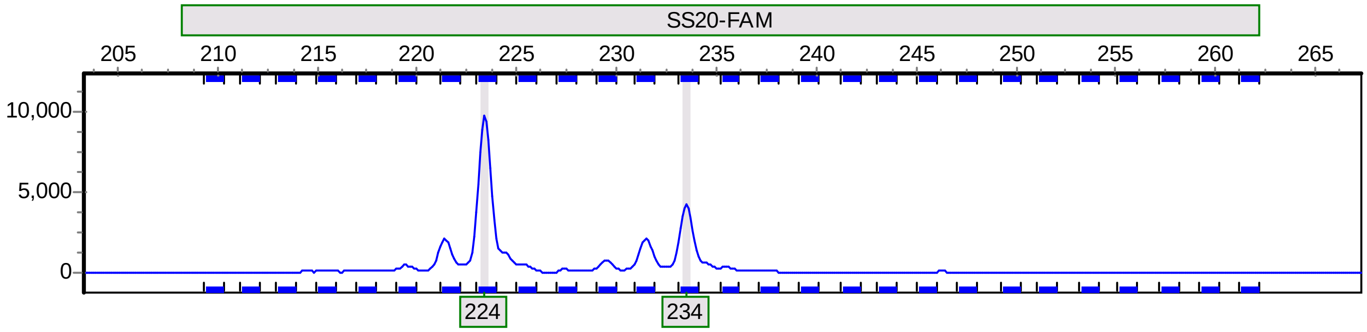

No	Size	Height	Area	Marker	Allele	Difference	Quality	Score	Allele Comments	Sample Comments
1	129.2	31406	215504	SSS13-FAM	129	0.10	Pass	500.0	[<Confirmed>]	
2	137.4	10448	69136	SSS13-FAM	137	0.10	Pass	500.0	[<Confirmed>]	
3	223.4	9685	70790	SS20-FAM	224	0.10	Pass	500.0	[<Confirmed>]	
4	233.5	4187	30910	SS20-FAM	234	0.10	Pass	500.0	[<Confirmed>]	

Allele Report

Sample 70: SSS13_SS20_SS11_SS21_SS02_SS19_HQZ15_B01.fsa

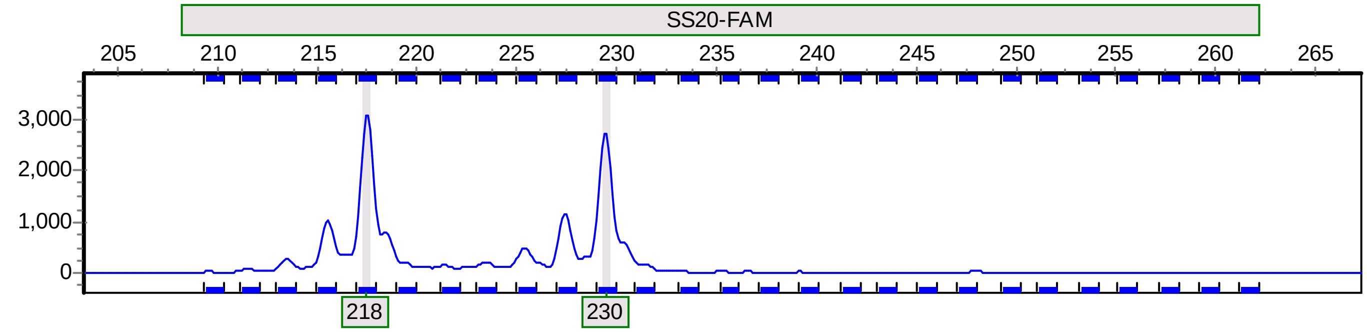

No	Size	Height	Area	Marker	Allele	Difference	Quality	Score	Allele Comments	Sample Comments
1	135.5	28819	191051	SSS13-FAM	135	0.10	Pass	500.0	[<Confirmed>]	
2	217.5	3051	22395	SS20-FAM	218	0.00	Pass	500.0	[<Confirmed>]	
3	229.5	2717	20109	SS20-FAM	230	0.00	Pass	422.0	[<Confirmed>]	

Sample 71: SSS13_SS20_SS11_SS21_SS02_SS19_HQZ16_P01.fsa

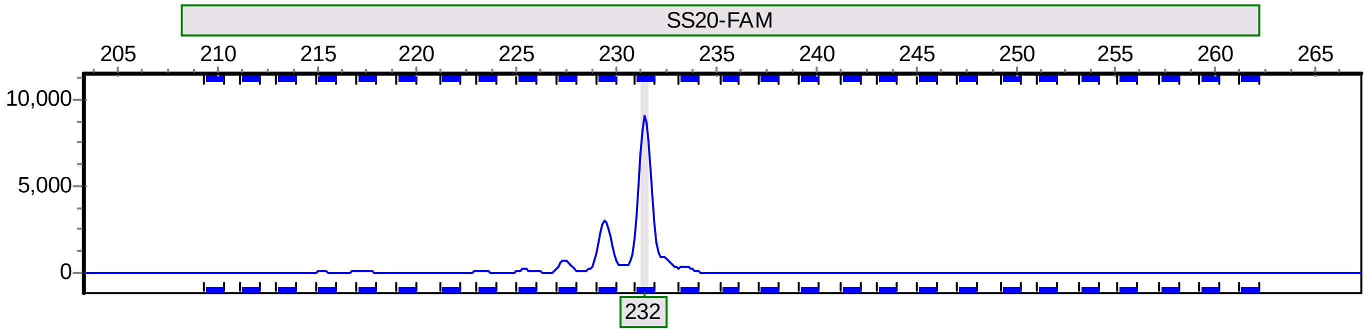

No	Size	Height	Area	Marker	Allele	Difference	Quality	Score	Allele Comments	Sample Comments
1	129.5	28745	179339	SSS13-FAM	129	0.20	Pass	500.0	[<Confirmed>]	
2	231.4	8977	63454	SS20-FAM	232	0.00	Pass	500.0	[<Confirmed>]	

Sample 72: SSS13_SS20_SS11_SS21_SS02_SS19_HQZ17-1_O03.fsa

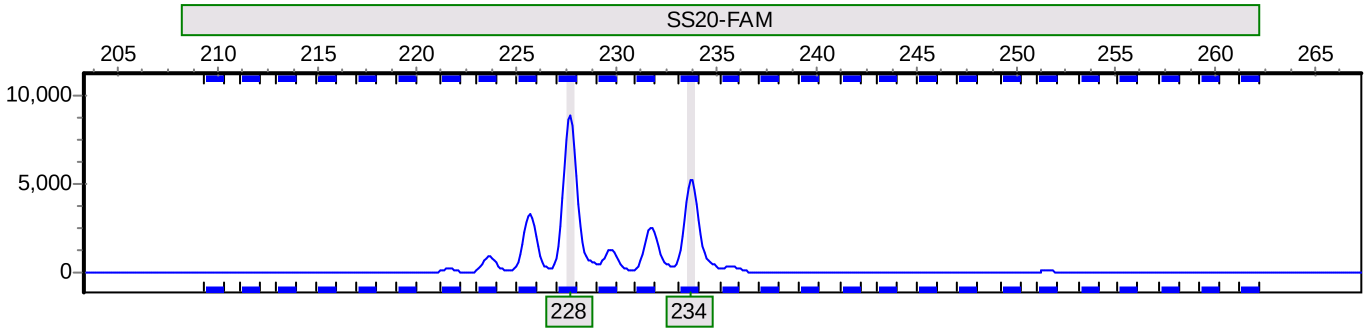

No	Size	Height	Area	Marker	Allele	Difference	Quality	Score	Allele Comments	Sample Comments
1	137.7	31430	221783	SSS13-FAM	137	0.20	Pass	500.0	[<Confirmed>]	
2	227.7	8791	65912	SS20-FAM	228	0.20	Pass	500.0	[<Confirmed>]	
3	233.7	5247	40553	SS20-FAM	234	0.10	Pass	500.0	[<Confirmed>]	

Allele Report

Sample 73: SSS13_SS20_SS11_SS21_SS02_SS19_HQZ17-2_N03.fsa

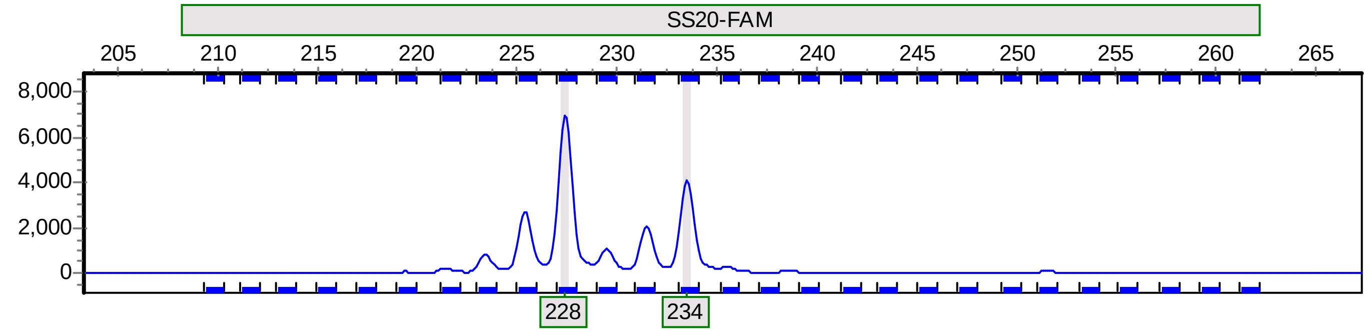

No	Size	Height	Area	Marker	Allele	Difference	Quality	Score	Allele Comments	Sample Comments
1	137.5	30952	210294	SSS13-FAM	137	0.00	Pass	500.0	[<Confirmed>]	
2	227.4	6928	53378	SS20-FAM	228	0.10	Pass	500.0	[<Confirmed>]	
3	233.5	4116	31889	SS20-FAM	234	0.10	Pass	500.0	[<Confirmed>]	

Sample 74: SSS13_SS20_SS11_SS21_SS02_SS19_HQZ18_K09.fsa

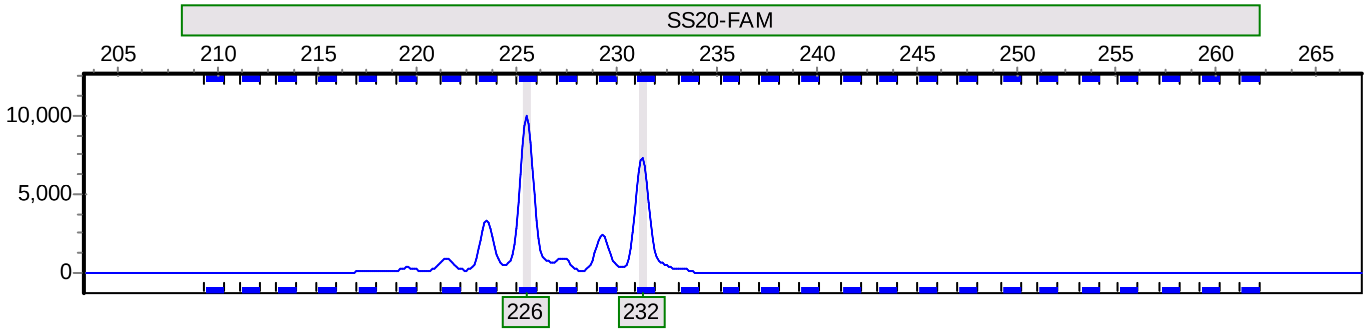

No	Size	Height	Area	Marker	Allele	Difference	Quality	Score	Allele Comments	Sample Comments
1	129.2	19989	133437	SSS13-FAM	129	0.10	Pass	500.0	[<Confirmed>]	
2	144.4	22849	166095	SSS13-FAM	143	1.00	Pass	500.0	[<Confirmed><Edited>]	
3	225.5	9916	76212	SS20-FAM	226	0.00	Pass	500.0	[<Confirmed>]	
4	231.3	7354	56092	SS20-FAM	232	0.10	Pass	500.0	[<Confirmed>]	

Sample 75: SSS13_SS20_SS11_SS21_SS02_SS19_HQZ19_L03.fsa

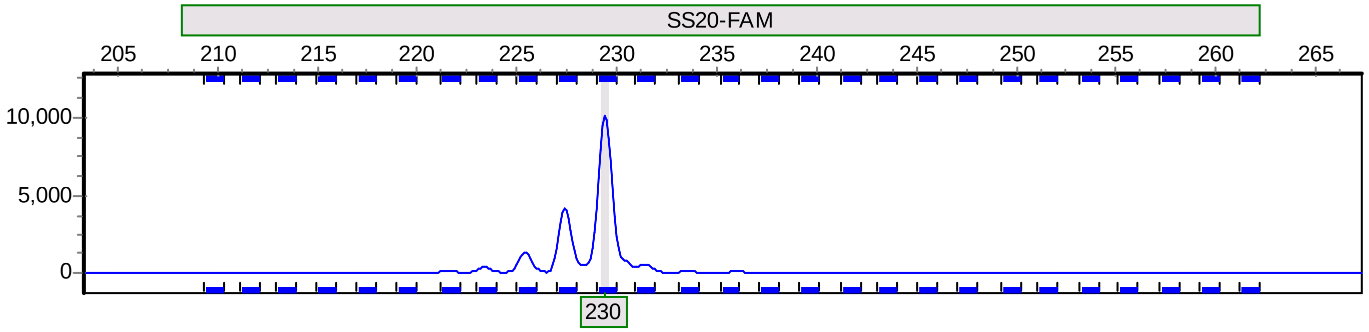

No	Size	Height	Area	Marker	Allele	Difference	Quality	Score	Allele Comments	Sample Comments
1	137.5	18250	121986	SSS13-FAM	137	0.00	Pass	500.0	[<Confirmed>]	
2	144.4	12537	91481	SSS13-FAM	143	1.00	Pass	500.0	[<Confirmed><Edited>]	
3	229.4	10086	77336	SS20-FAM	230	0.10	Pass	500.0	[<Confirmed>]	

Sample 76: SSS13_SS20_SS11_SS21_SS02_SS19_HQZ21_B03.fsa Run date and time: 10/09/2021 - 22:22:10 -> 10/09/2021 - 22:49:12

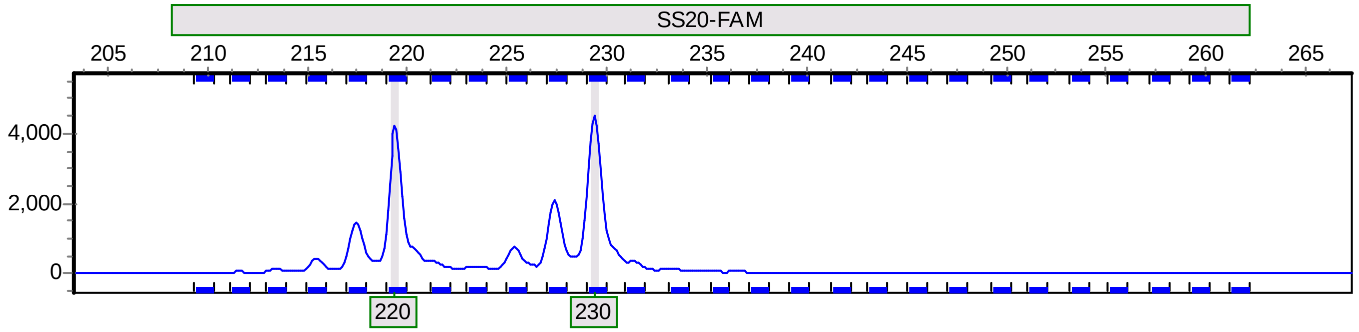

No	Size	Height	Area	Marker	Allele	Difference	Quality	Score	Allele Comments	Sample Comments
1	137.4	17500	120084	SSS13-FAM	137	0.10	Pass	500.0	[<Confirmed>]	
2	144.3	13098	98752	SSS13-FAM	143	0.50	Pass	500.0	[<Confirmed>]	
3	219.4	4284	32779	SS20-FAM	220	0.10	Pass	500.0	[<Confirmed>]	
4	229.4	4523	35049	SS20-FAM	230	0.10	Pass	500.0	[<Confirmed>]	

Sample 77: SSS13_SS20_SS11_SS21_SS02_SS19_HQZ22-1_M01.fsa

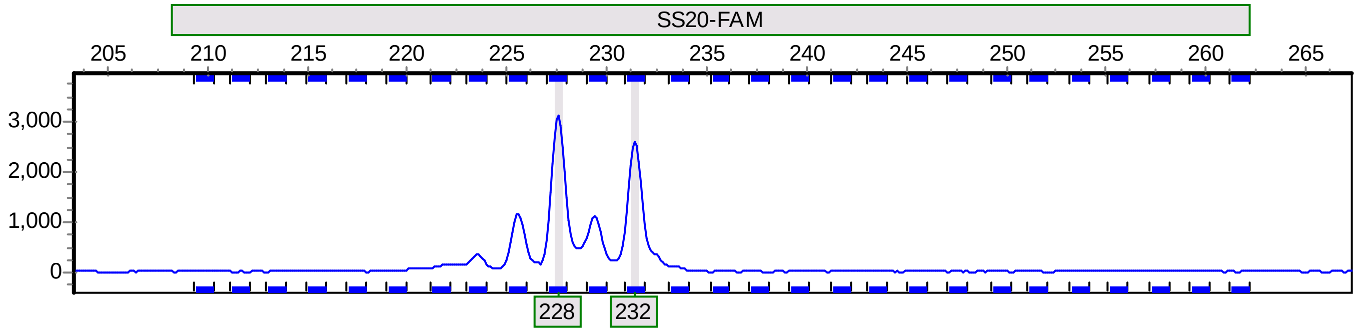

No	Size	Height	Area	Marker	Allele	Difference	Quality	Score	Allele Comments	Sample Comments
1	135.6	11783	76315	SSS13-FAM	135	0.20	Pass	500.0	[<Confirmed>]	
2	144.6	9093	64815	SSS13-FAM	143	1.00	Pass	500.0	[<Confirmed><Edited>]	
3	227.6	3108	23735	SS20-FAM	228	0.10	Pass	494.4	[<Confirmed>]	
4	231.4	2590	20749	SS20-FAM	232	0.00	Pass	381.3	[<Confirmed>]	

Sample 78: SSS13_SS20_SS11_SS21_SS02_SS19_HQZ22-2_O13.fsa

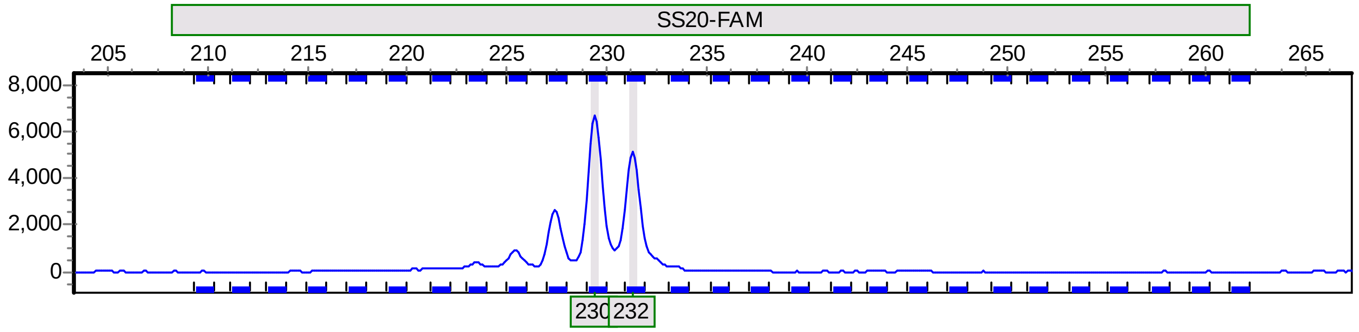

No	Size	Height	Area	Marker	Allele	Difference	Quality	Score	Allele Comments	Sample Comments
1	129.3	21568	146026	SSS13-FAM	129	0.00	Pass	500.0	[<Confirmed>]	
2	144.4	12256	92456	SSS13-FAM	143	1.00	Pass	500.0	[<Confirmed><Edited>]	
3	229.4	6636	53532	SS20-FAM	230	0.10	Pass	500.0	[<Confirmed>]	
4	231.3	5136	40917	SS20-FAM	232	0.10	Pass	500.0	[<Confirmed>]	

Allele Report

Sample 79: SSS13_SS20_SS11_SS21_SS02_SS19_HQZ23_A15.fsa

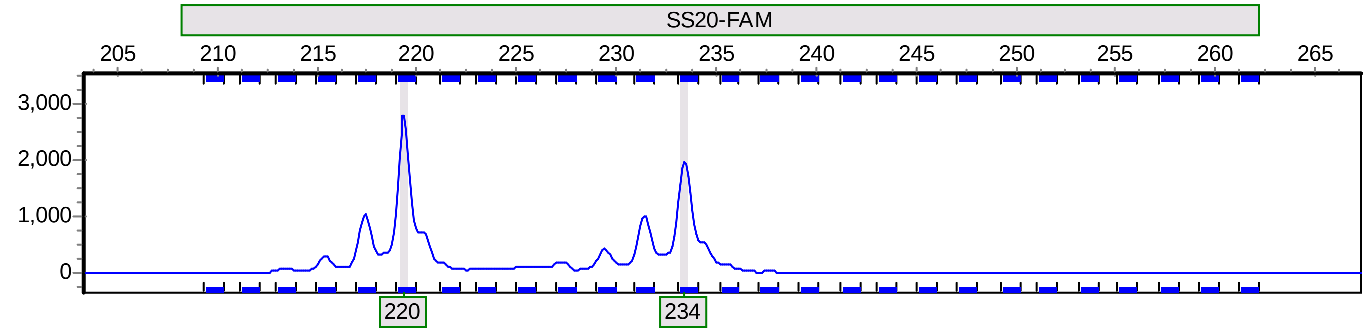

No	Size	Height	Area	Marker	Allele	Difference	Quality	Score	Allele Comments	Sample Comments
1	133.4	31191	217168	SSS13-FAM	133	0.00	Pass	500.0	[<Confirmed>]	
2	219.4	2756	20777	SS20-FAM	220	0.10	Pass	409.6	[<Confirmed>]	
3	233.4	1951	15747	SS20-FAM	234	0.20	Pass	237.6	[<Confirmed>]	

Sample 80: SSS13_SS20_SS11_SS21_SS02_SS19_HQZ24_E11.fsa

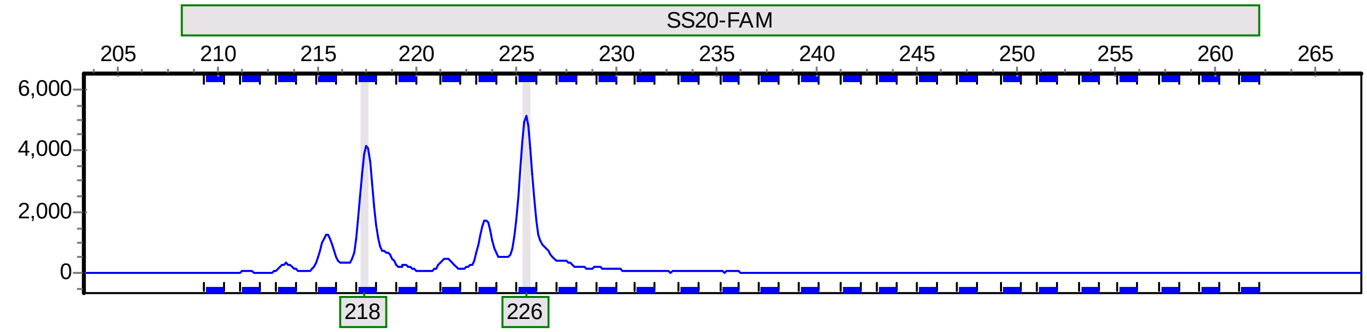

No	Size	Height	Area	Marker	Allele	Difference	Quality	Score	Allele Comments	Sample Comments
1	146.6	24196	175005	SSS13-FAM	147	0.10	Pass	500.0	[<Confirmed>]	
2	217.4	4147	32186	SS20-FAM	218	0.10	Pass	500.0	[<Confirmed>]	
3	225.5	5104	39197	SS20-FAM	226	0.00	Pass	500.0	[<Confirmed>]	

Sample 81: SSS13_SS20_SS11_SS21_SS02_SS19_HQZ25_N01.fsa

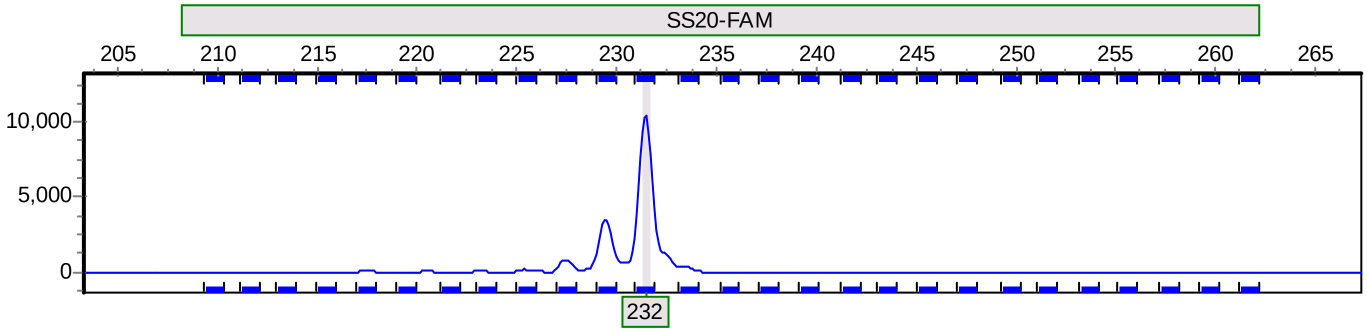

No	Size	Height	Area	Marker	Allele	Difference	Quality	Score	Allele Comments	Sample Comments
1	129.2	19711	125431	SSS13-FAM	129	0.10	Pass	500.0	[<Confirmed>]	
2	146.6	10128	70431	SSS13-FAM	147	0.10	Pass	500.0	[<Confirmed>]	
3	231.5	10318	79106	SS20-FAM	232	0.10	Pass	500.0	[<Confirmed>]	

Allele Report

Sample 82: SSS13_SS20_SS11_SS21_SS02_SS19_HQZ26_E03.fsa

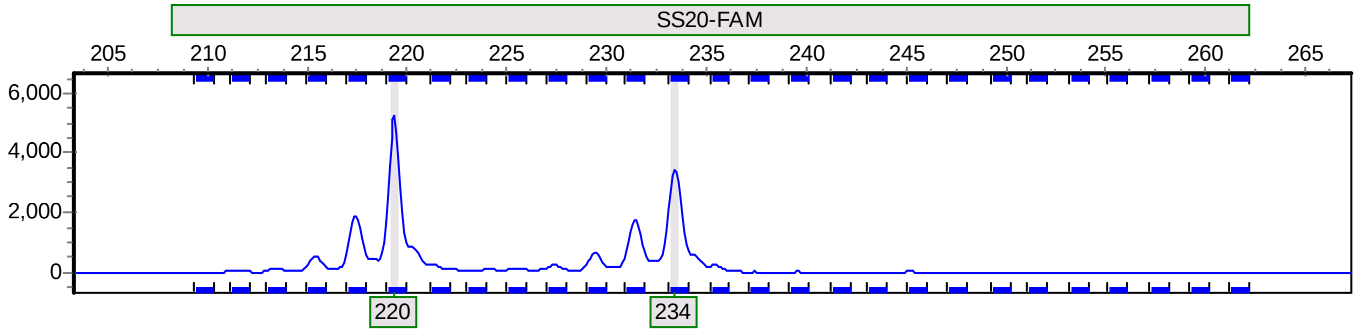

No	Size	Height	Area	Marker	Allele	Difference	Quality	Score	Allele Comments	Sample Comments
1	135.4	31156	213608	SSS13-FAM	135	0.00	Pass	500.0	[<Confirmed>]	
2	219.4	5212	38180	SS20-FAM	220	0.10	Pass	500.0	[<Confirmed>]	
3	233.4	3446	26773	SS20-FAM	234	0.20	Pass	500.0	[<Confirmed>]	

Sample 83: SSS13_SS20_SS11_SS21_SS02_SS19_HQZ27_G09.fsa

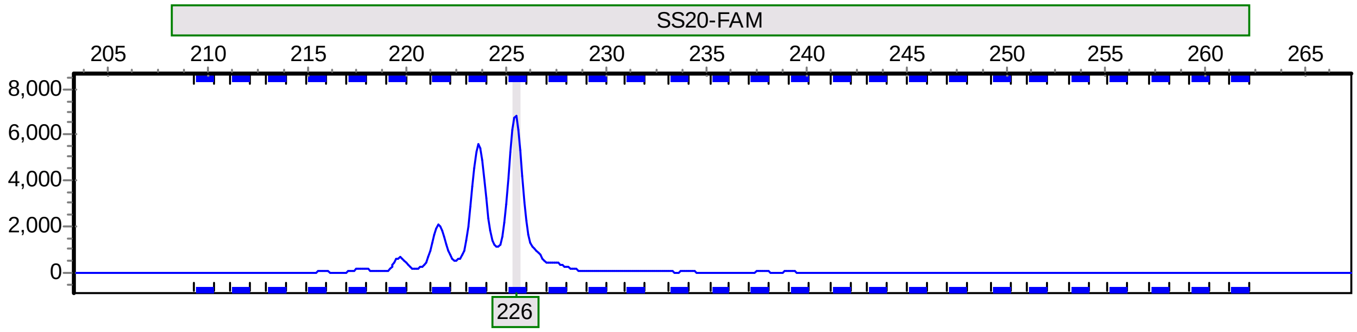

No	Size	Height	Area	Marker	Allele	Difference	Quality	Score	Allele Comments	Sample Comments
1	127.5	32018	242169	SSS13-FAM	127	0.30	Pass	500.0	[<Confirmed>]	
2	225.5	6793	54992	SS20-FAM	226	0.00	Pass	500.0	[<Confirmed>]	

Sample 84: SSS13_SS20_SS11_SS21_SS02_SS19_HQZ28_B05.fsa

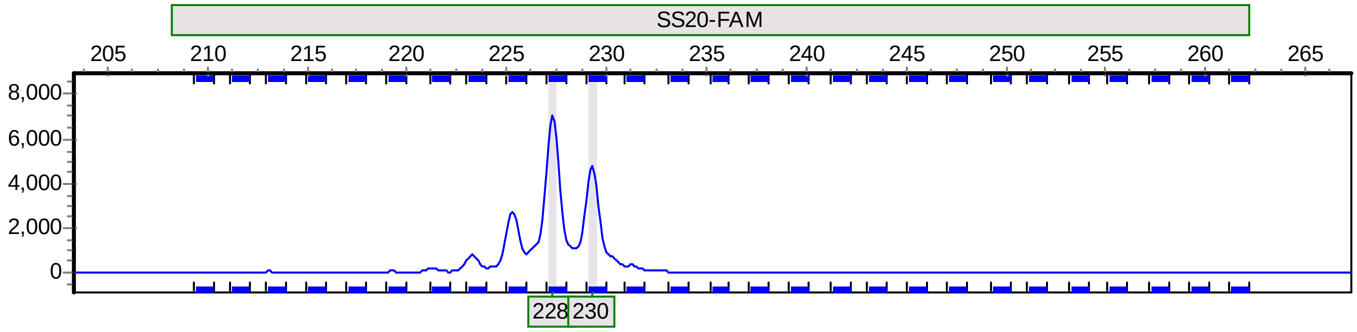

No	Size	Height	Area	Marker	Allele	Difference	Quality	Score	Allele Comments	Sample Comments
1	127.1	33165	227107	SSS13-FAM	127	0.10	Pass	500.0	[<SAT (Repaired)><Confirmed>]	
2	144.3	17088	125142	SSS13-FAM	143	0.50	Pass	500.0	[<Confirmed>]	
3	227.3	7006	55470	SS20-FAM	228	0.20	Pass	500.0	[<Confirmed>]	
4	229.3	4748	35990	SS20-FAM	230	0.20	Pass	500.0	[<Confirmed>]	

Sample 85: SSS13_SS20_SS11_SS21_SS02_SS19_HQZ29_C13.fsa

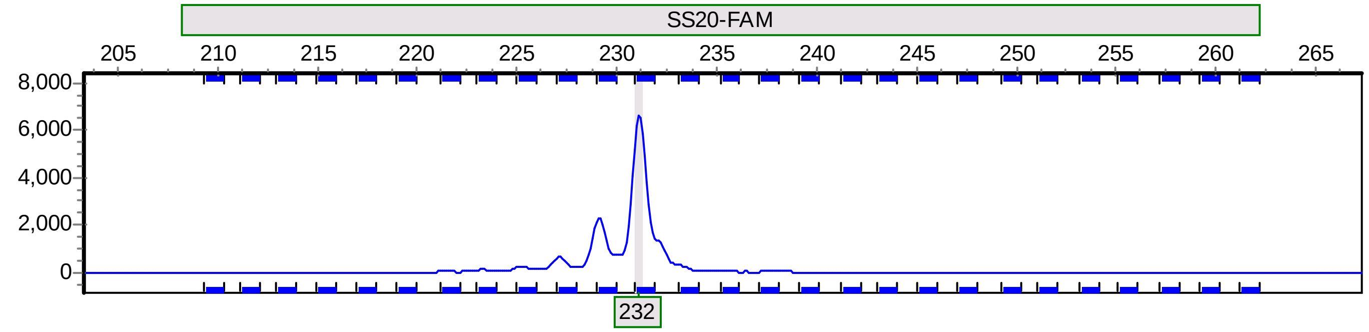

No	Size	Height	Area	Marker	Allele	Difference	Quality	Score	Allele Comments	Sample Comments
1	129.1	28582	191559	SSS13-FAM	129	0.20	Pass	500.0	[<Confirmed>]	
2	231.1	6575	53242	SS20-FAM	232	0.30	Pass	500.0	[<Confirmed>]	

Sample 86: SSS13_SS20_SS11_SS21_SS02_SS19_HQZ2_K03.fsa

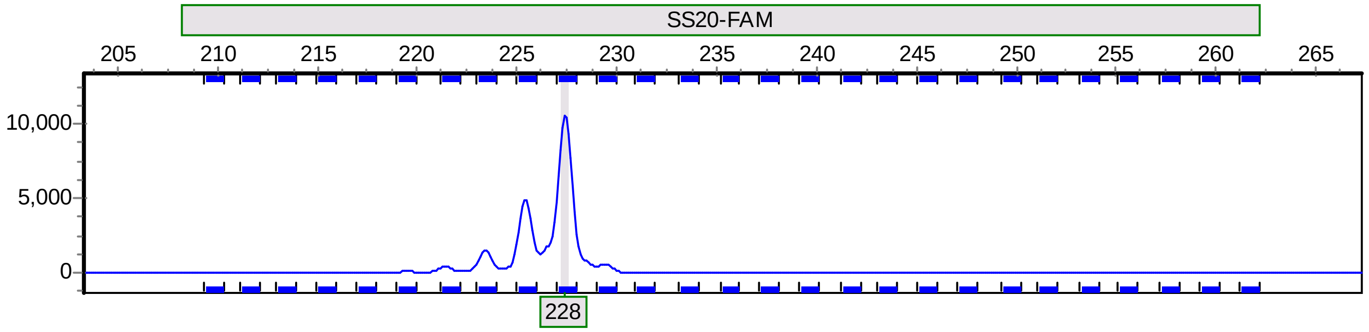

No	Size	Height	Area	Marker	Allele	Difference	Quality	Score	Allele Comments	Sample Comments
1	129.4	30950	233568	SSS13-FAM	129	0.10	Pass	500.0	[<Confirmed>]	
2	227.4	10499	82980	SS20-FAM	228	0.10	Pass	500.0	[<Confirmed>]	

Sample 87: SSS13_SS20_SS11_SS21_SS02_SS19_HQZ30_G15.fsa

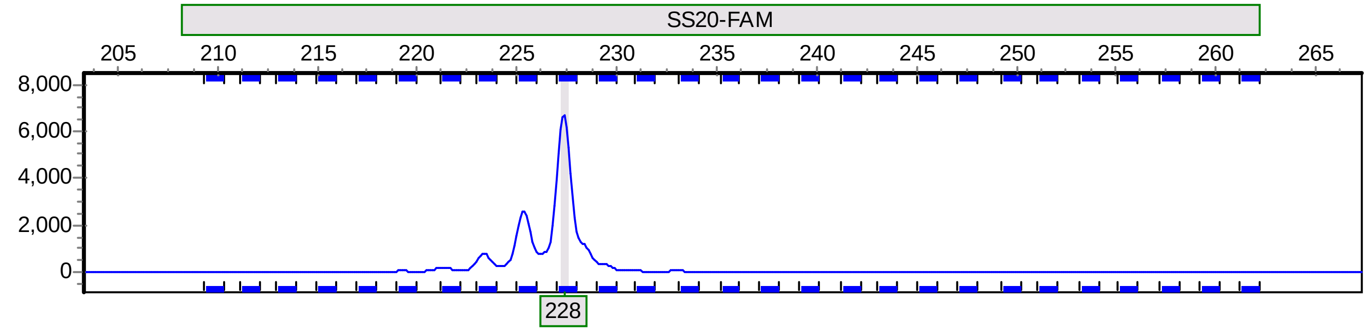

No	Size	Height	Area	Marker	Allele	Difference	Quality	Score	Allele Comments	Sample Comments
1	127.0	31248	228035	SSS13-FAM	127	0.20	Pass	500.0	[<Confirmed>]	
2	129.0	12030	85873	SSS13-FAM	129	0.30	Pass	500.0	[<Confirmed>]	
3	227.4	6660	54855	SS20-FAM	228	0.10	Pass	500.0	[<Confirmed>]	

Allele Report

Sample 88: SSS13_SS20_SS11_SS21_SS02_SS19_HQZ31_K13.fsa

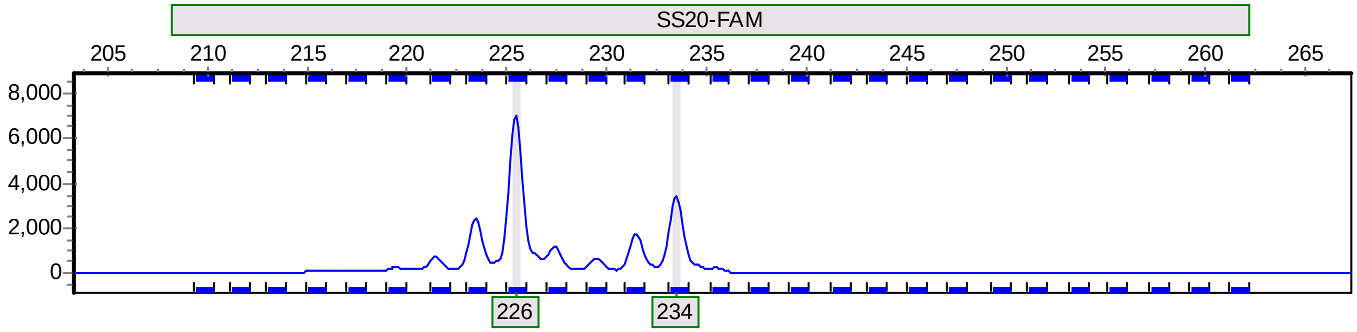

No	Size	Height	Area	Marker	Allele	Difference	Quality	Score	Allele Comments	Sample Comments
1	129.2	22471	150698	SSS13-FAM	129	0.10	Pass	500.0	[<Confirmed>]	
2	135.3	16906	113851	SSS13-FAM	135	0.10	Pass	500.0	[<Confirmed>]	
3	225.5	6965	53105	SS20-FAM	226	0.00	Pass	500.0	[<Confirmed>]	
4	233.5	3405	26540	SS20-FAM	234	0.10	Pass	500.0	[<Confirmed>]	

Sample 89: SSS13_SS20_SS11_SS21_SS02_SS19_HQZ32_J01.fsa

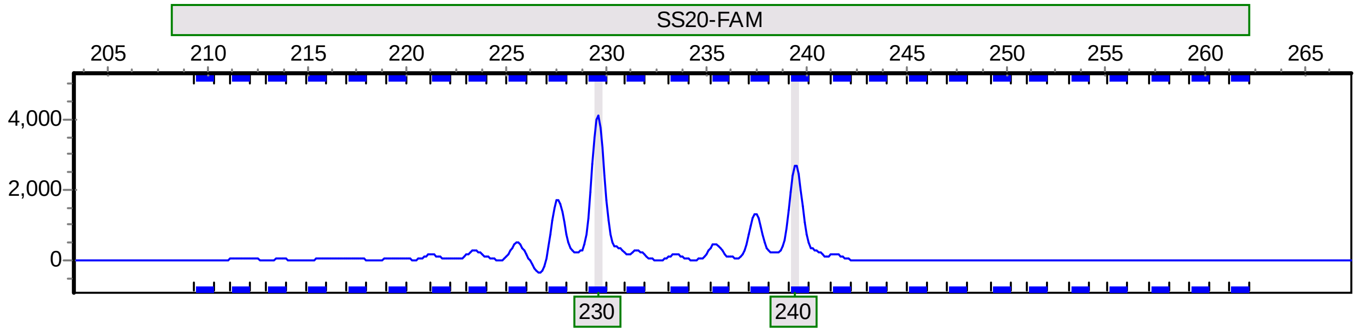

No	Size	Height	Area	Marker	Allele	Difference	Quality	Score	Allele Comments	Sample Comments
1	144.5	20540	145952	SSS13-FAM	143	1.00	Pass	500.0	[<Confirmed><Edited>]	
2	229.6	4085	30141	SS20-FAM	230	0.10	Pass	500.0	[<Confirmed>]	
3	239.5	2680	20516	SS20-FAM	240	0.20	Pass	424.2	[<Confirmed>]	

Sample 90: SSS13_SS20_SS11_SS21_SS02_SS19_HQZ33_I03.fsa

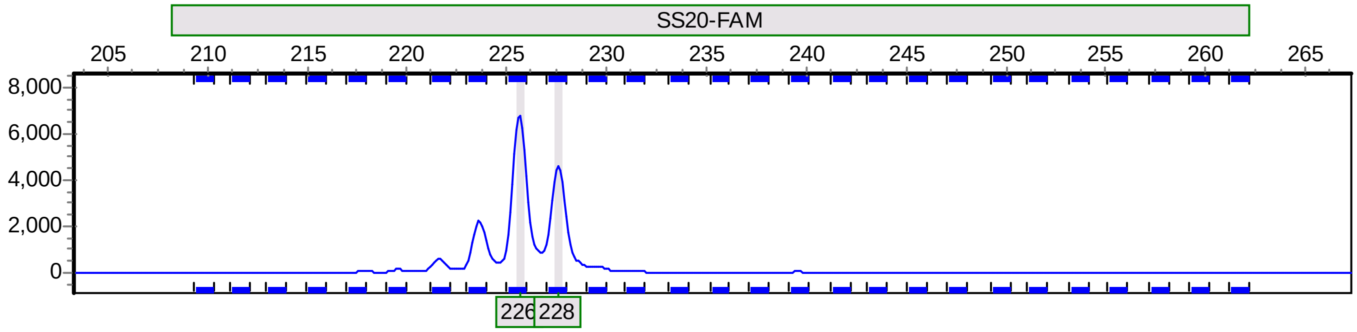

No	Size	Height	Area	Marker	Allele	Difference	Quality	Score	Allele Comments	Sample Comments
1	127.1	21198	137475	SSS13-FAM	127	0.10	Pass	500.0	[<Confirmed>]	
2	137.6	12268	82392	SSS13-FAM	137	0.10	Pass	500.0	[<Confirmed>]	
3	225.7	6774	53788	SS20-FAM	226	0.20	Pass	500.0	[<Confirmed>]	
4	227.6	4676	37450	SS20-FAM	228	0.10	Pass	500.0	[<Confirmed>]	

Allele Report

Sample 91: SSS13_SS20_SS11_SS21_SS02_SS19_HQZ34_M03.fsa

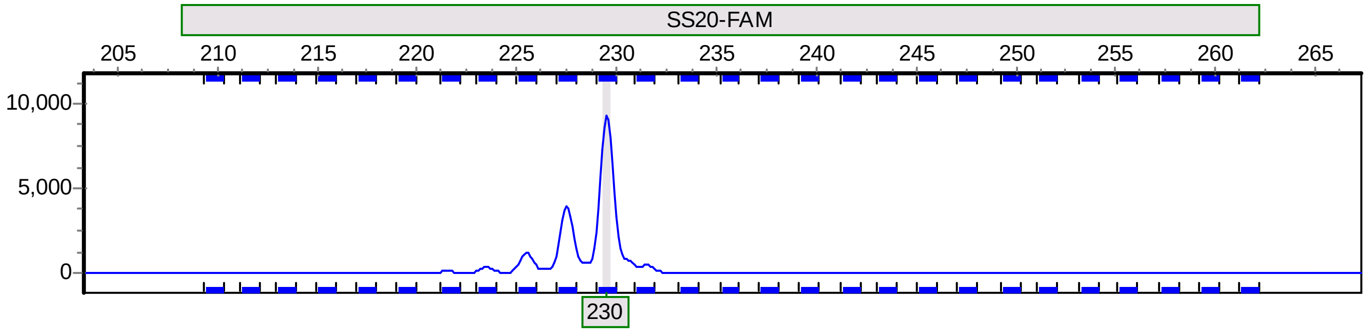

No	Size	Height	Area	Marker	Allele	Difference	Quality	Score	Allele Comments	Sample Comments
1	137.4	16136	108401	SSS13-FAM	137	0.10	Pass	500.0	[<Confirmed>]	
2	139.6	13419	103712	SSS13-FAM	139	0.10	Pass	500.0	[<Confirmed>]	
3	146.5	9295	67638	SSS13-FAM	147	0.00	Pass	500.0	[<Confirmed><Edited><Deleted>]	
4	229.5	9228	70525	SS20-FAM	230	0.00	Pass	500.0	[<Confirmed>]	

Sample 92: SSS13_SS20_SS11_SS21_SS02_SS19_HQZ35_A01.fsa

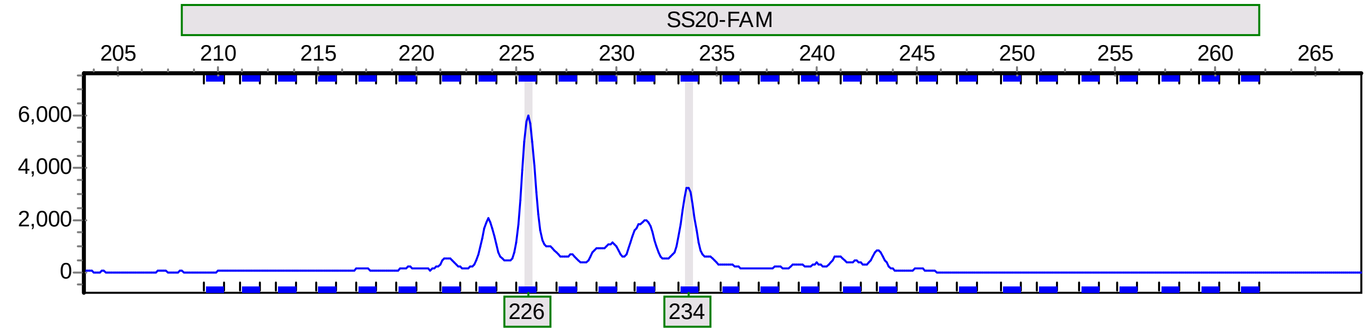

No	Size	Height	Area	Marker	Allele	Difference	Quality	Score	Allele Comments	Sample Comments
1	135.3	3427	25181	SSS13-FAM	135	0.10	Pass	495.8	[<Confirmed>]	
2	225.6	5973	47335	SS20-FAM	226	0.10	Pass	500.0	[<Confirmed>]	
3	233.6	3266	26443	SS20-FAM	234	0.00	Pass	479.0	[<Confirmed>]	

Sample 93: SSS13_SS20_SS11_SS21_SS02_SS19_HQZ36_E15.fsa

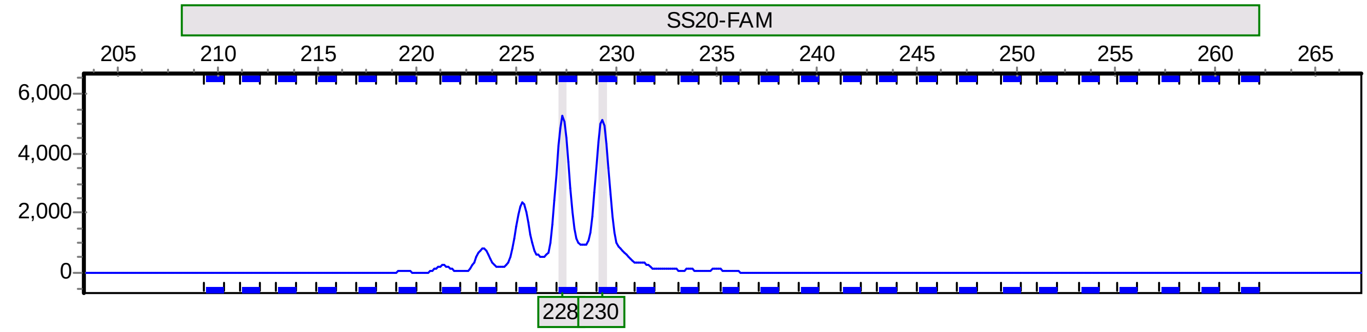

No	Size	Height	Area	Marker	Allele	Difference	Quality	Score	Allele Comments	Sample Comments
1	144.1	22456	175696	SSS13-FAM	143	0.30	Pass	500.0	[<Confirmed>]	
2	146.3	7777	63002	SSS13-FAM	147	0.20	Pass	500.0	[<Confirmed>]	
3	227.3	5219	40966	SS20-FAM	228	0.20	Pass	500.0	[<Confirmed>]	
4	229.3	5143	42453	SS20-FAM	230	0.20	Pass	500.0	[<Confirmed>]	

Sample 94: SSS13_SS20_SS11_SS21_SS02_SS19_HQZ37_F03.fsa

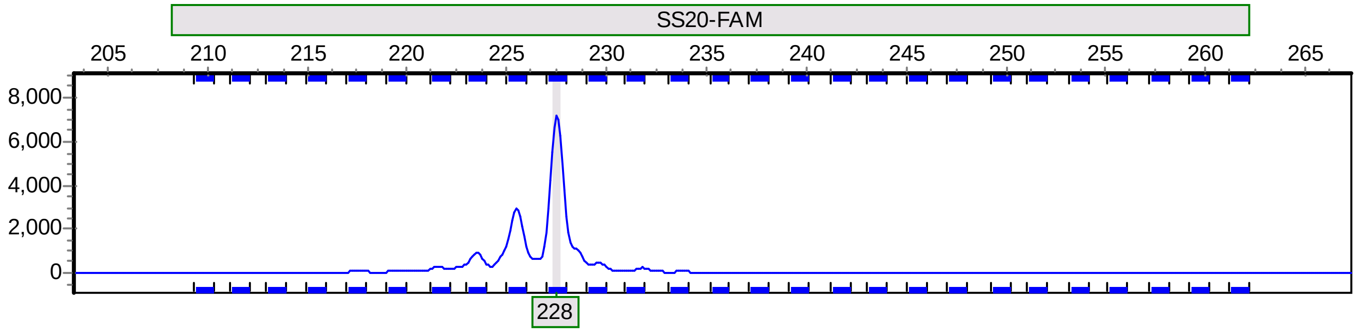

No	Size	Height	Area	Marker	Allele	Difference	Quality	Score	Allele Comments	Sample Comments
1	127.2	37459	264699	SSS13-FAM	127	0.00	Pass	500.0	[<SAT (Repaired)><Confirmed>]	
2	227.5	7142	55365	SS20-FAM	228	0.00	Pass	500.0	[<Confirmed>]	

Sample 95: SSS13_SS20_SS11_SS21_SS02_SS19_HQZ38_L01.fsa

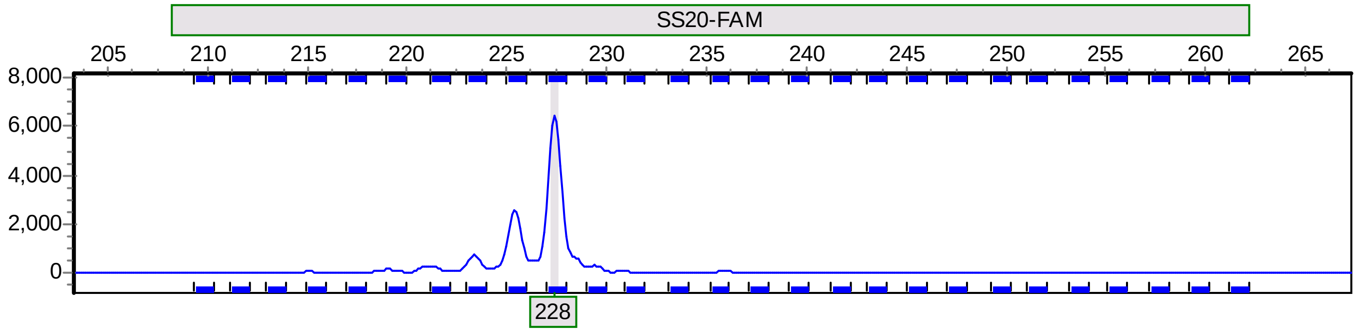

No	Size	Height	Area	Marker	Allele	Difference	Quality	Score	Allele Comments	Sample Comments
1	129.2	18782	122437	SSS13-FAM	129	0.10	Pass	500.0	[<Confirmed>]	
2	146.6	11054	80528	SSS13-FAM	147	0.10	Pass	500.0	[<Confirmed>]	
3	227.4	6405	48864	SS20-FAM	228	0.10	Pass	500.0	[<Confirmed>]	

Sample 96: SSS13_SS20_SS11_SS21_SS02_SS19_HQZ39_M15.fsa

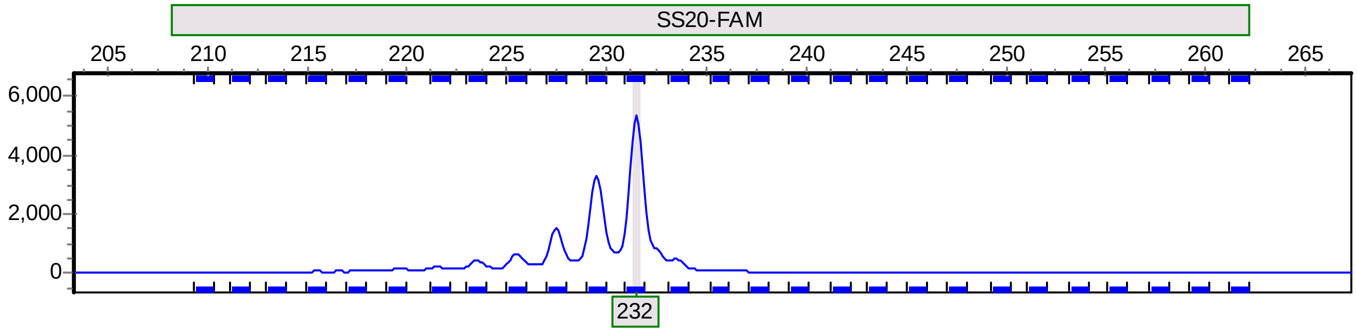

No	Size	Height	Area	Marker	Allele	Difference	Quality	Score	Allele Comments	Sample Comments
1	133.4	25349	177210	SSS13-FAM	133	0.00	Pass	500.0	[<Confirmed>]	
2	135.5	22046	153704	SSS13-FAM	135	0.10	Pass	500.0	[<Confirmed>]	
3	231.5	5320	43785	SS20-FAM	232	0.10	Pass	500.0	[<Confirmed>]	

Sample 97: SSS13_SS20_SS11_SS21_SS02_SS19_HQZ7_C03.fsa

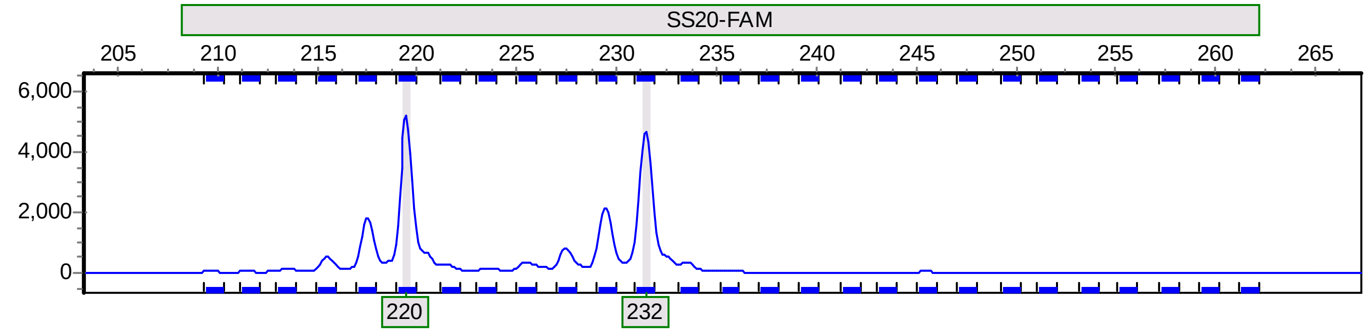

No	Size	Height	Area	Marker	Allele	Difference	Quality	Score	Allele Comments	Sample Comments
1	129.3	27348	175914	SSS13-FAM	129	0.00	Pass	500.0	[<Confirmed>]	
2	137.5	19714	128328	SSS13-FAM	137	0.00	Pass	500.0	[<Confirmed>]	
3	219.5	5185	38456	SS20-FAM	220	0.00	Pass	500.0	[<Confirmed>]	
4	231.5	4688	35505	SS20-FAM	232	0.10	Pass	500.0	[<Confirmed>]	

Sample 98: SSS13_SS20_SS11_SS21_SS02_SS19_HQZ9_K11.fsa

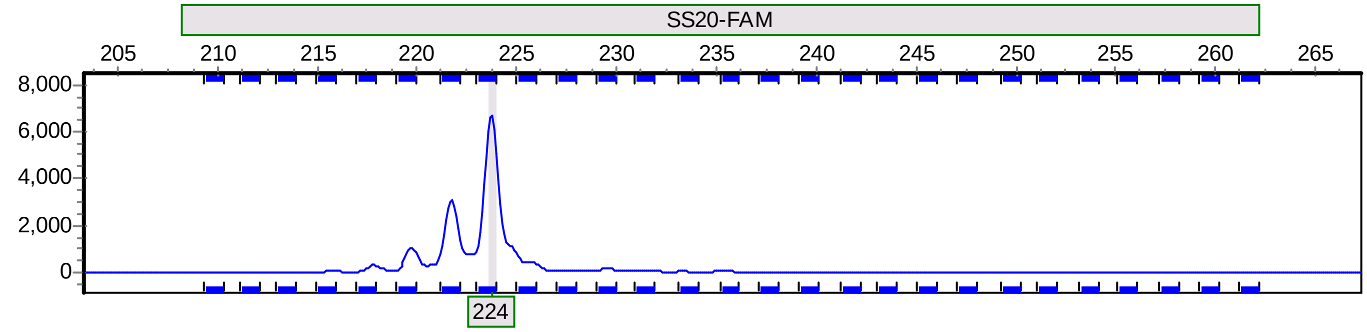

No	Size	Height	Area	Marker	Allele	Difference	Quality	Score	Allele Comments	Sample Comments
1	137.2	30379	207964	SSS13-FAM	137	0.30	Pass	500.0	[<Confirmed>]	
2	144.2	14064	103477	SSS13-FAM	143	0.40	Pass	500.0	[<Confirmed>]	
3	223.8	6656	52484	SS20-FAM	224	0.30	Pass	500.0	[<Confirmed>]	

Sample 99: SSS13_SS20_SS11_SS21_SS02_SS19_HRS24_I17.fsa

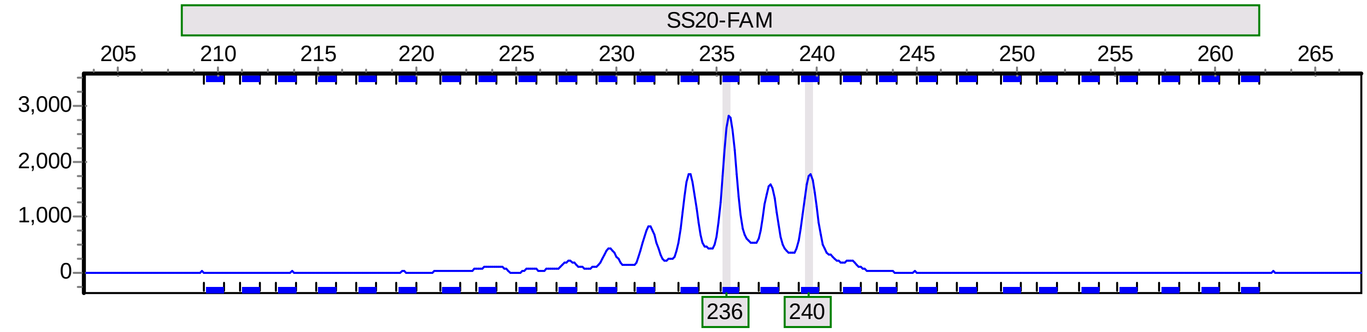

No	Size	Height	Area	Marker	Allele	Difference	Quality	Score	Allele Comments	Sample Comments
1	136.2	22919	161656	SSS13-FAM	135	1.00	Pass	500.0	[<Confirmed><Edited>]	
2	141.6	14610	114071	SSS13-FAM	141	0.10	Pass	500.0	[<Confirmed>]	
3	235.6	2806	23932	SS20-FAM	236	0.10	Pass	368.6	[<Confirmed>]	
4	239.7	1771	14699	SS20-FAM	240	0.00	Pass	179.7	[<Confirmed>]	

Allele Report

Sample 100: SSS13_SS20_SS11_SS21_SS02_SS19_HRS26_H09.fsa

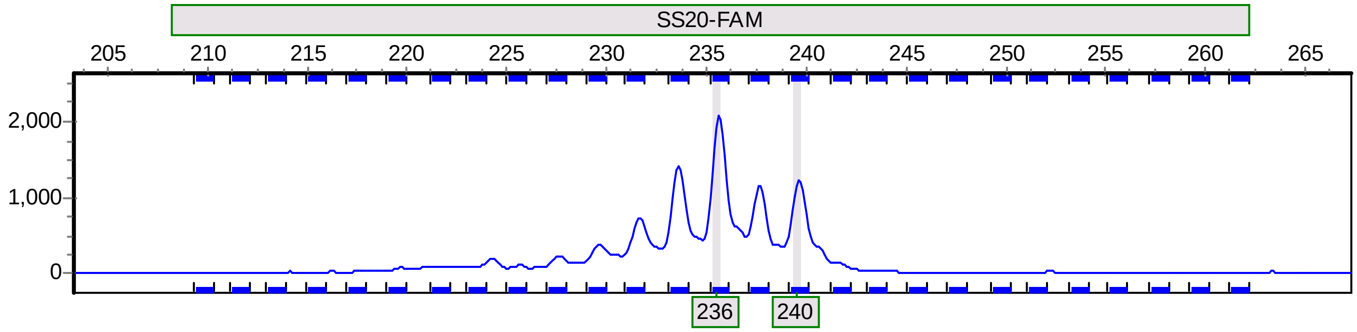

No	Size	Height	Area	Marker	Allele	Difference	Quality	Score	Allele Comments	Sample Comments
1	133.2	18798	129320	SSS13-FAM	133	0.20	Pass	500.0	[<Confirmed>]	
2	136.5	20326	141314	SSS13-FAM	137	1.00	Pass	500.0	[<Confirmed><Edited>]	
3	235.6	2075	17048	SS20-FAM	236	0.10	Pass	243.2	[<Confirmed>]	
4	237.7	1142	9322	SS20-FAM	238	0.00	Undetermined	89.2	[<Deleted>]	
5	239.6	1224	10388	SS20-FAM	240	0.10	Pass	97.0	[<Confirmed>]	

Sample 101: SSS13_SS20_SS11_SS21_SS02_SS19_HRS28_D13.fsa

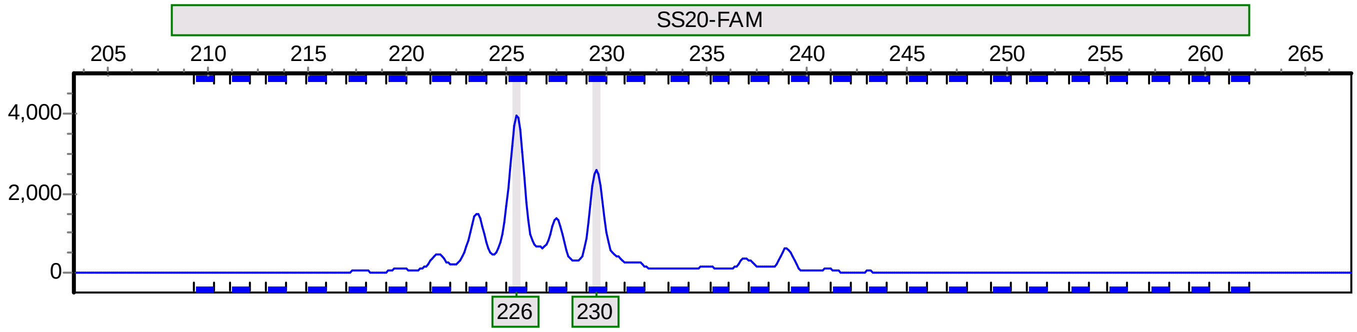

No	Size	Height	Area	Marker	Allele	Difference	Quality	Score	Allele Comments	Sample Comments
1	129.4	4580	32232	SSS13-FAM	129	0.10	Pass	500.0	[<Confirmed>]	
2	225.5	3952	34677	SS20-FAM	226	0.00	Pass	500.0	[<Confirmed>]	
3	229.5	2623	21415	SS20-FAM	230	0.00	Pass	368.8	[<Confirmed>]	

Sample 102: SSS13_SS20_SS11_SS21_SS02_SS19_HRS29_J05.fsa

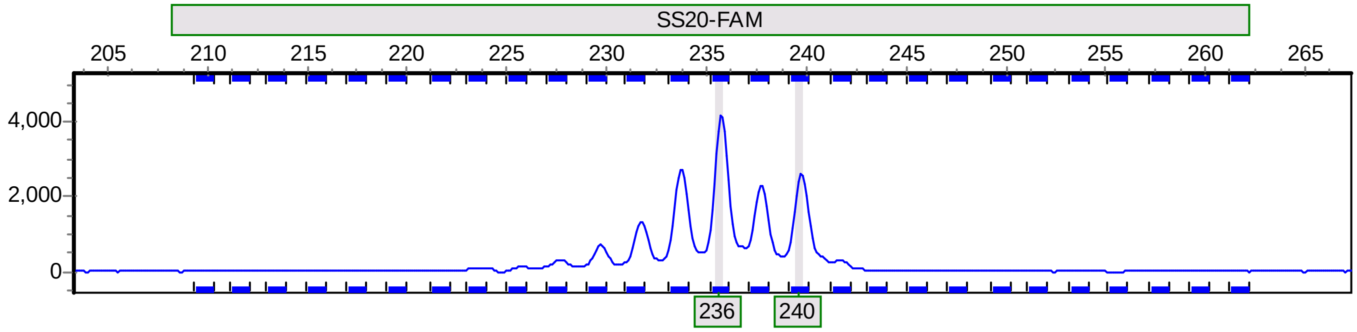

No	Size	Height	Area	Marker	Allele	Difference	Quality	Score	Allele Comments	Sample Comments
1	133.2	19044	130209	SSS13-FAM	133	0.20	Pass	500.0	[<Confirmed>]	
2	136.4	20646	138625	SSS13-FAM	135	1.00	Pass	500.0	[<Confirmed><Edited>]	
3	235.7	4137	32666	SS20-FAM	236	0.00	Pass	500.0	[<Confirmed>]	
4	239.7	2578	21822	SS20-FAM	240	0.00	Pass	336.4	[<Confirmed>]	

Allele Report

Sample 103: SSS13_SS20_SS11_SS21_SS02_SS19_HRS30_P05.fsa

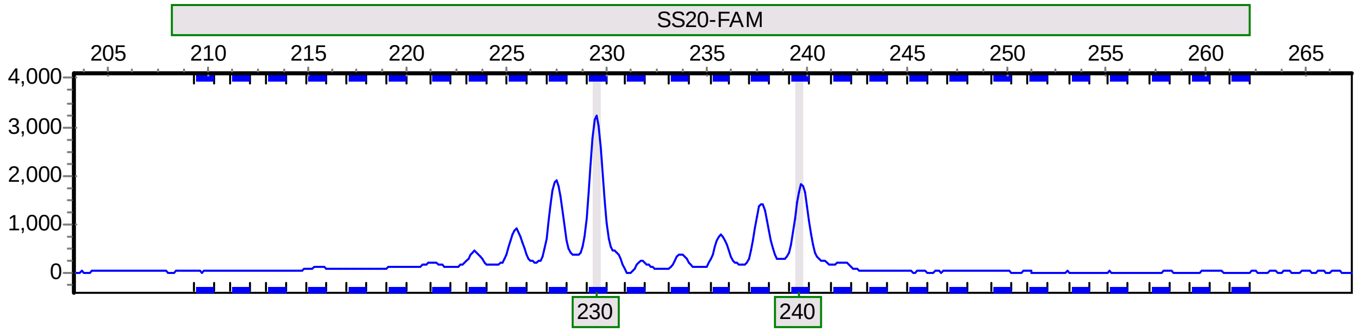

No	Size	Height	Area	Marker	Allele	Difference	Quality	Score	Allele Comments	Sample Comments
1	129.3	22475	156461	SSS13-FAM	129	0.00	Pass	500.0	[<Confirmed>]	
2	133.2	17818	120939	SSS13-FAM	133	0.20	Pass	500.0	[<Confirmed>]	
3	229.5	3214	24878	SS20-FAM	230	0.00	Pass	500.0	[<Confirmed>]	
4	239.7	1801	14464	SS20-FAM	240	0.00	Pass	206.4	[<Confirmed>]	

Sample 104: SSS13_SS20_SS11_SS21_SS02_SS19_HRS31_J11.fsa

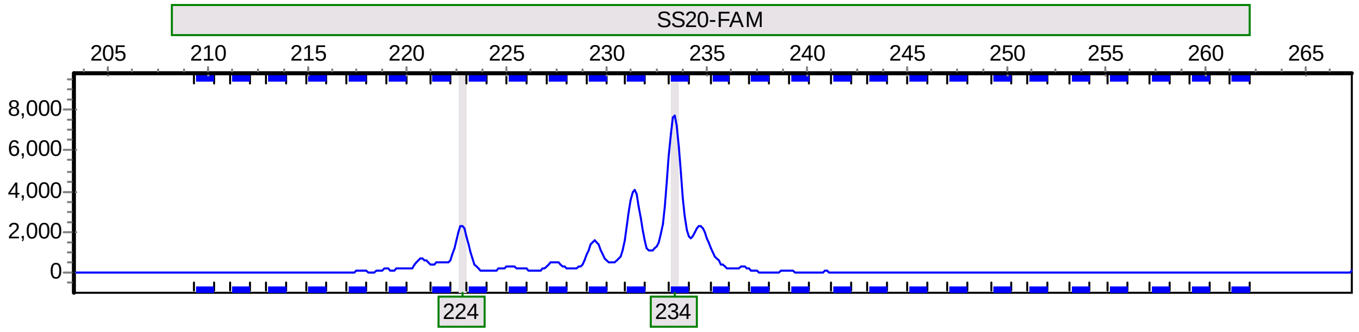

No	Size	Height	Area	Marker	Allele	Difference	Quality	Score	Allele Comments	Sample Comments
1	127.1	10707	73826	SSS13-FAM	127	0.10	Pass	500.0	[<Confirmed>]	
2	222.8	2313	18190	SS20-FAM	224	1.00	Pass	328.9	[<Confirmed><Edited>]	
3	233.4	7653	62200	SS20-FAM	234	0.20	Pass	500.0	[<Confirmed>]	

Sample 105: SSS13_SS20_SS11_SS21_SS02_SS19_HRS33_H07.fsa

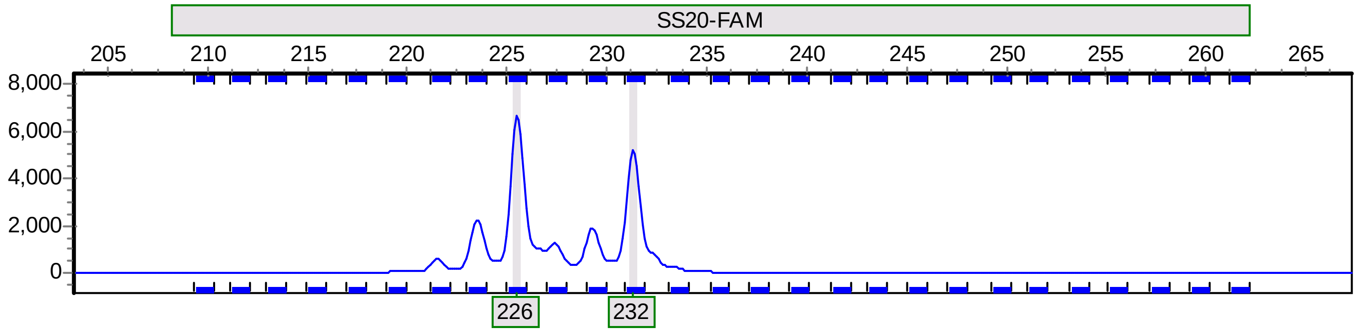

No	Size	Height	Area	Marker	Allele	Difference	Quality	Score	Allele Comments	Sample Comments
1	127.1	31502	225351	SSS13-FAM	127	0.10	Pass	500.0	[<Confirmed>]	
2	133.3	10177	69433	SSS13-FAM	133	0.10	Pass	500.0	[<Confirmed>]	
3	225.5	6606	50558	SS20-FAM	226	0.00	Pass	500.0	[<Confirmed>]	
4	231.3	5163	40423	SS20-FAM	232	0.10	Pass	500.0	[<Confirmed>]	

Allele Report

Sample 106: SSS13_SS20_SS11_SS21_SS02_SS19_HRS34_L09.fsa

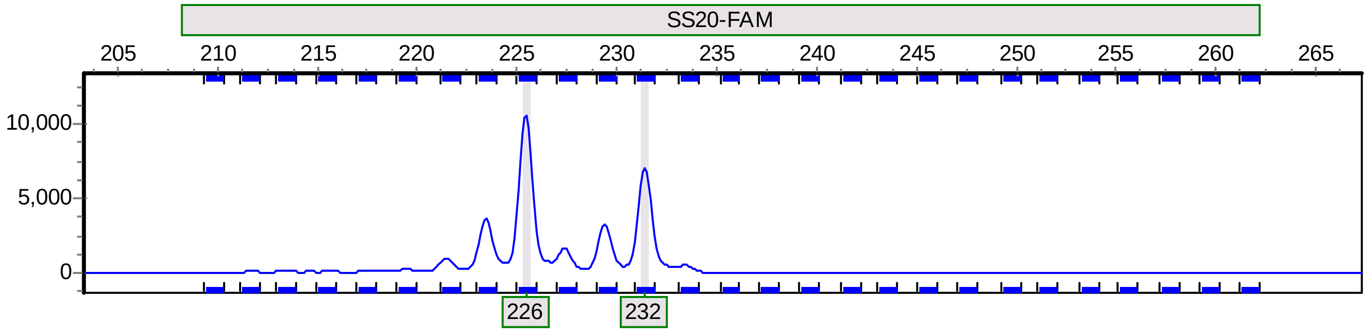

No	Size	Height	Area	Marker	Allele	Difference	Quality	Score	Allele Comments	Sample Comments
1	127.1	31334	241492	SSS13-FAM	127	0.10	Pass	500.0	[<Confirmed>]	
2	137.4	11623	79296	SSS13-FAM	137	0.10	Pass	500.0	[<Confirmed>]	
3	225.5	10455	80193	SS20-FAM	226	0.00	Pass	500.0	[<Confirmed>]	
4	231.4	7062	54309	SS20-FAM	232	0.00	Pass	500.0	[<Confirmed>]	

Sample 107: SSS13_SS20_SS11_SS21_SS02_SS19_HRS35_P09.fsa

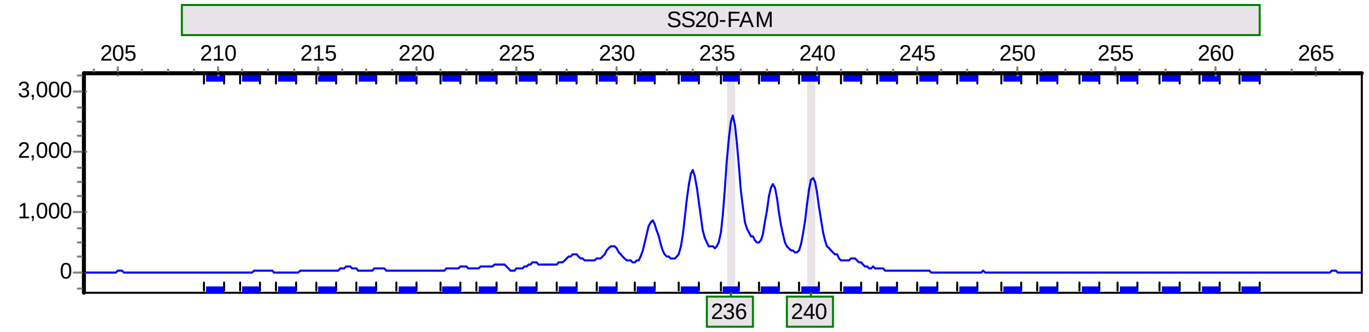

No	Size	Height	Area	Marker	Allele	Difference	Quality	Score	Allele Comments	Sample Comments
1	136.4	20683	142337	SSS13-FAM	135	1.00	Pass	500.0	[<Confirmed><Edited>]	
2	141.8	11790	89949	SSS13-FAM	141	0.30	Pass	500.0	[<Confirmed>]	
3	235.8	2579	21005	SS20-FAM	236	0.10	Pass	338.5	[<Confirmed>]	
4	237.8	1457	11571	SS20-FAM	238	0.10	Undetermined	137.9	[<Deleted>]	
5	239.8	1574	13479	SS20-FAM	240	0.10	Pass	155.3	[<Confirmed>]	

Sample 108: SSS13_SS20_SS11_SS21_SS02_SS19_HRS37_J13.fsa

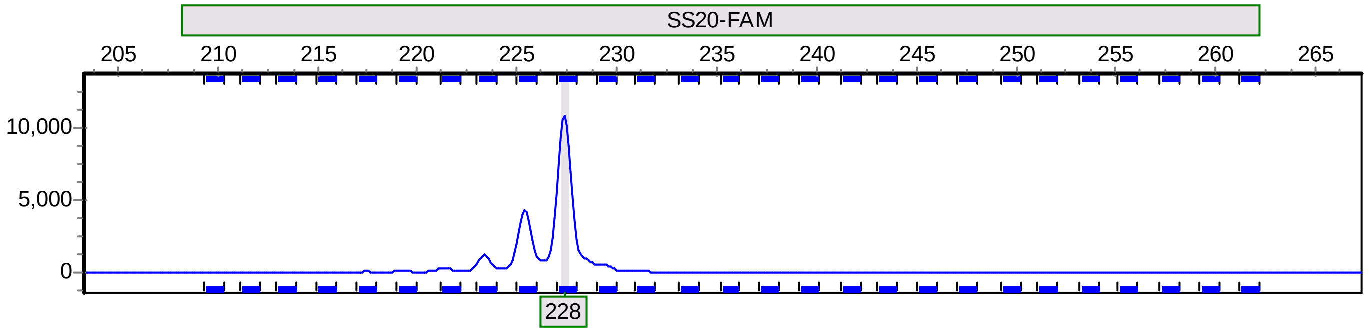

No	Size	Height	Area	Marker	Allele	Difference	Quality	Score	Allele Comments	Sample Comments
1	137.3	17390	115844	SSS13-FAM	137	0.20	Pass	500.0	[<Confirmed>]	
2	227.4	10844	83713	SS20-FAM	228	0.10	Pass	500.0	[<Confirmed>]	

Allele Report

Sample 109: SSS13_SS20_SS11_SS21_SS02_SS19_HRS38_J07.fsa

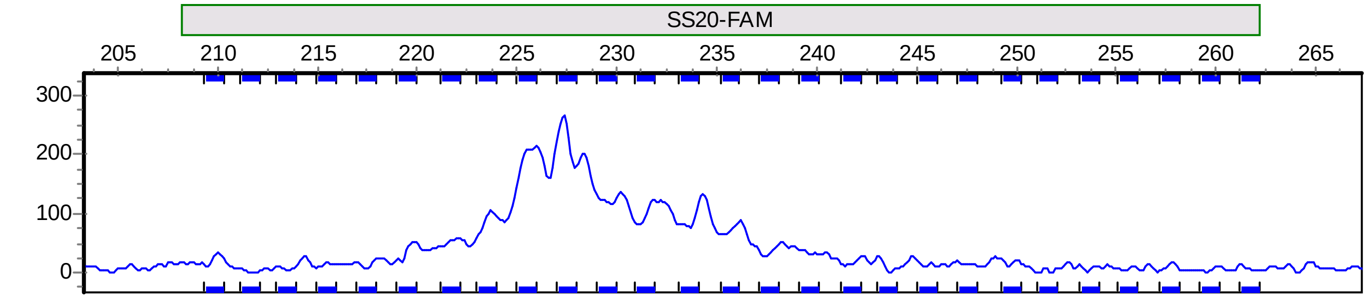

No	Size	Height	Area	Marker	Allele	Difference	Quality	Score	Allele Comments	Sample Comments
1	127.2	29786	199682	SSS13-FAM	127	0.00	Pass	500.0	[<Confirmed>]	
2	142.1	14341	112171	SSS13-FAM	141	0.60	Pass	500.0	[<Confirmed>]	

Sample 110: SSS13_SS20_SS11_SS21_SS02_SS19_HRS39_N07.fsa

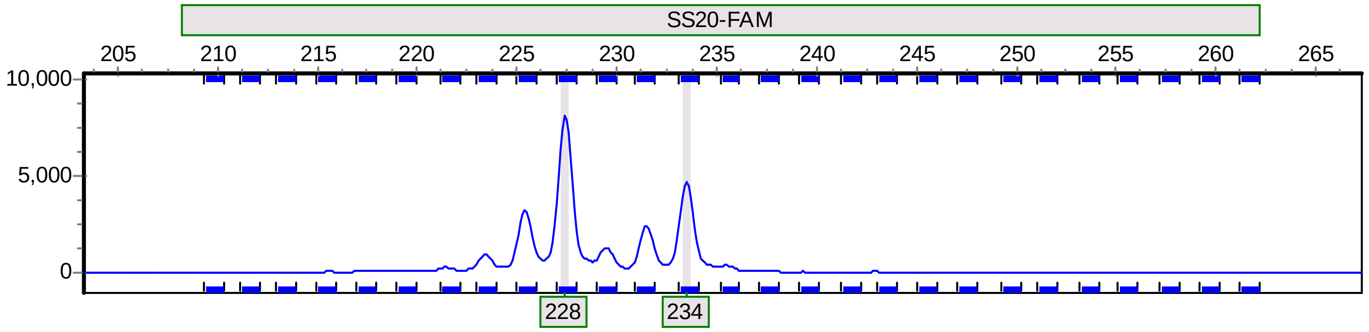

No	Size	Height	Area	Marker	Allele	Difference	Quality	Score	Allele Comments	Sample Comments
1	135.5	24851	168754	SSS13-FAM	135	0.10	Pass	500.0	[<Confirmed>]	
2	146.5	8381	61121	SSS13-FAM	147	0.00	Pass	500.0	[<Confirmed>]	
3	227.4	8041	64430	SS20-FAM	228	0.10	Pass	500.0	[<Confirmed>]	
4	233.5	4664	37197	SS20-FAM	234	0.10	Pass	500.0	[<Confirmed>]	

Sample 111: SSS13_SS20_SS11_SS21_SS02_SS19_HRS40_A07.fsa

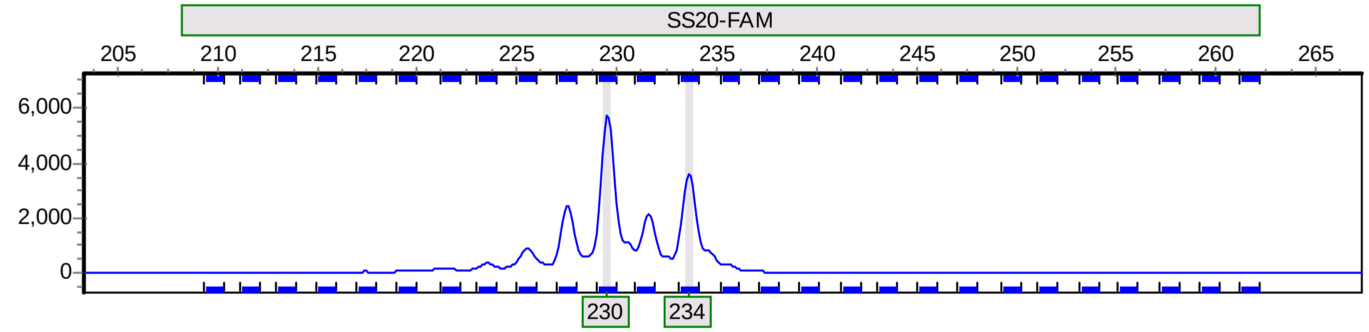

No	Size	Height	Area	Marker	Allele	Difference	Quality	Score	Allele Comments	Sample Comments
1	129.3	33185	243270	SSS13-FAM	129	0.00	Pass	500.0	[<SAT (Repaired)><Confirmed>]	
2	229.5	5708	44837	SS20-FAM	230	0.00	Pass	500.0	[<Confirmed>]	
3	233.6	3635	29918	SS20-FAM	234	0.00	Pass	500.0	[<Confirmed>]	

Allele Report

Sample 112: SSS13_SS20_SS11_SS21_SS02_SS19_HRS41_K07.fsa

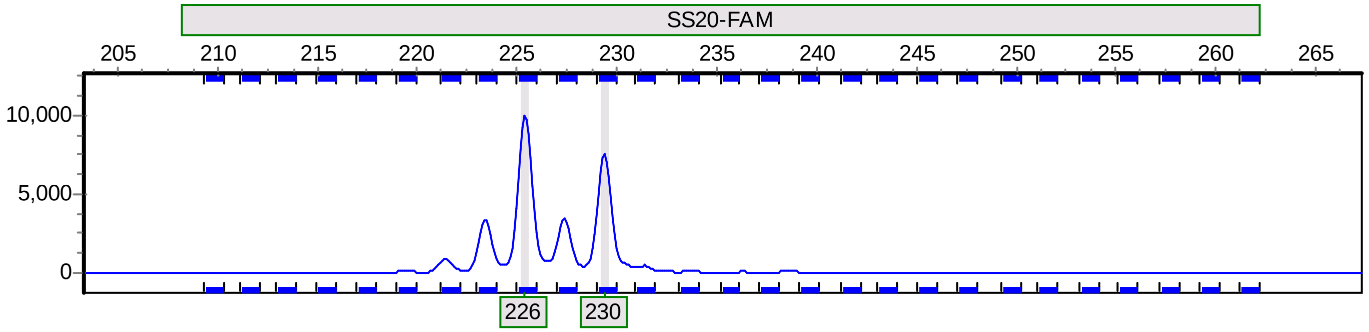

No	Size	Height	Area	Marker	Allele	Difference	Quality	Score	Allele Comments	Sample Comments
1	127.0	31338	219757	SSS13-FAM	127	0.20	Pass	500.0	[<Confirmed>]	
2	135.3	26829	181466	SSS13-FAM	135	0.10	Pass	500.0	[<Confirmed>]	
3	225.4	9971	78246	SS20-FAM	226	0.10	Pass	500.0	[<Confirmed>]	
4	229.4	7583	58489	SS20-FAM	230	0.10	Pass	500.0	[<Confirmed>]	

Sample 113: SSS13_SS20_SS11_SS21_SS02_SS19_HRS42_I07.fsa

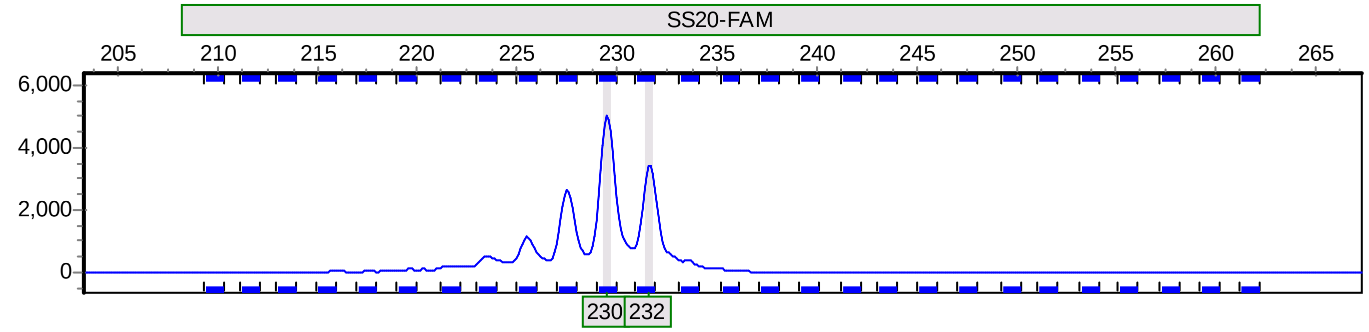

No	Size	Height	Area	Marker	Allele	Difference	Quality	Score	Allele Comments	Sample Comments
1	133.5	22738	156660	SSS13-FAM	133	0.10	Pass	500.0	[<Confirmed>]	
2	137.6	16473	114685	SSS13-FAM	137	0.10	Pass	500.0	[<Confirmed>]	
3	229.5	5018	41667	SS20-FAM	230	0.00	Pass	500.0	[<Confirmed>]	
4	231.6	3430	29631	SS20-FAM	232	0.20	Pass	467.0	[<Confirmed>]	

Sample 114: SSS13_SS20_SS11_SS21_SS02_SS19_HTHL11_M11.fsa

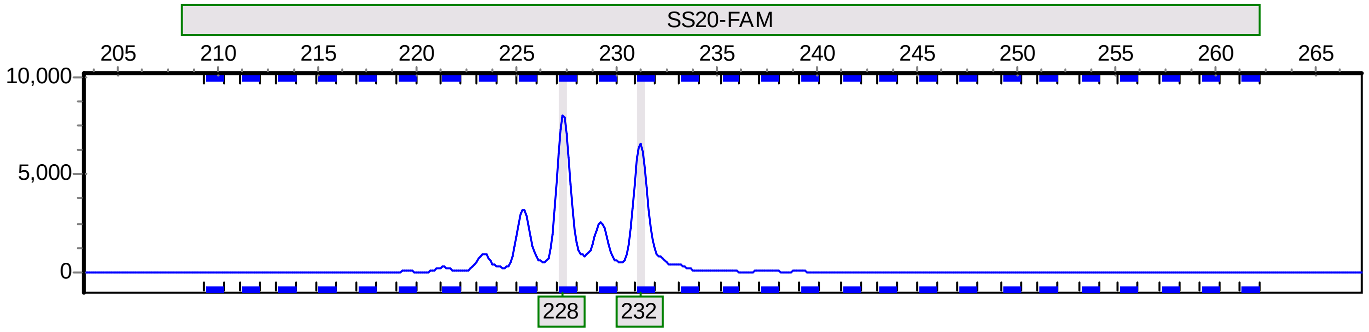

No	Size	Height	Area	Marker	Allele	Difference	Quality	Score	Allele Comments	Sample Comments
1	127.0	31561	268070	SSS13-FAM	127	0.20	Pass	500.0	[<Confirmed>]	
2	227.3	7965	62542	SS20-FAM	228	0.20	Pass	500.0	[<Confirmed>]	
3	231.2	6554	50864	SS20-FAM	232	0.20	Pass	500.0	[<Confirmed>]	

Sample 115: SSS13_SS20_SS11_SS21_SS02_SS19_HTHL13_H01.fsa

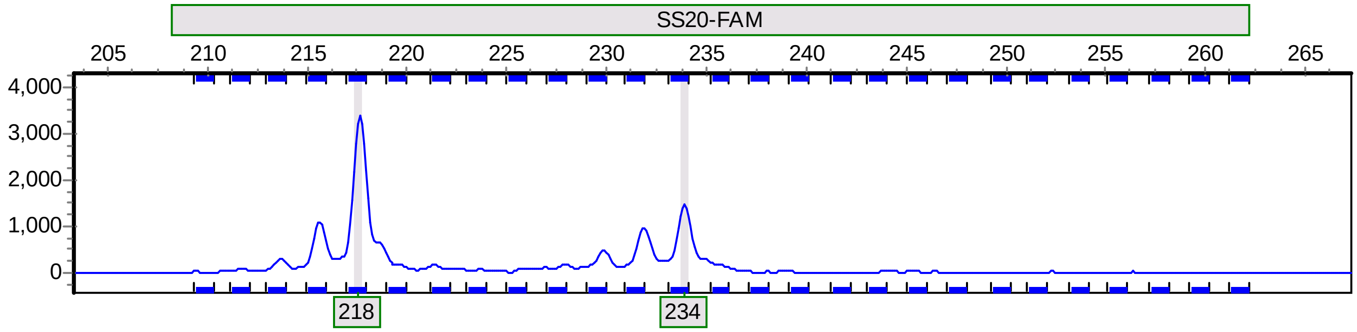

No	Size	Height	Area	Marker	Allele	Difference	Quality	Score	Allele Comments	Sample Comments
1	129.4	19036	125639	SSS13-FAM	129	0.10	Pass	500.0	[<Confirmed>]	
2	135.5	16391	108003	SSS13-FAM	135	0.10	Pass	500.0	[<Confirmed>]	
3	217.6	3359	25117	SS20-FAM	218	0.10	Pass	500.0	[<Confirmed>]	
4	233.9	1470	10969	SS20-FAM	234	0.30	Pass	174.3	[<Confirmed>]	

Sample 116: SSS13_SS20_SS11_SS21_SS02_SS19_HTHL14_F15.fsa

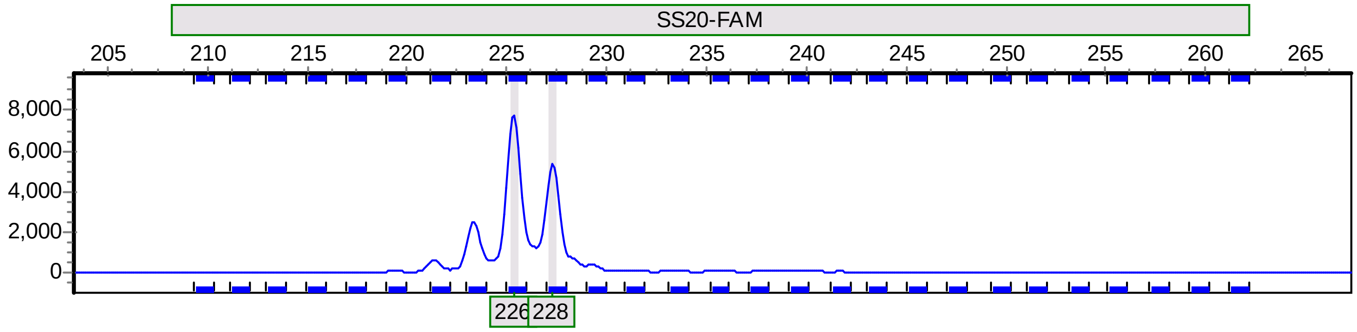

No	Size	Height	Area	Marker	Allele	Difference	Quality	Score	Allele Comments	Sample Comments
1	137.3	31421	223639	SSS13-FAM	137	0.20	Pass	500.0	[<Confirmed>]	
2	225.4	7705	60867	SS20-FAM	226	0.10	Pass	500.0	[<Confirmed>]	
3	227.3	5330	42731	SS20-FAM	228	0.20	Pass	500.0	[<Confirmed>]	

Sample 117: SSS13_SS20_SS11_SS21_SS02_SS19_HTHL15_F01.fsa

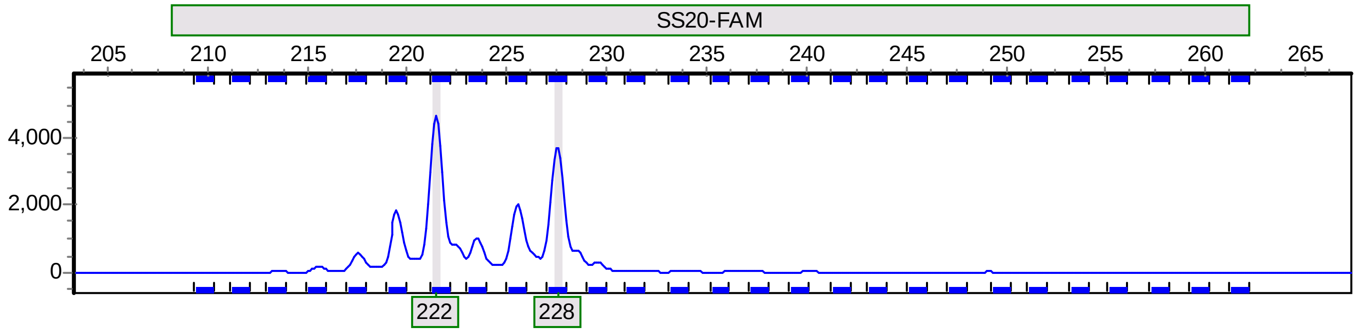

No	Size	Height	Area	Marker	Allele	Difference	Quality	Score	Allele Comments	Sample Comments
1	129.4	27855	177401	SSS13-FAM	129	0.10	Pass	500.0	[<Confirmed>]	
2	137.4	29299	188537	SSS13-FAM	137	0.10	Pass	500.0	[<Confirmed>]	
3	221.5	4608	33900	SS20-FAM	222	0.20	Pass	500.0	[<Confirmed>]	
4	227.6	3693	28749	SS20-FAM	228	0.10	Pass	500.0	[<Confirmed>]	

Allele Report

Sample 118: SSS13_SS20_SS11_SS21_SS02_SS19_HTHL1_E13.fsa

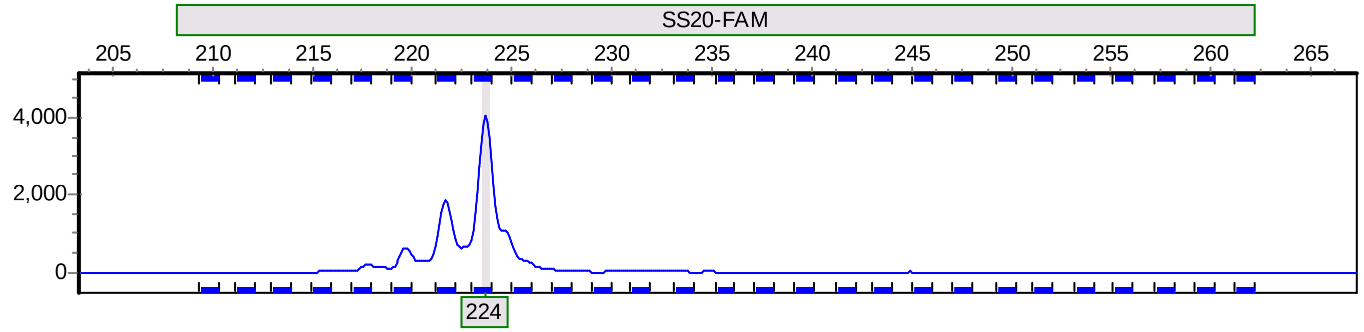

No	Size	Height	Area	Marker	Allele	Difference	Quality	Score	Allele Comments	Sample Comments
1	127.1	31171	221440	SSS13-FAM	127	0.10	Pass	500.0	[<Confirmed>]	
2	131.4	14486	100850	SSS13-FAM	131	0.10	Pass	500.0	[<Confirmed>]	
3	223.7	4012	34086	SS20-FAM	224	0.20	Pass	500.0	[<Confirmed>]	

Sample 119: SSS13_SS20_SS11_SS21_SS02_SS19_HTHL3_D05.fsa

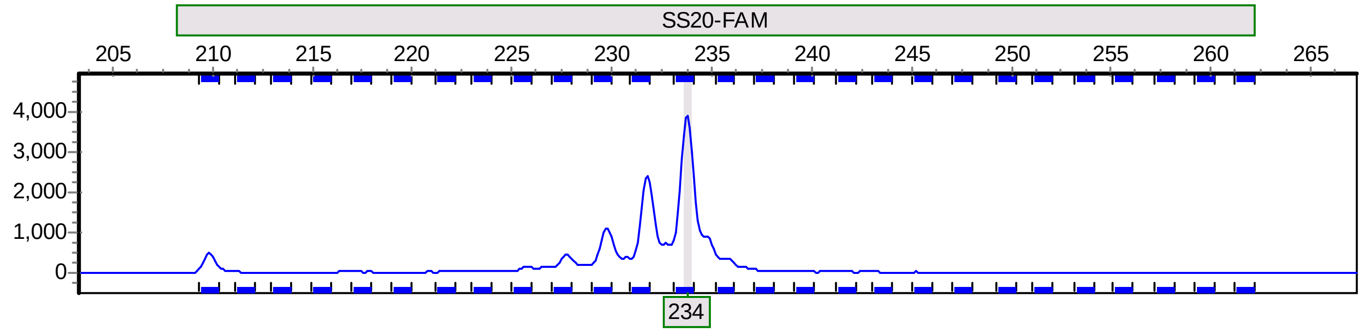

No	Size	Height	Area	Marker	Allele	Difference	Quality	Score	Allele Comments	Sample Comments
1	136.5	22501	152221	SSS13-FAM	135	1.00	Pass	500.0	[<Confirmed><Edited>]	
2	137.7	16759	112529	SSS13-FAM	137	0.20	Pass	500.0	[<Confirmed>]	
3	233.8	3898	30612	SS20-FAM	234	0.20	Pass	500.0	[<Confirmed>]	

Sample 120: SSS13_SS20_SS11_SS21_SS02_SS19_HTHL4_O15.fsa

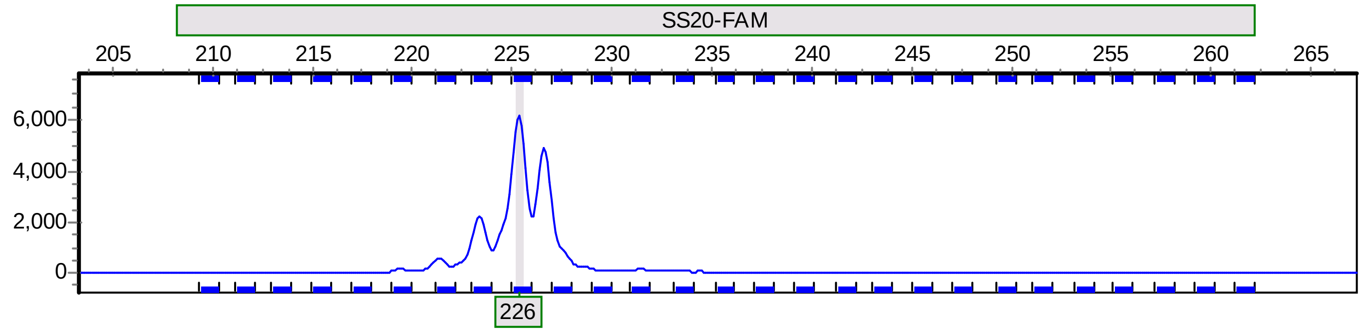

No	Size	Height	Area	Marker	Allele	Difference	Quality	Score	Allele Comments	Sample Comments
1	129.2	19525	134036	SSS13-FAM	129	0.10	Pass	500.0	[<Confirmed>]	
2	143.4	13043	101011	SSS13-FAM	143	0.40	Pass	500.0	[<Confirmed>]	
3	225.4	6139	52553	SS20-FAM	226	0.10	Pass	500.0	[<Confirmed>]	

Sample 121: SSS13_SS20_SS11_SS21_SS02_SS19_HTHL5_M13.fsa

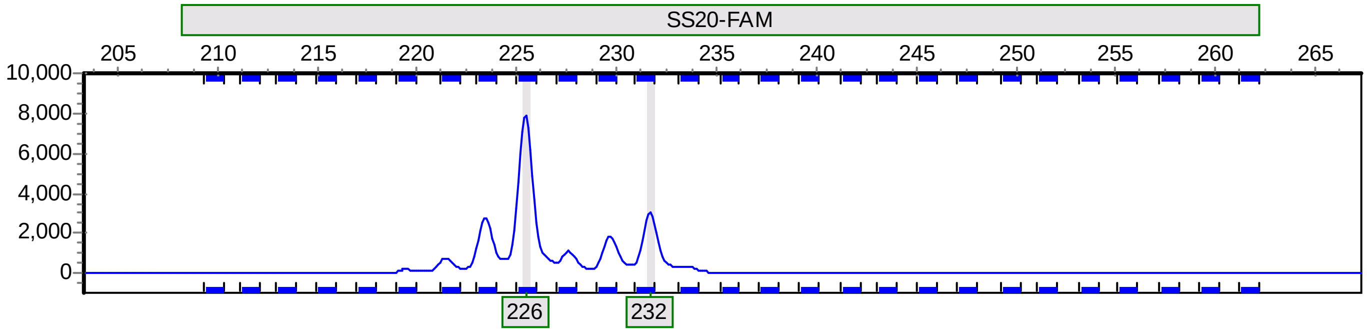

No	Size	Height	Area	Marker	Allele	Difference	Quality	Score	Allele Comments	Sample Comments
1	135.4	22904	159382	SSS13-FAM	135	0.00	Pass	500.0	[<Confirmed>]	
2	145.7	11806	90741	SSS13-FAM	145	0.40	Pass	500.0	[<Confirmed>]	
3	225.5	7856	63573	SS20-FAM	226	0.00	Pass	500.0	[<Confirmed>]	
4	231.7	3028	24609	SS20-FAM	232	0.30	Pass	426.6	[<Confirmed>]	

Sample 122: SSS13_SS20_SS11_SS21_SS02_SS19_HTHL6_P03.fsa

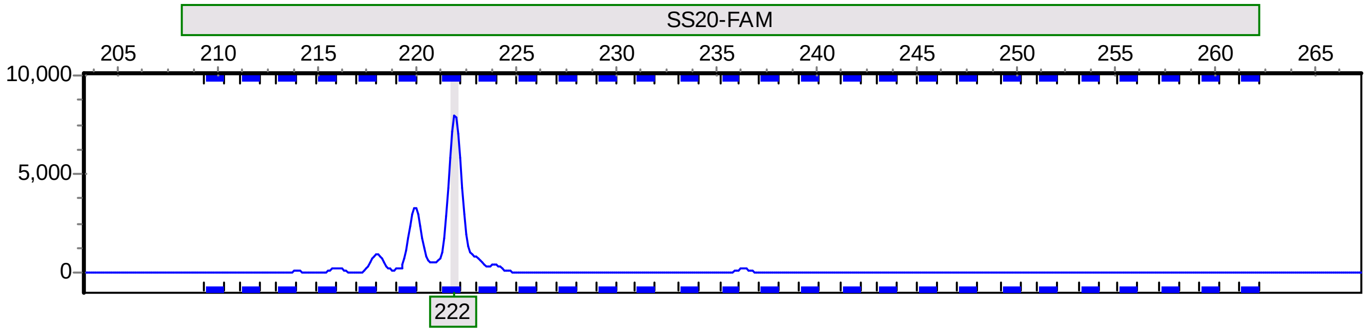

No	Size	Height	Area	Marker	Allele	Difference	Quality	Score	Allele Comments	Sample Comments
1	137.4	24157	157171	SSS13-FAM	137	0.10	Pass	500.0	[<Confirmed>]	
2	221.9	7922	59425	SS20-FAM	222	0.20	Pass	500.0	[<Confirmed>]	

Sample 123: SSS13_SS20_SS11_SS21_SS02_SS19_HTHL8_A03.fsa

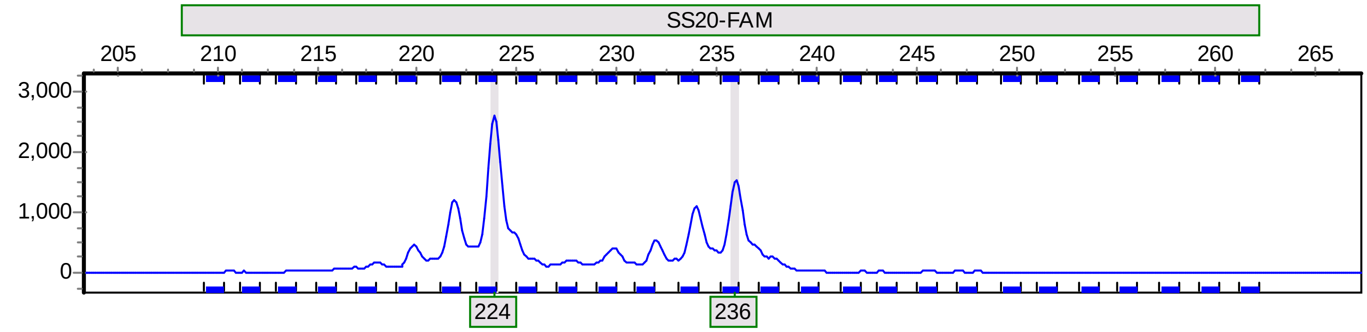

No	Size	Height	Area	Marker	Allele	Difference	Quality	Score	Allele Comments	Sample Comments
1	136.6	31114	220414	SSS13-FAM	135	1.00	Pass	500.0	[<Confirmed><Edited>]	
2	223.9	2589	20952	SS20-FAM	224	0.40	Pass	351.6	[<Confirmed>]	
3	236.0	1525	12578	SS20-FAM	236	0.30	Pass	147.9	[<Confirmed>]	

Allele Report

Sample 124: SSS13_SS20_SS11_SS21_SS02_SS19_HTHL9_O09.fsa

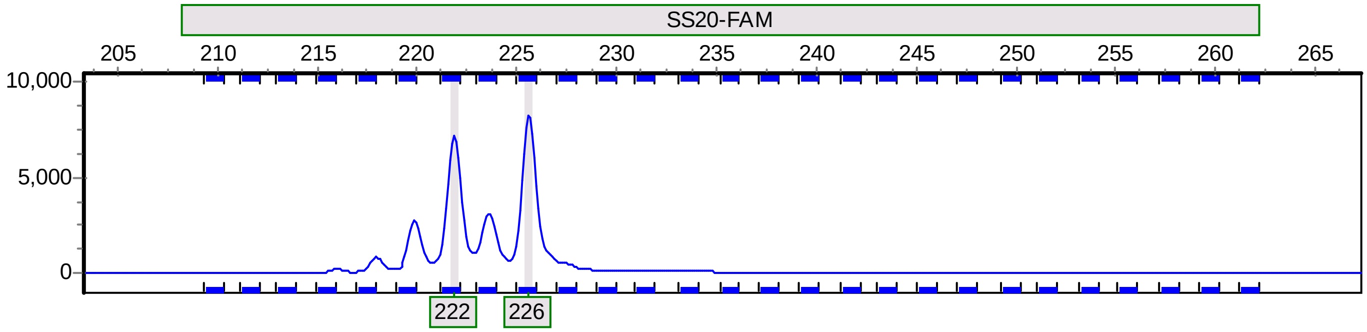

No	Size	Height	Area	Marker	Allele	Difference	Quality	Score	Allele Comments	Sample Comments
1	129.4	30354	213316	SSS13-FAM	129	0.10	Pass	500.0	[<Confirmed>]	
2	221.9	7130	57187	SS20-FAM	222	0.20	Pass	500.0	[<Confirmed>]	
3	225.6	8174	64170	SS20-FAM	226	0.10	Pass	500.0	[<Confirmed>]	
