# Supplementary material for: Genetic diversity of Schima superba based on physiological traits and SSR markers
Source: PLoS One. 2026 Apr 10;21(4):e0344465. doi: 10.1371/journal.pone.0344465 (PMC13068225; doi:10.1371/journal.pone.0344465)
Supplement: S1 File — (ZIP) [file pone.0344465.s003.zip › SS21.pdf]

Project Comments:

Sample 1: SSS13_SS20_SS11_SS21_SS02_SS19_HBB10_E05.fsa

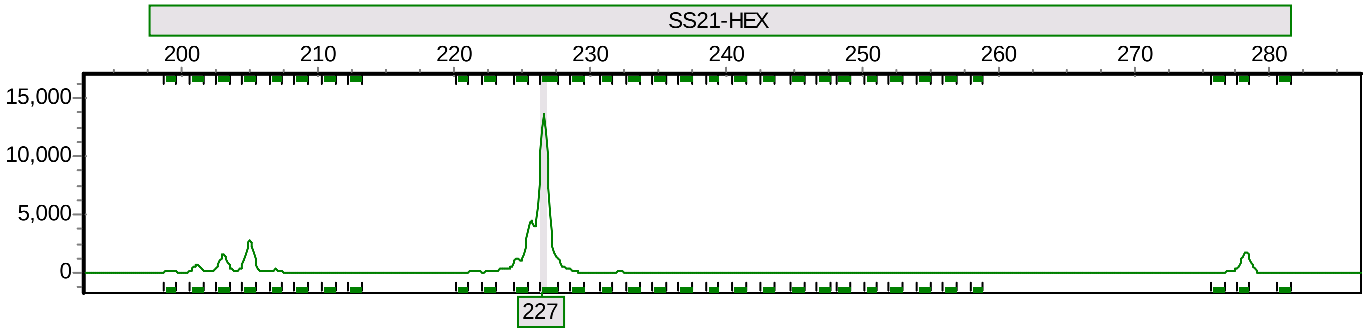

No	Size	Height	Area	Marker	Allele	Difference	Quality	Score	Allele Comments	Sample Comments
1	146.8	15580	85978	SS11-HEX	146	0.60	Pass	500.0	[<Confirmed>]	
2	169.4	6063	40805	SS11-HEX	170	0.10	Pass	500.0	[<Confirmed>]	
3	226.6	13619	99771	SS21-HEX	227	0.40	Pass	500.0	[<Confirmed>]	

Sample 2: SSS13_SS20_SS11_SS21_SS02_SS19_HBB12-2_D11.fsa

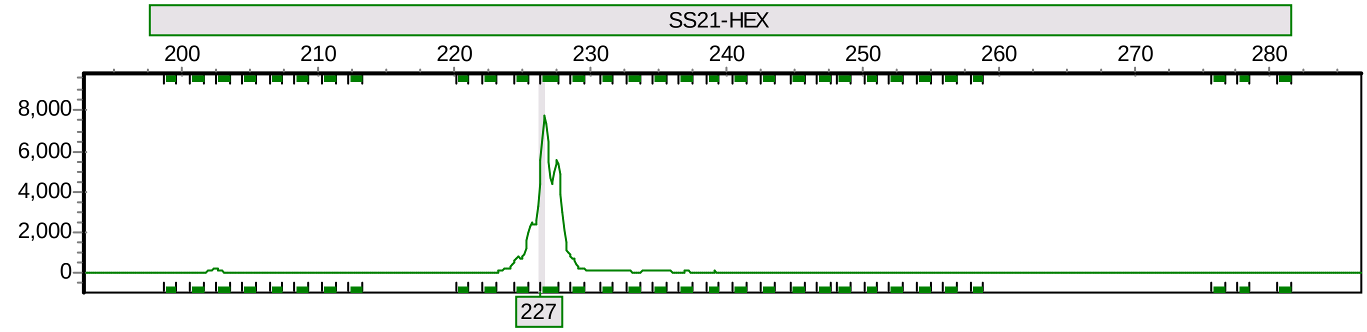

No	Size	Height	Area	Marker	Allele	Difference	Quality	Score	Allele Comments	Sample Comments
1	163.5	9255	62673	SS11-HEX	164	0.10	Pass	500.0	[<Confirmed>]	
2	167.4	6134	42104	SS11-HEX	168	0.00	Pass	500.0	[<Confirmed>]	
3	226.5	6699	104076	SS21-HEX	227	0.50	Pass	500.0	[<Confirmed>]	

Sample 3: SSS13_SS20_SS11_SS21_SS02_SS19_HBB13_C17.fsa

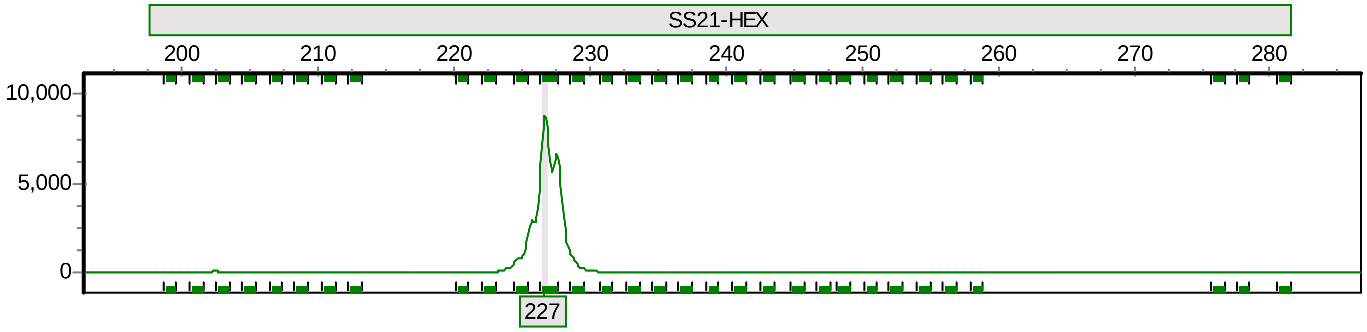

No	Size	Height	Area	Marker	Allele	Difference	Quality	Score	Allele Comments	Sample Comments
1	145.9	17041	128492	SS11-HEX	146	0.30	Pass	500.0	[<Confirmed>]	
2	163.6	7624	54283	SS11-HEX	164	0.20	Pass	500.0	[<Confirmed>]	
3	226.7	8721	73566	SS21-HEX	227	0.30	Pass	500.0	[<Confirmed>]	

Allele Report

Sample 4: SSS13_SS20_SS11_SS21_SS02_SS19_HBB14_G03.fsa

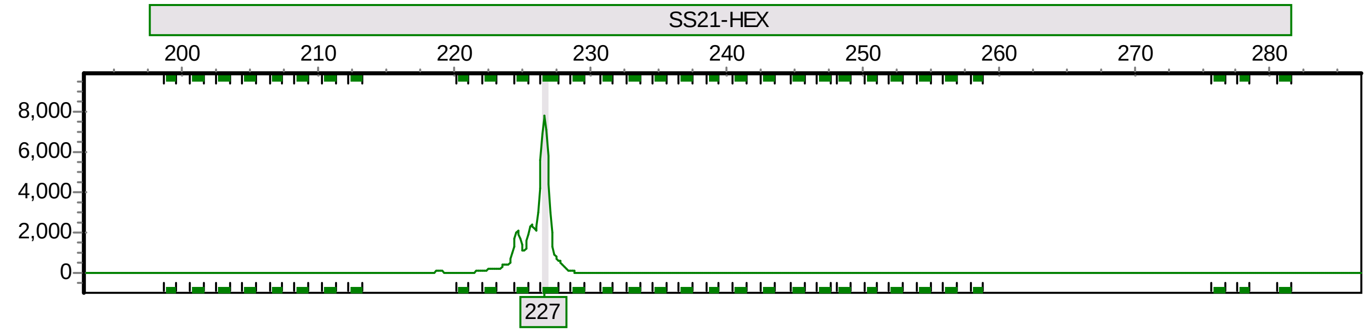

No	Size	Height	Area	Marker	Allele	Difference	Quality	Score	Allele Comments	Sample Comments
1	161.4	16873	110023	SS11-HEX	162	0.10	Pass	500.0	[<Confirmed>]	
2	163.5	5701	39029	SS11-HEX	164	0.10	Pass	500.0	[<Confirmed>]	
3	165.5	5614	37210	SS11-HEX	166	0.10	Undetermined	500.0	[<Deleted>]	
4	226.7	7787	60027	SS21-HEX	227	0.30	Pass	500.0	[<Confirmed>]	

Sample 5: SSS13_SS20_SS11_SS21_SS02_SS19_HBB15_B07.fsa

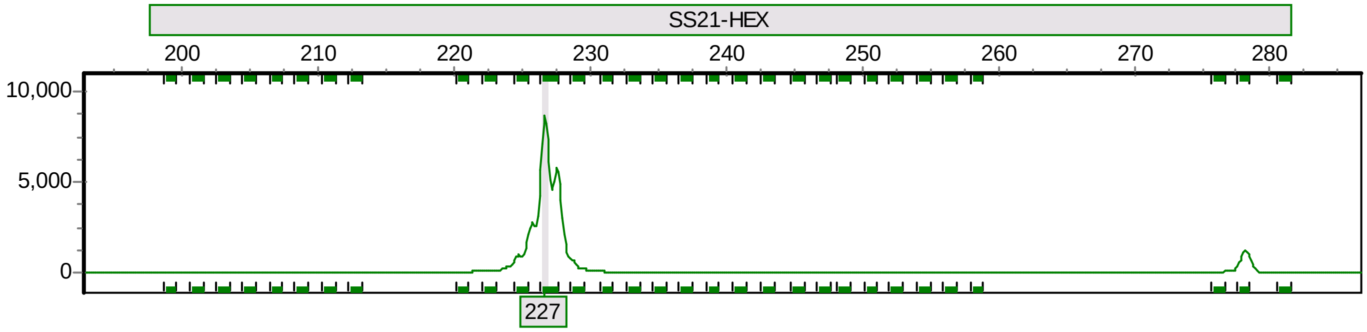

No	Size	Height	Area	Marker	Allele	Difference	Quality	Score	Allele Comments	Sample Comments
1	163.5	8283	56273	SS11-HEX	164	0.10	Pass	500.0	[<Confirmed>]	
2	165.4	4947	34112	SS11-HEX	166	0.00	Pass	500.0	[<Confirmed>]	
3	226.7	8667	63822	SS21-HEX	227	0.30	Pass	500.0	[<Confirmed>]	

Sample 6: SSS13_SS20_SS11_SS21_SS02_SS19_HBB16_G07.fsa

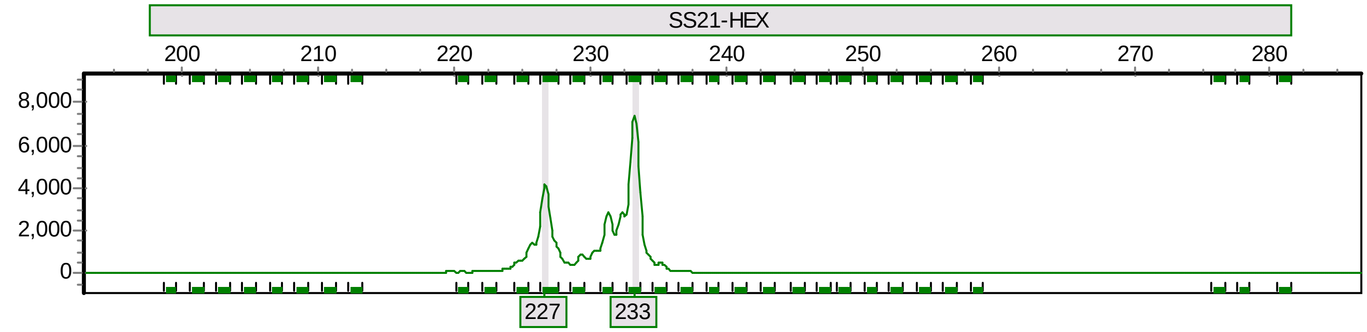

No	Size	Height	Area	Marker	Allele	Difference	Quality	Score	Allele Comments	Sample Comments
1	161.4	31082	211692	SS11-HEX	162	0.10	Pass	500.0	[<Confirmed>]	
2	226.7	4186	33602	SS21-HEX	227	0.30	Pass	500.0	[<Confirmed>]	
3	233.3	7334	59794	SS21-HEX	233	0.00	Pass	500.0	[<Confirmed>]	

Sample 7: SSS13_SS20_SS11_SS21_SS02_SS19_HBB17_K15.fsa

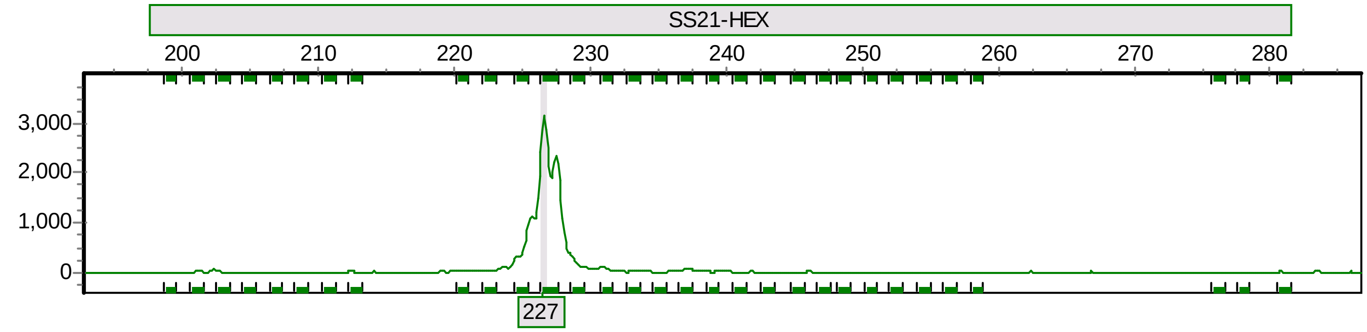

No	Size	Height	Area	Marker	Allele	Difference	Quality	Score	Allele Comments	Sample Comments
1	163.6	4330	31054	SS11-HEX	164	0.20	Pass	500.0	[<Confirmed>]	
2	165.6	2959	20892	SS11-HEX	166	0.20	Pass	500.0	[<Confirmed>]	
3	226.6	3129	24352	SS21-HEX	227	0.40	Pass	383.1	[<Confirmed>]	

Sample 8: SSS13_SS20_SS11_SS21_SS02_SS19_HBB18_O05.fsa

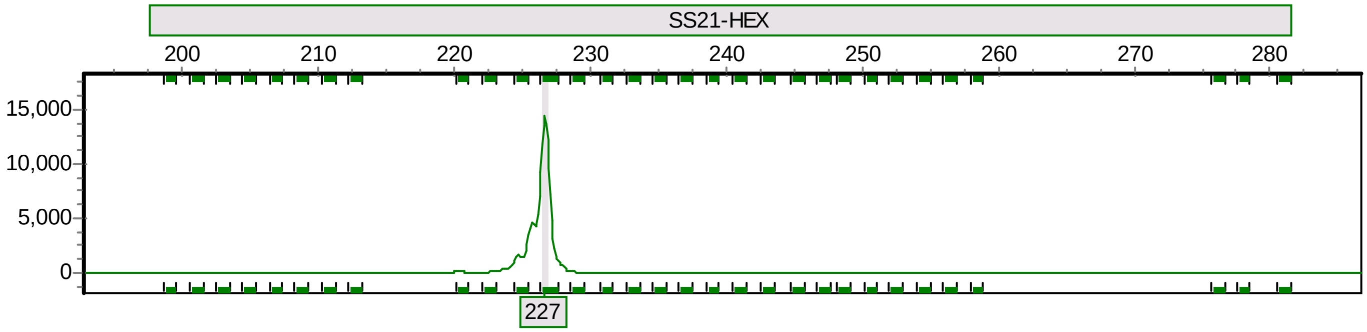

No	Size	Height	Area	Marker	Allele	Difference	Quality	Score	Allele Comments	Sample Comments
1	161.3	21505	148174	SS11-HEX	162	0.20	Pass	500.0	[<Confirmed>]	
2	226.7	14404	111950	SS21-HEX	227	0.30	Pass	500.0	[<Confirmed>]	

Sample 9: SSS13_SS20_SS11_SS21_SS02_SS19_HBB19_O07.fsa

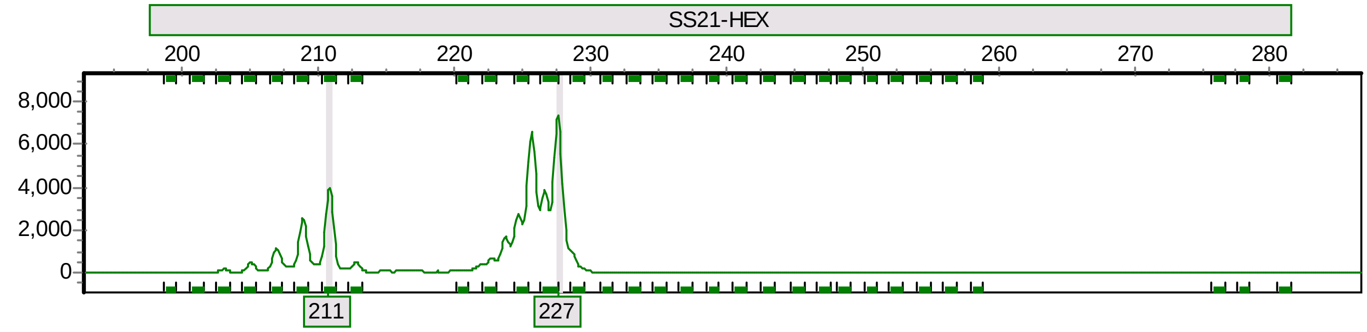

No	Size	Height	Area	Marker	Allele	Difference	Quality	Score	Allele Comments	Sample Comments
1	165.5	15886	104762	SS11-HEX	166	0.10	Pass	500.0	[<Confirmed>]	
2	210.9	3918	27754	SS21-HEX	211	0.00	Pass	500.0	[<Confirmed>]	
3	227.7	7285	54762	SS21-HEX	227	0.70	Pass	500.0	[<Confirmed>]	

Allele Report

Sample 10: SSS13_SS20_SS11_SS21_SS02_SS19_HBB1_B11.fsa

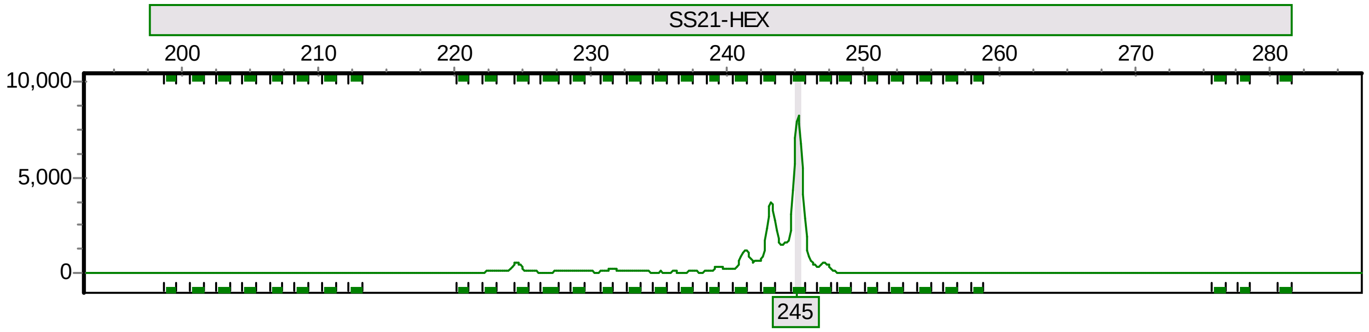

No	Size	Height	Area	Marker	Allele	Difference	Quality	Score	Allele Comments	Sample Comments
1	139.5	32005	283378	SS11-HEX	140	0.10	Pass	500.0	[<Confirmed>]	
2	245.3	8208	67070	SS21-HEX	245	0.00	Pass	500.0	[<Confirmed>]	

Sample 11: SSS13_SS20_SS11_SS21_SS02_SS19_HBB20_F11.fsa

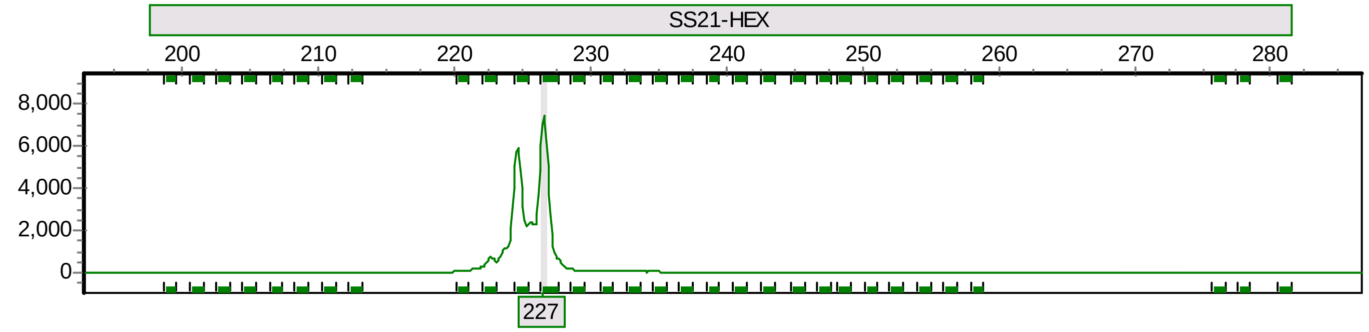

No	Size	Height	Area	Marker	Allele	Difference	Quality	Score	Allele Comments	Sample Comments
1	175.3	29345	205293	SS11-HEX	176	0.10	Pass	500.0	[<Confirmed>]	
2	226.6	7424	58231	SS21-HEX	227	0.40	Pass	500.0	[<Confirmed>]	

Sample 12: SSS13_SS20_SS11_SS21_SS02_SS19_HBB21_J09.fsa

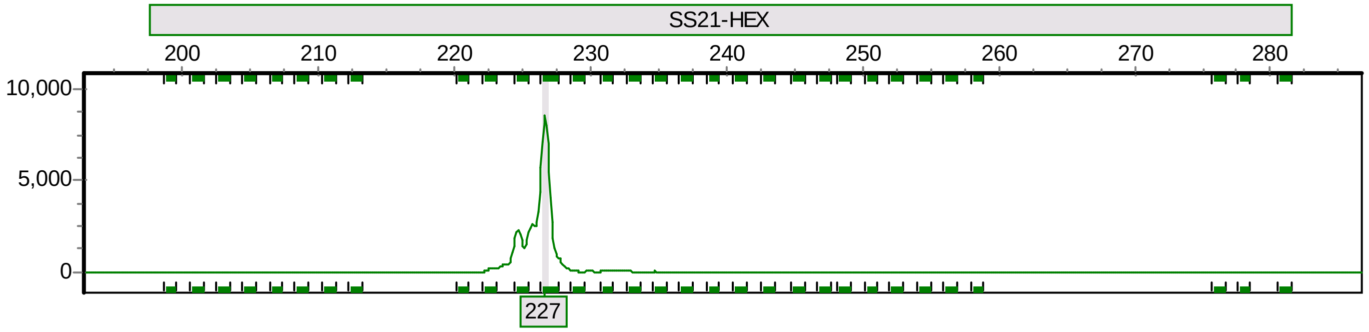

No	Size	Height	Area	Marker	Allele	Difference	Quality	Score	Allele Comments	Sample Comments
1	161.4	24091	162212	SS11-HEX	162	0.10	Pass	500.0	[<Confirmed>]	
2	226.7	8499	65886	SS21-HEX	227	0.30	Pass	500.0	[<Confirmed>]	

Allele Report

Sample 13: SSS13_SS20_SS11_SS21_SS02_SS19_HBB22_K05.fsa

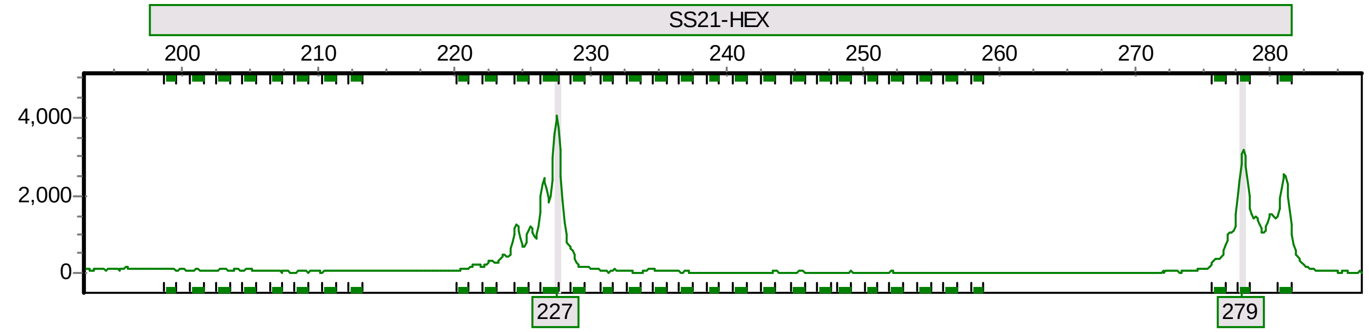

No	Size	Height	Area	Marker	Allele	Difference	Quality	Score	Allele Comments	Sample Comments
1	175.0	17373	120184	SS11-HEX	176	0.20	Pass	500.0	[<Confirmed>]	
2	227.6	4056	31493	SS21-HEX	227	0.60	Pass	500.0	[<Confirmed>]	
3	278.0	3165	27800	SS21-HEX	279	0.10	Pass	339.3	[<Confirmed>]	
4	281.0	2558	23610	SS21-HEX	281	0.10	Undetermined	225.9	[<Deleted>]	

Sample 14: SSS13_SS20_SS11_SS21_SS02_SS19_HBB23_B09.fsa

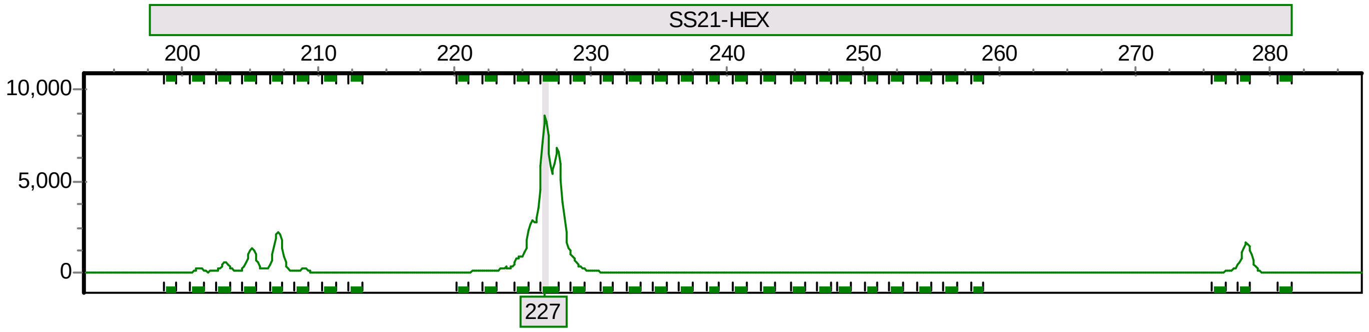

No	Size	Height	Area	Marker	Allele	Difference	Quality	Score	Allele Comments	Sample Comments
1	161.5	9144	62608	SS11-HEX	162	0.00	Pass	500.0	[<Confirmed>]	
2	165.4	6445	44228	SS11-HEX	166	0.00	Pass	500.0	[<Confirmed>]	
3	226.7	8562	65820	SS21-HEX	227	0.30	Pass	500.0	[<Confirmed>]	

Sample 15: SSS13_SS20_SS11_SS21_SS02_SS19_HBB25-1_F09.fsa

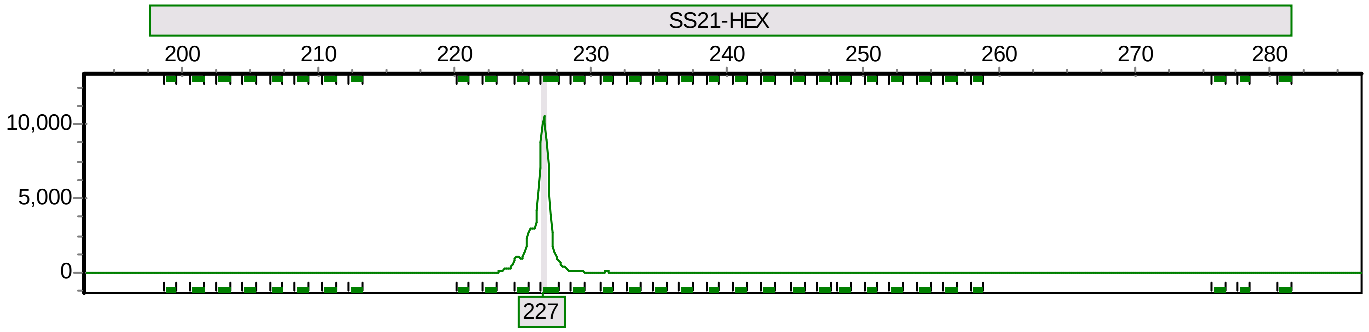

No	Size	Height	Area	Marker	Allele	Difference	Quality	Score	Allele Comments	Sample Comments
1	156.0	5971	38179	SS11-HEX	156	0.10	Pass	500.0	[<Confirmed>]	
2	161.6	4801	32602	SS11-HEX	162	0.10	Pass	500.0	[<Confirmed>]	
3	226.6	10427	84012	SS21-HEX	227	0.40	Pass	500.0	[<Confirmed>]	

Allele Report

Sample 16: SSS13_SS20_SS11_SS21_SS02_SS19_HBB25-2_N11.fsa

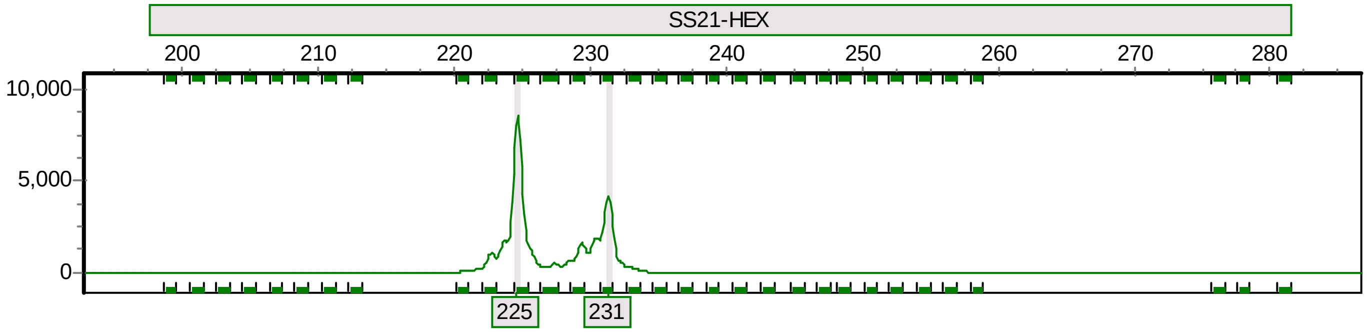

No	Size	Height	Area	Marker	Allele	Difference	Quality	Score	Allele Comments	Sample Comments
1	171.3	27132	187441	SS11-HEX	172	0.00	Pass	500.0	[<Confirmed>]	
2	224.7	8498	66088	SS21-HEX	225	0.30	Pass	500.0	[<Confirmed>]	
3	231.4	4180	34879	SS21-HEX	231	0.10	Pass	500.0	[<Confirmed>]	

Sample 17: SSS13_SS20_SS11_SS21_SS02_SS19_HBB26_D07.fsa

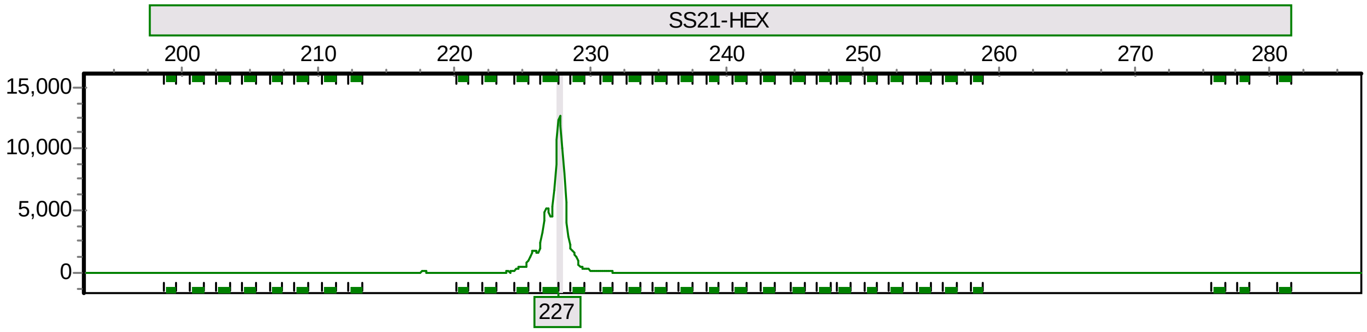

No	Size	Height	Area	Marker	Allele	Difference	Quality	Score	Allele Comments	Sample Comments
1	165.4	11607	78503	SS11-HEX	166	0.00	Pass	500.0	[<Confirmed>]	
2	226.8	5217	36710	SS21-HEX	227	0.20	Pass	500.0	[<Deleted>]	
3	227.8	12603	95877	SS21-HEX	227	1.00	Pass	500.0	[<Confirmed><Edited>]	

Sample 18: SSS13_SS20_SS11_SS21_SS02_SS19_HBB27_H05.fsa

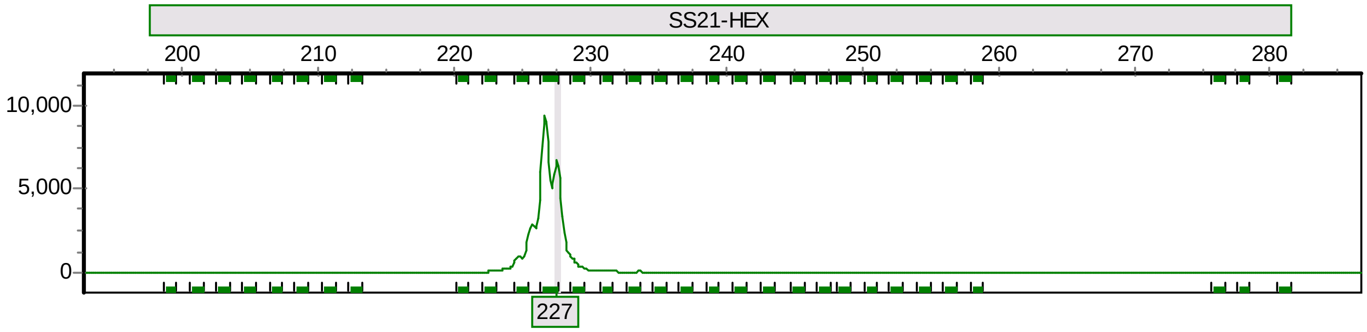

No	Size	Height	Area	Marker	Allele	Difference	Quality	Score	Allele Comments	Sample Comments
1	161.5	12572	83997	SS11-HEX	162	0.00	Pass	500.0	[<Confirmed>]	
2	226.5	7568	73096	SS21-HEX	227	0.50	Pass	500.0	[<Deleted>]	
3	227.6	6686	48190	SS21-HEX	227	0.60	Pass	500.0	[<Confirmed>]	

Allele Report

Sample 19: SSS13_SS20_SS11_SS21_SS02_SS19_HBB28_L11.fsa

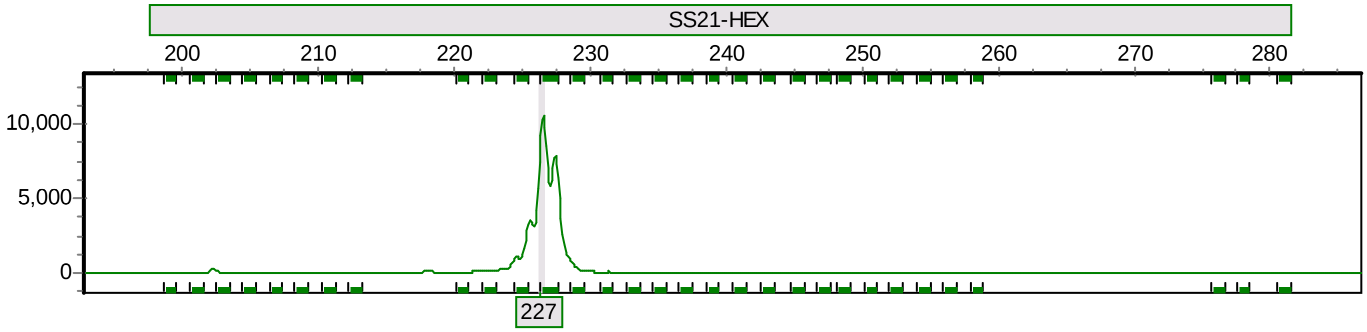

No	Size	Height	Area	Marker	Allele	Difference	Quality	Score	Allele Comments	Sample Comments
1	129.3	13387	63139	SS11-HEX	130	0.10	Pass	500.0	[<Confirmed>]	
2	131.5	4583	31345	SS11-HEX	132	0.10	Pass	500.0	[<Confirmed>]	
3	226.4	9252	139840	SS21-HEX	227	0.60	Pass	500.0	[<Confirmed>]	

Sample 20: SSS13_SS20_SS11_SS21_SS02_SS19_HBB2_C07.fsa

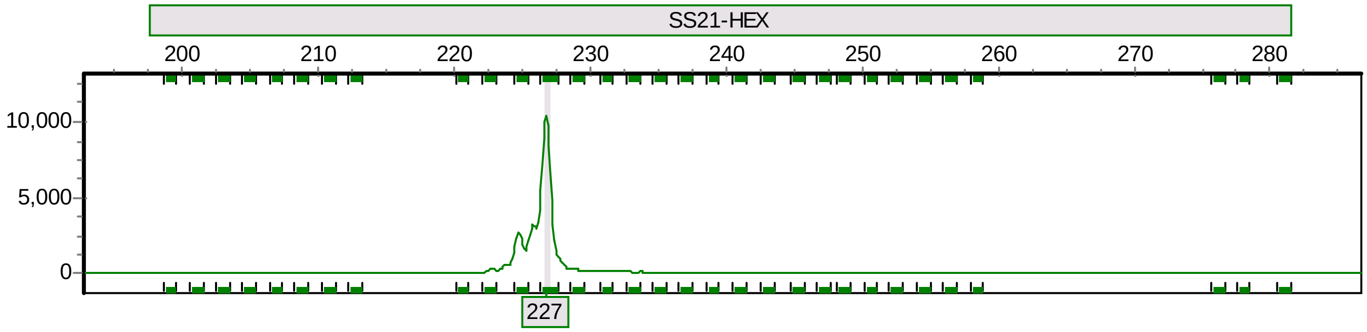

No	Size	Height	Area	Marker	Allele	Difference	Quality	Score	Allele Comments	Sample Comments
1	161.5	27757	190296	SS11-HEX	162	0.00	Pass	500.0	[<Confirmed>]	
2	226.8	10379	78334	SS21-HEX	227	0.20	Pass	500.0	[<Confirmed>]	

Sample 21: SSS13_SS20_SS11_SS21_SS02_SS19_HBB30_L13.fsa

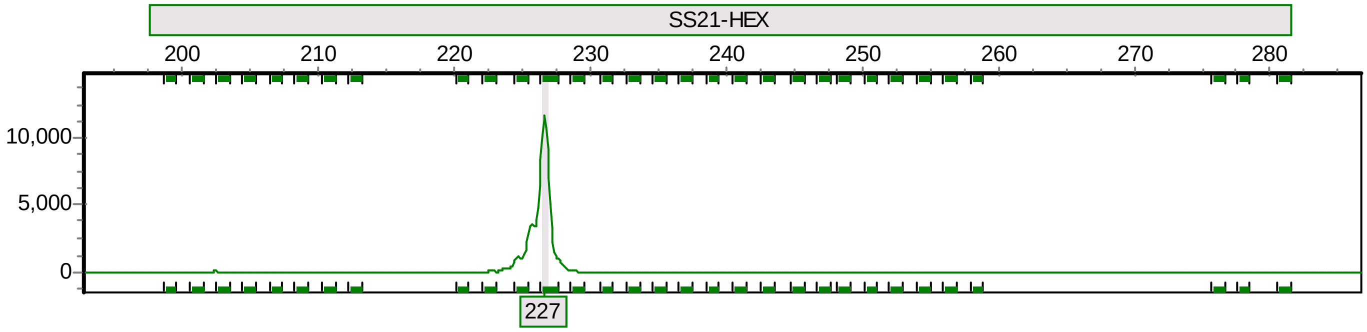

No	Size	Height	Area	Marker	Allele	Difference	Quality	Score	Allele Comments	Sample Comments
1	165.5	10324	69049	SS11-HEX	166	0.10	Pass	500.0	[<Confirmed>]	
2	226.7	11501	88061	SS21-HEX	227	0.30	Pass	500.0	[<Confirmed>]	

Sample 22: SSS13_SS20_SS11_SS21_SS02_SS19_HBB31_P07.fsa

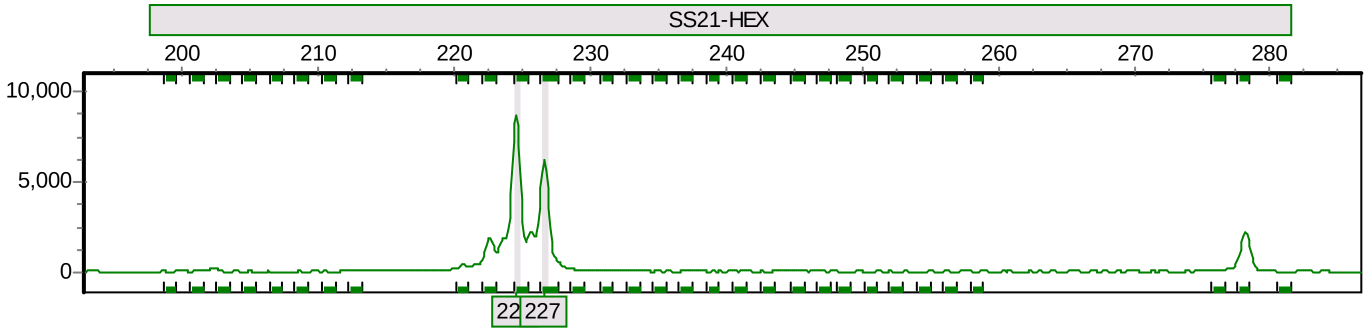

No	Size	Height	Area	Marker	Allele	Difference	Quality	Score	Allele Comments	Sample Comments
1	145.9	13446	97822	SS11-HEX	146	0.30	Pass	500.0	[<Confirmed>]	
2	161.4	7174	46412	SS11-HEX	162	0.10	Pass	500.0	[<Confirmed>]	
3	224.6	8644	64244	SS21-HEX	225	0.40	Pass	500.0	[<Confirmed>]	
4	226.7	6172	48958	SS21-HEX	227	0.30	Pass	500.0	[<Confirmed>]	

Sample 23: SSS13_SS20_SS11_SS21_SS02_SS19_HBB32_B15.fsa

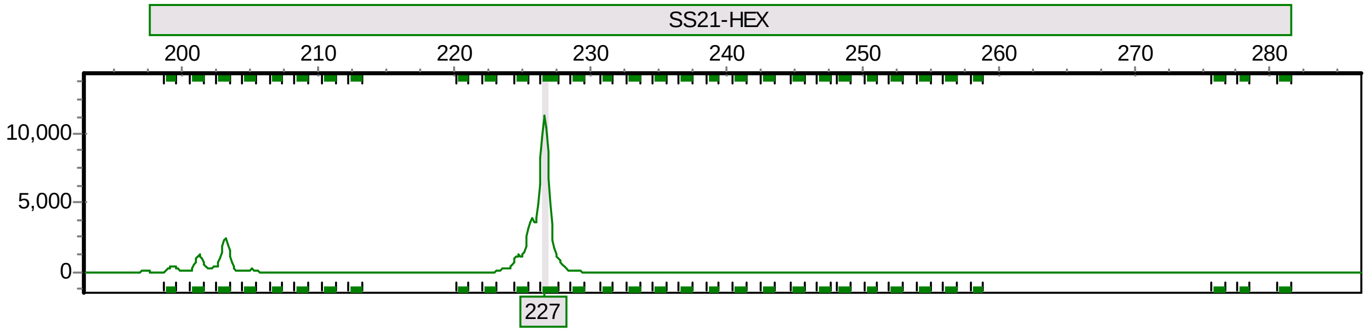

No	Size	Height	Area	Marker	Allele	Difference	Quality	Score	Allele Comments	Sample Comments
1	156.0	11132	70153	SS11-HEX	156	0.10	Pass	500.0	[<Confirmed>]	
2	165.5	6467	43706	SS11-HEX	166	0.10	Pass	500.0	[<Confirmed>]	
3	226.7	11200	89673	SS21-HEX	227	0.30	Pass	500.0	[<Confirmed>]	

Sample 24: SSS13_SS20_SS11_SS21_SS02_SS19_HBB33_N13.fsa

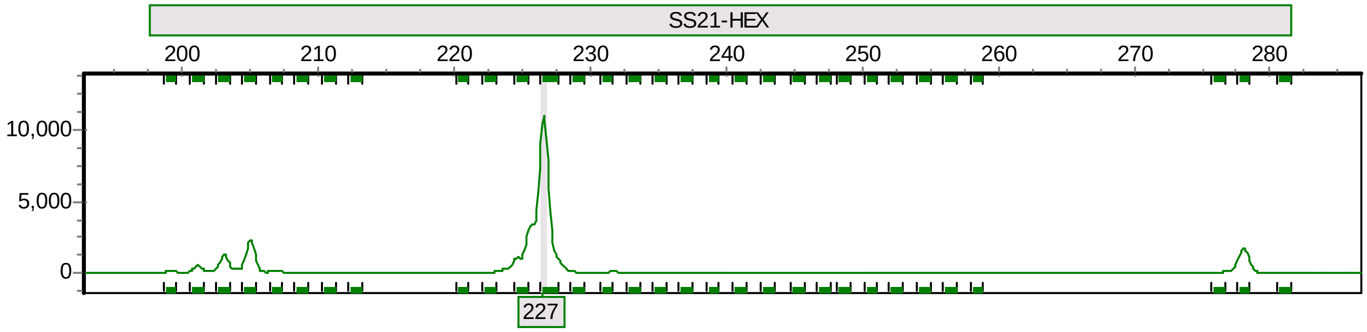

No	Size	Height	Area	Marker	Allele	Difference	Quality	Score	Allele Comments	Sample Comments
1	165.5	10102	71417	SS11-HEX	166	0.10	Pass	500.0	[<Confirmed>]	
2	226.6	10907	89303	SS21-HEX	227	0.40	Pass	500.0	[<Confirmed>]	

Sample 25: SSS13_SS20_SS11_SS21_SS02_SS19_HBB34_P13.fsa

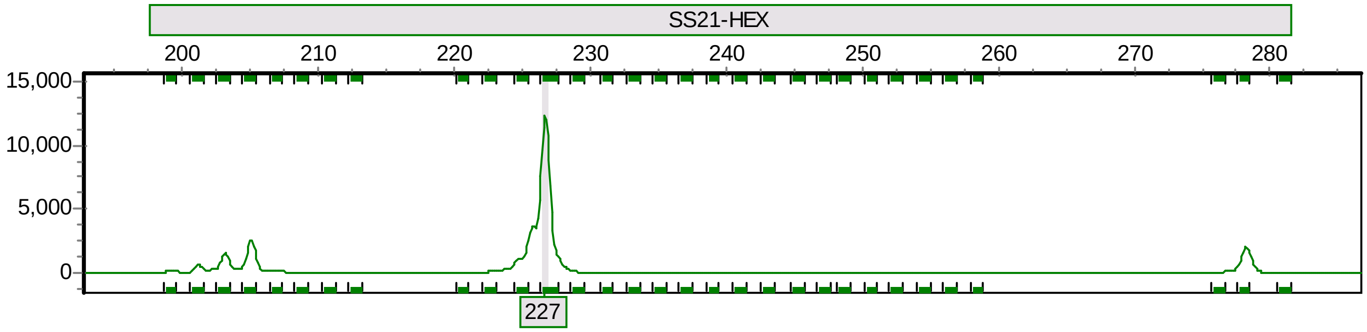

No	Size	Height	Area	Marker	Allele	Difference	Quality	Score	Allele Comments	Sample Comments
1	165.5	12171	81987	SS11-HEX	166	0.10	Pass	500.0	[<Confirmed>]	
2	226.7	12275	98204	SS21-HEX	227	0.30	Pass	500.0	[<Confirmed>]	

Sample 26: SSS13_SS20_SS11_SS21_SS02_SS19_HBB35_C15.fsa

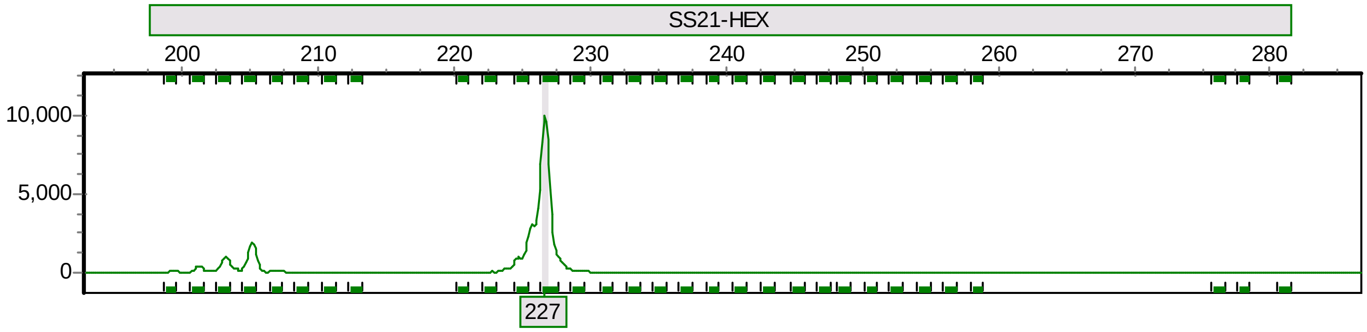

No	Size	Height	Area	Marker	Allele	Difference	Quality	Score	Allele Comments	Sample Comments
1	146.8	12089	71179	SS11-HEX	146	0.60	Pass	500.0	[<Confirmed>]	
2	165.6	5651	39602	SS11-HEX	166	0.20	Pass	500.0	[<Confirmed>]	
3	226.7	10008	81586	SS21-HEX	227	0.30	Pass	500.0	[<Confirmed>]	

Sample 27: SSS13_SS20_SS11_SS21_SS02_SS19_HBB36_G05.fsa

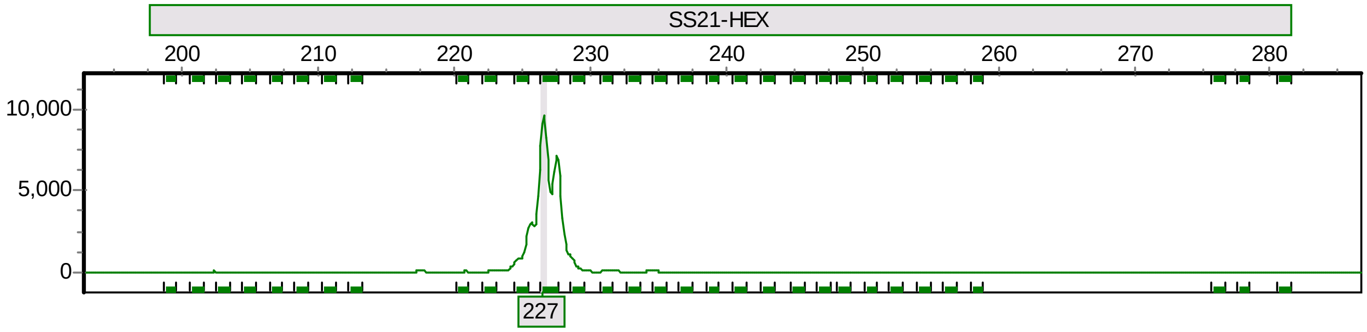

No	Size	Height	Area	Marker	Allele	Difference	Quality	Score	Allele Comments	Sample Comments
1	156.0	9468	60203	SS11-HEX	156	0.10	Pass	500.0	[<Confirmed>]	
2	163.4	6314	42853	SS11-HEX	164	0.00	Pass	500.0	[<Confirmed>]	
3	226.6	9536	75761	SS21-HEX	227	0.40	Pass	500.0	[<Confirmed>]	

Sample 28: SSS13_SS20_SS11_SS21_SS02_SS19_HBB37_N09.fsa

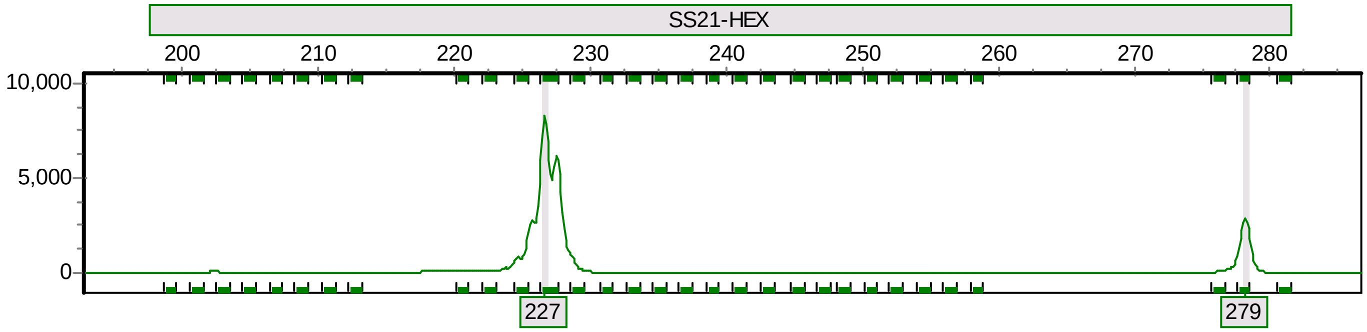

No	Size	Height	Area	Marker	Allele	Difference	Quality	Score	Allele Comments	Sample Comments
1	159.6	14802	93842	SS11-HEX	160	0.00	Pass	500.0	[<Confirmed>]	
2	226.7	8264	63448	SS21-HEX	227	0.30	Pass	500.0	[<Confirmed>]	
3	278.2	2883	24973	SS21-HEX	279	0.10	Pass	363.1	[<Confirmed>]	

Sample 29: SSS13_SS20_SS11_SS21_SS02_SS19_HBB38_M07.fsa

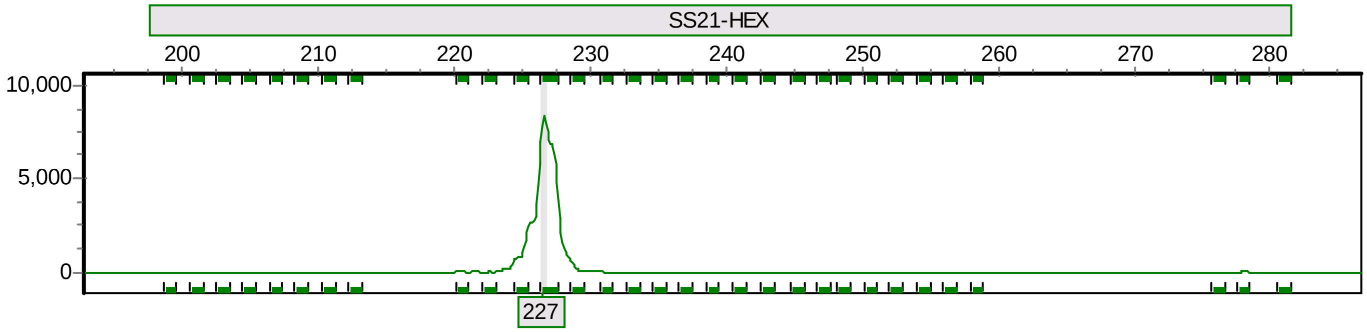

No	Size	Height	Area	Marker	Allele	Difference	Quality	Score	Allele Comments	Sample Comments
1	169.4	7494	52104	SS11-HEX	170	0.10	Pass	500.0	[<Confirmed>]	
2	226.6	8317	115133	SS21-HEX	227	0.40	Pass	500.0	[<Confirmed>]	

Sample 30: SSS13_SS20_SS11_SS21_SS02_SS19_HBB39_F05.fsa

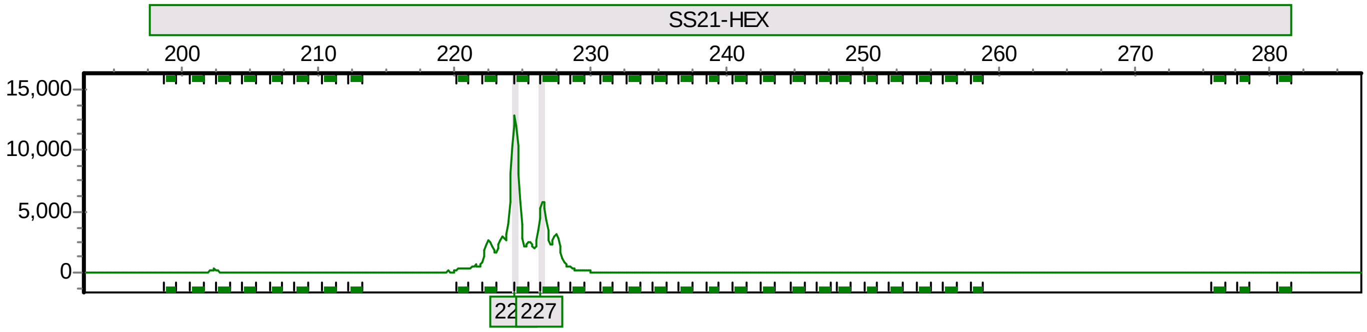

No	Size	Height	Area	Marker	Allele	Difference	Quality	Score	Allele Comments	Sample Comments
1	163.6	15199	99328	SS11-HEX	164	0.20	Pass	500.0	[<Confirmed>]	
2	224.5	12770	92861	SS21-HEX	225	0.50	Pass	500.0	[<Confirmed>]	
3	226.5	5749	42961	SS21-HEX	227	0.50	Pass	500.0	[<Confirmed>]	

Allele Report

Sample 31: SSS13_SS20_SS11_SS21_SS02_SS19_HBB40_D15.fsa

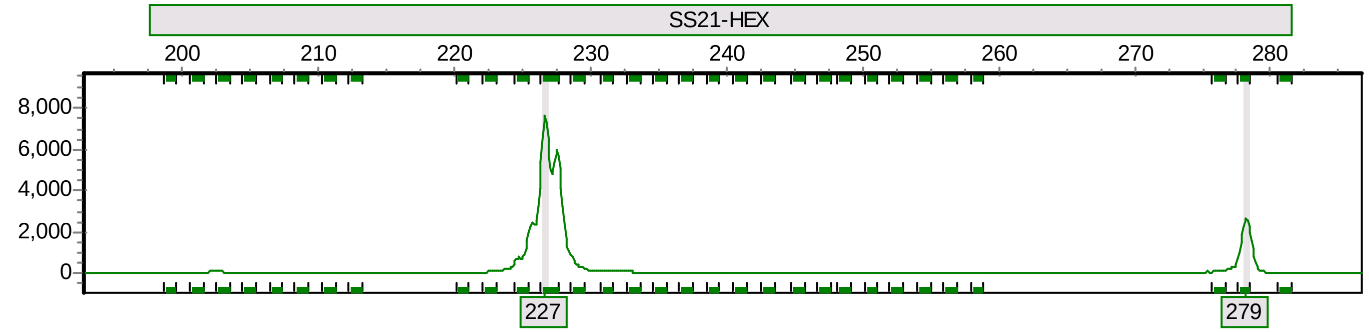

No	Size	Height	Area	Marker	Allele	Difference	Quality	Score	Allele Comments	Sample Comments
1	165.5	9316	64443	SS11-HEX	166	0.10	Pass	500.0	[<Confirmed>]	
2	226.7	7593	58515	SS21-HEX	227	0.30	Pass	500.0	[<Confirmed>]	
3	278.2	2635	22863	SS21-HEX	279	0.10	Pass	314.5	[<Confirmed>]	

Sample 32: SSS13_SS20_SS11_SS21_SS02_SS19_HBB41_L07.fsa

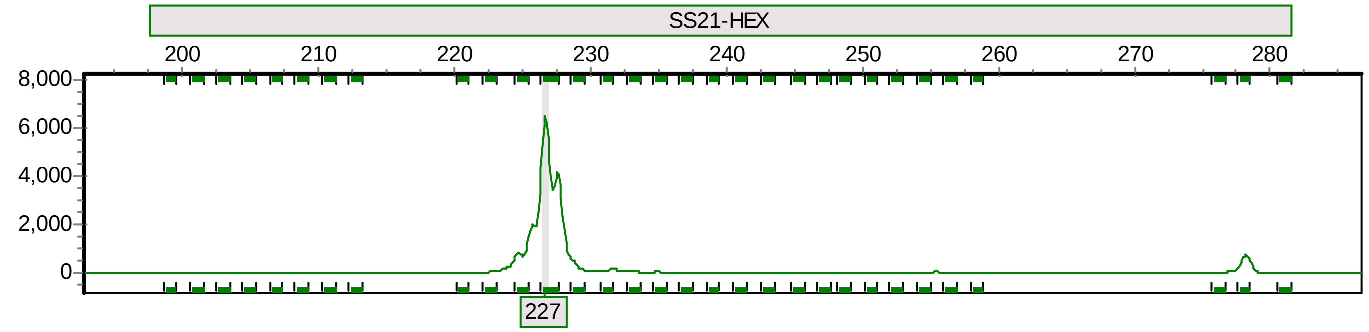

No	Size	Height	Area	Marker	Allele	Difference	Quality	Score	Allele Comments	Sample Comments
1	165.5	3960	27102	SS11-HEX	166	0.10	Pass	500.0	[<Confirmed>]	
2	226.7	6494	52214	SS21-HEX	227	0.30	Pass	500.0	[<Confirmed>]	

Sample 33: SSS13_SS20_SS11_SS21_SS02_SS19_HBB42_M05.fsa

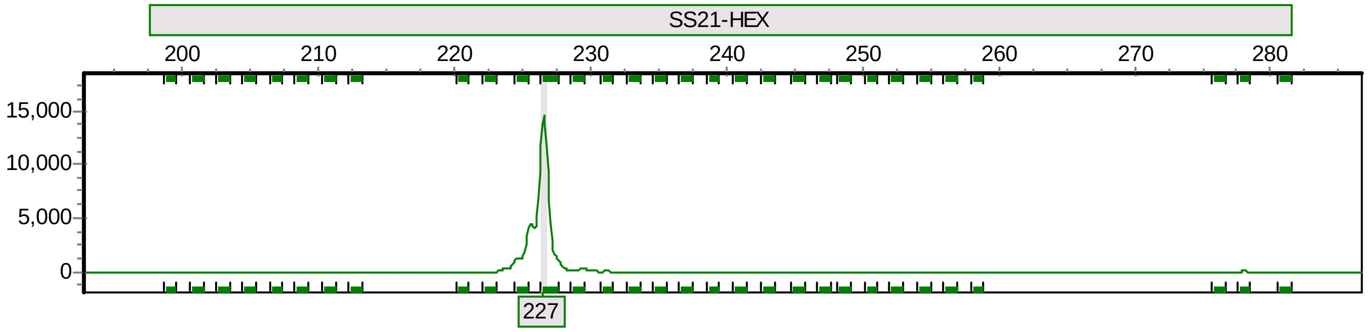

No	Size	Height	Area	Marker	Allele	Difference	Quality	Score	Allele Comments	Sample Comments
1	145.9	23317	165045	SS11-HEX	146	0.30	Pass	500.0	[<Confirmed>]	
2	226.6	14485	109747	SS21-HEX	227	0.40	Pass	500.0	[<Confirmed>]	

Allele Report

Sample 34: SSS13_SS20_SS11_SS21_SS02_SS19_HBB43_D09.fsa

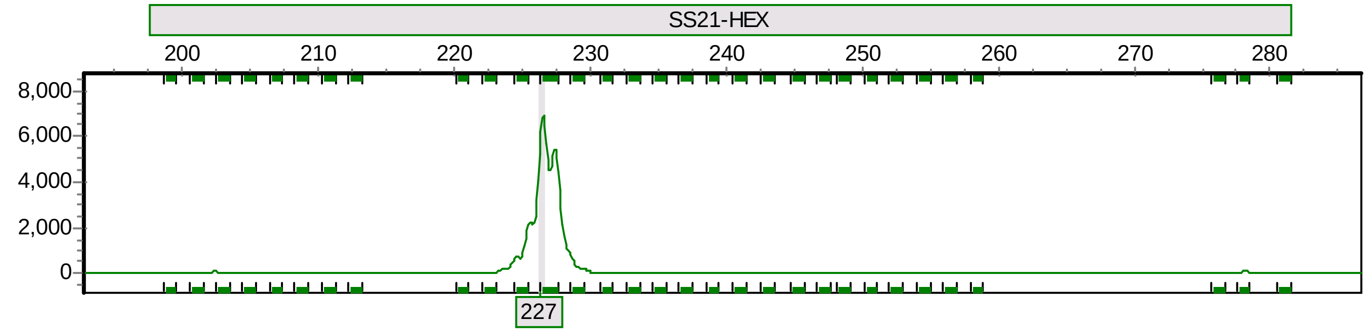

No	Size	Height	Area	Marker	Allele	Difference	Quality	Score	Allele Comments	Sample Comments
1	161.6	9900	69556	SS11-HEX	162	0.10	Pass	500.0	[<Confirmed>]	
2	226.5	6837	53876	SS21-HEX	227	0.50	Pass	500.0	[<Confirmed>]	

Sample 35: SSS13_SS20_SS11_SS21_SS02_SS19_HBB44_H11.fsa

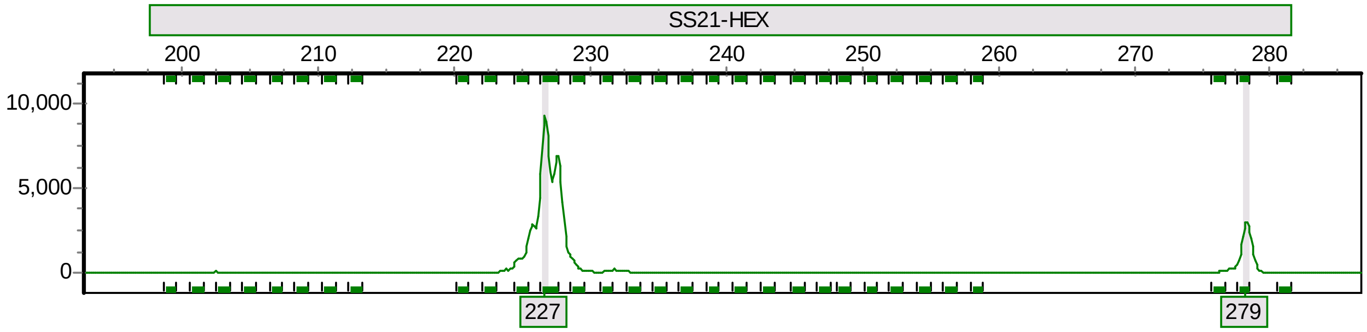

No	Size	Height	Area	Marker	Allele	Difference	Quality	Score	Allele Comments	Sample Comments
1	165.5	9278	63703	SS11-HEX	166	0.10	Pass	500.0	[<Confirmed>]	
2	226.7	9271	74528	SS21-HEX	227	0.30	Pass	500.0	[<Confirmed>]	
3	278.3	3007	25048	SS21-HEX	279	0.20	Pass	412.9	[<Confirmed>]	

Sample 36: SSS13_SS20_SS11_SS21_SS02_SS19_HBB45_F13.fsa

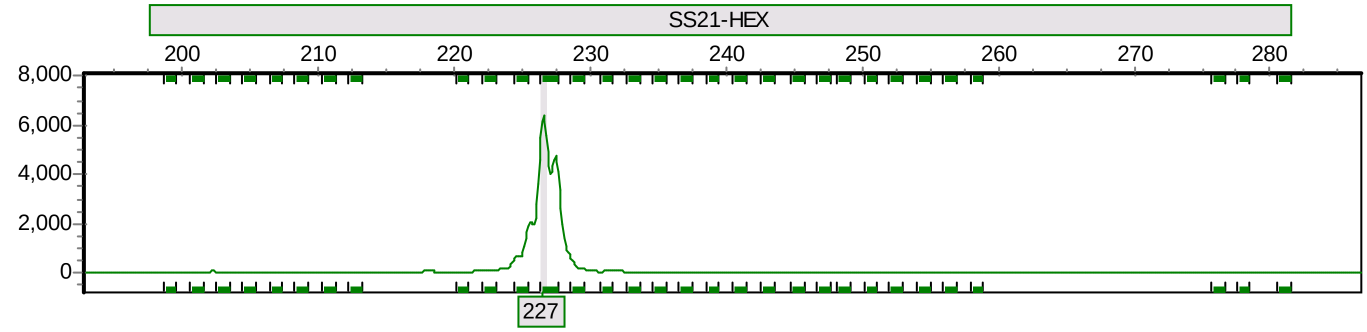

No	Size	Height	Area	Marker	Allele	Difference	Quality	Score	Allele Comments	Sample Comments
1	159.6	8077	53489	SS11-HEX	160	0.00	Pass	500.0	[<Confirmed>]	
2	226.6	6341	49555	SS21-HEX	227	0.40	Pass	500.0	[<Confirmed>]	

Allele Report

Sample 37: SSS13_SS20_SS11_SS21_SS02_SS19_HBB46_F07.fsa

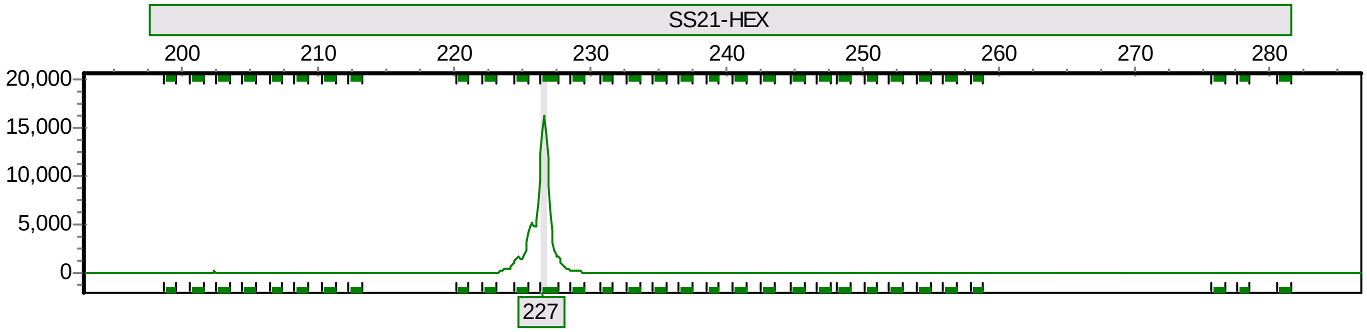

No	Size	Height	Area	Marker	Allele	Difference	Quality	Score	Allele Comments	Sample Comments
1	163.5	16323	109437	SS11-HEX	164	0.10	Pass	500.0	[<Confirmed>]	
2	226.6	16193	122938	SS21-HEX	227	0.40	Pass	500.0	[<Confirmed>]	

Sample 38: SSS13_SS20_SS11_SS21_SS02_SS19_HBB47_A05.fsa

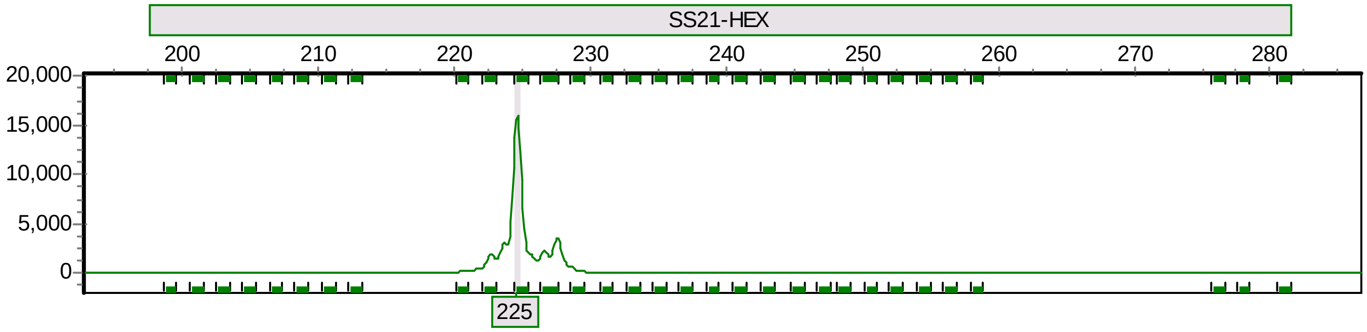

No	Size	Height	Area	Marker	Allele	Difference	Quality	Score	Allele Comments	Sample Comments
1	155.9	10458	64030	SS11-HEX	156	0.00	Pass	500.0	[<Confirmed>]	
2	165.5	19641	128489	SS11-HEX	166	0.10	Pass	500.0	[<Confirmed>]	
3	224.7	15890	116817	SS21-HEX	225	0.30	Pass	500.0	[<Confirmed>]	

Sample 39: SSS13_SS20_SS11_SS21_SS02_SS19_HBB48_B13.fsa

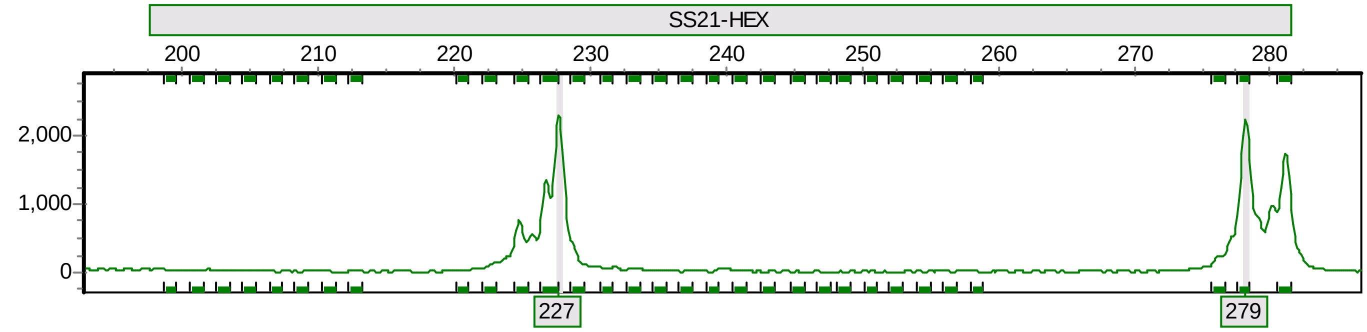

No	Size	Height	Area	Marker	Allele	Difference	Quality	Score	Allele Comments	Sample Comments
1	161.4	20646	140315	SS11-HEX	162	0.10	Pass	500.0	[<Confirmed>]	
2	227.7	2298	18546	SS21-HEX	227	0.70	Pass	238.1	[<Confirmed>]	
3	278.2	2262	19668	SS21-HEX	279	0.10	Pass	214.1	[<Confirmed>]	
4	281.1	1741	15171	SS21-HEX	281	0.00	Undetermined	128.7	[<Deleted>]	

Allele Report

Sample 40: SSS13_SS20_SS11_SS21_SS02_SS19_HBB49_L05.fsa

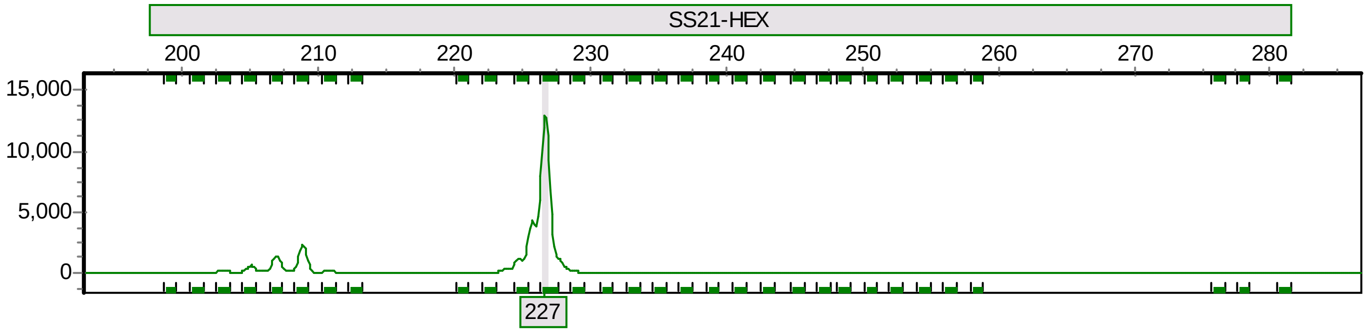

No	Size	Height	Area	Marker	Allele	Difference	Quality	Score	Allele Comments	Sample Comments
1	145.8	14311	102691	SS11-HEX	146	0.40	Pass	500.0	[<Confirmed>]	
2	165.3	6404	42645	SS11-HEX	166	0.10	Pass	500.0	[<Confirmed>]	
3	226.7	12829	96239	SS21-HEX	227	0.30	Pass	500.0	[<Confirmed>]	

Sample 41: SSS13_SS20_SS11_SS21_SS02_SS19_HBB4_E07.fsa

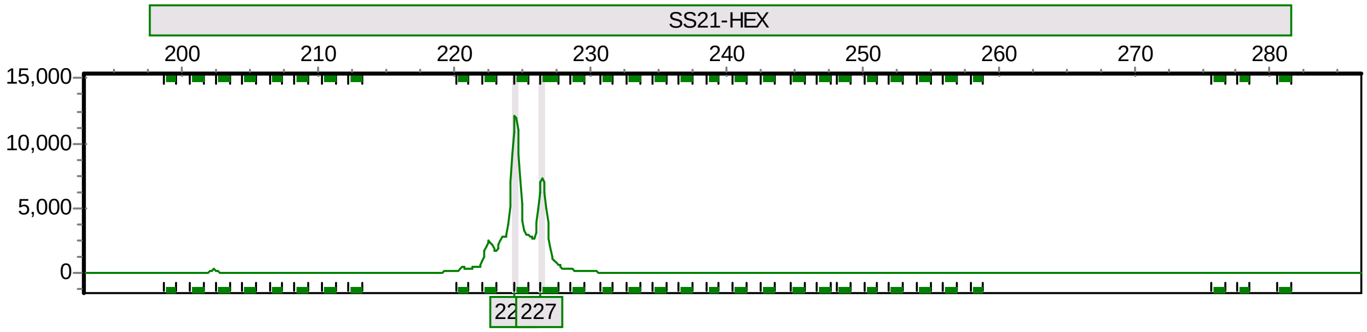

No	Size	Height	Area	Marker	Allele	Difference	Quality	Score	Allele Comments	Sample Comments
1	145.9	22812	163955	SS11-HEX	146	0.30	Pass	500.0	[<Confirmed>]	
2	169.3	8107	54941	SS11-HEX	170	0.00	Pass	500.0	[<Confirmed>]	
3	224.5	12051	96184	SS21-HEX	225	0.50	Pass	500.0	[<Confirmed>]	
4	226.5	7354	59468	SS21-HEX	227	0.50	Pass	500.0	[<Confirmed>]	

Sample 42: SSS13_SS20_SS11_SS21_SS02_SS19_HBB5_H13.fsa

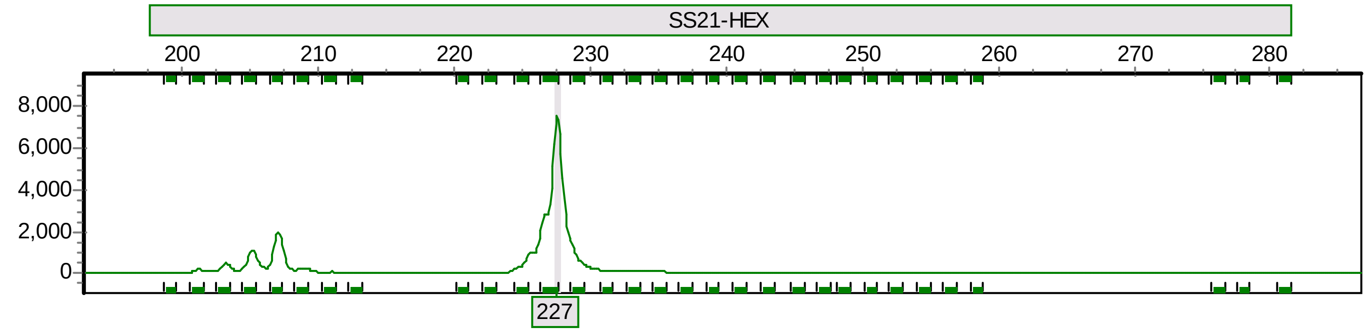

No	Size	Height	Area	Marker	Allele	Difference	Quality	Score	Allele Comments	Sample Comments
1	165.5	9502	71323	SS11-HEX	166	0.10	Pass	500.0	[<Confirmed>]	
2	227.6	7512	63723	SS21-HEX	227	0.60	Pass	500.0	[<Confirmed>]	

Sample 43: SSS13_SS20_SS11_SS21_SS02_SS19_HBB6_C05.fsa

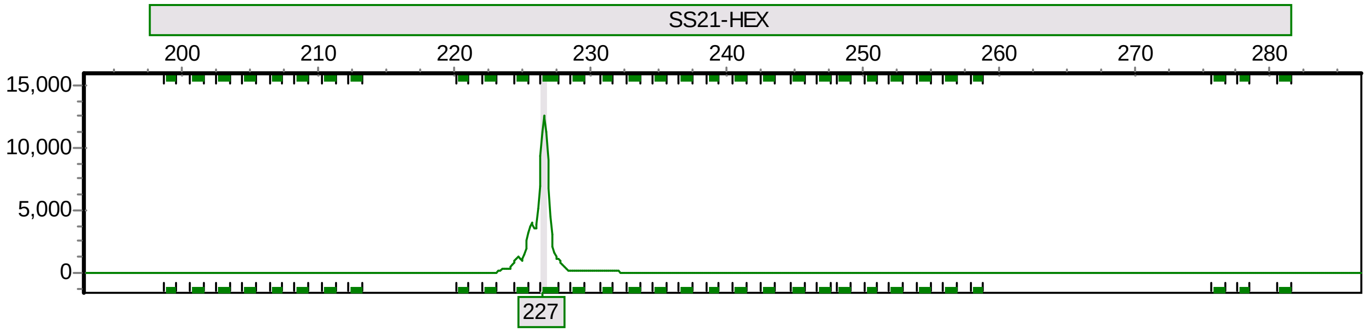

No	Size	Height	Area	Marker	Allele	Difference	Quality	Score	Allele Comments	Sample Comments
1	161.5	9019	58479	SS11-HEX	162	0.00	Pass	500.0	[<Confirmed>]	
2	165.4	6025	39768	SS11-HEX	166	0.00	Pass	500.0	[<Confirmed>]	
3	226.6	12531	92218	SS21-HEX	227	0.40	Pass	500.0	[<Confirmed>]	

Sample 44: SSS13_SS20_SS11_SS21_SS02_SS19_HBB7_N05.fsa

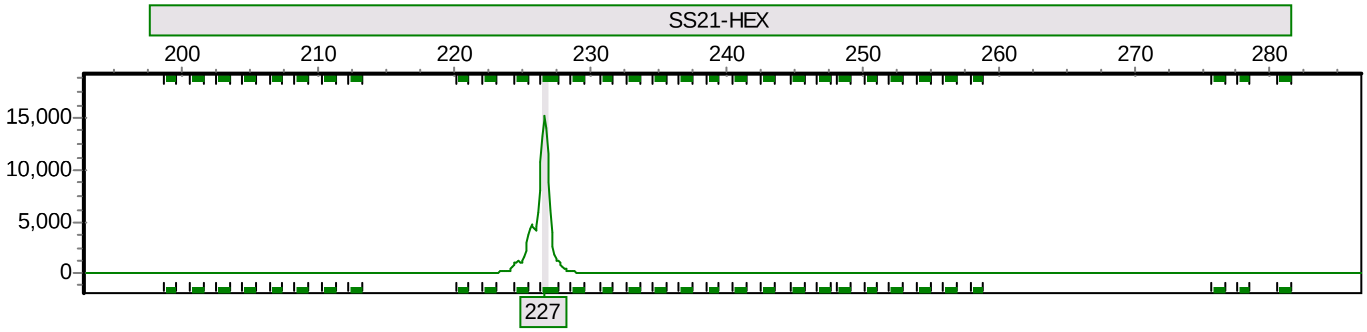

No	Size	Height	Area	Marker	Allele	Difference	Quality	Score	Allele Comments	Sample Comments
1	161.6	8279	54233	SS11-HEX	162	0.10	Pass	500.0	[<Confirmed>]	
2	171.4	5659	37475	SS11-HEX	172	0.10	Pass	500.0	[<Confirmed>]	
3	226.7	15168	112566	SS21-HEX	227	0.30	Pass	500.0	[<Confirmed>]	

Sample 45: SSS13_SS20_SS11_SS21_SS02_SS19_HBB8_P11.fsa

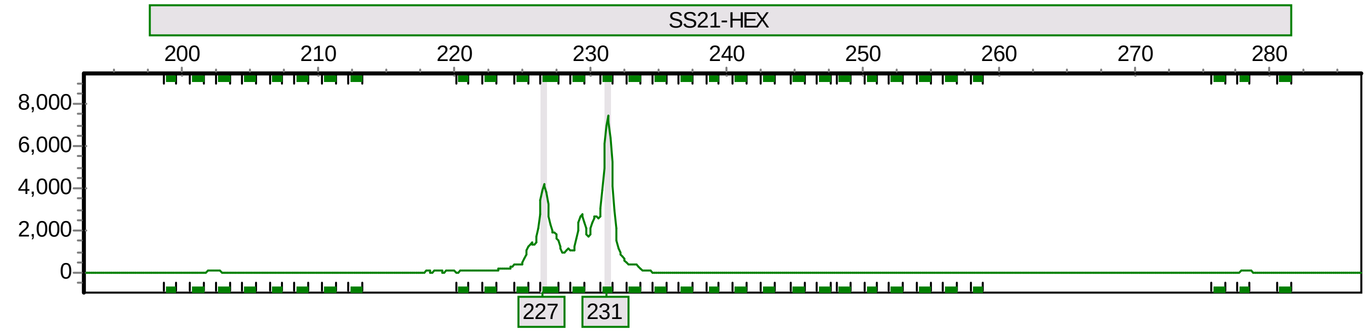

No	Size	Height	Area	Marker	Allele	Difference	Quality	Score	Allele Comments	Sample Comments
1	165.4	11245	77073	SS11-HEX	166	0.00	Pass	500.0	[<Confirmed>]	
2	226.6	4185	33895	SS21-HEX	227	0.40	Pass	500.0	[<Confirmed>]	
3	231.3	7361	59769	SS21-HEX	231	0.00	Pass	500.0	[<Confirmed>]	

Allele Report

Sample 46: SSS13_SS20_SS11_SS21_SS02_SS19_HBB9_I05.fsa

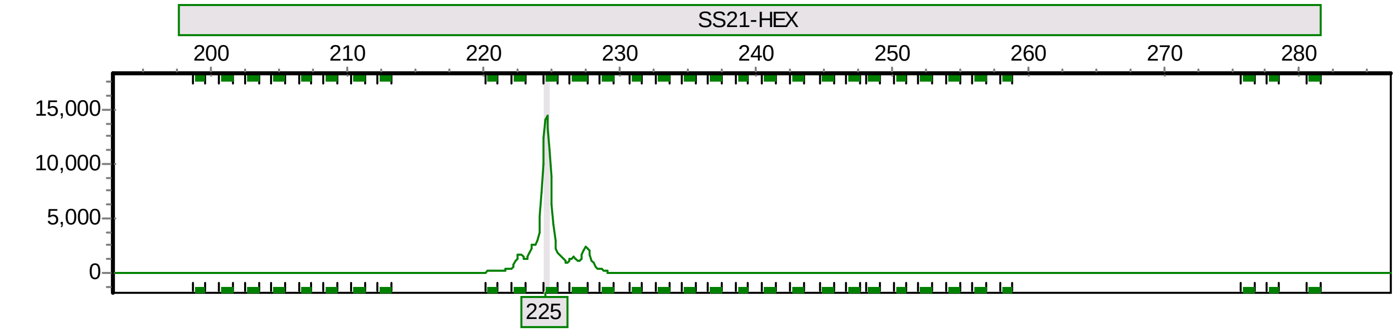

No	Size	Height	Area	Marker	Allele	Difference	Quality	Score	Allele Comments	Sample Comments
1	156.0	20586	134073	SS11-HEX	156	0.10	Pass	500.0	[<Confirmed>]	
2	224.7	14284	110439	SS21-HEX	225	0.30	Pass	500.0	[<Confirmed>]	

Sample 47: SSS13_SS20_SS11_SS21_SS02_SS19_HBN10_G11.fsa

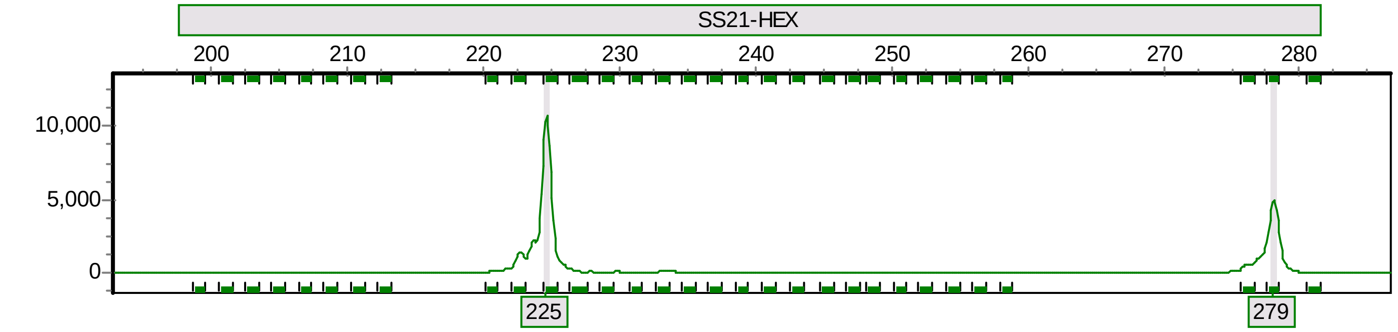

No	Size	Height	Area	Marker	Allele	Difference	Quality	Score	Allele Comments	Sample Comments
1	163.4	12259	84704	SS11-HEX	164	0.00	Pass	500.0	[<Confirmed>]	
2	167.4	11145	74811	SS11-HEX	168	0.00	Pass	500.0	[<Confirmed>]	
3	224.7	10647	82119	SS21-HEX	225	0.30	Pass	500.0	[<Confirmed>]	
4	278.1	4980	43052	SS21-HEX	279	0.00	Pass	500.0	[<Confirmed>]	

Sample 48: SSS13_SS20_SS11_SS21_SS02_SS19_HBN6_O01.fsa

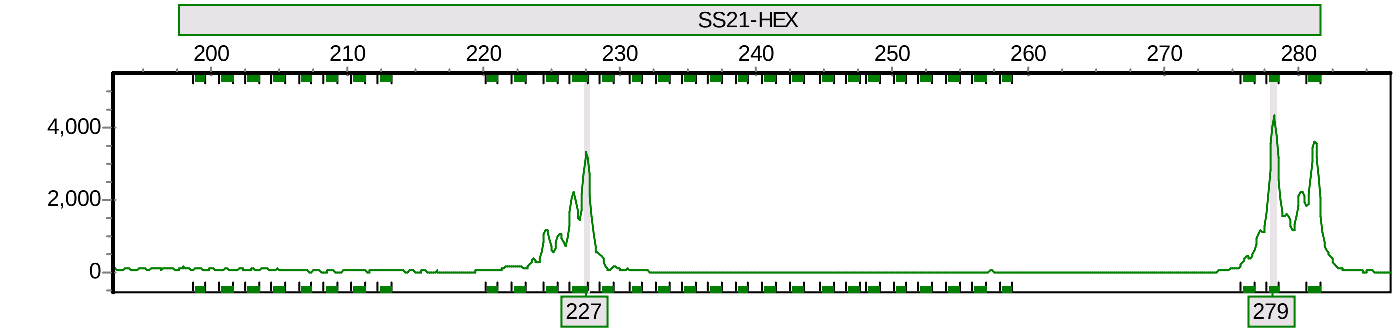

No	Size	Height	Area	Marker	Allele	Difference	Quality	Score	Allele Comments	Sample Comments
1	145.9	8343	58226	SS11-HEX	146	0.30	Pass	500.0	[<Confirmed>]	
2	163.3	19003	119376	SS11-HEX	164	0.10	Pass	500.0	[<Confirmed>]	
3	227.6	3366	23736	SS21-HEX	227	0.60	Pass	500.0	[<Confirmed>]	
4	278.1	4329	34306	SS21-HEX	279	0.00	Pass	500.0	[<Confirmed>]	
5	281.1	3646	31002	SS21-HEX	281	0.00	Undetermined	465.2	[<Deleted>]	

Sample 49: SSS13_SS20_SS11_SS21_SS02_SS19_HBN9_D03.fsa Run date and time: 10/09/2021 - 22:22:10 -> 10/09/2021 - 22:49:12

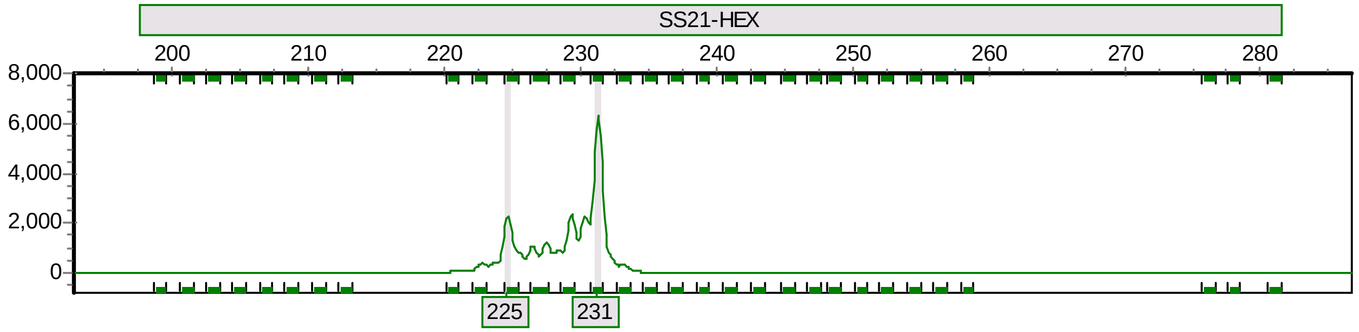

No	Size	Height	Area	Marker	Allele	Difference	Quality	Score	Allele Comments	Sample Comments
1	146.0	17221	122185	SS11-HEX	146	0.20	Pass	500.0	[<Confirmed>]	
2	163.6	6887	44741	SS11-HEX	164	0.20	Pass	500.0	[<Confirmed>]	
3	224.7	2312	17768	SS21-HEX	225	0.30	Pass	309.2	[<Confirmed>]	
4	231.3	6281	46615	SS21-HEX	231	0.00	Pass	500.0	[<Confirmed>]	

Sample 50: SSS13_SS20_SS11_SS21_SS02_SS19_HCW1_M09.fsa

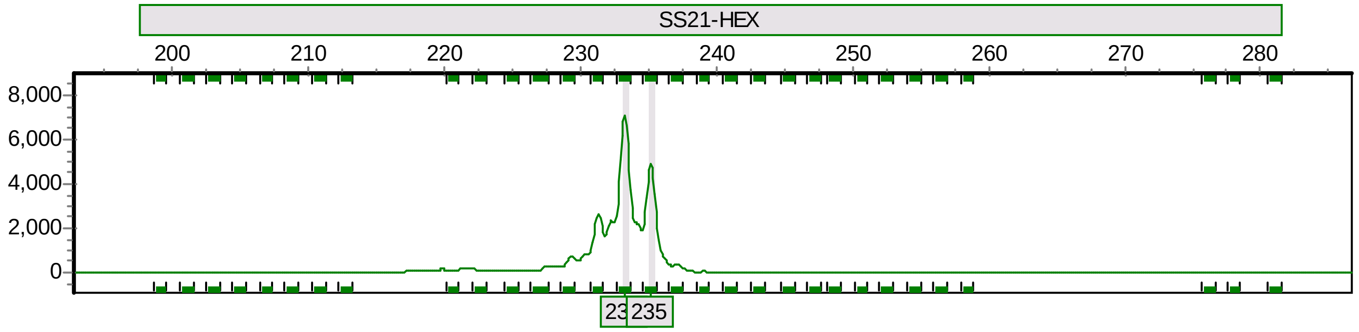

No	Size	Height	Area	Marker	Allele	Difference	Quality	Score	Allele Comments	Sample Comments
1	139.4	23522	175912	SS11-HEX	140	0.00	Pass	500.0	[<Confirmed>]	
2	145.9	18144	134221	SS11-HEX	146	0.30	Pass	500.0	[<Confirmed>]	
3	233.3	7063	55893	SS21-HEX	233	0.00	Pass	500.0	[<Confirmed>]	
4	235.2	4879	40457	SS21-HEX	235	0.00	Pass	500.0	[<Confirmed>]	

Sample 51: SSS13_SS20_SS11_SS21_SS02_SS19_HCW2_A11.fsa

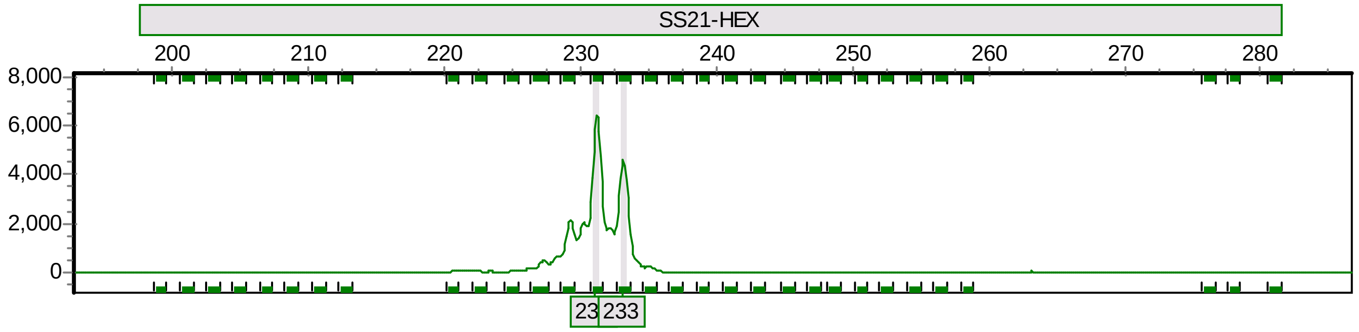

No	Size	Height	Area	Marker	Allele	Difference	Quality	Score	Allele Comments	Sample Comments
1	139.5	36479	281405	SS11-HEX	140	0.10	Pass	500.0	[<SAT (Repaired)><Confirmed>]	
2	231.2	6368	49032	SS21-HEX	231	0.10	Pass	500.0	[<Confirmed>]	
3	233.2	4569	36398	SS21-HEX	233	0.10	Pass	500.0	[<Confirmed>]	

Allele Report

Sample 52: SSS13_SS20_SS11_SS21_SS02_SS19_HCW3_G13.fsa

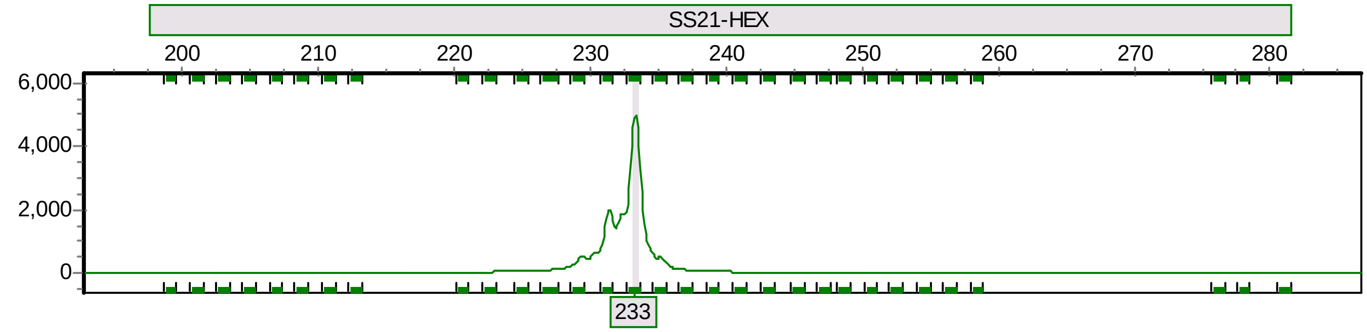

No	Size	Height	Area	Marker	Allele	Difference	Quality	Score	Allele Comments	Sample Comments
1	139.2	22541	176033	SS11-HEX	140	0.20	Pass	500.0	[<Confirmed>]	
2	233.4	4953	42940	SS21-HEX	233	0.10	Pass	500.0	[<Confirmed>]	

Sample 53: SSS13_SS20_SS11_SS21_SS02_SS19_HCW4_E09.fsa

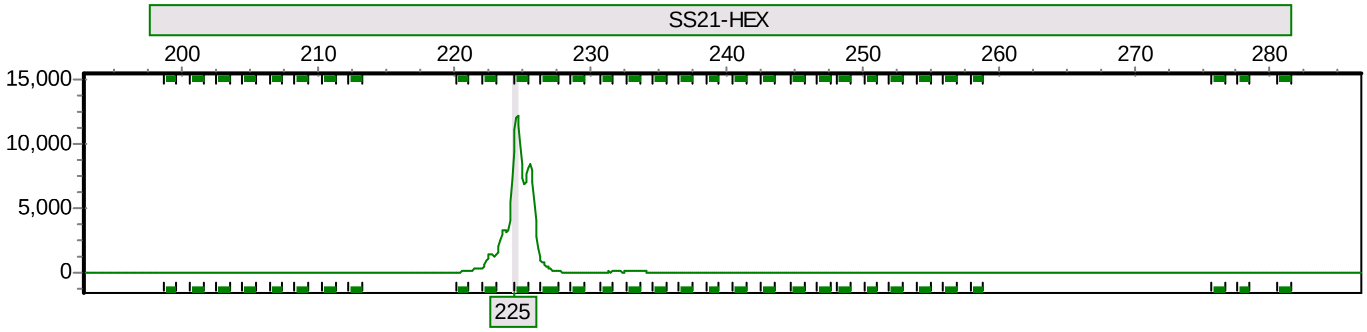

No	Size	Height	Area	Marker	Allele	Difference	Quality	Score	Allele Comments	Sample Comments
1	157.6	31007	202196	SS11-HEX	158	0.10	Pass	500.0	[<Confirmed>]	
2	224.5	11093	160536	SS21-HEX	225	0.50	Pass	500.0	[<Confirmed>]	

Sample 54: SSS13_SS20_SS11_SS21_SS02_SS19_HCW5_C11.fsa

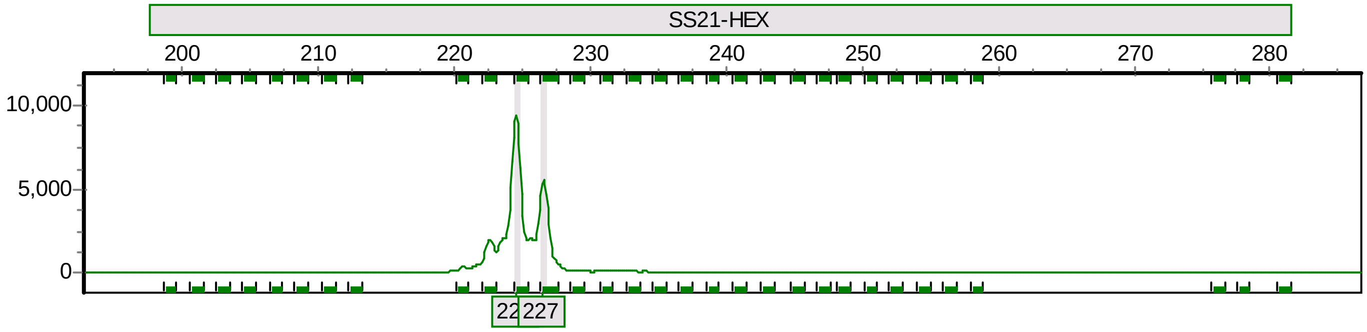

No	Size	Height	Area	Marker	Allele	Difference	Quality	Score	Allele Comments	Sample Comments
1	161.6	8371	57560	SS11-HEX	162	0.10	Pass	500.0	[<Confirmed>]	
2	165.5	6484	45090	SS11-HEX	166	0.10	Pass	500.0	[<Confirmed>]	
3	224.6	9378	75595	SS21-HEX	225	0.40	Pass	500.0	[<Confirmed>]	
4	226.6	5535	45062	SS21-HEX	227	0.40	Pass	500.0	[<Confirmed>]	

Allele Report

Sample 55: SSS13_SS20_SS11_SS21_SS02_SS19_HCW6_D01.fsa

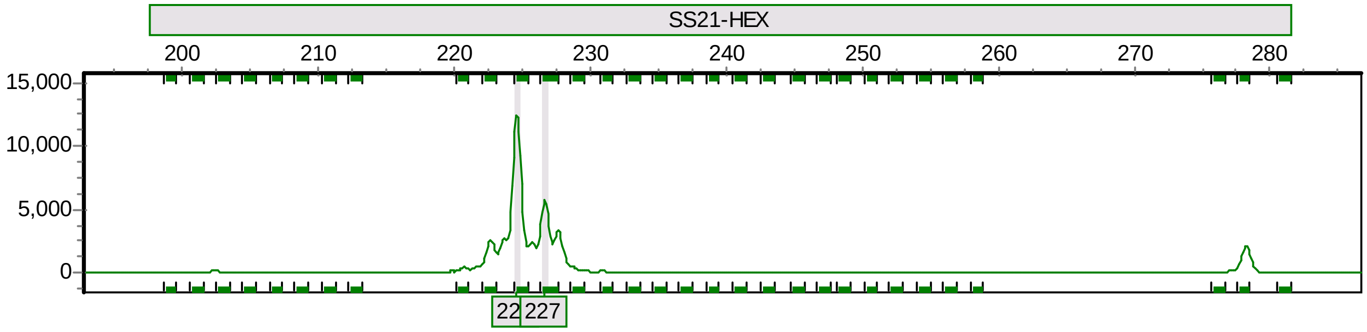

No	Size	Height	Area	Marker	Allele	Difference	Quality	Score	Allele Comments	Sample Comments
1	159.6	15549	92973	SS11-HEX	160	0.00	Pass	500.0	[<Confirmed>]	
2	161.5	11632	75296	SS11-HEX	162	0.00	Pass	500.0	[<Confirmed>]	
3	224.6	12336	92535	SS21-HEX	225	0.40	Pass	500.0	[<Confirmed>]	
4	226.7	5671	41445	SS21-HEX	227	0.30	Pass	500.0	[<Confirmed>]	

Sample 56: SSS13_SS20_SS11_SS21_SS02_SS19_HCW7_G17.fsa

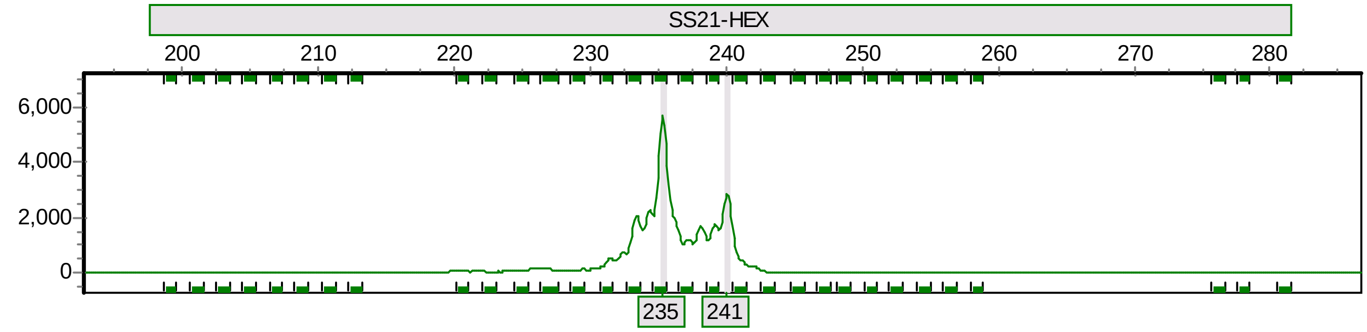

No	Size	Height	Area	Marker	Allele	Difference	Quality	Score	Allele Comments	Sample Comments
1	156.0	22997	150411	SS11-HEX	156	0.10	Pass	500.0	[<Confirmed>]	
2	159.5	18118	115291	SS11-HEX	160	0.10	Pass	500.0	[<Confirmed>]	
3	235.4	5645	45962	SS21-HEX	235	0.20	Pass	500.0	[<Confirmed>]	
4	239.2	1737	12494	SS21-HEX	239	0.10	Pass	46.4	[<Deleted>]	
5	240.1	2837	24650	SS21-HEX	241	1.00	Pass	299.2	[<Confirmed><Edited>]	

Sample 57: SSS13_SS20_SS11_SS21_SS02_SS19_HCW8_A17.fsa

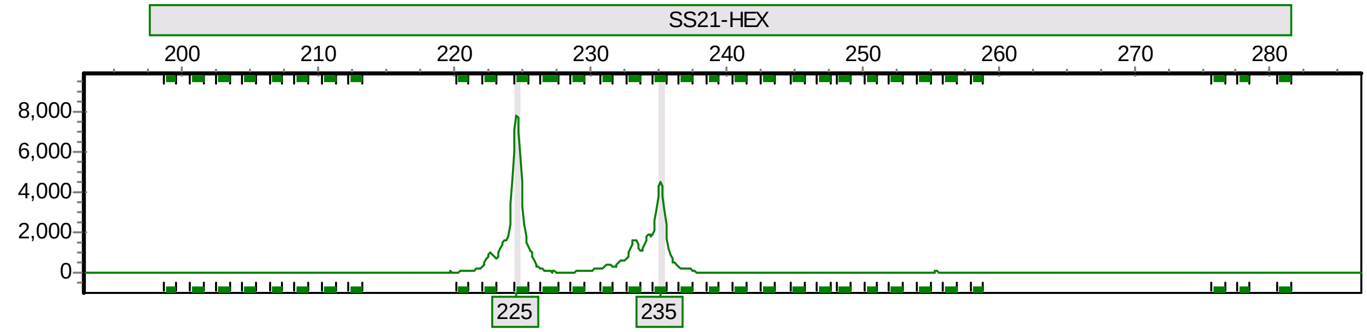

No	Size	Height	Area	Marker	Allele	Difference	Quality	Score	Allele Comments	Sample Comments
1	171.4	17700	125686	SS11-HEX	172	0.10	Pass	500.0	[<Confirmed>]	
2	224.6	7778	61816	SS21-HEX	225	0.40	Pass	500.0	[<Confirmed>]	
3	235.2	4507	37525	SS21-HEX	235	0.00	Pass	500.0	[<Confirmed>]	

Allele Report

Sample 58: SSS13_SS20_SS11_SS21_SS02_SS19_HGC1_C01.fsa

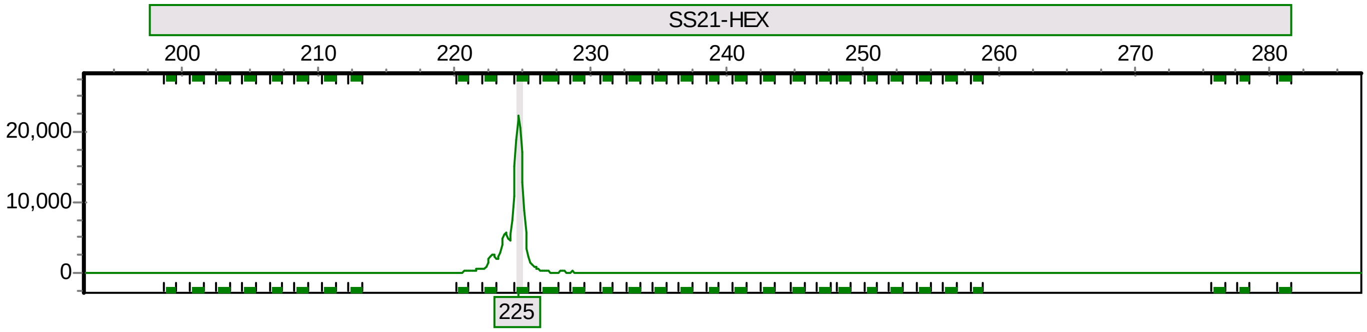

No	Size	Height	Area	Marker	Allele	Difference	Quality	Score	Allele Comments	Sample Comments
1	163.2	31654	211423	SS11-HEX	164	0.20	Pass	500.0	[<Confirmed>]	
2	224.8	22153	160749	SS21-HEX	225	0.20	Pass	500.0	[<Confirmed>]	

Sample 59: SSS13_SS20_SS11_SS21_SS02_SS19_HGC3_E17.fsa

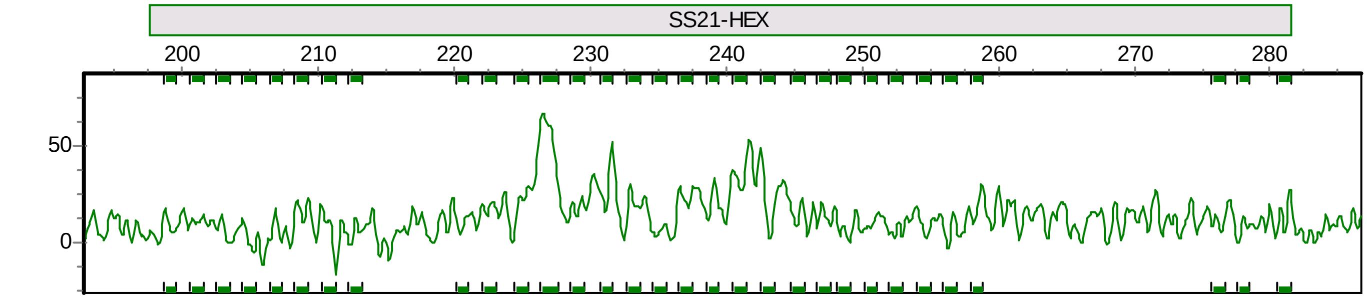

No	Size	Height	Area	Marker	Allele	Difference	Quality	Score	Allele Comments	Sample Comments

Sample 60: SSS13_SS20_SS11_SS21_SS02_SS19_HGC4_A13.fsa

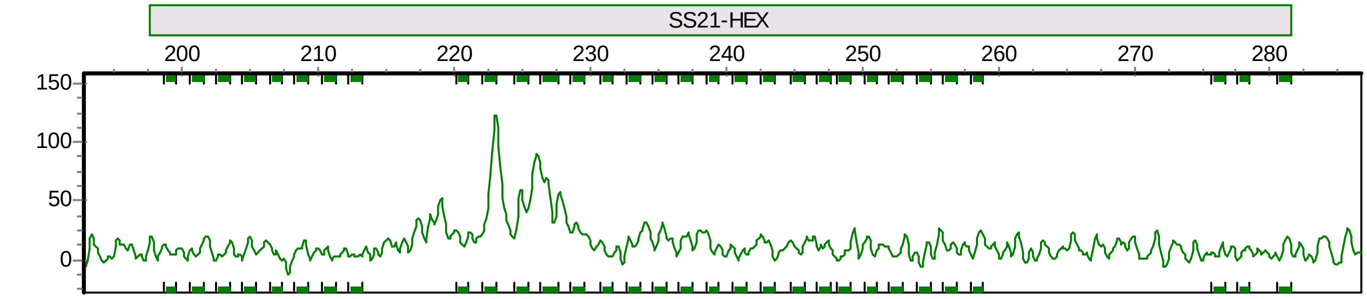

No	Size	Height	Area	Marker	Allele	Difference	Quality	Score	Allele Comments	Sample Comments

Allele Report

Sample 61: SSS13_SS20_SS11_SS21_SS02_SS19_HGY1_A09.fsa

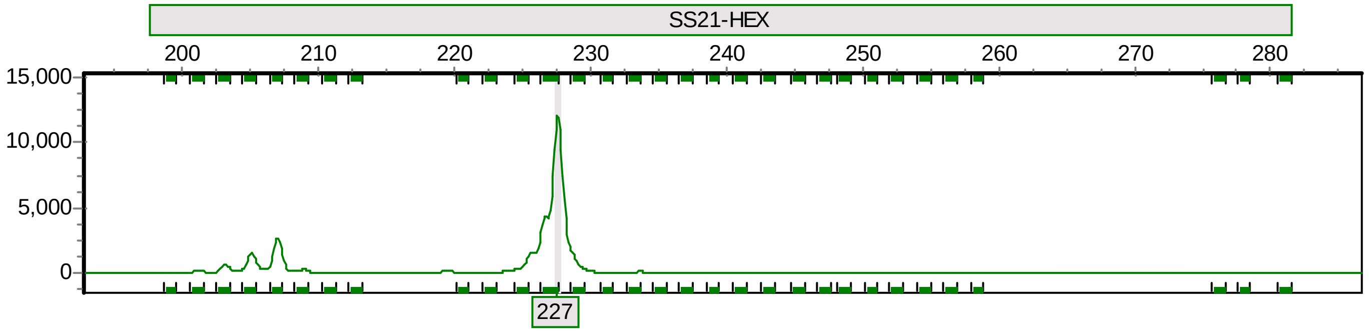

No	Size	Height	Area	Marker	Allele	Difference	Quality	Score	Allele Comments	Sample Comments
1	161.6	10680	75041	SS11-HEX	162	0.10	Pass	500.0	[<Confirmed>]	
2	165.5	7157	48958	SS11-HEX	166	0.10	Pass	500.0	[<Confirmed>]	
3	227.6	11969	98786	SS21-HEX	227	0.60	Pass	500.0	[<Confirmed>]	

Sample 62: SSS13_SS20_SS11_SS21_SS02_SS19_HGY2_I13.fsa

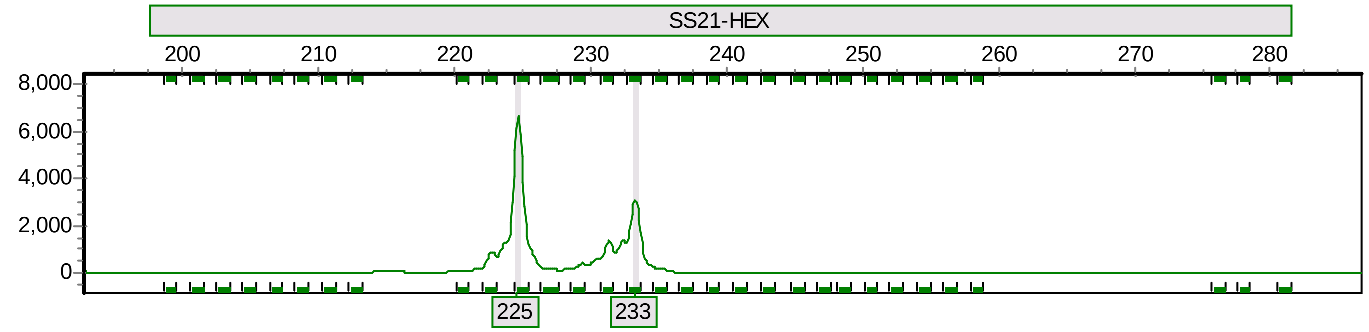

No	Size	Height	Area	Marker	Allele	Difference	Quality	Score	Allele Comments	Sample Comments
1	165.6	5739	40252	SS11-HEX	166	0.20	Pass	500.0	[<Confirmed>]	
2	173.4	15397	108557	SS11-HEX	174	0.10	Pass	500.0	[<Confirmed>]	
3	224.7	6631	53150	SS21-HEX	225	0.30	Pass	500.0	[<Confirmed>]	
4	233.3	3081	25482	SS21-HEX	233	0.00	Pass	402.2	[<Confirmed>]	

Sample 63: SSS13_SS20_SS11_SS21_SS02_SS19_HGY3_I11.fsa

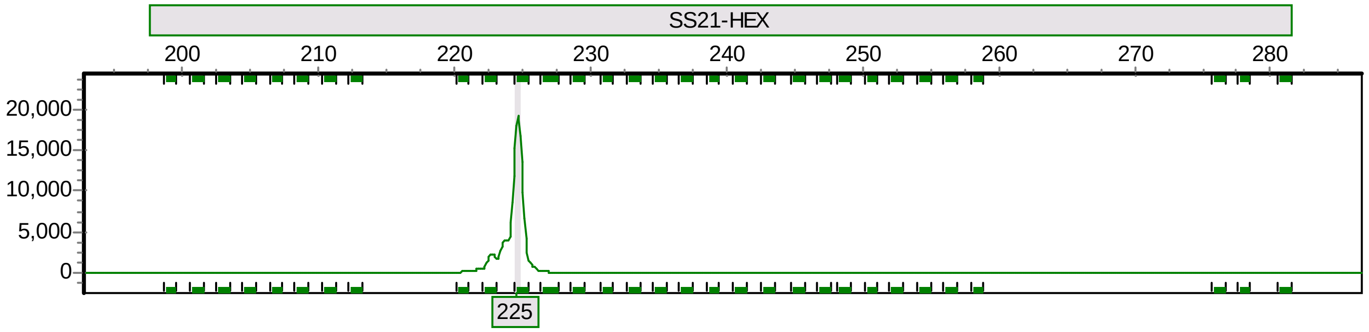

No	Size	Height	Area	Marker	Allele	Difference	Quality	Score	Allele Comments	Sample Comments
1	152.1	33141	251941	SS11-HEX	152	0.00	Pass	500.0	[<Confirmed>]	
2	173.4	21234	147745	SS11-HEX	174	0.10	Pass	500.0	[<Confirmed>]	
3	224.7	19064	146625	SS21-HEX	225	0.30	Pass	500.0	[<Confirmed>]	

Allele Report

Sample 64: SSS13_SS20_SS11_SS21_SS02_SS19_HGY4_I09.fsa

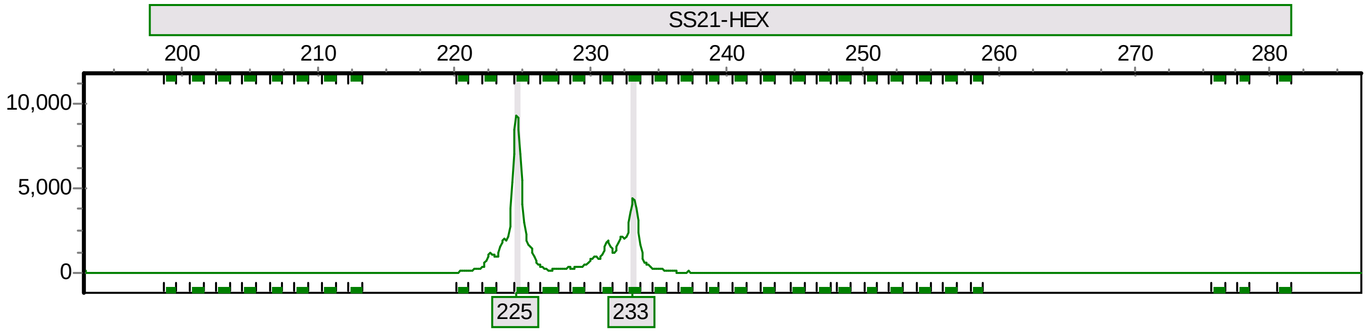

No	Size	Height	Area	Marker	Allele	Difference	Quality	Score	Allele Comments	Sample Comments
1	173.4	36378	261770	SS11-HEX	174	0.10	Pass	500.0	[<SAT (Repaired)><Confirmed>]	
2	224.6	9189	73280	SS21-HEX	225	0.40	Pass	500.0	[<Confirmed>]	
3	233.2	4388	34234	SS21-HEX	233	0.10	Pass	500.0	[<Confirmed>]	

Sample 65: SSS13_SS20_SS11_SS21_SS02_SS19_HGY5_I15.fsa

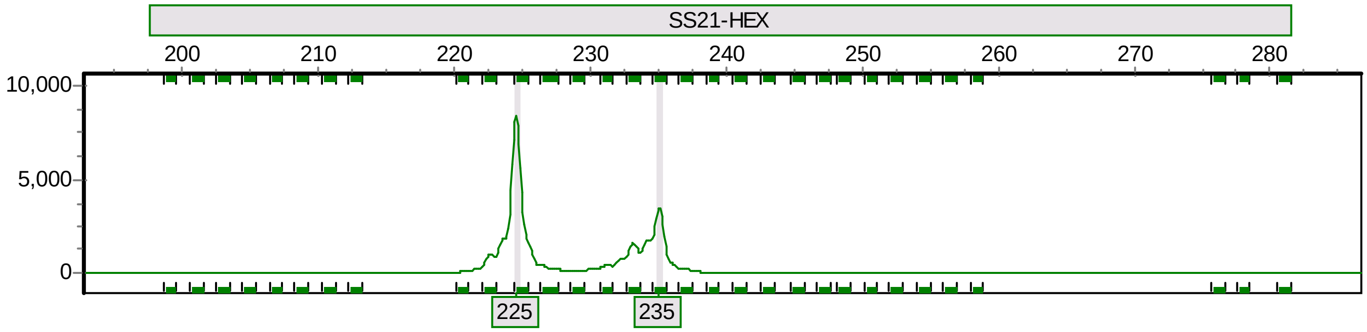

No	Size	Height	Area	Marker	Allele	Difference	Quality	Score	Allele Comments	Sample Comments
1	143.6	31479	243744	SS11-HEX	144	0.10	Pass	500.0	[<Confirmed>]	
2	224.6	8330	66716	SS21-HEX	225	0.40	Pass	500.0	[<Confirmed>]	
3	235.1	3465	27512	SS21-HEX	235	0.10	Pass	435.3	[<Confirmed>]	

Sample 66: SSS13_SS20_SS11_SS21_SS02_SS19_HQZ11_O11.fsa

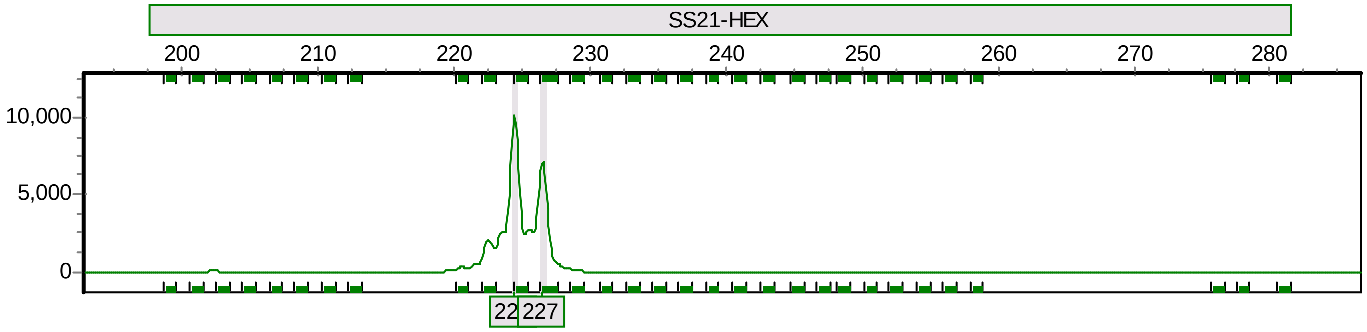

No	Size	Height	Area	Marker	Allele	Difference	Quality	Score	Allele Comments	Sample Comments
1	155.9	18589	119735	SS11-HEX	156	0.00	Pass	500.0	[<Confirmed>]	
2	224.5	10011	77073	SS21-HEX	225	0.50	Pass	500.0	[<Confirmed>]	
3	226.6	7038	57858	SS21-HEX	227	0.40	Pass	500.0	[<Confirmed>]	

Sample 67: SSS13_SS20_SS11_SS21_SS02_SS19_HQZ13-1_C09.fsa

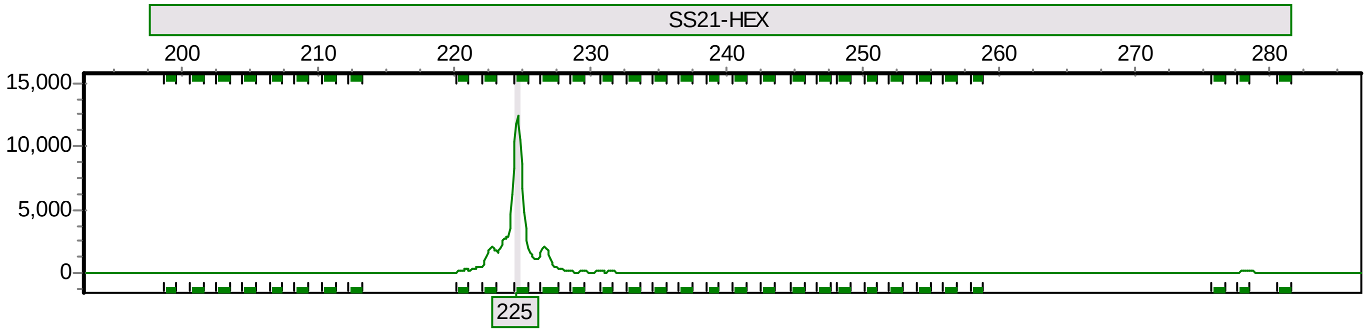

No	Size	Height	Area	Marker	Allele	Difference	Quality	Score	Allele Comments	Sample Comments
1	159.5	28221	179138	SS11-HEX	160	0.10	Pass	500.0	[<Confirmed>]	
2	224.7	12350	98842	SS21-HEX	225	0.30	Pass	500.0	[<Confirmed>]	

Sample 68: SSS13_SS20_SS11_SS21_SS02_SS19_HQZ13-2_J03.fsa

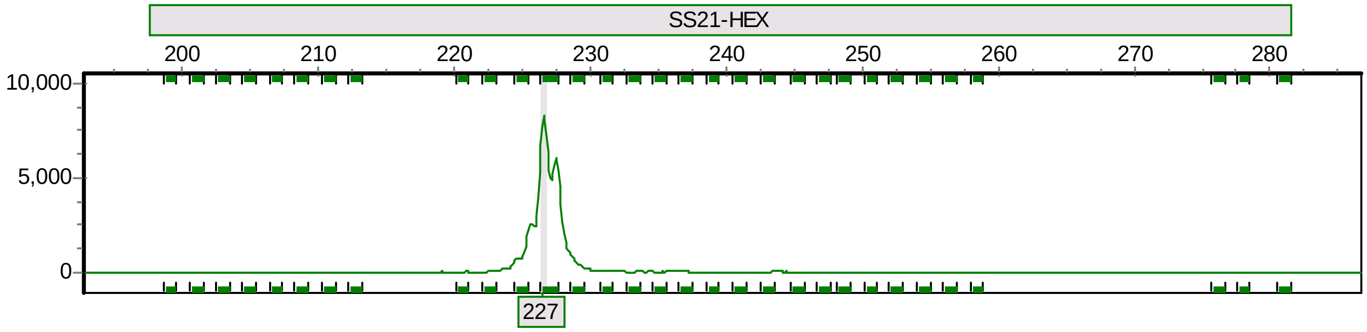

No	Size	Height	Area	Marker	Allele	Difference	Quality	Score	Allele Comments	Sample Comments
1	155.8	18011	113867	SS11-HEX	156	0.10	Pass	500.0	[<Confirmed>]	
2	226.6	8210	66686	SS21-HEX	227	0.40	Pass	500.0	[<Confirmed>]	

Sample 69: SSS13_SS20_SS11_SS21_SS02_SS19_HQZ14_H03.fsa

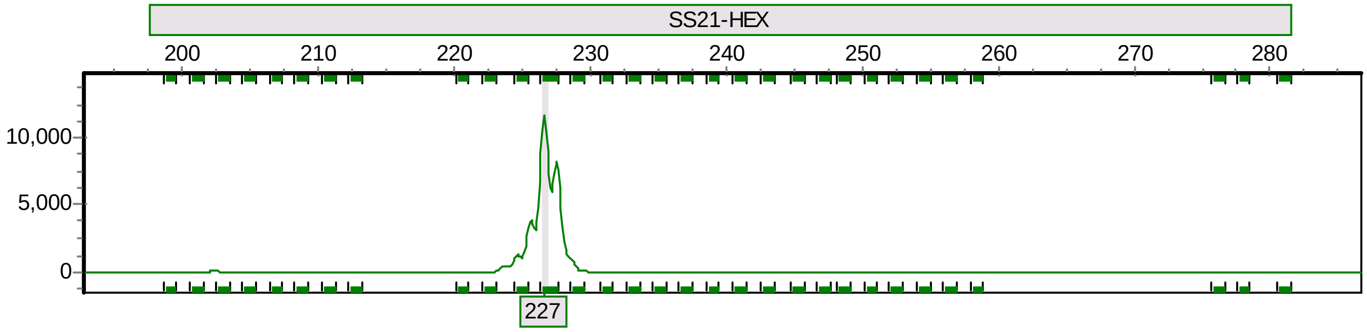

No	Size	Height	Area	Marker	Allele	Difference	Quality	Score	Allele Comments	Sample Comments
1	161.6	18899	121887	SS11-HEX	162	0.10	Pass	500.0	[<Confirmed>]	
2	226.7	11518	83678	SS21-HEX	227	0.30	Pass	500.0	[<Confirmed>]	

Sample 70: SSS13_SS20_SS11_SS21_SS02_SS19_HQZ15_B01.fsa Run date and time: 10/09/2021 - 22:22:10 -> 10/09/2021 - 22:49:12

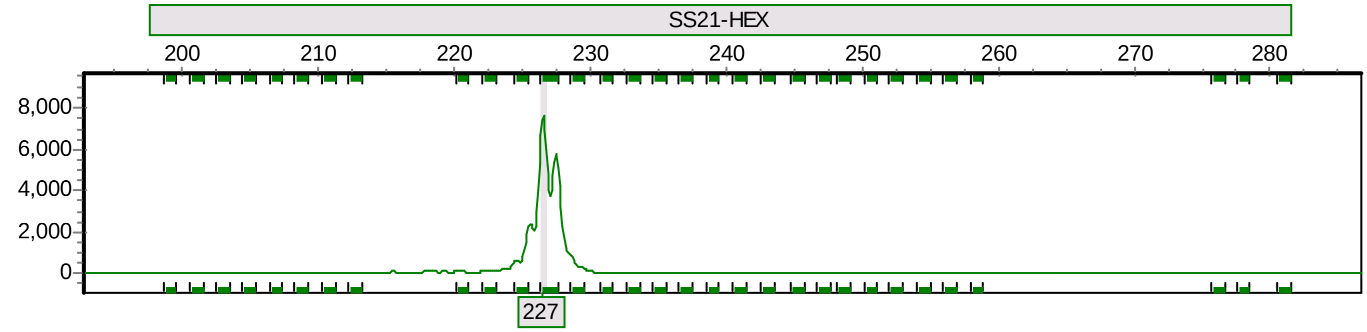

No	Size	Height	Area	Marker	Allele	Difference	Quality	Score	Allele Comments	Sample Comments
1	155.9	7291	46031	SS11-HEX	156	0.00	Pass	500.0	[<Confirmed>]	
2	165.4	5056	33376	SS11-HEX	166	0.00	Pass	500.0	[<Confirmed>]	
3	226.6	7589	55443	SS21-HEX	227	0.40	Pass	500.0	[<Confirmed>]	

Sample 71: SSS13_SS20_SS11_SS21_SS02_SS19_HQZ16_P01.fsa

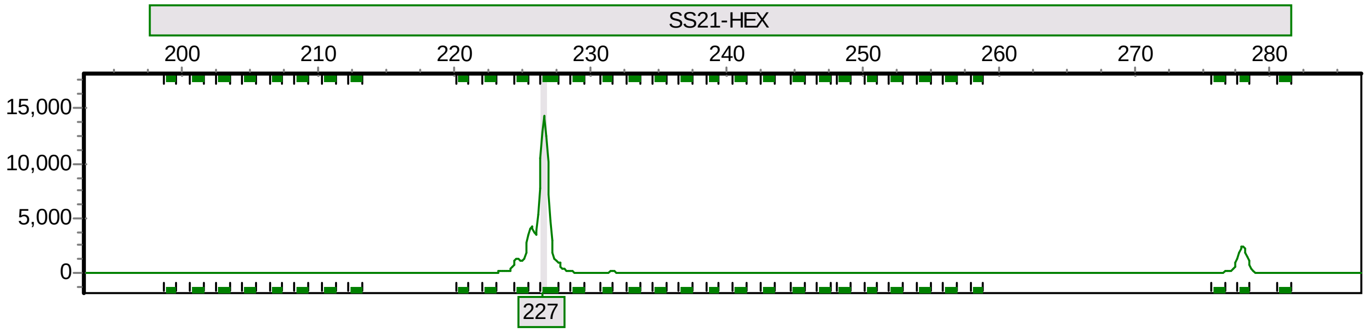

No	Size	Height	Area	Marker	Allele	Difference	Quality	Score	Allele Comments	Sample Comments
1	161.4	11173	69970	SS11-HEX	162	0.10	Pass	500.0	[<Confirmed>]	
2	226.6	14223	100609	SS21-HEX	227	0.40	Pass	500.0	[<Confirmed>]	

Sample 72: SSS13_SS20_SS11_SS21_SS02_SS19_HQZ17-1_O03.fsa

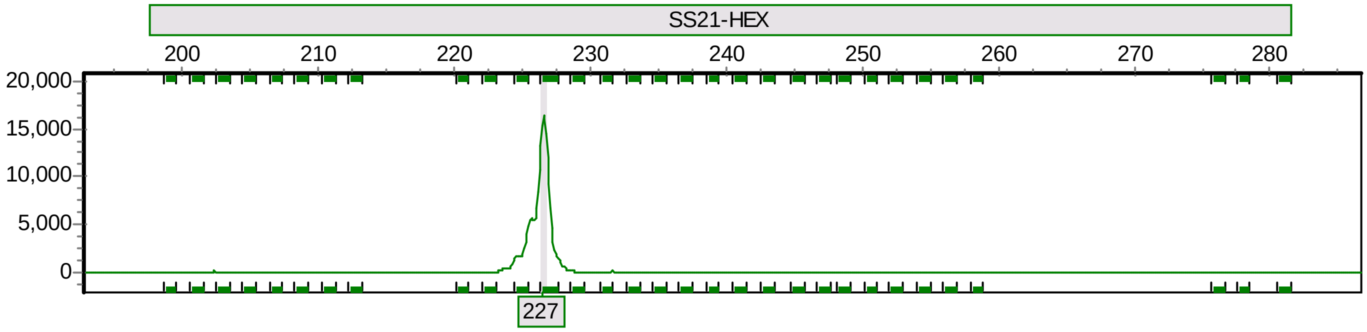

No	Size	Height	Area	Marker	Allele	Difference	Quality	Score	Allele Comments	Sample Comments
1	155.9	15860	98995	SS11-HEX	156	0.00	Pass	500.0	[<Confirmed>]	
2	159.6	12136	74637	SS11-HEX	160	0.00	Pass	500.0	[<Confirmed>]	
3	226.6	16275	133129	SS21-HEX	227	0.40	Pass	500.0	[<Confirmed>]	

Sample 73: SSS13_SS20_SS11_SS21_SS02_SS19_HQZ17-2_N03.fsa

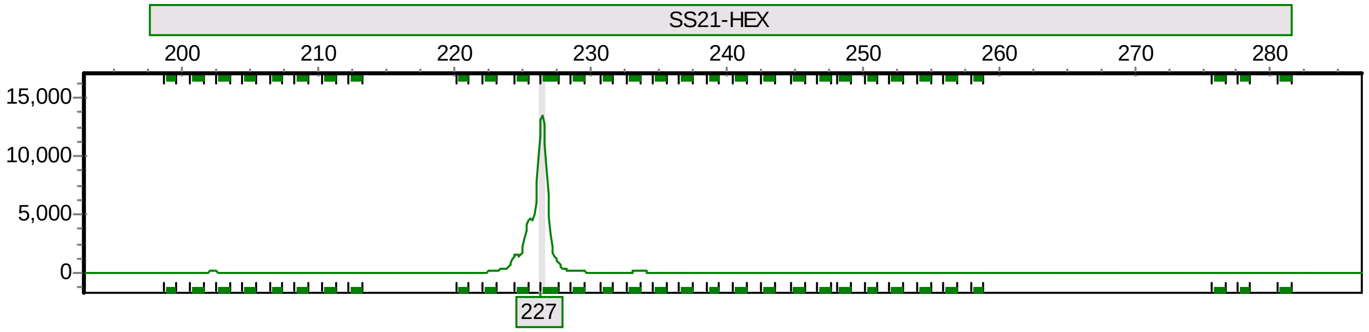

No	Size	Height	Area	Marker	Allele	Difference	Quality	Score	Allele Comments	Sample Comments
1	155.8	14584	91607	SS11-HEX	156	0.10	Pass	500.0	[<Confirmed>]	
2	159.5	10773	65200	SS11-HEX	160	0.10	Pass	500.0	[<Confirmed>]	
3	226.5	13368	109227	SS21-HEX	227	0.50	Pass	500.0	[<Confirmed>]	

Sample 74: SSS13_SS20_SS11_SS21_SS02_SS19_HQZ18_K09.fsa

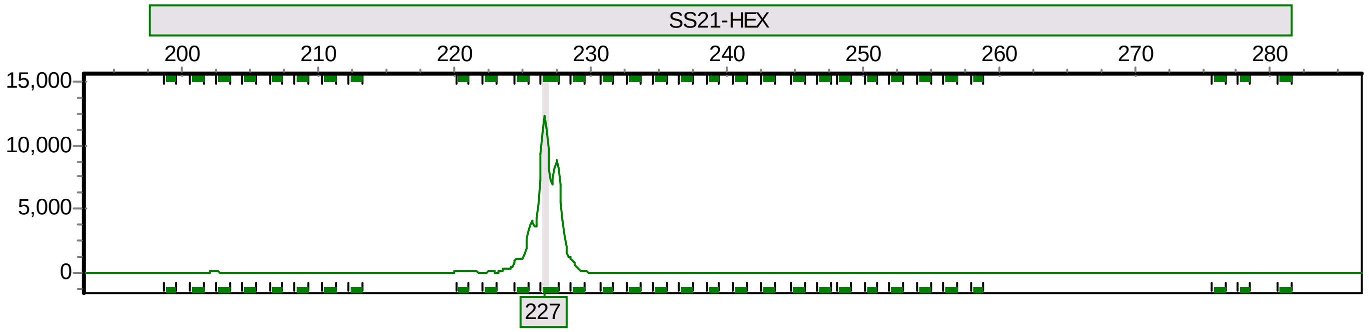

No	Size	Height	Area	Marker	Allele	Difference	Quality	Score	Allele Comments	Sample Comments
1	161.5	13668	91862	SS11-HEX	162	0.00	Pass	500.0	[<Confirmed>]	
2	165.4	10151	68880	SS11-HEX	166	0.00	Pass	500.0	[<Confirmed>]	
3	226.7	12228	98171	SS21-HEX	227	0.30	Pass	500.0	[<Confirmed>]	

Sample 75: SSS13_SS20_SS11_SS21_SS02_SS19_HQZ19_L03.fsa

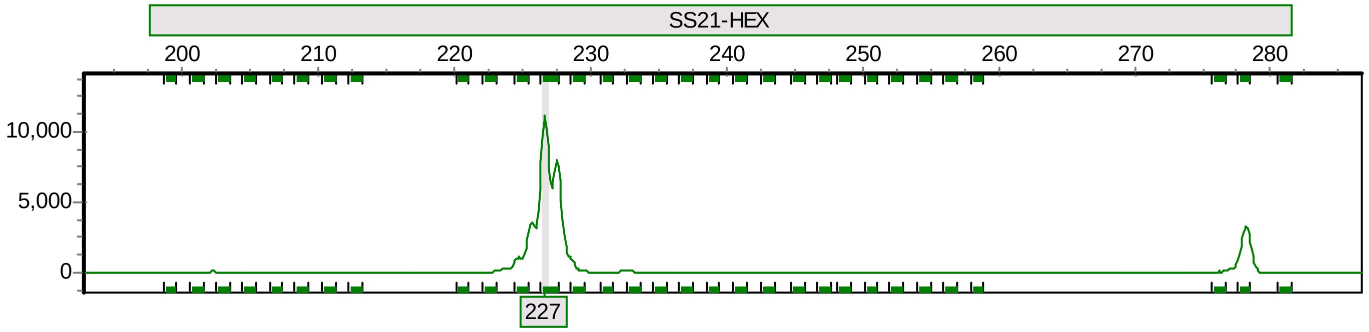

No	Size	Height	Area	Marker	Allele	Difference	Quality	Score	Allele Comments	Sample Comments
1	165.3	14005	91442	SS11-HEX	166	0.10	Pass	500.0	[<Confirmed>]	
2	226.7	11142	83127	SS21-HEX	227	0.30	Pass	500.0	[<Confirmed>]	

Sample 76: SSS13_SS20_SS11_SS21_SS02_SS19_HQZ21_B03.fsa

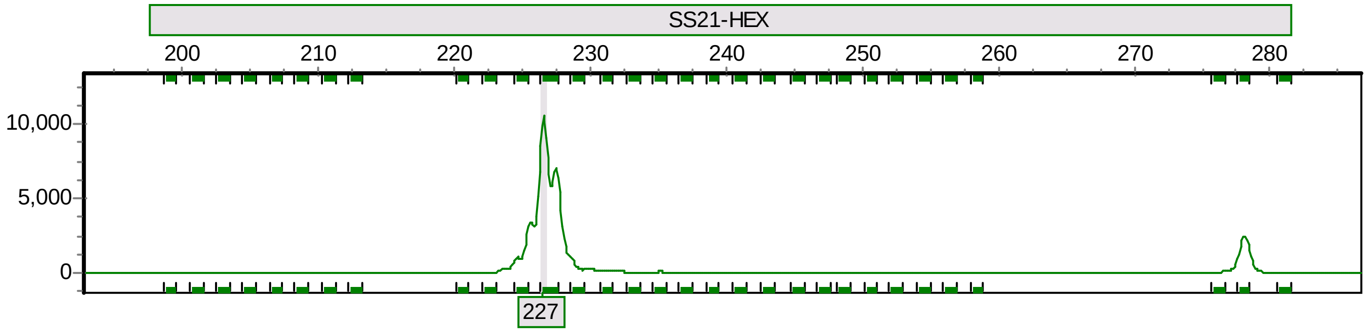

No	Size	Height	Area	Marker	Allele	Difference	Quality	Score	Allele Comments	Sample Comments
1	159.6	13545	84382	SS11-HEX	160	0.00	Pass	500.0	[<Confirmed>]	
2	226.6	10416	83255	SS21-HEX	227	0.40	Pass	500.0	[<Confirmed>]	

Sample 77: SSS13_SS20_SS11_SS21_SS02_SS19_HQZ22-1_M01.fsa

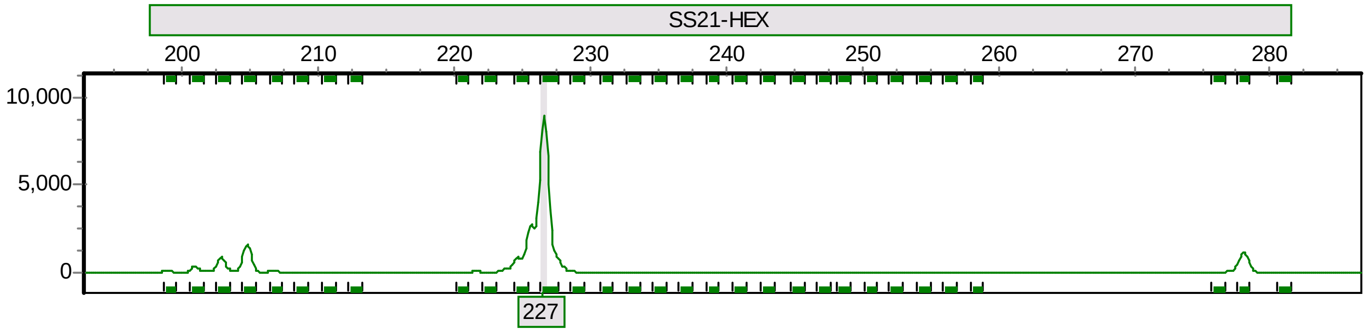

No	Size	Height	Area	Marker	Allele	Difference	Quality	Score	Allele Comments	Sample Comments
1	165.4	7486	49529	SS11-HEX	166	0.00	Pass	500.0	[<Confirmed>]	
2	226.6	8878	67853	SS21-HEX	227	0.40	Pass	500.0	[<Confirmed>]	

Sample 78: SSS13_SS20_SS11_SS21_SS02_SS19_HQZ22-2_O13.fsa

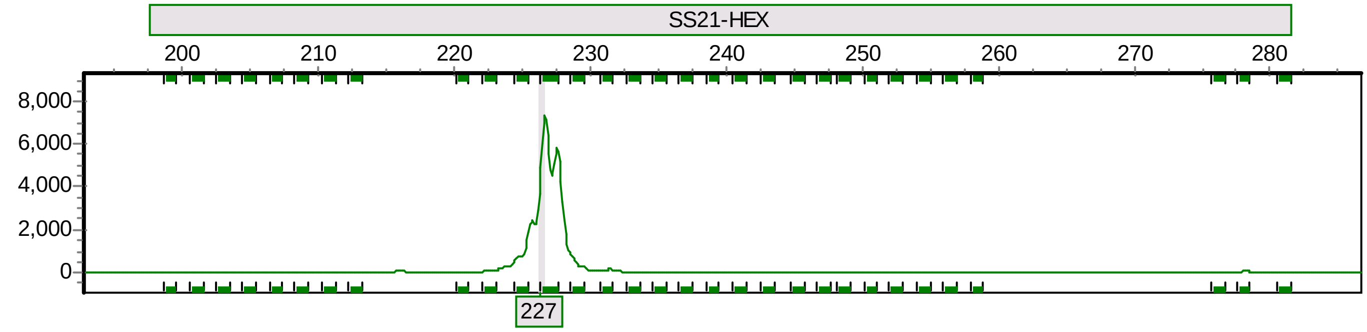

No	Size	Height	Area	Marker	Allele	Difference	Quality	Score	Allele Comments	Sample Comments
1	165.4	11194	76270	SS11-HEX	166	0.00	Pass	500.0	[<Confirmed>]	
2	226.5	6014	60034	SS21-HEX	227	0.50	Pass	500.0	[<Confirmed>]	
3	227.6	5793	43454	SS21-HEX	227	0.60	Pass	500.0	[<Deleted>]	

Sample 79: SSS13_SS20_SS11_SS21_SS02_SS19_HQZ23_A15.fsa

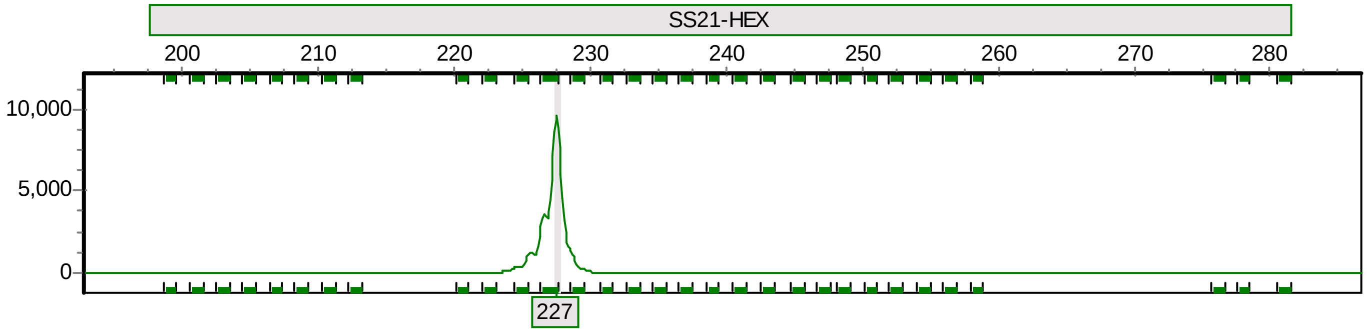

No	Size	Height	Area	Marker	Allele	Difference	Quality	Score	Allele Comments	Sample Comments
1	146.0	12173	89346	SS11-HEX	146	0.20	Pass	500.0	[<Confirmed>]	
2	165.5	5432	36755	SS11-HEX	166	0.10	Pass	500.0	[<Confirmed>]	
3	227.6	9532	78290	SS21-HEX	227	0.60	Pass	500.0	[<Confirmed>]	

Sample 80: SSS13_SS20_SS11_SS21_SS02_SS19_HQZ24_E11.fsa

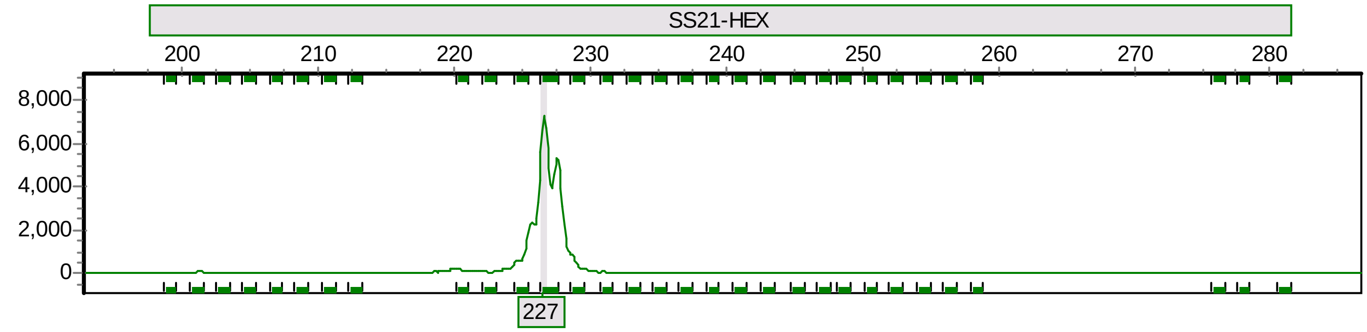

No	Size	Height	Area	Marker	Allele	Difference	Quality	Score	Allele Comments	Sample Comments
1	161.6	9669	66940	SS11-HEX	162	0.10	Pass	500.0	[<Confirmed>]	
2	165.5	6790	46218	SS11-HEX	166	0.10	Pass	500.0	[<Confirmed>]	
3	226.6	7241	55541	SS21-HEX	227	0.40	Pass	500.0	[<Confirmed>]	

Sample 81: SSS13_SS20_SS11_SS21_SS02_SS19_HQZ25_N01.fsa

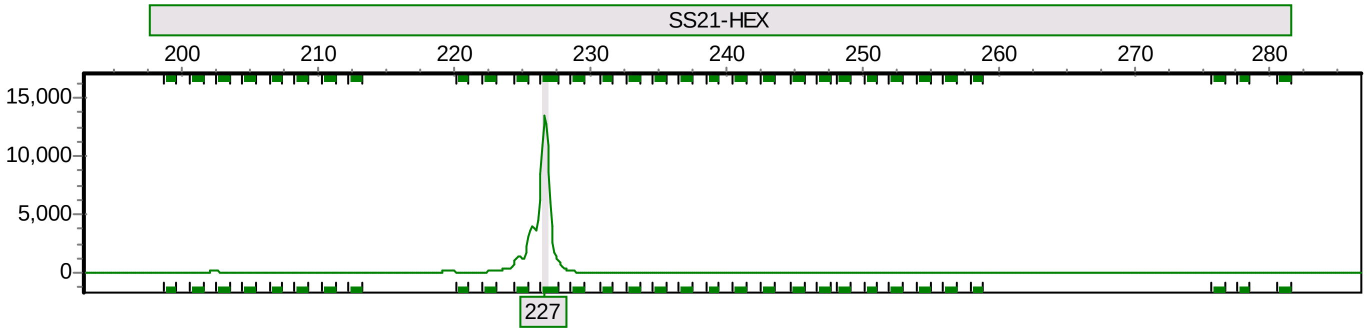

No	Size	Height	Area	Marker	Allele	Difference	Quality	Score	Allele Comments	Sample Comments
1	165.3	13535	88261	SS11-HEX	166	0.10	Pass	500.0	[<Confirmed>]	
2	226.7	13336	99201	SS21-HEX	227	0.30	Pass	500.0	[<Confirmed>]	

Sample 82: SSS13_SS20_SS11_SS21_SS02_SS19_HQZ26_E03.fsa

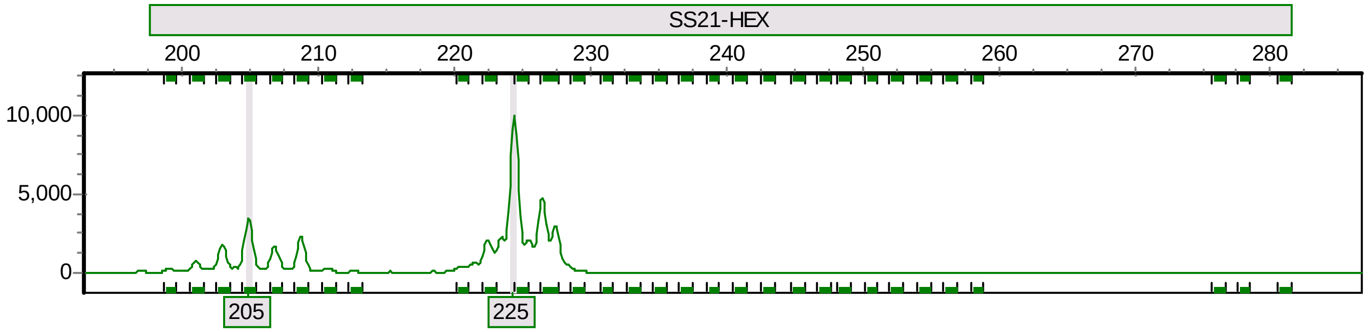

No	Size	Height	Area	Marker	Allele	Difference	Quality	Score	Allele Comments	Sample Comments
1	165.4	11883	76942	SS11-HEX	166	0.00	Pass	500.0	[<Confirmed>]	
2	167.4	7697	49996	SS11-HEX	168	0.00	Pass	500.0	[<Confirmed>]	
3	204.9	3460	23627	SS21-HEX	205	0.10	Pass	500.0	[<Confirmed>]	
4	224.4	9886	73101	SS21-HEX	225	1.00	Pass	500.0	[<Confirmed><Edited>]	
5	226.5	4734	34600	SS21-HEX	227	0.50	Pass	500.0	[<Deleted>]	

Sample 83: SSS13_SS20_SS11_SS21_SS02_SS19_HQZ27_G09.fsa

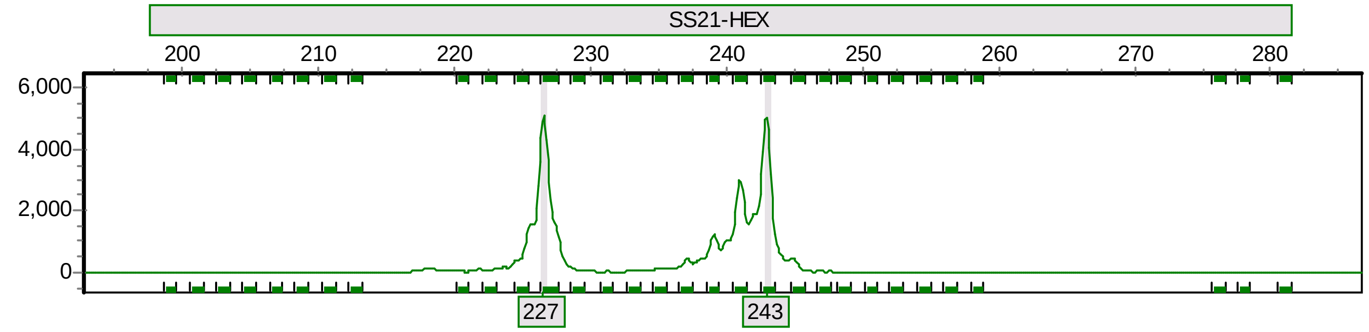

No	Size	Height	Area	Marker	Allele	Difference	Quality	Score	Allele Comments	Sample Comments
1	139.4	37240	308421	SS11-HEX	140	0.00	Pass	500.0	[<SAT (Repaired)><Confirmed>]	
2	226.6	5055	40700	SS21-HEX	227	0.40	Pass	500.0	[<Confirmed>]	
3	243.0	5015	42289	SS21-HEX	243	0.10	Pass	500.0	[<Confirmed>]	

Sample 84: SSS13_SS20_SS11_SS21_SS02_SS19_HQZ28_B05.fsa

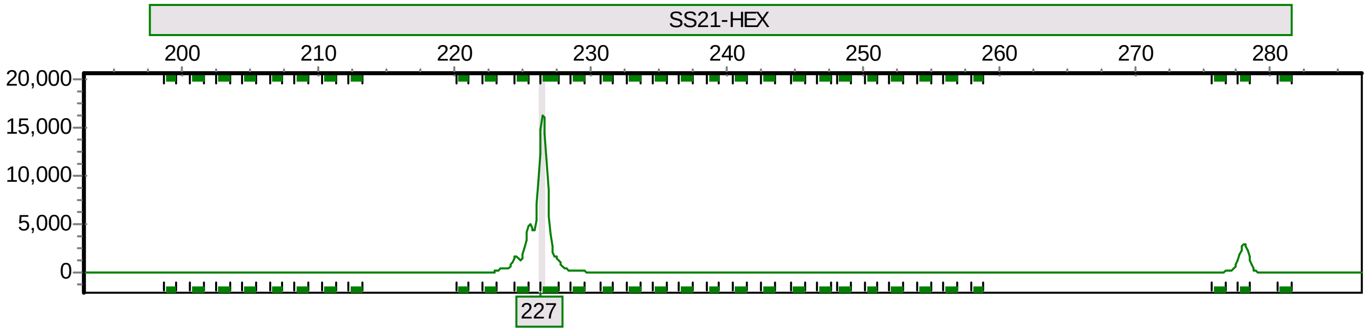

No	Size	Height	Area	Marker	Allele	Difference	Quality	Score	Allele Comments	Sample Comments
1	145.9	27356	196309	SS11-HEX	146	0.30	Pass	500.0	[<Confirmed>]	
2	225.6	4955	35751	SS21-HEX	225	0.60	Pass	282.3	[<Deleted>]	
3	226.5	16200	120616	SS21-HEX	227	0.50	Pass	500.0	[<Confirmed>]	

Sample 85: SSS13_SS20_SS11_SS21_SS02_SS19_HQZ29_C13.fsa

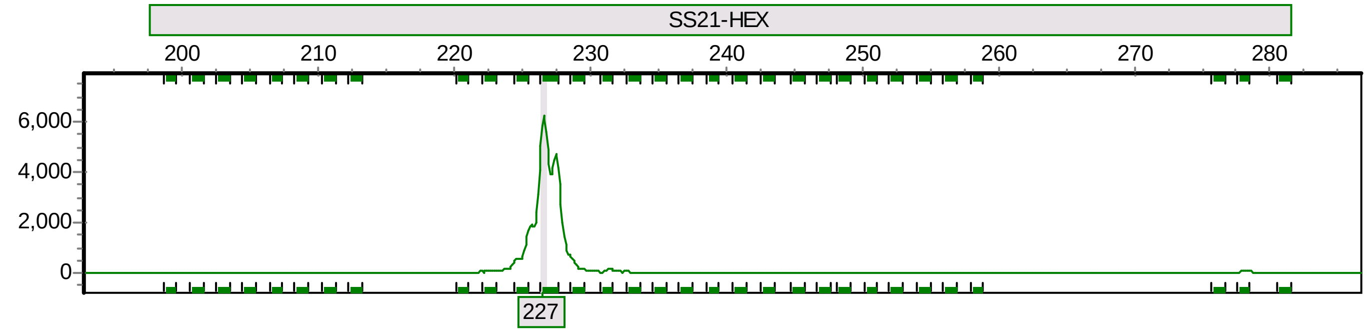

No	Size	Height	Area	Marker	Allele	Difference	Quality	Score	Allele Comments	Sample Comments
1	165.5	7699	53778	SS11-HEX	166	0.10	Pass	500.0	[<Confirmed>]	
2	226.6	6207	51358	SS21-HEX	227	0.40	Pass	500.0	[<Confirmed>]	

Sample 86: SSS13_SS20_SS11_SS21_SS02_SS19_HQZ2_K03.fsa

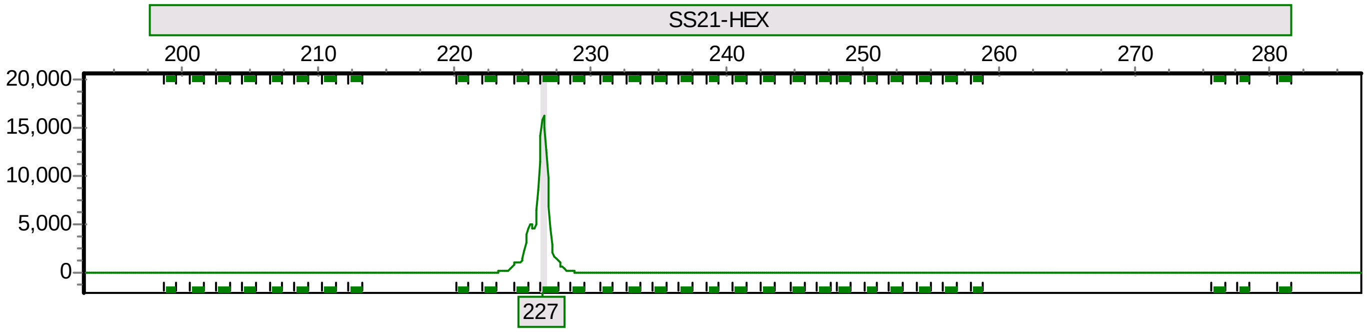

No	Size	Height	Area	Marker	Allele	Difference	Quality	Score	Allele Comments	Sample Comments
1	225.6	5043	34917	SS21-HEX	225	0.60	Pass	451.9	[<Deleted>]	
2	226.6	16243	122945	SS21-HEX	227	0.40	Pass	500.0	[<Confirmed>]	

Sample 87: SSS13_SS20_SS11_SS21_SS02_SS19_HQZ30_G15.fsa

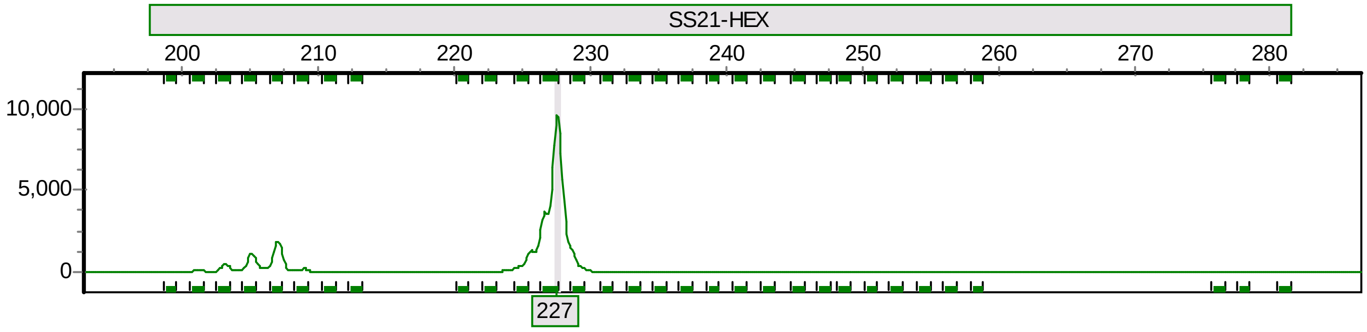

No	Size	Height	Area	Marker	Allele	Difference	Quality	Score	Allele Comments	Sample Comments
1	159.6	7800	50564	SS11-HEX	160	0.00	Pass	500.0	[<Confirmed>]	
2	165.4	5256	37507	SS11-HEX	166	0.00	Pass	500.0	[<Confirmed>]	
3	227.6	9534	79699	SS21-HEX	227	0.60	Pass	500.0	[<Confirmed>]	

Allele Report

Sample 88: SSS13_SS20_SS11_SS21_SS02_SS19_HQZ31_K13.fsa

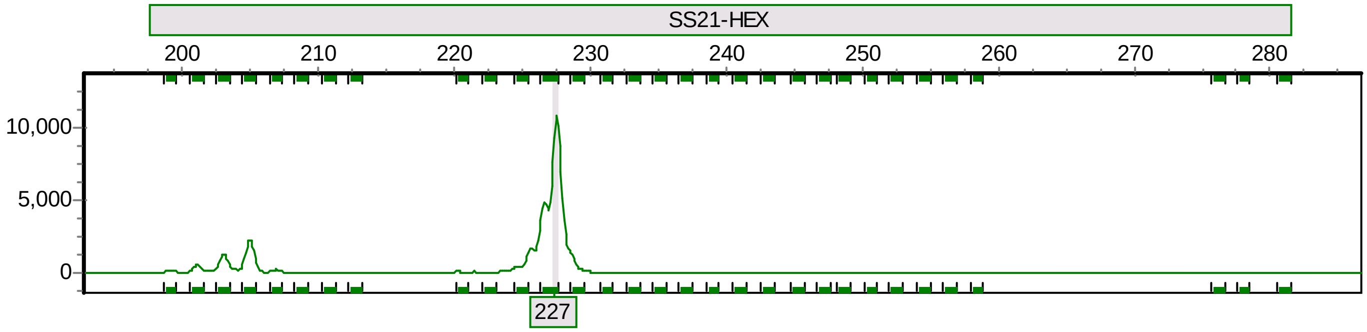

No	Size	Height	Area	Marker	Allele	Difference	Quality	Score	Allele Comments	Sample Comments
1	165.4	8887	61315	SS11-HEX	166	0.00	Pass	500.0	[<Confirmed>]	
2	167.3	5965	40761	SS11-HEX	168	0.10	Pass	500.0	[<Confirmed>]	
3	227.5	10487	82054	SS21-HEX	227	0.50	Pass	500.0	[<Confirmed>]	

Sample 89: SSS13_SS20_SS11_SS21_SS02_SS19_HQZ32_J01.fsa

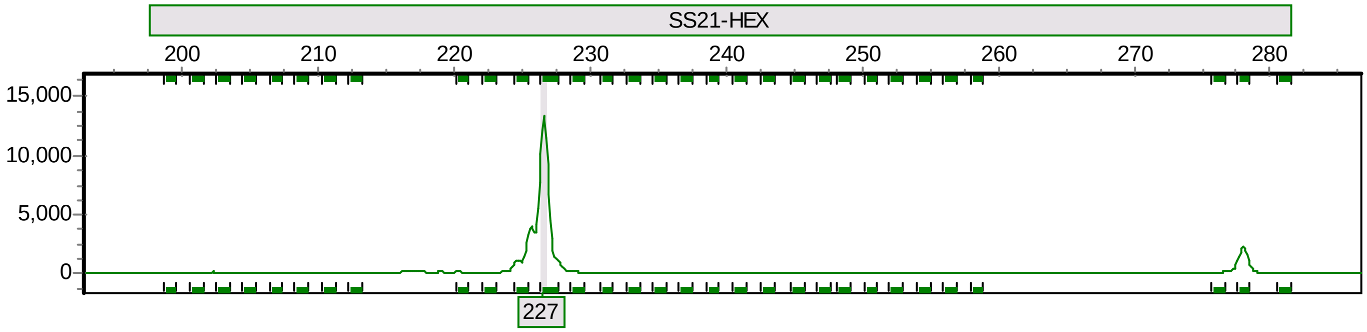

No	Size	Height	Area	Marker	Allele	Difference	Quality	Score	Allele Comments	Sample Comments
1	161.4	9192	60200	SS11-HEX	162	0.10	Pass	500.0	[<Confirmed>]	
2	165.3	6636	43263	SS11-HEX	166	0.10	Pass	500.0	[<Confirmed>]	
3	226.6	13282	95327	SS21-HEX	227	0.40	Pass	500.0	[<Confirmed>]	

Sample 90: SSS13_SS20_SS11_SS21_SS02_SS19_HQZ33_I03.fsa

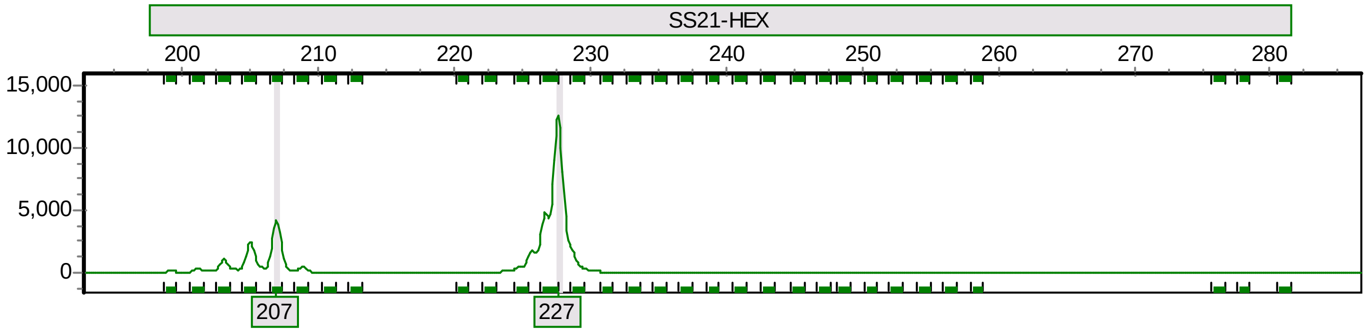

No	Size	Height	Area	Marker	Allele	Difference	Quality	Score	Allele Comments	Sample Comments
1	165.4	13375	89973	SS11-HEX	166	0.00	Pass	500.0	[<Confirmed>]	
2	207.0	4154	30692	SS21-HEX	207	0.00	Pass	500.0	[<Confirmed>]	
3	227.7	12483	97137	SS21-HEX	227	0.70	Pass	500.0	[<Confirmed>]	

SoftGenetics

GeneMarker V3.0.0

Allele Report

Sample 91: SSS13_SS20_SS11_SS21_SS02_SS19_HQZ34_M03.fsa

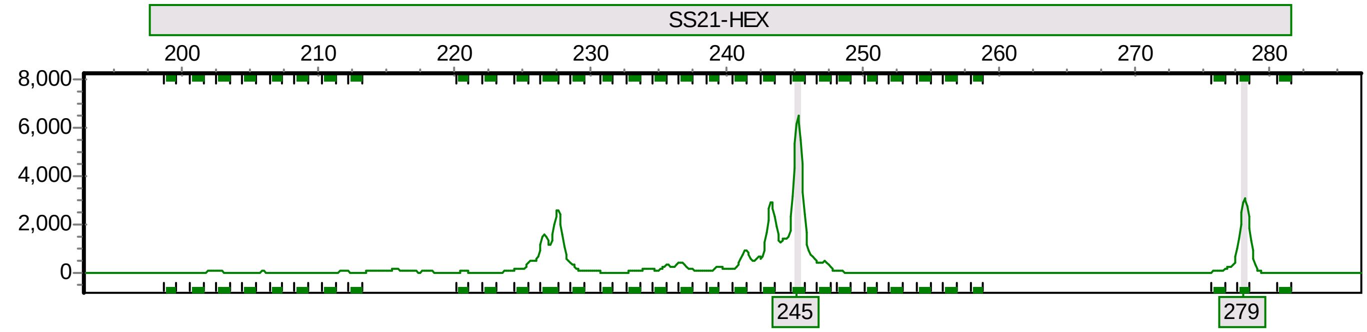

No	Size	Height	Area	Marker	Allele	Difference	Quality	Score	Allele Comments	Sample Comments
1	159.5	14699	90287	SS11-HEX	160	0.10	Pass	500.0	[<Confirmed>]	
2	227.6	2594	20372	SS21-HEX	227	0.60	Undetermined	313.7	[<Deleted>]	
3	245.3	6466	51602	SS21-HEX	245	0.00	Pass	500.0	[<Confirmed>]	
4	278.1	3065	25726	SS21-HEX	279	0.00	Pass	421.9	[<Confirmed>]	

Sample 92: SSS13_SS20_SS11_SS21_SS02_SS19_HQZ35_A01.fsa

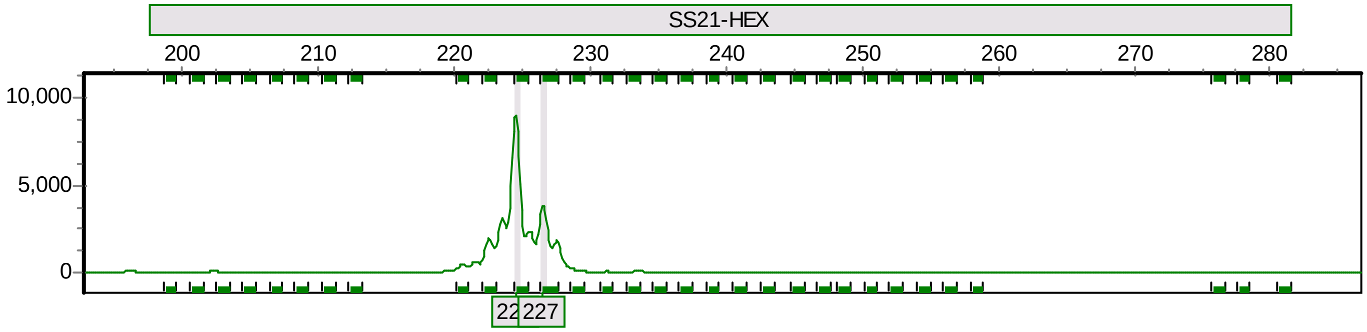

No	Size	Height	Area	Marker	Allele	Difference	Quality	Score	Allele Comments	Sample Comments
1	145.7	26850	190670	SS11-HEX	146	0.50	Pass	500.0	[<Confirmed>]	
2	224.6	8922	67090	SS21-HEX	225	0.40	Pass	500.0	[<Confirmed>]	
3	226.6	3844	28566	SS21-HEX	227	0.40	Pass	500.0	[<Confirmed>]	

Sample 93: SSS13_SS20_SS11_SS21_SS02_SS19_HQZ36_E15.fsa

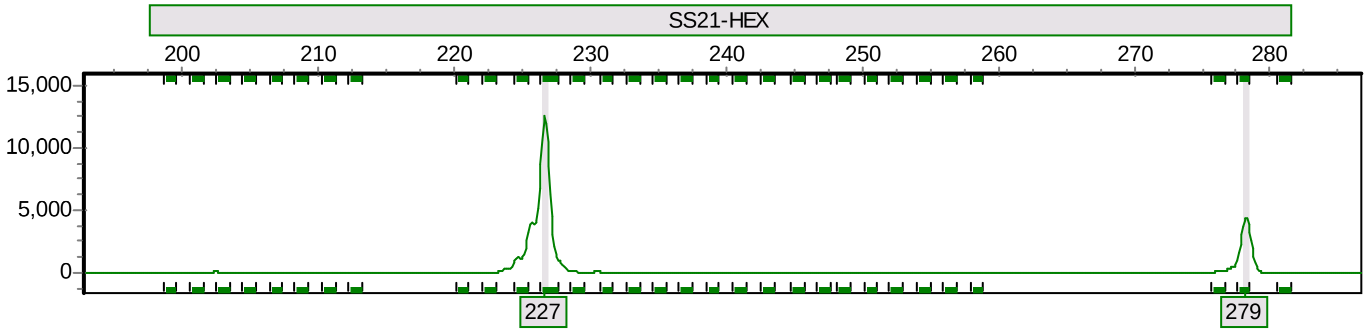

No	Size	Height	Area	Marker	Allele	Difference	Quality	Score	Allele Comments	Sample Comments
1	159.6	13403	86288	SS11-HEX	160	0.00	Pass	500.0	[<Confirmed>]	
2	226.7	12510	100628	SS21-HEX	227	0.30	Pass	500.0	[<Confirmed>]	
3	278.2	4448	38258	SS21-HEX	279	0.10	Pass	500.0	[<Confirmed>]	

Sample 94: SSS13_SS20_SS11_SS21_SS02_SS19_HQZ37_F03.fsa

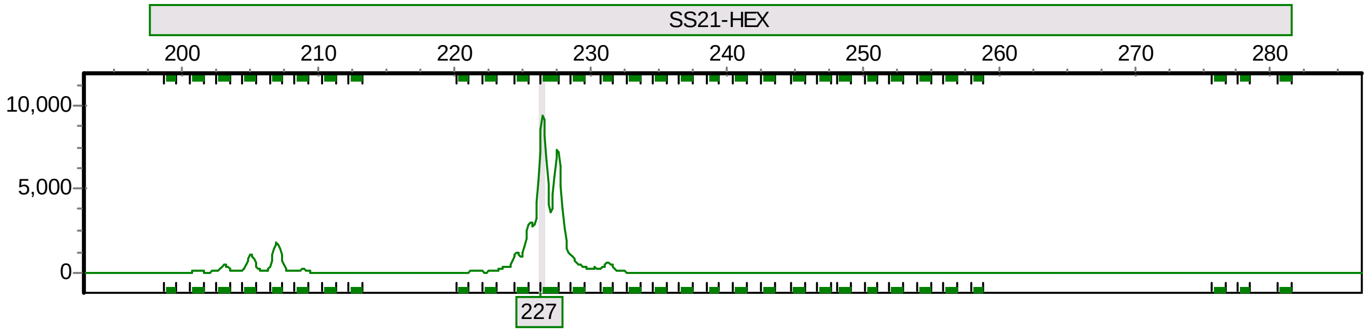

No	Size	Height	Area	Marker	Allele	Difference	Quality	Score	Allele Comments	Sample Comments
1	145.9	5536	39324	SS11-HEX	146	0.30	Pass	500.0	[<Confirmed><Edited>]	
2	163.4	6360	43250	SS11-HEX	164	0.00	Pass	500.0	[<Confirmed>]	
3	165.3	6302	40855	SS11-HEX	166	0.10	Pass	500.0	[<Deleted>]	
4	169.1	4939	33436	SS11-HEX	170	0.20	Undetermined	500.0	[<Deleted>]	
5	226.5	9311	67739	SS21-HEX	227	0.50	Pass	500.0	[<Confirmed>]	
6	227.6	7327	56043	SS21-HEX	227	0.60	Pass	500.0	[<Deleted>]	

Sample 95: SSS13_SS20_SS11_SS21_SS02_SS19_HQZ38_L01.fsa

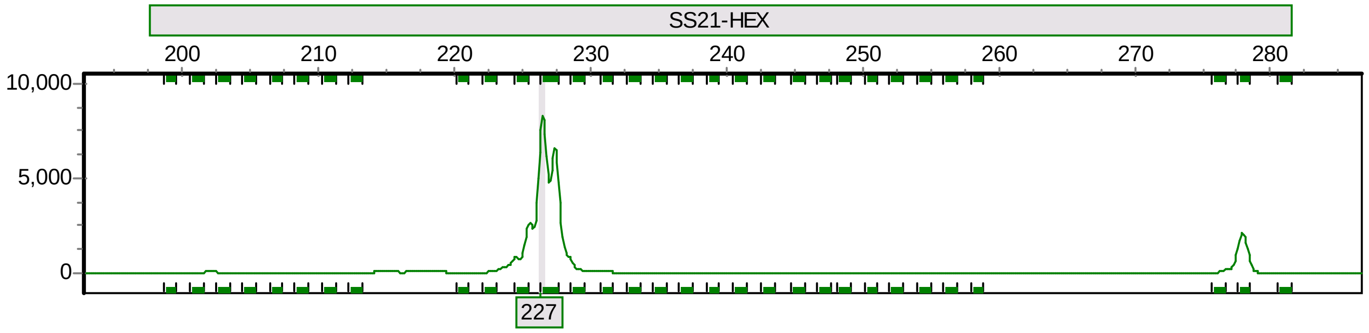

No	Size	Height	Area	Marker	Allele	Difference	Quality	Score	Allele Comments	Sample Comments
1	155.8	13509	84772	SS11-HEX	156	0.10	Pass	500.0	[<Confirmed>]	
2	226.5	8223	60099	SS21-HEX	227	0.50	Pass	500.0	[<Confirmed>]	

Sample 96: SSS13_SS20_SS11_SS21_SS02_SS19_HQZ39_M15.fsa

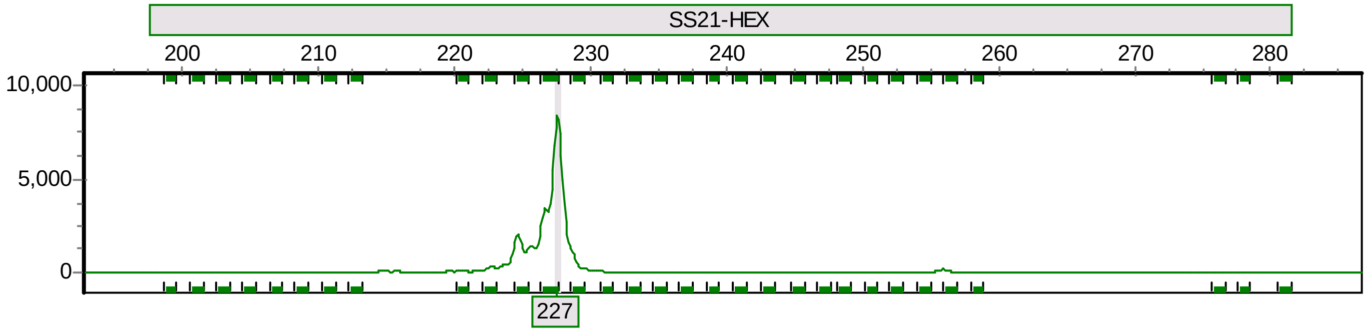

No	Size	Height	Area	Marker	Allele	Difference	Quality	Score	Allele Comments	Sample Comments
1	139.4	32648	276860	SS11-HEX	140	0.00	Pass	500.0	[<Confirmed>]	
2	227.6	8376	65902	SS21-HEX	227	0.60	Pass	500.0	[<Confirmed>]	

Sample 97: SSS13_SS20_SS11_SS21_SS02_SS19_HQZ7_C03.fsa

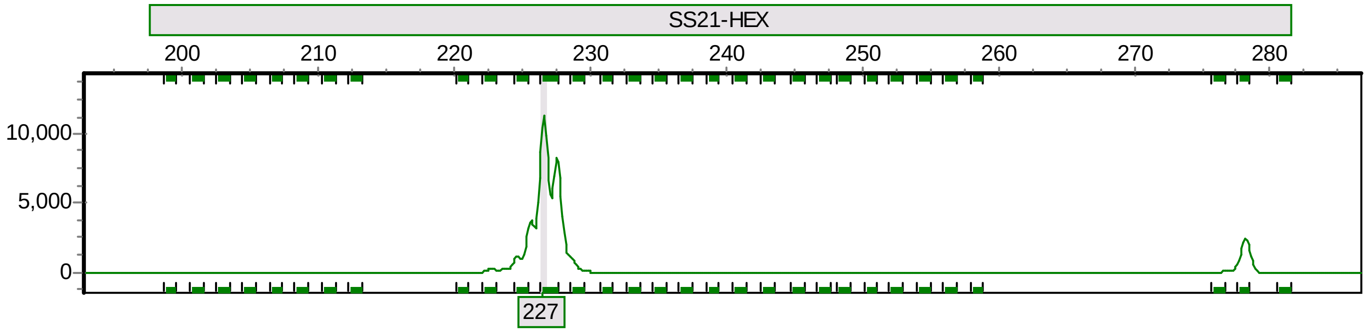

No	Size	Height	Area	Marker	Allele	Difference	Quality	Score	Allele Comments	Sample Comments
1	159.6	17777	106699	SS11-HEX	160	0.00	Pass	500.0	[<Confirmed>]	
2	161.5	12244	79044	SS11-HEX	162	0.00	Pass	500.0	[<Confirmed>]	
3	226.6	11168	82888	SS21-HEX	227	0.40	Pass	500.0	[<Confirmed>]	

Sample 98: SSS13_SS20_SS11_SS21_SS02_SS19_HQZ9_K11.fsa

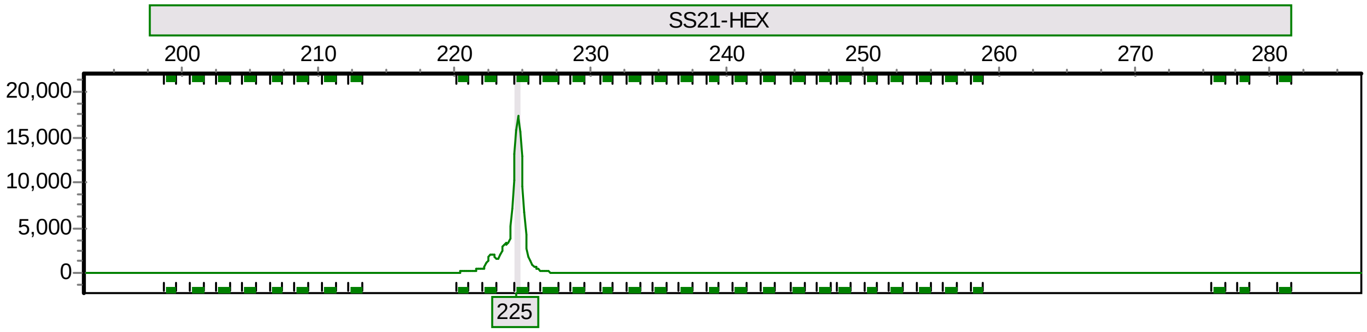

No	Size	Height	Area	Marker	Allele	Difference	Quality	Score	Allele Comments	Sample Comments
1	165.5	13207	89840	SS11-HEX	166	0.10	Pass	500.0	[<Confirmed>]	
2	171.4	9683	66977	SS11-HEX	172	0.10	Pass	500.0	[<Confirmed>]	
3	224.7	17297	134641	SS21-HEX	225	0.30	Pass	500.0	[<Confirmed>]	

Sample 99: SSS13_SS20_SS11_SS21_SS02_SS19_HRS24_I17.fsa

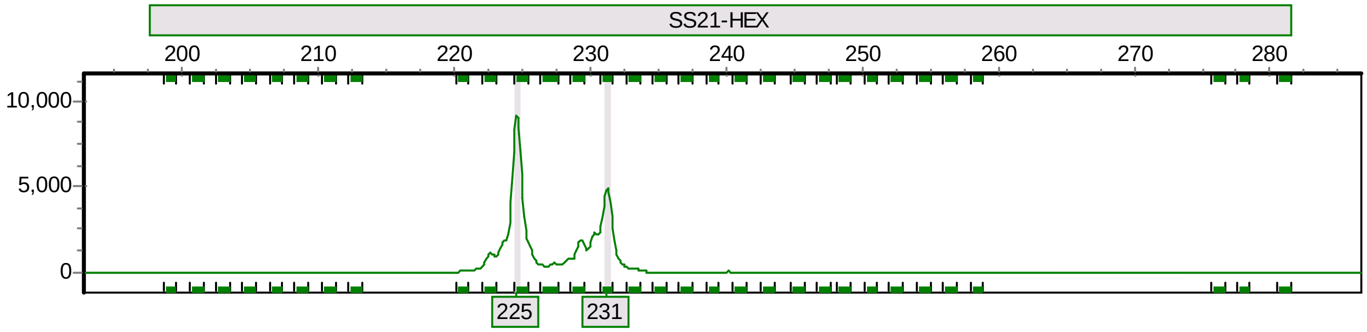

No	Size	Height	Area	Marker	Allele	Difference	Quality	Score	Allele Comments	Sample Comments
1	154.1	22084	146743	SS11-HEX	154	0.10	Pass	500.0	[<Confirmed>]	
2	171.2	18143	129890	SS11-HEX	172	0.10	Pass	500.0	[<Confirmed>]	
3	224.6	9088	77196	SS21-HEX	225	0.40	Pass	500.0	[<Confirmed>]	
4	231.3	4939	41609	SS21-HEX	231	0.00	Pass	500.0	[<Confirmed>]	

Sample 100: SSS13_SS20_SS11_SS21_SS02_SS19_HRS26_H09.fsa

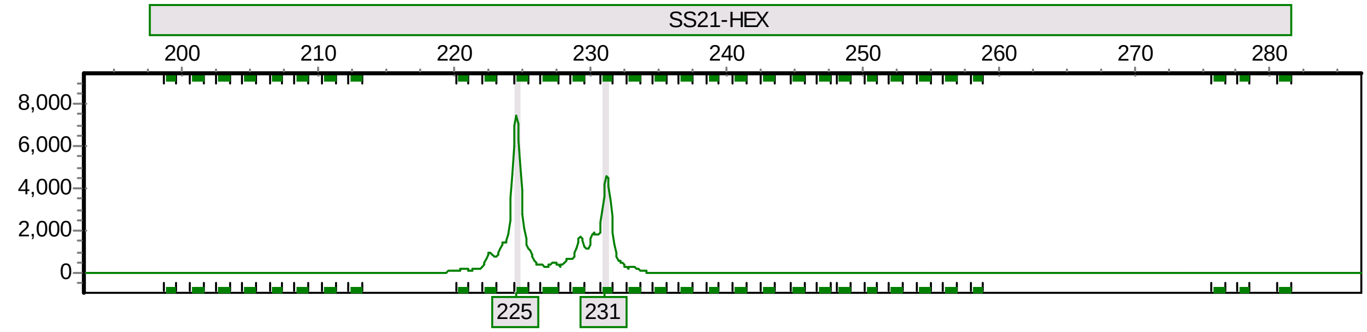

No	Size	Height	Area	Marker	Allele	Difference	Quality	Score	Allele Comments	Sample Comments
1	154.1	20196	127789	SS11-HEX	154	0.10	Pass	500.0	[<Confirmed>]	
2	171.3	14759	102461	SS11-HEX	172	0.00	Pass	500.0	[<Confirmed>]	
3	224.6	7410	58097	SS21-HEX	225	0.40	Pass	500.0	[<Confirmed>]	
4	231.2	4561	36558	SS21-HEX	231	0.10	Pass	500.0	[<Confirmed>]	

Sample 101: SSS13_SS20_SS11_SS21_SS02_SS19_HRS28_D13.fsa

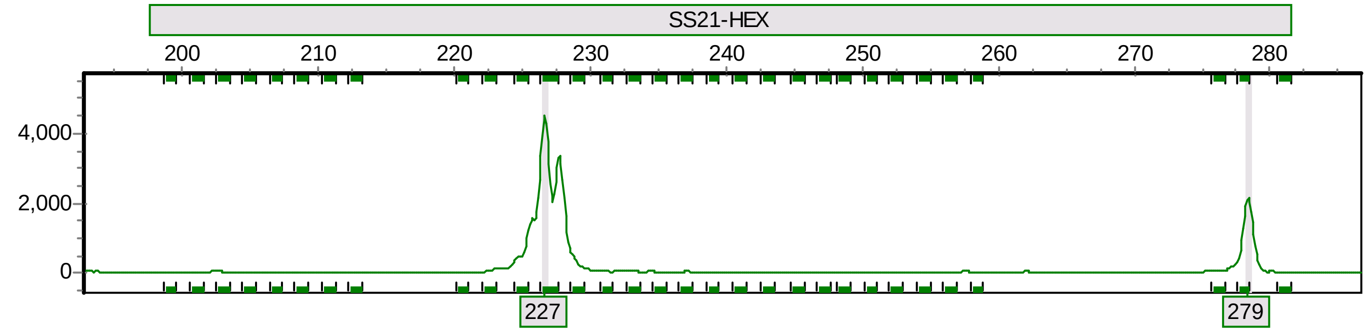

No	Size	Height	Area	Marker	Allele	Difference	Quality	Score	Allele Comments	Sample Comments
1	226.7	4509	36531	SS21-HEX	227	0.30	Pass	500.0	[<Confirmed>]	
2	278.4	2139	18482	SS21-HEX	279	0.30	Pass	235.0	[<Confirmed>]	

Sample 102: SSS13_SS20_SS11_SS21_SS02_SS19_HRS29_J05.fsa

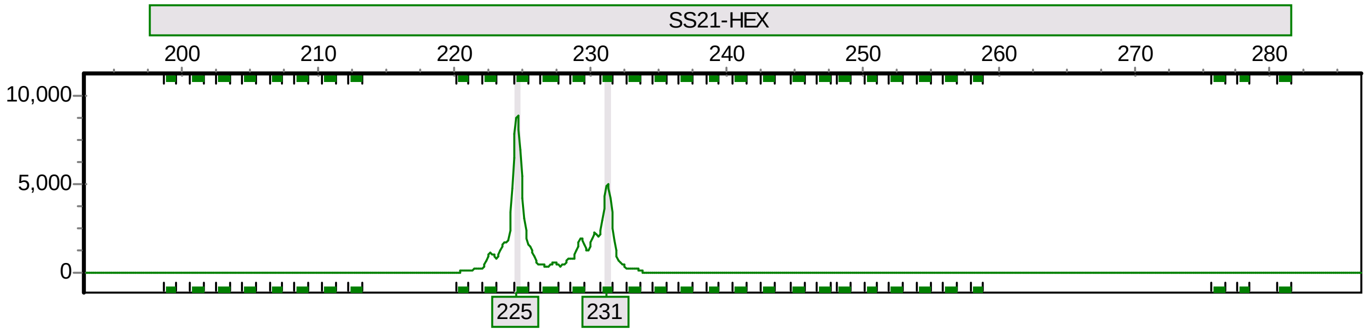

No	Size	Height	Area	Marker	Allele	Difference	Quality	Score	Allele Comments	Sample Comments
1	171.4	28097	194897	SS11-HEX	172	0.10	Pass	500.0	[<Confirmed>]	
2	224.7	8815	69930	SS21-HEX	225	0.30	Pass	500.0	[<Confirmed>]	
3	231.3	5026	41211	SS21-HEX	231	0.00	Pass	500.0	[<Confirmed>]	

Sample 103: SSS13_SS20_SS11_SS21_SS02_SS19_HRS30_P05.fsa

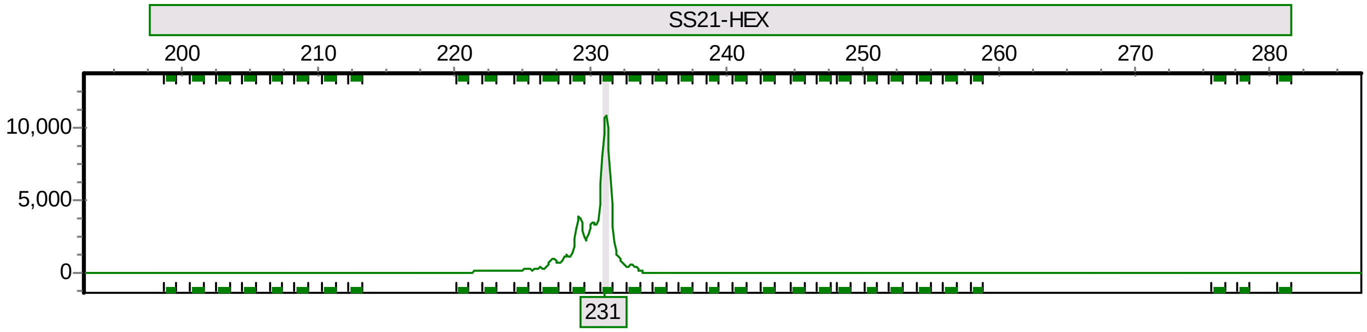

No	Size	Height	Area	Marker	Allele	Difference	Quality	Score	Allele Comments	Sample Comments
1	154.1	22824	145364	SS11-HEX	154	0.10	Pass	500.0	[<Confirmed>]	
2	171.2	18810	125906	SS11-HEX	172	0.10	Pass	500.0	[<Confirmed>]	
3	231.2	10788	87522	SS21-HEX	231	0.10	Pass	500.0	[<Confirmed>]	

Sample 104: SSS13_SS20_SS11_SS21_SS02_SS19_HRS31_J11.fsa

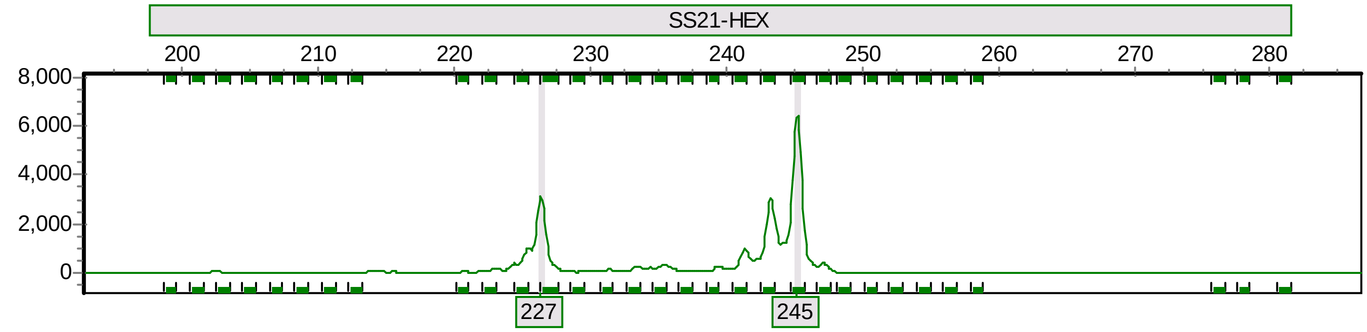

No	Size	Height	Area	Marker	Allele	Difference	Quality	Score	Allele Comments	Sample Comments
1	161.6	10610	70868	SS11-HEX	162	0.10	Pass	500.0	[<Confirmed>]	
2	226.4	3106	24597	SS21-HEX	227	0.60	Pass	472.8	[<Confirmed>]	
3	245.3	6382	51059	SS21-HEX	245	0.00	Pass	500.0	[<Confirmed>]	

Sample 105: SSS13_SS20_SS11_SS21_SS02_SS19_HRS33_H07.fsa

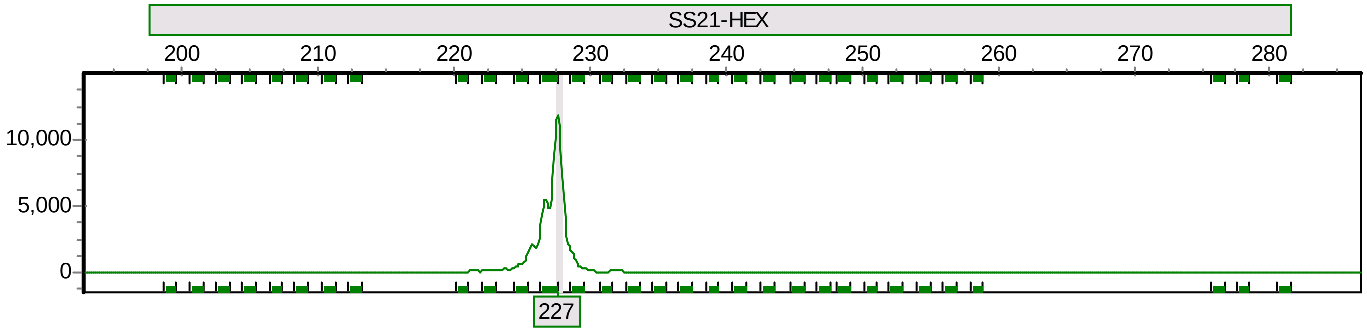

No	Size	Height	Area	Marker	Allele	Difference	Quality	Score	Allele Comments	Sample Comments
1	161.7	9352	63846	SS11-HEX	162	0.20	Pass	500.0	[<Confirmed>]	
2	165.6	7038	47871	SS11-HEX	166	0.20	Pass	500.0	[<Confirmed>]	
3	227.7	11676	90820	SS21-HEX	227	0.70	Pass	500.0	[<Confirmed>]	

Allele Report

Sample 106: SSS13_SS20_SS11_SS21_SS02_SS19_HRS34_L09.fsa

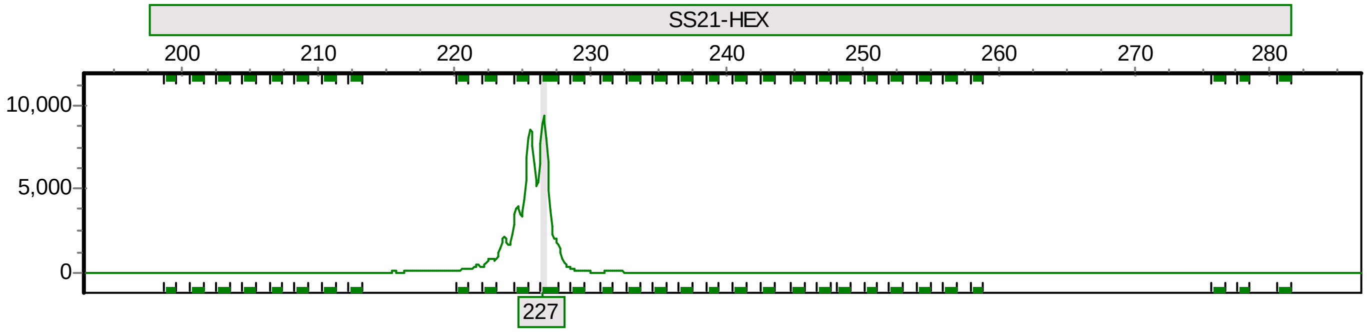

No	Size	Height	Area	Marker	Allele	Difference	Quality	Score	Allele Comments	Sample Comments
1	163.4	17444	116231	SS11-HEX	164	0.00	Pass	500.0	[<Confirmed>]	
2	225.6	8571	60990	SS21-HEX	225	0.60	Pass	500.0	[<Deleted>]	
3	226.6	9335	72698	SS21-HEX	227	0.40	Pass	500.0	[<Confirmed>]	

Sample 107: SSS13_SS20_SS11_SS21_SS02_SS19_HRS35_P09.fsa

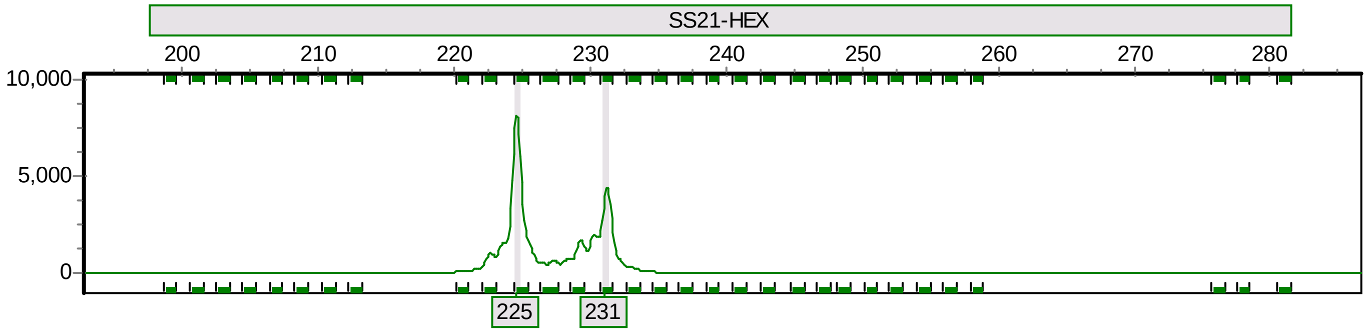

No	Size	Height	Area	Marker	Allele	Difference	Quality	Score	Allele Comments	Sample Comments
1	154.0	20193	131280	SS11-HEX	154	0.00	Pass	500.0	[<Confirmed>]	
2	171.2	15044	102620	SS11-HEX	172	0.10	Pass	500.0	[<Confirmed>]	
3	224.6	8122	64536	SS21-HEX	225	0.40	Pass	500.0	[<Confirmed>]	
4	231.2	4371	36241	SS21-HEX	231	0.10	Pass	500.0	[<Confirmed>]	

Sample 108: SSS13_SS20_SS11_SS21_SS02_SS19_HRS37_J13.fsa

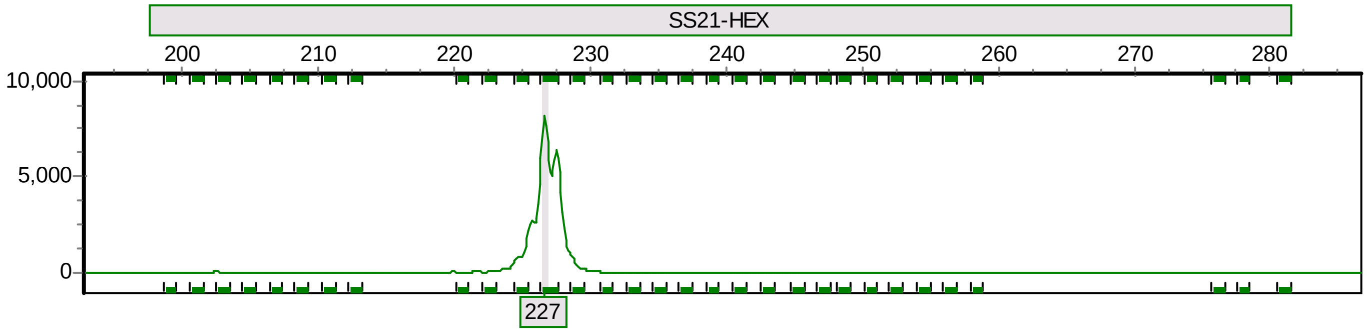

No	Size	Height	Area	Marker	Allele	Difference	Quality	Score	Allele Comments	Sample Comments
1	159.6	12534	78037	SS11-HEX	160	0.00	Pass	500.0	[<Confirmed>]	
2	161.5	8915	60026	SS11-HEX	162	0.00	Pass	500.0	[<Confirmed>]	
3	226.7	8154	66003	SS21-HEX	227	0.30	Pass	500.0	[<Confirmed>]	

Allele Report

Sample 109: SSS13_SS20_SS11_SS21_SS02_SS19_HRS38_J07.fsa

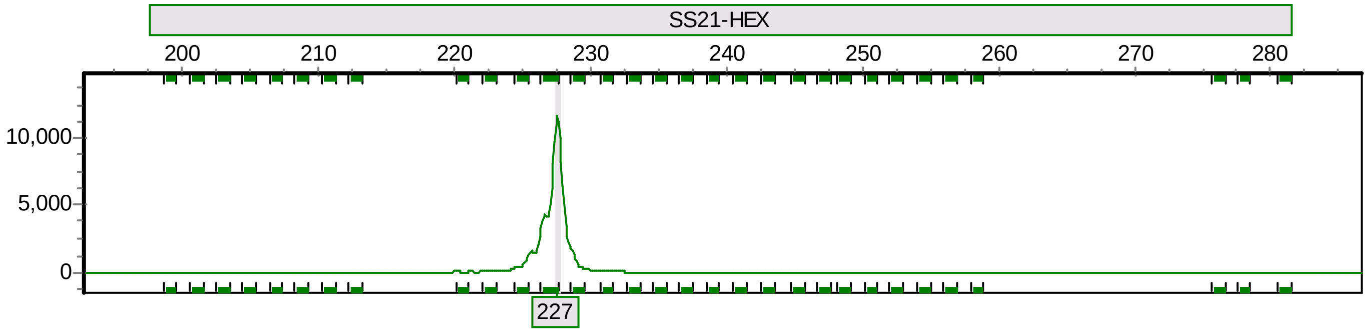

No	Size	Height	Area	Marker	Allele	Difference	Quality	Score	Allele Comments	Sample Comments
1	146.0	15736	116267	SS11-HEX	146	0.20	Pass	500.0	[<Confirmed>]	
2	165.6	6968	50063	SS11-HEX	166	0.20	Pass	500.0	[<Confirmed>]	
3	227.6	11526	95005	SS21-HEX	227	0.60	Pass	500.0	[<Confirmed>]	

Sample 110: SSS13_SS20_SS11_SS21_SS02_SS19_HRS39_N07.fsa

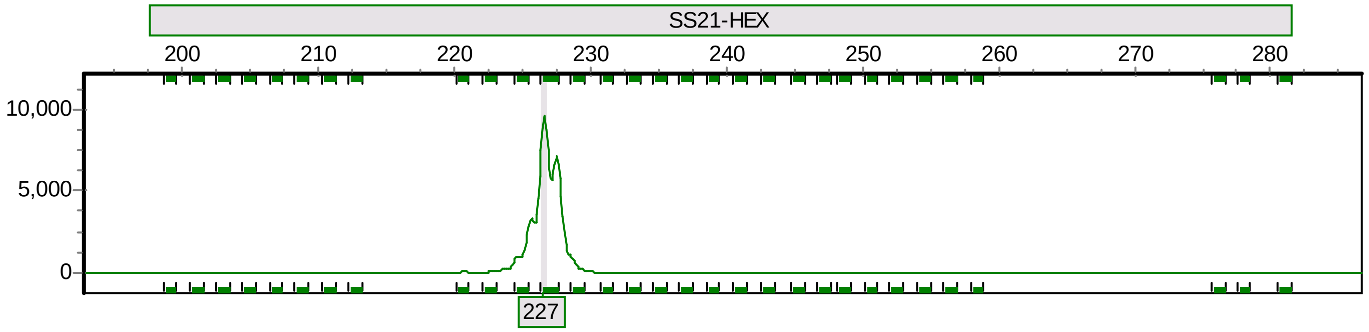

No	Size	Height	Area	Marker	Allele	Difference	Quality	Score	Allele Comments	Sample Comments
1	155.8	16061	101569	SS11-HEX	156	0.10	Pass	500.0	[<Confirmed>]	
2	163.4	9108	62084	SS11-HEX	164	0.00	Pass	500.0	[<Confirmed>]	
3	226.6	9559	73973	SS21-HEX	227	0.40	Pass	500.0	[<Confirmed>]	

Sample 111: SSS13_SS20_SS11_SS21_SS02_SS19_HRS40_A07.fsa

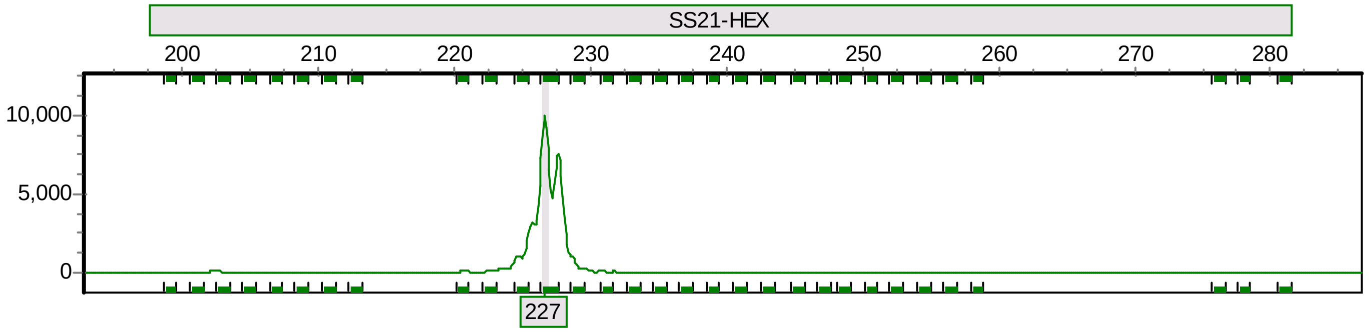

No	Size	Height	Area	Marker	Allele	Difference	Quality	Score	Allele Comments	Sample Comments
1	156.0	13055	82855	SS11-HEX	156	0.10	Pass	500.0	[<Confirmed>]	
2	165.4	8348	55375	SS11-HEX	166	0.00	Pass	500.0	[<Confirmed>]	
3	226.7	10007	78391	SS21-HEX	227	0.30	Pass	500.0	[<Confirmed>]	

Sample 112: SSS13_SS20_SS11_SS21_SS02_SS19_HRS41_K07.fsa

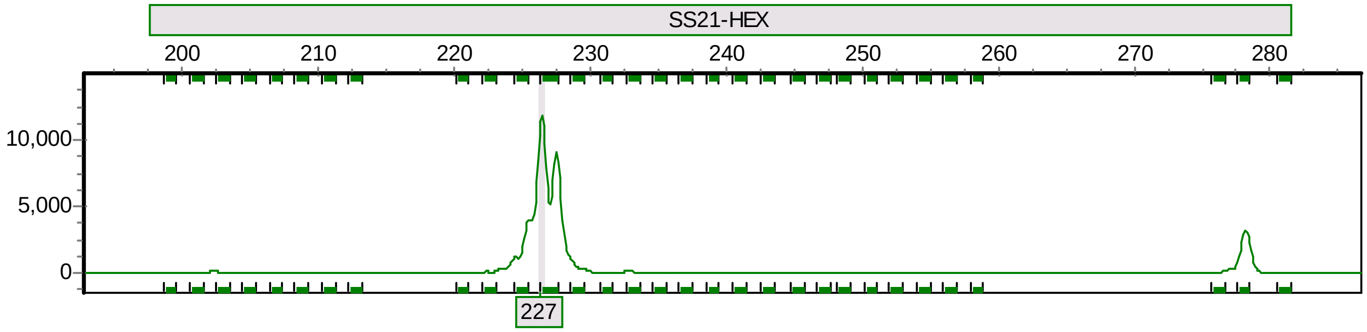

No	Size	Height	Area	Marker	Allele	Difference	Quality	Score	Allele Comments	Sample Comments
1	155.9	15786	100863	SS11-HEX	156	0.00	Pass	500.0	[<Confirmed>]	
2	163.4	10893	73177	SS11-HEX	164	0.00	Pass	500.0	[<Confirmed>]	
3	226.5	11694	93686	SS21-HEX	227	0.50	Pass	500.0	[<Confirmed>]	
4	227.6	9036	71760	SS21-HEX	227	0.60	Pass	500.0	[<Deleted>]	

Sample 113: SSS13_SS20_SS11_SS21_SS02_SS19_HRS42_I07.fsa

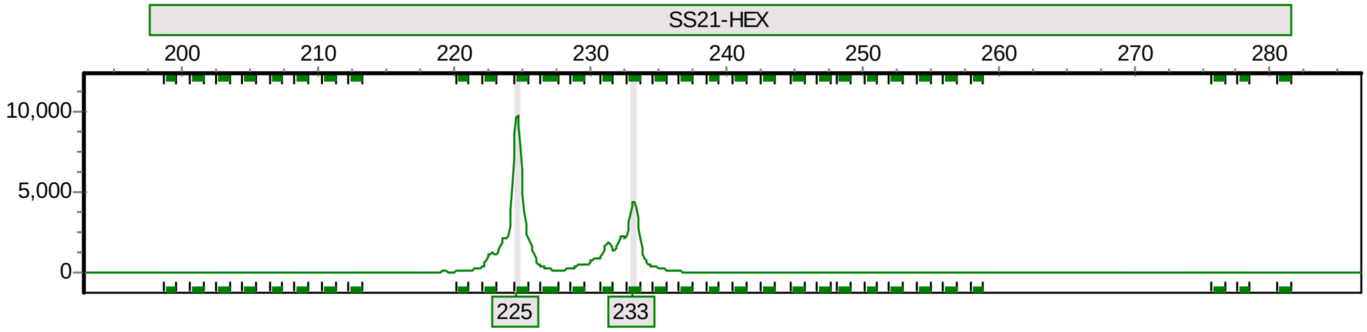

No	Size	Height	Area	Marker	Allele	Difference	Quality	Score	Allele Comments	Sample Comments
1	157.7	38078	269476	SS11-HEX	158	0.00	Pass	500.0	[<SAT (Repaired)><Confirmed>]	
2	224.7	9714	78890	SS21-HEX	225	0.30	Pass	500.0	[<Confirmed>]	
3	233.2	4416	37186	SS21-HEX	233	0.10	Pass	500.0	[<Confirmed>]	

Sample 114: SSS13_SS20_SS11_SS21_SS02_SS19_HTHL11_M11.fsa

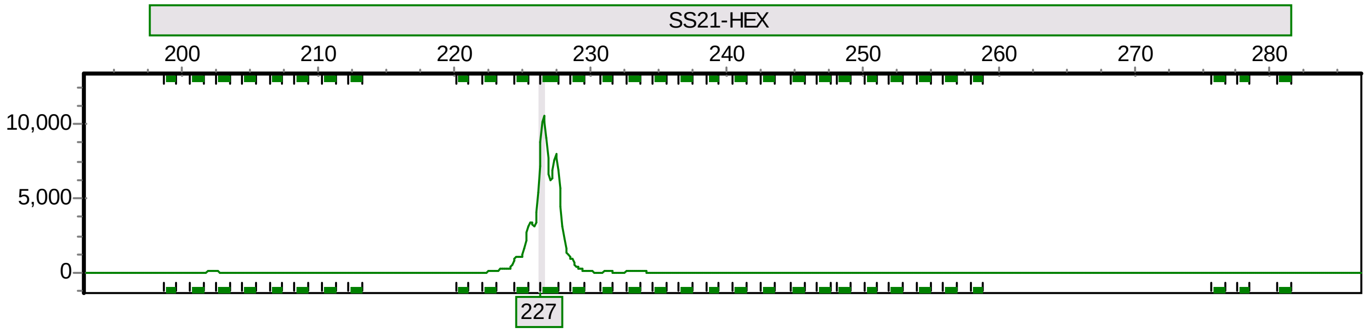

No	Size	Height	Area	Marker	Allele	Difference	Quality	Score	Allele Comments	Sample Comments
1	163.4	16020	107719	SS11-HEX	164	0.00	Pass	500.0	[<Confirmed>]	
2	226.4	8713	84977	SS21-HEX	227	0.60	Pass	500.0	[<Confirmed>]	
3	227.5	7906	58474	SS21-HEX	227	0.50	Pass	500.0	[<Deleted>]	

Sample 115: SSS13_SS20_SS11_SS21_SS02_SS19_HTHL13_H01.fsa

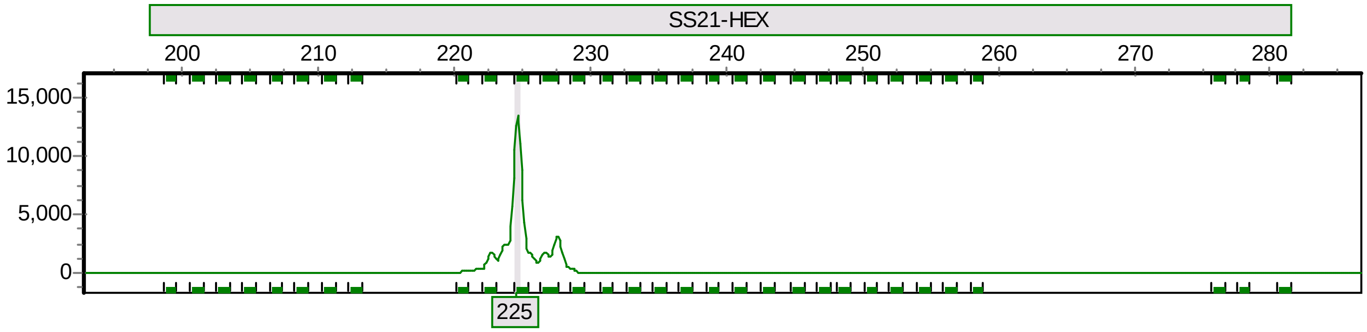

No	Size	Height	Area	Marker	Allele	Difference	Quality	Score	Allele Comments	Sample Comments
1	145.9	30522	220512	SS11-HEX	146	0.30	Pass	500.0	[<Confirmed>]	
2	224.7	13414	100240	SS21-HEX	225	0.30	Pass	500.0	[<Confirmed>]	

Sample 116: SSS13_SS20_SS11_SS21_SS02_SS19_HTHL14_F15.fsa

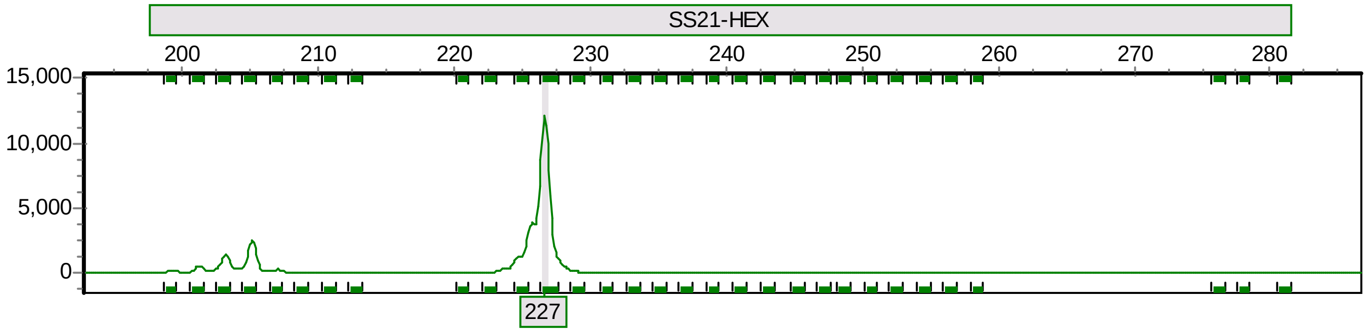

No	Size	Height	Area	Marker	Allele	Difference	Quality	Score	Allele Comments	Sample Comments
1	161.6	8017	56217	SS11-HEX	162	0.10	Pass	500.0	[<Confirmed>]	
2	169.4	5126	37103	SS11-HEX	170	0.10	Pass	500.0	[<Confirmed>]	
3	226.7	12048	96843	SS21-HEX	227	0.30	Pass	500.0	[<Confirmed>]	

Sample 117: SSS13_SS20_SS11_SS21_SS02_SS19_HTHL15_F01.fsa

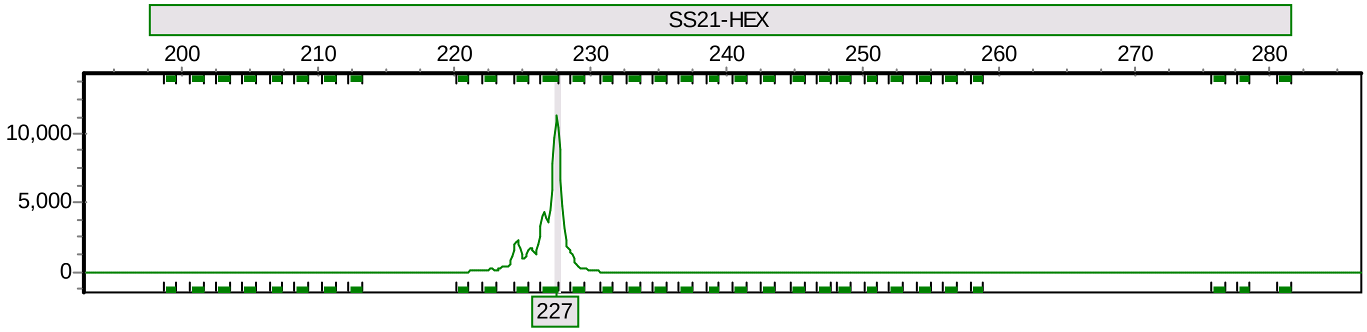

No	Size	Height	Area	Marker	Allele	Difference	Quality	Score	Allele Comments	Sample Comments
1	163.3	30661	196242	SS11-HEX	164	0.10	Pass	500.0	[<Confirmed>]	
2	227.6	11172	82982	SS21-HEX	227	0.60	Pass	500.0	[<Confirmed>]	

Sample 118: SSS13_SS20_SS11_SS21_SS02_SS19_HTHL1_E13.fsa

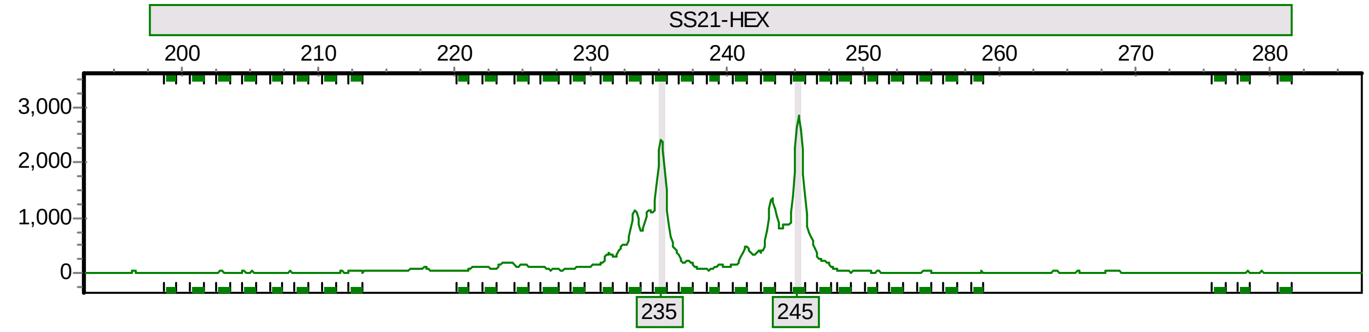

No	Size	Height	Area	Marker	Allele	Difference	Quality	Score	Allele Comments	Sample Comments
1	159.7	18263	119502	SS11-HEX	160	0.10	Pass	500.0	[<Confirmed>]	
2	235.2	2404	20143	SS21-HEX	235	0.00	Pass	250.3	[<Confirmed>]	
3	245.3	2847	23890	SS21-HEX	245	0.00	Pass	354.8	[<Confirmed>]	

Sample 119: SSS13_SS20_SS11_SS21_SS02_SS19_HTHL3_D05.fsa

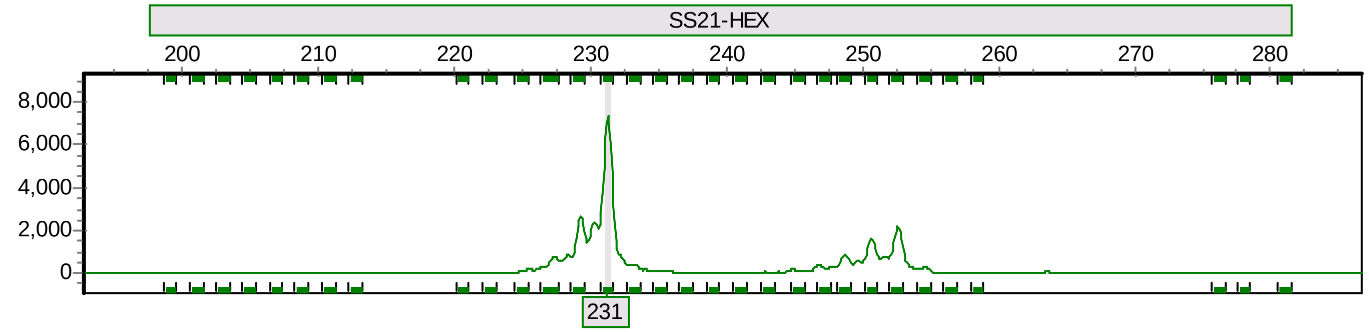

No	Size	Height	Area	Marker	Allele	Difference	Quality	Score	Allele Comments	Sample Comments
1	157.6	27211	164236	SS11-HEX	158	0.10	Pass	500.0	[<Confirmed>]	
2	173.1	12297	83517	SS11-HEX	174	0.20	Pass	500.0	[<Confirmed>]	
3	231.3	7287	54782	SS21-HEX	231	0.00	Pass	500.0	[<Confirmed>]	

Sample 120: SSS13_SS20_SS11_SS21_SS02_SS19_HTHL4_O15.fsa

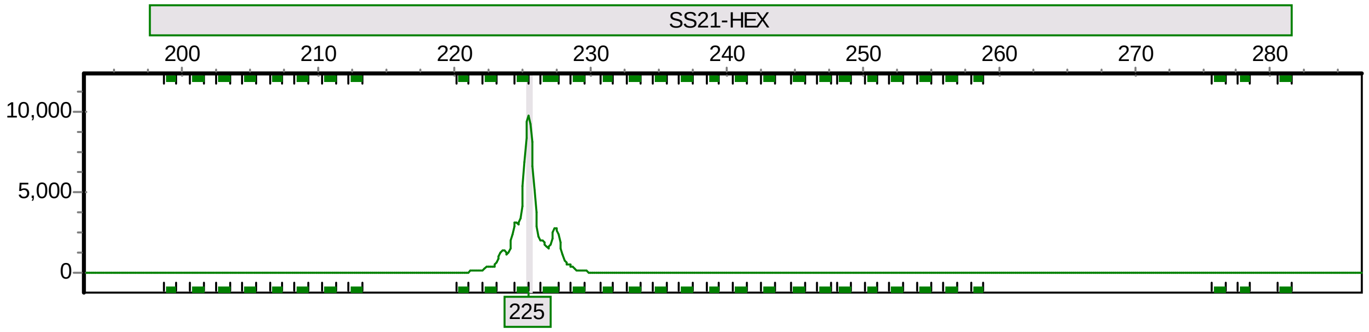

No	Size	Height	Area	Marker	Allele	Difference	Quality	Score	Allele Comments	Sample Comments
1	153.9	30140	202329	SS11-HEX	154	0.10	Pass	500.0	[<Confirmed>]	
2	225.5	9729	79498	SS21-HEX	225	0.50	Pass	500.0	[<Confirmed>]	

Allele Report

Sample 121: SSS13_SS20_SS11_SS21_SS02_SS19_HTHL5_M13.fsa

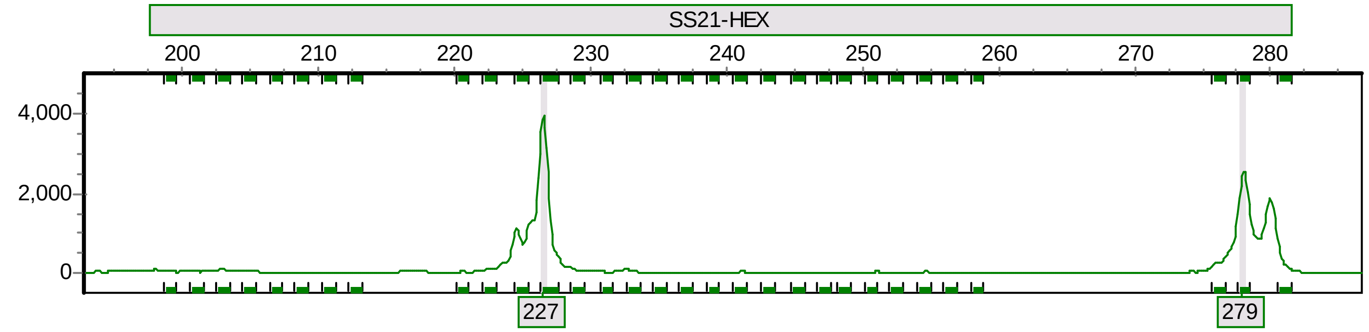

No	Size	Height	Area	Marker	Allele	Difference	Quality	Score	Allele Comments	Sample Comments
1	155.9	17051	113697	SS11-HEX	156	0.00	Pass	500.0	[<Confirmed>]	
2	226.6	3927	32380	SS21-HEX	227	0.40	Pass	500.0	[<Confirmed>]	
3	278.0	2554	23873	SS21-HEX	279	0.10	Pass	232.5	[<Confirmed>]	

Sample 122: SSS13_SS20_SS11_SS21_SS02_SS19_HTHL6_P03.fsa

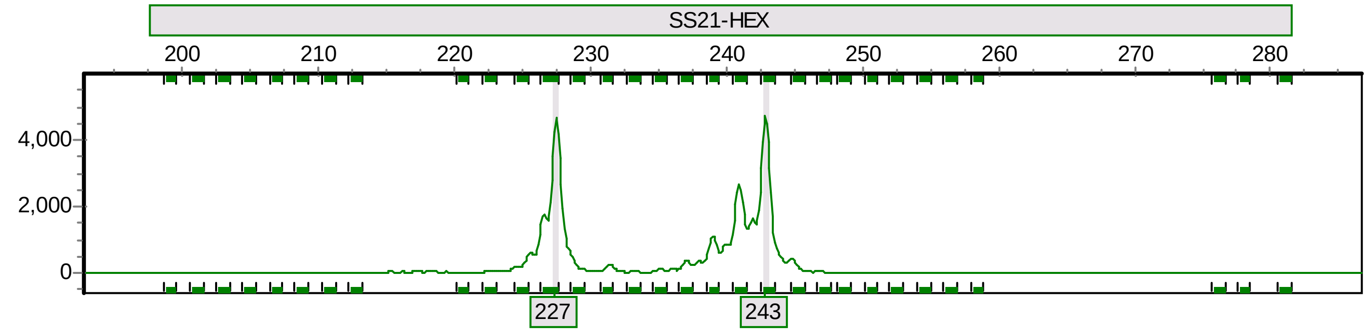

No	Size	Height	Area	Marker	Allele	Difference	Quality	Score	Allele Comments	Sample Comments
1	171.3	15927	105052	SS11-HEX	172	0.00	Pass	500.0	[<Confirmed>]	
2	227.5	4643	35336	SS21-HEX	227	0.50	Pass	500.0	[<Confirmed>]	
3	242.9	4702	37790	SS21-HEX	243	0.20	Pass	500.0	[<Confirmed>]	

Sample 123: SSS13_SS20_SS11_SS21_SS02_SS19_HTHL8_A03.fsa

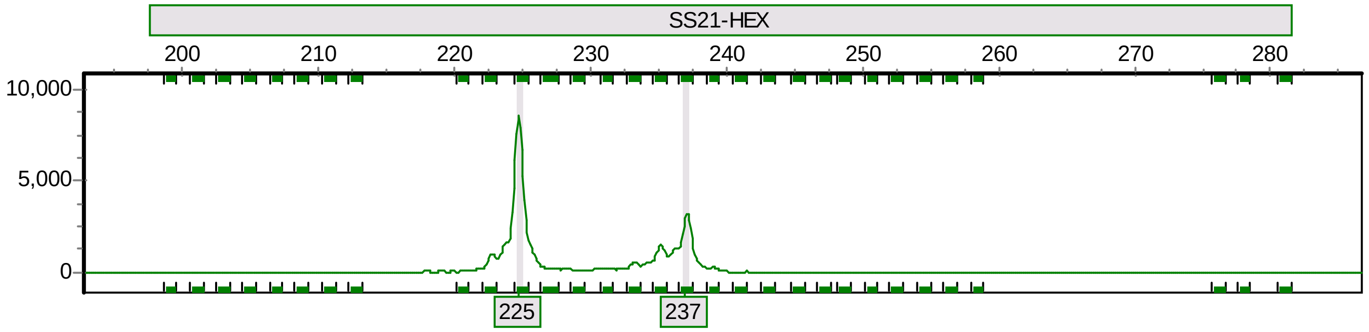

No	Size	Height	Area	Marker	Allele	Difference	Quality	Score	Allele Comments	Sample Comments
1	171.4	16648	116131	SS11-HEX	172	0.10	Pass	500.0	[<Confirmed>]	
2	224.8	8492	67191	SS21-HEX	225	0.20	Pass	500.0	[<Confirmed>]	
3	237.1	3215	24933	SS21-HEX	237	0.00	Pass	450.0	[<Confirmed>]	

Sample 124: SSS13_SS20_SS11_SS21_SS02_SS19_HTHL9_O09.fsa

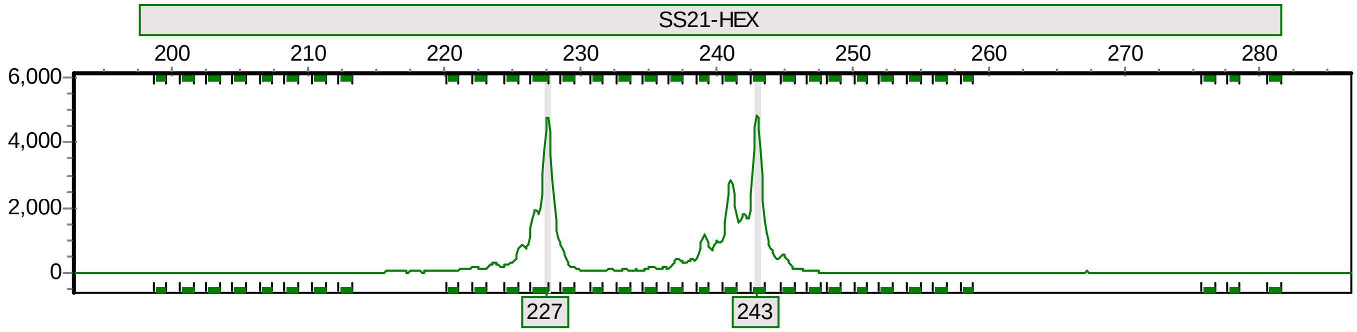

No	Size	Height	Area	Marker	Allele	Difference	Quality	Score	Allele Comments	Sample Comments
1	145.9	31613	247304	SS11-HEX	146	0.30	Pass	500.0	[<Confirmed>]	
2	227.6	4744	37481	SS21-HEX	227	0.60	Pass	500.0	[<Confirmed>]	
3	243.0	4786	40006	SS21-HEX	243	0.10	Pass	500.0	[<Confirmed>]	
