# Supplementary material for: Genetic diversity of Schima superba based on physiological traits and SSR markers
Source: PLoS One. 2026 Apr 10;21(4):e0344465. doi: 10.1371/journal.pone.0344465 (PMC13068225; doi:10.1371/journal.pone.0344465)
Supplement: S1 File — (ZIP) [file pone.0344465.s003.zip › SS23.pdf]

Allele Report

Project Comments:

Sample 1: SSS18_SS24_SS05_SS32_SS30_SS12_SS23_HBB10_E06.fsa

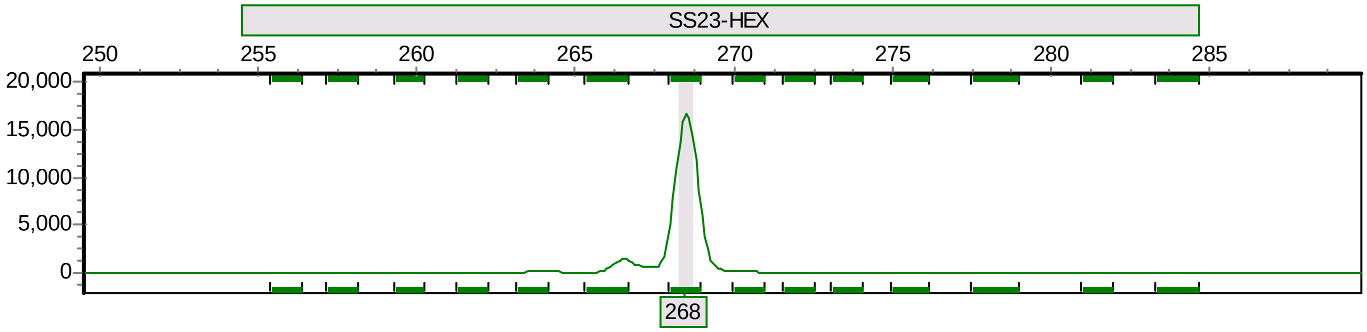

No	Size	Height	Area	Marker	Allele	Difference	Quality	Score	Allele Comments	Sample Comments
1	124.6	23701	147555	SS30-HEX	125	0.10	Pass	500.0	[<Confirmed>]	
2	203.3	5849	40824	SS12-HEX	203	0.00	Pass	500.0	[<Confirmed>]	
3	268.5	16747	131691	SS23-HEX	268	0.00	Pass	500.0	[<Confirmed>]	

Sample 2: SSS18_SS24_SS05_SS32_SS30_SS12_SS23_HBB12-2_D12.fsa

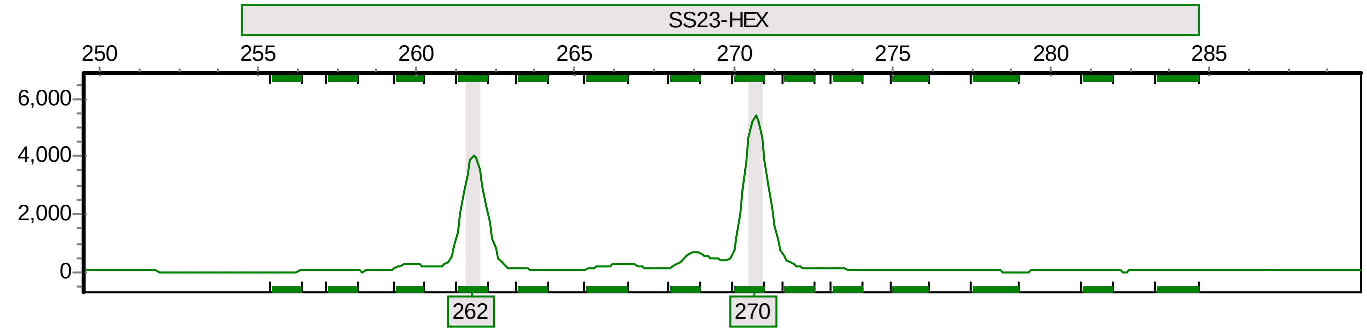

No	Size	Height	Area	Marker	Allele	Difference	Quality	Score	Allele Comments	Sample Comments
1	120.5	18597	120226	SS30-HEX	121	0.10	Pass	500.0	[<Confirmed>]	
2	138.9	7231	51876	SS30-HEX	139	0.20	Pass	500.0	[<Confirmed>]	
3	215.2	1926	14246	SS12-HEX	215	0.20	Pass	276.1	[<Confirmed>]	
4	261.8	4044	33950	SS23-HEX	262	0.00	Pass	500.0	[<Confirmed>]	
5	270.7	5384	45773	SS23-HEX	270	0.20	Pass	500.0	[<Confirmed>]	

Sample 3: SSS18_SS24_SS05_SS32_SS30_SS12_SS23_HBB13_C18.fsa

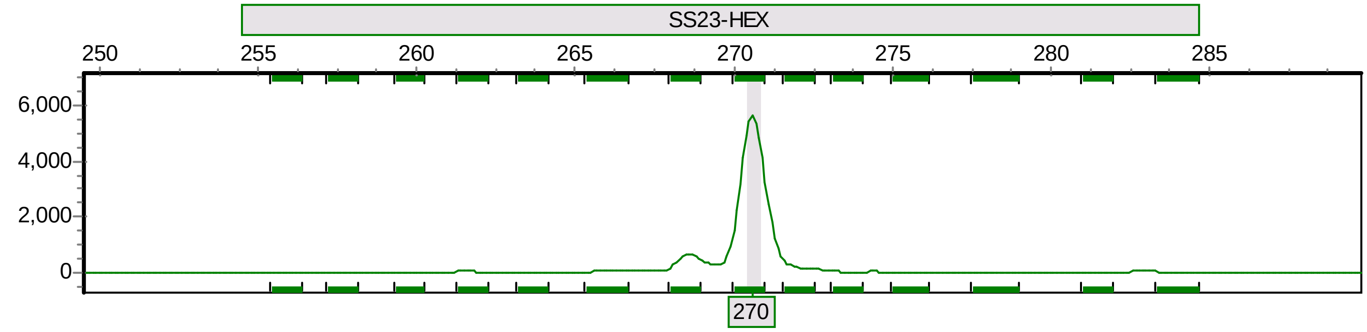

No	Size	Height	Area	Marker	Allele	Difference	Quality	Score	Allele Comments	Sample Comments
1	126.7	20070	133350	SS30-HEX	127	0.00	Pass	500.0	[<Confirmed>]	
2	139.0	7328	51970	SS30-HEX	139	0.10	Pass	500.0	[<Confirmed>]	
3	203.3	8113	62041	SS12-HEX	203	0.00	Pass	500.0	[<Confirmed>]	
4	270.6	5611	50110	SS23-HEX	270	0.10	Pass	500.0	[<Confirmed>]	

Sample 4: SSS18_SS24_SS05_SS32_SS30_SS12_SS23_HBB14_G04.fsa

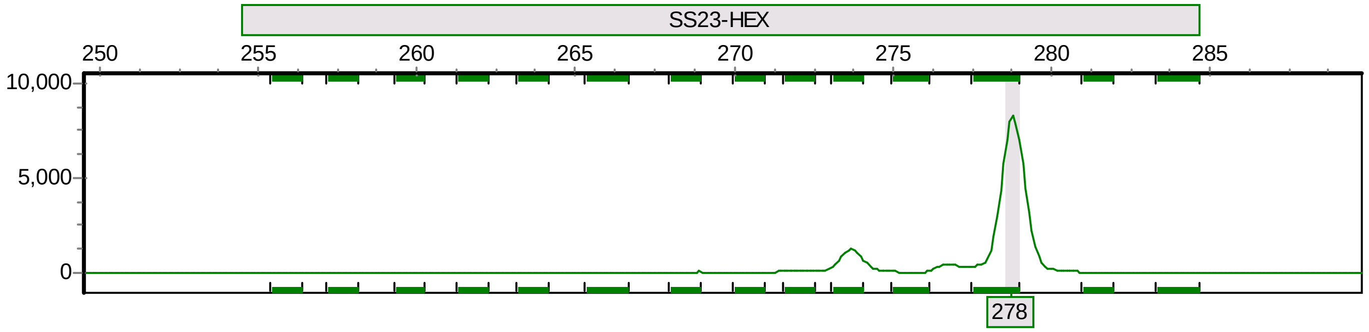

No	Size	Height	Area	Marker	Allele	Difference	Quality	Score	Allele Comments	Sample Comments
1	131.0	10158	64954	SS30-HEX	131	0.10	Pass	500.0	[<Confirmed>]	
2	135.0	7404	47206	SS30-HEX	135	0.10	Pass	500.0	[<Confirmed>]	
3	211.4	8023	56770	SS12-HEX	211	0.00	Pass	500.0	[<Confirmed>]	
4	217.4	5634	40709	SS12-HEX	217	0.00	Pass	500.0	[<Confirmed>]	
5	278.8	8288	69098	SS23-HEX	278	0.60	Pass	500.0	[<Confirmed>]	

Sample 5: SSS18_SS24_SS05_SS32_SS30_SS12_SS23_HBB15_B08.fsa

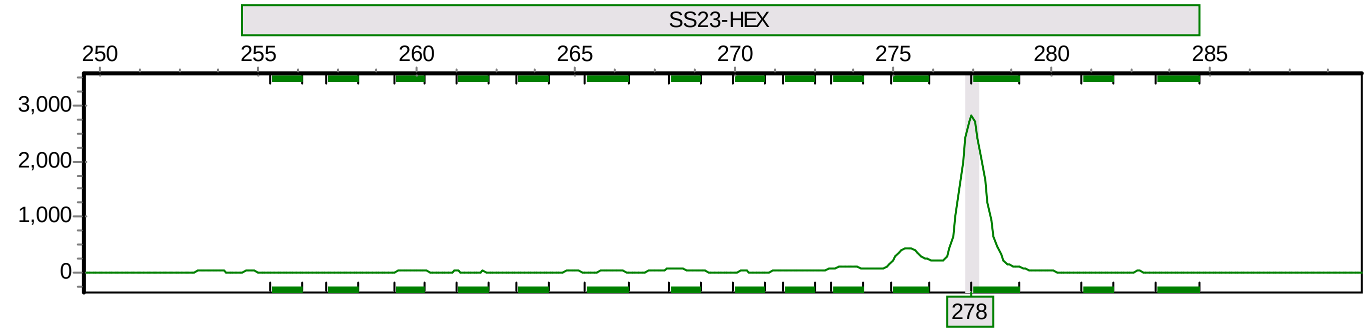

No	Size	Height	Area	Marker	Allele	Difference	Quality	Score	Allele Comments	Sample Comments
1	114.5	29687	180559	SS30-HEX	115	0.10	Pass	500.0	[<Confirmed>]	
2	203.3	3543	25549	SS12-HEX	203	0.00	Pass	500.0	[<Confirmed>]	
3	277.5	2795	24609	SS23-HEX	278	0.70	Pass	343.9	[<Confirmed>]	

Sample 6: SSS18_SS24_SS05_SS32_SS30_SS12_SS23_HBB16_G08.fsa

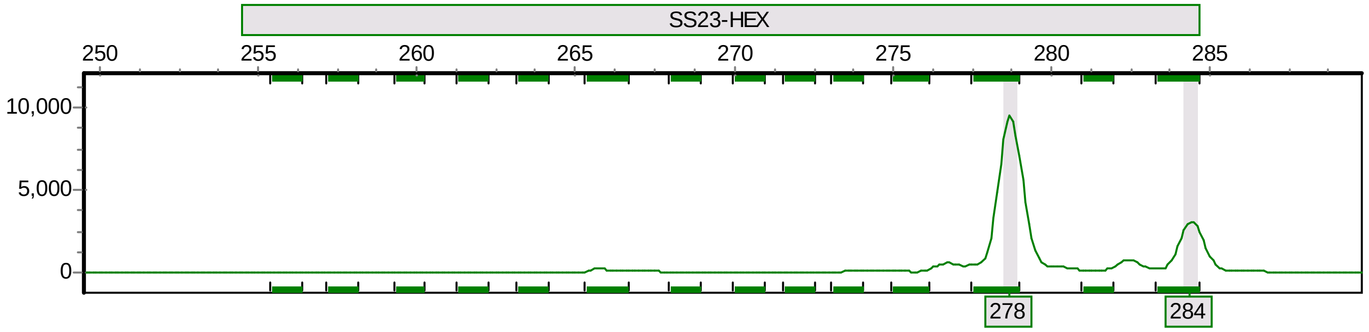

No	Size	Height	Area	Marker	Allele	Difference	Quality	Score	Allele Comments	Sample Comments
1	130.9	12759	81891	SS30-HEX	131	0.00	Pass	500.0	[<Confirmed>]	
2	134.9	7941	51791	SS30-HEX	135	0.00	Pass	500.0	[<Confirmed>]	
3	217.4	15000	112685	SS12-HEX	217	0.00	Pass	500.0	[<Confirmed>]	
4	278.7	9476	82175	SS23-HEX	278	0.50	Pass	500.0	[<Confirmed>]	
5	284.4	3123	27679	SS23-HEX	284	0.40	Pass	383.4	[<Confirmed>]	

Sample 7: SSS18_SS24_SS05_SS32_SS30_SS12_SS23_HBB17_K16.fsa

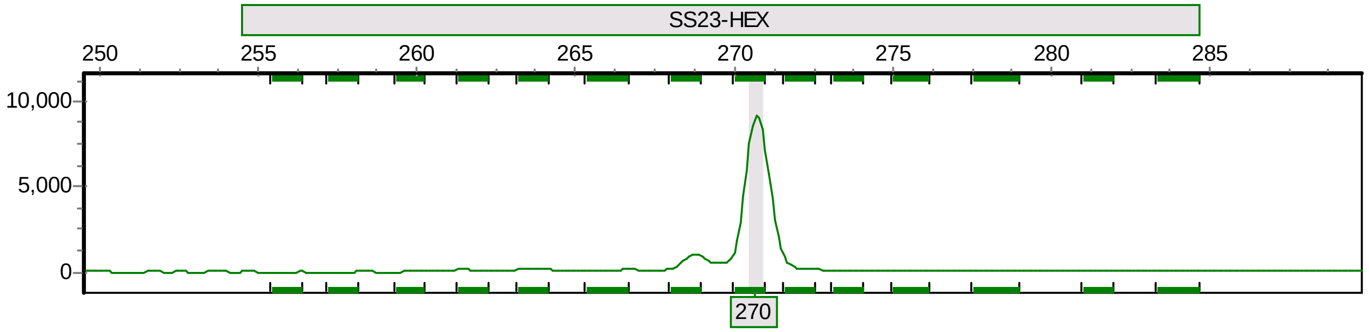

No	Size	Height	Area	Marker	Allele	Difference	Quality	Score	Allele Comments	Sample Comments
1	118.6	32245	228162	SS30-HEX	119	0.00	Pass	500.0	[<Confirmed>]	
2	207.3	1071	8400	SS12-HEX	207	0.00	Pass	107.9	[<Confirmed>]	
3	215.4	1489	11787	SS12-HEX	215	0.00	Pass	168.7	[<Confirmed>]	
4	270.7	9083	78247	SS23-HEX	270	0.20	Pass	500.0	[<Confirmed>]	

Sample 8: SSS18_SS24_SS05_SS32_SS30_SS12_SS23_HBB18_O06.fsa

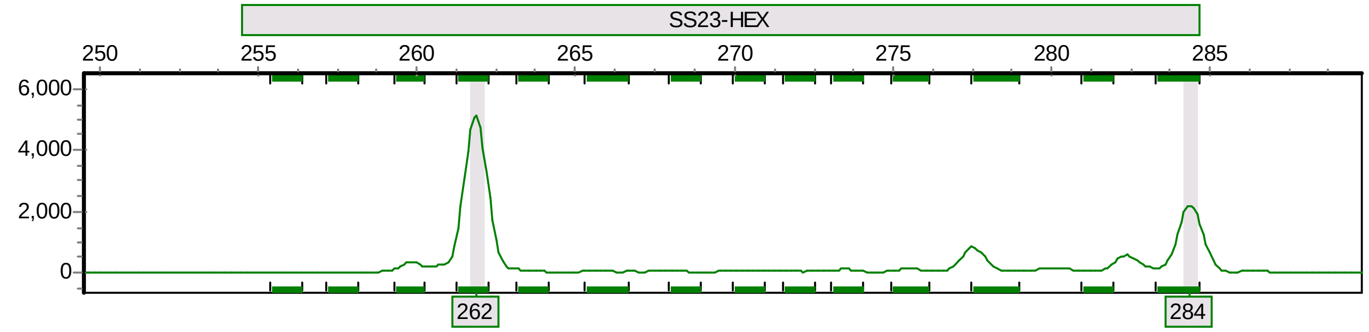

No	Size	Height	Area	Marker	Allele	Difference	Quality	Score	Allele Comments	Sample Comments
1	118.6	23012	143315	SS30-HEX	119	0.00	Pass	500.0	[<Confirmed>]	
2	205.5	9922	71401	SS12-HEX	205	0.10	Pass	500.0	[<Confirmed>]	
3	261.9	5092	42523	SS23-HEX	262	0.10	Pass	500.0	[<Confirmed>]	
4	284.4	2195	19425	SS23-HEX	284	0.40	Pass	226.2	[<Confirmed>]	

Sample 9: SSS18_SS24_SS05_SS32_SS30_SS12_SS23_HBB19_O08.fsa

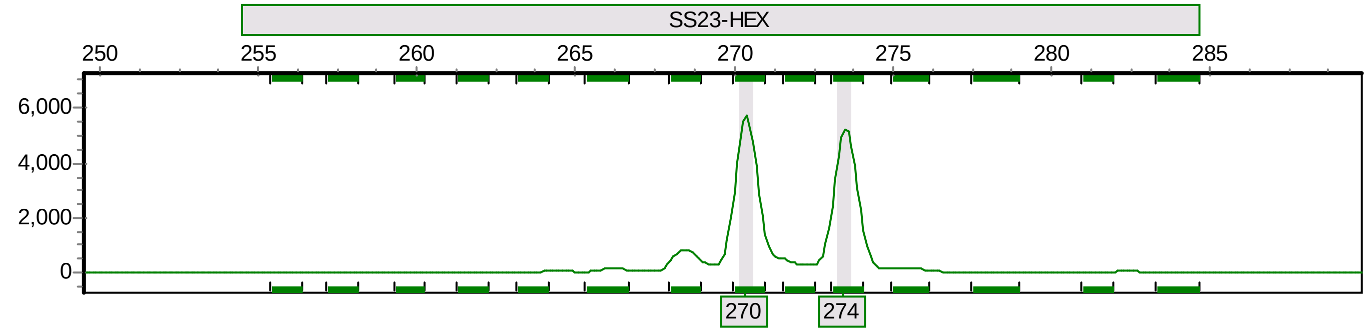

No	Size	Height	Area	Marker	Allele	Difference	Quality	Score	Allele Comments	Sample Comments
1	120.7	24351	158143	SS30-HEX	121	0.10	Pass	500.0	[<Confirmed>]	
2	122.5	16033	100447	SS30-HEX	123	0.10	Pass	500.0	[<Confirmed>]	
3	217.4	5983	43513	SS12-HEX	217	0.00	Pass	500.0	[<Confirmed>]	
4	270.4	5687	46146	SS23-HEX	270	0.10	Pass	500.0	[<Confirmed>]	
5	273.5	5204	43159	SS23-HEX	274	0.10	Pass	500.0	[<Confirmed>]	

Allele Report

Sample 10: SSS18_SS24_SS05_SS32_SS30_SS12_SS23_HBB1_B12.fsa

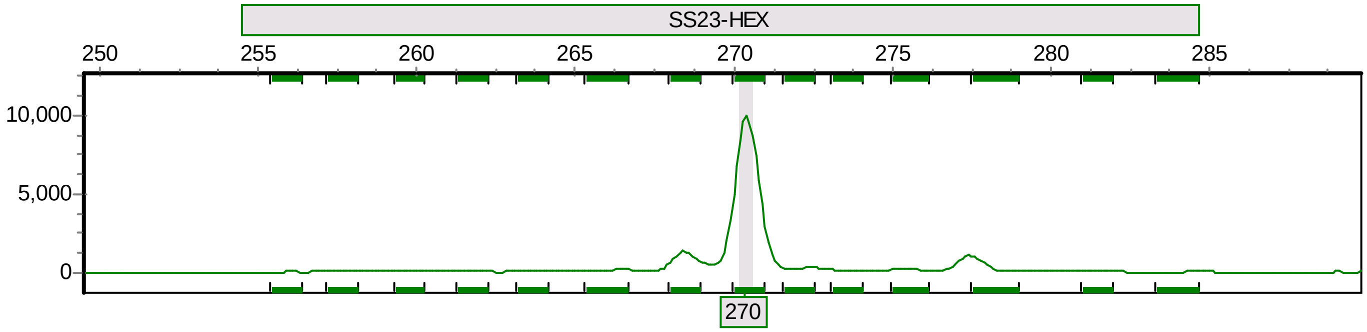

No	Size	Height	Area	Marker	Allele	Difference	Quality	Score	Allele Comments	Sample Comments
1	126.7	13329	87214	SS30-HEX	127	0.00	Pass	500.0	[<Confirmed>]	
2	132.9	9128	59988	SS30-HEX	133	0.00	Pass	500.0	[<Confirmed>]	
3	205.4	17542	127786	SS12-HEX	205	0.00	Pass	500.0	[<Confirmed>]	
4	270.4	9978	84010	SS23-HEX	270	0.10	Pass	500.0	[<Confirmed>]	

Sample 11: SSS18_SS24_SS05_SS32_SS30_SS12_SS23_HBB20_F12.fsa

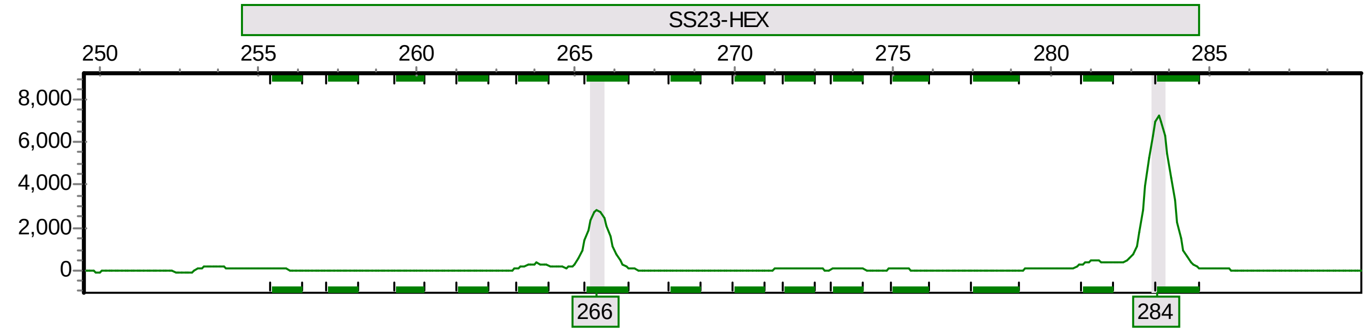

No	Size	Height	Area	Marker	Allele	Difference	Quality	Score	Allele Comments	Sample Comments
1	118.6	16460	105174	SS30-HEX	119	0.00	Pass	500.0	[<Confirmed>]	
2	128.9	10808	70608	SS30-HEX	129	0.10	Pass	500.0	[<Confirmed>]	
3	219.2	12637	95879	SS12-HEX	219	0.20	Pass	500.0	[<Confirmed>]	
4	265.7	2857	23324	SS23-HEX	266	0.30	Pass	398.8	[<Confirmed>]	
5	283.4	7174	63814	SS23-HEX	284	0.60	Pass	500.0	[<Confirmed>]	

Sample 12: SSS18_SS24_SS05_SS32_SS30_SS12_SS23_HBB21_J10.fsa

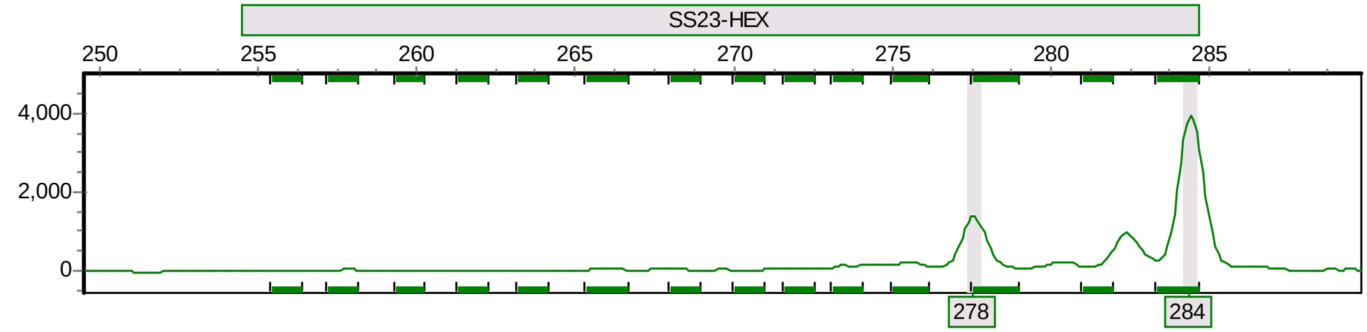

No	Size	Height	Area	Marker	Allele	Difference	Quality	Score	Allele Comments	Sample Comments
1	118.6	14113	88120	SS30-HEX	119	0.00	Pass	500.0	[<Confirmed>]	
2	139.2	6260	45391	SS30-HEX	139	0.10	Pass	500.0	[<Confirmed>]	
3	217.5	8797	66037	SS12-HEX	217	0.10	Pass	500.0	[<Confirmed>]	
4	277.6	1374	12312	SS23-HEX	278	0.60	Pass	113.1	[<Confirmed>]	
5	284.4	3922	35122	SS23-HEX	284	0.40	Pass	500.0	[<Confirmed>]	

Sample 13: SSS18_SS24_SS05_SS32_SS30_SS12_SS23_HBB22_K06.fsa

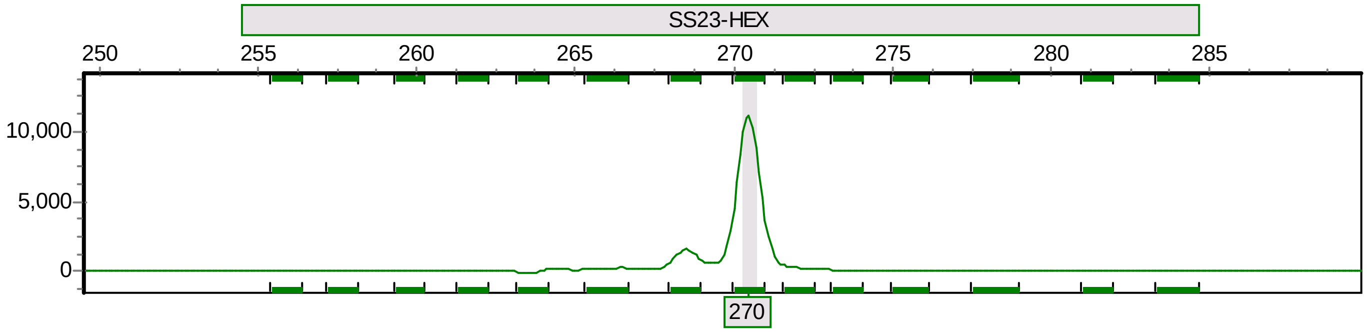

No	Size	Height	Area	Marker	Allele	Difference	Quality	Score	Allele Comments	Sample Comments
1	126.9	21108	131633	SS30-HEX	127	0.20	Pass	500.0	[<Confirmed>]	
2	217.4	15553	112785	SS12-HEX	217	0.00	Pass	500.0	[<Confirmed>]	
3	270.5	11173	91655	SS23-HEX	270	0.00	Pass	500.0	[<Confirmed>]	

Sample 14: SSS18_SS24_SS05_SS32_SS30_SS12_SS23_HBB23_B10.fsa

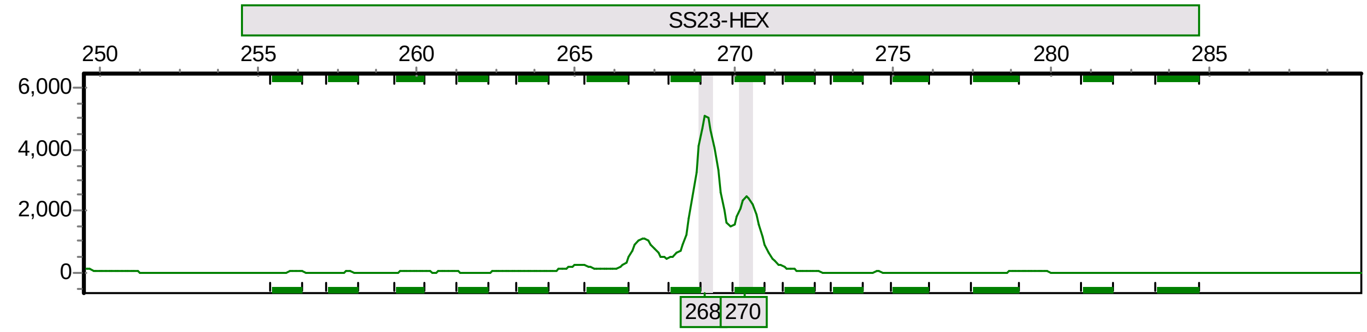

No	Size	Height	Area	Marker	Allele	Difference	Quality	Score	Allele Comments	Sample Comments
1	118.6	15946	103855	SS30-HEX	119	0.00	Pass	500.0	[<Confirmed>]	
2	128.7	9882	64471	SS30-HEX	129	0.10	Pass	500.0	[<Confirmed>]	
3	203.3	4174	30604	SS12-HEX	203	0.00	Pass	500.0	[<Confirmed>]	
4	229.3	1404	11409	SS12-HEX	229	0.00	Pass	147.9	[<Confirmed>]	
5	269.1	5070	44679	SS23-HEX	268	1.00	Pass	500.0	[<Confirmed><Edited>]	
6	270.4	2489	21398	SS23-HEX	270	0.10	Pass	235.2	[<Confirmed>]	

Sample 15: SSS18_SS24_SS05_SS32_SS30_SS12_SS23_HBB25-1_F10.fsa

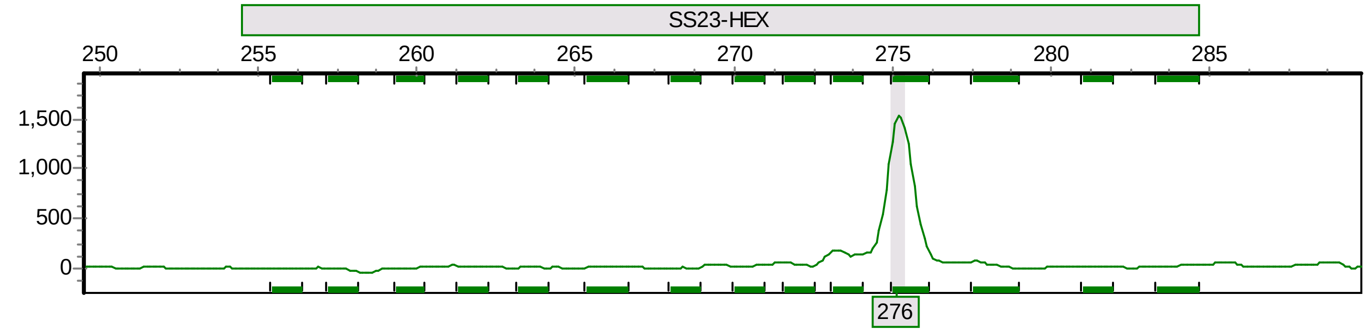

No	Size	Height	Area	Marker	Allele	Difference	Quality	Score	Allele Comments	Sample Comments
1	122.5	11170	72678	SS30-HEX	123	0.10	Pass	500.0	[<Confirmed>]	
2	128.8	7758	50525	SS30-HEX	129	0.00	Pass	500.0	[<Confirmed>]	
3	275.2	1526	14320	SS23-HEX	276	0.30	Pass	122.1	[<Confirmed>]	

Sample 16: SSS18_SS24_SS05_SS32_SS30_SS12_SS23_HBB25-2_N12.fsa

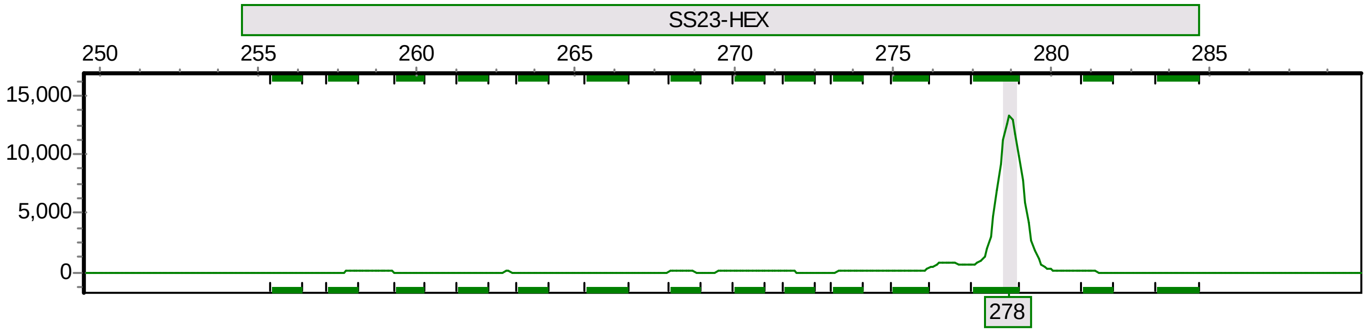

No	Size	Height	Area	Marker	Allele	Difference	Quality	Score	Allele Comments	Sample Comments
1	126.7	14027	88736	SS30-HEX	127	0.00	Pass	500.0	[<Confirmed>]	
2	215.4	14548	108535	SS12-HEX	215	0.00	Pass	500.0	[<Confirmed>]	
3	278.7	13204	114773	SS23-HEX	278	0.50	Pass	500.0	[<Confirmed>]	

Sample 17: SSS18_SS24_SS05_SS32_SS30_SS12_SS23_HBB26_D08.fsa

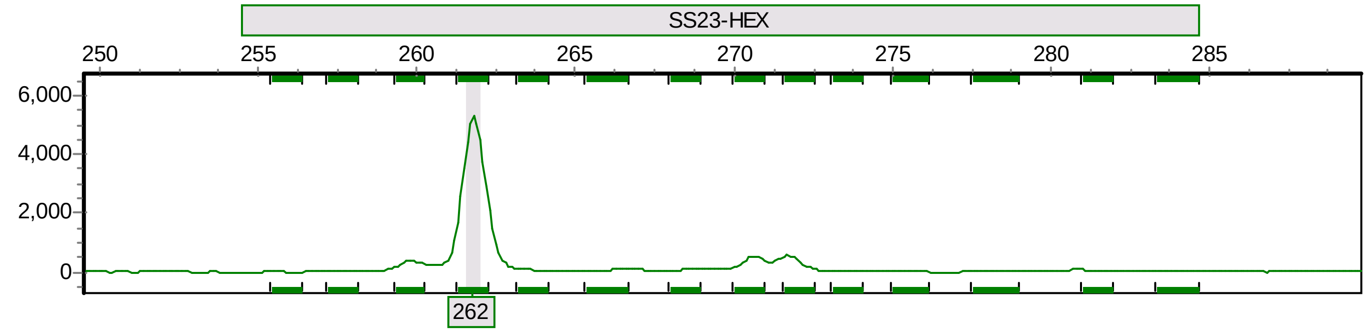

No	Size	Height	Area	Marker	Allele	Difference	Quality	Score	Allele Comments	Sample Comments
1	114.6	22323	139166	SS30-HEX	115	0.00	Pass	500.0	[<Confirmed>]	
2	118.6	19550	125339	SS30-HEX	119	0.00	Pass	500.0	[<Confirmed>]	
3	213.1	4750	34398	SS12-HEX	213	0.20	Pass	500.0	[<Confirmed>]	
4	229.3	2175	16807	SS12-HEX	229	0.00	Pass	300.9	[<Confirmed>]	
5	261.8	5267	42991	SS23-HEX	262	0.00	Pass	500.0	[<Confirmed>]	

Sample 18: SSS18_SS24_SS05_SS32_SS30_SS12_SS23_HBB27_H06.fsa

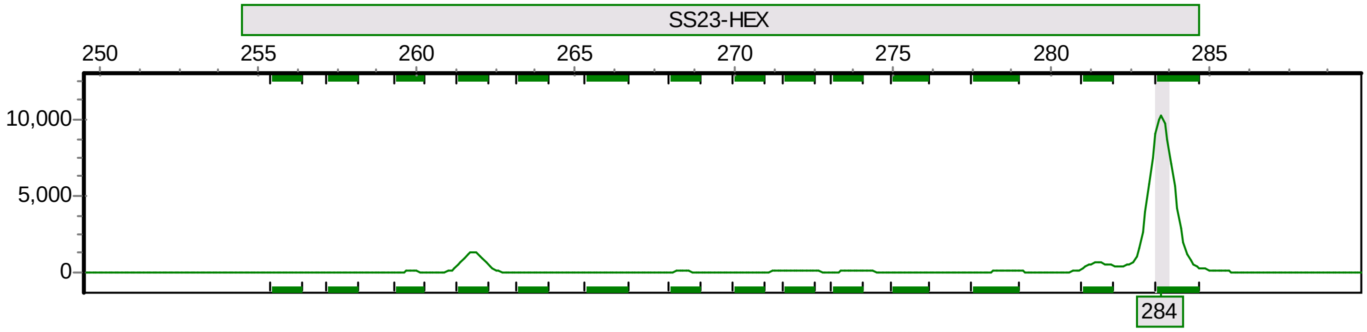

No	Size	Height	Area	Marker	Allele	Difference	Quality	Score	Allele Comments	Sample Comments
1	118.6	17719	110643	SS30-HEX	119	0.00	Pass	500.0	[<Confirmed>]	
2	126.7	12051	77971	SS30-HEX	127	0.00	Pass	500.0	[<Confirmed>]	
3	229.3	2317	17680	SS12-HEX	229	0.00	Pass	342.9	[<Confirmed>]	
4	283.5	10216	88269	SS23-HEX	284	0.50	Pass	500.0	[<Confirmed>]	

Sample 19: SSS18_SS24_SS05_SS32_SS30_SS12_SS23_HBB28_L12.fsa

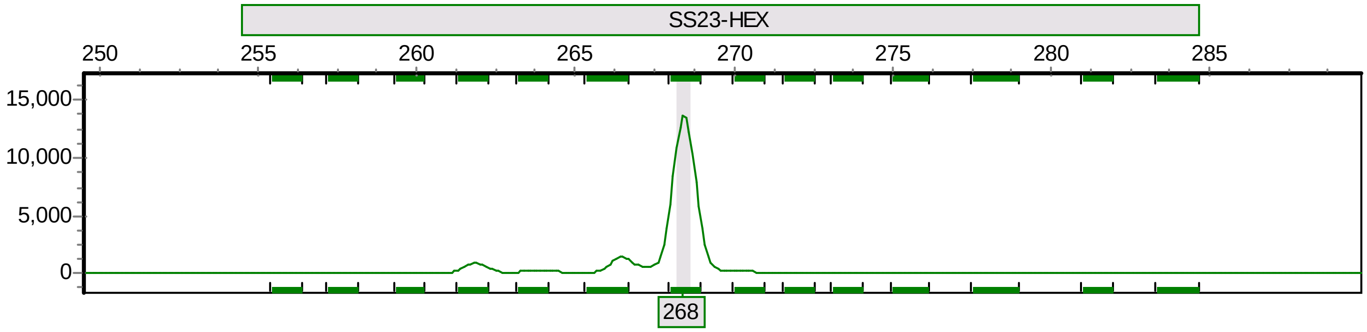

No	Size	Height	Area	Marker	Allele	Difference	Quality	Score	Allele Comments	Sample Comments
1	116.6	19263	117971	SS30-HEX	117	0.00	Pass	500.0	[<Confirmed>]	
2	128.7	12855	82021	SS30-HEX	129	0.10	Pass	500.0	[<Confirmed>]	
3	229.3	3324	25505	SS12-HEX	229	0.00	Pass	500.0	[<Confirmed>]	
4	268.4	13576	110465	SS23-HEX	268	0.10	Pass	500.0	[<Confirmed>]	

Sample 20: SSS18_SS24_SS05_SS32_SS30_SS12_SS23_HBB2_C08.fsa

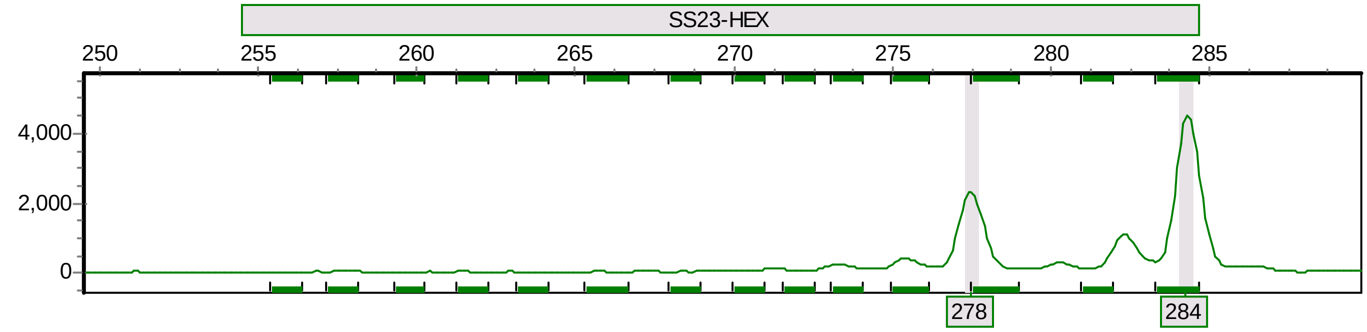

No	Size	Height	Area	Marker	Allele	Difference	Quality	Score	Allele Comments	Sample Comments
1	118.7	17648	111848	SS30-HEX	119	0.10	Pass	500.0	[<Confirmed>]	
2	133.0	8586	55564	SS30-HEX	133	0.10	Pass	500.0	[<Confirmed>]	
3	205.4	16120	115073	SS12-HEX	205	0.00	Pass	500.0	[<Confirmed>]	
4	277.5	2350	20497	SS23-HEX	278	0.70	Pass	252.7	[<Confirmed>]	
5	284.3	4528	39523	SS23-HEX	284	0.30	Pass	500.0	[<Confirmed>]	

Sample 21: SSS18_SS24_SS05_SS32_SS30_SS12_SS23_HBB30_L14.fsa

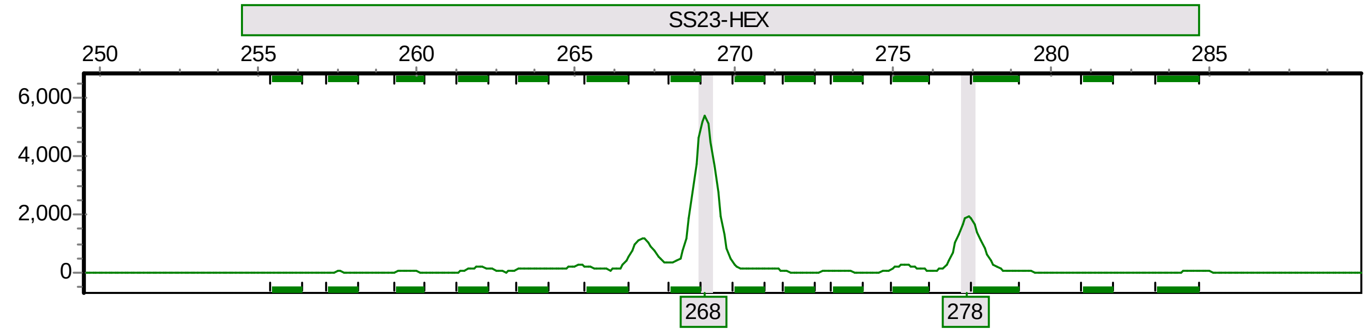

No	Size	Height	Area	Marker	Allele	Difference	Quality	Score	Allele Comments	Sample Comments
1	118.6	16551	103308	SS30-HEX	119	0.00	Pass	500.0	[<Confirmed>]	
2	126.7	10643	67578	SS30-HEX	127	0.00	Pass	500.0	[<Confirmed>]	
3	203.2	6759	49560	SS12-HEX	203	0.10	Pass	500.0	[<Confirmed>]	
4	269.1	5361	43682	SS23-HEX	268	1.00	Pass	500.0	[<Confirmed><Edited>]	
5	277.4	1941	16853	SS23-HEX	278	1.00	Pass	206.0	[<Confirmed><Edited>]	

Allele Report

Sample 22: SSS18_SS24_SS05_SS32_SS30_SS12_SS23_HBB31_P08.fsa

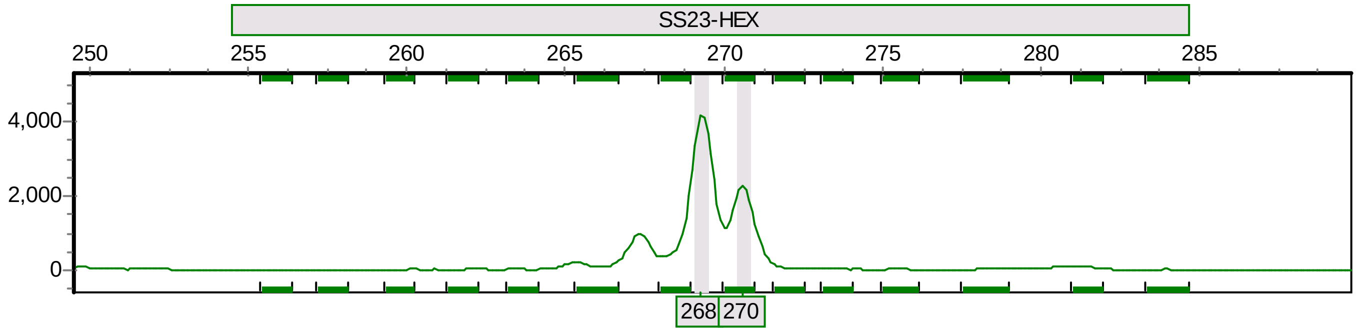

No	Size	Height	Area	Marker	Allele	Difference	Quality	Score	Allele Comments	Sample Comments
1	118.7	15443	95658	SS30-HEX	119	0.10	Pass	500.0	[<Confirmed>]	
2	128.8	9824	61897	SS30-HEX	129	0.00	Pass	500.0	[<Confirmed>]	
3	269.3	4113	33564	SS23-HEX	268	1.00	Pass	500.0	[<Confirmed><Edited>]	
4	270.6	2230	18733	SS23-HEX	270	0.10	Pass	240.9	[<Confirmed>]	

Sample 23: SSS18_SS24_SS05_SS32_SS30_SS12_SS23_HBB32_B16.fsa Run date and time: 10/09/2021 - 21:29:12 -> 10/09/2021 - 21:54:39

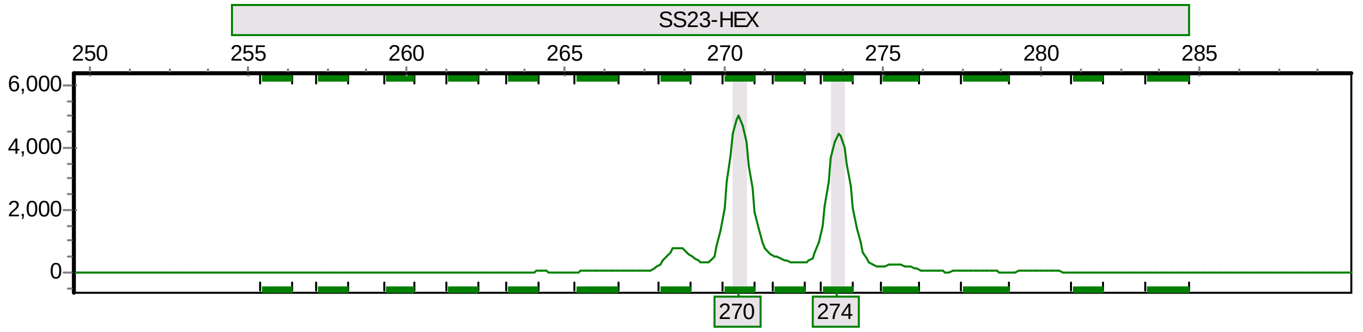

No	Size	Height	Area	Marker	Allele	Difference	Quality	Score	Allele Comments	Sample Comments
1	124.5	15709	103951	SS30-HEX	125	0.20	Pass	500.0	[<Confirmed>]	
2	139.0	8386	60240	SS30-HEX	139	0.10	Pass	500.0	[<Confirmed>]	
3	215.3	1320	10035	SS12-HEX	215	0.10	Pass	153.0	[<Confirmed>]	
4	270.5	5028	43104	SS23-HEX	270	0.00	Pass	500.0	[<Confirmed>]	
5	273.6	4485	38816	SS23-HEX	274	0.00	Pass	500.0	[<Confirmed>]	

Sample 24: SSS18_SS24_SS05_SS32_SS30_SS12_SS23_HBB33_N14.fsa

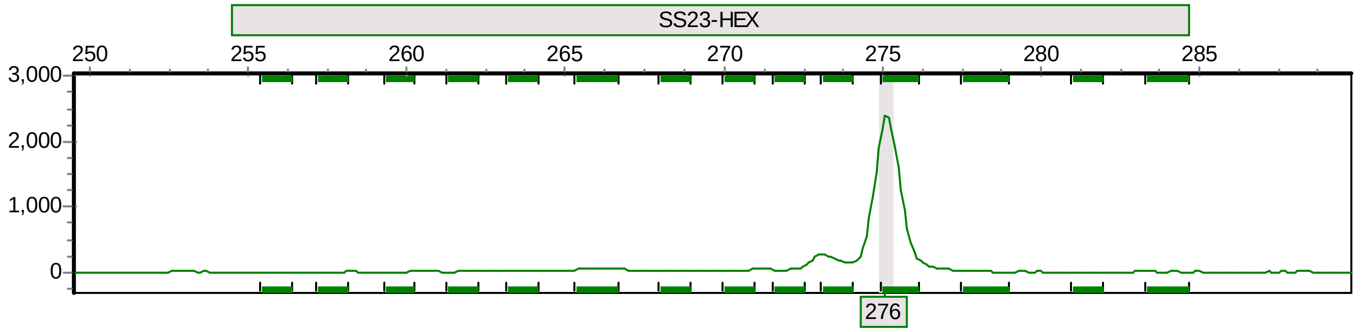

No	Size	Height	Area	Marker	Allele	Difference	Quality	Score	Allele Comments	Sample Comments
1	118.5	25021	161620	SS30-HEX	119	0.10	Pass	500.0	[<Confirmed>]	
2	198.6	526	4124	SS12-HEX	199	0.50	Pass	29.3	[<Confirmed>]	
3	275.1	2383	21709	SS23-HEX	276	0.40	Pass	255.6	[<Confirmed>]	

Allele Report

Sample 25: SSS18_SS24_SS05_SS32_SS30_SS12_SS23_HBB34_P14.fsa

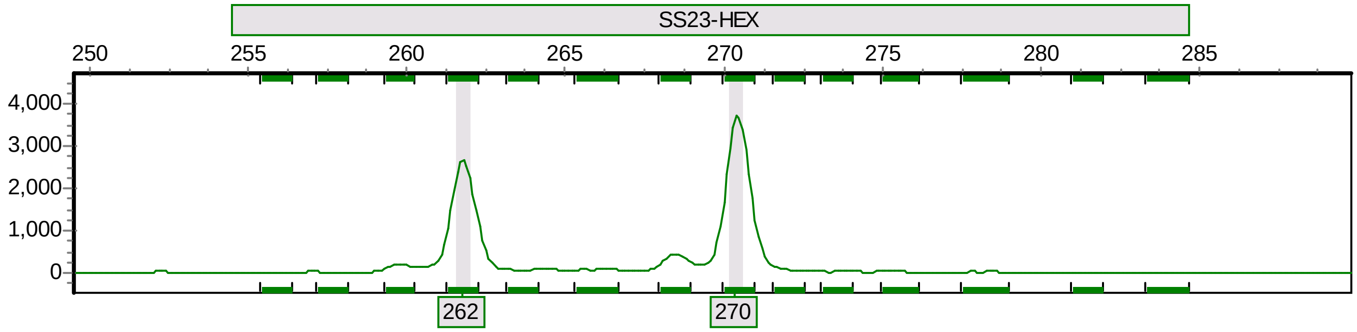

No	Size	Height	Area	Marker	Allele	Difference	Quality	Score	Allele Comments	Sample Comments
1	118.7	31607	204781	SS30-HEX	119	0.10	Pass	500.0	[<Confirmed>]	
2	207.3	546	4274	SS12-HEX	207	0.00	Pass	35.2	[<Confirmed>]	
3	215.3	431	3657	SS12-HEX	215	0.10	Pass	20.7	[<Confirmed>]	
4	261.8	2682	23048	SS23-HEX	262	0.00	Pass	328.0	[<Confirmed>]	
5	270.4	3706	31582	SS23-HEX	270	0.10	Pass	500.0	[<Confirmed>]	

Sample 26: SSS18_SS24_SS05_SS32_SS30_SS12_SS23_HBB35_C16.fsa

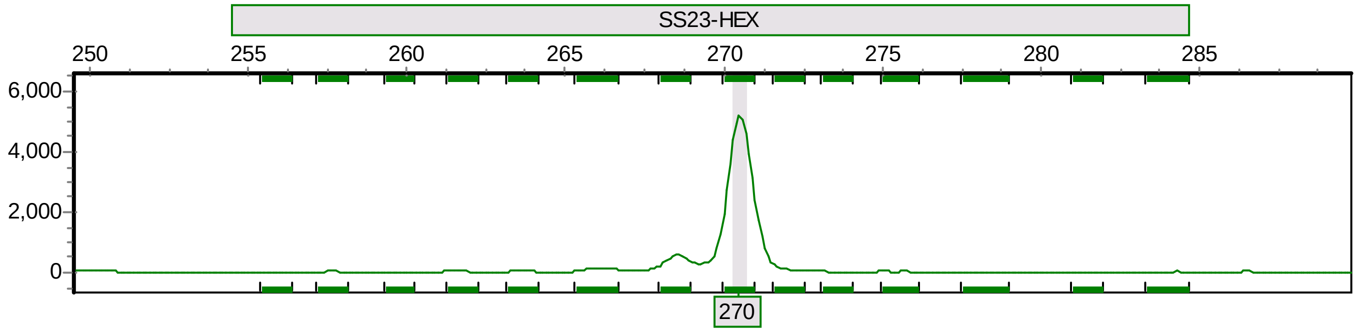

No	Size	Height	Area	Marker	Allele	Difference	Quality	Score	Allele Comments	Sample Comments
1	124.5	11385	75192	SS30-HEX	125	0.20	Pass	500.0	[<Confirmed>]	
2	134.8	7373	48273	SS30-HEX	135	0.10	Pass	500.0	[<Confirmed>]	
3	203.2	3657	27082	SS12-HEX	203	0.10	Pass	500.0	[<Confirmed>]	
4	270.5	5163	45847	SS23-HEX	270	0.00	Pass	500.0	[<Confirmed>]	

Sample 27: SSS18_SS24_SS05_SS32_SS30_SS12_SS23_HBB36_G06.fsa R

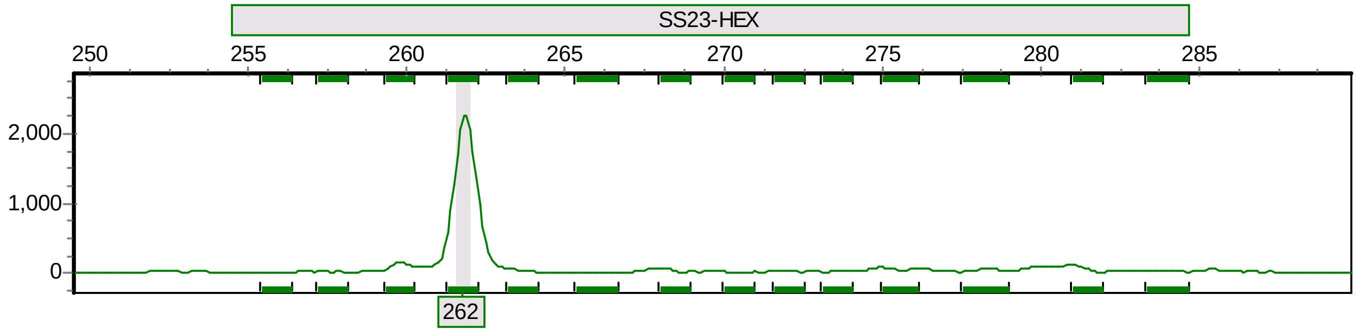

No	Size	Height	Area	Marker	Allele	Difference	Quality	Score	Allele Comments	Sample Comments
1	118.6	16923	106081	SS30-HEX	119	0.00	Pass	500.0	[<Confirmed>]	
2	139.1	6838	48664	SS30-HEX	139	0.00	Pass	500.0	[<Confirmed>]	
3	261.8	2258	17972	SS23-HEX	262	0.00	Pass	310.1	[<Confirmed>]	

Allele Report

Sample 28: SSS18_SS24_SS05_SS32_SS30_SS12_SS23_HBB37_N10.fsa

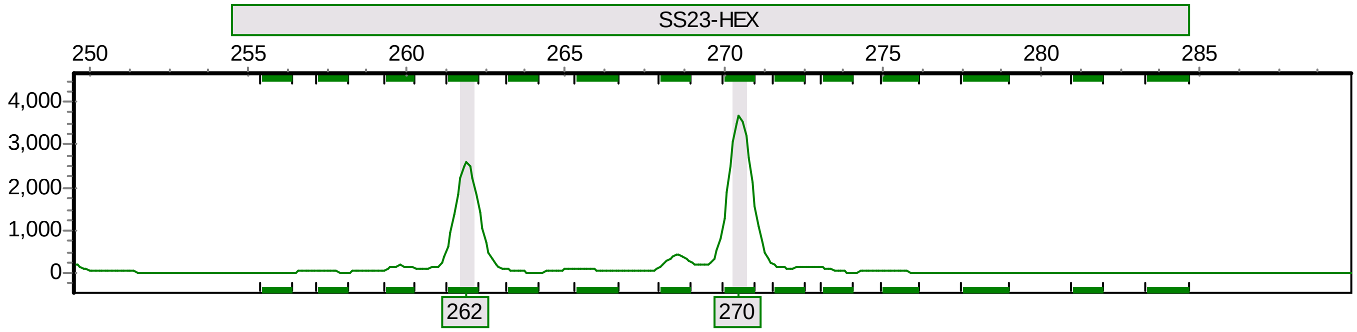

No	Size	Height	Area	Marker	Allele	Difference	Quality	Score	Allele Comments	Sample Comments
1	128.9	9978	64679	SS30-HEX	129	0.10	Pass	500.0	[<Confirmed>]	
2	139.1	6142	43834	SS30-HEX	139	0.00	Pass	500.0	[<Confirmed>]	
3	213.2	4131	31791	SS12-HEX	213	0.10	Pass	500.0	[<Confirmed>]	
4	261.9	2592	21948	SS23-HEX	262	0.10	Pass	326.8	[<Confirmed>]	
5	270.5	3648	31013	SS23-HEX	270	0.00	Pass	500.0	[<Confirmed>]	

Sample 29: SSS18_SS24_SS05_SS32_SS30_SS12_SS23_HBB38_M08.fsa

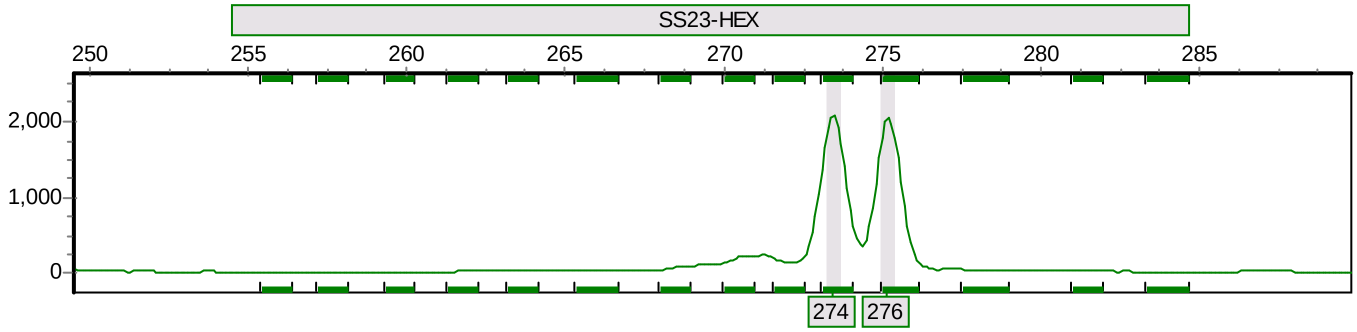

No	Size	Height	Area	Marker	Allele	Difference	Quality	Score	Allele Comments	Sample Comments
1	126.7	19785	127677	SS30-HEX	127	0.00	Pass	500.0	[<Confirmed>]	
2	273.5	2071	19010	SS23-HEX	274	0.10	Pass	197.5	[<Confirmed>]	
3	275.2	2047	18110	SS23-HEX	276	0.30	Pass	202.8	[<Confirmed>]	

Sample 30: SSS18_SS24_SS05_SS32_SS30_SS12_SS23_HBB39_F06.fsa

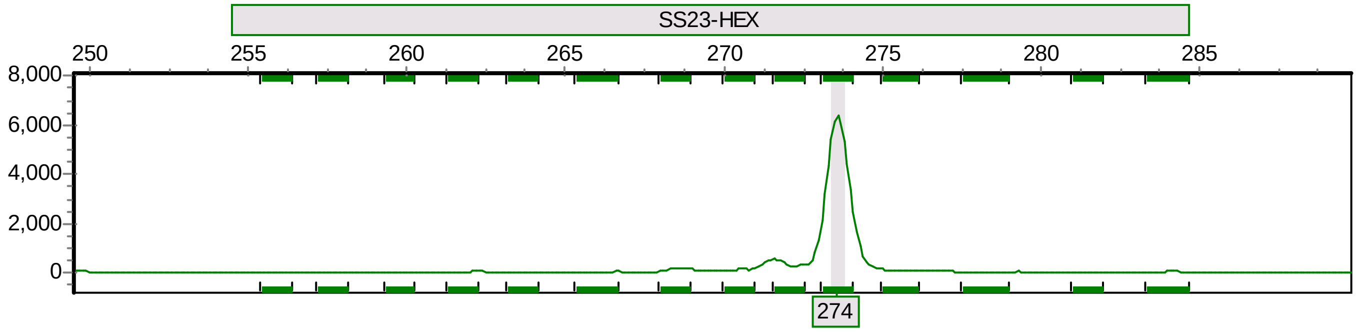

No	Size	Height	Area	Marker	Allele	Difference	Quality	Score	Allele Comments	Sample Comments
1	128.8	25753	163097	SS30-HEX	129	0.00	Pass	500.0	[<Confirmed>]	
2	207.3	984	7021	SS12-HEX	207	0.00	Pass	113.0	[<Confirmed>]	
3	217.3	681	5247	SS12-HEX	217	0.10	Pass	54.3	[<Confirmed>]	
4	273.6	6366	51919	SS23-HEX	274	0.00	Pass	500.0	[<Confirmed>]	

Sample 31: SSS18_SS24_SS05_SS32_SS30_SS12_SS23_HBB40_D16.fsa

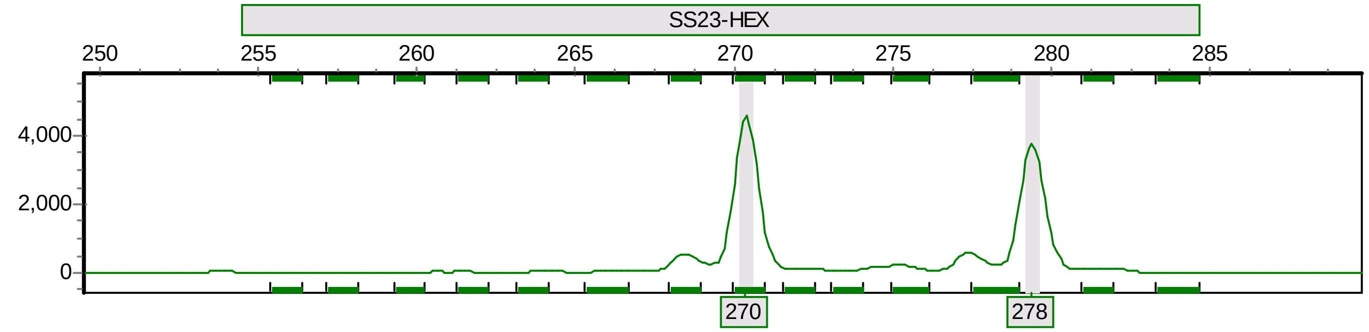

No	Size	Height	Area	Marker	Allele	Difference	Quality	Score	Allele Comments	Sample Comments
1	126.6	17675	117090	SS30-HEX	127	0.10	Pass	500.0	[<Confirmed>]	
2	128.7	12364	82294	SS30-HEX	129	0.10	Pass	500.0	[<Confirmed>]	
3	227.3	3206	24856	SS12-HEX	227	0.00	Pass	500.0	[<Confirmed>]	
4	270.4	4557	38855	SS23-HEX	270	0.10	Pass	500.0	[<Confirmed>]	
5	279.4	3744	32997	SS23-HEX	278	1.00	Pass	499.0	[<Confirmed><Edited>]	

Sample 32: SSS18_SS24_SS05_SS32_SS30_SS12_SS23_HBB41_L08.fsa

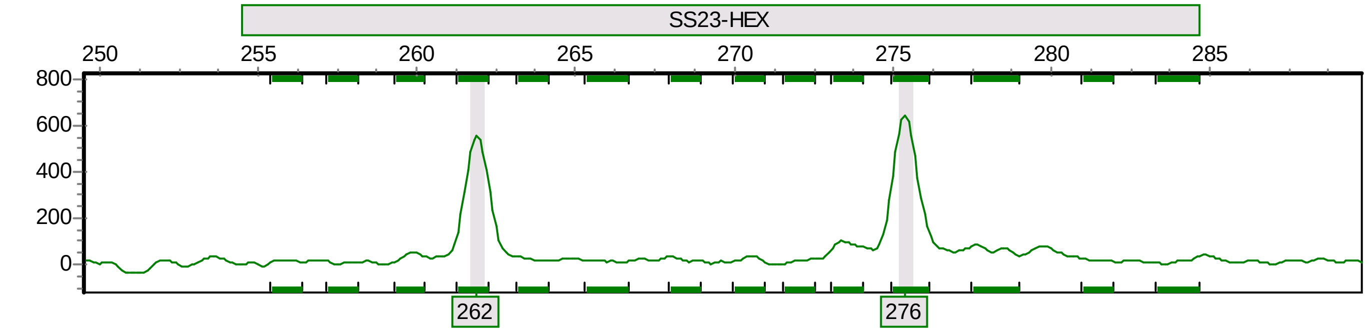

No	Size	Height	Area	Marker	Allele	Difference	Quality	Score	Allele Comments	Sample Comments
1	126.7	10537	67436	SS30-HEX	127	0.00	Pass	500.0	[<Confirmed>]	
2	128.7	7027	45480	SS30-HEX	129	0.10	Pass	500.0	[<Confirmed>]	
3	261.9	551	4787	SS23-HEX	262	0.10	Pass	27.1	[<Confirmed>]	
4	275.4	636	5684	SS23-HEX	276	0.10	Pass	31.4	[<Confirmed>]	

Sample 33: SSS18_SS24_SS05_SS32_SS30_SS12_SS23_HBB42_M06.fsa

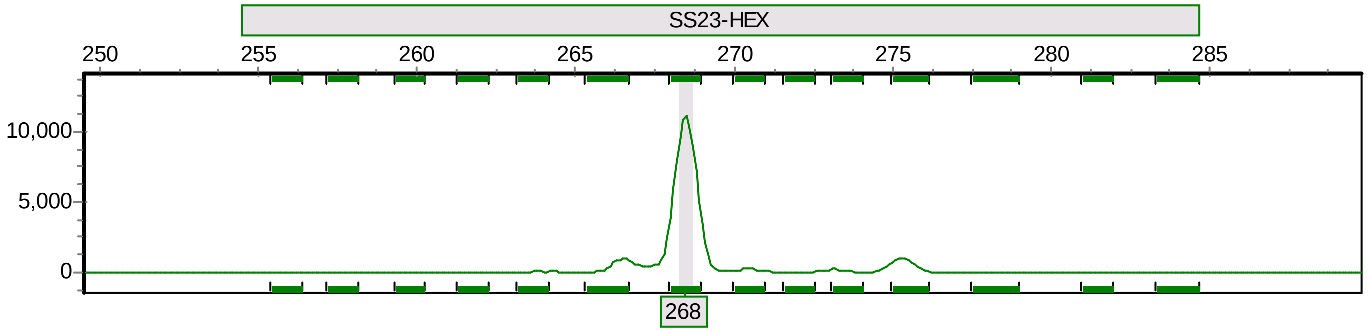

No	Size	Height	Area	Marker	Allele	Difference	Quality	Score	Allele Comments	Sample Comments
1	123.1	17443	120920	SS30-HEX	123	0.50	Pass	500.0	[<Confirmed>]	
2	125.3	11900	82519	SS30-HEX	125	1.00	Pass	500.0	[<Confirmed><Edited>]	
3	213.3	11457	81827	SS12-HEX	213	0.00	Pass	500.0	[<Confirmed>]	
4	268.5	11134	87347	SS23-HEX	268	0.00	Pass	500.0	[<Confirmed>]	

Sample 34: SSS18_SS24_SS05_SS32_SS30_SS12_SS23_HBB43_D10.fsa

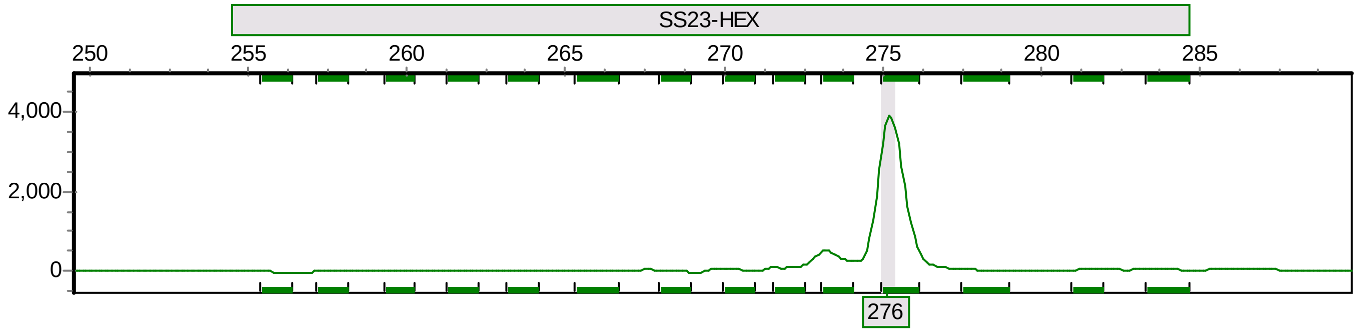

No	Size	Height	Area	Marker	Allele	Difference	Quality	Score	Allele Comments	Sample Comments
1	118.6	17097	112656	SS30-HEX	119	0.00	Pass	500.0	[<Confirmed>]	
2	122.7	10951	73077	SS30-HEX	123	0.10	Pass	500.0	[<Confirmed>]	
3	207.3	800	5929	SS12-HEX	207	0.00	Pass	73.2	[<Confirmed>]	
4	275.2	3883	35907	SS23-HEX	276	0.30	Pass	500.0	[<Confirmed>]	

Sample 35: SSS18_SS24_SS05_SS32_SS30_SS12_SS23_HBB44_H12.fsa

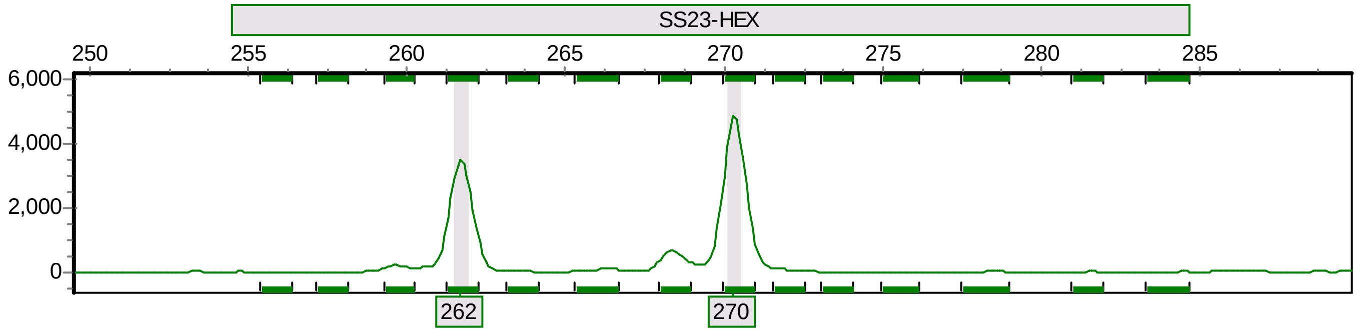

No	Size	Height	Area	Marker	Allele	Difference	Quality	Score	Allele Comments	Sample Comments
1	126.6	12330	80212	SS30-HEX	127	0.10	Pass	500.0	[<Confirmed>]	
2	139.0	7776	55424	SS30-HEX	139	0.10	Pass	500.0	[<Confirmed>]	
3	227.2	3202	24037	SS12-HEX	227	0.10	Pass	500.0	[<Confirmed>]	
4	261.7	3483	28444	SS23-HEX	262	0.10	Pass	500.0	[<Confirmed>]	
5	270.3	4840	38842	SS23-HEX	270	0.20	Pass	500.0	[<Confirmed>]	

Sample 36: SSS18_SS24_SS05_SS32_SS30_SS12_SS23_HBB45_F14.fsa

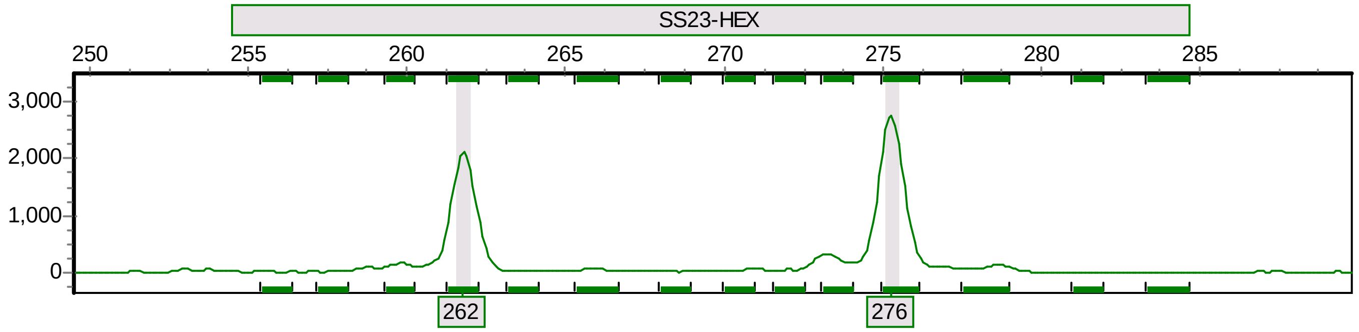

No	Size	Height	Area	Marker	Allele	Difference	Quality	Score	Allele Comments	Sample Comments
1	118.6	14006	93150	SS30-HEX	119	0.00	Pass	500.0	[<Confirmed>]	
2	128.7	10232	68464	SS30-HEX	129	0.10	Pass	500.0	[<Confirmed>]	
3	227.3	2102	17235	SS12-HEX	227	0.00	Pass	256.8	[<Confirmed>]	
4	261.8	2116	18350	SS23-HEX	262	0.00	Pass	226.5	[<Confirmed>]	
5	275.3	2739	24699	SS23-HEX	276	0.20	Pass	309.2	[<Confirmed>]	

Allele Report

Sample 37: SSS18_SS24_SS05_SS32_SS30_SS12_SS23_HBB46_F08.fsa

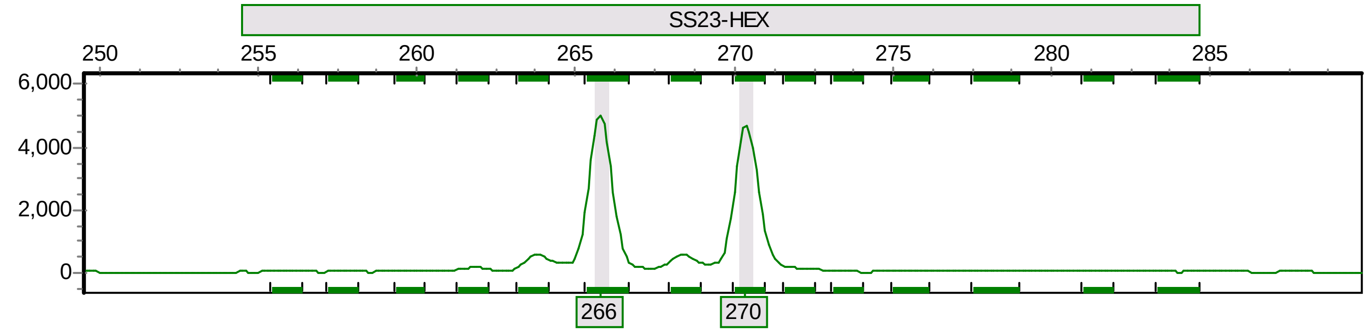

No	Size	Height	Area	Marker	Allele	Difference	Quality	Score	Allele Comments	Sample Comments
1	118.5	20813	132132	SS30-HEX	119	0.10	Pass	500.0	[<Confirmed>]	
2	126.7	13164	85595	SS30-HEX	127	0.00	Pass	500.0	[<Confirmed>]	
3	207.3	1151	8543	SS12-HEX	207	0.00	Pass	129.5	[<Confirmed>]	
4	265.8	4975	41160	SS23-HEX	266	0.20	Pass	500.0	[<Confirmed>]	
5	270.4	4704	39808	SS23-HEX	270	0.10	Pass	500.0	[<Confirmed>]	

Sample 38: SSS18_SS24_SS05_SS32_SS30_SS12_SS23_HBB47_A06.fsa

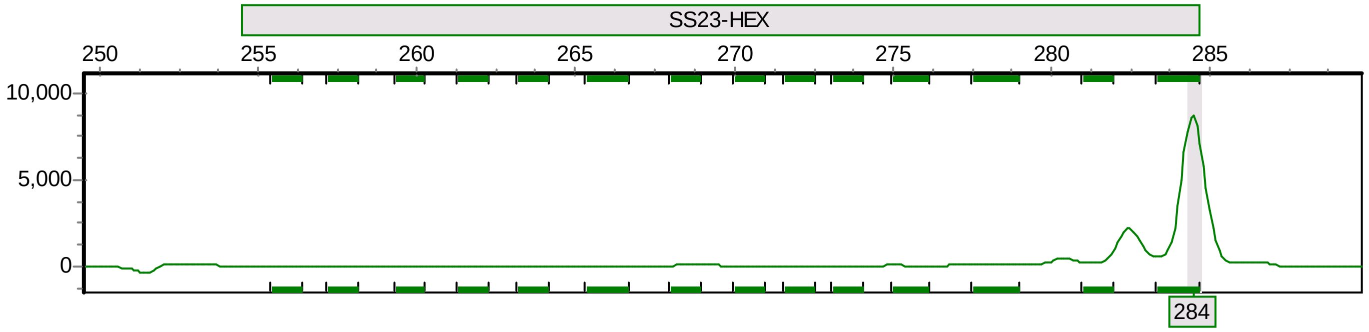

No	Size	Height	Area	Marker	Allele	Difference	Quality	Score	Allele Comments	Sample Comments
1	126.8	15256	95507	SS30-HEX	127	0.10	Pass	500.0	[<Confirmed>]	
2	146.1	6957	48850	SS30-HEX	145	0.10	Pass	500.0	[<Confirmed>]	
3	217.5	12668	93082	SS12-HEX	217	0.10	Pass	500.0	[<Confirmed>]	
4	284.5	8634	73582	SS23-HEX	284	0.50	Pass	500.0	[<Confirmed>]	

Sample 39: SSS18_SS24_SS05_SS32_SS30_SS12_SS23_HBB48_B14.fsa

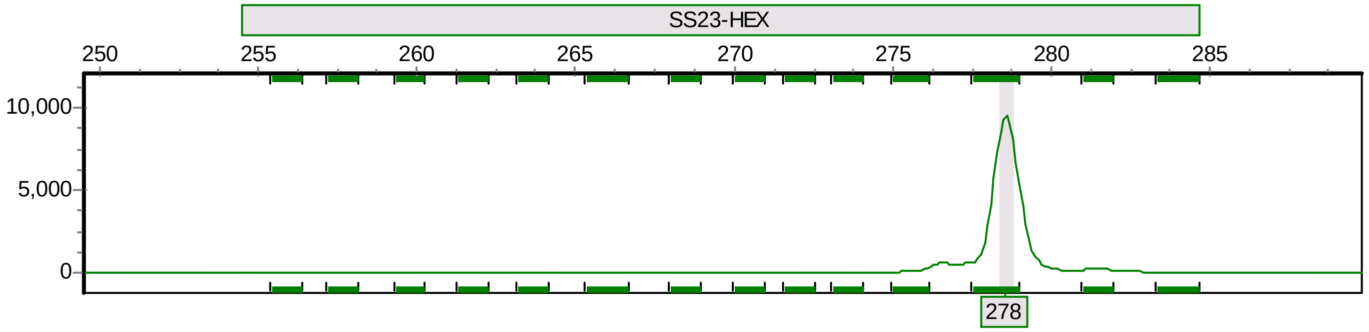

No	Size	Height	Area	Marker	Allele	Difference	Quality	Score	Allele Comments	Sample Comments
1	122.7	11496	74567	SS30-HEX	123	0.10	Pass	500.0	[<Confirmed>]	
2	132.9	7995	53130	SS30-HEX	133	0.00	Pass	500.0	[<Confirmed>]	
3	217.3	13098	98279	SS12-HEX	217	0.10	Pass	500.0	[<Confirmed>]	
4	278.6	9472	84151	SS23-HEX	278	0.40	Pass	500.0	[<Confirmed>]	

Sample 40: SSS18_SS24_SS05_SS32_SS30_SS12_SS23_HBB49_L06.fsa

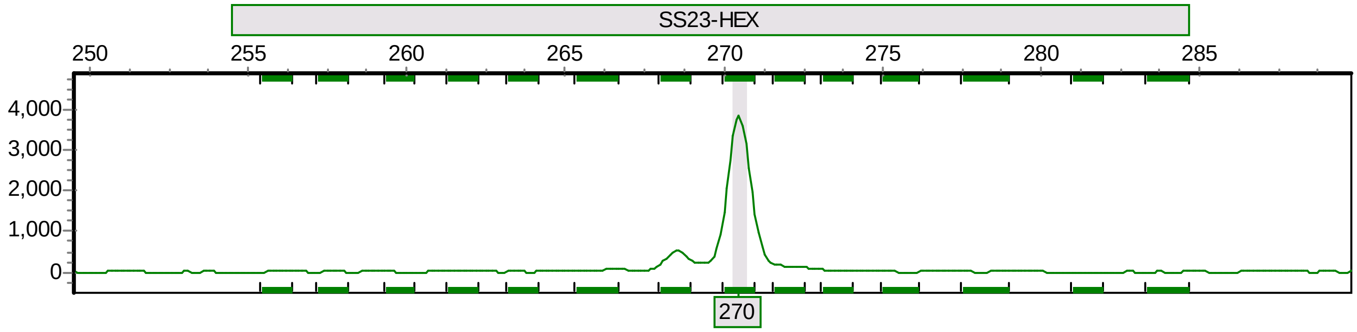

No	Size	Height	Area	Marker	Allele	Difference	Quality	Score	Allele Comments	Sample Comments
1	120.7	18028	111508	SS30-HEX	121	0.10	Pass	500.0	[<Confirmed>]	
2	128.7	11870	75280	SS30-HEX	129	0.10	Pass	500.0	[<Confirmed>]	
3	213.3	4799	35442	SS12-HEX	213	0.00	Pass	500.0	[<Confirmed>]	
4	270.5	3820	31649	SS23-HEX	270	0.00	Pass	500.0	[<Confirmed>]	

Sample 41: SSS18_SS24_SS05_SS32_SS30_SS12_SS23_HBB4_E08.fsa

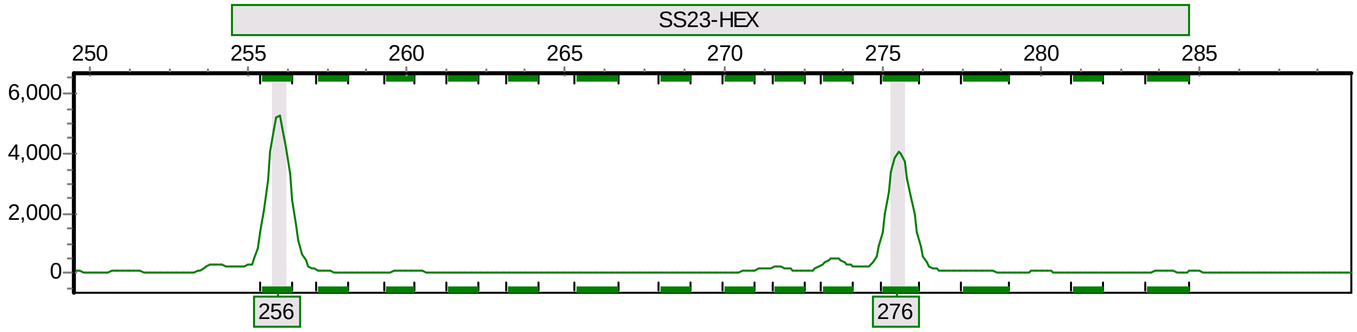

No	Size	Height	Area	Marker	Allele	Difference	Quality	Score	Allele Comments	Sample Comments
1	120.7	19732	124518	SS30-HEX	121	0.10	Pass	500.0	[<Confirmed>]	
2	134.9	9518	61990	SS30-HEX	135	0.00	Pass	500.0	[<Confirmed>]	
3	229.3	3202	24409	SS12-HEX	229	0.00	Pass	500.0	[<Confirmed>]	
4	256.0	5246	42178	SS23-HEX	256	0.10	Pass	500.0	[<Confirmed>]	
5	275.5	4085	35433	SS23-HEX	276	0.00	Pass	500.0	[<Confirmed>]	

Sample 42: SSS18_SS24_SS05_SS32_SS30_SS12_SS23_HBB5_H14.fsa

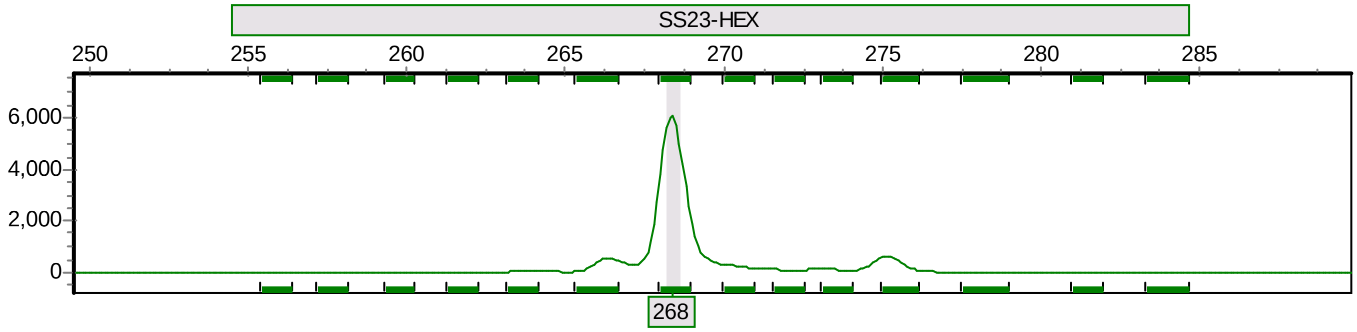

No	Size	Height	Area	Marker	Allele	Difference	Quality	Score	Allele Comments	Sample Comments
1	118.5	11517	79157	SS30-HEX	119	0.10	Pass	500.0	[<Confirmed>]	
2	128.6	7525	53653	SS30-HEX	129	0.20	Pass	500.0	[<Confirmed>]	
3	268.4	6028	53842	SS23-HEX	268	0.10	Pass	500.0	[<Confirmed>]	

Sample 43: SSS18_SS24_SS05_SS32_SS30_SS12_SS23_HBB6_C06.fsa

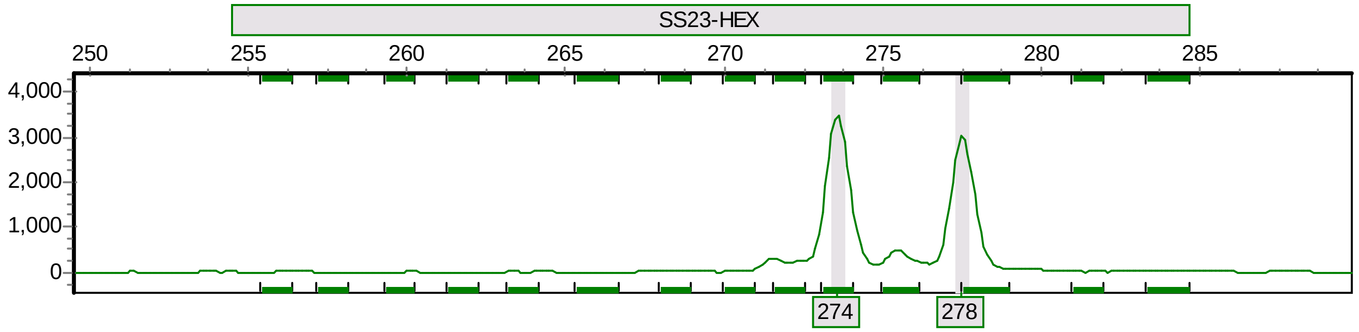

No	Size	Height	Area	Marker	Allele	Difference	Quality	Score	Allele Comments	Sample Comments
1	126.6	19631	124002	SS30-HEX	127	0.10	Pass	500.0	[<Confirmed>]	
2	217.5	13993	99882	SS12-HEX	217	0.10	Pass	500.0	[<Confirmed>]	
3	273.6	3457	29266	SS23-HEX	274	0.00	Pass	485.1	[<Confirmed>]	
4	277.5	3006	25032	SS23-HEX	278	0.70	Pass	414.9	[<Confirmed>]	

Sample 44: SSS18_SS24_SS05_SS32_SS30_SS12_SS23_HBB7_N06.fsa

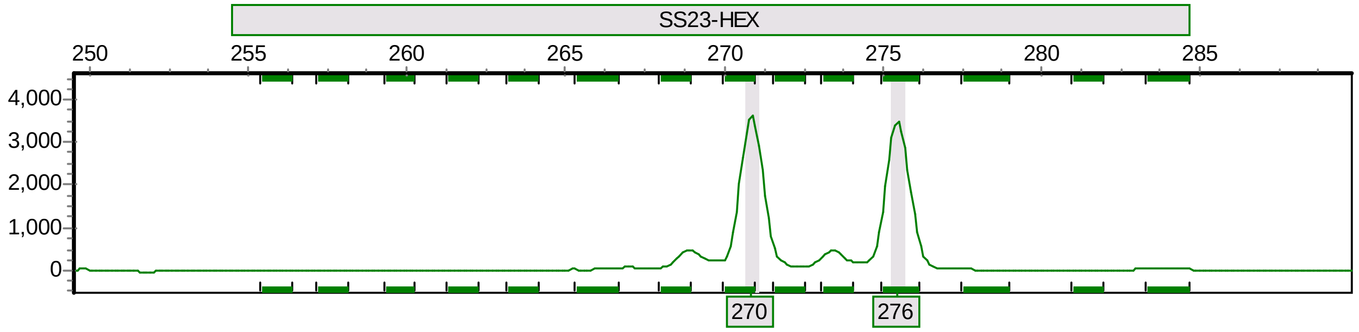

No	Size	Height	Area	Marker	Allele	Difference	Quality	Score	Allele Comments	Sample Comments
1	120.9	19019	116295	SS30-HEX	121	0.30	Pass	500.0	[<Confirmed>]	
2	128.9	11151	70330	SS30-HEX	129	0.10	Pass	500.0	[<Confirmed>]	
3	221.3	3732	27280	SS12-HEX	221	0.10	Pass	500.0	[<Confirmed>]	
4	270.9	3585	29500	SS23-HEX	270	0.40	Pass	500.0	[<Confirmed>]	
5	275.5	3452	29213	SS23-HEX	276	0.00	Pass	481.7	[<Confirmed>]	

Sample 45: SSS18_SS24_SS05_SS32_SS30_SS12_SS23_HBB8_P12.fsa

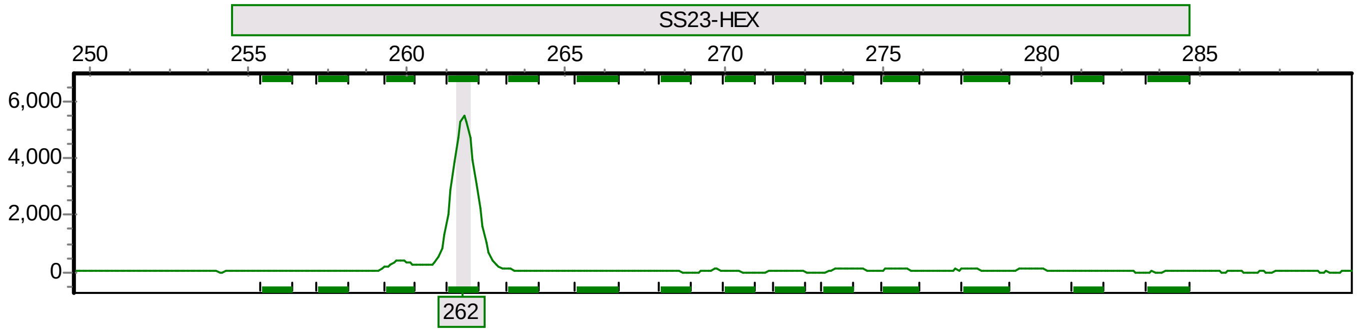

No	Size	Height	Area	Marker	Allele	Difference	Quality	Score	Allele Comments	Sample Comments
1	118.7	16968	107085	SS30-HEX	119	0.10	Pass	500.0	[<Confirmed>]	
2	137.1	7211	47950	SS30-HEX	137	0.10	Pass	500.0	[<Confirmed>]	
3	217.3	9794	75483	SS12-HEX	217	0.10	Pass	500.0	[<Confirmed>]	
4	261.8	5458	46377	SS23-HEX	262	0.00	Pass	500.0	[<Confirmed>]	

Allele Report

Sample 46: SSS18_SS24_SS05_SS32_SS30_SS12_SS23_HBB9_I06.fsa

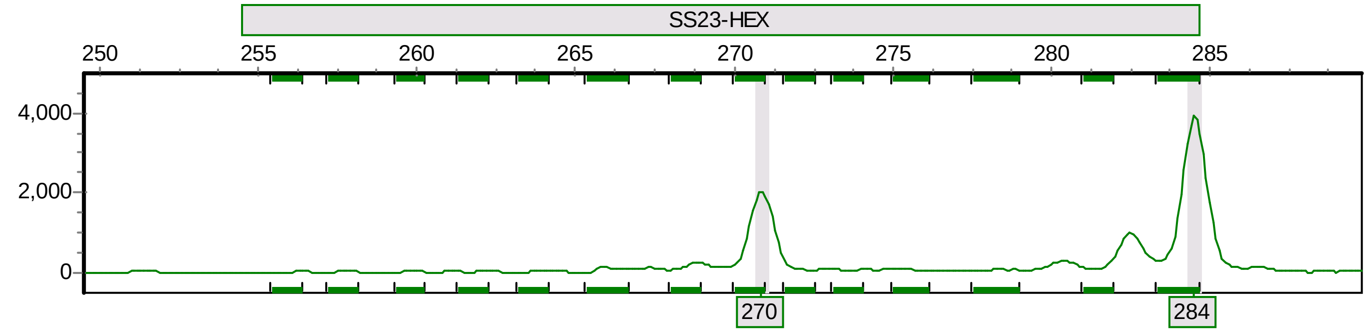

No	Size	Height	Area	Marker	Allele	Difference	Quality	Score	Allele Comments	Sample Comments
1	118.6	16428	104058	SS30-HEX	119	0.00	Pass	500.0	[<Confirmed>]	
2	141.5	5883	42998	SS30-HEX	141	0.10	Pass	500.0	[<Confirmed>]	
3	217.5	14671	109112	SS12-HEX	217	0.10	Pass	500.0	[<Confirmed>]	
4	270.9	2042	17254	SS23-HEX	270	0.40	Pass	227.5	[<Confirmed>]	
5	284.5	3936	33914	SS23-HEX	284	0.50	Pass	500.0	[<Confirmed>]	

Sample 47: SSS18_SS24_SS05_SS32_SS30_SS12_SS23_HBN10_G12.fsa

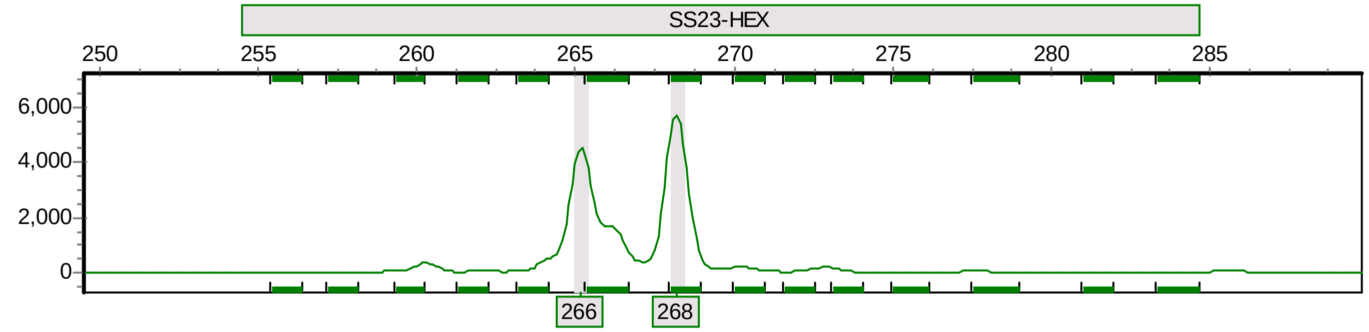

No	Size	Height	Area	Marker	Allele	Difference	Quality	Score	Allele Comments	Sample Comments
1	128.7	9587	61295	SS30-HEX	129	0.10	Pass	500.0	[<Confirmed>]	
2	141.4	5661	43313	SS30-HEX	141	0.00	Pass	500.0	[<Confirmed>]	
3	205.4	11564	86019	SS12-HEX	205	0.00	Pass	500.0	[<Confirmed>]	
4	207.3	7345	53334	SS12-HEX	207	0.00	Pass	500.0	[<Confirmed>]	
5	265.2	4517	37678	SS23-HEX	266	1.00	Pass	500.0	[<Confirmed><Edited>]	
6	268.2	5661	46447	SS23-HEX	268	0.30	Pass	500.0	[<Confirmed>]	

Sample 48: SSS18_SS24_SS05_SS32_SS30_SS12_SS23_HBN6_O02.fsa

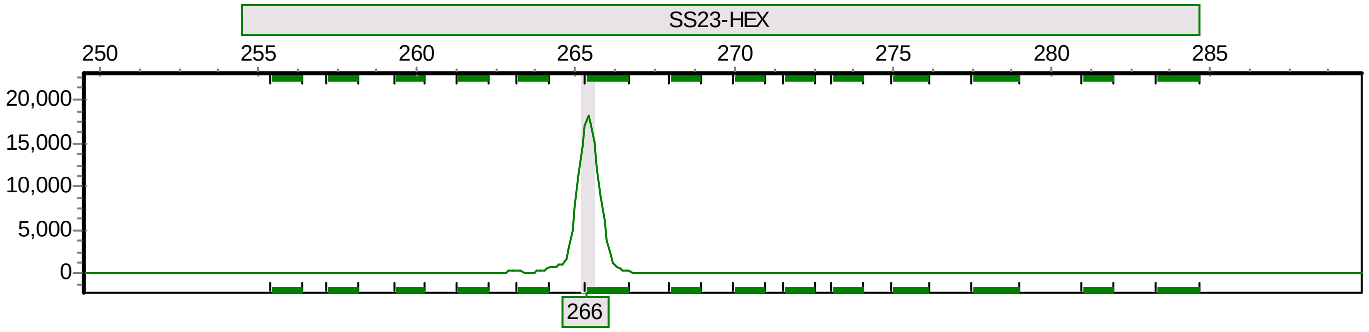

No	Size	Height	Area	Marker	Allele	Difference	Quality	Score	Allele Comments	Sample Comments
1	129.1	14316	85820	SS30-HEX	129	0.30	Pass	500.0	[<Confirmed>]	
2	135.3	10665	64507	SS30-HEX	135	0.40	Pass	500.0	[<Confirmed>]	
3	215.5	14040	95995	SS12-HEX	215	0.10	Pass	500.0	[<Confirmed>]	
4	265.4	18126	136208	SS23-HEX	266	0.60	Pass	500.0	[<Confirmed>]	

Allele Report

Sample 49: SSS18_SS24_SS05_SS32_SS30_SS12_SS23_HBN9_D04.fsa

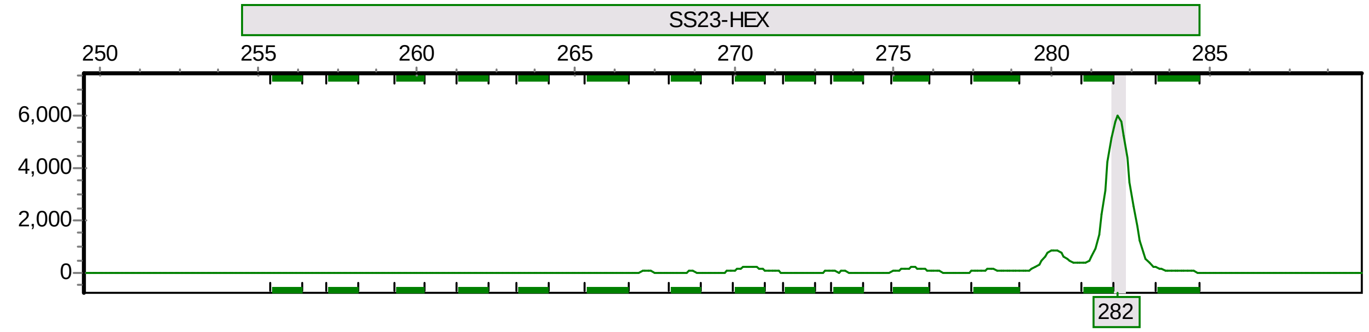

No	Size	Height	Area	Marker	Allele	Difference	Quality	Score	Allele Comments	Sample Comments
1	128.8	9755	62379	SS30-HEX	129	0.00	Pass	500.0	[<Confirmed>]	
2	209.6	7326	52011	SS12-HEX	209	0.20	Pass	500.0	[<Confirmed>]	
3	217.6	7509	55501	SS12-HEX	217	0.20	Pass	500.0	[<Confirmed>]	
4	282.1	6002	52040	SS23-HEX	282	1.00	Pass	500.0	[<Confirmed><Edited>]	

Sample 50: SSS18_SS24_SS05_SS32_SS30_SS12_SS23_HCW1_M10.fsa

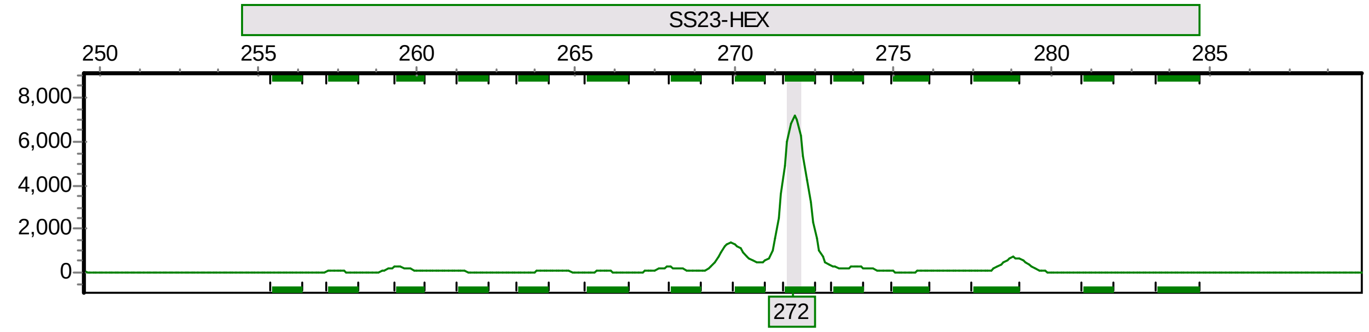

No	Size	Height	Area	Marker	Allele	Difference	Quality	Score	Allele Comments	Sample Comments
1	134.9	7559	50438	SS30-HEX	135	0.00	Pass	500.0	[<Confirmed>]	
2	143.6	5452	40029	SS30-HEX	143	0.10	Pass	500.0	[<Confirmed>]	
3	217.3	12993	98921	SS12-HEX	217	0.10	Pass	500.0	[<Confirmed>]	
4	271.9	7130	61830	SS23-HEX	272	0.20	Pass	500.0	[<Confirmed>]	

Sample 51: SSS18_SS24_SS05_SS32_SS30_SS12_SS23_HCW2_A12.fsa

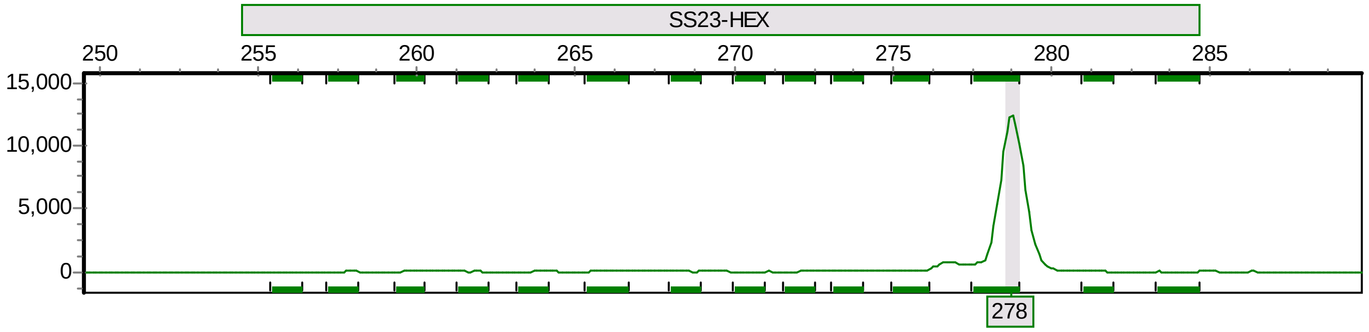

No	Size	Height	Area	Marker	Allele	Difference	Quality	Score	Allele Comments	Sample Comments
1	135.0	10559	69009	SS30-HEX	135	0.10	Pass	500.0	[<Confirmed>]	
2	141.4	7017	50599	SS30-HEX	141	0.00	Pass	500.0	[<Confirmed>]	
3	198.2	12250	55982	SS12-HEX	199	1.00	Pass	500.0	[<Confirmed><Edited>]	
4	217.4	16331	122256	SS12-HEX	217	0.00	Pass	500.0	[<Confirmed>]	
5	278.8	12315	107386	SS23-HEX	278	0.60	Pass	500.0	[<Confirmed>]	

Allele Report

Sample 52: SSS18_SS24_SS05_SS32_SS30_SS12_SS23_HCW3_G14.fsa

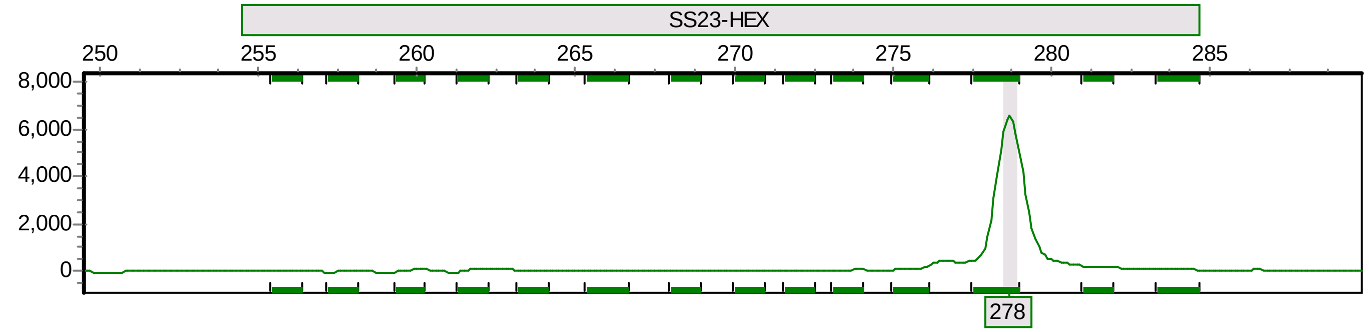

No	Size	Height	Area	Marker	Allele	Difference	Quality	Score	Allele Comments	Sample Comments
1	134.8	6853	49455	SS30-HEX	135	0.10	Pass	500.0	[<Confirmed>]	
2	141.3	4961	39427	SS30-HEX	141	0.10	Pass	500.0	[<Confirmed>]	
3	217.4	7168	58228	SS12-HEX	217	0.00	Pass	500.0	[<Confirmed>]	
4	278.7	6533	63225	SS23-HEX	278	0.50	Pass	500.0	[<Confirmed>]	

Sample 53: SSS18_SS24_SS05_SS32_SS30_SS12_SS23_HCW4_E10.fsa

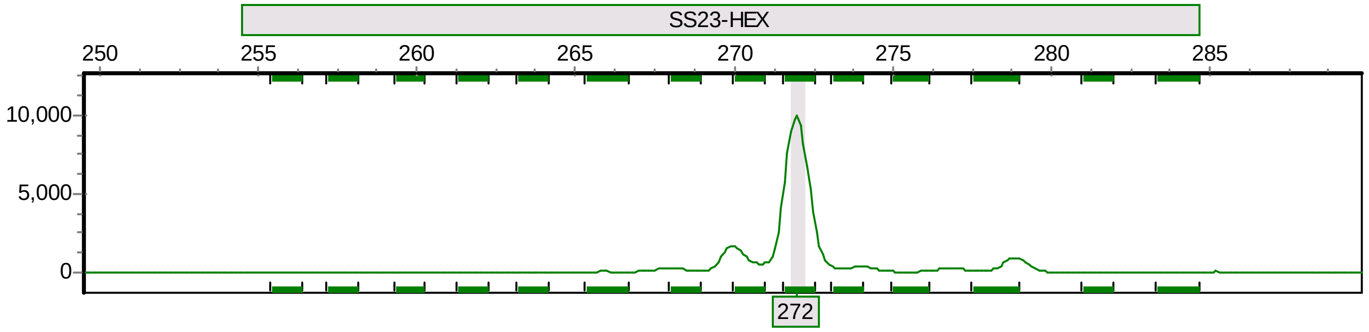

No	Size	Height	Area	Marker	Allele	Difference	Quality	Score	Allele Comments	Sample Comments
1	141.2	9631	73354	SS30-HEX	141	0.20	Pass	500.0	[<Confirmed>]	
2	143.6	5560	41731	SS30-HEX	143	0.10	Pass	500.0	[<Confirmed>]	
3	217.2	6002	45906	SS12-HEX	217	0.20	Pass	500.0	[<Confirmed>]	
4	272.0	9973	85599	SS23-HEX	272	0.10	Pass	500.0	[<Confirmed>]	

Sample 54: SSS18_SS24_SS05_SS32_SS30_SS12_SS23_HCW5_C12.fsa

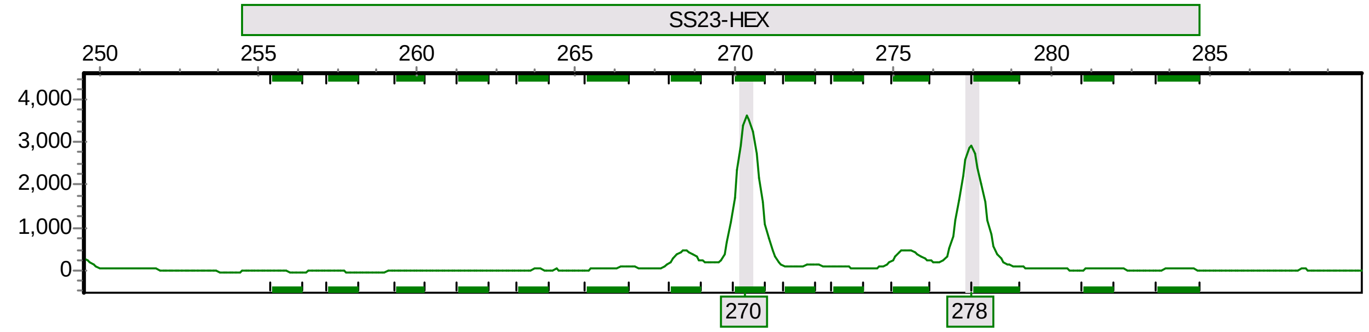

No	Size	Height	Area	Marker	Allele	Difference	Quality	Score	Allele Comments	Sample Comments
1	122.4	14004	89935	SS30-HEX	123	0.20	Pass	500.0	[<Confirmed>]	
2	134.8	8501	56042	SS30-HEX	135	0.10	Pass	500.0	[<Confirmed>]	
3	229.3	2556	19784	SS12-HEX	229	0.00	Pass	384.9	[<Confirmed>]	
4	270.4	3593	30062	SS23-HEX	270	0.10	Pass	500.0	[<Confirmed>]	
5	277.5	2897	25443	SS23-HEX	278	0.70	Pass	354.0	[<Confirmed>]	

Allele Report

Sample 55: SSS18_SS24_SS05_SS32_SS30_SS12_SS23_HCW6_D02.fsa

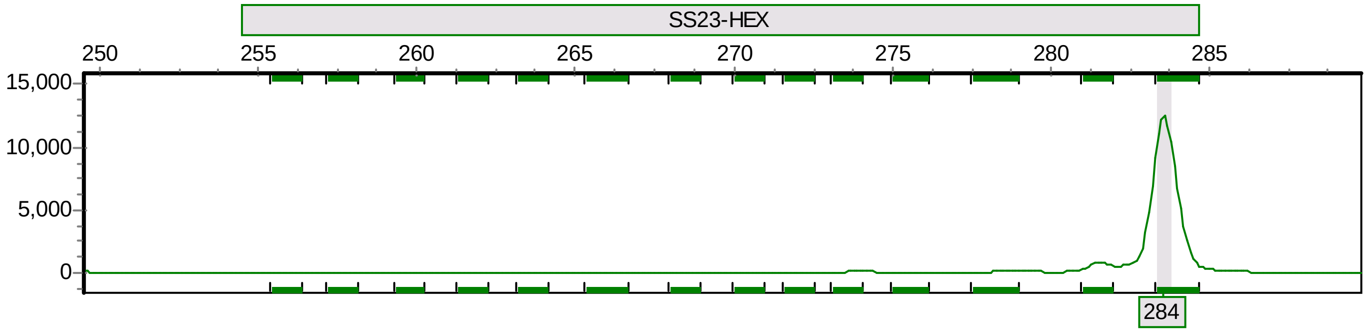

No	Size	Height	Area	Marker	Allele	Difference	Quality	Score	Allele Comments	Sample Comments
1	128.9	25391	161431	SS30-HEX	129	0.10	Pass	500.0	[<Confirmed>]	
2	203.3	9396	65165	SS12-HEX	203	0.00	Pass	500.0	[<Confirmed>]	
3	283.6	12437	107007	SS23-HEX	284	0.40	Pass	500.0	[<Confirmed>]	

Sample 56: SSS18_SS24_SS05_SS32_SS30_SS12_SS23_HCW7_G18.fsa

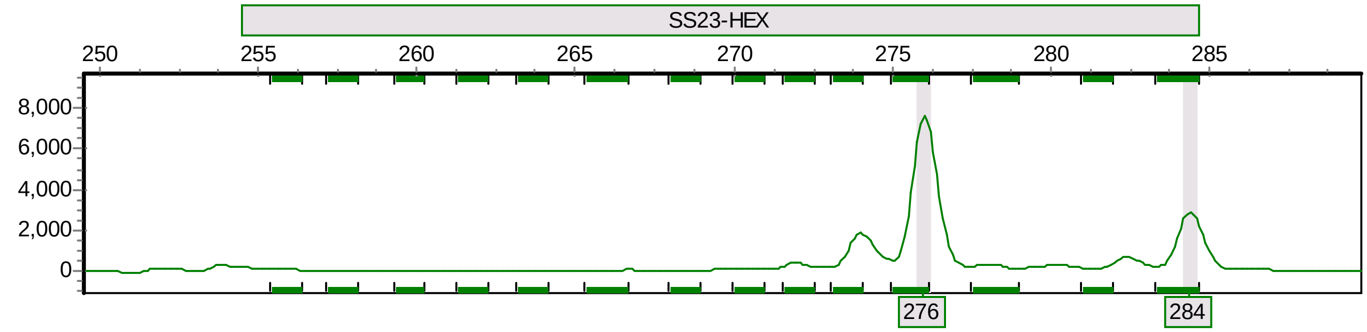

No	Size	Height	Area	Marker	Allele	Difference	Quality	Score	Allele Comments	Sample Comments
1	124.6	26157	168152	SS30-HEX	125	0.10	Pass	500.0	[<Confirmed>]	
2	213.4	8436	64047	SS12-HEX	213	0.10	Pass	500.0	[<Confirmed>]	
3	217.3	9298	72566	SS12-HEX	217	0.10	Pass	500.0	[<Confirmed>]	
4	276.0	7595	66826	SS23-HEX	276	0.50	Pass	500.0	[<Confirmed>]	
5	284.4	2896	26235	SS23-HEX	284	0.40	Pass	329.5	[<Confirmed>]	

Sample 57: SSS18_SS24_SS05_SS32_SS30_SS12_SS23_HCW8_A18.fsa

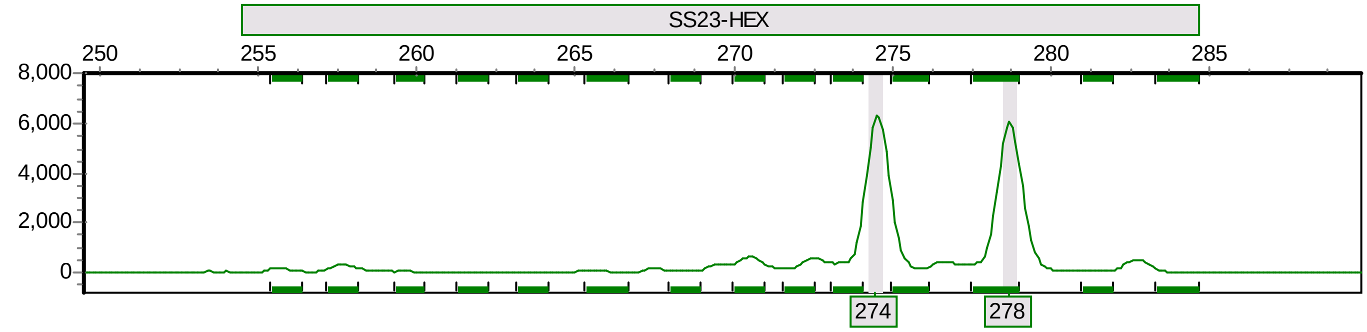

No	Size	Height	Area	Marker	Allele	Difference	Quality	Score	Allele Comments	Sample Comments
1	128.5	10706	70470	SS30-HEX	129	0.30	Pass	500.0	[<Confirmed>]	
2	143.5	5371	39642	SS30-HEX	143	0.20	Pass	500.0	[<Confirmed>]	
3	199.2	11249	85045	SS12-HEX	199	0.10	Pass	500.0	[<Confirmed>]	
4	207.3	9126	67047	SS12-HEX	207	0.00	Pass	500.0	[<Confirmed>]	
5	274.5	6275	52994	SS23-HEX	274	1.00	Pass	500.0	[<Confirmed><Edited>]	
6	278.7	6027	52790	SS23-HEX	278	0.50	Pass	500.0	[<Confirmed>]	

Allele Report

Sample 58: SSS18_SS24_SS05_SS32_SS30_SS12_SS23_HGC1_C02.fsa

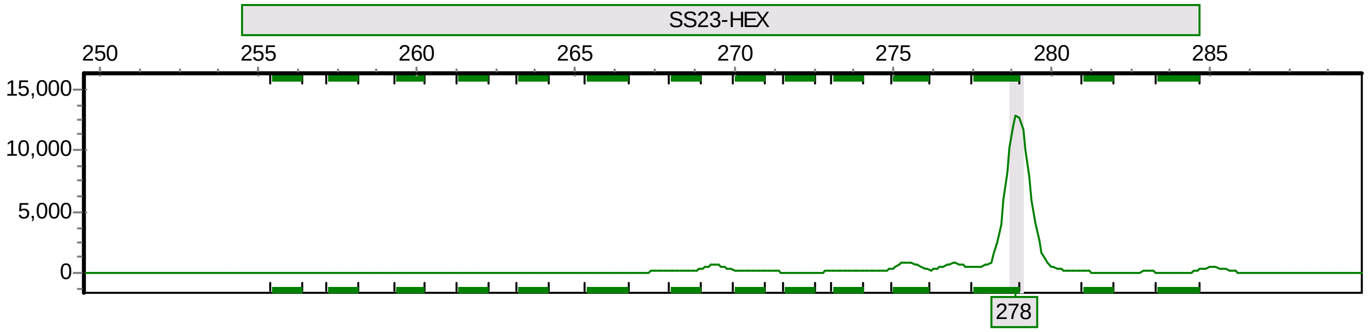

No	Size	Height	Area	Marker	Allele	Difference	Quality	Score	Allele Comments	Sample Comments
1	137.1	15282	95378	SS30-HEX	137	0.10	Pass	500.0	[<Confirmed>]	
2	139.2	9581	66857	SS30-HEX	139	0.10	Pass	500.0	[<Confirmed>]	
3	219.6	9346	66759	SS12-HEX	219	0.20	Pass	500.0	[<Confirmed>]	
4	223.6	4480	32967	SS12-HEX	223	0.20	Pass	500.0	[<Confirmed>]	
5	278.9	12818	108088	SS23-HEX	278	0.70	Pass	500.0	[<Confirmed>]	

Sample 59: SSS18_SS24_SS05_SS32_SS30_SS12_SS23_HGC3_E18.fsa

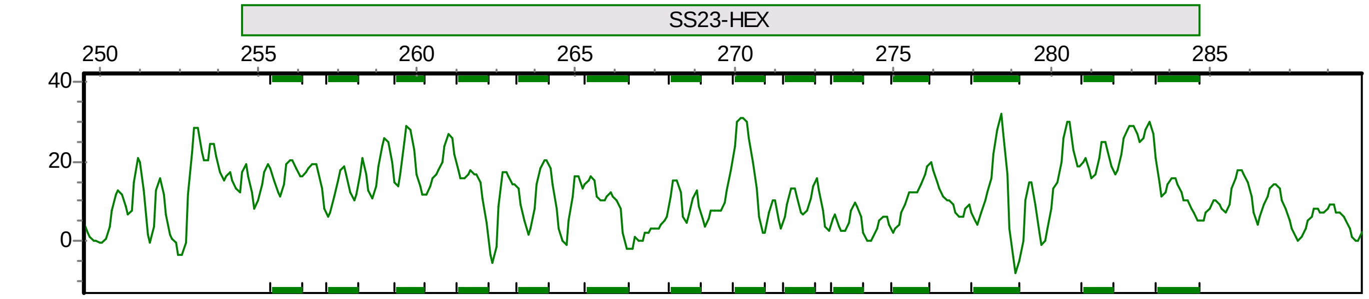

No	Size	Height	Area	Marker	Allele	Difference	Quality	Score	Allele Comments	Sample Comments

Sample 60: SSS18_SS24_SS05_SS32_SS30_SS12_SS23_HGC4_A14.fsa

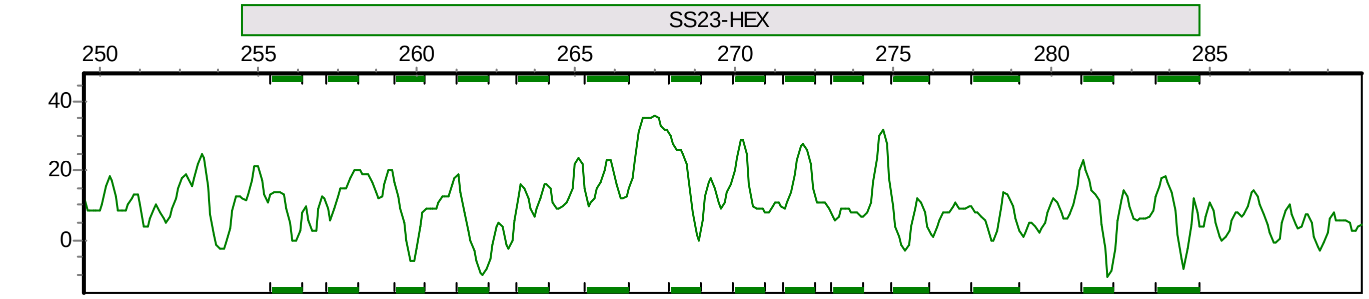

No	Size	Height	Area	Marker	Allele	Difference	Quality	Score	Allele Comments	Sample Comments

Sample 61: SSS18_SS24_SS05_SS32_SS30_SS12_SS23_HGY1_A10.fsa

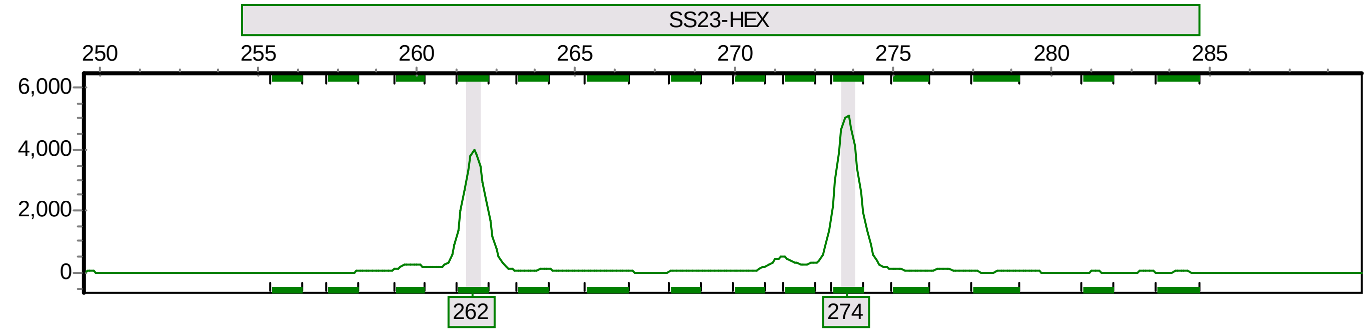

No	Size	Height	Area	Marker	Allele	Difference	Quality	Score	Allele Comments	Sample Comments
1	118.7	32248	224640	SS30-HEX	119	0.10	Pass	500.0	[<Confirmed>]	
2	207.3	1746	13416	SS12-HEX	207	0.00	Pass	226.4	[<Confirmed>]	
3	261.8	3980	33822	SS23-HEX	262	0.00	Pass	500.0	[<Confirmed>]	
4	273.6	5081	43928	SS23-HEX	274	0.00	Pass	500.0	[<Confirmed>]	

Sample 62: SSS18_SS24_SS05_SS32_SS30_SS12_SS23_HGY2_I14.fsa

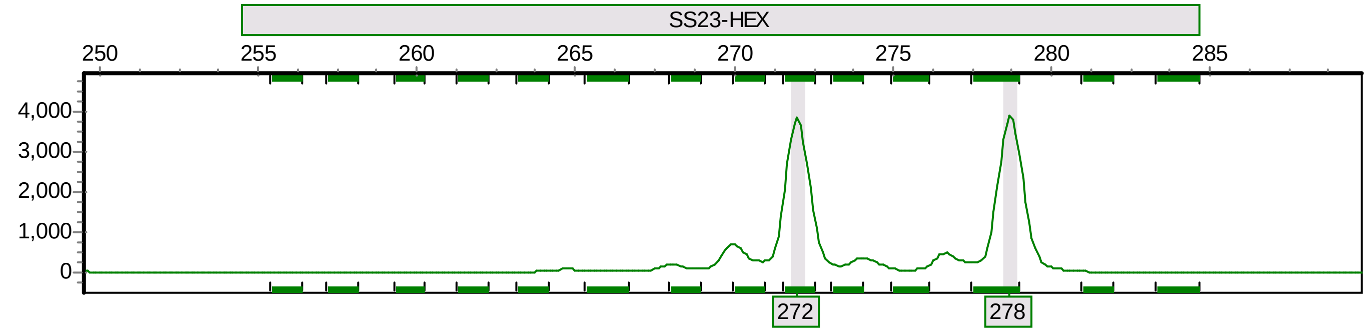

No	Size	Height	Area	Marker	Allele	Difference	Quality	Score	Allele Comments	Sample Comments
1	141.3	5163	40327	SS30-HEX	141	0.10	Pass	500.0	[<Confirmed>]	
2	158.2	1670	10771	SS30-HEX	159	0.10	Pass	295.6	[<Confirmed>]	
3	217.4	7381	56005	SS12-HEX	217	0.00	Pass	500.0	[<Confirmed>]	
4	272.0	3848	32768	SS23-HEX	272	0.10	Pass	500.0	[<Confirmed>]	
5	278.7	3893	34840	SS23-HEX	278	0.50	Pass	500.0	[<Confirmed>]	

Sample 63: SSS18_SS24_SS05_SS32_SS30_SS12_SS23_HGY3_I12.fsa

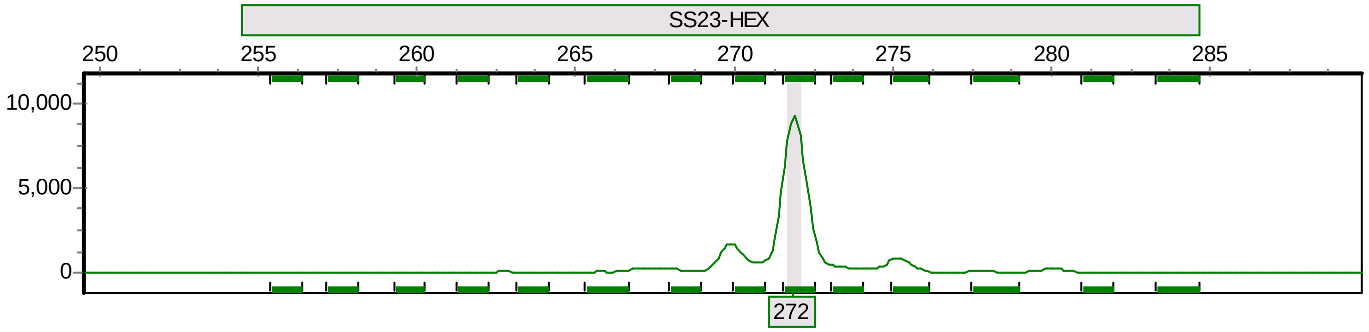

No	Size	Height	Area	Marker	Allele	Difference	Quality	Score	Allele Comments	Sample Comments
1	126.6	13789	88524	SS30-HEX	127	0.10	Pass	500.0	[<Confirmed>]	
2	205.4	11960	85824	SS12-HEX	205	0.00	Pass	500.0	[<Confirmed>]	
3	217.3	9057	67973	SS12-HEX	217	0.10	Pass	500.0	[<Confirmed>]	
4	271.9	9280	78612	SS23-HEX	272	0.20	Pass	500.0	[<Confirmed>]	

Sample 64: SSS18_SS24_SS05_SS32_SS30_SS12_SS23_HGY4_I10.fsa

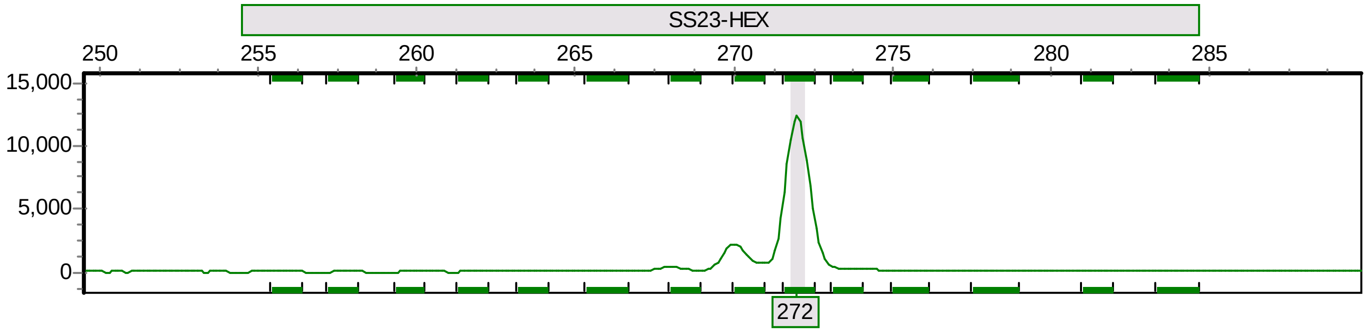

No	Size	Height	Area	Marker	Allele	Difference	Quality	Score	Allele Comments	Sample Comments
1	156.1	5269	33364	SS30-HEX	157	0.10	Pass	500.0	[<Confirmed>]	
2	158.0	5353	33873	SS30-HEX	159	0.10	Pass	500.0	[<Confirmed>]	
3	160.0	2409	15599	SS30-HEX	161	0.00	Undetermined	483.1	[<Deleted>]	
4	217.4	11809	88655	SS12-HEX	217	0.00	Pass	500.0	[<Confirmed>]	
5	219.3	5734	43828	SS12-HEX	219	0.10	Pass	500.0	[<Confirmed>]	
6	272.0	12380	103859	SS23-HEX	272	0.10	Pass	500.0	[<Confirmed>]	

Sample 65: SSS18_SS24_SS05_SS32_SS30_SS12_SS23_HGY5_I16.fsa

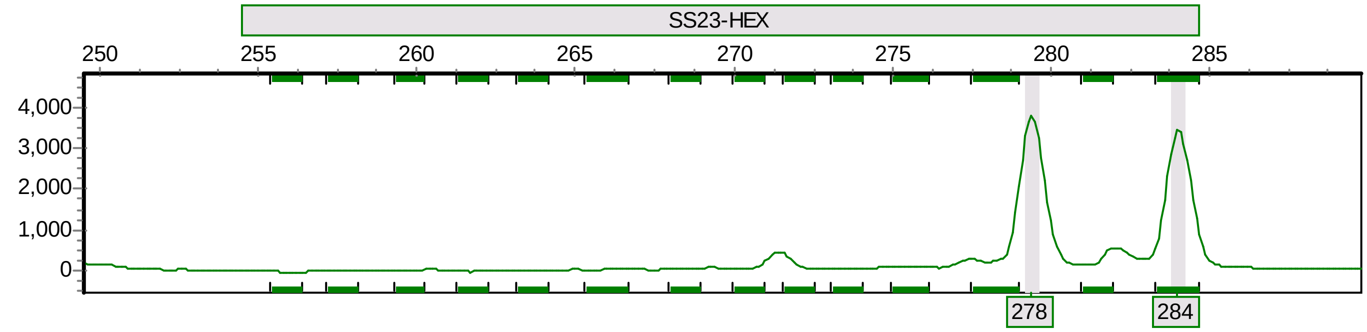

No	Size	Height	Area	Marker	Allele	Difference	Quality	Score	Allele Comments	Sample Comments
1	118.5	3612	23764	SS30-HEX	119	0.10	Pass	500.0	[<Confirmed>]	
2	143.7	9738	72325	SS30-HEX	143	0.00	Pass	500.0	[<Confirmed>]	
3	216.9	9222	71144	SS12-HEX	217	0.50	Pass	500.0	[<Confirmed>]	
4	218.8	9638	75290	SS12-HEX	219	1.00	Pass	500.0	[<Confirmed><Edited>]	
5	279.4	3794	33447	SS23-HEX	278	1.00	Pass	500.0	[<Confirmed><Edited>]	
6	284.0	3441	30881	SS23-HEX	284	0.00	Pass	433.4	[<Confirmed>]	

Sample 66: SSS18_SS24_SS05_SS32_SS30_SS12_SS23_HQZ11_O12.fsa

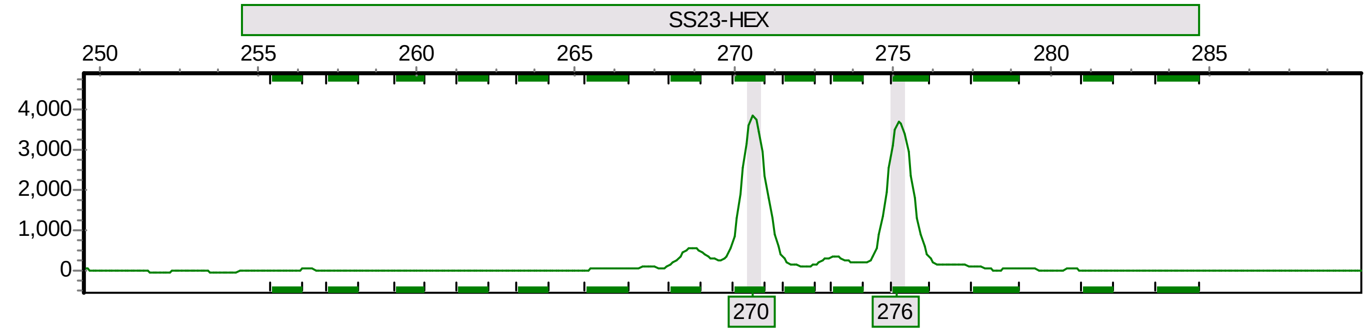

No	Size	Height	Area	Marker	Allele	Difference	Quality	Score	Allele Comments	Sample Comments
1	118.7	30472	195631	SS30-HEX	119	0.10	Pass	500.0	[<Confirmed>]	
2	213.1	4874	36871	SS12-HEX	213	0.20	Pass	500.0	[<Confirmed>]	
3	270.6	3822	33561	SS23-HEX	270	0.10	Pass	500.0	[<Confirmed>]	

Allele Report

4	275.2	3693	33324	SS23-HEX	276	0.30	Pass	464.7	[<Confirmed>]	

Sample 67: SSS18_SS24_SS05_SS32_SS30_SS12_SS23_HQZ13-1_C10.fsa

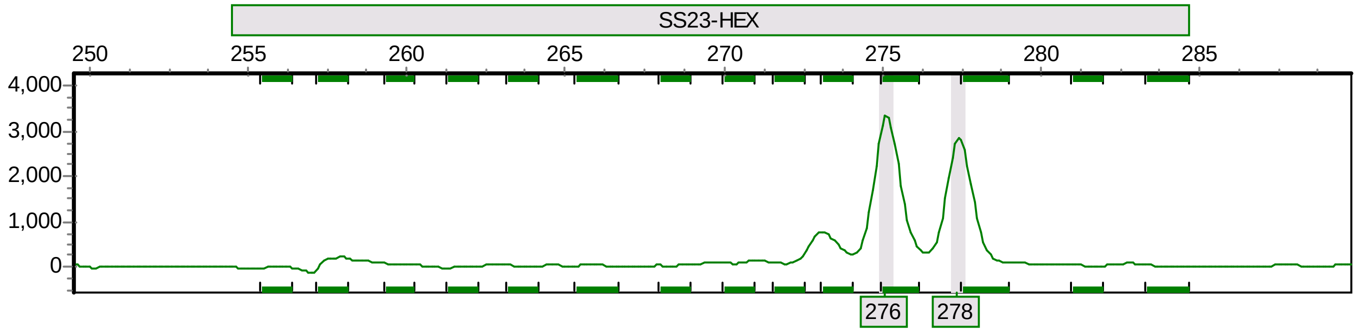

No	Size	Height	Area	Marker	Allele	Difference	Quality	Score	Allele Comments	Sample Comments
1	107.6	12135	55953	SS30-HEX	107	0.00	Pass	500.0	[<Confirmed>]	
2	122.5	12541	82051	SS30-HEX	123	0.10	Pass	500.0	[<Confirmed>]	
3	143.6	5449	41039	SS30-HEX	143	0.10	Undetermined	500.0	[<Deleted>]	
4	205.4	16023	120646	SS12-HEX	205	0.00	Pass	500.0	[<Confirmed>]	
5	275.1	3334	31775	SS23-HEX	276	0.40	Pass	385.5	[<Confirmed>]	
6	277.4	2867	26460	SS23-HEX	278	1.00	Pass	327.5	[<Confirmed><Edited>]	

Sample 68: SSS18_SS24_SS05_SS32_SS30_SS12_SS23_HQZ13-2_J04.fsa

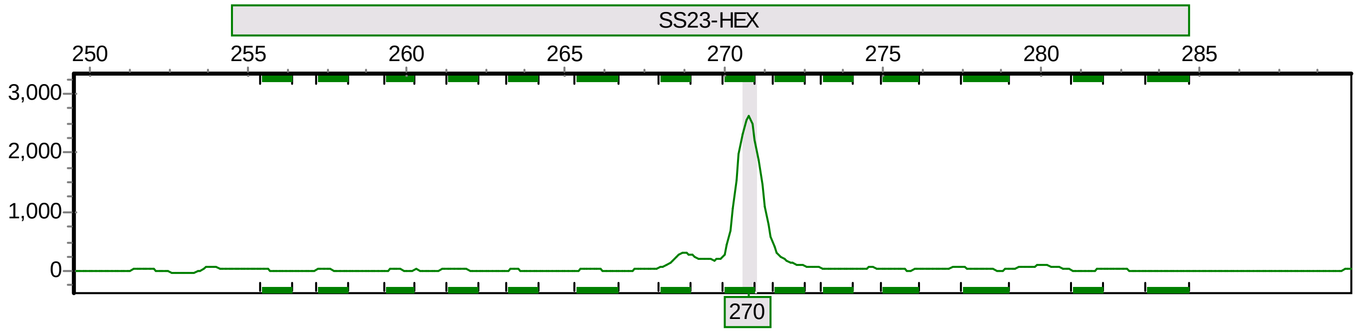

No	Size	Height	Area	Marker	Allele	Difference	Quality	Score	Allele Comments	Sample Comments
1	120.8	14892	93303	SS30-HEX	121	0.20	Pass	500.0	[<Confirmed>]	
2	139.2	6245	44869	SS30-HEX	139	0.10	Pass	500.0	[<Confirmed>]	
3	207.3	558	4344	SS12-HEX	207	0.00	Pass	37.8	[<Confirmed>]	
4	270.8	2616	22813	SS23-HEX	270	0.30	Pass	293.1	[<Confirmed>]	

Sample 69: SSS18_SS24_SS05_SS32_SS30_SS12_SS23_HQZ14_H04.fsa

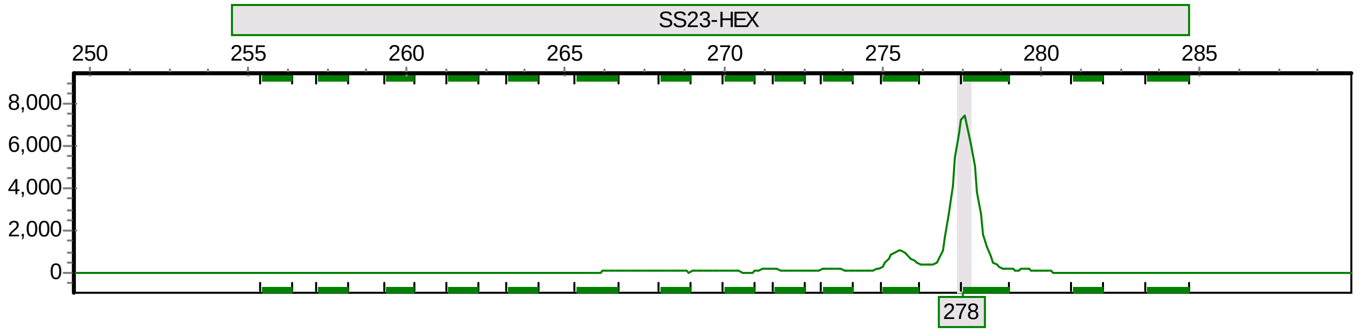

No	Size	Height	Area	Marker	Allele	Difference	Quality	Score	Allele Comments	Sample Comments
1	118.8	24676	150292	SS30-HEX	119	0.20	Pass	500.0	[<Confirmed>]	
2	126.9	14946	92013	SS30-HEX	127	0.20	Pass	500.0	[<Confirmed>]	
3	213.2	6862	49541	SS12-HEX	213	0.10	Pass	500.0	[<Confirmed>]	
4	277.6	7385	61560	SS23-HEX	278	0.60	Pass	500.0	[<Confirmed>]	

Sample 70: SSS18_SS24_SS05_SS32_SS30_SS12_SS23_HQZ15_B02.fsa

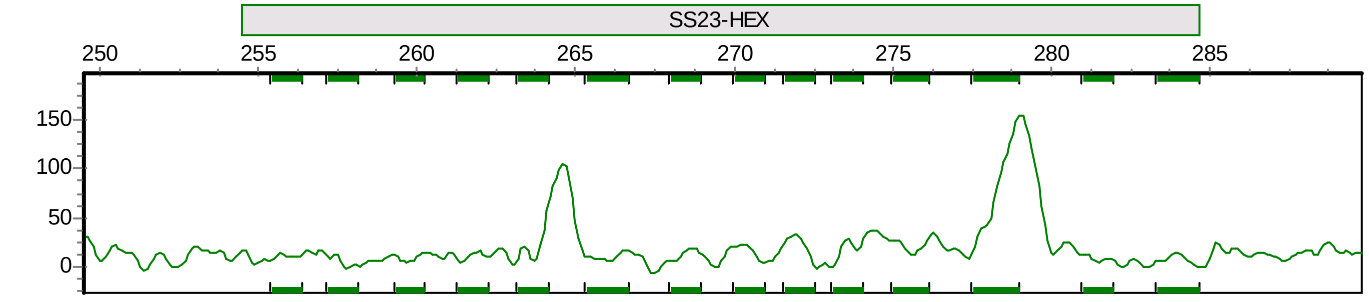

No	Size	Height	Area	Marker	Allele	Difference	Quality	Score	Allele Comments	Sample Comments
1	118.8	26826	168380	SS30-HEX	119	0.20	Pass	500.0	[<Confirmed>]	

Sample 71: SSS18_SS24_SS05_SS32_SS30_SS12_SS23_HQZ16_P02.fsa

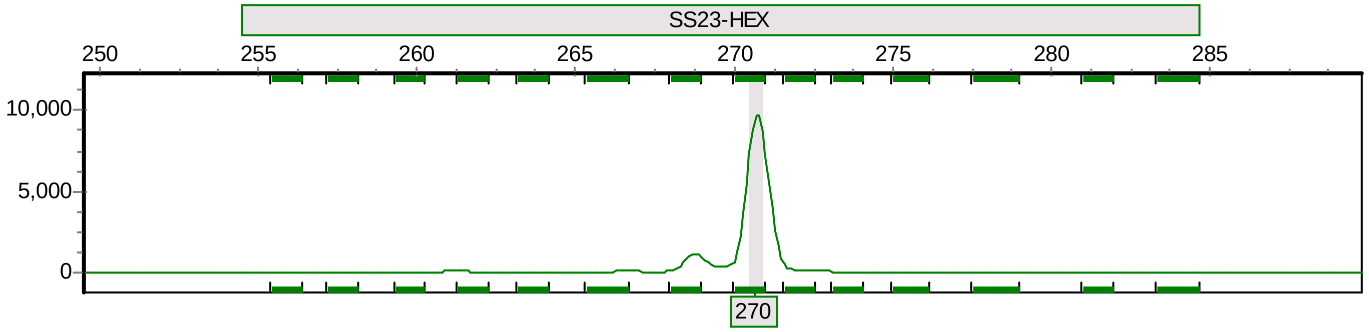

No	Size	Height	Area	Marker	Allele	Difference	Quality	Score	Allele Comments	Sample Comments
1	127.1	11559	69941	SS30-HEX	127	0.40	Pass	500.0	[<Confirmed>]	
2	139.5	7008	47797	SS30-HEX	139	0.40	Pass	500.0	[<Confirmed>]	
3	207.6	745	5200	SS12-HEX	207	0.30	Pass	75.8	[<Confirmed>]	
4	270.7	9635	74602	SS23-HEX	270	0.20	Pass	500.0	[<Confirmed>]	

Sample 72: SSS18_SS24_SS05_SS32_SS30_SS12_SS23_HQZ17-1_O04.fsa

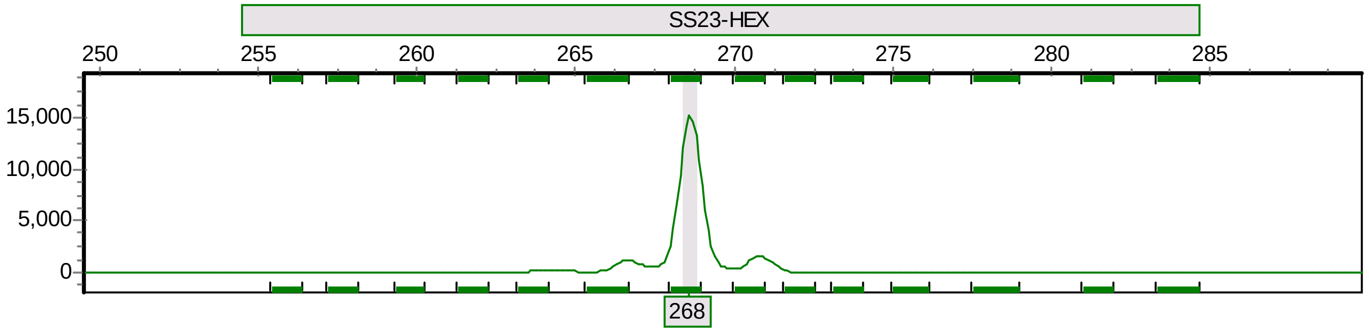

No	Size	Height	Area	Marker	Allele	Difference	Quality	Score	Allele Comments	Sample Comments
1	114.6	28091	169874	SS30-HEX	115	0.00	Pass	500.0	[<Confirmed>]	
2	209.5	8087	57702	SS12-HEX	209	0.10	Pass	500.0	[<Confirmed>]	
3	268.6	15175	120394	SS23-HEX	268	0.10	Pass	500.0	[<Confirmed>]	

Sample 73: SSS18_SS24_SS05_SS32_SS30_SS12_SS23_HQZ17-2_N04.fsa

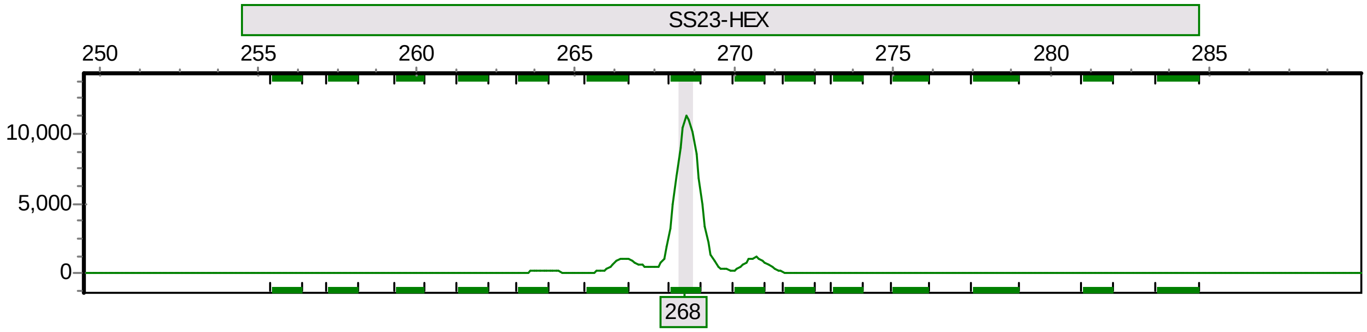

No	Size	Height	Area	Marker	Allele	Difference	Quality	Score	Allele Comments	Sample Comments
1	114.6	20126	122313	SS30-HEX	115	0.00	Pass	500.0	[<Confirmed>]	
2	209.4	4987	36674	SS12-HEX	209	0.00	Pass	500.0	[<Confirmed>]	
3	268.5	11278	92312	SS23-HEX	268	0.00	Pass	500.0	[<Confirmed>]	

Sample 74: SSS18_SS24_SS05_SS32_SS30_SS12_SS23_HQZ18_K10.fsa

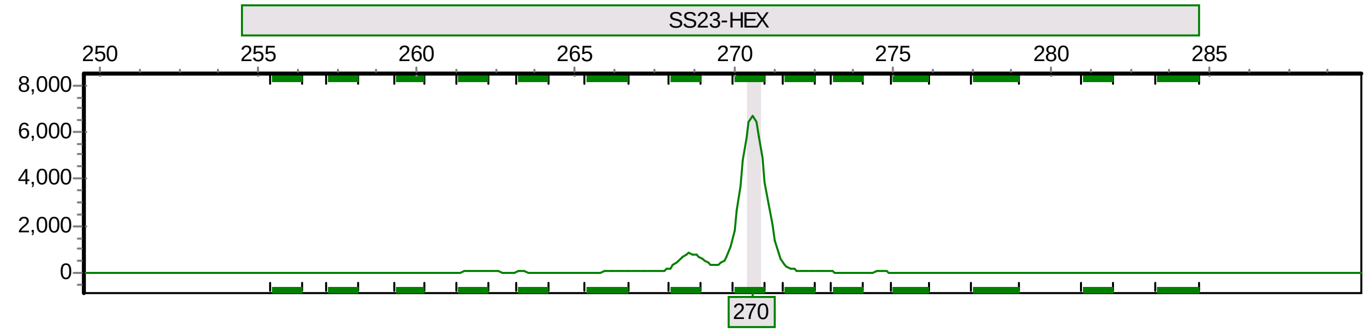

No	Size	Height	Area	Marker	Allele	Difference	Quality	Score	Allele Comments	Sample Comments
1	118.7	19257	120478	SS30-HEX	119	0.10	Pass	500.0	[<Confirmed>]	
2	126.8	13616	85381	SS30-HEX	127	0.10	Pass	500.0	[<Confirmed>]	
3	207.3	527	4183	SS12-HEX	207	0.00	Pass	32.8	[<Confirmed>]	
4	270.6	6657	58879	SS23-HEX	270	0.10	Pass	500.0	[<Confirmed>]	

Sample 75: SSS18_SS24_SS05_SS32_SS30_SS12_SS23_HQZ19_L04.fsa

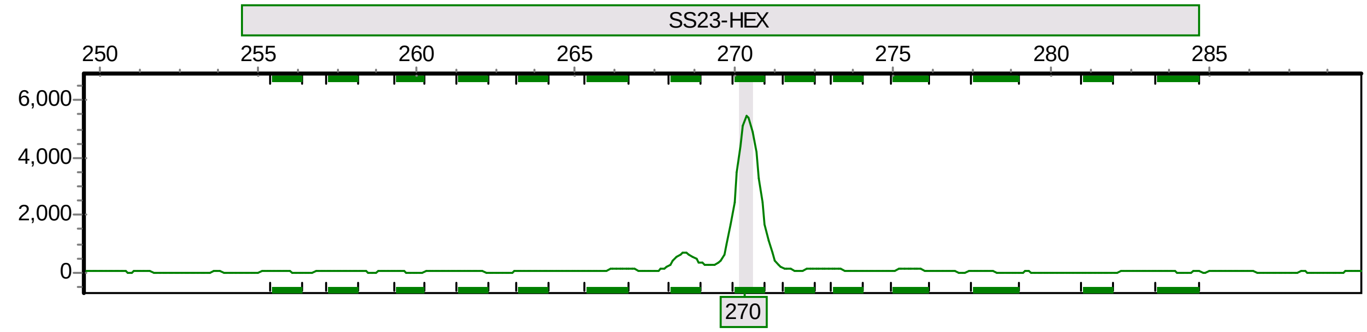

No	Size	Height	Area	Marker	Allele	Difference	Quality	Score	Allele Comments	Sample Comments
1	126.7	22320	142907	SS30-HEX	127	0.00	Pass	500.0	[<Confirmed>]	
2	207.2	781	5847	SS12-HEX	207	0.10	Pass	71.2	[<Confirmed>]	
3	270.4	5434	45086	SS23-HEX	270	0.10	Pass	500.0	[<Confirmed>]	

Sample 76: SSS18_SS24_SS05_SS32_SS30_SS12_SS23_HQZ21_B04.fsa

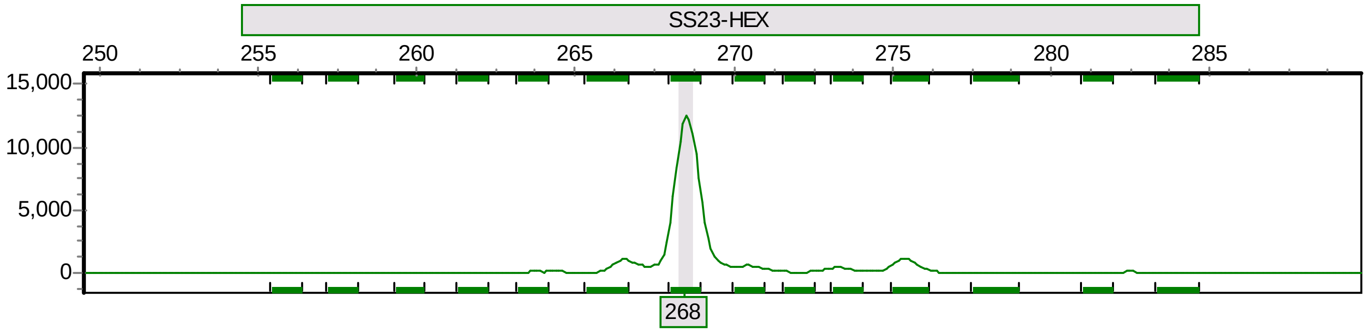

No	Size	Height	Area	Marker	Allele	Difference	Quality	Score	Allele Comments	Sample Comments
1	126.8	13874	87661	SS30-HEX	127	0.10	Pass	500.0	[<Confirmed>]	
2	139.1	8166	59992	SS30-HEX	139	0.00	Pass	500.0	[<Confirmed>]	
3	207.4	2415	18202	SS12-HEX	207	0.10	Pass	370.2	[<Confirmed>]	
4	268.5	12424	106685	SS23-HEX	268	0.00	Pass	500.0	[<Confirmed>]	

Sample 77: SSS18_SS24_SS05_SS32_SS30_SS12_SS23_HQZ22-1_M02.fsa

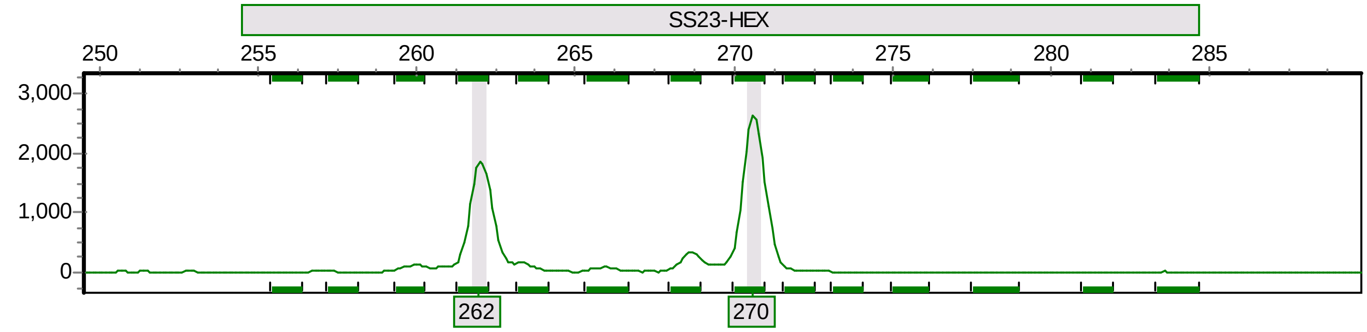

No	Size	Height	Area	Marker	Allele	Difference	Quality	Score	Allele Comments	Sample Comments
1	118.9	15551	96652	SS30-HEX	119	0.30	Pass	500.0	[<Confirmed>]	
2	127.0	11812	72819	SS30-HEX	127	0.30	Pass	500.0	[<Confirmed>]	
3	262.0	1860	14905	SS23-HEX	262	0.20	Pass	231.7	[<Confirmed>]	
4	270.6	2611	20695	SS23-HEX	270	0.10	Pass	386.5	[<Confirmed>]	

Sample 78: SSS18_SS24_SS05_SS32_SS30_SS12_SS23_HQZ22-2_O14.fsa

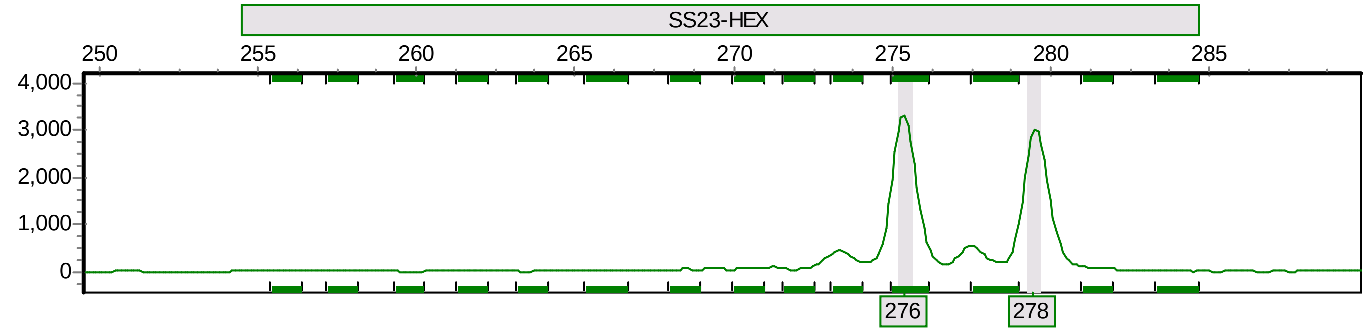

No	Size	Height	Area	Marker	Allele	Difference	Quality	Score	Allele Comments	Sample Comments
1	118.7	9993	62835	SS30-HEX	119	0.10	Pass	500.0	[<Confirmed>]	
2	139.2	4260	30627	SS30-HEX	139	0.10	Pass	500.0	[<Confirmed>]	
3	207.3	1058	8272	SS12-HEX	207	0.00	Pass	104.0	[<Confirmed>]	
4	275.4	3299	29080	SS23-HEX	276	0.10	Pass	414.6	[<Confirmed>]	
5	279.5	3007	27057	SS23-HEX	278	1.00	Pass	365.7	[<Confirmed><Edited>]	

Sample 79: SSS18_SS24_SS05_SS32_SS30_SS12_SS23_HQZ23_A16.fsa

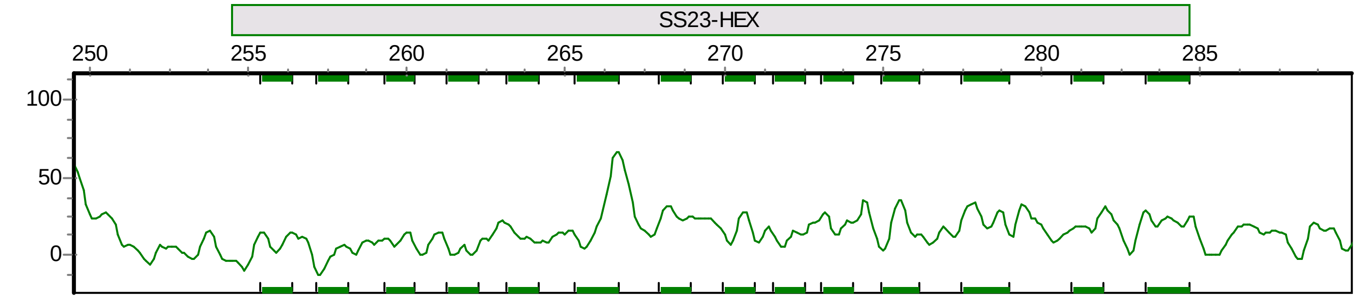

No	Size	Height	Area	Marker	Allele	Difference	Quality	Score	Allele Comments	Sample Comments
1	124.7	13066	85009	SS30-HEX	125	0.00	Pass	500.0	[<Confirmed>]	
2	139.0	6310	44635	SS30-HEX	139	0.10	Pass	500.0	[<Confirmed>]	

Sample 80: SSS18_SS24_SS05_SS32_SS30_SS12_SS23_HQZ24_E12.fsa

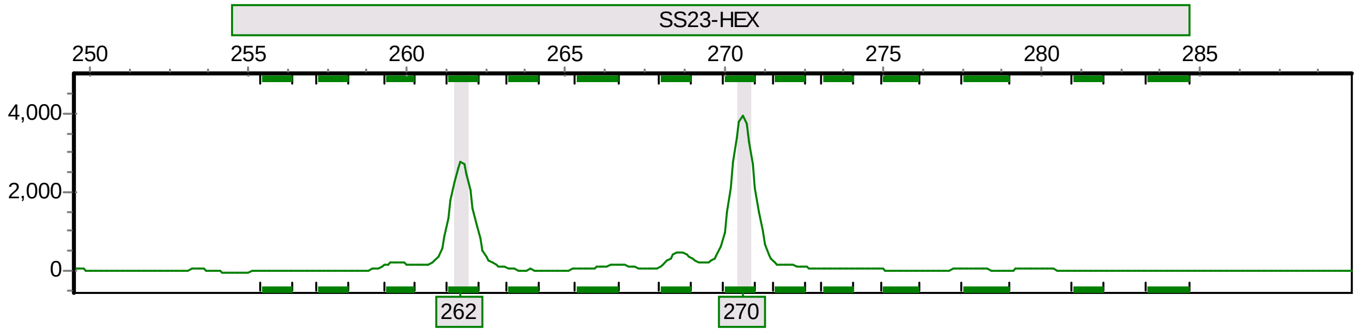

No	Size	Height	Area	Marker	Allele	Difference	Quality	Score	Allele Comments	Sample Comments
1	118.6	17582	113770	SS30-HEX	119	0.00	Pass	500.0	[<Confirmed>]	
2	124.6	12124	77582	SS30-HEX	125	0.10	Pass	500.0	[<Confirmed>]	
3	219.1	3115	23904	SS12-HEX	219	0.30	Pass	500.0	[<Confirmed>]	
4	261.7	2773	23064	SS23-HEX	262	0.10	Pass	372.6	[<Confirmed>]	
5	270.6	3942	32949	SS23-HEX	270	0.10	Pass	500.0	[<Confirmed>]	

Sample 81: SSS18_SS24_SS05_SS32_SS30_SS12_SS23_HQZ25_N02.fsa

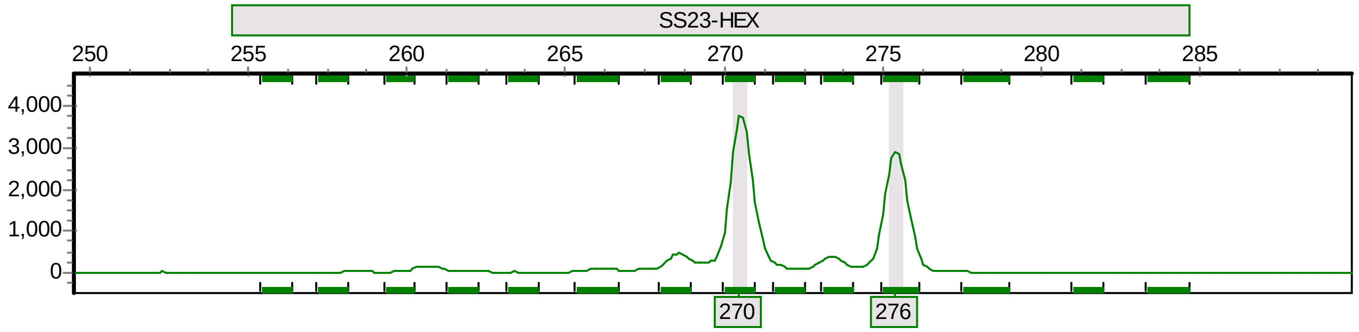

No	Size	Height	Area	Marker	Allele	Difference	Quality	Score	Allele Comments	Sample Comments
1	118.8	17325	104164	SS30-HEX	119	0.20	Pass	500.0	[<Confirmed>]	
2	124.9	12022	73085	SS30-HEX	125	0.20	Pass	500.0	[<Confirmed>]	
3	209.5	5539	39624	SS12-HEX	209	0.10	Pass	500.0	[<Confirmed>]	
4	270.5	3757	30676	SS23-HEX	270	0.00	Pass	500.0	[<Confirmed>]	
5	275.4	2911	24546	SS23-HEX	276	0.10	Pass	380.8	[<Confirmed>]	

Allele Report

Sample 82: SSS18_SS24_SS05_SS32_SS30_SS12_SS23_HQZ26_E04.fsa

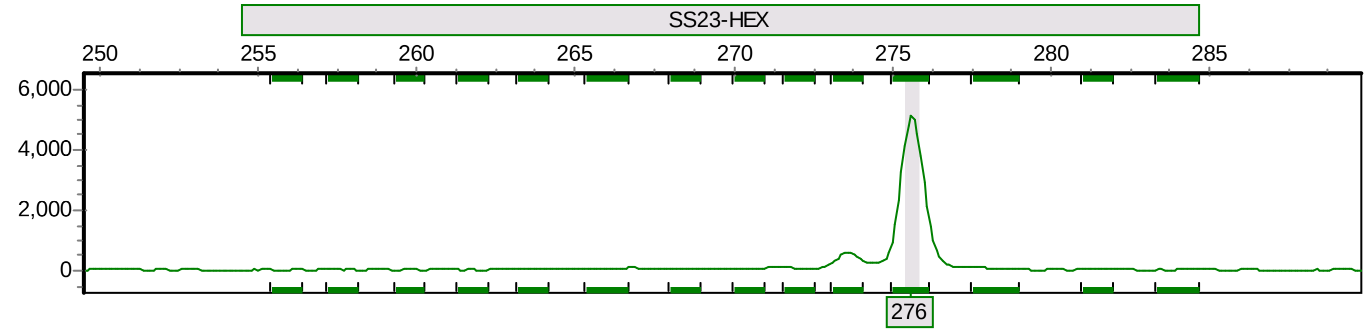

No	Size	Height	Area	Marker	Allele	Difference	Quality	Score	Allele Comments	Sample Comments
1	146.0	12907	89476	SS30-HEX	145	0.00	Pass	500.0	[<Confirmed>]	
2	207.2	493	3535	SS12-HEX	207	0.10	Pass	36.1	[<Confirmed>]	
3	215.3	500	3860	SS12-HEX	215	0.10	Pass	33.5	[<Confirmed>]	
4	275.6	5089	41529	SS23-HEX	276	0.10	Pass	500.0	[<Confirmed>]	

Sample 83: SSS18_SS24_SS05_SS32_SS30_SS12_SS23_HQZ27_G10.fsa

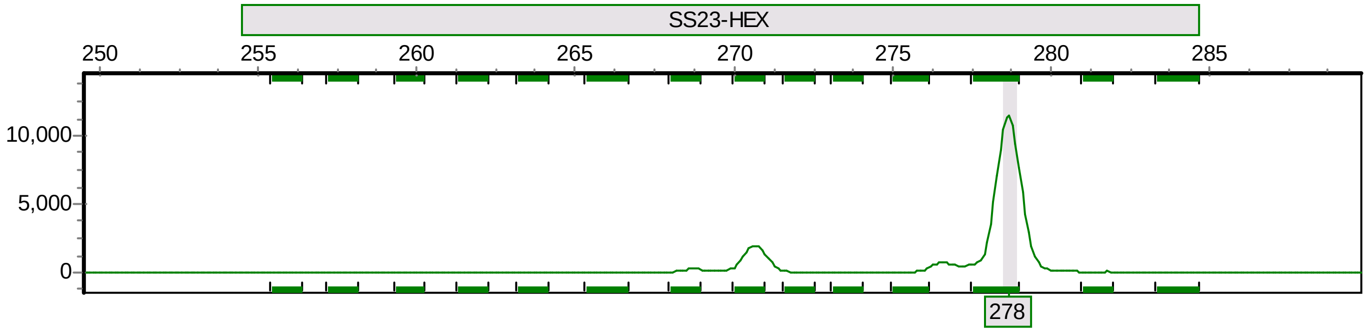

No	Size	Height	Area	Marker	Allele	Difference	Quality	Score	Allele Comments	Sample Comments
1	137.1	14116	96302	SS30-HEX	137	0.10	Pass	500.0	[<Confirmed>]	
2	139.2	8376	61829	SS30-HEX	139	0.10	Pass	500.0	[<Confirmed>]	
3	205.5	19309	141363	SS12-HEX	205	0.10	Pass	500.0	[<Confirmed>]	
4	278.7	11348	99725	SS23-HEX	278	0.50	Pass	500.0	[<Confirmed>]	

Sample 84: SSS18_SS24_SS05_SS32_SS30_SS12_SS23_HQZ28_B06.fsa

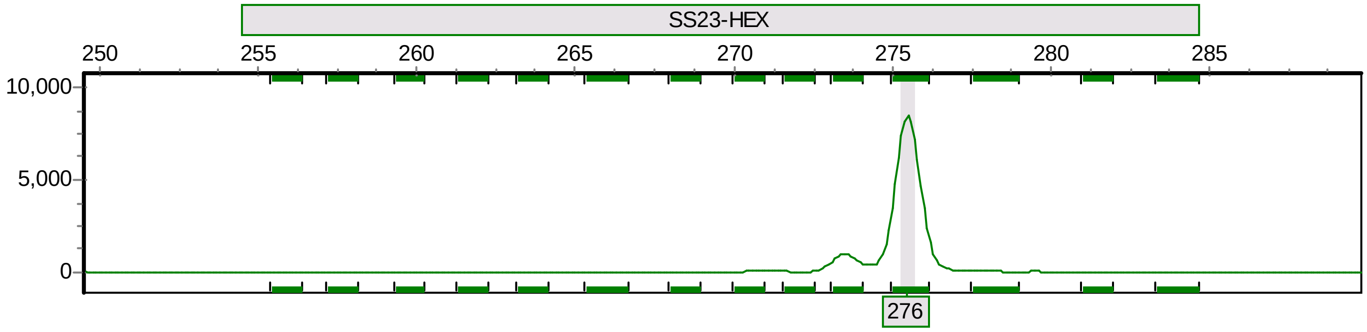

No	Size	Height	Area	Marker	Allele	Difference	Quality	Score	Allele Comments	Sample Comments
1	126.7	13859	86675	SS30-HEX	127	0.00	Pass	500.0	[<Confirmed>]	
2	139.0	8429	57057	SS30-HEX	139	0.10	Pass	500.0	[<Confirmed>]	
3	207.2	686	5064	SS12-HEX	207	0.10	Pass	58.9	[<Confirmed>]	
4	215.3	675	5024	SS12-HEX	215	0.10	Pass	52.3	[<Confirmed>]	
5	275.5	8441	73753	SS23-HEX	276	0.00	Pass	500.0	[<Confirmed>]	

Sample 85: SSS18_SS24_SS05_SS32_SS30_SS12_SS23_HQZ29_C14.fsa

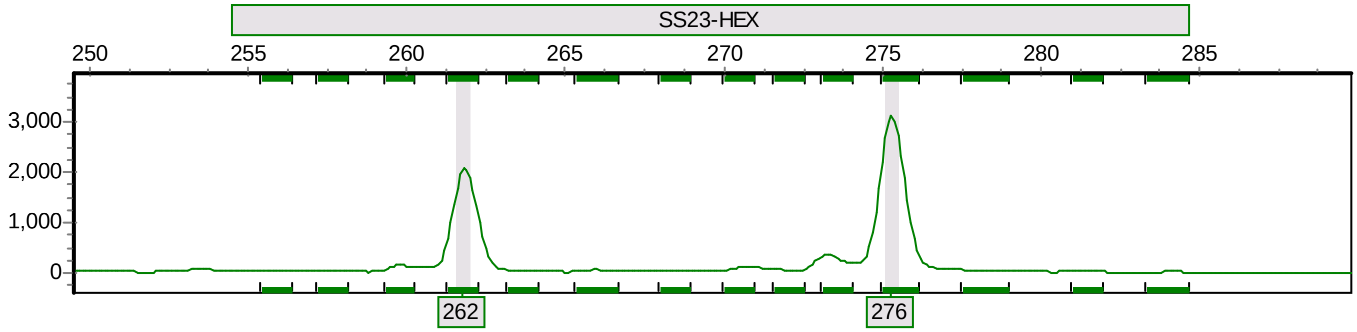

No	Size	Height	Area	Marker	Allele	Difference	Quality	Score	Allele Comments	Sample Comments
1	118.4	15700	102688	SS30-HEX	119	0.20	Pass	500.0	[<Confirmed>]	
2	128.6	10249	67967	SS30-HEX	129	0.20	Pass	500.0	[<Confirmed>]	
3	207.3	710	5404	SS12-HEX	207	0.00	Pass	61.1	[<Confirmed>]	
4	261.8	2074	18113	SS23-HEX	262	0.00	Pass	223.4	[<Confirmed>]	
5	275.3	3097	27550	SS23-HEX	276	0.20	Pass	379.5	[<Confirmed>]	

Sample 86: SSS18_SS24_SS05_SS32_SS30_SS12_SS23_HQZ2_K04.fsa

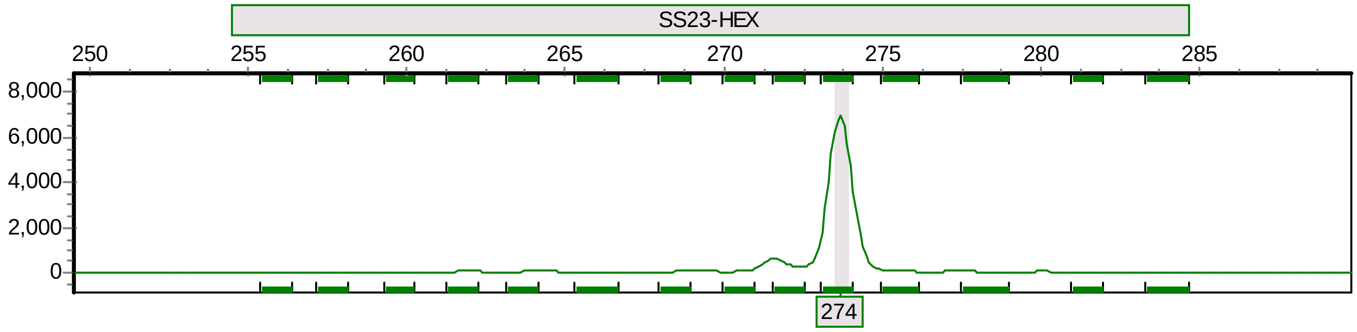

No	Size	Height	Area	Marker	Allele	Difference	Quality	Score	Allele Comments	Sample Comments
1	128.8	22988	144290	SS30-HEX	129	0.00	Pass	500.0	[<Confirmed>]	
2	205.3	1126	8383	SS12-HEX	205	0.10	Pass	124.8	[<Confirmed>]	
3	219.3	747	5828	SS12-HEX	219	0.10	Pass	58.0	[<Confirmed>]	
4	273.7	6901	58665	SS23-HEX	274	0.10	Pass	500.0	[<Confirmed>]	

Sample 87: SSS18_SS24_SS05_SS32_SS30_SS12_SS23_HQZ30_G16.fsa

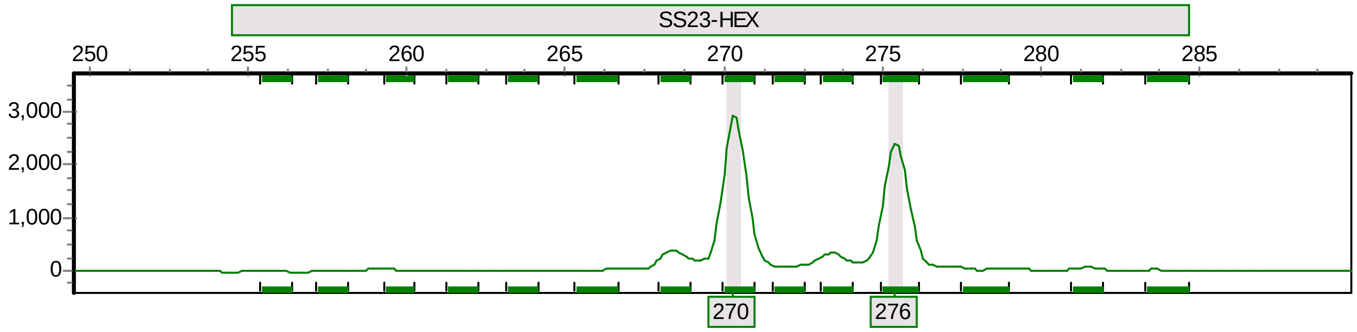

No	Size	Height	Area	Marker	Allele	Difference	Quality	Score	Allele Comments	Sample Comments
1	128.6	10225	68593	SS30-HEX	129	0.20	Pass	500.0	[<Confirmed>]	
2	134.8	7873	52573	SS30-HEX	135	0.10	Pass	500.0	[<Confirmed>]	
3	219.2	484	3996	SS12-HEX	219	0.20	Pass	25.7	[<Confirmed><Inserted>]	
4	270.3	2904	24717	SS23-HEX	270	0.20	Pass	389.0	[<Confirmed>]	
5	275.4	2389	21332	SS23-HEX	276	0.10	Pass	261.0	[<Confirmed>]	

Sample 88: SSS18_SS24_SS05_SS32_SS30_SS12_SS23_HQZ31_K14.fsa

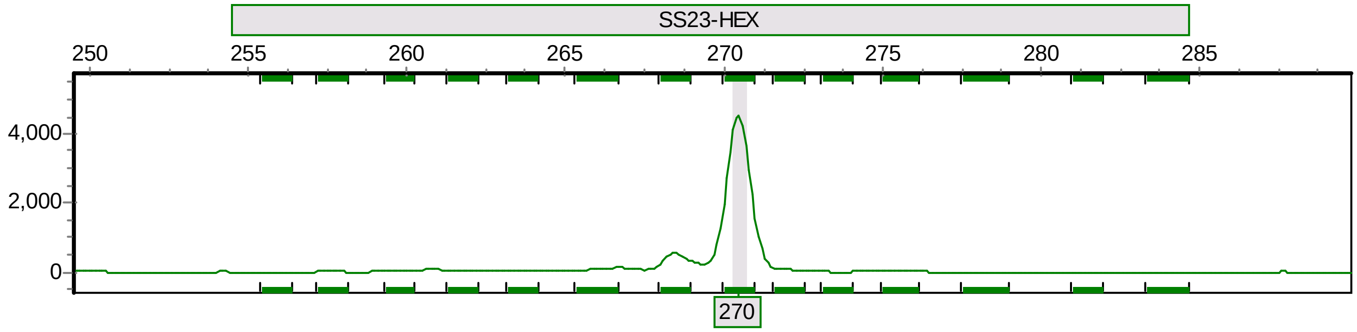

No	Size	Height	Area	Marker	Allele	Difference	Quality	Score	Allele Comments	Sample Comments
1	118.7	4359	27664	SS30-HEX	119	0.10	Undetermined	500.0	[<Deleted>]	
2	124.7	12268	78388	SS30-HEX	125	0.00	Pass	500.0	[<Confirmed>]	
3	145.9	5094	36450	SS30-HEX	145	0.10	Pass	500.0	[<Confirmed>]	
4	270.5	4492	38340	SS23-HEX	270	0.00	Pass	500.0	[<Confirmed>]	

Sample 89: SSS18_SS24_SS05_SS32_SS30_SS12_SS23_HQZ32_J02.fsa

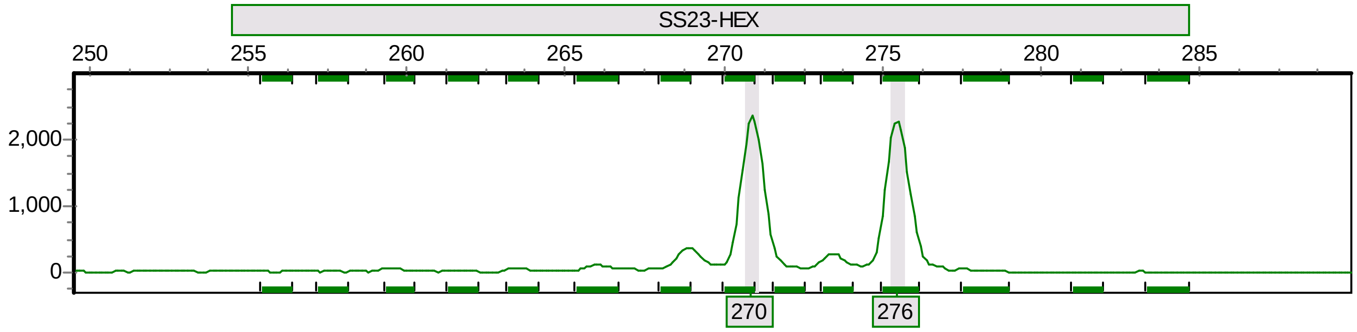

No	Size	Height	Area	Marker	Allele	Difference	Quality	Score	Allele Comments	Sample Comments
1	126.9	24040	150833	SS30-HEX	127	0.20	Pass	500.0	[<Confirmed>]	
2	270.9	2338	18609	SS23-HEX	270	0.40	Pass	322.2	[<Confirmed>]	
3	275.5	2273	18907	SS23-HEX	276	0.00	Pass	281.5	[<Confirmed>]	

Sample 90: SSS18_SS24_SS05_SS32_SS30_SS12_SS23_HQZ33_I04.fsa

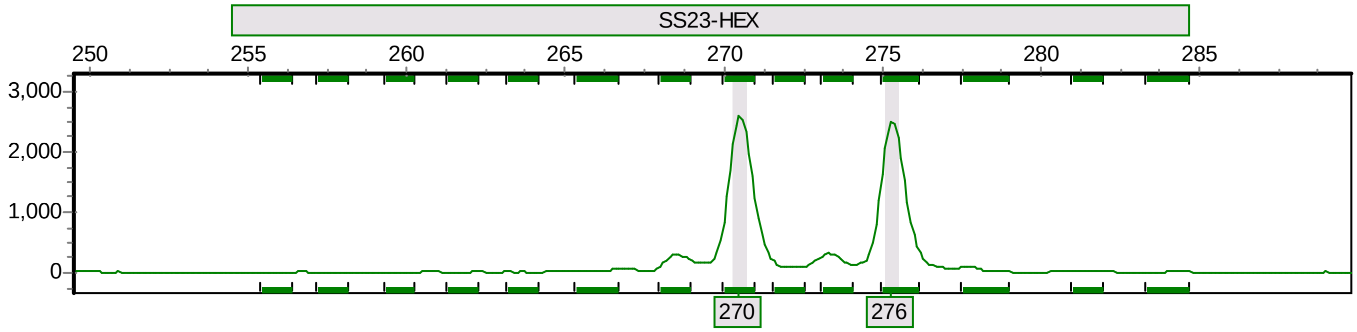

No	Size	Height	Area	Marker	Allele	Difference	Quality	Score	Allele Comments	Sample Comments
1	128.8	9949	62870	SS30-HEX	129	0.00	Pass	500.0	[<Confirmed>]	
2	135.0	6573	42285	SS30-HEX	135	0.10	Pass	500.0	[<Confirmed>]	
3	270.5	2588	22546	SS23-HEX	270	0.00	Pass	315.6	[<Confirmed>]	
4	275.3	2498	21715	SS23-HEX	276	0.20	Pass	302.4	[<Confirmed>]	

Sample 91: SSS18_SS24_SS05_SS32_SS30_SS12_SS23_HQZ34_M04.fsa

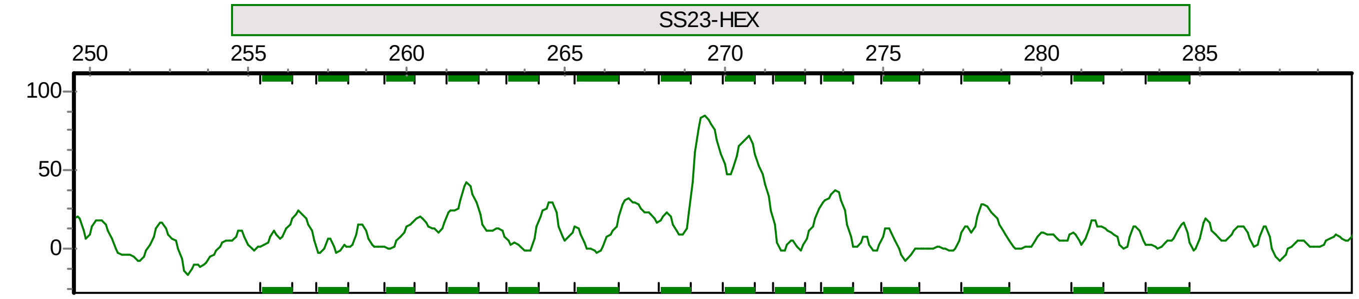

No	Size	Height	Area	Marker	Allele	Difference	Quality	Score	Allele Comments	Sample Comments
1	114.5	22016	134383	SS30-HEX	115	0.10	Pass	500.0	[<Confirmed>]	
2	213.2	5374	39071	SS12-HEX	213	0.10	Pass	500.0	[<Confirmed>]	

Sample 92: SSS18_SS24_SS05_SS32_SS30_SS12_SS23_HQZ35_A02.fsa

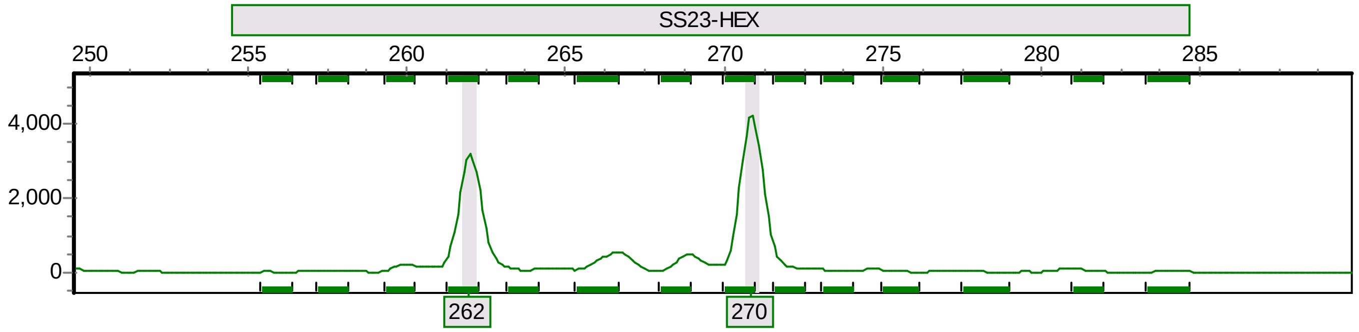

No	Size	Height	Area	Marker	Allele	Difference	Quality	Score	Allele Comments	Sample Comments
1	128.8	10191	64365	SS30-HEX	129	0.00	Pass	500.0	[<Confirmed>]	
2	145.9	5022	35881	SS30-HEX	145	0.10	Pass	500.0	[<Confirmed>]	
3	203.3	8557	60523	SS12-HEX	203	0.00	Pass	500.0	[<Confirmed>]	
4	262.0	3176	25869	SS23-HEX	262	0.20	Pass	468.4	[<Confirmed>]	
5	270.9	4197	34442	SS23-HEX	270	0.40	Pass	500.0	[<Confirmed>]	

Sample 93: SSS18_SS24_SS05_SS32_SS30_SS12_SS23_HQZ36_E16.fsa

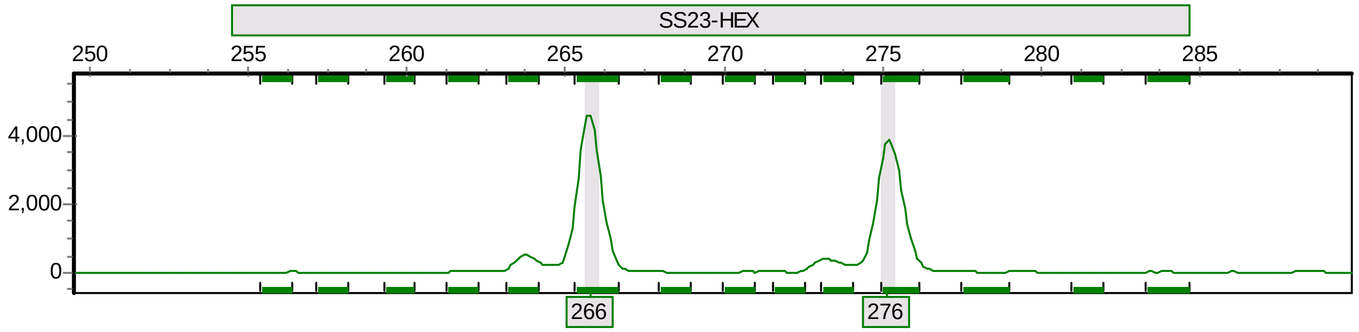

No	Size	Height	Area	Marker	Allele	Difference	Quality	Score	Allele Comments	Sample Comments
1	118.5	19520	125833	SS30-HEX	119	0.10	Pass	500.0	[<Confirmed>]	
2	126.6	12839	83785	SS30-HEX	127	0.10	Pass	500.0	[<Confirmed>]	
3	207.2	795	5897	SS12-HEX	207	0.10	Pass	74.2	[<Confirmed>]	
4	265.8	4550	37177	SS23-HEX	266	0.20	Pass	500.0	[<Confirmed>]	
5	275.2	3880	35091	SS23-HEX	276	0.30	Pass	499.8	[<Confirmed>]	

Allele Report

Sample 94: SSS18_SS24_SS05_SS32_SS30_SS12_SS23_HQZ37_F04.fsa

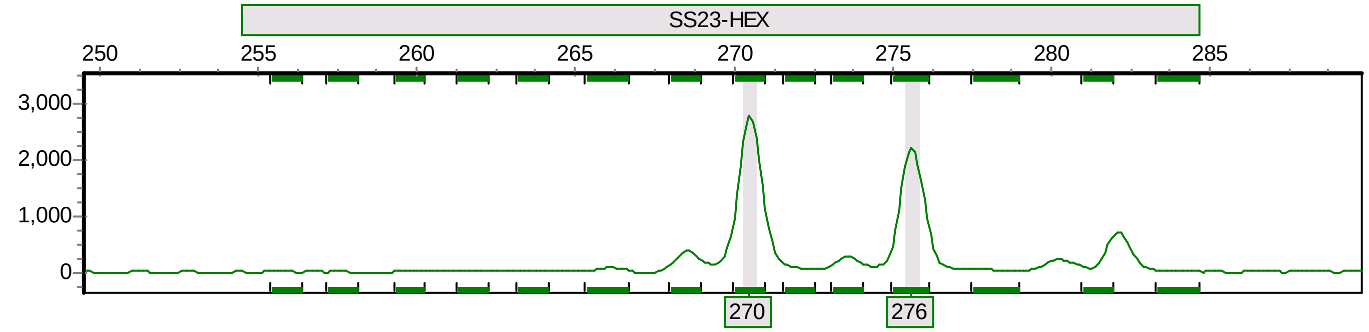

No	Size	Height	Area	Marker	Allele	Difference	Quality	Score	Allele Comments	Sample Comments
1	128.8	23449	145804	SS30-HEX	129	0.00	Pass	500.0	[<Confirmed>]	
2	209.5	2964	21429	SS12-HEX	209	0.10	Undetermined	500.0	[<Deleted>]	
3	215.3	3608	29081	SS12-HEX	215	0.10	Pass	500.0	[<Confirmed>]	
4	217.6	3733	27196	SS12-HEX	217	0.20	Pass	500.0	[<Confirmed>]	
5	270.5	2777	23289	SS23-HEX	270	0.00	Pass	371.7	[<Confirmed>]	
6	275.6	2225	18732	SS23-HEX	276	0.10	Pass	263.5	[<Confirmed>]	

Sample 95: SSS18_SS24_SS05_SS32_SS30_SS12_SS23_HQZ38_L02.fsa

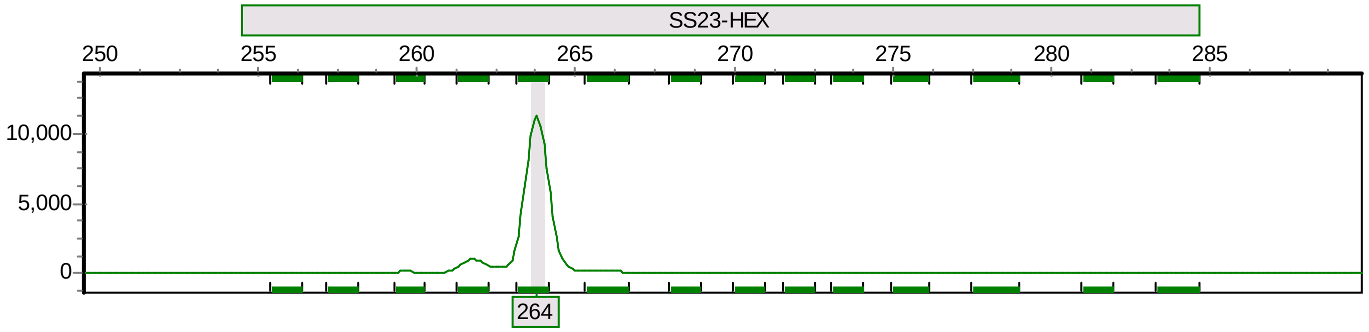

No	Size	Height	Area	Marker	Allele	Difference	Quality	Score	Allele Comments	Sample Comments
1	114.6	17454	106078	SS30-HEX	115	0.00	Pass	500.0	[<Confirmed>]	
2	122.7	11938	73586	SS30-HEX	123	0.10	Pass	500.0	[<Confirmed>]	
3	203.3	5501	39583	SS12-HEX	203	0.00	Pass	500.0	[<Confirmed>]	
4	263.8	11285	93113	SS23-HEX	264	0.10	Pass	500.0	[<Confirmed>]	

Sample 96: SSS18_SS24_SS05_SS32_SS30_SS12_SS23_HQZ39_M16.fsa

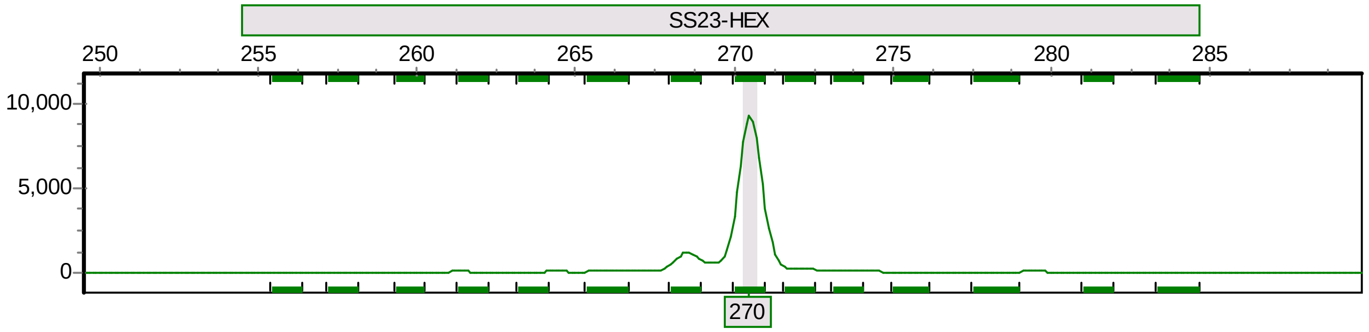

No	Size	Height	Area	Marker	Allele	Difference	Quality	Score	Allele Comments	Sample Comments
1	128.8	9841	65754	SS30-HEX	129	0.00	Pass	500.0	[<Confirmed>]	
2	139.2	7429	54625	SS30-HEX	139	0.10	Pass	500.0	[<Confirmed>]	
3	217.4	8337	65076	SS12-HEX	217	0.00	Pass	500.0	[<Confirmed>]	
4	270.5	9209	77914	SS23-HEX	270	0.00	Pass	500.0	[<Confirmed>]	

Sample 97: SSS18_SS24_SS05_SS32_SS30_SS12_SS23_HQZ7_C04.fsa

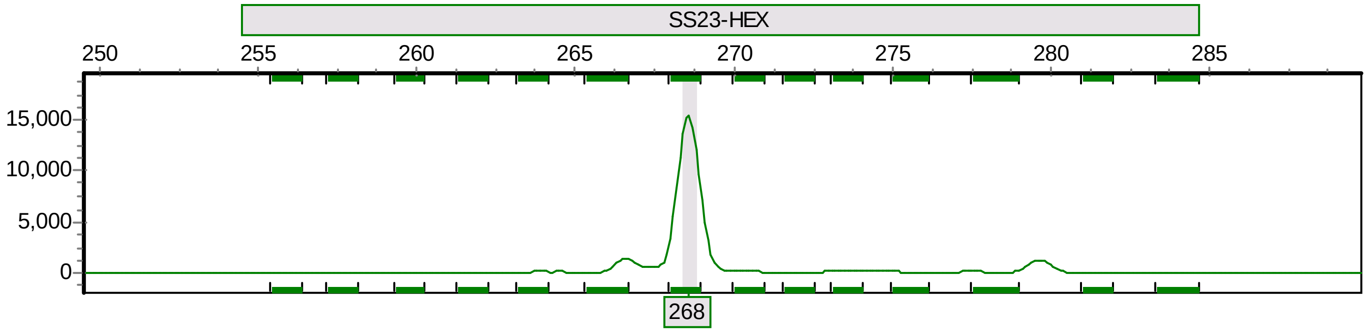

No	Size	Height	Area	Marker	Allele	Difference	Quality	Score	Allele Comments	Sample Comments
1	120.6	20099	124749	SS30-HEX	121	0.00	Pass	500.0	[<Confirmed>]	
2	128.7	13456	82655	SS30-HEX	129	0.10	Pass	500.0	[<Confirmed>]	
3	207.4	2261	16412	SS12-HEX	207	0.10	Pass	358.6	[<Confirmed>]	
4	268.6	15319	121580	SS23-HEX	268	0.10	Pass	500.0	[<Confirmed>]	

Sample 98: SSS18_SS24_SS05_SS32_SS30_SS12_SS23_HQZ9_K12.fsa

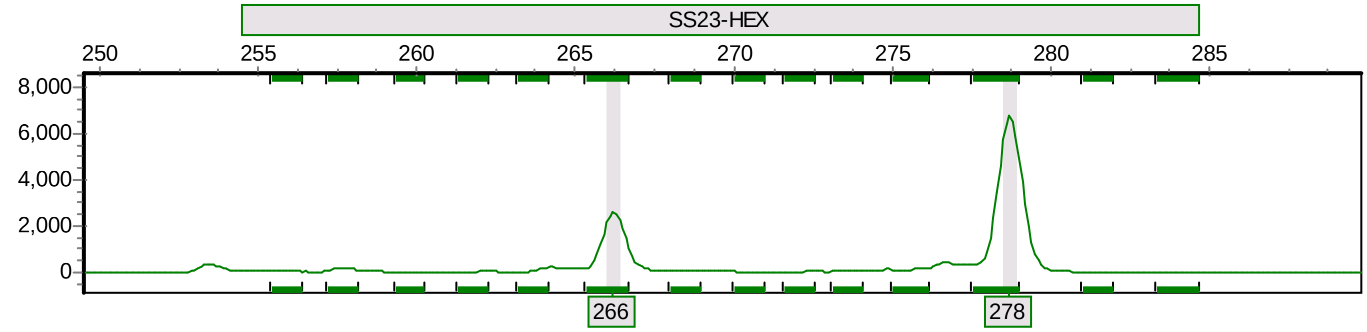

No	Size	Height	Area	Marker	Allele	Difference	Quality	Score	Allele Comments	Sample Comments
1	116.5	16373	102043	SS30-HEX	117	0.10	Pass	500.0	[<Confirmed>]	
2	217.4	10810	79743	SS12-HEX	217	0.00	Pass	500.0	[<Confirmed>]	
3	266.2	2603	21207	SS23-HEX	266	0.20	Pass	355.3	[<Confirmed>]	
4	278.7	6747	57298	SS23-HEX	278	0.50	Pass	500.0	[<Confirmed>]	

Sample 99: SSS18_SS24_SS05_SS32_SS30_SS12_SS23_HRS24_I18.fsa

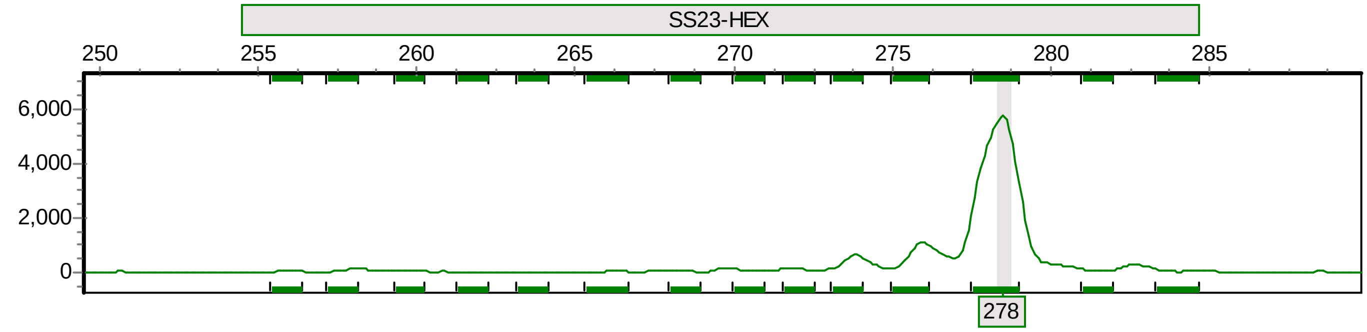

No	Size	Height	Area	Marker	Allele	Difference	Quality	Score	Allele Comments	Sample Comments
1	122.6	11774	76654	SS30-HEX	123	0.00	Pass	500.0	[<Confirmed>]	
2	143.6	5420	40738	SS30-HEX	143	0.10	Pass	500.0	[<Confirmed>]	
3	215.4	6852	53562	SS12-HEX	215	0.00	Pass	500.0	[<Confirmed>]	
4	219.4	4703	37105	SS12-HEX	219	0.00	Pass	500.0	[<Confirmed>]	
5	278.5	5750	78760	SS23-HEX	278	0.30	Pass	346.5	[<Confirmed>]	

Sample 100: SSS18_SS24_SS05_SS32_SS30_SS12_SS23_HRS26_H10.fsa

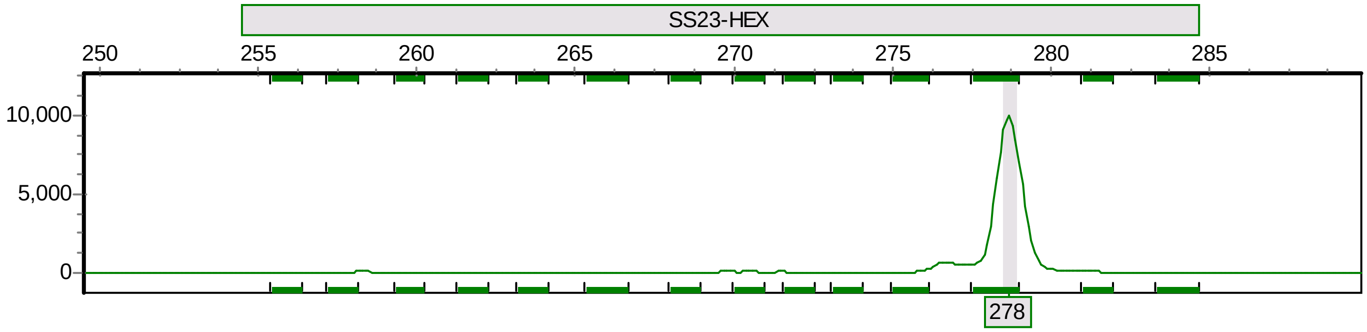

No	Size	Height	Area	Marker	Allele	Difference	Quality	Score	Allele Comments	Sample Comments
1	122.7	12252	78322	SS30-HEX	123	0.10	Pass	500.0	[<Confirmed>]	
2	215.3	7099	55046	SS12-HEX	215	0.10	Pass	500.0	[<Confirmed>]	
3	219.3	4493	35498	SS12-HEX	219	0.10	Pass	500.0	[<Confirmed>]	
4	278.7	9954	89489	SS23-HEX	278	0.50	Pass	500.0	[<Confirmed>]	

Sample 101: SSS18_SS24_SS05_SS32_SS30_SS12_SS23_HRS28_D14.fsa

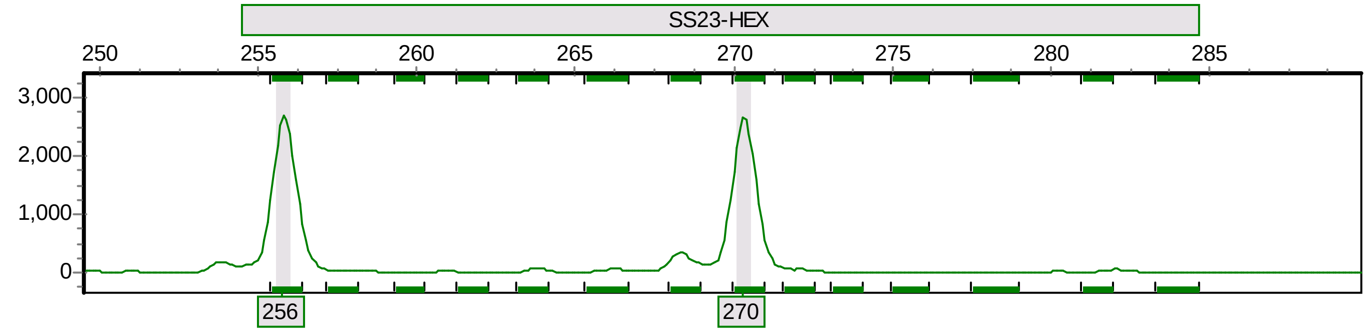

No	Size	Height	Area	Marker	Allele	Difference	Quality	Score	Allele Comments	Sample Comments
1	118.5	12564	82026	SS30-HEX	119	0.10	Pass	500.0	[<Confirmed>]	
2	141.3	4269	31409	SS30-HEX	141	0.10	Pass	500.0	[<Confirmed>]	
3	255.8	2691	22619	SS23-HEX	256	0.10	Pass	356.5	[<Confirmed>]	
4	270.3	2663	22510	SS23-HEX	270	0.20	Pass	342.2	[<Confirmed>]	

Sample 102: SSS18_SS24_SS05_SS32_SS30_SS12_SS23_HRS29_J06.fsa

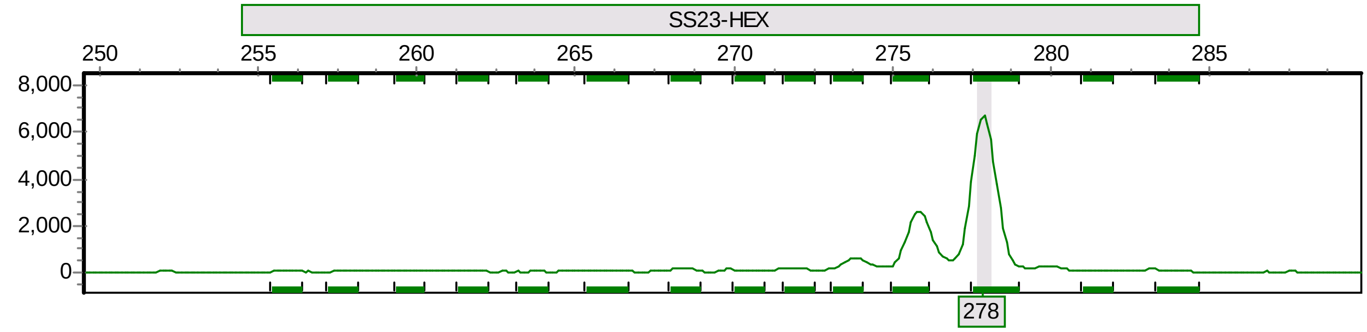

No	Size	Height	Area	Marker	Allele	Difference	Quality	Score	Allele Comments	Sample Comments
1	122.6	13108	82423	SS30-HEX	123	0.00	Pass	500.0	[<Confirmed>]	
2	215.3	7330	55526	SS12-HEX	215	0.10	Pass	500.0	[<Confirmed>]	
3	219.4	5463	40536	SS12-HEX	219	0.00	Pass	500.0	[<Confirmed>]	
4	277.9	6697	58938	SS23-HEX	278	0.30	Pass	500.0	[<Confirmed>]	

Allele Report

Sample 103: SSS18_SS24_SS05_SS32_SS30_SS12_SS23_HRS30_P06.fsa

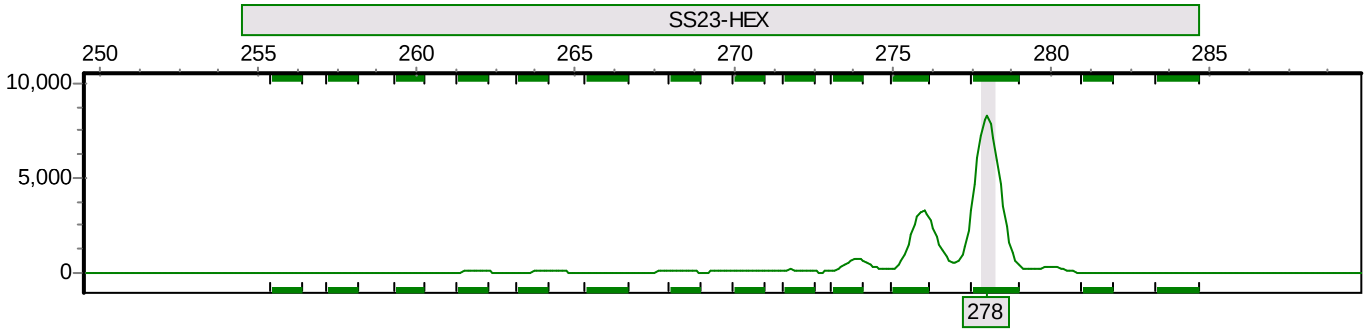

No	Size	Height	Area	Marker	Allele	Difference	Quality	Score	Allele Comments	Sample Comments
1	126.9	13153	83604	SS30-HEX	127	0.20	Pass	500.0	[<Confirmed>]	
2	215.5	7942	59737	SS12-HEX	215	0.10	Pass	500.0	[<Confirmed>]	
3	219.4	5268	39532	SS12-HEX	219	0.00	Pass	500.0	[<Confirmed>]	
4	278.0	8264	72968	SS23-HEX	278	0.20	Pass	500.0	[<Confirmed>]	

Sample 104: SSS18_SS24_SS05_SS32_SS30_SS12_SS23_HRS31_J12.fsa

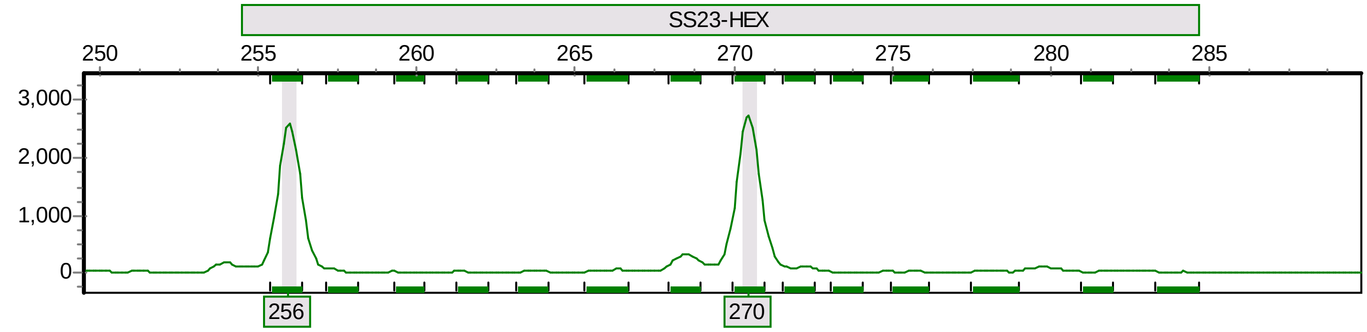

No	Size	Height	Area	Marker	Allele	Difference	Quality	Score	Allele Comments	Sample Comments
1	135.0	8241	53114	SS30-HEX	135	0.10	Pass	500.0	[<Confirmed>]	
2	145.9	4713	34087	SS30-HEX	145	0.10	Pass	500.0	[<Confirmed>]	
3	198.6	531	4362	SS12-HEX	199	0.50	Pass	31.7	[<Confirmed>]	
4	207.3	669	4976	SS12-HEX	207	0.00	Pass	57.0	[<Confirmed>]	
5	256.0	2594	21103	SS23-HEX	256	0.10	Pass	353.1	[<Confirmed>]	
6	270.5	2720	22104	SS23-HEX	270	0.00	Pass	367.8	[<Confirmed>]	

Sample 105: SSS18_SS24_SS05_SS32_SS30_SS12_SS23_HRS33_H08.fsa

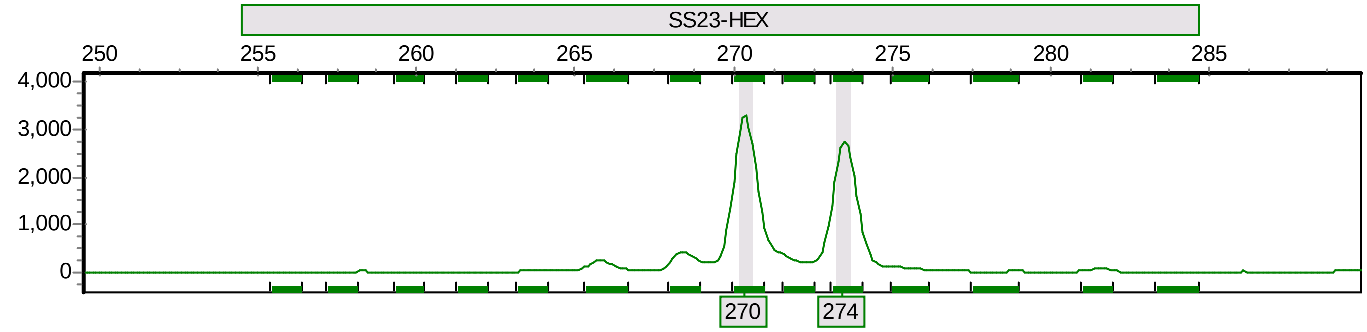

No	Size	Height	Area	Marker	Allele	Difference	Quality	Score	Allele Comments	Sample Comments
1	122.5	15086	95218	SS30-HEX	123	0.10	Pass	500.0	[<Confirmed>]	
2	124.5	10342	67901	SS30-HEX	125	0.20	Pass	500.0	[<Confirmed>]	
3	270.4	3277	27923	SS23-HEX	270	0.10	Pass	439.2	[<Confirmed>]	
4	273.5	2732	23771	SS23-HEX	274	0.10	Pass	334.8	[<Confirmed>]	

Sample 106: SSS18_SS24_SS05_SS32_SS30_SS12_SS23_HRS34_L10.fsa

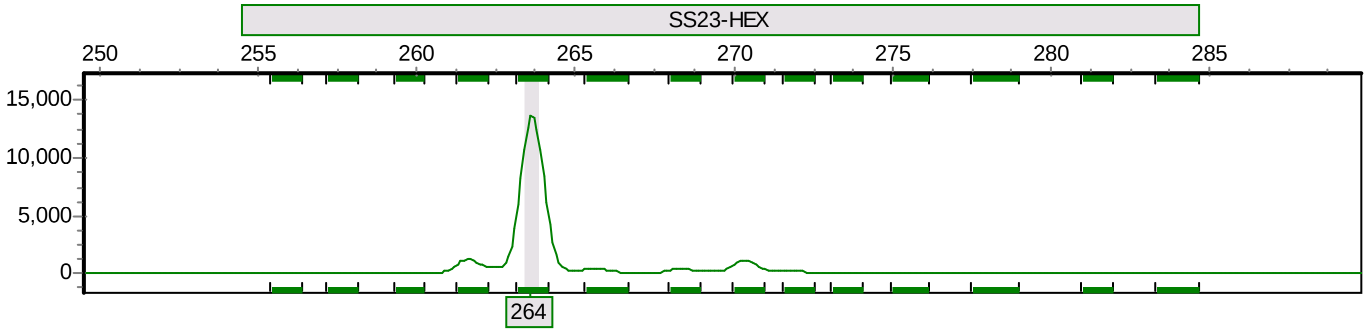

No	Size	Height	Area	Marker	Allele	Difference	Quality	Score	Allele Comments	Sample Comments
1	128.9	12740	80900	SS30-HEX	129	0.10	Pass	500.0	[<Confirmed>]	
2	145.9	6932	48520	SS30-HEX	145	0.10	Pass	500.0	[<Confirmed>]	
3	207.3	2321	17155	SS12-HEX	207	0.00	Pass	363.1	[<Confirmed>]	
4	217.4	1516	11428	SS12-HEX	217	0.00	Pass	188.0	[<Confirmed>]	
5	263.6	13580	112352	SS23-HEX	264	0.10	Pass	500.0	[<Confirmed>]	

Sample 107: SSS18_SS24_SS05_SS32_SS30_SS12_SS23_HRS35_P10.fsa

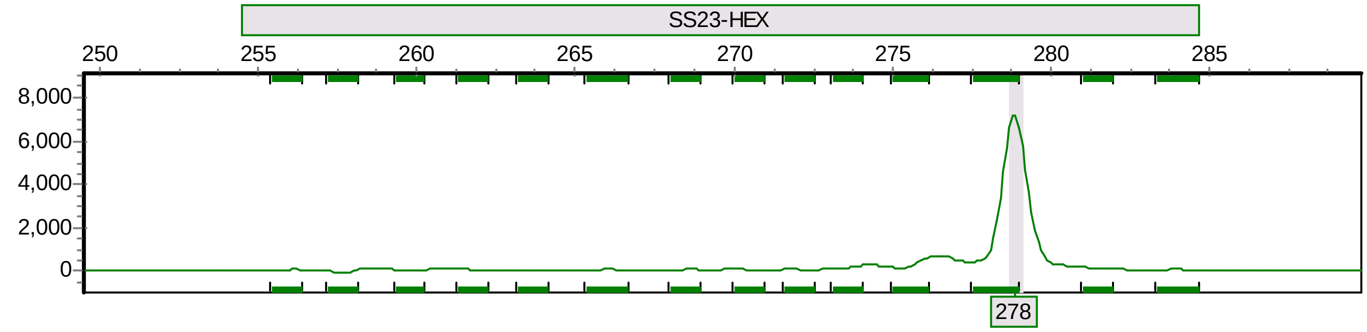

No	Size	Height	Area	Marker	Allele	Difference	Quality	Score	Allele Comments	Sample Comments
1	122.7	12223	78570	SS30-HEX	123	0.10	Pass	500.0	[<Confirmed>]	
2	143.8	4568	33553	SS30-HEX	143	0.10	Pass	500.0	[<Confirmed>]	
3	215.5	6430	48093	SS12-HEX	215	0.10	Pass	500.0	[<Confirmed>]	
4	219.5	4589	35555	SS12-HEX	219	0.10	Pass	500.0	[<Confirmed>]	
5	278.9	7142	63579	SS23-HEX	278	0.70	Pass	500.0	[<Confirmed>]	

Sample 108: SSS18_SS24_SS05_SS32_SS30_SS12_SS23_HRS37_J14.fsa

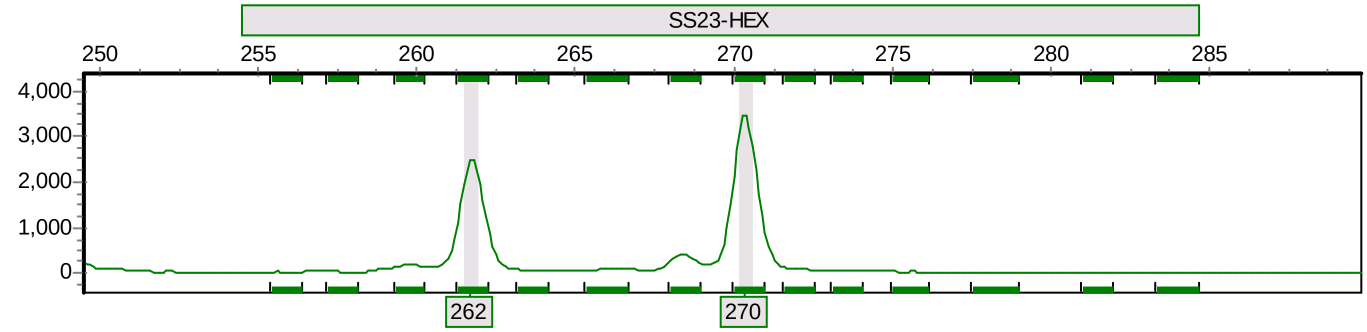

No	Size	Height	Area	Marker	Allele	Difference	Quality	Score	Allele Comments	Sample Comments
1	126.6	17794	118573	SS30-HEX	127	0.10	Pass	500.0	[<Confirmed>]	
2	128.8	9085	60452	SS30-HEX	129	0.00	Pass	500.0	[<Confirmed>]	
3	198.7	780	5949	SS12-HEX	199	0.40	Pass	63.0	[<Confirmed>]	
4	207.2	524	4301	SS12-HEX	207	0.10	Pass	31.5	[<Confirmed>]	
5	261.7	2494	21162	SS23-HEX	262	0.10	Pass	313.9	[<Confirmed>]	

Allele Report

6	270.4	3438	29788	SS23-HEX	270	0.10	Pass	450.6	[<Confirmed>]	

Sample 109: SSS18_SS24_SS05_SS32_SS30_SS12_SS23_HRS38_J08.fsa

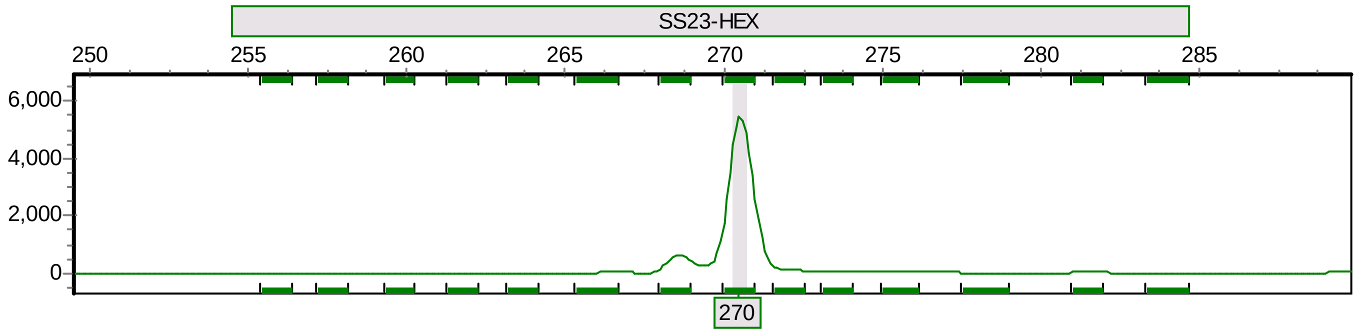

No	Size	Height	Area	Marker	Allele	Difference	Quality	Score	Allele Comments	Sample Comments
1	124.6	11886	78737	SS30-HEX	125	0.10	Pass	500.0	[<Confirmed>]	
2	135.0	8009	54160	SS30-HEX	135	0.10	Pass	500.0	[<Confirmed>]	
3	207.4	1289	10273	SS12-HEX	207	0.10	Pass	135.7	[<Confirmed>]	
4	270.5	5412	46647	SS23-HEX	270	0.00	Pass	500.0	[<Confirmed>]	

Sample 110: SSS18_SS24_SS05_SS32_SS30_SS12_SS23_HRS39_N08.fsa

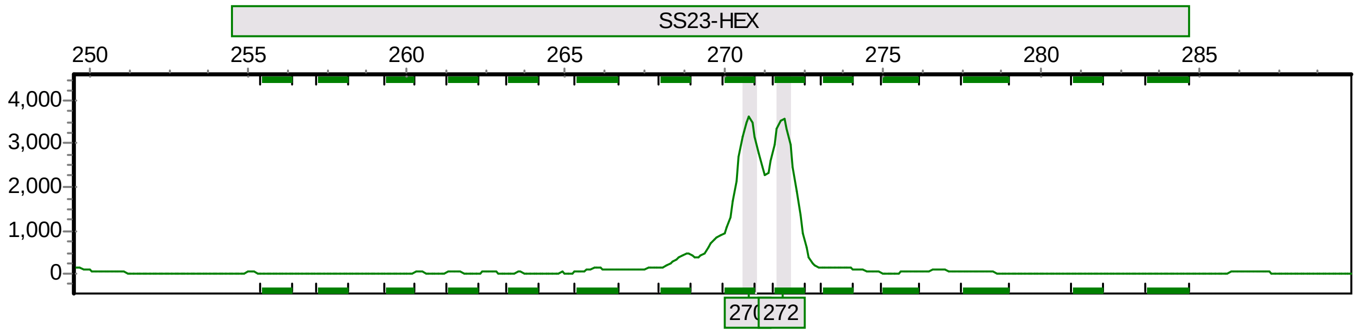

No	Size	Height	Area	Marker	Allele	Difference	Quality	Score	Allele Comments	Sample Comments
1	118.7	19991	127063	SS30-HEX	119	0.10	Pass	500.0	[<Confirmed>]	
2	120.8	16437	104085	SS30-HEX	121	0.20	Pass	500.0	[<Confirmed>]	
3	215.3	4865	36462	SS12-HEX	215	0.10	Pass	500.0	[<Confirmed>]	
4	270.8	3605	29810	SS23-HEX	270	0.30	Pass	378.7	[<Confirmed>]	
5	271.9	3569	31361	SS23-HEX	272	0.20	Pass	381.1	[<Confirmed>]	

Sample 111: SSS18_SS24_SS05_SS32_SS30_SS12_SS23_HRS40_A08.fsa

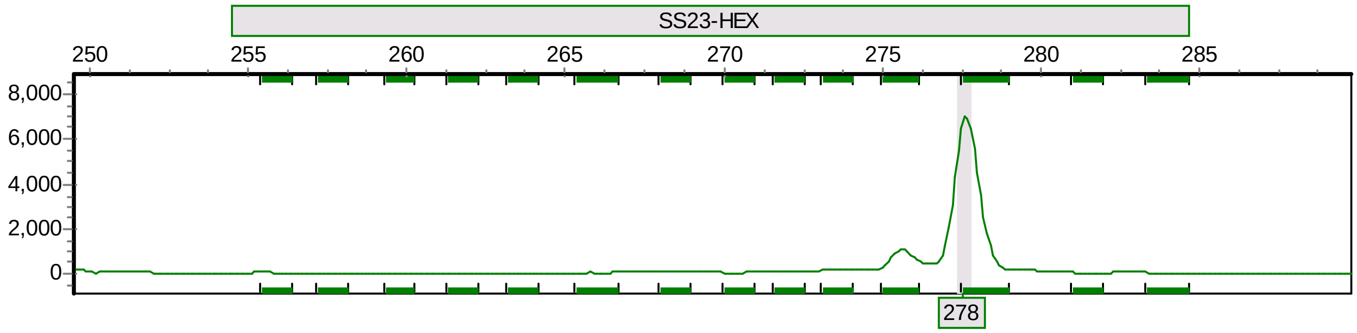

No	Size	Height	Area	Marker	Allele	Difference	Quality	Score	Allele Comments	Sample Comments
1	118.6	21564	134427	SS30-HEX	119	0.00	Pass	500.0	[<Confirmed>]	
2	128.8	14172	88516	SS30-HEX	129	0.00	Pass	500.0	[<Confirmed>]	
3	207.4	988	7206	SS12-HEX	207	0.10	Pass	106.1	[<Confirmed>]	
4	213.4	1083	8515	SS12-HEX	213	0.10	Pass	108.4	[<Confirmed>]	
5	277.6	7001	60433	SS23-HEX	278	0.60	Pass	500.0	[<Confirmed>]	

Allele Report

Sample 112: SSS18_SS24_SS05_SS32_SS30_SS12_SS23_HRS41_K08.fsa

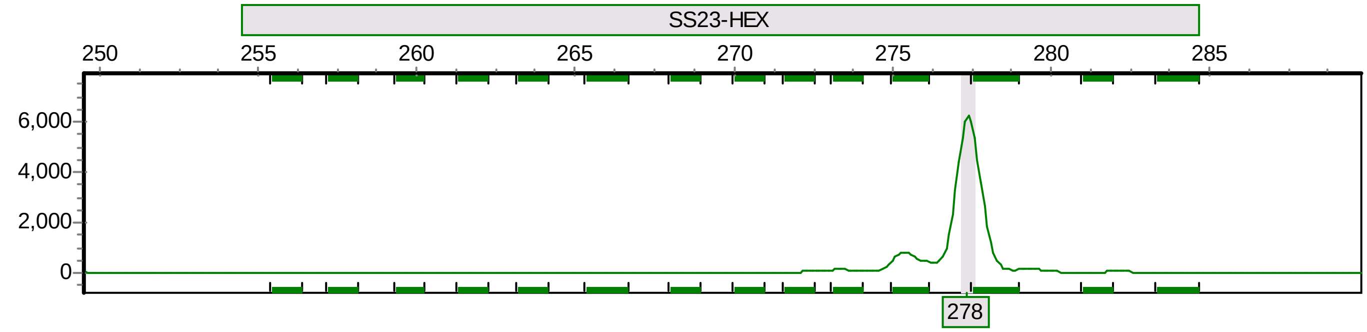

No	Size	Height	Area	Marker	Allele	Difference	Quality	Score	Allele Comments	Sample Comments
1	124.5	12468	79053	SS30-HEX	125	0.20	Pass	500.0	[<Confirmed>]	
2	141.3	6752	48626	SS30-HEX	141	0.10	Pass	500.0	[<Confirmed>]	
3	231.3	2368	18151	SS12-HEX	231	0.00	Pass	345.0	[<Confirmed>]	
4	277.4	6168	53203	SS23-HEX	278	1.00	Pass	500.0	[<Confirmed><Edited>]	

Sample 113:

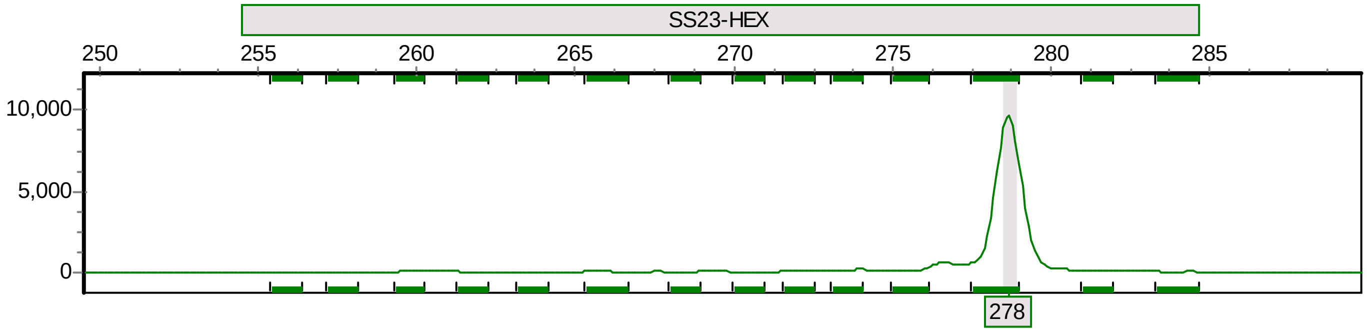

No	Size	Height	Area	Marker	Allele	Difference	Quality	Score	Allele Comments	Sample Comments
1	141.4	11111	82291	SS30-HEX	141	0.00	Pass	500.0	[<Confirmed>]	
2	205.4	13453	101836	SS12-HEX	205	0.00	Pass	500.0	[<Confirmed>]	
3	278.7	9619	87310	SS23-HEX	278	0.50	Pass	500.0	[<Confirmed>]	

Sample 114:

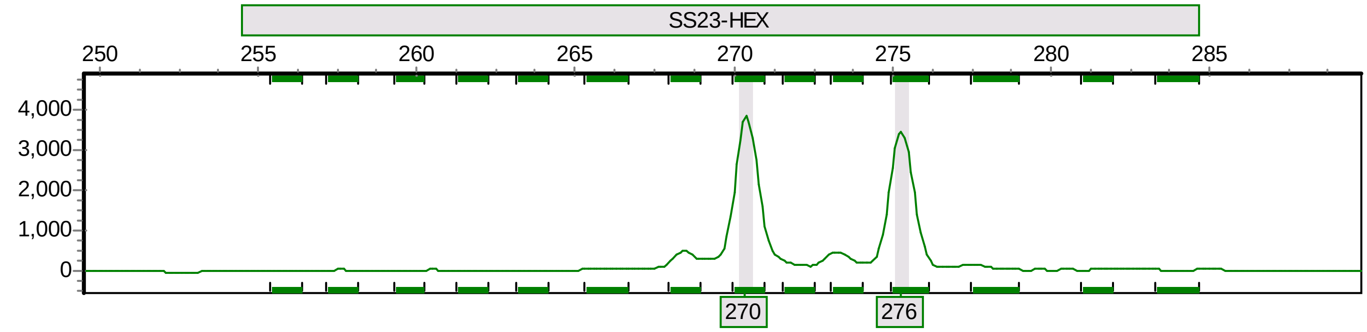

No	Size	Height	Area	Marker	Allele	Difference	Quality	Score	Allele Comments	Sample Comments
1	128.8	11060	70404	SS30-HEX	129	0.00	Pass	500.0	[<Confirmed>]	
2	139.0	8247	58306	SS30-HEX	139	0.10	Pass	500.0	[<Confirmed>]	
3	209.4	1062	7991	SS12-HEX	209	0.00	Pass	113.7	[<Confirmed>]	
4	270.4	3852	32566	SS23-HEX	270	0.10	Pass	500.0	[<Confirmed>]	
5	275.3	3492	30125	SS23-HEX	276	0.20	Pass	465.8	[<Confirmed>]	

Sample 115: SSS18_SS24_SS05_SS32_SS30_SS12_SS23_HTHL13_H02.fsa

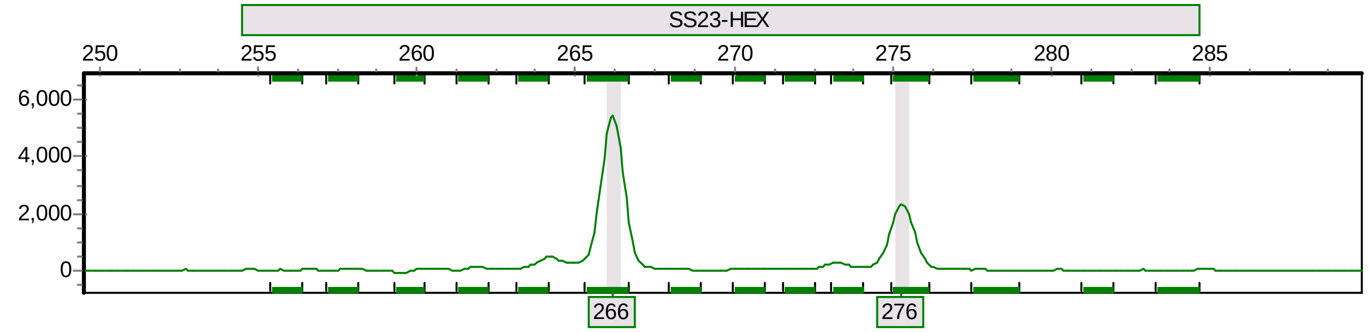

No	Size	Height	Area	Marker	Allele	Difference	Quality	Score	Allele Comments	Sample Comments
1	128.6	9851	62446	SS30-HEX	129	0.20	Pass	500.0	[<Confirmed>]	
2	148.3	4486	32261	SS30-HEX	149	0.00	Pass	500.0	[<Confirmed>]	
3	217.4	9552	69733	SS12-HEX	217	0.00	Pass	500.0	[<Confirmed>]	
4	266.2	5411	43370	SS23-HEX	266	0.20	Pass	500.0	[<Confirmed>]	
5	275.3	2303	20034	SS23-HEX	276	0.20	Pass	254.7	[<Confirmed>]	

Sample 116: SSS18_SS24_SS05_SS32_SS30_SS12_SS23_HTHL14_F16.fsa

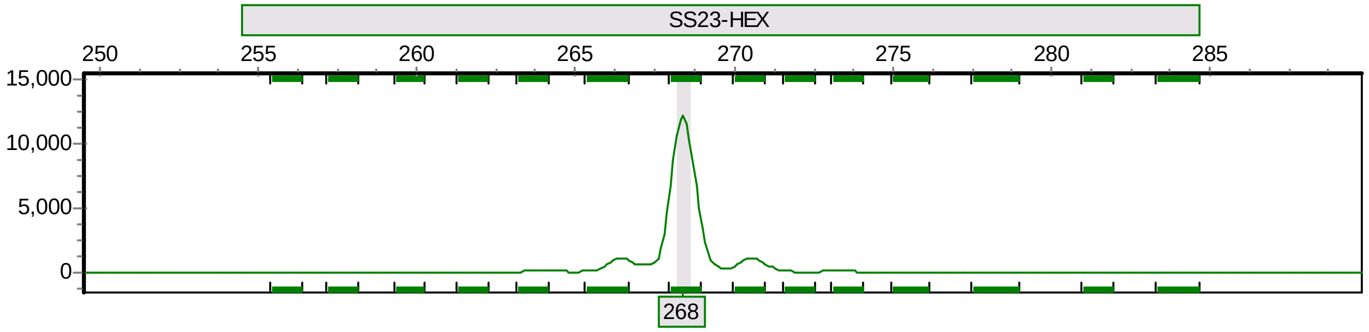

No	Size	Height	Area	Marker	Allele	Difference	Quality	Score	Allele Comments	Sample Comments
1	120.5	18306	123466	SS30-HEX	121	0.10	Pass	500.0	[<Confirmed>]	
2	124.4	11050	71785	SS30-HEX	125	0.30	Pass	500.0	[<Confirmed>]	
3	203.1	6504	49387	SS12-HEX	203	0.20	Pass	500.0	[<Confirmed>]	
4	268.4	12134	103755	SS23-HEX	268	0.10	Pass	500.0	[<Confirmed>]	

Sample 117: SSS18_SS24_SS05_SS32_SS30_SS12_SS23_HTHL15_F02.fsa

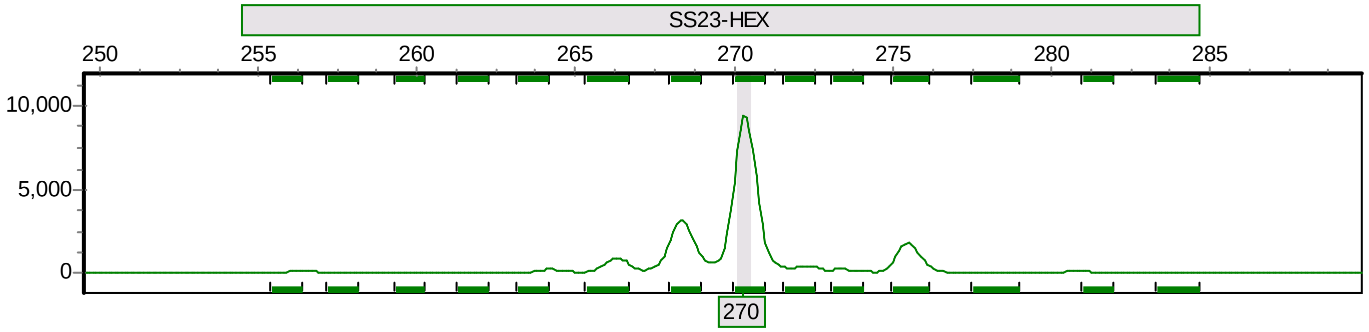

No	Size	Height	Area	Marker	Allele	Difference	Quality	Score	Allele Comments	Sample Comments
1	146.1	10573	72647	SS30-HEX	145	0.10	Pass	500.0	[<Confirmed>]	
2	215.5	14061	101410	SS12-HEX	215	0.10	Pass	500.0	[<Confirmed>]	
3	270.3	9344	75606	SS23-HEX	270	0.20	Pass	500.0	[<Confirmed>]	

Allele Report

Sample 118: SSS18_SS24_SS05_SS32_SS30_SS12_SS23_HTHL1_E14.fsa

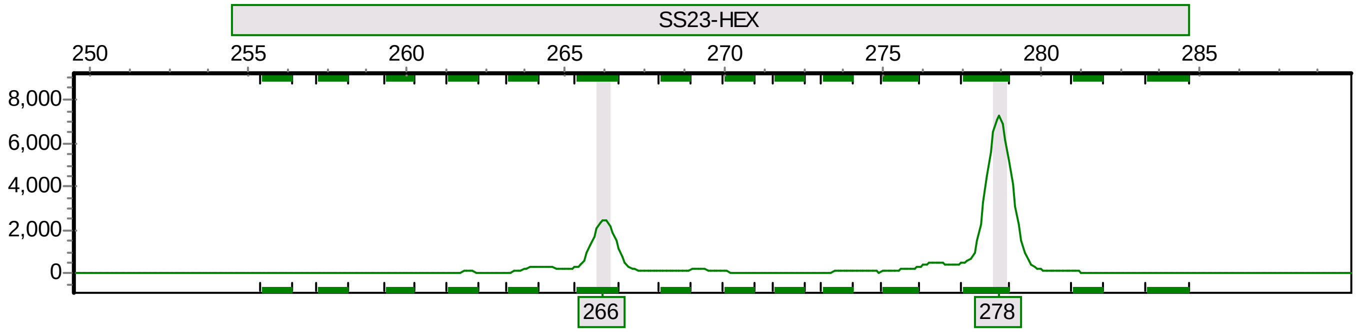

No	Size	Height	Area	Marker	Allele	Difference	Quality	Score	Allele Comments	Sample Comments
1	120.7	17050	118501	SS30-HEX	121	0.10	Pass	500.0	[<Confirmed>]	
2	122.5	10906	72911	SS30-HEX	123	0.10	Pass	500.0	[<Confirmed>]	
3	203.4	13829	103939	SS12-HEX	203	0.10	Pass	500.0	[<Confirmed>]	
4	266.2	2471	21619	SS23-HEX	266	0.20	Pass	287.4	[<Confirmed>]	
5	278.7	7258	66123	SS23-HEX	278	0.50	Pass	500.0	[<Confirmed>]	

Sample 119: SSS18_SS24_SS05_SS32_SS30_SS12_SS23_HTHL3_D06.fsa

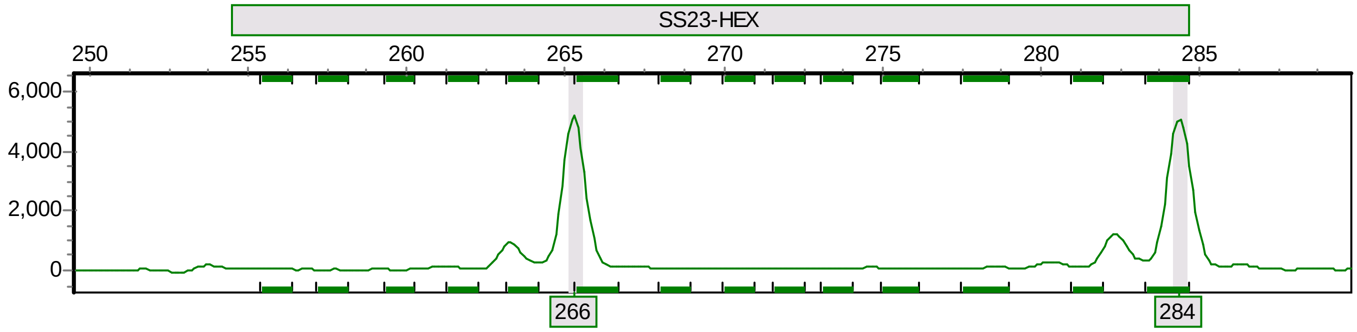

No	Size	Height	Area	Marker	Allele	Difference	Quality	Score	Allele Comments	Sample Comments
1	132.9	16499	105906	SS30-HEX	133	0.00	Pass	500.0	[<Confirmed>]	
2	205.5	12167	86256	SS12-HEX	205	0.10	Pass	500.0	[<Confirmed>]	
3	219.4	8453	60941	SS12-HEX	219	0.00	Pass	500.0	[<Confirmed>]	
4	265.3	5181	41184	SS23-HEX	266	0.70	Pass	500.0	[<Confirmed>]	
5	284.4	5098	44469	SS23-HEX	284	0.40	Pass	500.0	[<Confirmed>]	

Sample 120: SSS18_SS24_SS05_SS32_SS30_SS12_SS23_HTHL4_O16.fsa

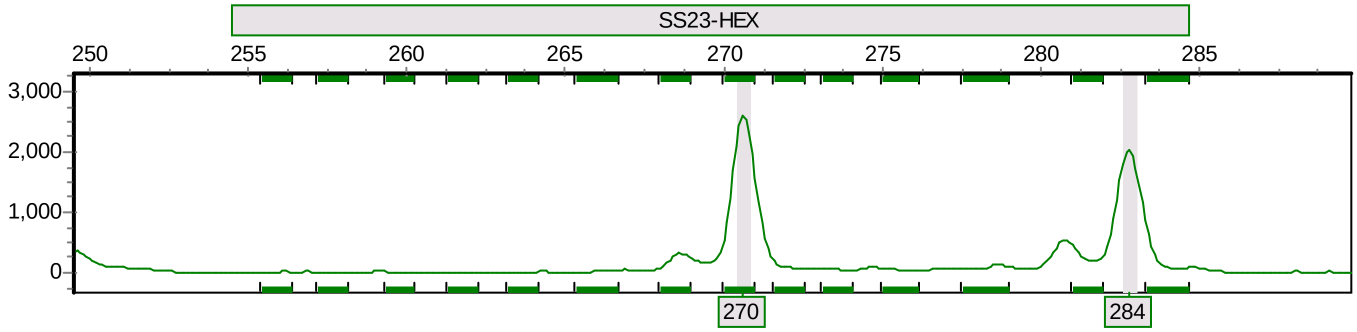

No	Size	Height	Area	Marker	Allele	Difference	Quality	Score	Allele Comments	Sample Comments
1	118.5	14367	92337	SS30-HEX	119	0.10	Pass	500.0	[<Confirmed>]	
2	132.8	7085	46473	SS30-HEX	133	0.10	Pass	500.0	[<Confirmed>]	
3	217.3	9118	70299	SS12-HEX	217	0.10	Pass	500.0	[<Confirmed>]	
4	270.6	2580	22137	SS23-HEX	270	0.10	Pass	319.5	[<Confirmed>]	
5	282.8	2025	18005	SS23-HEX	284	1.00	Pass	201.5	[<Confirmed><Edited>]	

Allele Report

Sample 121: SSS18_SS24_SS05_SS32_SS30_SS12_SS23_HTHL5_M14.fsa

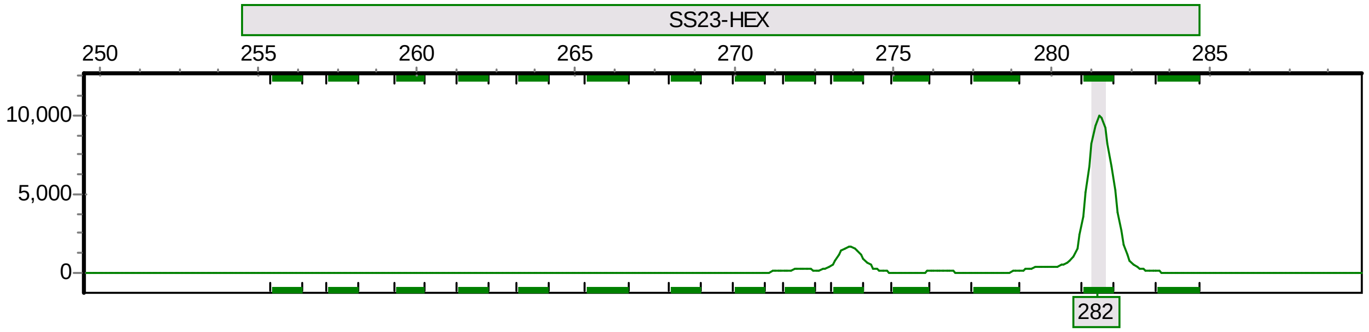

No	Size	Height	Area	Marker	Allele	Difference	Quality	Score	Allele Comments	Sample Comments
1	135.0	9037	61556	SS30-HEX	135	0.10	Pass	500.0	[<Confirmed>]	
2	143.8	5634	42360	SS30-HEX	143	0.10	Pass	500.0	[<Confirmed>]	
3	217.4	13704	106030	SS12-HEX	217	0.00	Pass	500.0	[<Confirmed>]	
4	281.5	9998	92636	SS23-HEX	282	0.00	Pass	500.0	[<Confirmed>]	

Sample 122: SSS18_SS24_SS05_SS32_SS30_SS12_SS23_HTHL6_P04.fsa

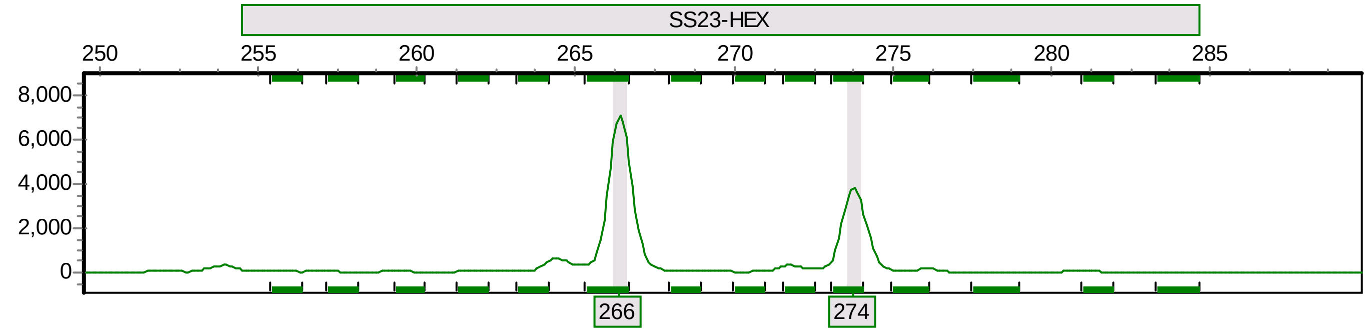

No	Size	Height	Area	Marker	Allele	Difference	Quality	Score	Allele Comments	Sample Comments
1	139.3	8757	61998	SS30-HEX	139	0.20	Pass	500.0	[<Confirmed>]	
2	152.5	4154	25165	SS30-HEX	153	0.10	Undetermined	500.0	[<Deleted>]	
3	154.4	4271	25802	SS30-HEX	155	0.10	Pass	500.0	[<Confirmed>]	
4	217.6	10905	80751	SS12-HEX	217	0.20	Pass	500.0	[<Confirmed>]	
5	266.4	7043	57731	SS23-HEX	266	0.40	Pass	500.0	[<Confirmed>]	
6	273.8	3846	32864	SS23-HEX	274	0.20	Pass	500.0	[<Confirmed>]	

Sample 123: SSS18_SS24_SS05_SS32_SS30_SS12_SS23_HTHL8_A04.fsa

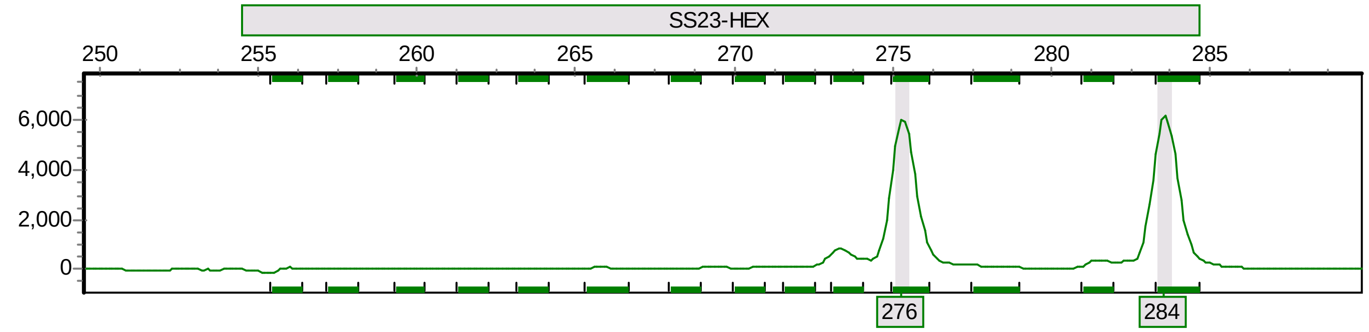

No	Size	Height	Area	Marker	Allele	Difference	Quality	Score	Allele Comments	Sample Comments
1	116.6	19073	121996	SS30-HEX	117	0.00	Pass	500.0	[<Confirmed>]	
2	213.4	6492	49646	SS12-HEX	213	0.10	Pass	500.0	[<Confirmed>]	
3	233.3	2328	18707	SS12-HEX	233	0.00	Pass	316.1	[<Confirmed>]	
4	275.3	6004	53170	SS23-HEX	276	0.20	Pass	500.0	[<Confirmed>]	
5	283.6	6158	56290	SS23-HEX	284	0.40	Pass	500.0	[<Confirmed>]	

Sample 124: SSS18_SS24_SS05_SS32_SS30_SS12_SS23_HTHL9_O10.fsa

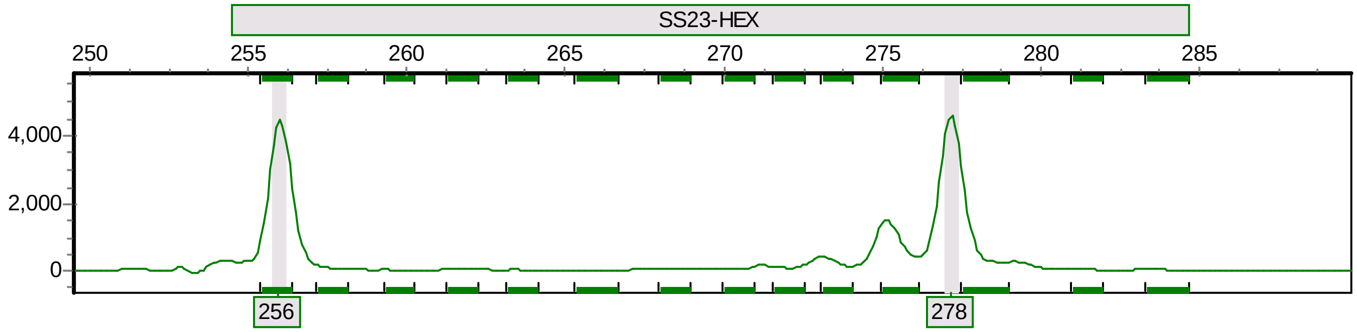

No	Size	Height	Area	Marker	Allele	Difference	Quality	Score	Allele Comments	Sample Comments
1	126.7	21556	139811	SS30-HEX	127	0.00	Pass	500.0	[<Confirmed>]	
2	205.4	17039	124277	SS12-HEX	205	0.00	Pass	500.0	[<Confirmed>]	
3	256.0	4459	36498	SS23-HEX	256	0.10	Pass	500.0	[<Confirmed>]	
4	277.2	4574	39689	SS23-HEX	278	1.00	Pass	500.0	[<Confirmed><Edited>]	
