# Supplementary material for: Genetic diversity of Schima superba based on physiological traits and SSR markers
Source: PLoS One. 2026 Apr 10;21(4):e0344465. doi: 10.1371/journal.pone.0344465 (PMC13068225; doi:10.1371/journal.pone.0344465)
Supplement: S1 File — (ZIP) [file pone.0344465.s003.zip › SS24.pdf]

Allele Report

Project Comments:

Sample 1: SSS18_SS24_SS05_SS32_SS30_SS12_SS23_HBB10_E06.fsa

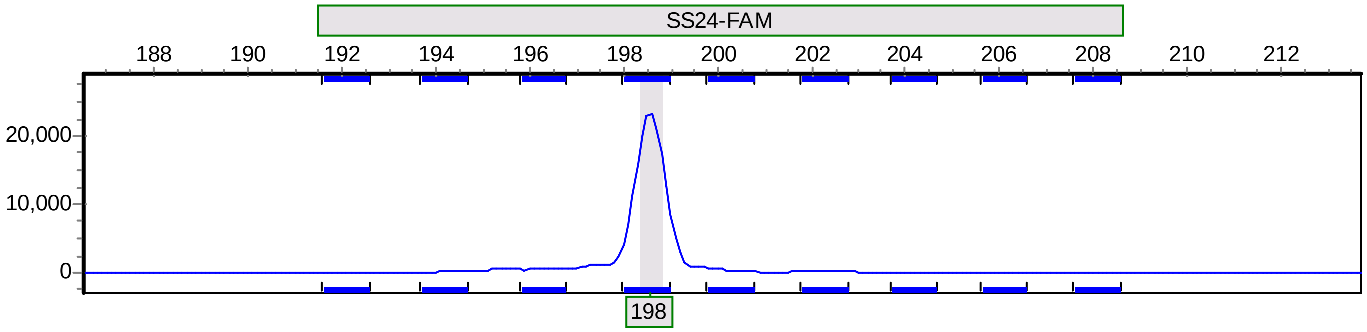

No	Size	Height	Area	Marker	Allele	Difference	Quality	Score	Allele Comments	Sample Comments
1	107.5	22433	144656	SSS18-FAM	108	0.00	Pass	500.0	[<Confirmed>]	
2	115.4	11212	74211	SSS18-FAM	116	0.10	Pass	500.0	[<Confirmed>]	
3	198.6	23446	165791	SS24-FAM	198	0.10	Pass	500.0	[<Confirmed>]	
4	245.6	11891	92595	SS05-FAM	245	0.00	Pass	500.0		
5	273.3	7950	66034	SS05-FAM	273	0.10	Pass	500.0		
6	326.0	6488	63580	SS32-FAM	326	0.00	Pass	500.0		

Sample 2: SSS18_SS24_SS05_SS32_SS30_SS12_SS23_HBB12-2_D12.fsa

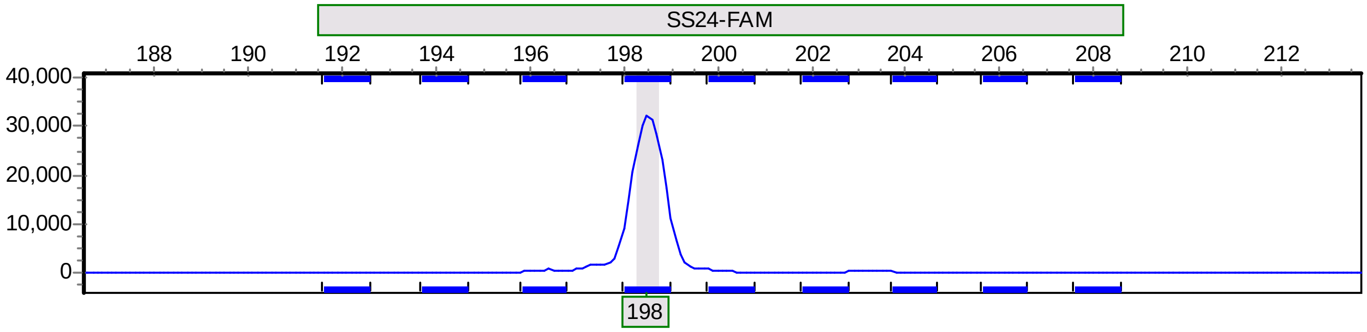

No	Size	Height	Area	Marker	Allele	Difference	Quality	Score	Allele Comments	Sample Comments
1	105.3	32994	265648	SSS18-FAM	106	0.10	Pass	500.0	[<SAT (Repaired)><Confirmed>]	
2	198.5	31943	249101	SS24-FAM	198	0.00	Pass	500.0	[<Confirmed>]	
3	247.5	13022	104882	SS05-FAM	247	0.10	Pass	500.0		
4	271.2	10048	85313	SS05-FAM	271	0.00	Pass	500.0		
5	325.9	7644	77135	SS32-FAM	326	0.10	Pass	500.0		

Sample 3: SSS18_SS24_SS05_SS32_SS30_SS12_SS23_HBB13_C18.fsa

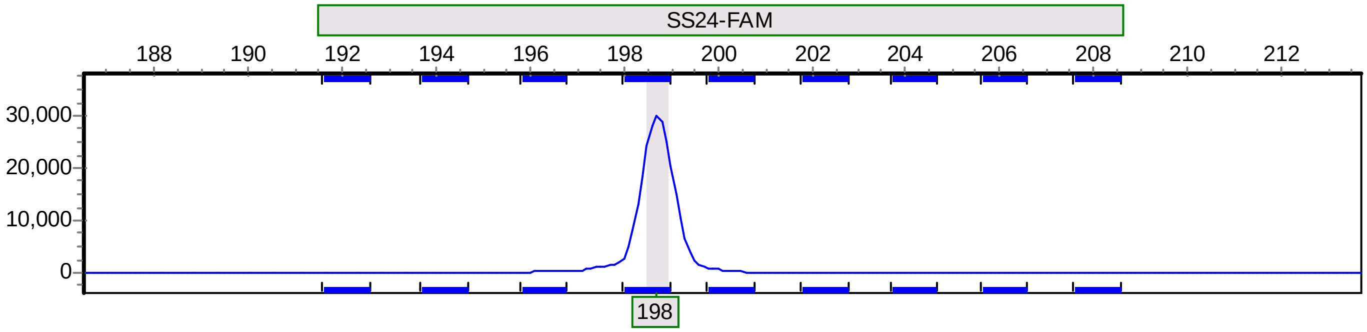

No	Size	Height	Area	Marker	Allele	Difference	Quality	Score	Allele Comments	Sample Comments
1	127.8	21392	149683	SSS18-FAM	128	0.00	Pass	500.0	[<Confirmed>]	
2	198.7	30005	228095	SS24-FAM	198	0.20	Pass	500.0	[<Confirmed>]	
3	245.7	30587	256988	SS05-FAM	245	0.10	Check	500.0		

No	Size	Height	Area	Marker	Allele	Difference	Quality	Score	Allele Comments	Sample Comments
4	321.9	4649	48409	SS32-FAM	322	0.10	Pass	484.8		
5	326.0	3450	36856	SS32-FAM	326	0.00	Pass	279.4		

Sample 4: SSS18_SS24_SS05_SS32_SS30_SS12_SS23_HBB14_G04.fsa

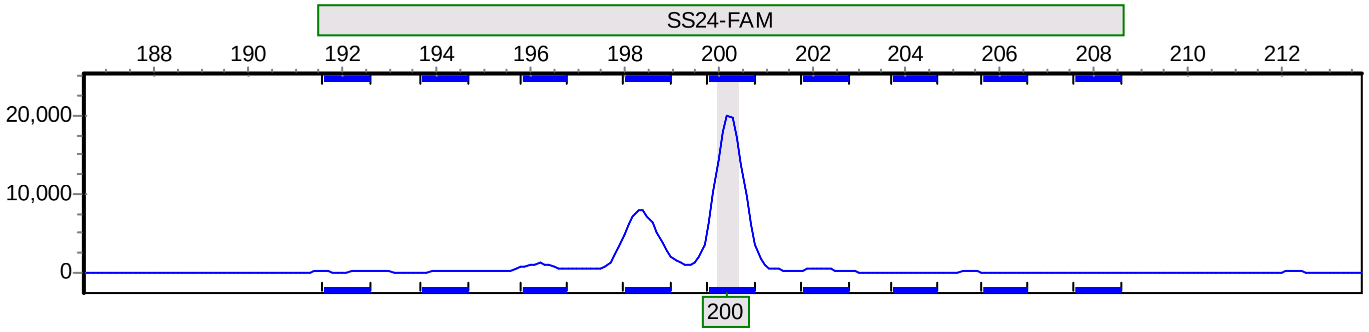

No	Size	Height	Area	Marker	Allele	Difference	Quality	Score	Allele Comments	Sample Comments
1	115.5	6479	42789	SSS18-FAM	116	0.00	Pass	500.0	[<Confirmed>]	
2	155.3	4132	25928	SSS18-FAM	156	0.00	Pass	500.0	[<Confirmed>]	
3	157.2	3848	24155	SSS18-FAM	158	0.00	Undetermined	500.0	[<Deleted>]	
4	159.1	2576	16187	SSS18-FAM	160	0.10	Undetermined	500.0	[<Deleted>]	
5	200.2	19768	136995	SS24-FAM	200	0.10	Pass	500.0	[<Confirmed>]	
6	245.1	15830	157076	SS05-FAM	245	0.50	Pass	500.0		
7	347.7	878	8849	SS32-FAM	348	0.00	Pass	42.4		

Sample 5: SSS18_SS24_SS05_SS32_SS30_SS12_SS23_HBB15_B08.fsa

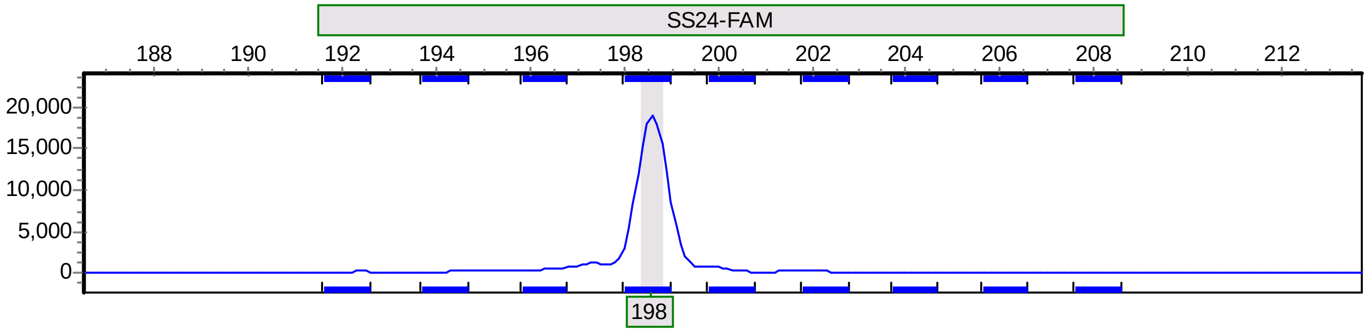

No	Size	Height	Area	Marker	Allele	Difference	Quality	Score	Allele Comments	Sample Comments
1	99.7	4422	74930	SSS18-FAM	100	0.00	Pass	115.4	[<Deleted>]	
2	117.4	12290	82363	SSS18-FAM	118	0.20	Pass	500.0	[<Confirmed>]	
3	198.6	18879	139795	SS24-FAM	198	0.10	Pass	500.0	[<Confirmed>]	
4	245.6	11253	90146	SS05-FAM	245	0.00	Pass	500.0		
5	326.0	3073	30982	SS32-FAM	326	0.00	Pass	288.0		
6	334.2	2102	21002	SS32-FAM	334	0.00	Pass	160.6		

Sample 6: SSS18_SS24_SS05_SS32_SS30_SS12_SS23_HBB16_G08.fsa

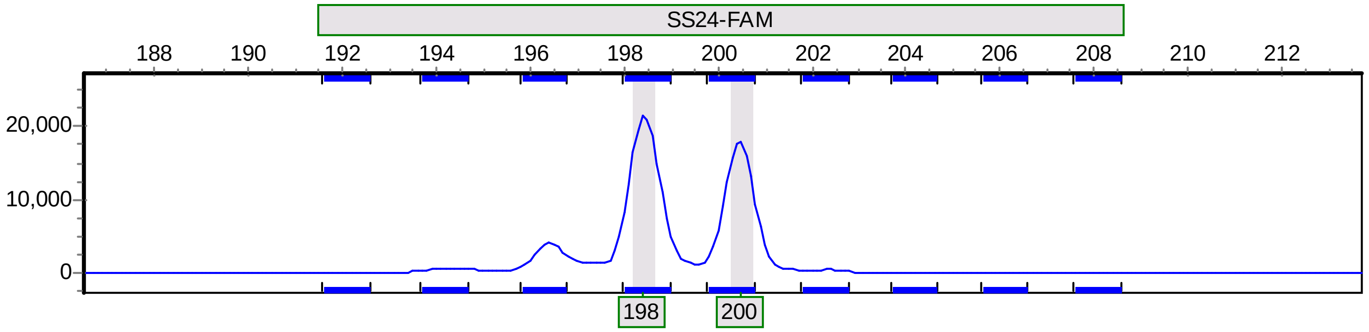

No	Size	Height	Area	Marker	Allele	Difference	Quality	Score	Allele Comments	Sample Comments

1	125.8	21045	141326	SSS18-FAM	126	0.10	Pass	500.0	[<Confirmed>]	
2	198.4	21191	158389	SS24-FAM	198	0.10	Pass	500.0	[<Confirmed>]	
3	200.5	17700	127268	SS24-FAM	200	0.20	Pass	500.0	[<Confirmed>]	
4	265.5	16740	134469	SS05-FAM	265	0.00	Pass	500.0		
5	347.8	1589	15569	SS32-FAM	348	0.10	Pass	98.0		
6	349.6	919	9614	SS32-FAM	350	0.00	Undetermined	39.5		
7	351.5	998	10057	SS32-FAM	352	0.00	Pass	52.0		

Sample 7: SSS18_SS24_SS05_SS32_SS30_SS12_SS23_HBB17_K16.fsa

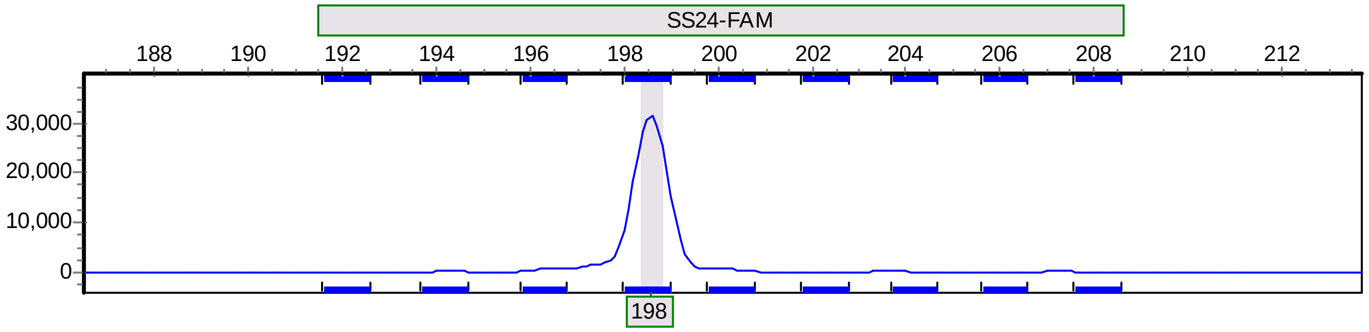

No	Size	Height	Area	Marker	Allele	Difference	Quality	Score	Allele Comments	Sample Comments
1	115.4	27650	192724	SSS18-FAM	116	0.10	Pass	500.0	[<Confirmed>]	
2	198.6	31253	259415	SS24-FAM	198	0.10	Pass	500.0	[<SAT (Repaired)><Confirmed>]	
3	247.6	11134	92625	SS05-FAM	247	0.00	Pass	500.0		
4	263.3	13033	113654	SS05-FAM	263	0.10	Pass	500.0		
5	325.8	7422	78030	SS32-FAM	326	0.20	Pass	500.0		

Sample 8: SSS18_SS24_SS05_SS32_SS30_SS12_SS23_HBB18_O06.fsa

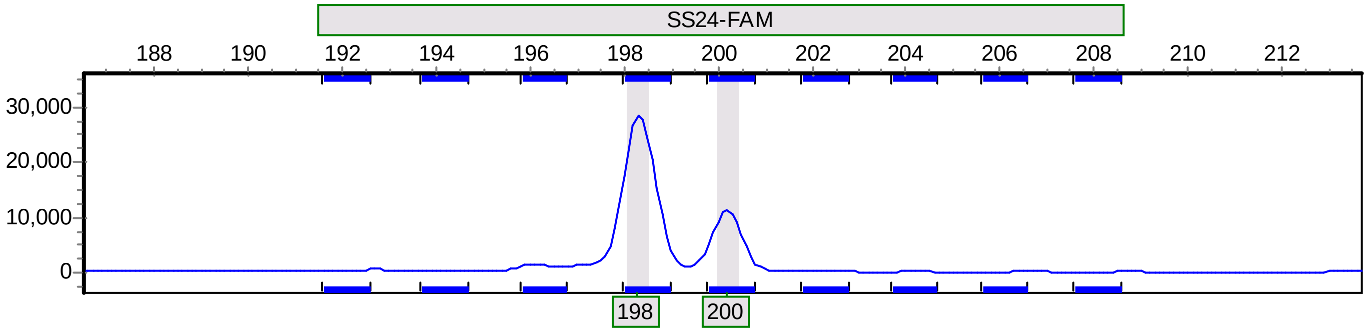

No	Size	Height	Area	Marker	Allele	Difference	Quality	Score	Allele Comments	Sample Comments
1	103.4	31017	208148	SSS18-FAM	104	0.00	Pass	500.0	[<Confirmed>]	
2	115.4	22473	144815	SSS18-FAM	116	0.10	Pass	500.0	[<Confirmed>]	
3	198.3	28501	220135	SS24-FAM	198	0.20	Pass	500.0	[<Confirmed>]	
4	200.2	11351	80233	SS24-FAM	200	0.10	Pass	500.0	[<Confirmed>]	
5	245.6	11627	117741	SS05-FAM	245	0.00	Pass	500.0		
6	247.6	9440	76359	SS05-FAM	247	0.00	Pass	500.0		
7	326.0	3048	31720	SS32-FAM	326	0.00	Pass	269.9		
8	332.1	2208	22199	SS32-FAM	332	0.00	Pass	162.3		

Allele Report

Sample 9: SSS18_SS24_SS05_SS32_SS30_SS12_SS23_HBB19_O08.fsa

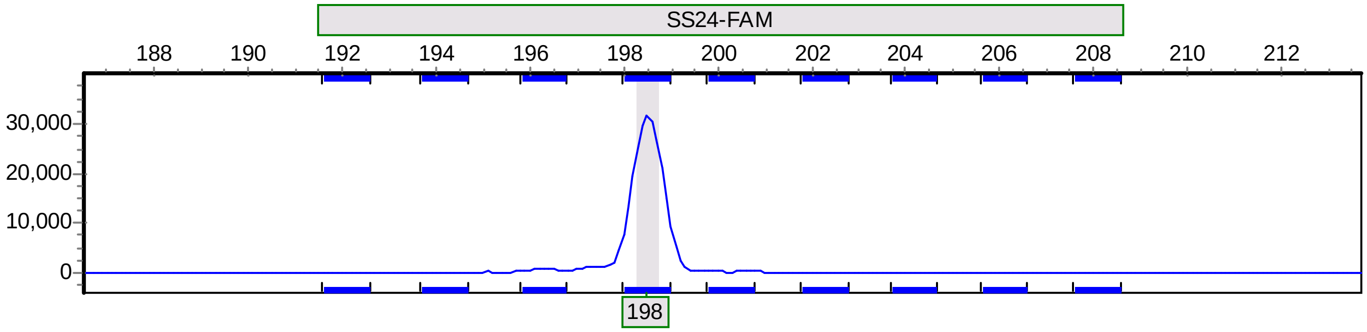

No	Size	Height	Area	Marker	Allele	Difference	Quality	Score	Allele Comments	Sample Comments
1	97.3	32829	252402	SSS18-FAM	98	0.10	Pass	500.0	[<SAT (Repaired)><Confirmed>]	
2	103.3	32687	226560	SSS18-FAM	104	0.10	Pass	500.0	[<SAT (Repaired)><Confirmed>]	
3	198.5	31359	229976	SS24-FAM	198	0.00	Pass	500.0	[<Confirmed>]	
4	245.6	25291	199112	SS05-FAM	245	0.00	Pass	500.0		
5	325.9	6077	58527	SS32-FAM	326	0.10	Pass	500.0		

Sample 10: SSS18_SS24_SS05_SS32_SS30_SS12_SS23_HBB1_B12.fsa

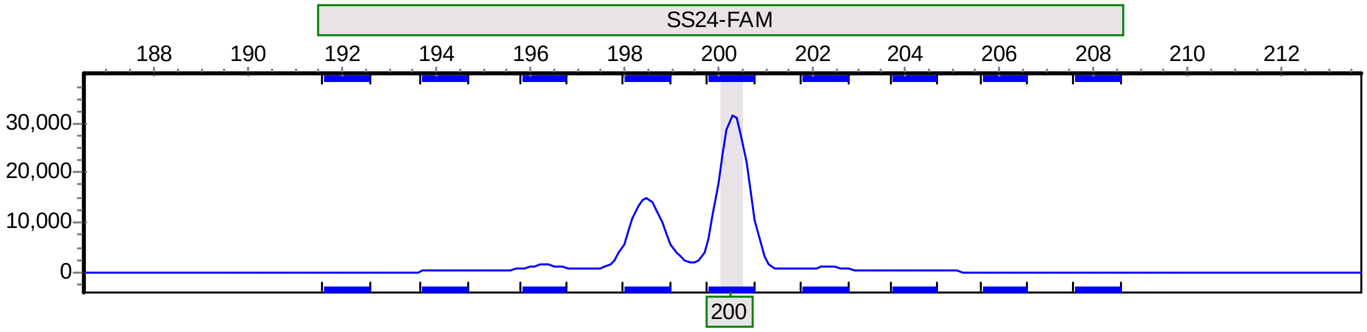

No	Size	Height	Area	Marker	Allele	Difference	Quality	Score	Allele Comments	Sample Comments
1	115.5	9149	61853	SSS18-FAM	116	0.00	Undetermined	500.0	[<Deleted>]	
2	136.0	14933	102295	SSS18-FAM	136	0.00	Pass	500.0	[<Confirmed>]	
3	138.1	14678	101180	SSS18-FAM	138	0.00	Pass	500.0	[<Confirmed>]	
4	200.3	31295	227330	SS24-FAM	200	0.00	Pass	500.0	[<Confirmed>]	
5	247.5	9646	82071	SS05-FAM	247	0.10	Pass	500.0		
6	326.0	4228	43050	SS32-FAM	326	0.00	Pass	409.0		
7	351.4	1318	13422	SS32-FAM	352	0.10	Pass	74.5		

Sample 11: SSS18_SS24_SS05_SS32_SS30_SS12_SS23_HBB20_F12.fsa

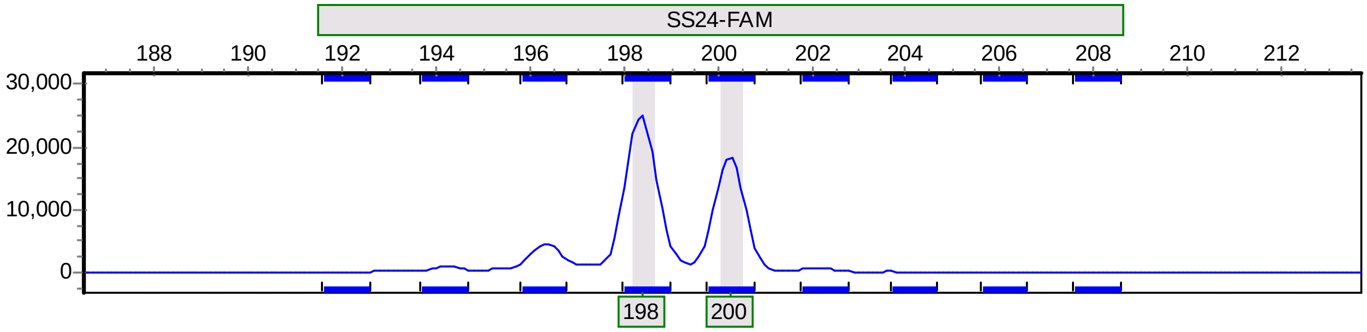

No	Size	Height	Area	Marker	Allele	Difference	Quality	Score	Allele Comments	Sample Comments
1	125.8	24737	171715	SSS18-FAM	126	0.10	Pass	500.0	[<Confirmed>]	
2	198.4	24888	191161	SS24-FAM	198	0.10	Pass	500.0	[<Confirmed>]	
3	200.3	18231	136718	SS24-FAM	200	0.00	Pass	500.0	[<Confirmed>]	

4	253.2	28133	237299	SS05-FAM	253	0.10	Pass	500.0		
5	323.8	3114	32439	SS32-FAM	324	0.10	Pass	242.3		

Sample 12: SSS18_SS24_SS05_SS32_SS30_SS12_SS23_HBB21_J10.fsa

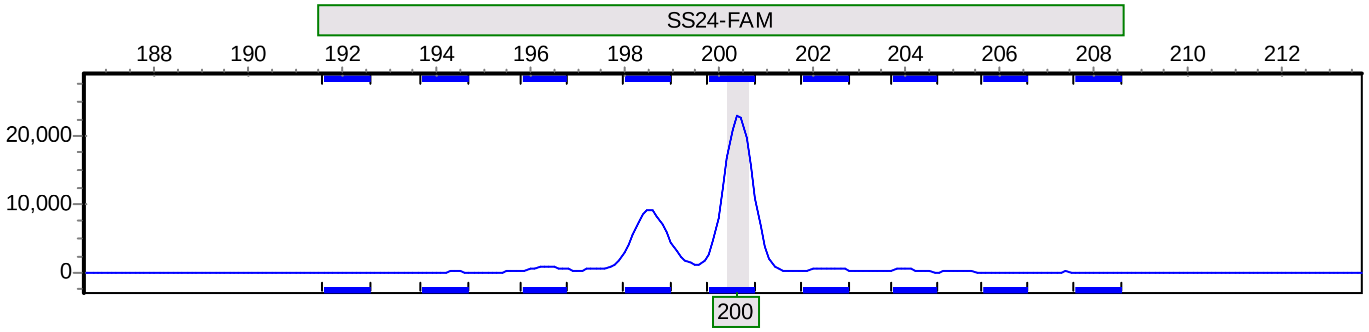

No	Size	Height	Area	Marker	Allele	Difference	Quality	Score	Allele Comments	Sample Comments
1	115.5	30799	202856	SSS18-FAM	116	0.00	Pass	500.0	[<Confirmed>]	
2	200.4	22962	161497	SS24-FAM	200	0.10	Pass	500.0	[<Confirmed>]	
3	245.1	16006	127636	SS05-FAM	245	0.50	Pass	500.0		
4	273.3	6102	53492	SS05-FAM	273	0.10	Pass	500.0		
5	325.9	3171	32505	SS32-FAM	326	0.10	Pass	260.2		
6	347.7	1334	13850	SS32-FAM	348	0.00	Pass	79.7		

Sample 13: SSS18_SS24_SS05_SS32_SS30_SS12_SS23_HBB22_K06.fsa

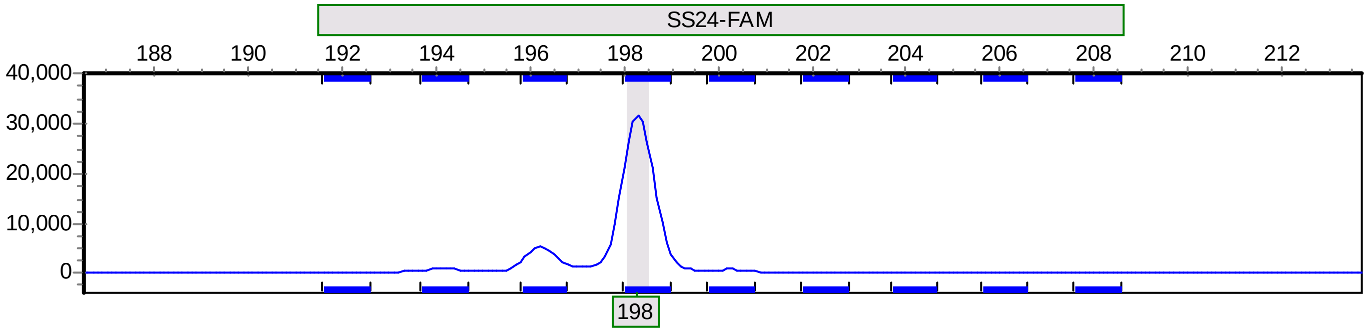

No	Size	Height	Area	Marker	Allele	Difference	Quality	Score	Allele Comments	Sample Comments
1	105.5	20850	133474	SSS18-FAM	106	0.10	Pass	500.0	[<Confirmed>]	
2	132.0	17341	115257	SSS18-FAM	132	0.10	Pass	500.0	[<Confirmed>]	
3	198.3	31446	241955	SS24-FAM	198	0.20	Pass	500.0	[<Confirmed>]	
4	263.5	17653	145198	SS05-FAM	263	0.10	Pass	500.0		
5	319.7	3963	39891	SS32-FAM	320	0.00	Pass	407.9		

Sample 14: SSS18_SS24_SS05_SS32_SS30_SS12_SS23_HBB23_B10.fsa

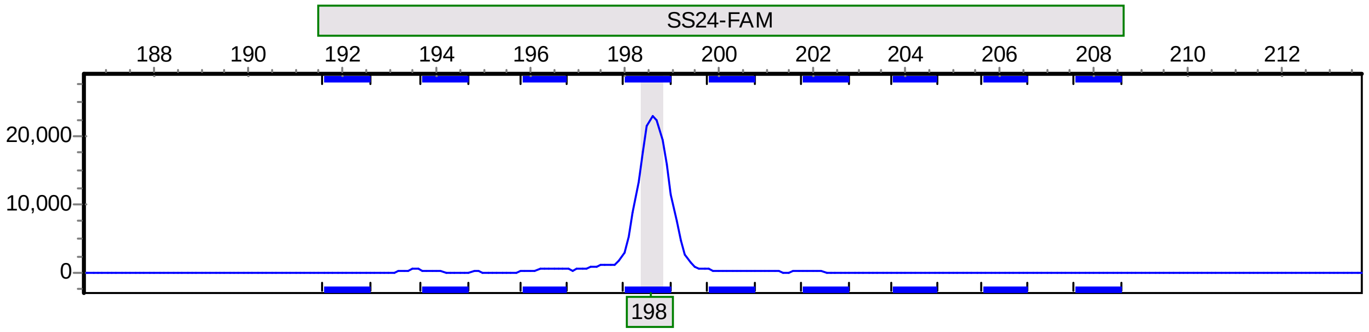

No	Size	Height	Area	Marker	Allele	Difference	Quality	Score	Allele Comments	Sample Comments
1	113.5	20682	138482	SSS18-FAM	114	0.00	Pass	500.0	[<Confirmed>]	
2	198.6	22738	167280	SS24-FAM	198	0.10	Pass	500.0	[<Confirmed>]	

Allele Report

3	247.6	11676	109592	SS05-FAM	247	0.00	Pass	500.0		
4	249.1	10904	92302	SS05-FAM	249	0.10	Pass	500.0		
5	326.0	3340	35433	SS32-FAM	326	0.00	Pass	292.8		
6	334.2	2026	21433	SS32-FAM	334	0.00	Pass	134.1		

Sample 15: SSS18_SS24_SS05_SS32_SS30_SS12_SS23_HBB25-1_F10.fsa

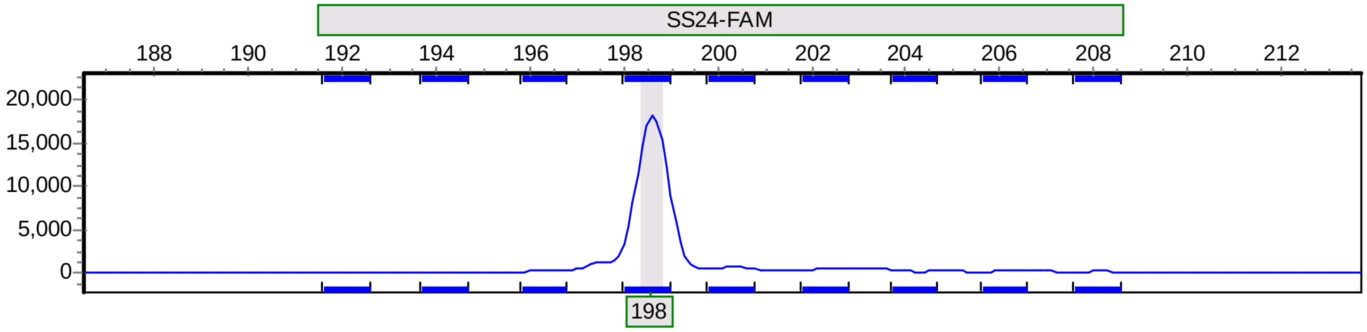

No	Size	Height	Area	Marker	Allele	Difference	Quality	Score	Allele Comments	Sample Comments
1	99.9	3965	66065	SSS18-FAM	100	0.20	Undetermined	104.7	[<Deleted>]	
2	105.4	2264	16029	SSS18-FAM	106	0.00	Pass	382.5	[<Confirmed>]	
3	107.4	1739	13034	SSS18-FAM	108	0.10	Pass	227.3	[<Confirmed>]	
4	198.6	18152	138164	SS24-FAM	198	0.10	Pass	500.0	[<Confirmed>]	
5	245.5	6182	52439	SS05-FAM	245	0.10	Pass	500.0		
6	273.1	6200	55968	SS05-FAM	273	0.10	Pass	500.0		
7	325.8	3793	40326	SS32-FAM	326	0.20	Pass	353.2		
8	327.9	2668	27556	SS32-FAM	328	0.10	Pass	210.1		

Sample 16: SSS18_SS24_SS05_SS32_SS30_SS12_SS23_HBB25-2_N12.fsa

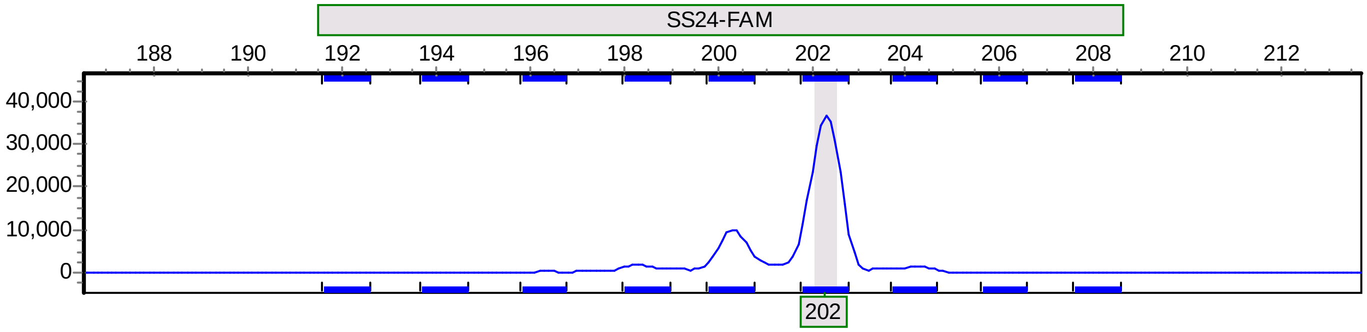

No	Size	Height	Area	Marker	Allele	Difference	Quality	Score	Allele Comments	Sample Comments
1	136.0	18104	124839	SSS18-FAM	136	0.00	Pass	500.0	[<Confirmed>]	
2	138.1	18757	129820	SSS18-FAM	138	0.00	Pass	500.0	[<Confirmed>]	
3	202.3	36360	267675	SS24-FAM	202	0.00	Pass	500.0	[<SAT (Repaired)><Confirmed>]	
4	257.6	14636	122020	SS05-FAM	257	0.10	Pass	500.0		
5	269.5	11958	98316	SS05-FAM	269	0.10	Pass	500.0		
6	343.9	1795	17556	SS32-FAM	344	0.10	Pass	129.9		

Sample 17: SSS18_SS24_SS05_SS32_SS30_SS12_SS23_HBB26_D08.fsa

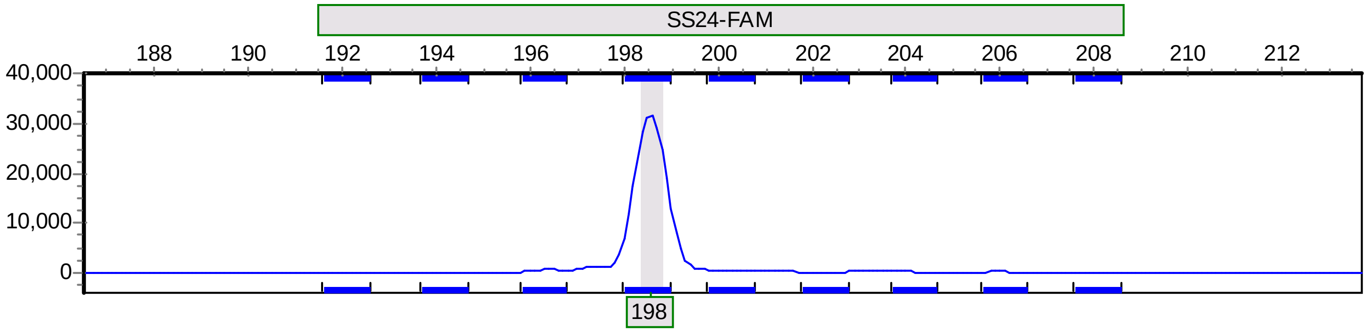

No	Size	Height	Area	Marker	Allele	Difference	Quality	Score	Allele Comments	Sample Comments
1	97.3	11882	87081	SSS18-FAM	98	0.10	Pass	500.0	[<Confirmed>]	
2	100.0	4529	79089	SSS18-FAM	100	0.30	Pass	91.1	[<Deleted>]	
3	198.6	31438	243361	SS24-FAM	198	0.10	Pass	500.0	[<Confirmed>]	
4	245.6	25982	208936	SS05-FAM	245	0.00	Pass	500.0		
5	319.7	4543	46675	SS32-FAM	320	0.00	Pass	476.3		
6	325.9	3825	37686	SS32-FAM	326	0.10	Pass	425.5		

Sample 18: SSS18_SS24_SS05_SS32_SS30_SS12_SS23_HBB27_H06.fsa

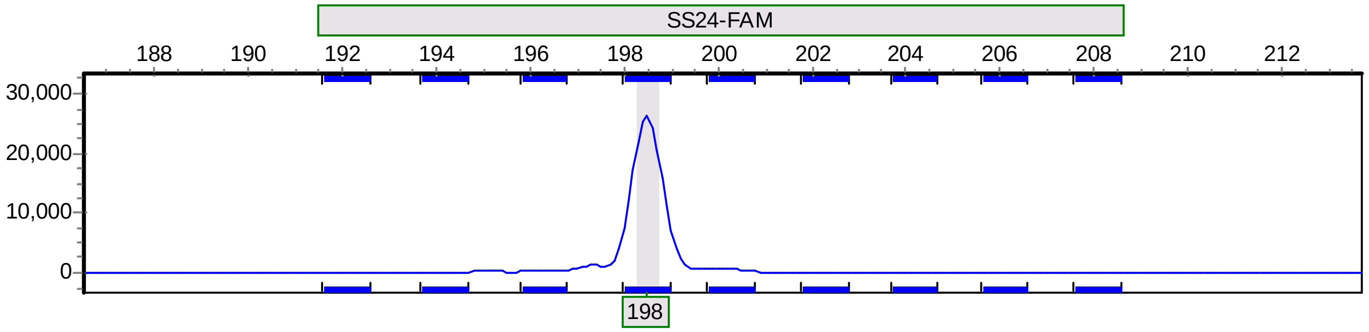

No	Size	Height	Area	Marker	Allele	Difference	Quality	Score	Allele Comments	Sample Comments
1	113.4	25595	169130	SSS18-FAM	114	0.10	Pass	500.0	[<Confirmed>]	
2	198.5	26077	189502	SS24-FAM	198	0.00	Pass	500.0	[<Confirmed>]	
3	247.5	8356	68517	SS05-FAM	247	0.10	Pass	500.0		
4	271.2	7684	66091	SS05-FAM	271	0.00	Pass	500.0		
5	319.8	4087	40770	SS32-FAM	320	0.10	Pass	430.3		
6	323.9	2742	28017	SS32-FAM	324	0.00	Pass	221.7		

Sample 19: SSS18_SS24_SS05_SS32_SS30_SS12_SS23_HBB28_L12.fsa

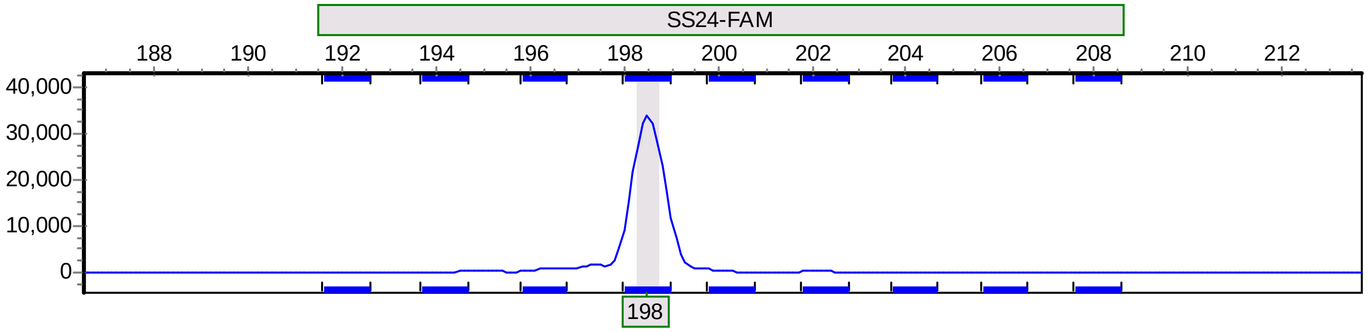

No	Size	Height	Area	Marker	Allele	Difference	Quality	Score	Allele Comments	Sample Comments
1	97.3	32868	281047	SSS18-FAM	98	0.10	Pass	500.0	[<SAT (Repaired)><Confirmed>]	
2	198.5	33827	258723	SS24-FAM	198	0.00	Pass	500.0	[<SAT (Repaired)><Confirmed>]	
3	245.6	26271	207800	SS05-FAM	245	0.00	Pass	500.0		

4	319.7	5433	54465	SS32-FAM	320	0.00	Pass	500.0		
5	325.9	4172	40619	SS32-FAM	326	0.10	Pass	444.7		

Sample 20: SSS18_SS24_SS05_SS32_SS30_SS12_SS23_HBB2_C08.fsa

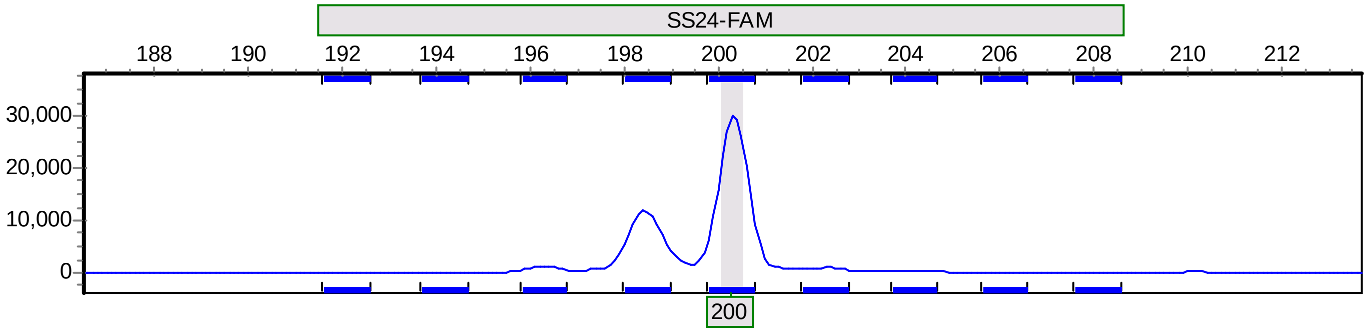

No	Size	Height	Area	Marker	Allele	Difference	Quality	Score	Allele Comments	Sample Comments
1	115.6	31673	222371	SSS18-FAM	116	0.10	Pass	500.0	[<Confirmed>]	
2	200.3	30010	212182	SS24-FAM	200	0.00	Pass	500.0	[<Confirmed>]	
3	273.2	6199	52681	SS05-FAM	273	0.00	Pass	500.0		
4	319.7	4517	44534	SS32-FAM	320	0.00	Pass	500.0		

Sample 21: SSS18_SS24_SS05_SS32_SS30_SS12_SS23_HBB30_L14.fsa

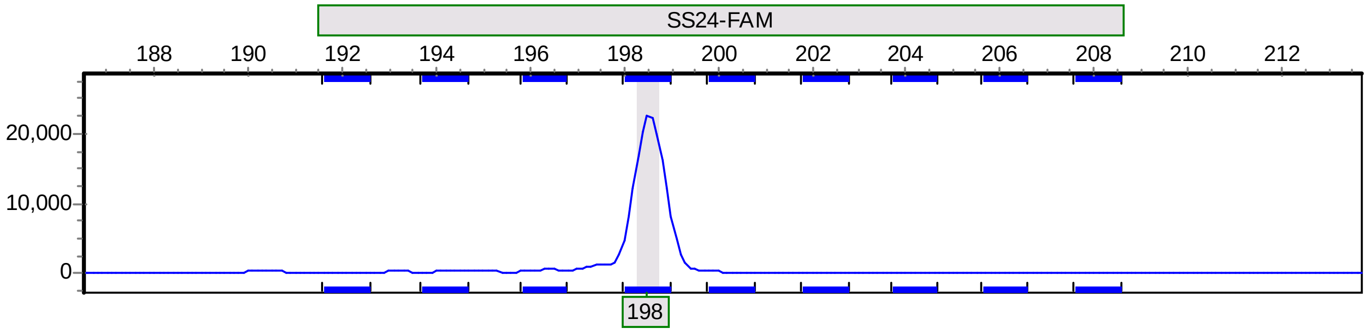

No	Size	Height	Area	Marker	Allele	Difference	Quality	Score	Allele Comments	Sample Comments
1	113.5	29992	200448	SSS18-FAM	114	0.00	Pass	500.0	[<Confirmed>]	
2	198.5	22546	165741	SS24-FAM	198	0.00	Pass	500.0	[<Confirmed>]	
3	245.6	9791	79228	SS05-FAM	245	0.00	Pass	500.0		
4	263.2	12109	101728	SS05-FAM	263	0.20	Pass	500.0		
5	319.6	4230	43469	SS32-FAM	320	0.10	Pass	442.1		
6	325.8	2715	28346	SS32-FAM	326	0.20	Pass	225.5		

Sample 22: SSS18_SS24_SS05_SS32_SS30_SS12_SS23_HBB31_P08.fsa

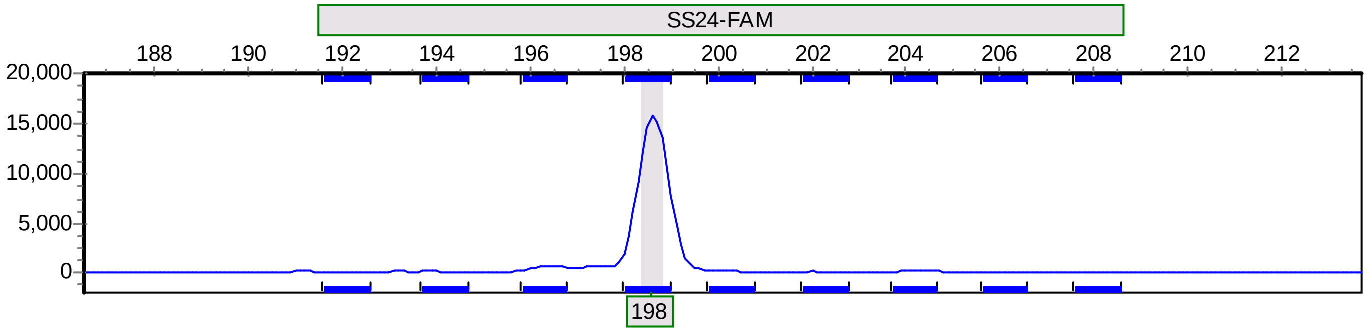

No	Size	Height	Area	Marker	Allele	Difference	Quality	Score	Allele Comments	Sample Comments
1	115.5	13473	87350	SSS18-FAM	116	0.00	Pass	500.0	[<Confirmed>]	
2	198.6	15754	113887	SS24-FAM	198	0.10	Pass	500.0	[<Confirmed>]	
3	249.3	17660	139808	SS05-FAM	249	0.10	Pass	500.0		

4	323.8	3176	32075	SS32-FAM	324	0.10	Pass	290.4		
5	327.9	2374	23498	SS32-FAM	328	0.10	Pass	204.1		

Sample 23: SSS18_SS24_SS05_SS32_SS30_SS12_SS23_HBB32_B16.fsa

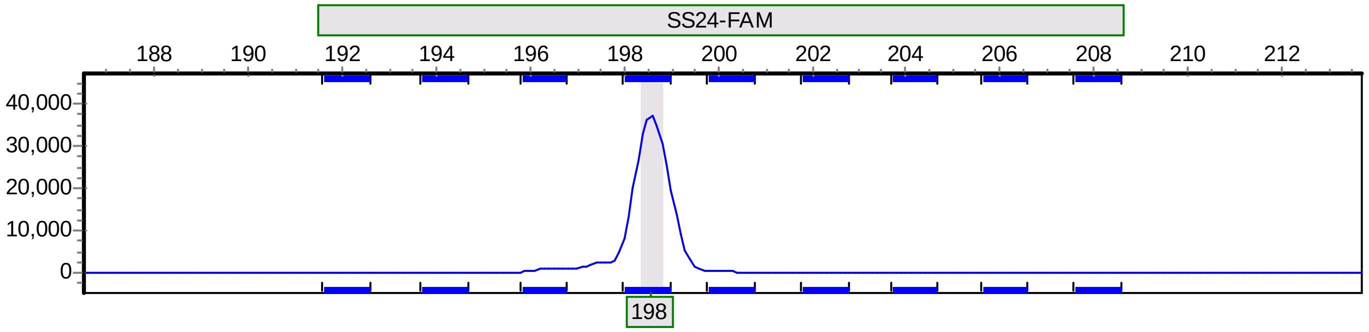

No	Size	Height	Area	Marker	Allele	Difference	Quality	Score	Allele Comments	Sample Comments
1	107.2	26997	210201	SSS18-FAM	108	0.30	Pass	500.0	[<SAT (Repaired)><Confirmed>]	
2	121.4	18867	127970	SSS18-FAM	122	0.30	Pass	500.0	[<Confirmed>]	
3	198.6	37136	305650	SS24-FAM	198	0.10	Pass	500.0	[<SAT (Repaired)><Confirmed>]	
4	245.6	31988	293092	SS05-FAM	245	0.00	Check	500.0		
5	323.9	7167	73545	SS32-FAM	324	0.00	Pass	500.0		
6	325.9	5154	51633	SS32-FAM	326	0.10	Pass	500.0		

Sample 24: SSS18_SS24_SS05_SS32_SS30_SS12_SS23_HBB33_N14.fsa

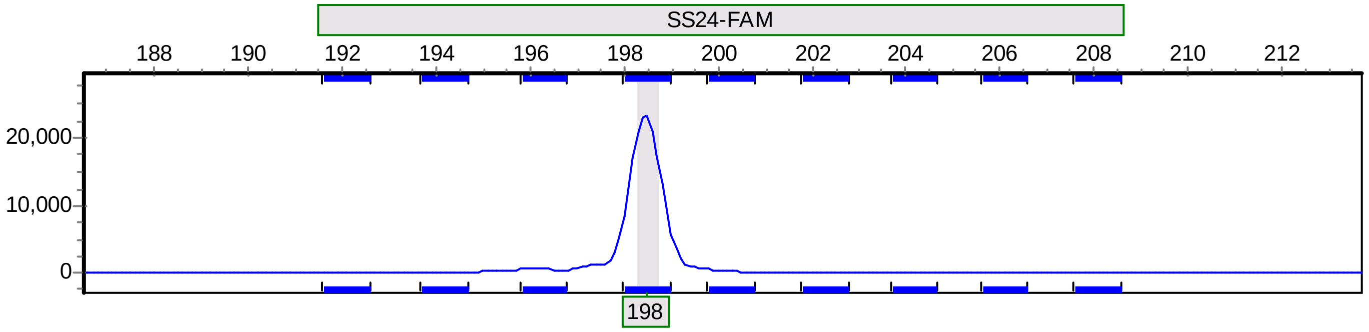

No	Size	Height	Area	Marker	Allele	Difference	Quality	Score	Allele Comments	Sample Comments
1	115.4	11208	76229	SSS18-FAM	116	0.10	Pass	500.0	[<Confirmed>]	
2	127.7	9867	68971	SSS18-FAM	128	0.10	Pass	500.0	[<Confirmed>]	
3	198.5	23255	176397	SS24-FAM	198	0.00	Pass	500.0	[<Confirmed>]	
4	245.5	20860	173206	SS05-FAM	245	0.10	Pass	500.0		
5	325.7	2831	30431	SS32-FAM	326	0.30	Pass	213.2		
6	331.9	1674	17851	SS32-FAM	332	0.20	Pass	101.9		

Sample 25: SSS18_SS24_SS05_SS32_SS30_SS12_SS23_HBB34_P14.fsa

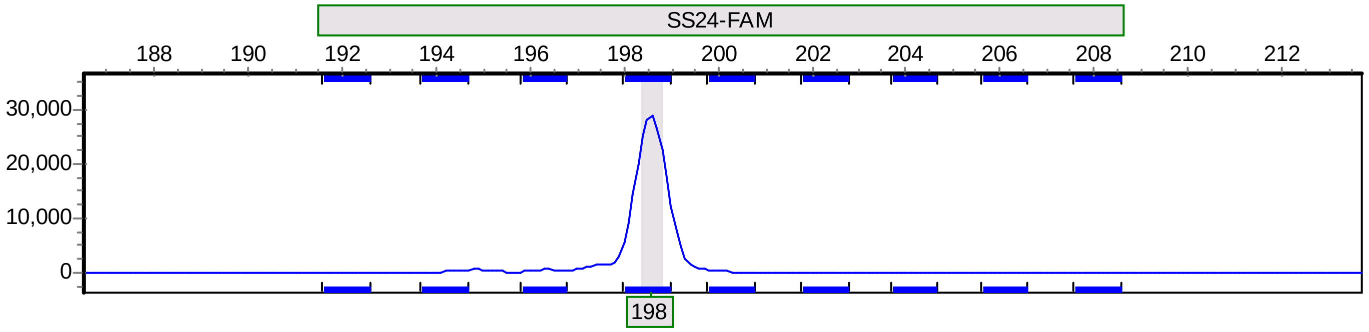

No	Size	Height	Area	Marker	Allele	Difference	Quality	Score	Allele Comments	Sample Comments
1	113.6	16473	110871	SSS18-FAM	114	0.10	Pass	500.0	[<Confirmed>]	

2	127.8	14320	95793	SSS18-FAM	128	0.00	Pass	500.0	[<Confirmed>]	
3	198.6	28695	215748	SS24-FAM	198	0.10	Pass	500.0	[<Confirmed>]	
4	245.6	27089	220440	SS05-FAM	245	0.00	Pass	500.0		
5	325.9	3368	35423	SS32-FAM	326	0.10	Pass	293.1		
6	332.0	2437	25792	SS32-FAM	332	0.10	Pass	179.7		

Sample 26: SSS18_SS24_SS05_SS32_SS30_SS12_SS23_HBB35_C16.fsa

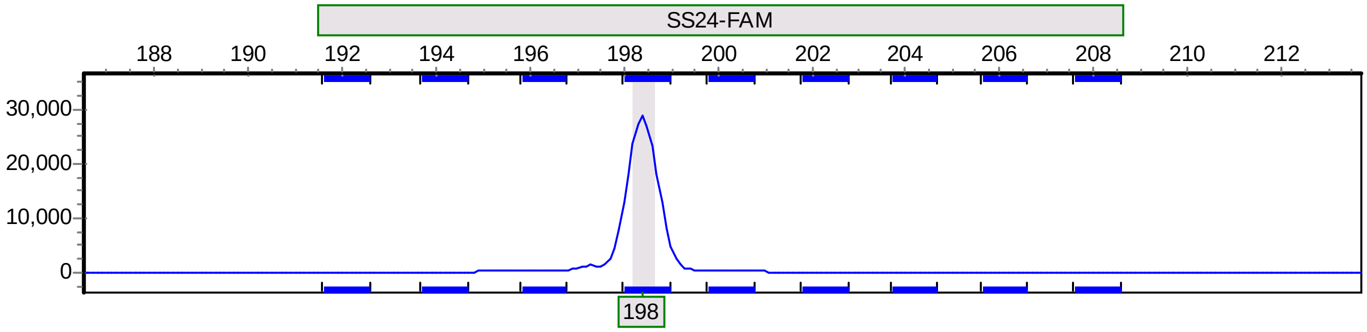

No	Size	Height	Area	Marker	Allele	Difference	Quality	Score	Allele Comments	Sample Comments
1	97.3	24858	178877	SSS18-FAM	98	0.10	Pass	500.0	[<Confirmed>]	
2	107.4	25589	172337	SSS18-FAM	108	0.10	Pass	500.0	[<Confirmed>]	
3	198.4	28760	210581	SS24-FAM	198	0.10	Pass	500.0	[<Confirmed>]	
4	249.0	7044	59532	SS05-FAM	249	0.20	Pass	500.0		
5	265.2	10880	91149	SS05-FAM	265	0.30	Pass	500.0		
6	319.7	4072	41102	SS32-FAM	320	0.00	Pass	413.3		
7	325.9	3202	33381	SS32-FAM	326	0.10	Pass	290.6		

Sample 27: SSS18_SS24_SS05_SS32_SS30_SS12_SS23_HBB36_G06.fsa

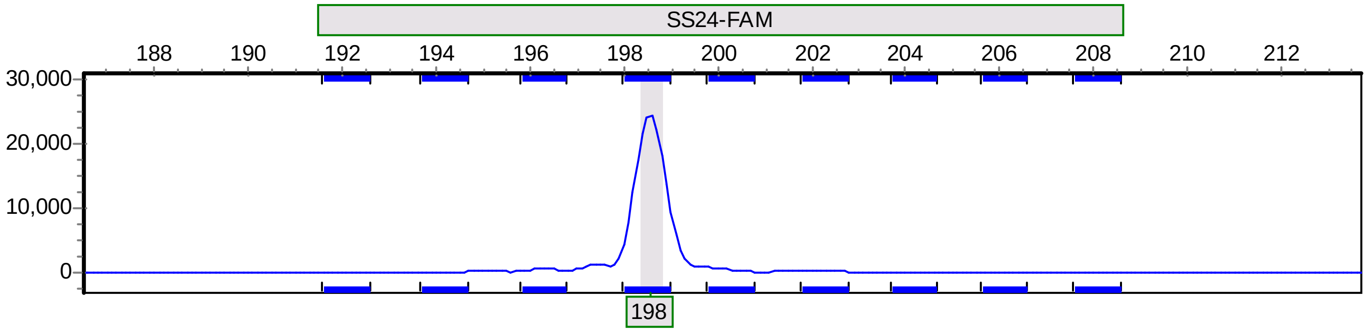

No	Size	Height	Area	Marker	Allele	Difference	Quality	Score	Allele Comments	Sample Comments
1	99.4	4419	74886	SSS18-FAM	100	0.30	Undetermined	116.2	[<Deleted>]	
2	115.5	10544	71276	SSS18-FAM	116	0.00	Pass	500.0	[<Confirmed>]	
3	127.7	9203	63774	SSS18-FAM	128	0.10	Pass	500.0	[<Confirmed>]	
4	198.6	24236	178903	SS24-FAM	198	0.10	Pass	500.0	[<Confirmed>]	
5	245.6	13547	107281	SS05-FAM	245	0.00	Pass	500.0		
6	319.8	3903	38604	SS32-FAM	320	0.10	Pass	408.1		
7	325.9	2929	29304	SS32-FAM	326	0.10	Pass	271.9		

Allele Report

Sample 28: SSS18_SS24_SS05_SS32_SS30_SS12_SS23_HBB37_N10.fsa

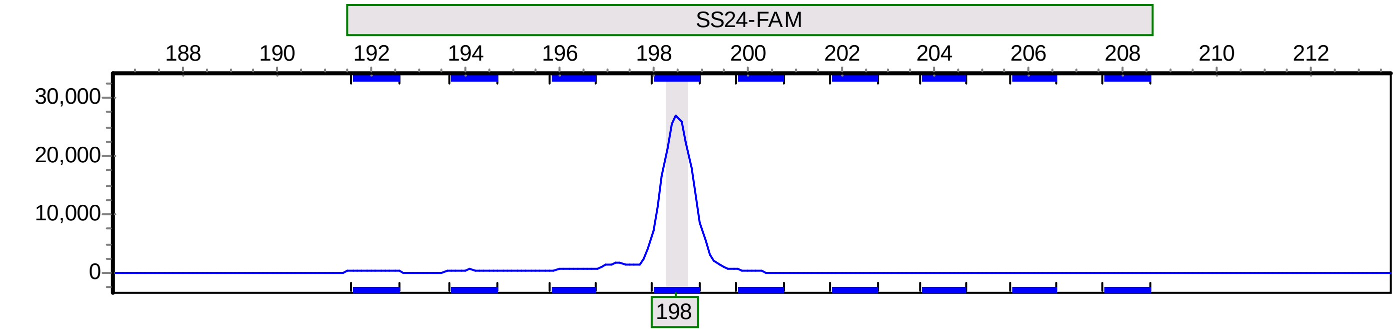

No	Size	Height	Area	Marker	Allele	Difference	Quality	Score	Allele Comments	Sample Comments
1	97.4	30550	221360	SSS18-FAM	98	0.00	Pass	500.0	[<Confirmed>]	
2	105.5	31457	216413	SSS18-FAM	106	0.10	Pass	500.0	[<Confirmed>]	
3	198.5	26642	199816	SS24-FAM	198	0.00	Pass	500.0	[<Confirmed>]	
4	247.6	12812	113070	SS05-FAM	247	0.00	Pass	500.0		
5	249.1	11265	93999	SS05-FAM	249	0.10	Pass	500.0		
6	327.9	5100	51792	SS32-FAM	328	0.10	Pass	500.0		

Sample 29: SSS18_SS24_SS05_SS32_SS30_SS12_SS23_HBB38_M08.fsa

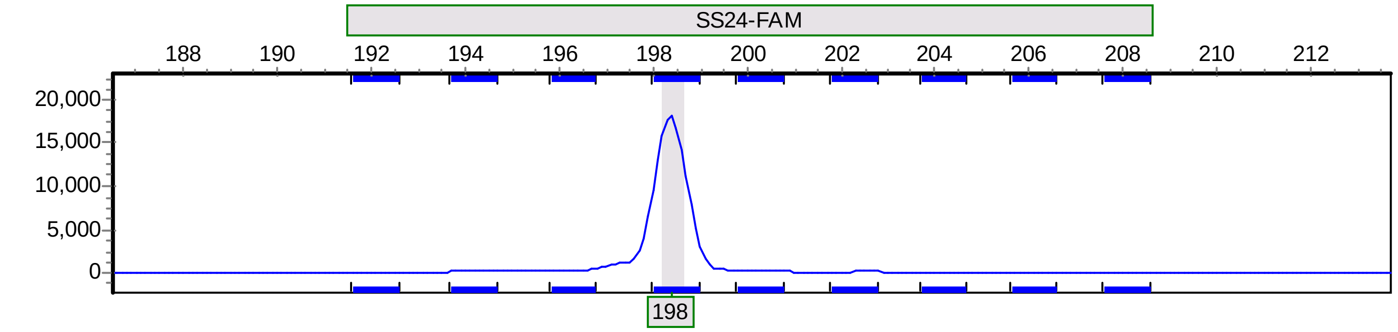

No	Size	Height	Area	Marker	Allele	Difference	Quality	Score	Allele Comments	Sample Comments
1	99.3	3493	57640	SSS18-FAM	100	0.40	Pass	72.8	[<Deleted>]	
2	127.7	11178	75441	SSS18-FAM	128	0.10	Pass	500.0	[<Confirmed>]	
3	198.4	17947	138991	SS24-FAM	198	0.10	Pass	500.0	[<Confirmed>]	
4	245.5	8546	70198	SS05-FAM	245	0.10	Pass	500.0		
5	249.0	7070	59202	SS05-FAM	249	0.20	Pass	500.0		
6	319.7	2971	31571	SS32-FAM	320	0.00	Pass	238.0		
7	332.1	1696	18119	SS32-FAM	332	0.00	Pass	96.3		

Sample 30: SSS18_SS24_SS05_SS32_SS30_SS12_SS23_HBB39_F06.fsa

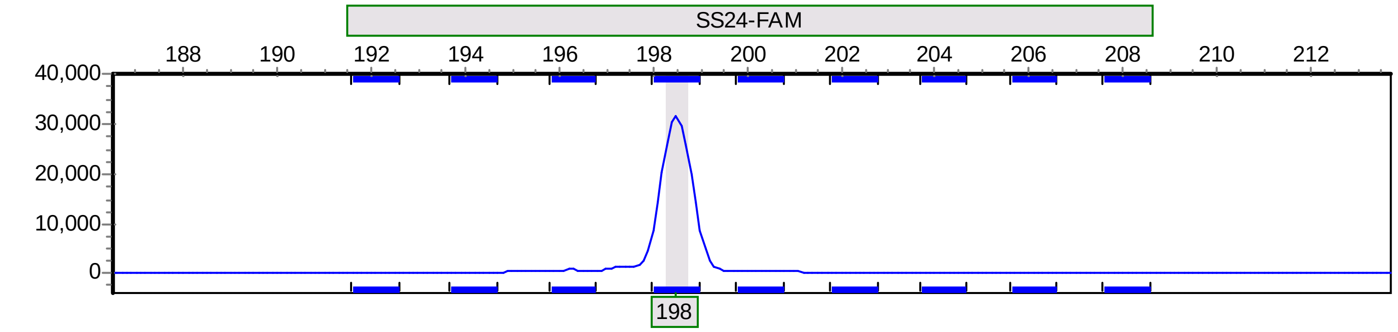

No	Size	Height	Area	Marker	Allele	Difference	Quality	Score	Allele Comments	Sample Comments
1	97.4	22963	160705	SSS18-FAM	98	0.00	Pass	500.0	[<Confirmed>]	
2	133.9	16487	111202	SSS18-FAM	134	0.00	Undetermined	500.0	[<Deleted>]	

3	136.0	17646	117961	SSS18-FAM	136	0.00	Pass	500.0	[<Confirmed>]	
4	198.5	31449	229851	SS24-FAM	198	0.00	Pass	500.0	[<Confirmed>]	
5	247.6	14425	124958	SS05-FAM	247	0.00	Pass	500.0		
6	249.0	13061	104756	SS05-FAM	249	0.20	Pass	500.0		
7	326.1	4850	50075	SS32-FAM	326	0.10	Pass	497.1		
8	328.1	3332	33356	SS32-FAM	328	0.10	Pass	327.2		

Sample 31: SSS18_SS24_SS05_SS32_SS30_SS12_SS23_HBB40_D16.fsa

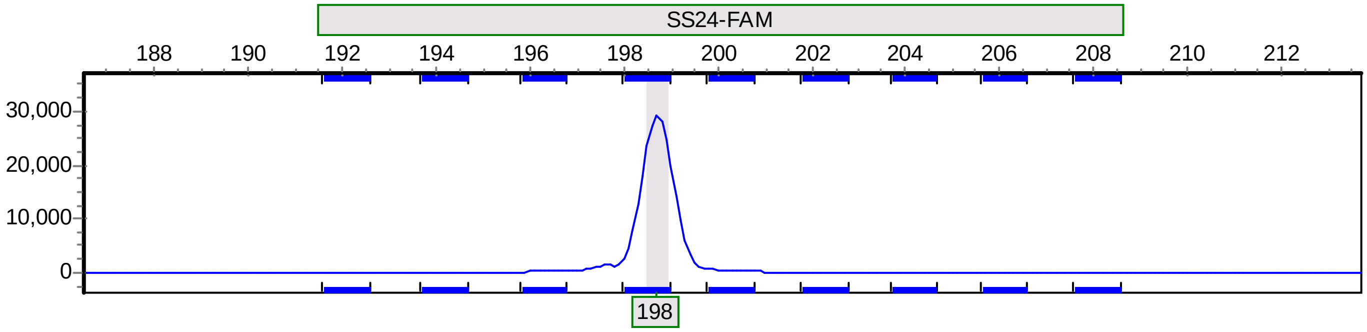

No	Size	Height	Area	Marker	Allele	Difference	Quality	Score	Allele Comments	Sample Comments
1	127.7	23368	162711	SSS18-FAM	128	0.10	Pass	500.0	[<Confirmed>]	
2	198.7	29066	218801	SS24-FAM	198	0.20	Pass	500.0	[<Confirmed>]	
3	245.5	27337	224736	SS05-FAM	245	0.10	Pass	500.0		
4	323.9	4928	49577	SS32-FAM	324	0.00	Pass	500.0		
5	332.0	3049	32226	SS32-FAM	332	0.10	Pass	254.5		

Sample 32: SSS18_SS24_SS05_SS32_SS30_SS12_SS23_HBB41_L08.fsa

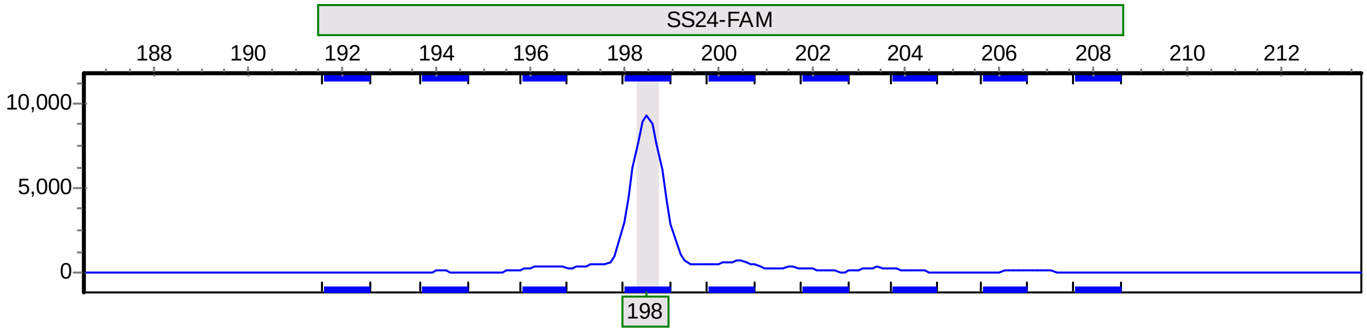

No	Size	Height	Area	Marker	Allele	Difference	Quality	Score	Allele Comments	Sample Comments
1	99.8	2824	45793	SSS18-FAM	100	0.10	Pass	58.7	[<Deleted>]	
2	115.5	7547	50636	SSS18-FAM	116	0.00	Pass	500.0	[<Confirmed>]	
3	198.5	9242	70962	SS24-FAM	198	0.00	Pass	500.0	[<Confirmed>]	
4	245.6	3916	32492	SS05-FAM	245	0.00	Pass	500.0		
5	323.9	1849	19406	SS32-FAM	324	0.00	Pass	124.1		
6	332.1	1291	13104	SS32-FAM	332	0.00	Pass	71.4		

Allele Report

Sample 33: SSS18_SS24_SS05_SS32_SS30_SS12_SS23_HBB42_M06.fsa

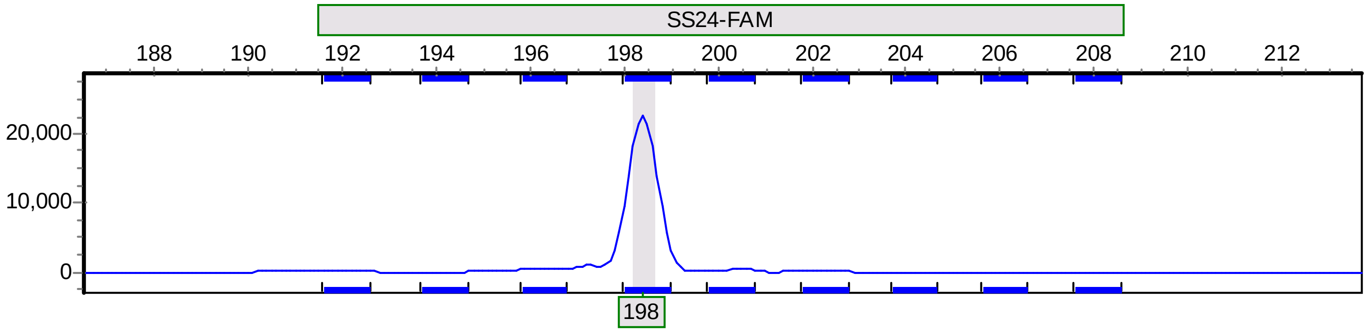

No	Size	Height	Area	Marker	Allele	Difference	Quality	Score	Allele Comments	Sample Comments
1	99.8	28193	187441	SSS18-FAM	100	0.10	Pass	500.0	[<Confirmed>]	
2	126.4	12991	94667	SSS18-FAM	126	1.00	Pass	500.0	[<Confirmed><Edited>]	
3	198.4	22489	159929	SS24-FAM	198	0.10	Pass	500.0	[<Confirmed>]	
4	245.6	22492	174728	SS05-FAM	245	0.00	Pass	500.0		
5	326.0	2941	29041	SS32-FAM	326	0.00	Pass	281.2		
6	332.1	1801	17792	SS32-FAM	332	0.00	Pass	129.7		

Sample 34: SSS18_SS24_SS05_SS32_SS30_SS12_SS23_HBB43_D10.fsa

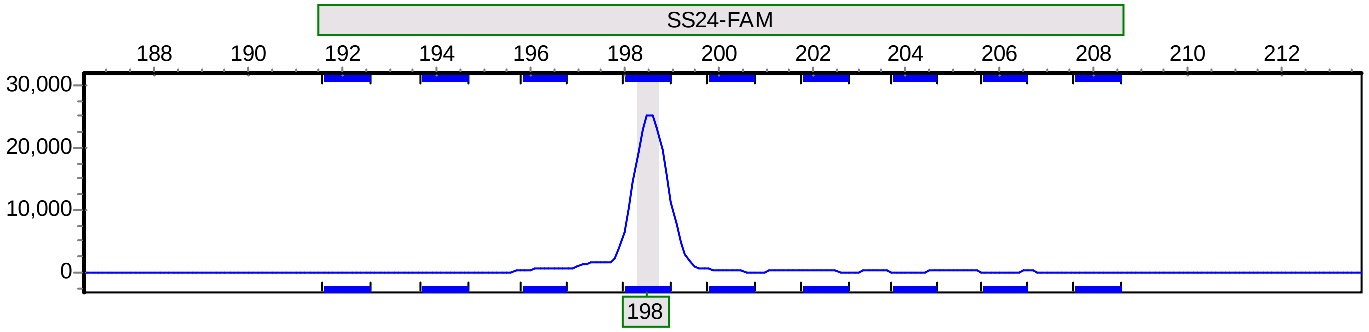

No	Size	Height	Area	Marker	Allele	Difference	Quality	Score	Allele Comments	Sample Comments
1	105.3	27440	182930	SSS18-FAM	106	0.10	Pass	500.0	[<Confirmed>]	
2	113.4	14791	99727	SSS18-FAM	114	0.10	Pass	500.0	[<Confirmed>]	
3	198.5	25119	204521	SS24-FAM	198	0.00	Pass	500.0	[<Confirmed>]	
4	245.6	7964	69946	SS05-FAM	245	0.00	Pass	500.0		
5	271.2	7963	71403	SS05-FAM	271	0.00	Pass	500.0		
6	325.9	5699	61094	SS32-FAM	326	0.10	Pass	500.0		

Sample 35: SSS18_SS24_SS05_SS32_SS30_SS12_SS23_HBB44_H12.fsa

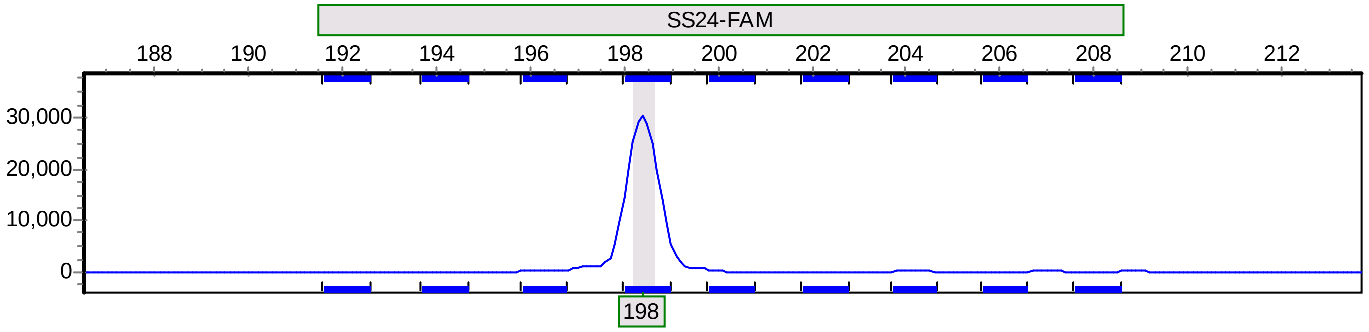

No	Size	Height	Area	Marker	Allele	Difference	Quality	Score	Allele Comments	Sample Comments
1	115.4	24696	166208	SSS18-FAM	116	0.10	Pass	500.0	[<Confirmed>]	
2	198.4	30098	228592	SS24-FAM	198	0.10	Pass	500.0	[<Confirmed>]	
3	245.5	26312	216045	SS05-FAM	245	0.10	Pass	500.0		

4	325.9	3792	40134	SS32-FAM	326	0.10	Pass	355.6		
5	332.0	2881	29425	SS32-FAM	332	0.10	Pass	249.6		

Sample 36: SSS18_SS24_SS05_SS32_SS30_SS12_SS23_HBB45_F14.fsa

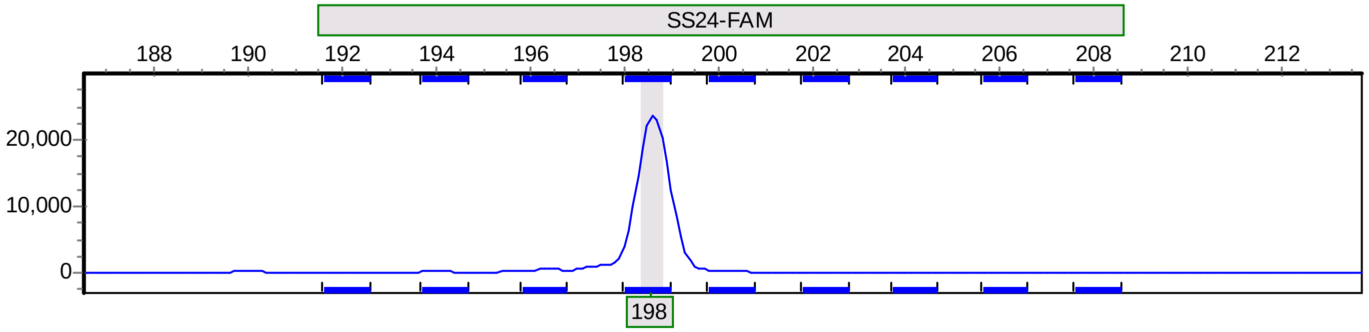

No	Size	Height	Area	Marker	Allele	Difference	Quality	Score	Allele Comments	Sample Comments
1	100.0	3836	63767	SSS18-FAM	100	0.30	Pass	102.3	[<Deleted>]	
2	127.7	11295	79996	SSS18-FAM	128	0.10	Pass	500.0	[<Confirmed>]	
3	198.6	23486	181435	SS24-FAM	198	0.10	Pass	500.0	[<Confirmed>]	
4	245.6	14600	124219	SS05-FAM	245	0.00	Pass	500.0		
5	323.8	3474	36816	SS32-FAM	324	0.10	Pass	301.9		
6	332.0	1976	21410	SS32-FAM	332	0.10	Pass	113.5		

Sample 37: SSS18_SS24_SS05_SS32_SS30_SS12_SS23_HBB46_F08.fsa

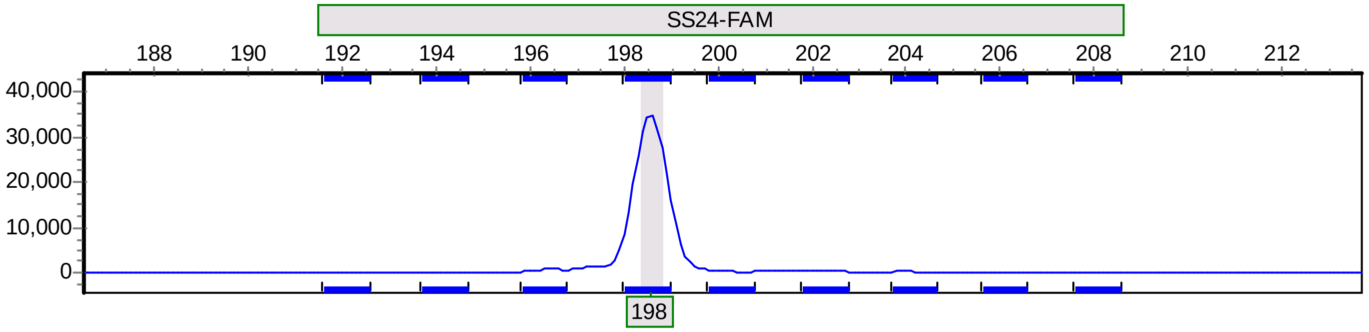

No	Size	Height	Area	Marker	Allele	Difference	Quality	Score	Allele Comments	Sample Comments
1	97.4	30635	222579	SSS18-FAM	98	0.00	Pass	500.0	[<Confirmed>]	
2	115.4	16951	114200	SSS18-FAM	116	0.10	Pass	500.0	[<Confirmed>]	
3	198.6	34595	277344	SS24-FAM	198	0.10	Pass	500.0	[<SAT (Repaired)><Confirmed>]	
4	245.6	22204	172990	SS05-FAM	245	0.00	Pass	500.0		
5	247.5	14093	114689	SS05-FAM	247	0.10	Pass	500.0		
6	326.0	3914	39104	SS32-FAM	326	0.00	Pass	412.4		
7	332.1	2664	28362	SS32-FAM	332	0.00	Pass	202.9		

Sample 38: SSS18_SS24_SS05_SS32_SS30_SS12_SS23_HBB47_A06.fsa

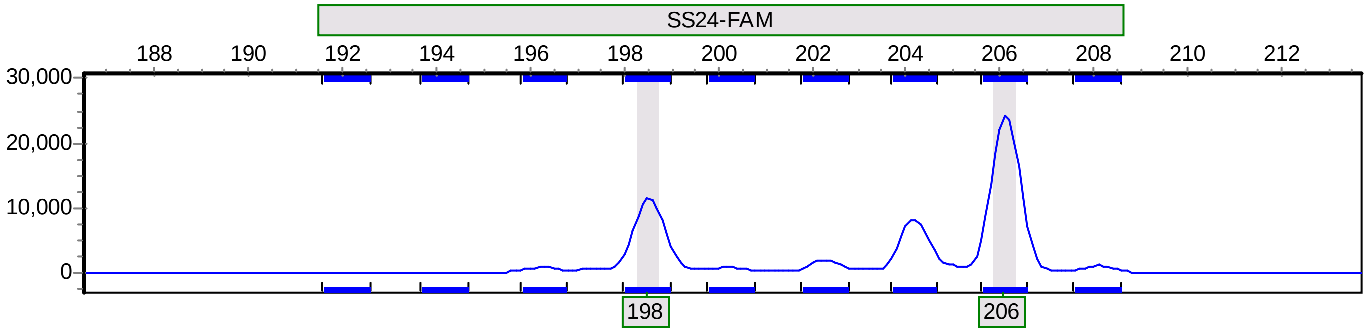

No	Size	Height	Area	Marker	Allele	Difference	Quality	Score	Allele Comments	Sample Comments

1	147.2	15556	114026	SSS18-FAM	148	0.10	Pass	500.0	[<Confirmed>]	
2	149.4	13036	95358	SSS18-FAM	150	0.00	Pass	500.0	[<Confirmed>]	
3	198.5	11518	85388	SS24-FAM	198	0.00	Pass	500.0	[<Confirmed>]	
4	206.1	24077	170593	SS24-FAM	206	0.00	Pass	500.0	[<Confirmed>]	
5	251.6	31662	256874	SS05-FAM	251	0.20	Check	500.0		
6	319.9	5929	56383	SS32-FAM	320	0.20	Pass	500.0		

Sample 39: SSS18_SS24_SS05_SS32_SS30_SS12_SS23_HBB48_B14.fsa

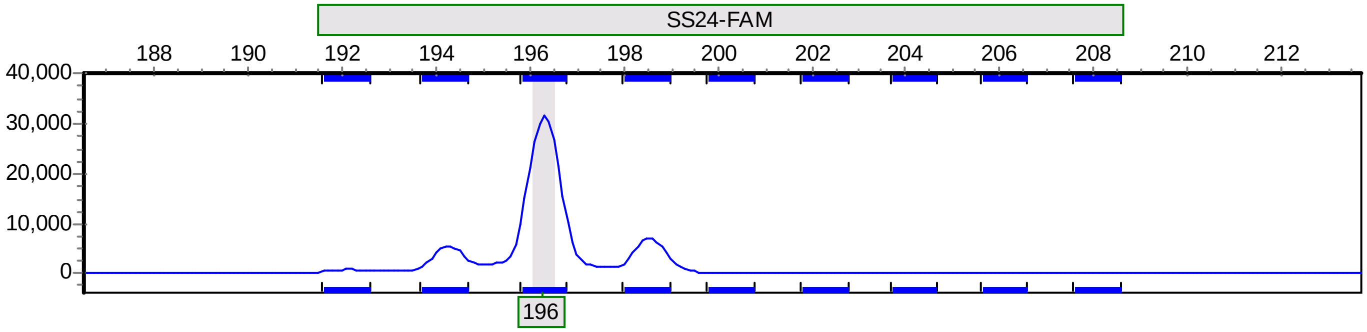

No	Size	Height	Area	Marker	Allele	Difference	Quality	Score	Allele Comments	Sample Comments
1	115.5	31888	231873	SSS18-FAM	116	0.00	Pass	500.0	[<Confirmed>]	
2	196.3	31472	243386	SS24-FAM	196	0.00	Pass	500.0	[<Confirmed>]	
3	245.5	7291	59692	SS05-FAM	245	0.10	Pass	500.0		
4	281.4	10976	102025	SS05-FAM	281	0.00	Pass	500.0		
5	325.9	3717	38850	SS32-FAM	326	0.10	Pass	336.7		
6	347.7	1425	14287	SS32-FAM	348	0.00	Pass	85.3		

Sample 40: SSS18_SS24_SS05_SS32_SS30_SS12_SS23_HBB49_L06.fsa

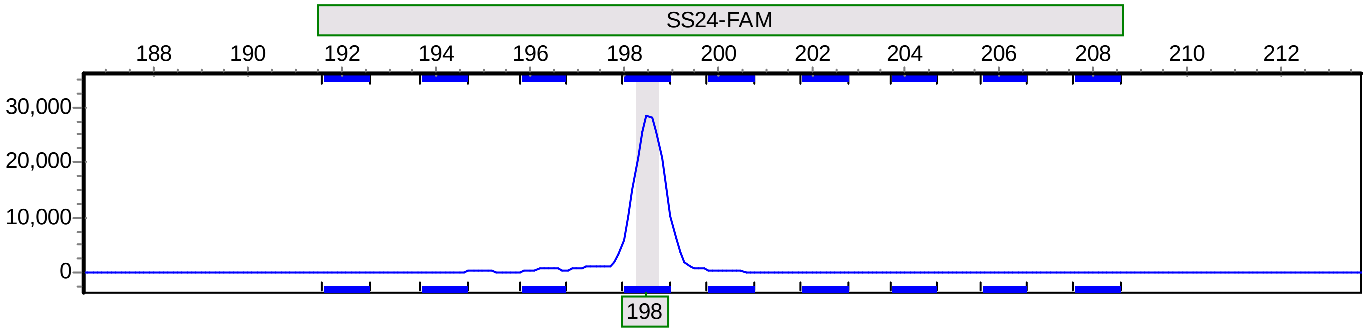

No	Size	Height	Area	Marker	Allele	Difference	Quality	Score	Allele Comments	Sample Comments
1	115.4	23185	150684	SSS18-FAM	116	0.10	Pass	500.0	[<Confirmed>]	
2	117.4	13505	88971	SSS18-FAM	118	0.20	Pass	500.0	[<Confirmed>]	
3	198.5	28223	207548	SS24-FAM	198	0.00	Pass	500.0	[<Confirmed>]	
4	245.7	24966	199669	SS05-FAM	245	0.10	Pass	500.0		
5	319.8	3991	40501	SS32-FAM	320	0.10	Pass	395.6		
6	326.0	2947	30759	SS32-FAM	326	0.00	Pass	258.2		

Sample 41: SSS18_SS24_SS05_SS32_SS30_SS12_SS23_HBB4_E08.fsa

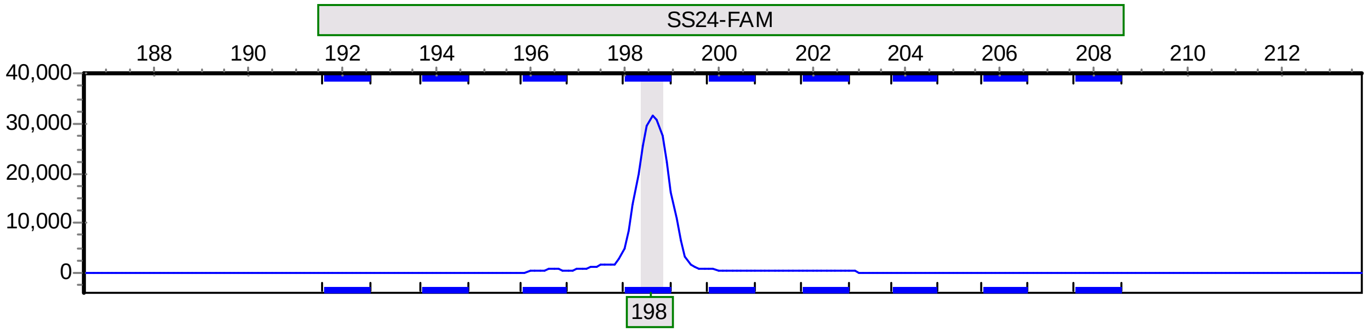

No	Size	Height	Area	Marker	Allele	Difference	Quality	Score	Allele Comments	Sample Comments
1	97.3	31705	239121	SSS18-FAM	98	0.10	Pass	500.0	[<Confirmed>]	
2	113.4	24015	159319	SSS18-FAM	114	0.10	Pass	500.0	[<Confirmed>]	
3	198.6	31386	239912	SS24-FAM	198	0.10	Pass	500.0	[<Confirmed>]	
4	245.6	16449	134889	SS05-FAM	245	0.00	Pass	500.0		
5	249.1	13523	109486	SS05-FAM	249	0.10	Pass	500.0		
6	326.0	6602	65062	SS32-FAM	326	0.00	Pass	500.0		

Sample 42: SSS18_SS24_SS05_SS32_SS30_SS12_SS23_HBB5_H14.fsa

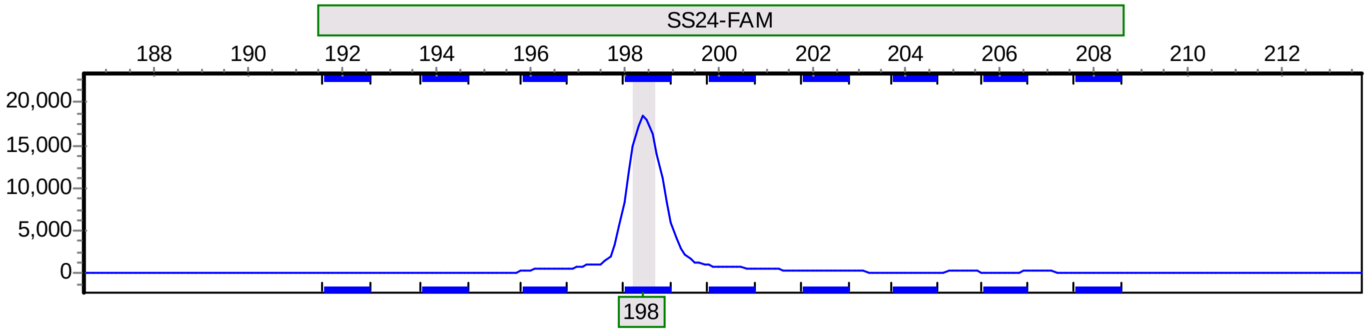

No	Size	Height	Area	Marker	Allele	Difference	Quality	Score	Allele Comments	Sample Comments
1	103.3	14564	102254	SSS18-FAM	104	0.10	Pass	500.0	[<Confirmed>]	
2	111.4	10748	76672	SSS18-FAM	112	0.10	Pass	500.0	[<Confirmed>]	
3	198.4	18335	154713	SS24-FAM	198	0.10	Pass	500.0	[<Confirmed>]	
4	245.4	9521	85883	SS05-FAM	245	0.20	Pass	500.0		
5	247.4	7358	67315	SS05-FAM	247	0.20	Pass	500.0		
6	326.0	4170	46294	SS32-FAM	326	0.00	Pass	326.1		

Sample 43: SSS18_SS24_SS05_SS32_SS30_SS12_SS23_HBB6_C06.fsa

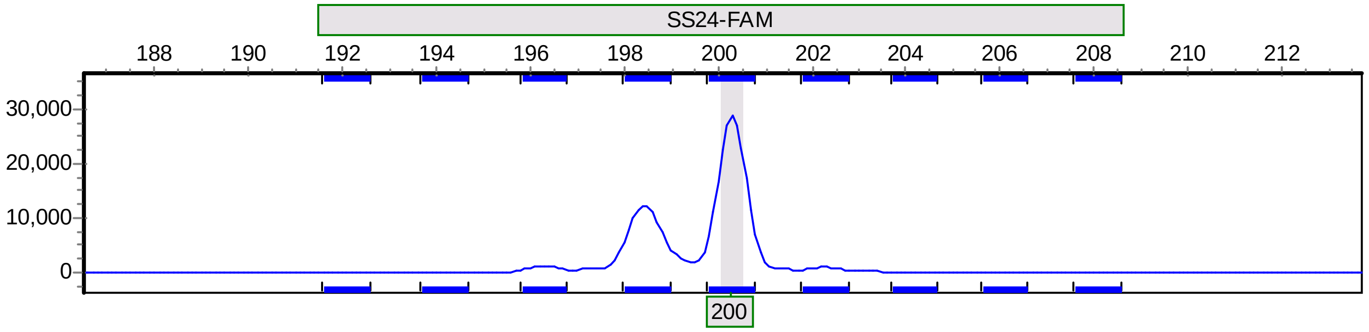

No	Size	Height	Area	Marker	Allele	Difference	Quality	Score	Allele Comments	Sample Comments
1	113.5	23705	156121	SSS18-FAM	114	0.00	Pass	500.0	[<Confirmed>]	
2	115.5	14443	95474	SSS18-FAM	116	0.00	Pass	500.0	[<Confirmed>]	
3	200.3	28848	197157	SS24-FAM	200	0.00	Pass	500.0	[<Confirmed>]	

4	245.6	24263	188853	SS05-FAM	245	0.00	Pass	500.0		
5	319.7	6488	62346	SS32-FAM	320	0.00	Pass	500.0		

Sample 44: SSS18_SS24_SS05_SS32_SS30_SS12_SS23_HBB7_N06.fsa

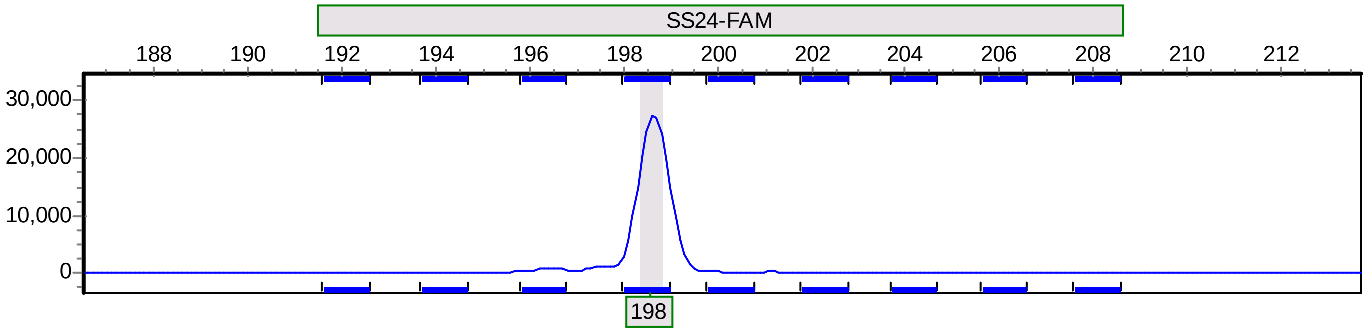

No	Size	Height	Area	Marker	Allele	Difference	Quality	Score	Allele Comments	Sample Comments
1	115.6	20444	130976	SSS18-FAM	116	0.10	Pass	500.0	[<Confirmed>]	
2	198.6	27132	198417	SS24-FAM	198	0.10	Pass	500.0	[<Confirmed>]	
3	245.8	16025	128279	SS05-FAM	245	0.20	Pass	500.0		
4	249.3	11326	91522	SS05-FAM	249	0.10	Pass	500.0		
5	326.1	3280	33013	SS32-FAM	326	0.10	Pass	300.1		
6	332.2	2398	24556	SS32-FAM	332	0.10	Pass	200.7		

Sample 45: SSS18_SS24_SS05_SS32_SS30_SS12_SS23_HBB8_P12.fsa

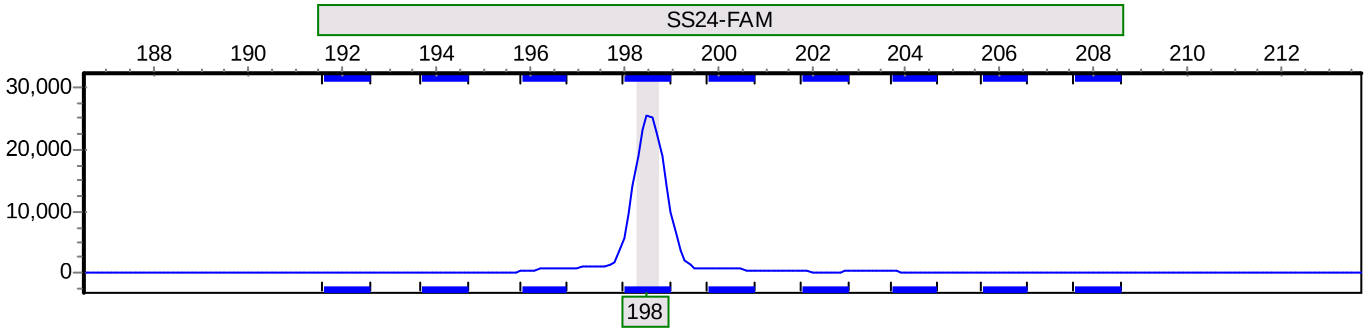

No	Size	Height	Area	Marker	Allele	Difference	Quality	Score	Allele Comments	Sample Comments
1	113.5	8239	56067	SSS18-FAM	114	0.00	Pass	500.0	[<Confirmed>]	
2	125.8	22886	154277	SSS18-FAM	126	0.10	Pass	500.0	[<Confirmed>]	
3	198.5	25301	191272	SS24-FAM	198	0.00	Pass	500.0	[<Confirmed>]	
4	273.3	16019	143285	SS05-FAM	273	0.10	Pass	500.0		
5	325.9	3065	32402	SS32-FAM	326	0.10	Pass	249.2		
6	342.1	1569	15965	SS32-FAM	342	0.00	Pass	96.6		

Sample 46: SSS18_SS24_SS05_SS32_SS30_SS12_SS23_HBB9_I06.fsa

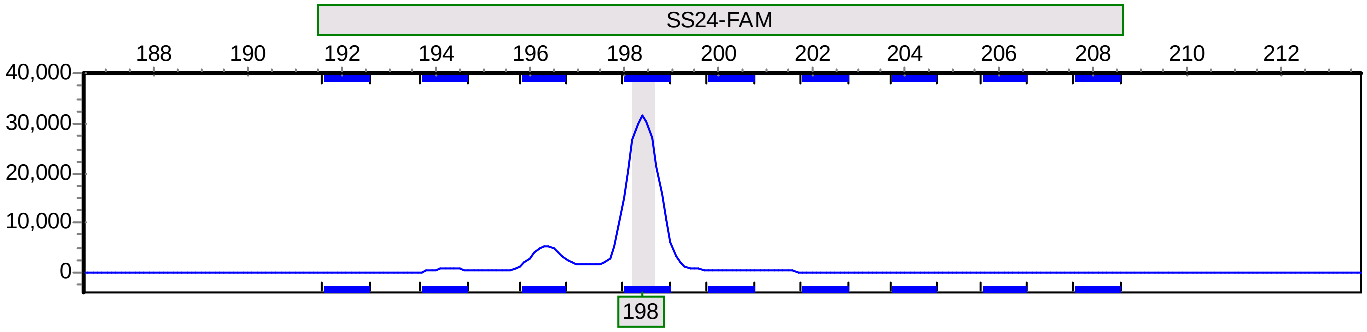

No	Size	Height	Area	Marker	Allele	Difference	Quality	Score	Allele Comments	Sample Comments
1	103.4	16918	112110	SSS18-FAM	104	0.00	Pass	500.0	[<Confirmed>]	

2	129.9	14766	100363	SSS18-FAM	130	0.10	Undetermined	500.0	[<Deleted>]	
3	132.0	15830	108010	SSS18-FAM	132	0.10	Pass	500.0	[<Confirmed>]	
4	198.4	31417	243565	SS24-FAM	198	0.10	Pass	500.0	[<Confirmed>]	
5	265.7	15715	132964	SS05-FAM	265	0.20	Pass	500.0		
6	326.1	2801	28141	SS32-FAM	326	0.10	Pass	239.5		
7	347.7	1126	11178	SS32-FAM	348	0.00	Pass	65.9		

Sample 47: SSS18_SS24_SS05_SS32_SS30_SS12_SS23_HBN10_G12.fsa

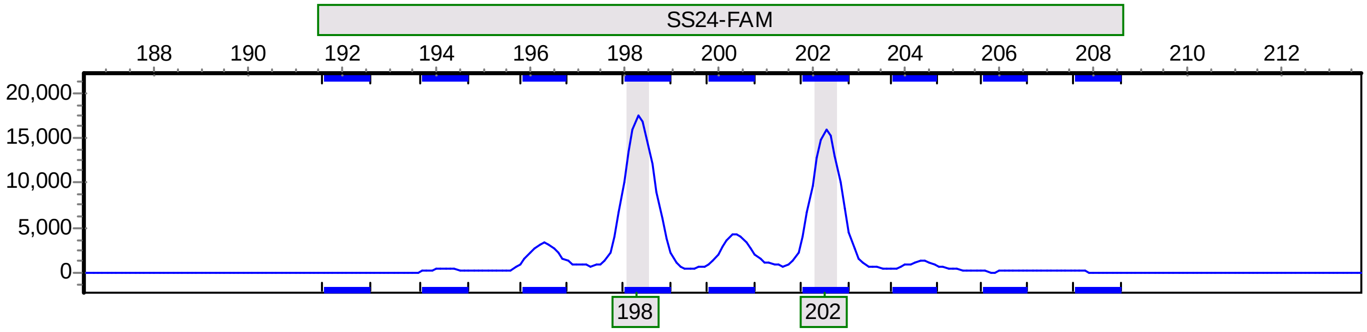

No	Size	Height	Area	Marker	Allele	Difference	Quality	Score	Allele Comments	Sample Comments
1	131.8	16449	114629	SSS18-FAM	132	0.10	Pass	500.0	[<Confirmed>]	
2	198.3	17372	128838	SS24-FAM	198	0.20	Pass	500.0	[<Confirmed>]	
3	202.3	15844	114873	SS24-FAM	202	0.00	Pass	500.0	[<Confirmed>]	
4	243.4	21511	172545	SS05-FAM	243	0.20	Pass	500.0		
5	351.4	934	9186	SS32-FAM	352	0.10	Pass	46.8		

Sample 48: SSS18_SS24_SS05_SS32_SS30_SS12_SS23_HBN6_O02.fsa

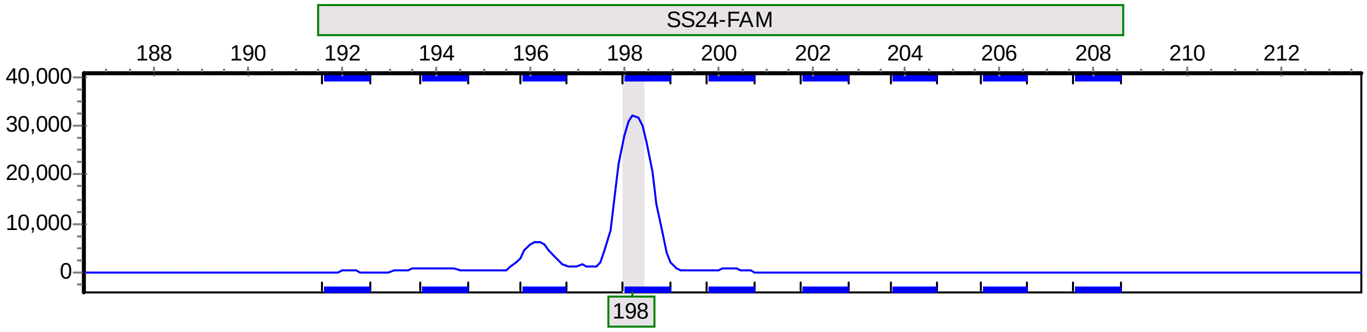

No	Size	Height	Area	Marker	Allele	Difference	Quality	Score	Allele Comments	Sample Comments
1	107.6	23074	141551	SSS18-FAM	108	0.10	Pass	500.0	[<Confirmed>]	
2	119.8	30856	197165	SSS18-FAM	120	0.20	Pass	500.0	[<Confirmed>]	
3	198.2	31887	267258	SS24-FAM	198	0.30	Pass	500.0	[<Confirmed>]	
4	243.7	36583	276669	SS05-FAM	243	0.10	Check	500.0	[<SAT (Repaired)>]	
5	330.2	3969	36927	SS32-FAM	330	0.10	Pass	500.0		
6	351.6	1525	14230	SS32-FAM	352	0.10	Pass	115.5		

Allele Report

Sample 49: SSS18_SS24_SS05_SS32_SS30_SS12_SS23_HBN9_D04.fsa

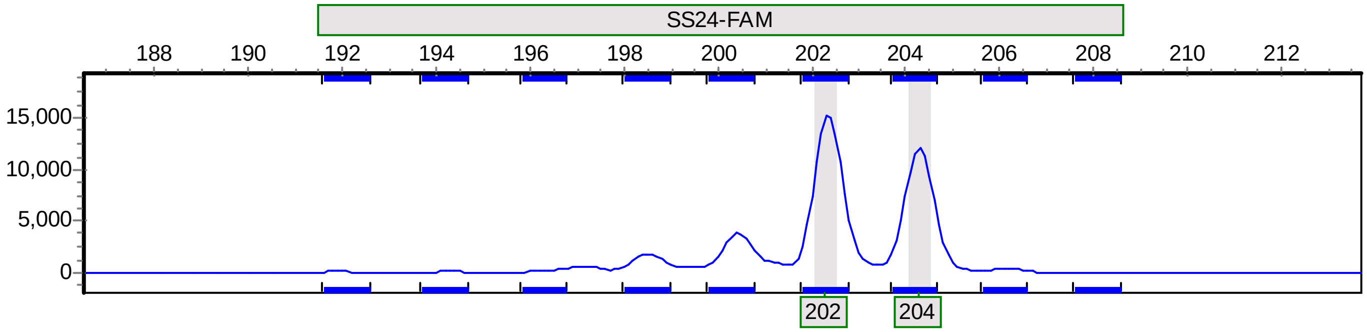

No	Size	Height	Area	Marker	Allele	Difference	Quality	Score	Allele Comments	Sample Comments
1	140.4	8209	63643	SSS18-FAM	140	0.10	Pass	500.0	[<Confirmed>]	
2	142.7	7836	58233	SSS18-FAM	142	0.10	Pass	500.0	[<Confirmed>]	
3	153.4	2651	17268	SSS18-FAM	154	0.00	Undetermined	500.0	[<Deleted>]	
4	155.4	2754	17604	SSS18-FAM	156	0.10	Undetermined	500.0	[<Deleted>]	
5	202.3	15083	105493	SS24-FAM	202	0.00	Pass	500.0	[<Confirmed>]	
6	204.3	12050	83944	SS24-FAM	204	0.10	Pass	500.0	[<Confirmed>]	
7	245.6	19697	156143	SS05-FAM	245	0.00	Pass	500.0		
8	247.6	14617	117631	SS05-FAM	247	0.00	Pass	500.0		
9	323.8	829	8314	SS32-FAM	324	0.10	Pass	34.4		
10	325.9	698	7660	SS32-FAM	326	0.10	Undetermined	21.5		
11	351.4	1121	10758	SS32-FAM	352	0.10	Pass	67.7		

Sample 50: SSS18_SS24_SS05_SS32_SS30_SS12_SS23_HCW1_M10.fsa

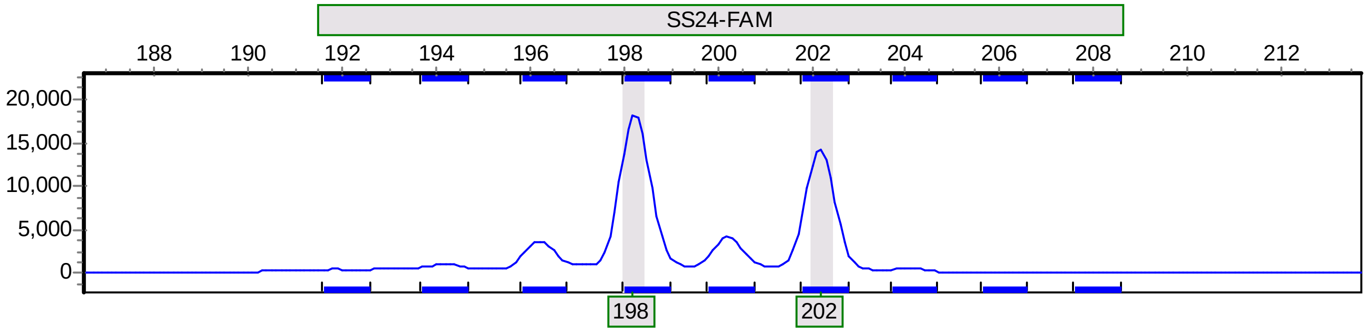

No	Size	Height	Area	Marker	Allele	Difference	Quality	Score	Allele Comments	Sample Comments
1	115.6	35378	264917	SSS18-FAM	116	0.10	Pass	500.0	[<SAT (Repaired)><Confirmed>]	
2	198.2	18103	136727	SS24-FAM	198	0.30	Pass	500.0	[<Confirmed>]	
3	202.2	14211	103690	SS24-FAM	202	0.10	Pass	500.0	[<Confirmed>]	
4	247.4	17040	140024	SS05-FAM	247	0.20	Pass	500.0		
5	259.3	13292	112684	SS05-FAM	259	0.10	Pass	500.0		
6	340.2	2090	20780	SS32-FAM	340	0.00	Pass	168.7		

Sample 51: SSS18_SS24_SS05_SS32_SS30_SS12_SS23_HCW2_A12.fsa

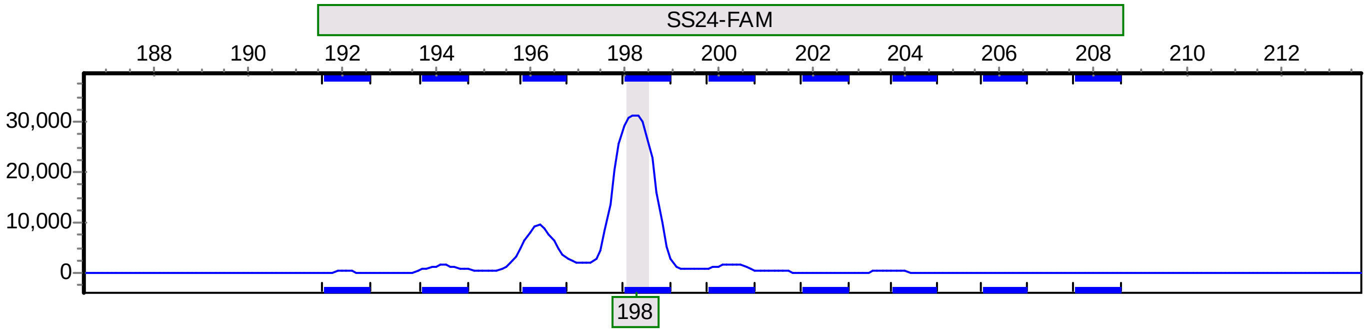

No	Size	Height	Area	Marker	Allele	Difference	Quality	Score	Allele Comments	Sample Comments
1	115.7	28933	196338	SSS18-FAM	116	0.20	Pass	500.0	[<Confirmed>]	
2	121.7	21703	145830	SSS18-FAM	122	0.00	Pass	500.0	[<Confirmed>]	
3	198.3	31081	298800	SS24-FAM	198	0.20	Pass	500.0	[<Confirmed>]	
4	259.4	19756	162901	SS05-FAM	259	0.00	Pass	500.0		
5	265.5	13851	114521	SS05-FAM	265	0.00	Pass	500.0		
6	338.2	3454	34598	SS32-FAM	338	0.10	Pass	331.0		

Sample 52: SSS18_SS24_SS05_SS32_SS30_SS12_SS23_HCW3_G14.fsa

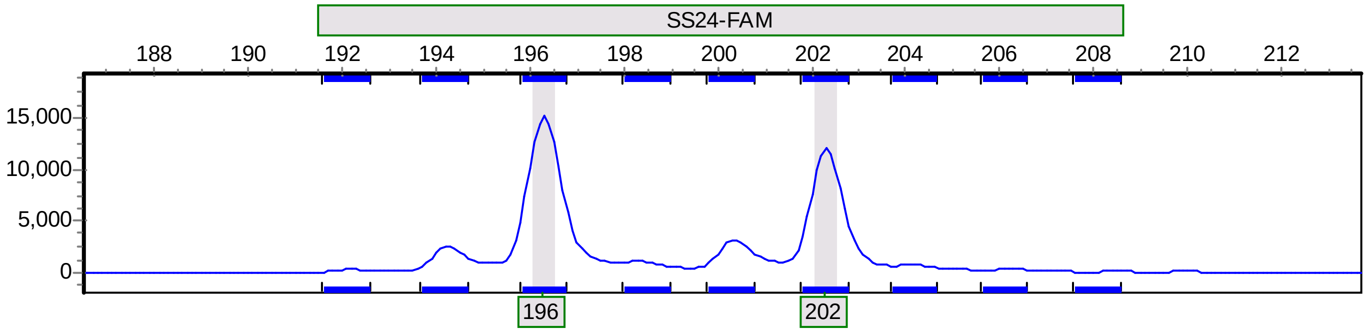

No	Size	Height	Area	Marker	Allele	Difference	Quality	Score	Allele Comments	Sample Comments
1	121.5	22031	163289	SSS18-FAM	122	0.20	Pass	500.0	[<Confirmed>]	
2	123.6	14388	107814	SSS18-FAM	124	0.10	Pass	500.0	[<Confirmed>]	
3	196.3	15071	121360	SS24-FAM	196	0.00	Pass	500.0	[<Confirmed>]	
4	202.3	12051	95433	SS24-FAM	202	0.00	Pass	500.0	[<Confirmed>]	
5	259.4	15298	140221	SS05-FAM	259	0.00	Pass	500.0		
6	261.4	12388	114715	SS05-FAM	261	0.10	Pass	500.0		
7	345.8	1405	14975	SS32-FAM	346	0.00	Pass	68.0		
8	359.1	559	6426	SS32-FAM	360	0.20	Pass	12.6		

Sample 53: SSS18_SS24_SS05_SS32_SS30_SS12_SS23_HCW4_E10.fsa

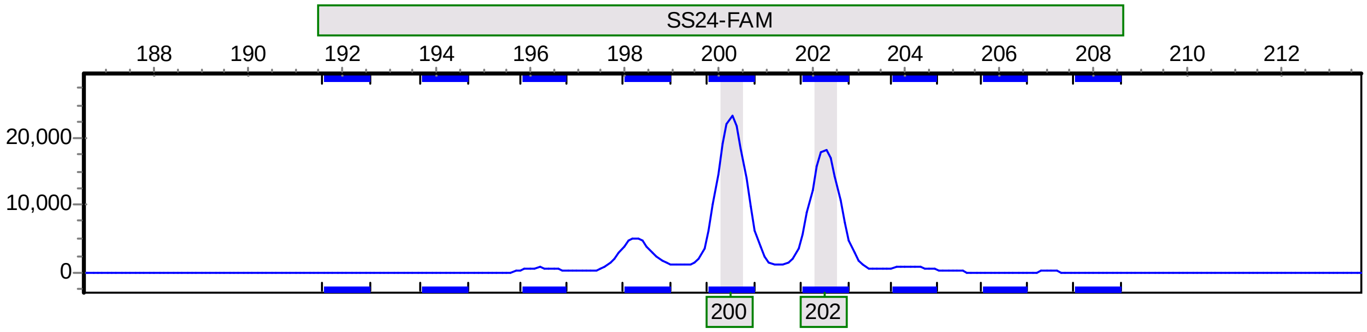

No	Size	Height	Area	Marker	Allele	Difference	Quality	Score	Allele Comments	Sample Comments
1	115.6	23181	157228	SSS18-FAM	116	0.10	Pass	500.0	[<Confirmed>]	

2	131.8	13050	91154	SSS18-FAM	132	0.10	Pass	500.0	[<Confirmed>]
3	200.3	23007	165845	SS24-FAM	200	0.00	Pass	500.0	[<Confirmed>]
4	202.3	18217	135052	SS24-FAM	202	0.00	Pass	500.0	[<Confirmed>]
5	340.2	2391	24370	SS32-FAM	340	0.00	Pass	179.0	
6	357.2	788	8132	SS32-FAM	358	0.10	Pass	31.0	

Sample 54: SSS18_SS24_SS05_SS32_SS30_SS12_SS23_HCW5_C12.fsa

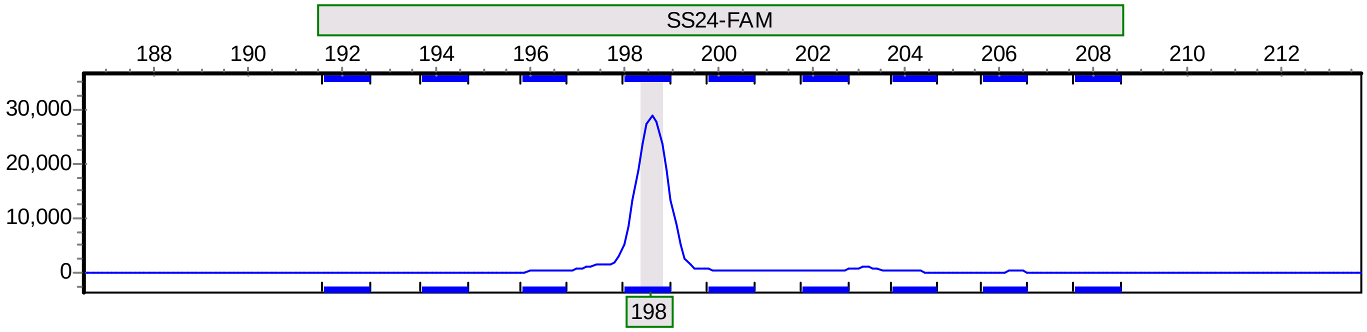

No	Size	Height	Area	Marker	Allele	Difference	Quality	Score	Allele Comments	Sample Comments
1	97.2	28411	202011	SSS18-FAM	98	0.20	Pass	500.0	[<Confirmed>]	
2	113.3	16918	112055	SSS18-FAM	114	0.20	Pass	500.0	[<Confirmed>]	
3	198.6	28635	216033	SS24-FAM	198	0.10	Pass	500.0	[<Confirmed>]	
4	249.0	22182	181265	SS05-FAM	249	0.20	Pass	500.0		
5	325.9	5576	56774	SS32-FAM	326	0.10	Pass	500.0		

Sample 55: SSS18_SS24_SS05_SS32_SS30_SS12_SS23_HCW6_D02.fsa

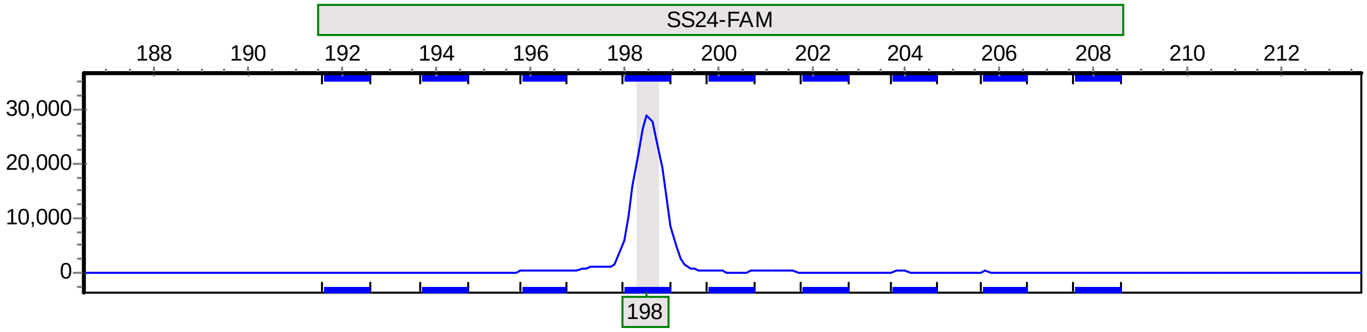

No	Size	Height	Area	Marker	Allele	Difference	Quality	Score	Allele Comments	Sample Comments
1	127.7	18476	121027	SSS18-FAM	128	0.10	Pass	500.0	[<Confirmed>]	
2	198.5	28879	203328	SS24-FAM	198	0.00	Pass	500.0	[<Confirmed>]	
3	247.7	14082	122433	SS05-FAM	247	0.10	Pass	500.0		
4	249.1	13567	111197	SS05-FAM	249	0.10	Pass	500.0		
5	328.0	3732	37569	SS32-FAM	328	0.00	Pass	367.1		
6	332.1	2798	29070	SS32-FAM	332	0.00	Pass	246.7		

Allele Report

Sample 56: SSS18_SS24_SS05_SS32_SS30_SS12_SS23_HCW7_G18.fsa

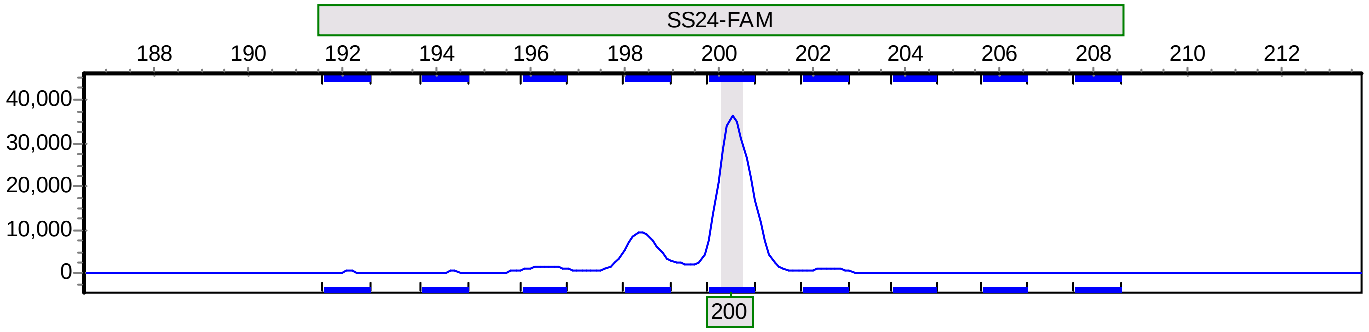

No	Size	Height	Area	Marker	Allele	Difference	Quality	Score	Allele Comments	Sample Comments
1	129.7	31581	227431	SSS18-FAM	130	0.10	Pass	500.0	[<Confirmed>]	
2	200.3	36192	285127	SS24-FAM	200	0.00	Pass	500.0	[<SAT (Repaired)><Confirmed>]	
3	251.4	30655	267121	SS05-FAM	251	0.00	Check	500.0		
4	253.3	30222	257730	SS05-FAM	253	0.00	Check	500.0		
5	342.1	2686	26746	SS32-FAM	342	0.00	Pass	229.9		
6	349.5	1727	17064	SS32-FAM	350	0.10	Pass	128.3		

Sample 57: SSS18_SS24_SS05_SS32_SS30_SS12_SS23_HCW8_A18.fsa

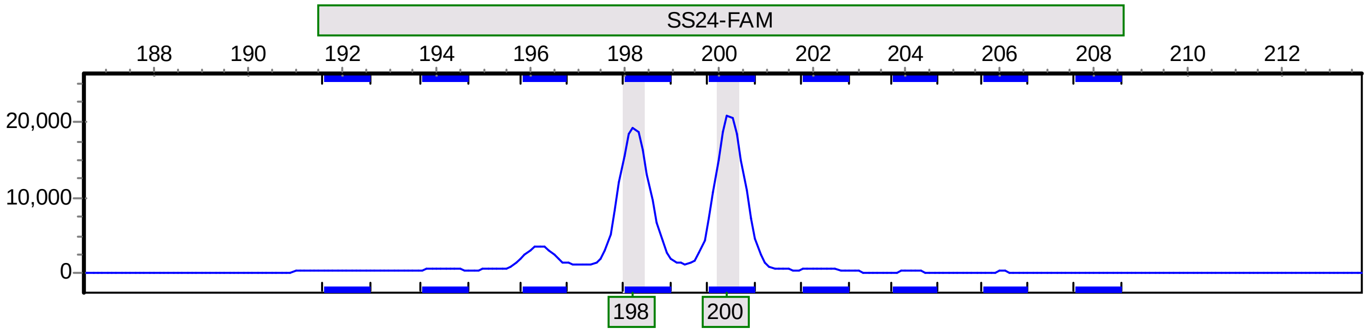

No	Size	Height	Area	Marker	Allele	Difference	Quality	Score	Allele Comments	Sample Comments
1	125.6	15206	103535	SSS18-FAM	126	0.10	Pass	500.0	[<Confirmed>]	
2	135.9	10177	71394	SSS18-FAM	136	0.10	Undetermined	500.0	[<Deleted>]	
3	137.9	10522	73960	SSS18-FAM	138	0.20	Pass	500.0	[<Confirmed>]	
4	198.2	19440	146922	SS24-FAM	198	0.30	Pass	500.0	[<Confirmed>]	
5	200.2	20821	150173	SS24-FAM	200	0.10	Pass	500.0	[<Confirmed>]	
6	257.6	12386	105362	SS05-FAM	257	0.10	Pass	500.0		
7	269.5	9558	80177	SS05-FAM	269	0.10	Pass	500.0		
8	336.1	2569	26697	SS32-FAM	336	0.10	Pass	185.7		

Sample 58: SSS18_SS24_SS05_SS32_SS30_SS12_SS23_HGC1_C02.fsa

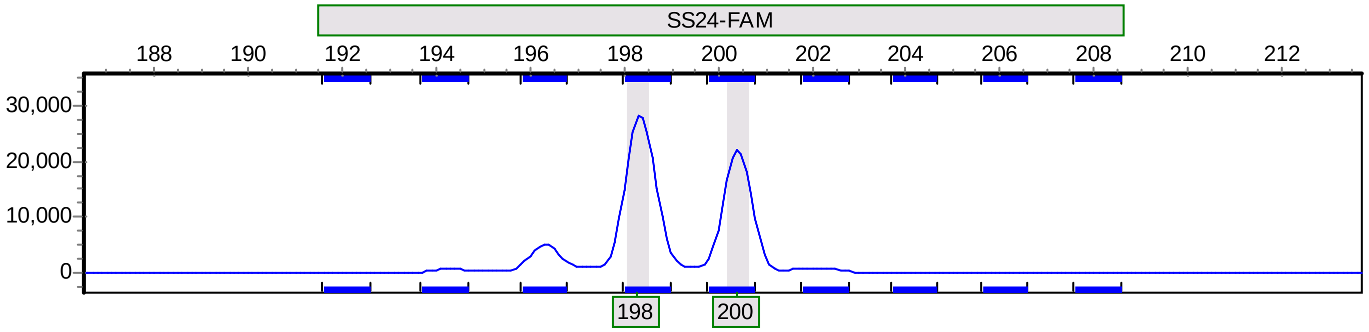

No	Size	Height	Area	Marker	Allele	Difference	Quality	Score	Allele Comments	Sample Comments
1	125.8	31976	229190	SSS18-FAM	126	0.10	Pass	500.0	[<Confirmed>]	

2	127.9	24474	162382	SSS18-FAM	128	0.10	Pass	500.0	[<Confirmed>]	
3	198.3	27958	204502	SS24-FAM	198	0.20	Pass	500.0	[<Confirmed>]	
4	200.4	22027	151153	SS24-FAM	200	0.10	Pass	500.0	[<Confirmed>]	
5	247.7	26223	208311	SS05-FAM	247	0.10	Pass	500.0		
6	269.7	9834	79385	SS05-FAM	269	0.30	Pass	500.0		
7	340.3	3680	34004	SS32-FAM	340	0.10	Pass	442.1		
8	349.7	2152	20143	SS32-FAM	350	0.10	Pass	196.3		

Sample 59: SSS18_SS24_SS05_SS32_SS30_SS12_SS23_HGC3_E18.fsa

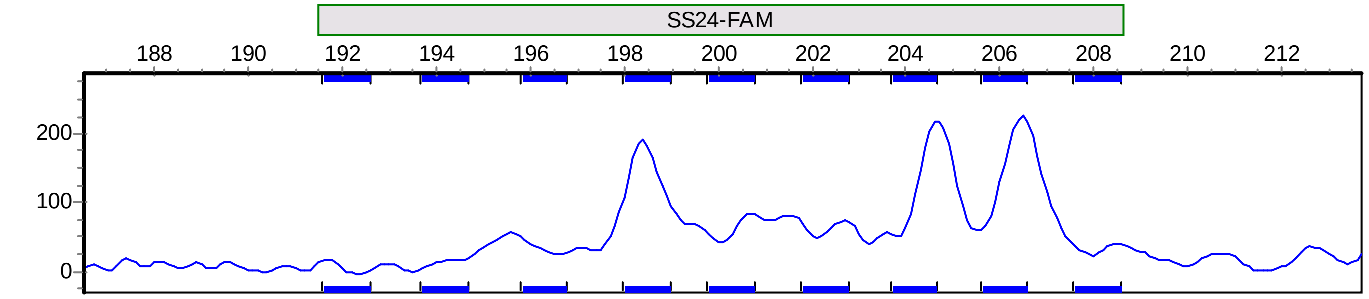

No	Size	Height	Area	Marker	Allele	Difference	Quality	Score	Allele Comments	Sample Comments
1	117.5	620	4461	SSS18-FAM	118	0.10	Pass	53.4	[<Confirmed>]	
2	253.3	781	6619	SS05-FAM	253	0.00	Pass	54.5		

Sample 60: SSS18_SS24_SS05_SS32_SS30_SS12_SS23_HGC4_A14.fsa

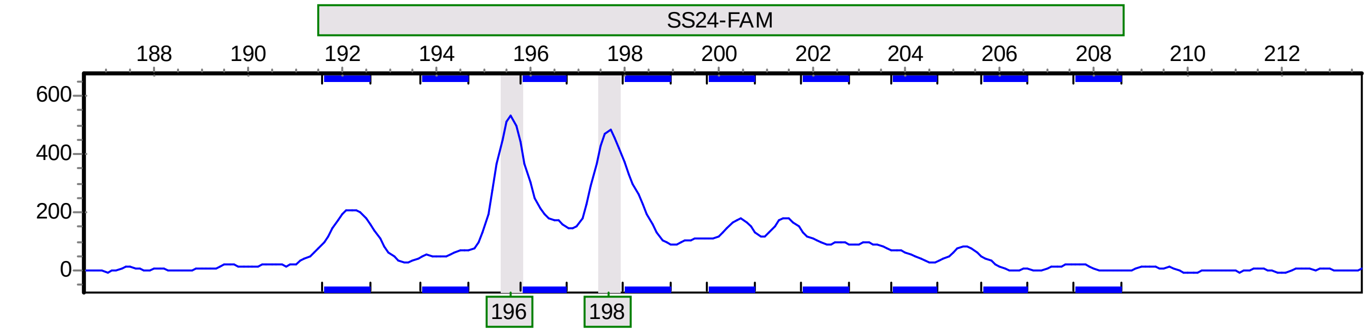

No	Size	Height	Area	Marker	Allele	Difference	Quality	Score	Allele Comments	Sample Comments
1	195.6	532	4365	SS24-FAM	196	1.00	Pass	28.9	[<Confirmed><Edited>]	
2	197.7	482	5174	SS24-FAM	198	1.00	Pass	15.2	[<Confirmed><Edited>]	

Sample 61: SSS18_SS24_SS05_SS32_SS30_SS12_SS23_HGY1_A10.fsa

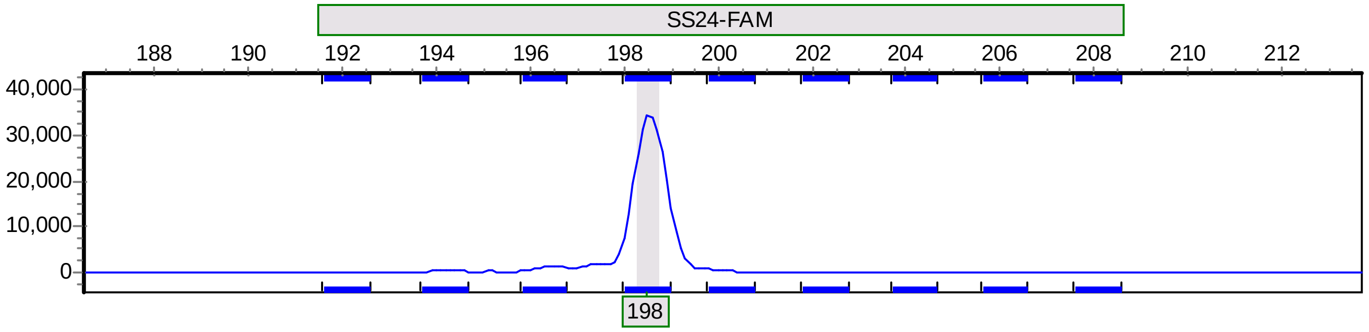

No	Size	Height	Area	Marker	Allele	Difference	Quality	Score	Allele Comments	Sample Comments
1	115.5	27463	184565	SSS18-FAM	116	0.00	Pass	500.0	[<Confirmed>]	
2	198.5	34073	264144	SS24-FAM	198	0.00	Pass	500.0	[<SAT (Repaired)><Confirmed>]	
3	245.6	22507	187786	SS05-FAM	245	0.00	Pass	500.0		
4	319.7	5151	51846	SS32-FAM	320	0.00	Pass	500.0		

5	325.9	3390	35901	SS32-FAM	326	0.10	Pass	286.6		

Sample 62: SSS18_SS24_SS05_SS32_SS30_SS12_SS23_HGY2_I14.fsa

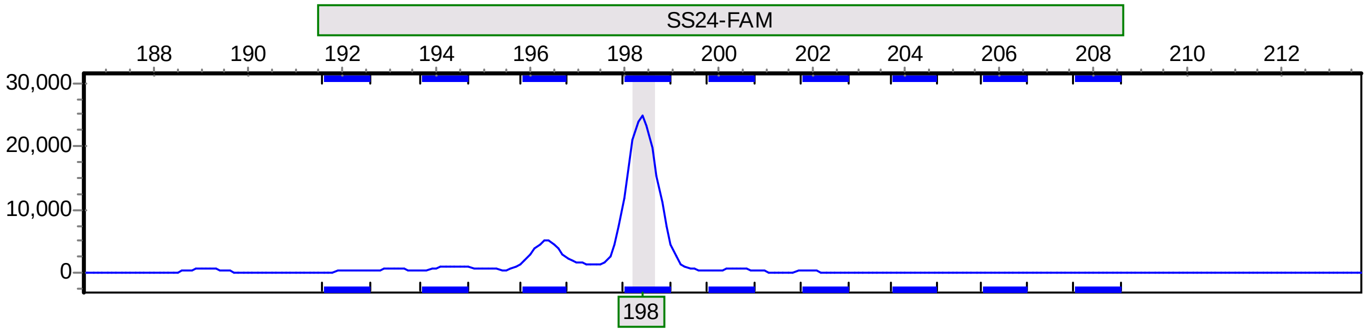

No	Size	Height	Area	Marker	Allele	Difference	Quality	Score	Allele Comments	Sample Comments
1	131.8	9825	67875	SSS18-FAM	132	0.10	Pass	500.0	[<Confirmed>]	
2	140.2	5855	47226	SSS18-FAM	140	0.10	Pass	500.0	[<Confirmed>]	
3	142.6	5276	40861	SSS18-FAM	142	0.00	Undetermined	500.0	[<Deleted>]	
4	198.4	24792	186075	SS24-FAM	198	0.10	Pass	500.0	[<Confirmed>]	
5	334.3	1870	19205	SS32-FAM	334	0.10	Pass	120.2		
6	340.3	1417	14146	SS32-FAM	340	0.10	Pass	94.5		

Sample 63: SSS18_SS24_SS05_SS32_SS30_SS12_SS23_HGY3_I12.fsa

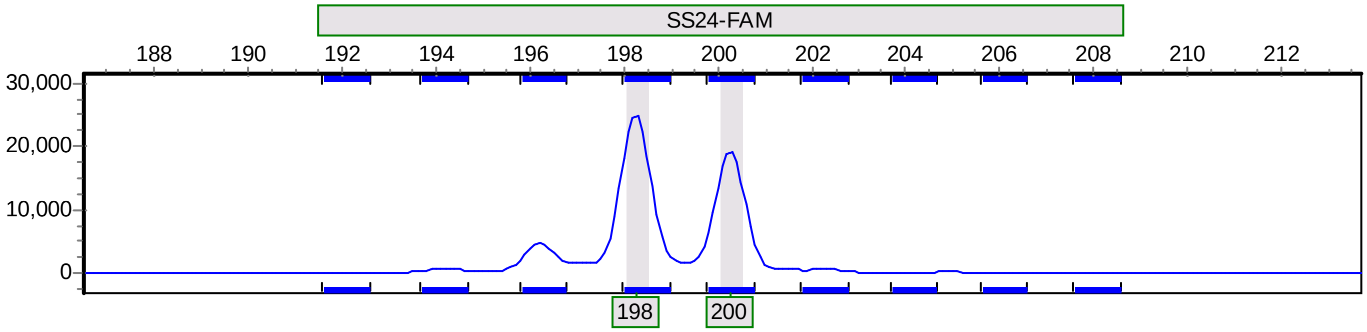

No	Size	Height	Area	Marker	Allele	Difference	Quality	Score	Allele Comments	Sample Comments
1	131.8	20175	138566	SSS18-FAM	132	0.10	Pass	500.0	[<Confirmed>]	
2	133.8	11769	82435	SSS18-FAM	134	0.10	Pass	500.0	[<Confirmed>]	
3	135.9	10222	72014	SSS18-FAM	136	0.10	Undetermined	500.0	[<Deleted>]	
4	198.3	24796	185914	SS24-FAM	198	0.20	Pass	500.0	[<Confirmed>]	
5	200.3	19297	141888	SS24-FAM	200	0.00	Pass	500.0	[<Confirmed>]	
6	338.3	3580	36078	SS32-FAM	338	0.00	Pass	359.3		
7	340.3	2502	23496	SS32-FAM	340	0.10	Pass	251.5		

Sample 64: SSS18_SS24_SS05_SS32_SS30_SS12_SS23_HGY4_I10.fsa

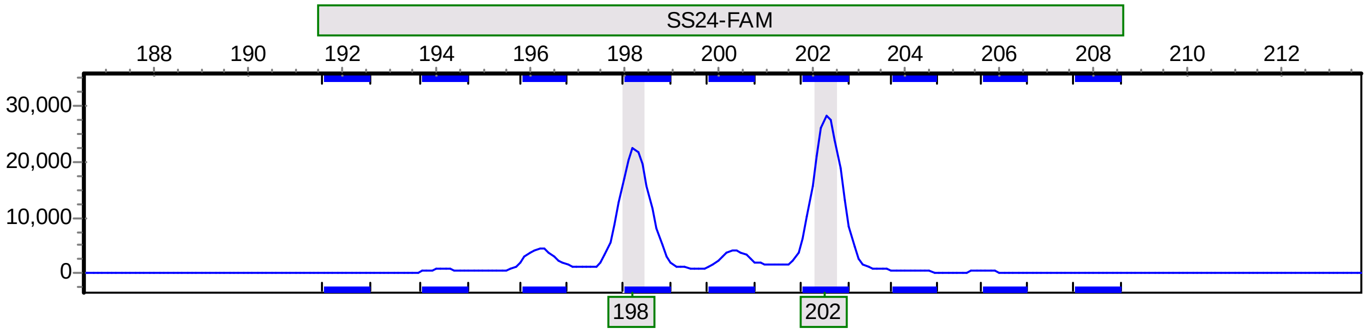

No	Size	Height	Area	Marker	Allele	Difference	Quality	Score	Allele Comments	Sample Comments
1	115.6	25132	170836	SSS18-FAM	116	0.10	Pass	500.0	[<Confirmed>]	

Allele Report

2	123.8	16855	114988	SSS18-FAM	124	0.10	Pass	500.0	[<Confirmed>]	
3	198.2	22461	167869	SS24-FAM	198	0.30	Pass	500.0	[<Confirmed>]	
4	202.3	28213	199950	SS24-FAM	202	0.00	Pass	500.0	[<Confirmed>]	
5	259.4	18048	154619	SS05-FAM	259	0.00	Pass	500.0		
6	261.5	14003	117921	SS05-FAM	261	0.00	Pass	500.0		
7	338.3	3889	40179	SS32-FAM	338	0.00	Pass	347.3		
8	340.3	2550	24811	SS32-FAM	340	0.10	Pass	221.2		

Sample 65: SSS18_SS24_SS05_SS32_SS30_SS12_SS23_HGY5_I16.fsa

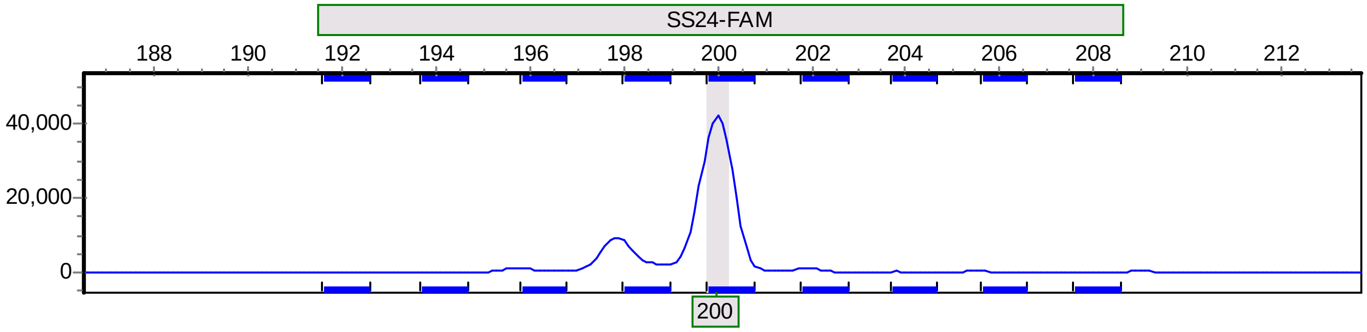

No	Size	Height	Area	Marker	Allele	Difference	Quality	Score	Allele Comments	Sample Comments
1	115.6	29250	200231	SSS18-FAM	116	0.10	Pass	500.0	[<Confirmed>]	
2	138.1	10260	74036	SSS18-FAM	138	0.00	Undetermined	500.0	[<Deleted>]	
3	140.3	10631	84020	SSS18-FAM	140	0.00	Pass	500.0	[<Confirmed>]	
4	200.0	41964	336297	SS24-FAM	200	0.30	Pass	500.0	[<SAT (Repaired)><Confirmed>]	
5	340.3	2303	23328	SS32-FAM	340	0.10	Pass	172.2		
6	345.9	1684	16611	SS32-FAM	346	0.10	Pass	122.1		

Sample 66: SSS18_SS24_SS05_SS32_SS30_SS12_SS23_HQZ11_O12.fsa

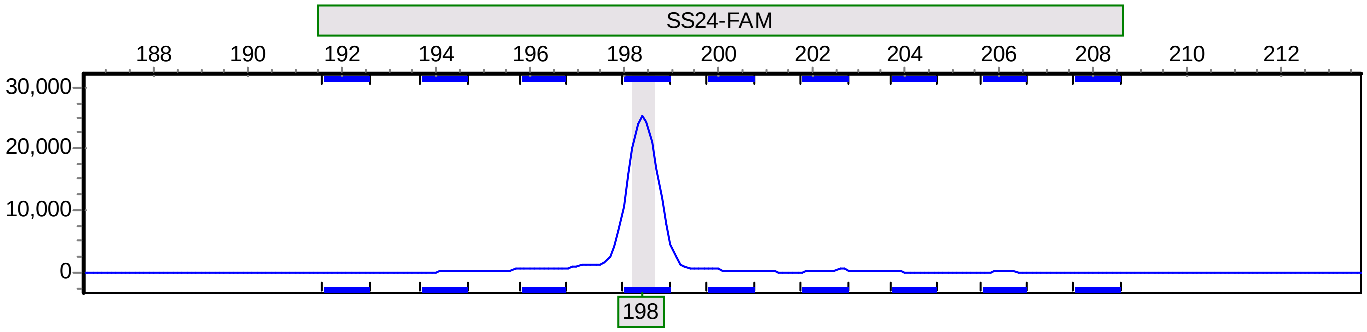

No	Size	Height	Area	Marker	Allele	Difference	Quality	Score	Allele Comments	Sample Comments
1	113.5	31630	227267	SSS18-FAM	114	0.00	Pass	500.0	[<Confirmed>]	
2	198.4	25202	187519	SS24-FAM	198	0.10	Pass	500.0	[<Confirmed>]	
3	249.0	4173	34897	SS05-FAM	249	0.20	Pass	500.0		
4	271.2	5171	44688	SS05-FAM	271	0.00	Pass	500.0		
5	325.8	3334	35379	SS32-FAM	326	0.20	Pass	284.0		
6	332.0	2456	25146	SS32-FAM	332	0.10	Pass	182.9		

Sample 67: SSS18_SS24_SS05_SS32_SS30_SS12_SS23_HQZ13-1_C10.fsa

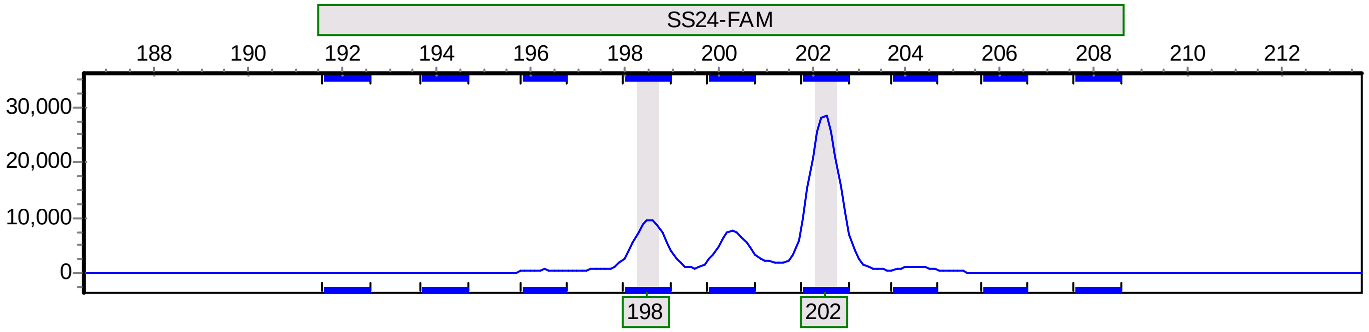

No	Size	Height	Area	Marker	Allele	Difference	Quality	Score	Allele Comments	Sample Comments
1	107.6	31276	269141	SSS18-FAM	108	0.10	Pass	500.0	[<Confirmed>]	
2	198.5	9466	77088	SS24-FAM	198	0.00	Pass	500.0	[<Confirmed>]	
3	202.3	28232	210892	SS24-FAM	202	0.00	Pass	500.0	[<Confirmed>]	
4	257.4	30691	262686	SS05-FAM	257	0.10	Check	500.0		
5	327.9	3043	32425	SS32-FAM	328	0.10	Pass	249.7		
6	334.1	2791	29970	SS32-FAM	334	0.10	Pass	211.7		

Sample 68: SSS18_SS24_SS05_SS32_SS30_SS12_SS23_HQZ13-2_J04.fsa

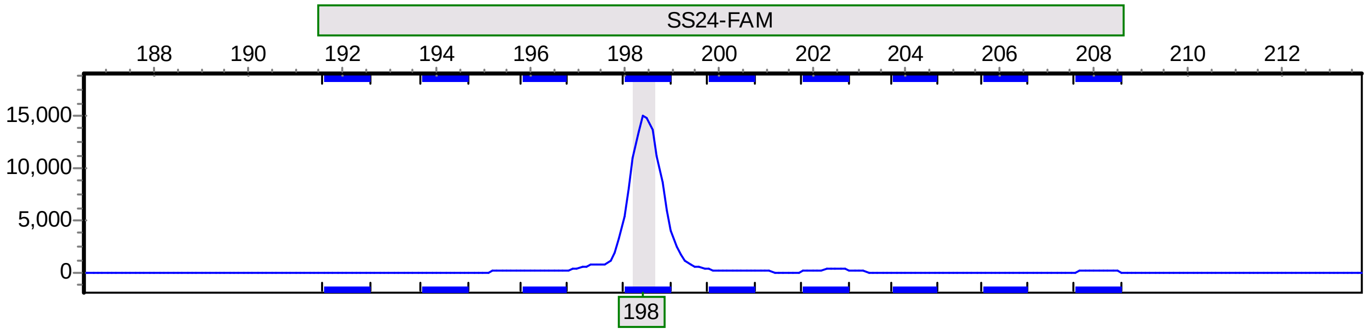

No	Size	Height	Area	Marker	Allele	Difference	Quality	Score	Allele Comments	Sample Comments
1	105.4	23937	161158	SSS18-FAM	106	0.00	Pass	500.0	[<Confirmed>]	
2	107.4	17046	112823	SSS18-FAM	108	0.10	Pass	500.0	[<Confirmed>]	
3	198.4	14936	114912	SS24-FAM	198	0.10	Pass	500.0	[<Confirmed>]	
4	253.4	9044	76954	SS05-FAM	253	0.10	Pass	500.0		
5	271.3	3917	35108	SS05-FAM	271	0.10	Pass	500.0		
6	319.8	2832	30302	SS32-FAM	320	0.10	Pass	213.8		
7	328.1	1960	20206	SS32-FAM	328	0.10	Pass	130.6		

Sample 69: SSS18_SS24_SS05_SS32_SS30_SS12_SS23_HQZ14_H04.fsa

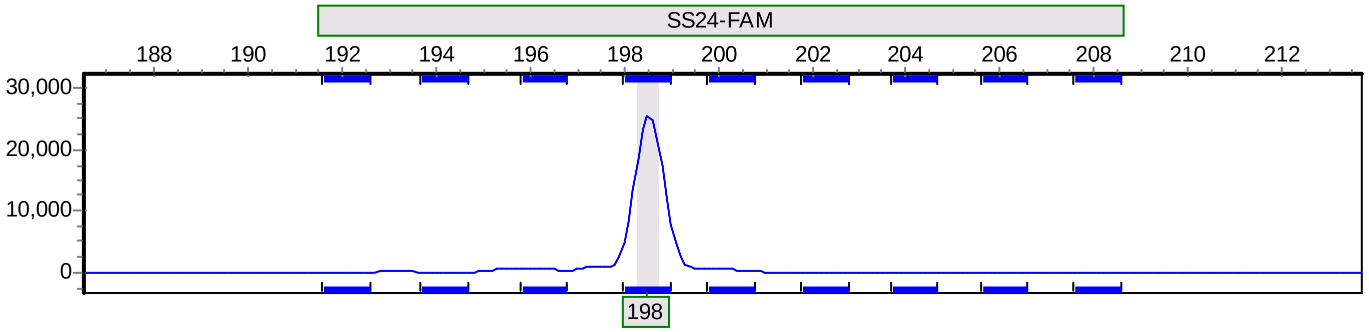

No	Size	Height	Area	Marker	Allele	Difference	Quality	Score	Allele Comments	Sample Comments
1	113.6	27759	178414	SSS18-FAM	114	0.10	Pass	500.0	[<Confirmed>]	
2	115.6	17450	111416	SSS18-FAM	116	0.10	Pass	500.0	[<Confirmed>]	

No	Size	Height	Area	Marker	Allele	Difference	Quality	Score	Allele Comments	Sample Comments
3	198.5	25386	176906	SS24-FAM	198	0.00	Pass	500.0	[<Confirmed>]	
4	271.3	10468	85673	SS05-FAM	271	0.10	Pass	500.0		
5	321.8	4327	43691	SS32-FAM	322	0.00	Pass	479.6		
6	326.0	3246	31681	SS32-FAM	326	0.00	Pass	321.1		

Sample 70: SSS18_SS24_SS05_SS32_SS30_SS12_SS23_HQZ15_B02.fsa

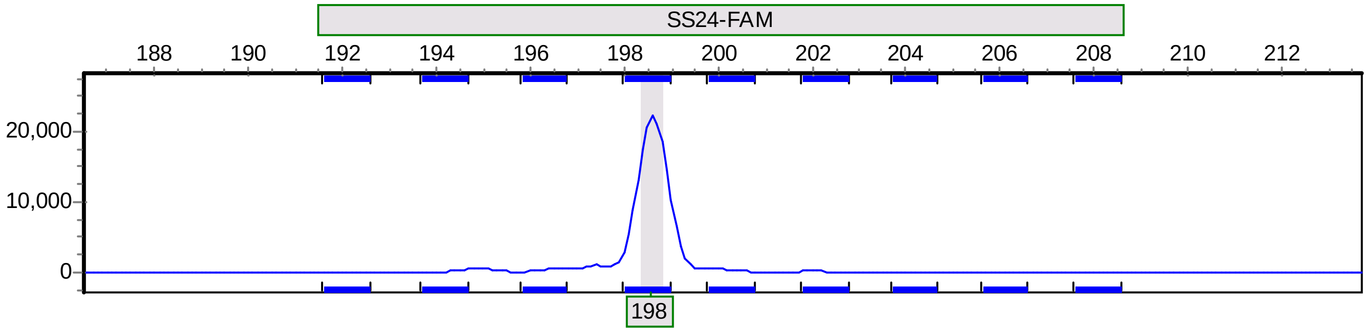

No	Size	Height	Area	Marker	Allele	Difference	Quality	Score	Allele Comments	Sample Comments
1	103.5	22079	141085	SSS18-FAM	104	0.10	Pass	500.0	[<Confirmed>]	
2	127.9	8833	58043	SSS18-FAM	128	0.10	Pass	500.0	[<Confirmed>]	
3	198.6	22303	160757	SS24-FAM	198	0.10	Pass	500.0	[<Confirmed>]	
4	245.8	14685	117473	SS05-FAM	245	0.20	Pass	500.0		
5	319.8	3654	36699	SS32-FAM	320	0.10	Pass	358.9		
6	328.0	2417	23885	SS32-FAM	328	0.00	Pass	214.4		

Sample 71: SSS18_SS24_SS05_SS32_SS30_SS12_SS23_HQZ16_P02.fsa

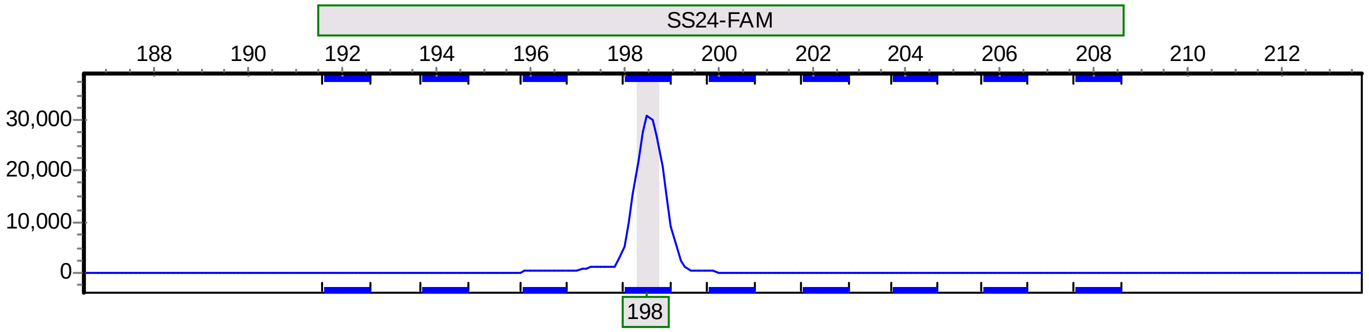

No	Size	Height	Area	Marker	Allele	Difference	Quality	Score	Allele Comments	Sample Comments
1	128.0	16783	105115	SSS18-FAM	128	0.20	Pass	500.0	[<Confirmed>]	
2	198.5	30531	209662	SS24-FAM	198	0.00	Pass	500.0	[<Confirmed>]	
3	246.0	25107	185181	SS05-FAM	245	0.40	Pass	500.0		
4	326.1	3504	33455	SS32-FAM	326	0.10	Pass	396.4		
5	332.3	2395	23295	SS32-FAM	332	0.20	Pass	218.1		

Sample 72: SSS18_SS24_SS05_SS32_SS30_SS12_SS23_HQZ17-1_O04.fsa

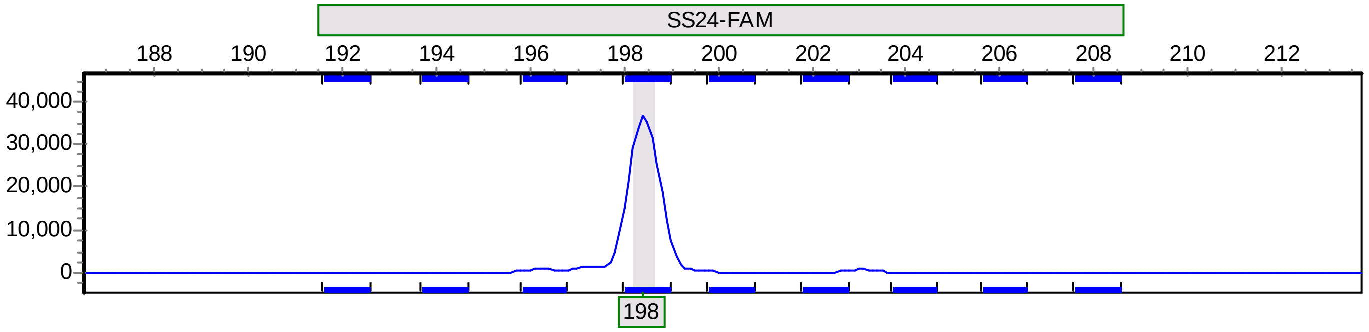

No	Size	Height	Area	Marker	Allele	Difference	Quality	Score	Allele Comments	Sample Comments

1	105.2	45151	337836	SSS18-FAM	106	0.20	Pass	500.0	[<SAT (Repaired)><Confirmed>]	
2	198.4	36365	269584	SS24-FAM	198	0.10	Pass	500.0	[<SAT (Repaired)><Confirmed>]	
3	245.8	31609	255263	SS05-FAM	245	0.20	Check	500.0		
4	319.8	5400	53014	SS32-FAM	320	0.10	Pass	500.0		
5	332.2	2794	27847	SS32-FAM	332	0.10	Pass	249.4		

Sample 73: SSS18_SS24_SS05_SS32_SS30_SS12_SS23_HQZ17-2_N04.fsa

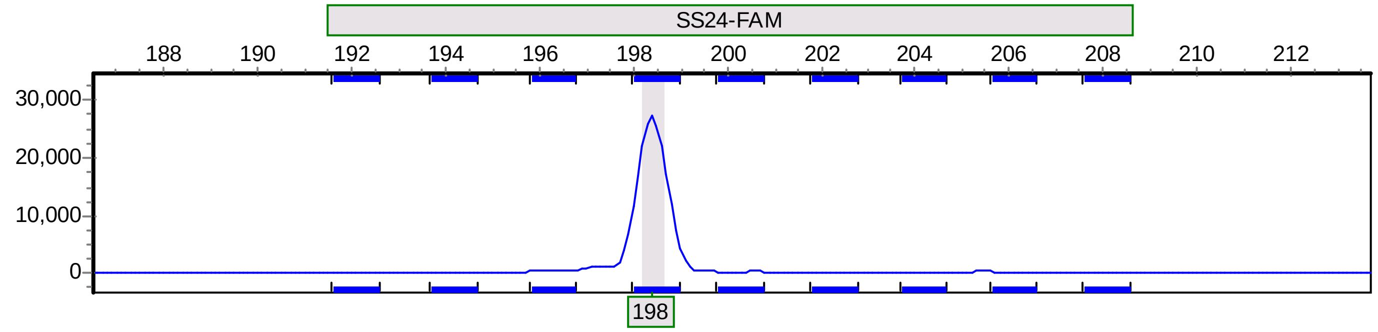

No	Size	Height	Area	Marker	Allele	Difference	Quality	Score	Allele Comments	Sample Comments
1	105.3	36149	271851	SSS18-FAM	106	0.10	Pass	500.0	[<SAT (Repaired)><Confirmed>]	
2	198.4	27228	194849	SS24-FAM	198	0.10	Pass	500.0	[<Confirmed>]	
3	245.6	23193	182654	SS05-FAM	245	0.00	Pass	500.0		
4	319.7	4023	40350	SS32-FAM	320	0.00	Pass	398.0		
5	332.0	2046	20779	SS32-FAM	332	0.10	Pass	146.6		

Sample 74: SSS18_SS24_SS05_SS32_SS30_SS12_SS23_HQZ18_K10.fsa

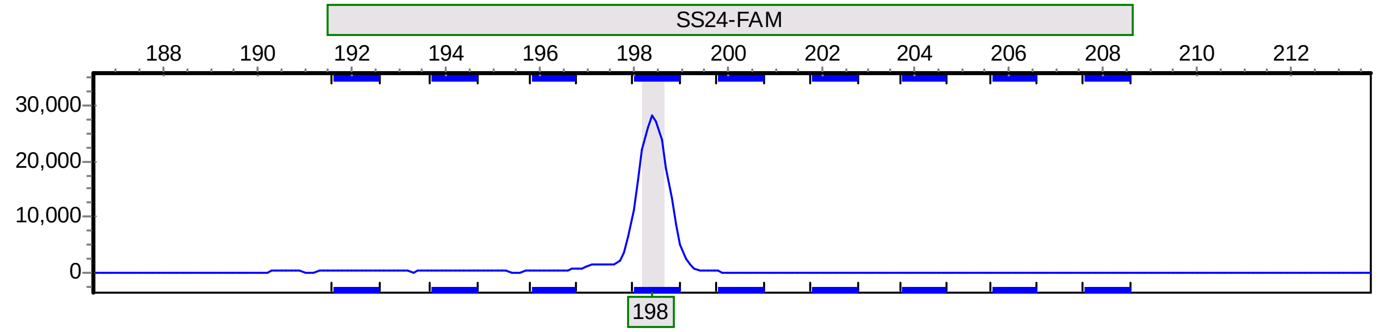

No	Size	Height	Area	Marker	Allele	Difference	Quality	Score	Allele Comments	Sample Comments
1	113.5	17970	119748	SSS18-FAM	114	0.00	Pass	500.0	[<Confirmed>]	
2	127.8	12346	82719	SSS18-FAM	128	0.00	Pass	500.0	[<Confirmed>]	
3	198.4	27990	202949	SS24-FAM	198	0.10	Pass	500.0	[<Confirmed>]	
4	245.7	20989	165179	SS05-FAM	245	0.10	Pass	500.0		
5	247.7	16622	134806	SS05-FAM	247	0.10	Pass	500.0		
6	326.0	3775	38026	SS32-FAM	326	0.00	Pass	359.3		
7	332.1	2596	26211	SS32-FAM	332	0.00	Pass	215.1		

Allele Report

Sample 75: SSS18_SS24_SS05_SS32_SS30_SS12_SS23_HQZ19_L04.fsa

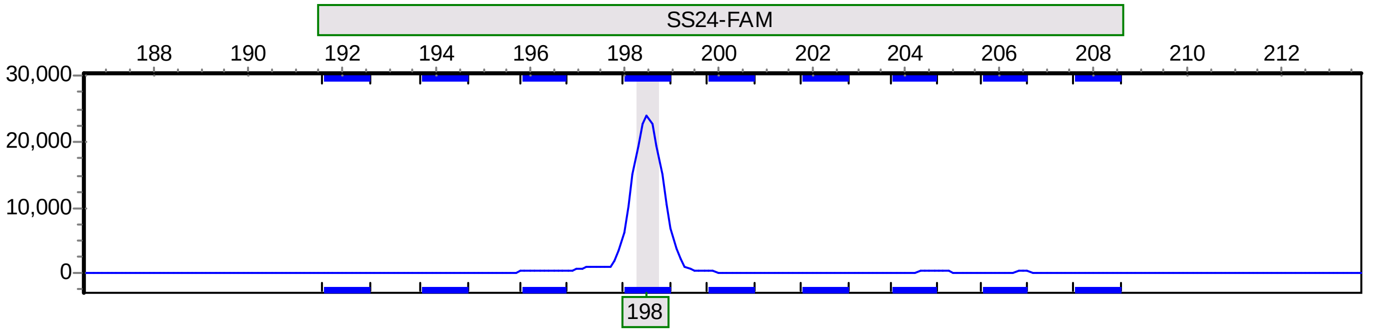

No	Size	Height	Area	Marker	Allele	Difference	Quality	Score	Allele Comments	Sample Comments
1	113.5	14357	94025	SSS18-FAM	114	0.00	Pass	500.0	[<Confirmed>]	
2	115.5	9178	60670	SSS18-FAM	116	0.00	Pass	500.0	[<Confirmed>]	
3	198.5	23861	172529	SS24-FAM	198	0.00	Pass	500.0	[<Confirmed>]	
4	245.6	11892	97200	SS05-FAM	245	0.00	Pass	500.0		
5	247.6	10289	82150	SS05-FAM	247	0.00	Pass	500.0		
6	332.2	3314	33600	SS32-FAM	332	0.10	Pass	295.9		
7	334.2	2100	21721	SS32-FAM	334	0.00	Pass	162.1		

Sample 76: SSS18_SS24_SS05_SS32_SS30_SS12_SS23_HQZ21_B04.fsa

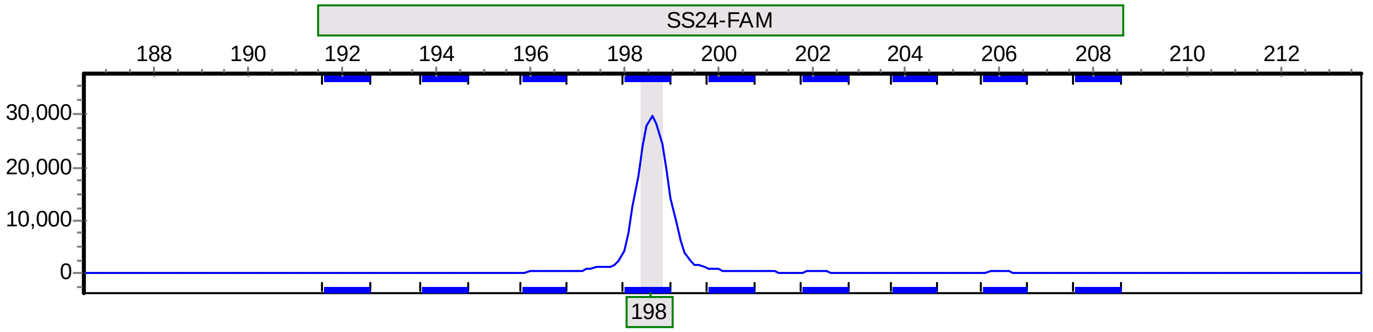

No	Size	Height	Area	Marker	Allele	Difference	Quality	Score	Allele Comments	Sample Comments
1	113.5	27963	187073	SSS18-FAM	114	0.00	Pass	500.0	[<Confirmed>]	
2	115.5	18586	126749	SSS18-FAM	116	0.00	Pass	500.0	[<Confirmed>]	
3	198.6	29540	219398	SS24-FAM	198	0.10	Pass	500.0	[<Confirmed>]	
4	245.7	30616	256517	SS05-FAM	245	0.10	Check	500.0		
5	326.0	3762	39986	SS32-FAM	326	0.00	Pass	344.5		
6	332.2	2600	27030	SS32-FAM	332	0.10	Pass	187.7		

Sample 77: SSS18_SS24_SS05_SS32_SS30_SS12_SS23_HQZ22-1_M02.fsa

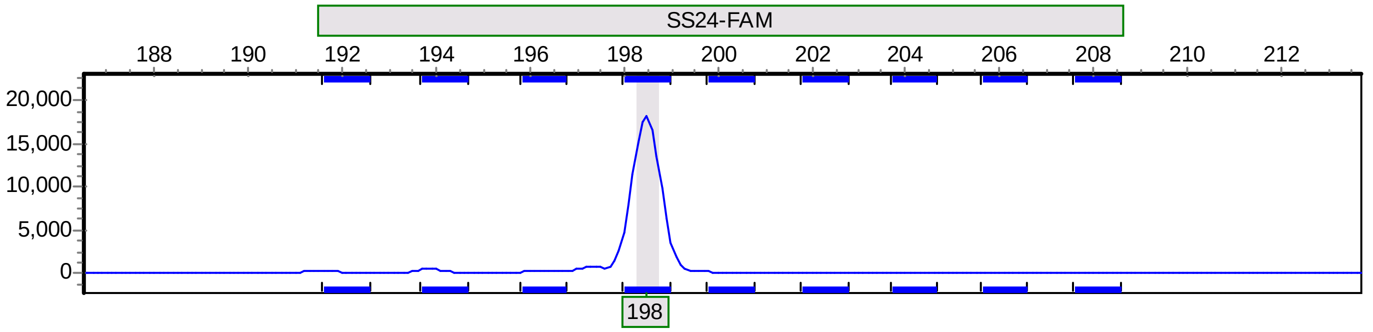

No	Size	Height	Area	Marker	Allele	Difference	Quality	Score	Allele Comments	Sample Comments
1	113.7	19273	125492	SSS18-FAM	114	0.20	Pass	500.0	[<Confirmed>]	
2	115.7	14646	93097	SSS18-FAM	116	0.20	Pass	500.0	[<Confirmed>]	

3	198.5	18032	122446	SS24-FAM	198	0.00	Pass	500.0	[<Confirmed>]	
4	245.9	17566	134477	SS05-FAM	245	0.30	Pass	500.0		
5	326.0	2652	26450	SS32-FAM	326	0.00	Pass	247.5		
6	332.1	1883	18872	SS32-FAM	332	0.00	Pass	136.0		

Sample 78: SSS18_SS24_SS05_SS32_SS30_SS12_SS23_HQZ22-2_O14.fsa

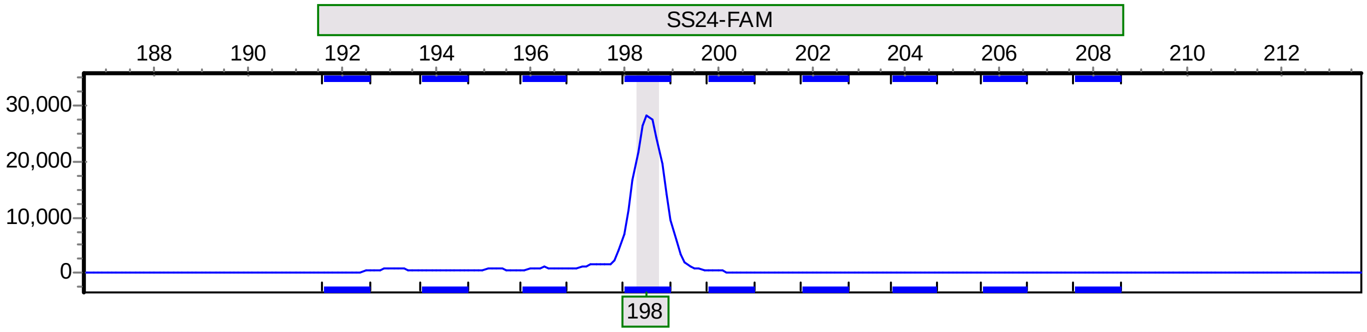

No	Size	Height	Area	Marker	Allele	Difference	Quality	Score	Allele Comments	Sample Comments
1	127.9	21842	148434	SSS18-FAM	128	0.10	Pass	500.0	[<Confirmed>]	
2	198.5	28034	208830	SS24-FAM	198	0.00	Pass	500.0	[<Confirmed>]	
3	245.6	18793	151557	SS05-FAM	245	0.00	Pass	500.0		
4	326.1	3780	37948	SS32-FAM	326	0.10	Pass	369.5		
5	332.2	2452	25786	SS32-FAM	332	0.10	Pass	189.2		

Sample 79: SSS18_SS24_SS05_SS32_SS30_SS12_SS23_HQZ23_A16.fsa

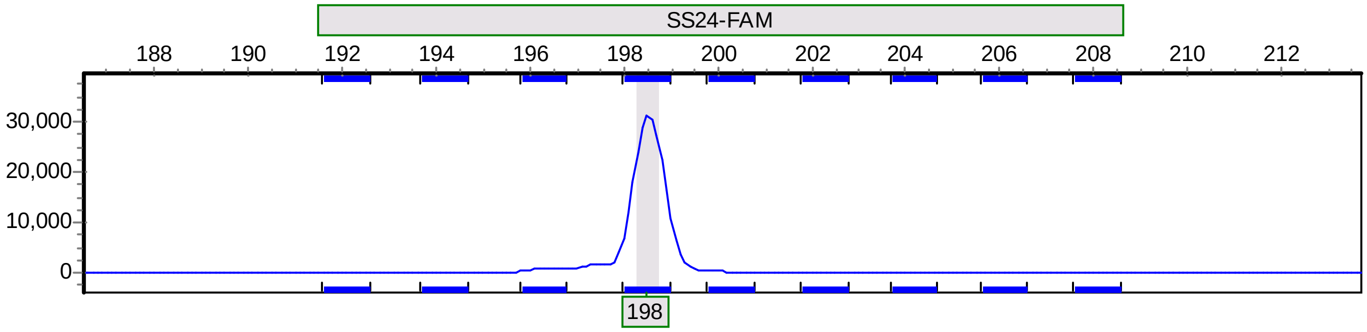

No	Size	Height	Area	Marker	Allele	Difference	Quality	Score	Allele Comments	Sample Comments
1	115.4	19829	135227	SSS18-FAM	116	0.10	Pass	500.0	[<Confirmed>]	
2	198.5	30888	229437	SS24-FAM	198	0.00	Pass	500.0	[<Confirmed>]	
3	247.6	25913	208236	SS05-FAM	247	0.00	Pass	500.0		
4	325.9	6409	64655	SS32-FAM	326	0.10	Pass	500.0		

Sample 80: SSS18_SS24_SS05_SS32_SS30_SS12_SS23_HQZ24_E12.fsa

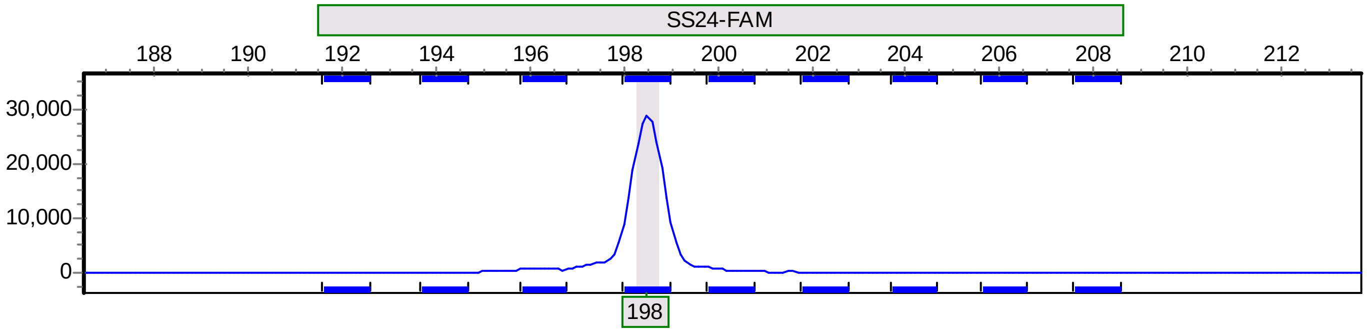

No	Size	Height	Area	Marker	Allele	Difference	Quality	Score	Allele Comments	Sample Comments
1	103.4	30121	199402	SSS18-FAM	104	0.00	Pass	500.0	[<Confirmed>]	
2	113.5	17271	115338	SSS18-FAM	114	0.00	Pass	500.0	[<Confirmed>]	

3	198.5	28790	221527	SS24-FAM	198	0.00	Pass	500.0	[<Confirmed>]	
4	247.5	15356	127178	SS05-FAM	247	0.10	Pass	500.0		
5	325.9	2824	28745	SS32-FAM	326	0.10	Pass	231.3		
6	332.1	2271	23087	SS32-FAM	332	0.00	Pass	171.2		

Sample 81: SSS18_SS24_SS05_SS32_SS30_SS12_SS23_HQZ25_N02.fsa

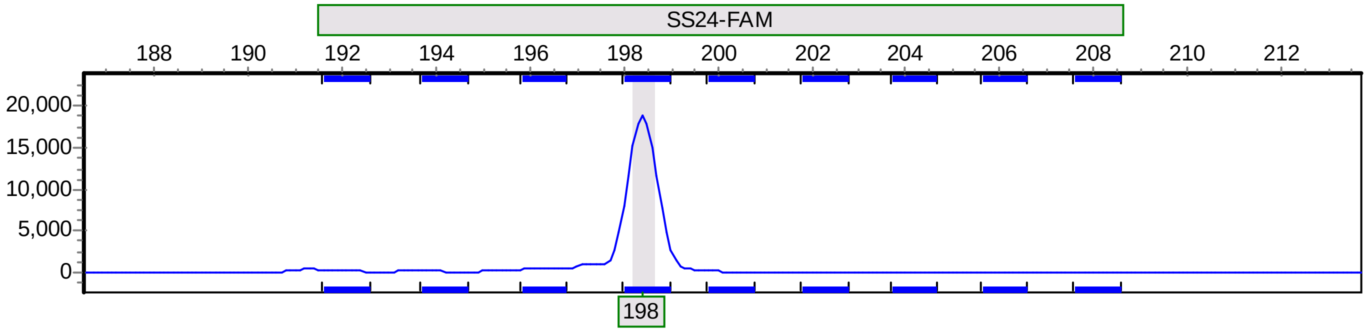

No	Size	Height	Area	Marker	Allele	Difference	Quality	Score	Allele Comments	Sample Comments
1	97.5	26246	177265	SSS18-FAM	98	0.10	Pass	500.0	[<Confirmed>]	
2	115.7	15296	98949	SSS18-FAM	116	0.20	Pass	500.0	[<Confirmed>]	
3	198.4	18805	132938	SS24-FAM	198	0.10	Pass	500.0	[<Confirmed>]	
4	245.7	11011	83963	SS05-FAM	245	0.10	Pass	500.0		
5	271.4	7272	58048	SS05-FAM	271	0.20	Pass	500.0		
6	332.2	3353	34030	SS32-FAM	332	0.10	Pass	306.7		
7	334.3	2184	22272	SS32-FAM	334	0.10	Pass	154.8		

Sample 82: SSS18_SS24_SS05_SS32_SS30_SS12_SS23_HQZ26_E04.fsa

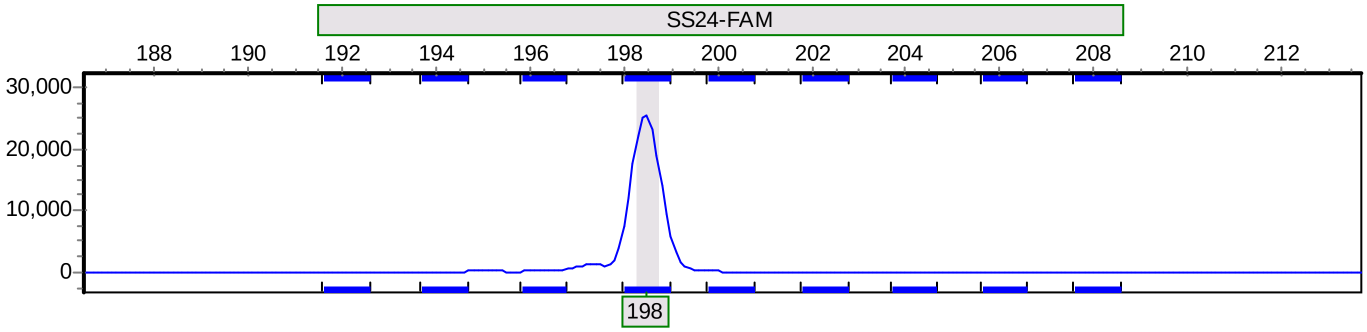

No	Size	Height	Area	Marker	Allele	Difference	Quality	Score	Allele Comments	Sample Comments
1	113.5	26195	169866	SSS18-FAM	114	0.00	Pass	500.0	[<Confirmed>]	
2	198.5	25266	180731	SS24-FAM	198	0.00	Pass	500.0	[<Confirmed>]	
3	247.7	13462	120759	SS05-FAM	247	0.10	Pass	500.0		
4	249.1	11227	89963	SS05-FAM	249	0.10	Pass	500.0		
5	326.0	6764	65124	SS32-FAM	326	0.00	Pass	500.0		

Allele Report

Sample 83: SSS18_SS24_SS05_SS32_SS30_SS12_SS23_HQZ27_G10.fsa

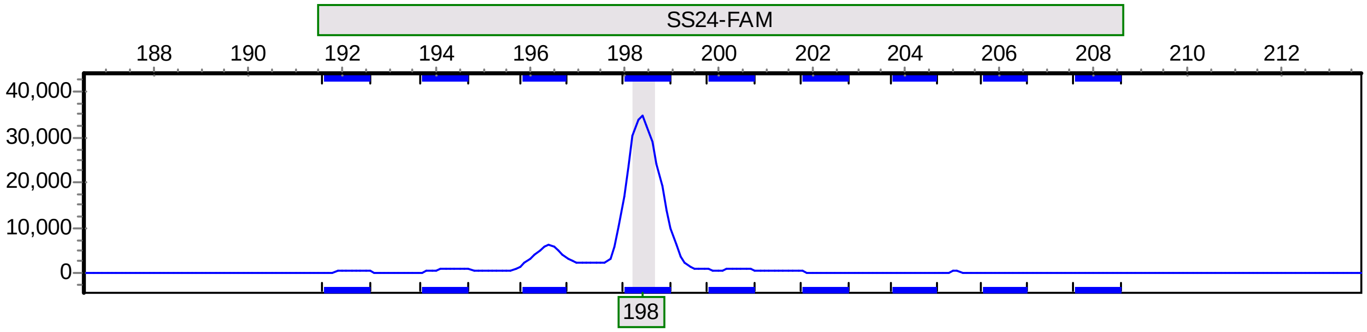

No	Size	Height	Area	Marker	Allele	Difference	Quality	Score	Allele Comments	Sample Comments
1	99.5	5105	82592	SSS18-FAM	100	0.20	Undetermined	109.8	[<Deleted>]	
2	103.4	15774	104814	SSS18-FAM	104	0.00	Pass	500.0	[<Confirmed>]	
3	142.6	12615	97299	SSS18-FAM	142	0.00	Pass	500.0	[<Confirmed>]	
4	144.9	11487	88644	SSS18-FAM	144	0.00	Undetermined	500.0	[<Deleted>]	
5	198.4	34543	279628	SS24-FAM	198	0.10	Pass	500.0	[<SAT (Repaired)><Confirmed>]	
6	247.6	6563	53654	SS05-FAM	247	0.00	Pass	500.0		
7	283.5	10153	91425	SS05-FAM	283	0.00	Pass	500.0		
8	326.0	4168	44020	SS32-FAM	326	0.00	Pass	393.0		
9	347.7	1551	15159	SS32-FAM	348	0.00	Pass	113.8		

Sample 84: SSS18_SS24_SS05_SS32_SS30_SS12_SS23_HQZ28_B06.fsa

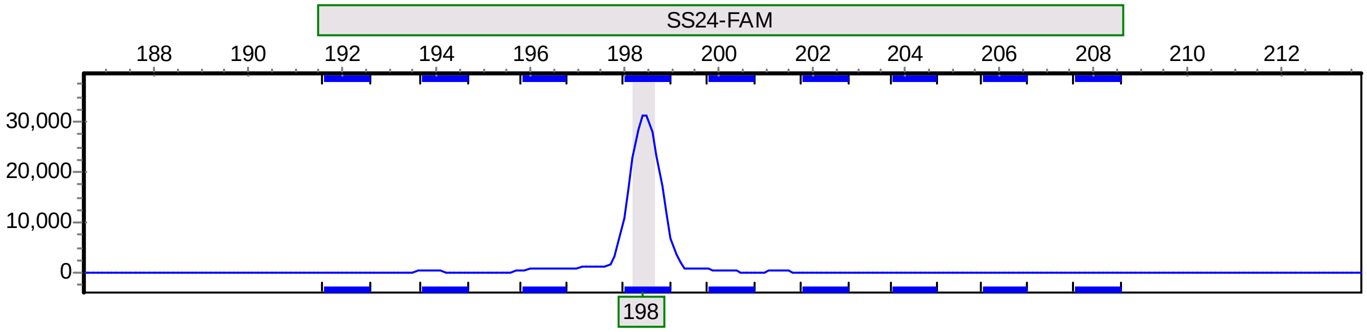

No	Size	Height	Area	Marker	Allele	Difference	Quality	Score	Allele Comments	Sample Comments
1	105.4	31461	225366	SSS18-FAM	106	0.00	Pass	500.0	[<Confirmed>]	
2	115.5	19707	127881	SSS18-FAM	116	0.00	Pass	500.0	[<Confirmed>]	
3	198.4	31128	231334	SS24-FAM	198	0.10	Pass	500.0	[<Confirmed>]	
4	245.6	19577	158696	SS05-FAM	245	0.00	Pass	500.0		
5	249.1	11996	96726	SS05-FAM	249	0.10	Pass	500.0		
6	326.0	4287	42278	SS32-FAM	326	0.00	Pass	486.8		
7	332.2	2838	27838	SS32-FAM	332	0.10	Pass	273.9		

Allele Report

Sample 85: SSS18_SS24_SS05_SS32_SS30_SS12_SS23_HQZ29_C14.fsa

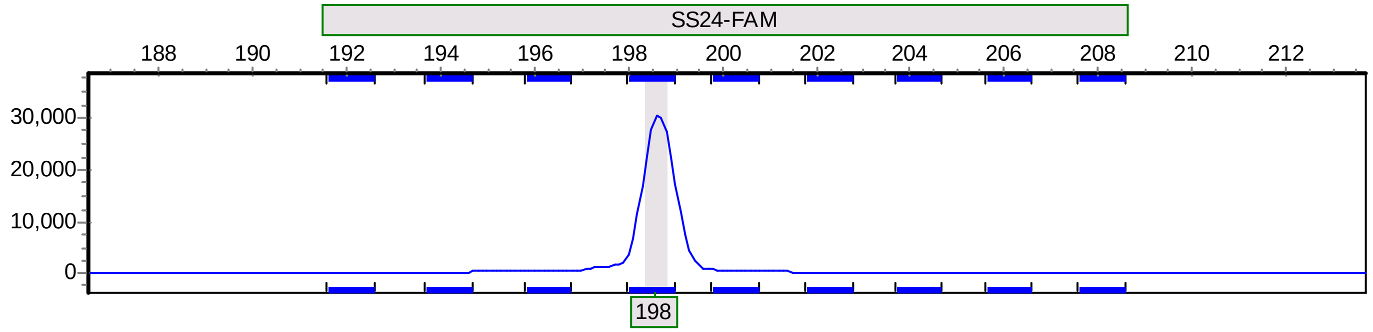

No	Size	Height	Area	Marker	Allele	Difference	Quality	Score	Allele Comments	Sample Comments
1	115.3	21581	144752	SSS18-FAM	116	0.20	Pass	500.0	[<Confirmed>]	
2	198.6	30299	229713	SS24-FAM	198	0.10	Pass	500.0	[<Confirmed>]	
3	245.6	22442	183920	SS05-FAM	245	0.00	Pass	500.0		
4	325.9	3703	39166	SS32-FAM	326	0.10	Pass	316.1		
5	332.0	2646	27764	SS32-FAM	332	0.10	Pass	208.0		

Sample 86: SSS18_SS24_SS05_SS32_SS30_SS12_SS23_HQZ2_K04.fsa

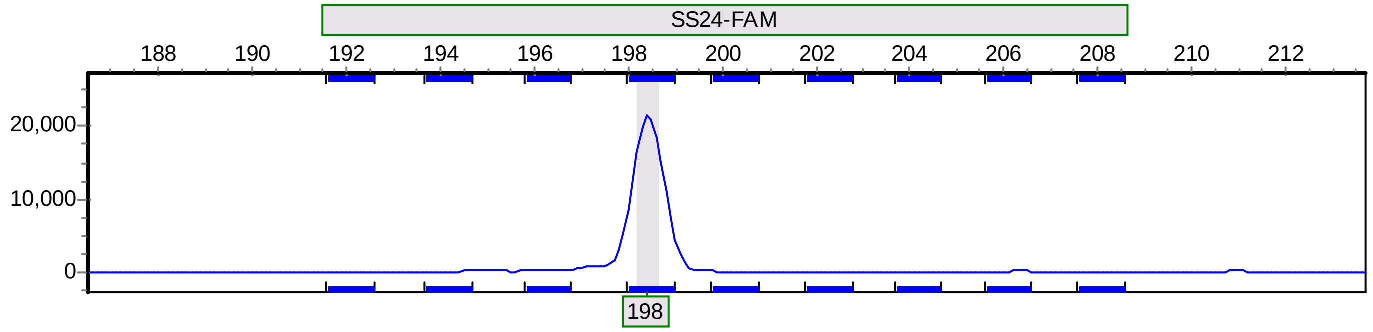

No	Size	Height	Area	Marker	Allele	Difference	Quality	Score	Allele Comments	Sample Comments
1	107.6	22632	143840	SSS18-FAM	108	0.10	Pass	500.0	[<Confirmed>]	
2	115.6	14210	91761	SSS18-FAM	116	0.10	Pass	500.0	[<Confirmed>]	
3	198.4	21338	159795	SS24-FAM	198	0.10	Pass	500.0	[<Confirmed>]	
4	245.7	11533	92069	SS05-FAM	245	0.10	Pass	500.0		
5	263.4	9329	77091	SS05-FAM	263	0.00	Pass	500.0		
6	319.8	3909	39245	SS32-FAM	320	0.10	Pass	399.5		
7	326.0	2605	25997	SS32-FAM	326	0.00	Pass	226.2		

Sample 87: SSS18_SS24_SS05_SS32_SS30_SS12_SS23_HQZ30_G16.fsa

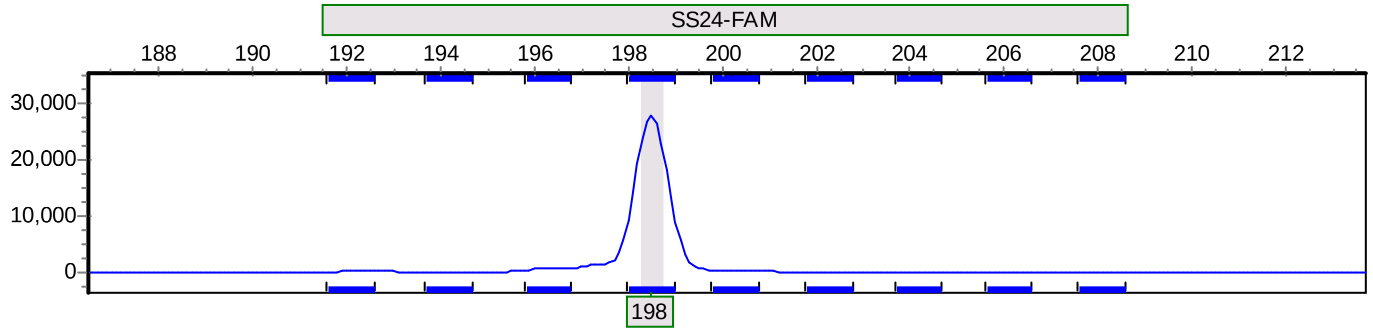

No	Size	Height	Area	Marker	Allele	Difference	Quality	Score	Allele Comments	Sample Comments
1	113.4	24980	169860	SSS18-FAM	114	0.10	Pass	500.0	[<Confirmed>]	
2	198.5	27869	219761	SS24-FAM	198	0.00	Pass	500.0	[<Confirmed>]	
3	245.5	19642	159957	SS05-FAM	245	0.10	Pass	500.0		

4	325.8	4044	41921	SS32-FAM	326	0.20	Pass	353.2		
5	327.8	2826	29234	SS32-FAM	328	0.20	Pass	225.5		

Sample 88: SSS18_SS24_SS05_SS32_SS30_SS12_SS23_HQZ31_K14.fsa

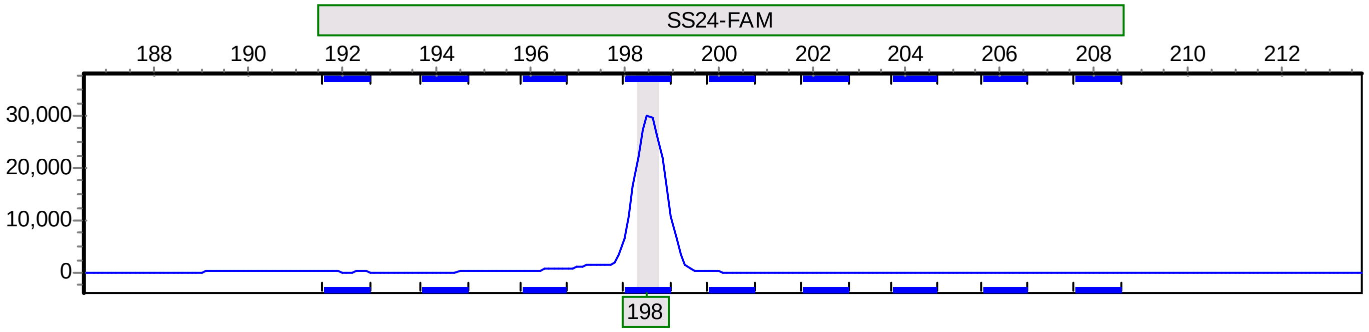

No	Size	Height	Area	Marker	Allele	Difference	Quality	Score	Allele Comments	Sample Comments
1	107.5	30722	212153	SSS18-FAM	108	0.00	Pass	500.0	[<Confirmed>]	
2	198.5	30020	221470	SS24-FAM	198	0.00	Pass	500.0	[<Confirmed>]	
3	247.6	21545	171979	SS05-FAM	247	0.00	Pass	500.0		
4	319.7	4056	40516	SS32-FAM	320	0.00	Pass	421.7		
5	325.9	3549	35672	SS32-FAM	326	0.10	Pass	350.3		

Sample 89: SSS18_SS24_SS05_SS32_SS30_SS12_SS23_HQZ32_J02.fsa

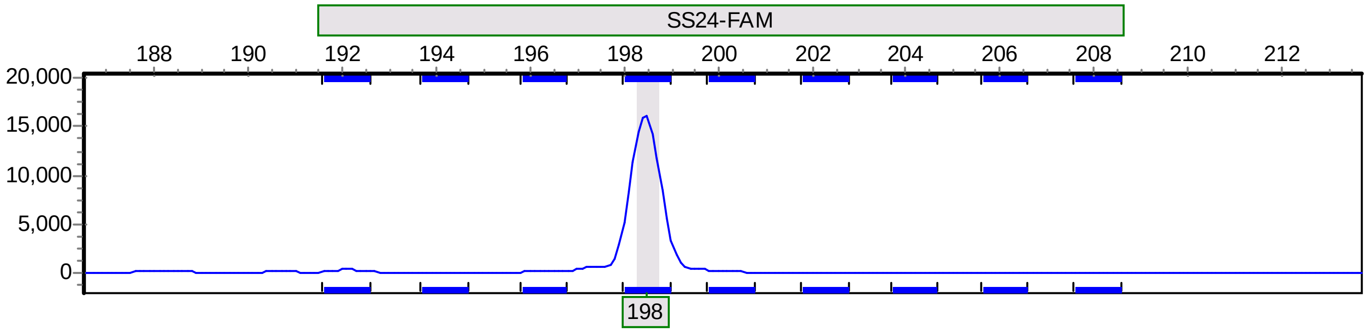

No	Size	Height	Area	Marker	Allele	Difference	Quality	Score	Allele Comments	Sample Comments
1	99.3	3422	54940	SSS18-FAM	100	0.40	Undetermined	114.3	[<Deleted>]	
2	128.0	8498	55555	SSS18-FAM	128	0.20	Pass	500.0	[<Confirmed>]	
3	132.0	4416	29453	SSS18-FAM	132	0.10	Undetermined	500.0	[<Deleted>]	
4	134.1	4785	32010	SSS18-FAM	134	0.20	Pass	500.0	[<Confirmed>]	
5	198.5	15967	113913	SS24-FAM	198	0.00	Pass	500.0	[<Confirmed>]	
6	245.8	15075	115940	SS05-FAM	245	0.20	Pass	500.0		
7	332.3	3299	33108	SS32-FAM	332	0.20	Pass	312.2		

Sample 90: SSS18_SS24_SS05_SS32_SS30_SS12_SS23_HQZ33_I04.fsa

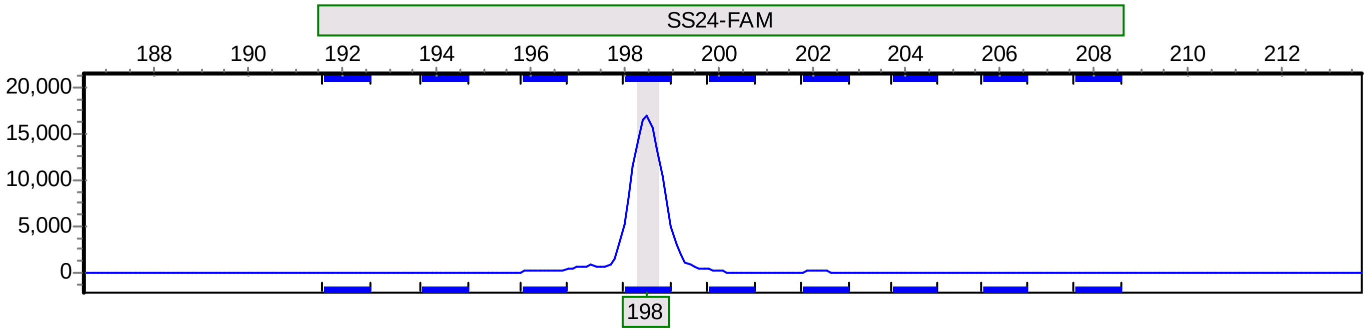

No	Size	Height	Area	Marker	Allele	Difference	Quality	Score	Allele Comments	Sample Comments
1	113.4	13826	92678	SSS18-FAM	114	0.10	Pass	500.0	[<Confirmed>]	

2	198.5	16836	126933	SS24-FAM	198	0.00	Pass	500.0	[<Confirmed>]	
3	245.6	8397	72193	SS05-FAM	245	0.00	Pass	500.0		
4	247.6	6887	58613	SS05-FAM	247	0.00	Pass	500.0		
5	319.7	2500	25742	SS32-FAM	320	0.00	Pass	186.4		
6	328.0	1659	16765	SS32-FAM	328	0.00	Pass	109.4		

Sample 91: SSS18_SS24_SS05_SS32_SS30_SS12_SS23_HQZ34_M04.fsa

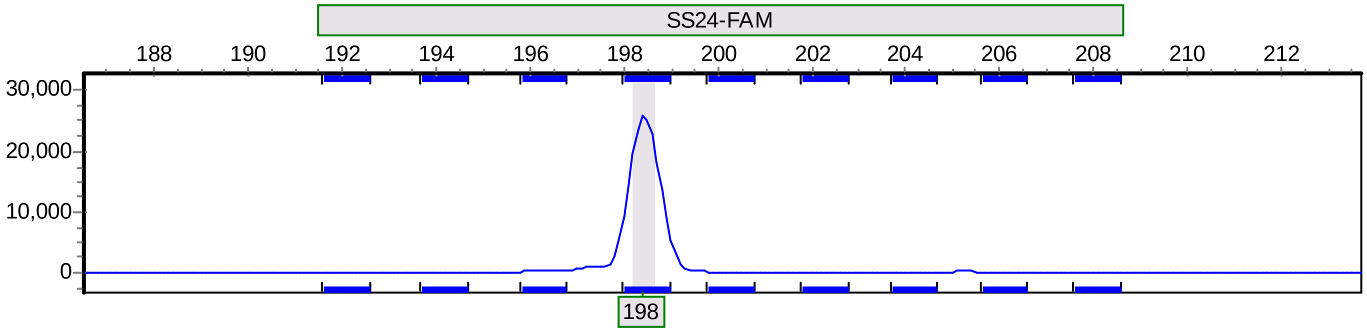

No	Size	Height	Area	Marker	Allele	Difference	Quality	Score	Allele Comments	Sample Comments
1	97.3	30651	216570	SSS18-FAM	98	0.10	Pass	500.0	[<Confirmed>]	
2	115.4	15932	102789	SSS18-FAM	116	0.10	Pass	500.0	[<Confirmed>]	
3	198.4	25709	187359	SS24-FAM	198	0.10	Pass	500.0	[<Confirmed>]	
4	245.6	13383	107446	SS05-FAM	245	0.00	Pass	500.0		
5	247.6	11887	95869	SS05-FAM	247	0.00	Pass	500.0		
6	328.1	5207	51662	SS32-FAM	328	0.10	Pass	500.0		

Sample 92: SSS18_SS24_SS05_SS32_SS30_SS12_SS23_HQZ35_A02.fsa

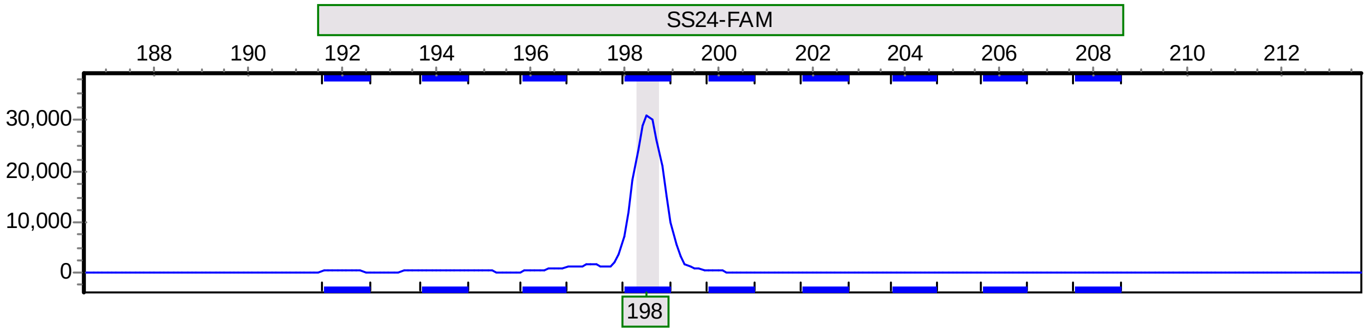

No	Size	Height	Area	Marker	Allele	Difference	Quality	Score	Allele Comments	Sample Comments
1	113.4	20351	132935	SSS18-FAM	114	0.10	Pass	500.0	[<Confirmed>]	
2	121.5	13002	84492	SSS18-FAM	122	0.20	Pass	500.0	[<Confirmed>]	
3	198.5	30807	223465	SS24-FAM	198	0.00	Pass	500.0	[<Confirmed>]	
4	247.8	10992	98322	SS05-FAM	247	0.20	Pass	500.0		
5	249.2	9362	76925	SS05-FAM	249	0.00	Pass	500.0		
6	319.8	3299	32732	SS32-FAM	320	0.10	Pass	339.3		
7	326.1	2357	23057	SS32-FAM	326	0.10	Pass	192.1		

Allele Report

Sample 93: SSS18_SS24_SS05_SS32_SS30_SS12_SS23_HQZ36_E16.fsa

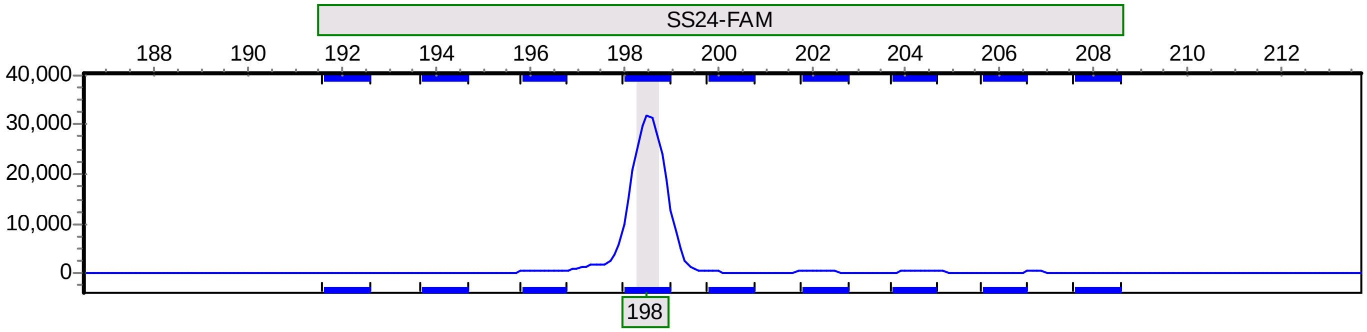

No	Size	Height	Area	Marker	Allele	Difference	Quality	Score	Allele Comments	Sample Comments
1	97.4	30310	220468	SSS18-FAM	98	0.00	Pass	500.0	[<Confirmed>]	
2	115.4	16653	112187	SSS18-FAM	116	0.10	Pass	500.0	[<Confirmed>]	
3	198.5	31599	258985	SS24-FAM	198	0.00	Pass	500.0	[<Confirmed>]	
4	245.5	20727	173275	SS05-FAM	245	0.10	Pass	500.0		
5	247.5	14506	122077	SS05-FAM	247	0.10	Pass	500.0		
6	325.8	4204	44707	SS32-FAM	326	0.20	Pass	390.0		
7	332.0	2652	28887	SS32-FAM	332	0.10	Pass	177.5		

Sample 94: SSS18_SS24_SS05_SS32_SS30_SS12_SS23_HQZ37_F04.fsa

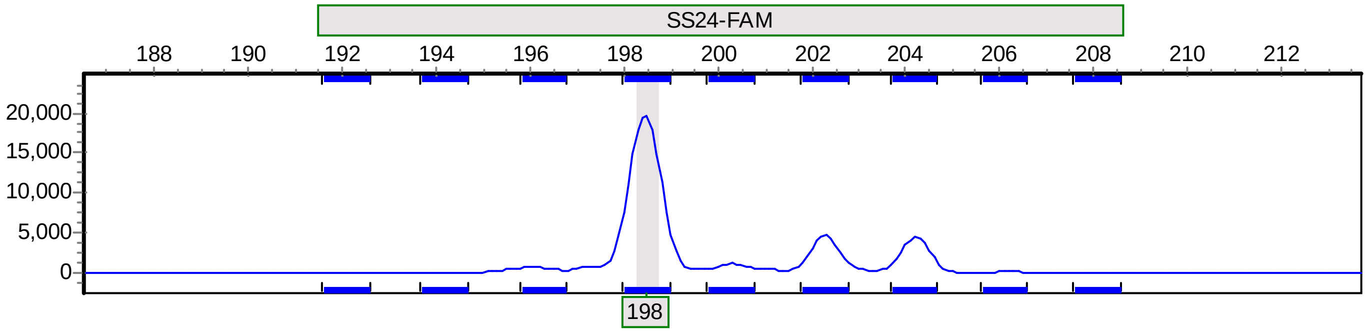

No	Size	Height	Area	Marker	Allele	Difference	Quality	Score	Allele Comments	Sample Comments
1	113.5	22438	142562	SSS18-FAM	114	0.00	Pass	500.0	[<Confirmed>]	
2	198.5	19519	150595	SS24-FAM	198	0.00	Pass	500.0	[<Confirmed>]	
3	245.7	23000	182464	SS05-FAM	245	0.10	Pass	500.0		
4	323.9	4548	44964	SS32-FAM	324	0.00	Pass	500.0		
5	326.0	3250	32494	SS32-FAM	326	0.00	Pass	310.4		

Sample 95: SSS18_SS24_SS05_SS32_SS30_SS12_SS23_HQZ38_L02.fsa

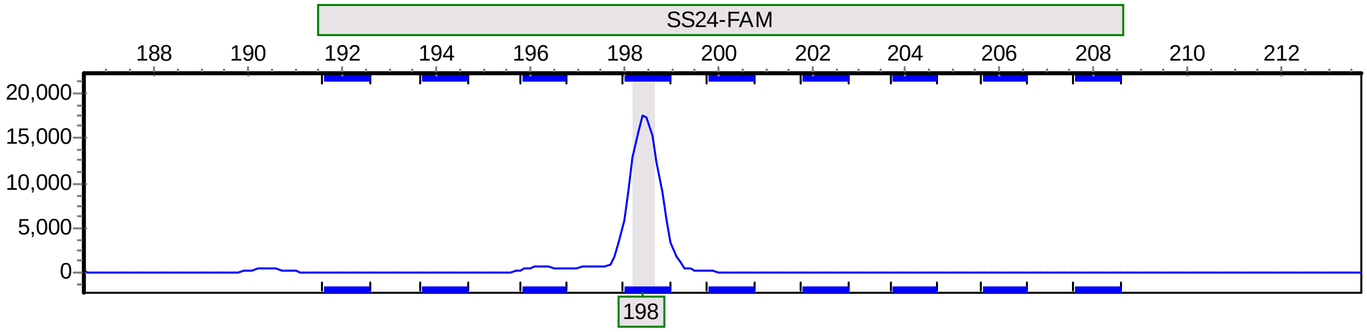

No	Size	Height	Area	Marker	Allele	Difference	Quality	Score	Allele Comments	Sample Comments
1	113.5	11234	72152	SSS18-FAM	114	0.00	Pass	500.0	[<Confirmed>]	
2	127.7	8431	56126	SSS18-FAM	128	0.10	Pass	500.0	[<Confirmed>]	
3	198.4	17428	123915	SS24-FAM	198	0.10	Pass	500.0	[<Confirmed>]	

4	245.7	10655	83695	SS05-FAM	245	0.10	Pass	500.0		
5	247.7	6681	53807	SS05-FAM	247	0.10	Pass	500.0		
6	319.8	2987	31138	SS32-FAM	320	0.10	Pass	256.3		
7	328.1	2124	22028	SS32-FAM	328	0.10	Pass	154.6		

Sample 96: SSS18_SS24_SS05_SS32_SS30_SS12_SS23_HQZ39_M16.fsa

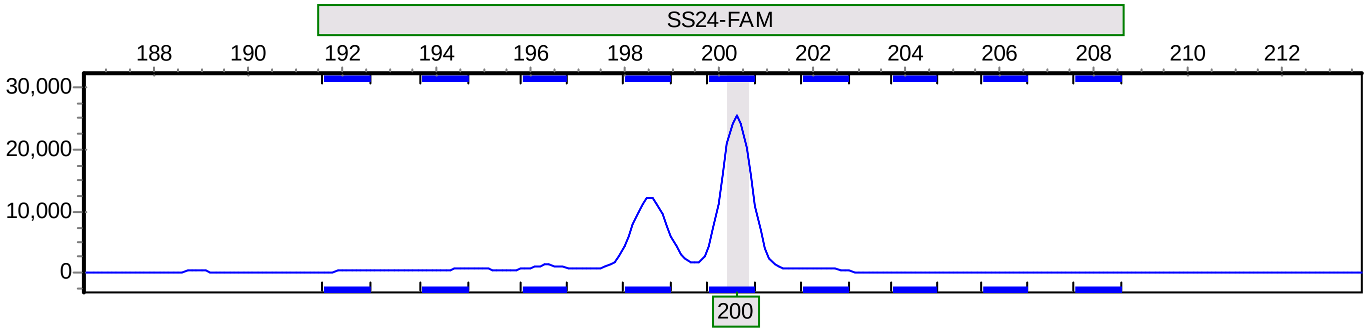

No	Size	Height	Area	Marker	Allele	Difference	Quality	Score	Allele Comments	Sample Comments
1	97.4	22034	158629	SSS18-FAM	98	0.00	Pass	500.0	[<Confirmed>]	
2	115.6	31313	228567	SSS18-FAM	116	0.10	Pass	500.0	[<Confirmed>]	
3	200.4	25355	184349	SS24-FAM	200	0.10	Pass	500.0	[<Confirmed>]	
4	247.6	8283	72143	SS05-FAM	247	0.00	Pass	500.0		
5	323.8	3545	36283	SS32-FAM	324	0.10	Pass	315.3		
6	347.6	1314	13622	SS32-FAM	348	0.10	Pass	78.0		

Sample 97: SSS18_SS24_SS05_SS32_SS30_SS12_SS23_HQZ7_C04.fsa

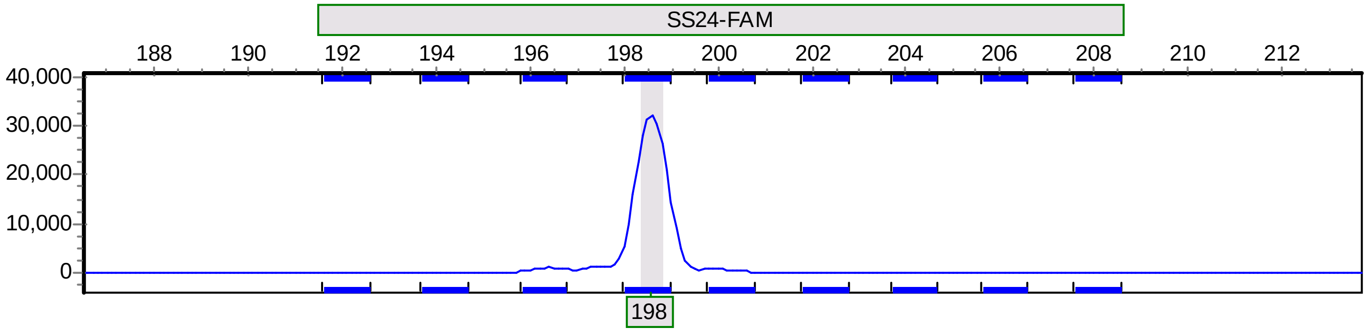

No	Size	Height	Area	Marker	Allele	Difference	Quality	Score	Allele Comments	Sample Comments
1	113.4	31550	213539	SSS18-FAM	114	0.10	Pass	500.0	[<Confirmed>]	
2	198.6	31886	241966	SS24-FAM	198	0.10	Pass	500.0	[<Confirmed>]	
3	245.8	31100	240992	SS05-FAM	245	0.20	Check	500.0		
4	319.8	4877	47042	SS32-FAM	320	0.10	Pass	500.0		
5	326.0	3669	35565	SS32-FAM	326	0.00	Pass	396.4		

Sample 98: SSS18_SS24_SS05_SS32_SS30_SS12_SS23_HQZ9_K12.fsa

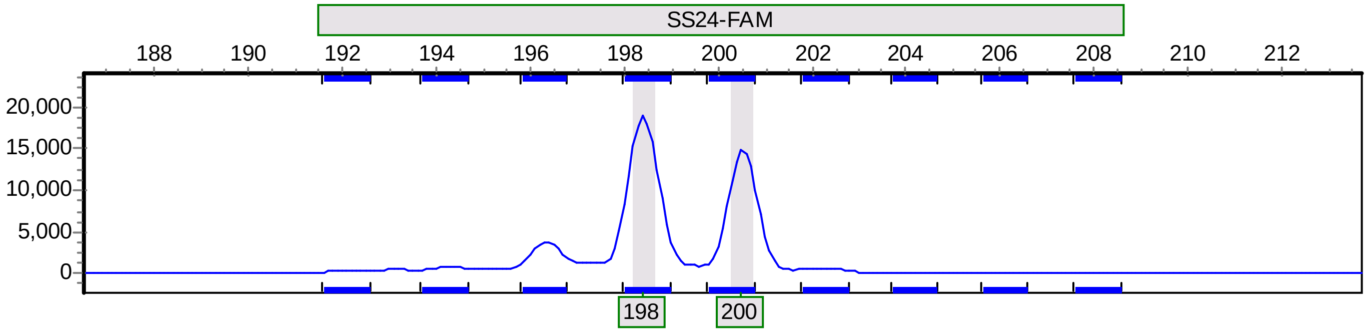

No	Size	Height	Area	Marker	Allele	Difference	Quality	Score	Allele Comments	Sample Comments

1	140.2	11805	94924	SSS18-FAM	140	0.10	Pass	500.0	[<Confirmed>]	
2	142.5	7942	62079	SSS18-FAM	142	0.10	Pass	500.0	[<Confirmed>]	
3	144.7	6179	47303	SSS18-FAM	144	0.20	Undetermined	500.0	[<Deleted>]	
4	198.4	18894	139945	SS24-FAM	198	0.10	Pass	500.0	[<Confirmed>]	
5	200.5	14771	104842	SS24-FAM	200	0.20	Pass	500.0	[<Confirmed>]	
6	253.3	21163	177109	SS05-FAM	253	0.00	Pass	500.0		
7	257.6	10287	83849	SS05-FAM	257	0.10	Pass	500.0		
8	334.3	2641	26318	SS32-FAM	334	0.10	Pass	236.5		
9	349.5	844	8206	SS32-FAM	350	0.10	Pass	46.4		

Sample 99: SSS18_SS24_SS05_SS32_SS30_SS12_SS23_HRS24_I18.fsa

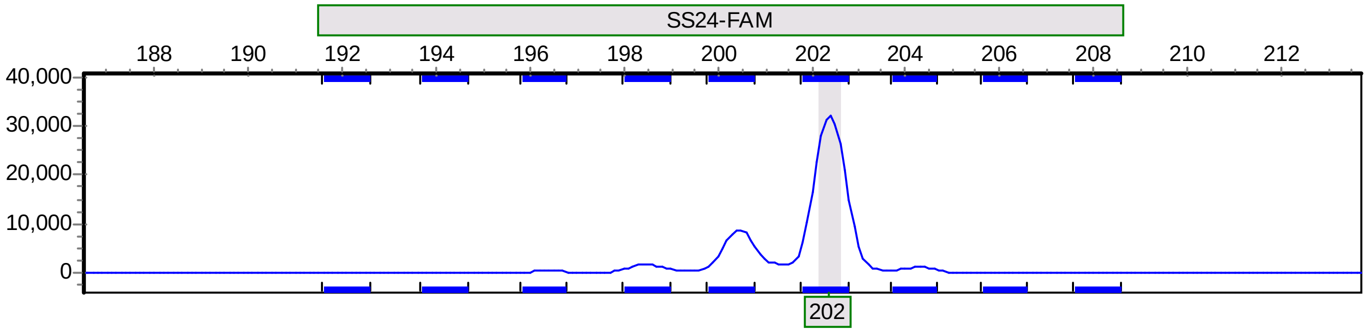

No	Size	Height	Area	Marker	Allele	Difference	Quality	Score	Allele Comments	Sample Comments
1	127.8	23828	164533	SSS18-FAM	128	0.00	Pass	500.0	[<Confirmed>]	
2	202.4	31872	246459	SS24-FAM	202	0.10	Pass	500.0	[<Confirmed>]	
3	257.5	12496	108846	SS05-FAM	257	0.00	Pass	500.0		
4	269.4	9668	83538	SS05-FAM	269	0.00	Pass	500.0		
5	344.0	1786	17915	SS32-FAM	344	0.00	Pass	126.1		
6	351.4	1054	11319	SS32-FAM	352	0.10	Pass	49.3		

Sample 100: SSS18_SS24_SS05_SS32_SS30_SS12_SS23_HRS26_H10.fsa

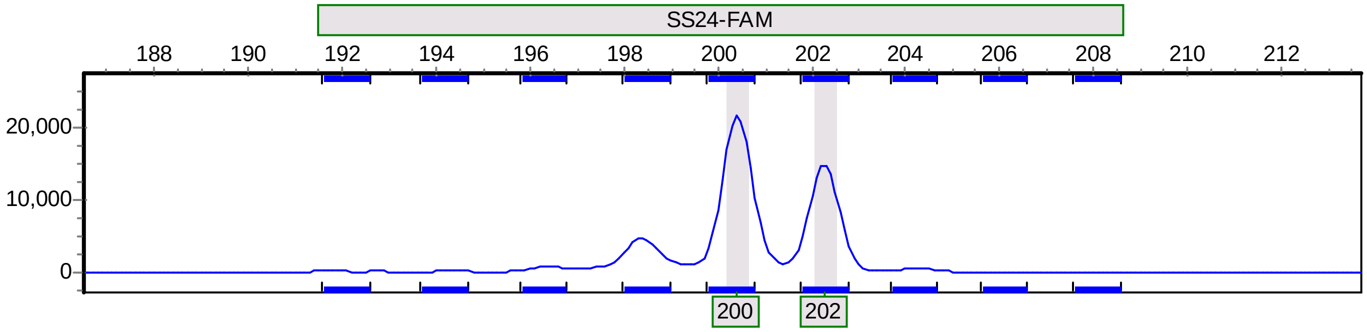

No	Size	Height	Area	Marker	Allele	Difference	Quality	Score	Allele Comments	Sample Comments
1	136.1	11605	83161	SSS18-FAM	136	0.10	Pass	500.0	[<Confirmed>]	
2	138.1	11264	80054	SSS18-FAM	138	0.00	Pass	500.0	[<Confirmed>]	
3	200.4	21605	159434	SS24-FAM	200	0.10	Pass	500.0	[<Confirmed>]	
4	202.3	14878	110212	SS24-FAM	202	0.00	Pass	500.0	[<Confirmed>]	
5	257.5	12098	101543	SS05-FAM	257	0.00	Pass	500.0		
6	269.4	10007	84860	SS05-FAM	269	0.00	Pass	500.0		
7	343.9	1469	15299	SS32-FAM	344	0.10	Pass	79.5		

Sample 101: SSS18_SS24_SS05_SS32_SS30_SS12_SS23_HRS28_D14.fsa

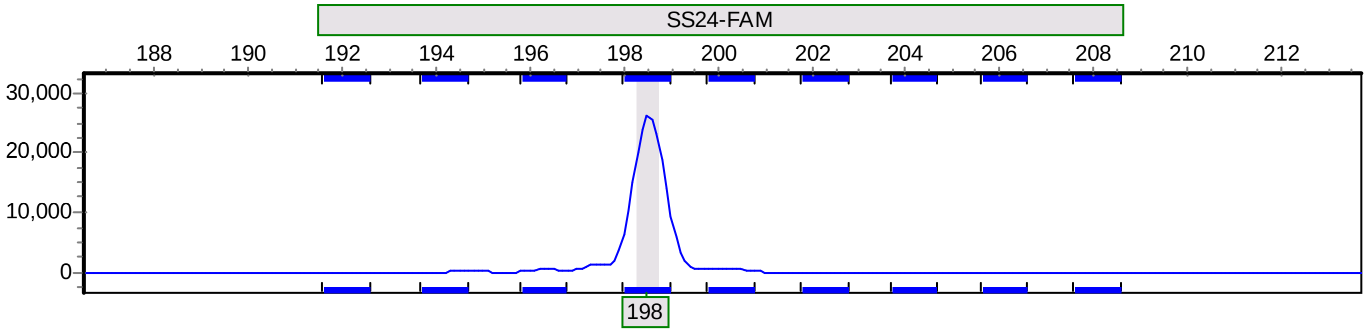

No	Size	Height	Area	Marker	Allele	Difference	Quality	Score	Allele Comments	Sample Comments
1	105.2	24371	164148	SSS18-FAM	106	0.20	Pass	500.0	[<Confirmed>]	
2	113.4	13007	88083	SSS18-FAM	114	0.10	Pass	500.0	[<Confirmed>]	
3	198.5	26033	196887	SS24-FAM	198	0.00	Pass	500.0	[<Confirmed>]	
4	245.5	11365	94000	SS05-FAM	245	0.10	Pass	500.0		
5	247.4	10398	86318	SS05-FAM	247	0.20	Pass	500.0		
6	319.6	3803	38059	SS32-FAM	320	0.10	Pass	395.9		
7	327.9	2658	27918	SS32-FAM	328	0.10	Pass	204.4		

Sample 102: SSS18_SS24_SS05_SS32_SS30_SS12_SS23_HRS29_J06.fsa

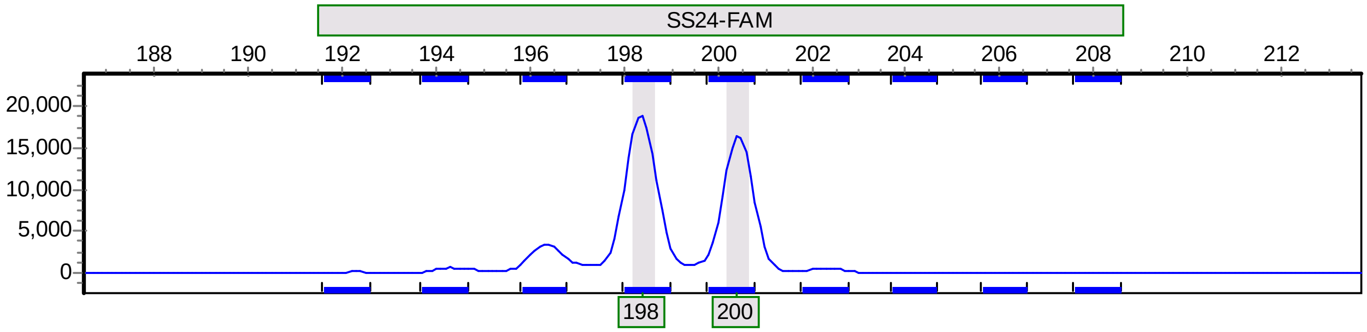

No	Size	Height	Area	Marker	Allele	Difference	Quality	Score	Allele Comments	Sample Comments
1	127.8	19655	132869	SSS18-FAM	128	0.00	Pass	500.0	[<Confirmed>]	
2	198.4	18688	142691	SS24-FAM	198	0.10	Pass	500.0	[<Confirmed>]	
3	200.4	16404	119818	SS24-FAM	200	0.10	Pass	500.0	[<Confirmed>]	
4	257.5	12633	102919	SS05-FAM	257	0.00	Pass	500.0		
5	269.4	10093	83135	SS05-FAM	269	0.00	Pass	500.0		
6	351.4	2123	22458	SS32-FAM	352	0.10	Pass	156.3		

Sample 103: SSS18_SS24_SS05_SS32_SS30_SS12_SS23_HRS30_P06.fsa

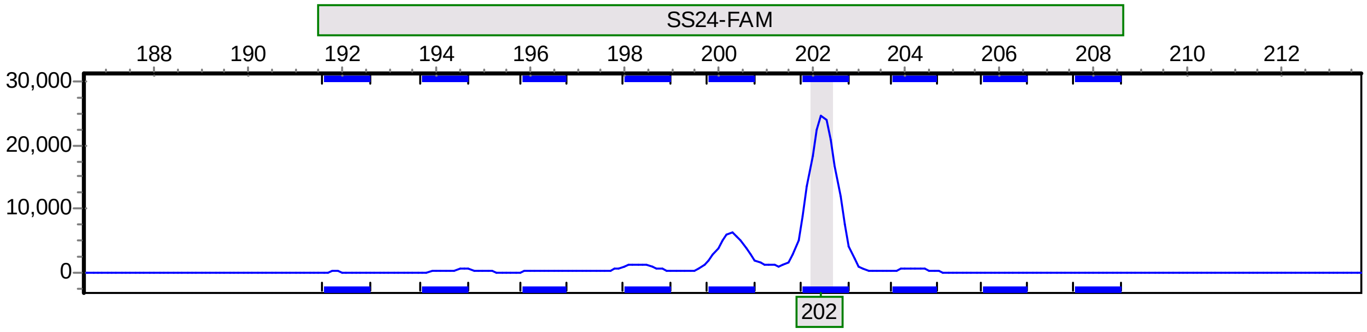

No	Size	Height	Area	Marker	Allele	Difference	Quality	Score	Allele Comments	Sample Comments
1	136.1	13957	94871	SSS18-FAM	136	0.10	Pass	500.0	[<Confirmed>]	
2	138.2	14180	96528	SSS18-FAM	138	0.10	Pass	500.0	[<Confirmed>]	

3	202.2	24584	173855	SS24-FAM	202	0.10	Pass	500.0	[<Confirmed>]	
4	261.5	9203	76416	SS05-FAM	261	0.00	Pass	500.0		
5	269.5	9081	77626	SS05-FAM	269	0.10	Pass	500.0		
6	351.5	2435	25753	SS32-FAM	352	0.00	Pass	178.1		

Sample 104: SSS18_SS24_SS05_SS32_SS30_SS12_SS23_HRS31_J12.fsa

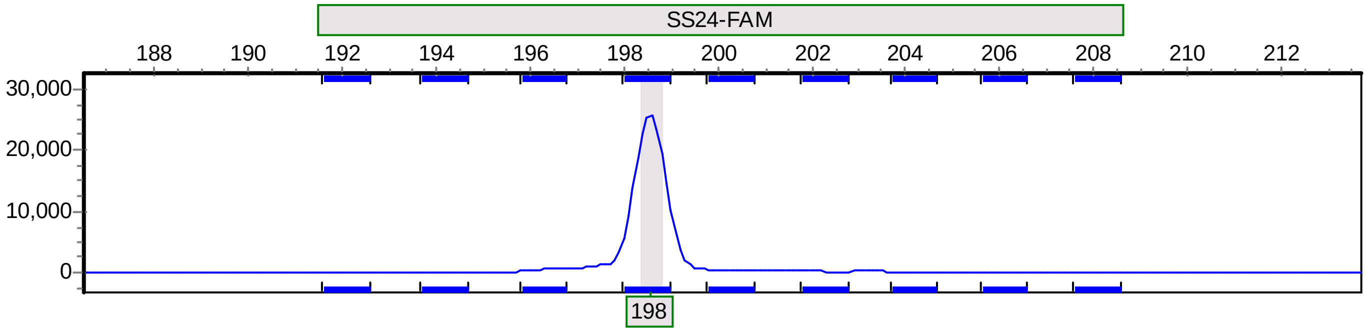

No	Size	Height	Area	Marker	Allele	Difference	Quality	Score	Allele Comments	Sample Comments
1	97.4	23821	169096	SSS18-FAM	98	0.00	Pass	500.0	[<Confirmed>]	
2	113.5	12306	83005	SSS18-FAM	114	0.00	Pass	500.0	[<Confirmed>]	
3	198.6	25550	193756	SS24-FAM	198	0.10	Pass	500.0	[<Confirmed>]	
4	245.6	15995	130871	SS05-FAM	245	0.00	Pass	500.0		
5	247.6	12026	101653	SS05-FAM	247	0.00	Pass	500.0		
6	319.8	4155	42061	SS32-FAM	320	0.10	Pass	421.0		
7	326.0	3607	35738	SS32-FAM	326	0.00	Pass	367.1		

Sample 105: SSS18_SS24_SS05_SS32_SS30_SS12_SS23_HRS33_H08.fsa

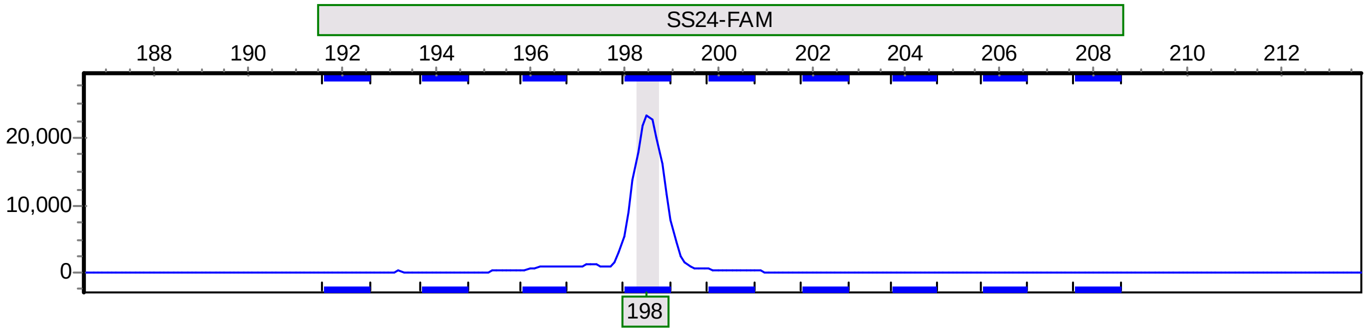

No	Size	Height	Area	Marker	Allele	Difference	Quality	Score	Allele Comments	Sample Comments
1	105.3	24181	158368	SSS18-FAM	106	0.10	Pass	500.0	[<Confirmed>]	
2	129.7	9171	62522	SSS18-FAM	130	0.10	Pass	500.0	[<Confirmed>]	
3	198.5	23152	170936	SS24-FAM	198	0.00	Pass	500.0	[<Confirmed>]	
4	245.5	13503	110929	SS05-FAM	245	0.10	Pass	500.0		
5	247.5	10986	92071	SS05-FAM	247	0.10	Pass	500.0		
6	323.9	4228	41015	SS32-FAM	324	0.00	Pass	455.2		
7	326.0	3027	30697	SS32-FAM	326	0.00	Pass	267.6		

Allele Report

Sample 106: SSS18_SS24_SS05_SS32_SS30_SS12_SS23_HRS34_L10.fsa

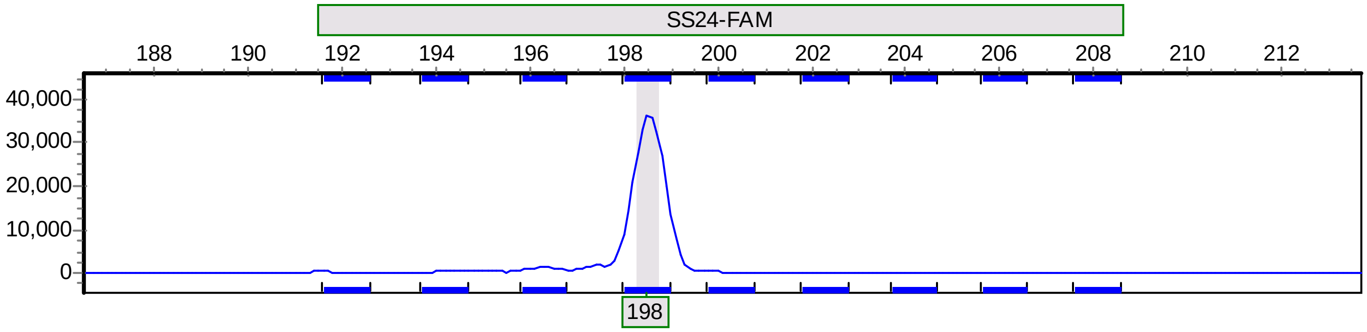

No	Size	Height	Area	Marker	Allele	Difference	Quality	Score	Allele Comments	Sample Comments
1	97.3	34766	299250	SSS18-FAM	98	0.10	Pass	500.0	[<SAT (Repaired)><Confirmed>]	
2	198.5	36035	275845	SS24-FAM	198	0.00	Pass	500.0	[<SAT (Repaired)><Confirmed>]	
3	271.1	12177	103539	SS05-FAM	271	0.10	Pass	500.0		
4	324.0	6019	62081	SS32-FAM	324	0.10	Pass	500.0		
5	326.0	4325	44205	SS32-FAM	326	0.00	Pass	428.8		

Sample 107: SSS18_SS24_SS05_SS32_SS30_SS12_SS23_HRS35_P10.fsa

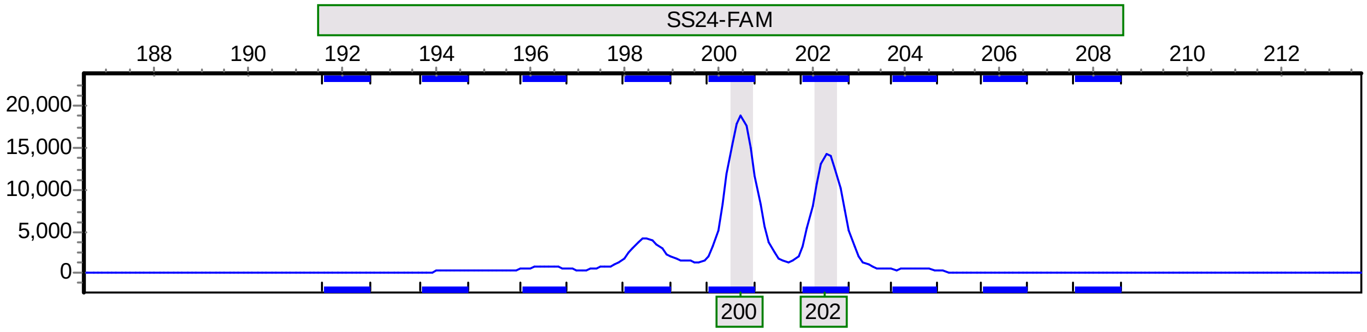

No	Size	Height	Area	Marker	Allele	Difference	Quality	Score	Allele Comments	Sample Comments
1	127.9	19154	130991	SSS18-FAM	128	0.10	Pass	500.0	[<Confirmed>]	
2	200.5	18787	138223	SS24-FAM	200	0.20	Pass	500.0	[<Confirmed>]	
3	202.3	14335	106145	SS24-FAM	202	0.00	Pass	500.0	[<Confirmed>]	
4	257.7	10535	89739	SS05-FAM	257	0.20	Pass	500.0		
5	269.6	8597	73147	SS05-FAM	269	0.20	Pass	500.0		
6	344.0	1407	14334	SS32-FAM	344	0.00	Pass	86.0		

Sample 108: SSS18_SS24_SS05_SS32_SS30_SS12_SS23_HRS37_J14.fsa

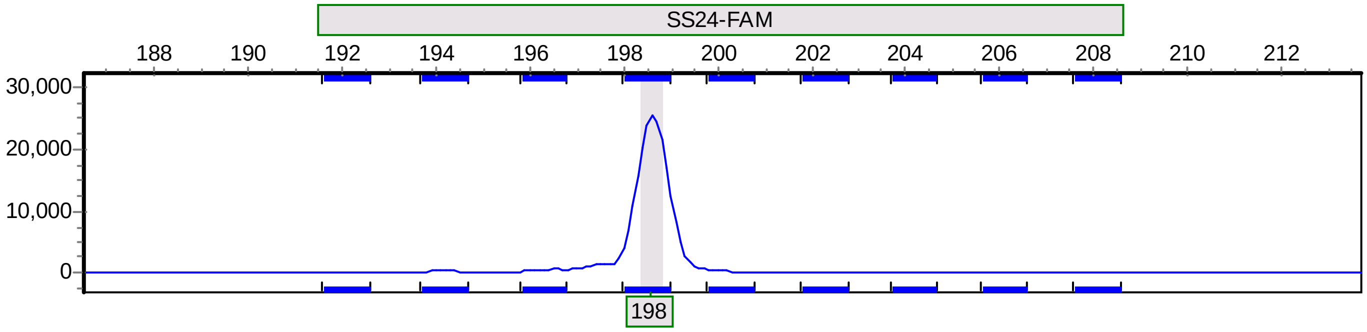

No	Size	Height	Area	Marker	Allele	Difference	Quality	Score	Allele Comments	Sample Comments
1	105.5	31355	241093	SSS18-FAM	106	0.10	Pass	500.0	[<Confirmed>]	
2	198.6	25307	189524	SS24-FAM	198	0.10	Pass	500.0	[<Confirmed>]	
3	245.5	14517	119911	SS05-FAM	245	0.10	Pass	500.0		
4	249.0	11916	99331	SS05-FAM	249	0.20	Pass	500.0		

Allele Report

5	319.7	4509	45647	SS32-FAM	320	0.00	Pass	464.9		
6	325.9	3081	31515	SS32-FAM	326	0.10	Pass	256.6		

Sample 109: SSS18_SS24_SS05_SS32_SS30_SS12_SS23_HRS38_J08.fsa

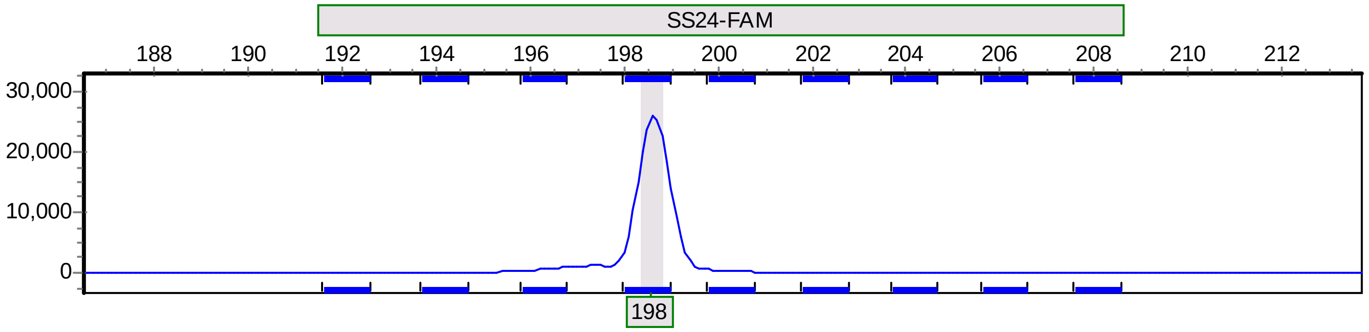

No	Size	Height	Area	Marker	Allele	Difference	Quality	Score	Allele Comments	Sample Comments
1	113.5	31047	217952	SSS18-FAM	114	0.00	Pass	500.0	[<Confirmed>]	
2	198.6	25895	195521	SS24-FAM	198	0.10	Pass	500.0	[<Confirmed>]	
3	245.7	26735	223911	SS05-FAM	245	0.10	Pass	500.0		
4	325.8	4883	52681	SS32-FAM	326	0.20	Pass	460.2		

Sample 110: SSS18_SS24_SS05_SS32_SS30_SS12_SS23_HRS39_N08.fsa

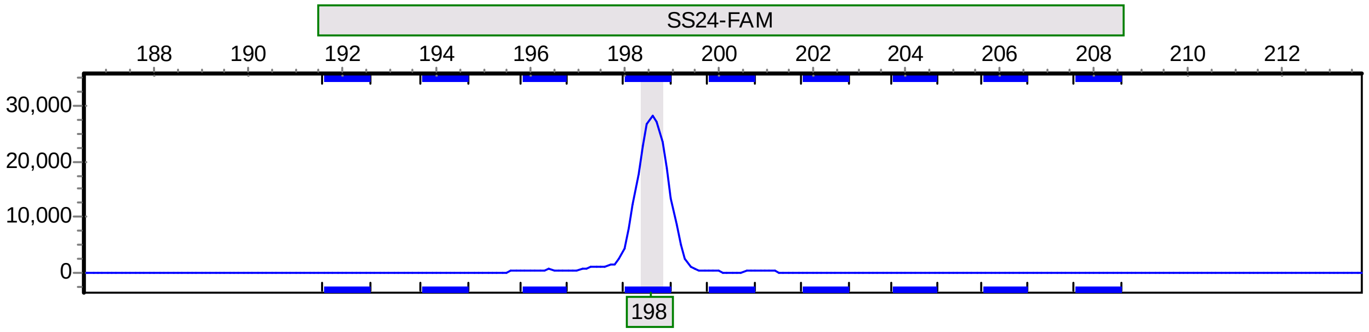

No	Size	Height	Area	Marker	Allele	Difference	Quality	Score	Allele Comments	Sample Comments
1	103.4	31936	223452	SSS18-FAM	104	0.00	Pass	500.0	[<Confirmed>]	
2	115.6	19227	128048	SSS18-FAM	116	0.10	Pass	500.0	[<Confirmed>]	
3	198.6	27936	208671	SS24-FAM	198	0.10	Pass	500.0	[<Confirmed>]	
4	245.7	14678	126051	SS05-FAM	245	0.10	Pass	500.0		
5	249.2	13259	110189	SS05-FAM	249	0.00	Pass	500.0		
6	328.1	3540	37535	SS32-FAM	328	0.10	Pass	302.8		
7	332.1	2453	25462	SS32-FAM	332	0.00	Pass	188.4		

Sample 111: SSS18_SS24_SS05_SS32_SS30_SS12_SS23_HRS40_A08.fsa

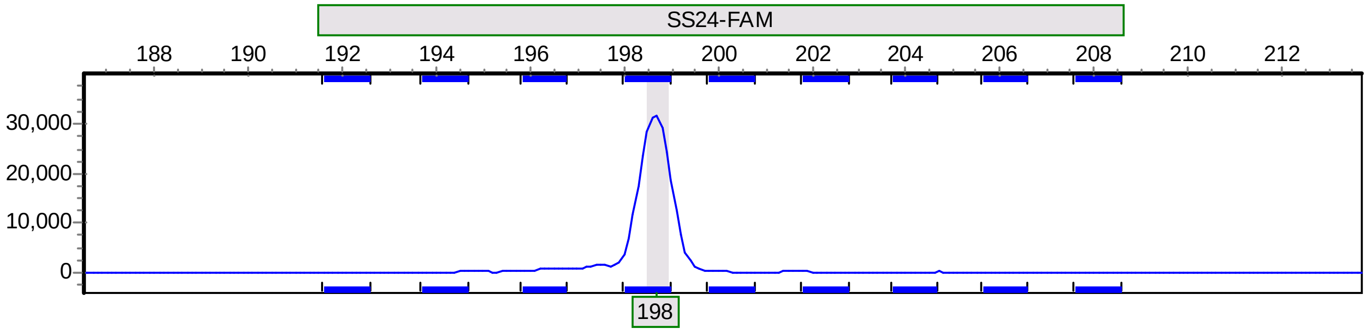

No	Size	Height	Area	Marker	Allele	Difference	Quality	Score	Allele Comments	Sample Comments
1	105.5	29404	192381	SSS18-FAM	106	0.10	Pass	500.0	[<Confirmed>]	
2	115.5	18093	121509	SSS18-FAM	116	0.00	Pass	500.0	[<Confirmed>]	

3	198.7	31346	239635	SS24-FAM	198	0.20	Pass	500.0	[<Confirmed>]	
4	249.2	19482	162178	SS05-FAM	249	0.00	Pass	500.0		
5	326.1	5642	57093	SS32-FAM	326	0.10	Pass	500.0		
6	328.2	4201	41093	SS32-FAM	328	0.20	Pass	434.7		

Sample 112: SSS18_SS24_SS05_SS32_SS30_SS12_SS23_HRS41_K08.fsa

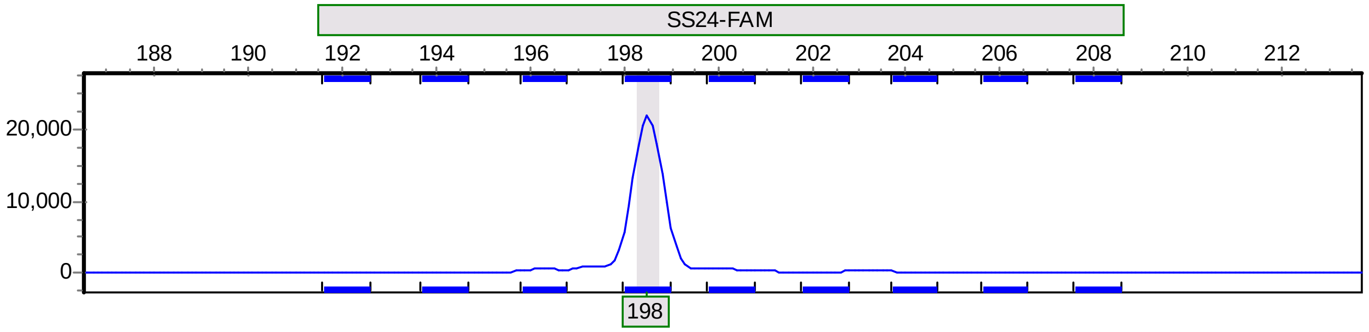

No	Size	Height	Area	Marker	Allele	Difference	Quality	Score	Allele Comments	Sample Comments
1	105.3	31886	224310	SSS18-FAM	106	0.10	Pass	500.0	[<Confirmed>]	
2	113.4	19296	126550	SSS18-FAM	114	0.10	Pass	500.0	[<Confirmed>]	
3	198.5	21926	158278	SS24-FAM	198	0.00	Pass	500.0	[<Confirmed>]	
4	245.6	12120	100896	SS05-FAM	245	0.00	Pass	500.0		
5	249.0	11404	93108	SS05-FAM	249	0.20	Pass	500.0		
6	319.6	4243	43890	SS32-FAM	320	0.10	Pass	411.8		
7	332.0	2089	21676	SS32-FAM	332	0.10	Pass	152.3		

Sample 113: SSS18_SS24_SS05_SS32_SS30_SS12_SS23_HRS42_I08.fsa

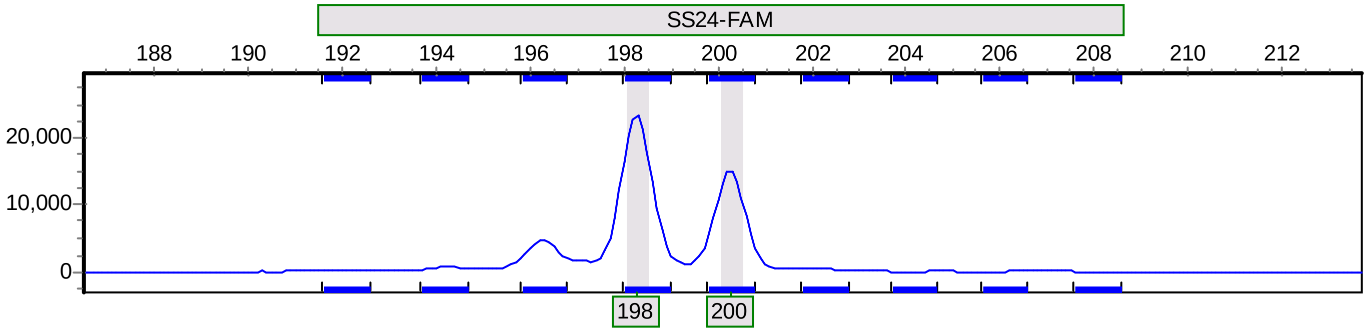

No	Size	Height	Area	Marker	Allele	Difference	Quality	Score	Allele Comments	Sample Comments
1	121.8	29687	207667	SSS18-FAM	122	0.10	Pass	500.0	[<Confirmed>]	
2	123.8	18957	131813	SSS18-FAM	124	0.10	Pass	500.0	[<Confirmed>]	
3	198.3	23104	174526	SS24-FAM	198	0.20	Pass	500.0	[<Confirmed>]	
4	200.3	14819	111411	SS24-FAM	200	0.00	Pass	500.0	[<Confirmed>]	
5	259.3	16864	146365	SS05-FAM	259	0.10	Pass	500.0		
6	334.2	2596	27515	SS32-FAM	334	0.00	Pass	195.3		

Sample 114:

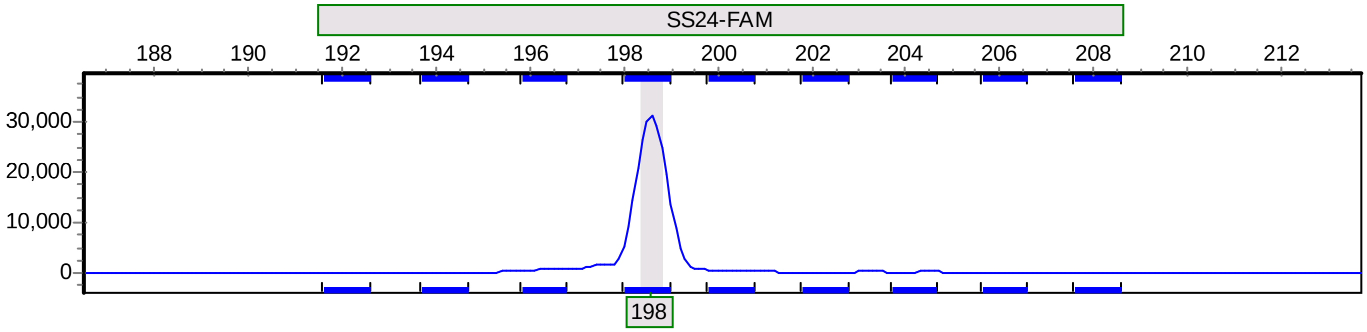

No	Size	Height	Area	Marker	Allele	Difference	Quality	Score	Allele Comments	Sample Comments
1	97.4	31280	232365	SSS18-FAM	98	0.00	Pass	500.0	[<Confirmed>]	
2	115.5	18163	121498	SSS18-FAM	116	0.00	Pass	500.0	[<Confirmed>]	
3	198.6	31022	230237	SS24-FAM	198	0.10	Pass	500.0	[<Confirmed>]	
4	271.2	11388	95890	SS05-FAM	271	0.00	Pass	500.0		
5	319.8	4804	48090	SS32-FAM	320	0.10	Pass	500.0		
6	326.0	3536	35749	SS32-FAM	326	0.00	Pass	340.8		

Sample 115: SSS18_SS24_SS05_SS32_SS30_SS12_SS23_HTHL13_H02.fsa

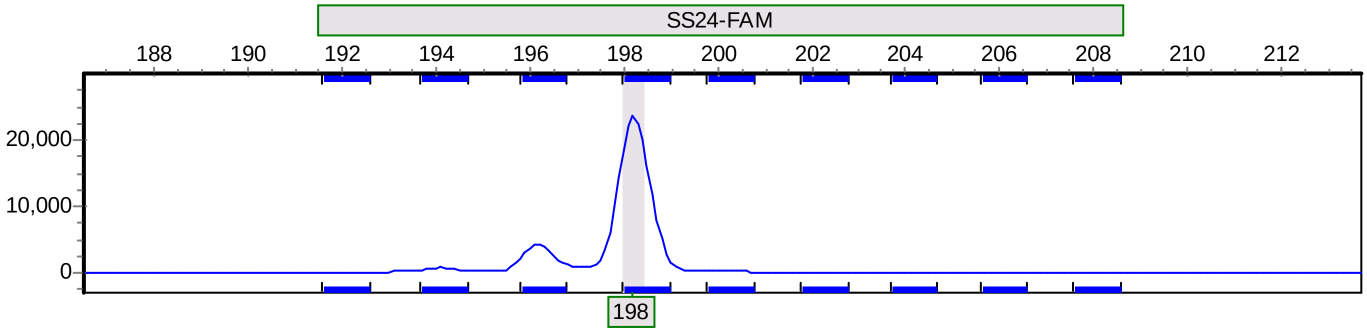

No	Size	Height	Area	Marker	Allele	Difference	Quality	Score	Allele Comments	Sample Comments
1	105.4	10751	69021	SSS18-FAM	106	0.00	Pass	500.0	[<Confirmed>]	
2	131.8	12119	81690	SSS18-FAM	132	0.10	Pass	500.0	[<Confirmed>]	
3	198.2	23499	176346	SS24-FAM	198	0.30	Pass	500.0	[<Confirmed>]	
4	261.6	13120	109125	SS05-FAM	261	0.10	Pass	500.0		
5	326.0	2641	26351	SS32-FAM	326	0.00	Pass	232.8		
6	338.3	1662	17299	SS32-FAM	338	0.00	Pass	106.6		

Sample 116: SSS18_SS24_SS05_SS32_SS30_SS12_SS23_HTHL14_F16.fsa

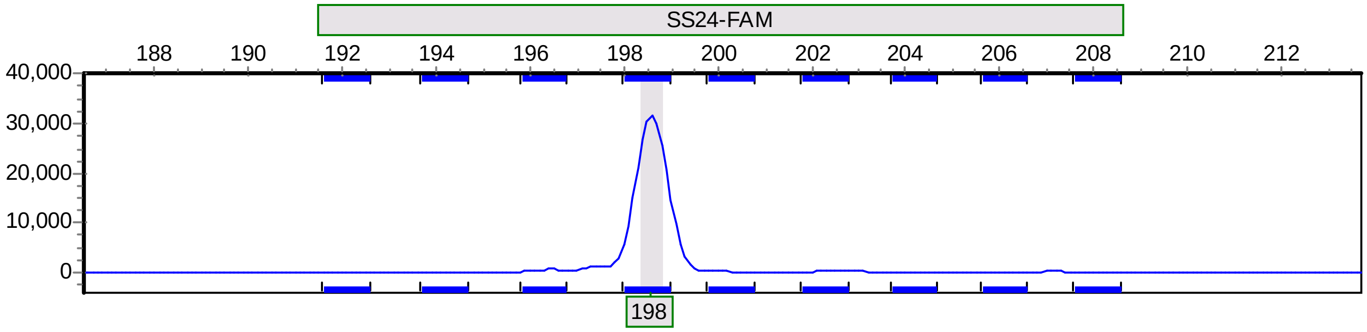

No	Size	Height	Area	Marker	Allele	Difference	Quality	Score	Allele Comments	Sample Comments
1	113.4	25268	172611	SSS18-FAM	114	0.10	Pass	500.0	[<Confirmed>]	
2	198.6	31378	238683	SS24-FAM	198	0.10	Pass	500.0	[<Confirmed>]	
3	245.5	10912	92294	SS05-FAM	245	0.10	Pass	500.0		

4	273.0	8276	74687	SS05-FAM	273	0.20	Pass	500.0
5	319.6	4641	48944	SS32-FAM	320	0.10	Pass	452.2
6	325.9	3526	36981	SS32-FAM	326	0.10	Pass	317.7

Sample 117: SSS18_SS24_SS05_SS32_SS30_SS12_SS23_HTHL15_F02.fsa

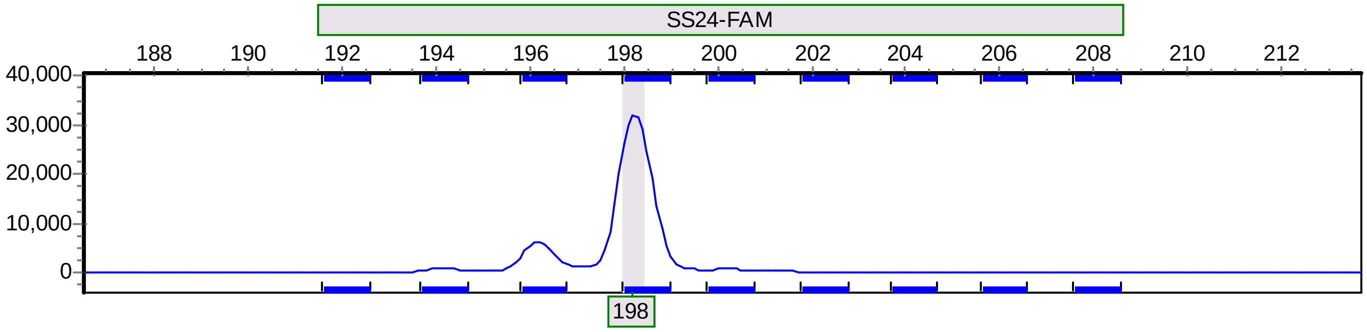

No	Size	Height	Area	Marker	Allele	Difference	Quality	Score	Allele Comments	Sample Comments
1	121.6	8744	55888	SSS18-FAM	122	0.10	Undetermined	500.0	[<Deleted>]	
2	136.1	14845	98575	SSS18-FAM	136	0.10	Pass	500.0	[<Confirmed>]	
3	138.1	15284	103313	SSS18-FAM	138	0.00	Pass	500.0	[<Confirmed>]	
4	198.2	31825	257829	SS24-FAM	198	0.30	Pass	500.0	[<Confirmed>]	
5	267.7	22123	178894	SS05-FAM	267	0.20	Pass	500.0		
6	326.0	3729	36936	SS32-FAM	326	0.00	Pass	398.8		

Sample 118: SSS18_SS24_SS05_SS32_SS30_SS12_SS23_HTHL1_E14.fsa

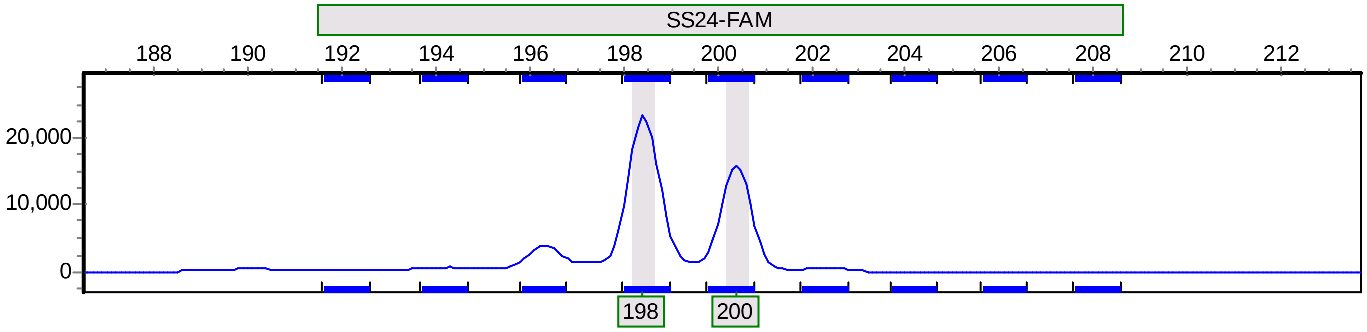

No	Size	Height	Area	Marker	Allele	Difference	Quality	Score	Allele Comments	Sample Comments
1	138.1	11233	80142	SSS18-FAM	138	0.00	Pass	500.0	[<Confirmed>]	
2	142.6	7498	59700	SSS18-FAM	142	0.00	Pass	500.0	[<Confirmed>]	
3	144.9	6899	53997	SSS18-FAM	144	0.00	Undetermined	500.0	[<Deleted>]	
4	198.4	23174	177225	SS24-FAM	198	0.10	Pass	500.0	[<Confirmed>]	
5	200.4	15874	116875	SS24-FAM	200	0.10	Pass	500.0	[<Confirmed>]	
6	263.4	17448	151568	SS05-FAM	263	0.00	Pass	500.0		
7	344.0	2283	22811	SS32-FAM	344	0.00	Pass	188.6		
8	345.8	1353	13293	SS32-FAM	346	0.00	Pass	79.7		

Allele Report

Sample 119: SSS18_SS24_SS05_SS32_SS30_SS12_SS23_HTHL3_D06.fsa

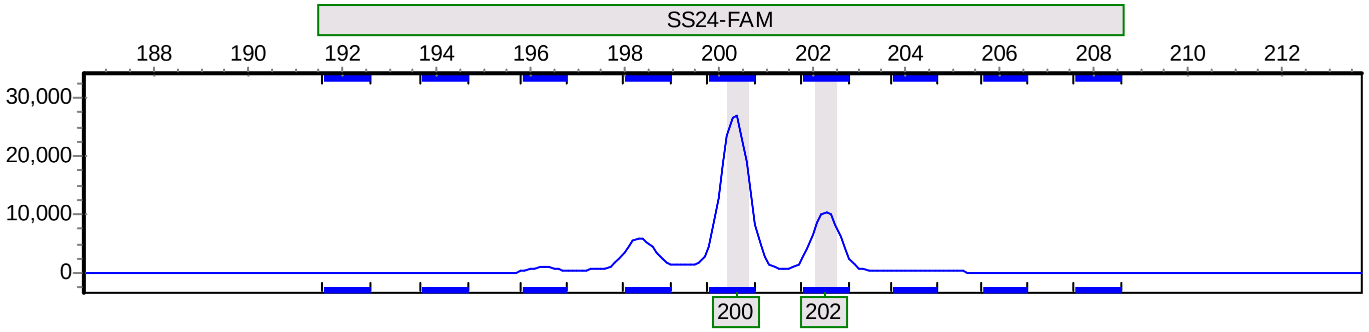

No	Size	Height	Area	Marker	Allele	Difference	Quality	Score	Allele Comments	Sample Comments
1	123.8	24779	162954	SSS18-FAM	124	0.10	Pass	500.0	[<Confirmed>]	
2	127.8	16090	107494	SSS18-FAM	128	0.00	Pass	500.0	[<Confirmed>]	
3	200.4	26795	184295	SS24-FAM	200	0.10	Pass	500.0	[<Confirmed>]	
4	202.3	10526	73695	SS24-FAM	202	0.00	Pass	500.0	[<Confirmed>]	
5	253.3	27038	213550	SS05-FAM	253	0.00	Pass	500.0		
6	330.1	3081	32865	SS32-FAM	330	0.00	Pass	162.8		

Sample 120: SSS18_SS24_SS05_SS32_SS30_SS12_SS23_HTHL4_O16.fsa

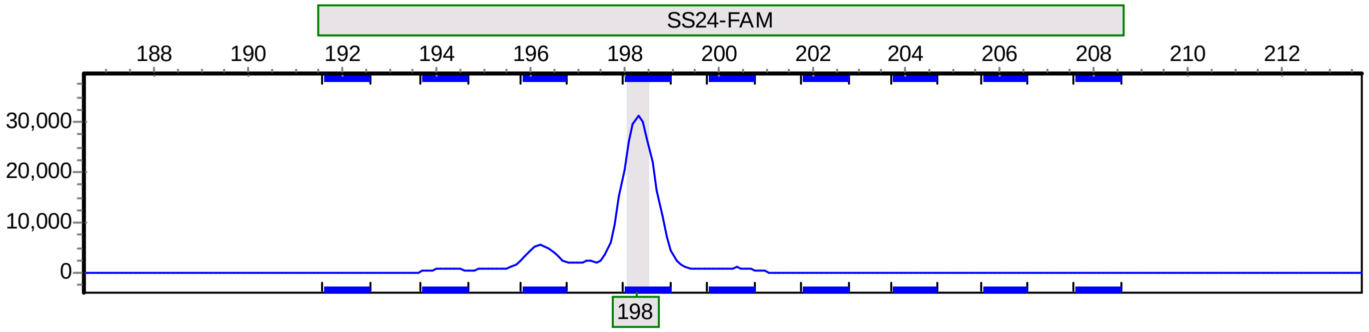

No	Size	Height	Area	Marker	Allele	Difference	Quality	Score	Allele Comments	Sample Comments
1	119.5	28866	195606	SSS18-FAM	120	0.10	Pass	500.0	[<Confirmed>]	
2	198.3	30857	246342	SS24-FAM	198	0.20	Pass	500.0	[<Confirmed>]	
3	249.4	25621	207422	SS05-FAM	249	0.20	Pass	500.0		
4	325.8	3235	33793	SS32-FAM	326	0.20	Pass	278.8		
5	338.1	1624	17187	SS32-FAM	338	0.20	Pass	97.8		

Sample 121: SSS18_SS24_SS05_SS32_SS30_SS12_SS23_HTHL5_M14.fsa

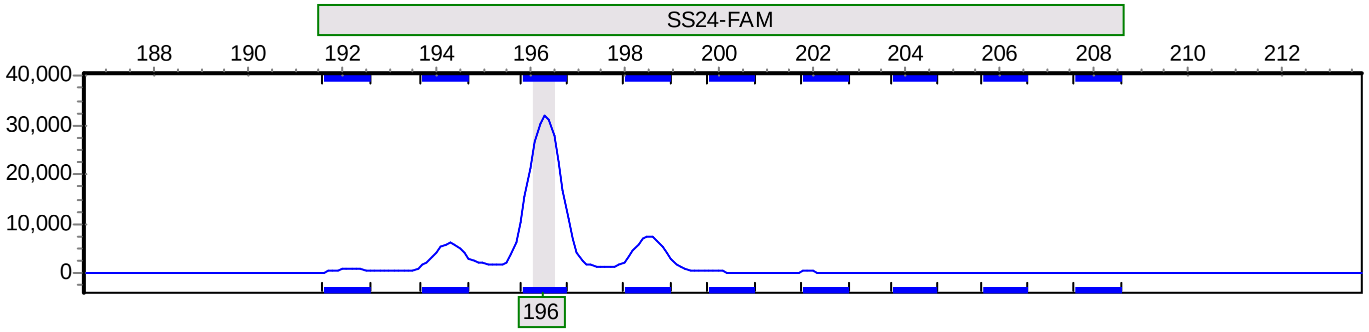

No	Size	Height	Area	Marker	Allele	Difference	Quality	Score	Allele Comments	Sample Comments
1	105.3	21015	139808	SSS18-FAM	106	0.10	Pass	500.0	[<Confirmed>]	
2	119.6	30451	211221	SSS18-FAM	120	0.00	Pass	500.0	[<Confirmed>]	
3	196.3	31709	252939	SS24-FAM	196	0.00	Pass	500.0	[<Confirmed>]	
4	247.6	9379	83268	SS05-FAM	247	0.00	Pass	500.0		

5	330.2	2905	30880	SS32-FAM	330	0.10	Pass	225.4		
6	344.0	1701	17264	SS32-FAM	344	0.00	Pass	113.7		

Sample 122: SSS18_SS24_SS05_SS32_SS30_SS12_SS23_HTHL6_P04.fsa

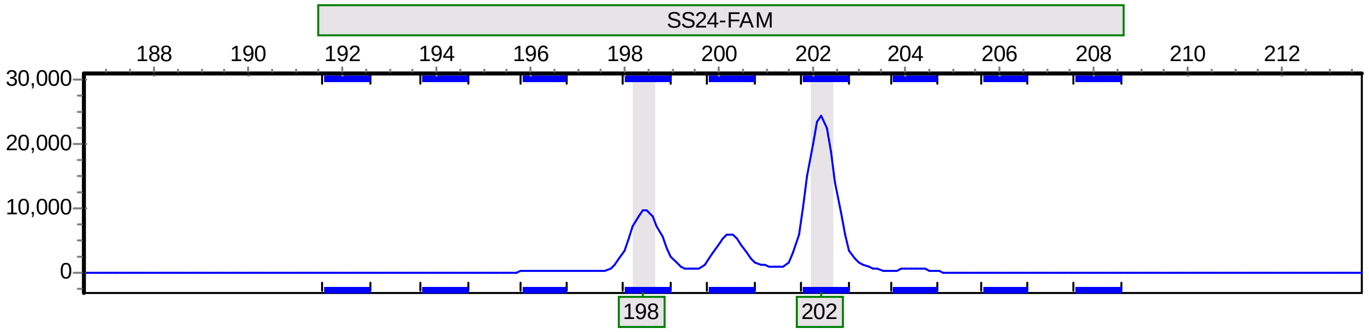

No	Size	Height	Area	Marker	Allele	Difference	Quality	Score	Allele Comments	Sample Comments
1	117.7	29926	191253	SSS18-FAM	118	0.10	Pass	500.0	[<Confirmed>]	
2	198.4	9755	74804	SS24-FAM	198	0.10	Pass	500.0	[<Confirmed>]	
3	202.2	24282	168170	SS24-FAM	202	0.10	Pass	500.0	[<Confirmed>]	
4	253.6	30817	247306	SS05-FAM	253	0.30	Check	500.0		
5	319.9	4397	44658	SS32-FAM	320	0.20	Pass	442.1		
6	338.4	2218	22332	SS32-FAM	338	0.10	Pass	169.2		

Sample 123: SSS18_SS24_SS05_SS32_SS30_SS12_SS23_HTHL8_A04.fsa

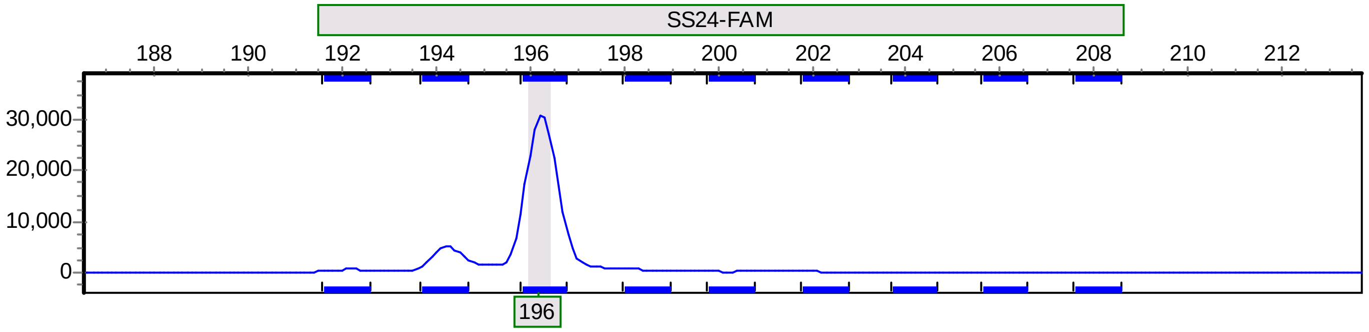

No	Size	Height	Area	Marker	Allele	Difference	Quality	Score	Allele Comments	Sample Comments
1	147.1	9670	72602	SSS18-FAM	148	0.00	Pass	500.0	[<Confirmed>]	
2	149.3	7837	58505	SSS18-FAM	150	0.10	Pass	500.0	[<Confirmed>]	
3	196.2	30533	230658	SS24-FAM	196	0.10	Pass	500.0	[<Confirmed>]	
4	247.5	16098	137219	SS05-FAM	247	0.10	Pass	500.0		
5	255.6	16323	139924	SS05-FAM	255	0.10	Pass	500.0		
6	340.2	1958	19745	SS32-FAM	340	0.00	Pass	146.3		
7	353.3	914	9916	SS32-FAM	354	0.30	Pass	36.7		

Sample 124: SSS18_SS24_SS05_SS32_SS30_SS12_SS23_HTHL9_O10.fsa

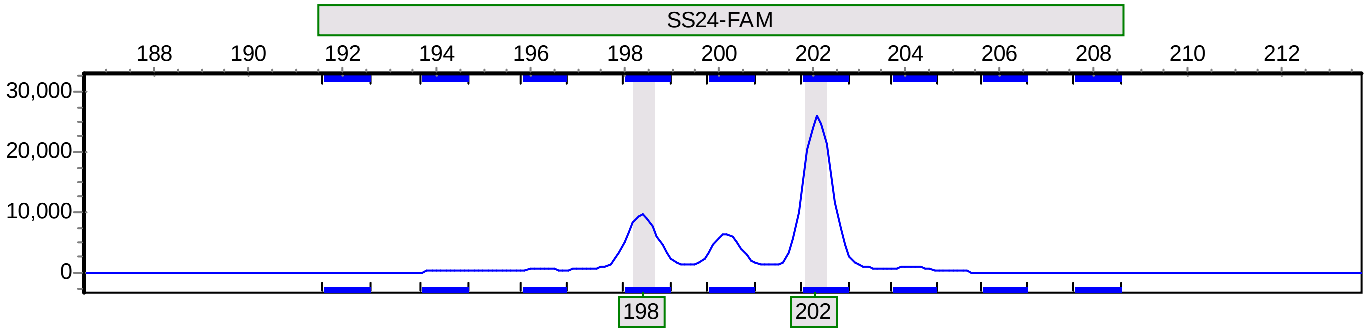

No	Size	Height	Area	Marker	Allele	Difference	Quality	Score	Allele Comments	Sample Comments

1	117.7	32004	236657	SSS18-FAM	118	0.10	Pass	500.0	[<SAT (Repaired)><Confirmed>]
2	198.4	9601	75431	SS24-FAM	198	0.10	Pass	500.0	[<Confirmed>]
3	202.1	25950	183102	SS24-FAM	202	0.20	Pass	500.0	[<Confirmed>]
4	253.3	35570	297280	SS05-FAM	253	0.00	Check	500.0	[<SAT (Repaired)>]
5	327.9	3136	31748	SS32-FAM	328	0.10	Pass	275.3	
6	343.9	1674	16828	SS32-FAM	344	0.10	Pass	110.7	
