# Supplementary material for: Genetic diversity of Schima superba based on physiological traits and SSR markers
Source: PLoS One. 2026 Apr 10;21(4):e0344465. doi: 10.1371/journal.pone.0344465 (PMC13068225; doi:10.1371/journal.pone.0344465)
Supplement: S1 File — (ZIP) [file pone.0344465.s003.zip › SS36.pdf]

Allele Report

Project Comments:

Sample 1: SS08_SS10_SSS42_SS16_SS27_SS36_SS22_HBB10_E05.fsa

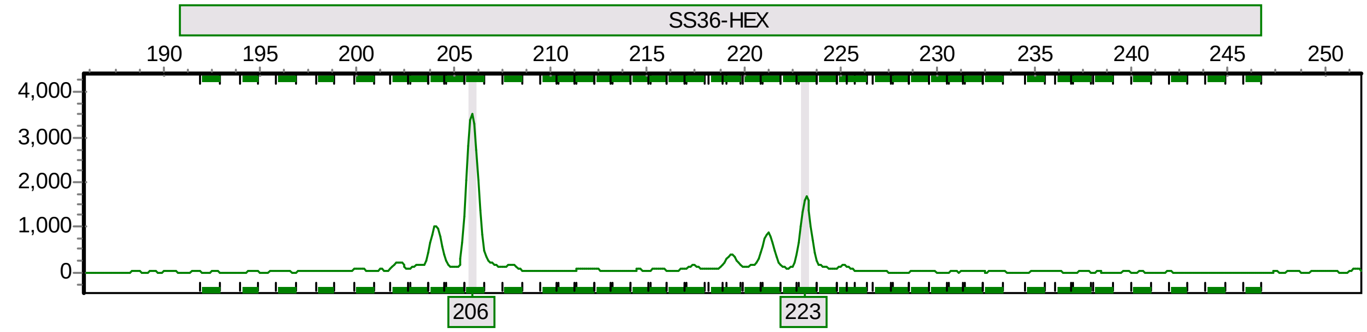

No	Size	Height	Area	Marker	Allele	Difference	Quality	Score	Allele Comments	Sample Comments
1	114.2	9511	63631	SS27-HEX	114	0.00	Pass	500.0	[<Confirmed>]	
2	118.2	7312	48831	SS27-HEX	118	0.40	Pass	500.0	[<Confirmed>]	
3	206.0	3533	23476	SS36-HEX	206	0.10	Pass	500.0	[<Confirmed>]	
4	223.2	1684	11714	SS36-HEX	223	0.10	Pass	260.2	[<Confirmed>]	
5	299.9	4393	38079	SS22-HEX	300	0.10	Pass	500.0		
6	304.1	2984	25560	SS22-HEX	304	0.00	Pass	373.1		

Sample 2: SS08_SS10_SSS42_SS16_SS27_SS36_SS22_HBB12-2_D11.fsa

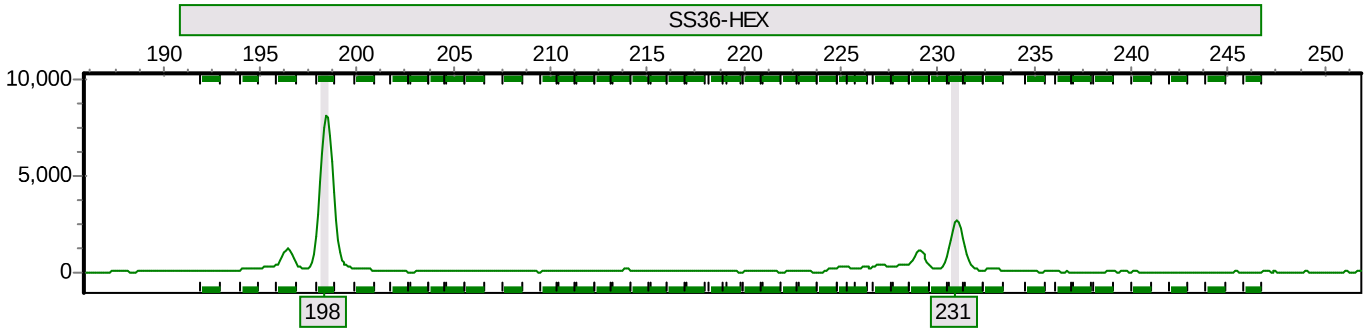

No	Size	Height	Area	Marker	Allele	Difference	Quality	Score	Allele Comments	Sample Comments
1	116.2	11543	77718	SS27-HEX	116	0.00	Pass	500.0	[<Confirmed>]	
2	119.2	3963	24479	SS27-HEX	118	0.60	Undetermined	500.0	[<Deleted>]	
3	120.2	7945	56226	SS27-HEX	120	0.60	Pass	500.0	[<Confirmed>]	
4	198.4	8068	59068	SS36-HEX	198	0.00	Pass	500.0	[<Confirmed>]	
5	231.0	2692	20189	SS36-HEX	231	0.00	Pass	441.1	[<Confirmed>]	
6	300.1	5378	50052	SS22-HEX	300	0.10	Pass	500.0		
7	310.5	5028	46879	SS22-HEX	310	0.20	Pass	500.0		

Sample 3: SS08_SS10_SSS42_SS16_SS27_SS36_SS22_HBB13_C17.fsa

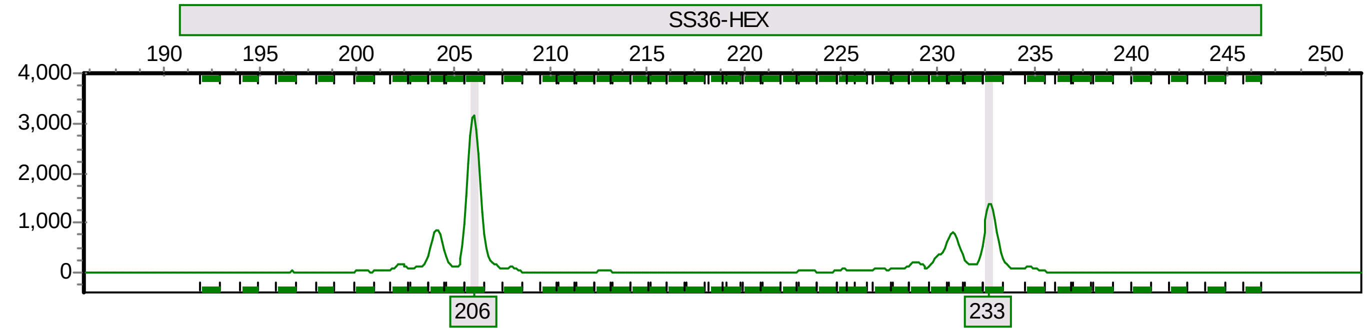

No	Size	Height	Area	Marker	Allele	Difference	Quality	Score	Allele Comments	Sample Comments
1	114.1	10018	68718	SS27-HEX	114	0.10	Pass	500.0	[<Confirmed>]	

2	116.1	6925	48342	SS27-HEX	116	0.10	Pass	500.0	[<Confirmed>]	
3	206.1	3149	22961	SS36-HEX	206	0.00	Pass	500.0	[<Confirmed>]	
4	232.7	1383	11168	SS36-HEX	233	0.20	Pass	153.7	[<Confirmed>]	
5	304.0	4060	38340	SS22-HEX	304	0.10	Pass	454.8		
6	310.2	1806	17913	SS22-HEX	310	0.10	Pass	137.9		

Sample 4: SS08_SS10_SSS42_SS16_SS27_SS36_SS22_HBB14_G03.fsa

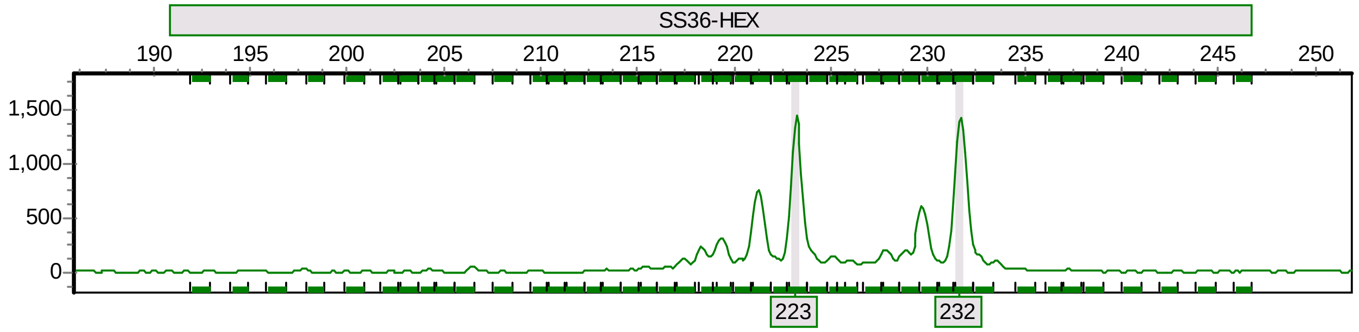

No	Size	Height	Area	Marker	Allele	Difference	Quality	Score	Allele Comments	Sample Comments
1	116.0	10631	71476	SS27-HEX	116	0.20	Pass	500.0	[<Confirmed>]	
2	119.1	10053	67374	SS27-HEX	118	0.50	Pass	500.0	[<Confirmed>]	
3	223.2	1434	9921	SS36-HEX	223	0.10	Pass	204.6	[<Confirmed>]	
4	231.7	1429	10321	SS36-HEX	232	0.20	Pass	193.5	[<Confirmed>]	
5	297.9	2998	24120	SS22-HEX	298	0.10	Pass	424.4		
6	303.9	3308	29497	SS22-HEX	304	0.20	Pass	418.7		

Sample 5: SS08_SS10_SSS42_SS16_SS27_SS36_SS22_HBB15_B07.fsa

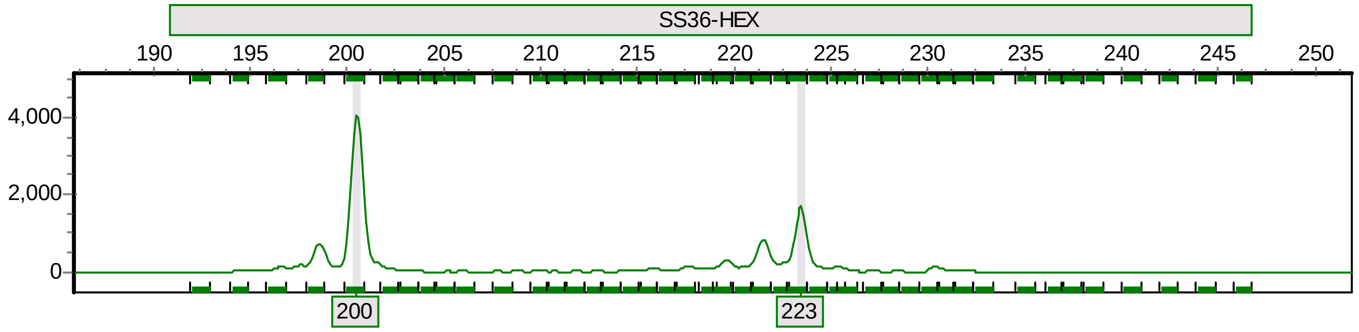

No	Size	Height	Area	Marker	Allele	Difference	Quality	Score	Allele Comments	Sample Comments
1	116.4	9148	59196	SS27-HEX	116	0.20	Pass	500.0	[<Confirmed>]	
2	118.4	3539	24289	SS27-HEX	118	0.20	Pass	489.6	[<Deleted>]	
3	119.3	6009	40634	SS27-HEX	118	1.00	Pass	500.0	[<Confirmed><Edited>]	
4	200.5	4009	28001	SS36-HEX	200	0.10	Pass	500.0	[<Confirmed>]	
5	223.5	1680	12994	SS36-HEX	223	0.20	Pass	221.7	[<Confirmed>]	
6	294.2	6378	54843	SS22-HEX	294	0.00	Pass	500.0		
7	296.1	4377	38559	SS22-HEX	296	0.00	Pass	500.0		

Sample 6: SS08_SS10_SSS42_SS16_SS27_SS36_SS22_HBB16_G07.fsa

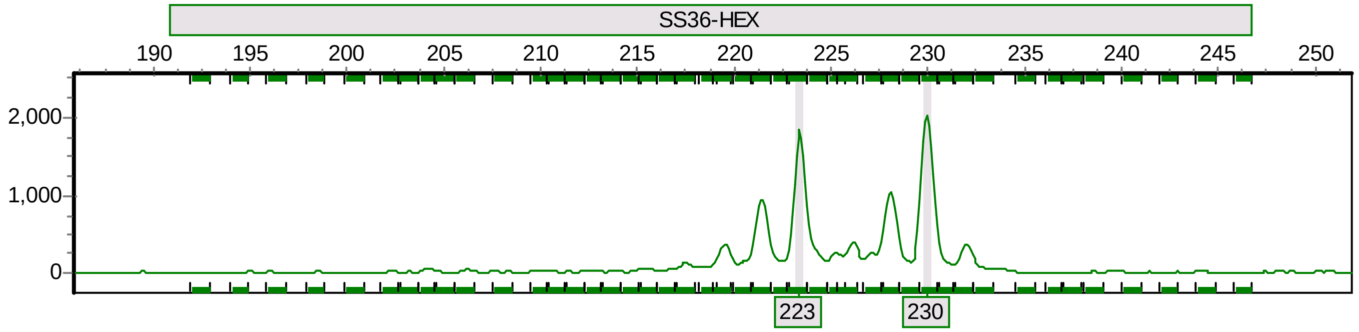

No	Size	Height	Area	Marker	Allele	Difference	Quality	Score	Allele Comments	Sample Comments
1	119.0	19938	131009	SS27-HEX	118	0.40	Pass	500.0	[<Confirmed>]	
2	223.4	1855	13489	SS36-HEX	223	0.10	Pass	267.2	[<Confirmed>]	
3	230.0	2026	15012	SS36-HEX	230	0.10	Pass	299.5	[<Confirmed>]	
4	302.1	3397	31525	SS22-HEX	302	0.00	Pass	395.9		
5	306.3	2442	22549	SS22-HEX	306	0.10	Pass	239.5		

Sample 7: SS08_SS10_SSS42_SS16_SS27_SS36_SS22_HBB17_K15.fsa

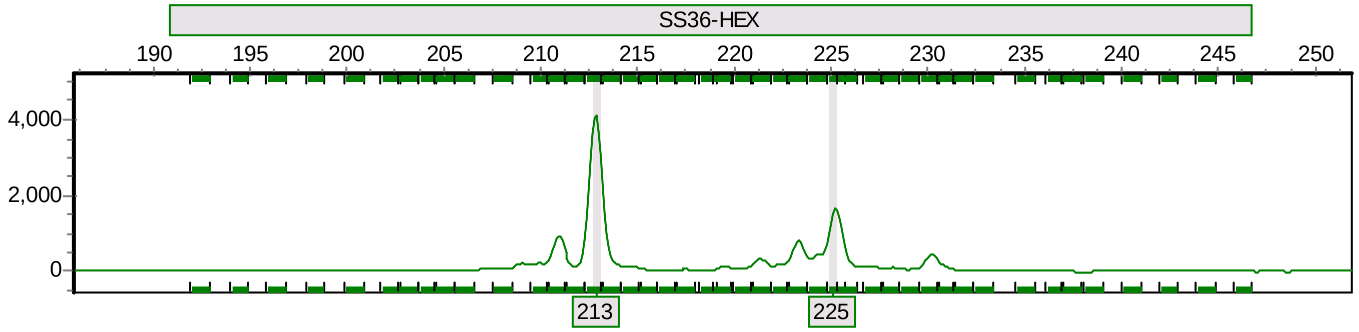

No	Size	Height	Area	Marker	Allele	Difference	Quality	Score	Allele Comments	Sample Comments
1	114.1	9175	63059	SS27-HEX	114	0.10	Pass	500.0	[<Confirmed>]	
2	119.0	6263	43059	SS27-HEX	118	0.40	Pass	500.0	[<Confirmed>]	
3	212.9	4081	30035	SS36-HEX	213	0.10	Pass	500.0	[<Confirmed>]	
4	225.2	1642	12612	SS36-HEX	225	0.10	Pass	189.8	[<Confirmed>]	
5	304.0	4409	43558	SS22-HEX	304	0.10	Pass	500.0		
6	310.2	1821	18092	SS22-HEX	310	0.10	Pass	135.0		

Sample 8: SS08_SS10_SSS42_SS16_SS27_SS36_SS22_HBB18_O05.fsa

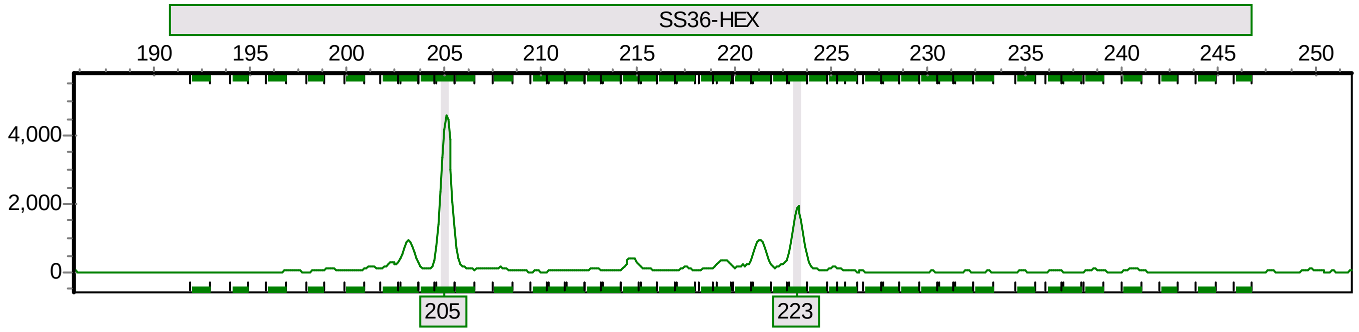

No	Size	Height	Area	Marker	Allele	Difference	Quality	Score	Allele Comments	Sample Comments
1	118.2	9487	61387	SS27-HEX	118	0.40	Pass	500.0	[<Confirmed>]	
2	205.1	4591	31065	SS36-HEX	205	0.00	Pass	500.0	[<Confirmed>]	
3	223.3	1933	13984	SS36-HEX	223	0.00	Pass	297.7	[<Confirmed>]	
4	290.2	4892	40352	SS22-HEX	290	0.10	Pass	500.0		

No	Size	Height	Area	Marker	Allele	Difference	Quality	Score	Allele Comments	Sample Comments
5	299.8	3403	29529	SS22-HEX	300	0.20	Pass	468.6		

Sample 9: SS08_SS10_SSS42_SS16_SS27_SS36_SS22_HBB19_O07.fsa Run date and time: 10/09/2021 - 19:59:30 -> 10/09/2021 - 20:38:12

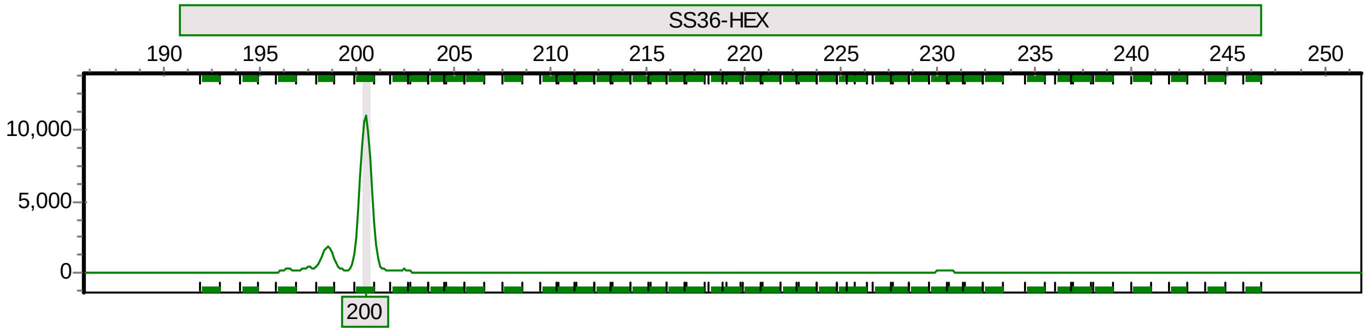

No	Size	Height	Area	Marker	Allele	Difference	Quality	Score	Allele Comments	Sample Comments
1	114.2	16908	109943	SS27-HEX	114	0.00	Pass	500.0	[<Confirmed>]	
2	200.5	10985	71835	SS36-HEX	200	0.10	Pass	500.0	[<Confirmed>]	
3	298.0	4230	36009	SS22-HEX	298	0.00	Pass	500.0		
4	301.9	2885	26174	SS22-HEX	302	0.20	Pass	330.0		

Sample 10: SS08_SS10_SSS42_SS16_SS27_SS36_SS22_HBB1_B11.fsa

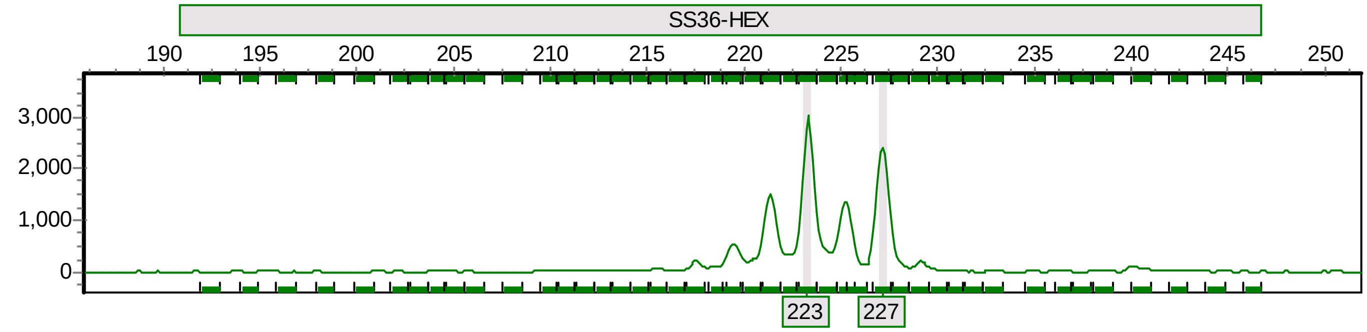

No	Size	Height	Area	Marker	Allele	Difference	Quality	Score	Allele Comments	Sample Comments
1	116.3	19301	133931	SS27-HEX	116	0.10	Pass	500.0	[<Confirmed>]	
2	223.3	3009	23180	SS36-HEX	223	0.00	Pass	499.3	[<Confirmed>]	
3	227.2	2406	18155	SS36-HEX	227	0.00	Pass	369.1	[<Confirmed>]	
4	300.1	5185	47180	SS22-HEX	300	0.10	Pass	500.0		
5	304.2	5649	53221	SS22-HEX	304	0.10	Pass	500.0		

Sample 11: SS08_SS10_SSS42_SS16_SS27_SS36_SS22_HBB20_F11.fsa

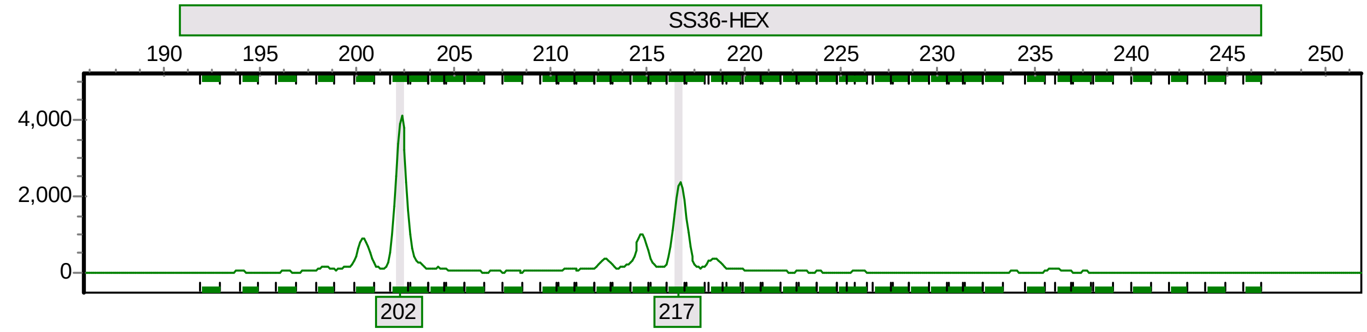

No	Size	Height	Area	Marker	Allele	Difference	Quality	Score	Allele Comments	Sample Comments
1	121.4	17865	120831	SS27-HEX	120	0.60	Pass	500.0	[<Confirmed>]	
2	202.3	4103	28667	SS36-HEX	202	0.00	Pass	500.0	[<Confirmed>]	
3	216.7	2368	17475	SS36-HEX	217	0.10	Pass	380.7	[<Confirmed>]	
4	296.2	5489	48099	SS22-HEX	296	0.10	Pass	500.0		
5	302.3	3860	35335	SS22-HEX	302	0.20	Pass	474.1		

Allele Report

Sample 12: SS08_SS10_SSS42_SS16_SS27_SS36_SS22_HBB21_J09.fsa

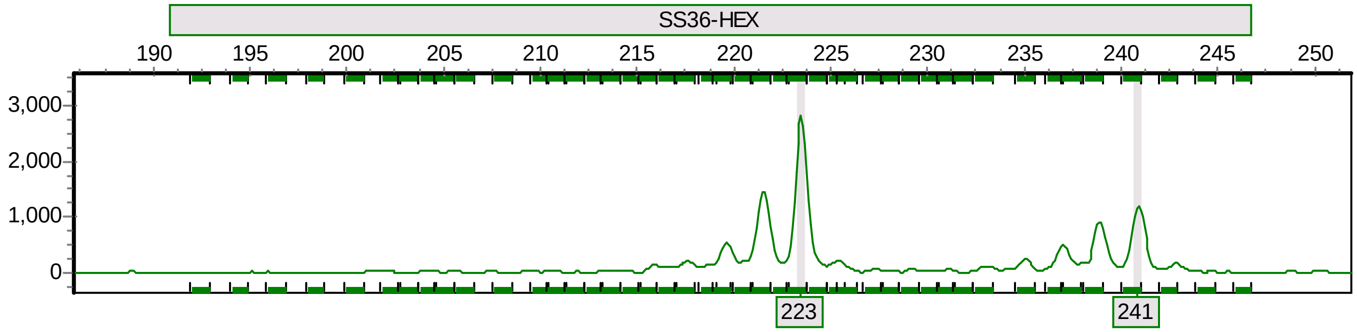

No	Size	Height	Area	Marker	Allele	Difference	Quality	Score	Allele Comments	Sample Comments
1	114.4	5497	39012	SS27-HEX	114	0.20	Pass	500.0	[<Confirmed>]	
2	128.4	10536	73299	SS27-HEX	128	0.20	Pass	500.0	[<Confirmed>]	
3	223.5	2807	21000	SS36-HEX	223	0.20	Pass	464.9	[<Confirmed>]	
4	240.9	1202	9674	SS36-HEX	241	0.30	Pass	117.6	[<Confirmed>]	
5	300.2	4083	40046	SS22-HEX	300	0.20	Pass	471.0		
6	304.4	4242	40367	SS22-HEX	304	0.30	Pass	500.0		

Sample 13: SS08_SS10_SSS42_SS16_SS27_SS36_SS22_HBB22_K05.fsa

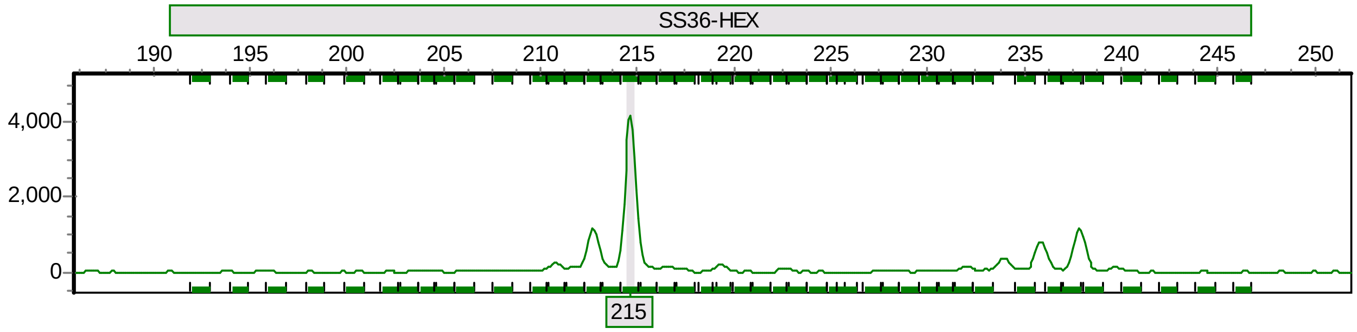

No	Size	Height	Area	Marker	Allele	Difference	Quality	Score	Allele Comments	Sample Comments
1	114.3	9286	60614	SS27-HEX	114	0.10	Pass	500.0	[<Deleted>]	
2	115.3	18159	117262	SS27-HEX	116	1.00	Pass	500.0	[<Confirmed><Edited>]	
3	214.7	4133	28044	SS36-HEX	215	0.00	Pass	500.0	[<Confirmed>]	
4	297.9	3249	27430	SS22-HEX	298	0.10	Pass	448.1		
5	303.9	3698	32583	SS22-HEX	304	0.20	Pass	493.2		

Sample 14: SS08_SS10_SSS42_SS16_SS27_SS36_SS22_HBB23_B09.fsa

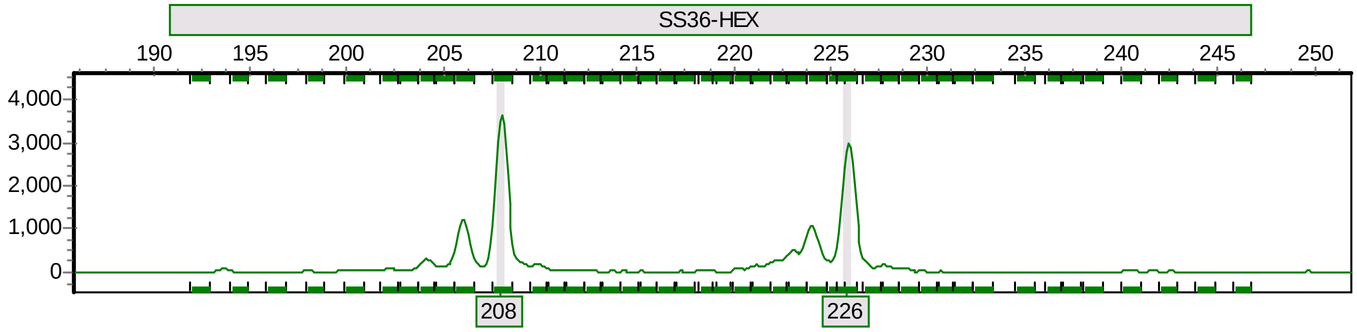

No	Size	Height	Area	Marker	Allele	Difference	Quality	Score	Allele Comments	Sample Comments
1	114.3	8112	56026	SS27-HEX	114	0.10	Pass	500.0	[<Confirmed>]	
2	119.1	5396	37074	SS27-HEX	118	0.50	Pass	500.0	[<Confirmed>]	
3	208.0	3614	26716	SS36-HEX	208	0.10	Pass	500.0	[<Confirmed>]	
4	225.9	2982	23404	SS36-HEX	226	0.00	Pass	469.5	[<Confirmed>]	

5	294.2	5414	47688	SS22-HEX	294	0.00	Pass	500.0		
6	310.3	2438	24051	SS22-HEX	310	0.00	Pass	202.2		

Sample 15: SS08_SS10_SSS42_SS16_SS27_SS36_SS22_HBB25-1_F09.fsa

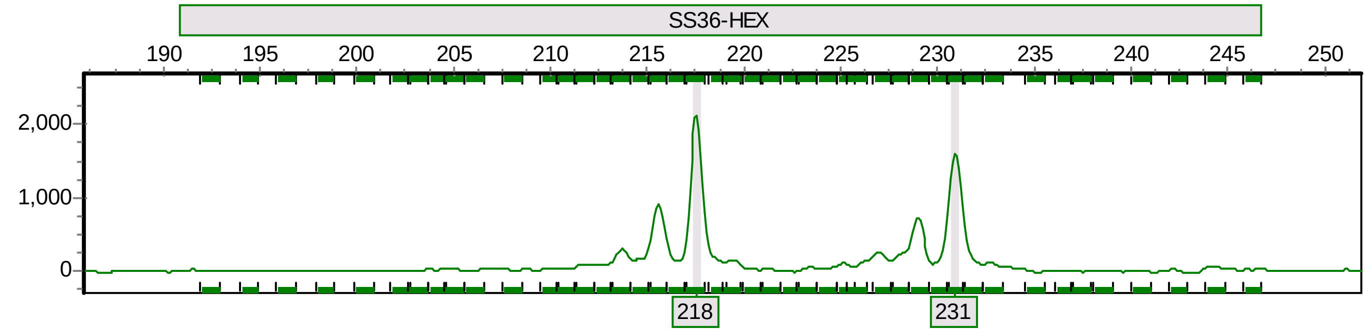

No	Size	Height	Area	Marker	Allele	Difference	Quality	Score	Allele Comments	Sample Comments
1	116.2	14887	101335	SS27-HEX	116	0.00	Pass	500.0	[<Confirmed>]	
2	217.6	2097	15563	SS36-HEX	218	0.10	Pass	315.8	[<Confirmed>]	
3	230.9	1598	12509	SS36-HEX	231	0.10	Pass	196.3	[<Confirmed>]	
4	294.3	6820	60374	SS22-HEX	294	0.10	Pass	500.0		

Sample 16: SS08_SS10_SSS42_SS16_SS27_SS36_SS22_HBB25-2_N11.fsa

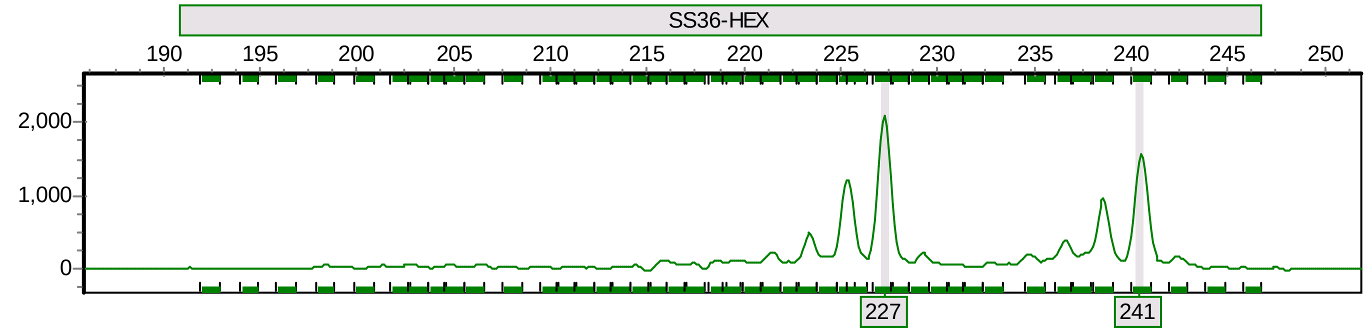

No	Size	Height	Area	Marker	Allele	Difference	Quality	Score	Allele Comments	Sample Comments
1	112.1	12683	86391	SS27-HEX	112	0.10	Pass	500.0	[<Confirmed>]	
2	126.1	6367	43997	SS27-HEX	126	1.00	Pass	500.0	[<Confirmed><Edited>]	
3	227.3	2075	15612	SS36-HEX	227	0.10	Pass	301.8	[<Confirmed>]	
4	240.5	1567	12103	SS36-HEX	241	0.10	Pass	193.7	[<Confirmed>]	
5	300.2	3249	30063	SS22-HEX	300	0.20	Pass	350.0		

Sample 17: SS08_SS10_SSS42_SS16_SS27_SS36_SS22_HBB26_D07.fsa

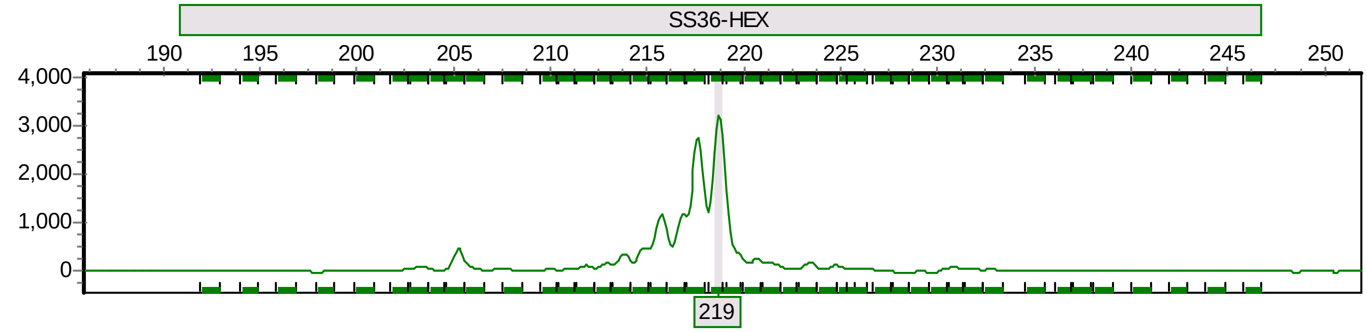

No	Size	Height	Area	Marker	Allele	Difference	Quality	Score	Allele Comments	Sample Comments
1	114.2	9275	62806	SS27-HEX	114	0.00	Pass	500.0	[<Confirmed>]	
2	119.2	5794	42120	SS27-HEX	118	0.60	Pass	500.0	[<Confirmed>]	
3	217.7	2737	20589	SS36-HEX	218	0.20	Pass	350.7	[<Deleted>]	
4	218.7	3192	23844	SS36-HEX	219	0.00	Pass	500.0	[<Confirmed>]	

5	294.3	6417	54141	SS22-HEX	294	0.10	Pass	500.0	
6	296.2	5003	43700	SS22-HEX	296	0.10	Pass	500.0	

Sample 18: SS08_SS10_SSS42_SS16_SS27_SS36_SS22_HBB27_H05.fsa

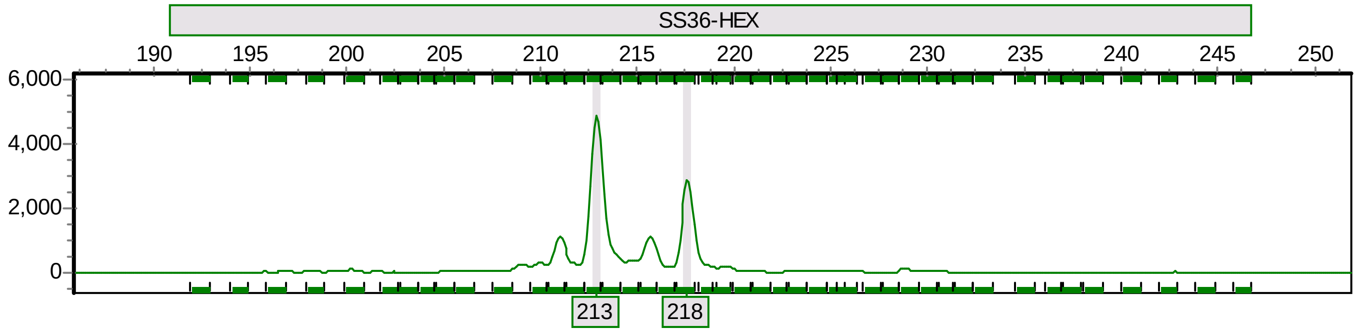

No	Size	Height	Area	Marker	Allele	Difference	Quality	Score	Allele Comments	Sample Comments
1	114.3	12771	86108	SS27-HEX	114	0.10	Pass	500.0	[<Confirmed>]	
2	116.3	8399	57858	SS27-HEX	116	0.10	Pass	500.0	[<Confirmed>]	
3	212.9	4872	35486	SS36-HEX	213	0.10	Pass	500.0	[<Confirmed>]	
4	217.6	2872	20760	SS36-HEX	218	0.10	Pass	500.0	[<Confirmed>]	
5	290.4	6468	56555	SS22-HEX	290	0.10	Pass	500.0		
6	296.2	4772	41383	SS22-HEX	296	0.10	Pass	500.0		

Sample 19: SS08_SS10_SSS42_SS16_SS27_SS36_SS22_HBB28_L11.fsa

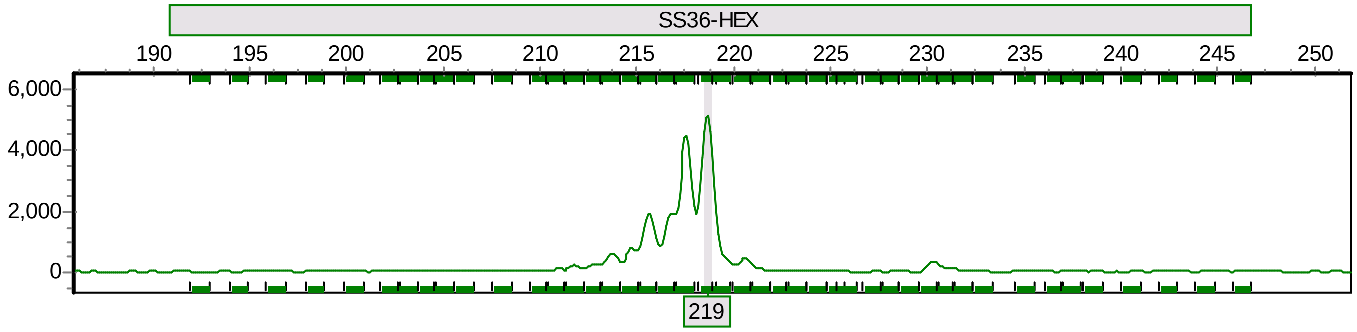

No	Size	Height	Area	Marker	Allele	Difference	Quality	Score	Allele Comments	Sample Comments
1	112.4	15599	104445	SS27-HEX	112	0.20	Pass	500.0	[<Confirmed>]	
2	114.4	11091	76453	SS27-HEX	114	0.20	Pass	500.0	[<Confirmed>]	
3	217.6	4503	33377	SS36-HEX	218	0.10	Pass	500.0	[<Deleted>]	
4	218.7	5129	38099	SS36-HEX	219	0.00	Pass	500.0	[<Confirmed>]	
5	296.1	11086	94886	SS22-HEX	296	0.00	Pass	500.0		

Sample 20: SS08_SS10_SSS42_SS16_SS27_SS36_SS22_HBB2_C07.fsa

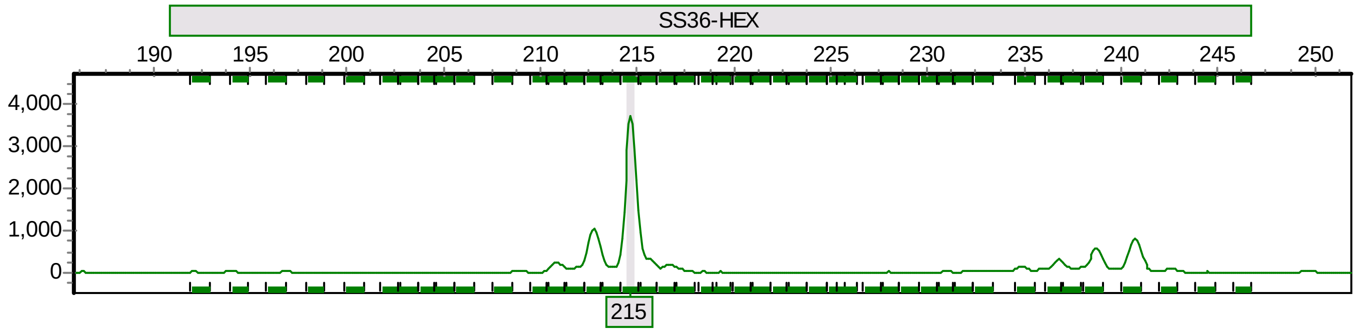

No	Size	Height	Area	Marker	Allele	Difference	Quality	Score	Allele Comments	Sample Comments
1	114.2	3607	24865	SS27-HEX	114	0.00	Pass	500.0	[<Confirmed>]	
2	128.1	9182	63007	SS27-HEX	128	0.10	Pass	500.0	[<Confirmed>]	

3	214.7	3692	25337	SS36-HEX	215	0.00	Pass	500.0	[<Confirmed>]	
4	300.0	4047	36044	SS22-HEX	300	0.00	Pass	500.0		
5	304.1	4192	37936	SS22-HEX	304	0.00	Pass	500.0		

Sample 21: SS08_SS10_SSS42_SS16_SS27_SS36_SS22_HBB30_L13.fsa

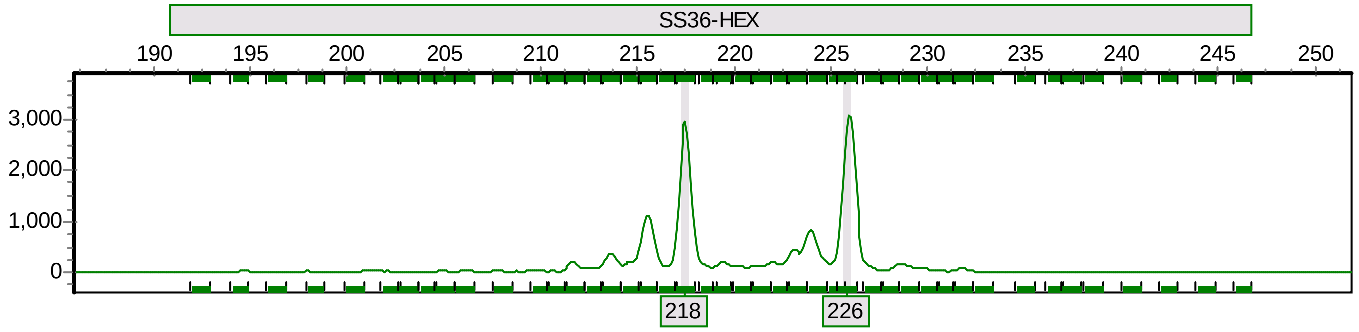

No	Size	Height	Area	Marker	Allele	Difference	Quality	Score	Allele Comments	Sample Comments
1	116.3	9718	69887	SS27-HEX	116	0.10	Pass	500.0	[<Confirmed>]	
2	119.2	6928	47871	SS27-HEX	118	0.60	Pass	500.0	[<Confirmed>]	
3	217.5	2977	21448	SS36-HEX	218	0.00	Pass	500.0	[<Confirmed>]	
4	225.9	3065	22967	SS36-HEX	226	0.00	Pass	500.0	[<Confirmed>]	
5	304.3	7509	70248	SS22-HEX	304	0.20	Pass	500.0		

Sample 22: SS08_SS10_SSS42_SS16_SS27_SS36_SS22_HBB31_P07.fsa

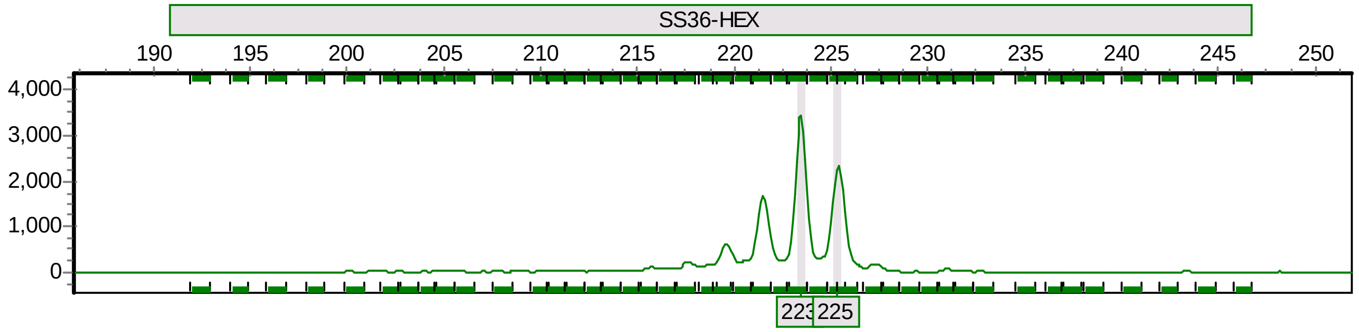

No	Size	Height	Area	Marker	Allele	Difference	Quality	Score	Allele Comments	Sample Comments
1	116.4	9764	66232	SS27-HEX	116	0.20	Pass	500.0	[<Confirmed>]	
2	118.3	3507	23609	SS27-HEX	118	0.30	Pass	500.0	[<Deleted>]	
3	119.3	6125	40868	SS27-HEX	118	1.00	Pass	500.0	[<Confirmed><Edited>]	
4	223.5	3413	24361	SS36-HEX	223	0.20	Pass	500.0	[<Confirmed>]	
5	225.4	2309	16951	SS36-HEX	225	0.10	Pass	379.5	[<Confirmed>]	
6	294.3	5572	47128	SS22-HEX	294	0.10	Pass	500.0		
7	306.3	2103	20066	SS22-HEX	306	0.10	Pass	192.9		

Sample 23: SS08_SS10_SSS42_SS16_SS27_SS36_SS22_HBB32_B15.fsa

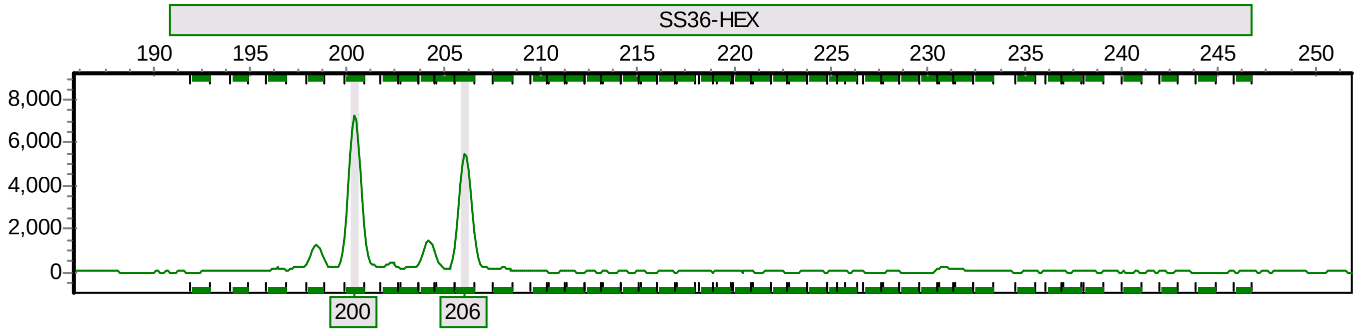

No	Size	Height	Area	Marker	Allele	Difference	Quality	Score	Allele Comments	Sample Comments

1	114.4	10344	69875	SS27-HEX	114	0.20	Pass	500.0	[<Confirmed>]	
2	118.4	7173	50212	SS27-HEX	118	0.20	Pass	500.0	[<Confirmed>]	
3	200.4	7205	50519	SS36-HEX	200	0.00	Pass	500.0	[<Confirmed>]	
4	206.1	5467	38944	SS36-HEX	206	0.00	Pass	500.0	[<Confirmed>]	
5	294.4	6010	52153	SS22-HEX	294	0.20	Pass	500.0		
6	310.5	2545	23961	SS22-HEX	310	0.20	Pass	261.5		

Sample 24: SS08_SS10_SSS42_SS16_SS27_SS36_SS22_HBB33_N13.fsa

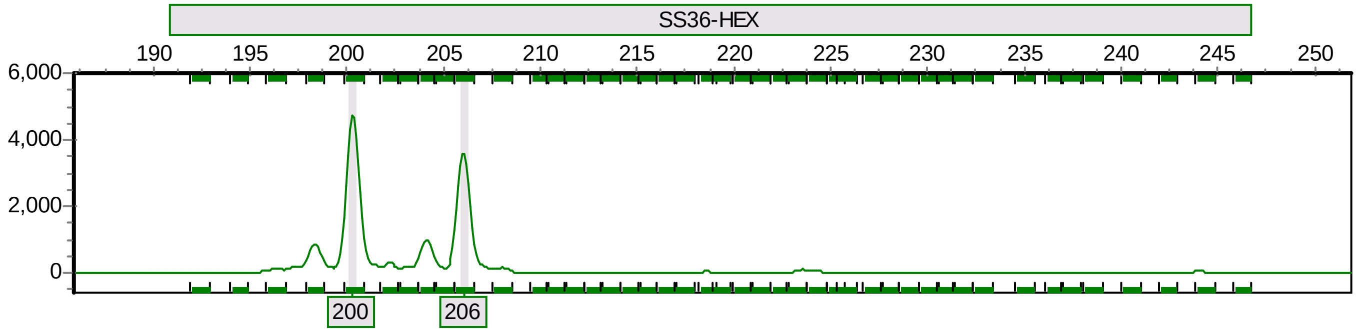

No	Size	Height	Area	Marker	Allele	Difference	Quality	Score	Allele Comments	Sample Comments
1	116.2	7543	52631	SS27-HEX	116	0.00	Pass	500.0	[<Confirmed>]	
2	119.2	5169	37354	SS27-HEX	118	0.60	Pass	500.0	[<Confirmed>]	
3	200.3	4717	34365	SS36-HEX	200	0.10	Pass	500.0	[<Confirmed>]	
4	206.1	3613	26846	SS36-HEX	206	0.00	Pass	500.0	[<Confirmed>]	
5	296.2	4387	41309	SS22-HEX	296	0.10	Pass	500.0		
6	300.0	2820	27603	SS22-HEX	300	0.00	Pass	275.0		

Sample 25: SS08_SS10_SSS42_SS16_SS27_SS36_SS22_HBB34_P13.fsa

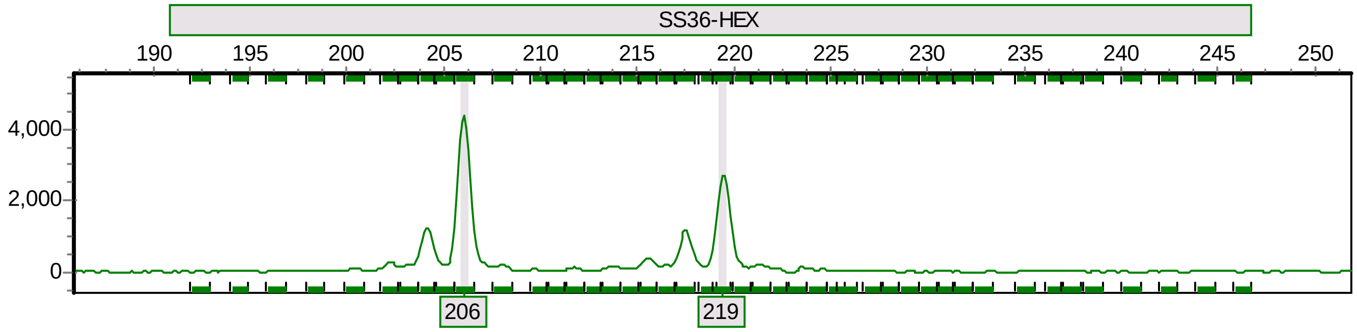

No	Size	Height	Area	Marker	Allele	Difference	Quality	Score	Allele Comments	Sample Comments
1	110.4	21207	140424	SS27-HEX	110	0.10	Pass	500.0	[<Confirmed>]	
2	206.1	4365	31570	SS36-HEX	206	0.00	Pass	500.0	[<Confirmed>]	
3	219.5	2695	20563	SS36-HEX	219	0.10	Pass	422.6	[<Confirmed>]	
4	294.2	5121	46285	SS22-HEX	294	0.00	Pass	500.0		
5	300.0	3741	35034	SS22-HEX	300	0.00	Pass	448.5		

Allele Report

Sample 26: SS08_SS10_SSS42_SS16_SS27_SS36_SS22_HBB35_C15.fsa

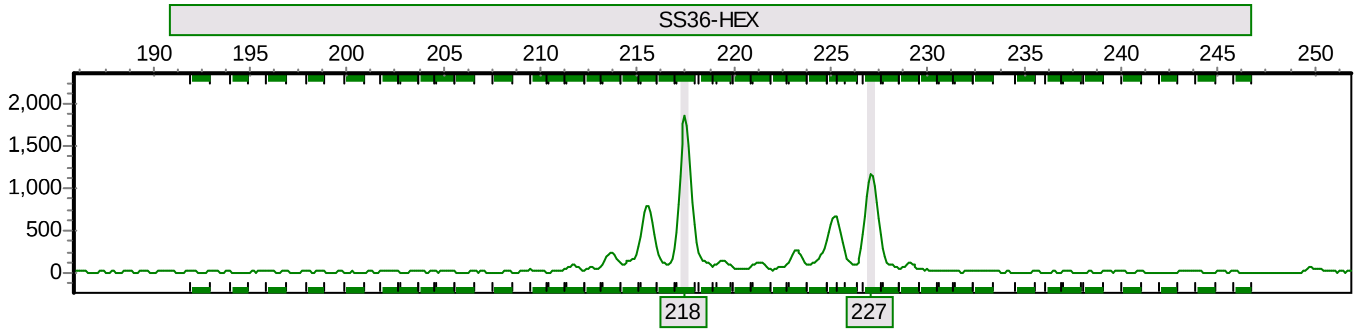

No	Size	Height	Area	Marker	Allele	Difference	Quality	Score	Allele Comments	Sample Comments
1	114.1	7071	50884	SS27-HEX	114	0.10	Pass	500.0	[<Confirmed>]	
2	119.1	5105	35257	SS27-HEX	118	0.50	Pass	500.0	[<Confirmed>]	
3	217.5	1852	13558	SS36-HEX	218	0.00	Pass	271.0	[<Confirmed>]	
4	227.1	1169	8897	SS36-HEX	227	0.10	Pass	128.7	[<Confirmed>]	
5	304.0	3607	34979	SS22-HEX	304	0.10	Pass	391.0		
6	310.2	1563	14600	SS22-HEX	310	0.10	Pass	123.5		

Sample 27: SS08_SS10_SSS42_SS16_SS27_SS36_SS22_HBB36_G05.fsa

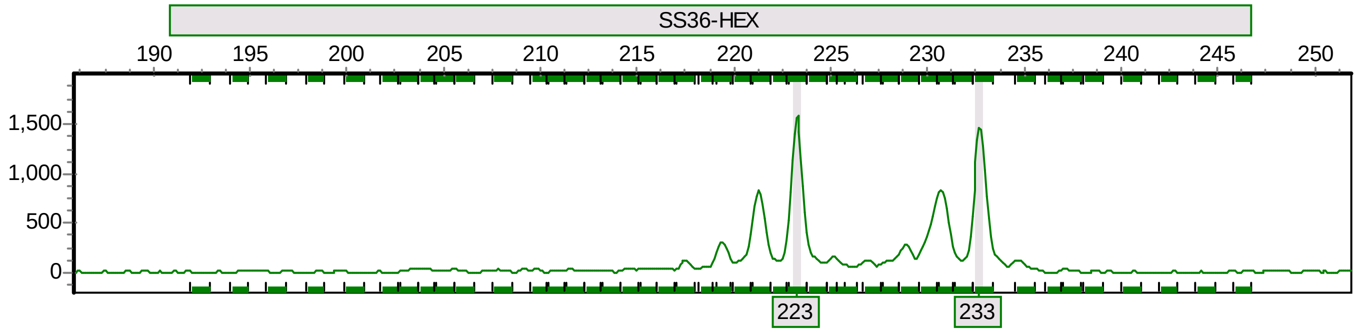

No	Size	Height	Area	Marker	Allele	Difference	Quality	Score	Allele Comments	Sample Comments
1	116.2	15112	103982	SS27-HEX	116	0.00	Pass	500.0	[<Confirmed>]	
2	223.3	1567	11720	SS36-HEX	223	0.00	Pass	208.0	[<Confirmed>]	
3	232.7	1456	11068	SS36-HEX	233	0.20	Pass	181.8	[<Confirmed>]	
4	294.0	4408	37013	SS22-HEX	294	0.20	Pass	500.0		
5	310.2	1961	18095	SS22-HEX	310	0.10	Pass	173.6		

Sample 28: SS08_SS10_SSS42_SS16_SS27_SS36_SS22_HBB37_N09.fsa

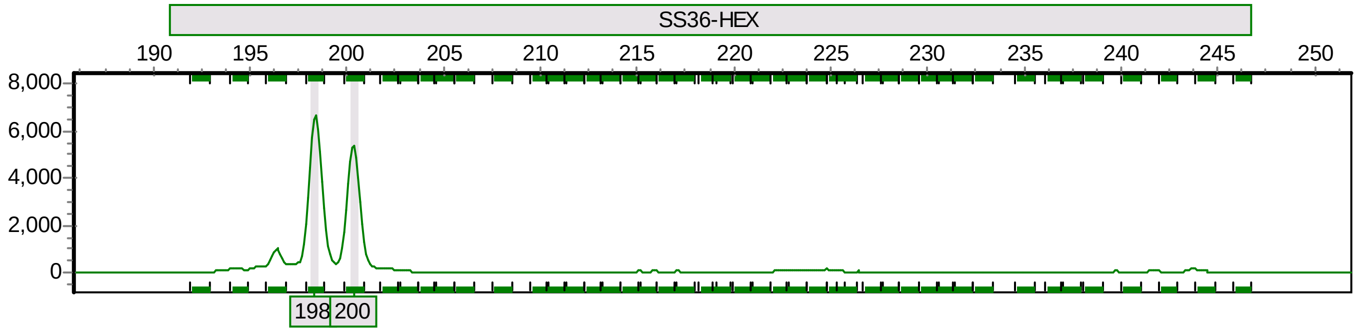

No	Size	Height	Area	Marker	Allele	Difference	Quality	Score	Allele Comments	Sample Comments
1	112.2	13947	94123	SS27-HEX	112	0.00	Pass	500.0	[<Confirmed>]	
2	114.2	9507	64964	SS27-HEX	114	0.00	Pass	500.0	[<Confirmed>]	
3	198.4	6617	48581	SS36-HEX	198	0.00	Pass	500.0	[<Confirmed>]	
4	200.4	5357	38796	SS36-HEX	200	0.00	Pass	500.0	[<Confirmed>]	

5	296.2	4124	36788	SS22-HEX	296	0.10	Pass	500.0		
6	304.2	4275	41510	SS22-HEX	304	0.10	Pass	500.0		

Sample 29: SS08_SS10_SSS42_SS16_SS27_SS36_SS22_HBB38_M07.fsa

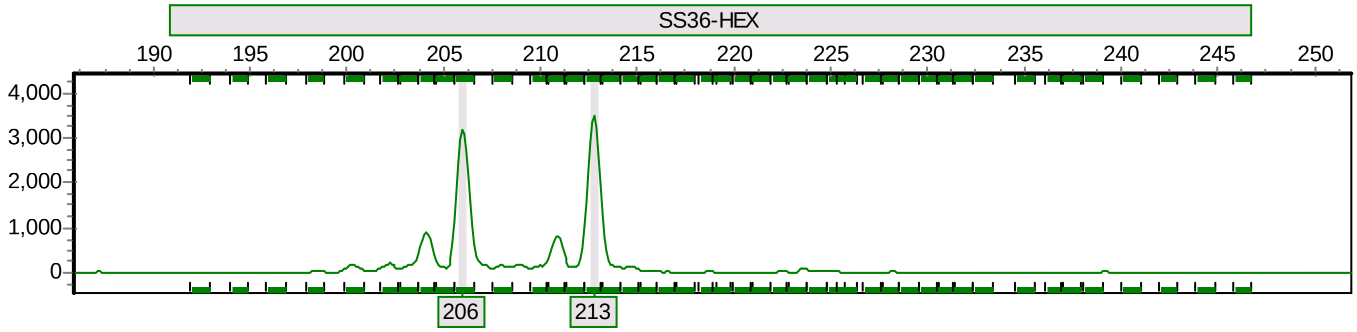

No	Size	Height	Area	Marker	Allele	Difference	Quality	Score	Allele Comments	Sample Comments
1	110.3	10877	72708	SS27-HEX	110	0.00	Pass	500.0	[<Confirmed>]	
2	114.2	8092	54563	SS27-HEX	114	0.00	Pass	500.0	[<Confirmed>]	
3	206.0	3196	22509	SS36-HEX	206	0.10	Pass	500.0	[<Confirmed>]	
4	212.8	3476	24504	SS36-HEX	213	0.00	Pass	500.0	[<Confirmed>]	
5	299.8	5026	45983	SS22-HEX	300	0.20	Pass	500.0		

Sample 30: SS08_SS10_SSS42_SS16_SS27_SS36_SS22_HBB39_F05.fsa

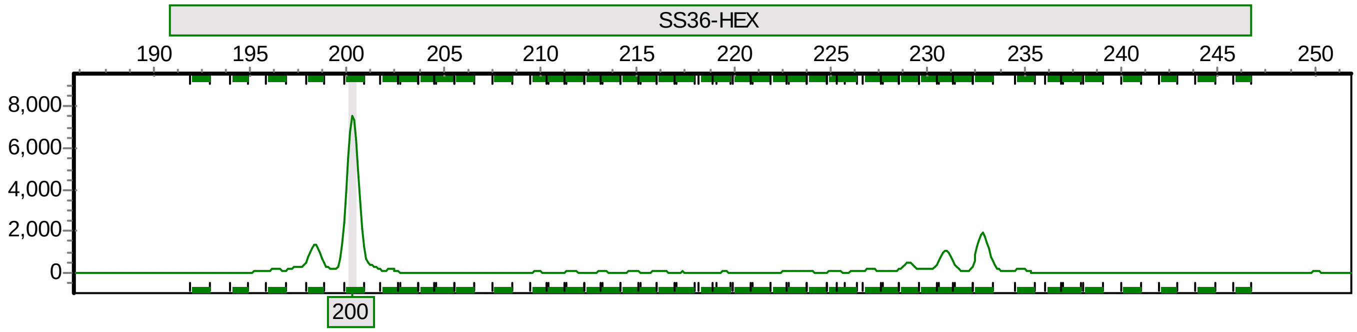

No	Size	Height	Area	Marker	Allele	Difference	Quality	Score	Allele Comments	Sample Comments
1	114.3	15400	101991	SS27-HEX	114	0.10	Pass	500.0	[<Confirmed>]	
2	116.3	10805	74387	SS27-HEX	116	0.10	Pass	500.0	[<Confirmed>]	
3	200.3	7503	51159	SS36-HEX	200	0.10	Pass	500.0	[<Confirmed>]	
4	290.3	7783	64391	SS22-HEX	290	0.00	Pass	500.0		
5	298.0	5387	44888	SS22-HEX	298	0.00	Pass	500.0		

Sample 31: SS08_SS10_SSS42_SS16_SS27_SS36_SS22_HBB40_D15.fsa

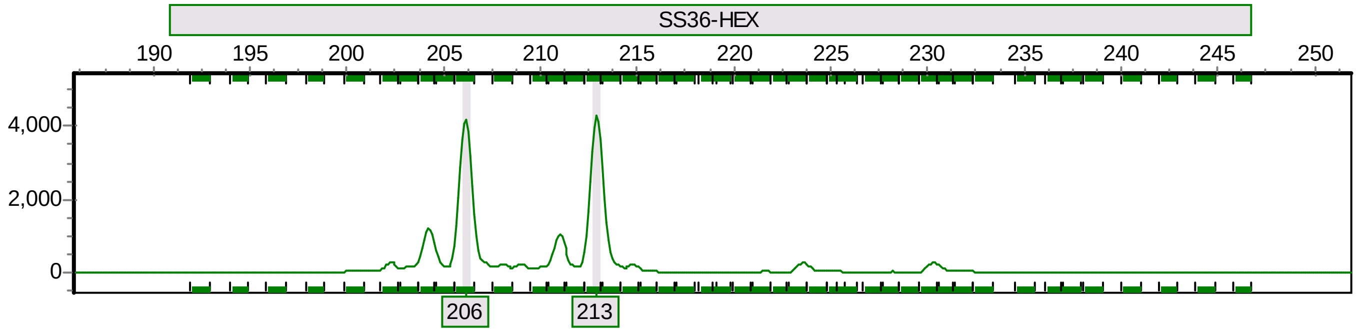

No	Size	Height	Area	Marker	Allele	Difference	Quality	Score	Allele Comments	Sample Comments
1	110.3	10893	73460	SS27-HEX	110	0.00	Pass	500.0	[<Confirmed>]	
2	114.3	8424	60565	SS27-HEX	114	0.10	Pass	500.0	[<Confirmed>]	
3	206.2	4194	30143	SS36-HEX	206	0.10	Pass	500.0	[<Confirmed>]	

4	212.9	4279	31071	SS36-HEX	213	0.10	Pass	500.0	[<Confirmed>]	
5	296.4	6531	58605	SS22-HEX	296	0.30	Pass	500.0		
6	300.2	4036	37326	SS22-HEX	300	0.20	Pass	500.0		

Sample 32: SS08_SS10_SSS42_SS16_SS27_SS36_SS22_HBB41_L07.fsa

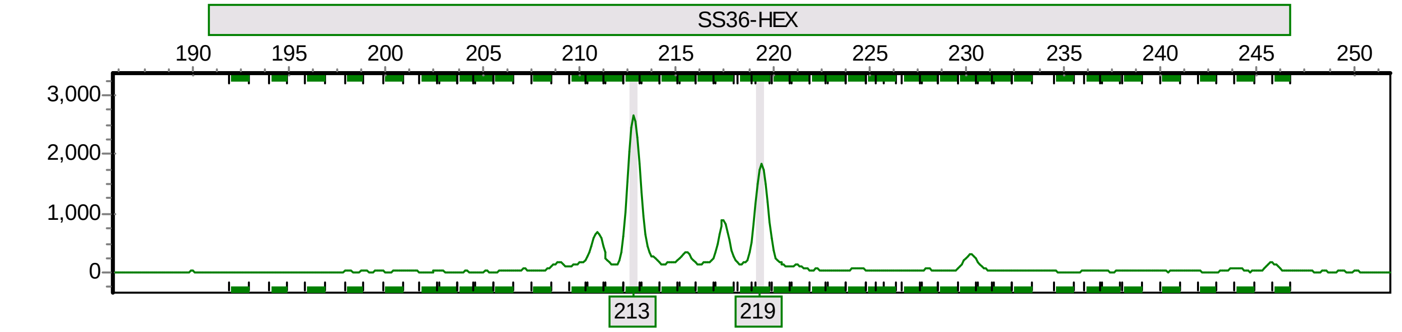

No	Size	Height	Area	Marker	Allele	Difference	Quality	Score	Allele Comments	Sample Comments
1	119.1	9042	61225	SS27-HEX	118	0.50	Pass	500.0	[<Confirmed>]	
2	212.8	2636	19743	SS36-HEX	213	0.00	Pass	436.2	[<Confirmed>]	
3	219.4	1837	13682	SS36-HEX	219	0.00	Pass	262.0	[<Confirmed>]	
4	296.3	3777	34100	SS22-HEX	296	0.20	Pass	500.0		
5	300.1	2397	22572	SS22-HEX	300	0.10	Pass	236.7		

Sample 33: SS08_SS10_SSS42_SS16_SS27_SS36_SS22_HBB42_M05.fsa

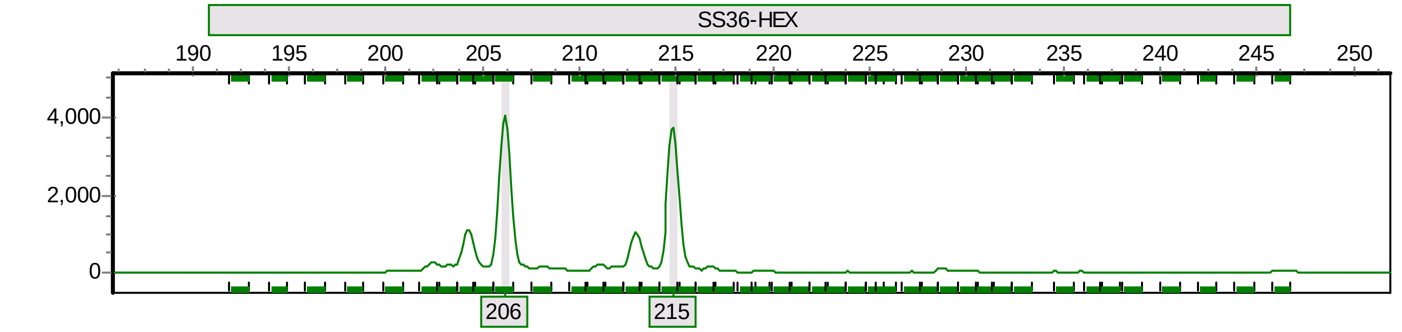

No	Size	Height	Area	Marker	Allele	Difference	Quality	Score	Allele Comments	Sample Comments
1	110.2	11923	78199	SS27-HEX	110	0.10	Pass	500.0	[<Confirmed>]	
2	116.2	9070	60451	SS27-HEX	116	0.00	Pass	500.0	[<Confirmed>]	
3	206.2	4039	26712	SS36-HEX	206	0.10	Pass	500.0	[<Confirmed>]	
4	214.9	3730	25639	SS36-HEX	215	0.20	Pass	500.0	[<Confirmed>]	
5	300.0	6597	56978	SS22-HEX	300	0.00	Pass	500.0		

Sample 34: SS08_SS10_SSS42_SS16_SS27_SS36_SS22_HBB43_D09.fsa

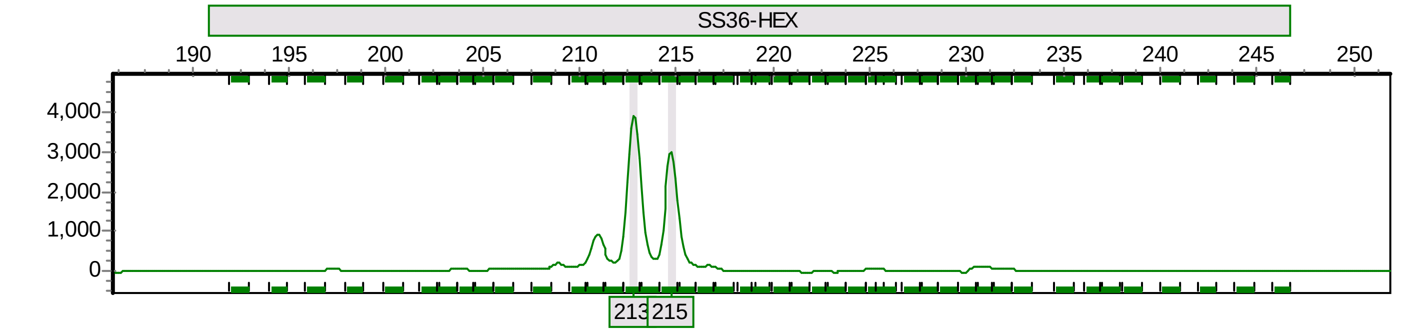

No	Size	Height	Area	Marker	Allele	Difference	Quality	Score	Allele Comments	Sample Comments
1	114.2	7954	56777	SS27-HEX	114	0.00	Pass	500.0	[<Confirmed>]	
2	119.2	6227	45742	SS27-HEX	118	0.60	Pass	500.0	[<Confirmed>]	

3	212.8	3876	29611	SS36-HEX	213	0.00	Pass	500.0	[<Confirmed>]	
4	214.8	2966	22733	SS36-HEX	215	0.10	Pass	470.7	[<Confirmed>]	
5	296.1	4804	43576	SS22-HEX	296	0.00	Pass	500.0		
6	300.1	3340	32576	SS22-HEX	300	0.10	Pass	345.5		

Sample 35: SS08_SS10_SSS42_SS16_SS27_SS36_SS22_HBB44_H11.fsa

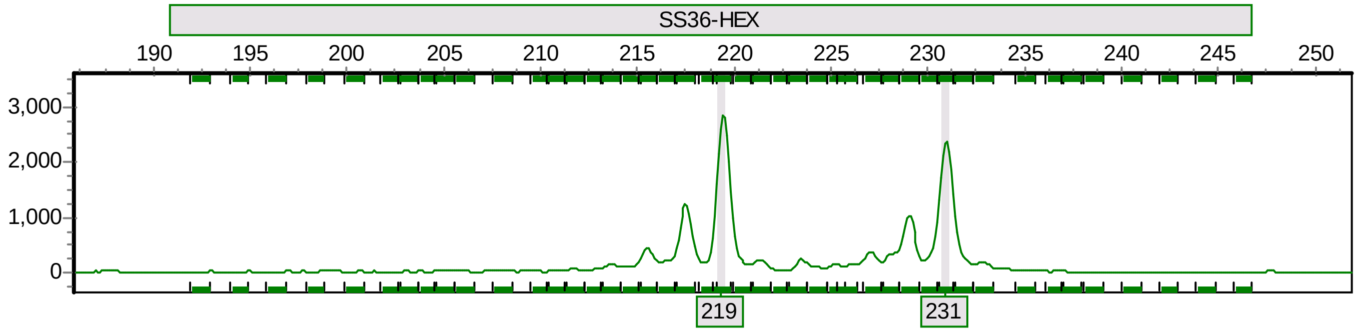

No	Size	Height	Area	Marker	Allele	Difference	Quality	Score	Allele Comments	Sample Comments
1	110.4	9589	65652	SS27-HEX	110	0.10	Pass	500.0	[<Confirmed>]	
2	119.2	5881	41959	SS27-HEX	118	0.60	Pass	500.0	[<Confirmed>]	
3	219.4	2835	20963	SS36-HEX	219	0.00	Pass	489.2	[<Confirmed>]	
4	231.0	2377	18781	SS36-HEX	231	0.00	Pass	337.0	[<Confirmed>]	
5	296.3	5497	50123	SS22-HEX	296	0.20	Pass	500.0		
6	300.1	4289	40854	SS22-HEX	300	0.10	Pass	500.0		

Sample 36: SS08_SS10_SSS42_SS16_SS27_SS36_SS22_HBB45_F13.fsa

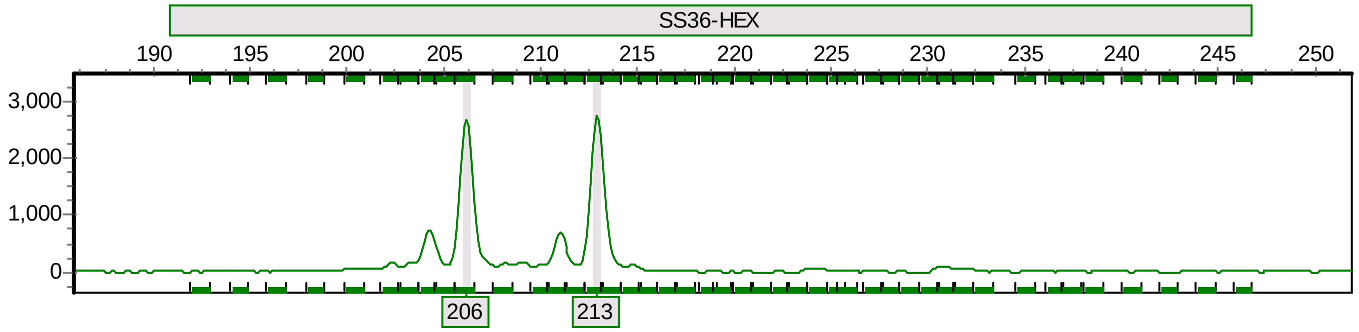

No	Size	Height	Area	Marker	Allele	Difference	Quality	Score	Allele Comments	Sample Comments
1	119.2	10394	72380	SS27-HEX	118	0.60	Pass	500.0	[<Confirmed>]	
2	206.2	2670	19647	SS36-HEX	206	0.10	Pass	451.7	[<Confirmed>]	
3	212.9	2724	20646	SS36-HEX	213	0.10	Pass	445.4	[<Confirmed>]	
4	300.1	5169	49374	SS22-HEX	300	0.10	Pass	500.0		

Sample 37: SS08_SS10_SSS42_SS16_SS27_SS36_SS22_HBB46_F07.fsa

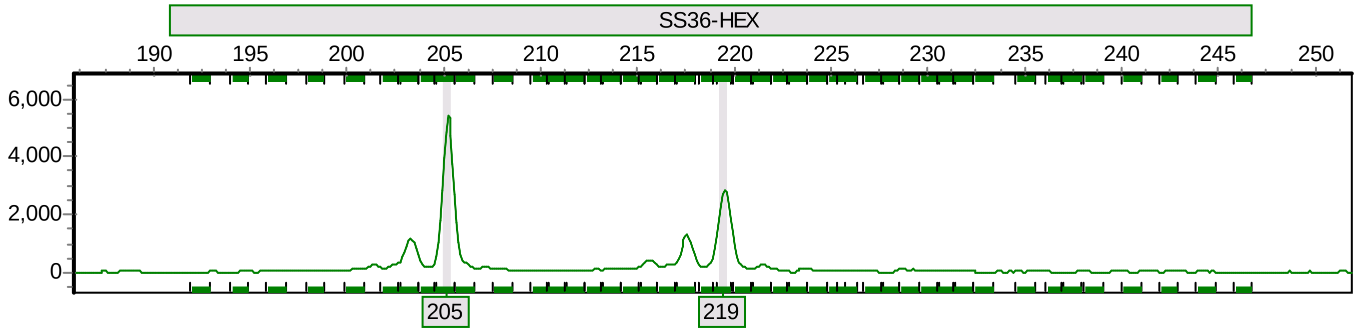

No	Size	Height	Area	Marker	Allele	Difference	Quality	Score	Allele Comments	Sample Comments
1	110.4	11572	76767	SS27-HEX	110	0.10	Pass	500.0	[<Confirmed>]	

2	114.3	9178	60450	SS27-HEX	114	0.10	Pass	500.0	[<Confirmed>]	
3	205.2	5389	37876	SS36-HEX	205	0.10	Pass	500.0	[<Confirmed>]	
4	219.5	2869	21210	SS36-HEX	219	0.10	Pass	495.9	[<Confirmed>]	
5	296.2	6280	55776	SS22-HEX	296	0.10	Pass	500.0		
6	300.0	4568	41664	SS22-HEX	300	0.00	Pass	500.0		

Sample 38: SS08_SS10_SSS42_SS16_SS27_SS36_SS22_HBB47_A05.fsa

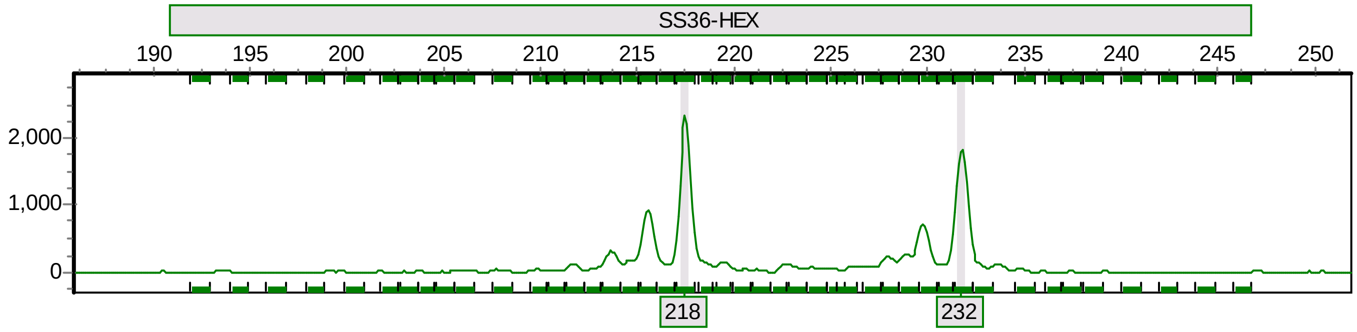

No	Size	Height	Area	Marker	Allele	Difference	Quality	Score	Allele Comments	Sample Comments
1	114.2	4598	30398	SS27-HEX	114	0.00	Pass	500.0	[<Confirmed>]	
2	123.1	13894	91567	SS27-HEX	122	0.10	Pass	500.0	[<Confirmed>]	
3	217.5	2301	15693	SS36-HEX	218	0.00	Pass	421.5	[<Confirmed>]	
4	231.8	1802	12990	SS36-HEX	232	0.10	Pass	265.7	[<Confirmed>]	
5	302.0	5064	44571	SS22-HEX	302	0.10	Pass	500.0		
6	304.1	4470	39720	SS22-HEX	304	0.00	Pass	500.0		

Sample 39: SS08_SS10_SSS42_SS16_SS27_SS36_SS22_HBB48_B13.fsa

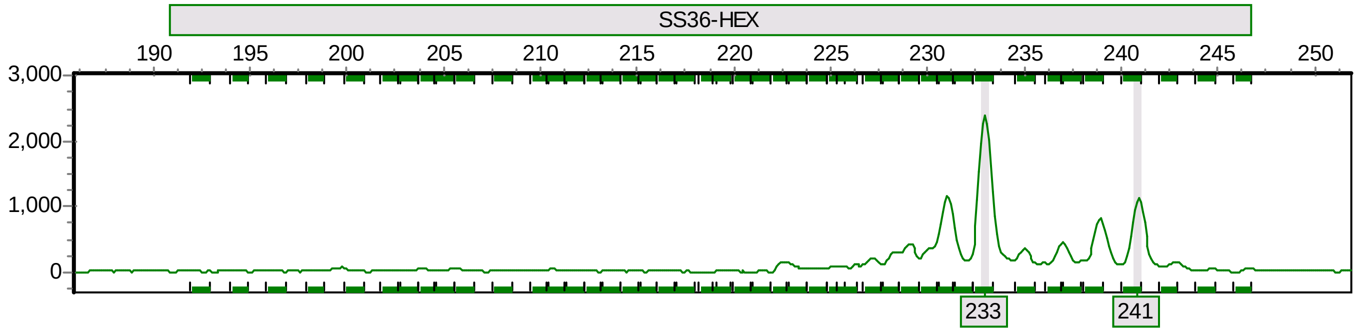

No	Size	Height	Area	Marker	Allele	Difference	Quality	Score	Allele Comments	Sample Comments
1	123.4	13967	95334	SS27-HEX	122	0.40	Pass	500.0	[<Confirmed>]	
2	233.0	2373	18578	SS36-HEX	233	0.10	Pass	341.9	[<Confirmed>]	
3	240.9	1110	8928	SS36-HEX	241	0.30	Pass	109.2	[<Confirmed>]	
4	298.2	7976	72179	SS22-HEX	298	0.20	Pass	500.0		

Sample 40: SS08_SS10_SSS42_SS16_SS27_SS36_SS22_HBB49_L05.fsa

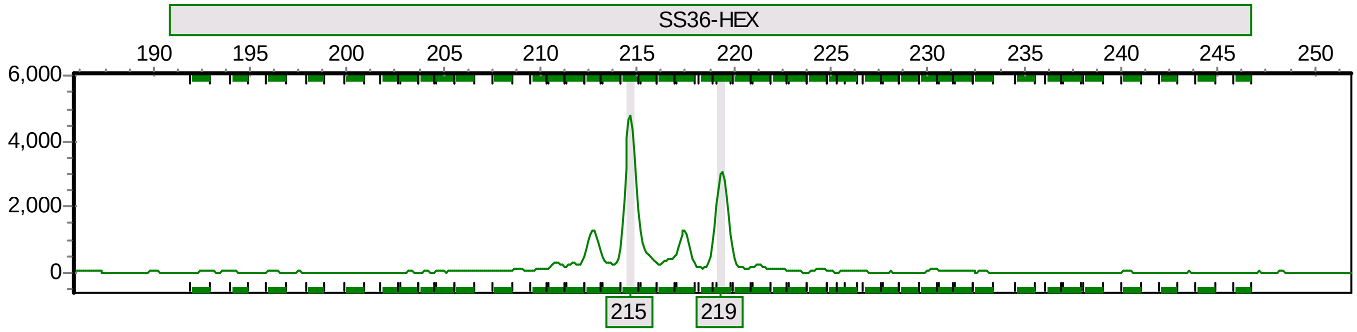

No	Size	Height	Area	Marker	Allele	Difference	Quality	Score	Allele Comments	Sample Comments

1	118.2	8784	58494	SS27-HEX	118	0.40	Pass	500.0	[<Confirmed>]	
2	122.2	6524	42388	SS27-HEX	122	1.00	Pass	500.0	[<Confirmed><Edited>]	
3	214.7	4754	34401	SS36-HEX	215	0.00	Pass	500.0	[<Confirmed>]	
4	219.4	3054	21681	SS36-HEX	219	0.00	Pass	500.0	[<Confirmed>]	
5	304.2	9381	86853	SS22-HEX	304	0.10	Pass	500.0		

Sample 41: SS08_SS10_SSS42_SS16_SS27_SS36_SS22_HBB4_E07.fsa

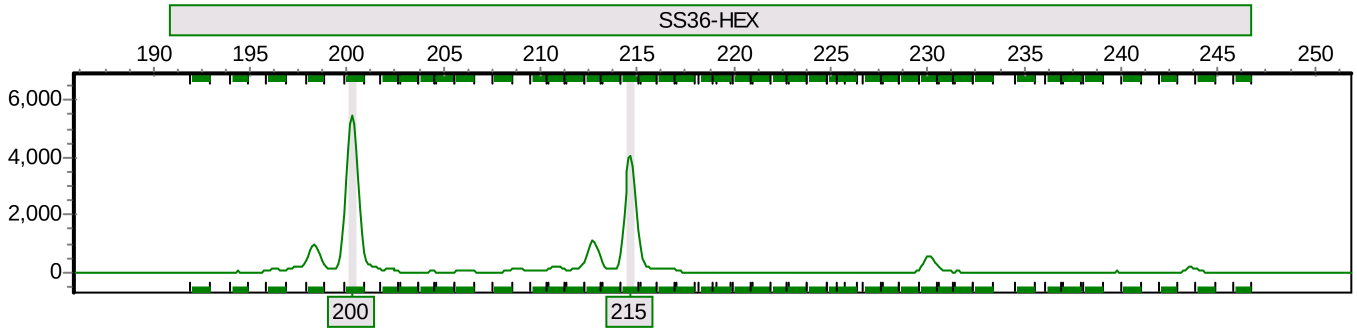

No	Size	Height	Area	Marker	Allele	Difference	Quality	Score	Allele Comments	Sample Comments
1	116.2	19104	131126	SS27-HEX	116	0.00	Pass	500.0	[<Confirmed>]	
2	200.3	5423	36998	SS36-HEX	200	0.10	Pass	500.0	[<Confirmed>]	
3	214.7	4042	28681	SS36-HEX	215	0.00	Pass	500.0	[<Confirmed>]	
4	299.8	5005	43721	SS22-HEX	300	0.20	Pass	500.0		
5	304.0	5641	53186	SS22-HEX	304	0.10	Pass	500.0		

Sample 42: SS08_SS10_SSS42_SS16_SS27_SS36_SS22_HBB5_H13.fsa

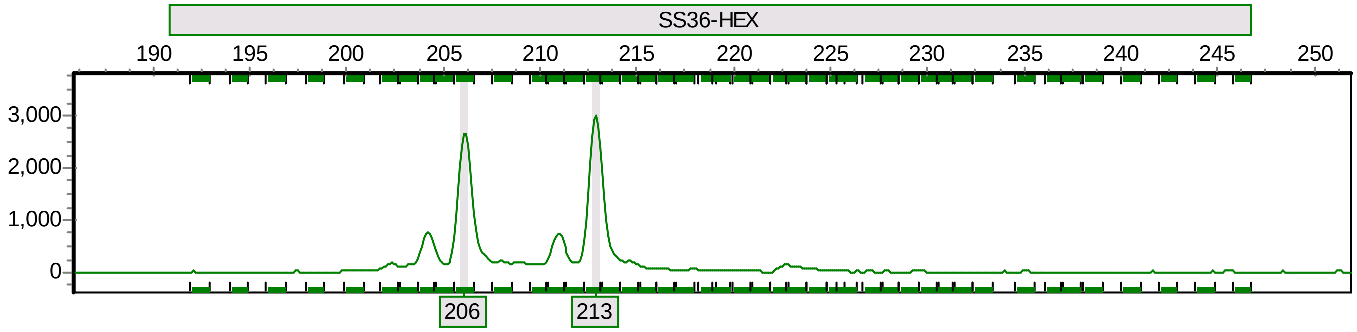

No	Size	Height	Area	Marker	Allele	Difference	Quality	Score	Allele Comments	Sample Comments
1	118.3	8421	63417	SS27-HEX	118	0.30	Pass	500.0	[<Confirmed>]	
2	120.3	5628	42249	SS27-HEX	120	0.50	Pass	500.0	[<Confirmed>]	
3	206.1	2682	20882	SS36-HEX	206	0.00	Pass	408.7	[<Confirmed>]	
4	212.9	3001	22772	SS36-HEX	213	0.10	Pass	475.2	[<Confirmed>]	
5	304.2	4129	42592	SS22-HEX	304	0.10	Pass	369.8		
6	310.3	1983	22524	SS22-HEX	310	0.00	Pass	107.5		

Allele Report

Sample 43: SS08_SS10_SSS42_SS16_SS27_SS36_SS22_HBB6_C05.fsa

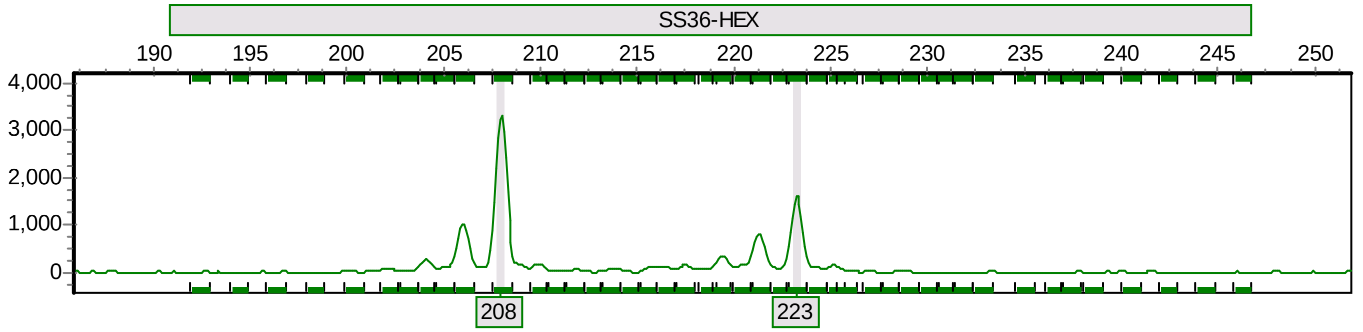

No	Size	Height	Area	Marker	Allele	Difference	Quality	Score	Allele Comments	Sample Comments
1	119.1	19386	130453	SS27-HEX	118	0.50	Pass	500.0	[<Confirmed>]	
2	208.0	3301	22345	SS36-HEX	208	0.10	Pass	500.0	[<Confirmed>]	
3	223.3	1621	11505	SS36-HEX	223	0.00	Pass	233.6	[<Confirmed>]	
4	299.9	3917	32981	SS22-HEX	300	0.10	Pass	500.0		
5	306.1	2947	25830	SS22-HEX	306	0.10	Pass	367.7		

Sample 44: SS08_SS10_SSS42_SS16_SS27_SS36_SS22_HBB7_N05.fsa

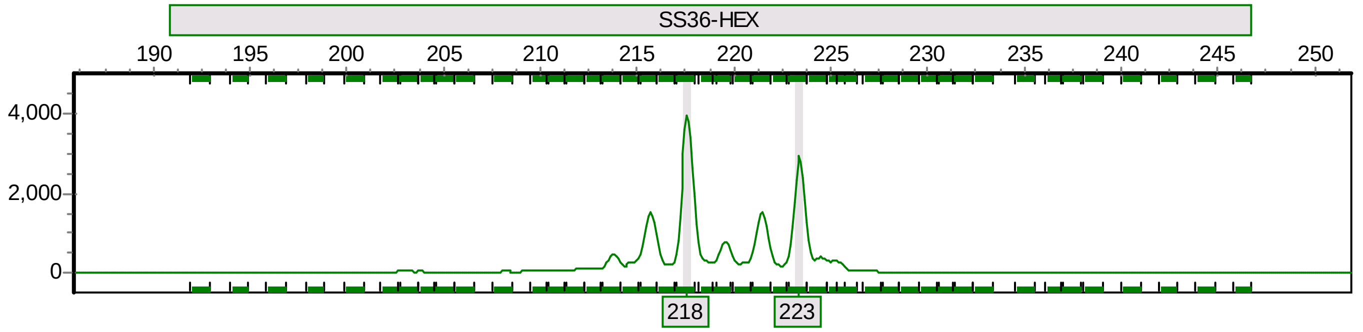

No	Size	Height	Area	Marker	Allele	Difference	Quality	Score	Allele Comments	Sample Comments
1	118.3	13028	84439	SS27-HEX	118	0.30	Pass	500.0	[<Confirmed>]	
2	217.6	3952	28004	SS36-HEX	218	0.10	Pass	500.0	[<Confirmed>]	
3	223.4	2956	21088	SS36-HEX	223	0.10	Pass	500.0	[<Confirmed>]	
4	300.0	3545	31393	SS22-HEX	300	0.00	Pass	446.4		
5	310.3	2090	19350	SS22-HEX	310	0.00	Pass	191.7		

Sample 45: SS08_SS10_SSS42_SS16_SS27_SS36_SS22_HBB8_P11.fsa

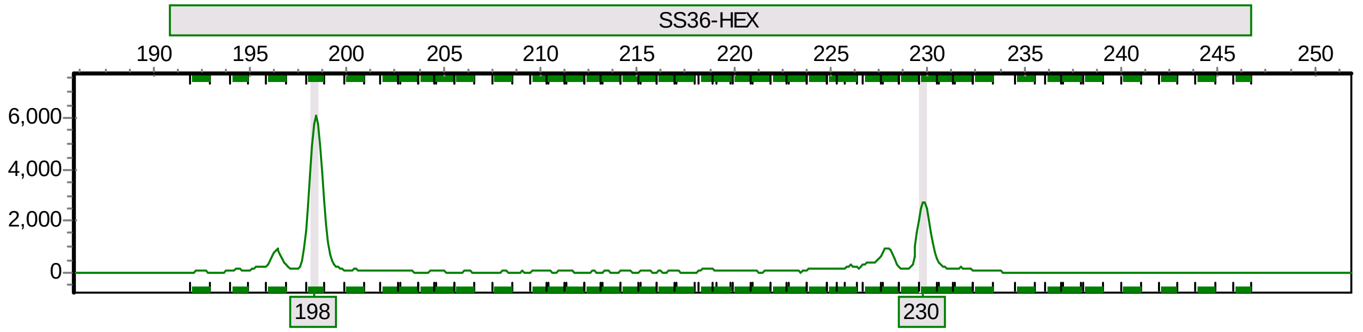

No	Size	Height	Area	Marker	Allele	Difference	Quality	Score	Allele Comments	Sample Comments
1	119.1	16654	116781	SS27-HEX	118	0.50	Pass	500.0	[<Confirmed>]	
2	198.4	6051	44742	SS36-HEX	198	0.00	Pass	500.0	[<Confirmed>]	
3	229.8	2726	21216	SS36-HEX	230	0.30	Pass	423.9	[<Confirmed>]	
4	294.1	4841	43865	SS22-HEX	294	0.10	Pass	500.0		
5	300.1	3650	35085	SS22-HEX	300	0.10	Pass	410.6		

Allele Report

Sample 46: SS08_SS10_SSS42_SS16_SS27_SS36_SS22_HBB9_I05.fsa

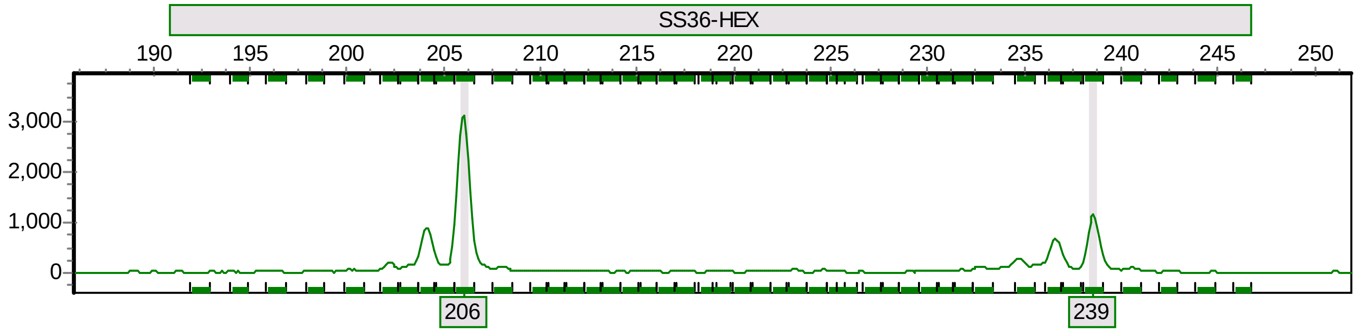

No	Size	Height	Area	Marker	Allele	Difference	Quality	Score	Allele Comments	Sample Comments
1	123.2	13525	91400	SS27-HEX	122	0.20	Pass	500.0	[<Confirmed>]	
2	206.1	3121	21835	SS36-HEX	206	0.00	Pass	500.0	[<Confirmed>]	
3	238.6	1164	8830	SS36-HEX	239	0.00	Pass	126.6	[<Confirmed>]	
4	290.3	3978	33200	SS22-HEX	290	0.00	Pass	500.0		
5	302.1	2530	23777	SS22-HEX	302	0.00	Pass	251.0		

Sample 47: SS08_SS10_SSS42_SS16_SS27_SS36_SS22_HBN10_G11.fsa

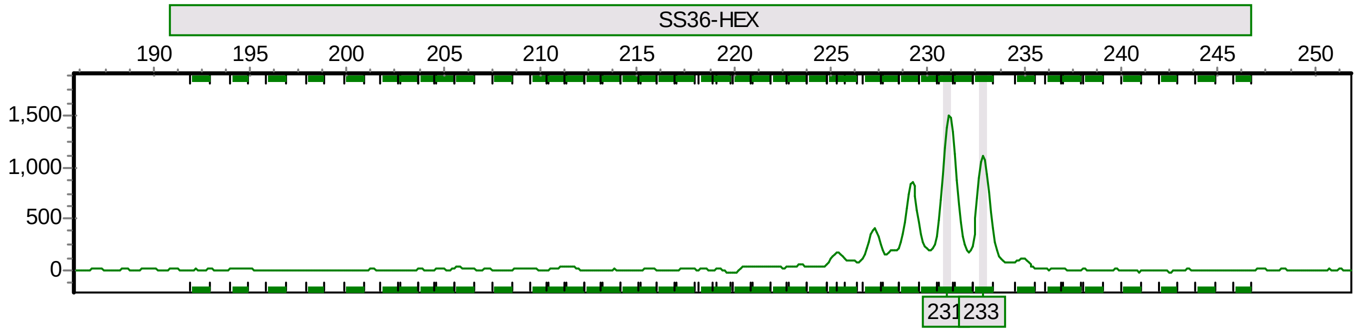

No	Size	Height	Area	Marker	Allele	Difference	Quality	Score	Allele Comments	Sample Comments
1	119.1	12844	86023	SS27-HEX	118	0.50	Pass	500.0	[<Confirmed>]	
2	121.1	8196	54696	SS27-HEX	120	0.30	Pass	500.0	[<Confirmed>]	
3	231.1	1486	12010	SS36-HEX	231	0.10	Pass	154.1	[<Confirmed>]	
4	232.9	1106	8607	SS36-HEX	233	0.00	Pass	112.1	[<Confirmed>]	
5	292.2	4023	33635	SS22-HEX	292	0.00	Pass	500.0		
6	306.2	2063	18799	SS22-HEX	306	0.00	Pass	195.3		

Sample 48: SS08_SS10_SSS42_SS16_SS27_SS36_SS22_HBN6_O01.fsa

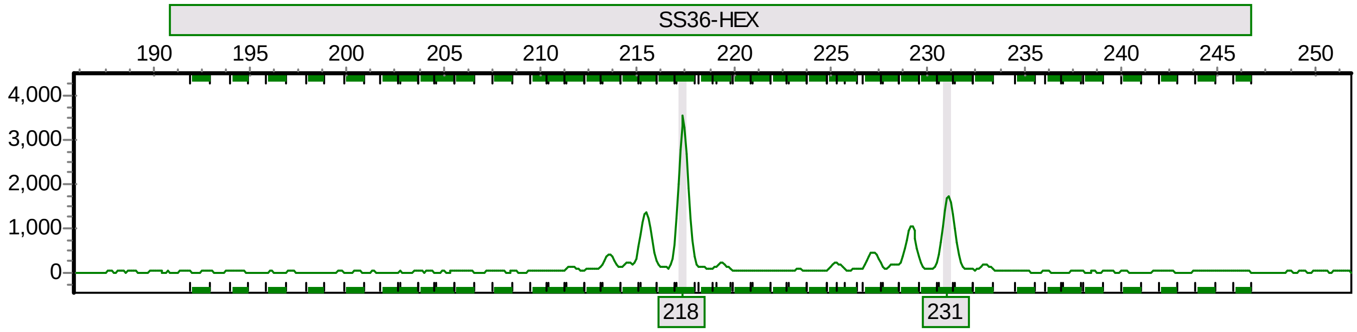

No	Size	Height	Area	Marker	Allele	Difference	Quality	Score	Allele Comments	Sample Comments
1	121.0	18959	120633	SS27-HEX	120	0.20	Pass	500.0	[<Confirmed>]	
2	217.4	3516	22420	SS36-HEX	218	0.10	Pass	500.0	[<Confirmed>]	
3	231.1	1710	11617	SS36-HEX	231	0.10	Pass	275.7	[<Confirmed>]	
4	297.8	6479	50950	SS22-HEX	298	0.20	Pass	500.0		

5	299.7	4060	32381	SS22-HEX	300	0.30	Pass	500.0		

Sample 49: SS08_SS10_SSS42_SS16_SS27_SS36_SS22_HBN9_D03.fsa

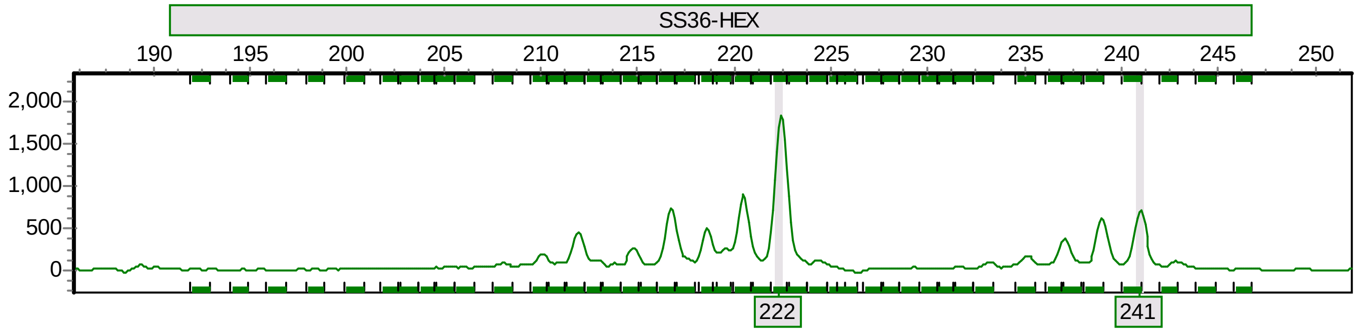

No	Size	Height	Area	Marker	Allele	Difference	Quality	Score	Allele Comments	Sample Comments
1	119.1	9169	60250	SS27-HEX	118	0.50	Pass	500.0	[<Deleted>]	
2	120.1	18591	125577	SS27-HEX	120	1.00	Pass	500.0	[<Confirmed><Edited>]	
3	216.7	730	5097	SS36-HEX	217	0.10	Pass	73.9	[<Deleted>]	
4	222.4	1818	13209	SS36-HEX	222	0.00	Pass	268.3	[<Confirmed>]	
5	241.0	699	5347	SS36-HEX	241	0.40	Pass	57.7	[<Confirmed><Edited>]	
6	298.1	4117	34921	SS22-HEX	298	0.10	Pass	500.0		

Sample 50: SS08_SS10_SSS42_SS16_SS27_SS36_SS22_HCW1_M09.fsa

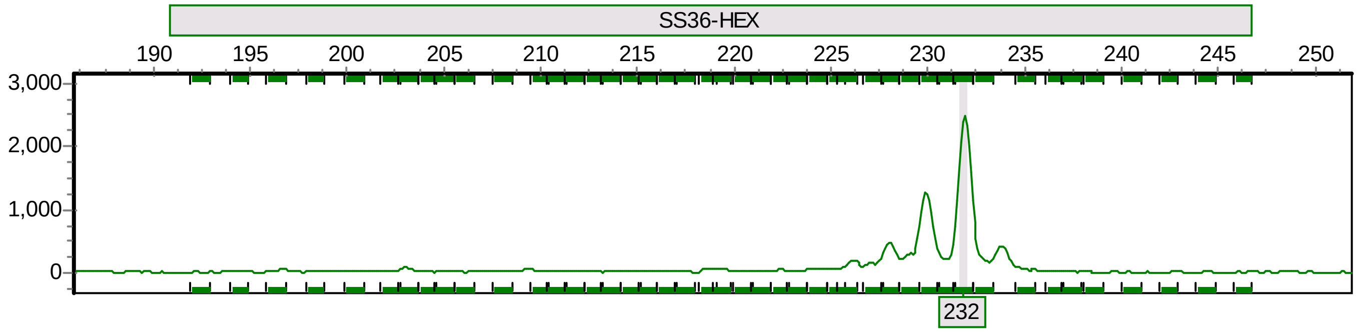

No	Size	Height	Area	Marker	Allele	Difference	Quality	Score	Allele Comments	Sample Comments
1	119.2	13135	92010	SS27-HEX	118	0.60	Pass	500.0	[<Confirmed>]	
2	121.2	8969	61624	SS27-HEX	120	0.40	Pass	500.0	[<Confirmed>]	
3	231.9	2480	18520	SS36-HEX	232	0.00	Pass	380.8	[<Confirmed>]	
4	286.3	4333	37097	SS22-HEX	286	0.10	Pass	500.0		
5	300.0	2113	20154	SS22-HEX	300	0.00	Pass	194.5		

Sample 51: SS08_SS10_SSS42_SS16_SS27_SS36_SS22_HCW2_A11.fsa

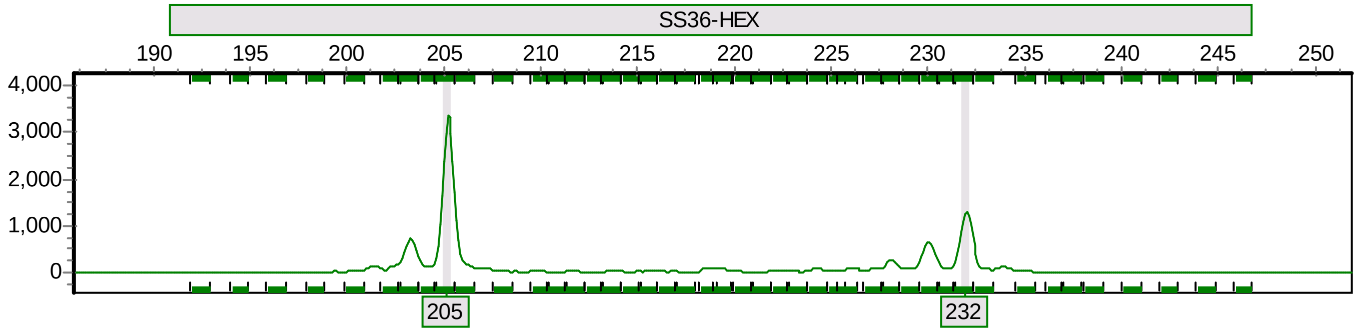

No	Size	Height	Area	Marker	Allele	Difference	Quality	Score	Allele Comments	Sample Comments
1	118.2	9929	69419	SS27-HEX	118	0.40	Pass	500.0	[<Confirmed>]	
2	205.2	3345	23499	SS36-HEX	205	0.10	Pass	500.0	[<Confirmed>]	
3	232.0	1298	9639	SS36-HEX	232	0.10	Pass	155.4	[<Confirmed>]	

Allele Report

4	302.1	2444	22354	SS22-HEX	302	0.00	Pass	247.5		
5	308.2	2031	18959	SS22-HEX	308	0.10	Pass	189.6		

Sample 52: SS08_SS10_SSS42_SS16_SS27_SS36_SS22_HCW3_G13.fsa

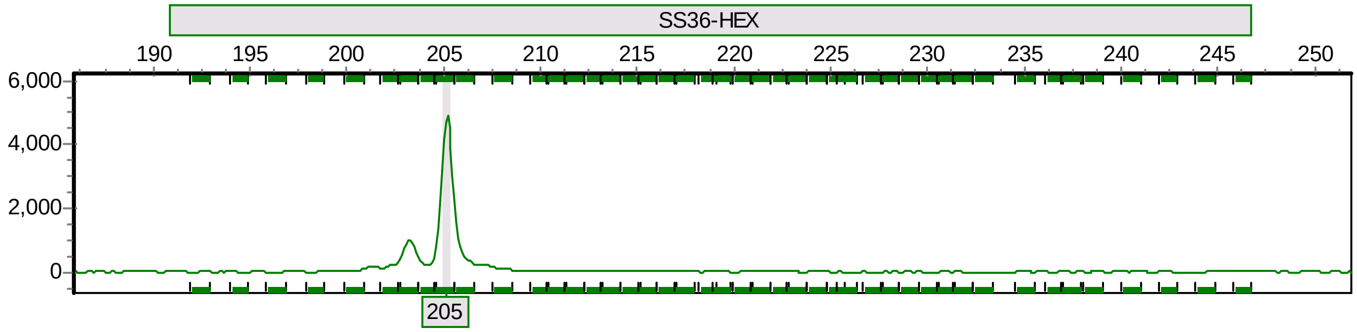

No	Size	Height	Area	Marker	Allele	Difference	Quality	Score	Allele Comments	Sample Comments
1	112.3	3208	20506	SS27-HEX	112	0.10	Pass	389.7	[<Deleted>]	
2	113.3	7404	53695	SS27-HEX	114	1.00	Pass	500.0	[<Confirmed><Edited>]	
3	119.1	6459	47753	SS27-HEX	118	0.50	Pass	500.0	[<Confirmed>]	
4	205.2	4892	35984	SS36-HEX	205	0.10	Pass	500.0	[<Confirmed>]	
5	298.0	2785	27273	SS22-HEX	298	0.00	Pass	272.8		
6	299.9	1907	19252	SS22-HEX	300	0.10	Pass	140.1		

Sample 53: SS08_SS10_SSS42_SS16_SS27_SS36_SS22_HCW4_E09.fsa

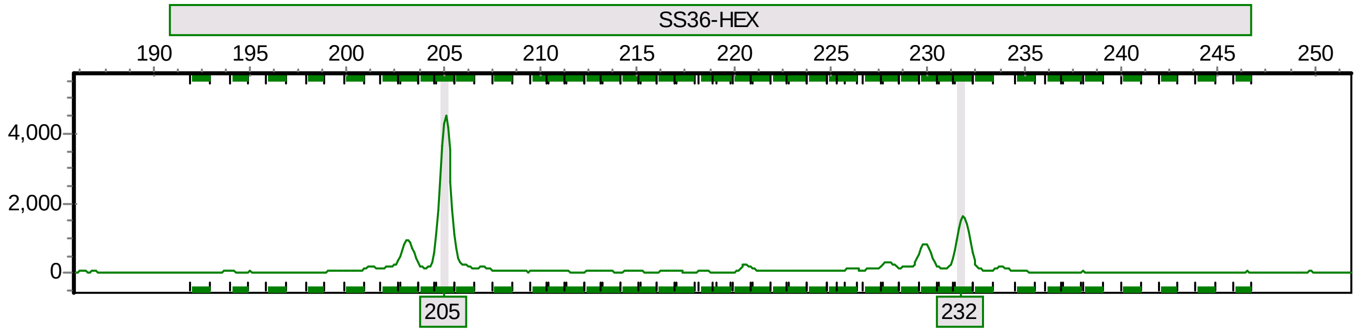

No	Size	Height	Area	Marker	Allele	Difference	Quality	Score	Allele Comments	Sample Comments
1	118.0	17941	123388	SS27-HEX	118	0.60	Pass	500.0	[<Confirmed>]	
2	205.1	4530	31259	SS36-HEX	205	0.00	Pass	500.0	[<Confirmed>]	
3	231.8	1628	12239	SS36-HEX	232	0.10	Pass	214.5	[<Confirmed>]	
4	297.9	5194	44993	SS22-HEX	298	0.10	Pass	500.0		
5	299.9	3045	27508	SS22-HEX	300	0.10	Pass	356.3		

Sample 54: SS08_SS10_SSS42_SS16_SS27_SS36_SS22_HCW5_C11.fsa

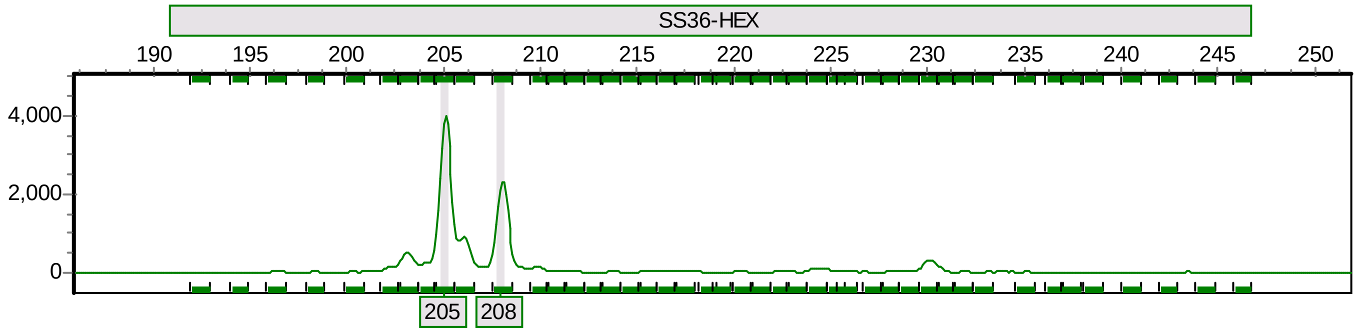

No	Size	Height	Area	Marker	Allele	Difference	Quality	Score	Allele Comments	Sample Comments
1	118.9	8108	57342	SS27-HEX	118	0.30	Pass	500.0	[<Confirmed>]	
2	205.1	3998	28353	SS36-HEX	205	0.00	Pass	500.0	[<Confirmed>]	

3	208.0	2323	16438	SS36-HEX	208	0.10	Pass	397.2	[<Confirmed>]	
4	294.1	3548	29742	SS22-HEX	294	0.10	Pass	498.0		
5	304.1	3367	31174	SS22-HEX	304	0.00	Pass	390.7		

Sample 55: SS08_SS10_SSS42_SS16_SS27_SS36_SS22_HCW6_D01.fsa

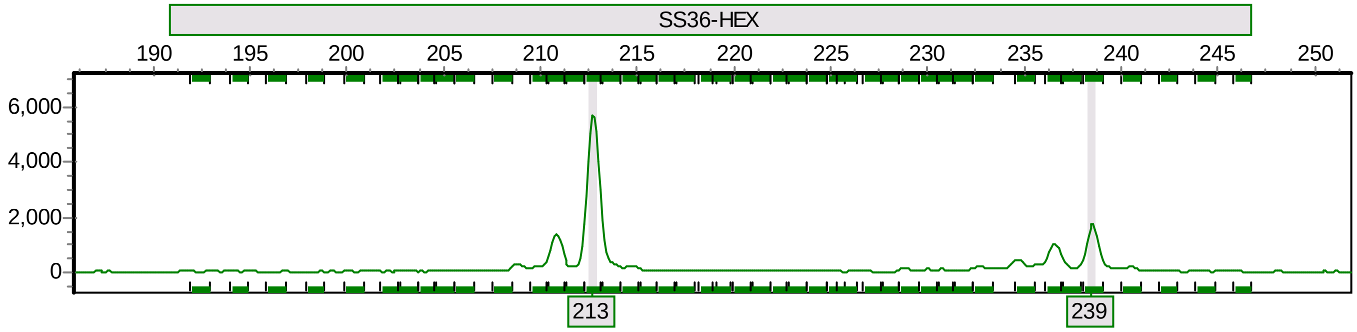

No	Size	Height	Area	Marker	Allele	Difference	Quality	Score	Allele Comments	Sample Comments
1	116.2	21899	140115	SS27-HEX	116	0.00	Pass	500.0	[<Confirmed>]	
2	212.7	5695	39957	SS36-HEX	213	0.10	Pass	500.0	[<Confirmed>]	
3	238.5	1757	13372	SS36-HEX	239	0.10	Pass	239.8	[<Confirmed>]	
4	296.2	6284	52687	SS22-HEX	296	0.10	Pass	500.0		
5	304.2	6066	54243	SS22-HEX	304	0.10	Pass	500.0		

Sample 56: SS08_SS10_SSS42_SS16_SS27_SS36_SS22_HCW7_G17.fsa

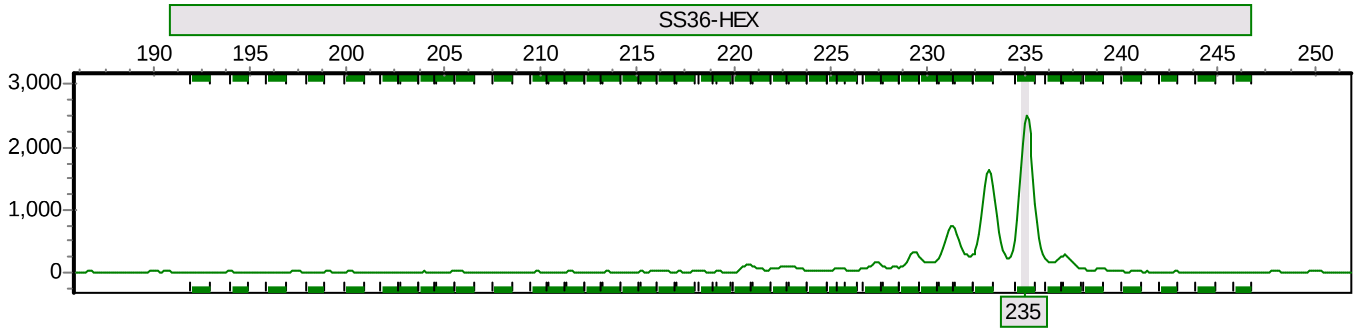

No	Size	Height	Area	Marker	Allele	Difference	Quality	Score	Allele Comments	Sample Comments
1	114.3	4523	30472	SS27-HEX	114	0.10	Pass	500.0	[<Deleted>]	
2	115.3	9907	70602	SS27-HEX	116	1.00	Pass	500.0	[<Confirmed><Edited>]	
3	121.2	8923	60124	SS27-HEX	120	0.40	Pass	500.0	[<Confirmed>]	
4	235.1	2485	21210	SS36-HEX	235	0.00	Pass	310.4	[<Confirmed>]	
5	298.1	4296	39654	SS22-HEX	298	0.10	Pass	500.0		
6	314.6	1656	16531	SS22-HEX	314	0.00	Pass	113.0		

Sample 57: SS08_SS10_SSS42_SS16_SS27_SS36_SS22_HCW8_A17.fsa

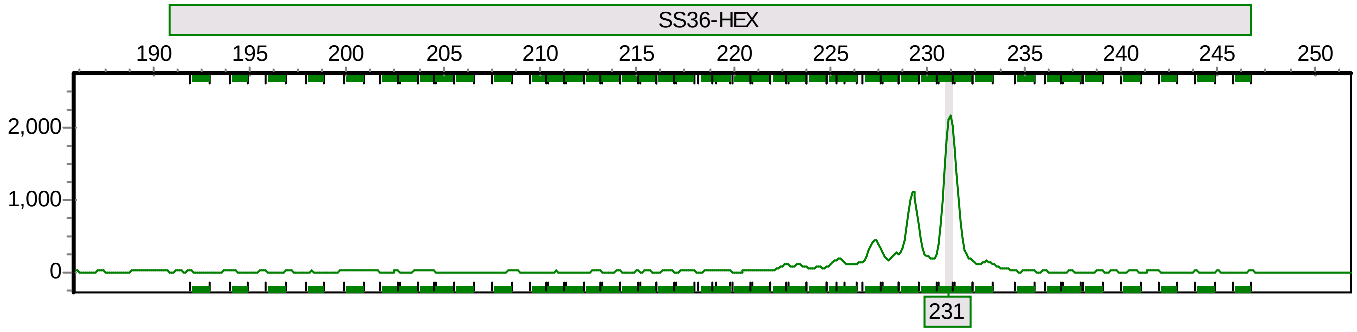

No	Size	Height	Area	Marker	Allele	Difference	Quality	Score	Allele Comments	Sample Comments
1	120.1	9207	60470	SS27-HEX	120	1.00	Pass	500.0	[<Confirmed><Edited>]	

2	121.2	7951	54311	SS27-HEX	120	0.40	Pass	500.0	[<Deleted>]	
3	231.2	2154	16054	SS36-HEX	231	0.20	Pass	314.4	[<Confirmed>]	
4	292.1	3426	29265	SS22-HEX	292	0.10	Pass	466.6		
5	304.0	1832	16988	SS22-HEX	304	0.10	Pass	164.3		

Sample 58: SS08_SS10_SSS42_SS16_SS27_SS36_SS22_HGC1_C01.fsa

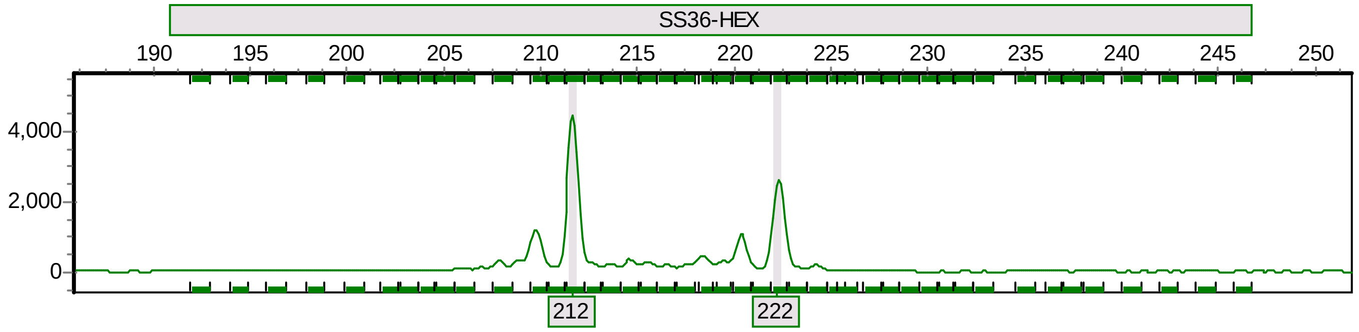

No	Size	Height	Area	Marker	Allele	Difference	Quality	Score	Allele Comments	Sample Comments
1	112.2	9351	57459	SS27-HEX	112	0.00	Pass	500.0	[<Deleted>]	
2	113.2	15643	99732	SS27-HEX	114	1.00	Pass	500.0	[<Confirmed><Edited>]	
3	133.1	5701	37081	SS27-HEX	132	1.00	Pass	500.0	[<Confirmed><Edited>]	
4	211.7	4407	29529	SS36-HEX	212	0.10	Pass	500.0	[<Confirmed>]	
5	222.3	2611	17583	SS36-HEX	222	0.10	Pass	500.0	[<Confirmed>]	
6	308.1	4182	37484	SS22-HEX	308	0.20	Pass	500.0		
7	312.2	2656	22704	SS22-HEX	312	0.10	Pass	333.2		

Sample 59: SS08_SS10_SSS42_SS16_SS27_SS36_SS22_HGC3_E17.fsa

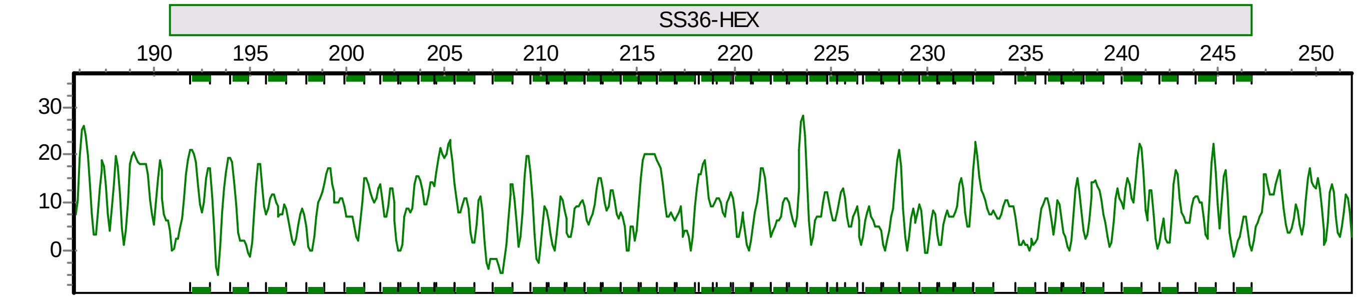

No	Size	Height	Area	Marker	Allele	Difference	Quality	Score	Allele Comments	Sample Comments

Sample 60: SS08_SS10_SSS42_SS16_SS27_SS36_SS22_HGC4_A13.fsa 2

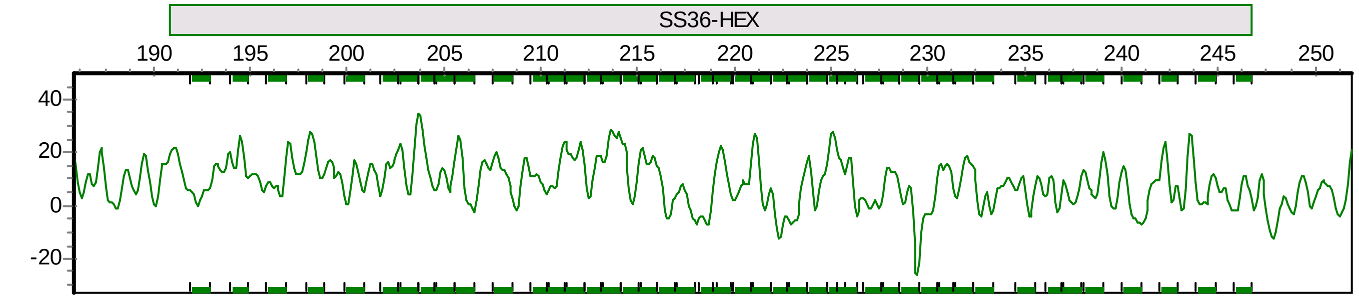

No	Size	Height	Area	Marker	Allele	Difference	Quality	Score	Allele Comments	Sample Comments

Sample 61: SS08_SS10_SSS42_SS16_SS27_SS36_SS22_HGY1_A09.fsa

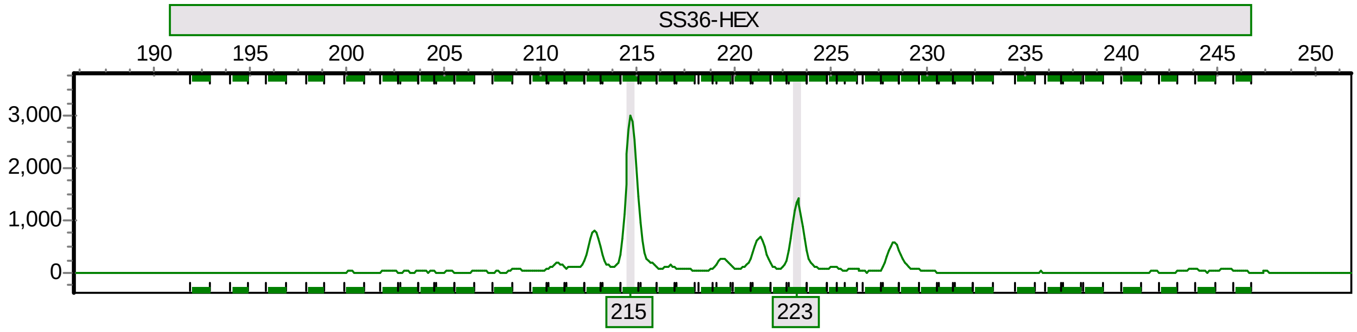

No	Size	Height	Area	Marker	Allele	Difference	Quality	Score	Allele Comments	Sample Comments
1	116.1	9456	64159	SS27-HEX	116	0.10	Pass	500.0	[<Confirmed>]	
2	118.1	6767	48056	SS27-HEX	118	0.50	Pass	500.0	[<Confirmed>]	
3	214.7	2994	21509	SS36-HEX	215	0.00	Pass	500.0	[<Confirmed>]	
4	223.3	1412	10431	SS36-HEX	223	0.00	Pass	174.0	[<Confirmed>]	
5	296.0	3811	33345	SS22-HEX	296	0.10	Pass	493.2		
6	299.9	2450	22399	SS22-HEX	300	0.10	Pass	250.6		

Sample 62: SS08_SS10_SSS42_SS16_SS27_SS36_SS22_HGY2_I13.fsa

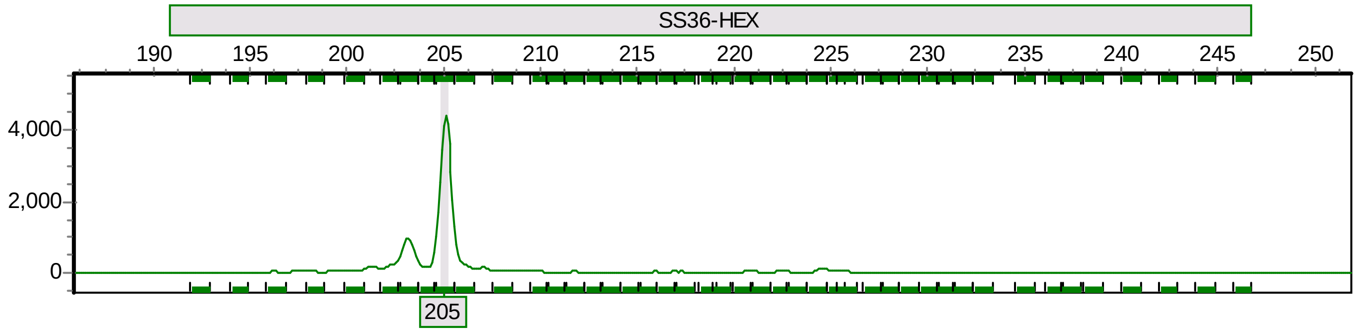

No	Size	Height	Area	Marker	Allele	Difference	Quality	Score	Allele Comments	Sample Comments
1	121.1	13126	92611	SS27-HEX	120	0.30	Pass	500.0	[<Confirmed>]	
2	205.1	4390	31276	SS36-HEX	205	0.00	Pass	500.0	[<Confirmed>]	
3	298.1	2546	23011	SS22-HEX	298	0.10	Pass	265.2		
4	300.0	1925	17588	SS22-HEX	300	0.00	Pass	187.7		

Sample 63: SS08_SS10_SSS42_SS16_SS27_SS36_SS22_HGY3_I11.fsa

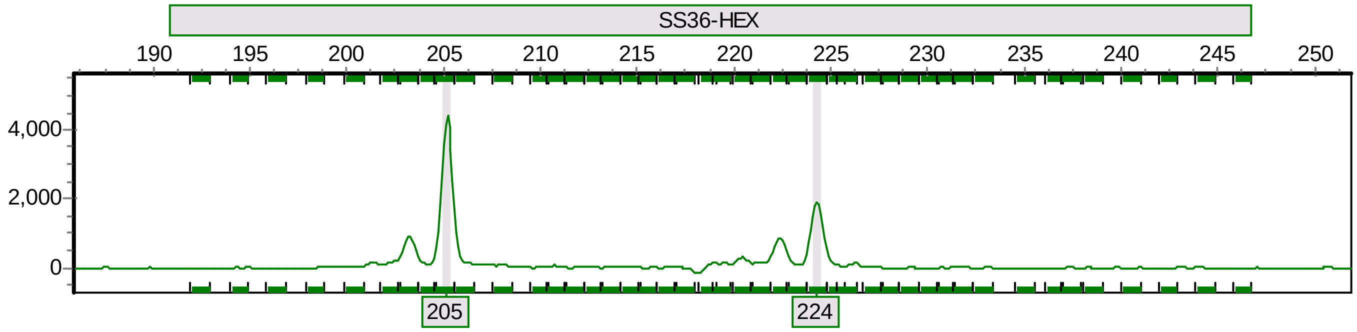

No	Size	Height	Area	Marker	Allele	Difference	Quality	Score	Allele Comments	Sample Comments
1	113.3	13133	89571	SS27-HEX	114	1.00	Pass	500.0	[<Confirmed><Edited>]	
2	125.3	6826	48007	SS27-HEX	126	0.10	Pass	500.0	[<Confirmed>]	
3	205.2	4385	30341	SS36-HEX	205	0.10	Pass	500.0	[<Confirmed>]	
4	224.3	1919	13524	SS36-HEX	224	0.00	Pass	305.7	[<Confirmed>]	
5	294.2	4040	34940	SS22-HEX	294	0.00	Pass	500.0		

6	300.0	2989	26006	SS22-HEX	300	0.00	Pass	380.0		

Sample 64: SS08_SS10_SSS42_SS16_SS27_SS36_SS22_HGY4_I09.fsa

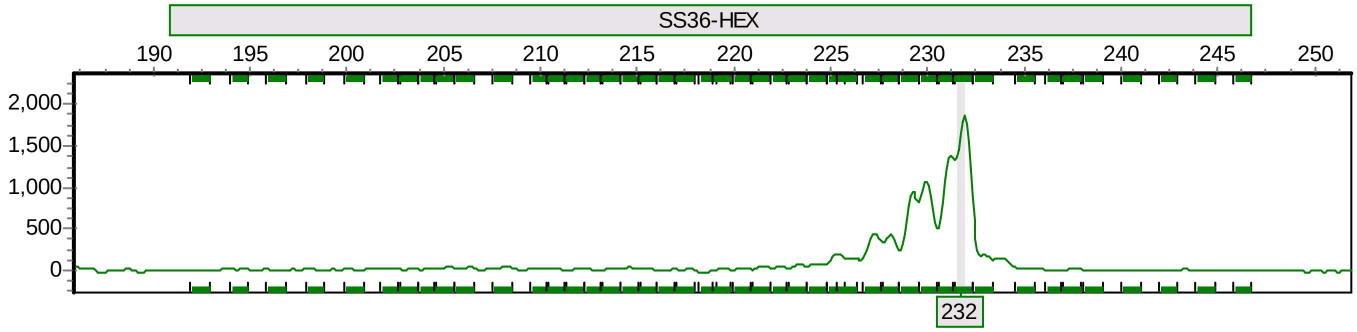

No	Size	Height	Area	Marker	Allele	Difference	Quality	Score	Allele Comments	Sample Comments
1	119.0	18828	125955	SS27-HEX	118	0.40	Pass	500.0	[<Confirmed>]	
2	231.8	1782	14363	SS36-HEX	232	0.10	Pass	96.5	[<Confirmed>]	
3	298.0	6148	50782	SS22-HEX	298	0.00	Pass	500.0		

Sample 65: SS08_SS10_SSS42_SS16_SS27_SS36_SS22_HGY5_I15.fsa

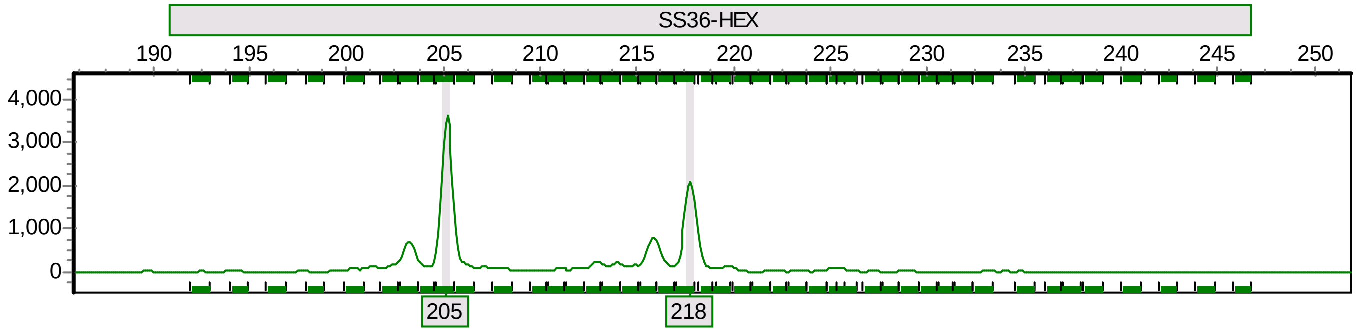

No	Size	Height	Area	Marker	Allele	Difference	Quality	Score	Allele Comments	Sample Comments
1	119.0	18134	126193	SS27-HEX	118	0.40	Pass	500.0	[<Confirmed>]	
2	205.2	3607	25157	SS36-HEX	205	0.10	Pass	500.0	[<Confirmed>]	
3	217.8	2090	15422	SS36-HEX	218	0.30	Pass	317.7	[<Confirmed>]	
4	286.4	4670	39627	SS22-HEX	286	0.00	Pass	500.0		
5	300.1	2323	21194	SS22-HEX	300	0.10	Pass	227.8		

Sample 66: SS08_SS10_SSS42_SS16_SS27_SS36_SS22_HQZ11_O11.fsa

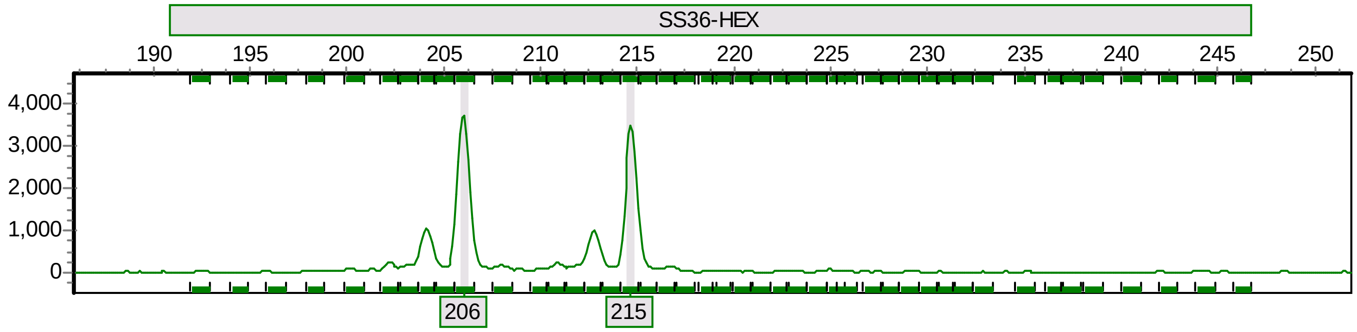

No	Size	Height	Area	Marker	Allele	Difference	Quality	Score	Allele Comments	Sample Comments
1	110.2	10704	72604	SS27-HEX	110	0.10	Pass	500.0	[<Confirmed>]	
2	119.1	6714	48096	SS27-HEX	118	0.50	Pass	500.0	[<Confirmed>]	
3	206.1	3700	26134	SS36-HEX	206	0.00	Pass	500.0	[<Confirmed>]	
4	214.7	3492	24440	SS36-HEX	215	0.00	Pass	500.0	[<Confirmed>]	
5	299.9	5963	55211	SS22-HEX	300	0.10	Pass	500.0		

Allele Report

Sample 67: SS08_SS10_SSS42_SS16_SS27_SS36_SS22_HQZ13-1_C09.fsa

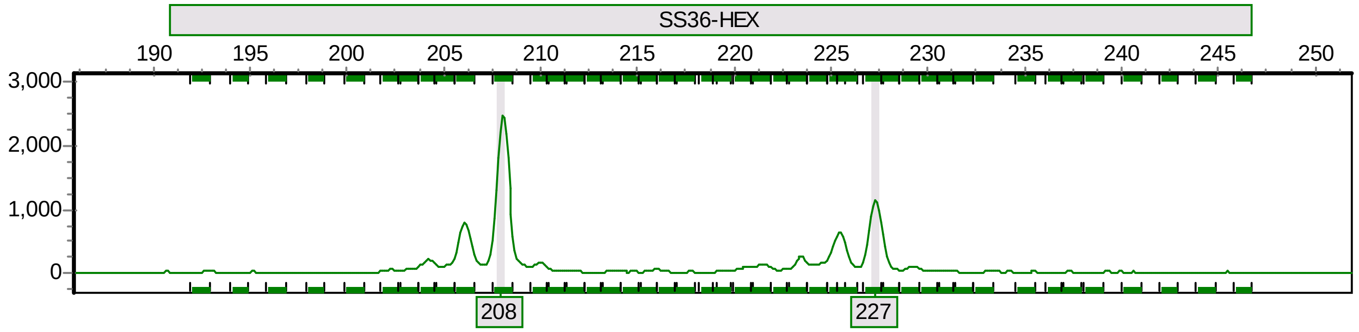

No	Size	Height	Area	Marker	Allele	Difference	Quality	Score	Allele Comments	Sample Comments
1	121.1	10923	76327	SS27-HEX	120	0.30	Pass	500.0	[<Confirmed>]	
2	208.0	2463	18158	SS36-HEX	208	0.10	Pass	403.1	[<Confirmed>]	
3	227.3	1140	8598	SS36-HEX	227	0.10	Pass	124.8	[<Confirmed>]	
4	294.0	2702	24498	SS22-HEX	294	0.20	Pass	305.7		
5	299.9	2272	21711	SS22-HEX	300	0.10	Pass	207.1		

Sample 68: SS08_SS10_SSS42_SS16_SS27_SS36_SS22_HQZ13-2_J03.fsa

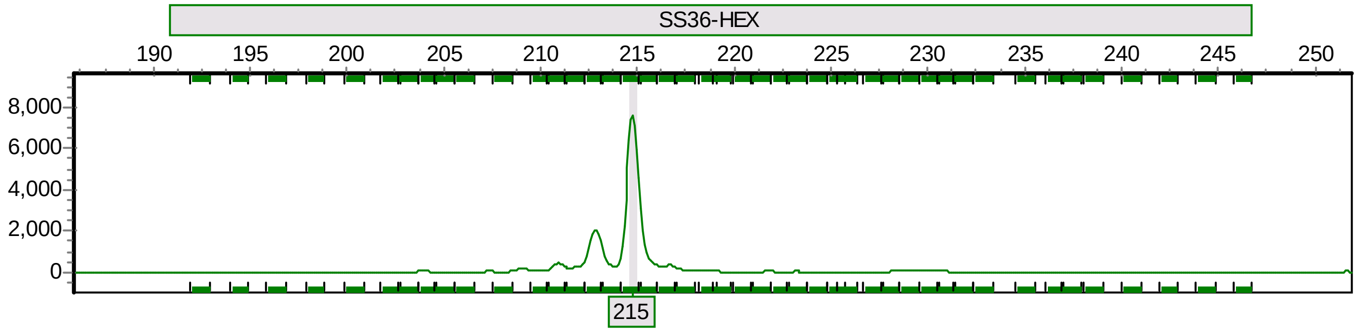

No	Size	Height	Area	Marker	Allele	Difference	Quality	Score	Allele Comments	Sample Comments
1	112.3	11651	78024	SS27-HEX	112	0.10	Pass	500.0	[<Confirmed>]	
2	114.2	8848	60240	SS27-HEX	114	0.00	Pass	500.0	[<Confirmed>]	
3	214.8	7541	55071	SS36-HEX	215	0.10	Pass	500.0	[<Confirmed>]	
4	304.1	8326	80148	SS22-HEX	304	0.00	Pass	500.0		

Sample 69: SS08_SS10_SSS42_SS16_SS27_SS36_SS22_HQZ14_H03.fsa

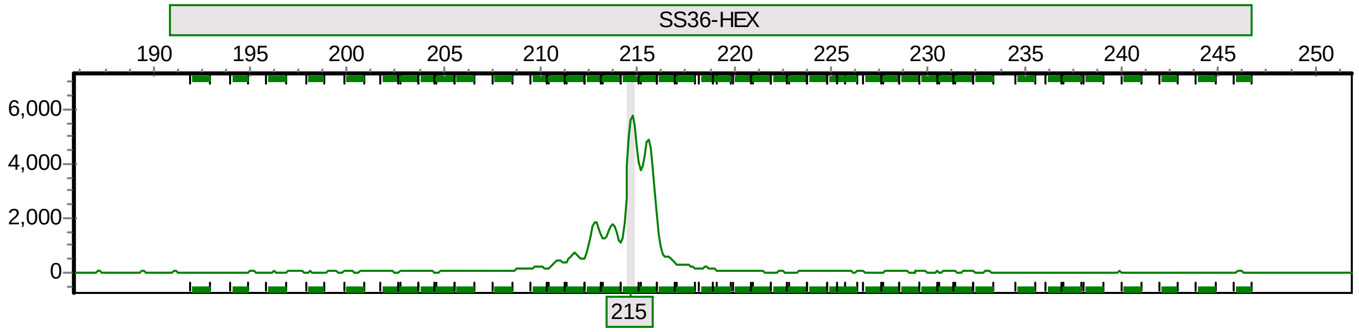

No	Size	Height	Area	Marker	Allele	Difference	Quality	Score	Allele Comments	Sample Comments
1	114.5	13415	88875	SS27-HEX	114	0.30	Pass	500.0	[<Confirmed>]	
2	118.4	10010	66474	SS27-HEX	118	0.20	Pass	500.0	[<Confirmed>]	
3	213.7	1787	11870	SS36-HEX	214	0.00	Pass	141.7	[<Deleted>]	
4	214.7	5666	75792	SS36-HEX	215	0.00	Pass	500.0	[<Confirmed>]	
5	290.4	9050	73727	SS22-HEX	290	0.10	Pass	500.0		
6	304.3	6324	57680	SS22-HEX	304	0.20	Pass	500.0		

Allele Report

Sample 70: SS08_SS10_SSS42_SS16_SS27_SS36_SS22_HQZ15_B01.fsa

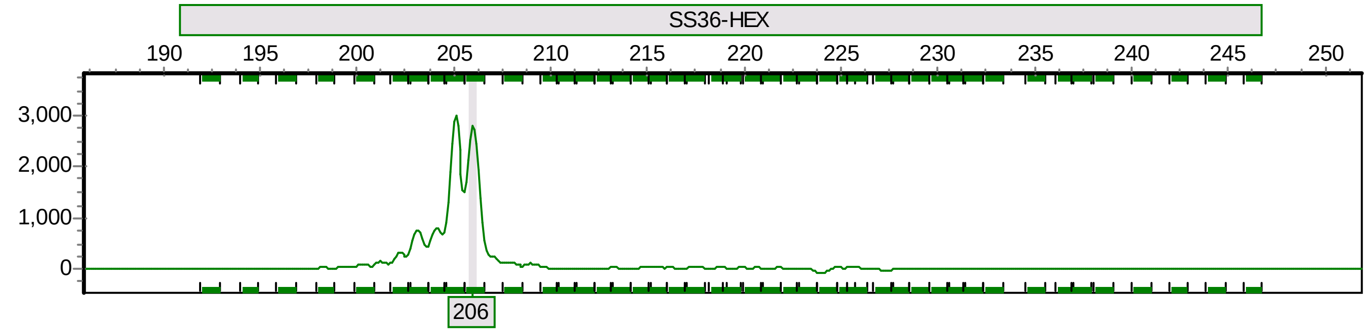

No	Size	Height	Area	Marker	Allele	Difference	Quality	Score	Allele Comments	Sample Comments
1	105.5	3694	23385	SS27-HEX	105	0.20	Undetermined	500.0	[<Deleted>]	
2	106.5	8628	58346	SS27-HEX	106	0.10	Pass	500.0	[<Confirmed>]	
3	112.4	6592	44843	SS27-HEX	112	0.20	Pass	500.0	[<Confirmed>]	
4	205.1	2987	20970	SS36-HEX	205	0.00	Pass	490.3	[<Deleted>]	
5	206.0	2787	18871	SS36-HEX	206	0.10	Pass	397.7	[<Confirmed>]	
6	298.1	4546	40554	SS22-HEX	298	0.10	Pass	500.0		
7	300.1	3280	29579	SS22-HEX	300	0.10	Pass	382.2		

Sample 71: SS08_SS10_SSS42_SS16_SS27_SS36_SS22_HQZ16_P01.fsa

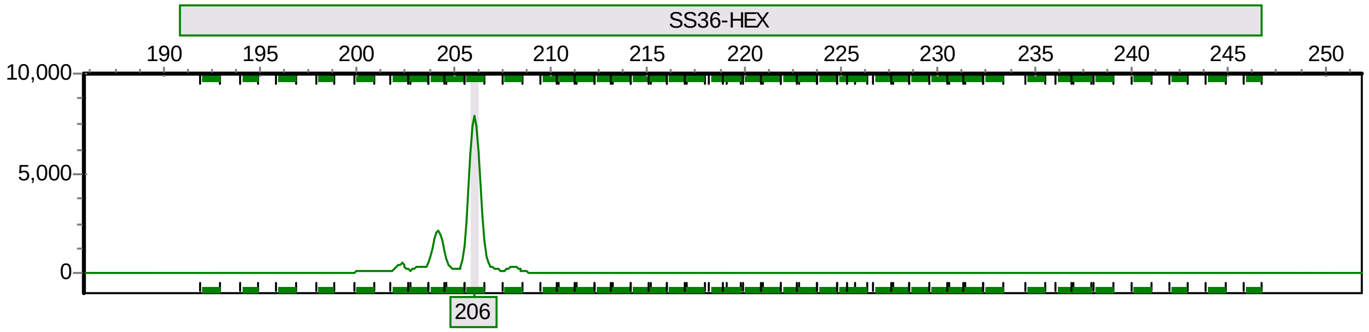

No	Size	Height	Area	Marker	Allele	Difference	Quality	Score	Allele Comments	Sample Comments
1	118.1	10420	68401	SS27-HEX	118	0.50	Pass	500.0	[<Confirmed>]	
2	206.1	7854	50215	SS36-HEX	206	0.00	Pass	500.0	[<Confirmed>]	
3	296.2	3731	29786	SS22-HEX	296	0.10	Pass	500.0		
4	304.1	4041	34809	SS22-HEX	304	0.00	Pass	500.0		

Sample 72: SS08_SS10_SSS42_SS16_SS27_SS36_SS22_HQZ17-1_O03.fsa

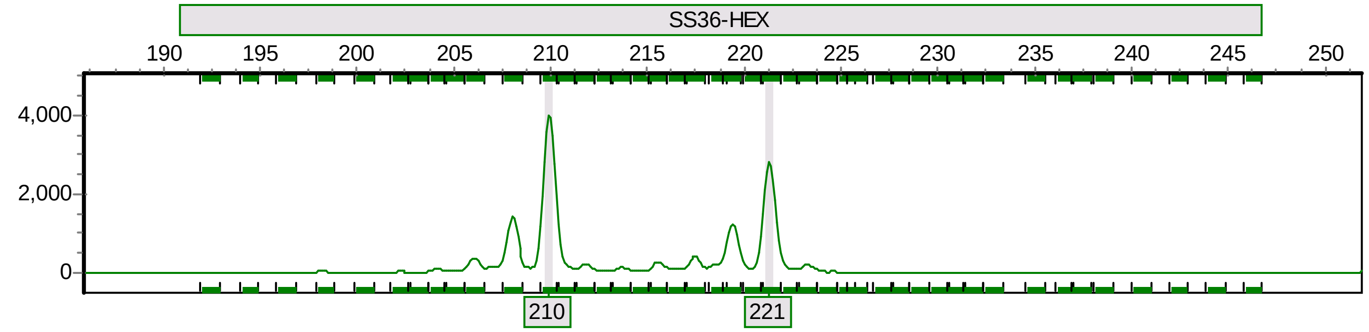

No	Size	Height	Area	Marker	Allele	Difference	Quality	Score	Allele Comments	Sample Comments
1	119.1	6984	46142	SS27-HEX	118	0.50	Pass	500.0	[<Deleted>]	
2	209.9	3987	27260	SS36-HEX	210	0.10	Pass	500.0	[<Confirmed>]	
3	221.3	2830	19438	SS36-HEX	221	0.10	Pass	500.0	[<Confirmed>]	
4	294.0	5226	42388	SS22-HEX	294	0.20	Pass	500.0		

5	304.0	5099	45701	SS22-HEX	304	0.10	Pass	500.0

Sample 73: SS08_SS10_SSS42_SS16_SS27_SS36_SS22_HQZ17-2_N03.fsa

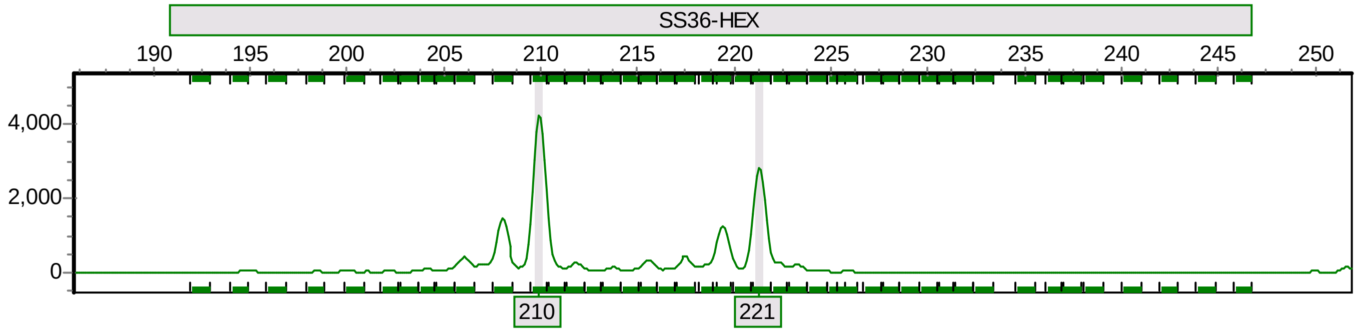

No	Size	Height	Area	Marker	Allele	Difference	Quality	Score	Allele Comments	Sample Comments
1	119.1	6038	40609	SS27-HEX	118	0.50	Pass	500.0	[<Deleted>]	
2	120.1	14989	102491	SS27-HEX	120	1.00	Pass	500.0	[<Confirmed><Edited>]	
3	209.9	4180	29766	SS36-HEX	210	0.10	Pass	500.0	[<Confirmed>]	
4	221.3	2810	20250	SS36-HEX	221	0.10	Pass	499.5	[<Confirmed>]	
5	294.2	5292	45651	SS22-HEX	294	0.00	Pass	500.0		
6	304.2	4897	46446	SS22-HEX	304	0.10	Pass	500.0		

Sample 74: SS08_SS10_SSS42_SS16_SS27_SS36_SS22_HQZ18_K09.fsa

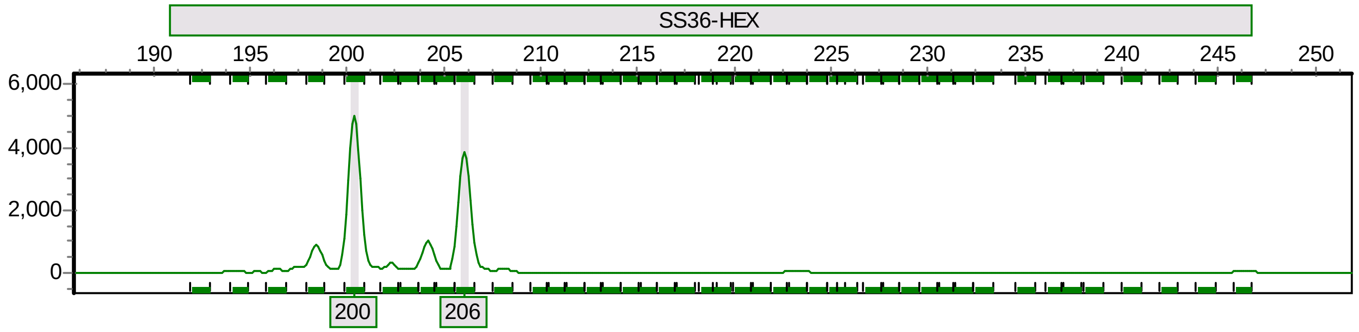

No	Size	Height	Area	Marker	Allele	Difference	Quality	Score	Allele Comments	Sample Comments
1	114.2	9869	65839	SS27-HEX	114	0.00	Pass	500.0	[<Confirmed>]	
2	119.1	6517	44197	SS27-HEX	118	0.50	Pass	500.0	[<Confirmed>]	
3	200.4	4973	33934	SS36-HEX	200	0.00	Pass	500.0	[<Confirmed>]	
4	206.1	3865	26273	SS36-HEX	206	0.00	Pass	500.0	[<Confirmed>]	
5	294.2	4379	37154	SS22-HEX	294	0.00	Pass	500.0		
6	300.0	3286	29041	SS22-HEX	300	0.00	Pass	411.2		

Sample 75: SS08_SS10_SSS42_SS16_SS27_SS36_SS22_HQZ19_L03.fsa

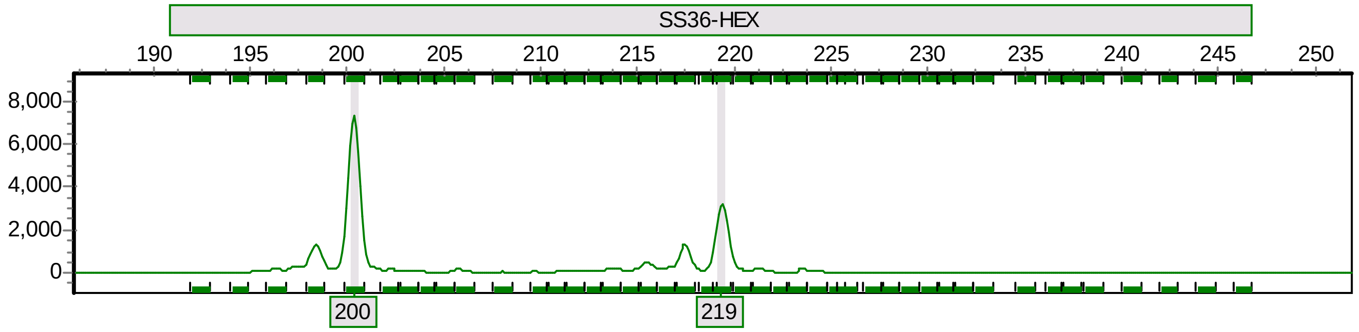

No	Size	Height	Area	Marker	Allele	Difference	Quality	Score	Allele Comments	Sample Comments
1	110.3	13387	89474	SS27-HEX	110	0.00	Pass	500.0	[<Confirmed>]	
2	116.2	9547	63934	SS27-HEX	116	0.00	Pass	500.0	[<Confirmed>]	

3	200.4	7283	48906	SS36-HEX	200	0.00	Pass	500.0	[<Confirmed>]	
4	219.4	3161	22860	SS36-HEX	219	0.00	Pass	500.0	[<Confirmed>]	
5	296.2	4835	41840	SS22-HEX	296	0.10	Pass	500.0		
6	304.2	5080	47017	SS22-HEX	304	0.10	Pass	500.0		

Sample 76: SS08_SS10_SSS42_SS16_SS27_SS36_SS22_HQZ21_B03.fsa

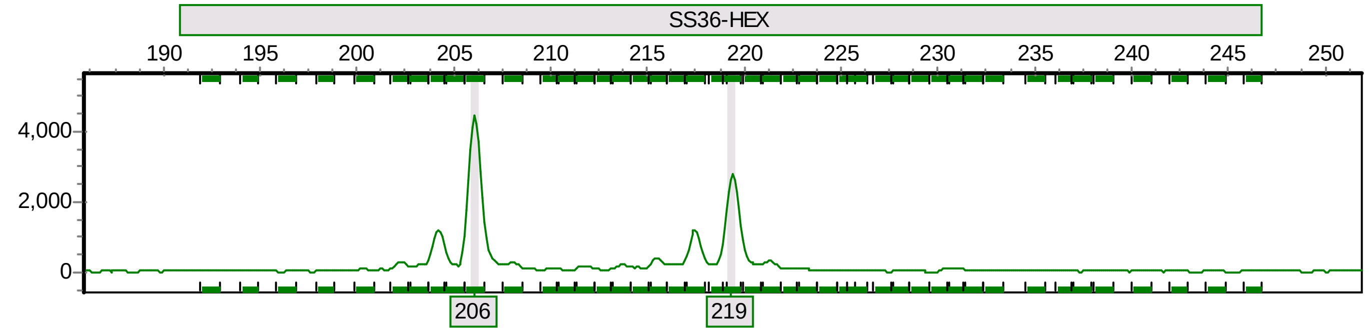

No	Size	Height	Area	Marker	Allele	Difference	Quality	Score	Allele Comments	Sample Comments
1	110.2	21732	144571	SS27-HEX	110	0.10	Pass	500.0	[<Confirmed>]	
2	206.1	4424	32020	SS36-HEX	206	0.00	Pass	500.0	[<Confirmed>]	
3	219.4	2766	20915	SS36-HEX	219	0.00	Pass	458.5	[<Confirmed>]	
4	296.1	5390	47003	SS22-HEX	296	0.00	Pass	500.0		
5	312.4	3717	36523	SS22-HEX	312	0.10	Pass	398.0		

Sample 77: SS08_SS10_SSS42_SS16_SS27_SS36_SS22_HQZ22-1_M01.fsa

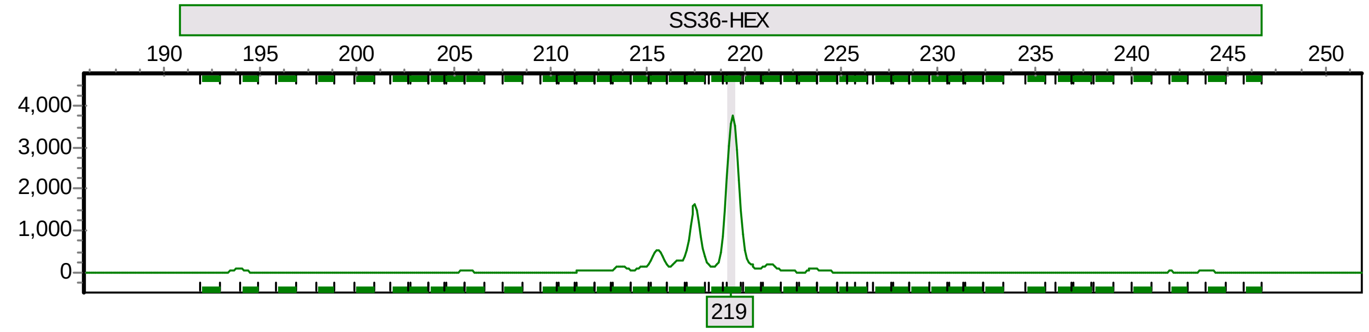

No	Size	Height	Area	Marker	Allele	Difference	Quality	Score	Allele Comments	Sample Comments
1	116.2	7832	53042	SS27-HEX	116	0.00	Pass	500.0	[<Confirmed>]	
2	119.1	6224	40608	SS27-HEX	118	0.50	Pass	500.0	[<Confirmed>]	
3	219.4	3733	25663	SS36-HEX	219	0.00	Pass	500.0	[<Confirmed>]	
4	296.0	3447	28921	SS22-HEX	296	0.10	Pass	500.0		
5	299.8	2473	20834	SS22-HEX	300	0.20	Pass	305.7		

Sample 78: SS08_SS10_SSS42_SS16_SS27_SS36_SS22_HQZ22-2_O13.fsa

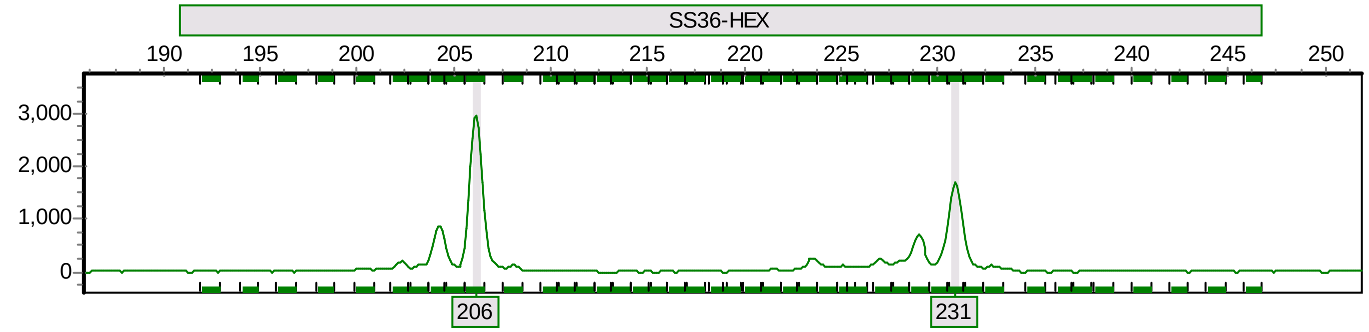

No	Size	Height	Area	Marker	Allele	Difference	Quality	Score	Allele Comments	Sample Comments
1	110.3	9131	61793	SS27-HEX	110	0.00	Pass	500.0	[<Confirmed>]	

2	119.1	5538	38613	SS27-HEX	118	0.50	Pass	500.0	[<Confirmed>]	
3	206.2	2928	20955	SS36-HEX	206	0.10	Pass	500.0	[<Confirmed>]	
4	230.9	1682	13290	SS36-HEX	231	0.10	Pass	199.1	[<Confirmed>]	
5	299.9	4783	44239	SS22-HEX	300	0.10	Pass	500.0		

Sample 79: SS08_SS10_SSS42_SS16_SS27_SS36_SS22_HQZ23_A15.fsa

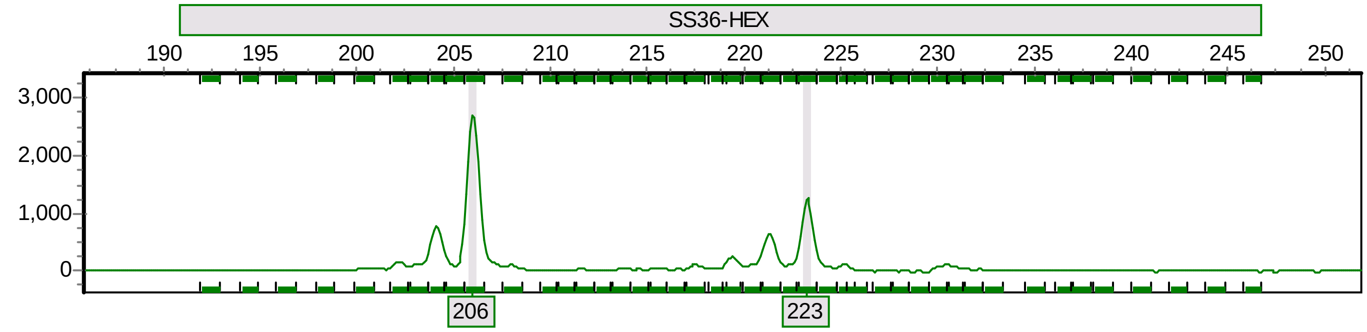

No	Size	Height	Area	Marker	Allele	Difference	Quality	Score	Allele Comments	Sample Comments
1	110.2	8324	55031	SS27-HEX	110	0.10	Pass	500.0	[<Confirmed>]	
2	122.1	4962	33820	SS27-HEX	122	1.00	Pass	500.0	[<Confirmed><Edited>]	
3	206.0	2674	18688	SS36-HEX	206	0.10	Pass	497.1	[<Confirmed>]	
4	223.3	1261	9328	SS36-HEX	223	0.00	Pass	151.3	[<Confirmed>]	
5	300.0	2773	25048	SS22-HEX	300	0.00	Pass	289.4		
6	304.1	3051	28012	SS22-HEX	304	0.00	Pass	350.3		

Sample 80: SS08_SS10_SSS42_SS16_SS27_SS36_SS22_HQZ24_E11.fsa

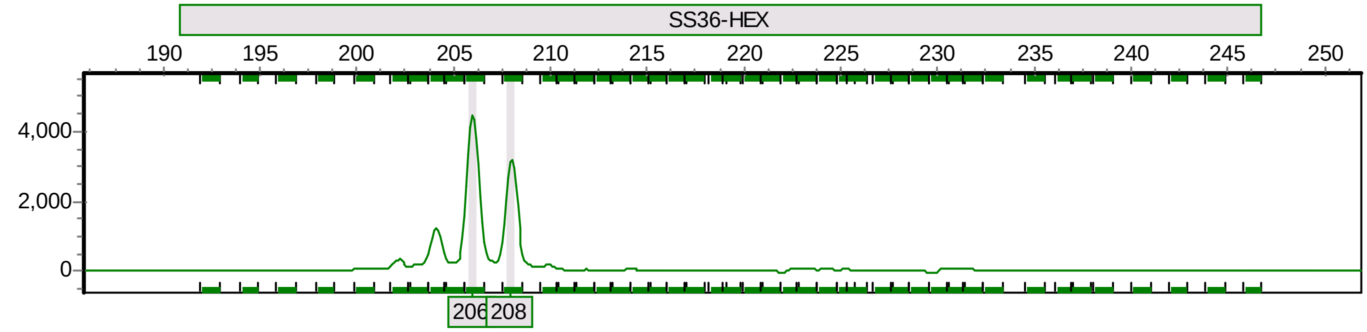

No	Size	Height	Area	Marker	Allele	Difference	Quality	Score	Allele Comments	Sample Comments
1	114.1	9225	63142	SS27-HEX	114	0.10	Pass	500.0	[<Confirmed>]	
2	119.1	5942	41497	SS27-HEX	118	0.50	Pass	500.0	[<Confirmed>]	
3	206.0	4438	31718	SS36-HEX	206	0.10	Pass	500.0	[<Confirmed>]	
4	208.0	3208	22522	SS36-HEX	208	0.10	Pass	500.0	[<Confirmed>]	
5	290.2	5763	47952	SS22-HEX	290	0.10	Pass	500.0		
6	304.1	3972	36738	SS22-HEX	304	0.00	Pass	484.6		

Sample 81: SS08_SS10_SSS42_SS16_SS27_SS36_SS22_HQZ25_N01.fsa

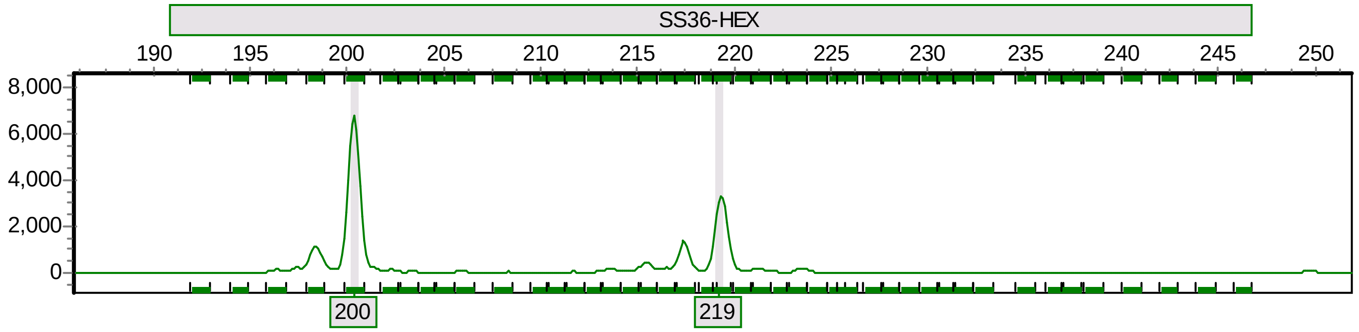

No	Size	Height	Area	Marker	Allele	Difference	Quality	Score	Allele Comments	Sample Comments
1	116.2	11299	76268	SS27-HEX	116	0.00	Pass	500.0	[<Confirmed>]	
2	119.1	7981	52066	SS27-HEX	118	0.50	Pass	500.0	[<Confirmed>]	
3	200.4	6732	44704	SS36-HEX	200	0.00	Pass	500.0	[<Confirmed>]	
4	219.3	3331	23350	SS36-HEX	219	0.10	Pass	500.0	[<Confirmed>]	
5	299.8	8993	76552	SS22-HEX	300	0.20	Pass	500.0		

Sample 82: SS08_SS10_SSS42_SS16_SS27_SS36_SS22_HQZ26_E03.fsa

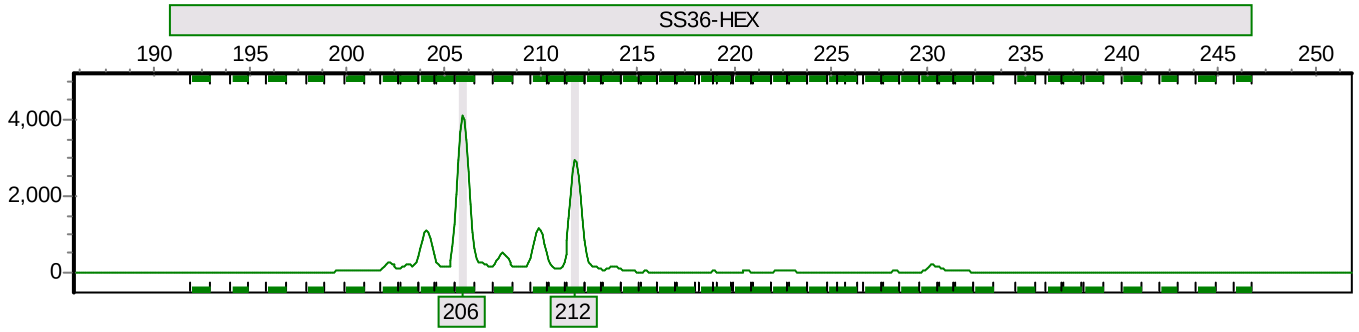

No	Size	Height	Area	Marker	Allele	Difference	Quality	Score	Allele Comments	Sample Comments
1	114.2	14374	93995	SS27-HEX	114	0.00	Pass	500.0	[<Confirmed>]	
2	116.1	9161	61536	SS27-HEX	116	0.10	Pass	500.0	[<Confirmed>]	
3	206.0	4090	27182	SS36-HEX	206	0.10	Pass	500.0	[<Confirmed>]	
4	211.8	2931	19885	SS36-HEX	212	0.00	Pass	500.0	[<Confirmed>]	
5	294.1	5393	43171	SS22-HEX	294	0.10	Pass	500.0		
6	299.8	4016	32674	SS22-HEX	300	0.20	Pass	500.0		

Sample 83: SS08_SS10_SSS42_SS16_SS27_SS36_SS22_HQZ27_G09.fsa

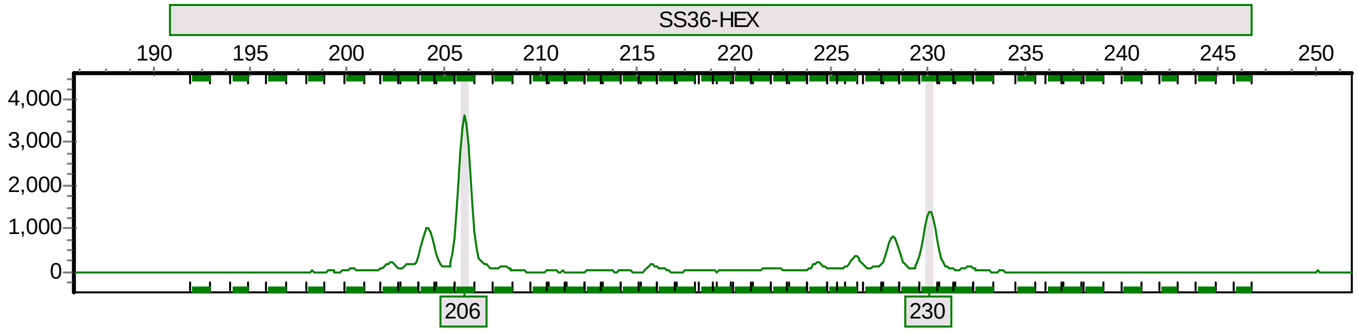

No	Size	Height	Area	Marker	Allele	Difference	Quality	Score	Allele Comments	Sample Comments
1	114.3	6126	41260	SS27-HEX	114	0.10	Pass	500.0	[<Deleted>]	
2	115.3	15783	105961	SS27-HEX	116	1.00	Pass	500.0	[<Confirmed><Edited>]	
3	206.1	3593	24719	SS36-HEX	206	0.00	Pass	500.0	[<Confirmed>]	
4	230.1	1396	10641	SS36-HEX	230	0.00	Pass	166.9	[<Confirmed>]	

5	304.1	4391	40540	SS22-HEX	304	0.00	Pass	500.0		
6	312.4	2251	20827	SS22-HEX	312	0.10	Pass	218.3		

Sample 84: SS08_SS10_SSS42_SS16_SS27_SS36_SS22_HQZ28_B05.fsa

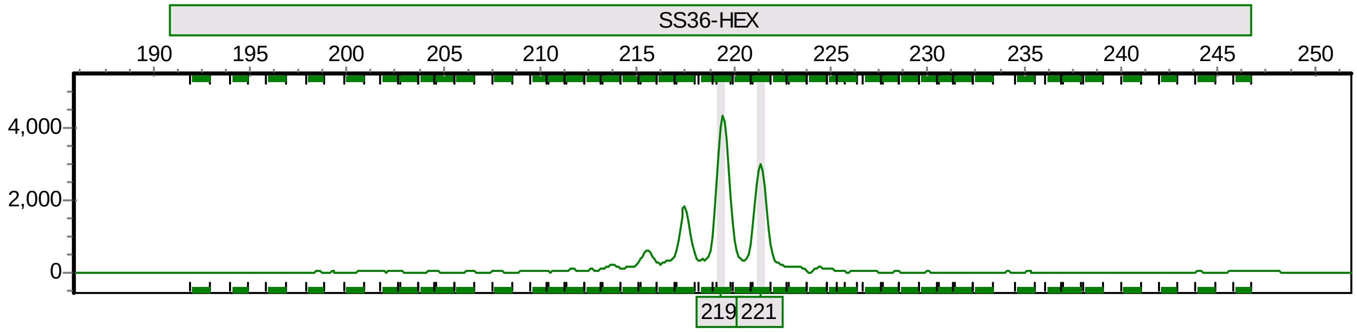

No	Size	Height	Area	Marker	Allele	Difference	Quality	Score	Allele Comments	Sample Comments
1	110.2	11460	75828	SS27-HEX	110	0.10	Pass	500.0	[<Confirmed>]	
2	118.1	7594	51910	SS27-HEX	118	0.50	Pass	500.0	[<Confirmed>]	
3	219.4	4296	31166	SS36-HEX	219	0.00	Pass	500.0	[<Confirmed>]	
4	221.4	2998	21100	SS36-HEX	221	0.00	Pass	500.0	[<Confirmed>]	
5	296.2	6157	51121	SS22-HEX	296	0.10	Pass	500.0		
6	310.5	2894	26865	SS22-HEX	310	0.20	Pass	290.6		

Sample 85: SS08_SS10_SSS42_SS16_SS27_SS36_SS22_HQZ29_C13.fsa

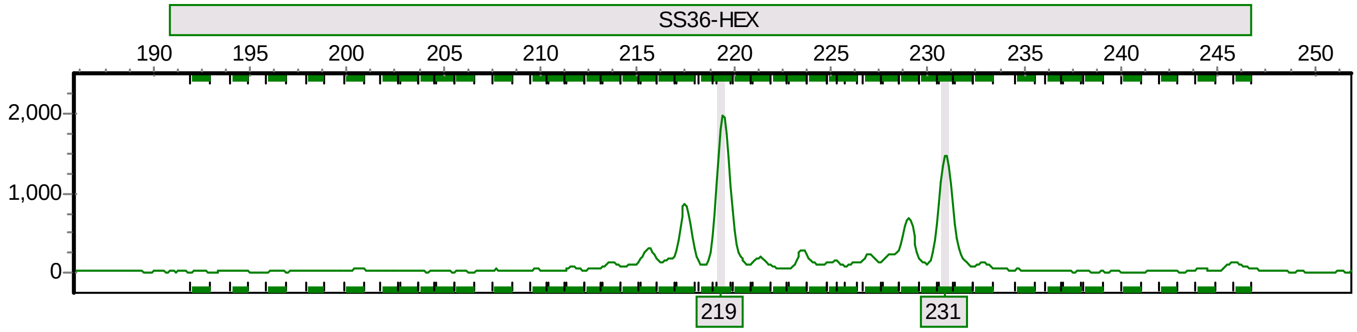

No	Size	Height	Area	Marker	Allele	Difference	Quality	Score	Allele Comments	Sample Comments
1	110.2	8879	61735	SS27-HEX	110	0.10	Pass	500.0	[<Confirmed>]	
2	119.1	5078	35234	SS27-HEX	118	0.50	Pass	500.0	[<Confirmed>]	
3	219.4	1975	15137	SS36-HEX	219	0.00	Pass	278.8	[<Confirmed>]	
4	230.9	1480	11906	SS36-HEX	231	0.10	Pass	166.1	[<Confirmed>]	
5	299.8	5866	53288	SS22-HEX	300	0.20	Pass	500.0		

Sample 86: SS08_SS10_SSS42_SS16_SS27_SS36_SS22_HQZ2_K03.fsa

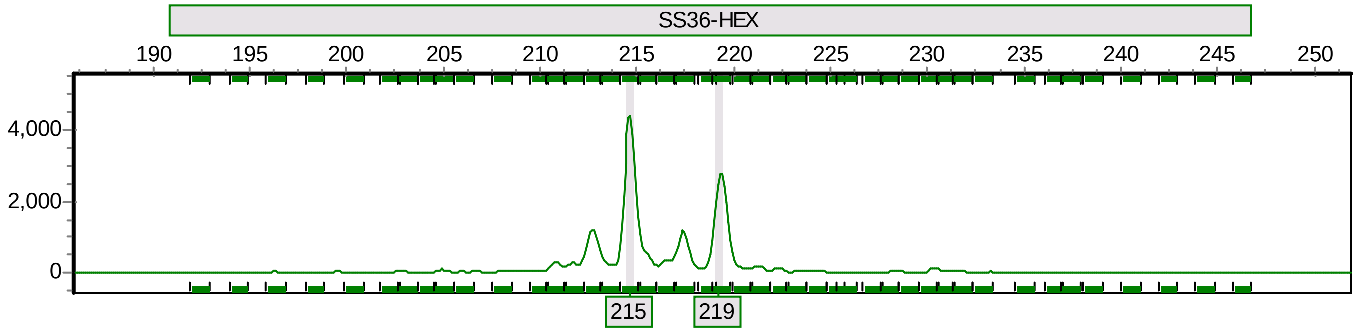

No	Size	Height	Area	Marker	Allele	Difference	Quality	Score	Allele Comments	Sample Comments
1	110.2	13404	91722	SS27-HEX	110	0.10	Pass	500.0	[<Confirmed>]	
2	119.1	7561	50019	SS27-HEX	118	0.50	Pass	500.0	[<Confirmed>]	

3	214.7	4397	30131	SS36-HEX	215	0.00	Pass	500.0	[<Confirmed>]	
4	219.3	2786	19678	SS36-HEX	219	0.10	Pass	500.0	[<Confirmed>]	
5	294.1	4980	41158	SS22-HEX	294	0.10	Pass	500.0		
6	312.3	3212	28930	SS22-HEX	312	0.00	Pass	391.3		

Sample 87: SS08_SS10_SSS42_SS16_SS27_SS36_SS22_HQZ30_G15.fsa

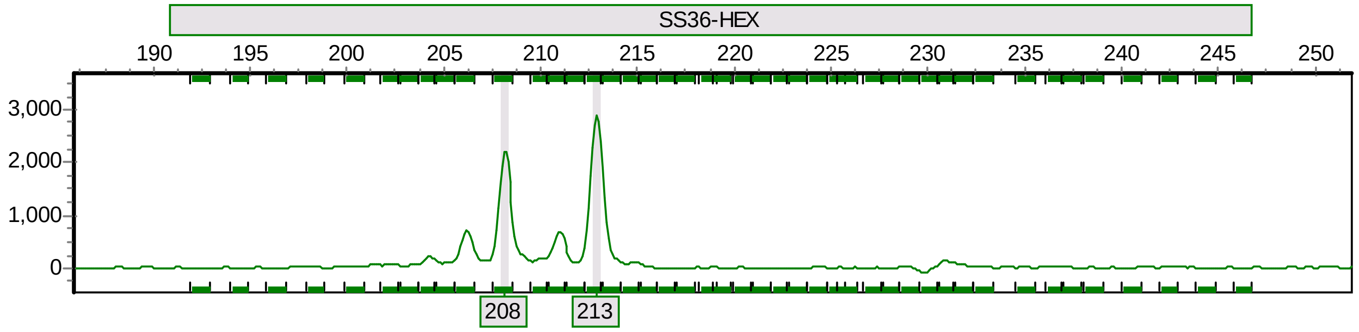

No	Size	Height	Area	Marker	Allele	Difference	Quality	Score	Allele Comments	Sample Comments
1	119.0	7225	47954	SS27-HEX	118	0.40	Pass	500.0	[<Confirmed>]	
2	208.2	2203	16497	SS36-HEX	208	0.10	Pass	334.2	[<Confirmed>]	
3	212.9	2879	20911	SS36-HEX	213	0.10	Pass	500.0	[<Confirmed>]	
4	299.9	2875	27319	SS22-HEX	300	0.10	Pass	306.7		
5	304.1	3182	30466	SS22-HEX	304	0.00	Pass	327.2		

Sample 88: SS08_SS10_SSS42_SS16_SS27_SS36_SS22_HQZ31_K13.fsa

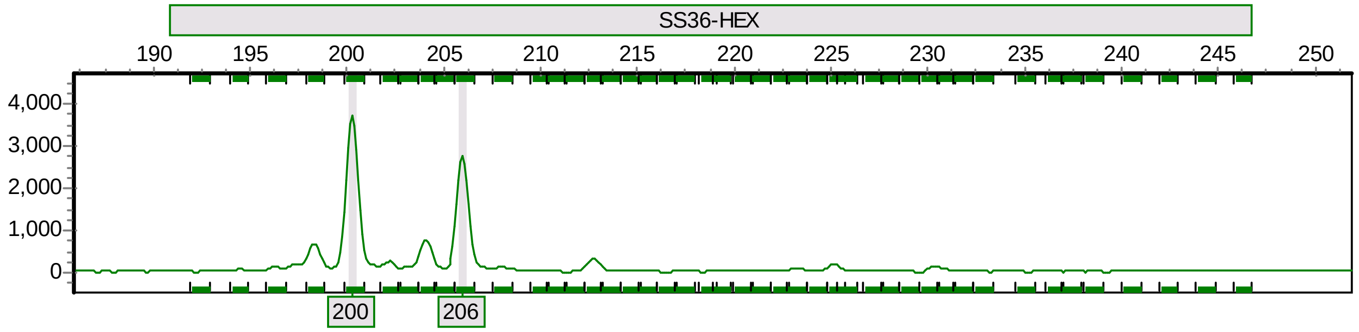

No	Size	Height	Area	Marker	Allele	Difference	Quality	Score	Allele Comments	Sample Comments
1	118.1	7427	50080	SS27-HEX	118	0.50	Pass	500.0	[<Confirmed>]	
2	122.1	5028	34884	SS27-HEX	122	1.00	Pass	500.0	[<Confirmed><Edited>]	
3	200.3	3689	25016	SS36-HEX	200	0.10	Pass	500.0	[<Confirmed>]	
4	206.0	2740	18664	SS36-HEX	206	0.10	Pass	500.0	[<Confirmed>]	
5	299.8	4758	42549	SS22-HEX	300	0.20	Pass	500.0		

Sample 89: SS08_SS10_SSS42_SS16_SS27_SS36_SS22_HQZ32_J01.fsa

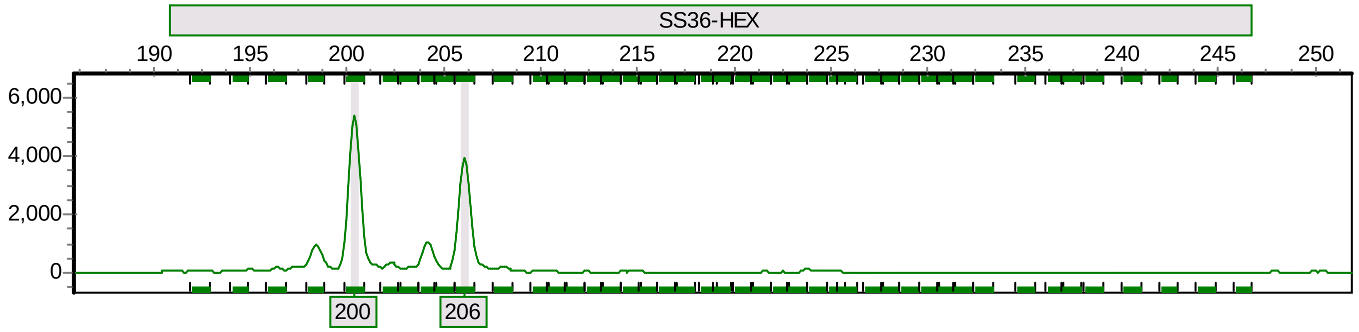

No	Size	Height	Area	Marker	Allele	Difference	Quality	Score	Allele Comments	Sample Comments
1	110.4	11690	75281	SS27-HEX	110	0.10	Pass	500.0	[<Confirmed>]	

2	120.3	7292	49494	SS27-HEX	120	0.50	Pass	500.0	[<Confirmed>]	
3	200.4	5376	35486	SS36-HEX	200	0.00	Pass	500.0	[<Confirmed>]	
4	206.1	3944	25740	SS36-HEX	206	0.00	Pass	500.0	[<Confirmed>]	
5	296.2	4427	36177	SS22-HEX	296	0.10	Pass	500.0		
6	312.4	3119	27586	SS22-HEX	312	0.10	Pass	393.9		

Sample 90: SS08_SS10_SSS42_SS16_SS27_SS36_SS22_HQZ33_I03.fsa

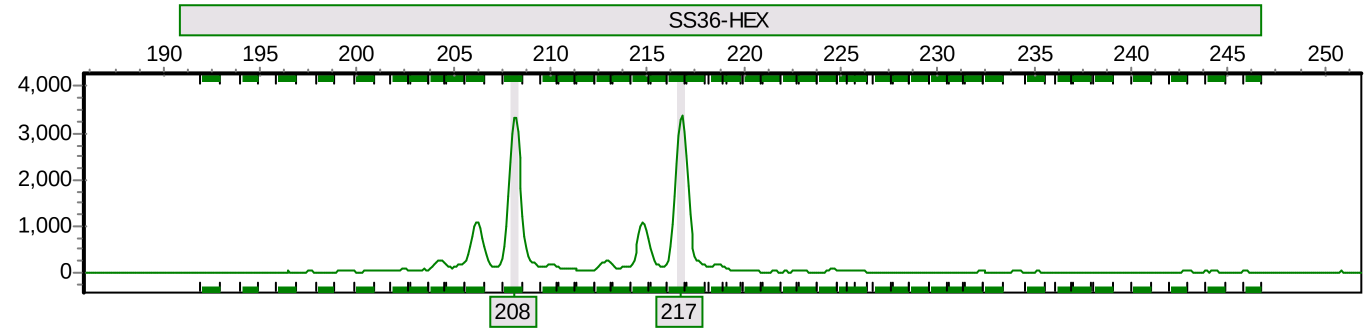

No	Size	Height	Area	Marker	Allele	Difference	Quality	Score	Allele Comments	Sample Comments
1	114.2	9615	64398	SS27-HEX	114	0.00	Pass	500.0	[<Confirmed>]	
2	119.1	6665	46156	SS27-HEX	118	0.50	Pass	500.0	[<Confirmed>]	
3	208.2	3344	23962	SS36-HEX	208	0.10	Pass	500.0	[<Confirmed>]	
4	216.8	3350	24245	SS36-HEX	217	0.20	Pass	500.0	[<Confirmed>]	
5	299.9	3834	34802	SS22-HEX	300	0.10	Pass	500.0		
6	304.2	2687	24529	SS22-HEX	304	0.10	Pass	288.2		

Sample 91: SS08_SS10_SSS42_SS16_SS27_SS36_SS22_HQZ34_M03.fsa

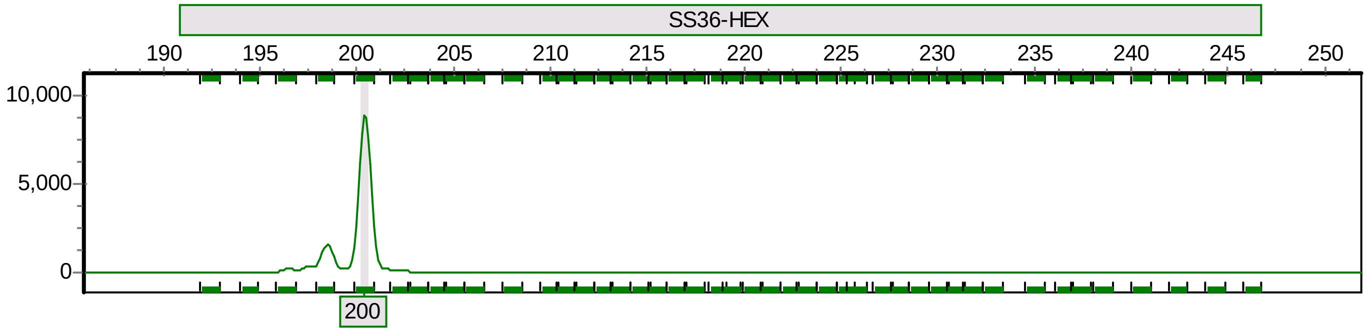

No	Size	Height	Area	Marker	Allele	Difference	Quality	Score	Allele Comments	Sample Comments
1	112.2	11245	72704	SS27-HEX	112	0.00	Pass	500.0	[<Confirmed>]	
2	116.2	8381	56998	SS27-HEX	116	0.00	Pass	500.0	[<Confirmed>]	
3	200.4	8820	59827	SS36-HEX	200	0.00	Pass	500.0	[<Confirmed>]	
4	296.1	3293	27786	SS22-HEX	296	0.00	Pass	455.2		
5	304.0	3193	29195	SS22-HEX	304	0.10	Pass	373.9		

Sample 92: SS08_SS10_SSS42_SS16_SS27_SS36_SS22_HQZ35_A01.fsa

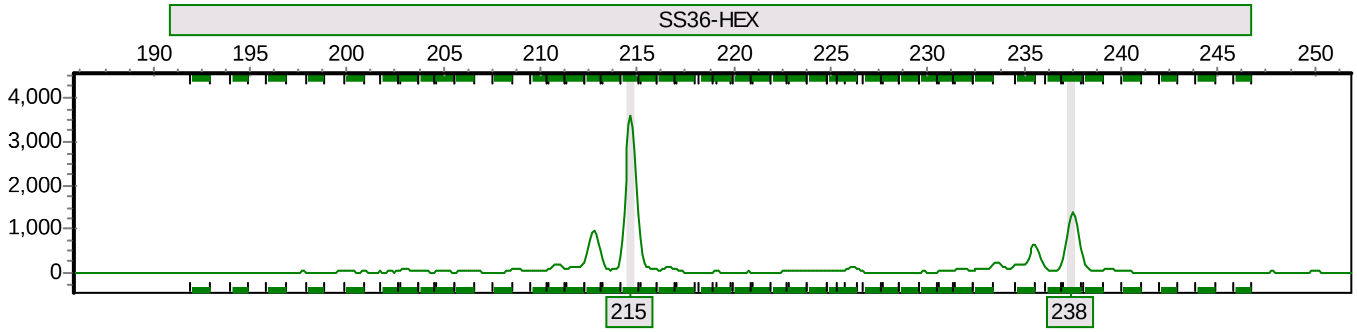

No	Size	Height	Area	Marker	Allele	Difference	Quality	Score	Allele Comments	Sample Comments
1	118.2	6313	41118	SS27-HEX	118	0.40	Pass	500.0	[<Confirmed>]	
2	121.2	3117	19559	SS27-HEX	120	0.40	Pass	500.0	[<Deleted>]	
3	122.2	4388	29133	SS27-HEX	122	1.00	Pass	500.0	[<Confirmed><Edited>]	
4	214.7	3571	23653	SS36-HEX	215	0.00	Pass	500.0	[<Confirmed>]	
5	237.5	1374	9610	SS36-HEX	238	0.00	Pass	189.6	[<Confirmed>]	
6	299.8	2715	22685	SS22-HEX	300	0.20	Pass	368.0		
7	304.0	3019	26355	SS22-HEX	304	0.10	Pass	369.6		

Sample 93: SS08_SS10_SSS42_SS16_SS27_SS36_SS22_HQZ36_E15.fsa

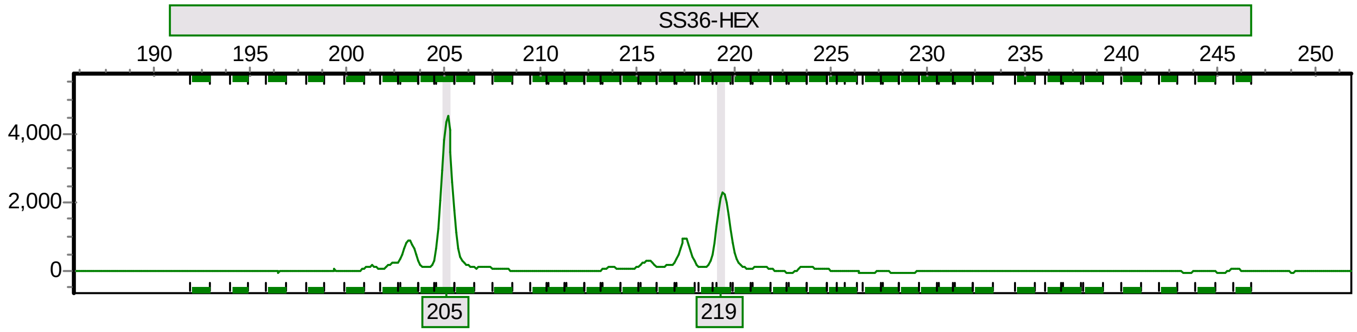

No	Size	Height	Area	Marker	Allele	Difference	Quality	Score	Allele Comments	Sample Comments
1	110.1	10393	70686	SS27-HEX	110	0.20	Pass	500.0	[<Confirmed>]	
2	114.1	8007	53574	SS27-HEX	114	0.10	Pass	500.0	[<Confirmed>]	
3	205.2	4523	32158	SS36-HEX	205	0.10	Pass	500.0	[<Confirmed>]	
4	219.4	2311	17311	SS36-HEX	219	0.00	Pass	356.9	[<Confirmed>]	
5	296.1	8358	74499	SS22-HEX	296	0.00	Pass	500.0		

Sample 94: SS08_SS10_SSS42_SS16_SS27_SS36_SS22_HQZ37_F03.fsa

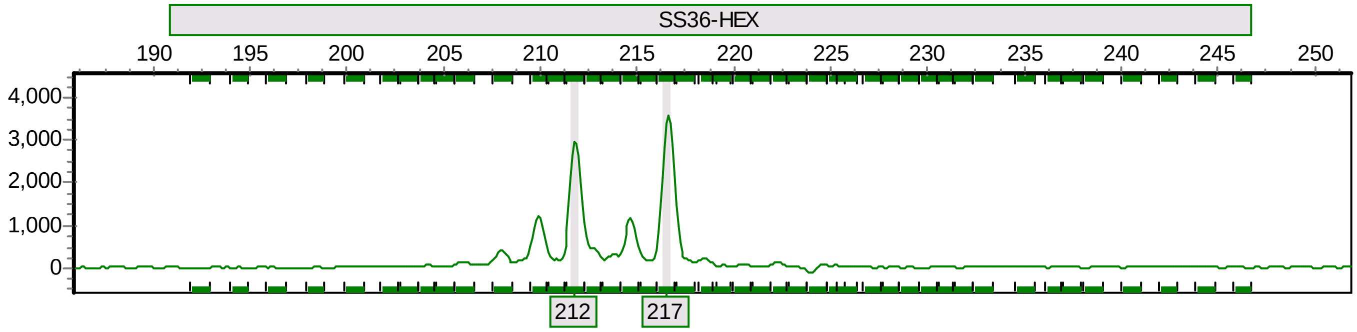

No	Size	Height	Area	Marker	Allele	Difference	Quality	Score	Allele Comments	Sample Comments
1	118.3	12105	83370	SS27-HEX	118	0.30	Pass	500.0	[<Confirmed>]	
2	120.2	4316	28560	SS27-HEX	120	0.60	Pass	500.0	[<Deleted>]	
3	211.8	2974	21250	SS36-HEX	212	0.00	Pass	500.0	[<Confirmed>]	

4	216.6	3556	24836	SS36-HEX	217	0.00	Pass	500.0	[<Confirmed>]	
5	294.2	5894	49024	SS22-HEX	294	0.00	Pass	500.0		
6	304.3	3619	32261	SS22-HEX	304	0.20	Pass	467.9		

Sample 95: SS08_SS10_SSS42_SS16_SS27_SS36_SS22_HQZ38_L01.fsa

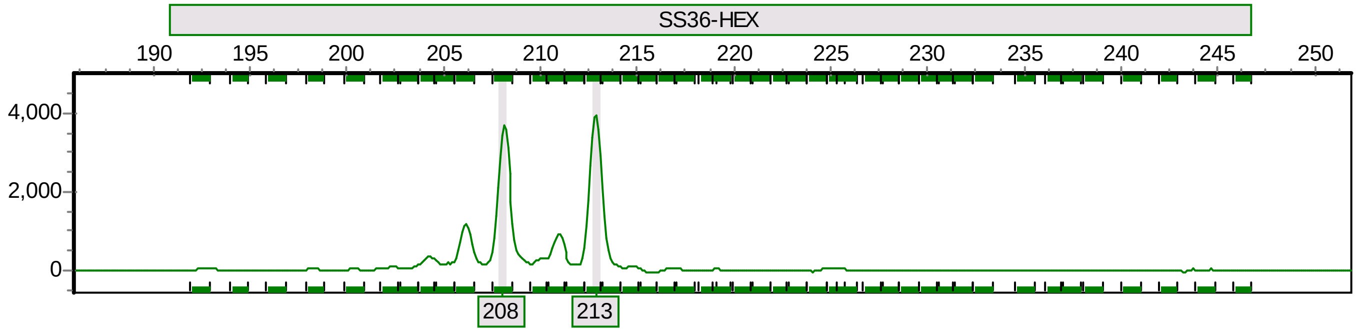

No	Size	Height	Area	Marker	Allele	Difference	Quality	Score	Allele Comments	Sample Comments
1	114.3	9221	60817	SS27-HEX	114	0.10	Pass	500.0	[<Confirmed>]	
2	118.3	6537	45408	SS27-HEX	118	0.30	Pass	500.0	[<Confirmed>]	
3	208.1	3683	26589	SS36-HEX	208	0.00	Pass	500.0	[<Confirmed>]	
4	212.9	3913	27336	SS36-HEX	213	0.10	Pass	500.0	[<Confirmed>]	
5	294.2	5782	51396	SS22-HEX	294	0.00	Pass	500.0		
6	296.1	4489	38082	SS22-HEX	296	0.00	Pass	500.0		

Sample 96: SS08_SS10_SSS42_SS16_SS27_SS36_SS22_HQZ39_M15.fsa

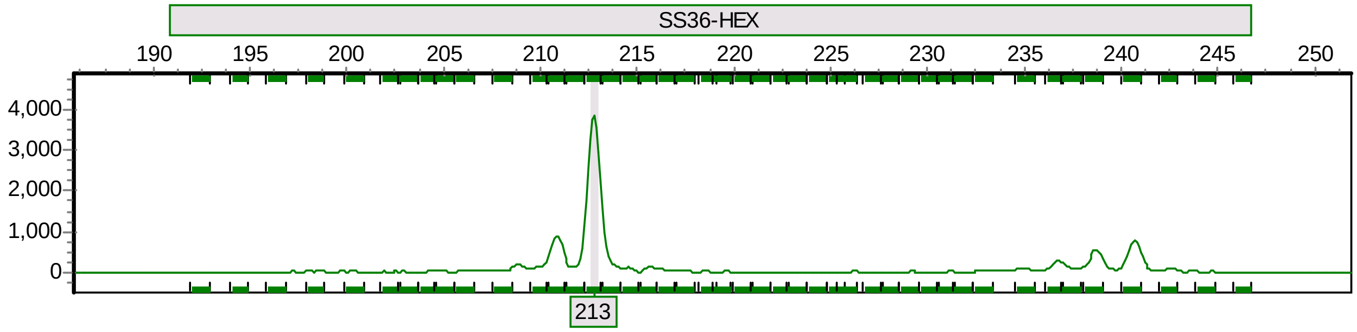

No	Size	Height	Area	Marker	Allele	Difference	Quality	Score	Allele Comments	Sample Comments
1	116.1	13476	92357	SS27-HEX	116	0.10	Pass	500.0	[<Confirmed>]	
2	212.8	3813	27690	SS36-HEX	213	0.00	Pass	500.0	[<Confirmed>]	
3	290.2	4441	39401	SS22-HEX	290	0.10	Pass	500.0		
4	310.3	1693	16633	SS22-HEX	310	0.00	Pass	127.0		

Sample 97: SS08_SS10_SSS42_SS16_SS27_SS36_SS22_HQZ7_C03.fsa

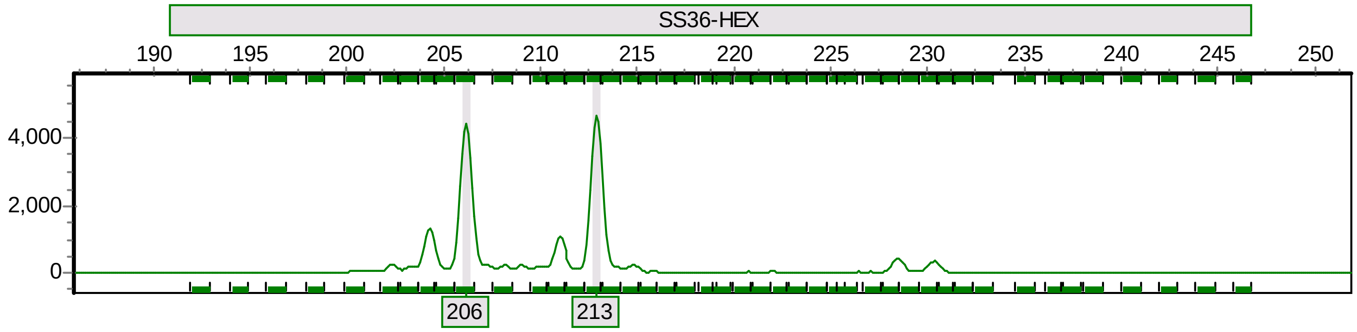

No	Size	Height	Area	Marker	Allele	Difference	Quality	Score	Allele Comments	Sample Comments
1	110.3	13134	87445	SS27-HEX	110	0.00	Pass	500.0	[<Confirmed>]	
2	116.3	9441	64605	SS27-HEX	116	0.10	Pass	500.0	[<Confirmed>]	

3	206.2	4430	29451	SS36-HEX	206	0.10	Pass	500.0	[<Confirmed>]	
4	212.9	4620	30746	SS36-HEX	213	0.10	Pass	500.0	[<Confirmed>]	
5	298.1	5219	42290	SS22-HEX	298	0.10	Pass	500.0		
6	306.1	3330	28792	SS22-HEX	306	0.10	Pass	444.4		

Sample 98: SS08_SS10_SSS42_SS16_SS27_SS36_SS22_HQZ9_K11.fsa

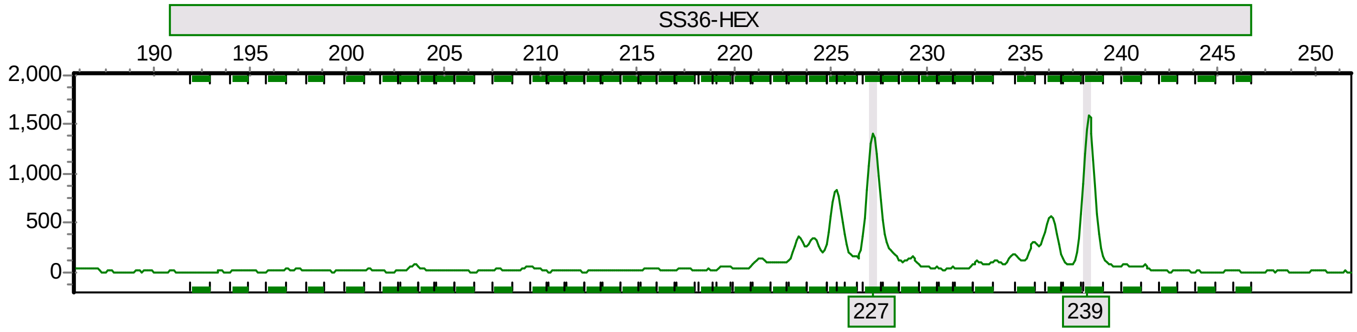

No	Size	Height	Area	Marker	Allele	Difference	Quality	Score	Allele Comments	Sample Comments
1	123.2	10782	72315	SS27-HEX	122	0.20	Pass	500.0	[<Confirmed>]	
2	125.2	7827	53815	SS27-HEX	126	0.00	Pass	500.0	[<Confirmed>]	
3	227.2	1399	10698	SS36-HEX	227	0.00	Pass	170.8	[<Confirmed>]	
4	238.3	1583	11950	SS36-HEX	239	0.30	Pass	204.1	[<Confirmed>]	
5	296.0	5647	47301	SS22-HEX	296	0.10	Pass	500.0		

Sample 99: SS08_SS10_SSS42_SS16_SS27_SS36_SS22_HRS24_I17.fsa

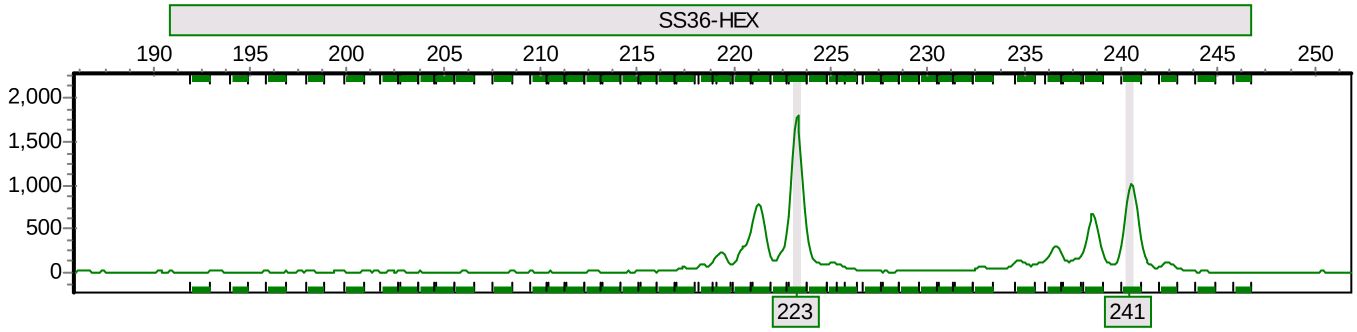

No	Size	Height	Area	Marker	Allele	Difference	Quality	Score	Allele Comments	Sample Comments
1	112.1	13355	93410	SS27-HEX	112	0.10	Pass	500.0	[<Confirmed>]	
2	126.1	5885	40601	SS27-HEX	126	1.00	Pass	500.0	[<Confirmed><Edited>]	
3	223.3	1792	13962	SS36-HEX	223	0.00	Pass	235.5	[<Confirmed>]	
4	240.5	1011	8120	SS36-HEX	241	0.10	Pass	93.4	[<Confirmed>]	
5	299.9	2561	23767	SS22-HEX	300	0.10	Pass	267.0		

Sample 100: SS08_SS10_SSS42_SS16_SS27_SS36_SS22_HRS26_H09.fsa

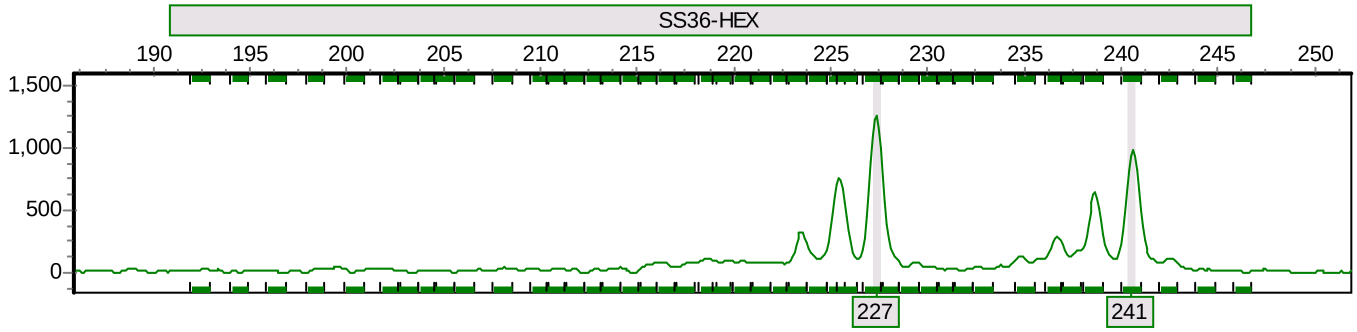

No	Size	Height	Area	Marker	Allele	Difference	Quality	Score	Allele Comments	Sample Comments
1	125.1	9793	70263	SS27-HEX	126	0.10	Pass	500.0	[<Confirmed>]	

2	227.4	1256	9646	SS36-HEX	227	0.20	Pass	134.2	[<Confirmed>]	
3	240.6	985	8014	SS36-HEX	241	0.00	Pass	90.5	[<Confirmed>]	
4	298.2	4807	43825	SS22-HEX	298	0.20	Pass	500.0		
5	300.1	2946	28612	SS22-HEX	300	0.10	Pass	292.4		

Sample 101: SS08_SS10_SSS42_SS16_SS27_SS36_SS22_HRS28_D13.fsa

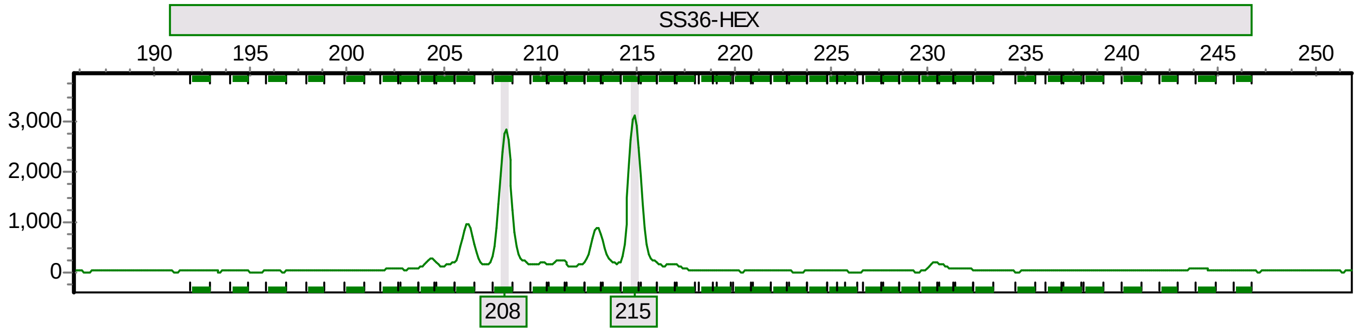

No	Size	Height	Area	Marker	Allele	Difference	Quality	Score	Allele Comments	Sample Comments
1	114.3	7389	50802	SS27-HEX	114	0.10	Pass	500.0	[<Confirmed>]	
2	118.2	5624	39310	SS27-HEX	118	0.40	Pass	500.0	[<Confirmed>]	
3	208.2	2807	20901	SS36-HEX	208	0.10	Pass	472.5	[<Confirmed>]	
4	214.9	3086	22905	SS36-HEX	215	0.20	Pass	500.0	[<Confirmed>]	
5	298.2	3528	32298	SS22-HEX	298	0.20	Pass	403.1		
6	304.2	3956	38189	SS22-HEX	304	0.10	Pass	451.1		

Sample 102: SS08_SS10_SSS42_SS16_SS27_SS36_SS22_HRS29_J05.fsa

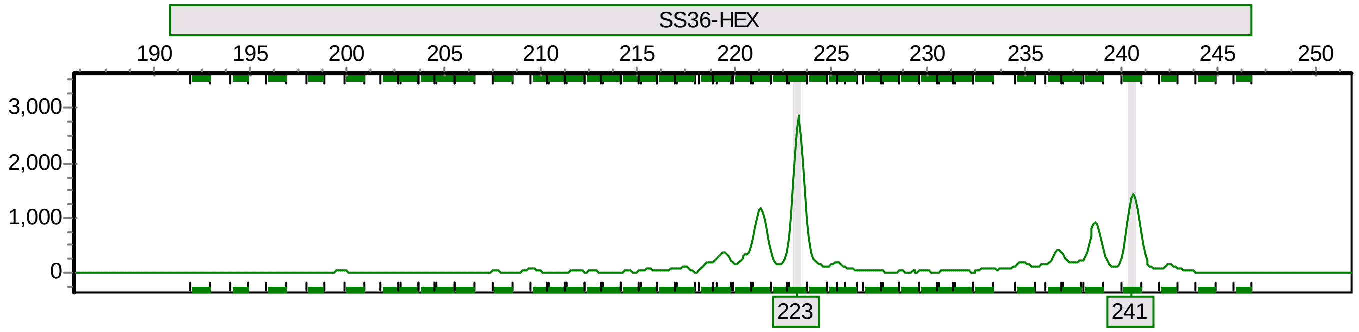

No	Size	Height	Area	Marker	Allele	Difference	Quality	Score	Allele Comments	Sample Comments
1	123.3	15991	110814	SS27-HEX	122	0.30	Pass	500.0	[<Confirmed>]	
2	125.3	10313	71051	SS27-HEX	126	0.10	Pass	500.0	[<Confirmed>]	
3	223.3	2845	20873	SS36-HEX	223	0.00	Pass	496.5	[<Confirmed>]	
4	240.6	1426	11147	SS36-HEX	241	0.00	Pass	165.0	[<Confirmed>]	
5	298.2	6500	57343	SS22-HEX	298	0.20	Pass	500.0		
6	300.1	3867	36370	SS22-HEX	300	0.10	Pass	475.6		

Sample 103: SS08_SS10_SSS42_SS16_SS27_SS36_SS22_HRS30_P05.fsa

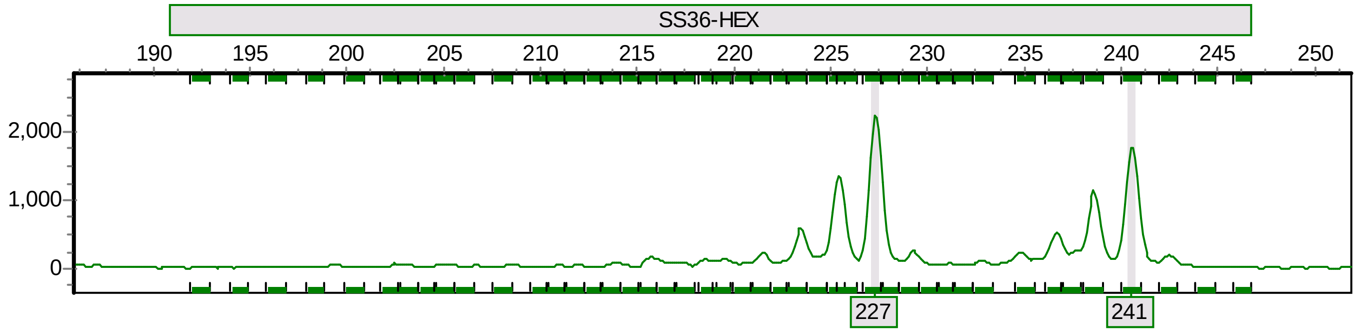

No	Size	Height	Area	Marker	Allele	Difference	Quality	Score	Allele Comments	Sample Comments
1	123.3	17494	119411	SS27-HEX	122	0.30	Pass	500.0	[<Confirmed>]	
2	125.3	10626	72054	SS27-HEX	126	0.10	Pass	500.0	[<Confirmed>]	
3	227.3	2223	16442	SS36-HEX	227	0.10	Pass	348.4	[<Confirmed>]	
4	240.6	1757	13517	SS36-HEX	241	0.00	Pass	232.0	[<Confirmed>]	
5	298.1	8798	77024	SS22-HEX	298	0.10	Pass	500.0		

Sample 104: SS08_SS10_SSS42_SS16_SS27_SS36_SS22_HRS31_J11.fsa

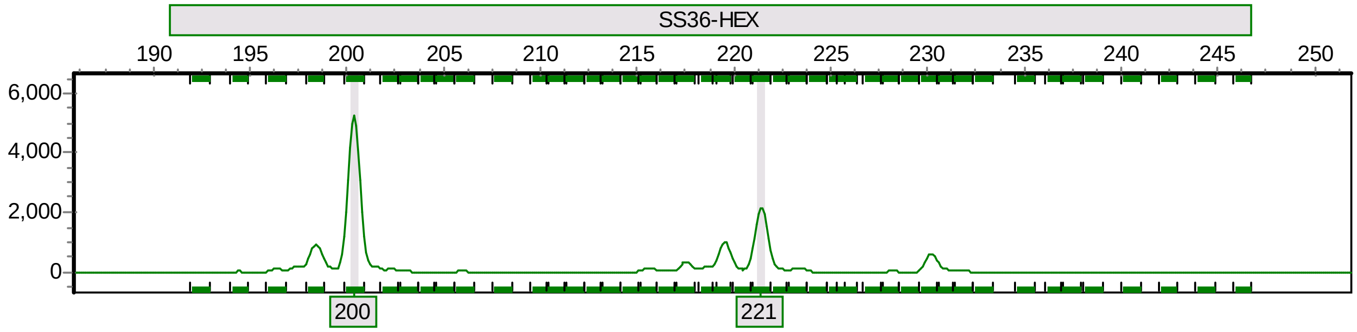

No	Size	Height	Area	Marker	Allele	Difference	Quality	Score	Allele Comments	Sample Comments
1	110.3	9697	66148	SS27-HEX	110	0.00	Pass	500.0	[<Confirmed>]	
2	118.2	6656	45529	SS27-HEX	118	0.40	Pass	500.0	[<Confirmed>]	
3	200.4	5214	35583	SS36-HEX	200	0.00	Pass	500.0	[<Confirmed>]	
4	221.4	2164	15922	SS36-HEX	221	0.00	Pass	333.7	[<Confirmed>]	
5	302.2	4598	42611	SS22-HEX	302	0.10	Pass	500.0		
6	304.3	4417	40783	SS22-HEX	304	0.20	Pass	500.0		

Sample 105: SS08_SS10_SSS42_SS16_SS27_SS36_SS22_HRS33_H07.fsa

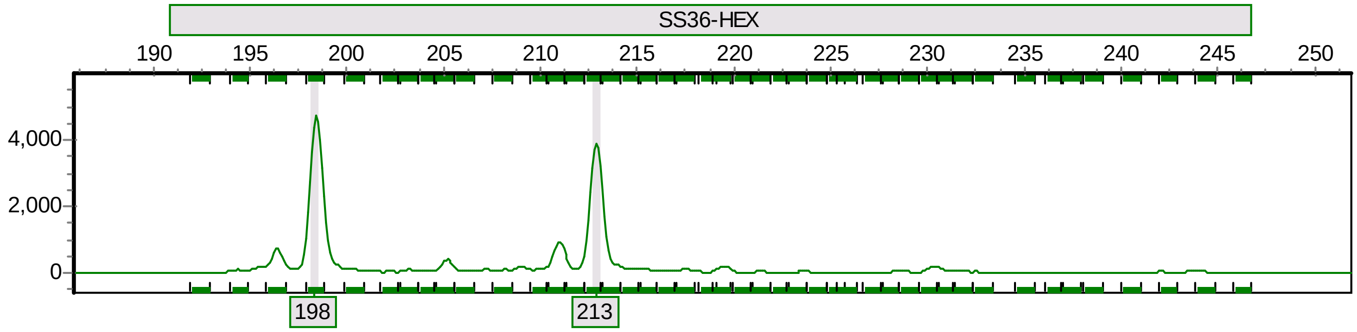

No	Size	Height	Area	Marker	Allele	Difference	Quality	Score	Allele Comments	Sample Comments
1	116.2	8061	57350	SS27-HEX	116	0.00	Pass	500.0	[<Confirmed>]	
2	119.2	2639	16573	SS27-HEX	118	0.60	Undetermined	324.7	[<Deleted>]	
3	120.2	5627	38315	SS27-HEX	120	0.60	Pass	500.0	[<Confirmed>]	
4	198.4	4703	34043	SS36-HEX	198	0.00	Pass	500.0	[<Confirmed>]	

5	212.9	3911	28097	SS36-HEX	213	0.10	Pass	500.0	[<Confirmed>]	
6	298.3	6172	56547	SS22-HEX	298	0.30	Pass	500.0		
7	300.3	4260	40652	SS22-HEX	300	0.30	Pass	497.6		

Sample 106: SS08_SS10_SSS42_SS16_SS27_SS36_SS22_HRS34_L09.fsa

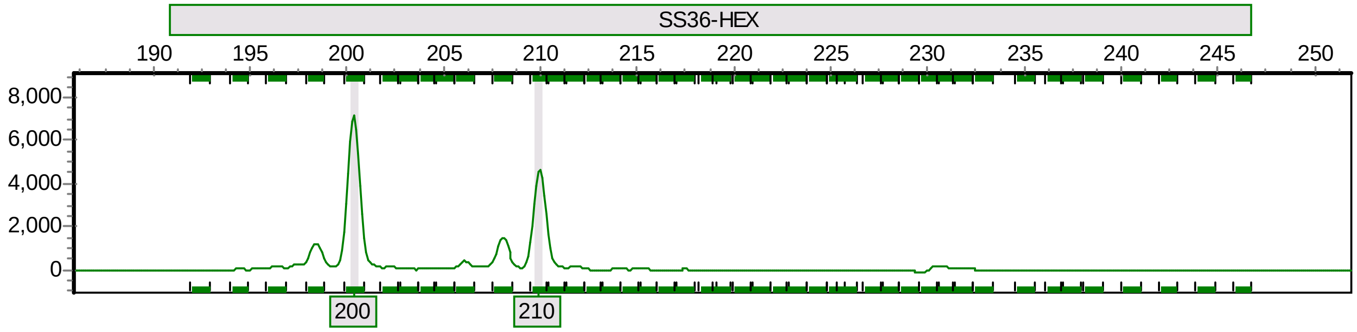

No	Size	Height	Area	Marker	Allele	Difference	Quality	Score	Allele Comments	Sample Comments
1	114.1	15638	104059	SS27-HEX	114	0.10	Pass	500.0	[<Confirmed>]	
2	116.1	11002	73477	SS27-HEX	116	0.10	Pass	500.0	[<Confirmed>]	
3	200.4	7098	48138	SS36-HEX	200	0.00	Pass	500.0	[<Confirmed>]	
4	210.0	4601	32203	SS36-HEX	210	0.00	Pass	500.0	[<Confirmed>]	
5	290.2	8011	67603	SS22-HEX	290	0.10	Pass	500.0		
6	302.0	3987	37988	SS22-HEX	302	0.10	Pass	465.8		

Sample 107: SS08_SS10_SSS42_SS16_SS27_SS36_SS22_HRS35_P09.fsa

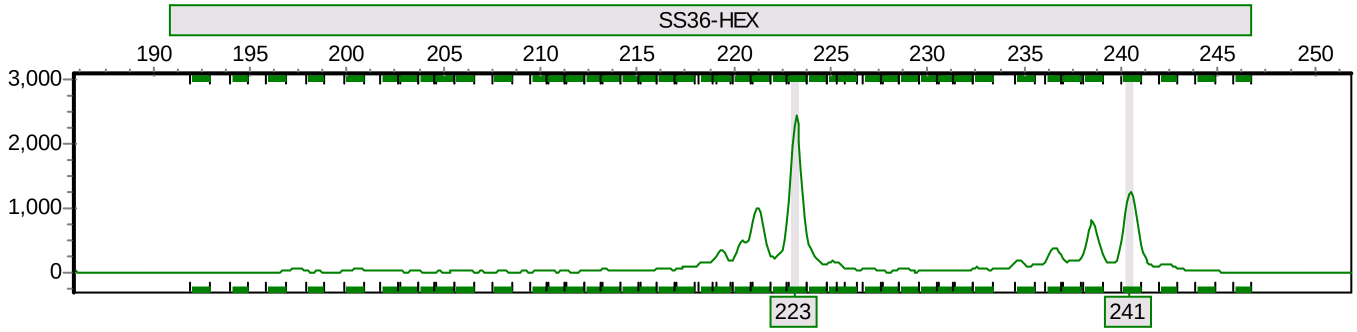

No	Size	Height	Area	Marker	Allele	Difference	Quality	Score	Allele Comments	Sample Comments
1	125.2	10438	68454	SS27-HEX	126	0.00	Pass	500.0	[<Confirmed>]	
2	223.2	2418	17872	SS36-HEX	223	0.10	Pass	366.2	[<Confirmed>]	
3	240.5	1254	9927	SS36-HEX	241	0.10	Pass	123.8	[<Confirmed>]	
4	298.1	3700	33753	SS22-HEX	298	0.10	Pass	462.0		

Sample 108: SS08_SS10_SSS42_SS16_SS27_SS36_SS22_HRS37_J13.fsa

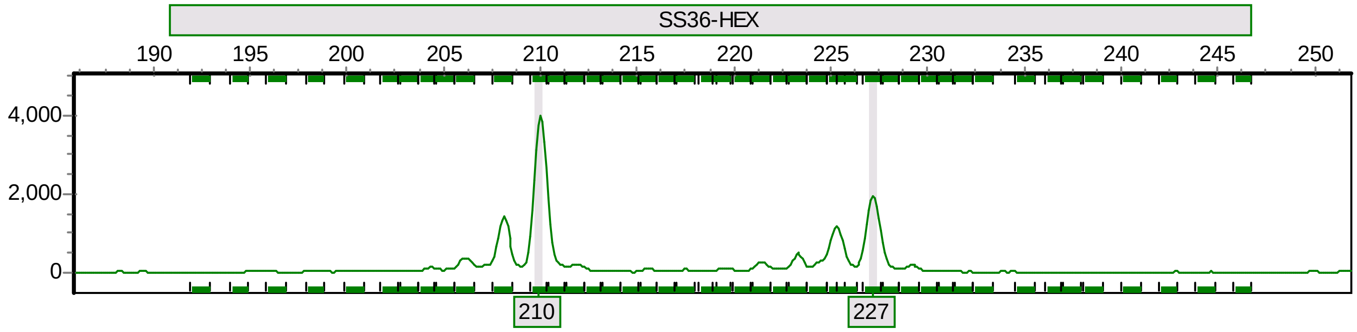

No	Size	Height	Area	Marker	Allele	Difference	Quality	Score	Allele Comments	Sample Comments
1	116.3	9972	70552	SS27-HEX	116	0.10	Pass	500.0	[<Confirmed>]	
2	120.3	7065	48863	SS27-HEX	120	0.50	Pass	500.0	[<Confirmed>]	

3	210.0	3978	28714	SS36-HEX	210	0.00	Pass	500.0	[<Confirmed>]	
4	227.2	1966	15443	SS36-HEX	227	0.00	Pass	262.6	[<Confirmed>]	
5	290.5	5144	45910	SS22-HEX	290	0.20	Pass	500.0		
6	300.1	2988	28639	SS22-HEX	300	0.10	Pass	314.7		

Sample 109: SS08_SS10_SSS42_SS16_SS27_SS36_SS22_HRS38_J07.fsa

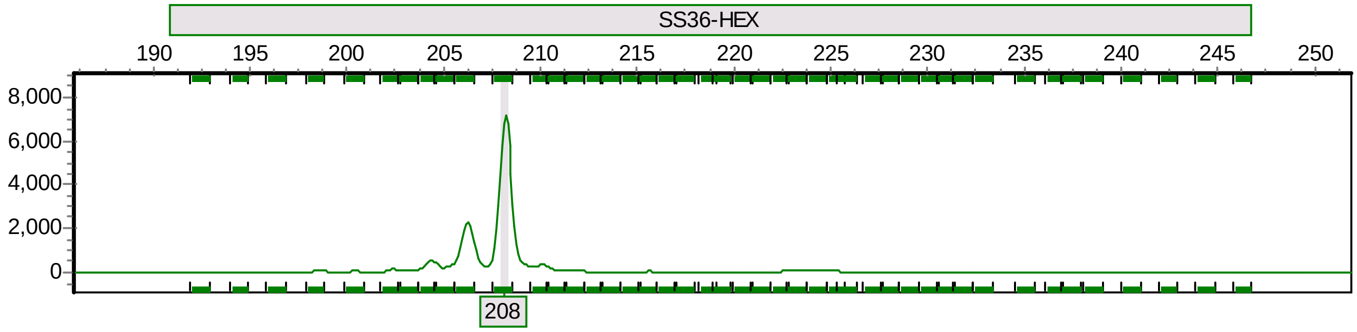

No	Size	Height	Area	Marker	Allele	Difference	Quality	Score	Allele Comments	Sample Comments
1	116.3	11638	79340	SS27-HEX	116	0.10	Pass	500.0	[<Confirmed>]	
2	118.3	7928	56618	SS27-HEX	118	0.30	Pass	500.0	[<Confirmed>]	
3	208.2	7115	51795	SS36-HEX	208	0.10	Pass	500.0	[<Confirmed>]	
4	290.5	5332	47857	SS22-HEX	290	0.20	Pass	500.0		
5	300.1	2810	26338	SS22-HEX	300	0.10	Pass	281.8		

Sample 110: SS08_SS10_SSS42_SS16_SS27_SS36_SS22_HRS39_N07.fsa

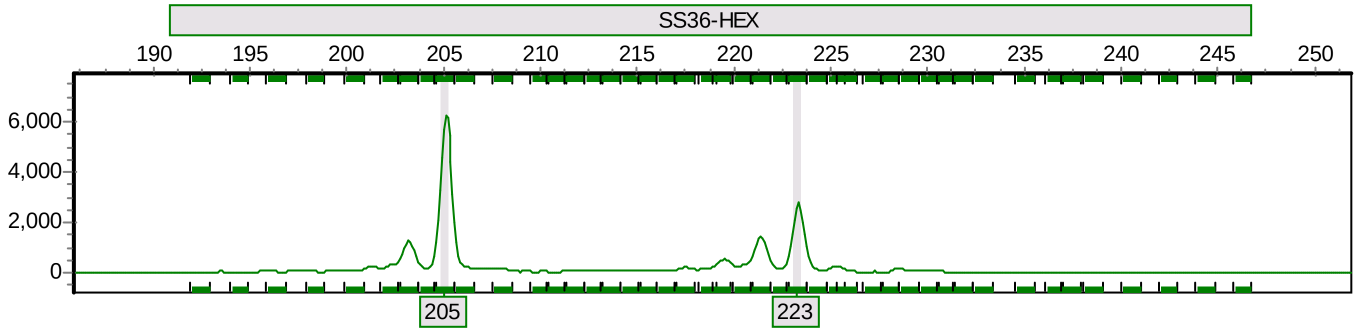

No	Size	Height	Area	Marker	Allele	Difference	Quality	Score	Allele Comments	Sample Comments
1	112.2	12532	85165	SS27-HEX	112	0.00	Pass	500.0	[<Confirmed>]	
2	118.2	8865	61076	SS27-HEX	118	0.40	Pass	500.0	[<Confirmed>]	
3	205.1	6234	43987	SS36-HEX	205	0.00	Pass	500.0	[<Confirmed>]	
4	223.3	2837	20972	SS36-HEX	223	0.00	Pass	491.7	[<Confirmed>]	
5	300.0	7465	68983	SS22-HEX	300	0.00	Pass	500.0		

Sample 111: SS08_SS10_SSS42_SS16_SS27_SS36_SS22_HRS40_A07.fsa

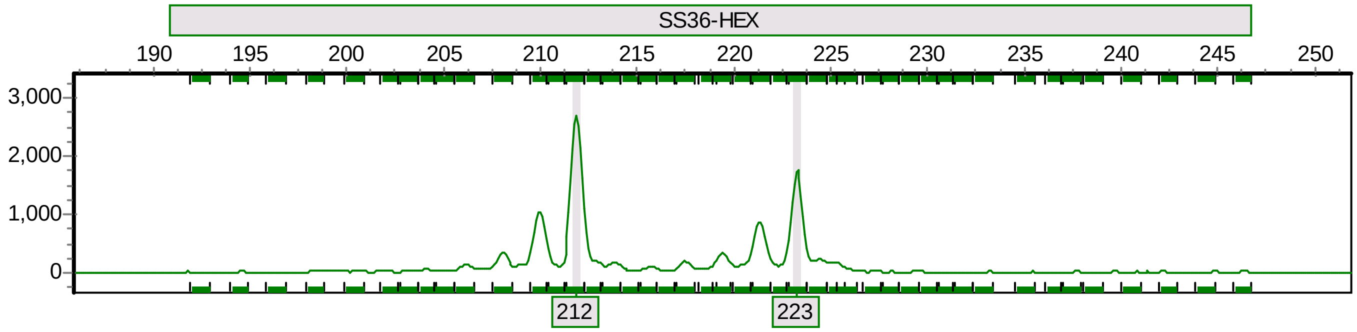

No	Size	Height	Area	Marker	Allele	Difference	Quality	Score	Allele Comments	Sample Comments
1	112.2	9695	64448	SS27-HEX	112	0.00	Pass	500.0	[<Confirmed>]	

2	119.2	3009	20397	SS27-HEX	118	0.60	Undetermined	453.5	[<Deleted>]	
3	120.2	6260	43230	SS27-HEX	120	0.60	Pass	500.0	[<Confirmed>]	
4	211.9	2667	18378	SS36-HEX	212	0.10	Pass	500.0	[<Confirmed>]	
5	223.3	1757	12691	SS36-HEX	223	0.00	Pass	260.5	[<Confirmed>]	
6	299.9	4048	35564	SS22-HEX	300	0.10	Pass	500.0		
7	304.0	4159	37014	SS22-HEX	304	0.10	Pass	500.0		

Sample 112: SS08_SS10_SSS42_SS16_SS27_SS36_SS22_HRS41_K07.fsa

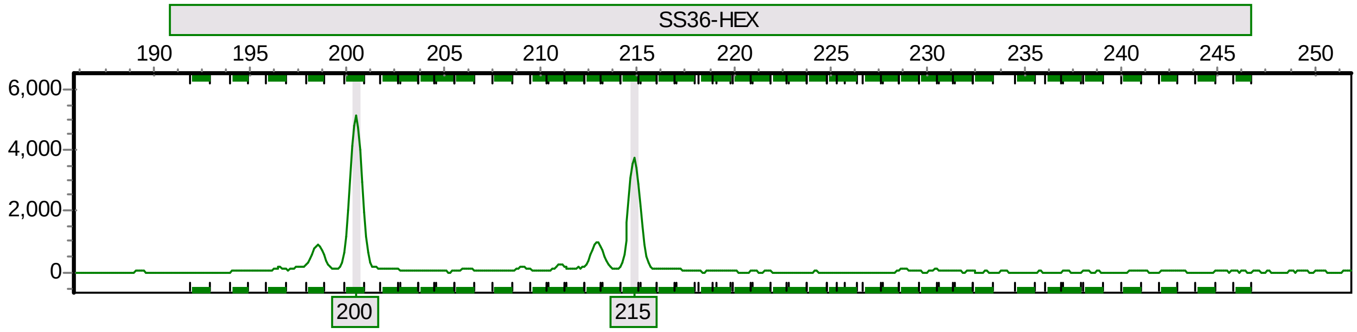

No	Size	Height	Area	Marker	Allele	Difference	Quality	Score	Allele Comments	Sample Comments
1	114.1	12131	83378	SS27-HEX	114	0.10	Pass	500.0	[<Confirmed>]	
2	118.1	8216	55559	SS27-HEX	118	0.50	Pass	500.0	[<Confirmed>]	
3	200.5	5117	34611	SS36-HEX	200	0.10	Pass	500.0	[<Confirmed>]	
4	214.9	3723	26251	SS36-HEX	215	0.20	Pass	500.0	[<Confirmed>]	
5	298.1	3924	33403	SS22-HEX	298	0.10	Pass	500.0		
6	304.1	4368	40755	SS22-HEX	304	0.00	Pass	500.0		

Sample 113: SS08_SS10_SSS42_SS16_SS27_SS36_SS22_HRS42_I07.fsa

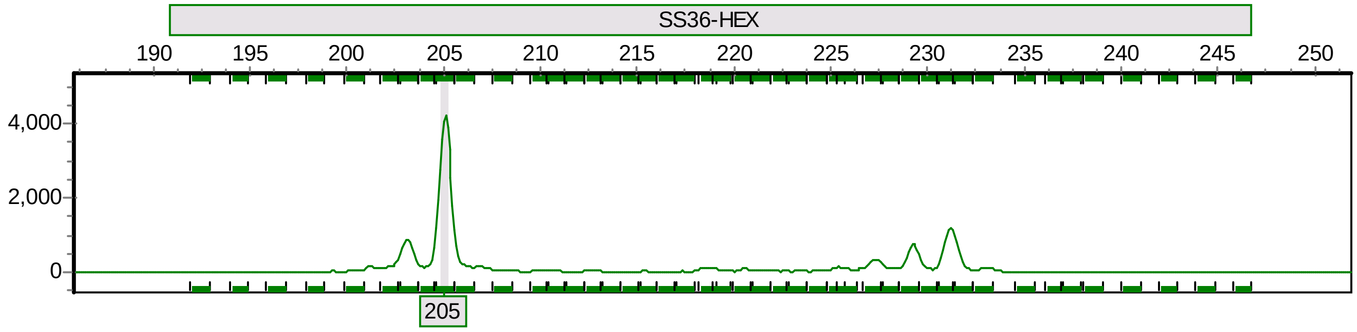

No	Size	Height	Area	Marker	Allele	Difference	Quality	Score	Allele Comments	Sample Comments
1	119.1	15002	106749	SS27-HEX	118	0.50	Pass	500.0	[<Confirmed>]	
2	121.1	9812	66705	SS27-HEX	120	0.30	Pass	500.0	[<Confirmed>]	
3	205.1	4188	30544	SS36-HEX	205	0.00	Pass	500.0	[<Confirmed>]	
4	298.0	3292	30306	SS22-HEX	298	0.00	Pass	381.7		
5	308.3	2003	19756	SS22-HEX	308	0.00	Pass	164.7		

Sample 114: SS08_SS10_SSS42_SS16_SS27_SS36_SS22_HTHL11_M11.fsa

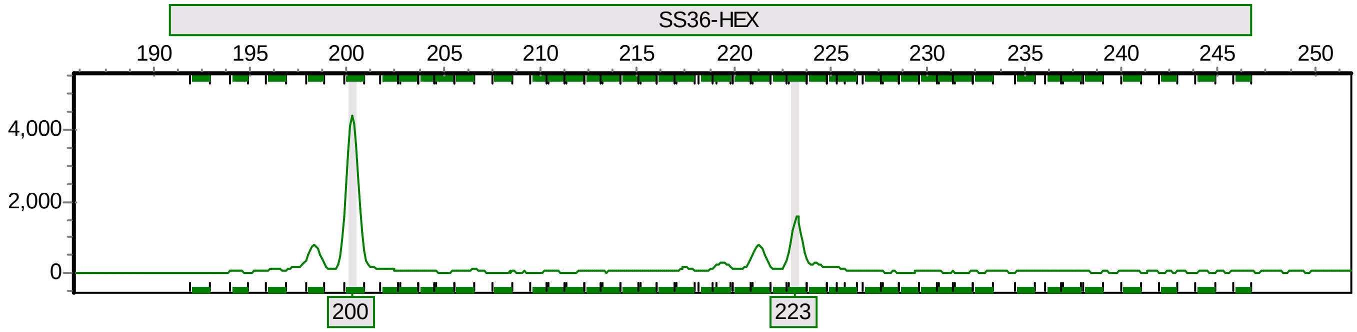

No	Size	Height	Area	Marker	Allele	Difference	Quality	Score	Allele Comments	Sample Comments
1	114.1	9441	65597	SS27-HEX	114	0.10	Pass	500.0	[<Confirmed>]	
2	118.0	6766	46318	SS27-HEX	118	0.60	Pass	500.0	[<Confirmed>]	
3	200.3	4386	29728	SS36-HEX	200	0.10	Pass	500.0	[<Confirmed>]	
4	223.2	1575	11598	SS36-HEX	223	0.10	Pass	213.2	[<Confirmed>]	
5	299.9	2630	23584	SS22-HEX	300	0.10	Pass	280.3		
6	306.1	3075	28882	SS22-HEX	306	0.10	Pass	337.0		

Sample 115: SS08_SS10_SSS42_SS16_SS27_SS36_SS22_HTHL13_H01.fsa

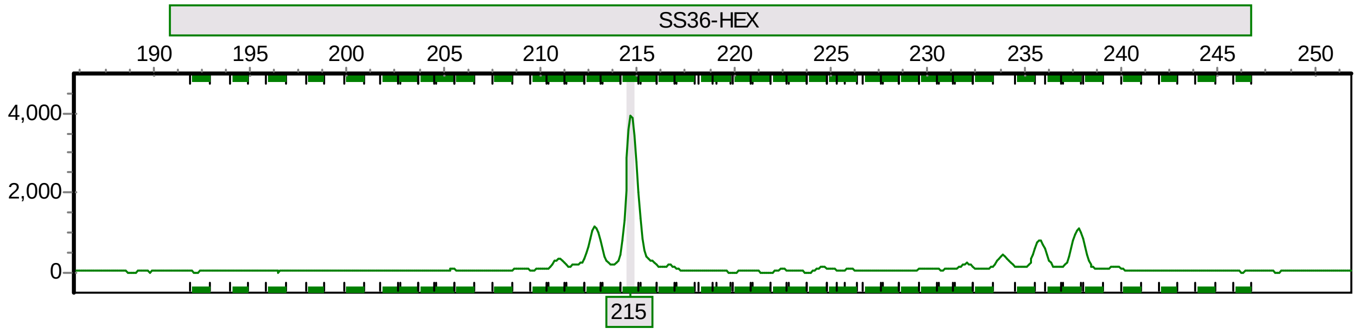

No	Size	Height	Area	Marker	Allele	Difference	Quality	Score	Allele Comments	Sample Comments
1	123.2	14971	97262	SS27-HEX	122	0.20	Pass	500.0	[<Confirmed>]	
2	214.7	3935	28374	SS36-HEX	215	0.00	Pass	500.0	[<Confirmed>]	
3	300.0	4300	38723	SS22-HEX	300	0.00	Pass	500.0		
4	308.3	3879	36018	SS22-HEX	308	0.00	Pass	466.6		

Sample 116: SS08_SS10_SSS42_SS16_SS27_SS36_SS22_HTHL14_F15.fsa

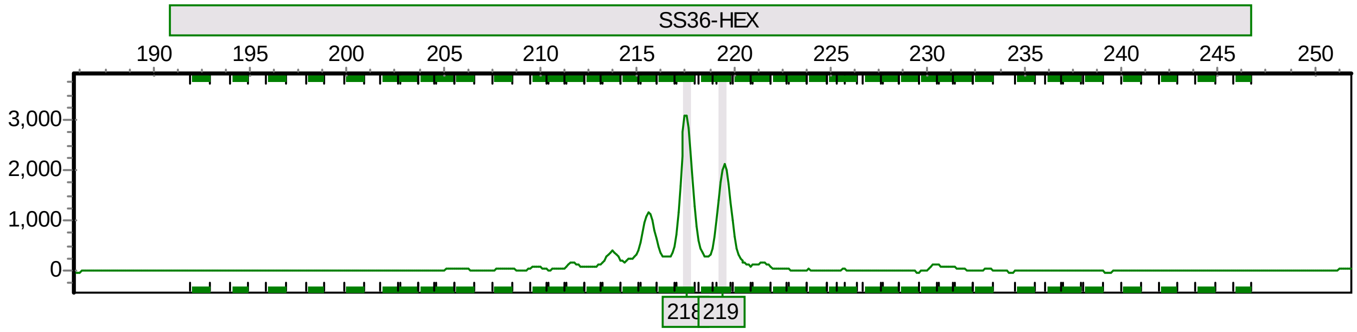

No	Size	Height	Area	Marker	Allele	Difference	Quality	Score	Allele Comments	Sample Comments
1	118.3	13504	92296	SS27-HEX	118	0.30	Pass	500.0	[<Confirmed>]	
2	217.6	3047	23931	SS36-HEX	218	0.10	Pass	482.9	[<Confirmed>]	
3	219.5	2102	15962	SS36-HEX	219	0.10	Pass	295.4	[<Confirmed>]	
4	300.1	5985	57732	SS22-HEX	300	0.10	Pass	500.0		

Allele Report

Sample 117: SS08_SS10_SSS42_SS16_SS27_SS36_SS22_HTHL15_F01.fsa

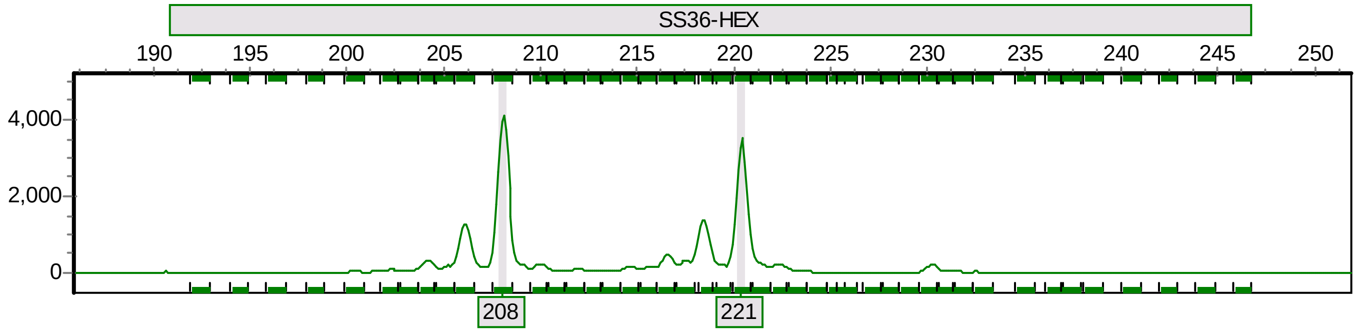

No	Size	Height	Area	Marker	Allele	Difference	Quality	Score	Allele Comments	Sample Comments
1	125.2	16742	111440	SS27-HEX	126	0.00	Pass	500.0	[<Confirmed>]	
2	208.1	4093	27966	SS36-HEX	208	0.00	Pass	500.0	[<Confirmed>]	
3	220.4	3538	24537	SS36-HEX	221	0.10	Pass	500.0	[<Confirmed>]	
4	290.3	7732	62542	SS22-HEX	290	0.00	Pass	500.0		
5	302.1	4780	43733	SS22-HEX	302	0.00	Pass	500.0		

Sample 118: SS08_SS10_SSS42_SS16_SS27_SS36_SS22_HTHL1_E13.fsa

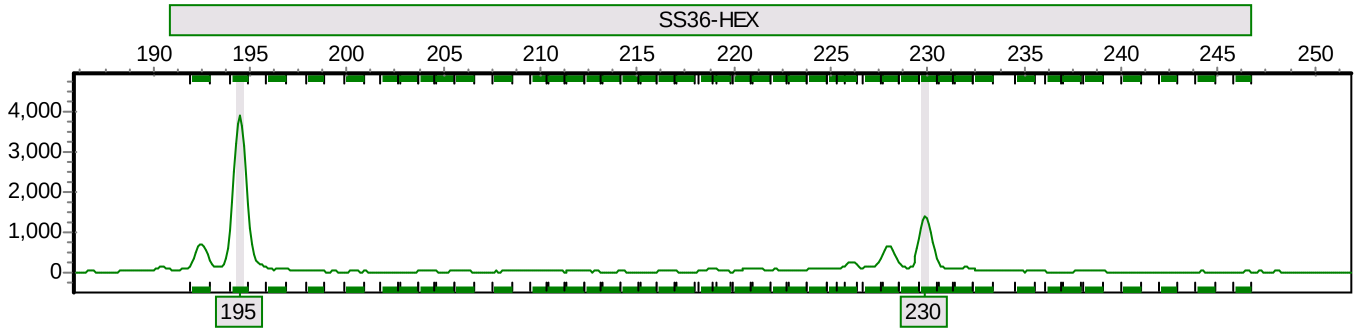

No	Size	Height	Area	Marker	Allele	Difference	Quality	Score	Allele Comments	Sample Comments
1	104.3	6642	43692	SS27-HEX	104	0.00	Pass	500.0	[<Deleted>]	
2	105.3	15432	102666	SS27-HEX	105	0.00	Pass	500.0	[<Confirmed>]	
3	125.1	5714	40772	SS27-HEX	126	0.10	Pass	500.0	[<Confirmed><Edited>]	
4	194.5	3866	28241	SS36-HEX	195	0.00	Pass	500.0	[<Confirmed>]	
5	229.9	1371	10733	SS36-HEX	230	0.20	Pass	154.6	[<Confirmed>]	
6	290.2	3980	34713	SS22-HEX	290	0.10	Pass	500.0		
7	302.0	2296	22073	SS22-HEX	302	0.10	Pass	204.3		

Sample 119: SS08_SS10_SSS42_SS16_SS27_SS36_SS22_HTHL3_D05.fsa

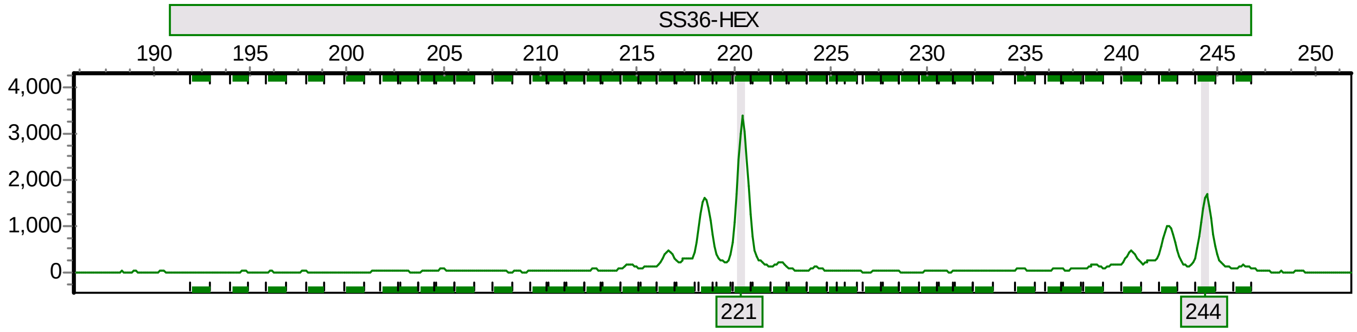

No	Size	Height	Area	Marker	Allele	Difference	Quality	Score	Allele Comments	Sample Comments
1	116.3	4906	30424	SS27-HEX	116	0.10	Pass	500.0	[<Deleted>]	
2	117.4	13221	87135	SS27-HEX	118	1.00	Pass	500.0	[<Confirmed><Edited>]	
3	123.2	10711	71033	SS27-HEX	122	0.20	Pass	500.0	[<Confirmed>]	

4	220.4	3356	24390	SS36-HEX	221	0.10	Pass	500.0	[<Confirmed>]	
5	244.4	1689	13105	SS36-HEX	244	0.00	Pass	215.0	[<Confirmed>]	
6	294.2	6278	52244	SS22-HEX	294	0.00	Pass	500.0		
7	302.2	4583	41973	SS22-HEX	302	0.10	Pass	500.0		

Sample 120: SS08_SS10_SSS42_SS16_SS27_SS36_SS22_HTHL4_O15.fsa

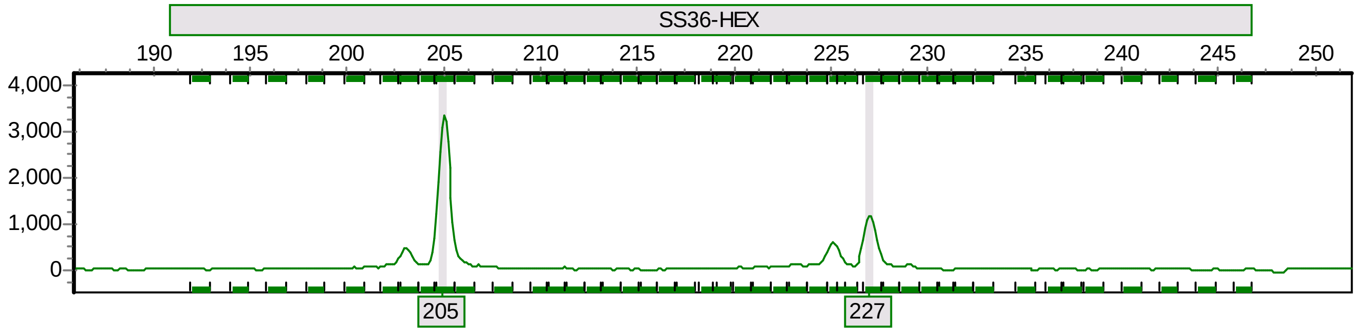

No	Size	Height	Area	Marker	Allele	Difference	Quality	Score	Allele Comments	Sample Comments
1	120.0	2424	16864	SS27-HEX	120	1.00	Pass	394.0	[<Confirmed><Edited>]	
2	127.1	7689	54554	SS27-HEX	128	1.00	Pass	500.0	[<Confirmed><Edited>]	
3	205.0	3329	23538	SS36-HEX	205	0.10	Pass	500.0	[<Confirmed>]	
4	227.0	1164	9006	SS36-HEX	227	0.20	Pass	126.0	[<Confirmed>]	
5	290.2	2930	26183	SS22-HEX	290	0.10	Pass	355.4		
6	300.0	1806	17361	SS22-HEX	300	0.00	Pass	147.4		

Sample 121: SS08_SS10_SSS42_SS16_SS27_SS36_SS22_HTHL5_M13.fsa

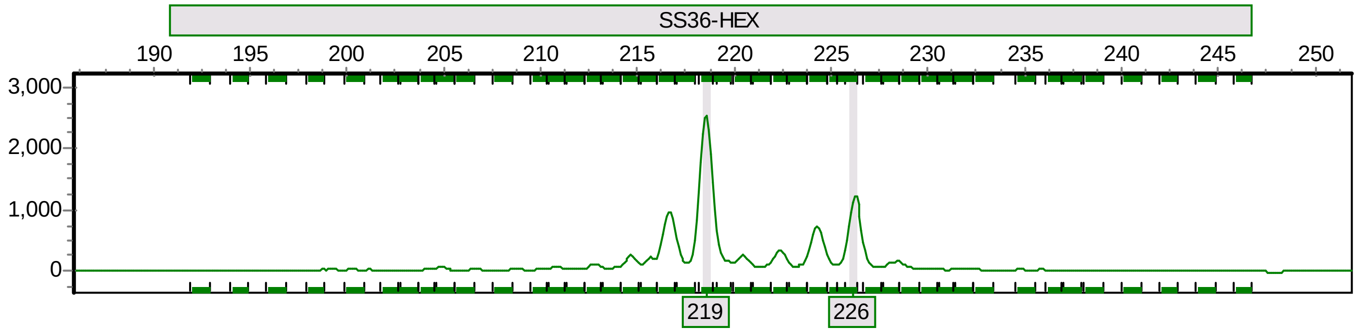

No	Size	Height	Area	Marker	Allele	Difference	Quality	Score	Allele Comments	Sample Comments
1	112.2	19071	130221	SS27-HEX	112	0.00	Pass	500.0	[<Confirmed>]	
2	218.6	2530	18910	SS36-HEX	219	0.10	Pass	402.8	[<Confirmed>]	
3	226.2	1235	9614	SS36-HEX	226	0.30	Pass	135.7	[<Confirmed>]	
4	304.1	2028	20280	SS22-HEX	304	0.00	Pass	160.0		
5	316.4	1245	12420	SS22-HEX	316	0.00	Pass	74.2		

Sample 122: SS08_SS10_SSS42_SS16_SS27_SS36_SS22_HTHL6_P03.fsa

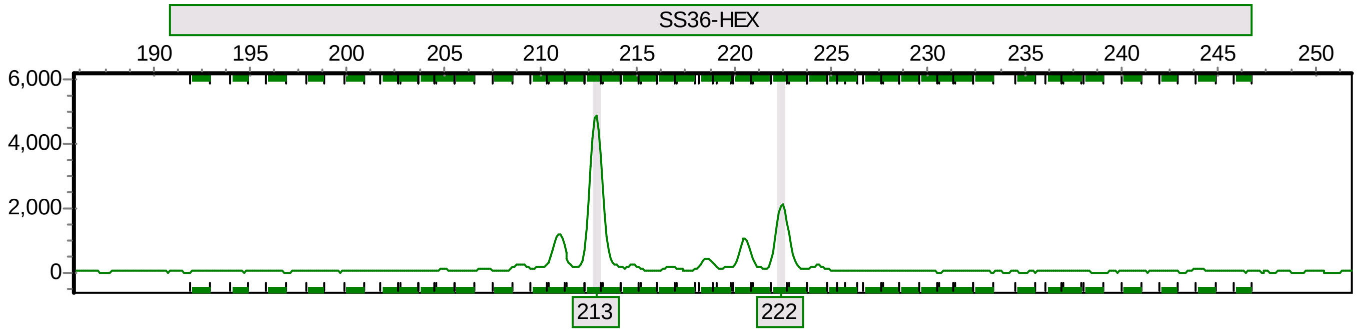

No	Size	Height	Area	Marker	Allele	Difference	Quality	Score	Allele Comments	Sample Comments

1	119.1	18795	122968	SS27-HEX	118	0.50	Pass	500.0	[<Confirmed>]
2	212.9	4825	33825	SS36-HEX	213	0.10	Pass	500.0	[<Confirmed>]
3	222.5	2083	15243	SS36-HEX	222	0.10	Pass	319.0	[<Confirmed>]
4	296.0	9015	77720	SS22-HEX	296	0.10	Pass	500.0	

Sample 123: SS08_SS10_SSS42_SS16_SS27_SS36_SS22_HTHL8_A03.fsa

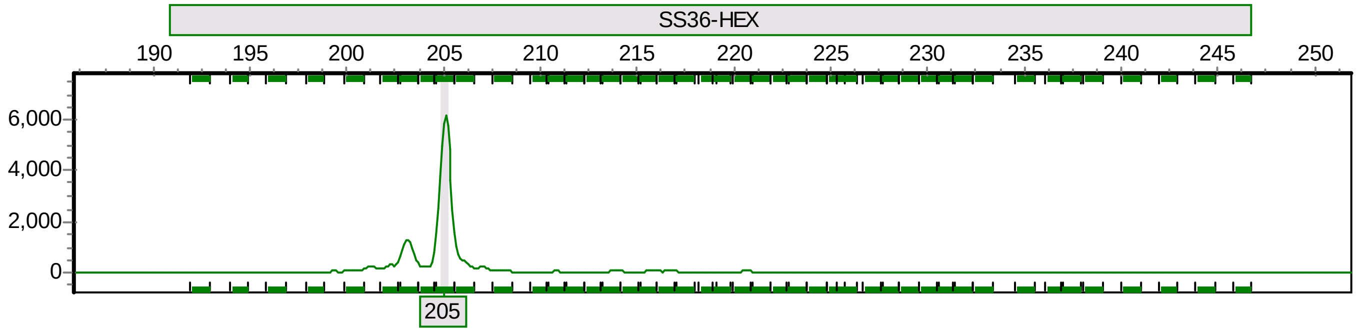

No	Size	Height	Area	Marker	Allele	Difference	Quality	Score	Allele Comments	Sample Comments
1	130.0	6281	42882	SS27-HEX	130	0.10	Pass	500.0	[<Confirmed>]	
2	136.1	3923	28119	SS27-HEX	136	0.00	Pass	500.0	[<Confirmed>]	
3	205.1	6090	42519	SS36-HEX	205	0.00	Pass	500.0	[<Confirmed>]	
4	284.2	5648	46326	SS22-HEX	284	0.10	Pass	500.0		
5	299.8	2671	24084	SS22-HEX	300	0.20	Pass	294.8		

Sample 124: SS08_SS10_SSS42_SS16_SS27_SS36_SS22_HTHL9_O09.fsa

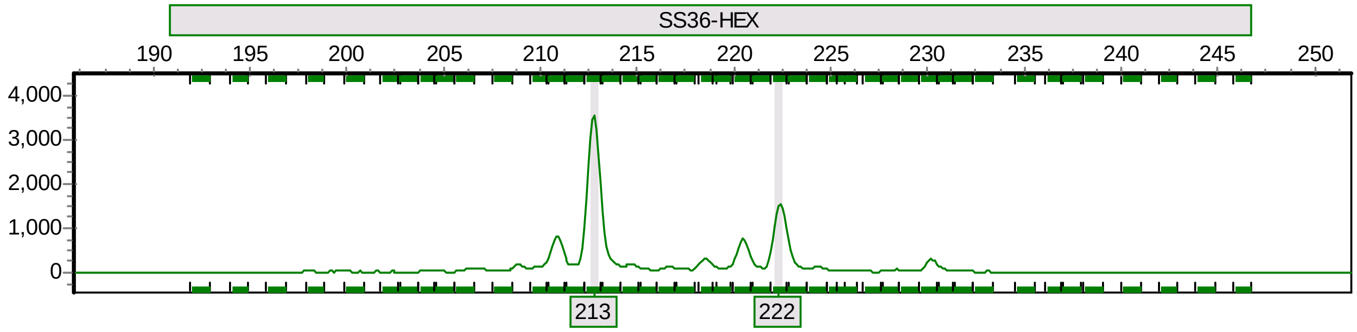

No	Size	Height	Area	Marker	Allele	Difference	Quality	Score	Allele Comments	Sample Comments
1	114.2	3916	29172	SS27-HEX	114	0.00	Pass	500.0	[<Confirmed>]	
2	123.2	10275	70701	SS27-HEX	122	0.20	Pass	500.0	[<Confirmed>]	
3	212.8	3530	25622	SS36-HEX	213	0.00	Pass	500.0	[<Confirmed>]	
4	222.4	1565	11618	SS36-HEX	222	0.00	Pass	197.0	[<Confirmed>]	
5	296.0	3685	32795	SS22-HEX	296	0.10	Pass	469.5		
6	299.9	3059	28828	SS22-HEX	300	0.10	Pass	350.0		
