# Supplementary material for: Genetic diversity of Schima superba based on physiological traits and SSR markers
Source: PLoS One. 2026 Apr 10;21(4):e0344465. doi: 10.1371/journal.pone.0344465 (PMC13068225; doi:10.1371/journal.pone.0344465)
Supplement: Attachment — Submitted filename: Plos One review .pdf [file pone.0344465.s006.pdf]

The review of the manuscript titled “Genetic Diversity of Schima superba Based on Physiological Traits and SSR Markers” has been reviewed in detail. This subject is important and timely because Schima superba is a tree species of economic importance which acts as a major source of reforestation and landscape development in subtropical China.

The genetic diversity of 122 single genotypes was assessed by the authors based on physiological characteristics and simple sequence repeat (SSR) markers. Interestingly, the markers SS30 and SS32 had close correlations with chlorophyll and malondialdehyde content, which points to the possibility of their involvement in the improvement of plant defense mechanisms during extreme stress factors like drought and salinity. These data offer useful information and have the potential towards coming up with trait-specific markers to enhance the breeding programs in order to have stress-tolerant lines.

The paper is well structured and in a logical scientific manner. The authors have sufficiently answered all the questions of the reviewers and the modified version is satisfactory. The discussion, results and the methodology are well presented and scientifically justified.

Regarding the revision and the quality of the work in general, I would recommend the **acceptance of this manuscript**. In my opinion, this research offers a valuable platform on which molecular breeding programs can be implemented to make stress-tolerant lines.
