# Supplementary material for: Genetic diversity of Schima superba based on physiological traits and SSR markers
Source: PLoS One. 2026 Apr 10;21(4):e0344465. doi: 10.1371/journal.pone.0344465 (PMC13068225; doi:10.1371/journal.pone.0344465)
Supplement: Attachment — Submitted filename: Schimasuperba-PlosOne.docx [file pone.0344465.s004.docx]

This is a well-structured and scientifically sound study that effectively integrates physiological trait assessment with SSR-based genetic analysis across a substantial number of Schima superba populations. The combination of phenotypic and molecular data strengthens the study’s relevance for conservation, breeding, and adaptive management. The work provides important insights into population variability, genetic clustering, and marker–trait associations, offering valuable resources for future improvement and management programs. However, before publication, the below-mentioned minor issues must be addressed:

1. Provide all abbreviations after keywords used in the manuscript.
2. Include a discussion and figure regarding SSR based dendrogram in this genetic diversity analysis section.
3. The dendrogram generated from SSR markers needs improvement in clarity and formatting.
4. Include bootstrap values, proper clustering labels, and scale on the dendrogram.
5. Provide a more detailed discussion interpreting the clustering pattern and its biological significance.
6. AMOVA results should be presented clearly, preferably with a figure.
7. Discuss how the STRUCTURE groups correlate with geographical origin or genetic diversity.
8. Revise the conclusion section to improve. Highlight key findings, practical implications, novelty, and future directions.
9. The reference list is comprehensive; ensure consistent formatting according to journal guidelines.
