# Supplementary material for: Genetic diversity of Schima superba based on physiological traits and SSR markers
Source: PLoS One. 2026 Apr 10;21(4):e0344465. doi: 10.1371/journal.pone.0344465 (PMC13068225; doi:10.1371/journal.pone.0344465)
Supplement: Attachment — Submitted filename: Response to Reviewers.docx [file pone.0344465.s007.docx]

**Response to Reviewers**

First of all, we sincerely thank the editors and reviewers for their valuable comments concerning our manuscript entitled **"Genetic diversity of *Schima superba* based on physiological traits and SSR markers"(Manuscript ID: PONE-D-25-59450)**. Those comments are very valuable and helpful for us to revise and improve our research. We carefully considered all comments and revised our manuscript accordingly to make our results convincing. To facilitate the review process, all changes have been made and clearly marked using the Track Changes function in Microsoft Word. The responses to the reviewer's comments are listed below.

**Responses to editors and reviewers (original comments by editors and reviewers are in blue.)**

**To Editor:**

**1. Comment:** The amplified products were separated and analyzed using capillary electrophoresis on an ABI 3130xl Genetic Analyzer (Applied Biosystems, USA), and allele sizes were determined using GeneMarker software. Authors have given average value of Numbers of allele as more than 15 which is very high and interesting. In this regard, authors are requested to provide a clear and representative figure illustrating the differentiation of alleles, preferably in the form of a peak (electropherogram) profile.

**Response:**

Thank you for this helpful comment. We agree that a representative capillary electropherogram would improve the clarity of allele differentiation. Therefore, we have provided the electropherogram peak profiles for all SSR loci and samples in the Supplementary Material (S1 File), which clearly illustrates allele separation and sizing based on the ABI 3130xl Genetic Analyzer and GeneMarker outputs.

2. **Comment:** The conclusion is very general. The authors are requested to strengthen this section by specifically highlighting the populations or accessions identified as promising for further research, based on the parameters evaluated in the study.

**Response:**

Thank you for the suggestion. We strengthened the Conclusion by explicitly identifying HTHL and HBB as the most promising provenances for further utilization based on combined physiological and SSR diversity parameters, and highlighting SS30 and SS32 as SSR loci with potential value for marker-assisted selection. (Revised Manuscript with Track Changes, lines 491–502)

3. **Comment:** quality of figures is not good please check that.

**Response:**

Thank you for pointing this out. We have carefully checked the quality of all figures and re-uploaded high-resolution versions in the revised manuscript. In particular, Fig. 4 has been replaced with a clearer, higher-quality image with improved readability.

**To Reviewer:**

**1. Comment:** Provide all abbreviations after keywords used in the manuscript.

**Response:**

We appreciate the reviewer's advice. We have inserted a table of abbreviations after the abstract section to facilitate reading. This table lists all abbreviations used in the manuscript along with their full definitions. Additionally, we confirmed that all abbreviations are fully defined when first mentioned in the text. (Revised Manuscript with Track Changes, line 30)

**2.** **Comment:** Include a discussion and figure regarding SSR based dendrogram in this genetic diversity analysis section.

**Response:**

Thank you for the suggestion. We have revised the genetic diversity analysis section by including the dendrogram (Fig 1) and adding corresponding discussion to interpret the clustering pattern and its biological significance in the revised manuscript. (Revised Manuscript with Track Changes, lines 223–231)

**3. Comment:** The dendrogram generated from SSR markers needs improvement in clarity and formatting.

**Response:**

Thank you for the comment. We have carefully checked and improved the clarity and formatting of the SSR-based dendrogram and all other figures. The updated high-resolution dendrogram (Fig1 and Fig 4) has been replaced in the revised manuscript to ensure optimal readability.(Revised Manuscript with Track Changes, lines 371–378)

**4. Comment:** Include bootstrap values, proper clustering labels, and scale on the dendrogram.

**Response:**

Thank you for this suggestion. We have updated the SSR-based dendrogram (Fig. 4) by adding bootstrap values, appropriate clustering labels, and a scale bar to improve the interpretability and presentation quality in the revised manuscript.

**5. Comment:** Provide a more detailed discussion interpreting the clustering pattern and its biological significance.

**Response:**

Thank you for this valuable comment. We have expanded the Discussion section by adding a more detailed interpretation of the SSR-based clustering pattern and explaining its biological significance, including the potential implications for genetic differentiation, germplasm conservation, and future utilization of *S. superba* populations. (Revised Manuscript with Track Changes, lines 384–396)

**6. Comment:** AMOVA results should be presented clearly, preferably with a figure.

**Response:**

We appreciate the reviewer’s suggestion to visualize the AMOVA results. We carefully considered converting the data into a figure; however, we found that a graphical representation (e.g., pie charts or bar plots) tends to obscure the precise statistical values, particularly the variance components, which is critical for the interpretation of our findings. Therefore, we respectfully prefer to retain Table 7, as it allows readers to access the exact numerical results directly.

**7. Comment:** Discuss how the STRUCTURE groups correlate with geographical origin or genetic diversity.

**Response:**

Thank you for this important suggestion. We have revised the Discussion section to further explain how the STRUCTURE-defined genetic groups correlate with geographical origins and patterns of genetic diversity among the sampled provenances, and we highlighted the potential implications for germplasm conservation and utilization in the revised manuscript. (Revised Manuscript with Track Changes, lines 392–404)

**8.Comment:** Revise the conclusion section to improve. Highlight key findings, practical implications, novelty, and future directions.

**Response:**

Thank you for the suggestion. We have revised the Conclusion section to better highlight the key findings, practical implications, novelty, and future directions. The revised conclusion now summarizes the major genetic diversity and population structure results, emphasizes the most promising provenances for further utilization, proposes conservation and breeding implications, and outlines future validation and application of the associated SSR loci.(Revised Manuscript with Track Changes, lines 491–502)

**9. Comment:** The reference list is comprehensive; ensure consistent formatting according to journal guidelines.

**Response:**

We thank the reviewer for the positive comment and the reminder regarding formatting. We have carefully double-checked the entire reference list and reformatted it to ensure strict compliance with the PLOS One’s author guidelines.
